# Supplementary material for: Peritumoral radiomics features predict distant metastasis in locally advanced NSCLC
Source: PLoS One. 2018 Nov 2;13(11):e0206108. doi: 10.1371/journal.pone.0206108 (PMC6214508; doi:10.1371/journal.pone.0206108)
Supplement: S7 File — (PDF) [file pone.0206108.s007.pdf]

X	general_info_BoundingBox
1	(6, 6, 5, 11, 11, 14)
2	(6, 5, 6, 19, 21, 17)
3	(6, 5, 5, 8, 13, 7)
4	(5, 6, 5, 18, 20, 21)
5	(6, 6, 6, 9, 9, 7)
6	(6, 6, 6, 11, 11, 9)
8	(5, 6, 6, 12, 10, 5)
9	(6, 6, 6, 15, 14, 12)
10	(5, 6, 6, 21, 18, 15)
11	(5, 6, 6, 25, 19, 24)
12	(5, 6, 5, 11, 13, 10)
13	(6, 5, 6, 14, 16, 19)
14	(6, 5, 6, 19, 18, 16)
15	(5, 5, 6, 14, 16, 12)
16	(5, 6, 6, 15, 12, 13)
17	(6, 6, 6, 33, 28, 36)
18	(6, 6, 6, 15, 9, 10)
19	(6, 6, 5, 21, 19, 21)
20	(6, 6, 6, 10, 11, 11)
21	(6, 5, 6, 21, 24, 24)
22	(6, 6, 6, 23, 24, 34)
23	(6, 6, 6, 15, 12, 21)
24	(5, 5, 6, 20, 28, 31)
25	(5, 6, 6, 11, 10, 9)
26	(6, 6, 5, 18, 22, 16)
27	(6, 6, 6, 10, 15, 10)
29	(5, 6, 5, 19, 27, 20)
30	(5, 5, 6, 16, 32, 15)
31	(5, 5, 5, 13, 25, 28)
32	(6, 6, 6, 20, 20, 20)
33	(6, 6, 6, 19, 29, 27)
34	(6, 5, 6, 34, 33, 32)
35	(6, 5, 6, 17, 17, 18)
36	(6, 5, 6, 5, 5, 5)
37	(6, 6, 5, 8, 9, 10)
38	(6, 6, 6, 18, 17, 15)
39	(5, 5, 6, 21, 32, 30)
40	(6, 6, 6, 22, 25, 18)
41	(6, 6, 5, 11, 14, 10)
42	(6, 5, 6, 14, 13, 13)
43	(6, 5, 6, 14, 19, 16)
44	(6, 6, 6, 16, 13, 12)
45	(5, 6, 6, 25, 14, 14)
46	(5, 5, 6, 14, 14, 15)
48	(5, 6, 5, 20, 17, 14)
49	(6, 6, 6, 22, 35, 26)
50	(6, 5, 1, 22, 21, 28)
51	(6, 5, 5, 10, 10, 9)
52	(6, 6, 5, 5, 6, 6)
53	(6, 5, 6, 8, 12, 10)
54	(6, 6, 5, 27, 25, 25)
55	(5, 5, 6, 12, 12, 11)
56	(5, 6, 6, 20, 23, 30)
57	(6, 6, 5, 11, 11, 11)
58	(5, 6, 6, 29, 44, 34)
59	(6, 6, 5, 12, 13, 11)
60	(5, 6, 6, 25, 20, 21)
61	(6, 6, 5, 18, 13, 15)
62	(6, 6, 5, 12, 13, 12)
63	(6, 6, 6, 16, 12, 9)
64	(6, 5, 5, 17, 19, 16)
65	(5, 6, 6, 26, 28, 33)
66	(5, 6, 6, 22, 20, 35)
67	(5, 5, 6, 23, 27, 19)
68	(6, 6, 6, 8, 7, 6)
69	(5, 6, 5, 22, 15, 15)
70	(6, 6, 6, 10, 8, 8)
71	(6, 6, 6, 24, 21, 23)
72	(6, 6, 6, 10, 9, 9)
73	(6, 6, 6, 19, 23, 20)
74	(6, 6, 5, 25, 25, 22)
75	(5, 6, 6, 14, 14, 12)
76	(6, 6, 6, 25, 31, 29)
77	(6, 5, 6, 31, 39, 37)
78	(6, 6, 5, 37, 40, 36)
79	(5, 6, 6, 15, 17, 12)
80	(6, 6, 6, 15, 17, 10)
81	(6, 5, 6, 10, 10, 11)
82	(6, 5, 5, 21, 19, 12)
83	(5, 5, 5, 19, 29, 22)
84	(6, 6, 6, 12, 11, 6)
85	(6, 6, 6, 18, 23, 11)
86	(5, 6, 5, 20, 24, 26)
87	(6, 5, 6, 6, 7, 5)
88	(6, 5, 6, 24, 27, 26)
89	(6, 6, 6, 33, 23, 23)
90	(6, 6, 6, 12, 11, 11)
91	(5, 6, 6, 16, 26, 13)
92	(6, 6, 6, 8, 9, 5)
93	(6, 6, 6, 7, 6, 3)
94	(5, 5, 5, 28, 34, 21)
95	(6, 6, 6, 11, 12, 14)
96	(5, 5, 6, 14, 17, 11)
107	(6, 6, 6, 24, 25, 23)
108	(6, 6, 5, 26, 29, 28)
112	(6, 6, 6, 7, 7, 7)
114	(6, 6, 6, 16, 13, 13)
126	(6, 6, 5, 5, 6, 6)
135	(6, 6, 6, 9, 10, 7)
139	(6, 5, 5, 17, 15, 12)

general_info_GeneralSettings

{'distances': [1], 'verbose': True, 'additionalInfo': True, 'enableCExtensions': True, 'force2D': False, 'interpolator': 3, 'resampledPixelSpacing': [3, 3, 3], 'label': 1, 'normalizeScale': 1, 'normalize': False, 'force2Ddimension': 0, 'removeOutliers': None, 'minimumROISi
{'distances': [1], 'verbose': True, 'additionalInfo': True, 'enableCExtensions': True, 'force2D': False, 'interpolator': 3, 'resampledPixelSpacing': [3, 3, 3], 'label': 1, 'normalizeScale': 1, 'normalize': False, 'force2Ddimension': 0, 'removeOutliers': None, 'minimumROISi
{'distances': [1], 'verbose': True, 'additionalInfo': True, 'enableCExtensions': True, 'force2D': False, 'interpolator': 3, 'resampledPixelSpacing': [3, 3, 3], 'label': 1, 'normalizeScale': 1, 'normalize': False, 'force2Ddimension': 0, 'removeOutliers': None, 'minimumROISi
{'distances': [1], 'verbose': True, 'additionalInfo': True, 'enableCExtensions': True, 'force2D': False, 'interpolator': 3, 'resampledPixelSpacing': [3, 3, 3], 'label': 1, 'normalizeScale': 1, 'normalize': False, 'force2Ddimension': 0, 'removeOutliers': None, 'minimumROISi
{'distances': [1], 'verbose': True, 'additionalInfo': True, 'enableCExtensions': True, 'force2D': False, 'interpolator': 3, 'resampledPixelSpacing': [3, 3, 3], 'label': 1, 'normalizeScale': 1, 'normalize': False, 'force2Ddimension': 0, 'removeOutliers': None, 'minimumROISi
{'distances': [1], 'verbose': True, 'additionalInfo': True, 'enableCExtensions': True, 'force2D': False, 'interpolator': 3, 'resampledPixelSpacing': [3, 3, 3], 'label': 1, 'normalizeScale': 1, 'normalize': False, 'force2Ddimension': 0, 'removeOutliers': None, 'minimumROISi
{'distances': [1], 'verbose': True, 'additionalInfo': True, 'enableCExtensions': True, 'force2D': False, 'interpolator': 3, 'resampledPixelSpacing': [3, 3, 3], 'label': 1, 'normalizeScale': 1, 'normalize': False, 'force2Ddimension': 0, 'removeOutliers': None, 'minimumROISi
{'distances': [1], 'verbose': True, 'additionalInfo': True, 'enableCExtensions': True, 'force2D': False, 'interpolator': 3, 'resampledPixelSpacing': [3, 3, 3], 'label': 1, 'normalizeScale': 1, 'normalize': False, 'force2Ddimension': 0, 'removeOutliers': None, 'minimumROISi
{'distances': [1], 'verbose': True, 'additionalInfo': True, 'enableCExtensions': True, 'force2D': False, 'interpolator': 3, 'resampledPixelSpacing': [3, 3, 3], 'label': 1, 'normalizeScale': 1, 'normalize': False, 'force2Ddimension': 0, 'removeOutliers': None, 'minimumROISi
{'distances': [1], 'verbose': True, 'additionalInfo': True, 'enableCExtensions': True, 'force2D': False, 'interpolator': 3, 'resampledPixelSpacing': [3, 3, 3], 'label': 1, 'normalizeScale': 1, 'normalize': False, 'force2Ddimension': 0, 'removeOutliers': None, 'minimumROISi
{'distances': [1], 'verbose': True, 'additionalInfo': True, 'enableCExtensions': True, 'force2D': False, 'interpolator': 3, 'resampledPixelSpacing': [3, 3, 3], 'label': 1, 'normalizeScale': 1, 'normalize': False, 'force2Ddimension': 0, 'removeOutliers': None, 'minimumROISi
{'distances': [1], 'verbose': True, 'additionalInfo': True, 'enableCExtensions': True, 'force2D': False, 'interpolator': 3, 'resampledPixelSpacing': [3, 3, 3], 'label': 1, 'normalizeScale': 1, 'normalize': False, 'force2Ddimension': 0, 'removeOutliers': None, 'minimumROISi
{'distances': [1], 'verbose': True, 'additionalInfo': True, 'enableCExtensions': True, 'force2D': False, 'interpolator': 3, 'resampledPixelSpacing': [3, 3, 3], 'label': 1, 'normalizeScale': 1, 'normalize': False, 'force2Ddimension': 0, 'removeOutliers': None, 'minimumROISi
{'distances': [1], 'verbose': True, 'additionalInfo': True, 'enableCExtensions': True, 'force2D': False, 'interpolator': 3, 'resampledPixelSpacing': [3, 3, 3], 'label': 1, 'normalizeScale': 1, 'normalize': False, 'force2Ddimension': 0, 'removeOutliers': None, 'minimumROISi
{'distances': [1], 'verbose': True, 'additionalInfo': True, 'enableCExtensions': True, 'force2D': False, 'interpolator': 3, 'resampledPixelSpacing': [3, 3, 3], 'label': 1, 'normalizeScale': 1, 'normalize': False, 'force2Ddimension': 0, 'removeOutliers': None, 'minimumROISi
{'distances': [1], 'verbose': True, 'additionalInfo': True, 'enableCExtensions': True, 'force2D': False, 'interpolator': 3, 'resampledPixelSpacing': [3, 3, 3], 'label': 1, 'normalizeScale': 1, 'normalize': False, 'force2Ddimension': 0, 'removeOutliers': None, 'minimumROISi
{'distances': [1], 'verbose': True, 'additionalInfo': True, 'enableCExtensions': True, 'force2D': False, 'interpolator': 3, 'resampledPixelSpacing': [3, 3, 3], 'label': 1, 'normalizeScale': 1, 'normalize': False, 'force2Ddimension': 0, 'removeOutliers': None, 'minimumROISi
{'distances': [1], 'verbose': True, 'additionalInfo': True, 'enableCExtensions': True, 'force2D': False, 'interpolator': 3, 'resampledPixelSpacing': [3, 3, 3], 'label': 1, 'normalizeScale': 1, 'normalize': False, 'force2Ddimension': 0, 'removeOutliers': None, 'minimumROISi
{'distances': [1], 'verbose': True, 'additionalInfo': True, 'enableCExtensions': True, 'force2D': False, 'interpolator': 3, 'resampledPixelSpacing': [3, 3, 3], 'label': 1, 'normalizeScale': 1, 'normalize': False, 'force2Ddimension': 0, 'removeOutliers': None, 'minimumROISi
{'distances': [1], 'verbose': True, 'additionalInfo': True, 'enableCExtensions': True, 'force2D': False, 'interpolator': 3, 'resampledPixelSpacing': [3, 3, 3], 'label': 1, 'normalizeScale': 1, 'normalize': False, 'force2Ddimension': 0, 'removeOutliers': None, 'minimumROISi
{'distances': [1], 'verbose': True, 'additionalInfo': True, 'enableCExtensions': True, 'force2D': False, 'interpolator': 3, 'resampledPixelSpacing': [3, 3, 3], 'label': 1, 'normalizeScale': 1, 'normalize': False, 'force2Ddimension': 0, 'removeOutliers': None, 'minimumROISi
{'distances': [1], 'verbose': True, 'additionalInfo': True, 'enableCExtensions': True, 'force2D': False, 'interpolator': 3, 'resampledPixelSpacing': [3, 3, 3], 'label': 1, 'normalizeScale': 1, 'normalize': False, 'force2Ddimension': 0, 'removeOutliers': None, 'minimumROISi
{'distances': [1], 'verbose': True, 'additionalInfo': True, 'enableCExtensions': True, 'force2D': False, 'interpolator': 3, 'resampledPixelSpacing': [3, 3, 3], 'label': 1, 'normalizeScale': 1, 'normalize': False, 'force2Ddimension': 0, 'removeOutliers': None, 'minimumROISi
{'distances': [1], 'verbose': True, 'additionalInfo': True, 'enableCExtensions': True, 'force2D': False, 'interpolator': 3, 'resampledPixelSpacing': [3, 3, 3], 'label': 1, 'normalizeScale': 1, 'normalize': False, 'force2Ddimension': 0, 'removeOutliers': None, 'minimumROISi
{'distances': [1], 'verbose': True, 'additionalInfo': True, 'enableCExtensions': True, 'force2D': False, 'interpolator': 3, 'resampledPixelSpacing': [3, 3, 3], 'label': 1, 'normalizeScale': 1, 'normalize': False, 'force2Ddimension': 0, 'removeOutliers': None, 'minimumROISi
{'distances': [1], 'verbose': True, 'additionalInfo': True, 'enableCExtensions': True, 'force2D': False, 'interpolator': 3, 'resampledPixelSpacing': [3, 3, 3], 'label': 1, 'normalizeScale': 1, 'normalize': False, 'force2Ddimension': 0, 'removeOutliers': None, 'minimumROISi
{'distances': [1], 'verbose': True, 'additionalInfo': True, 'enableCExtensions': True, 'force2D': False, 'interpolator': 3, 'resampledPixelSpacing': [3, 3, 3], 'label': 1, 'normalizeScale': 1, 'normalize': False, 'force2Ddimension': 0, 'removeOutliers': None, 'minimumROISi
{'distances': [1], 'verbose': True, 'additionalInfo': True, 'enableCExtensions': True, 'force2D': False, 'interpolator': 3, 'resampledPixelSpacing': [3, 3, 3], 'label': 1, 'normalizeScale': 1, 'normalize': False, 'force2Ddimension': 0, 'removeOutliers': None, 'minimumROISi
{'distances': [1], 'verbose': True, 'additionalInfo': True, 'enableCExtensions': True, 'force2D': False, 'interpolator': 3, 'resampledPixelSpacing': [3, 3, 3], 'label': 1, 'normalizeScale': 1, 'normalize': False, 'force2Ddimension': 0, 'removeOutliers': None, 'minimumROISi
{'distances': [1], 'verbose': True, 'additionalInfo': True, 'enableCExtensions': True, 'force2D': False, 'interpolator': 3, 'resampledPixelSpacing': [3, 3, 3], 'label': 1, 'normalizeScale': 1, 'normalize': False, 'force2Ddimension': 0, 'removeOutliers': None, 'minimumROISi
{'distances': [1], 'verbose': True, 'additionalInfo': True, 'enableCExtensions': True, 'force2D': False, 'interpolator': 3, 'resampledPixelSpacing': [3, 3, 3], 'label': 1, 'normalizeScale': 1, 'normalize': False, 'force2Ddimension': 0, 'removeOutliers': None, 'minimumROISi
{'distances': [1], 'verbose': True, 'additionalInfo': True, 'enableCExtensions': True, 'force2D': False, 'interpolator': 3, 'resampledPixelSpacing': [3, 3, 3], 'label': 1, 'normalizeScale': 1, 'normalize': False, 'force2Ddimension': 0, 'removeOutliers': None, 'minimumROISi
{'distances': [1], 'verbose': True, 'additionalInfo': True, 'enableCExtensions': True, 'force2D': False, 'interpolator': 3, 'resampledPixelSpacing': [3, 3, 3], 'label': 1, 'normalizeScale': 1, 'normalize': False, 'force2Ddimension': 0, 'removeOutliers': None, 'minimumROISi
{'distances': [1], 'verbose': True, 'additionalInfo': True, 'enableCExtensions': True, 'force2D': False, 'interpolator': 3, 'resampledPixelSpacing': [3, 3, 3], 'label': 1, 'normalizeScale': 1, 'normalize': False, 'force2Ddimension': 0, 'removeOutliers': None, 'minimumROISi
{'distances': [1], 'verbose': True, 'additionalInfo': True, 'enableCExtensions': True, 'force2D': False, 'interpolator': 3, 'resampledPixelSpacing': [3, 3, 3], 'label': 1, 'normalizeScale': 1, 'normalize': False, 'force2Ddimension': 0, 'removeOutliers': None, 'minimumROISi
{'distances': [1], 'verbose': True, 'additionalInfo': True, 'enableCExtensions': True, 'force2D': False, 'interpolator': 3, 'resampledPixelSpacing': [3, 3, 3], 'label': 1, 'normalizeScale': 1, 'normalize': False, 'force2Ddimension': 0, 'removeOutliers': None, 'minimumROISi
{'distances': [1], 'verbose': True, 'additionalInfo': True, 'enableCExtensions': True, 'force2D': False, 'interpolator': 3, 'resampledPixelSpacing': [3, 3, 3], 'label': 1, 'normalizeScale': 1, 'normalize': False, 'force2Ddimension': 0, 'removeOutliers': None, 'minimumROISi
{'distances': [1], 'verbose': True, 'additionalInfo': True, 'enableCExtensions': True, 'force2D': False, 'interpolator': 3, 'resampledPixelSpacing': [3, 3, 3], 'label': 1, 'normalizeScale': 1, 'normalize': False, 'force2Ddimension': 0, 'removeOutliers': None, 'minimumROISi
{'distances': [1], 'verbose': True, 'additionalInfo': True, 'enableCExtensions': True, 'force2D': False, 'interpolator': 3, 'resampledPixelSpacing': [3, 3, 3], 'label': 1, 'normalizeScale': 1, 'normalize': False, 'force2Ddimension': 0, 'removeOutliers': None, 'minimumROISi
{'distances': [1], 'verbose': True, 'additionalInfo': True, 'enableCExtensions': True, 'force2D': False, 'interpolator': 3, 'resampledPixelSpacing': [3, 3, 3], 'label': 1, 'normalizeScale': 1, 'normalize': False, 'force2Ddimension': 0, 'removeOutliers': None, 'minimumROISi
{'distances': [1], 'verbose': True, 'additionalInfo': True, 'enableCExtensions': True, 'force2D': False, 'interpolator': 3, 'resampledPixelSpacing': [3, 3, 3], 'label': 1, 'normalizeScale': 1, 'normalize': False, 'force2Ddimension': 0, 'removeOutliers': None, 'minimumROISi
{'distances': [1], 'verbose': True, 'additionalInfo': True, 'enableCExtensions': True, 'force2D': False, 'interpolator': 3, 'resampledPixelSpacing': [3, 3, 3], 'label': 1, 'normalizeScale': 1, 'normalize': False, 'force2Ddimension': 0, 'removeOutliers': None, 'minimumROISi
{'distances': [1], 'verbose': True, 'additionalInfo': True, 'enableCExtensions': True, 'force2D': False, 'interpolator': 3, 'resampledPixelSpacing': [3, 3, 3], 'label': 1, 'normalizeScale': 1, 'normalize': False, 'force2Ddimension': 0, 'removeOutliers': None, 'minimumROISi
{'distances': [1], 'verbose': True, 'additionalInfo': True, 'enableCExtensions': True, 'force2D': False, 'interpolator': 3, 'resampledPixelSpacing': [3, 3, 3], 'label': 1, 'normalizeScale': 1, 'normalize': False, 'force2Ddimension': 0, 'removeOutliers': None, 'minimumROISi
{'distances': [1], 'verbose': True, 'additionalInfo': True, 'enableCExtensions': True, 'force2D': False, 'interpolator': 3, 'resampledPixelSpacing': [3, 3, 3], 'label': 1, 'normalizeScale': 1, 'normalize': False, 'force2Ddimension': 0, 'removeOutliers': None, 'minimumROISi
{'distances': [1], 'verbose': True, 'additionalInfo': True, 'enableCExtensions': True, 'force2D': False, 'interpolator': 3, 'resampledPixelSpacing': [3, 3, 3], 'label': 1, 'normalizeScale': 1, 'normalize': False, 'force2Ddimension': 0, 'removeOutliers': None, 'minimumROISi
{'distances': [1], 'verbose': True, 'additionalInfo': True, 'enableCExtensions': True, 'force2D': False, 'interpolator': 3, 'resampledPixelSpacing': [3, 3, 3], 'label': 1, 'normalizeScale': 1, 'normalize': False, 'force2Ddimension': 0, 'removeOutliers': None, 'minimumROISi
{'distances': [1], 'verbose': True, 'additionalInfo': True, 'enableCExtensions': True, 'force2D': False, 'interpolator': 3, 'resampledPixelSpacing': [3, 3, 3], 'label': 1, 'normalizeScale': 1, 'normalize': False, 'force2Ddimension': 0, 'removeOutliers': None, 'minimumROISi
{'distances': [1], 'verbose': True, 'additionalInfo': True, 'enableCExtensions': True, 'force2D': False, 'interpolator': 3, 'resampledPixelSpacing': [3, 3, 3], 'label': 1, 'normalizeScale': 1, 'normalize': False, 'force2Ddimension': 0, 'removeOutliers': None, 'minimumROISi
{'distances': [1], 'verbose': True, 'additionalInfo': True, 'enableCExtensions': True, 'force2D': False, 'interpolator': 3, 'resampledPixelSpacing': [3, 3, 3], 'label': 1, 'normalizeScale': 1, 'normalize': False, 'force2Ddimension': 0, 'removeOutliers': None, 'minimumROISi
{'distances': [1], 'verbose': True, 'additionalInfo': True, 'enableCExtensions': True, 'force2D': False, 'interpolator': 3, 'resampledPixelSpacing': [3, 3, 3], 'label': 1, 'normalizeScale': 1, 'normalize': False, 'force2Ddimension': 0, 'removeOutliers': None, 'minimumROISi
{'distances': [1], 'verbose': True, 'additionalInfo': True, 'enableCExtensions': True, 'force2D': False, 'interpolator': 3, 'resampledPixelSpacing': [3, 3, 3], 'label': 1, 'normalizeScale': 1, 'normalize': False, 'force2Ddimension': 0, 'removeOutliers': None, 'minimumROISi
{'distances': [1], 'verbose': True, 'additionalInfo': True, 'enableCExtensions': True, 'force2D': False, 'interpolator': 3, 'resampledPixelSpacing': [3, 3, 3], 'label': 1, 'normalizeScale': 1, 'normalize': False, 'force2Ddimension': 0, 'removeOutliers': None, 'minimumROISi
{'distances': [1], 'verbose': True, 'additionalInfo': True, 'enableCExtensions': True, 'force2D': False, 'interpolator': 3, 'resampledPixelSpacing': [3, 3, 3], 'label': 1, 'normalizeScale': 1, 'normalize': False, 'force2Ddimension': 0, 'removeOutliers': None, 'minimumROISi
{'distances': [1], 'verbose': True, 'additionalInfo': True, 'enableCExtensions': True, 'force2D': False, 'interpolator': 3, 'resampledPixelSpacing': [3, 3, 3], 'label': 1, 'normalizeScale': 1, 'normalize': False, 'force2Ddimension': 0, 'removeOutliers': None, 'minimumROISi
{'distances': [1], 'verbose': True, 'additionalInfo': True, 'enableCExtensions': True, 'force2D': False, 'interpolator': 3, 'resampledPixelSpacing': [3, 3, 3], 'label': 1, 'normalizeScale': 1, 'normalize': False, 'force2Ddimension': 0, 'removeOutliers': None, 'minimumROISi
{'distances': [1], 'verbose': True, 'additionalInfo': True, 'enableCExtensions': True, 'force2D': False, 'interpolator': 3, 'resampledPixelSpacing': [3, 3, 3], 'label': 1, 'normalizeScale': 1, 'normalize': False, 'force2Ddimension': 0, 'removeOutliers': None, 'minimumROISi
{'distances': [1], 'verbose': True, 'additionalInfo': True, 'enableCExtensions': True, 'force2D': False, 'interpolator': 3, 'resampledPixelSpacing': [3, 3, 3], 'label': 1, 'normalizeScale': 1, 'normalize': False, 'force2Ddimension': 0, 'removeOutliers': None, 'minimumROISi
{'distances': [1], 'verbose': True, 'additionalInfo': True, 'enableCExtensions': True, 'force2D': False, 'interpolator': 3, 'resampledPixelSpacing': [3, 3, 3], 'label': 1, 'normalizeScale': 1, 'normalize': False, 'force2Ddimension': 0, 'removeOutliers': None, 'minimumROISi
{'distances': [1], 'verbose': True, 'additionalInfo': True, 'enableCExtensions': True, 'force2D': False, 'interpolator': 3, 'resampledPixelSpacing': [3, 3, 3], 'label': 1, 'normalizeScale': 1, 'normalize': False, 'force2Ddimension': 0, 'removeOutliers': None, 'minimumROISi
{'distances': [1], 'verbose': True, 'additionalInfo': True, 'enableCExtensions': True, 'force2D': False, 'interpolator': 3, 'resampledPixelSpacing': [3, 3, 3], 'label': 1, 'normalizeScale': 1, 'normalize': False, 'force2Ddimension': 0, 'removeOutliers': None, 'minimumROISi
{'distances': [1], 'verbose': True, 'additionalInfo': True, 'enableCExtensions': True, 'force2D': False, 'interpolator': 3, 'resampledPixelSpacing': [3, 3, 3], 'label': 1, 'normalizeScale': 1, 'normalize': False, 'force2Ddimension': 0, 'removeOutliers': None, 'minimumROISi
{'distances': [1], 'verbose': True, 'additionalInfo': True, 'enableCExtensions': True, 'force2D': False, 'interpolator': 3, 'resampledPixelSpacing': [3, 3, 3], 'label': 1, 'normalizeScale': 1, 'normalize': False, 'force2Ddimension': 0, 'removeOutliers': None, 'minimumROISi
{'distances': [1], 'verbose': True, 'additionalInfo': True, 'enableCExtensions': True, 'force2D': False, 'interpolator': 3, 'resampledPixelSpacing': [3, 3, 3], 'label': 1, 'normalizeScale': 1, 'normalize': False, 'force2Ddimension': 0, 'removeOutliers': None, 'minimumROISi
{'distances': [1], 'verbose': True, 'additionalInfo': True, 'enableCExtensions': True, 'force2D': False, 'interpolator': 3, 'resampledPixelSpacing': [3, 3, 3], 'label': 1, 'normalizeScale': 1, 'normalize': False, 'force2Ddimension': 0, 'removeOutliers': None, 'minimumROISi
{'distances': [1], 'verbose': True, 'additionalInfo': True, 'enableCExtensions': True, 'force2D': False, 'interpolator': 3, 'resampledPixelSpacing': [3, 3, 3], 'label': 1, 'normalizeScale': 1, 'normalize': False, 'force2Ddimension': 0, 'removeOutliers': None, 'minimumROISi
{'distances': [1], 'verbose': True, 'additionalInfo': True, 'enableCExtensions': True, 'force2D': False, 'interpolator': 3, 'resampledPixelSpacing': [3, 3, 3], 'label': 1, 'normalizeScale': 1, 'normalize': False, 'force2Ddimension': 0, 'removeOutliers': None, 'minimumROISi
{'distances': [1], 'verbose': True, 'additionalInfo': True, 'enableCExtensions': True, 'force2D': False, 'interpolator': 3, 'resampledPixelSpacing': [3, 3, 3], 'label': 1, 'normalizeScale': 1, 'normalize': False, 'force2Ddimension': 0, 'removeOutliers': None, 'minimumROISi
{'distances': [1], 'verbose': True, 'additionalInfo': True, 'enableCExtensions': True, 'force2D': False, 'interpolator': 3, 'resampledPixelSpacing': [3, 3, 3], 'label': 1, 'normalizeScale': 1, 'normalize': False, 'force2Ddimension': 0, 'removeOutliers': None, 'minimumROISi
{'distances': [1], 'verbose': True, 'additionalInfo': True, 'enableCExtensions': True, 'force2D': False, 'interpolator': 3, 'resampledPixelSpacing': [3, 3, 3], 'label': 1, 'normalizeScale': 1, 'normalize': False, 'force2Ddimension': 0, 'removeOutliers': None, 'minimumROISi
{'distances': [1], 'verbose': True, 'additionalInfo': True, 'enableCExtensions': True, 'force2D': False, 'interpolator': 3, 'resampledPixelSpacing': [3, 3, 3], 'label': 1, 'normalizeScale': 1, 'normalize': False, 'force2Ddimension': 0, 'removeOutliers': None, 'minimumROISi
{'distances': [1], 'verbose': True, 'additionalInfo': True, 'enableCExtensions': True, 'force2D': False, 'interpolator': 3, 'resampledPixelSpacing': [3, 3, 3], 'label': 1, 'normalizeScale': 1, 'normalize': False, 'force2Ddimension': 0, 'removeOutliers': None, 'minimumROISi
{'distances': [1], 'verbose': True, 'additionalInfo': True, 'enableCExtensions': True, 'force2D': False, 'interpolator': 3, 'resampledPixelSpacing': [3, 3, 3], 'label': 1, 'normalizeScale': 1, 'normalize': False, 'force2Ddimension': 0, 'removeOutliers': None, 'minimumROISi
{'distances': [1], 'verbose': True, 'additionalInfo': True, 'enableCExtensions': True, 'force2D': False, 'interpolator': 3, 'resampledPixelSpacing': [3, 3, 3], 'label': 1, 'normalizeScale': 1, 'normalize': False, 'force2Ddimension': 0, 'removeOutliers': None, 'minimumROISi
{'distances': [1], 'verbose': True, 'additionalInfo': True, 'enableCExtensions': True, 'force2D': False, 'interpolator': 3, 'resampledPixelSpacing': [3, 3, 3], 'label': 1, 'normalizeScale': 1, 'normalize': False, 'force2Ddimension': 0, 'removeOutliers': None, 'minimumROISi
{'distances': [1], 'verbose': True, 'additionalInfo': True, 'enableCExtensions': True, 'force2D': False, 'interpolator': 3, 'resampledPixelSpacing': [3, 3, 3], 'label': 1, 'normalizeScale': 1, 'normalize': False, 'force2Ddimension': 0, 'removeOutliers': None, 'minimumROISi
{'distances': [1], 'verbose': True, 'additionalInfo': True, 'enableCExtensions': True, 'force2D': False, 'interpolator': 3, 'resampledPixelSpacing': [3, 3, 3], 'label': 1, 'normalizeScale': 1, 'normalize': False, 'force2Ddimension': 0, 'removeOutliers': None, 'minimumROISi
{'distances': [1], 'verbose': True, 'additionalInfo': True, 'enableCExtensions': True, 'force2D': False, 'interpolator': 3, 'resampledPixelSpacing': [3, 3, 3], 'label': 1, 'normalizeScale': 1, 'normalize': False, 'force2Ddimension': 0, 'removeOutliers': None, 'minimumROISi
{'distances': [1], 'verbose': True, 'additionalInfo': True, 'enableCExtensions': True, 'force2D': False, 'interpolator': 3, 'resampledPixelSpacing': [3, 3, 3], 'label': 1, 'normalizeScale': 1, 'normalize': False, 'force2Ddimension': 0, 'removeOutliers': None, 'minimumROISi
{'distances': [1], 'verbose': True, 'additionalInfo': True, 'enableCExtensions': True, 'force2D': False, 'interpolator': 3, 'resampledPixelSpacing': [3, 3, 3], 'label': 1, 'normalizeScale': 1, 'normalize': False, 'force2Ddimension': 0, 'removeOutliers': None, 'minimumROISi
{'distances': [1], 'verbose': True, 'additionalInfo': True, 'enableCExtensions': True, 'force2D': False, 'interpolator': 3, 'resampledPixelSpacing': [3, 3, 3], 'label': 1, 'normalizeScale': 1, 'normalize': False, 'force2Ddimension': 0, 'removeOutliers': None, 'minimumROISi
{'distances': [1], 'verbose': True, 'additionalInfo': True, 'enableCExtensions': True, 'force2D': False, 'interpolator': 3, 'resampledPixelSpacing': [3, 3, 3], 'label': 1, 'normalizeScale': 1, 'normalize': False, 'force2Ddimension': 0, 'removeOutliers': None, 'minimumROISi
{'distances': [1], 'verbose': True, 'additionalInfo': True, 'enableCExtensions': True, 'force2D': False, 'interpolator': 3, 'resampledPixelSpacing': [3, 3, 3], 'label': 1, 'normalizeScale': 1, 'normalize': False, 'force2Ddimension': 0, 'removeOutliers': None, 'minimumROISi
{'distances': [1], 'verbose': True, 'additionalInfo': True, 'enableCExtensions': True, 'force2D': False, 'interpolator': 3, 'resampledPixelSpacing': [3, 3, 3], 'label': 1, 'normalizeScale': 1, 'normalize': False, 'force2Ddimension': 0, 'removeOutliers': None, 'minimumROISi
{'distances': [1], 'verbose': True, 'additionalInfo': True, 'enableCExtensions': True, 'force2D': False, 'interpolator': 3, 'resampledPixelSpacing': [3, 3, 3], 'label': 1, 'normalizeScale': 1, 'normalize': False, 'force2Ddimension': 0, 'removeOutliers': None, 'minimumROISi
{'distances': [1], 'verbose': True, 'additionalInfo': True, 'enableCExtensions': True, 'force2D': False, 'interpolator': 3, 'resampledPixelSpacing': [3, 3, 3], 'label': 1, 'normalizeScale': 1, 'normalize': False, 'force2Ddimension': 0, 'removeOutliers': None, 'minimumROISi
{'distances': [1], 'verbose': True, 'additionalInfo': True, 'enableCExtensions': True, 'force2D': False, 'interpolator': 3, 'resampledPixelSpacing': [3, 3, 3], 'label': 1, 'normalizeScale': 1, 'normalize': False, 'force2Ddimension': 0, 'removeOutliers': None, 'minimumROISi
{'distances': [1], 'verbose': True, 'additionalInfo': True, 'enableCExtensions': True, 'force2D': False, 'interpolator': 3, 'resampledPixelSpacing': [3, 3, 3], 'label': 1, 'normalizeScale': 1, 'normalize': False, 'force2Ddimension': 0, 'removeOutliers': None, 'minimumROISi
{'distances': [1], 'verbose': True, 'additionalInfo': True, 'enableCExtensions': True, 'force2D': False, 'interpolator': 3, 'resampledPixelSpacing': [3, 3, 3], 'label': 1, 'normalizeScale': 1, 'normalize': False, 'force2Ddimension': 0, 'removeOutliers': None, 'minimumROISi
{'distances': [1], 'verbose': True, 'additionalInfo': True, 'enableCExtensions': True, 'force2D': False, 'interpolator': 3, 'resampledPixelSpacing': [3, 3, 3], 'label': 1, 'normalizeScale': 1, 'normalize': False, 'force2Ddimension': 0, 'removeOutliers': None, 'minimumROISi
{'distances': [1], 'verbose': True, 'additionalInfo': True, 'enableCExtensions': True, 'force2D': False, 'interpolator': 3, 'resampledPixelSpacing': [3, 3, 3], 'label': 1, 'normalizeScale': 1, 'normalize': False, 'force2Ddimension': 0, 'removeOutliers': None, 'minimumROISi
{'distances': [1], 'verbose': True, 'additionalInfo': True, 'enableCExtensions': True, 'force2D': False, 'interpolator': 3, 'resampledPixelSpacing': [3, 3, 3], 'label': 1, 'normalizeScale': 1, 'normalize': False, 'force2Ddimension': 0, 'removeOutliers': None, 'minimumROISi
{'distances': [1], 'verbose': True, 'additionalInfo': True, 'enableCExtensions': True, 'force2D': False, 'interpolator': 3, 'resampledPixelSpacing': [3, 3, 3], 'label': 1, 'normalizeScale': 1, 'normalize': False, 'force2Ddimension': 0, 'removeOutliers': None, 'minimumROISi
{'distances': [1], 'verbose': True, 'additionalInfo': True, 'enableCExtensions': True, 'force2D': False, 'interpolator': 3, 'resampledPixelSpacing': [3, 3, 3], 'label': 1, 'normalizeScale': 1, 'normalize': False, 'force2Ddimension': 0, 'removeOutliers': None, 'minimumROISi
{'distances': [1], 'verbose': True, 'additionalInfo': True, 'enableCExtensions': True, 'force2D': False, 'interpolator': 3, 'resampledPixelSpacing': [3, 3, 3], 'label': 1, 'normalizeScale': 1, 'normalize': False, 'force2Ddimension': 0, 'removeOutliers': None, 'minimumROISi

general_info_ImageHash	general_info_ImageSpacing	general_info_InputImages	general_info_MaskHash	general_info_Version	general_info_VolumeNum
7d71e808fe6671534a3019b7be487ba8a0f98390	(1.0, 1.0, 1.0)	{'LoG': {}, 'Original': {}, 'Wavelet': {}}	b579ce2620b5a51e83afa8f0fe926b517e8e1c29	1.1.1-beta1	1
acd8228c36628cbfaefc90db997bb9ba7b57d5b7	(1.0, 1.0, 1.0)	{'LoG': {}, 'Original': {}, 'Wavelet': {}}	a5975900f7d36f6010c68a8a665208388946894e	1.1.1-beta1	1
a7529fcb4f8c9ad8d0d680fb4c32473100b7d25e	(1.0, 1.0, 1.0)	{'LoG': {}, 'Original': {}, 'Wavelet': {}}	d2bbdc2855bd6bec7d5c31e2f64ef0c3ff7c64bf	1.1.1-beta1	1
dbc259ac4a070f69d98a1a383291a01e3a986bc5	(1.0, 1.0, 1.0)	{'LoG': {}, 'Original': {}, 'Wavelet': {}}	eb4e12bcdf3564db96dda237a7f6cc3e73f93ca6	1.1.1-beta1	1
89de885ea7aa7e2148a38702fb9ea9a34295bb7a	(1.0, 1.0, 1.0)	{'LoG': {}, 'Original': {}, 'Wavelet': {}}	34167b00ac532b95d6477ad7b25c874242418556	1.1.1-beta1	1
60fced09f191524b644aded04cb0252ee2b6fc2b	(1.0, 1.0, 1.0)	{'LoG': {}, 'Original': {}, 'Wavelet': {}}	5742109a4734bfa2ce866f6593d05833fb67ed6a	1.1.1-beta1	1
4995f6ecbf9ab9416d2b5b690525de6bf6f58dd9	(1.0, 1.0, 1.0)	{'LoG': {}, 'Original': {}, 'Wavelet': {}}	2593c96b591c4ab182eadb07e267ab1e3caffbee	1.1.1-beta1	1
cd5f1a7045e07876b5e7fb747ce134ffc613ac6b	(1.0, 1.0, 1.0)	{'LoG': {}, 'Original': {}, 'Wavelet': {}}	042f27a1df65a72a6b184ee9da4a8286e1c600ea	1.1.1-beta1	1
de70f76ee5c3c953e9b5710409ec3282e7258cde	(1.0, 1.0, 1.0)	{'LoG': {}, 'Original': {}, 'Wavelet': {}}	db80b7b9ab80301978c693cd40fe86b0a7ce34cd	1.1.1-beta1	1
be3af75292a035c2daeed04f83ce4a3e84e6f871	(1.0, 1.0, 1.0)	{'LoG': {}, 'Original': {}, 'Wavelet': {}}	e9736a26ad8519c28ec825d335f10c20887a4c63	1.1.1-beta1	1
778f4b4a8ea6a18c8da5046951d7aed66d34a6b2	(1.0, 1.0, 1.0)	{'LoG': {}, 'Original': {}, 'Wavelet': {}}	5a26482df4f38b1ecb569ce43dc4af3c14d02e7f	1.1.1-beta1	1
43820772bb86565ffd947ea76a7e037d208aa784	(1.0, 1.0, 1.0)	{'LoG': {}, 'Original': {}, 'Wavelet': {}}	45a9a94841e06fc1c200f857b8c7740f9cac18f8	1.1.1-beta1	1
5ae037077e6ef4bfb56a0af8bd4e0f4ba3c34c57	(1.0, 1.0, 1.0)	{'LoG': {}, 'Original': {}, 'Wavelet': {}}	f02ad611ade01763dccc048f05fccea90afed981	1.1.1-beta1	1
4824fd3555f87f78f06c42bcaece5153075edba7	(1.0, 1.0, 1.0)	{'LoG': {}, 'Original': {}, 'Wavelet': {}}	138d0cde4b499852ee925989a1a733ca4eda7f26	1.1.1-beta1	1
069fd1be988755e457288f8d74b75c3c15fefbc4	(1.0, 1.0, 1.0)	{'LoG': {}, 'Original': {}, 'Wavelet': {}}	0d953f76a06a805d5170e319a171a13bf539d746	1.1.1-beta1	1
cc30fa586f37ea761ac29cb87ac70d41ad928a62	(1.0, 1.0, 0.9999999999999999)	{'LoG': {}, 'Original': {}, 'Wavelet': {}}	6a72ae648acb4067883e429b9d8149ad00aacf0a	1.1.1-beta1	1
24235a029862f96ddac62459ec91f1189d5dc2d9	(1.0, 1.0, 1.0)	{'LoG': {}, 'Original': {}, 'Wavelet': {}}	8ca99f426c7b9938084e1e4f69da35c4e58342c1	1.1.1-beta1	1
95880cb500e05e8a3cd7fd96ee5264dcb1e450d9	(1.0, 1.0, 0.9999999999999999)	{'LoG': {}, 'Original': {}, 'Wavelet': {}}	891d47a4d0ab10d5183fe2a7b881cf21b1a13b84	1.1.1-beta1	1
d02f4eeadf32f7bc50c30c0d58b7691f5080827b	(1.0, 1.0, 1.0)	{'LoG': {}, 'Original': {}, 'Wavelet': {}}	edcdf23dec215aac032d36adc4cf183c7002cbe0	1.1.1-beta1	1
1ba33fcb87a615e09a14f5560918e3602925af8c	(1.0, 1.0, 1.0)	{'LoG': {}, 'Original': {}, 'Wavelet': {}}	6354a12e6d9c2e7d94617b95a198e3b7d56f79c5	1.1.1-beta1	1
56955aa6d8ad23566e6500c3745a0238a7b67410	(1.0, 1.0, 1.0)	{'LoG': {}, 'Original': {}, 'Wavelet': {}}	90c0c6e21fb9348bb10e518011861672340904a5	1.1.1-beta1	2
78c291ea9c332118a69a05e48f1c08b14d5e7c7f	(1.0, 1.0, 0.9999999999999999)	{'LoG': {}, 'Original': {}, 'Wavelet': {}}	974bf31f6802f25808abe8f51881410b0a3cb8df	1.1.1-beta1	1
03fd1116f7ebbc9b0236bacd710d54e70f43222b	(1.0, 1.0, 1.0)	{'LoG': {}, 'Original': {}, 'Wavelet': {}}	743573c372c12aa8793bfa3bc5e553961b813cd8	1.1.1-beta1	1
e83cabcb67c6acdba6290c6523c55038a9d3bec9	(1.0, 1.0, 1.0)	{'LoG': {}, 'Original': {}, 'Wavelet': {}}	803bc25fd5600065216b8039ec98cf900994ba97	1.1.1-beta1	1
bab90b53bb641677fc25b517e30253e88e6c1551	(1.0, 1.0, 0.9999999999999999)	{'LoG': {}, 'Original': {}, 'Wavelet': {}}	6a1facbc81d275a99a1a9bef9c304e72901bf067	1.1.1-beta1	1
331a674ae99f13aad88c4d4d11ba8a24a766d352	(1.0, 1.0, 1.0)	{'LoG': {}, 'Original': {}, 'Wavelet': {}}	3d814518e405e8247c43cc4c0a534bd8dea98e34	1.1.1-beta1	1
df35a3918b83254f64f08cd4d9c7ea699dc12827	(1.0, 1.0, 1.0)	{'LoG': {}, 'Original': {}, 'Wavelet': {}}	b2bbfda99f141dbf72b9340bfc65161be90ab6d5	1.1.1-beta1	1
90a2432541c8208da8e51d3eed094103df5926b0	(1.0, 1.0, 1.0)	{'LoG': {}, 'Original': {}, 'Wavelet': {}}	5a8f23fe8ee692fbdc7ddc3bf403a8d27d301609	1.1.1-beta1	1
1b368f9f9427ad60615923d9ece22534d031e675	(1.0, 1.0, 1.0)	{'LoG': {}, 'Original': {}, 'Wavelet': {}}	45301f461688accbf71c77c37ea67ef26b169135	1.1.1-beta1	1
4b5e7d8d777c8fcef9804e75c9fc5e48ab1a0412	(1.0, 1.0, 1.0)	{'LoG': {}, 'Original': {}, 'Wavelet': {}}	70552138bb1f394629472a3b009f87dc47194054	1.1.1-beta1	1
86c04aea820ab661dfd5c877cbf21e4cf8defd7d	(1.0, 1.0, 1.0)	{'LoG': {}, 'Original': {}, 'Wavelet': {}}	64fc6b9f0cb86b0b3feba0c3e2bb4c7b3f9e5438	1.1.1-beta1	4
3bc54655740cee2499184a8461d0ed43db11a9ac	(1.0, 1.0, 1.0)	{'LoG': {}, 'Original': {}, 'Wavelet': {}}	b47dc5e284abdafa2b99430823183e777f62a360	1.1.1-beta1	2
bd7e8746c1d829d9a88e7d61e8e554f2b32f7740	(1.0, 1.0, 1.0)	{'LoG': {}, 'Original': {}, 'Wavelet': {}}	a80a36b48ca65d41a52797ff1324b0430e9f1630	1.1.1-beta1	1
f3c7c875021eddbd98b64055ac1af6012a53427c	(1.0, 1.0, 1.0)	{'LoG': {}, 'Original': {}, 'Wavelet': {}}	e124d8fe4e866481864295e07c198ec1565af769	1.1.1-beta1	1
b843096e4fc13a50b9dd6fd33ac8111282f33a52	(1.0, 1.0, 1.0)	{'LoG': {}, 'Original': {}, 'Wavelet': {}}	28cf76b2791992cbc9403c53fe01b407d39c5328	1.1.1-beta1	1
62f24f52dfe1b55bef588eae84ed26a62bec8c90	(1.0, 1.0, 1.0)	{'LoG': {}, 'Original': {}, 'Wavelet': {}}	954484c5519bf22702bc0ea0f7b46ce866bdbfcf	1.1.1-beta1	1
001216bee1af2b3881edcf5e01759d697a0150ff	(1.0, 1.0, 1.0)	{'LoG': {}, 'Original': {}, 'Wavelet': {}}	a0cbf7ddf967169fb7ca62ad3091c802843d16eb	1.1.1-beta1	1
425d9499496351afcb3b43d88e01590e34770cc2	(1.0, 1.0, 1.0)	{'LoG': {}, 'Original': {}, 'Wavelet': {}}	f847f68475356e7d70bfdd5c26912f0b332e37d0	1.1.1-beta1	1
e5b1250a787ca1ed95ef710f82c70cfe8b39a0b0	(1.0, 1.0, 1.0)	{'LoG': {}, 'Original': {}, 'Wavelet': {}}	35b90ab5ff9c3e12b87adf9eb98d76607e10ae74	1.1.1-beta1	1
92005832804dbdf44d0453ea2aaf4b3be7cf95ee	(1.0, 1.0, 1.0)	{'LoG': {}, 'Original': {}, 'Wavelet': {}}	3a8ff3001abf6ebb6260780e40708c218dcd054f	1.1.1-beta1	1
2c3510223208746f011c6cc80d234d225f19eb6c	(1.0, 1.0, 1.0)	{'LoG': {}, 'Original': {}, 'Wavelet': {}}	a46a391f828a88c9f9c1e71cb488a4e6e3813b23	1.1.1-beta1	1
7b9e96024ec8928dc40f07539fda7467232b1db0	(1.0, 1.0, 1.0)	{'LoG': {}, 'Original': {}, 'Wavelet': {}}	4152d71d11525284cf259f571ab331094fd9410a	1.1.1-beta1	1
f1a89c80faf8e38162c556eca91776d271727649	(1.0, 1.0, 1.0)	{'LoG': {}, 'Original': {}, 'Wavelet': {}}	e5e881b054cfa11b0cf0cd9c3004764a41a64d5f	1.1.1-beta1	1
ecf3abdee7a7aa29590029351da7e80de30b5748	(1.0, 1.0, 1.0)	{'LoG': {}, 'Original': {}, 'Wavelet': {}}	e345e94e6b53c2b832860aa6dfeb1461986f89cf	1.1.1-beta1	1
7e56a6fa7582b1efd41526d493b9dd57779f00b0	(1.0, 1.0, 1.0)	{'LoG': {}, 'Original': {}, 'Wavelet': {}}	3c1e404ac0df34823f61e306a92e823e3bf11792	1.1.1-beta1	1
124810c6a78ec91138f06ece964dbe2c6aaa17fe	(1.0, 1.0, 1.0)	{'LoG': {}, 'Original': {}, 'Wavelet': {}}	23e0f34b41e6b7ee066a176633f04eb9404fb640	1.1.1-beta1	2
f60bb3e1c364756a8ccbe1d8f9fc0855f7ccd1f6	(1.0, 1.0, 1.0)	{'LoG': {}, 'Original': {}, 'Wavelet': {}}	7abbb0cc73c5e5af35f333addbe1e47ff4495cad	1.1.1-beta1	1
6a36618ca2f75a85a5128fc656f10e6180d8cc57	(1.0, 1.0, 1.0)	{'LoG': {}, 'Original': {}, 'Wavelet': {}}	20955bcc6010d0907fab2f923ff57bb28c6b4b3d	1.1.1-beta1	1
08940100339b89405caae24ed3babf29aa4c22bb	(1.0, 1.0, 1.0)	{'LoG': {}, 'Original': {}, 'Wavelet': {}}	d241d9929f0077cd759afe106de146d493e0e66c	1.1.1-beta1	1
39ccd562aa9d39222569d219fb2eb8003df0d30c	(1.0, 1.0, 1.0)	{'LoG': {}, 'Original': {}, 'Wavelet': {}}	50c595ec862717a93846ea6c187689fa885f4c03	1.1.1-beta1	1
2c2efec1dbcd2cd1429b1960dadedb922e5aa603	(1.0, 1.0, 1.0)	{'LoG': {}, 'Original': {}, 'Wavelet': {}}	c3f733fbd58fdbe2dd22a242c4b62eadf0b11acf	1.1.1-beta1	1
ff839a7865e4a66e9ed3f151a45992caa8c43eb4	(1.0, 1.0, 1.0)	{'LoG': {}, 'Original': {}, 'Wavelet': {}}	5289f06e0dfcec8d1ea53db339fff94ef70060db	1.1.1-beta1	1
40b3697fef4a5dc9de3a9bc4bb29356337cf24da	(1.0, 1.0, 1.0)	{'LoG': {}, 'Original': {}, 'Wavelet': {}}	2fdbfd177adb72cc737f39750db736695b89bf45	1.1.1-beta1	1
8b1df46b0cfc61193922dedcc4296bb21c2e773c	(1.0, 1.0, 1.0)	{'LoG': {}, 'Original': {}, 'Wavelet': {}}	1190253fc53ec3d7875534f4438ca86a7f38a56b	1.1.1-beta1	1
303aa75ad2b720c7d7329d77466a4c9967dcdd29	(1.0, 1.0, 1.0)	{'LoG': {}, 'Original': {}, 'Wavelet': {}}	b023b8a9650d14b3a8ea9865b7088f78bc8000be	1.1.1-beta1	1
dcd6cede20943bb052f20e3f05664f6ce47536a3	(1.0, 1.0, 1.0)	{'LoG': {}, 'Original': {}, 'Wavelet': {}}	420dea5b6b276dbff82549724b82368837ea0757	1.1.1-beta1	1
882c441365cfbede7ef6456bba09d3eb7759da54	(1.0, 1.0, 1.0)	{'LoG': {}, 'Original': {}, 'Wavelet': {}}	468983939177a3f02da4100316c42e5e04eac1ff	1.1.1-beta1	1
082856f2a12425cee867d722afa3b93a8d1c8f30	(1.0, 1.0, 1.0)	{'LoG': {}, 'Original': {}, 'Wavelet': {}}	2b99e6a308e989696996604f27d745c5672df1c0	1.1.1-beta1	1
0f850fec7bb5bc5714a52d63844862f8e428496d	(1.0, 1.0, 1.0)	{'LoG': {}, 'Original': {}, 'Wavelet': {}}	282763d9a4f3e641601708d88380a501c49abb99	1.1.1-beta1	1
425351daad40d6a5866008f88407c9c301ab9d74	(1.0, 1.0, 1.0)	{'LoG': {}, 'Original': {}, 'Wavelet': {}}	553cbbbfdc1b40aa3be288e8bb00a0935327803d	1.1.1-beta1	1
286477e11a09101dd019b73125ded81c1fb2f575	(1.0, 1.0, 1.0)	{'LoG': {}, 'Original': {}, 'Wavelet': {}}	db101623aafe726602c1ba8ad335e240630f1ba8	1.1.1-beta1	1
05028e3b7ffbbe3f7d7095782ca680d0b92bd767	(1.0, 1.0, 1.0)	{'LoG': {}, 'Original': {}, 'Wavelet': {}}	46411ad24ce74d2521c1a18b99aa5e3d34b9a861	1.1.1-beta1	1
a1ef95f7dc3da464757d168b8b06989cff9fa3d9	(1.0, 1.0, 1.0)	{'LoG': {}, 'Original': {}, 'Wavelet': {}}	8d65b00f147d030cd48f3b1c785d5ec84aaaa97f	1.1.1-beta1	1
53e9cc2d3a433a1c0d3f6efa685675ffdc80dd24	(1.0, 1.0, 1.0)	{'LoG': {}, 'Original': {}, 'Wavelet': {}}	185558ad98bd35afb222db42bee6ea0625467d23	1.1.1-beta1	1
99adb35d859ae1c7f7c50bfcdd3d64a7cbf59bf0	(1.0, 1.0, 1.0)	{'LoG': {}, 'Original': {}, 'Wavelet': {}}	4f0dabca38f7a05298fd5970078e522f86f90e21	1.1.1-beta1	1
bec1de8d9329b8187163106b831361859cd7bf20	(1.0, 1.0, 1.0)	{'LoG': {}, 'Original': {}, 'Wavelet': {}}	c5ed7a46f168f5f5f714a0a30746d2b960be188a	1.1.1-beta1	1
2ca04a708c5b413bac22cde7644c886624834bf3	(1.0, 1.0, 1.0)	{'LoG': {}, 'Original': {}, 'Wavelet': {}}	b3ddc3e475d550675b36ab19295d357ccc04d498	1.1.1-beta1	1
68faced8e8d9f36b5bf826127fc1ef66dc61dcfc	(1.0, 1.0, 1.0)	{'LoG': {}, 'Original': {}, 'Wavelet': {}}	5db1e6b9ecc796887505ebe993892fabfcf325f8	1.1.1-beta1	1
5627858df0e21252bee88b75a293758b47022e9a	(1.0, 1.0, 1.0)	{'LoG': {}, 'Original': {}, 'Wavelet': {}}	12b228f5f66531ad25b1c7d0560ff5c6afae9c16	1.1.1-beta1	1
8d140ccfe8a2e02737dd8387970765181737d54f	(1.0, 1.0, 1.0)	{'LoG': {}, 'Original': {}, 'Wavelet': {}}	e2e2330360a2a6cb3e8fd397014649e7be7183d3	1.1.1-beta1	1
f8fcbac275778cd107756269b7306710466567cc	(1.0, 1.0, 1.0)	{'LoG': {}, 'Original': {}, 'Wavelet': {}}	33e5dbb0862b482ea548803a6916ad3c5118bb4f	1.1.1-beta1	1
ceb6b308f0598622f4c495c05a54895ecfa308f4	(1.0, 1.0, 1.0)	{'LoG': {}, 'Original': {}, 'Wavelet': {}}	9199df64dfa55c4b7dcf617698c38102a4a9c104	1.1.1-beta1	1
e567a92e50fcdc68a599ba598cdd80985307759c	(1.0, 1.0, 1.0)	{'LoG': {}, 'Original': {}, 'Wavelet': {}}	b98a859d1c4ed214091ac916efa5ca0b3e25bab6	1.1.1-beta1	1
bb83d60db485faac72a5af79b593e920ddc3806a	(1.0, 1.0, 1.0)	{'LoG': {}, 'Original': {}, 'Wavelet': {}}	5ebd593caed4911fff08c5f2e4f2c6312d527561	1.1.1-beta1	2
a73d9a89b71cea5258e59ad0bfee7eb241a94258	(1.0, 1.0, 1.0)	{'LoG': {}, 'Original': {}, 'Wavelet': {}}	852b71e75ba6969c5bae510208b593bafbbade1d	1.1.1-beta1	1
6f7dbc63238a8f35b442abd4d0df881a5c90a25c	(1.0, 1.0, 1.0)	{'LoG': {}, 'Original': {}, 'Wavelet': {}}	112add3a0e5311851fb41ddfd3d78ef6567bfbe0	1.1.1-beta1	1
ab6ef8e1c396418632c92d0f36915895773e5e7c	(1.0, 1.0, 1.0)	{'LoG': {}, 'Original': {}, 'Wavelet': {}}	d6c63c23d0bd8e41c72f640377ac72434800321c	1.1.1-beta1	1
d9f46cc9c5c2019d299615d06d6993a53e2bd258	(1.0, 1.0, 1.0)	{'LoG': {}, 'Original': {}, 'Wavelet': {}}	1b2b02f076a79f8065bd5c071a99d58449ab0c64	1.1.1-beta1	1
5b2de6388b14d2c1e149157e5ff79b32e4fcc5e4	(1.0, 1.0, 1.0)	{'LoG': {}, 'Original': {}, 'Wavelet': {}}	a1de9c1d81d71109753f8b477f8a40259239e900	1.1.1-beta1	1
341adba7f1fdbadb81a40490335321ac03811a01	(1.0, 1.0, 1.0)	{'LoG': {}, 'Original': {}, 'Wavelet': {}}	1457001c73140bd2f4c6cd6c331814ecca6dc0ae	1.1.1-beta1	1
7505a548e249926abe434b898d8fc2e5763f238d	(1.0, 1.0, 1.0)	{'LoG': {}, 'Original': {}, 'Wavelet': {}}	938daf80d6f7d0465f5b4443121b48dd50ff586e	1.1.1-beta1	1
eb16fbadd3bea123c4b0fae2635d350cb7791af5	(1.0, 1.0, 1.0)	{'LoG': {}, 'Original': {}, 'Wavelet': {}}	ec6e9eca0c51aa69e20e0b97b3e1681ef2997d6d	1.1.1-beta1	1
2f247bc2a882d7a7b134d0035e403c3a80a8d389	(1.0, 1.0, 1.0)	{'LoG': {}, 'Original': {}, 'Wavelet': {}}	01ad058b85addfe603cc3d6d15c3f7405c9359e7	1.1.1-beta1	1
88011fc8e4004e2da019b1e69eeb737f6b1b8a5c	(1.0, 1.0, 1.0)	{'LoG': {}, 'Original': {}, 'Wavelet': {}}	cca78c06af2e2c5cfd42675d3e925733f7ecc307	1.1.1-beta1	1
27878584982c4556d58290555364c0bf9271a6b2	(1.0, 1.0, 1.0)	{'LoG': {}, 'Original': {}, 'Wavelet': {}}	1c5d6928e0cff9dab61c72bb7e841c32f20ab031	1.1.1-beta1	1
bb975f93f3220af49485a0f8e3cbc6248da84fe9	(1.0, 1.0, 1.0)	{'LoG': {}, 'Original': {}, 'Wavelet': {}}	c5235bd4bdd93b70ad4cd787995e7b551f546e11	1.1.1-beta1	1
c038c6ada1b41149a97d5d58676ae2c625c43d0e	(1.0, 1.0, 1.0)	{'LoG': {}, 'Original': {}, 'Wavelet': {}}	32cf9a63a0ccfb6a496f750149d33f8e7d10a2f3	1.1.1-beta1	1
7b65b3b77f26f606f1ad35358001d7167203c041	(1.0, 1.0, 1.0)	{'LoG': {}, 'Original': {}, 'Wavelet': {}}	61e91fcde06513839f60828947cbfdf52f30ef1f	1.1.1-beta1	1
1e75d9bc15f8092472c67d8c924d8786f94c1e78	(1.0, 1.0, 1.0)	{'LoG': {}, 'Original': {}, 'Wavelet': {}}	5fd834ca4e5ee04cad9ac904dfeb49328a7deacc	1.1.1-beta1	1
38e36f9da7fef61903791c86f3239e06c671c416	(1.0, 1.0, 1.0)	{'LoG': {}, 'Original': {}, 'Wavelet': {}}	e3f59bcbb4c94f6173323d259d67a4fb3065f69d	1.1.1-beta1	1
fd24cf5e6b7434ab0bfc65f6c8a8ca90b14ee927	(1.0, 1.0, 1.0)	{'LoG': {}, 'Original': {}, 'Wavelet': {}}	a189be6c67c11db75867fe8f78928737727fb21f	1.1.1-beta1	1
a2f8c625d6f5fe6dfeda8762d8a180e51e1e0860	(1.0, 1.0, 1.0)	{'LoG': {}, 'Original': {}, 'Wavelet': {}}	fff9df1a9b83daf2bb7a85a3722934fede7e9584	1.1.1-beta1	1
98c354e74e35b0436885e9fa927228ab2a4fab7d	(1.0, 1.0, 1.0)	{'LoG': {}, 'Original': {}, 'Wavelet': {}}	155b688c41e10b86f19ba48a6b550873f4f7f2db	1.1.1-beta1	1
c907b4bccf40f0ddf63e2ab0ea5b2631e2a3fa9e	(1.0, 1.0, 1.0)	{'LoG': {}, 'Original': {}, 'Wavelet': {}}	e9354777f0f5033109cbcd01da03188174e6d4c6	1.1.1-beta1	1
bab82adc990efd6447998cba8f0e2c5158114246	(1.0, 1.0, 1.0)	{'LoG': {}, 'Original': {}, 'Wavelet': {}}	a76665de9ba4cc0fe36a3716855f8c4315a032b9	1.1.1-beta1	1
a82808907bd5f4f4342a69e2ffa3a4b2a00a0435	(1.0, 1.0, 1.0)	{'LoG': {}, 'Original': {}, 'Wavelet': {}}	7faa374f6f5d2bc5e541e8c7d58ee3a238bc789d	1.1.1-beta1	1
a3ef48a21870bef679038cdf1da0a554774524e5	(1.0, 1.0, 1.0)	{'LoG': {}, 'Original': {}, 'Wavelet': {}}	fe1dde5917e46a75e07320a5ddd876850e42fe01	1.1.1-beta1	1
060480befcbe7178b54f76651aef987231588545	(1.0, 1.0, 1.0)	{'LoG': {}, 'Original': {}, 'Wavelet': {}}	b285ec54b96c327a4e829d1fc67a37db8bee52b7	1.1.1-beta1	1
d271553e8e5eca7e73252b2e6e52b09a3019aca8	(1.0, 1.0, 1.0)	{'LoG': {}, 'Original': {}, 'Wavelet': {}}	a086edf3e468cc6224a5b5d18efedf1a962f1a71	1.1.1-beta1	1
1751d2ed951684b0961cc9a1157a921e2543d279	(1.0, 1.0, 1.0)	{'LoG': {}, 'Original': {}, 'Wavelet': {}}	2e99cd96df3736da16f6d1aa283176a4a4bf8d26	1.1.1-beta1	1

general_info_VoxelNum	original_shape_Maximum3DDiameter	original_shape_Compactness2	original_shape_Maximum2DDiameterSlice	original_shape_Sphericity	original_shape_Compactness1	original_shape_Elongation
706	41.02438299	0.304485403	36.61966685	0.672752767	0.029274003	1.157472739
2077	63.35613625	0.1253789	63.07138812	0.50050469	0.018784996	1.137390524
434	40.02499219	0.315583706	39	0.680829226	0.029802738	1.480940021
2635	78.05767099	0.108420745	63.78087488	0.476837932	0.017468478	1.178934735
359	31.32091953	0.553696999	27.65863337	0.821152948	0.039476166	1.075111024
643	37.34969879	0.402277541	33.54101966	0.738202076	0.033648195	1.0357752
337	42.53234064	0.521060506	40.80441153	0.804691448	0.038295078	1.830784002
1002	48.09365863	0.301376511	45.69463864	0.670455257	0.029124171	1.355209273
2079	65.17668295	0.123068678	59.77457654	0.497411526	0.018611126	1.194525969
3098	87.10338685	0.077870826	61.18823416	0.427029877	0.014804245	1.465355195
827	48.09365863	0.298184799	41.67733197	0.668080045	0.028969542	1.254166298
1482	71.8748913	0.199087073	50.91168825	0.583912385	0.023671207	1.643811888
2074	61.4817046	0.135084557	56.36488268	0.513099866	0.019498524	1.084775367
1118	48.55924217	0.24533485	45.89117562	0.626017415	0.026277164	1.21209068
911	53.24471805	0.294092801	39	0.665009932	0.02877008	1.398284468
6947	127.6322843	0.021048189	98.27003612	0.276103288	0.00769673	1.369685278
698	49.29503018	0.43816177	45.69463864	0.759529816	0.035116896	1.512482278
2332	85.74963557	0.112790709	63.28506933	0.483160151	0.017817039	1.802162194
582	42.42640687	0.440910852	32.44996148	0.761114968	0.035226887	1.429327672
2801	85.4341852	0.061325928	58.94064811	0.394349575	0.013137748	1.420209869
5224	107.5825265	0.05090897	72.56031973	0.370622205	0.011970052	1.488584403
1720	71.37226352	0.163227567	45.69463864	0.546509651	0.021433625	1.476832609
2731	98.08669635	0.058990512	79.20227269	0.389278773	0.012885164	1.298122314
481	41.78516483	0.475209907	34.98571137	0.780360291	0.036571403	1.22014039
1204	70.61161378	0.13298103	61.55485359	0.510422603	0.019346113	1.697038763
694	48.18713521	0.319433069	42.95346319	0.683586207	0.029983948	1.705796292
3466	83.03011502	0.100745436	82.54089895	0.465309365	0.016838817	1.098530056
1433	97.5807358	0.096779193	96.84007435	0.459121185	0.016504025	3.06820024
1765	90.09994451	0.069686876	72.56031973	0.411513099	0.014004718	1.121019503
2542	68.73863542	0.109708476	58.94064811	0.478718335	0.01757191	1.072039453
3179	105.6834897	0.053800779	72.99315036	0.377510923	0.012305327	1.768113456
5930	129.0697486	0.025746416	92.0271699	0.295283328	0.008512503	1.166443702
2033	66.06814664	0.144327778	55.80322571	0.524545672	0.020154584	1.208053984
104	18	0.819962945	15	0.935976063	0.048039225	1.052410496
332	38.18376618	0.483956064	25.63201124	0.785118683	0.036906414	1.549334513
2020	61.84658438	0.148581151	54.08326913	0.529648695	0.020449408	1.043286666
2880	103.1406806	0.063305027	91.24143795	0.398546868	0.013348055	1.372326207
3354	86.79285685	0.080625681	75.89466384	0.432007346	0.015063836	1.142471745
835	44.39594576	0.384232932	40.80441153	0.726995174	0.032884873	1.202990163
1049	47.52893855	0.229019216	42.95346319	0.611820429	0.025388371	1.062107579
1474	55.64171097	0.203321986	54.74486277	0.588023633	0.023921645	1.098021642
953	48.92851929	0.275541771	47.4341649	0.650722487	0.02784791	1.105955238
1642	73.23933369	0.170206321	72.99315036	0.554189842	0.021887024	1.479627385
1025	56.60388679	0.267038448	40.80441153	0.643958577	0.027414844	1.474191638
1878	66.13622306	0.158372017	53.66563146	0.541035982	0.021112424	1.106793852
2228	118.8696765	0.055763126	98.95453501	0.382046041	0.012527731	1.393509657
4266	85.85452813	0.084862078	68.41052551	0.439445026	0.015454527	1.273091453
583	36.61966685	0.391118402	34.20526275	0.731312085	0.033178214	1.05890169
134	21.21320344	0.822906564	17.49285568	0.937094759	0.048125376	1.138241693
497	37.34969879	0.527848804	31.32091953	0.808170848	0.038543722	1.342481209
3424	87.20665112	0.061292004	80.77747211	0.394276846	0.013134114	1.181732459
827	42.42640687	0.282357555	35.11409973	0.656044256	0.028190228	1.120927016
3821	90.09994451	0.070309105	63.5688603	0.412734263	0.014067103	1.38344691
591	36.24913792	0.382444368	34.98571137	0.725865391	0.032808246	1.15300422
6523	154.2011673	0.014945268	128.26535	0.246320887	0.006485609	1.78376296
879	44.59820624	0.309830439	40.24922359	0.676666528	0.029529828	1.134363136
2193	99	0.166608774	70.22819946	0.550257482	0.021654483	1.827696947
1293	68.47627326	0.265927473	54.08326913	0.643064303	0.027357757	1.706122359
814	43.78355856	0.323886454	41.67733197	0.686748303	0.030192235	1.115985732
881	48.09365863	0.233389352	45.89117562	0.615687514	0.025629456	1.343067921
2119	63.5688603	0.133487674	56.04462508	0.51107	0.019382931	1.107982008
5478	106.1508361	0.033752732	76.89603371	0.323173923	0.009746604	1.387310674
3090	118.224363	0.038171065	70.80254233	0.336701275	0.010364922	1.692366793
2731	82.15838363	0.083284728	75.23961722	0.436705294	0.015310225	1.323064982
217	24.55605832	0.746283943	24.18677324	0.90705725	0.045830113	1.104814609
1553	72.62231062	0.104035271	60.67124525	0.470320094	0.017111543	1.541572375
394	33.27160952	0.578738647	30.88689042	0.833350106	0.040358974	1.189020627
3197	81.88406438	0.065885397	70.80254233	0.403889959	0.013617377	1.277686613
446	31.46426545	0.569497942	28.46049894	0.828890928	0.040035472	1.073353008
2011	69.84268036	0.081679198	67.08203932	0.433880856	0.015161934	1.133716314
3040	90.44888059	0.069070072	76.66159404	0.410295389	0.013942602	1.35215091
1129	52.13444159	0.234907886	47.4341649	0.61701994	0.0257127	1.370230739
5168	104.1393297	0.032360198	90.24965374	0.318666974	0.009543429	1.141704262
6716	127.2084903	0.027551503	117.8812962	0.302028852	0.008805855	1.221063418
9180	123.073149	0.025351834	115.4123044	0.293767081	0.008447021	1.027186751
1116	62.20932406	0.280387296	56.60388679	0.654514759	0.028091701	1.495184969
1117	55.72252686	0.264416528	53.66563146	0.641844069	0.027279925	1.321723685
610	38.06573262	0.396459186	34.20526275	0.734625775	0.033403973	1.154952343
1764	65.31462317	0.170403589	61.18823416	0.554403861	0.021899704	1.034765553
2846	91.6351461	0.079506927	82.54089895	0.429999868	0.014958959	1.080678048
504	45.49725266	0.46601511	44.59820624	0.775294434	0.036215866	1.295816422
1341	72.0624729	0.142702499	69.26037828	0.522569261	0.020040782	1.734910899
2792	82.81304245	0.046607715	73.05477397	0.359875767	0.011453225	1.054698352
145	21.21320344	0.755044586	20.1246118	0.910592773	0.046098328	1.17553699
3391	85.38149682	0.051796293	78.74642849	0.372763081	0.012073918	1.108288022
5476	99.13626985	0.068918085	98.95453501	0.40999422	0.013927253	1.412734726
778	41.35214626	0.282054283	34.98571137	0.655809293	0.028175084	1.087683019
1552	88.43641784	0.203397992	73.05477397	0.588096896	0.023926116	2.329822057
183	29.08607914	0.594528223	28.3019434	0.840860901	0.040905821	1.587516475
105	22.44994432	0.657620663	21.63330765	0.869611252	0.043021604	1.193885701
3975	124.8519123	0.062250689	96.93296653	0.396321884	0.013236433	1.716211788
706	48.92851929	0.290166374	32.44996148	0.662037151	0.028577381	1.592850109
1022	51.70106382	0.243157744	48.46648326	0.624160147	0.026160313	1.323953316
4090	93.1933474	0.06672959	78.74642849	0.405607663	0.013704339	1.197101982
5806	96.04686356	0.044744207	85.90692638	0.355014102	0.011221923	1.025034466
253	28.14249456	0.739036849	23.43074903	0.904111578	0.045607044	1.156163764
1216	53.16013544	0.215722069	47.4341649	0.599742546	0.02464031	1.356139869
123	21.21320344	0.768128444	17.49285568	0.915822444	0.046496022	1.255845816
379	31.32091953	0.616853495	30	0.851256962	0.041666775	1.028701923
807	58.55766389	0.26374757	50.28916384	0.641302337	0.027245395	1.502992265

original_shape_SurfaceVolumeRatio	original_shape_Volume	original_shape_SphericalDisproportion	original_shape_Flatness	original_shape_SurfaceArea	original_shape_Maximum2DDiameterColumn
0.269095113	19062	1.486430156	1.127304415	5129.491039	40.02499219
0.252430922	56079	1.997983276	1.172006132	14156.07365	57.93962375
0.312724161	11718	1.46879711	1.237281793	3664.501719	27.65863337
0.244755166	71145	2.097148596	1.258379411	17413.10629	65.7951366
0.276210805	9693	1.217799926	1.247420787	2677.311333	30
0.252998114	17361	1.354642628	1.150910578	4392.300253	34.98571137
0.287865874	9099	1.24271235	1.446924826	2619.291592	29.54657341
0.240272368	27054	1.491523841	1.053971054	6500.328647	46.95742753
0.253919193	56133	2.010407776	1.129963712	14253.24605	62.64183905
0.258946953	83646	2.341756525	1.062671795	21659.87679	85.80209788
0.257058834	22329	1.496826626	1.095779976	5739.866701	36.12478374
0.242139578	40014	1.712585699	1.326368858	9688.973085	53.66563146
0.246353111	55998	1.948938338	1.097808003	13795.28151	59.09314681
0.248101384	30186	1.59739965	1.021149881	7489.188377	42.95346319
0.25005101	24597	1.50373694	1.183709663	6150.504688	49.2036584
0.305979402	187569	3.621832998	1.150745095	57392.25044	122.4499898
0.239257872	18846	1.316604007	1.337196499	4509.053847	42.95346319
0.251591277	62964	2.069707111	1.078242094	15841.19316	60
0.253671462	15714	1.313861955	1.064429653	3986.193355	34.98571137
0.289986062	75627	2.535821171	1.243177118	21930.77587	75.71657678
0.250666931	141048	2.69816537	1.135072132	35356.06932	105.3422992
0.246181578	46440	1.829793854	1.346646684	11432.6725	65.8634952
0.296252198	73737	2.568853147	1.745783125	21844.74831	86.58521814
0.263644884	12987	1.281459361	1.481188727	3423.956113	33.9411255
0.29686396	32508	1.959160888	1.274020891	9650.453608	51
0.266348193	18738	1.462873285	1.064090813	4990.832445	28.46049894
0.228917094	93582	2.149107831	1.316673132	21422.51947	67.41661516
0.311425001	38691	2.178074184	1.260297618	12049.3447	43.68065934
0.324138762	47655	2.4300563	2.421664122	15446.83269	84.48076704
0.246731326	68634	2.08891101	1.069101385	16934.15785	64.41273166
0.290404386	85833	2.648929972	1.449020724	24926.27966	83.57032966
0.301605353	160110	3.386577921	1.182750136	48290.033	106.0235823
0.242586764	54891	1.906411688	1.144697414	13315.83009	61.55485359
0.366229801	2808	1.068403391	1.111970098	1028.373282	16.97056275
0.296516043	8964	1.273692783	1.381639715	2657.969814	34.20526275
0.240763791	54540	1.888043925	1.191392136	13131.25719	55.15432893
0.284284146	77760	2.509115189	1.827479322	22105.93516	88.28363382
0.249278057	90558	2.314775452	1.269766561	22574.12233	70.80254233
0.235470144	22545	1.375524949	1.160861984	5308.674401	34.20526275
0.259306697	28323	1.634466507	1.066701803	7344.343569	44.29446918
0.240880842	39798	1.700611922	1.316706162	9586.575762	48.83646179
0.251730645	25731	1.536753408	1.262170167	6477.281215	46.57252409
0.246554684	44334	1.804435815	1.404363092	10930.75536	72.24956747
0.248273471	27675	1.552894916	1.129430646	6870.968311	45.69463864
0.241493173	50706	1.848305906	1.278675026	12245.15284	64.89992296
0.323054234	60156	2.617485574	3.015407031	19433.65052	96.04686356
0.226178524	115182	2.275597493	1.159296014	26051.69478	85.38149682
0.263858204	15741	1.367405272	1.135358539	4153.391984	32.44996148
0.336158756	3618	1.067127941	1.146421357	1216.22238	19.20937271
0.251810727	13419	1.237362128	1.069870404	3379.048141	29.69848481
0.271258701	92448	2.53628893	1.169785649	25077.32437	80.04998438
0.261774836	22329	1.52428741	1.056813312	5845.170307	38.18376618
0.249823526	103167	2.422866451	1.237331352	25773.5437	88.84255737
0.264633174	15957	1.377665904	1.102862973	4222.751562	32.44996148
0.350250827	176121	4.059745036	1.04461016	61686.52595	98.95453501
0.248690131	23733	1.477832816	1.06318268	5902.162876	38.41874542
0.225484851	59211	1.817331037	1.58114774	13351.18351	73.23933369
0.230095942	34911	1.55505444	1.232417628	8032.879425	55.31726674
0.251395292	21978	1.456137563	1.198806817	5525.165726	39.11521443
0.273113953	23787	1.624200552	1.250656561	6496.561588	46.57252409
0.24556822	57213	1.956679123	1.093163785	14049.69457	54.08326913
0.282956201	147906	3.094309065	1.103659768	41850.91993	97.67292358
0.328699176	83430	2.969991726	1.434644458	27423.37229	111.3642672
0.264078988	73737	2.289873774	1.171501044	19472.3923	63.28506933
0.295738768	5859	1.102466244	1.174706395	1732.733442	22.84731932
0.295968518	41931	2.126211516	1.501333413	12410.25591	66.40783086
0.263857819	10638	1.199975848	1.232196806	2806.919475	30
0.270926987	86319	2.475921912	1.16530292	23386.14662	72.12489168
0.254538725	12042	1.206431349	1.073445087	3065.155324	28.46049894
0.29434389	54297	2.304780187	1.331148212	15981.99018	59.09314681
0.27121167	82080	2.437268433	1.384666817	22261.05384	72.12489168
0.250899054	30483	1.620693166	1.134776282	7648.155854	45
0.292584722	139536	3.138072288	1.3686016	40826.10182	96.74709298
0.282885623	181332	3.310941963	1.228695689	51296.21572	114.2365966
0.262066906	247860	3.404057375	1.311514355	64955.90323	112.3298714
0.237440808	30132	1.527849428	1.294783844	7154.566416	45.79301257
0.242055867	30159	1.558010813	1.334317223	7300.162881	43.26661531
0.258733957	16470	1.361237292	1.036188698	4261.348265	36.61966685
0.240641447	47628	1.803739242	1.628004975	11461.27083	61.84658438
0.264534863	76842	2.325582107	1.555742749	20327.38792	67.41661516
0.261267882	13608	1.289832554	1.782663441	3555.333341	35.11409973
0.279732387	36207	1.913621935	1.321727833	10128.27053	52.39274759
0.318105952	75384	2.778736698	1.722996976	23980.09908	80.49844719
0.336963352	3915	1.098185742	1.138421687	1319.211523	19.20937271
0.287841923	91557	2.682669102	1.385128574	26353.94292	77.4209274
0.223064601	147852	2.439058775	1.090466324	32980.54738	96.42095208
0.267254753	21006	1.524833532	1.033379618	5613.953344	35.11409973
0.236746414	41904	1.700400064	1.378229048	9920.621748	45
0.337666789	4941	1.189257342	1.164268979	1668.411604	21.63330765
0.392923454	2835	1.149939123	1.914062226	1113.937992	18.97366596
0.256764969	107325	2.523201571	1.127595482	27557.30031	84.05355436
0.273450639	19062	1.510489251	1.161083346	5212.516072	41.78516483
0.256399123	27594	1.602152917	1.063288933	7075.077413	40.24922359
0.248512907	110430	2.465436654	1.266641239	27443.28027	82.37718131
0.252633877	156762	2.816789512	1.214863564	39603.39189	87.20665112
0.281903628	6831	1.106058172	1.183184849	1925.683682	25.45584412
0.251818028	32832	1.667382123	1.033131858	8267.689503	48.83646179
0.353929327	3321	1.091914712	1.087202109	1175.399295	17.49285568
0.261671118	10233	1.174733418	1.303887652	2677.68055	26.83281573
0.269986596	21789	1.559326924	1.367530932	5882.737931	55.80322571

original_shape_Maximum2DDiameterRow	log.sigma.5.0.mm.3D_gldm_GrayLevelVariance	log.sigma.5.0.mm.3D_gldm_HighGrayLevelEmphasis	log.sigma.5.0.mm.3D_gldm_GrayLevelNonUniformityNormalized
40.02499219	43.45610871	309.8484419	0.043259315
59.77457654	21.85941957	202.3134328	0.065741694
36.49657518	19.13135233	160.3248848	0.063762237
72.62231062	29.3407755	360.940038	0.051829661
28.3019434	45.19857853	395.1699164	0.043039703
33.54101966	24.15144794	202.8289269	0.059231006
25.63201124	14.82555979	87.25222552	0.080435682
42.63801121	50.90029123	350.5149701	0.039430122
52.39274759	39.88662872	412.4564695	0.044472069
71.30918594	22.45282052	190.9496449	0.060361315
43.26661531	29.41200622	250.2237001	0.053948582
59.16924877	29.19687123	196.1504723	0.051836614
53.16013544	28.99886086	164.0694311	0.051914738
47.4341649	27.17668914	288.1234347	0.055075349
36.49657518	27.75716484	251.2777168	0.054857029
113.0884609	12.31167314	262.4996401	0.114485835
32.44996148	32.81600315	277.3037249	0.050512722
70.29224708	36.35367778	226.478988	0.047070514
40.36087214	41.53674673	438.8608247	0.049178682
82.97590012	16.60394302	131.1802928	0.073534328
103.4456379	13.3824355	187.931853	0.079988198
63.5688603	26.42147073	206.152907	0.054969578
96.93296653	21.17963585	252.0838521	0.065275601
34.20526275	30.45899698	235.8835759	0.050790756
66.40783086	14.16485124	104.0207641	0.073913367
45.79301257	32.49373593	240.3270893	0.049622537
76.4852927	14.3313951	179.8612233	0.08816634
94.20191081	22.37702174	153.4180042	0.059912179
85.38149682	33.74696402	287.7926346	0.047429961
64.41273166	22.46910967	205.9213218	0.062200043
99.04544412	18.12694566	150.8222712	0.068355056
104.6565813	25.34016033	258.3838111	0.058501347
52.39274759	33.5363042	288.372848	0.048112925
16.97056275	22.75508506	124.1057692	0.065643491
33.9411255	25.66167078	274.1987952	0.062146175
60	33.05881066	261.7420792	0.048546711
101.8233765	29.09930363	175.4756944	0.05388479
72.99315036	20.38349176	169.4651163	0.062980718
41.78516483	25.2104041	329.7233533	0.067813116
39.11521443	31.52614365	247.1315539	0.051698426
54.08326913	21.5790868	256.3297151	0.06500463
42.63801121	43.74717273	333.818468	0.043788379
49.2036584	32.49353229	271.6309379	0.048947319
55.31726674	43.77889732	320.0195122	0.042986794
55.07267925	28.40148556	225.8503727	0.052949856
93.77099765	14.8455408	80.41337522	0.079887445
82.97590012	23.99944661	243.0269573	0.057191711
33.9411255	32.88664829	238.8559177	0.04998985
19.20937271	18.29494319	140.6119403	0.067052796
36.24913792	45.77288277	474.1046278	0.049726933
80.77747211	22.49585117	190.5858645	0.060509344
40.36087214	33.8978432	230.5755744	0.05074942
87.20665112	28.57283524	248.6226119	0.054562929
34.20526275	21.76266674	202.4399323	0.062502684
149.2179614	17.73286435	169.4534723	0.089446842
41.67733197	37.77706593	328.7531286	0.046014902
65.7951366	34.44178665	304.1171911	0.046585028
47.4341649	34.76159869	497.8600155	0.054585671
40.36087214	28.06567048	210.514742	0.054135552
35.11409973	20.89214738	164.7832009	0.060955137
61.77378085	36.66105113	207.6422841	0.046598106
99.72462083	32.67523601	276.6518802	0.050419075
96.74709298	13.9945807	134.6530744	0.083078937
77.12976079	14.78741831	172.0322226	0.081212079
20.1246118	30.18488394	202.1935484	0.053876702
48.83646179	26.986739	218.166774	0.056122603
29.69848481	43.11747275	369.4974619	0.045736814
68.47627326	38.2386633	245.0153269	0.04628391
28.3019434	42.49832593	334.1726457	0.047246074
64.76109943	27.05646542	190.0726007	0.05429437
88.23264702	31.32295663	217.6993421	0.050485197
46.86149806	40.89855104	277.4968999	0.043239626
94.86832981	20.4958615	159.8436533	0.064701943
115.4123044	16.59164879	217.6392198	0.070206145
118.3765179	19.24111496	191.5196078	0.078150664
51.26402247	42.72455631	373.4363799	0.044123598
48.37354649	28.58409908	205.2336616	0.051550507
32.44996148	41.7361032	257.2	0.043418436
54.74486277	30.90692246	168.0119048	0.049825561
89.19641248	21.82088607	291.9992973	0.059811727
32.31098884	52.88378685	350.0357143	0.040320295
67.08203932	30.3234589	254.9209545	0.050370882
77.12976079	29.94919736	299.1583095	0.053924537
20.1246118	10.6870868	102.1862069	0.091652794
81.60882305	22.16067216	160.0967266	0.060177595
75.17978452	17.556249	191.3424032	0.073051888
40.36087214	41.46686349	245.9421594	0.043668757
75	48.72342596	622.9001289	0.041935414
24.18677324	22.89993729	186.4043716	0.065902237
16.15549442	21.18603175	119.8571429	0.068662132
89.04493248	27.18601005	352.8636478	0.058969123
47.4341649	38.98042677	194.6997167	0.044908474
49.47726751	16.23934211	101.0342466	0.06924759
85.80209788	20.19157113	168.108313	0.064098254
88.23264702	19.84355844	141.44299	0.065017492
25.45584412	60.86159759	446.7588933	0.042072209
45	29.77950788	269.7236842	0.051951231
19.20937271	18.13576575	90.04065041	0.066693106
28.46049894	19.16548896	197.2717678	0.065990908
43.68065934	38.79459316	236.9516729	0.048760152

log.sigma.5.0.mm.3D_gldm_DependenceEntropy	log.sigma.5.0.mm.3D_gldm_DependenceNonUniformity	log.sigma.5.0.mm.3D_gldm_GrayLevelNonUniformity	log.sigma.5.0.mm.3D_gldm_SmallDependenceEmphasis
6.630199096	176.5014164	30.54107649	0.338786917
7.065647417	283.966779	136.5454983	0.207420611
6.242193957	92.28110599	27.67281106	0.258533536
6.947771806	512.944592	136.5711575	0.268633643
6.462984839	86.77158774	15.45125348	0.308563912
6.324226221	152.5583204	38.08553655	0.24740415
6.224330721	48.54302671	27.10682493	0.1828464
6.749674567	260.9001996	39.50898204	0.363136488
6.955733956	431.9023569	92.45743146	0.292144301
6.873800459	522.1078115	186.9993544	0.233265969
6.430531759	196.8573156	44.61547763	0.275805434
6.825160007	294.2537112	76.82186235	0.242591389
6.947614165	380.6422372	107.6711668	0.19877547
6.541515437	250.1144902	61.57423971	0.265663702
6.657023678	183.6520307	49.97475302	0.263804496
7.227108309	531.3618828	795.3330934	0.118212914
6.431343878	168.9885387	35.25787966	0.305083324
6.923420097	482.4871355	109.7684391	0.283831921
6.643876684	128.1580756	28.62199313	0.312071139
6.859605447	415.8711175	205.9696537	0.173755331
6.983310496	673.2320061	417.8583461	0.153575583
6.762706485	331.8093023	94.54767442	0.24059662
6.682072198	498.5825705	178.2676675	0.226295886
6.604734153	92.09355509	24.43035343	0.278712363
6.522120194	191.269103	88.99169435	0.223906783
6.466643229	159.3832853	34.43804035	0.340762088
7.180844346	350.4616272	305.5845355	0.138425825
6.630994427	273.8709002	85.85415213	0.228862391
6.622941598	429.0067989	83.71388102	0.324055305
7.193475639	338.3587726	158.1125098	0.210711251
6.639674057	534.6533501	217.3007235	0.221681686
7.172631955	933.13457	346.9129848	0.214716062
6.898640948	428.03394	97.813576	0.262031276
5.386697502	24.53846154	6.826923077	0.364252137
6.083960918	73.69277108	20.63253012	0.29309488
6.783182275	442.6148515	98.06435644	0.283631199
6.874962163	530.7381944	155.1881944	0.261441752
6.972837846	525.8449612	211.2373286	0.18161429
6.751778679	134.0754491	56.6239521	0.208493686
6.752481542	213.5719733	54.23164919	0.317242449
6.950251006	225.2713704	95.81682497	0.207165909
6.632621475	238.0912907	41.73032529	0.322717561
6.623856672	395.8148599	80.37149817	0.299081167
6.691758733	254.2663415	44.06146341	0.339256894
6.928924266	339.5697551	99.43982961	0.243126382
6.787530145	278.989228	177.989228	0.18416746
7.029519627	712.2165963	243.9798406	0.216874249
6.365157706	144.7701544	29.14408233	0.320606284
5.453012818	33.04477612	8.985074627	0.319369818
6.437998319	122.0301811	24.71428571	0.343459228
6.970706311	568.0251168	207.1839953	0.212776795
6.323794868	213.1378476	41.96977025	0.334329887
7.014646144	713.1277153	208.4849516	0.255309882
6.390234751	123.0067682	36.93908629	0.244347936
7.045536072	733.5877664	583.4617507	0.182605948
6.621488807	214.2172924	40.44709898	0.363908266
6.845775078	473.4532604	102.1609667	0.321407316
6.83800927	258.9938128	70.57927301	0.285807668
6.707499178	152.1670762	44.06633907	0.263246715
6.39584022	190.0011351	53.7014756	0.247771239
6.706651509	473.2982539	98.74138745	0.306172452
7.151980571	1001.01862	276.1956919	0.264646672
6.815180823	427.6957929	256.7139159	0.174177793
6.998265409	311.3976565	221.7901867	0.148147867
5.992722922	53.28571429	11.69124424	0.352431476
6.7901246	297.1751449	87.15840309	0.255780231
6.413330732	102.9898477	18.02030457	0.321183728
7.003420649	656.0678761	147.9696591	0.286923221
6.345710021	117.4529148	21.07174888	0.390249751
6.743957568	393.031825	109.1859771	0.261752296
6.965710659	588.4013158	153.475	0.278345707
6.704285813	276.7289637	48.81753764	0.318542151
6.984369651	804.5386997	334.379644	0.205736586
7.047921377	903.8037522	471.5044669	0.173547134
7.206320368	1088.963181	717.4230937	0.167380691
6.688778037	278.4050179	49.24193548	0.351857314
6.469636612	268.6902417	57.58191585	0.293962112
6.4636745	167.4590164	26.4852459	0.348942623
6.69027695	385.3730159	87.89229025	0.26889086
6.661889743	551.7434996	170.2241743	0.244026957
6.471944363	135.8571429	20.32142857	0.400708774
6.581721044	306.9045488	67.54735272	0.317995297
6.942623069	526.1361032	150.5573066	0.249470961
5.459824265	29.89655172	13.28965517	0.234358238
6.768235046	616.2214686	204.0622235	0.225518031
7.058056288	738.6263696	400.0321402	0.165178809
6.591824786	186.940874	33.97429306	0.350008737
6.756604584	406.1765464	65.08376289	0.364911204
6.030448612	32.97814208	12.06010929	0.333717908
5.564072604	22.48571429	7.20952381	0.359843915
7.173142442	607.8432704	234.4022642	0.227005135
6.482063656	171.9915014	31.70538244	0.355686921
6.816751982	143.4403131	70.77103718	0.194308297
6.95940486	663.60489	262.1618582	0.204151735
7.068313123	819.3551498	377.4915605	0.198446129
6.06563634	79.29249012	10.64426877	0.446551383
6.822046981	236.7944079	63.17269737	0.25221693
5.233749745	35.3902439	8.203252033	0.34730804
6.266829765	73.27440633	25.01055409	0.222922965
6.944491515	144.2242875	39.34944238	0.282805027

log.sigma.5.0.mm.3D_gldm_DependenceNonUniformityNormalized	log.sigma.5.0.mm.3D_gldm_DependenceVariance	log.sigma.5.0.mm.3D_gldm_LargeDependenceEmphasis
0.250002006	1.83284514	8.218130312
0.136719682	8.246322088	25.55464612
0.212629276	2.667862983	11.77880184
0.194665879	3.041146441	12.49297913
0.241703587	1.375579023	8.158774373
0.237260218	1.617137785	9.690513219
0.14404459	4.285852653	19.93175074
0.260379441	1.275337548	6.936127745
0.207745241	2.257866232	10.44588745
0.168530604	4.550093544	16.73014848
0.238037867	1.400888104	8.888754534
0.198551762	2.58510493	12.37921727
0.18353049	2.925367943	14.66441659
0.223716002	1.727119409	9.817531306
0.201593887	2.054070207	10.88364435
0.076487964	22.47862211	67.9687635
0.242103924	1.594773442	8.498567335
0.206898429	3.194259008	12.07118353
0.220202879	2.02251981	9.467353952
0.148472373	7.425557838	24.4673331
0.128872896	9.542111418	31.06929556
0.192912385	2.524222553	12.41744186
0.182564105	2.95954996	13.88026364
0.191462693	2.623268399	11.74844075
0.158861381	3.533848964	16.08803987
0.229658913	1.642709432	8.250720461
0.101114145	20.60737878	53.5389498
0.191117167	2.66918206	13.01395673
0.243063342	1.432867289	7.930311615
0.133107306	10.76754273	29.56805665
0.168182872	3.55663944	15.6234665
0.157358275	7.282461204	22.03946037
0.21054301	2.499536303	11.41121495
0.235946746	1.518860947	7.673076923
0.221966178	1.598417767	9.078313253
0.219116263	2.131394961	10.13861386
0.184284095	3.005011092	12.97569444
0.156781443	5.088535161	20.19499106
0.1605694	5.598402237	19.62275449
0.20359578	3.15351949	11.52430887
0.152829966	6.119239132	20.95793758
0.249833463	1.32403885	7.682056663
0.241056553	1.502508008	8.462850183
0.248064723	1.330613682	7.485853659
0.180814566	3.283170073	14.03514377
0.125219582	7.707986972	26.96858169
0.166951851	3.867845253	16.34036568
0.248319304	1.40789493	7.861063465
0.246602807	1.263533081	7.701492537
0.245533564	1.360436259	7.535211268
0.165895186	7.301400504	21.37266355
0.25772412	1.256919943	7.311970979
0.186633791	5.013449483	15.99293379
0.20813328	2.087253529	11.25042301
0.112461715	11.02020626	34.19668864
0.24370568	1.646265212	7.757679181
0.21589296	2.107862163	9.52621979
0.200304573	2.638993115	11.41763341
0.18693744	3.056160919	12.97788698
0.215665307	2.000600391	10.75709421
0.223359251	1.925931386	9.348277489
0.182734323	4.092103553	14.57466229
0.138412878	7.67885129	25.92750809
0.114023309	11.73989621	37.03368729
0.245556287	1.437745546	7.516129032
0.191355534	2.775402198	12.50032196
0.261395553	1.188925249	7.375634518
0.205213599	3.950852031	13.06380982
0.263347343	1.205694866	6.497757848
0.195440987	2.287202089	11.49726504
0.193553064	3.78288608	13.46644737
0.2451098	1.469968407	8.045172719
0.155676993	5.491735879	19.88119195
0.13457471	8.121345787	26.98094104
0.11862344	9.946212995	32.27647059
0.249466862	1.497944528	7.578853047
0.240546322	1.70731809	8.915846016
0.274522978	1.011351787	6.537704918
0.218465428	2.109139196	10.40249433
0.193866303	2.420578697	12.19114547
0.269557823	1.171563996	6.26984127
0.228862453	1.689923877	8.670395227
0.188444163	3.164538366	13.33954155
0.206183115	1.774554102	11.19310345
0.181722639	2.990916594	14.01503981
0.13488429	7.45495371	26.44010226
0.2402839	1.458184918	7.676092545
0.261711692	1.28140444	6.909793814
0.180208427	4.132401684	13.73224044
0.21414966	1.816780045	8.676190476
0.152916546	8.497546774	23.71245283
0.243614025	1.561163319	7.705382436
0.140352557	6.319556834	23.17612524
0.162250584	4.933814121	18.62200489
0.141122141	8.770720989	26.20702721
0.313409052	0.777890609	4.889328063
0.194732243	2.726343382	12.39967105
0.28772556	0.93198493	6.300813008
0.193336164	2.943825231	13.83905013
0.178716589	5.746355081	16.71747212

log.sigma.5.0.mm.3D_gldm_LargeDependenceLowGrayLevelEmphasis	log.sigma.5.0.mm.3D_gldm_SmallDependenceHighGrayLevelEmphasis	log.sigma.5.0.mm.3D_gldm_LargeDependenceHighGrayLevelEmphasis
0.105154401	121.3293814	2098.627479
0.216155918	54.46924581	4651.119403
0.178234755	48.49836195	1485.14977
0.050856806	98.81363605	4341.486528
0.063963121	124.8860469	3132.941504
0.124018266	54.26305064	1884.751166
1.09100876	21.24833805	1287.827893
0.075492314	136.3722332	2111.808383
0.05311382	138.7925446	3758.782588
0.200626955	57.75269784	2432.41898
0.083530652	66.97093894	2183.568319
0.203178686	54.4541625	2058.560729
0.361605513	35.1752707	2279.109932
0.0630194	78.12574107	2947.52415
0.08685609	76.11172545	2446.801317
0.301019537	36.53627816	16414.67669
0.278304362	95.64743483	2047.753582
0.216468274	81.34022917	1908.34434
0.062385732	140.5805054	4218.032646
0.385235887	29.51305882	2713.046055
0.231276163	36.02406873	5203.795942
0.168566462	58.0808317	2193.381977
0.096323829	69.01627031	2900.342365
0.207545971	63.73943063	3071.363825
0.44646809	30.57020354	1366.429402
0.116598282	101.5117707	1530.618156
0.408601374	31.55852604	8575.308425
0.349542924	45.10276567	1476.043266
0.06154021	91.34119376	2295.027762
0.255306839	54.07279029	6482.147128
0.257248035	45.04216102	1740.73671
0.147084075	65.93679433	4672.422597
0.085076132	80.78173519	3136.269552
0.37772527	53.165211	835.0096154
0.07457397	85.05338437	2414.325301
0.084494819	75.84637959	2710.243069
0.232291393	60.65520068	1755.050694
0.259576403	32.00492788	3412.812761
0.077944754	68.74627019	7017.656287
0.144383409	90.47770496	2413.322212
0.117767303	61.86518972	4811.911805
0.079386827	109.1504152	2607.390346
0.075169056	81.26439757	2270.416565
0.163902529	117.0621713	2224.844878
0.135964161	61.06445595	2850.123003
1.26233998	22.91859819	1255.386445
0.117749247	59.75288506	3686.451242
0.144399309	81.6809999	1676.180103
0.181267539	56.96035862	925.880597
0.047117778	162.9519552	3582.386318
0.212130765	50.25558969	3470.810164
0.11820462	80.34956637	1624.677146
0.114988681	75.88192801	3280.751374
0.14050692	53.92870701	2135.708968
0.331222177	39.61152478	5104.488272
0.053561114	133.423446	2337.615472
0.070302033	109.0029829	2444.764706
0.032834073	140.8326634	5794.135344
0.195044356	67.26656727	2070.64742
0.521815713	44.04277061	1581.402951
0.209425882	78.76393814	1414.361491
0.110602052	79.68029402	3619.828952
0.309842912	28.89497354	2990.784466
0.293056939	33.51699543	5763.185646
0.131674442	81.23961127	1319.963134
0.109366177	64.6134542	2322.947199
0.126487927	131.6636097	2652.659898
0.162200869	81.73451864	2725.134814
0.090101169	139.5082268	2035.038117
0.161808911	60.57985835	1782.530085
0.181596741	73.393808	2225.330263
0.096640799	93.34644654	2052.092117
0.264356395	42.77843979	2620.389319
0.185636862	47.98751128	5132.454288
0.23417471	37.50904983	5527.686928
0.063955606	145.4079657	2800.301971
0.148072324	69.99736086	1498.508505
0.177169455	102.4081753	1477.613115
0.337976219	54.74622257	1356.168367
0.070516884	83.0109214	3053.722417
0.130936715	154.3844257	1906.531746
0.08232751	82.44744075	2238.337808
0.070289341	79.68883461	3701.51361
0.202483598	27.02429502	1047.593103
0.218969195	43.75370214	1822.465939
0.232320325	38.95849037	4047.141527
0.176507792	97.77401516	1617.94473
0.018531728	226.7959121	4264.402706
0.127690828	65.21485417	2497.300546
0.259195367	59.52016138	759.3047619
0.088739719	92.14963227	7674.221635
0.320211248	89.2379822	1065.416431
0.638678674	23.84180795	2122.453033
0.206046525	40.00911172	2929.380196
0.397932735	34.14915362	3490.803996
0.073362793	197.4563889	2178.094862
0.097891258	70.08235578	3165.116776
0.756865928	39.01783198	463.0650407
0.183560217	44.55189319	2880.522427
0.268460406	93.15175353	3385.360595

log.sigma.5.0.mm.3D_gldm_SmallDependenceLowGrayLevelEmphasis	log.sigma.5.0.mm.3D_gldm_LowGrayLevelEmphasis	log.sigma.5.0.mm.3D_gldzm_DistanceZoneVariabilityNormalized
0.00378231	0.011553468	0.921185964
0.002313107	0.010498945	0.948448862
0.005127521	0.01539052	0.958639159
0.001681302	0.004883143	0.863195755
0.004231441	0.009046203	0.918515165
0.004249015	0.013898198	0.940941534
0.003987276	0.045051246	0.877077562
0.005298018	0.011231521	0.822510498
0.001674835	0.005179855	0.936991986
0.002030556	0.010642386	0.971637079
0.00464601	0.011471227	0.89188217
0.003129986	0.01478489	0.932057132
0.004729832	0.024399699	0.913579444
0.002686301	0.007960169	0.919497211
0.00298707	0.009334232	0.933190455
0.000809417	0.00517186	0.952150672
0.002835781	0.017727038	0.79204557
0.003902756	0.015963518	0.960264794
0.002707974	0.00942957	0.876067037
0.002564493	0.017350184	0.973639357
0.001501553	0.008242299	0.938558494
0.002371076	0.012147037	0.908259544
0.001628648	0.006799182	0.940030187
0.00454414	0.019134135	0.82486685
0.004261776	0.023651167	0.917088889
0.003442088	0.013394475	0.920665874
0.001605328	0.010275436	0.916988252
0.003252	0.02091835	0.965527855
0.003668307	0.008846465	0.944335432
0.002145503	0.013483575	0.971569896
0.002297796	0.01327156	0.978184472
0.001410558	0.006952435	0.978424684
0.002182098	0.008837998	0.914932982
0.011943893	0.045939117	0.878071834
0.005713079	0.011209656	0.818656875
0.003612405	0.010067004	0.892100098
0.003009324	0.015692172	0.962110021
0.002703122	0.013880283	0.967124287
0.002990336	0.007606329	0.841900888
0.00378074	0.011665196	0.95568781
0.002060976	0.007032676	0.929633253
0.002863711	0.010325129	0.883989479
0.003236147	0.009218918	0.93482137
0.003163366	0.014296217	0.85707201
0.002578795	0.010985247	0.905
0.004270023	0.036228653	1
0.001754172	0.007985101	0.912513034
0.004640177	0.016799563	0.933961575
0.008202148	0.031937832	0.834710744
0.004371763	0.008792299	0.792387543
0.002420995	0.011163886	0.943906476
0.004576909	0.014082305	0.959319885
0.002308205	0.008504397	0.912724143
0.003814192	0.015626797	0.900277008
0.001728938	0.011256499	0.958134348
0.003362881	0.008133914	0.865123967
0.002405347	0.007049003	0.803700561
0.001893486	0.004105528	0.770673668
0.003551223	0.013904996	0.904302626
0.003467503	0.022823454	0.957010023
0.003543083	0.017225179	0.928578571
0.002088914	0.008221226	0.933299832
0.00276617	0.013803802	0.97723879
0.001338926	0.009399579	0.96036864
0.008282833	0.019787225	0.959201389
0.00312514	0.009698162	0.942555502
0.002796606	0.015544732	0.798109277
0.002999545	0.01286337	0.958983215
0.007545362	0.014323749	0.876103412
0.003143235	0.01358511	0.929325997
0.002700344	0.01254915	0.91322314
0.003748257	0.012213211	0.925874507
0.002302487	0.013610332	0.962339849
0.001090621	0.006837385	0.972270508
0.001925834	0.00897585	0.957637277
0.002552985	0.008638772	0.849847712
0.004290352	0.014194903	0.861568025
0.006521329	0.023029825	0.909637707
0.005719297	0.028547802	0.865016265
0.001313244	0.005943842	0.944689859
0.003885299	0.016213016	0.835296671
0.004351536	0.010698401	0.841070901
0.001783394	0.005995973	0.930964166
0.011079209	0.026165564	0.785841761
0.003236257	0.014290194	0.980046373
0.001323277	0.008606792	0.845956913
0.003830142	0.017445877	0.936116296
0.001704856	0.003384708	0.816556079
0.010114952	0.017017144	0.896415838
0.015091897	0.033615671	0.878071834
0.001258263	0.004634719	0.923479363
0.006450071	0.027508557	0.961797942
0.004953569	0.031889342	0.888801653
0.002678532	0.012875572	0.941536961
0.003501142	0.018586922	0.956644092
0.00495155	0.015374369	0.714300502
0.002804495	0.01060422	0.978554799
0.01307094	0.080411458	0.931122449
0.003177664	0.01836489	0.818385879
0.004058211	0.018565183	0.896504875

log.sigma.5.0.mm.3D_gldzm_LowIntensityEmphasis	log.sigma.5.0.mm.3D_gldzm_LargeDistanceEmphasis	log.sigma.5.0.mm.3D_gldzm_HighIntensitySmallDistanceEmphasis
0.010761816	1.123287671	352.2714041
0.011256583	1.079429735	264.3559063
0.018371149	1.063380282	185.8838028
0.006145651	1.221570926	350.0999414
0.012759567	1.127659574	389.7801418
0.015865206	1.091370558	221.1675127
0.029490381	1.197368421	118.6151316
0.013469656	1.295302013	363.9524609
0.005675231	1.097693351	464.3510855
0.009236469	1.043165468	252.2434053
0.015815964	1.172043011	238.4480287
0.013886981	1.105633803	225.2769953
0.026310957	1.135775862	179.2042026
0.010004399	1.12605042	286.7338936
0.011255922	1.103806228	283.3226644
0.007114799	1.073578595	311.3436455
0.01222985	1.353612167	293.1321293
0.013362108	1.060836502	290.8754753
0.010711117	1.199115044	436.9568584
0.015445176	1.040076336	181.2810115
0.009831414	1.095182139	245.2388367
0.01114755	1.144578313	236.1194779
0.007300227	1.092827004	308.3895921
0.019140656	1.290909091	218.6439394
0.019853834	1.13	137.7975
0.012182265	1.124137931	286.2887931
0.014157585	1.130165289	240.0397727
0.015162686	1.052631579	200.641604
0.01085821	1.085959885	280.60351
0.012613258	1.043269231	259.9939904
0.010625672	1.033088235	206.893076
0.006523344	1.032719836	312.7690866
0.009323047	1.13354531	300.881558
0.044355263	1.195652174	135.5163043
0.017947554	1.302521008	275.7037815
0.012278765	1.17167382	260.0797568
0.012352413	1.057954545	229.4960227
0.015359471	1.050151976	182.1512158
0.014289222	1.259615385	314.7608173
0.011482192	1.068010076	280.4093199
0.009632798	1.109550562	301.3679775
0.010235997	1.185483871	326.6572581
0.010601029	1.101180438	269.4026138
0.011021632	1.232394366	334.1625587
0.01205159	1.15	246.6708333
0.024771134	1	132.1412804
0.008732508	1.137535817	274.1048233
0.016292149	1.102564103	252.1634615
0.034916639	1.272727273	169.4136364
0.012283562	1.352941176	448.7794118
0.011618338	1.086642599	236.4623947
0.013499343	1.06231454	236.4176558
0.009390135	1.13718723	294.9402502
0.018405856	1.157894737	218.0467836
0.009858407	1.064171123	224.7610772
0.009227573	1.218181818	349.8831169
0.007399119	1.330960854	324.9854686
0.006219746	1.396355353	463.6634396
0.013175546	1.151162791	259.3217054
0.017055504	1.065934066	180.3177656
0.01277282	1.111258278	263.8357616
0.00827772	1.103629977	298.0395199
0.015909763	1.034539474	170.7561678
0.010108462	1.060674157	240.1820225
0.023117943	1.0625	224.9895833
0.011764651	1.088794926	253.5480973
0.013725115	1.341772152	381.4240506
0.010949123	1.06284153	286.3909381
0.017570256	1.199052133	343.457346
0.012826878	1.110047847	230.8452951
0.010674086	1.136363636	260.4393133
0.01267556	1.115646259	293.2165533
0.011257283	1.057595993	216.1202003
0.006103445	1.0421875	289.5693359
0.011586005	1.064950263	231.4704506
0.008513034	1.245283019	391.1357442
0.014287254	1.224438903	229.7911471
0.018045774	1.14229249	288.3063241
0.025793204	1.218370884	199.1256499
0.006069845	1.08539604	341.990099
0.012908067	1.271653543	368.5954724
0.013009208	1.261121857	248.016441
0.007051912	1.107398568	314.2168854
0.045555829	1.365853659	116.5304878
0.014114344	1.030235162	200.0103583
0.008582656	1.252279635	235.4571935
0.013053082	1.099099099	277.8611111
0.004350855	1.30647482	590.0082734
0.02784249	1.164383562	186.359589
0.041149338	1.195652174	153.6195652
0.00539899	1.119544592	400.4656072
0.018055733	1.058441558	250.3449675
0.030917549	1.177272727	123.4556818
0.014020154	1.090419806	201.3148547
0.018959302	1.066508314	178.3240301
0.01437945	1.517985612	409.7589928
0.012186204	1.032520325	282.4457995
0.040464837	1.107142857	108.6830357
0.022500662	1.303030303	193.2929293
0.015364911	1.164233577	314.8987226

log.sigma.5.0.mm.3D_gldzm_LowIntensityLargeDistanceEmphasis	log.sigma.5.0.mm.3D_gldzm_HighIntensityEmphasis	log.sigma.5.0.mm.3D_gldzm_DistanceZoneVariability	log.sigma.5.0.mm.3D_gldzm_ZonePercentage
0.011747253	358.4486301	268.9863014	0.413597734
0.011852085	267.6232179	465.688391	0.236398652
0.018953288	187.7112676	136.1267606	0.32718894
0.006969749	367.2708089	736.3059789	0.323719165
0.013277908	399.4397163	129.5106383	0.39275766
0.0167401	224.0304569	185.3654822	0.306376361
0.080742535	121.0131579	66.65789474	0.225519288
0.015243085	379.1319911	367.6621924	0.446107784
0.00607026	471.5671642	690.5630936	0.354497354
0.010007286	253.5131894	810.3453237	0.269205939
0.016821461	246.4265233	248.8351254	0.337363966
0.015595112	230.0657277	397.056338	0.287449393
0.027741014	184.4849138	423.9008621	0.223722276
0.010756772	293.5658263	328.2605042	0.319320215
0.011823086	288.8685121	269.6920415	0.317233809
0.007457923	316.090301	854.0791527	0.129120484
0.014845309	309.1673004	208.3079848	0.376790831
0.014005556	292.5931559	757.6489227	0.338336192
0.011623943	450.8849558	197.9911504	0.388316151
0.015949322	182.3301527	510.1870229	0.187076044
0.010557225	249.2056404	798.7132785	0.162901991
0.012196148	245.1706827	452.313253	0.289534884
0.008391313	312.5710267	668.3614627	0.260344196
0.04574347	227.0848485	136.1030303	0.343035343
0.021860255	140.75	275.1266667	0.249169435
0.013150054	291.0034483	266.9931034	0.417867435
0.015067146	246.7541322	443.822314	0.139642239
0.015694099	202.1829574	385.245614	0.278436846
0.011471716	284.4040115	659.1461318	0.395467422
0.012913867	261.8365385	606.2596154	0.245476003
0.010874879	208.1568627	798.1985294	0.256684492
0.006704502	314.9107021	1435.349012	0.247386172
0.010020514	308.5151033	575.4928458	0.309394983
0.118149998	136.4782609	40.39130435	0.442307692
0.021437395	285.8067227	97.42016807	0.358433735
0.013105181	269.9456366	623.5779685	0.346039604
0.012822122	232.1431818	846.6568182	0.305555556
0.015817196	183.9133739	636.3677812	0.196183661
0.016359745	325.5096154	175.1153846	0.249101796
0.012179022	282.4382872	379.4080605	0.378455672
0.01011423	309.1629213	330.9494382	0.241519674
0.011369399	337.4032258	328.844086	0.390346275
0.011371288	273.2765599	554.3490725	0.361144945
0.013878624	343.0446009	365.1126761	0.415609756
0.013054426	254.1555556	488.7	0.287539936
0.024771134	132.1412804	453	0.203321364
0.009444983	282.2932187	955.4011461	0.245428973
0.017251908	255.3076923	218.5470085	0.401372213
0.109802539	170.7090909	45.90909091	0.410447761
0.013700653	475.9411765	161.6470588	0.410462777
0.012480415	239.5824308	784.3862816	0.242698598
0.014036324	238.4540059	323.2908012	0.407496977
0.010374559	301.8093184	1057.847282	0.303323737
0.02061027	222.7134503	153.9473684	0.289340102
0.01036062	227.2964095	1254.197861	0.200674536
0.010379061	362.5220779	333.0727273	0.437997725
0.010639948	338.059312	677.519573	0.384404925
0.007797148	493.4123007	338.3257403	0.339520495
0.015034632	264.0542636	233.3100775	0.316953317
0.018727066	181.7326007	261.2637363	0.309875142
0.014146368	267.6304636	701.0768212	0.356300142
0.008739783	305.691452	1594.076112	0.311792625
0.016205556	171.9638158	594.1611842	0.196763754
0.010478505	242.8314607	427.3640449	0.162943977
0.023918724	226.2708333	92.08333333	0.442396313
0.012261511	258.8520085	445.8287526	0.304571797
0.016596461	395.4746835	126.1012658	0.401015228
0.011443169	288.9489982	1052.96357	0.343446982
0.018932308	351.7677725	184.8578199	0.47309417
0.01387711	234.8835726	582.6874003	0.311785182
0.01194395	265.9235977	944.2727273	0.340131579
0.013609236	297.8866213	408.3106576	0.39061116
0.011684371	218.4015025	1152.883139	0.231811146
0.006445019	291.2328125	1244.50625	0.190589637
0.011904267	235.6606202	1636.602106	0.186165577
0.009822767	405.3102725	405.3773585	0.427419355
0.01717241	236.2418953	345.4887781	0.358997314
0.019076668	294.916996	230.1383399	0.414754098
0.044221865	203.745234	499.1143847	0.327097506
0.006496073	347.4740099	763.3094059	0.283907238
0.014598953	382.7716535	212.1653543	0.503968254
0.016667633	258.7079304	434.8336557	0.385533184
0.007401374	323.3699284	780.1479714	0.300143266
0.152649325	117.5365854	32.2195122	0.282758621
0.01446342	200.8174692	875.181411	0.263344146
0.009844713	252.0202634	834.9594732	0.180241052
0.01387288	283.1651652	311.7267267	0.428020566
0.005242887	620.9568345	567.5064748	0.447809278
0.028855444	193.7260274	65.43835616	0.398907104
0.109627599	155.673913	40.39130435	0.438095238
0.005868395	410.5009488	973.3472486	0.265157233
0.019523536	251.3506494	296.2337662	0.436260623
0.032975805	127.8227273	195.5363636	0.215264188
0.015200349	203.9354144	874.6878364	0.227139364
0.019592864	180.6191607	1208.241489	0.217533586
0.019170315	440.8920863	99.28776978	0.549407115
0.012295097	285.3360434	361.0867209	0.303453947
0.049765432	109.0178571	52.14285714	0.455284553
0.029095913	200.7171717	81.02020202	0.26121372
0.016946796	321.1094891	245.6423358	0.33952912

log.sigma.5.0.mm.3D_gldzm_IntensityVariabilityNormalized	log.sigma.5.0.mm.3D_gldzm_LowIntensitySmallDistanceEmphasis	log.sigma.5.0.mm.3D_gldzm_IntensityVariability
0.040603303	0.010515457	11.85616438
0.050816945	0.011107707	24.95112016
0.061198175	0.018225614	8.690140845
0.045451609	0.005939627	38.77022274
0.043005885	0.012629982	6.063829787
0.055064547	0.015646482	10.84771574
0.07998615	0.016677342	6.078947368
0.037480794	0.013026298	16.75391499
0.040692676	0.005576474	29.99050204
0.054894214	0.009043765	45.78177458
0.04952403	0.01556459	13.8172043
0.049053318	0.013459949	20.89671362
0.050692999	0.025953442	23.52155172
0.051275412	0.009816306	18.30532213
0.048981693	0.011114131	14.15570934
0.0518724	0.007029018	46.52954292
0.050383842	0.011575986	13.25095057
0.044475608	0.013201246	35.09125475
0.044717676	0.010482911	10.10619469
0.057120797	0.01531914	29.93129771
0.064447577	0.009649962	54.84488837
0.053757197	0.0108854	26.77108434
0.054227223	0.007027455	38.55555556
0.051092746	0.012489953	8.43030303
0.067333333	0.019352228	20.2
0.045755054	0.011940318	13.26896552
0.054717915	0.013930195	26.48347107
0.057015974	0.015029833	22.74937343
0.047700758	0.010704834	33.29512894
0.050213675	0.012538106	31.33333333
0.061403667	0.010563371	50.10539216
0.050305076	0.006478055	73.79754601
0.044330593	0.00914868	27.88394277
0.061436673	0.025906579	2.826086957
0.048795989	0.017075093	5.806722689
0.046465316	0.012072161	32.47925608
0.049000517	0.012234985	43.12045455
0.058591476	0.01524504	38.55319149
0.049879808	0.013771592	10.375
0.04553674	0.011307985	18.07808564
0.052281909	0.00951244	18.61235955
0.042548271	0.009952646	15.82795699
0.048790129	0.010408465	28.93254637
0.040611431	0.010307383	17.30046948
0.046268861	0.01180088	24.98518519
0.065138469	0.024771134	29.50772627
0.048460649	0.008554389	50.7382999
0.050807217	0.016052209	11.88888889
0.06446281	0.016195164	3.545454545
0.039023453	0.011929289	7.960784314
0.052257512	0.011402819	43.42599278
0.052769682	0.013365097	17.78338279
0.045367276	0.009144029	52.58067299
0.051810814	0.017854752	8.859649123
0.053881543	0.009732854	70.53093965
0.04310339	0.008939701	16.59480519
0.045787724	0.006588912	38.59905101
0.042709409	0.005825396	18.74943052
0.052220419	0.012710775	13.47286822
0.060070577	0.016637614	16.3992674
0.046989167	0.012429434	35.47682119
0.046771907	0.008162204	79.88641686
0.05808518	0.015835815	35.31578947
0.05674536	0.010015951	25.25168539
0.052517361	0.022917747	5.041666667
0.047106097	0.011640436	22.28118393
0.041419644	0.013007279	6.544303797
0.043533366	0.010825611	47.7996357
0.041755576	0.017229743	8.81042654
0.049741942	0.01256432	31.18819777
0.047321439	0.01035662	48.9303675
0.043608373	0.012442141	19.23129252
0.05382092	0.011150511	64.47746244
0.061185303	0.006018051	78.3171875
0.047867946	0.011506439	81.80631949
0.040764034	0.008185601	19.44444444
0.051013364	0.013565965	20.4563591
0.043915699	0.017788051	11.11067194
0.050031688	0.021186039	28.86828423
0.056979708	0.005963288	46.03960396
0.039432079	0.012485345	10.01574803
0.050533318	0.012094602	26.12572534
0.04610933	0.006964547	38.63961814
0.077929804	0.018782455	3.195121951
0.0552098	0.014027075	49.30235162
0.055241955	0.008267142	54.52380952
0.044053062	0.012848132	14.66966967
0.039722582	0.004127847	27.60719424
0.059485832	0.027589252	4.342465753
0.061436673	0.024029773	2.826086957
0.044624436	0.005281639	47.0341556
0.04579187	0.017688782	14.1038961
0.066983471	0.030402985	14.73636364
0.052927961	0.013725106	49.17007535
0.055090715	0.018800912	69.57957245
0.037937995	0.013181734	5.273381295
0.043646859	0.01215898	16.10569106
0.073979592	0.038139688	4.142857143
0.064381186	0.020851849	6.373737374
0.042330438	0.01496944	11.59854015

log.sigma.5.0.mm.3D_gldzm_HighIntensityLargeDistanceEmphasis	log.sigma.5.0.mm.3D_gldzm_SmallDistanceEmphasis	log.sigma.5.0.mm.3D_glcm_SumVariance	log.sigma.5.0.mm.3D_glcm_Homogeneity1
383.1575342	0.969178082	812.8793538	0.299269376
280.6924644	0.980142566	528.0506495	0.414190573
195.0211268	0.98415493	372.6145558	0.363769414
435.954279	0.944607268	1042.087591	0.342266164
438.0780142	0.968085106	1079.090064	0.310299244
235.4822335	0.97715736	481.161083	0.33584812
130.6052632	0.950657895	172.0732893	0.432297043
439.8501119	0.926174497	899.7829932	0.280843306
500.431479	0.975576662	1154.266166	0.327251504
258.5923261	0.989208633	493.7258272	0.38557197
278.3405018	0.956989247	628.2718524	0.314779513
249.2206573	0.973591549	502.0292895	0.350823702
205.6077586	0.966056034	404.2627231	0.382963362
320.8935574	0.968487395	796.2114914	0.339983976
311.0519031	0.974048443	677.3958297	0.341783438
335.0769231	0.981605351	769.7929051	0.55439812
373.3079848	0.911596958	708.6370967	0.308981808
299.4638783	0.984790875	585.6747692	0.332939439
506.5973451	0.950221239	1198.213067	0.298789594
186.5267176	0.989980916	308.0492472	0.431624322
265.0728555	0.976204465	495.0612856	0.458581644
281.375502	0.963855422	541.0295883	0.354907432
329.2967651	0.976793249	690.1554497	0.380080575
260.8484848	0.927272727	622.6261026	0.342870344
152.56	0.9675	224.9304212	0.408700598
309.862069	0.968965517	604.2607564	0.307664705
273.6115702	0.967458678	482.4639209	0.497964913
208.3483709	0.986842105	356.3601616	0.376839243
299.6060172	0.978510029	787.8177164	0.30998459
269.2067308	0.989182692	538.9675514	0.421771757
213.2120098	0.991727941	360.0825133	0.39494082
323.4771643	0.991820041	723.3915803	0.392848428
339.0492846	0.966613672	791.7430487	0.337861201
140.326087	0.951086957	274.5581401	0.32477898
326.2184874	0.924369748	704.5893412	0.330157984
309.4091559	0.957081545	702.8804572	0.322679343
242.7318182	0.985511364	440.2994239	0.356955759
190.9620061	0.987462006	429.9157601	0.416741584
368.5048077	0.935096154	934.215729	0.384696459
290.5541562	0.982997481	638.1137833	0.332184671
340.3426966	0.97261236	706.0040021	0.394919728
380.3870968	0.953629032	878.4029243	0.293762835
288.772344	0.97470489	706.4674987	0.312132456
378.57277	0.941901408	847.3379261	0.298551817
284.0944444	0.9625	585.4545004	0.362345651
132.1412804	1	156.2855852	0.456405482
315.0468004	0.965616046	668.4671322	0.379748535
267.8846154	0.974358974	587.6934717	0.306766285
175.8909091	0.931818182	301.5328716	0.330170283
584.5882353	0.911764706	1285.620247	0.287498761
252.0625752	0.97833935	490.9609828	0.396296943
246.5994065	0.984421365	555.2048726	0.296658604
329.285591	0.965703192	669.4273179	0.353609834
241.380117	0.960526316	500.1605914	0.364331738
237.4377387	0.983957219	450.1124915	0.464824302
413.0779221	0.945454545	873.1522485	0.293116178
390.3546856	0.917259786	789.9466003	0.305128347
612.4077449	0.900911162	1405.65336	0.323592937
282.9844961	0.962209302	515.8878538	0.360803599
187.3919414	0.983516484	390.9930144	0.353440258
282.8092715	0.97218543	510.2393687	0.31777492
336.2991803	0.974092506	769.5813542	0.356458463
176.7944079	0.991365132	333.071992	0.445120373
253.4292135	0.984831461	446.7779653	0.472995276
231.3958333	0.984375	468.4097202	0.305550247
280.0676533	0.977801268	596.0910971	0.360262105
451.6772152	0.914556962	927.5534943	0.292695948
299.1812386	0.984289617	641.1627435	0.333934112
385.0094787	0.950236967	844.3183307	0.272395117
251.0366826	0.972488038	485.6567573	0.35057943
287.860735	0.965909091	570.9119214	0.341418241
316.5668934	0.971088435	710.8441413	0.297571406
227.5267112	0.985601002	401.39336	0.401735602
297.8867188	0.989453125	592.3876248	0.434545371
252.421299	0.983762434	532.2980478	0.453905436
462.0083857	0.938679245	985.5359738	0.284138839
262.0448878	0.943890274	493.7669095	0.326460464
321.3596838	0.964426877	651.0802067	0.281712749
222.2235702	0.945407279	385.2573499	0.325244995
369.4096535	0.97865099	824.2870453	0.361439003
439.476378	0.932086614	861.832951	0.260614515
301.4738878	0.934719536	674.2836383	0.318665976
359.9821002	0.973150358	863.0291788	0.356866774
121.5609756	0.908536585	212.6027565	0.380831225
204.0459127	0.992441209	395.285482	0.375519152
318.2725431	0.936930091	520.8046378	0.432183915
304.3813814	0.975225225	613.9020068	0.295889955
744.7510791	0.923381295	1752.704979	0.274256857
223.1917808	0.95890411	477.8467344	0.376281903
163.8913043	0.951086957	243.5856726	0.344631596
450.642315	0.970113852	1014.441415	0.384987328
255.3733766	0.98538961	453.0477084	0.297119238
145.2909091	0.955681818	223.7397892	0.430215056
214.4176534	0.977395048	424.0684476	0.396032106
189.7996833	0.983372922	339.3033795	0.423578244
565.4244604	0.870503597	1207.816654	0.255940049
296.897019	0.991869919	745.0889396	0.350498496
110.3571429	0.973214286	169.622023	0.318381268
230.4141414	0.924242424	493.7769745	0.383762878
345.9525547	0.958941606	609.7595278	0.357149589

log.sigma.5.0.mm.3D_glcm_Homogeneity2	log.sigma.5.0.mm.3D_glcm_ClusterShade	log.sigma.5.0.mm.3D_glcm_MaximumProbability	log.sigma.5.0.mm.3D_glcm_Idmn	log.sigma.5.0.mm.3D_glcm_SumVariance2
0.203291396	137.3157018	0.011851262	0.976418849	136.0768818
0.338547415	224.513993	0.031037441	0.983409593	69.94907634
0.273074954	60.11727989	0.019840696	0.971695652	57.85591961
0.252476742	-8.842626932	0.011306183	0.98344701	87.40225748
0.215546311	-353.3020019	0.013618813	0.974916086	156.8814724
0.241834862	-59.00519812	0.015025442	0.974233224	70.91078041
0.353837051	156.3567183	0.043638619	0.971101052	48.62178628
0.185815343	105.1727825	0.009614337	0.9738757	151.4552263
0.235285113	136.0031185	0.01132952	0.983463083	123.0016042
0.301199283	206.4709732	0.01646621	0.984778046	72.70136413
0.220481161	-132.395975	0.013446639	0.972206565	86.24431354
0.260269548	248.9629425	0.012364572	0.981005435	95.75201559
0.296421337	361.1301411	0.01330006	0.980719526	98.15464292
0.246673797	-268.0472472	0.013718859	0.978622453	87.30180183
0.249435584	124.5032711	0.013521943	0.982309827	86.94638405
0.504823792	34.05533178	0.115680009	0.992474691	42.87561997
0.212804048	-276.9497386	0.012337365	0.975205878	106.8777708
0.243638688	451.3081802	0.012182903	0.978040949	112.6684528
0.205882205	-846.1103021	0.014594294	0.973102793	129.8187762
0.355472993	171.2783863	0.038643363	0.983855722	51.87234528
0.387720969	57.84537771	0.031115629	0.990350047	41.42297623
0.264086362	267.7659648	0.012042087	0.985038996	89.79074444
0.294439006	193.7530105	0.017535046	0.986243199	70.17557982
0.25009004	-250.7705445	0.0150204	0.973633643	103.8507569
0.326630735	60.30052332	0.018974521	0.977963346	42.37126185
0.213065545	214.6005971	0.013037508	0.974262523	96.74874814
0.43515171	136.4233775	0.085295359	0.990279835	46.49835262
0.289810265	165.1176002	0.015718998	0.981288565	71.82309794
0.214763021	-80.8121237	0.009741373	0.973613685	104.8622492
0.347285333	10.53317078	0.026436196	0.984932239	72.71285935
0.311208236	239.5405685	0.021414085	0.983734866	56.23872421
0.310940587	182.9968559	0.026583164	0.98751427	80.58293337
0.24671856	54.46238987	0.010983913	0.981078391	102.9058977
0.22900763	17.66020769	0.026369512	0.955856439	70.30494649
0.237589198	-327.9623169	0.021095018	0.972750768	88.13946716
0.22969929	-6.114925155	0.010353797	0.976654642	101.0540267
0.269250355	436.4198702	0.012746859	0.984283499	91.8845289
0.336907865	-11.50342178	0.021274602	0.984247488	67.87332641
0.300802756	-447.1130575	0.030154649	0.985601077	81.29910486
0.24049115	-24.3785204	0.014693811	0.978195535	95.42454419
0.314123789	78.896447	0.02565315	0.989238869	66.40704806
0.198303243	-74.20377814	0.011293601	0.974689893	134.3836991
0.217331968	-116.0231243	0.011327899	0.975246944	96.05783864
0.203913726	-66.10739433	0.011937205	0.975677875	138.0174106
0.274688194	284.1084437	0.013224086	0.980474107	88.94402394
0.385893169	196.6898366	0.033226034	0.985080021	42.11351419
0.293076506	50.36791818	0.013329598	0.985795634	77.0671614
0.211212162	-124.2529636	0.012615608	0.971156598	102.1485766
0.233462628	-23.02001536	0.026362059	0.959083546	56.21776387
0.193457214	-433.9148253	0.01509804	0.980186355	150.3578733
0.31340841	194.3042172	0.02584686	0.98443739	73.1441561
0.200915746	-96.66565507	0.011564723	0.960877572	96.69135332
0.266235301	329.7178661	0.018607496	0.984311818	84.47285449
0.274916577	-70.58043237	0.018874489	0.979449207	68.72359661
0.398935432	254.4714438	0.066495825	0.985248177	61.84985111
0.197979325	-74.45103901	0.011181865	0.977633609	111.4513357
0.210568081	36.7521789	0.009275286	0.976359487	98.49864929
0.2323416	-288.4845922	0.015163754	0.98524843	112.6895525
0.270288528	225.4949336	0.016420117	0.980556393	93.09286126
0.258797581	-84.84799162	0.015348986	0.974988571	65.90491399
0.225245679	362.682214	0.010709648	0.978442782	109.1004042
0.26903535	390.6512623	0.015059756	0.985781258	107.6997655
0.371844484	182.6947093	0.037041732	0.987333774	45.63307273
0.40826232	186.9766685	0.042628652	0.98604763	44.63327253
0.207654138	196.8070446	0.017442937	0.966896111	94.71860243
0.273332109	342.2405691	0.013945978	0.982538088	85.93199032
0.197996419	-436.2850941	0.015636047	0.97158722	136.9016252
0.245735145	273.4888926	0.017515524	0.977047008	119.4318754
0.175826283	-399.6394138	0.01629626	0.96769074	125.5507102
0.260840374	288.351443	0.013035368	0.979113053	87.44209743
0.252582969	250.3669317	0.013024996	0.979314763	97.76362143
0.202300588	149.2618991	0.009179848	0.972782179	122.5025262
0.322177434	253.6213727	0.022043119	0.984352554	65.9723477
0.360496261	141.2357667	0.022158026	0.988483442	53.19775787
0.384766595	399.9906125	0.038361897	0.988914857	65.02041925
0.189053876	42.2118664	0.010274451	0.979533508	123.4717793
0.233083296	55.15532366	0.010070359	0.97838927	87.41313292
0.185525094	42.35539003	0.011588287	0.9698114	129.8303875
0.233615284	267.1041452	0.01127292	0.972755196	94.30816118
0.27136109	19.25534413	0.013273703	0.985093083	68.45327278
0.166696328	-265.7080779	0.012870789	0.967129023	150.5463934
0.223804935	-107.0533261	0.012126454	0.979614875	96.30414927
0.270398589	340.0955891	0.018163741	0.98460275	95.0849634
0.290105234	-0.326205999	0.036685205	0.963412551	33.1015742
0.288511515	243.7695326	0.015157892	0.981545475	67.2508272
0.356968054	257.1954608	0.023802338	0.98703363	57.18158055
0.200521598	306.4013506	0.0103463	0.975699485	127.9170012
0.17931596	-456.8247389	0.007498139	0.975829889	154.0659947
0.288714434	37.29698233	0.034246697	0.97168942	63.56063279
0.25085293	325.7359858	0.032992897	0.965839716	63.57043062
0.304898691	180.7524243	0.034662461	0.986004607	80.67419128
0.2020163	374.7124156	0.010008615	0.974188395	117.240193
0.352683065	117.4261665	0.025715189	0.979796693	51.32086502
0.313286558	212.4729575	0.016730398	0.98481902	63.79425275
0.346449421	120.3029724	0.033831924	0.984549206	62.3492422
0.159095186	-1109.410851	0.015190134	0.968856741	183.302949
0.260347841	20.53912025	0.014037298	0.979363376	93.15089772
0.223565209	57.5119679	0.024869424	0.939120505	48.96989158
0.295233228	-202.4719646	0.023894378	0.978794763	69.8497848
0.27135847	221.8228213	0.02622423	0.981326975	126.0421739

log.sigma.5.0.mm.3D_glcm_Contrast	log.sigma.5.0.mm.3D_glcm_DifferenceEntropy	log.sigma.5.0.mm.3D_glcm_InverseVariance	log.sigma.5.0.mm.3D_glcm_Entropy	log.sigma.5.0.mm.3D_glcm_Dissimilarity
27.37864345	3.345446494	0.207495278	8.318367533	4.206891962
14.8414066	3.01667857	0.316052863	7.705211859	2.827524058
14.86025717	2.949224107	0.273221039	7.304386773	3.055261034
20.08927332	3.210764156	0.258204571	8.227142716	3.520778983
25.97795438	3.347773105	0.219649781	7.95262524	4.039580407
18.72528299	3.139743479	0.24503732	7.755929602	3.465462674
9.268533185	2.602481787	0.340017822	6.702690854	2.305678861
34.43398207	3.571621456	0.195918098	8.688585771	4.696366155
23.88640783	3.35778519	0.24279796	8.534790993	3.798764376
14.43815177	3.025930736	0.300945816	7.856782881	2.905001542
23.36509804	3.26307099	0.220768542	7.993132791	3.911137768
18.14302133	3.14092064	0.262663811	8.089566224	3.335522318
13.95286114	2.9784106	0.296374111	7.964205322	2.872552253
17.80177888	3.12215151	0.258705833	8.003529147	3.366340868
19.19541108	3.1776319	0.255904553	7.974317596	3.433475075
8.079415816	2.560053329	0.362616928	6.66533334	1.799749456
23.89536755	3.240127506	0.220765407	8.0921786	3.934886474
24.0916002	3.318601573	0.24703532	8.399384837	3.806757528
31.59364334	3.485572669	0.204759193	8.208253554	4.455863349
10.69287079	2.834524977	0.331814055	7.367572047	2.446760754
8.422277432	2.700886936	0.360573189	7.155671094	2.150900007
17.12689743	3.112271961	0.267507704	8.022826409	3.235055127
14.7671458	3.025659168	0.297952053	7.829519232	2.946721049
19.28025068	3.174671235	0.24248917	7.845469615	3.46584136
10.41349774	2.802248058	0.328744261	7.21391072	2.505783452
26.65716864	3.387265411	0.221339616	8.120806824	4.091103584
7.987583613	2.650175266	0.3621171	6.98955883	1.977139571
14.46105134	3.006199416	0.288731473	7.76205153	2.959949443
23.71977839	3.268289246	0.218391907	8.293267023	3.952033934
14.36334692	3.014013624	0.319208413	7.773452392	2.760275221
12.50837903	2.933953892	0.310936237	7.574557333	2.728307453
15.98084912	3.077194428	0.286067149	7.993054052	3.009942699
20.49060337	3.230963776	0.25326969	8.317036617	3.560491719
19.67173513	3.077904222	0.228133116	6.485045253	3.616560609
21.49369812	3.208350503	0.242371138	7.542456586	3.669486224
22.37728863	3.277226244	0.235425636	8.305217402	3.783879966
19.11241818	3.199062727	0.268355967	8.100045386	3.370247695
10.35969386	2.817144471	0.332517852	7.605244596	2.473288844
15.48955912	3.051050286	0.291415924	7.575871138	2.99676718
22.41411849	3.284210986	0.243267389	8.138849802	3.705018971
15.43345049	3.051825407	0.310891081	7.702494552	2.909496826
29.56351074	3.449029707	0.202949382	8.480985855	4.364637581
23.83763817	3.262427812	0.227247638	8.23665235	3.917250891
28.4417406	3.424249329	0.219218362	8.519397921	4.24231407
17.51673954	3.136479085	0.276270394	8.061125338	3.232413613
9.104671946	2.713890718	0.356697314	6.954597445	2.218130089
14.32409172	3.033470686	0.293037738	7.996297408	2.923119572
24.37535922	3.281644106	0.219200688	8.023478408	3.995570027
18.25531201	3.035681559	0.238633507	6.716019369	3.457270605
33.56210145	3.547424343	0.200806929	8.216481141	4.610207804
13.77781831	3.002288193	0.296339889	7.83019764	2.835681582
26.92861325	3.330726404	0.206866421	8.041618114	4.224798374
20.19642172	3.235362088	0.262315779	8.191958628	3.469101338
14.78076326	3.008145262	0.288483351	7.592688181	3.026755669
13.22190021	2.912273613	0.325167511	7.375619927	2.523130532
29.12941152	3.444177337	0.211152089	8.370005972	4.335103952
27.58130149	3.341973784	0.216249865	8.397591837	4.165031386
24.66565154	3.350691515	0.236229097	8.308321162	3.901295407
16.19200432	3.068214196	0.271861381	7.901619759	3.145250491
14.12698076	2.896392852	0.263783283	7.592194311	3.052446875
25.04643523	3.379762118	0.234001347	8.402732571	3.943257488
19.26396173	3.217781901	0.264114361	8.384029909	3.399926212
9.655778931	2.754130696	0.342124625	7.179094714	2.320264508
10.02181443	2.742545378	0.362192803	7.035779632	2.228387277
24.48917815	3.269792738	0.207140294	7.396584387	3.994434978
17.70851578	3.143046082	0.280147378	8.007477382	3.264753361
31.58234143	3.465717329	0.201202018	8.050442619	4.473352763
25.25504874	3.387668224	0.243465111	8.540275187	3.881956288
33.61240574	3.477231816	0.182381479	8.109705296	4.757167253
18.70095609	3.181577707	0.26030493	8.091078874	3.387724516
22.59612627	3.265628181	0.248061102	8.292887028	3.682660124
28.14163132	3.42108304	0.206219217	8.462515252	4.271662112
14.89528929	3.04935273	0.304324144	7.797037805	2.884385326
11.55256603	2.880787258	0.332399985	7.459512829	2.503474427
11.20844152	2.853251815	0.350926179	7.476259633	2.375381575
32.93502189	3.48729003	0.197557497	8.503828761	4.605596644
20.67694528	3.187975058	0.250989509	8.03682297	3.622281495
31.35906257	3.466887804	0.195084319	8.295940209	4.552357812
23.02082656	3.275755533	0.236870177	8.19504031	3.813785566
14.9485348	3.030575459	0.281567729	7.855149835	3.065275854
43.93030966	3.63719599	0.172796336	8.337493038	5.338491903
22.08814127	3.266808006	0.233235917	8.191590754	3.768953116
19.79108524	3.217557477	0.267886286	8.21294303	3.42862444
11.68098019	2.792276307	0.285198026	6.456840029	2.739559114
15.38414499	3.047247194	0.283250641	7.82578009	3.02666349
10.67920116	2.831566531	0.341442826	7.442402015	2.430963424
28.25734314	3.412611188	0.206564635	8.328235729	4.286246493
37.29783348	3.621750178	0.186459863	8.837075274	4.898151365
13.56512098	2.74445014	0.29225706	6.685163769	2.903432256
18.34106276	2.963954421	0.243758519	6.337914629	3.384510449
19.06076291	3.19779209	0.283853051	8.074145922	3.250207722
30.56448577	3.480298447	0.208510609	8.233173168	4.35017177
9.523553456	2.752903264	0.339057211	7.232134877	2.35556018
13.4586305	2.977231093	0.307461257	7.735411688	2.792125109
11.92337001	2.917706761	0.323502606	7.618424435	2.570772219
38.99244296	3.52022689	0.175553232	7.796557022	5.116412909
18.5168091	3.177028812	0.262650988	8.081311342	3.362500536
20.58505686	3.068133061	0.241548653	6.644223111	3.684216624
11.92987049	2.868580932	0.297841827	7.322661989	2.734939332
21.70374985	3.242613937	0.265596558	8.055467063	3.522867066

log.sigma.5.0.mm.3D_glcm_DifferenceVariance	log.sigma.5.0.mm.3D_glcm_Idn	log.sigma.5.0.mm.3D_glcm_Idm	log.sigma.5.0.mm.3D_glcm_Correlation	log.sigma.5.0.mm.3D_glcm_Autocorrelation
8.483833957	0.892842325	0.203291396	0.667293452	275.2496682
6.354227216	0.91685829	0.338547415	0.651610945	188.5084134
4.913422894	0.885356596	0.273074954	0.588405966	138.297095
6.902009812	0.910871889	0.252476742	0.627158412	343.3679069
8.837019591	0.891248901	0.215546311	0.718248068	353.764918
6.32169871	0.888861537	0.241834862	0.583530853	172.7103688
3.26604829	0.889079813	0.353837051	0.680649899	71.7758062
11.72695838	0.888245873	0.185815343	0.631409273	300.4672495
8.954094087	0.911717957	0.235285113	0.675009399	377.8982683
5.670012864	0.916435643	0.301199283	0.67080804	178.6053734
7.188738581	0.884142283	0.220481161	0.576373589	218.0132614
6.346349549	0.905282794	0.260269548	0.683033375	180.9622236
5.33398328	0.906205553	0.296421337	0.74951608	150.6273579
6.09849345	0.89825016	0.246673797	0.663129587	270.4187176
6.85350492	0.908082339	0.249435584	0.641918335	234.4120175
4.668865048	0.950363558	0.504823792	0.68489403	257.382579
7.130337639	0.890179052	0.212804048	0.638628672	243.3813655
8.666583603	0.899955242	0.243638688	0.650255597	206.3068194
10.44841955	0.888020555	0.205882205	0.615509719	386.9326465
4.510794963	0.916147852	0.355472993	0.657967548	118.8312657
3.658803269	0.934364769	0.387720969	0.661839215	177.3650863
6.172948461	0.915054156	0.264086362	0.681757103	193.3349022
5.706612849	0.919952739	0.294439006	0.655575041	238.3678231
6.693524602	0.889320031	0.25009004	0.687902858	217.2338154
3.877335831	0.899667652	0.326630735	0.60541968	90.8250335
9.253440174	0.890115515	0.213065545	0.570937912	210.2657647
3.95140838	0.937927994	0.43515171	0.706371257	173.5167599
5.256309355	0.906591721	0.289810265	0.665299083	134.6623759
7.137559095	0.88639274	0.214763021	0.631478572	267.6321959
6.435439777	0.921064575	0.347285333	0.670603565	192.5721883
4.768000619	0.913111612	0.311208236	0.636266818	135.5418695
6.344332079	0.92495438	0.310940587	0.671792627	249.2717443
7.250805896	0.90513908	0.24671856	0.669580977	270.1029604
5.990904382	0.856534391	0.22900763	0.556509519	102.441621
7.206258761	0.887439358	0.237589198	0.610376674	240.0763751
7.443443955	0.893989107	0.22969929	0.638689234	242.2594754
7.336572144	0.914568418	0.269250355	0.656981968	160.8993375
4.031303707	0.914792361	0.336907865	0.735259956	159.1147009
6.016074703	0.918925399	0.300802756	0.682130988	309.8493531
8.190187468	0.899287661	0.24049115	0.622609957	222.0416937
6.648450891	0.930838448	0.314123789	0.627355596	242.3122202
9.600841918	0.889501343	0.198303243	0.641250148	294.5331055
7.367561459	0.890720221	0.217331968	0.606925594	242.5969658
9.507536609	0.892370994	0.203913726	0.662104316	285.9622954
6.617443523	0.905426894	0.274688194	0.670894639	206.8003455
3.931106843	0.92054778	0.385893169	0.642744228	66.84176161
5.510924121	0.918135449	0.293076506	0.687428041	232.9683902
7.574048368	0.882002776	0.211212162	0.617169721	205.6956137
5.74823081	0.861873039	0.233462628	0.510226597	112.0924428
11.39083022	0.901913225	0.193457214	0.638298546	413.4611926
5.423797581	0.91577624	0.31340841	0.683540916	177.8894299
7.985173016	0.864202738	0.200915746	0.566552671	194.2818002
7.582433973	0.914557292	0.266235301	0.616030107	231.9349993
5.321032263	0.901352799	0.274916577	0.64741166	179.6563644
6.432285283	0.925752028	0.398935432	0.653394178	163.2101965
9.496982838	0.895450144	0.197979325	0.587960462	292.2160526
8.381994516	0.894134258	0.210568081	0.56953971	267.3550612
8.626254904	0.915378248	0.2323416	0.645846656	449.0371952
5.901590581	0.904498877	0.270288528	0.703378912	185.6359779
4.233181009	0.888830916	0.258797581	0.647416254	145.5012098
9.006521534	0.899115229	0.225245679	0.627963208	181.9783526
7.21108334	0.918004224	0.26903535	0.699025536	264.1107638
3.929824954	0.925162861	0.371844484	0.651553193	126.4648842
4.794227023	0.926392345	0.40826232	0.631479259	161.7288418
7.995500924	0.874609538	0.207654138	0.589224084	166.0413166
6.583515046	0.909848685	0.273332109	0.659516374	209.851354
10.48105083	0.884537738	0.197996419	0.626314813	307.3768717
9.622119803	0.898425208	0.245735145	0.650818998	223.4508536
9.876449022	0.874141357	0.175826283	0.581606936	281.5664716
6.712082237	0.901223289	0.260840374	0.649287029	175.5041452
7.830257292	0.902770901	0.252582969	0.628841088	201.6817473
9.026184165	0.885553948	0.202300588	0.628871632	243.9161339
6.174446459	0.917451826	0.322177434	0.634781214	148.8376689
5.049264194	0.929348995	0.360496261	0.644169182	207.6652874
5.377219541	0.933073964	0.384766595	0.707637695	189.7357893
10.02665942	0.899634398	0.189053876	0.581072327	325.4535991
6.679370748	0.897922757	0.233083296	0.619963994	177.2291003
9.737098188	0.878973968	0.185525094	0.61524108	224.2978313
7.506366237	0.88713632	0.233615284	0.607816797	142.0806963
5.20513224	0.914175467	0.27136109	0.64371981	278.3223311
13.34437419	0.875490064	0.166696328	0.5525935	285.3676097
7.22516216	0.899881909	0.223804935	0.629288803	233.1975361
7.386510829	0.91546573	0.270398589	0.656679583	290.849778
3.838906155	0.869981247	0.290105234	0.481464001	84.13546111
5.698895368	0.907899804	0.288511515	0.62745589	146.7795634
4.489174982	0.924520803	0.356968054	0.685209405	186.1555165
9.172016946	0.891046611	0.200521598	0.640955978	213.6859181
12.27258283	0.891906056	0.17931596	0.61484591	548.6792097
3.703699808	0.885977181	0.288714434	0.637705713	169.2539764
5.922960516	0.875293259	0.25085293	0.558107447	91.84262132
7.900883442	0.92192482	0.304898691	0.618724838	334.1735437
10.90273562	0.890351733	0.2020163	0.590436935	161.709173
3.738975682	0.905268954	0.352683065	0.685809771	90.86075881
5.278950812	0.916948978	0.313286558	0.652789893	156.355301
5.086272381	0.91821412	0.346449421	0.67786115	129.3471752
10.65780868	0.876156751	0.159095186	0.649374952	386.977364
6.77979442	0.901886277	0.260347841	0.67032844	255.5913458
6.229017311	0.834719104	0.223565209	0.415055624	67.01473094
4.162647125	0.899415853	0.295233228	0.710268842	177.6661123
8.307632763	0.909384051	0.27135847	0.710994663	213.794344

log.sigma.5.0.mm.3D_glcm_SumEntropy	log.sigma.5.0.mm.3D_glcm_AverageIntensity	log.sigma.5.0.mm.3D_glcm_Energy	log.sigma.5.0.mm.3D_glcm_SumSquares	log.sigma.5.0.mm.3D_glcm_ClusterProminence
5.485070004	15.89047278	0.003854563	41.77138941	49553.73071
5.033874943	13.25763097	0.007929952	21.75784147	17758.43356
4.845247031	11.29140072	0.007778687	18.1790442	8044.329959
5.242825476	18.20408133	0.004365308	27.05481467	22596.12383
5.467453089	18.00434396	0.004781391	46.07806181	55812.98229
5.016911935	12.63655397	0.005592841	22.44924559	12654.85594
4.628488572	7.868172366	0.012771403	14.47257987	5835.375706
5.581458085	16.6369937	0.00297621	46.76553249	57236.02609
5.469533513	18.93650084	0.003623993	36.47669798	44022.2966
5.096731473	12.84760169	0.005857812	22.32628427	15101.13981
5.16430447	14.25885903	0.004788845	27.9021674	17903.26866
5.264832411	12.75882942	0.004602341	29.1942166	24567.53663
5.270165428	11.38631464	0.005068731	28.11140152	24882.79676
5.189344686	15.9499961	0.004880404	26.75331529	19694.13996
5.194703442	14.85525062	0.005057802	27.54244517	22666.13502
4.577883998	15.80949121	0.028776478	13.0283182	8951.098653
5.310779653	15.00846717	0.004553759	33.69301737	29712.07999
5.392356349	13.67758823	0.003877322	35.31062672	35231.65706
5.386013108	19.2715498	0.004373082	40.3588374	49632.38662
4.830584516	10.41786455	0.009136021	15.64130402	9233.454943
4.709980451	13.0115007	0.010666686	12.56564136	5984.690878
5.227924154	13.29850527	0.004841681	27.37221032	23837.74011
5.068212504	15.05931681	0.006376516	21.90139846	15784.28757
5.230108836	14.00847992	0.005286539	30.88632904	23674.45149
4.691260159	9.100866325	0.008546964	13.1961899	4730.614212
5.237967865	14.00566139	0.004505094	32.17002097	24366.27007
4.724005595	12.80868511	0.017050625	13.7402684	8150.117143
5.070193143	10.97412302	0.005996951	21.68954095	14380.85849
5.320885227	15.80446254	0.003804361	32.93846339	24325.37248
5.089272793	13.38581555	0.007503104	22.36795914	16496.41456
4.894495178	11.16696511	0.007353132	17.27897382	11068.99654
5.182770523	15.4105874	0.005868523	24.91955344	19415.63497
5.345245074	15.90382241	0.003996375	31.61578541	28373.55231
4.658536904	9.47241786	0.012339407	22.4941704	10152.25357
5.065295271	14.96799082	0.006723932	27.73579884	21482.06244
5.306603432	14.99922629	0.003831605	31.69735907	23581.16489
5.226040093	12.02764779	0.004774566	28.74567923	24425.11864
5.033849346	12.03031429	0.006926313	19.55825507	11594.84983
5.052834441	17.20751242	0.008347441	24.57623393	22819.15043
5.255064753	14.34610583	0.004653532	30.19218576	25215.76507
5.012756812	15.30141566	0.007360426	21.13333622	14473.37724
5.484432537	16.51518206	0.003448625	41.56193309	44831.1982
5.26289804	15.05491905	0.004023856	30.65039577	21549.96654
5.527202353	16.2116463	0.003447041	42.41802936	49810.75155
5.208629974	13.77683308	0.004901845	27.05126573	21326.19612
4.623182037	7.653516739	0.012130799	12.80454653	5749.113106
5.162224215	14.80488845	0.005237539	23.4823245	16289.73032
5.259853166	13.68658221	0.004601608	32.26364307	24647.19193
4.596320971	10.12514272	0.010816178	18.61826897	7423.862858
5.511709672	19.79066024	0.004309324	44.87692992	69888.31595
5.097453249	12.79368562	0.006153365	22.13168648	15621.4211
5.183190911	13.29662482	0.004446001	30.90499164	18703.85302
5.198813976	14.81993365	0.004849414	26.96534924	23628.08038
5.010340919	12.8906131	0.006711057	20.90021377	13677.38444
4.876478412	12.31707648	0.014747824	19.19608551	14332.5083
5.363825396	16.67788389	0.003766225	35.6840052	32299.77212
5.302796678	16.00544525	0.003621418	32.59723421	24420.57898
5.371602392	20.8545047	0.004403126	33.67595612	38187.46618
5.238052286	12.92639278	0.005427556	27.79787528	23557.77687
4.996323124	11.51167655	0.006387295	20.00797369	10833.61261
5.346027013	12.77568502	0.003674144	34.50742075	29014.35373
5.385818725	15.6593642	0.004089754	32.152898	37237.67009
4.722553603	10.84494779	0.011096228	13.96578613	8766.06981
4.690943208	12.37553043	0.012801183	13.72961313	7074.810139
5.039835454	12.21447585	0.006816797	30.40481934	22722.57045
5.183667468	13.97726606	0.005204158	26.93211533	20726.22265
5.410799984	16.82962505	0.004701556	42.48424041	48912.76682
5.436113764	14.22250799	0.003589803	37.02031281	37384.3172
5.351400415	16.22552083	0.004481674	40.77168396	40521.57122
5.209632859	12.6174917	0.004762914	27.09734958	20416.77874
5.295315609	13.61977639	0.00417696	31.06257603	24196.57245
5.431099994	14.9556772	0.003394046	38.6651759	36690.44831
5.015103917	11.69679882	0.006620812	20.72471276	13749.97213
4.869117861	14.10294554	0.008129164	16.7807464	8315.032769
4.937851015	13.33363557	0.011174529	19.76305183	16693.62701
5.441541872	17.64641843	0.003422814	38.97295234	43803.23856
5.182759367	12.70895413	0.004510913	27.55350891	18071.78432
5.430781883	14.30025302	0.003767062	42.09972965	39144.85726
5.236642655	11.17282531	0.004174867	29.84754427	21226.90538
5.061783535	16.38570698	0.005475955	21.5682609	13245.61466
5.498647952	16.32163089	0.003755905	49.54828916	57775.28451
5.260133611	14.74808649	0.00414346	30.39113559	21322.42573
5.274692228	16.65323949	0.004767615	29.12825258	28325.97131
4.353893122	8.873570658	0.013685576	11.1956386	2725.835133
5.023636485	11.57648771	0.005742938	20.83252469	13121.74085
4.889980782	13.22930402	0.008815807	17.25621533	10408.74956
5.433838286	13.85949527	0.003729902	40.22006813	40955.44762
5.60154661	22.99748581	0.002690624	46.68001334	58473.70943
4.68644598	12.51931238	0.011955341	19.28143844	8182.902132
4.530774594	8.968285469	0.013924387	20.47787334	12586.40094
5.150636063	17.98781413	0.006535856	24.97467775	23724.83247
5.342853127	11.95676261	0.003923229	38.54793631	34274.2798
4.803422707	8.966848392	0.008832541	15.21110462	6992.866703
4.999958864	12.01016682	0.006571518	19.63864294	13272.9747
4.96729092	10.80727831	0.007692423	18.63061463	11203.20812
5.455945107	18.98994034	0.005187531	55.67582146	82480.42424
5.252428816	15.44990971	0.004783713	28.57395653	22627.76208
4.498642827	7.737337837	0.011008033	17.38873711	5144.726802
4.959654486	12.77308406	0.007803568	20.44491382	12153.02336
5.408018476	13.85505916	0.005315329	38.59497524	43845.62831

log.sigma.5.0.mm.3D_glcm_SumAverage	log.sigma.5.0.mm.3D_glcm_Imc2	log.sigma.5.0.mm.3D_glcm_Imc1	log.sigma.5.0.mm.3D_glcm_DifferenceAverage	log.sigma.5.0.mm.3D_glcm_Id
31.49846318	0.928944814	-0.217950854	4.206891962	0.299269376
26.43679515	0.844988132	-0.161177841	2.827524058	0.414190573
22.58280145	0.888046531	-0.200823467	3.055261034	0.363769414
36.14028714	0.806781356	-0.132253008	3.520778983	0.342266164
35.8318333	0.969190886	-0.301871538	4.039580407	0.310299244
25.26886275	0.863265878	-0.166953099	3.465462674	0.33584812
15.73634473	0.909033977	-0.246796486	2.305678861	0.432297043
32.93328468	0.893986113	-0.173271227	4.696366155	0.280843306
37.58217045	0.854809957	-0.150525214	3.798764376	0.327251504
25.61534305	0.819610797	-0.142990749	2.905001542	0.38557197
28.44236205	0.869848888	-0.167159495	3.911137768	0.314779513
25.42001043	0.863794988	-0.16678827	3.335522318	0.350823702
22.76513438	0.877687482	-0.182918078	2.872552253	0.382963362
31.81382358	0.856621752	-0.160370939	3.366340868	0.339983976
29.4933096	0.883158677	-0.178342453	3.433475075	0.341783438
31.53916847	0.873750339	-0.207278067	1.799749456	0.55439812
29.8369449	0.907255432	-0.200509966	3.934886474	0.308981808
27.14021941	0.844698355	-0.14952736	3.806757528	0.332939439
38.0686435	0.924629846	-0.214735809	4.455863349	0.298789594
20.8357291	0.811257894	-0.146113364	2.446760754	0.431624322
26.00864154	0.794187024	-0.140935386	2.150900007	0.458581644
26.46952772	0.856534298	-0.162724965	3.235055127	0.354907432
29.96726745	0.817455852	-0.141265166	2.946721049	0.380080575
28.0048247	0.922994016	-0.223282112	3.46584136	0.342870344
18.20173265	0.785841379	-0.136255053	2.505783452	0.408700598
27.76402576	0.888843127	-0.178341365	4.091103584	0.307664705
25.60335376	0.856483241	-0.183581414	1.977139571	0.497964913
21.93648327	0.843103185	-0.159674565	2.959949443	0.376839243
31.45200183	0.843898904	-0.150062434	3.952033934	0.30998459
26.68181055	0.85674404	-0.16538388	2.760275221	0.421771757
22.32508447	0.777812844	-0.127919981	2.728307453	0.39494082
30.53591195	0.820409495	-0.146883831	3.009942699	0.392848428
31.59061235	0.839998573	-0.146930592	3.560491719	0.337861201
18.94483572	0.978550142	-0.395596144	3.616560609	0.32477898
29.88826275	0.941607523	-0.256129238	3.669486224	0.330157984
29.83814701	0.829275042	-0.139335555	3.783879966	0.322679343
23.89155192	0.823920111	-0.141337861	3.370247695	0.356955759
24.06062858	0.846759209	-0.164288405	2.473288844	0.416741584
34.25614932	0.89464676	-0.202865817	2.99676718	0.384696459
28.54952861	0.868184776	-0.163307451	3.705018971	0.332184671
30.30223353	0.845515582	-0.157963265	2.909496826	0.394919728
32.75923008	0.892505006	-0.177928673	4.364637581	0.293762835
29.96765679	0.834710079	-0.144896677	3.917250891	0.312132456
32.15784907	0.897137865	-0.180327519	4.24231407	0.298551817
27.49055889	0.844511136	-0.153782464	3.232413613	0.362345651
15.30703348	0.799826018	-0.151758421	2.218130089	0.456405482
29.48088477	0.813322197	-0.136316061	2.923119572	0.379748535
27.28967815	0.9112381	-0.202888725	3.995570027	0.306766285
20.25028545	0.967198545	-0.342717689	3.457270605	0.330170283
39.19895964	0.949634608	-0.248664239	4.610207804	0.287498761
25.53731295	0.825741867	-0.147419258	2.835681582	0.396296943
26.59324963	0.860472461	-0.160755771	4.224798374	0.296658604
29.38424826	0.78430202	-0.121958088	3.469101338	0.353609834
25.77881178	0.885542526	-0.188780735	3.026755669	0.364331738
24.58043792	0.84711458	-0.168775292	2.523130532	0.464824302
32.96075745	0.871279842	-0.163538479	4.335103952	0.293116178
31.5963277	0.80649872	-0.132038235	4.165031386	0.305128347
41.32635713	0.876626759	-0.169171538	3.901295407	0.323592937
25.79711716	0.897524962	-0.194074324	3.145250491	0.360803599
23.02335309	0.856122332	-0.174803812	3.052446875	0.353440258
25.37278839	0.817767735	-0.131200294	3.943257488	0.31777492
31.11225434	0.829079837	-0.140461431	3.399926212	0.356458463
21.67634352	0.804332888	-0.149872074	2.320264508	0.445120373
24.74398894	0.837666532	-0.170861142	2.228387277	0.472995276
24.36438012	0.961849109	-0.300914795	3.994434978	0.305550247
27.76887128	0.846398031	-0.154578036	3.264753361	0.360262105
33.51934308	0.957310259	-0.271956786	4.473352763	0.292695948
28.27692978	0.822804767	-0.132873286	3.881956288	0.333934112
32.15742214	0.938645635	-0.234784846	4.757167253	0.272395117
25.16460333	0.832620388	-0.143860843	3.387724516	0.35057943
27.04693215	0.813483443	-0.136227564	3.682660124	0.341418241
29.68313832	0.877808592	-0.164823467	4.271662112	0.297571406
23.32925828	0.784992831	-0.128106974	2.884385326	0.401735602
28.0891358	0.799135458	-0.139208376	2.503474427	0.434545371
26.55367398	0.833675471	-0.157614484	2.375381575	0.453905436
34.79930365	0.881445284	-0.168516631	4.605596644	0.284138839
25.33816316	0.855247609	-0.158566253	3.622281495	0.326460464
28.25727295	0.919925921	-0.205304465	4.552357812	0.281712749
22.29144856	0.817481654	-0.138430018	3.813785566	0.325244995
32.55400964	0.803567998	-0.13401003	3.065275854	0.361439003
32.16092125	0.948591362	-0.245943343	5.338491903	0.260614515
29.3002737	0.83796309	-0.145961437	3.768953116	0.318665976
32.98618727	0.829454961	-0.144575933	3.42862444	0.356866774
17.74714132	0.918250154	-0.255196762	2.739559114	0.380831225
23.1345999	0.778626994	-0.127677115	3.02666349	0.375519152
26.42129422	0.80770125	-0.146380152	2.430963424	0.432183915
27.47604259	0.906802512	-0.192453521	4.286246493	0.295889955
45.58001892	0.863409892	-0.149294614	4.898151365	0.274256857
25.03862476	0.95893663	-0.324390296	2.903432256	0.376281903
17.93657094	0.982267399	-0.424143722	3.384510449	0.344631596
35.70812883	0.807517894	-0.134761756	3.250207722	0.384987328
23.66420383	0.90480575	-0.189724491	4.35017177	0.297119238
17.93369678	0.849462796	-0.174299709	2.35556018	0.430215056
23.9805877	0.791985446	-0.132904279	2.792125109	0.396032106
21.60832328	0.808745038	-0.141217231	2.570772219	0.423578244
37.46097077	0.982308701	-0.354598847	5.116412909	0.255940049
30.78355464	0.869592396	-0.166437698	3.362500536	0.350498496
15.47467567	0.95612089	-0.313660064	3.684216624	0.318381268
25.54616813	0.920208316	-0.23251352	2.734939332	0.383762878
27.40017105	0.933640437	-0.232074617	3.522867066	0.357149589

log.sigma.5.0.mm.3D_glcm_ClusterTendency	log.sigma.5.0.mm.3D_firstorder_InterquartileRange	log.sigma.5.0.mm.3D_firstorder_Skewness	log.sigma.5.0.mm.3D_firstorder_Uniformity
136.0768818	219.6123695	0.120951329	0.043259315
69.94907634	137.5040092	0.40750604	0.065741694
57.85591961	164.8047719	0.079767584	0.063762237
87.40225748	187.4931297	-0.029109475	0.051829661
156.8814724	254.7696991	-0.280485174	0.043039703
70.91078041	180.5699253	-0.250306282	0.059231006
48.62178628	138.5425644	0.367886149	0.080435682
151.4552263	257.1126137	-0.072620929	0.039430122
123.0016042	208.5134163	0.046497199	0.044472069
72.70136413	157.3192997	0.416536791	0.060361315
86.24431354	198.7477417	-0.334381375	0.053948582
95.75201559	186.5920124	0.2843724	0.051836614
98.15464292	194.7957211	0.301138952	0.051914738
87.30180183	186.1762714	-0.346553961	0.055075349
86.94638405	175.2731228	0.240667719	0.054857029
42.87561997	70.00573921	0.283821473	0.114485835
106.8777708	201.8516293	-0.310035342	0.050512722
112.6684528	211.4301205	0.393315475	0.047070514
129.8187762	201.352541	-0.700696167	0.049178682
51.87234528	127.2696724	0.43075481	0.073534328
41.42297623	116.679772	0.288918466	0.079988198
89.79074444	178.9796371	0.301316371	0.054969578
70.17557982	144.8784199	0.429094181	0.065275601
103.8507569	212.9482346	-0.262453677	0.050790756
42.37126185	133.6559529	0.242680556	0.073913367
96.74874814	193.9703732	0.203312469	0.049622537
46.49835262	104.4119143	0.493344495	0.08816634
71.82309794	157.7814772	0.283505268	0.059912179
104.8622492	231.8777161	-0.136852277	0.047429961
72.71285935	150.8087027	0.095466876	0.062200043
56.23872421	145.224103	0.502974604	0.068355056
80.58293337	169.2838631	0.385560894	0.058501347
102.9058977	207.5865479	0.059187086	0.048112925
70.30494649	185.1525187	0.033857734	0.065643491
88.13946716	163.6853809	-0.489593732	0.062146175
101.0540267	233.0982361	-0.0636697	0.048546711
91.8845289	195.9761391	0.550123604	0.05388479
67.87332641	158.8791046	-0.000856623	0.062980718
81.29910486	139.0440788	-0.622233997	0.067813116
95.42454419	181.0796127	0.007067917	0.051698426
66.40704806	139.1168194	0.296218658	0.06500463
134.3836991	232.5514603	-0.175306152	0.043788379
96.05783864	223.6640415	-0.181661331	0.048947319
138.0174106	222.2899017	-0.063746263	0.042986794
88.94402394	188.3341866	0.25349964	0.052949856
42.11351419	136.7990708	0.699704862	0.079887445
77.0671614	167.0451393	0.143524845	0.057191711
102.1485766	217.5864084	-0.245117391	0.04998985
56.21776387	150.1070023	-0.171534705	0.067052796
150.3578733	186.2359238	-0.439762353	0.049726933
73.1441561	165.9090109	0.315562148	0.060509344
96.69135332	249.3593445	-0.245576543	0.05074942
84.47285449	173.8320847	0.446963385	0.054562929
68.72359661	152.657505	-0.200885641	0.062502684
61.84985111	97.74832726	0.5852842	0.089446842
111.4513357	208.2518902	-0.128113955	0.046014902
98.49864929	222.4855995	0.042394975	0.046585028
112.6895525	175.1173553	-0.484815969	0.054585671
93.09286126	184.9770226	0.155060247	0.054135552
65.90491399	166.2828903	-0.189699461	0.060955137
109.1004042	236.6766472	0.301665019	0.046598106
107.6997655	192.6456394	0.316188585	0.050419075
45.63307273	106.7251186	0.507816296	0.083078937
44.63327253	113.2171402	0.672966029	0.081212079
94.71860243	201.0679131	0.049333589	0.053876702
85.93199032	178.5778275	0.474927646	0.056122603
136.9016252	220.2075787	-0.378451977	0.045736814
119.4318754	217.3617401	0.223664608	0.04628391
125.5507102	224.4404672	-0.382500805	0.047246074
87.44209743	187.1449356	0.36371477	0.05429437
97.76362143	205.8982906	0.3398595	0.050485197
122.5025262	240.7151413	0.030427151	0.043239626
65.9723477	142.8408165	0.485033519	0.064701943
53.19775787	135.7731361	0.404090105	0.070206145
65.02041925	108.2778969	0.784486709	0.078150664
123.4717793	246.3589745	-0.074134821	0.044123598
87.41313292	205.2604485	-0.018451247	0.051550507
129.8303875	237.3470917	0.00581008	0.043418436
94.30816118	222.7717605	0.209606519	0.049825561
68.45327278	168.353919	0.141887356	0.059811727
150.5463934	263.6816201	-0.196502055	0.040320295
96.30414927	214.0492401	-0.149412518	0.050370882
95.0849634	185.2632217	0.393120174	0.053924537
33.1015742	107.8496132	-0.119907413	0.091652794
67.2508272	168.6647911	0.438768865	0.060177595
57.18158055	130.1759558	0.633201625	0.073051888
127.9170012	232.4581881	0.178351759	0.043668757
154.0659947	251.8365154	-0.495136657	0.041935414
63.56063279	204.8112144	-0.073946758	0.065902237
63.57043062	151.93153	0.413092701	0.068662132
80.67419128	158.4637108	0.241280268	0.058969123
117.240193	234.6254621	0.284854083	0.044908474
51.32086502	142.2647018	0.317692943	0.06924759
63.79425275	154.047905	0.3875691	0.064098254
62.3492422	152.432518	0.259240289	0.065017492
183.302949	286.4722633	-0.463118051	0.042072209
93.15089772	180.6121826	-0.074088789	0.051951231
48.96989158	168.2705173	0.055913983	0.066693106
69.8497848	148.2780209	-0.424168385	0.065990908
126.0421739	194.7439384	0.286461075	0.048760152

log.sigma.5.0.mm.3D_firstorder_MeanAbsoluteDeviation	log.sigma.5.0.mm.3D_firstorder_Energy	log.sigma.5.0.mm.3D_firstorder_RobustMeanAbsoluteDeviation	log.sigma.5.0.mm.3D_firstorder_Median
131.6622774	19821766.41	91.80871193	-31.01939392
89.15507532	29796003.99	58.2684631	-28.05907631
91.43649137	8761082.96	68.31192558	-93.44990158
109.472097	49060271.75	77.92823243	15.35609245
137.5188705	10255788.58	101.4838749	-20.16877747
101.2904624	16596547.7	73.76073119	-88.4389267
78.37516898	3345240.56	56.73084056	7.901723862
147.3193654	32866273.9	107.8609513	-15.09683466
126.9176617	53754084.18	89.03243638	-32.94900131
94.70086083	47824671.48	66.55289994	-45.93871689
112.2729888	18729444.95	83.08693495	-41.31168747
109.2645062	29201610.83	78.4059867	-42.51844597
110.5555468	37575504.06	81.0325558	-0.436579436
107.4586538	19024382.46	78.31518731	5.306252241
104.5149995	16945926.34	72.59382649	34.99415207
60.12074926	53671563.35	32.95917114	3.650893211
116.3853177	18053529.66	82.16686109	-56.26034164
122.7088538	53568805.82	88.12847091	-33.7288208
126.9119081	15015167.2	86.18076436	38.09223366
78.65803429	30104333.96	53.07195949	-22.13406944
71.29741141	60458665.13	48.49930769	-62.18571472
103.0559045	28281948.44	73.55937793	-8.104179859
89.79137285	38857448.4	60.76288896	-45.74875259
115.1128582	9288512.442	86.49027274	33.73172379
76.54833616	17536088.03	55.21988162	-78.15855789
116.3788021	16028657.23	83.18760882	-62.39849091
70.11590534	31793586.32	44.58997542	-21.40383148
94.25829045	28289740.22	66.06150765	-81.28633118
122.9562984	37477590.06	94.19515338	-10.09100437
93.04095595	49422400.19	63.22298019	-69.61969757
85.19879849	43707251.84	59.79752637	-61.30716705
100.6001377	95854501.08	70.36654432	0.640729547
117.817669	42865848	84.95536821	-10.93474102
100.1069567	1872327.89	75.73295559	67.23913956
100.4526253	5944453.633	68.86567603	-24.25758457
121.2634647	44052043.57	92.74445884	-25.03794098
110.3659432	55710166.12	80.58752227	-54.08483124
91.91024599	42761188.59	66.49908695	2.989079833
96.46753222	13139973.61	62.75031737	22.55182457
111.1962787	22763906.24	76.70581663	-42.46776199
89.51222892	21157611.6	59.55049072	-35.49675369
135.7837035	27292454.49	99.14709167	-16.18147087
120.0628746	37780293.47	90.77550151	-37.71664619
133.4297333	28442727.44	94.78161941	-10.77713203
108.6877558	33583318.18	78.37035886	-12.15401316
78.47829285	23305198.15	56.41243384	-57.17102623
98.29309424	64124343.44	69.25700593	7.791370869
118.8406028	18151247.23	88.12094608	-84.70782471
86.79715173	1550813.138	61.92411455	15.28323603
127.567248	14161392.45	81.48537812	20.65718651
94.38899057	48613312.94	66.57837874	-14.55650902
125.802594	27899669.12	100.1286994	-100.2201843
105.2401311	68879297.85	72.18770742	-28.43455124
91.98975148	7979430.972	63.48802589	-1.628923893
75.38094109	72002900.15	45.36803513	-6.243593216
125.2545526	24036372.02	89.99216885	-50.71207428
122.0287862	53016349.65	90.95335981	-48.25421524
114.4453011	28763765.67	75.88729576	0.312221944
106.2877617	18800157.03	74.9189116	-76.89801025
93.60996926	12693364.09	67.88675154	-27.65908241
126.6213579	51060313.19	96.02401775	-43.20464325
112.7296175	111397737.1	78.35003355	-1.522044361
70.48225975	27273390.75	46.01226046	10.70008135
74.25625104	26727873.4	49.38819492	-33.82705688
112.2479864	4876906.05	81.07733406	-54.55591583
104.2785196	27806714.85	73.24674597	16.93308258
130.0636441	11069443.58	89.62401833	-19.46465111
124.6216056	76363327.79	88.96756425	-0.822728455
131.414993	17187713.22	92.28326297	-81.35189438
105.6558527	34640908.83	76.13548683	-29.03917122
115.2249573	60745475.77	84.41021297	-40.23618317
132.1277932	31903154.9	97.89217695	-44.7742157
88.45169698	67200411.8	60.63755822	-23.87006283
81.51659041	76700359.03	57.31292055	-39.06919098
80.30265938	112122012	49.22081214	4.319744349
133.663802	33430173.83	97.82504882	-48.31993866
112.5060663	27109677.42	85.28372437	-72.18600464
133.4847934	18996752.87	98.60968885	-74.15685654
116.9773778	49790154.72	88.90229674	-103.1020737
95.21676089	46040125.66	69.38512018	-57.33372116
149.5564089	29046366.69	108.7915856	-136.2050552
115.1609014	27222225.08	86.8257428	-28.12976456
107.4108582	53563832.18	73.61729765	14.28766632
66.4546748	1080759.584	46.84879267	19.32926941
95.58326917	46739405.95	69.16611627	-7.600265503
82.12625257	59985905.19	56.08122003	-23.79539776
132.7628722	22355583.64	98.50064532	-52.91953659
143.210231	53196953.32	104.0631863	-32.94246101
101.7829806	2643179.638	78.59983042	-33.0421524
90.83132689	1417499.355	60.46864993	-21.44186974
99.63034328	68079865.91	65.84976169	-14.62581825
128.4654684	24263988.9	94.73248338	-102.3812752
81.23807405	13651550.26	58.29311345	-62.34408569
90.27733631	56333061.69	63.7520366	-37.06498909
89.6907381	72439552.13	64.47516983	-6.68351984
163.0906096	11916042.48	122.2086213	-61.67111206
109.8204511	22461696.4	77.52083103	6.739589453
88.94305265	1460597.674	66.85861674	24.39482117
88.66626271	4691539.169	63.06683008	29.49085045
122.6264747	22470325.98	84.08130704	-60.00167465

log.sigma.5.0.mm.3D_firstorder_TotalEnergy	log.sigma.5.0.mm.3D_firstorder_Maximum	log.sigma.5.0.mm.3D_firstorder_RootMeanSquared	log.sigma.5.0.mm.3D_firstorder_90Percentile	log.sigma.5.0.mm.3D_firstorder_Minimum
535187693	398.1594238	167.5594115	183.4881897	-407.9721069
804492107.6	371.9824829	119.7735063	122.4983841	-329.9846191
236549239.9	174.0602417	142.0803544	62.61198349	-356.6549988
1324627337	420.4545898	136.4503535	191.2645721	-420.552887
276906291.7	296.5324097	169.0196918	198.1220459	-458.6394348
448106788	214.1236877	160.6583835	40.32708511	-400.5035706
90321495.13	246.5287323	99.63197204	169.4770538	-166.2814789
887389395.4	408.2093201	181.1095595	200.5433884	-446.8782349
1451360273	412.8890076	160.7972025	182.9411774	-475.4846802
1291266130	379.0649109	124.2468225	126.3159233	-344.0827332
505695013.7	258.7205811	150.4907127	95.73009644	-407.4573364
788443492.5	390.8519592	140.3716169	145.0425644	-332.4332275
1014538610	363.7368469	134.600921	190.1280579	-263.0894775
513658326.3	296.261261	130.4470861	146.6594894	-382.8499146
457540011.2	457.1081848	136.3871545	192.442688	-307.5517273
1449132211	413.6767883	87.8968828	112.0473602	-351.8797913
487445300.8	284.5861206	160.8249224	104.7923325	-449.6646118
1446357757	437.786499	151.5624823	196.0738373	-335.1509705
405409514.3	330.7303772	160.6214691	198.1971466	-474.4384766
812817017	340.6596985	103.6711598	111.7461853	-265.7367554
1632383959	332.4829712	107.5790471	64.04805489	-368.5857239
763612607.8	485.8036804	128.2302354	164.0655151	-322.1489258
1049151107	384.8393555	119.2823782	126.7756271	-376.3655396
250789835.9	312.4599915	138.9634365	191.969574	-324.3959656
473474376.8	220.779068	120.68495	53.37378044	-276.3450928
432773745.3	364.5209961	151.9738395	144.2221939	-384.0940857
858426830.5	369.3963928	95.7757321	110.0454941	-310.7468567
763822985.9	313.8582764	140.5048723	83.95223999	-329.7859192
1011894932	310.2831726	145.7180911	169.924765	-385.8077698
1334404805	326.986969	139.435752	71.55333481	-396.3745117
1180095800	341.7412415	117.2550311	90.34071808	-301.651886
2588071529	504.6586914	127.1390344	193.3702301	-327.5950928
1157377896	395.9094543	145.206822	168.3874023	-384.9477234
50552853.04	300.2579346	134.1758279	225.3003616	-172.1832428
160500248.1	243.0044556	133.8094945	95.2542717	-414.2804871
1189405176	339.8654785	147.6751244	143.6584335	-391.5691528
1504174485	505.5345459	139.0820178	147.9699448	-318.2186584
1154552092	308.5236816	112.9128463	134.9955673	-291.2206421
354779287.6	356.5192871	125.4451886	133.2675446	-404.6130371
614625468.6	370.1736145	147.3111602	132.6489136	-383.4190674
571255513.3	500.2965393	119.8076579	126.2181595	-382.3634644
736896271.3	362.5746765	169.229023	171.088089	-436.8304138
1020067924	304.6922302	151.6862054	121.5473999	-402.9432373
767953641	386.1714783	166.5803181	196.7483398	-424.3597717
906749591	364.7976074	133.7254316	165.0420975	-335.0354309
629240350.1	358.134613	102.2748395	104.5012527	-218.5806122
1731357273	419.3085938	122.6029839	169.5204849	-338.1012573
490083675.1	233.3429413	176.4489014	68.18047485	-439.965271
41871954.73	230.5424805	107.5789588	157.6324158	-233.4862061
382357596.2	476.0808411	168.8009105	207.3927826	-488.6722107
1312559449	397.1264648	119.1545776	140.1997711	-314.5205688
753291066.3	165.9445343	183.6736141	66.46630554	-428.4102783
1859741042	496.6290588	134.2628423	159.975235	-355.0317993
215444636.2	320.0254211	116.196279	148.4840698	-321.3477173
1944078304	407.4779053	105.0633648	137.7797089	-298.3385315
648982044.6	375.0443115	165.3636391	136.719812	-454.4836426
1431441441	351.0911255	155.4839607	139.5878693	-446.1661072
776621673.1	432.7358704	149.1501205	141.4377289	-534.843689
507604239.9	297.9707336	151.9737346	105.0514282	-377.8724365
342720830.5	233.3171844	120.032932	110.5937653	-321.7480164
1378628456	457.5341187	155.2302149	173.7787994	-349.6297913
3007738903	507.443634	142.6025102	181.081456	-368.0835571
736381550.1	414.3852844	93.94860322	130.0318222	-242.5188141
721652581.7	313.8302307	98.92848372	113.3873444	-310.9238892
131676463.3	270.282074	149.9140468	115.0955551	-372.3064575
750781300.8	459.2610474	133.8101673	218.4653992	-275.6763916
298874976.7	317.6845703	167.6157346	169.3383804	-459.6026611
2061809850	447.1474609	154.5507457	203.8417206	-345.161438
464068256.9	236.4366455	196.309637	85.7385025	-523.5387573
935304538.5	383.8950806	131.2467638	163.6076202	-302.2322083
1640127846	439.9500122	141.3579301	173.8891266	-346.3918457
861385182.3	344.0638123	168.1008252	158.1242065	-415.0268555
1814411118	444.4550781	114.0314678	134.9490082	-275.4103699
2070909694	375.1882019	106.8669352	106.2262535	-350.8617554
3027294324	455.2832947	110.515731	165.3986191	-284.879303
902614693.4	450.3404846	173.0761477	130.1026382	-493.2848816
731961290.3	348.3182373	155.7885691	91.5904007	-379.3166504
512912327.5	346.383728	176.4715779	143.0294128	-415.5417175
1344334177	321.7208252	168.0050931	97.58599548	-371.9139404
1243083393	315.9849548	127.1893659	106.1434326	-447.2855225
784251900.6	278.2954102	240.0659908	60.79467773	-559.2689209
735000077.1	377.0725098	142.4778724	135.3867188	-389.8057251
1446223469	476.4621887	138.5090381	204.2701462	-360.1842651
29180508.78	219.8858185	86.33373833	142.3398804	-195.0445557
1261963961	422.511322	117.4025958	170.0777588	-274.9756165
1619619440	365.8861084	104.66293	143.2728729	-301.8298035
603600758.2	407.6058044	169.5130772	167.0437759	-390.9030762
1436317740	287.6853027	185.1388329	144.169754	-635.0585938
71365850.23	186.8864441	120.1815484	142.2904785	-323.1766663
38272482.57	275.5072327	116.1894739	150.1801697	-245.5810852
1838156380	426.5775146	130.870204	160.1029846	-431.3642578
655127700.2	404.2454529	185.3867747	107.3344574	-395.4390259
368591856.9	231.849411	115.5754354	82.9941658	-266.4323425
1520992666	393.0995178	117.3599792	107.7804237	-316.6188049
1955867908	376.2067871	111.6990199	132.1532745	-260.964447
321733147	265.1311035	217.0229991	133.4469818	-553.501709
606465802.9	333.4066467	135.9109628	187.4663239	-374.5265808
39436137.21	244.2566376	108.9714542	170.0521729	-161.5878296
126671557.6	262.9585876	111.2597471	156.72612	-285.7202148
606698801.6	411.0315552	166.8660244	130.9079559	-388.8544312

log.sigma.5.0.mm.3D_firstorder_Entropy	log.sigma.5.0.mm.3D_firstorder_StandardDeviation	log.sigma.5.0.mm.3D_firstorder_Range	log.sigma.5.0.mm.3D_firstorder_Variance	log.sigma.5.0.mm.3D_firstorder_10Percentile
4.71675895	164.8616981	806.1315308	27179.37952	-247.793663
4.220169579	116.7880183	701.9671021	13639.44121	-174.1885315
4.105607161	109.1845425	530.7152405	11921.26431	-229.739711
4.467934756	135.3198175	841.0074768	18311.45302	-160.4125488
4.676502787	167.8778154	755.1718445	28182.96092	-256.8691589
4.260634897	123.0171945	614.6272583	15133.23014	-286.3509094
3.843286563	96.27781103	412.8102112	9269.416897	-92.7213623
4.81746739	178.6512852	855.0875549	31916.2817	-278.5601532
4.678418399	157.9651406	888.3736877	24952.98564	-239.4741791
4.253190837	118.289559	723.147644	13992.41976	-182.9445969
4.387852159	135.2908869	666.1779175	18303.62409	-265.2539551
4.433685718	134.7362313	723.2851868	18153.85202	-218.929364
4.408385768	134.5632789	626.8263245	18107.27603	-172.2435272
4.367880499	130.1691767	679.1111755	16944.01456	-194.2459518
4.414550751	131.5216955	764.6599121	17297.95639	-131.534256
3.669876176	87.51321217	765.5565796	7658.562304	-94.45500031
4.504423516	143.7132154	734.2507324	20653.48828	-275.596698
4.587347326	150.4911827	772.9374695	22647.59608	-199.1963531
4.592726471	160.2814998	805.1688538	25690.15916	-229.6776367
4.016911719	101.7052967	606.3964539	10343.96737	-151.8186798
3.902849467	91.17261752	701.0686951	8312.446186	-167.7809723
4.366068216	128.2125445	807.9526062	16438.45658	-171.3127243
4.208778245	114.7267426	761.204895	13162.22547	-165.9628906
4.426426537	137.3491673	636.855957	18864.79376	-169.8009644
3.922075724	94.14938889	497.1241608	8864.107429	-197.0265366
4.511312618	142.5368017	748.6150818	20316.73984	-236.0134399
3.880927092	94.39903727	680.1432495	8911.178237	-128.3826675
4.24987782	117.9373059	643.6441956	13909.20813	-228.9649597
4.513526322	145.0251246	696.0909424	21032.28676	-215.3899689
4.265094445	118.2503902	723.3614807	13983.15479	-227.3961853
4.092991839	106.3216114	643.3931274	11304.28506	-175.0618011
4.338068701	125.6886136	832.2537842	15797.62758	-135.2254761
4.549475317	144.6031359	780.8571777	20910.06692	-205.0505005
4.084217244	118.2201796	472.4411774	13976.01085	-93.01577606
4.275197514	126.4668554	657.2849426	15993.86551	-226.6750916
4.498057354	143.5864028	731.4346313	20617.05507	-236.2538132
4.39434834	134.5979076	823.7532043	18116.59674	-196.432254
4.169531883	112.6902127	599.7443237	12699.08404	-165.2334946
4.242268533	125.4402141	761.1323242	15735.24731	-177.8727631
4.483219965	140.1673133	753.5926819	19646.87573	-243.7946686
4.217850134	116.0776432	882.6600037	13474.01925	-175.8547256
4.687767933	165.2088279	799.4050903	27293.95682	-275.7666443
4.484378797	142.752588	707.6354675	20378.30137	-256.4671631
4.719335408	165.1357147	810.53125	27269.80427	-256.523822
4.414163436	133.2402304	699.8330383	17752.959	-182.4389984
3.868019459	96.06455497	576.7152252	9228.398722	-142.5207092
4.327391341	122.3765071	757.4098511	14976.00948	-152.1025085
4.475350511	142.7678911	673.3082123	20382.67074	-308.1835938
4.05809638	106.5680052	464.0286865	11356.73973	-120.490696
4.670735559	168.7446733	964.7530518	28474.76477	-235.3961853
4.253180154	118.3983434	711.6470337	14018.16771	-165.3730911
4.390428195	145.4918908	594.3548126	21167.8903	-328.2681152
4.424204553	133.2249575	851.6608582	17748.88931	-182.4133453
4.224649318	116.1870055	641.3731384	13499.42024	-158.9726563
3.954378459	104.9568775	705.8164368	11015.94614	-138.2157013
4.612420659	153.7658127	829.5279541	23643.92516	-274.0726135
4.562319396	146.5303707	797.2572327	21471.14952	-250.2404602
4.516925667	147.1943396	967.5795593	21666.17362	-234.9961792
4.404035794	132.4177824	675.8431702	17534.4691	-249.6004456
4.188524122	114.0875282	555.0652008	13015.9641	-202.4118195
4.552164739	151.1031941	807.1639099	22832.17526	-226.1319
4.52994779	142.5719384	875.5271912	20326.75761	-181.4474258
3.890983705	93.16257874	656.9040985	8679.266078	-109.8747772
3.898849296	95.93974792	624.7541199	9204.43523	-136.046402
4.38091854	137.7893131	642.5885315	18985.8948	-243.6005493
4.367287761	129.6435496	734.937439	16807.44994	-124.4810745
4.664508815	163.739724	777.2872314	26810.69722	-269.5338074
4.606941815	154.5225484	792.3088989	23877.21797	-213.3366486
4.622865727	162.2765717	759.9754028	26333.68571	-347.1871796
4.379001424	129.9471394	686.1272888	16886.25905	-180.874649
4.476390413	139.7458476	786.3418579	19528.90193	-194.4697418
4.665856985	159.585794	759.0906677	25467.62565	-272.88125
4.181418145	112.6928084	719.865448	12699.66906	-159.8112503
4.03411735	101.6363517	726.0499573	10329.94799	-161.7258072
4.060521298	109.4127773	740.1625977	11971.15584	-113.5671043
4.697053315	163.0462318	943.6253662	26584.07369	-279.6516113
4.397468521	133.5861182	727.6348877	17845.25097	-267.0236145
4.666093669	161.6430459	761.9254456	26128.4743	-293.0070587
4.441504963	138.9064286	693.6347656	19294.9959	-270.4937317
4.24416102	116.27517	763.2704773	13519.91516	-194.4121857
4.810545625	181.8604636	837.5643311	33073.22823	-426.4947723
4.439150522	137.3480516	766.8782349	18864.48728	-225.360611
4.450824751	136.6519099	836.6464539	18673.74447	-149.3887711
3.675787262	82.13914637	414.9303741	6746.839367	-78.67941284
4.229474783	117.2543956	697.4869385	13748.59328	-137.4664917
4.038278867	104.3685666	667.7159119	10892.7977	-125.7950172
4.654322041	160.3800191	798.5088806	25721.75054	-266.0393768
4.756286928	174.0003324	922.7438965	30276.11568	-327.9045197
4.084007434	118.8114667	510.0631104	14116.16462	-161.54086
4.087594227	115.3749442	521.0883179	13311.37775	-160.7147095
4.391268598	130.3780382	857.9417725	16998.43284	-176.0264832
4.599345233	155.8645443	799.6844788	24293.75618	-309.4385681
4.008325549	100.3544336	498.2817535	10071.01235	-187.2886917
4.177940995	112.447113	709.7183228	12644.35322	-171.6420731
4.145859223	111.0870444	637.1712341	12340.33143	-159.830307
4.758199165	194.6485182	818.6328125	37888.04564	-393.7610168
4.451146415	135.9107817	707.9332275	18471.74059	-174.4971771
3.982319974	105.7617336	405.8444672	11185.5443	-123.9071655
4.102362234	109.3364707	548.6788025	11954.46382	-128.1581085
4.598262192	155.6185102	799.8859863	24217.12072	-269.3591064

log.sigma.5.0.mm.3D_firstorder_Kurtosis	log.sigma.5.0.mm.3D_firstorder_Mean	log.sigma.5.0.mm.3D_glrlm_ShortRunLowGrayLevelEmphasis	log.sigma.5.0.mm.3D_glrlm_GrayLevelVariance
2.642985203	-29.94623294	0.010907196	43.77605243
3.551931992	-26.57539487	0.009782974	23.05466187
2.297620778	-90.91514057	0.014599635	19.24813611
2.713770742	17.52843236	0.004705649	29.86074673
2.376066301	-19.61365088	0.008772254	45.07222737
2.450924938	-103.3338572	0.01328713	24.24775948
2.355915699	25.63421455	0.038331032	14.93708077
2.365523614	-29.73870971	0.010904136	50.98110896
2.670106363	-30.04587666	0.004919447	40.31179032
2.883550323	-38.01122404	0.009683438	22.85271941
2.329828964	-65.90774243	0.011167727	29.56437983
2.667200015	-39.37434224	0.013703033	29.38416272
2.482445294	3.183065116	0.022673033	29.0352783
2.513091195	-8.510446708	0.007704801	27.32189388
3.111241215	36.10400987	0.00897249	28.23828217
5.196242678	8.203639553	0.004655435	14.33210418
2.682541917	-72.18841591	0.015546929	32.80835882
2.660283538	-17.98860608	0.014831255	36.76203822
3.035122787	10.44495986	0.009174065	41.90299259
3.29586227	-20.09333244	0.015904389	17.26329101
3.34527484	-57.10346031	0.007513997	14.0577325
2.956765925	-2.129953277	0.011190731	26.50483526
3.308542023	-32.65057864	0.006334227	21.62889995
2.261447485	21.11972828	0.017899965	30.51502271
2.592969688	-75.50330946	0.02125861	14.36026034
2.56351201	-52.71914308	0.012654612	32.75682529
3.880862236	-16.18062487	0.009454533	15.84834693
2.737437578	-76.3702233	0.018783969	22.56732517
2.121575557	-14.1942	0.008588853	33.64484026
3.146721765	-73.88622443	0.01266109	23.61584968
3.157942999	-49.44145291	0.011891978	18.38618344
2.926803433	19.14958226	0.006396254	26.13645484
2.553619002	-13.22702654	0.008466386	33.87105866
2.060983345	63.45976629	0.043215833	22.71009805
2.924346281	-43.7163049	0.011024107	26.00740052
2.191211542	-34.50923481	0.009746306	33.12214976
2.882107288	-35.03157065	0.01437421	29.49561773
2.501795509	-7.087087256	0.012847475	20.58986149
3.620810922	-1.117155498	0.007613579	26.22873175
2.739861899	-45.31779121	0.010963697	31.99153852
3.403335803	-29.66236083	0.006703398	22.47328016
2.350413169	-36.66749778	0.009858089	43.5720723
2.223855182	-51.28745991	0.008853591	32.46224525
2.529786183	-21.89059416	0.012979556	43.93749091
2.587810575	-11.38121469	0.01037936	28.86174251
2.972459463	-35.09621171	0.030383521	15.3102243
2.799881872	7.448636304	0.007494958	24.49747676
2.310750787	-103.6896526	0.015852026	32.80326916
2.421987279	14.71368889	0.030959045	18.55692908
3.435117669	4.356904213	0.008665083	46.59711604
2.885564312	-13.40319545	0.010358804	23.16208923
1.881814398	-112.1075654	0.013338892	33.57140092
3.118501553	-16.66197807	0.008093129	29.46303352
2.87041442	-1.467994355	0.015015289	22.14886306
3.947569232	-4.729109137	0.010192888	19.09724641
2.534209774	-60.83755397	0.007898006	38.12854737
2.349239822	-52.00108186	0.006688129	34.54898199
3.33772419	-24.07456797	0.004043227	35.45084657
2.63582112	-74.57577963	0.012852971	28.35763232
2.434365553	-37.30872102	0.019196318	20.72601452
2.29880161	-35.55621425	0.015884198	36.63464064
3.036563267	2.952679535	0.007713771	33.16989574
3.948014909	12.12740571	0.012838464	14.77724635
3.462280864	-24.13316515	0.008619647	15.91089548
2.492819116	-59.06205741	0.019122904	30.31786967
2.891298597	33.13172101	0.009255766	27.52407858
2.729707574	-35.83765126	0.01452317	43.39604443
2.580726338	-2.952122014	0.012025152	38.69820217
2.580344252	-110.4707557	0.013926923	42.62440138
2.648348353	-18.4242761	0.012730498	27.35989365
2.496387478	-21.28761299	0.011589176	31.65369333
2.302147558	-52.8229285	0.011685256	40.83308074
3.168695423	-17.42144072	0.012493989	21.1731095
2.989621166	-33.0241405	0.006157782	17.18024098
4.142277558	15.57468967	0.00841092	20.66159577
2.634709962	-58.06271783	0.008212898	43.01198914
2.30524916	-80.1550204	0.013319261	28.55845997
2.297973033	-70.80779283	0.021804632	41.79278669
2.211098575	-94.50246235	0.026436678	30.89367841
2.689018042	-51.54822638	0.005596268	22.0231334
2.365422761	-156.7113643	0.015119134	52.76881061
2.257369943	-37.88742332	0.010336807	30.22579504
3.015917222	22.60551205	0.005718431	30.56886415
2.646399086	26.5833596	0.025734205	10.89411678
2.751236071	5.897135992	0.013226191	22.37395012
3.242614829	-7.844184243	0.007736432	18.24416197
2.395258223	-54.89018859	0.016107275	41.33927919
2.55568542	-63.24770172	0.003324676	48.92171513
2.095286094	-18.09530202	0.017164914	23.27042316
2.815714762	-13.73375771	0.032449712	21.61414594
3.290323709	-11.3391997	0.004391528	28.64445191
2.530075599	-100.371809	0.025158225	39.07603058
2.555273228	-57.32947688	0.029211709	16.66692263
3.116154955	-33.60076624	0.012084045	20.71370889
2.78828056	-11.67645582	0.017229399	20.49486829
2.253136848	-95.97362401	0.014801411	60.89094204
2.601707705	-0.221842927	0.010246276	30.34313843
2.062278221	26.25325772	0.073982512	17.99173887
2.770108062	20.59775458	0.017471319	19.36871817
2.982697283	-60.22582003	0.017240677	39.85097492

log.sigma.5.0.mm.3D_glrlm_LowGrayLevelRunEmphasis	log.sigma.5.0.mm.3D_glrlm_GrayLevelNonUniformityNormalized	log.sigma.5.0.mm.3D_glrlm_RunVariance	log.sigma.5.0.mm.3D_glrlm_GrayLevelNonUniformity
0.011475429	0.042968196	0.069993189	28.55356653
0.010631727	0.062691205	0.209835375	114.4084615
0.015467036	0.06336151	0.097426317	25.36438818
0.004948466	0.051211559	0.109164329	124.1935691
0.009143947	0.043028944	0.070195625	14.49443126
0.013984603	0.058899417	0.092331258	35.19580795
0.043644485	0.077756143	0.146369501	23.22391682
0.011299121	0.039359486	0.065301749	37.34524195
0.005189779	0.044112609	0.096383288	85.14948065
0.010513941	0.059518731	0.13805844	166.734949
0.011636533	0.053685583	0.087805518	41.43357217
0.014673346	0.051637436	0.117290235	70.25866329
0.024378382	0.051751664	0.139038048	97.31698024
0.008062493	0.054653756	0.094246425	56.77138769
0.00943091	0.054334586	0.109096968	45.74905114
0.005350442	0.097336574	0.612086187	528.2514741
0.016937931	0.050364723	0.080634729	32.95666273
0.015812761	0.04654827	0.111428117	100.2937757
0.009568634	0.048728318	0.082657373	26.47877924
0.017345033	0.070828024	0.218153586	174.5842857
0.008309739	0.077330876	0.240664925	347.4857522
0.012028552	0.054879229	0.118494963	86.60151867
0.0067908	0.064197873	0.122895859	159.801316
0.019106012	0.050571149	0.097729062	22.43123555
0.023254378	0.073385096	0.135579118	79.70548944
0.013323517	0.049234181	0.075431809	32.10745896
0.010616727	0.078200164	0.408845418	221.835643
0.02044193	0.05952728	0.116690301	78.04219499
0.008924154	0.047468856	0.083401006	78.79162463
0.01379764	0.059741868	0.223192068	132.4113787
0.0129995	0.067441771	0.144408678	194.0249161
0.006943959	0.056521358	0.187644614	298.5929742
0.008903786	0.047780344	0.107173829	89.71940413
0.045588382	0.0650376	0.059852133	6.378288045
0.011444108	0.061202082	0.080012241	18.97024778
0.010185943	0.04833583	0.102683089	90.77710599
0.015478319	0.053290718	0.119634148	140.7588532
0.013936015	0.062001957	0.177086804	184.9112238
0.007978616	0.064727356	0.156693924	48.3741972
0.01160584	0.050870537	0.100881613	49.48294685
0.007176195	0.062632766	0.170199339	82.24375291
0.010318721	0.043700758	0.074050062	39.20974506
0.00925556	0.048916848	0.0864149	75.27230695
0.013903381	0.042779593	0.071115432	41.35253724
0.01105511	0.052267384	0.131325126	89.56789585
0.034789392	0.076886128	0.222668703	149.0270719
0.008038479	0.056473373	0.139605133	217.4729371
0.016699252	0.050051008	0.073007645	27.45219916
0.032249797	0.066380515	0.062425475	8.368215019
0.008946225	0.048730017	0.069561765	22.83777588
0.011195681	0.059102095	0.173359403	180.9795932
0.01399605	0.050827411	0.072649087	39.67311546
0.008586513	0.053214293	0.138506637	185.2657589
0.015819142	0.061742676	0.115219122	33.65094478
0.011335159	0.080368799	0.303981075	448.2501093
0.008197963	0.0457554	0.073242376	37.9394772
0.0070396	0.046527479	0.087782484	95.27121738
0.004204306	0.053473054	0.107864719	63.93653578
0.013803968	0.053503597	0.110503235	39.95591011
0.021505625	0.061035512	0.11296365	49.72006819
0.016908645	0.046290556	0.09144189	91.58690459
0.008233915	0.049737012	0.124651275	249.0327823
0.01397825	0.079450822	0.223939795	214.8447177
0.009561306	0.077270757	0.31205548	178.4068226
0.019905693	0.053516207	0.065980702	10.95803331
0.009781777	0.055397741	0.109807127	79.04427881
0.015367004	0.045413093	0.073313694	16.87174545
0.012796654	0.045548658	0.118855795	134.3233977
0.01441945	0.046792648	0.057993293	19.82736354
0.013543864	0.053803487	0.106237857	99.73907878
0.012423135	0.049889776	0.116867198	139.3406147
0.012228669	0.043221003	0.074902455	45.86009284
0.013560683	0.063107492	0.182938645	291.197751
0.006796648	0.068712611	0.231342722	402.2234508
0.009192269	0.072780412	0.26716604	572.8214326
0.008611071	0.043883576	0.070755761	46.21593662
0.014090665	0.051643164	0.082772296	53.94436624
0.022795266	0.043350407	0.062584768	25.06754329
0.028219007	0.049625261	0.096404613	81.20331396
0.005950999	0.059377309	0.111646178	155.1607412
0.015941671	0.040271288	0.056484501	19.31563391
0.010786191	0.050369095	0.082406567	63.2776003
0.006050697	0.052990277	0.121391812	135.5069968
0.026883601	0.090277346	0.088194149	12.04755322
0.014191787	0.059627644	0.139762661	184.1437909
0.008569107	0.070706213	0.214324001	337.2703332
0.017103074	0.043586807	0.071256505	31.96366257
0.003428696	0.041792256	0.065513191	61.44146039
0.01773243	0.063673633	0.104836143	10.72040332
0.033914838	0.067685908	0.063480382	6.667302581
0.004709442	0.055531658	0.19761606	196.2021791
0.026792995	0.044835449	0.070442007	29.85349322
0.032047494	0.068190534	0.182611333	61.37187467
0.012983176	0.063128492	0.17666874	231.4380387
0.018755166	0.06270603	0.228354116	319.6684923
0.015321068	0.041808087	0.047301826	10.16125602
0.010774883	0.051224126	0.113010307	57.23623895
0.078273105	0.066900887	0.055879854	7.81149243
0.018606787	0.065410359	0.115558695	22.59585513
0.018523816	0.047166567	0.129198931	34.68286311

log.sigma.5.0.mm.3D_glrlm_LongRunEmphasis	log.sigma.5.0.mm.3D_glrlm_ShortRunHighGrayLevelEmphasis	log.sigma.5.0.mm.3D_glrlm_RunLengthNonUniformity	log.sigma.5.0.mm.3D_glrlm_ShortRunEmphasis
1.199782559	301.1810106	592.1957399	0.955895291
1.510309755	190.7887741	1472.71875	0.916282618
1.278949754	154.7805795	345.089648	0.941329499
1.293734761	341.0367715	2089.328598	0.941973797
1.208545748	378.0909206	298.0731977	0.952699498
1.252340056	193.6029858	522.0276815	0.948043921
1.434405395	83.32973332	239.1233216	0.910318145
1.1812251	340.233764	857.156849	0.961090054
1.2583326	396.8553063	1686.492365	0.947983309
1.363644695	184.3315304	2342.816074	0.930836729
1.240072379	238.6281267	681.2414694	0.951182239
1.307502987	187.8571669	1169.64769	0.940942649
1.359706855	154.3419622	1586.343819	0.933667125
1.254791901	272.6486871	908.5547285	0.948318406
1.28332214	240.9207949	730.9933759	0.945179634
2.289075179	229.9227423	3716.575157	0.850668011
1.223134404	267.9882837	581.6558955	0.953678758
1.285436945	221.4073528	1876.298641	0.946292784
1.233006535	417.2606176	477.9412641	0.950340377
1.516901541	124.3008498	1997.616983	0.917604862
1.598382958	174.5473687	3482.851564	0.900843533
1.313912196	197.4308541	1354.005577	0.939630715
1.32997302	241.3257435	2105.311047	0.935052964
1.276561974	219.8409944	381.4801233	0.941562053
1.369649442	99.6597577	902.6633885	0.927694857
1.209600367	234.84276	580.5219551	0.95529659
1.915511931	164.2671613	2072.181939	0.876977974
1.31684431	148.875964	1117.439805	0.937531141
1.218638061	275.4520694	1490.524731	0.957450412
1.545096636	188.7902944	1761.745517	0.910390672
1.371430766	146.1581299	2417.487056	0.931848113
1.453934733	245.8390414	4354.840105	0.924335999
1.281127476	274.1672595	1630.739871	0.945439981
1.187274997	120.3558309	87.35443444	0.955009958
1.230946184	261.8029276	272.0274379	0.949534523
1.262804431	248.8084961	1654.189401	0.95048359
1.312144572	170.4385997	2266.872029	0.940682371
1.447922975	156.5227915	2434.78248	0.920991119
1.413760045	301.787584	614.0064955	0.923070858
1.266388364	238.4848712	850.111312	0.9478524
1.437775955	240.9180475	1072.976606	0.921345076
1.203125301	319.4035053	802.5609275	0.957253164
1.228434972	259.7111922	1370.300201	0.954875567
1.196820876	308.9679465	867.3509442	0.958341425
1.337229728	214.9567827	1459.252743	0.937335176
1.555397951	79.49448455	1532.859562	0.907366861
1.370186055	228.6252782	3205.107025	0.928914936
1.205723626	230.8097853	489.8979615	0.95605862
1.195872441	136.4038931	111.8783902	0.953145666
1.19594779	454.2891002	419.6823366	0.957560956
1.428231166	181.5033942	2537.513942	0.926558271
1.197789674	222.3699253	703.3808527	0.959419299
1.347183788	238.7594248	2963.948165	0.937364816
1.294393939	193.1089183	473.3787934	0.945311478
1.689353067	158.2548313	4331.764347	0.900738129
1.200659392	317.9950358	746.3428724	0.958735508
1.238340273	293.8083263	1807.952993	0.951517413
1.281088673	470.0732665	1040.119708	0.945928181
1.30282864	203.7137619	638.1396319	0.938919354
1.294731822	157.5501919	710.4981043	0.944953652
1.239587709	203.2498219	1751.68064	0.953077633
1.325252137	262.79852	4270.580776	0.938193487
1.548751246	126.2296351	2163.378133	0.911061596
1.723966067	159.160624	1764.365613	0.893903734
1.189806523	196.4521448	183.4133794	0.957829826
1.299701116	208.9847185	1223.220352	0.940111786
1.201904876	355.7838921	333.2812701	0.957666278
1.296791709	236.4029936	2568.53278	0.946242082
1.167543344	323.8880905	384.4188706	0.962569437
1.286069136	183.3521409	1601.491129	0.943174706
1.305784253	211.1086939	2407.512595	0.941778004
1.208194842	267.1547505	945.3296886	0.955672713
1.444239436	152.6989932	3813.051629	0.925393198
1.554401756	204.8144782	4666.555624	0.911352083
1.637565801	176.6612329	6096.085155	0.900232109
1.195969583	359.6980391	946.4266719	0.958614016
1.22888309	198.8188028	923.4951793	0.952145615
1.176327535	250.3255426	523.1998448	0.961583244
1.263339135	162.9688942	1431.103045	0.947121636
1.30025377	279.5768611	2241.326247	0.940550682
1.163510838	341.3173231	436.6358353	0.963420703
1.223828124	243.2924705	1115.516145	0.95392381
1.318509802	283.6923794	2190.622411	0.939470811
1.271298847	96.98987114	113.5189142	0.937415831
1.349349332	153.9177181	2634.487866	0.937819212
1.539896916	180.5041481	3767.27847	0.907621742
1.198885271	238.8797978	657.4979924	0.957751571
1.181039365	599.4538108	1328.655021	0.961201386
1.296154448	176.0780824	144.5506065	0.93958009
1.203165851	117.8043753	86.51948194	0.950117793
1.469665043	331.6962388	2906.484547	0.923788364
1.196386705	191.8845347	597.4995619	0.958179201
1.479962909	94.28362723	720.4864073	0.912724579
1.427947837	158.4603329	3047.232395	0.927622327
1.530349091	132.6290765	4125.127281	0.917412616
1.133558592	434.2047238	225.412354	0.970680895
1.300898858	255.0874321	960.9997407	0.941383111
1.166840837	87.89357906	105.5565644	0.961315979
1.322552592	183.5458214	291.3950949	0.933947592
1.337509996	229.2283776	623.7806314	0.935913568

log.sigma.5.0.mm.3D_glrlm_LongRunHighGrayLevelEmphasis	log.sigma.5.0.mm.3D_glrlm_RunPercentage	log.sigma.5.0.mm.3D_glrlm_LongRunLowGrayLevelEmphasis	log.sigma.5.0.mm.3D_glrlm_RunEntropy
366.3110955	0.941272608	0.014000802	5.005974337
297.6662155	0.878448946	0.015269057	4.81300522
200.548786	0.922367955	0.019451945	4.468936587
464.9808767	0.920216027	0.006155607	4.886914651
476.7862724	0.938290122	0.010678988	4.945454149
253.5484858	0.929178131	0.017070647	4.605842496
120.0875337	0.886327323	0.066651251	4.339099465
408.9704627	0.946952249	0.013095319	5.091200257
509.8497228	0.928404928	0.006465133	5.051673252
250.2533029	0.904231018	0.01477364	4.738805711
311.5498754	0.933215515	0.013766077	4.718120099
251.7832584	0.918094052	0.019478824	4.839728341
220.9375025	0.90668348	0.032902699	4.866949929
363.7847957	0.929062887	0.009688299	4.730121227
318.2759255	0.924174618	0.011686324	4.789784687
582.920661	0.779052386	0.011127502	4.720539457
334.9405652	0.937403571	0.023294182	4.805342768
279.4461756	0.923868584	0.020767798	4.978337522
541.7635016	0.933518372	0.011232937	4.914213776
191.6540938	0.879685826	0.02620884	4.602377728
291.5239086	0.860010013	0.012911236	4.584374606
265.9123485	0.917486583	0.016112169	4.779967691
326.9328911	0.911359603	0.009023443	4.670598951
304.480376	0.922277307	0.024223836	4.782853253
138.1819586	0.902185024	0.033399544	4.403050419
282.84511	0.939592108	0.016283729	4.814112102
331.4674663	0.817746016	0.018165295	4.732316993
195.0480797	0.914756562	0.028509676	4.676927609
351.2911253	0.94042275	0.010557829	4.822757603
318.8999492	0.871694002	0.019445854	4.882085597
197.9285446	0.904856389	0.018757522	4.571895239
363.3693721	0.890712155	0.010043247	4.885918088
367.4474916	0.923644481	0.011156338	4.942633427
145.9913339	0.943047337	0.055104106	4.301166919
336.8280856	0.933271548	0.013219745	4.589883205
331.5804325	0.929626809	0.012347381	4.862195752
222.2674267	0.917013889	0.020958398	4.82441879
244.456892	0.888972983	0.0198219	4.713770454
472.0678624	0.894426532	0.009766167	4.76127059
306.3647261	0.927183398	0.014950673	4.849592574
360.7423657	0.890303726	0.009708263	4.758730377
402.5105282	0.941480345	0.012374087	4.982057638
333.7958904	0.936990537	0.011194641	4.812968718
380.6123617	0.9430394	0.017775835	5.012415155
298.4559193	0.912345376	0.014372379	4.855632424
114.6297436	0.869665792	0.059141466	4.488062843
328.7955114	0.902629017	0.010758854	4.828309901
285.729222	0.940757356	0.020330208	4.754058592
165.4206618	0.94087256	0.03747702	4.30523997
568.5764864	0.942888098	0.010118127	4.961300605
264.721568	0.894185838	0.015684984	4.779419933
276.2573907	0.94381918	0.016960876	4.670954867
325.5694006	0.911018058	0.011187729	4.888756905
261.064645	0.922035663	0.019462356	4.596472219
276.668454	0.853300157	0.018356843	4.683259624
391.1895056	0.943379715	0.009588078	4.901758645
370.7009735	0.933705146	0.008738092	4.910596112
639.5994695	0.92450473	0.005009711	4.905758169
265.0619531	0.917312417	0.018213201	4.80796291
210.5777215	0.924648564	0.031931279	4.54290528
248.8289783	0.933676988	0.022066149	4.901341472
361.4997154	0.913935462	0.010771799	4.981276209
201.7091561	0.874159821	0.020828161	4.511934263
287.9202489	0.845027181	0.015542603	4.622967248
238.0444066	0.943637008	0.023172983	4.621324308
277.5637606	0.918519986	0.012405323	4.790849286
443.4646763	0.942795783	0.018822024	4.919163121
311.115097	0.922355093	0.016664415	5.006749715
389.2566868	0.949982753	0.016435859	4.86670814
238.1341114	0.921738133	0.017522203	4.785455505
274.655871	0.918775304	0.016613283	4.894496215
333.0435099	0.939837842	0.014662602	4.971493546
221.9219778	0.892563706	0.019675014	4.722016819
327.4255316	0.871432171	0.010698676	4.655723124
304.7370638	0.856711916	0.014022481	4.777111293
445.8117476	0.943617315	0.010312617	4.993316292
247.7787566	0.9351973	0.017595983	4.726284971
299.2591487	0.948045397	0.027765403	4.921037183
207.2545573	0.927699285	0.03660074	4.812055076
372.5402489	0.918238824	0.007688544	4.669636534
403.1682586	0.951617827	0.019305338	5.031335531
313.0743498	0.936843917	0.012820499	4.76559031
390.6429533	0.915775843	0.007749429	4.889119799
128.9028548	0.920424403	0.031663322	4.02675389
209.9250343	0.91075925	0.019450835	4.682731049
282.0925862	0.870877114	0.013279777	4.676130708
290.4333082	0.942554874	0.021582912	4.938936168
737.0362891	0.947214512	0.003886109	5.044171925
241.8403679	0.919293821	0.02036163	4.415449852
139.7237208	0.937728938	0.0398054	4.335113882
508.8589889	0.888437349	0.006559336	4.955767243
224.9204624	0.943124864	0.034552693	4.887062056
146.1363981	0.880550956	0.046121875	4.569046822
236.2585027	0.89616325	0.017910585	4.699651596
212.5231216	0.877858449	0.027449818	4.741257331
508.742524	0.960474308	0.017423727	4.923888546
349.045473	0.918838563	0.013306949	4.860690702
102.9747757	0.94934334	0.099491094	4.178696822
263.1214963	0.911508017	0.02341365	4.505926649
307.1187682	0.911066629	0.024621538	5.021679911

log.sigma.5.0.mm.3D_glrlm_HighGrayLevelRunEmphasis	log.sigma.5.0.mm.3D_glrlm_RunLengthNonUniformityNormalized	log.sigma.5.0.mm.3D_glszm_GrayLevelVariance
312.6982089	0.890654309	46.78104476
205.6002793	0.805879283	30.61374393
162.5495749	0.85952247	20.71935132
361.609874	0.860280583	36.90928782
395.9502013	0.883756638	44.87882903
203.467636	0.873075756	26.89175191
89.53215009	0.795373677	12.68888504
352.5693661	0.903033332	54.32612145
416.0413107	0.873094531	46.49131302
195.0037089	0.835472105	26.34974035
250.3704016	0.881164622	32.05074447
198.1368009	0.858167457	33.27620181
164.8222516	0.842308444	29.57899357
287.6270922	0.873949199	29.89008937
253.3005636	0.86712048	34.96799607
267.0683462	0.682214319	29.28455933
279.338693	0.887110189	33.70827972
230.9039177	0.869988862	38.9400968
438.8619952	0.878525079	47.33730128
133.7124126	0.808973709	23.73362202
191.225864	0.773866172	20.63103752
208.3786053	0.855683198	28.46627071
255.5042415	0.844680742	28.28581602
234.5139038	0.85872905	32.27092746
105.8940035	0.829174407	16.8731
243.5212545	0.889633747	36.6559453
184.1352858	0.728957337	27.60403576
156.4375661	0.850678333	24.20462183
287.6606184	0.896233865	33.05887472
206.505803	0.793601396	30.64061473
154.2806632	0.838562165	21.44607242
262.8081178	0.822793885	30.36646812
289.2618429	0.867757574	39.32561084
125.0578733	0.889161811	23.93383743
274.9149304	0.876700989	34.3577431
261.6527311	0.879771284	34.92226172
178.7420283	0.857291297	32.82012397
169.756862	0.815070817	23.45609566
327.932532	0.819884987	38.7935466
249.7434866	0.873254438	36.96753358
259.2924575	0.815888685	30.76057316
333.6574705	0.894107579	42.15475778
271.769812	0.889532946	32.42500334
321.2889607	0.896794837	47.05923097
227.7129879	0.85017838	35.11555556
84.50022686	0.788208224	17.79767944
244.7804135	0.831265987	32.46001994
240.1954751	0.892005351	32.60946746
141.9737573	0.885524061	22.75504132
474.0561762	0.894901117	61.69896194
193.6033782	0.82745846	28.79116834
231.0705555	0.900083023	29.02071868
252.166954	0.850265196	37.09445306
203.3362632	0.867858927	30.62357649
172.9803558	0.775403253	27.23278169
330.3960425	0.898621859	43.08769776
306.7385882	0.881637714	36.37991188
497.2613388	0.868990961	46.74959138
214.2317778	0.853219405	29.65578992
165.8615351	0.868557661	21.38093896
210.967792	0.885021323	34.53239068
278.6537834	0.851847207	36.77369858
136.95008	0.796671991	23.3484678
175.3559505	0.761751034	23.79146067
203.7168303	0.895334076	31.43576389
220.5661894	0.856012269	35.11871952
370.4177223	0.895781359	48.52511617
247.777144	0.870164745	40.0682015
335.2524141	0.906635895	45.76653714
192.5879152	0.862840315	31.12729918
221.4552169	0.860340785	33.16932702
278.6266447	0.890429886	40.43331739
162.9136324	0.82492628	26.4240547
221.685127	0.795757393	21.092771
194.3408036	0.773233142	33.91757607
374.2827796	0.897787788	48.75770561
207.2416242	0.882899768	29.84317262
258.910387	0.904220137	40.93671202
170.2778633	0.872502995	30.57423054
294.9936642	0.856722183	24.67586358
352.303171	0.909122776	51.64628929
254.8616045	0.88700858	30.03737528
300.7652654	0.855146057	37.68732236
102.8242114	0.849350366	16.06781678
162.4497451	0.851691872	25.32173217
195.7383827	0.787922177	25.22337705
247.9333132	0.895738908	39.13010308
623.0892327	0.903352954	51.63663578
186.7830167	0.856267028	27.916307
122.1002284	0.87742688	27.3936673
356.4570791	0.821631721	39.11612399
197.9682856	0.896599227	38.64547141
102.3201589	0.798325087	18.81014463
169.6411335	0.829643444	27.66620358
143.1744997	0.808227147	25.34743466
446.7433118	0.926566941	63.02323896
270.4601976	0.858899182	39.38184943
90.87676716	0.90334569	16.37723214
196.7112332	0.842212567	23.20865218
241.3544504	0.847067377	44.8705312

log.sigma.5.0.mm.3D_glszm_SmallAreaHighGrayLevelEmphasis	log.sigma.5.0.mm.3D_glszm_GrayLevelNonUniformityNormalized	log.sigma.5.0.mm.3D_glszm_SizeZoneNonUniformityNormalized
224.0373966	0.040603303	0.322457309
163.3235049	0.050816945	0.287102675
99.95887573	0.061198175	0.265423527
203.8289906	0.045451609	0.289307856
222.5320316	0.043005885	0.291484332
108.7997454	0.055064547	0.229457085
62.8040718	0.07998615	0.231648199
232.4557654	0.037480794	0.346285703
287.0136357	0.040692676	0.307464159
151.7841026	0.054894214	0.28586052
123.4858929	0.04952403	0.270872676
126.843469	0.049053318	0.263572043
89.30228755	0.050692999	0.223989298
158.2950567	0.051275412	0.262591311
166.9830329	0.048981693	0.276086254
174.8299682	0.0518724	0.264947322
183.6414647	0.050383842	0.301392242
178.3639596	0.044475608	0.301649262
259.7702713	0.044717676	0.307110972
98.40908006	0.057120797	0.241783696
131.6763809	0.064447577	0.243737581
135.1897428	0.053757197	0.255963613
175.8674936	0.054227223	0.263599336
128.4768655	0.051092746	0.30137741
88.60475339	0.067333333	0.308155556
194.4362656	0.045755054	0.341640904
138.8202192	0.054717915	0.276594836
108.82513	0.057015974	0.239854021
160.0069593	0.047700758	0.313486753
160.8949211	0.050213675	0.292457676
124.308144	0.061403667	0.273599697
175.9811666	0.050305076	0.267453158
173.8100384	0.044330593	0.275737348
95.33416124	0.061436673	0.364839319
164.1695095	0.048795989	0.291716687
148.4143498	0.046465316	0.283476702
148.7094834	0.049000517	0.304323347
90.15136472	0.058591476	0.238818932
170.0093822	0.049879808	0.246440459
182.1511505	0.04553674	0.344707472
167.6357864	0.052281909	0.26415541
194.2726356	0.042548271	0.303141982
148.4098757	0.048790129	0.29090087
209.6621059	0.040611431	0.326004541
140.7888349	0.046268861	0.272318244
78.98740436	0.065138469	0.282214718
159.1990136	0.048460649	0.274381071
142.6659639	0.050807217	0.298999196
110.6210547	0.06446281	0.296528926
287.3382489	0.039023453	0.34174356
137.8022487	0.052257512	0.257768684
140.156929	0.052769682	0.316151415
175.2165234	0.045367276	0.282528376
118.9530153	0.051810814	0.250162443
135.4621775	0.053881543	0.303589593
239.4494395	0.04310339	0.359716647
211.9967507	0.045787724	0.339355997
285.8923124	0.042709409	0.317033432
151.6808361	0.052220419	0.293371793
89.77797113	0.060070577	0.241798495
166.3840376	0.046989167	0.328675058
178.628925	0.046771907	0.305575361
91.09711822	0.05808518	0.231631968
127.2185955	0.05674536	0.239964651
144.1372379	0.052517361	0.335503472
146.6504487	0.047106097	0.278555753
239.5590493	0.041419644	0.28889601
171.353049	0.043533366	0.302056065
231.8394439	0.041755576	0.371532535
140.2371825	0.049741942	0.292232321
157.4319972	0.047321439	0.294798888
168.9100742	0.043608373	0.303993706
125.1694188	0.05382092	0.265446585
157.9273624	0.061185303	0.249855957
126.3967413	0.047867946	0.259745929
263.5932978	0.040764034	0.340277503
137.8471733	0.051013364	0.279108961
184.3953233	0.043915699	0.326563452
116.7106592	0.050031688	0.267459428
198.3790929	0.056979708	0.278116116
248.3172549	0.039432079	0.37007874
152.1181412	0.050533318	0.318520403
174.3686203	0.04610933	0.268664453
62.68538754	0.077929804	0.251635931
110.5727127	0.0552098	0.259109987
127.4150328	0.055241955	0.240154429
178.9979467	0.044053062	0.347203059
373.8783335	0.039722582	0.344655039
129.3348884	0.059485832	0.39388253
116.9433998	0.061436673	0.38563327
238.7251446	0.044624436	0.2898635
168.109368	0.04579187	0.364100186
75.8351422	0.066983471	0.298677686
110.6152865	0.052927961	0.26085551
103.9270018	0.055090715	0.287511229
286.1330755	0.037937995	0.405724341
152.925521	0.043646859	0.275886634
69.45902679	0.073979592	0.304846939
105.3984859	0.064381186	0.252117131
221.3866349	0.042330438	0.333235655

log.sigma.5.0.mm.3D_glszm_SizeZoneNonUniformity	log.sigma.5.0.mm.3D_glszm_GrayLevelNonUniformity	log.sigma.5.0.mm.3D_glszm_LargeAreaEmphasis	log.sigma.5.0.mm.3D_glszm_ZoneVariance
94.15753425	11.85616438	12.48630137	6.640504785
140.9674134	24.95112016	166.8533605	148.9592544
37.69014085	8.690140845	20.63380282	11.29260067
246.7796014	38.77022274	38.47948417	28.93696202
41.09929078	6.063829787	12.82978723	6.347165636
45.20304569	10.84771574	24.49238579	13.83895488
17.60526316	6.078947368	57.32894737	37.66672438
154.7897092	16.75391499	10.06263982	5.037821119
226.6010855	29.99050204	24.17232022	16.21486867
238.4076739	45.78177458	79.39568345	65.59722007
75.5734767	13.8172043	19.65949821	10.8732673
112.2816901	20.89671362	39.01877934	26.91622033
103.9310345	23.52155172	83.84913793	63.86977928
93.74509804	18.30532213	22.66666667	12.85941828
79.78892734	14.15570934	24.5916955	14.65502089
237.657748	46.52954292	3447.651059	3387.67061
79.2661597	13.25095057	20.53231939	13.48864376
238.0012674	35.09125475	36.22813688	27.49232877
69.40707965	10.10619469	13.62831858	6.996554155
126.6946565	29.93129771	248.6507634	220.07725
207.4206816	54.84488837	680.9494712	643.2663197
127.4698795	26.77108434	34.87951807	22.95067821
187.419128	38.55555556	69.50070323	54.74689281
49.72727273	8.43030303	21.4	12.90189164
92.44666667	20.2	78.33333333	62.22648889
99.07586207	13.26896552	13.4137931	7.686848989
133.8719008	26.48347107	1041.078512	989.7963425
95.70175439	22.74937343	41.72180451	28.82308528
218.8137536	33.29512894	13.75787966	7.363792169
182.4935897	31.33333333	160.0352564	143.4400785
223.2573529	50.10539216	133.6727941	118.4952768
392.3537832	73.79754601	130.5153374	114.1754463
173.4387917	27.88394277	32.19554849	21.74898456
16.7826087	2.826086957	8.391304348	3.279773157
34.71428571	5.806722689	16.3697479	8.5861168
198.1502146	32.47925608	21.04148784	12.6902974
267.8045455	43.12045455	40.90681818	30.19607438
157.1428571	38.55319149	216.768997	190.7868922
51.25961538	10.375	79.88942308	63.77383044
136.8488665	18.07808564	24.88916877	17.90732763
94.03932584	18.61235955	128.5168539	111.3735324
112.7688172	15.82795699	12.86827957	6.305317089
172.5042159	28.93254637	18.54300169	10.87579945
138.8779343	17.30046948	12.43896714	6.649634111
147.0518519	24.98518519	44.59259259	32.49765432
127.8432671	29.50772627	262.0573951	237.8675009
287.2769819	50.7382999	87.03151862	70.42998005
69.96581197	11.88888889	13.26068376	7.053345752
16.30909091	3.545454545	10.10909091	4.17322314
69.71568627	7.960784314	12.94607843	7.010644944
214.2057762	43.42599278	99.26113117	82.28395464
106.5430267	17.78338279	14.15727003	8.135124902
327.4503883	52.58067299	54.41156169	43.54262139
42.77777778	8.859649123	35	23.05509388
397.3987777	70.53093965	1155.103896	1130.271681
138.4909091	16.59480519	10.63636364	5.423740934
286.0771056	38.59905101	18.71293001	11.94552585
139.1776765	18.74943052	37.61047836	28.93550781
75.68992248	13.47286822	35.27131783	25.31704825
66.01098901	16.3992674	25.73992674	15.32571214
248.1496689	35.47682119	40.19470199	32.31757204
521.9227166	79.88641686	62.94028103	52.65376462
140.8322368	35.31578947	180.7664474	154.937316
106.7842697	25.25168539	538.411236	500.7475016
32.20833333	5.041666667	9.427083333	4.317599826
131.756871	22.28118393	37.43551797	26.65547157
45.64556962	6.544303797	10.93670886	4.718314373
331.6575592	47.7996357	34.60564663	26.12789689
78.39336493	8.81042654	8.265402844	3.797488825
183.2296651	31.18819777	41.89952153	31.61251396
304.8220503	48.9303675	29.51644101	20.87261354
134.0612245	19.23129252	14.47845805	7.924393643
318.0050083	64.47746244	144.0767947	125.4674374
319.815625	78.3171875	309.9171875	282.3874902
443.9057929	81.80631949	1108.616735	1079.763053
162.312369	19.44444444	11.08595388	5.612119774
111.9226933	20.4563591	17.72319202	9.963980324
82.62055336	11.11067194	12.1027668	6.289521786
154.3240901	28.86828423	31.44887348	22.10244827
224.7178218	46.03960396	52.25247525	39.84603838
94	10.01574803	6.716535433	2.779279559
164.6750484	26.12572534	14.94970986	8.221857241
225.1408115	38.63961814	44.33174224	33.23123587
10.31707317	3.195121951	29.92682927	17.41939322
231.3852184	49.30235162	63.86674132	49.44715963
237.0324215	54.52380952	414.0790274	383.2973293
115.6186186	14.66966967	12.26426426	6.805796788
239.5352518	27.60719424	10.09208633	5.105379639
28.75342466	4.342465753	16.64383562	10.35954213
17.73913043	2.826086957	9.5	4.289697543
305.516129	47.0341556	226.1508539	211.9277938
112.1428571	14.1038961	12.51298701	7.25877045
65.70909091	14.73636364	104.2727273	82.69247934
242.3347686	49.17007535	141.883746	122.5010052
363.1266825	69.57957245	349.3903405	328.2580115
56.39568345	5.273381295	5.043165468	1.730241706
101.802168	16.10569106	39.32791328	28.46829856
17.07142857	4.142857143	7.803571429	2.979272959
24.95959596	6.373737374	40.67676768	26.02101826
91.30656934	11.59854015	33.69708029	25.02255048

log.sigma.5.0.mm.3D_glszm_ZonePercentage	log.sigma.5.0.mm.3D_glszm_LargeAreaLowGrayLevelEmphasis	log.sigma.5.0.mm.3D_glszm_LargeAreaHighGrayLevelEmphasis	log.sigma.5.0.mm.3D_glszm_HighGrayLevelZoneEmphasis
0.413597734	0.141027359	3158.25	358.4486301
0.236398652	1.12526434	30409.24847	267.6232179
0.32718894	0.304830185	2769.373239	187.7112676
0.323719165	0.146003904	12912.70457	367.2708089
0.39275766	0.075726159	5258.780142	399.4397163
0.306376361	0.312653746	4439.700508	224.0304569
0.225519288	2.375997814	3411.578947	121.0131579
0.446107784	0.11292845	2973.552573	379.1319911
0.354497354	0.11355454	8051.537313	471.5671642
0.269205939	0.821765966	10903.88249	253.5131894
0.337363966	0.17796282	4890.856631	246.4265233
0.287449393	0.546007816	6205.396714	230.0657277
0.223722276	1.608317652	11990.29741	184.4849138
0.319320215	0.142540107	6593.932773	293.5658263
0.317233809	0.179302675	5526.747405	288.8685121
0.129120484	14.51000781	834119.0357	316.090301
0.376790831	0.293175076	5033.285171	309.1673004
0.338336192	0.658579614	4755.040558	292.5931559
0.388316151	0.086117185	5880.163717	450.8849558
0.187076044	3.43644379	27372.26336	182.3301527
0.162901991	4.948819508	101411.8895	249.2056404
0.289534884	0.461079472	5560.688755	245.1706827
0.260344196	0.458065067	13007.19128	312.5710267
0.343035343	0.267410616	5713.10303	227.0848485
0.249169435	1.660698046	6221.636667	140.75
0.417867435	0.165926523	2329.424138	291.0034483
0.139642239	7.444841967	156158.4773	246.7541322
0.278436846	0.823304477	4566.110276	202.1829574
0.395467422	0.094702586	4277.26361	284.4040115
0.245476003	1.052437856	33446.24038	261.8365385
0.256684492	1.980395193	11389.79412	208.1568627
0.247386172	0.842340627	24414.48943	314.9107021
0.309394983	0.20883558	8589.837838	308.5151033
0.442307692	0.330363667	984.0217391	136.4782609
0.358433735	0.113138983	4485.714286	285.8067227
0.346039604	0.178837252	5465.646638	269.9456366
0.305555556	0.793560881	4546.697727	232.1431818
0.196183661	2.446540133	35488.7234	183.9133739
0.249101796	0.287567071	27829.24519	325.5096154
0.378455672	0.290717191	5192.465995	282.4382872
0.241519674	0.666560721	28384.42135	309.1629213
0.390346275	0.133725873	4175.965054	337.4032258
0.361144945	0.158727872	4777.716695	273.2765599
0.415609756	0.179062444	3452.2723	343.0446009
0.287539936	0.380393817	8923.831481	254.1555556
0.203321364	8.494867754	11287.24724	132.1412804
0.245428973	0.570769284	19163.42216	282.2932187
0.401372213	0.20211354	2841.166667	255.3076923
0.410447761	0.230827905	1169.090909	170.7090909
0.410462777	0.068035411	6141.764706	475.9411765
0.242698598	1.056190687	14789.1769	239.5824308
0.407496977	0.269644737	2787.673591	238.4540059
0.303323737	0.372960527	10537.5522	301.8093184
0.289340102	0.367172825	5971.80117	222.7134503
0.200674536	8.09736087	180960.3934	227.2964095
0.437997725	0.074474625	3082.01039	362.5220779
0.384404925	0.145801992	4490.492289	338.059312
0.339520495	0.089073629	19968.71298	493.4123007
0.316953317	0.51083744	4727.988372	264.0542636
0.309875142	0.599626412	3588.424908	181.7326007
0.356300142	1.01773438	3400.919205	267.6304636
0.311792625	0.374668217	15002.99356	305.691452
0.196763754	2.189559926	19370.6875	171.9638158
0.162943977	4.102314994	80067.28989	242.8314607
0.442396313	0.147113948	1737.104167	226.2708333
0.304571797	0.308319348	6458.150106	258.8520085
0.401015228	0.131534202	4038.987342	395.4746835
0.343446982	0.423343134	6728.916211	288.9489982
0.47309417	0.119308306	2624.611374	351.7677725
0.311785182	0.659220365	4870.91547	234.8835726
0.340131579	0.370161672	4530.760155	265.9235977
0.39061116	0.196179924	3285.505669	297.8866213
0.231811146	1.710115351	17486.29883	218.4015025
0.190589637	2.178086813	55051.46797	291.2328125
0.186165577	7.279417659	175925.1492	235.6606202
0.427419355	0.07648993	4073.712788	405.3102725
0.358997314	0.27959375	2830.194514	236.2418953
0.414754098	0.304049663	2523.881423	294.916996
0.327097506	0.946873613	3085.571924	203.745234
0.283907238	0.299664348	11246.71782	347.4740099
0.503968254	0.110397351	2039.047244	382.7716535
0.385533184	0.140439106	3785.862669	258.7079304
0.300143266	0.216117914	11753.4821	323.3699284
0.282758621	0.494231437	2703.585366	117.5365854
0.263344146	1.015750344	6567.319149	200.8174692
0.180241052	3.384690339	57533.30598	252.0202634
0.428020566	0.235287975	2304.72973	283.1651652
0.447809278	0.026683152	6337.736691	620.9568345
0.398907104	0.165888062	3002.90411	193.7260274
0.438095238	0.254561472	882.1521739	155.673913
0.265157233	0.763993734	71217.1129	410.5009488
0.436260623	0.621666795	1401.876623	251.3506494
0.215264188	2.878412015	8184.159091	127.8227273
0.227139364	1.534668948	18292.42519	203.9354144
0.217533586	4.273084194	45478.75059	180.6191607
0.549407115	0.066265098	2304.215827	440.8920863
0.303453947	0.256444662	9796.867209	285.3360434
0.455284553	0.958513369	515.1607143	109.0178571
0.26121372	0.396368843	8208.787879	200.7171717
0.33952912	0.550080903	6403.328467	321.1094891

log.sigma.5.0.mm.3D_glszm_SmallAreaEmphasis	log.sigma.5.0.mm.3D_glszm_LowGrayLevelZoneEmphasis	log.sigma.5.0.mm.3D_glszm_ZoneEntropy	log.sigma.5.0.mm.3D_glszm_SmallAreaLowGrayLevelEmphasis
0.582541509	0.010761816	6.373668493	0.00661053
0.551874318	0.011256583	6.556943865	0.006567554
0.496103271	0.018371149	5.83595412	0.011763569
0.549204529	0.006145651	6.812073818	0.003925137
0.548282267	0.012759567	6.113535108	0.009420598
0.466221716	0.015865206	6.298912371	0.009398836
0.481974549	0.029490381	5.30734931	0.005515905
0.604064986	0.013469656	6.636617867	0.01001198
0.567251485	0.005675231	6.802207518	0.003510642
0.54911558	0.009236469	6.627381525	0.004314121
0.529055977	0.015815964	6.4368184	0.010993691
0.523763229	0.013886981	6.67698763	0.006203298
0.475475208	0.026310957	6.731454583	0.011535754
0.522963377	0.010004399	6.528790375	0.006066877
0.539502039	0.011255922	6.506171248	0.006785403
0.5222479	0.007114799	6.823038012	0.004050763
0.551831555	0.01222985	6.205407213	0.004036865
0.561597295	0.013362108	6.722951487	0.00711674
0.566171232	0.010711117	6.317479047	0.004404328
0.497839718	0.015445176	6.66722642	0.006718744
0.500471128	0.009831414	6.682471988	0.00591615
0.513118334	0.01114755	6.649315688	0.004666228
0.522481316	0.007300227	6.708734894	0.004087764
0.559600225	0.019140656	5.984089603	0.008988455
0.574346499	0.019853834	6.024249804	0.010151649
0.602136247	0.012182265	6.220706377	0.005088131
0.543457943	0.014157585	6.627922033	0.006946858
0.489705559	0.015162686	6.496362455	0.005290956
0.573984634	0.01085821	6.535291641	0.007428431
0.554359317	0.012613258	6.608545381	0.004354039
0.534263536	0.010625672	6.507296959	0.00513689
0.52953612	0.006523344	6.949948439	0.003414991
0.538188417	0.009323047	6.806747662	0.003972726
0.616950113	0.044355263	4.854977445	0.017302542
0.553558078	0.017947554	5.869144549	0.014191305
0.542907459	0.012278765	6.751430622	0.007823298
0.56878861	0.012352413	6.719088026	0.005510808
0.49784271	0.015359471	6.735144766	0.008182963
0.503190603	0.014289222	6.448979321	0.009613472
0.606313442	0.011482192	6.397156062	0.007587598
0.518002244	0.009632798	6.534882348	0.006343226
0.566345079	0.010235997	6.527931063	0.004513797
0.549080809	0.010601029	6.549372321	0.006680469
0.58684411	0.011021632	6.546030143	0.004739156
0.535992153	0.01205159	6.789064569	0.005654981
0.546941867	0.024771134	6.289373682	0.011475813
0.538651623	0.008732508	6.941610452	0.004336464
0.554165434	0.016292149	6.137667136	0.00774845
0.547438524	0.034916639	5.085638577	0.011036388
0.60417973	0.012283562	6.394972156	0.009051374
0.520763634	0.011618338	6.85519048	0.006346841
0.575968177	0.013499343	6.184352652	0.008049338
0.541126846	0.009390135	6.867491897	0.005032675
0.507342607	0.018405856	6.219904562	0.007595789
0.567939432	0.009858407	6.669368273	0.004958462
0.621015761	0.009227573	6.395754763	0.006120159
0.601974019	0.007399119	6.625362859	0.004775671
0.579567031	0.006219746	6.642702739	0.004745843
0.55541266	0.013175546	6.258759186	0.007997474
0.485322667	0.017055504	6.306048627	0.006260544
0.591185633	0.01277282	6.525791948	0.005328249
0.570024068	0.00827772	6.892754642	0.004486971
0.487026198	0.015909763	6.779943028	0.008674622
0.486871205	0.010108462	6.556918512	0.004005355
0.590648717	0.023117943	5.548235548	0.015422857
0.538898545	0.011764651	6.665573282	0.007613952
0.546369988	0.013725115	6.258637179	0.003511184
0.56374442	0.010949123	6.821350317	0.005316268
0.630145102	0.017570256	6.161825152	0.013989354
0.554958778	0.012826878	6.621957332	0.006117579
0.554227437	0.010674086	6.722272371	0.004682601
0.562720782	0.01267556	6.610048102	0.006541858
0.525106454	0.011257283	6.812830079	0.005019362
0.506249245	0.006103445	6.753363445	0.003183765
0.520734334	0.011586005	7.109343767	0.006790761
0.60166436	0.008513034	6.621805519	0.004226656
0.537879532	0.014287254	6.452808452	0.008536097
0.588911649	0.018045774	6.323450031	0.009355456
0.524430764	0.025793204	6.651222618	0.009636465
0.540129348	0.006069845	6.625422836	0.002650802
0.624591896	0.012908067	6.207798515	0.004749764
0.580787337	0.013009208	6.421282411	0.009170138
0.52960708	0.007051912	6.895697366	0.00422449
0.504567125	0.045555829	4.900115724	0.032365755
0.519935038	0.014114344	6.781328953	0.008035814
0.494253836	0.008582656	6.857102887	0.003903322
0.607095966	0.013053082	6.282018257	0.005388057
0.603708674	0.004350855	6.681009134	0.003252025
0.648814281	0.02784249	5.249698662	0.023194695
0.637000302	0.041149338	4.906941783	0.029526745
0.552732423	0.00539899	6.913979613	0.003392581
0.613119056	0.018055733	6.145579971	0.009348768
0.566087118	0.030917549	6.057120565	0.012338474
0.521311433	0.014020154	6.847292508	0.006937726
0.551928008	0.018959302	6.722128832	0.009601346
0.654752198	0.01437945	5.909212771	0.006145404
0.534813507	0.012186204	6.635578832	0.005386507
0.544701151	0.040464837	4.930222503	0.008617528
0.513261907	0.022500662	5.705276383	0.006037815
0.590130603	0.015364911	6.313696985	0.007435071

log.sigma.5.0.mm.3D_ngtdm_Coarseness	log.sigma.5.0.mm.3D_ngtdm_Complexity	log.sigma.5.0.mm.3D_ngtdm_Strength	log.sigma.5.0.mm.3D_ngtdm_Busyness	log.sigma.5.0.mm.3D_ngtdm_Contrast
0.018813704	1.143593261	6.049534445	0.12101205	0.000242449
0.006993386	0.223954272	1.82858884	0.355631408	3.73E-05
0.026493736	0.660879961	3.825402883	0.181036081	0.000301057
0.005104302	0.28545208	1.739651612	0.325478635	3.68E-05
0.03638593	1.81476675	10.7760007	0.059835839	0.00056621
0.016721068	0.734842898	3.568791494	0.171995825	0.00021061
0.038863285	0.349749426	3.332853491	0.271185373	0.00038253
0.012086056	1.063232581	4.503879749	0.159227806	0.000203114
0.0065193	0.508326415	2.792895654	0.225024477	5.65E-05
0.004369043	0.178758072	1.330534818	0.59496335	2.52E-05
0.011501962	0.797663965	2.961787791	0.210274311	0.000203904
0.009680967	0.38444642	2.778933618	0.299156713	7.31E-05
0.006749578	0.18264933	1.4785515	0.681423731	6.48E-05
0.011003558	0.463565177	2.790861098	0.203661573	0.000109833
0.014754638	0.745385026	4.74546063	0.140177852	0.000104953
0.002427844	0.054926098	1.019124382	0.580040405	3.17E-06
0.017416766	0.924655233	4.785428003	0.126471617	0.000209189
0.005608788	0.320737785	1.845623994	0.512469939	6.03E-05
0.020078889	1.52916246	7.676127147	0.071027286	0.000281174
0.004890971	0.116170667	1.020768474	0.761241369	2.55E-05
0.002697114	0.079162293	0.836180563	0.798681256	7.25E-06
0.00717111	0.454649139	2.964948799	0.30303077	4.89E-05
0.004744927	0.227289697	1.713172504	0.39555406	2.31E-05
0.026304687	0.893324216	5.372515362	0.117602097	0.000342681
0.009768574	0.197316795	1.321396076	0.588182099	7.86E-05
0.016917428	1.027469006	4.410137109	0.167605487	0.00023138
0.004684431	0.092634239	1.323957161	0.499318881	1.10E-05
0.008694799	0.288662431	1.90827602	0.439280936	7.67E-05
0.005895115	0.393709661	1.593663842	0.42104792	0.000101601
0.00579058	0.200232947	1.649487715	0.394054055	2.94E-05
0.003798782	0.138020073	0.980014309	0.864611479	2.39E-05
0.002313305	0.133102014	1.048550376	0.744802781	1.09E-05
0.006762987	0.339640915	2.126351302	0.315568551	5.86E-05
0.079856383	2.843540801	10.71793087	0.075375504	0.002322117
0.030314772	1.567989357	7.877255195	0.073986358	0.000406697
0.005688595	0.338848789	1.639931803	0.422861756	7.37E-05
0.004188617	0.296753627	1.906962576	0.641471312	3.39E-05
0.004100597	0.095217793	0.830391521	0.837030712	2.66E-05
0.014954043	0.728349749	5.711377555	0.09808175	9.10E-05
0.011755234	0.682820385	3.417430421	0.194329412	0.0001266
0.010211308	0.385759865	3.465507659	0.188876212	4.41E-05
0.012056318	0.957930078	4.173830255	0.161003614	0.000193904
0.006549219	0.445400645	1.891429774	0.342072063	9.44E-05
0.012371149	0.829925259	4.031178933	0.170719083	0.000173903
0.007030057	0.287220924	1.820058294	0.406346436	6.28E-05
0.005215894	0.135192767	1.204548551	1.175844123	3.40E-05
0.003381292	0.133146799	1.039670215	0.629627667	1.78E-05
0.018171693	1.041815144	4.542656044	0.146806808	0.00030974
0.072104347	1.978292238	8.360654864	0.077563346	0.00133047
0.023391393	2.767917572	13.16787554	0.045085029	0.00025845
0.003965555	0.144086411	1.120208376	0.678741515	2.36E-05
0.0101934	0.700509077	2.225377812	0.315345877	0.000322745
0.003343218	0.224496596	1.310712457	0.589064244	2.41E-05
0.023202874	0.641204544	4.57936491	0.126620516	0.000169838
0.002244115	0.066213608	0.674900089	1.076458717	8.37E-06
0.014278977	1.00819297	4.767084848	0.132680619	0.000170783
0.005294088	0.41404541	1.777312807	0.375240629	6.32E-05
0.009082381	0.811981496	4.587229886	0.118678229	7.71E-05
0.017003361	0.563628386	4.098582065	0.180392375	0.000144379
0.01257421	0.366463017	2.049227174	0.326103745	0.000154978
0.005352475	0.38023307	1.772144456	0.593615398	7.84E-05
0.002498705	0.167558115	1.149603674	0.695667174	1.61E-05
0.004523553	0.120394201	1.238128312	0.640469478	1.53E-05
0.005136023	0.122876526	1.128007928	0.55610053	2.02E-05
0.049691135	2.100071074	9.592641529	0.074684911	0.000936645
0.009297846	0.38017298	2.883697233	0.271805814	5.78E-05
0.029764479	2.150733966	9.437321553	0.062199301	0.000496803
0.003870842	0.250831128	1.244035916	0.664092408	4.97E-05
0.023212406	1.906542377	7.172461311	0.085911181	0.000491142
0.006615066	0.272324989	1.744493549	0.479984551	5.59E-05
0.004058659	0.235204903	1.271893722	0.695353934	4.19E-05
0.009904077	0.710827042	2.917660701	0.266063847	0.000177834
0.002529446	0.11239972	0.834742379	1.064270211	1.38E-05
0.002070718	0.08030094	0.711600138	0.948688681	6.89E-06
0.001637023	0.052682816	0.609941961	1.314240665	5.37E-06
0.01042283	1.162424158	4.945481271	0.132852056	0.000124311
0.010420562	0.56721971	2.912853549	0.260364127	0.000117584
0.018083086	1.379482988	5.247178216	0.146345314	0.00035194
0.006253395	0.353143365	1.606805927	0.60642175	9.52E-05
0.004535466	0.214756603	1.461717686	0.419313273	2.51E-05
0.020036858	2.492544224	7.674144643	0.090592837	0.000496185
0.008445116	0.524242035	2.545185464	0.269789486	0.000100071
0.005353971	0.251561929	1.916663375	0.357572341	3.23E-05
0.061043966	1.12090472	5.433480992	0.108960419	0.000818726
0.003663496	0.151309249	0.978571946	0.921193199	2.77E-05
0.002590306	0.072347976	0.704178122	1.005918301	1.04E-05
0.014634916	1.129333141	4.843489856	0.18419762	0.000242182
0.006841006	0.837539786	3.201840719	0.172657312	0.000123512
0.059054218	1.300330104	8.293987282	0.08463096	0.001022931
0.086359948	3.224171445	13.93755842	0.064432507	0.001677881
0.003718162	0.199160969	1.428531805	0.387485872	1.86E-05
0.01593267	1.336160012	5.620262699	0.188897037	0.000256467
0.014525893	0.194889068	1.947088587	0.446632477	8.96E-05
0.003179763	0.125464177	0.949503548	0.860242985	1.78E-05
0.002239497	0.074350131	0.583208703	1.484702717	1.39E-05
0.039204637	4.185526595	16.18312528	0.040352407	0.0011408
0.011797759	0.420798582	2.898011113	0.209186602	9.93E-05
0.063519788	1.729909051	5.53259482	0.168911763	0.002297044
0.033878886	0.720749	5.821534904	0.098254888	0.000270767
0.017978551	0.872085713	6.463544849	0.131529534	0.000158987

log.sigma.4.5.mm.3D_gldm_GrayLevelVariance	log.sigma.4.5.mm.3D_gldm_HighGrayLevelEmphasis	log.sigma.4.5.mm.3D_gldm_GrayLevelNonUniformityNormalized	log.sigma.4.5.mm.3D_gldm_DependenceEntropy
41.54122295	308.2110482	0.044848285	6.645929187
22.09175626	179.2397689	0.065074089	7.107019174
20.59547347	150.6290323	0.062010236	6.237738944
30.20998254	401.4777989	0.050494835	6.942105251
45.93834623	399.9860724	0.043877686	6.515485035
22.54350113	161.9891135	0.060193639	6.204374849
15.21316556	97.66172107	0.077794116	6.096784571
49.78340823	350.0548902	0.040135298	6.742843807
40.89238707	371.4708995	0.04386729	6.923730427
22.25410509	220.1930278	0.061500349	6.919377654
27.38564968	225.3542926	0.056095004	6.342110455
30.04265855	201.6491228	0.051559788	6.841300411
27.79317704	164.0318226	0.053991235	6.905518142
27.84529059	260.9400716	0.05362086	6.598267268
27.13349584	217.428101	0.055845074	6.66777176
12.44545569	231.2912048	0.116667273	7.167302982
33.99602631	257.1088825	0.049638345	6.45358537
35.69615724	200.4309605	0.047589434	6.96625437
44.19088107	439.257732	0.046574792	6.550641387
16.53360934	133.1674402	0.073754324	6.913657148
13.61173017	194.3556662	0.079599707	6.9838423
27.02976981	182.7424419	0.054660627	6.704701826
21.77355856	225.7162212	0.064555872	6.747086402
31.60039938	294.3638254	0.050384464	6.388787091
14.40675807	91.02740864	0.074153431	6.480397964
33.06714614	242.2737752	0.049236353	6.557821259
14.54023639	208.9278707	0.090529416	7.122438972
22.44688722	137.7732031	0.060200469	6.604050536
35.4175753	296.0067989	0.046879118	6.590526596
22.65049606	214.5743509	0.062662146	7.187907806
18.33700417	153.2884555	0.068365149	6.676543381
24.97301653	225.0595278	0.059176565	7.128456798
33.2627669	261.6546975	0.048304549	6.872533182
28.07683062	178.375	0.05510355	5.364921901
29.73997496	316.5933735	0.055341849	6.159771324
32.93237526	266.9920792	0.048880992	6.736523678
29.42156527	178.7788194	0.053686343	6.875953494
19.99147619	171.3753727	0.063330073	6.974578726
25.30305999	301.3005988	0.06662842	6.728236415
31.24223806	223.6024786	0.0523182	6.638209561
22.20582913	259.541384	0.063171867	6.916488008
43.35009012	303.293809	0.043587985	6.589114284
32.0119785	246.2545676	0.049787773	6.619029895
43.42374872	321.9717073	0.043120048	6.742092351
27.69693616	223.9504792	0.053867947	6.89816669
14.50693878	99.05116697	0.080028864	6.683675173
24.66231038	242.9688233	0.056121309	6.962415922
31.88441521	200.2161235	0.05059593	6.364213387
24.21140566	212.761194	0.061706393	5.6829599
47.24121793	472.4969819	0.051305823	6.392925055
22.21857497	190.6571262	0.060983424	6.909851667
33.59642302	216.5163241	0.052106286	6.368734832
28.73696944	249.2062287	0.053979232	7.019791159
21.53881832	203.1725888	0.06318981	6.467796593
17.8843739	170.020696	0.089683554	7.063550012
38.73442906	307.112628	0.045882887	6.621409234
37.30281006	347.1623347	0.044563091	6.766227152
34.42546187	463.4887858	0.054567727	6.794379746
27.67190264	195.0786241	0.054585298	6.651807579
20.22296663	168.0896708	0.06175394	6.3974701
35.7260753	205.6106654	0.047893383	6.75161841
32.62098145	277.8481198	0.050123225	7.162809859
14.04979724	134.6300971	0.083649312	6.838381513
15.01469693	200.447089	0.080861868	7.016964946
31.76504067	234.4009217	0.052050373	5.888321139
27.69544935	219.2852543	0.055889583	6.859654643
41.94050993	363.1142132	0.049112319	6.309034505
38.55378493	244.1626525	0.045580443	6.993960928
39.32307105	276.8699552	0.050352913	6.26647487
27.75978167	168.7916459	0.053738006	6.718055865
31.98394207	219.6108553	0.050407072	6.982926614
39.63898698	251.0593446	0.043851563	6.590327304
20.64683342	160.5982972	0.064780271	6.968078092
16.9369963	220.3263848	0.069816207	7.074261076
19.1045324	191.6862745	0.078347193	7.185601962
43.0953739	378.6810036	0.043521409	6.70378509
27.95744613	213.3267681	0.052560374	6.572197331
41.13457673	230.4	0.043681806	6.415831905
31.86250681	229.1184807	0.049031139	6.729762965
22.49307556	267.6366831	0.059047749	6.678222669
54.70973482	371.1825397	0.038840073	6.455123758
33.54899929	295.0909769	0.04790297	6.599785801
29.73509864	333.732808	0.054036143	6.928962366
15.52028537	167.7448276	0.082330559	5.638996812
22.61612204	158.8947213	0.059482397	6.74683693
17.39763723	193.3668736	0.073412516	7.082442028
41.3744771	222.5848329	0.043701799	6.571566858
49.01228171	587.0908505	0.042294944	6.688941647
21.33207919	215.0437158	0.066977216	5.84582411
23.62394558	149.7047619	0.074829932	5.339218885
27.97914551	319.8344654	0.058638756	7.194018
40.38498824	179.7988669	0.044551357	6.427785737
16.71486878	108.5332681	0.069385457	6.72474357
20.45623956	197.7366748	0.063802942	6.960042553
19.65747752	141.7664485	0.065346122	7.057901585
66.70199503	468.284585	0.040197472	6.120271281
29.12258632	309.0518092	0.053052231	6.754924696
22.57875603	124.2113821	0.061273052	5.451269156
20.48292618	195.5039578	0.065127645	6.232648713
37.29789067	271.1945477	0.048628098	6.95011473

log.sigma.4.5.mm.3D_gldm_DependenceNonUniformity	log.sigma.4.5.mm.3D_gldm_GrayLevelNonUniformity	log.sigma.4.5.mm.3D_gldm_SmallDependenceEmphasis
167.0538244	31.66288952	0.361265658
282.2007703	135.158883	0.217315795
90.50230415	26.9124424	0.309101498
533.7294118	133.0538899	0.274158194
85.70194986	15.75208914	0.333803729
153.4385692	38.70451011	0.235004523
58.76854599	26.21661721	0.188829074
256.9361277	40.21556886	0.358549126
454.4699375	91.2000962	0.306938179
506.2601679	190.5280826	0.248696635
198.8428053	46.39056832	0.277698646
289.1592443	76.41160594	0.259533699
374.1407907	111.9778206	0.205884785
241.940966	59.94812165	0.281502345
180.2074643	50.87486279	0.276043238
535.0656398	810.4875486	0.129262625
172.8108883	34.64756447	0.319675847
459.983705	110.9785592	0.275060219
146.2199313	27.10652921	0.329006902
399.5083899	206.5858622	0.186339251
661.3541348	415.8288668	0.169537079
340.3488372	94.01627907	0.254059726
490.8103259	176.3020871	0.24086628
107.5239085	24.23492723	0.271480714
195.7857143	89.2807309	0.242494211
157.0778098	34.17002882	0.329921627
356.5193306	313.7749567	0.152306069
259.2623866	86.26727146	0.266025451
440.5399433	82.74164306	0.36288085
334.8583792	159.2871755	0.212076529
517.968229	217.3328091	0.238460445
946.7824621	350.917032	0.226856393
428.7343827	98.20314806	0.272902199
38.34615385	5.730769231	0.411258013
82.70481928	18.37349398	0.304759036
444.6544554	98.73960396	0.287317328
525.2277778	154.6166667	0.259842115
517.470483	212.4090638	0.191870285
140.4299401	55.63473054	0.205287928
226.7006673	54.88179218	0.319713771
226.9389417	93.11533243	0.207718395
236.3955929	41.53934942	0.353555576
393.8708892	81.75152253	0.309798593
242.2370732	44.19804878	0.32538385
348.627263	101.1640043	0.25937944
296.8554758	178.3043088	0.198825585
755.846226	239.4135021	0.233180183
140.2830189	29.4974271	0.330698317
30.7761194	8.268656716	0.332989221
123.028169	25.49899396	0.346022507
594.0566589	208.807243	0.219222914
198.7750907	43.09189843	0.330541827
695.7678618	206.2546454	0.263706585
116.0829103	37.34517766	0.23061455
723.2971026	585.0058255	0.1800574
216.8634812	40.33105802	0.354192522
511.744186	97.72685819	0.348739469
269.0030936	70.55607115	0.299029573
152.7248157	44.43243243	0.264666387
192.3257662	54.40522134	0.241641881
448.0599339	101.4860783	0.302603045
1017.16831	274.5750274	0.263776396
418.6686084	258.4763754	0.189121945
313.6693519	220.8337605	0.158279781
61.11059908	11.29493088	0.340865335
290.8763683	86.79652286	0.282894642
96.1928934	19.35025381	0.368666373
666.7829215	145.7206756	0.290931667
115.4843049	22.4573991	0.396341555
407.6752859	108.0671308	0.285016105
578.4552632	153.2375	0.288888801
287.5385297	49.50841453	0.317077029
794.5789474	334.7844427	0.211414743
889.5807028	468.8856462	0.186546402
1119.881917	719.2272331	0.177770715
264.8799283	48.56989247	0.356440631
248.5022381	58.70993733	0.285679569
173.704918	26.64590164	0.355231302
381.292517	86.49092971	0.303519504
550.7104708	168.0498946	0.248648768
145.0198413	19.57539683	0.429861662
320.5555556	64.23788218	0.349928537
520.6196275	150.8689112	0.266833338
33.8137931	11.93793103	0.303559387
630.8613978	201.7048068	0.246904099
737.1267348	402.0069394	0.17720472
187.0899743	34	0.344546984
403.1713918	65.64175258	0.379498844
34.46448087	12.2568306	0.328904548
23.8952381	7.857142857	0.354685185
606.8344654	233.0890566	0.244078081
175.5269122	31.45325779	0.391590159
153.0469667	70.91193738	0.199639247
668.0415648	260.9540342	0.214793862
827.1215983	379.3995866	0.203288813
79.90909091	10.16996047	0.458875714
235.7302632	64.51151316	0.255966856
34.67479675	7.536585366	0.379150858
73.32189974	24.68337731	0.20717674
136.6158612	39.24287485	0.325659156

log.sigma.4.5.mm.3D_gldm_DependenceNonUniformityNormalized	log.sigma.4.5.mm.3D_gldm_DependenceVariance	log.sigma.4.5.mm.3D_gldm_LargeDependenceEmphasis
0.236620148	1.827741174	8.141643059
0.135869413	8.677093666	26.07366394
0.208530655	2.559238888	10.61751152
0.202553856	3.079655924	12.20303605
0.238724094	1.628028957	8.181058496
0.23862919	1.38728114	9.818040435
0.174387377	2.80506124	15.71810089
0.256423281	1.272564651	7.047904192
0.218600259	2.088933033	9.677248677
0.163415161	4.657148597	16.80568108
0.240438701	1.43652923	8.753325272
0.1951142	2.78606253	12.31174089
0.180395753	2.882224613	14.54869817
0.216405157	1.939999552	10.03041145
0.197812804	2.511520976	11.52469813
0.077021108	22.34391164	66.31682741
0.247580069	1.320136945	7.76504298
0.197248587	3.255897514	12.59348199
0.251236995	1.677708105	8.144329897
0.142630628	8.736379023	26.27097465
0.126599184	9.443386514	30.15773354
0.197877231	2.342855598	11.70930233
0.179718171	3.220486914	13.98205785
0.223542429	1.92929664	10.00623701
0.16261272	3.649669982	15.46179402
0.226336902	2.007208763	9.051873199
0.102861896	21.74949464	53.54529717
0.18092281	2.856972694	12.77529658
0.249597702	1.546278038	7.506515581
0.131730283	11.10499795	30.2895358
0.162934328	3.816957006	15.80088078
0.159659774	7.231118416	21.39561551
0.210887547	2.334033939	10.8622725
0.368713018	0.576923077	4.576923077
0.249110901	1.268870663	7.885542169
0.220125968	1.950494069	9.796039604
0.182370756	3.170570988	13.33958333
0.154284581	5.21121462	20.06380441
0.168179569	4.187543476	17.13892216
0.216111218	2.743892454	10.56148713
0.15396129	5.136973944	19.4165536
0.248054137	1.412463431	7.426023085
0.239872649	1.658578632	8.567600487
0.236328852	1.487356573	8.116097561
0.18563752	3.289682337	13.44941427
0.133238544	6.397779687	23.45601436
0.177179143	3.215656719	14.33614627
0.240622674	1.63179744	8.20754717
0.229672533	1.526843395	8.194029851
0.247541588	1.39614346	7.511066398
0.173497856	7.435007629	20.80957944
0.240356821	1.560153174	8.088270859
0.182090516	5.605175218	16.79377126
0.196417784	2.40791798	12.45177665
0.110884118	11.39466573	35.14441208
0.246716133	1.49794277	7.575654152
0.233353482	1.758633001	8.263109895
0.208045703	2.542693748	10.8066512
0.187622624	2.965849477	12.7027027
0.218303934	1.953973467	10.80476731
0.211448765	2.378194457	10.36101935
0.185682422	4.399474095	14.94121942
0.135491459	7.927787099	25.91197411
0.114855127	11.70420901	36.1724643
0.281615664	1.127949203	6.760368664
0.187299658	3.601572098	13.34256278
0.244144399	1.664381973	7.700507614
0.208565193	2.975207931	11.55239287
0.258933419	1.223632086	6.515695067
0.202722668	2.403728771	10.94430632
0.190281337	3.908543975	13.59210526
0.25468426	1.456452444	7.91408326
0.153749796	5.519041775	19.82933437
0.132456924	9.277582164	28.22781418
0.121991494	10.03844628	31.43159041
0.237347606	1.525956758	7.749103943
0.222472908	2.256212886	10.21396598
0.284762161	1.131846278	6.550819672
0.216152221	2.462309943	10.36848073
0.193503328	2.386190824	12.04286718
0.28773778	1.214285714	5.908730159
0.239042174	1.614419548	7.94108874
0.186468348	3.33073774	13.25143266
0.233198573	1.412128419	8.462068966
0.186039929	2.875482927	13.0849307
0.134610434	7.972005473	26.67494522
0.240475545	1.688556116	8.100257069
0.259775381	1.261423571	6.731958763
0.188330497	2.913732868	11.52459016
0.227573696	1.900589569	8.561904762
0.152662759	8.797276943	23.71144654
0.248621689	1.832251282	7.671388102
0.149752414	7.051975904	22.81800391
0.163335346	5.327209665	18.81711491
0.1424598	7.537843126	24.09094041
0.315846209	0.818166195	4.833992095
0.193857124	2.64283241	12.20394737
0.281908917	1.130411792	6.349593496
0.193461477	2.225896506	12.87862797
0.169288552	4.910941898	15.34696406

log.sigma.4.5.mm.3D_gldm_LargeDependenceLowGrayLevelEmphasis	log.sigma.4.5.mm.3D_gldm_SmallDependenceHighGrayLevelEmphasis	log.sigma.4.5.mm.3D_gldm_LargeDependenceHighGrayLevelEmphasis
0.072135698	130.6087286	2084.630312
0.291379656	50.12811658	4156.832451
0.24165924	58.30443664	1110.052995
0.041579537	108.7232269	4880.535104
0.056762433	144.1411652	3245.607242
0.24359279	44.36808655	1427.796267
0.551970308	24.83785125	1209.543027
0.072324025	136.3308717	2184.877246
0.066506415	127.824521	3157.529582
0.143497008	68.6621213	2885.705617
0.099179772	60.03984289	1990.935913
0.207346888	63.94467574	1906.608637
0.280259137	38.22649333	2250.007715
0.092891746	78.14478936	2600.22093
0.128306422	69.72466778	2085.13831
0.340417487	35.48600559	13984.09097
0.28240936	92.70360776	1757.206304
0.383385504	69.03987052	1734.81175
0.058746681	150.0435221	3363.546392
0.375576472	31.70154821	2984.36487
0.215268819	41.22065469	5211.49464
0.243984823	54.57541559	1719.634302
0.123159553	67.74547472	2543.144269
0.121308365	79.65059067	3232.700624
0.679347155	29.46014646	1067.240033
0.11762945	99.11432866	1681.976945
0.318675057	39.11059728	10125.30525
0.437961499	48.90293188	1239.167481
0.059543392	105.634157	2298.822663
0.213284229	56.04316578	6797.222659
0.24379785	48.06187671	1769.708713
0.212428079	61.52550412	3893.215683
0.098466098	74.63435664	2695.311363
0.086557822	82.41840278	726.8942308
0.061438208	101.4478606	2586.629518
0.082450045	78.79157522	2610.042574
0.246153855	61.09425806	1786.919792
0.246395798	34.98297824	3372.210197
0.103120859	62.4674002	5450.807186
0.160411913	81.49355549	2066.193518
0.117626195	65.06567705	4424.664858
0.159021822	116.8689797	2047.293809
0.10095838	78.21307404	2068.373934
0.087251785	110.5919961	2485.546341
0.121709471	64.48760803	2698.903621
0.639920993	28.16908953	1456.622083
0.105140192	63.30904656	3239.92241
0.3663908	73.47181044	1417.780446
0.131229014	90.73697761	1473.074627
0.158034301	164.5310716	3561.422535
0.198176778	51.19486952	3457.051402
0.133971553	70.72874669	1685.781137
0.117560921	77.28468029	3574.98168
0.193533152	53.0302128	2270.208122
0.327941848	39.76488036	5250.956462
0.071320767	123.4401874	2101.410694
0.051506311	136.0273132	2471.891929
0.034667398	136.4993418	5157
0.289250393	65.85966638	1866.486486
0.216818587	43.41818371	1638.716232
0.207665031	78.17533437	1469.392638
0.101548478	75.87029944	3831.580504
0.305597331	31.10605296	3032.422977
0.224957094	39.50112887	6829.182351
0.130965561	90.52107015	1369.331797
0.110855327	72.20513248	2541.773342
0.12471518	153.1089608	2589.591371
0.157333749	83.01466842	2337.789803
0.148025017	120.7679142	1690.55157
0.256402607	59.20766055	1454.241671
0.191831986	76.28253211	2212.335197
0.139014055	92.53034531	1614.981399
0.249345088	44.6777512	2681.81308
0.196827489	51.59846114	5550.1757
0.223309201	38.75223353	5476.366885
0.047076372	151.2458754	2743.756272
0.142321506	70.08032478	1769.102954
0.191100998	92.44916895	1270.042623
0.133617135	81.14488836	1821.379252
0.08953445	77.26561501	2763.1026
0.096908239	179.5866093	1783.698413
0.059910768	106.8309369	2378.980611
0.05893547	95.37679084	4200.916905
0.087201458	55.42879693	1403.151724
0.215259559	47.68085848	1679.590091
0.215847864	41.1223517	4280.123265
0.330564745	91.33470362	1385.268638
0.022636255	230.9180305	3887.75451
0.088172155	74.78794348	2279.273224
0.272378827	60.05505026	948.1238095
0.104783916	88.5580049	6920.60327
1.040642337	93.36031409	862.9660057
0.52176786	24.62141691	2257.963796
0.151952586	49.56643355	3487.538631
0.378628593	35.11504972	3149.173958
0.047716565	210.9647167	2386.671937
0.060740038	76.70144993	3731.541941
0.609181947	53.98341915	616.5853659
0.288449378	38.99008958	2603.124011
0.151513319	115.7055689	3307.873606

log.sigma.4.5.mm.3D_gldm_SmallDependenceLowGrayLevelEmphasis	log.sigma.4.5.mm.3D_gldm_LowGrayLevelEmphasis	log.sigma.4.5.mm.3D_gldzm_DistanceZoneVariabilityNormalized
0.004141526	0.009773173	0.906476754
0.003390325	0.014657945	0.918515165
0.004879988	0.020733424	0.887410807
0.001505955	0.004198158	0.899844101
0.004387399	0.008918674	0.895743163
0.00391364	0.022351353	0.877452298
0.004092234	0.034525344	0.824459877
0.004147751	0.009854633	0.852028009
0.002120781	0.006510623	0.917240526
0.001890555	0.007957741	0.964337296
0.005205003	0.012846698	0.878330831
0.0034821	0.014815298	0.930842636
0.003916009	0.019332322	0.910945227
0.00289185	0.00928711	0.893470343
0.003819902	0.011676377	0.905891579
0.00088699	0.006292679	0.947565841
0.003820475	0.021444248	0.821698361
0.003705712	0.022238031	0.969316917
0.002620541	0.008917552	0.850618018
0.002802644	0.016072253	0.972608025
0.001484944	0.007893822	0.945332031
0.003845073	0.016507548	0.918364281
0.001670219	0.008585524	0.964251954
0.002874246	0.013610155	0.783460208
0.005908483	0.038031029	0.936116296
0.003482635	0.012702587	0.957466919
0.001341989	0.007630297	0.925028922
0.003819517	0.023889386	0.960969927
0.003502218	0.008509283	0.952349641
0.00222258	0.010997771	0.951397987
0.002437582	0.013183068	0.973038745
0.001712177	0.009857766	0.967549409
0.003011185	0.011430065	0.91839666
0.016097237	0.025664836	0.895061728
0.004728426	0.00987659	0.85491971
0.003308932	0.009890394	0.846822081
0.002957052	0.014645382	0.962361592
0.002413578	0.012483654	0.957010023
0.002615696	0.010711735	0.8698
0.004174928	0.015367668	0.941658658
0.00197224	0.007061541	0.915731318
0.003020837	0.013499295	0.876123611
0.004096849	0.012572904	0.916567709
0.00304968	0.011217554	0.846194178
0.003619526	0.011005865	0.905
0.003372122	0.021118127	0.995992
0.001883532	0.008069113	0.891361339
0.004864998	0.028739666	0.932286857
0.006258683	0.024486091	0.802469136
0.003359494	0.014221622	0.754092736
0.002490403	0.0110102	0.940649729
0.006159642	0.015788435	0.919450983
0.002401098	0.008625504	0.920392711
0.002998865	0.017164221	0.95183661
0.001697418	0.01095979	0.960310408
0.004304528	0.011379521	0.901982386
0.002125486	0.005947044	0.779838965
0.002189843	0.004561553	0.751676454
0.0036464	0.020009509	0.919261314
0.003674009	0.017082664	0.969472988
0.004089182	0.016004219	0.912355936
0.00249388	0.008183674	0.929780852
0.003201585	0.014338211	0.958165608
0.001442964	0.007449233	0.980081697
0.005702771	0.021416977	0.96001666
0.004020152	0.011088029	0.945398308
0.003950709	0.014972807	0.76446281
0.002890382	0.012633121	0.951461136
0.00815639	0.019533844	0.873882704
0.005082468	0.021648541	0.941148217
0.002842676	0.013222332	0.905687314
0.003722779	0.014727378	0.910454484
0.002356497	0.013295252	0.961767526
0.001068505	0.007245209	0.961932543
0.002110511	0.009184505	0.952012354
0.002796473	0.007145784	0.832215239
0.003733767	0.012040068	0.861688277
0.00683182	0.023403667	0.889273356
0.003013963	0.012072016	0.851127221
0.001381247	0.006998605	0.93439058
0.005665018	0.012210613	0.841480954
0.004208982	0.008956561	0.872520776
0.001657562	0.005119109	0.926987006
0.011618764	0.018570358	0.808673469
0.003342811	0.01579346	0.96346444
0.001537556	0.008530325	0.824782385
0.004253771	0.022586549	0.941743545
0.001750283	0.003771641	0.809539921
0.008586553	0.013369698	0.919063678
0.016086792	0.028533109	0.737283951
0.001589228	0.005638605	0.921991176
0.006889625	0.046052584	0.988095665
0.005018308	0.027322628	0.922306545
0.002325418	0.009336254	0.920678641
0.003230972	0.018195448	0.95382613
0.007249076	0.012717627	0.683396152
0.002824289	0.007171141	0.968093499
0.008679538	0.055808037	0.842449465
0.003636392	0.021979237	0.777585544
0.00332689	0.011826867	0.88859072

log.sigma.4.5.mm.3D_gldzm_LowIntensityEmphasis	log.sigma.4.5.mm.3D_gldzm_LargeDistanceEmphasis	log.sigma.4.5.mm.3D_gldzm_HighIntensitySmallDistanceEmphasis
0.010796824	1.147540984	353.3360656
0.016704965	1.127659574	231.6532882
0.017953922	1.179640719	184.5179641
0.005344633	1.15862069	384.9043103
0.012493845	1.165517241	412.3551724
0.018687434	1.196721311	187.5874317
0.029850429	1.291666667	133.7916667
0.010899241	1.24137931	368.2275862
0.006857163	1.129750983	409.5448886
0.007781941	1.054483541	280.238933
0.017035824	1.195205479	213.0111301
0.014615699	1.107594937	242.9915612
0.020481206	1.140127389	187.0355626
0.010410422	1.169354839	266.4905914
0.01361315	1.148514851	244.6765677
0.007910559	1.080829016	278.6183938
0.013785287	1.296819788	271.934629
0.013962014	1.046753247	257.1363636
0.009512995	1.243902439	440.7601626
0.014194961	1.041666667	180.6076389
0.008965947	1.084375	250.8898438
0.016004152	1.127906977	217.4578488
0.007761427	1.054616385	283.5802991
0.014231063	1.370588235	267.5411765
0.030276992	1.099099099	121.6614114
0.012396554	1.065217391	294.1865942
0.010052415	1.117021277	266.3568262
0.015716988	1.059734513	187.2577434
0.009348873	1.073264781	291.5510925
0.01170623	1.074766355	265.211838
0.01081613	1.041002278	206.5842825
0.007928758	1.049492386	276.1135787
0.012175948	1.127853881	270.3649163
0.034191282	1.166666667	193.9166667
0.014337146	1.236220472	312.1673228
0.011845435	1.250720461	264.5738473
0.011868397	1.057562077	235.2756772
0.01268492	1.065934066	187.1888736
0.015081473	1.21	295.465
0.014235668	1.090225564	253.6817043
0.009112933	1.132231405	309.4876033
0.010656946	1.199017199	314.9195332
0.013865087	1.13085622	248.7600969
0.011348783	1.251851852	332.2493827
0.0140031	1.15	246.35
0.016328118	1.006024096	149.4262048
0.00853289	1.172925764	269.4069869
0.014863111	1.105263158	222.7335526
0.028086916	1.333333333	248.0185185
0.013689598	1.430693069	437.7623762
0.011464815	1.091836735	232.4348073
0.017306849	1.126126126	213.5908408
0.009481083	1.124584718	289.7084718
0.017542398	1.074074074	226.7993827
0.009797868	1.060765191	225.5697674
0.012970519	1.15503876	331.3624031
0.005987914	1.377825619	369.5831539
0.006714191	1.435789474	430.8168421
0.018287435	1.126436782	246.4051724
0.015972815	1.046511628	185.7228682
0.013751816	1.137795276	258.1141732
0.00956211	1.109311741	288.962406
0.017036514	1.064122137	167.398855
0.008870762	1.030181087	261.2706237
0.021524476	1.06122449	259.8163265
0.014280445	1.084269663	248.3970037
0.013206464	1.409090909	387.2769886
0.010480685	1.074666667	285.8384444
0.019094765	1.202898551	292.2983092
0.020034088	1.091040462	205.7695087
0.011253592	1.148854962	261.898855
0.012789947	1.140939597	285.0307606
0.011204155	1.058489033	219.4843623
0.006066267	1.058231488	284.7846873
0.012141868	1.073796791	219.0639037
0.008006251	1.277310924	401.1811975
0.013012268	1.224226804	235.751933
0.020731536	1.176470588	249.3382353
0.010523785	1.242990654	258.586838
0.006385108	1.101873536	311.470726
0.012676115	1.260377358	397.0707547
0.011157886	1.205263158	296.8223684
0.006094426	1.113839286	346.0276228
0.032500559	1.321428571	179.9241071
0.014585741	1.055842813	197.3503102
0.009337902	1.291066282	224.3667147
0.015156806	1.09009009	256.228979
0.004435266	1.319775596	572.0052595
0.023281045	1.126760563	219.2042254
0.040618712	1.466666667	170.0166667
0.006435914	1.121972318	358.450692
0.021867382	1.017964072	232.0666168
0.027741737	1.12145749	129.0546559
0.010754031	1.124117053	233.0613017
0.01760774	1.070938215	177.2616323
0.01543858	1.591549296	412.8239437
0.010823132	1.048648649	301.9033784
0.036254455	1.25862069	138.737069
0.02492044	1.382352941	180.3063725
0.011743229	1.177631579	346.0024671

log.sigma.4.5.mm.3D_gldzm_LowIntensityLargeDistanceEmphasis	log.sigma.4.5.mm.3D_gldzm_HighIntensityEmphasis	log.sigma.4.5.mm.3D_gldzm_DistanceZoneVariability	log.sigma.4.5.mm.3D_gldzm_ZonePercentage
0.011874706	360.2852459	276.4754098	0.432011331
0.0179805	235.6847195	474.8723404	0.248916707
0.021095814	188.1736527	148.1976048	0.384792627
0.005959856	397.1896552	782.8643678	0.330170778
0.01325131	422.6068966	129.8827586	0.403899721
0.022094104	191.2021858	160.5737705	0.284603421
0.090837119	136.625	59.36111111	0.213649852
0.01236612	380.2965517	370.6321839	0.434131737
0.00745986	417.7771953	699.8545216	0.367003367
0.008231946	282.569807	849.5811578	0.284377017
0.018492894	220.4417808	256.4726027	0.353083434
0.022597256	246.7763713	441.2194093	0.319838057
0.021987325	192.8174098	429.0552017	0.227097396
0.011633612	274.4623656	332.3709677	0.33273703
0.014661528	251.9735974	274.4851485	0.332601537
0.008609067	282.2704663	914.4010363	0.138908882
0.01605448	285.3286219	232.540636	0.405444126
0.014642057	258.0792208	746.374026	0.330188679
0.010773795	457.8455285	209.2520325	0.422680412
0.014796409	181.6753472	560.2222222	0.205640843
0.009711276	255.178125	907.51875	0.183767228
0.026811128	222.1569767	473.875969	0.3
0.008886507	285.4421326	741.5097529	0.281581838
0.04117141	284.6941176	133.1882353	0.353430353
0.032292071	123.6006006	311.7267267	0.276578073
0.012988528	296.3876812	264.2608696	0.397694524
0.010680969	273.608156	521.7163121	0.162723601
0.016538027	188.6283186	434.3584071	0.315422191
0.00988415	294.8624679	740.9280206	0.440793201
0.012492757	268.6744548	610.7975078	0.252557042
0.012006393	207.998861	854.3280182	0.27618748
0.008417364	278.4511421	1524.857868	0.265767285
0.012849401	278.1628615	603.3866058	0.323167732
0.104321597	195.1111111	48.33333333	0.519230769
0.016329622	321.2913386	108.5748031	0.38253012
0.013300495	278.0317003	587.6945245	0.343564356
0.012276051	238.0699774	852.6523702	0.307638889
0.013314943	189.4574176	696.7032967	0.217054264
0.017348562	303.145	173.96	0.239520958
0.015415185	255.8245614	375.7218045	0.38036225
0.009981722	317.7272727	332.4104683	0.246268657
0.012203554	325.1523342	356.5823096	0.427072403
0.015155691	252.7027464	567.355412	0.376979294
0.015681171	341.3753086	342.708642	0.395121951
0.01533305	252.6810345	524.9	0.308839191
0.01640249	149.5481928	496.0040161	0.223518851
0.009676422	278.4253275	1020.608734	0.268401313
0.01635929	225.0526316	212.5614035	0.391080617
0.095975247	251.0740741	43.33333333	0.402985075
0.015622347	465.3415842	152.3267327	0.406438632
0.012780672	235.0759637	829.6530612	0.257593458
0.018291399	218.2642643	306.1771772	0.402660218
0.010655684	295.6187708	1108.152824	0.315100759
0.019083322	228.9567901	154.1975309	0.274111675
0.010426171	227.6774194	1280.093773	0.204353825
0.014135739	338.620155	349.0671835	0.440273038
0.010306152	387.5888052	724.4703983	0.423620611
0.009237376	457.0652632	357.0463158	0.367362722
0.019513828	250.5632184	239.9272031	0.320638821
0.016783713	186.7751938	250.124031	0.292849035
0.01533692	263.1929134	695.2152231	0.359603587
0.010100013	296.1266628	1607.591093	0.315626141
0.017863423	169.2091603	627.5984733	0.21197411
0.009027369	262.7585513	487.1006036	0.181984621
0.022308914	261.0714286	94.08163265	0.451612903
0.014702149	253.6666667	504.8426966	0.343850612
0.017230875	402.2727273	134.5454545	0.446700508
0.011106158	288.9111111	1070.393778	0.351892399
0.020768409	299.6570048	180.8937198	0.464125561
0.021225556	208.0693642	651.2745665	0.344107409
0.012706851	267.2519084	949.1603053	0.344736842
0.014031807	290.6465324	406.9731544	0.395925598
0.011794436	221.4809098	1183.935825	0.238196594
0.006418806	287.8957584	1338.048167	0.207117332
0.012507706	223.9016043	1780.263102	0.203703704
0.009279538	419.5042017	396.1344538	0.426523297
0.016962311	241.4716495	334.3350515	0.347358997
0.022667196	255.8345588	241.8823529	0.445901639
0.020178835	266.7071651	546.423676	0.363945578
0.007025161	317.1967213	797.969555	0.300070274
0.015013733	409.8037736	222.9924528	0.525793651
0.01807907	307.0315789	497.3368421	0.425055928
0.006531539	355.1305804	830.5803571	0.320916905
0.112396392	181.1428571	45.28571429	0.386206897
0.015201867	198.7269907	931.6701138	0.285166618
0.011095561	245.1325648	858.598463	0.190102264
0.015794623	261.0870871	313.6006006	0.428020566
0.005652352	600.85554	577.2019635	0.459407216
0.023821684	226.6619718	65.25352113	0.387978142
0.13551124	174.6	33.17777778	0.428571429
0.007010191	366.8226644	1065.821799	0.29081761
0.022140509	232.4011976	330.0239521	0.473087819
0.0289973	132.6315789	227.8097166	0.241682975
0.012710593	237.6316852	912.3925328	0.242298289
0.018514703	179.3302822	1250.466056	0.225800896
0.039555124	451.6549296	97.04225352	0.561264822
0.010987663	306.0486486	358.1945946	0.304276316
0.109533909	140.0689655	48.86206897	0.471544715
0.037881246	188.6078431	79.31372549	0.269129288
0.014137394	353.0756579	270.1315789	0.376703841

log.sigma.4.5.mm.3D_gldzm_IntensityVariabilityNormalized	log.sigma.4.5.mm.3D_gldzm_LowIntensitySmallDistanceEmphasis	log.sigma.4.5.mm.3D_gldzm_IntensityVariability
0.040644988	0.010527354	12.39672131
0.050458492	0.016386081	26.08704062
0.059198967	0.017168448	9.886227545
0.045588585	0.005190827	39.66206897
0.041426873	0.012304479	6.006896552
0.060825943	0.017835767	11.13114754
0.076003086	0.014603756	5.472222222
0.038689391	0.010532522	16.82988506
0.040285553	0.006706489	30.7378768
0.052518743	0.00766944	46.26901249
0.052308125	0.016671556	15.2739726
0.048514305	0.01262031	22.99578059
0.048814241	0.020104676	22.99150743
0.05074286	0.010104625	18.87634409
0.048110752	0.013351056	14.57755776
0.052413756	0.007735932	50.57927461
0.048733284	0.013217988	13.79151943
0.046085343	0.013792003	35.48571429
0.044880693	0.009197796	11.04065041
0.059353299	0.014044598	34.1875
0.063361545	0.008779615	60.82708333
0.052753741	0.013302408	27.22093023
0.055162583	0.007480157	42.42002601
0.04982699	0.007495976	8.470588235
0.069321574	0.029773222	23.08408408
0.04484352	0.012248561	12.37681159
0.052713646	0.009895277	29.73049645
0.059881353	0.015511728	27.06637168
0.046649176	0.009215054	36.29305913
0.049305616	0.011509599	31.65420561
0.06266053	0.010518564	55.01594533
0.050833086	0.007806606	80.11294416
0.043815693	0.012007585	28.7869102
0.054183813	0.016658703	2.925925926
0.051646103	0.013839027	6.559055118
0.047388484	0.01148167	32.88760807
0.049421908	0.011766483	43.78781038
0.05809232	0.012527414	42.29120879
0.0472	0.014514701	9.44
0.04602358	0.013940789	18.36340852
0.051780009	0.008895735	18.79614325
0.042855677	0.010270294	17.44226044
0.048608809	0.013542436	30.08885299
0.041670477	0.010265686	16.87654321
0.04646849	0.013670612	26.95172414
0.067660199	0.016309525	33.69477912
0.048802273	0.008247007	55.87860262
0.053978147	0.014489066	12.30701754
0.055555556	0.011114833	3
0.037545339	0.013206411	7.584158416
0.052822641	0.011135851	46.58956916
0.052187322	0.017060712	17.37837838
0.045617598	0.009187432	54.92358804
0.053726566	0.017157167	8.703703704
0.054946032	0.009640793	73.24306077
0.041310285	0.012679214	15.9870801
0.045227284	0.004908355	42.01614639
0.044609418	0.006083395	21.18947368
0.051716798	0.017980837	13.49808429
0.061354486	0.015770091	15.82945736
0.046255537	0.01335554	35.24671916
0.046624436	0.009427634	80.61364951
0.05872152	0.016829787	38.46259542
0.056787405	0.00883161	28.22334004
0.052269888	0.021328366	5.12244898
0.045224368	0.01417502	24.14981273
0.040418388	0.012200361	7.113636364
0.043742025	0.010324317	49.20977778
0.046138766	0.018676354	9.550724638
0.049584016	0.019736221	34.31213873
0.048177918	0.010890278	50.49045802
0.045028002	0.012479482	20.12751678
0.052904225	0.011056585	65.12510154
0.062175917	0.005978132	86.48670022
0.049367726	0.012050409	92.31764706
0.041381258	0.007687929	19.69747899
0.050643001	0.012024758	19.64948454
0.044685337	0.020247621	12.15441176
0.049465747	0.008110022	31.75700935
0.05639805	0.006225094	48.16393443
0.038234247	0.012091711	10.13207547
0.048261003	0.00942759	27.50877193
0.045734016	0.005985148	40.97767857
0.071428571	0.0125266	4
0.055724108	0.01443171	53.885212
0.053736293	0.008898487	55.93948127
0.044720396	0.014997352	14.89189189
0.041172849	0.004130995	29.35624123
0.059313628	0.023145885	4.211267606
0.065679012	0.01689558	2.955555556
0.04400091	0.006292345	50.8650519
0.046147227	0.0217991	15.41317365
0.062630104	0.027427847	15.46963563
0.051173987	0.010264891	50.71342079
0.05650539	0.017380999	74.07856598
0.037492561	0.009409444	5.323943662
0.044134405	0.010782	16.32972973
0.067776457	0.017934591	3.931034483
0.064013841	0.021680239	6.529411765
0.042222126	0.011144688	12.83552632

log.sigma.4.5.mm.3D_gldzm_HighIntensityLargeDistanceEmphasis	log.sigma.4.5.mm.3D_gldzm_SmallDistanceEmphasis	log.sigma.4.5.mm.3D_glcm_SumVariance	log.sigma.4.5.mm.3D_glcm_Homogeneity1
388.0819672	0.963114754	801.7789957	0.299479316
251.8104449	0.968085106	454.561377	0.41052328
202.7964072	0.95508982	341.3238935	0.349763013
446.3310345	0.960344828	1166.915161	0.332867221
463.6137931	0.95862069	1086.440557	0.303460977
205.6612022	0.950819672	362.9657105	0.337642369
147.9583333	0.927083333	195.8120229	0.416500594
428.5724138	0.939655172	895.0814208	0.275799132
450.706422	0.967562254	1018.418835	0.314733116
291.8933031	0.986379115	590.4846758	0.380276389
250.1643836	0.95119863	548.0710561	0.316546518
261.9156118	0.973101266	519.7727137	0.343941624
215.9447983	0.964968153	404.1829277	0.37822891
306.3494624	0.95766129	703.0145121	0.334498698
281.1617162	0.962871287	571.1325241	0.339430025
296.8787565	0.979792746	664.6291225	0.548647457
338.9045936	0.925795053	642.7161961	0.300296201
261.8506494	0.988311688	504.9296419	0.335646319
526.1869919	0.93902439	1186.161278	0.285585888
185.9461806	0.989583333	312.7794519	0.430434319
272.33125	0.97890625	517.2725818	0.448615837
240.9534884	0.968023256	462.3255892	0.346193337
292.8894668	0.986345904	600.8679193	0.373125105
353.3058824	0.907352941	828.9435903	0.328336577
131.3573574	0.975225225	188.279544	0.397175226
305.192029	0.983695652	604.2761133	0.305483487
302.6134752	0.970744681	581.5165082	0.485826436
194.1106195	0.985066372	310.2855975	0.37381221
308.1079692	0.981683805	820.453761	0.301225725
282.5249221	0.981308411	566.8184655	0.419651469
213.6571754	0.989749431	366.6616105	0.390598433
287.8013959	0.987626904	611.991228	0.390612967
309.3546423	0.96803653	705.2918935	0.332670072
199.8888889	0.958333333	418.9300034	0.287981752
357.7874016	0.940944882	834.6793852	0.30721704
331.8631124	0.937319885	721.3944236	0.31917965
249.2471783	0.985609481	449.1347346	0.353567786
198.5315934	0.983516484	435.2361788	0.409495877
333.865	0.9475	836.0878686	0.373287695
264.39599	0.977443609	561.7060009	0.326306584
350.6859504	0.966942149	714.8349388	0.387873333
366.0835381	0.9502457	783.010934	0.290785858
268.4733441	0.967285945	617.9452715	0.310197848
377.8790123	0.937037037	844.8190005	0.296953781
278.0051724	0.9625	577.3806941	0.352442252
150.0361446	0.998493976	206.8429392	0.442861522
314.49869	0.956768559	667.2862736	0.362756191
234.3289474	0.973684211	464.8637972	0.309940663
263.2962963	0.916666667	491.5629904	0.307158993
575.6584158	0.892326733	1275.53886	0.284026492
245.6405896	0.977040816	491.1711595	0.389405191
236.957958	0.968468468	513.4405731	0.301850839
319.2599668	0.968853821	665.8963368	0.349741413
237.5864198	0.981481481	500.6069267	0.363144512
236.108027	0.984808702	447.7952456	0.464959092
367.6511628	0.96124031	808.0860963	0.286477626
459.6114101	0.905543595	911.8912783	0.289741031
562.0589474	0.891052632	1285.262858	0.31470005
267.1954023	0.968390805	466.435975	0.355726898
190.9844961	0.988372093	401.1137456	0.356548461
283.507874	0.965551181	500.6838585	0.320842051
324.78369	0.972672065	772.6644547	0.35036705
176.4503817	0.983969466	331.960283	0.440234536
268.7102616	0.992454728	537.7498364	0.465609236
266.0918367	0.984693878	557.5630018	0.293878802
274.7453184	0.978932584	591.7756475	0.352844026
462.2556818	0.897727273	906.4944601	0.288042214
301.2017778	0.981333333	634.4666498	0.327634544
329.0917874	0.949275362	667.5015348	0.27861677
217.2687861	0.977239884	417.5102799	0.342290655
288.6641221	0.96278626	575.212573	0.336394234
313.1096197	0.964765101	626.9183727	0.297230282
229.4670999	0.985377742	401.7857303	0.398985717
300.3400431	0.985442128	599.5342481	0.429955349
243.2524064	0.981550802	532.1551544	0.444470948
492.7962185	0.930672269	1001.882002	0.279502957
264.3505155	0.943943299	522.7890195	0.326962528
281.8198529	0.955882353	562.2357795	0.282709445
299.1884735	0.939252336	579.0045756	0.315470844
340.1007026	0.974531616	744.0383358	0.353549464
460.7358491	0.93490566	917.9169816	0.252826493
347.8684211	0.948684211	793.1240411	0.30045988
391.5424107	0.971540179	969.1934738	0.350163704
186.0178571	0.919642857	385.5002866	0.338743296
204.2337125	0.986039297	388.3243812	0.367452601
328.1959654	0.927233429	528.9479775	0.424842098
280.5195195	0.977477477	537.1781154	0.296610413
716.256662	0.920056101	1626.602915	0.268760286
256.4929577	0.968309859	565.9073512	0.36865812
192.9333333	0.883333333	318.9684596	0.325925051
400.3105536	0.96950692	903.4694416	0.378859424
233.739521	0.995508982	405.2172357	0.290625183
146.9392713	0.969635628	244.4645511	0.41750045
255.913219	0.968970737	518.3548972	0.389673643
187.6048818	0.982265446	338.8022962	0.416613673
606.9788732	0.852112676	1259.399054	0.243972132
322.6297297	0.987837838	879.4330307	0.346483344
145.3965517	0.935344828	250.910974	0.297535989
221.8137255	0.904411765	484.8907394	0.375365772
381.3684211	0.955592105	705.3391731	0.347085486

log.sigma.4.5.mm.3D_glcm_Homogeneity2	log.sigma.4.5.mm.3D_glcm_ClusterShade	log.sigma.4.5.mm.3D_glcm_MaximumProbability	log.sigma.4.5.mm.3D_glcm_Idmn	log.sigma.4.5.mm.3D_glcm_SumVariance2
0.205054163	215.5979133	0.013212751	0.976893004	127.4214044
0.335144499	229.4030345	0.030951895	0.982821499	70.46358786
0.25810129	92.20884028	0.019980056	0.970264613	62.03043522
0.24267811	-17.53412446	0.012705835	0.983203787	89.05104152
0.208169455	-248.4705931	0.013935931	0.974163504	157.4812887
0.243507509	-41.59773614	0.015287037	0.968052834	65.97719157
0.338233364	170.290463	0.033041272	0.973041858	48.84200027
0.180993009	183.4496281	0.009643605	0.973236842	146.6447633
0.222833347	194.9052659	0.010229861	0.980911946	122.8082224
0.296117577	205.9195758	0.015292971	0.984208123	70.63230222
0.223173739	-110.3438517	0.012070056	0.970513373	79.43505257
0.253473988	286.6178666	0.011852621	0.980191134	96.9338322
0.291423616	404.2594194	0.013364035	0.979188029	92.50253797
0.240652575	-195.4775674	0.013106088	0.976905862	89.31240468
0.247697516	191.1429769	0.013697331	0.978774567	86.02348472
0.498495466	57.73689702	0.122636509	0.991690836	43.27865975
0.204498768	-203.0170148	0.010871887	0.972347872	108.8755936
0.247495773	509.2828705	0.011632338	0.97598105	111.1249862
0.192211307	-706.0326467	0.012264589	0.971583779	137.9158459
0.354984326	154.0444905	0.041919445	0.982506283	51.09333444
0.376287248	72.61464764	0.030742069	0.989697456	41.92310525
0.254503434	367.6032675	0.011518159	0.982474914	91.20501613
0.287181246	211.316357	0.017430516	0.98468201	71.7425317
0.234902339	-426.0946888	0.016272674	0.974160677	108.449919
0.312916774	68.50773967	0.020484629	0.975376719	42.25957522
0.212613842	248.4865467	0.01282311	0.972968278	96.28956675
0.420433928	146.4347819	0.09239369	0.990131197	46.98545121
0.288531205	227.1362869	0.016516624	0.979839644	70.85597386
0.2072869	-154.0300964	0.010945969	0.972412186	108.4024472
0.345966603	37.63801496	0.029639426	0.98443736	71.91961746
0.307133934	250.59154	0.020903172	0.982284842	56.41657757
0.309411267	215.2934056	0.02790558	0.986009087	80.56301711
0.241811694	51.8499059	0.010056926	0.979499665	101.8311264
0.191591485	64.75525137	0.022086767	0.95378356	88.5533247
0.212552663	-414.1866908	0.016878842	0.968758065	99.62646277
0.22638405	-35.52121296	0.011823651	0.976771776	98.77567469
0.266590195	442.8268068	0.013033225	0.982736423	91.77452561
0.328671124	15.01635766	0.021139595	0.982522985	65.1246679
0.288486852	-399.8437691	0.02434874	0.982799558	81.64715576
0.234413983	-1.635897447	0.015312832	0.975335072	94.27603137
0.306203684	64.68332166	0.021297257	0.988199288	67.46484901
0.196327254	72.43227087	0.011069797	0.973363075	133.2952779
0.216038966	-99.61635479	0.009795798	0.972130227	94.22308586
0.202949036	36.47129896	0.010901007	0.975127691	135.3313321
0.263117188	241.9583573	0.015181423	0.978657917	84.76663616
0.370408477	178.0613201	0.029301614	0.983689842	40.28108709
0.273880933	55.38902693	0.012330532	0.983357052	77.85145111
0.216539576	-30.4480199	0.011442921	0.968267235	97.08992908
0.210277242	-69.40637064	0.023857897	0.958459856	75.10498637
0.192019925	-540.9246748	0.01649781	0.977128884	153.3861586
0.306244181	196.9138846	0.029706205	0.9842391	71.86206481
0.208491487	-67.41679272	0.012566687	0.957156475	95.96145297
0.262636824	299.7853277	0.019096911	0.983777612	84.03381001
0.2730872	-117.9569541	0.019019938	0.977989624	68.07877246
0.400101335	236.8972752	0.067097014	0.984023266	61.5546578
0.191335713	-80.6675414	0.010920804	0.973764042	116.4249807
0.195815314	52.59051951	0.008005021	0.9733737	104.9879979
0.223076671	-318.3182762	0.014081971	0.982759942	107.2376406
0.26562831	251.4753342	0.014820037	0.978597593	89.9512029
0.263355563	-70.39919289	0.016294755	0.974859674	63.52751435
0.229884715	420.5026491	0.012234268	0.976132573	106.0198758
0.263372311	359.5658983	0.014013462	0.984923034	104.6392236
0.366881567	169.1932465	0.037165691	0.98612865	45.51600971
0.400359662	179.0587237	0.04391782	0.985490096	44.88171345
0.196781698	75.0814856	0.018568837	0.964663054	99.27975484
0.266556143	365.7210709	0.015891248	0.98073945	87.29637243
0.194682709	-481.8363533	0.019263565	0.971342949	127.2147678
0.239796293	271.4844965	0.012393445	0.975236614	118.6190383
0.1819243	-297.9399816	0.01352535	0.965158551	119.6119238
0.253077019	338.2088436	0.012009211	0.975002865	89.02078266
0.24842506	255.3386165	0.011626217	0.978427341	98.43074113
0.203004551	179.8865508	0.008618312	0.971978716	118.4285538
0.320078429	267.1037974	0.022610338	0.983078753	65.82821553
0.355389312	139.0235015	0.024497984	0.987294691	53.79212397
0.373920174	349.7489418	0.038617622	0.987579087	63.18485064
0.184924679	78.50260125	0.010667651	0.977656311	123.8844701
0.234675437	94.22026518	0.011086882	0.979217123	84.28722809
0.18754772	287.427867	0.009439495	0.964975838	129.0604965
0.224182943	204.0689691	0.010975759	0.972650429	96.98524778
0.263132274	67.14285936	0.012756347	0.983101542	70.55559819
0.159308071	-110.777066	0.011674525	0.966101279	153.3890309
0.205314687	-118.2980487	0.010305924	0.977776125	102.7694984
0.263844494	283.5525888	0.020405202	0.983577132	93.01691373
0.24379676	-59.42978083	0.030872813	0.963223517	49.13366617
0.28037476	234.1750499	0.013872672	0.979845944	67.95153169
0.348719807	251.0857596	0.026325818	0.985396086	56.62602454
0.202408945	410.4365923	0.010162148	0.973091253	127.1525994
0.174924469	-465.0510342	0.008091576	0.973623569	152.4230108
0.280143579	40.0589183	0.026069951	0.972974683	56.91383673
0.232037898	266.4591413	0.035339758	0.961417674	70.52576628
0.298789704	173.8759932	0.036802986	0.984443978	82.65774906
0.196142127	449.180199	0.01059317	0.970263208	120.1137403
0.338806266	127.102823	0.035836631	0.976104217	51.83944258
0.307132588	178.9959819	0.016739992	0.983997101	63.88279614
0.339036068	106.4291178	0.028229602	0.982069055	60.65489593
0.149890929	-842.0140302	0.015300979	0.965702554	197.9790821
0.257166997	-18.99352586	0.014612027	0.978980517	88.11149168
0.202065846	61.7682312	0.022432765	0.942937137	63.15533107
0.286744146	-221.5122203	0.02404201	0.977912069	74.54498313
0.260885353	234.7437042	0.017107872	0.982020477	111.6145311

log.sigma.4.5.mm.3D_glcm_Contrast	log.sigma.4.5.mm.3D_glcm_DifferenceEntropy	log.sigma.4.5.mm.3D_glcm_InverseVariance	log.sigma.4.5.mm.3D_glcm_Entropy	log.sigma.4.5.mm.3D_glcm_Dissimilarity
28.48920886	3.385924082	0.213232273	8.287744141	4.264747356
16.51712593	3.08754306	0.309517918	7.764130991	2.953920928
17.14049278	3.055812077	0.263592046	7.41789118	3.277659433
22.88338147	3.30710462	0.245589204	8.325009248	3.745925843
28.64078881	3.405970719	0.20956255	7.963444975	4.221621744
18.45627747	3.143285151	0.242789995	7.72864697	3.444618549
10.76649717	2.7000743	0.349817409	6.870737609	2.472204501
37.35502576	3.629702302	0.184612732	8.687540182	4.894758215
27.76658434	3.461348773	0.230512424	8.636658771	4.100047993
16.04717369	3.099449341	0.295095951	7.892450106	3.039524978
23.160806	3.261095926	0.228569176	7.958067215	3.883457795
20.30445693	3.220354036	0.25612166	8.170618228	3.503367869
15.17571969	3.039782553	0.28947968	7.961861226	2.978449416
19.32756477	3.187537104	0.246549519	8.078653062	3.503079829
20.46782158	3.232909796	0.249877399	8.005775547	3.537938246
8.984806357	2.611561389	0.359985402	6.696048883	1.880548974
26.89402303	3.325714082	0.213878141	8.187499756	4.159521718
24.95996048	3.355791593	0.247448428	8.40419697	3.841235903
35.49650797	3.552237871	0.192753106	8.292140643	4.751391855
11.66728209	2.894081611	0.326315191	7.407675845	2.530190781
9.653774997	2.788798727	0.34893999	7.247100109	2.29066369
19.03745947	3.193511507	0.255651983	8.090518354	3.404522674
16.54394574	3.109718426	0.287225005	7.914297177	3.10570065
21.90699088	3.262620395	0.237308017	7.917066474	3.696029589
11.73627496	2.889785285	0.312874878	7.2835984	2.66016367
30.00178396	3.480556372	0.214326813	8.178917001	4.302191472
9.316595156	2.749157078	0.345258537	7.061538223	2.130953217
15.69476244	3.065607891	0.295795397	7.764492658	3.05244382
26.66710414	3.356381765	0.219823053	8.36002162	4.170059681
15.90800897	3.077304276	0.315387184	7.799437523	2.871151681
13.70177979	3.0007258	0.305385221	7.625171317	2.8383198
17.01257025	3.124022491	0.286499657	8.027920334	3.084788778
22.3454888	3.297690999	0.250098056	8.368518622	3.696057653
27.47927622	3.260870166	0.215561078	6.611543852	4.275352558
26.77553891	3.365580828	0.214386946	7.736314807	4.120248611
23.80915142	3.328876688	0.233335169	8.324563441	3.882719897
21.11837812	3.270273138	0.261987756	8.153759258	3.517353061
11.57136478	2.894985538	0.320309942	7.654341718	2.602081698
17.5284551	3.125184317	0.280741929	7.681849779	3.19024699
23.98920844	3.338351172	0.240028578	8.154164256	3.823171881
17.00216347	3.117842927	0.30205304	7.778003095	3.041414245
31.30944196	3.495975663	0.206779048	8.509603051	4.465696433
25.32706077	3.325224557	0.223790564	8.276023712	4.019128969
30.94219256	3.496618964	0.207529065	8.554332244	4.40570825
19.27060054	3.206569607	0.261951631	8.113843891	3.395475923
9.998616408	2.77424675	0.353664871	7.016883391	2.33550805
16.90743833	3.14588299	0.27416277	8.115615074	3.18406671
25.18510168	3.327728751	0.227114336	8.018099094	4.024073862
26.84841665	3.305518805	0.190878653	6.863103992	4.150153276
37.19615966	3.620044339	0.200880122	8.204957568	4.822858632
14.95546802	3.061572066	0.2929022	7.882691404	2.947164345
27.50515883	3.36650436	0.214146874	8.024869927	4.233383178
22.13788844	3.301077237	0.252316185	8.253613438	3.618868467
15.90276151	3.070059399	0.270594404	7.612272565	3.127230475
14.4277056	2.958535886	0.321847845	7.399723493	2.604637514
32.62220803	3.531769957	0.197327461	8.444944588	4.572996435
33.27922851	3.472965383	0.204393991	8.568268337	4.569650782
27.64989898	3.425955693	0.227330529	8.364277816	4.114205982
17.96632012	3.152028649	0.266312223	7.93078109	3.287979867
14.24703351	2.92519565	0.271074733	7.583585386	3.040553805
26.32453941	3.42523566	0.23343086	8.407323782	4.003680728
21.67578855	3.301730393	0.257236456	8.439303024	3.57989779
10.63427037	2.81732372	0.336759329	7.229838068	2.417546768
11.27592793	2.81501687	0.35649482	7.11182419	2.349660303
28.40382306	3.373030165	0.199096636	7.434892845	4.292355453
20.98777613	3.263777621	0.268155999	8.109202655	3.513485152
33.91083338	3.507079896	0.2038362	7.978192093	4.624597995
27.4238709	3.447482678	0.241600375	8.59242955	4.034572261
31.99184156	3.466278906	0.188466058	8.05681979	4.615808018
21.17471203	3.273058531	0.257604859	8.189142483	3.578874093
25.17167039	3.344500507	0.24356997	8.348308622	3.861184502
29.09191892	3.454731395	0.21046653	8.469917537	4.320453646
16.22632401	3.104499307	0.302880165	7.834684841	2.980899868
12.81637907	2.951333894	0.321732017	7.525748192	2.617473583
12.640507	2.932009845	0.339885714	7.547325754	2.517093795
36.18530118	3.560445216	0.188462076	8.533862176	4.809591596
22.64715263	3.268319367	0.243541323	8.064343736	3.745464248
32.30478015	3.499109532	0.198600422	8.29588484	4.592349583
26.60935244	3.379124951	0.229498132	8.334643965	4.072283789
17.05928507	3.13296086	0.267429915	7.95394413	3.257584719
48.03269943	3.705365854	0.166826418	8.41713584	5.587131022
27.32702906	3.417737735	0.213259715	8.368950569	4.19267446
22.43368062	3.3066146	0.258921398	8.275728791	3.624589122
18.00258597	3.059566165	0.245515813	6.675076119	3.391106597
16.92230842	3.110907819	0.281302764	7.900219103	3.16192744
12.11732719	2.918641456	0.328957989	7.533204268	2.572271435
29.68093817	3.460595005	0.205772094	8.334472557	4.36466988
40.9638361	3.691176742	0.182837475	8.856987073	5.120277791
14.17214532	2.82797358	0.292383121	6.725908355	2.970936292
22.86346489	3.14108652	0.222861641	6.31768317	3.791285347
21.34100154	3.275130248	0.276424544	8.152256328	3.416885368
33.51207544	3.549003542	0.20392597	8.25731938	4.547844389
11.43598687	2.879970799	0.325985985	7.346371588	2.558652945
15.2547805	3.062507833	0.30042481	7.824704976	2.946614371
12.96165995	2.971686331	0.320263655	7.652349286	2.67256903
48.5313206	3.660016638	0.161672069	7.85823029	5.686750865
20.25377157	3.244842968	0.261020912	8.112282191	3.490189672
26.36849587	3.242608858	0.210022196	6.746375398	4.185425927
13.58284803	2.952095301	0.288684559	7.380839958	2.90168258
24.87296298	3.357267269	0.257236251	8.096581692	3.747879824

log.sigma.4.5.mm.3D_glcm_DifferenceVariance	log.sigma.4.5.mm.3D_glcm_Idn	log.sigma.4.5.mm.3D_glcm_Idm	log.sigma.4.5.mm.3D_glcm_Correlation	log.sigma.4.5.mm.3D_glcm_Autocorrelation
9.085103801	0.894485117	0.205054163	0.636512099	271.1989151
7.29501661	0.916371751	0.335144499	0.621676446	165.2079735
5.784681453	0.882905072	0.25810129	0.565252775	127.9170656
8.022610768	0.910568421	0.24267811	0.593127311	379.1270639
9.892174794	0.890250978	0.208169455	0.694392201	355.2992603
6.195466847	0.877513341	0.243507509	0.564850391	135.1377666
3.889351617	0.892961093	0.338233364	0.640728665	80.00895389
12.66974964	0.886992547	0.180993009	0.596246753	298.1729554
10.39070268	0.905711091	0.222833347	0.632064815	336.9188272
6.460210486	0.915685999	0.296117577	0.633078203	207.9176372
7.180690885	0.881386718	0.223173739	0.551184652	193.1007642
7.282682943	0.904123854	0.253473988	0.655745585	186.1086125
5.88381367	0.903408696	0.291423616	0.716125167	150.1178833
6.666753372	0.894793602	0.240652575	0.645926407	242.261751
7.379067793	0.900404697	0.247697516	0.619631016	201.4465273
5.267433113	0.948493413	0.498495466	0.658047097	225.9325632
8.149431928	0.885058402	0.204498768	0.607401013	222.6706085
9.322482013	0.896710356	0.247495773	0.63487815	180.5271308
11.39544832	0.884565487	0.192211307	0.596224803	383.2259382
5.050145904	0.913927509	0.354984326	0.62814455	120.0538532
4.251093823	0.932726062	0.376287248	0.625654495	183.8806215
6.94550082	0.908848134	0.254503434	0.656711344	167.9969251
6.501960772	0.916292745	0.287181246	0.628591192	210.9758725
7.644126402	0.890184802	0.234902339	0.666421903	279.0361086
4.394029854	0.894471814	0.312916774	0.565469174	77.97315458
10.75270329	0.888703063	0.212613842	0.529030951	209.4515509
4.630931147	0.937632084	0.420433928	0.668952789	203.1077563
5.939351778	0.904357648	0.288531205	0.63777624	119.0324207
8.253018875	0.884651819	0.2072869	0.606819347	276.7191507
7.346252881	0.920854415	0.345966603	0.638953592	200.6834487
5.332681525	0.910241994	0.307133934	0.609399831	137.3057834
6.922025683	0.921482285	0.309411267	0.653466325	215.0589392
8.109849022	0.902208193	0.241811694	0.641918028	243.4169667
8.38857779	0.853218467	0.191591485	0.522423545	146.4305807
8.848877649	0.879521437	0.212552663	0.580054959	278.5314208
8.118130974	0.894761037	0.22638405	0.613352299	247.3262451
8.29134861	0.91152608	0.266590195	0.627435848	163.1388263
4.5706303	0.911078636	0.328671124	0.69834122	160.3317738
6.716804404	0.912014371	0.288486852	0.649295589	280.6270785
8.863022884	0.893743447	0.234413983	0.596890374	197.9721073
7.408374224	0.92814654	0.306203684	0.602112297	244.7380064
10.44894506	0.887533231	0.196327254	0.621476321	265.1505165
8.036559975	0.885283662	0.216038966	0.580473031	215.164832
10.50365402	0.891797379	0.202949036	0.631592935	284.389511
7.285462468	0.901326338	0.263117188	0.629942902	203.8430066
4.276106149	0.916824531	0.370408477	0.60091814	84.19813042
6.45839858	0.911769585	0.273880933	0.644416765	232.0008724
8.202892008	0.878036282	0.216539576	0.589975144	166.772569
8.855572834	0.862332282	0.210277242	0.474693571	170.4707795
12.90578914	0.896043477	0.192019925	0.612924961	409.2897349
5.950856532	0.915481922	0.306244181	0.656052694	177.5809339
8.434188677	0.859871161	0.208491487	0.55636525	180.9781836
8.406726915	0.913594079	0.262636824	0.5849663	230.3857452
5.820880486	0.898699339	0.2730872	0.623062783	179.3110101
7.190322558	0.923950454	0.400101335	0.626078511	162.0730654
10.7867609	0.888021024	0.191335713	0.565361921	271.959929
10.17680575	0.888426167	0.195815314	0.526282092	302.8122735
9.767344022	0.90949823	0.223076671	0.595780552	413.1077744
6.770473875	0.901021445	0.26562831	0.666559404	169.3904915
4.453244276	0.88944086	0.263355563	0.634211335	148.5039503
9.800820235	0.895403036	0.229884715	0.60349275	178.544889
8.324975288	0.916403903	0.263372311	0.66028501	264.2593099
4.431960408	0.922564254	0.366881567	0.622832039	125.830988
5.471735706	0.925533095	0.400359662	0.596832721	189.2673765
9.347601271	0.871122974	0.196781698	0.556917872	193.3488245
8.123673752	0.906851726	0.266556143	0.614117372	207.7621813
11.27784503	0.884386825	0.194682709	0.580543717	299.9562262
10.5529823	0.895156028	0.239796293	0.62447621	220.7945908
9.71873668	0.87051718	0.1819243	0.580902995	228.2638387
7.842898875	0.893631025	0.253077019	0.617185032	153.1466664
8.934963973	0.901576934	0.24842506	0.598390798	202.3853683
9.559671451	0.884638812	0.203004551	0.608112085	217.6480128
6.918880029	0.915287125	0.320078429	0.607864235	148.5657145
5.711112068	0.926664134	0.355389312	0.616235891	209.547094
6.093575164	0.929626519	0.373920174	0.668453963	189.2809908
11.2553081	0.89596226	0.184924679	0.550510924	329.2630233
7.715126822	0.900911929	0.234675437	0.579842987	185.6135125
10.31079752	0.871461402	0.18754772	0.602139202	196.0688887
8.940028523	0.887674188	0.224182943	0.571477706	202.8287892
6.068247793	0.909635975	0.263132274	0.612856635	254.044213
14.59263439	0.873669917	0.159308071	0.526738263	301.4484199
8.984537476	0.895880624	0.205314687	0.58303622	268.3458927
8.607592586	0.913590137	0.263844494	0.612819231	321.4486163
6.025158141	0.869814006	0.24379676	0.467957428	138.8665933
6.356341527	0.904482605	0.28037476	0.600885259	144.2282732
5.206283328	0.92084285	0.348719807	0.647888141	188.2521591
9.914629945	0.886692876	0.202408945	0.624023954	189.1347896
13.5765971	0.88788673	0.174924469	0.581970497	511.5710137
4.142270069	0.888127418	0.280143579	0.58916269	195.6219488
7.309308376	0.867978068	0.232037898	0.518774721	115.2705495
9.020877931	0.918595373	0.298789704	0.591077744	301.2367838
12.03677421	0.883325623	0.196142127	0.567154757	145.191979
4.652253431	0.898660485	0.338806266	0.637395255	97.4213097
6.15385026	0.915685329	0.307132588	0.616173659	185.3925692
5.580227755	0.912707954	0.339036068	0.646889797	128.824927
13.29272408	0.871274401	0.149890929	0.608155851	400.0025595
7.63437646	0.901803378	0.257166997	0.628730143	294.9632053
7.692412374	0.838929875	0.202065846	0.42055267	92.9817528
4.845683514	0.89814746	0.286744146	0.693969807	174.7598215
10.0089659	0.911441132	0.260885353	0.639739053	241.9950259

log.sigma.4.5.mm.3D_glcm_SumEntropy	log.sigma.4.5.mm.3D_glcm_AverageIntensity	log.sigma.4.5.mm.3D_glcm_Energy	log.sigma.4.5.mm.3D_glcm_SumSquares	log.sigma.4.5.mm.3D_glcm_ClusterProminence
5.429580362	15.83942895	0.003971634	39.74219978	45316.61684
5.036628873	12.36312903	0.007715949	22.39941428	17406.35316
4.892423073	10.80011864	0.007252243	19.792732	9427.267622
5.252400292	19.20239987	0.00406217	27.71500742	22802.06914
5.470335529	18.05292454	0.004773082	46.79374303	58121.52004
4.970755247	11.10074696	0.005682067	21.10836726	10377.77078
4.667879502	8.39387274	0.01133324	14.90212436	6607.052632
5.557402653	16.64529527	0.002992812	46.13584631	55625.12479
5.465746626	17.86933498	0.003371988	37.65140162	44855.75491
5.076019605	14.01469823	0.005825244	22.47470779	14305.71329
5.112396751	13.38815728	0.004845013	25.80724778	14986.66203
5.27870474	12.99523626	0.004415626	30.25770796	25818.7068
5.217548985	11.44130427	0.005186315	27.04446672	23411.29154
5.211324649	15.02700754	0.004556125	27.59808849	19425.52442
5.181816387	13.68984472	0.004998536	27.73764434	21702.54304
4.559183059	14.77888212	0.029993664	13.38782179	9192.058875
5.332042329	14.30333296	0.004164949	34.96151286	30049.46735
5.373134482	12.701521	0.003857082	35.20899915	34453.18068
5.444864485	19.12988858	0.003956206	43.18945188	52385.6963
4.81830607	10.49721696	0.009120175	15.69015413	8830.528455
4.716340397	13.27577315	0.010152715	13.10375396	6057.984896
5.226809498	12.28911943	0.004689915	28.12655922	25198.94635
5.081049241	14.11685401	0.006067683	22.83474195	16473.04198
5.245723891	16.12206874	0.005063581	33.41263737	27855.25953
4.690139657	8.386514081	0.008171625	13.49896255	4848.781411
5.237314341	14.03437933	0.004340974	32.99718119	25077.35443
4.714566317	13.94457261	0.017529671	14.41258147	8465.925663
5.044161587	10.26445268	0.006003151	21.77451102	14063.35046
5.333519737	16.14710803	0.003706895	34.8396339	25727.00806
5.079969103	13.73200409	0.007640158	22.7167093	16354.8801
4.891889735	11.25801858	0.007242104	17.64362983	11237.36804
5.172722591	14.21806339	0.005865367	25.25161066	19382.05543
5.337295429	15.05504662	0.003860271	31.89543382	27326.07498
4.728264378	11.44902966	0.011114311	29.00815023	16216.09781
5.156401009	16.20252032	0.00566133	32.31739227	26693.12341
5.285521579	15.23492746	0.003842359	31.61343477	22539.93273
5.218575386	12.15419649	0.004665887	29.30728192	23927.66826
5.005743346	12.12169609	0.006670785	19.17400817	10830.07222
5.065539061	16.33797789	0.007458409	25.26883954	23163.63041
5.242242433	13.4827326	0.004582147	30.22769865	24389.34359
5.027613131	15.39601569	0.006840546	21.80393029	14909.17027
5.471719213	15.60634014	0.00336948	41.86892481	44768.40074
5.253133247	14.09742378	0.003890034	30.26330817	20492.91468
5.506758774	16.1973735	0.003365198	42.21890658	48637.20373
5.188731053	13.72386109	0.004767001	26.45323438	20146.1665
4.601059407	8.753103735	0.011426255	12.56992587	5199.95198
5.167518523	14.79678474	0.004736209	24.39471121	16261.7026
5.218904528	12.21218095	0.00456606	30.8768255	22155.29891
4.764699005	12.58117622	0.009669033	25.48835075	14287.95448
5.501243245	19.70268438	0.004470132	46.55058784	73426.79707
5.084931365	12.81315831	0.006097634	22.20181364	15238.40205
5.16938959	12.79939902	0.004599871	30.86665295	17882.00057
5.197800652	14.79064304	0.004674546	27.27422898	23703.44539
4.991411753	12.89456504	0.00664097	21.01959128	13675.92565
4.865873596	12.28621723	0.015147193	19.41856801	13928.12551
5.387009861	16.02789808	0.003567829	38.07392507	35000.39226
5.350010855	17.11473401	0.003195008	35.31159373	27823.04407
5.336577193	20.03126772	0.004224026	33.01158083	34684.27118
5.205165136	12.32603933	0.005288122	27.39042132	22284.48583
4.965813733	11.66838883	0.006424143	19.44363696	9909.373272
5.322798606	12.66582745	0.003786167	33.95720857	28456.88222
5.363985709	15.75590898	0.003946064	32.03008271	36133.68316
4.718644448	10.82995876	0.010992999	14.20824845	8789.644591
4.696402256	13.45991295	0.012520194	14.21512137	7260.013042
5.097563855	13.29700892	0.006656752	32.69957959	25045.35042
5.193262999	13.92313606	0.004959656	28.08883338	22317.05227
5.348875489	16.75611257	0.005180187	40.64426746	45371.05651
5.429848496	14.16118231	0.00334465	37.4182465	36253.66896
5.323652397	14.41659445	0.004508917	38.57086974	34029.86934
5.215397007	11.70020518	0.004454902	28.09529845	21813.29074
5.299111292	13.70766777	0.004063654	32.09451759	24558.82611
5.401398785	14.0764911	0.003353855	37.88622111	33621.47625
5.008775712	11.70559005	0.006556053	21.08090383	13884.42716
4.874006455	14.18325731	0.007938578	17.30248963	8529.092341
4.925964611	13.35099768	0.010937987	19.68551113	15797.14751
5.430066007	17.80047204	0.003354554	39.84263028	43614.29351
5.151491341	13.11798077	0.004481657	27.49961194	17678.80266
5.407612109	13.20913508	0.00372187	41.83836761	39352.27763
5.264855551	13.72004883	0.003860294	32.18732219	21786.05358
5.07543795	15.61896043	0.005098754	22.70980114	13492.82068
5.522205839	16.82094717	0.003498608	51.19547617	60273.55534
5.315485054	15.96005391	0.003649141	33.24347107	24859.06047
5.259583738	17.60458956	0.004621293	28.94716311	26751.76397
4.557580453	11.4464917	0.011573482	16.78406304	6326.122873
5.033025362	11.47461776	0.005458924	21.3883391	13319.79954
4.884592189	13.33246961	0.008494643	17.54719544	10354.90939
5.423384721	12.92462695	0.003725982	40.28323711	40305.98803
5.591640335	22.22634812	0.002668422	47.07098267	57313.27782
4.637012214	13.59821871	0.011289403	17.77149551	7172.085406
4.571421303	10.16013648	0.01456002	23.34730779	15193.96058
5.167812627	17.05231783	0.006483853	26.25734323	24671.00482
5.345204652	11.23039764	0.003879807	40.04456628	35781.21149
4.809916767	9.344190587	0.008608295	15.81885736	7242.694203
5.005386336	13.20001975	0.006294824	20.28591481	13126.93115
4.946356344	10.81299789	0.007422329	18.43352893	10484.87241
5.505988004	19.35571752	0.004949157	61.59490275	92831.04108
5.216214273	16.76448587	0.004774347	27.75551494	21100.56805
4.604525958	9.149668431	0.010315639	22.38095673	8786.145901
5.003497609	12.62858312	0.007546992	22.03195779	13966.48632
5.319103348	15.04417672	0.004948796	35.39196915	38324.32789

log.sigma.4.5.mm.3D_glcm_SumAverage	log.sigma.4.5.mm.3D_glcm_Imc2	log.sigma.4.5.mm.3D_glcm_Imc1	log.sigma.4.5.mm.3D_glcm_DifferenceAverage	log.sigma.4.5.mm.3D_glcm_Id
31.39640705	0.921679621	-0.209340861	4.264747356	0.299479316
24.6344241	0.837094586	-0.153420035	2.953920928	0.41052328
21.60023728	0.889697989	-0.197929195	3.277659433	0.349763013
38.08262679	0.789320654	-0.121763542	3.745925843	0.332867221
35.94565161	0.968826697	-0.300365049	4.221621744	0.303460977
22.20149393	0.839908212	-0.151403351	3.444618549	0.337642369
16.78774548	0.899427136	-0.231181034	2.472204501	0.416500594
32.91158185	0.889251205	-0.169312382	4.894758215	0.275799132
35.39173553	0.838967462	-0.137902139	4.100047993	0.314733116
27.87606352	0.797772278	-0.130105257	3.039524978	0.380276389
26.756823	0.845591865	-0.15237089	3.883457795	0.316546518
25.84074609	0.853594287	-0.157714259	3.503367869	0.343941624
22.87121414	0.854195003	-0.165458808	2.978449416	0.37822891
29.98338103	0.847184879	-0.152958172	3.503079829	0.334498698
27.20637354	0.86828745	-0.166643758	3.537938246	0.339430025
29.48596238	0.867962627	-0.200304629	1.880548974	0.548647457
28.43196819	0.898473017	-0.189082029	4.159521718	0.300296201
25.21634781	0.831763108	-0.141872712	3.841235903	0.335646319
37.81743513	0.931130462	-0.219542304	4.751391855	0.285585888
20.99443392	0.793456644	-0.135696735	2.530190781	0.430434319
26.51874027	0.767334892	-0.125971192	2.29066369	0.448615837
24.4900507	0.844715808	-0.152195382	3.404522674	0.346193337
28.08335957	0.802088943	-0.131245812	3.10570065	0.373125105
32.08590221	0.921265014	-0.217486737	3.696029589	0.328336577
16.77302816	0.763398126	-0.123335112	2.66016367	0.397175226
27.77393881	0.88262447	-0.171822055	4.302191472	0.305483487
27.83414114	0.838672156	-0.168188876	2.130953217	0.485826436
20.51562498	0.831077634	-0.151744892	3.05244382	0.37381221
32.01501033	0.833813015	-0.142408776	4.170059681	0.301225725
27.32585834	0.846260752	-0.157731503	2.871151681	0.419651469
22.50514647	0.762785733	-0.119623918	2.8383198	0.390598433
28.22493437	0.807249268	-0.139116897	3.084788778	0.390612967
29.90234371	0.827172281	-0.137365596	3.696057653	0.332670072
22.89805932	0.990495694	-0.466259342	4.275352558	0.287981752
32.26295442	0.945231708	-0.253571556	4.120248611	0.30721704
30.23739612	0.814889809	-0.130677962	3.882719897	0.31917965
24.12219359	0.807033345	-0.130985187	3.517353061	0.353567786
24.24339219	0.819808612	-0.146448721	2.602081698	0.409495877
32.53106385	0.887058519	-0.19334288	3.19024699	0.373287695
26.86092017	0.859689621	-0.156218079	3.823171881	0.326306584
30.47021075	0.840883365	-0.152512664	3.041414245	0.387873333
30.9589304	0.890472937	-0.174396774	4.465696433	0.290785858
28.13611704	0.816733684	-0.133628677	4.019128969	0.310197848
32.14057492	0.893131128	-0.175423412	4.40570825	0.296953781
27.38313169	0.819723075	-0.137771596	3.395475923	0.352442252
17.50620747	0.768281119	-0.132724078	2.33550805	0.442861522
29.44574845	0.781440302	-0.118748444	3.18406671	0.362756191
24.39081466	0.896421239	-0.188146525	4.024073862	0.309940663
25.16235245	0.980706538	-0.388119727	4.150153276	0.307158993
38.99325651	0.952131894	-0.254550261	4.822858632	0.284026492
25.56123306	0.807764615	-0.135598442	2.947164345	0.389405191
25.59879804	0.849931309	-0.154482943	4.233383178	0.301850839
29.31930148	0.769179101	-0.11453349	3.618868467	0.349741413
25.78671565	0.879601838	-0.182432668	3.127230475	0.363144512
24.51833847	0.841063469	-0.16383571	2.604637514	0.464959092
31.68487848	0.869349043	-0.159540155	4.572996435	0.286477626
33.7543752	0.791479558	-0.121935656	4.569650782	0.289741031
39.65604284	0.85458078	-0.152662571	4.114205982	0.31470005
24.60519997	0.885020123	-0.181444088	3.287979867	0.355726898
23.33677766	0.848269011	-0.167914667	3.040553805	0.356548461
25.18740113	0.801117697	-0.123035591	4.003680728	0.320842051
31.20965295	0.806554804	-0.127586196	3.57989779	0.35036705
21.64314711	0.79230883	-0.141458691	2.417546768	0.440234536
26.896742	0.827438633	-0.161194803	2.349660303	0.465609236
26.49901595	0.969254256	-0.318890871	4.292355453	0.293878802
27.65285496	0.832665553	-0.143487377	3.513485152	0.352844026
33.25504225	0.951666414	-0.262366346	4.624597995	0.288042214
28.14153628	0.804707237	-0.122835338	4.034572261	0.327634544
28.7264629	0.929271333	-0.22290259	4.615808018	0.27861677
23.33912222	0.810683071	-0.130259693	3.578874093	0.342290655
27.13396378	0.79964529	-0.128162501	3.861184502	0.336394234
27.94717018	0.861122896	-0.15348281	4.320453646	0.297230282
23.3375297	0.768905328	-0.120059057	2.980899868	0.398985717
28.23466207	0.785083569	-0.130504525	2.617473583	0.429955349
26.58104882	0.807178016	-0.140711435	2.517093795	0.444470948
35.05793721	0.881970341	-0.168183169	4.809591596	0.279502957
26.08977017	0.839423839	-0.147770853	3.745464248	0.326962528
26.21638532	0.91632906	-0.201013926	4.592349583	0.282709445
27.21835633	0.800938834	-0.128567569	4.072283789	0.315470844
31.02677623	0.783409224	-0.12184116	3.257584719	0.353549464
33.16451376	0.94696282	-0.241619503	5.587131022	0.252826493
31.5892105	0.828265383	-0.13591386	4.19267446	0.30045988
34.85965252	0.812415441	-0.133448522	3.624589122	0.350163704
22.8929834	0.955579893	-0.310539357	3.391106597	0.338743296
22.93120836	0.762870318	-0.119110556	3.16192744	0.367452601
26.61720534	0.779122159	-0.12984353	2.572271435	0.424842098
25.6693544	0.900669846	-0.185970161	4.36466988	0.296610413
43.98228108	0.8539972	-0.142643814	5.120277791	0.268760286
27.19643742	0.949174536	-0.29959835	2.970936292	0.36865812
20.32027297	0.98337189	-0.431875966	3.791285347	0.325925051
33.81749177	0.800690613	-0.128658506	3.416885368	0.378859424
22.22606688	0.896208383	-0.181808031	4.547844389	0.290625183
18.68838117	0.821922017	-0.152544368	2.558652945	0.41750045
26.32348358	0.769011867	-0.120880659	2.946614371	0.389673643
21.62328065	0.785379455	-0.12848449	2.67256903	0.416613673
38.08198708	0.98572001	-0.370119874	5.686750865	0.243972132
33.34554678	0.847727089	-0.150639364	3.490189672	0.346483344
18.29933686	0.97283156	-0.35947912	4.185425927	0.297535989
25.25716625	0.922942553	-0.233885533	2.90168258	0.375365772
29.684893	0.919657899	-0.21059144	3.747879824	0.347085486

log.sigma.4.5.mm.3D_glcm_ClusterTendency	log.sigma.4.5.mm.3D_firstorder_InterquartileRange	log.sigma.4.5.mm.3D_firstorder_Skewness	log.sigma.4.5.mm.3D_firstorder_Uniformity
127.4214044	213.3373852	0.17447898	0.044848285
70.46358786	136.8523598	0.40424824	0.065074089
62.03043522	177.5179417	0.134357525	0.062010236
89.05104152	196.3603058	-0.075441853	0.050494835
157.4812887	234.9963264	-0.246974348	0.043877686
65.97719157	179.0614794	-0.223080373	0.060193639
48.84200027	120.4314346	0.383387924	0.077794116
146.6447633	253.8022175	-0.043806696	0.040135298
122.8082224	215.2356606	0.097875769	0.04386729
70.63230222	155.1713047	0.468967278	0.061500349
79.43505257	198.8698502	-0.32890127	0.056095004
96.9338322	189.3769684	0.347340712	0.051559788
92.50253797	187.7154884	0.381901261	0.053991235
89.31240468	196.9120045	-0.252219122	0.05362086
86.02348472	171.130497	0.261736511	0.055845074
43.27865975	68.98709393	0.343042423	0.116667273
108.8755936	206.9243908	-0.253139861	0.049638345
111.1249862	212.3059769	0.436795098	0.047589434
137.9158459	215.0585499	-0.557434555	0.046574792
51.09333444	129.1017723	0.398818696	0.073754324
41.92310525	116.0039989	0.331456919	0.079599707
91.20501613	184.3486443	0.372163497	0.054660627
71.7425317	148.5554848	0.444361333	0.064555872
108.449919	204.3069382	-0.343402702	0.050384464
42.25957522	129.1344486	0.31023164	0.074153431
96.28956675	200.1778364	0.272721369	0.049236353
46.98545121	101.4512844	0.516283335	0.090529416
70.85597386	160.5430927	0.388981913	0.060200469
108.4024472	245.5644073	-0.151855854	0.046879118
71.91961746	150.6947656	0.17418654	0.062662146
56.41657757	143.6675968	0.52896654	0.068365149
80.56301711	169.4065666	0.420670606	0.059176565
101.8311264	211.8345337	0.044014774	0.048304549
88.5533247	198.1806831	0.018366417	0.05510355
99.62646277	185.5193796	-0.504917919	0.055341849
98.77567469	232.0250225	-0.05387023	0.048880992
91.77452561	197.6617804	0.552200739	0.053686343
65.1246679	158.0728016	0.034201633	0.063330073
81.64715576	133.9328213	-0.554789135	0.06662842
94.27603137	192.0901947	0.007572029	0.0523182
67.46484901	143.6224527	0.263135079	0.063171867
133.2952779	237.3201675	-0.07945045	0.043587985
94.22308586	226.6223602	-0.203435856	0.049787773
135.3313321	221.1310654	-0.019259577	0.043120048
84.76663616	185.1439056	0.224320963	0.053867947
40.28108709	133.1647596	0.679886357	0.080028864
77.85145111	171.3272362	0.14845329	0.056121309
97.08992908	220.139369	-0.177458631	0.05059593
75.10498637	159.2902527	-0.296103423	0.061706393
153.3861586	179.7369614	-0.485399533	0.051305823
71.86206481	166.4055367	0.332887719	0.060983424
95.96145297	255.4517756	-0.2236743	0.052106286
84.03381001	175.5234222	0.406720606	0.053979232
68.07877246	146.9249535	-0.262720514	0.06318981
61.5546578	99.74084282	0.547892039	0.089683554
116.4249807	213.6368275	-0.098554742	0.045882887
104.9879979	237.4700928	0.020891955	0.044563091
107.2376406	179.1852722	-0.497759065	0.054567727
89.9512029	186.1013122	0.193457512	0.054585298
63.52751435	165.3852196	-0.155631365	0.06175394
106.0198758	234.7347412	0.343365813	0.047893383
104.6392236	196.0623302	0.320423608	0.050123225
45.51600971	104.6419163	0.471850499	0.083649312
44.88171345	110.7754173	0.62383682	0.080861868
99.27975484	199.8271065	-0.080017332	0.052050373
87.29637243	175.5958099	0.490523363	0.055889583
127.2147678	198.8083029	-0.410997655	0.049112319
118.6190383	226.2204208	0.220421371	0.045580443
119.6119238	229.5797174	-0.36507879	0.050352913
89.02078266	185.03405	0.39881903	0.053738006
98.43074113	211.3242283	0.356441955	0.050407072
118.4285538	246.4568405	0.06355415	0.043851563
65.82821553	143.212491	0.512756995	0.064780271
53.79212397	136.6478314	0.400077042	0.069816207
63.18485064	107.3422384	0.712083613	0.078347193
123.8844701	248.9135017	-0.031331347	0.043521409
84.28722809	209.0788574	0.065486484	0.052560374
129.0604965	237.3875675	0.127325838	0.043681806
96.98524778	224.1314392	0.202085162	0.049031139
70.55559819	172.7735214	0.209065779	0.059047749
153.3890309	264.3661221	-0.139437373	0.038840073
102.7694984	227.6114502	-0.140946248	0.04790297
93.01691373	188.7289009	0.300597397	0.054036143
49.13366617	130.0939255	-0.382076806	0.082330559
67.95153169	173.950695	0.422464588	0.059482397
56.62602454	129.0715399	0.615779617	0.073412516
127.1525994	238.5867853	0.238111092	0.043701799
152.4230108	258.4961567	-0.499668618	0.042294944
56.91383673	193.7077255	-0.067297685	0.066977216
70.52576628	159.5203896	0.275499194	0.074829932
82.65774906	162.1300964	0.246021928	0.058638756
120.1137403	244.1566882	0.322039533	0.044551357
51.83944258	139.1168056	0.312071206	0.069385457
63.88279614	155.8610964	0.353287916	0.063802942
60.65489593	153.512022	0.259246537	0.065346122
197.9790821	326.7150574	-0.371983714	0.040197472
88.11149168	177.4049282	-0.102916668	0.053052231
63.15533107	181.5016937	-0.011764418	0.061273052
74.54498313	148.3801708	-0.464220935	0.065127645
111.6145311	194.4519958	0.344811374	0.048628098

log.sigma.4.5.mm.3D_firstorder_MeanAbsoluteDeviation	log.sigma.4.5.mm.3D_firstorder_Energy	log.sigma.4.5.mm.3D_firstorder_RobustMeanAbsoluteDeviation	log.sigma.4.5.mm.3D_firstorder_Median
127.5819629	18784892.05	88.60925209	-33.04889107
90.03384104	29833855.76	59.25050212	-24.91845131
94.89950772	8235041.658	70.61568466	-78.35889435
111.8015484	50627870.88	80.45383106	19.72442627
137.2173032	10449930.63	99.17328148	-12.98468208
98.38141271	14530335.73	72.52505183	-79.34416962
76.50875727	3230062.088	52.56982688	3.220030546
145.8390195	32090382.42	107.0700343	-12.26688242
128.4458447	55308224.75	90.22682369	-36.56697464
93.92319775	46899897.24	65.70509953	-44.99240875
109.3699371	17249406.62	81.94963406	-38.21777725
110.7091185	29555207.92	79.12759484	-40.43164825
107.8736113	36064507.5	78.60606264	-1.061830699
110.1816579	19427246.59	82.03111139	8.737744331
103.2581464	16243412.85	71.82176538	31.52790642
60.04576691	54149476.97	32.53402404	2.358610392
119.2531082	17703525	85.71954512	-46.85752678
121.811816	52606892.56	87.7985532	-32.60829735
132.1242039	16118713.39	90.87204567	33.64246941
78.67693325	29679509.27	53.38180432	-19.160923
71.91773858	58000098.11	48.81291063	-57.15045166
104.5658936	29078962.6	74.71724909	-6.350009441
91.31633655	39492583.23	61.88867498	-45.68045044
116.6135317	9621328.507	86.13273067	34.84367371
76.99130192	16547834.03	55.074022	-76.26910782
116.0980119	16132424.3	81.9993987	-64.60771179
69.76083656	31841670.02	43.7779093	-18.5765419
94.72996024	26919686.3	66.60016571	-78.69030762
126.6559894	39115892.69	97.98215586	-1.559182048
93.13714867	46994171.12	63.22966215	-61.93058586
85.6049965	43318606.06	60.00333693	-60.12175369
99.5668343	93781779.89	69.43095559	-1.534805477
117.9477036	42436022.13	85.7295759	-7.41683197
110.9181966	2028553.125	82.86558532	39.60605431
109.7883094	6676504.748	76.36982247	-18.5664854
121.5031128	43238732.22	93.26218352	-22.84551144
111.5803695	55826273.8	81.98479669	-52.24685478
91.21176796	41843222.17	66.3427503	3.23551023
95.32545178	13190190.18	60.93863378	20.68393135
111.5923011	22192403.04	78.13227297	-32.53975296
91.11944019	21445423.85	61.25126546	-29.9211216
134.903513	26793238.08	98.83392648	-18.43029213
119.624805	36349857.41	91.42592267	-31.32967758
133.0249675	28159483.79	94.62104035	-10.43830109
106.9698988	32711793.51	76.80936096	-13.59192133
77.46685566	22427920.61	55.86315971	-52.9764843
99.81246199	65578481.29	70.94031338	6.534384251
118.1921564	16145919.89	89.04122142	-70.4032135
96.11428124	1976751.409	65.3507536	11.31532669
129.3346413	14663863.62	82.02076885	27.39900208
93.92300705	47988987.76	66.63476665	-13.46890163
126.4523073	25525354.49	102.5003064	-82.88767242
105.8081259	69390517.86	73.14502412	-24.69505119
91.80387703	7968776.638	63.26838359	2.371567249
76.11122077	72864161.43	46.15424974	-4.566620827
126.5251142	23697707.66	90.88571191	-42.58376694
127.4101916	55944035.45	95.55839504	-44.84646225
114.4962512	28307753.71	76.18078934	6.501895905
105.8125886	17396533.28	74.76093842	-67.90420151
91.91801468	11979459.96	67.1606714	-24.06128883
125.7235554	50208605.92	95.35870893	-46.93196869
113.1061837	111520729	79.07419282	0.533398002
70.47725064	27470564.88	45.82300171	11.62539673
73.8996289	26696933.28	48.59289586	-29.38769531
114.420414	4976060.305	82.50213694	-56.20290756
105.0836731	28607806.35	72.99734518	17.49959183
127.0676385	10943728.94	85.16959068	-17.91279316
126.1582907	76871724.04	91.30945762	-1.0517205
128.3749053	15615799.48	91.99168767	-71.18259048
106.7855123	35374327.28	76.73374459	-27.662714
116.6500132	61852264.04	85.80783949	-39.28856659
131.2847569	30668672.09	98.65918404	-43.42778015
88.9007133	67741255.44	61.16993045	-21.81886101
82.12910158	77176995.27	57.70987068	-35.66366386
79.88320838	111734214.3	49.03296374	7.685521841
134.570364	33290151.13	99.23992357	-43.53464317
111.2937791	25358472.07	84.24593101	-65.82236481
131.7477148	18391020.29	97.02468858	-71.85259628
118.3898908	48092511.83	89.77218236	-96.83890915
97.22530959	45872043.23	71.21715389	-55.47480392
151.729816	27502824.73	110.7642814	-129.3307571
121.581994	29481818.33	92.37408411	-20.96928024
107.4722315	53423129.37	74.21037687	16.83238411
76.14387762	1456449.843	51.62759228	21.23301506
96.86199493	47741145.34	70.42432164	-7.09273243
81.37392346	59363745.61	55.18984735	-20.99051285
133.215211	22011461.52	99.60024575	-54.18372154
144.6816176	52549553.88	106.1039297	-24.92644501
97.50529612	2461153.924	74.16277916	-31.05732155
95.14692133	1645999.842	62.85745596	-24.90848923
100.9783746	69565111.37	67.22430414	-11.24271965
131.5373398	23659109.76	98.40058681	-100.0638618
82.40833323	12973074.15	58.97088807	-49.65940857
90.66472972	55588706.3	64.13872908	-30.9143734
89.66968738	71752280.08	64.76623292	-5.121492624
173.1381387	12465386.75	132.3189637	-59.51681137
108.0235189	22171944.76	75.13706921	11.88215446
99.75584207	1789676.599	73.9641414	12.75130272
90.44882751	4927951.463	63.42780356	30.85391045
119.5115644	21234607.84	81.53382778	-56.18813324

log.sigma.4.5.mm.3D_firstorder_TotalEnergy	log.sigma.4.5.mm.3D_firstorder_Maximum	log.sigma.4.5.mm.3D_firstorder_RootMeanSquared	log.sigma.4.5.mm.3D_firstorder_90Percentile	log.sigma.4.5.mm.3D_firstorder_Minimum
507192085.3	417.1393738	163.1180426	176.8087997	-401.0256958
805514105.4	405.2608032	119.8495602	126.0065033	-322.6225586
222346124.8	221.6657715	137.748867	77.58814621	-346.3121033
1366952514	437.6737671	138.6131784	195.9908203	-427.155304
282148126.9	302.6502991	170.6119628	213.1920105	-456.6540833
392319064.8	194.4595032	150.3253896	51.30500031	-368.0233459
87211676.39	256.2740173	97.90175673	161.3166779	-193.0821533
866440325.3	449.4336243	178.959017	196.7314117	-425.3314819
1493322068	433.2673645	163.1051278	183.7490082	-456.9980774
1266297225	377.6947632	123.039693	130.3019669	-356.5655518
465733978.6	256.7643433	144.4223226	92.48349915	-382.313385
797990614	413.1094055	141.2189271	148.7605057	-349.9212646
973741702.5	372.4314575	131.8668484	185.3865952	-264.5558167
524535658.1	310.8047485	131.8210373	153.4020767	-354.7445374
438572146.8	427.7446899	133.5301856	192.3210754	-286.4067078
1462035878	432.0059814	88.28735045	111.7664688	-345.3651733
477995175.1	304.6390381	159.2583321	112.0827675	-424.4124146
1420386099	447.5340576	150.1955452	195.7426727	-321.7028198
435205261.7	373.8947144	166.4193013	210.6874252	-453.2406921
801346750.4	346.3106384	102.9370703	112.428627	-254.7250061
1566002649	362.9118652	105.368981	70.45292206	-373.5119019
785131990.2	497.1899109	130.0245116	169.269162	-299.7278442
1066299747	401.3722534	120.2532772	129.6381226	-368.8803711
259775869.7	305.0290222	141.4311209	187.4746857	-364.5426331
446791518.8	237.2254333	117.2350127	63.53211403	-272.9027405
435575456	389.2144165	152.4649735	146.5944855	-377.8259277
859725090.6	385.1824341	95.84812896	113.1601334	-338.1495056
726831530.1	345.3501587	137.0603707	95.33805542	-316.071106
1056129103	334.2801514	148.868999	176.3164581	-380.2156982
1268842620	351.0600281	135.9672218	83.2218338	-393.8313599
1169602364	346.0568237	116.73255	97.74995117	-309.7597351
2532108057	515.1109619	125.756919	191.1124222	-318.71521
1145772598	404.5005493	144.4769764	167.084082	-369.3640442
54770934.38	312.6590881	139.6614425	231.0895874	-245.9579163
180265628.2	244.6188049	141.8095697	116.6216873	-445.528656
1167445770	355.286377	146.3055467	150.1733704	-386.427948
1507309393	501.7914429	139.2268755	155.0827194	-317.2812805
1129766999	323.8504028	111.6943052	135.7934265	-277.3063354
356135134.9	365.6124268	125.6846647	143.4983398	-395.8618164
599194882.1	374.8088379	145.4502331	133.0800781	-362.9453735
579026443.9	506.2559509	120.619792	131.2285858	-396.9732056
723417428.1	396.5288696	167.6741646	172.0909058	-418.7731628
981446150.1	304.1697388	148.7869288	126.0430305	-387.959259
760306062.4	407.1051331	165.7488073	194.5319641	-408.2222595
883218424.8	366.0924377	131.9788635	162.9412247	-329.6226196
605553856.4	329.6971741	100.3314091	106.9962013	-228.5857849
1770618995	424.5612488	123.9853156	171.3715897	-334.1549683
435939837	260.2061462	166.4167818	80.72507629	-396.3009033
53372288.03	255.74617	121.4573019	156.3920609	-311.9682922
395924317.7	450.0537109	171.7694844	210.5000122	-485.5483093
1295702669	424.5169678	118.3869731	137.8430649	-315.9094238
689184571.3	158.1026154	175.6843743	78.93819733	-419.6582642
1873543982	507.5281372	134.7601685	157.8774414	-368.8497925
215156969.2	319.7432861	116.118679	146.898819	-309.6540222
1967332359	401.605835	105.6898536	138.0619537	-293.0955505
639838106.7	390.0300293	164.1945475	148.6790009	-444.7350769
1510488957	365.3594971	159.7193666	151.1952972	-463.5101318
764309350.2	429.8476868	147.9631068	143.8524231	-521.9158325
469706398.6	310.0187073	146.1905008	114.421508	-365.5038452
323445419	232.5446625	116.6086228	109.5544815	-300.6368103
1355632360	447.9127502	153.9301183	169.8474457	-339.6643372
3011059682	543.7527466	142.6812107	182.414061	-370.853241
741705251.9	418.5674744	94.28759435	129.7465302	-246.0350342
720817198.7	311.140564	98.87120747	116.8800125	-326.1083984
134353628.2	266.2333374	151.4303585	123.144931	-391.2666931
772410771.5	485.5157166	135.7239688	216.2871155	-289.2389832
295480681.3	336.7109985	166.6612185	159.4176987	-465.446991
2075536549	441.4383545	155.0643605	200.0981689	-335.7905579
421626586	233.1183472	187.1176189	82.48269272	-474.2964172
955106836.6	398.5303955	132.628867	170.5162506	-297.0776978
1670011129	457.4165955	142.6398944	177.0452179	-340.9743652
828054146.4	350.7849426	164.8164346	154.867926	-394.2388
1829013897	434.3682251	114.4894234	138.4061661	-284.9017029
2083778872	376.1961975	107.1984706	110.923912	-371.4864197
3016823786	470.1372986	110.3244444	163.7415222	-291.5515442
898834080.6	469.94104	172.7133015	139.2035446	-494.2493286
684678745.9	389.0614319	150.6728332	97.2001358	-377.5161133
496557547.9	319.8681335	173.635289	143.4511551	-383.1359253
1298497819	324.3112183	165.1161067	109.1669235	-406.3411255
1238545167	348.3460083	126.9569834	117.2905731	-424.8715515
742576267.8	307.8728027	233.6002926	83.40577164	-553.8774414
796009095	416.0766907	148.2732285	149.5569153	-410.1163635
1442424493	487.4134827	138.326999	200.3716354	-383.7950439
39324145.75	231.1104431	100.2221616	143.4076874	-267.7762146
1289010924	422.3180542	118.6540378	169.939682	-273.1745911
1602821131	374.4926453	104.1187458	142.1802597	-309.8453064
594309461.1	411.2622681	168.2033501	165.2761353	-373.6722717
1418837955	319.8850708	184.0088276	146.0885406	-621.4572144
66451155.94	194.0187836	115.9695156	140.8541626	-327.8419495
44441995.73	272.1745605	125.2045885	124.3483459	-293.0187378
1878258007	456.9324341	132.2900486	163.798761	-410.7748108
638795963.5	420.2764587	183.0614293	125.4931488	-368.2608337
350273002	240.0991364	112.6668111	92.39241943	-269.8834839
1500895070	393.3476257	116.5820344	112.3292999	-328.0271301
1937311562	373.8443909	111.1678835	133.0051041	-259.4368591
336565442.2	314.3788452	221.9691496	166.691153	-555.0003052
598642508.6	344.1383667	135.0315045	190.8487549	-389.6210632
48321268.16	255.3091431	120.6242772	177.1077789	-224.5022125
133054689.5	275.4507446	114.0285511	157.1112	-299.3151855
573334411.8	454.7907715	162.2128875	135.6687103	-422.8779907

log.sigma.4.5.mm.3D_firstorder_Entropy	log.sigma.4.5.mm.3D_firstorder_StandardDeviation	log.sigma.4.5.mm.3D_firstorder_Range	log.sigma.4.5.mm.3D_firstorder_Variance	log.sigma.4.5.mm.3D_firstorder_10Percentile
4.682330173	160.4685494	818.1650696	25750.15535	-243.0813446
4.225718672	117.4464309	727.8833618	13793.66414	-175.4404877
4.15995288	113.9111481	567.9778748	12975.74966	-219.0873795
4.487946751	137.2799635	864.829071	18845.78837	-167.3159332
4.674088502	169.8016138	759.3043823	28832.58805	-251.6195404
4.209446937	118.6169077	562.4828491	14069.97079	-267.834906
3.907762282	96.82162142	449.3561707	9374.426375	-108.7927155
4.801582709	176.5857905	874.7651062	31182.54142	-273.6385284
4.701725669	159.7372919	890.2654419	25516.00244	-244.2001678
4.238652528	117.7255059	734.2603149	13859.29474	-176.5909698
4.321055026	131.055268	639.0777283	17175.48326	-255.2499969
4.456094239	136.9124224	763.0306702	18745.01141	-208.4470306
4.365600008	131.8050512	636.9872742	17372.57151	-163.6532974
4.381355973	131.6925124	665.5492859	17342.91783	-189.7558105
4.385461307	129.5836202	714.1513977	16791.91462	-136.0531464
3.661684125	87.95518275	777.3711548	7736.114172	-96.3056076
4.519633664	145.7478683	729.0514526	21242.44111	-273.5353149
4.563173044	149.2527845	769.2368774	22276.39369	-195.7890579
4.648461047	166.1174526	827.1354065	27595.00805	-242.8826263
4.011940608	101.4119095	601.0356445	10284.3754	-151.5876617
3.909568843	92.11315741	736.4237671	8484.833768	-162.2684845
4.377919631	130.0241369	796.9177551	16906.27618	-170.4654037
4.226622099	116.3755682	770.2526245	13543.27288	-164.1645813
4.449485182	140.2938617	669.5716553	19682.36763	-182.3777008
3.929703576	94.95127816	510.1281738	9015.745223	-191.0453506
4.525120097	143.6045664	767.0403442	20622.27148	-229.3840607
3.878287944	94.82711515	723.3319397	8992.181768	-124.4189682
4.237034977	118.3831542	661.4212646	14014.5712	-218.7850067
4.529587061	148.5924945	714.4958496	22079.72943	-215.1304413
4.263839243	118.7820262	744.8913879	14109.16976	-218.1535889
4.098042444	106.8113432	655.8165588	11408.66303	-172.5433014
4.322164353	124.713558	833.8261719	15553.47156	-134.6975357
4.541732606	144.1480572	773.8645935	20778.66238	-201.353067
4.315792754	132.4629805	558.6170044	17546.44121	-127.9501518
4.395225127	136.3715262	690.1474609	18597.19316	-231.37556
4.491525085	143.2432115	741.714325	20518.61763	-228.9872925
4.395523983	135.4501704	819.0727234	18346.74866	-193.7129822
4.156508033	111.5893979	601.1567383	12452.19373	-162.5396454
4.251155537	125.658601	761.4742432	15790.08399	-179.1667328
4.465726074	139.5697509	737.7542114	19479.71537	-246.6485168
4.248067981	117.5051415	903.2291565	13807.45828	-174.7649506
4.687528139	164.0966375	815.3020325	26927.70644	-270.6126526
4.46134998	141.2833545	692.1289978	19960.98626	-250.096344
4.718840258	164.5517252	815.3273926	27077.27025	-252.8640228
4.402469711	131.3952723	695.7150574	17264.71757	-179.2101303
3.860074989	94.83275499	558.282959	8993.251418	-140.6105957
4.344147854	123.7894616	758.716217	15323.8308	-155.4407883
4.437557709	141.1162487	656.5070496	19913.79565	-286.9337891
4.227647843	121.3528891	567.7144623	14726.52369	-154.7792511
4.667002196	171.7395093	935.6020203	29494.45906	-257.3227844
4.245999682	117.6683331	740.4263916	13845.83662	-162.2624054
4.352481337	144.8172044	577.7608795	20972.02269	-310.7076599
4.434811507	133.7492296	876.3779297	17888.85643	-186.4681854
4.2048912	116.1162304	629.3973083	13482.97896	-157.060791
3.954730863	105.6079438	694.7013855	11153.03778	-141.342923
4.623912745	155.5864392	834.7651062	24207.14006	-269.4139404
4.615723978	152.4805466	828.8696289	23250.31709	-251.9302094
4.51480132	146.64576	951.7635193	21504.97894	-225.4432617
4.391341767	131.4364926	675.5225525	17275.5516	-234.3006577
4.164592118	111.6386921	533.1814728	12463.19758	-192.9453278
4.528530179	149.5596681	787.5770874	22368.09433	-220.254834
4.533142041	142.6198842	914.6059875	20340.43136	-178.836821
3.890023897	93.50874017	664.6025085	8743.884487	-110.1330795
3.914678366	96.33781278	637.2489624	9280.97417	-135.5299377
4.43343106	140.3285647	657.5000305	19692.10608	-246.3723267
4.386380546	131.5024913	774.7546997	17292.90522	-125.6309509
4.615931476	161.9603833	802.1579895	26231.16575	-267.5665161
4.610795188	155.0172555	777.2289124	24030.34951	-214.3526154
4.534954188	156.9435107	707.4147644	24631.26556	-335.4229431
4.393170557	131.685333	695.6080933	17341.02693	-176.6282959
4.48346246	141.2327859	798.3909607	19946.69983	-192.1226944
4.63752636	157.3235746	745.0237427	24750.70711	-262.5847046
4.178591849	113.2472465	719.269928	12824.93885	-159.3618561
4.045960572	102.63335	747.6826172	10533.60454	-160.6045227
4.063570931	109.1537119	761.6888428	11914.53282	-115.970343
4.704809853	163.8622929	964.1903687	26850.85103	-278.9832306
4.384183495	132.3403647	766.5775452	17513.97212	-251.973999
4.642276355	159.6164073	703.0040588	25477.39748	-283.4777191
4.479238745	140.7609298	730.6523438	19813.63935	-263.8491241
4.258379298	118.334412	773.2175598	14003.03306	-191.6370316
4.836213731	184.5612844	861.7502441	34062.86771	-404.3270477
4.507611488	144.5491752	826.1930542	20894.46404	-234.0008545
4.449788577	136.3657137	871.2085266	18595.60786	-151.6602173
3.892811944	98.1007727	498.8866577	9623.761603	-113.5104858
4.243886295	118.5703372	695.4926453	14058.92487	-140.9528656
4.039198217	103.9466484	684.3379517	10804.90572	-124.848587
4.642750134	160.2706613	784.9345398	25686.68487	-264.8855591
4.745692483	174.7561258	941.3422852	30539.70352	-320.049704
4.049659896	115.0822176	521.860733	13243.9168	-143.6035614
4.038552745	120.8672201	565.1932983	14608.8849	-191.3670166
4.407486633	131.8805306	867.7072449	17392.47436	-177.8101288
4.597527917	157.9614789	788.5372925	24951.82882	-297.3891144
4.018803435	102.1452105	509.9826202	10433.64403	-180.6438431
4.189251948	112.7948386	721.3747559	12722.67561	-169.0946747
4.136039596	110.603576	633.28125	12233.15103	-157.5390244
4.830415307	204.2194587	869.3791504	41705.58733	-385.3869141
4.433832339	134.9179371	733.7594299	18202.84976	-168.5686874
4.138506142	119.6851022	479.8113556	14324.5237	-147.1790161
4.136966886	112.5071362	574.7659302	12657.85569	-147.0326019
4.595585587	152.6280874	877.6687622	23295.33305	-259.8250061

log.sigma.4.5.mm.3D_firstorder_Kurtosis	log.sigma.4.5.mm.3D_firstorder_Mean	log.sigma.4.5.mm.3D_glrlm_ShortRunLowGrayLevelEmphasis	log.sigma.4.5.mm.3D_glrlm_GrayLevelVariance
2.720991646	-29.28037692	0.009428952	42.1759489
3.427883571	-23.87996932	0.013761986	23.22889771
2.345341264	-77.45321621	0.019268076	20.75715864
2.614969933	19.17876081	0.004058361	30.66640683
2.426608463	-16.60884724	0.008748677	46.08682548
2.306546843	-92.34582809	0.02083457	22.60231866
2.638077743	14.50267551	0.031343959	15.38389693
2.393316184	-29.04803509	0.009458121	49.85355876
2.663185117	-32.97393326	0.006159915	41.36116585
2.959819764	-35.76969827	0.00731695	22.74057803
2.243119846	-60.68215565	0.012435493	27.40270676
2.762117363	-34.6088712	0.013658662	30.32036676
2.608384791	4.036607988	0.017998442	27.92021751
2.344008397	-5.819624932	0.008797992	27.84624832
3.040417645	32.2241502	0.011108838	27.50709125
5.292382277	7.651279512	0.00572983	14.50501866
2.568993095	-64.19326464	0.019232185	33.95090052
2.682386412	-16.80202652	0.019820072	35.94144228
2.802417594	10.01877193	0.008614869	44.39533522
3.249860199	-17.6540376	0.014772136	17.25831775
3.395039702	-51.16432724	0.007190975	14.33084565
3.004770399	-0.312136818	0.01505954	27.10743697
3.275519879	-30.29154682	0.00794666	22.24895813
2.43351672	17.89956256	0.012836847	31.69702275
2.702618448	-68.76265683	0.034353119	14.60381496
2.648215927	-51.2181283	0.012016611	33.44430548
4.049033554	-13.95285125	0.007056318	16.19570673
2.755755989	-69.07223771	0.021275745	22.56418806
2.063324206	-9.069147486	0.008209657	35.24879232
3.22676766	-66.16581938	0.010444044	23.96368424
3.160826847	-47.09379141	0.011924975	18.63296144
2.973867089	16.16573901	0.008929419	25.75858696
2.524961767	-9.743425449	0.011042628	33.70363011
2.111824125	44.25920578	0.025524322	28.15193482
2.848221469	-38.89422704	0.009668305	29.93120881
2.183949943	-29.77743028	0.009546691	32.92829953
2.813751236	-32.20829385	0.013285172	29.71489712
2.509111276	-4.839844284	0.01144103	20.16089349
3.629320366	2.559483337	0.010522783	26.09331877
2.686710936	-40.93964985	0.014530771	31.68437328
3.35734785	-27.23372812	0.006652186	23.02238316
2.355849736	-34.45169127	0.012212738	43.12192138
2.167963993	-46.65365937	0.012082751	31.96580142
2.527902411	-19.88458847	0.010703899	43.61416423
2.659884658	-12.39769451	0.010572538	28.24233628
2.923366359	-32.75881921	0.018158447	14.88061298
2.741481767	6.966181162	0.00760832	25.06928793
2.230838469	-88.20855747	0.025958846	31.56155711
2.744705258	5.035125679	0.023882155	24.70766915
3.426876796	3.208849273	0.012924533	47.97024472
2.908459815	-13.02454516	0.010246539	22.88197716
1.795561048	-99.46344401	0.015111358	33.26356961
3.095711695	-16.47563645	0.008210506	29.67179973
2.87954768	-0.75408652	0.01603568	21.89335016
3.849958489	-4.160212612	0.009998427	19.2663929
2.537771013	-52.46626891	0.01106433	39.15552041
2.280900481	-47.53902601	0.005663504	37.32338906
3.27918248	-19.70030572	0.00449723	35.07805675
2.637391988	-64.00086649	0.018234931	27.86688895
2.37578527	-33.68045907	0.015882978	20.13986243
2.331815309	-36.4195963	0.014799887	35.77691806
3.08429415	4.182884121	0.007808101	33.10281367
3.991794896	12.09404651	0.013453269	14.87320157
3.492594116	-22.23828896	0.006859107	16.19544484
2.5302481	-56.91262952	0.020550941	31.95087073
3.022356287	33.58705856	0.01076266	28.489004
2.894904988	-39.30389317	0.014104541	42.4889112
2.510995696	-3.821831985	0.011753139	38.870997
2.463140904	-101.8908129	0.018566569	39.2835888
2.699720931	-15.79206829	0.020095957	28.04333635
2.495697551	-19.98598656	0.012225794	32.2692255
2.256901291	-49.12992984	0.013796741	39.51915986
3.200740865	-16.81931078	0.01225752	21.38623798
3.017887536	-30.95008176	0.006509894	17.57494393
4.117914329	16.0296668	0.00868009	20.52867289
2.623058436	-54.58052304	0.006934299	43.36256903
2.427909339	-72.03006689	0.011307899	27.97076926
2.303001363	-68.35068481	0.022117037	40.94074398
2.226864038	-86.31158297	0.01130647	31.89329296
2.629215125	-45.98959188	0.006482024	22.61010043
2.360827497	-143.1999615	0.011632955	54.60562157
2.242515967	-33.02251117	0.008723966	33.44490768
2.933869885	23.21100587	0.004896207	30.34455805
2.930481315	20.51146191	0.01872434	15.77876443
2.713346622	4.455987204	0.014672908	22.84210018
3.309683236	-5.983937403	0.007802133	18.14672422
2.358896467	-51.04588258	0.01998777	41.21217917
2.513155293	-57.61549371	0.003672567	48.90686343
2.23849354	-14.3182312	0.013481502	21.69621553
2.895890669	-32.66961994	0.027362589	23.74880624
3.265023825	-10.40108617	0.005350134	29.46780357
2.468631341	-92.51842029	0.038417174	40.20429775
2.597105404	-47.54120622	0.025131239	17.16770788
3.11068362	-29.47363435	0.008788562	21.04869153
2.77055215	-11.18692514	0.016770005	20.20416249
2.146769623	-86.97537601	0.012559117	66.47487413
2.698472233	5.536916395	0.007039035	29.78191259
2.154098061	15.02306735	0.049880942	22.34170065
2.849362084	18.56487998	0.020088767	20.59310448
3.180624458	-54.93348541	0.011189766	38.3982976

log.sigma.4.5.mm.3D_glrlm_LowGrayLevelRunEmphasis	log.sigma.4.5.mm.3D_glrlm_GrayLevelNonUniformityNormalized	log.sigma.4.5.mm.3D_glrlm_RunVariance	log.sigma.4.5.mm.3D_glrlm_GrayLevelNonUniformity
0.009826008	0.04426841	0.069649838	29.43539418
0.014921631	0.061993972	0.205298261	113.0847618
0.020552546	0.061325055	0.088358306	24.73772997
0.004258634	0.049970845	0.106524204	121.450982
0.009062357	0.043515201	0.076235892	14.6876749
0.022191661	0.059961759	0.099331746	35.73441055
0.034273617	0.076510621	0.131619483	23.22181481
0.009858274	0.040050719	0.065538478	37.96430143
0.006493635	0.043498319	0.086154057	84.33337374
0.007909777	0.060479042	0.131545684	169.4621343
0.012984355	0.055833083	0.087546658	43.14408852
0.014678049	0.051214746	0.104876991	69.81573731
0.019321631	0.053753215	0.138229469	101.118229
0.009293609	0.053321624	0.094217032	55.38986317
0.011735345	0.05524948	0.10555919	46.45920725
0.006545906	0.098349132	0.603460711	536.7016747
0.020665533	0.049629033	0.070460997	32.5936196
0.021555021	0.047186754	0.112873994	101.3440869
0.008981291	0.046349186	0.073659493	25.37634139
0.01611137	0.070606099	0.224084853	173.6067037
0.007947773	0.07674999	0.231830706	346.2482806
0.016243315	0.054553062	0.111111762	86.40935898
0.008544965	0.063389537	0.119393932	157.951208
0.013609714	0.050034599	0.08658751	22.35959024
0.037410048	0.07352882	0.133290109	80.22917141
0.012665785	0.048791039	0.076393521	31.70647249
0.007874123	0.078607978	0.412059897	224.0258933
0.02317014	0.059790732	0.113283331	78.59759401
0.008541874	0.046815177	0.071290955	78.04976606
0.011321238	0.059741329	0.229772142	132.1569795
0.012980708	0.067328492	0.139322007	193.8008778
0.009753962	0.057130502	0.176258208	302.8334773
0.011546036	0.047892214	0.101130142	90.1772082
0.026029661	0.055111684	0.04150602	5.511593312
0.010029597	0.05492802	0.067119602	17.13360779
0.009990074	0.048677525	0.094032117	91.52299376
0.014376249	0.053180918	0.119286671	140.2912737
0.012457922	0.062438769	0.169817296	186.4502945
0.011097391	0.064423508	0.151490705	48.44196134
0.015338383	0.051384669	0.093896077	50.18472567
0.007156713	0.061286354	0.151601883	80.70184107
0.013086656	0.043500862	0.070857488	39.13812591
0.012624996	0.04971237	0.082173297	76.52146585
0.011220718	0.043002017	0.075638647	41.40614274
0.011160085	0.053081302	0.118711678	91.31553535
0.020456396	0.077636	0.185667752	152.1830323
0.008117258	0.055569599	0.125698565	215.7852771
0.027857304	0.050744748	0.076178066	27.8052214
0.024826056	0.061019864	0.064139383	7.676643628
0.013845753	0.049924685	0.067027542	23.4087005
0.011052387	0.059330704	0.162804911	182.4034371
0.01579221	0.051941379	0.078726719	40.38814676
0.008719416	0.052571615	0.135413355	182.8004904
0.017168958	0.062461337	0.109025519	33.83678024
0.011081833	0.080101982	0.313320364	445.5790308
0.011480329	0.045533545	0.069857992	37.77249201
0.005936281	0.04455486	0.074080626	91.88053233
0.004669611	0.053474087	0.094862048	64.17003839
0.019713975	0.054120215	0.10611095	40.46554632
0.016925286	0.061754375	0.113685211	50.26317052
0.015774352	0.047404772	0.094453094	93.40180346
0.008273308	0.0495276	0.124391337	247.8942565
0.014591702	0.079768513	0.223696739	216.0570055
0.007588283	0.07657857	0.294063086	177.4801253
0.021433476	0.051878618	0.056394604	10.663399
0.01129901	0.054774149	0.11298285	78.15263557
0.014856114	0.048124698	0.075990523	17.90276765
0.012519654	0.045159235	0.104601441	133.6706897
0.019420088	0.049871541	0.059880125	21.1321112
0.021458717	0.053270839	0.095394948	99.21160832
0.013103149	0.049839606	0.113615831	139.198222
0.014567126	0.043888285	0.073842734	46.61224487
0.013279148	0.06304491	0.179376121	291.0513272
0.007197888	0.068143443	0.235534061	398.6977506
0.009440824	0.072742743	0.256973622	575.1303859
0.007203471	0.043284515	0.074574155	45.53225819
0.011971782	0.052595196	0.096159091	54.63430527
0.023128	0.043738409	0.060646575	25.31494454
0.01200201	0.04875617	0.09090417	80.00842431
0.006950026	0.058699665	0.103615844	153.5097112
0.012114712	0.038788843	0.054897037	18.67316194
0.009053488	0.047947683	0.068369328	60.5506793
0.005170282	0.052838874	0.117007825	135.3414197
0.019202051	0.081344977	0.070182089	11.04592009
0.01570849	0.058948966	0.125499128	183.019894
0.008566957	0.070804902	0.214941953	338.1814339
0.021665208	0.043649834	0.072993206	31.95763426
0.003799153	0.042192452	0.063310185	62.11465714
0.013889377	0.065753516	0.090513045	11.1421544
0.028659272	0.073286888	0.07225818	7.232779683
0.005734857	0.054880271	0.197064672	194.268014
0.042653335	0.044562289	0.067176509	29.74667674
0.027445392	0.067583627	0.175549046	61.18585422
0.009429091	0.062640898	0.164842079	229.9167681
0.018283706	0.063377641	0.208506775	324.6024443
0.012844964	0.040010994	0.045643543	9.732452878
0.007348102	0.052144095	0.112285911	58.30791531
0.053669374	0.061761008	0.054665154	7.219125002
0.021791241	0.064633566	0.108250893	22.36336639
0.011902648	0.04757138	0.118401553	35.10232612

log.sigma.4.5.mm.3D_glrlm_LongRunEmphasis	log.sigma.4.5.mm.3D_glrlm_ShortRunHighGrayLevelEmphasis	log.sigma.4.5.mm.3D_glrlm_RunLengthNonUniformity	log.sigma.4.5.mm.3D_glrlm_ShortRunEmphasis
1.198210173	300.0701931	593.928254	0.956671861
1.506169681	168.8453844	1465.571155	0.914838645
1.252563386	147.3807611	352.1462881	0.946321006
1.285525214	378.5470919	2101.117189	0.943443397
1.211184139	383.680433	301.2986073	0.955855342
1.26552705	155.470529	519.336589	0.946974896
1.375133135	93.37408939	250.4639587	0.923755461
1.183590205	339.5850064	854.6691767	0.960345793
1.237273168	357.6872807	1703.680613	0.950452979
1.356068651	211.7115741	2336.301044	0.929880863
1.237376252	214.4563831	683.5712607	0.951945396
1.291264167	195.0119625	1169.20575	0.940097692
1.358427349	154.6475306	1588.07395	0.933793192
1.254464252	248.1833566	908.7696391	0.948485564
1.283880837	209.1603636	727.9654637	0.943655392
2.259115871	204.279461	3771.735033	0.854705019
1.204975183	248.5646341	585.7539031	0.954985342
1.294199489	195.9655825	1858.710962	0.943948483
1.205456382	422.4027495	488.3576519	0.955999961
1.52973343	126.0488669	1984.908279	0.916120298
1.579910846	180.9726085	3510.991338	0.902333612
1.296558908	176.1683111	1365.327907	0.941923099
1.324074203	216.7511453	2108.380166	0.93517268
1.247682647	276.6025948	389.2082516	0.946498578
1.355346728	88.04358732	915.4368091	0.931584642
1.218465925	236.5495085	574.4165894	0.952448937
1.902999728	190.5951942	2097.225992	0.88023067
1.306149523	134.6263286	1125.421325	0.939468359
1.19550003	283.4699335	1502.501439	0.959340125
1.556363397	197.0850993	1753.487337	0.909477321
1.364631351	148.5818407	2413.034227	0.931242404
1.433490032	214.809256	4380.589213	0.925536701
1.269012943	249.0692013	1641.623683	0.946909856
1.125123535	174.7197656	92.69938555	0.970490945
1.202185988	301.4537389	276.4712818	0.953359246
1.250297059	254.1778857	1652.817687	0.950176391
1.315436462	173.7620232	2256.46077	0.93920475
1.436994451	158.8267989	2440.259849	0.921192772
1.395469076	278.2658882	627.7644355	0.928281197
1.249930695	215.558352	857.7156112	0.949919464
1.409807508	244.7969414	1074.932827	0.921128766
1.193781841	293.6165996	808.9740515	0.959149675
1.223346975	235.6252896	1370.982392	0.954754363
1.209655868	309.3611963	857.5155304	0.955565146
1.315579535	213.4941428	1471.437271	0.939082936
1.48650383	96.95374093	1568.148787	0.912140152
1.334993434	229.423515	3273.814915	0.934205202
1.210893853	193.9041562	489.0237458	0.955791876
1.200369948	206.5344478	111.1709856	0.951970263
1.191934078	452.7017873	419.7776945	0.957587544
1.406712478	181.228483	2553.928353	0.927912816
1.212594029	207.7963478	696.4285986	0.957056411
1.347557031	238.2957149	2947.711556	0.935682117
1.301385896	193.3932294	461.0615856	0.938017753
1.705918465	158.61404	4304.499825	0.899309598
1.195502856	297.2559155	744.850127	0.958261314
1.207802114	336.3030418	1840.058298	0.955710699
1.260160868	437.8003237	1046.871782	0.946852053
1.294510458	189.073823	639.89659	0.939679081
1.293561569	160.4719323	709.4982983	0.945352525
1.252157449	201.5818709	1729.381934	0.949809335
1.326008816	262.7116979	4261.951563	0.937567508
1.545275354	126.0909471	2173.510872	0.912254514
1.694100515	184.0603835	1772.933786	0.89459133
1.172267626	227.6947552	184.6714345	0.959049143
1.303299378	209.7663458	1223.461375	0.940257887
1.201663858	352.0680969	335.4779797	0.959618543
1.273138324	235.896506	2581.344258	0.94705169
1.169244897	268.646952	384.6330216	0.962880039
1.263617668	163.6955127	1617.700621	0.945540091
1.302344718	213.1510437	2403.696043	0.941263474
1.205307313	244.4858172	948.3891299	0.956393681
1.43951972	153.2387937	3813.468004	0.925279862
1.559420496	207.0735474	4662.65125	0.91136688
1.614885169	176.4374554	6159.635818	0.902548246
1.202854649	365.7834628	944.476793	0.958103228
1.255306852	206.1749664	912.1410199	0.949506856
1.172663687	224.6063183	524.3871944	0.96200133
1.25012254	222.4657663	1437.867071	0.948383822
1.290479159	255.8887246	2235.545403	0.939393077
1.153279218	364.1724031	442.3095739	0.967012202
1.197707573	282.2334838	1126.756427	0.956069683
1.309275335	315.9865381	2197.907872	0.940359121
1.214247204	159.7707453	119.4518165	0.950040762
1.322157921	153.1924234	2663.153574	0.940101743
1.535911151	181.5366308	3783.944616	0.90897367
1.20448429	217.3419955	653.9398352	0.956212169
1.175954026	566.2165042	1334.528146	0.96230073
1.263542542	204.7719762	146.7202086	0.943072812
1.210981013	146.4527928	87.60186019	0.953526955
1.465407816	301.0137223	2915.367074	0.924267701
1.187422512	178.5515957	601.7824692	0.959982357
1.455956272	101.5257127	732.1129987	0.917069425
1.412389763	186.1798556	3045.008351	0.927143068
1.498598471	133.2021012	4155.612067	0.918542305
1.12824929	453.7960491	225.7783828	0.971554541
1.297251824	291.2420879	961.3716213	0.941636928
1.163865788	121.6265726	106.046034	0.962114556
1.313354137	181.8117169	292.2711553	0.933789961
1.318844485	263.2576942	627.6422262	0.936985892

log.sigma.4.5.mm.3D_glrlm_LongRunHighGrayLevelEmphasis	log.sigma.4.5.mm.3D_glrlm_RunPercentage	log.sigma.4.5.mm.3D_glrlm_LongRunLowGrayLevelEmphasis	log.sigma.4.5.mm.3D_glrlm_RunEntropy
363.0458531	0.941817389	0.011555064	4.977926844
262.345175	0.878041554	0.021047231	4.820832284
182.15475	0.929280397	0.02620094	4.496241074
516.2366126	0.922288717	0.005242491	4.897064511
483.6531171	0.940004285	0.010411117	4.941783501
203.1542641	0.926785501	0.028358807	4.564272672
129.7056718	0.900251084	0.047551273	4.35216099
410.2887812	0.946031015	0.011609545	5.081032728
452.9265085	0.932511933	0.00808714	5.061059898
287.9369988	0.904404827	0.010888856	4.731510074
280.6534429	0.934424705	0.015474059	4.653872571
252.9207881	0.919755009	0.019282427	4.861949265
220.3917694	0.907091462	0.026138181	4.824335872
327.4045564	0.929062887	0.011545607	4.741017318
273.0637759	0.922992485	0.01481555	4.768059859
505.8611184	0.783415088	0.013126574	4.695307869
306.4618495	0.940819925	0.027978656	4.811320321
248.6846169	0.920932841	0.030175303	4.962920598
526.9656684	0.940655564	0.010535858	4.933117814
196.1174729	0.877406421	0.02434764	4.607716103
297.8941583	0.863411474	0.012241301	4.580660994
231.5184237	0.920751342	0.021787966	4.778206933
289.9471549	0.912289102	0.011406962	4.685680593
371.0473386	0.929154006	0.016813884	4.786767502
118.5627641	0.906273959	0.052888541	4.393645575
288.0271549	0.936377743	0.015489833	4.843839657
384.31958	0.821585512	0.013526607	4.723369426
172.100326	0.91733319	0.033034866	4.651391689
355.0787826	0.944563086	0.010064921	4.8267129
333.8626213	0.869999395	0.015873571	4.890773777
199.8059427	0.905316137	0.01840244	4.579064687
311.3332556	0.893708652	0.014227562	4.859604022
329.7681032	0.926141738	0.014377582	4.929141268
199.4269788	0.961538462	0.02807573	4.445501354
381.8625623	0.93952734	0.011517098	4.678573172
334.2178206	0.93073115	0.012063919	4.853824389
226.3468712	0.915811966	0.019801517	4.829241529
244.478256	0.890234393	0.017921179	4.69684614
423.8832143	0.900046062	0.013800637	4.73198048
275.546974	0.930923224	0.019164464	4.823855688
357.1485655	0.893121804	0.009697514	4.775940775
358.7666941	0.944143999	0.016895055	4.967373984
300.899991	0.937365314	0.015236195	4.790844919
387.4179507	0.939437148	0.013496127	5.025684234
291.2475168	0.915867945	0.014043833	4.834228673
136.8316501	0.87960917	0.032960795	4.444116902
320.5473027	0.910202315	0.010606875	4.813316607
240.2337947	0.939833751	0.036631429	4.725551367
251.0260386	0.939150402	0.028637771	4.477511053
564.5299387	0.943352422	0.017570609	4.954704889
261.776989	0.89780284	0.015186734	4.760342831
262.0865098	0.94019161	0.019124536	4.652971336
327.3727158	0.909810158	0.011325502	4.90545167
261.8843236	0.916569049	0.022031885	4.609545146
279.9172607	0.851024187	0.018014535	4.694803077
363.4550203	0.943642251	0.013333891	4.918875312
413.3167854	0.940369708	0.007199399	4.934623096
586.883381	0.92789577	0.005489626	4.893730493
243.8461069	0.918446418	0.0263037	4.789319941
214.9410531	0.92403737	0.022434054	4.526102733
248.1232897	0.929792718	0.020505372	4.898145447
364.3102781	0.913584408	0.010574849	4.985302085
201.9018006	0.875354742	0.021314188	4.50937387
332.9163719	0.84821001	0.012095579	4.632471816
271.2161984	0.94718185	0.025014965	4.661301261
280.6779814	0.918420922	0.013888524	4.812648974
432.9644524	0.9439672	0.017979693	4.881768022
303.740467	0.925819879	0.016261649	4.999650006
322.2455009	0.949982753	0.022904923	4.778753027
207.7705505	0.926060513	0.027574407	4.78395616
275.8338108	0.918775304	0.017415514	4.901848081
297.1055488	0.940723581	0.018033979	4.941088098
223.0199674	0.892965587	0.01907443	4.72111971
333.3277596	0.871031292	0.011338383	4.666749044
302.1062256	0.860566449	0.014027511	4.76624092
452.19048	0.942514475	0.008426107	5.001335074
262.7827169	0.929963501	0.015219226	4.736840314
266.4334216	0.948928121	0.028270775	4.892022988
280.2881289	0.930315716	0.01514889	4.839172929
339.0033386	0.918941564	0.009100713	4.682452303
422.6931175	0.955128205	0.014238856	5.044610833
353.6211979	0.941719727	0.010499751	4.814233994
433.604171	0.917318713	0.006569542	4.884771086
203.3997902	0.936339523	0.021193741	4.182461564
204.3914837	0.91556836	0.020937596	4.679548853
285.9859432	0.872127325	0.01296451	4.675558365
261.4083808	0.941071782	0.029095195	4.935865992
689.9932079	0.948503172	0.004361434	5.021660182
270.3667161	0.925598991	0.015716633	4.377854912
177.0534591	0.939194139	0.034055268	4.274180661
459.6878579	0.889927431	0.007913913	4.967041941
205.212366	0.9455219	0.061967224	4.863932
154.6692186	0.885744393	0.038869393	4.560901973
275.8859157	0.897197668	0.012846964	4.712997917
208.379775	0.881978855	0.026597337	4.716893509
532.4913333	0.96138644	0.014016981	4.989021286
401.6006342	0.919534413	0.008843116	4.84342728
142.1146093	0.950594121	0.069000896	4.304691091
258.2828485	0.91292876	0.028876918	4.521191299
344.1152179	0.914212182	0.015373375	5.012764202

log.sigma.4.5.mm.3D_glrlm_HighGrayLevelRunEmphasis	log.sigma.4.5.mm.3D_glrlm_RunLengthNonUniformityNormalized	log.sigma.4.5.mm.3D_glszm_GrayLevelVariance
311.2306475	0.892702525	49.48568664
182.238025	0.802515295	30.64669328
153.5023841	0.870642749	22.27308258
401.3550376	0.863342421	36.75724799
400.6545088	0.891596821	49.71747919
163.1262825	0.870862305	23.7614142
99.57068948	0.822012172	15.31925154
352.0330954	0.901303466	52.89456467
374.2675476	0.878360969	47.70650447
224.4785257	0.833109846	27.65831831
225.1313697	0.882872669	28.92272237
204.8988066	0.85604431	35.11684381
164.9495051	0.842642665	31.45729599
261.2555037	0.874284467	30.02763325
219.85456	0.864117745	34.53278001
235.769709	0.689066535	28.22320492
258.8138798	0.890001351	34.50361473
204.6281761	0.86466308	35.75950245
440.6360794	0.891309106	46.05254809
135.7675934	0.805901963	22.2408824
197.8395313	0.776775546	20.48210829
185.1494837	0.860261344	29.8832815
229.2069751	0.844962958	27.23192094
293.0436854	0.8697254	33.56484429
92.9965655	0.837437002	16.94691087
245.6582181	0.883183778	38.18004358
213.2837754	0.734741776	28.99633758
140.9477894	0.85460014	22.26397917
295.6453793	0.899871051	34.35954858
215.5259091	0.791582192	32.17604885
156.89142	0.836780023	20.73304025
229.1635122	0.82503167	29.72862247
262.4724177	0.871086535	39.34982358
179.2201789	0.925906431	29.94101509
316.2723358	0.885240008	33.62837126
267.1781986	0.878399849	33.96986936
182.3546548	0.854213271	32.15071669
171.9987634	0.815611666	22.55799232
300.2921511	0.832159692	36.965775
225.4709858	0.877594353	36.87357491
263.0500721	0.815000962	31.71674673
304.8527789	0.898611811	42.96186515
246.5207508	0.889485354	32.04348042
322.9458453	0.890156648	47.82419753
225.7657119	0.853948352	35.72845125
103.1275997	0.797462945	17.02093918
244.4789011	0.842355086	31.64729582
201.6010504	0.891391496	28.06894429
215.0169987	0.882139268	31.59533608
472.4349797	0.894784315	63.16040094
193.2849156	0.829723224	28.03584026
216.8139279	0.894636611	29.59382806
252.3961675	0.846586106	37.4080384
204.7354631	0.850472871	27.91239902
173.6296839	0.772560788	26.68883537
308.8493249	0.896946708	46.0068639
349.8370842	0.891079055	37.58874723
462.7149057	0.871080535	44.16286316
198.6193242	0.854780081	30.3649095
169.051881	0.868828807	20.78492879
209.4453567	0.877411947	35.70576291
279.2756955	0.850402564	36.76579066
136.7367591	0.799232372	23.0022959
203.2901951	0.762781689	24.28292896
236.0873644	0.897840291	35.15212411
221.4627329	0.856274874	37.89587454
365.091792	0.900678228	50.71022727
247.0025879	0.871390716	40.39340247
277.9340939	0.90726665	39.46607855
171.2389057	0.867729747	31.54818905
223.5920623	0.859010342	32.88151914
253.6145491	0.892311075	38.42665746
163.540915	0.824626087	27.27370442
224.2147275	0.795624563	20.52271328
193.9821608	0.777781413	32.84120678
380.3844855	0.896827722	47.88948521
215.5072584	0.877047746	30.84777208
232.1419531	0.905281411	39.46343804
232.1053736	0.874606933	31.0224959
270.3385176	0.853767451	24.52235245
374.127363	0.917806182	56.07994304
295.1234582	0.891414742	33.33228686
335.0762676	0.856805406	37.56704276
167.9611296	0.878092637	21.30102041
161.2110107	0.856439817	24.68895902
197.091151	0.790528521	26.31429544
225.3164507	0.892140353	39.22919316
587.6893656	0.906085151	47.91317724
216.0919385	0.863565927	27.09660782
151.8211735	0.886462635	28.99555556
323.1995739	0.822551553	39.23145302
183.3700697	0.900790283	37.4058948
109.7879756	0.807000084	19.94518841
199.4211673	0.828269849	29.03181509
143.5513782	0.810267141	24.17484979
467.3190981	0.927969792	64.78481452
309.1025893	0.858952923	39.91792549
125.4268437	0.905796464	20.58145065
194.987943	0.842621105	24.16493656
276.7597987	0.849224769	47.86096563

log.sigma.4.5.mm.3D_glszm_SmallAreaHighGrayLevelEmphasis	log.sigma.4.5.mm.3D_glszm_GrayLevelNonUniformityNormalized	log.sigma.4.5.mm.3D_glszm_SizeZoneNonUniformityNormalized
242.4496468	0.040644988	0.370857296
146.73106	0.050458492	0.304879737
117.3015786	0.059198967	0.320664061
216.1383144	0.045588585	0.289784648
266.2403411	0.041426873	0.323376932
99.31721636	0.060825943	0.22858252
77.50975896	0.076003086	0.249228395
237.6202606	0.038689391	0.3443044
254.2825378	0.040285553	0.316201996
175.5601446	0.052518743	0.32087286
104.5560163	0.052308125	0.250797523
142.0678376	0.048514305	0.272321031
103.3250177	0.048814241	0.243395044
162.4569801	0.05074286	0.293950168
151.5019627	0.048110752	0.300526092
163.9435859	0.052413756	0.295407662
168.8856088	0.048733284	0.291238497
151.987704	0.046085343	0.296987688
253.2588805	0.044880693	0.293707449
100.5955988	0.059353299	0.263581453
142.041087	0.063361545	0.266271701
125.3298479	0.052753741	0.279437834
169.1935894	0.055162583	0.279330223
151.1412707	0.04982699	0.26615917
79.32650857	0.069321574	0.320996672
195.8598609	0.04484352	0.339345726
151.4042625	0.052713646	0.275621196
117.0679965	0.059881353	0.313336988
176.7286982	0.046649176	0.352578294
160.657043	0.049305616	0.288069797
126.7701483	0.06266053	0.302252998
159.6937704	0.050833086	0.282465085
154.8981087	0.043815693	0.292591898
125.5543158	0.054183813	0.351165981
185.472179	0.051646103	0.275466551
156.7357726	0.047388484	0.295492862
148.4208776	0.049421908	0.298177316
95.26703676	0.05809232	0.247218784
157.4011196	0.0472	0.24155
162.7959192	0.04602358	0.343295582
175.5783607	0.051780009	0.258854511
205.7180682	0.042855677	0.34271864
143.1484533	0.048608809	0.297407617
203.1904826	0.041670477	0.319408627
145.3127766	0.04646849	0.296147444
87.99610434	0.067660199	0.292229158
157.5969447	0.048802273	0.276625541
139.3776513	0.053978147	0.332987073
179.9454345	0.055555556	0.331961591
290.4740562	0.037545339	0.352661504
132.5158037	0.052822641	0.249854742
122.0198872	0.052187322	0.31949968
176.6433405	0.045617598	0.30263049
123.3720534	0.053726566	0.245160799
135.2119263	0.054946032	0.288079334
219.5510701	0.041310285	0.332972778
248.9556136	0.045227284	0.351734159
260.2773221	0.044609418	0.309172299
152.4521519	0.051716798	0.289969319
91.06762065	0.061354486	0.231867075
166.1169001	0.046255537	0.329358436
161.2574277	0.046624436	0.291344435
97.3668871	0.05872152	0.269436513
135.9265502	0.056787405	0.240869766
150.7076446	0.052269888	0.294460641
157.5605311	0.045224368	0.31225715
274.0042111	0.040418388	0.362086777
172.2582309	0.043742025	0.305271309
208.7608714	0.046138766	0.399379215
127.9678683	0.049584016	0.303100672
164.694725	0.048177918	0.323475103
169.9085949	0.045028002	0.294976703
131.3614595	0.052904225	0.27759392
163.0123583	0.062175917	0.271078686
121.0447989	0.049367726	0.261049501
279.3634488	0.041381258	0.362421439
142.6908778	0.050643001	0.288141673
155.4026825	0.044685337	0.312013408
164.2720875	0.049465747	0.317951107
178.3588116	0.05639805	0.272630794
281.9674392	0.038234247	0.393919544
190.2099566	0.048261003	0.347159126
207.5468571	0.045734016	0.296932298
107.2934005	0.071428571	0.290816327
115.4228664	0.055724108	0.281591378
133.643855	0.053736293	0.269604247
168.5853836	0.044720396	0.336615895
386.9981238	0.041172849	0.363206647
149.7537364	0.059313628	0.385836144
108.8009397	0.065679012	0.372839506
216.7831575	0.04400091	0.306458855
168.6606276	0.046147227	0.389347054
64.56336261	0.062630104	0.244242653
133.5680936	0.051173987	0.270467507
105.0083558	0.05650539	0.287256047
301.6988568	0.037492561	0.411723864
157.4731942	0.044134405	0.283520818
89.1740398	0.067776457	0.343043995
79.61745266	0.064013841	0.20165321
258.1034798	0.042222126	0.417416898

log.sigma.4.5.mm.3D_glszm_SizeZoneNonUniformity	log.sigma.4.5.mm.3D_glszm_GrayLevelNonUniformity	log.sigma.4.5.mm.3D_glszm_LargeAreaEmphasis	log.sigma.4.5.mm.3D_glszm_ZoneVariance
113.1114754	12.39672131	13.94098361	8.582897071
157.622824	26.08704062	152.0909091	135.9513411
53.5508982	9.886227545	18.28742515	11.53365126
252.1126437	39.66206897	38.23103448	29.05779495
46.88965517	6.006896552	13.19310345	7.063210464
41.83060109	11.13114754	31.41530055	19.0694855
17.94444444	5.472222222	70.51388889	48.60628858
149.7724138	16.82988506	10.96551724	5.659643282
241.2621232	30.7378768	20.60419397	13.17981892
282.6889898	46.26901249	78.59477866	66.22929779
73.23287671	15.2739726	15.35958904	7.338278758
129.0801688	22.99578059	32.3164557	22.54093895
114.6390658	22.99150743	79.50318471	60.11327933
109.3494624	18.87634409	22.30645161	13.27416464
91.05940594	14.57755776	27.74587459	18.70622706
285.0683938	50.57927461	3396.353368	3344.528289
82.4204947	13.79151943	10.71378092	4.63049857
228.6805195	35.48571429	43.11428571	33.94204082
72.25203252	11.04065041	9.536585366	3.939321832
151.8229167	34.1875	209.0052083	185.3579252
255.6208333	60.82708333	514.6458333	485.0340972
144.1899225	27.22093023	34.30232558	23.19121447
214.8049415	42.42002601	90.9869961	78.37479983
45.24705882	8.470588235	19.66470588	11.65913495
106.8918919	23.08408408	73.05705706	59.98438078
93.65942029	12.37681159	15.94927536	9.626601554
155.4503546	29.73049645	843.9219858	806.1561667
141.6283186	27.06637168	40.04646018	29.9953158
274.3059126	36.29305913	11.98586118	6.83914493
184.94081	31.65420561	163.5015576	147.823905
265.3781321	55.01594533	102.215262	89.10558398
445.1649746	80.11294416	92.37817259	78.22033388
192.2328767	28.7869102	34.04718417	24.47206874
18.96296296	2.925925926	5.259259259	1.550068587
34.98425197	6.559055118	13.35433071	6.520429041
205.0720461	32.88760807	24.00864553	15.53668746
264.1851016	43.78781038	39.59142212	29.02525363
179.9752747	42.29120879	157.7335165	136.5077512
48.31	9.44	79.165	61.734375
136.9749373	18.36340852	26.01253133	19.10050816
93.96418733	18.79614325	105.9559229	89.46740129
139.4864865	17.44226044	11.73218673	6.249455173
184.095315	30.08885299	15.98061389	8.943979163
129.3604938	16.87654321	13.16790123	6.762627648
171.7655172	26.95172414	41.83793103	31.35373365
145.5301205	33.69477912	234.6144578	214.5987161
316.7362445	55.87860262	61.84104803	47.95972922
75.92105263	12.30701754	15.36403509	8.825696368
17.92592593	3	11.40740741	5.249657064
71.23762376	7.584158416	16.31188119	10.25833252
220.3718821	46.58956916	94.96598639	79.89539338
106.3933934	17.37837838	14.78978979	8.622099577
364.3671096	54.92358804	60.6038206	50.53215955
39.71604938	8.703703704	38.19135802	24.88237311
384.0097524	73.24306077	1280.864966	1256.918885
128.8604651	15.9870801	9.930232558	4.771347876
326.7610334	42.01614639	13.16146394	7.589018366
146.8568421	21.18947368	24.42736842	17.01751136
75.68199234	13.49808429	35.70881226	25.98206133
59.82170543	15.82945736	29.18217054	17.52179857
250.9711286	35.24671916	34.85958005	27.12650953
503.7345286	80.61364951	45.15211105	35.11395207
176.480916	38.46259542	236.8824427	214.6270963
119.7122736	28.22334004	471.0201207	440.8254274
28.85714286	5.12244898	7.704081633	2.801020408
166.7453184	24.14981273	29.17041199	20.71255383
63.72727273	7.113636364	9.75	4.738507231
343.4302222	49.20977778	29.976	21.90029906
82.67149758	9.550724638	9.256038647	4.613783285
209.7456647	34.31213873	29.56791908	21.12267993
339.0019084	50.49045802	34.73664122	26.32221316
131.8545861	20.12751678	13.50111857	7.121821339
341.7181154	65.12510154	139.754671	122.12968
377.0704529	86.48670022	285.6534867	262.3421533
488.1625668	92.31764706	1014.147594	990.04842
172.512605	19.69747899	13.6302521	8.133394534
111.7989691	19.64948454	21.92010309	13.63223377
84.86764706	12.15441176	8.727941176	3.698475346
204.1246106	31.75700935	26.72897196	19.17931697
232.8266979	48.16393443	37.04683841	25.94093095
104.3886792	10.13207547	5.833962264	2.216788893
197.8807018	27.50877193	11.84035088	6.305475531
266.0513393	40.97767857	46.89955357	37.18965242
16.28571429	4	12.48214286	5.777742347
272.2988625	53.885212	49.45294726	37.15584934
280.6580211	55.93948127	527.5254563	499.8544203
112.0930931	14.89189189	13.01501502	7.556547539
258.9663394	29.35624123	9.467040673	4.728939019
27.3943662	4.211267606	15.3943662	8.75104146
16.77777778	2.955555556	12.55555556	7.111111111
354.266436	50.8650519	186.8849481	175.0611066
130.0419162	15.41317365	9.676646707	5.208612715
60.32793522	15.46963563	86.93927126	69.81910866
268.0332997	50.71342079	139.691221	122.6579009
376.5926773	74.07856598	294.7719298	275.1587198
58.46478873	5.323943662	4.866197183	1.691777425
104.9027027	16.32972973	42.60540541	31.80441198
19.89655172	3.931034483	7.155172414	2.6578478
20.56862745	6.529411765	34.97058824	21.16426374
126.8947368	12.83552632	26.97039474	19.92346563

log.sigma.4.5.mm.3D_glszm_ZonePercentage	log.sigma.4.5.mm.3D_glszm_LargeAreaLowGrayLevelEmphasis	log.sigma.4.5.mm.3D_glszm_LargeAreaHighGrayLevelEmphasis	log.sigma.4.5.mm.3D_glszm_HighGrayLevelZoneEmphasis
0.432011331	0.115487304	3405.931148	360.2852459
0.248916707	1.268015003	24356.32302	235.6847195
0.384792627	0.4102674	1746.976048	188.1736527
0.330170778	0.123779245	14414.17701	397.1896552
0.403899721	0.075406598	5128.351724	422.6068966
0.284603421	0.734410672	4022.398907	191.2021858
0.213649852	1.904252395	4973.861111	136.625
0.434131737	0.104469129	3297.455172	380.2965517
0.367003367	0.118142486	6365.221494	417.7771953
0.284377017	0.663245226	11987.51192	282.569807
0.353083434	0.182604814	3337.890411	220.4417808
0.319838057	0.473525247	4762.409283	246.7763713
0.227097396	1.430470791	10595.32484	192.8174098
0.33273703	0.178017868	5641.336022	274.4623656
0.332601537	0.259724179	5044.663366	251.9735974
0.138908882	16.48230381	712991.9896	282.2704663
0.405444126	0.303318187	2440.416961	285.3286219
0.330188679	1.030246565	4574.136364	258.0792208
0.422680412	0.066293557	3971.325203	457.8455285
0.205640843	3.110021494	21981.24653	181.6753472
0.183767228	3.572370537	80438.1125	255.178125
0.3	0.601593024	4417.625969	222.1569767
0.281581838	0.672276731	14652.91678	285.4421326
0.353430353	0.152922201	6689.605882	284.6941176
0.276578073	2.269271617	4405.660661	123.6006006
0.397694524	0.176139404	2839.768116	296.3876812
0.162723601	4.909454763	152528.4433	273.608156
0.315422191	1.115441593	3429.820796	188.6283186
0.440793201	0.100633217	3333.380463	294.8624679
0.252557042	0.920264976	35960.53894	268.6744548
0.27618748	1.484905971	8995.43508	207.998861
0.265767285	0.754828789	15673.29378	278.4511421
0.323167732	0.249107337	8117.908676	278.1628615
0.519230769	0.102440625	861.9074074	195.1111111
0.38253012	0.083394416	4473.173228	321.2913386
0.343564356	0.197049233	6331.51585	278.0317003
0.307638889	0.658951426	4467.545147	238.0699774
0.217054264	1.73800584	24177.38874	189.4574176
0.239520958	0.360508233	25791.26	303.145
0.38036225	0.350192183	4957.531328	255.8245614
0.246268657	0.556767055	23942.39118	317.7272727
0.427072403	0.161478943	3294.936118	325.1523342
0.376979294	0.177412844	3841.279483	252.7027464
0.395121951	0.117519982	3984.437037	341.3753086
0.308839191	0.341092517	8397.063793	252.6810345
0.223518851	5.015208052	13174.76707	149.5481928
0.268401313	0.431726673	12657.11179	278.4253275
0.391080617	0.610548536	2372.333333	225.0526316
0.402985075	0.164157264	2228.740741	251.0740741
0.406438632	0.132292403	7812.975248	465.3415842
0.257593458	0.989490384	13730.30385	235.0759637
0.402660218	0.303470465	2636.345345	218.2642643
0.315100759	0.435261121	11480.75498	295.6187708
0.274111675	0.443262781	6744.820988	228.9567901
0.204353825	8.893188788	198954.7764	227.6774194
0.440273038	0.086657875	2755.979328	338.620155
0.423620611	0.080934797	3847.753498	387.5888052
0.367362722	0.066230296	12257.12211	457.0652632
0.320638821	0.506760553	5234.321839	250.5632184
0.292849035	0.579302119	3864.523256	186.7751938
0.359603587	0.692404707	3502.652231	263.1929134
0.315626141	0.273014226	10881.59861	296.1266628
0.21197411	2.394158576	28985.37557	169.2091603
0.181984621	2.779513006	86291.60362	262.7585513
0.451612903	0.129151236	1591.928571	261.0714286
0.343850612	0.224297589	5494.333333	253.6666667
0.446700508	0.136938538	3318.448864	402.2727273
0.351892399	0.361484433	5866.369778	288.9111111
0.464125561	0.224616585	2392.405797	299.6570048
0.344107409	0.59749616	3147.395954	208.0693642
0.344736842	0.412169837	5559.487595	267.2519084
0.395925598	0.220097704	2550.42953	290.6465324
0.238196594	1.638036487	18170.30707	221.4809098
0.207117332	1.865604224	53004.1294	287.8957584
0.203703704	6.25586397	170210.5337	223.9016043
0.426523297	0.077748287	4679.029412	419.5042017
0.347358997	0.270572837	3759.670103	241.4716495
0.445901639	0.206285137	1750.286765	255.8345588
0.363945578	0.32697324	3998.758567	266.7071651
0.300070274	0.260151258	7292.704918	317.1967213
0.525793651	0.077867444	1875.169811	409.8037736
0.425055928	0.092349383	3326.829825	307.0315789
0.320916905	0.182398324	14737.51563	355.1305804
0.386206897	0.133582479	2006.232143	181.1428571
0.285166618	0.752384845	5355.236815	198.7269907
0.190102264	4.082083389	74542.80211	245.1325648
0.428020566	0.401610089	1992.807808	261.0870871
0.459407216	0.029646799	5693.791024	600.85554
0.387978142	0.124776616	3180.802817	226.6619718
0.428571429	0.261034782	1423.888889	174.6
0.29081761	0.687859399	54753.93945	366.8226644
0.473087819	0.888225646	1007.113772	232.4011976
0.241682975	1.829023403	7537.234818	132.6315789
0.242298289	1.106380294	23235.71847	237.6316852
0.225800896	3.225514181	40960.43021	179.3302822
0.561264822	0.043880258	2507.985915	451.6549296
0.304276316	0.182741884	13173.02973	306.0486486
0.471544715	0.464885173	723.862069	140.0689655
0.269129288	0.419643673	7170.137255	188.6078431
0.376703841	0.266495168	5506.980263	353.0756579

log.sigma.4.5.mm.3D_glszm_SmallAreaEmphasis	log.sigma.4.5.mm.3D_glszm_LowGrayLevelZoneEmphasis	log.sigma.4.5.mm.3D_glszm_ZoneEntropy	log.sigma.4.5.mm.3D_glszm_SmallAreaLowGrayLevelEmphasis
0.628816083	0.010796824	6.320373547	0.007778896
0.569544663	0.016704965	6.545704324	0.009395785
0.576563012	0.017953922	5.83485117	0.007721276
0.548458305	0.005344633	6.797638755	0.003466091
0.586135081	0.012493845	6.107336376	0.009287529
0.477910522	0.018687434	6.115327642	0.005937353
0.506696341	0.029850429	5.223550481	0.00675573
0.605980274	0.010899241	6.595359707	0.007820484
0.579410158	0.006857163	6.803232891	0.004294068
0.581736901	0.007781941	6.506471393	0.004439961
0.501453376	0.017035824	6.433766815	0.011890616
0.528921647	0.014615699	6.6394866	0.007141488
0.505744238	0.020481206	6.797040626	0.009738668
0.559114477	0.010410422	6.455604947	0.006385079
0.562197346	0.01361315	6.387309673	0.008623761
0.559614388	0.007910559	6.69030964	0.003597361
0.550141953	0.013785287	6.316305881	0.004751655
0.556966244	0.013962014	6.677558112	0.005889069
0.547894916	0.009512995	6.336647928	0.004028368
0.522538692	0.014194961	6.541314851	0.007651108
0.527118484	0.008965947	6.592286432	0.005232837
0.542966046	0.016004152	6.603155421	0.008765573
0.539895377	0.007761427	6.630018598	0.003129519
0.507569893	0.014231063	6.03489239	0.00502537
0.584057315	0.030276992	5.941753063	0.010313841
0.597152349	0.012396554	6.205548937	0.005675516
0.539323661	0.010052415	6.645528068	0.005006789
0.574490529	0.015716988	6.163403028	0.006027165
0.612021258	0.009348873	6.388731663	0.006351114
0.546479639	0.01170623	6.615193938	0.005400307
0.566040202	0.01081613	6.416207697	0.005081348
0.543890756	0.007928758	6.864715613	0.003514313
0.553151234	0.012175948	6.75004477	0.005428627
0.588029835	0.034191282	5.180451391	0.027611979
0.530850198	0.014337146	5.977632498	0.010484405
0.558010079	0.011845435	6.680930718	0.00695824
0.562654457	0.011868397	6.696450562	0.005705588
0.505300526	0.01268492	6.712492729	0.006827921
0.498188138	0.015081473	6.488287377	0.00558962
0.602599346	0.014235668	6.299151406	0.007034331
0.512192193	0.009112933	6.541640137	0.005912239
0.604025204	0.010656946	6.425005482	0.004767256
0.55712234	0.013865087	6.543801832	0.007657986
0.582038753	0.011348783	6.553199302	0.004862339
0.558270647	0.0140031	6.622135068	0.008840402
0.554748428	0.016328118	6.20671712	0.009457835
0.541158733	0.00853289	6.891253384	0.004222404
0.59643517	0.014863111	6.017627571	0.005131808
0.592670449	0.028086916	5.018323845	0.008413923
0.61282047	0.013689598	6.305509414	0.006045104
0.508204363	0.011464815	6.864592253	0.006208478
0.58153049	0.017306849	6.185681676	0.012278227
0.563042781	0.009481083	6.839163778	0.005266864
0.503038839	0.017542398	6.179649367	0.00559397
0.548422958	0.009797868	6.671467164	0.004494358
0.593863237	0.012970519	6.494578429	0.00743609
0.611013786	0.005987914	6.540461011	0.00383154
0.571417834	0.006714191	6.668504392	0.005085372
0.550909597	0.018287435	6.247715457	0.006405488
0.483269442	0.015972815	6.303016354	0.006895093
0.589882407	0.013751816	6.543908184	0.00753921
0.55421081	0.00956211	6.9574063	0.005712853
0.532934341	0.017036514	6.646610235	0.009923666
0.487672978	0.008870762	6.549156647	0.0051932
0.534461986	0.021524476	5.683238288	0.007738967
0.573717464	0.014280445	6.602553244	0.008979252
0.621751787	0.013206464	6.150891586	0.005476509
0.564026446	0.010480685	6.783937967	0.004897786
0.655927189	0.019094765	5.963022599	0.014425095
0.565467705	0.020034088	6.576552595	0.009568841
0.586542403	0.011253592	6.604265185	0.004853513
0.546658529	0.012789947	6.552867347	0.005721984
0.538904375	0.011204155	6.784785752	0.005219351
0.531807089	0.006066267	6.666462951	0.002706923
0.520056362	0.012141868	7.038284155	0.006947819
0.623938289	0.008006251	6.511019697	0.005066875
0.54492478	0.013012268	6.456084158	0.007925863
0.566000559	0.020731536	6.273020148	0.009814149
0.579517885	0.010523785	6.46919015	0.004828866
0.533204058	0.006385108	6.629750892	0.002612573
0.648648392	0.012676115	6.261651948	0.009099264
0.608404276	0.011157886	6.378620288	0.008424299
0.559967719	0.006094426	6.811212391	0.003856346
0.547876994	0.032500559	5.265131208	0.027530636
0.547278179	0.014585741	6.712976717	0.006913647
0.530438942	0.009337902	6.776142118	0.004696861
0.594235161	0.015156806	6.282382518	0.005737749
0.623143898	0.004435266	6.540820748	0.003162892
0.642035603	0.023281045	5.138839549	0.020607253
0.625523125	0.040618712	4.924969207	0.034915598
0.568384487	0.006435914	6.849838894	0.003957361
0.644739561	0.021867382	6.052547145	0.007195819
0.494745935	0.027741737	6.202835481	0.012753764
0.531519654	0.010754031	6.800156906	0.006449043
0.550396916	0.01760774	6.692163971	0.008098599
0.661767719	0.01543858	5.911805528	0.011500438
0.541343799	0.010823132	6.602056752	0.007370409
0.597567954	0.036254455	5.038846943	0.008839447
0.430591773	0.02492044	5.850381504	0.007593935
0.671983712	0.011743229	6.177820592	0.006339203

log.sigma.4.5.mm.3D_ngtdm_Coarseness	log.sigma.4.5.mm.3D_ngtdm_Complexity	log.sigma.4.5.mm.3D_ngtdm_Strength	log.sigma.4.5.mm.3D_ngtdm_Busyness	log.sigma.4.5.mm.3D_ngtdm_Contrast
0.017593179	1.30558875	6.184437859	0.116670217	0.000227316
0.006740637	0.244446421	1.800170866	0.397371119	4.04E-05
0.025465341	0.775197616	4.014232415	0.190899813	0.000321675
0.004778007	0.336468707	1.751597379	0.314990379	3.87E-05
0.03373403	2.077977001	10.82273308	0.058996767	0.000572417
0.016122115	0.556168946	2.592825192	0.252482245	0.00025457
0.037520877	0.491617176	4.00778073	0.202344471	0.000342106
0.011228831	1.227899338	4.515138844	0.15902317	0.000199649
0.006047997	0.553237871	2.55288333	0.260743898	6.41E-05
0.004076506	0.20427178	1.320392827	0.551024734	2.48E-05
0.010684045	0.765141973	2.612198359	0.244454775	0.000207662
0.009151262	0.443146082	2.854990375	0.299882363	7.57E-05
0.006322061	0.193656689	1.387967475	0.710727435	6.51E-05
0.010438885	0.490901891	2.595260423	0.231413499	0.000119138
0.013822263	0.685884727	3.811692495	0.182630799	0.000124174
0.002301441	0.05921574	0.971690887	0.654243617	3.45E-06
0.01583761	1.01656093	4.427277548	0.146398224	0.000237456
0.005356177	0.312207393	1.686163419	0.62047553	6.53E-05
0.019387292	1.612648122	6.976511149	0.078600323	0.000326067
0.004623068	0.122647677	0.960713524	0.791914427	2.68E-05
0.002518413	0.093605951	0.84877525	0.800859597	7.52E-06
0.006712568	0.46130331	2.627069613	0.373222303	5.76E-05
0.004562352	0.226068854	1.532859323	0.469085816	2.71E-05
0.024961069	1.073325449	5.750794155	0.104099183	0.00035362
0.008962615	0.217111215	1.27507815	0.699315947	8.75E-05
0.016541587	1.179293609	4.568281238	0.159830478	0.000233499
0.004366494	0.118659004	1.446560154	0.440750267	1.06E-05
0.008302169	0.309462803	1.889647305	0.506468551	8.10E-05
0.005577601	0.454213576	1.644430019	0.406312522	0.000105611
0.005603691	0.228309665	1.711198741	0.37933332	2.93E-05
0.003605574	0.146116414	0.91528323	0.90893296	2.58E-05
0.002213875	0.123102922	0.881005784	0.931549099	1.27E-05
0.006308344	0.36574676	2.020163983	0.352885047	6.19E-05
0.077707007	4.463884866	13.08522536	0.060395336	0.002602191
0.029615335	1.755055038	7.331741119	0.077176904	0.000539563
0.005441691	0.38253686	1.720055408	0.406023865	7.10E-05
0.003959457	0.316493388	1.793233796	0.683009579	3.65E-05
0.003773747	0.103821298	0.758257986	0.907243251	2.83E-05
0.013688516	0.702392757	4.387680783	0.126257482	0.000116805
0.010960282	0.669898086	3.007442759	0.231345962	0.000141695
0.009689138	0.467019145	3.62169295	0.179797853	4.31E-05
0.011559308	1.00615436	3.992437925	0.182086472	0.000201429
0.006124207	0.431168879	1.665137035	0.399601078	0.000104047
0.011466298	0.960570163	4.050263703	0.172704919	0.000173578
0.006483808	0.30949878	1.670265074	0.427476837	6.63E-05
0.004847954	0.156168137	1.133236714	0.994321397	3.29E-05
0.003054342	0.149654236	0.931102522	0.708031785	2.06E-05
0.017235702	0.914839125	3.714111259	0.196639488	0.000361487
0.065335348	3.216216997	10.57903829	0.055335559	0.001617776
0.022231589	2.535982084	11.34449921	0.05128814	0.00031089
0.003798255	0.165557742	1.180710432	0.660701635	2.29E-05
0.009570931	0.659676221	1.994168659	0.369114443	0.000353703
0.003180019	0.257001512	1.355791238	0.583599099	2.42E-05
0.020561212	0.706414029	4.204370191	0.138815632	0.000182552
0.002197985	0.0698806	0.639625524	1.100506583	8.92E-06
0.013187103	1.080254284	4.392336155	0.14875923	0.000189638
0.004974079	0.454589186	1.647866135	0.394436731	7.60E-05
0.008551489	0.868804859	4.204566376	0.130245272	8.21E-05
0.015717743	0.616475951	3.822214962	0.201439971	0.000154361
0.012109281	0.378788932	1.998458117	0.331490076	0.0001523
0.005046235	0.39405393	1.678693532	0.625144423	7.94E-05
0.002358177	0.192686492	1.168777497	0.697032667	1.63E-05
0.004258539	0.128230394	1.163094772	0.675372185	1.63E-05
0.004924533	0.14413282	1.143011974	0.495814414	2.05E-05
0.04873983	2.497937818	9.738727269	0.067660723	0.001001496
0.008597979	0.455576656	2.874791594	0.276154546	6.19E-05
0.026545629	2.468701339	9.365784316	0.063125705	0.000481222
0.003674941	0.26689069	1.179917953	0.709921263	5.30E-05
0.021495516	1.664059601	5.957548936	0.107883123	0.000519038
0.006199403	0.270655726	1.512281531	0.608120522	6.72E-05
0.003841418	0.272058105	1.336309894	0.686862717	4.26E-05
0.009402275	0.748094163	2.84405313	0.288741798	0.000177531
0.002397428	0.108757466	0.712907677	1.199327911	1.58E-05
0.001986697	0.084822834	0.670521946	0.987837252	7.46E-06
0.001540182	0.057129901	0.558479374	1.38465992	5.81E-06
0.009727505	1.337856159	4.913496948	0.136772935	0.000127938
0.009704481	0.718724757	3.295523983	0.24103203	0.000106862
0.017303679	1.202739623	4.337505017	0.199194352	0.000410252
0.006006635	0.434652072	1.678101474	0.483070918	9.17E-05
0.004186723	0.233220039	1.342822608	0.487693001	2.83E-05
0.0194593	2.654918926	7.336869769	0.094620249	0.000544863
0.007990807	0.624202247	2.541716819	0.262356396	0.000116121
0.004951289	0.293039583	1.851621101	0.337731995	3.30E-05
0.057501569	2.124358348	8.590599588	0.064374306	0.000927639
0.003500212	0.160189494	0.930206992	0.972669551	3.00E-05
0.002429714	0.07792493	0.645725999	1.054176126	1.12E-05
0.014036922	1.095531395	4.371869898	0.224688491	0.000268891
0.006392555	0.944287428	3.171972387	0.177576372	0.000124309
0.054156456	1.442452163	7.713568412	0.08347163	0.001025324
0.069208469	4.153250604	13.84079637	0.059208373	0.001920241
0.003520264	0.227300404	1.440772221	0.41449849	1.94E-05
0.014708261	1.22772074	4.546551953	0.25516296	0.000326123
0.0131688	0.214380133	1.707767353	0.474362958	0.000103812
0.003004863	0.143073828	0.931987317	0.779814297	1.81E-05
0.002099872	0.071426215	0.482770654	1.694841302	1.58E-05
0.035399862	5.447014118	16.87019609	0.040790132	0.001268621
0.010993824	0.49419611	2.960010003	0.189672572	9.69E-05
0.065224781	2.695590516	7.745740702	0.099210139	0.002271709
0.032571673	0.758728928	5.610025149	0.104680606	0.000309124
0.01648202	1.198089297	7.127385015	0.111180246	0.000142796

log.sigma.4.0.mm.3D_gldm_GrayLevelVariance	log.sigma.4.0.mm.3D_gldm_HighGrayLevelEmphasis	log.sigma.4.0.mm.3D_gldm_GrayLevelNonUniformityNormalized	log.sigma.4.0.mm.3D_gldm_DependenceEntropy
38.14571981	307.4589235	0.047043151	6.566964565
22.30369345	209.033221	0.064789893	7.075428118
22.51753594	142.859447	0.061001508	6.183753383
31.25976992	366.8098672	0.049565008	6.893589476
45.6371071	370.6406685	0.044824295	6.454579643
21.34370624	173.1353033	0.060106567	6.327301191
14.97181449	109.2403561	0.078392871	6.178403169
48.23155685	352.1457086	0.040744858	6.680013515
41.26843552	371.3573834	0.043600299	6.978062171
21.99004377	223.3221433	0.062853187	6.874577485
25.68706108	203.6045949	0.058337927	6.318265692
30.12638937	207.7962213	0.05184572	6.873939607
26.4263225	163.1832208	0.056002637	6.819293636
27.85666088	207.1243292	0.053214435	6.522487321
25.84725775	185.3106476	0.056890957	6.540327963
12.50638782	202.1224989	0.118068908	7.130003343
34.6138558	269.3882521	0.048784493	6.487678649
34.37380788	200.7860206	0.048720318	6.95200096
45.36257543	365.7233677	0.043409974	6.657563989
16.29131509	114.372724	0.074174432	6.990606106
14.00816437	201.9090735	0.07943855	6.956013091
27.66871282	185.3755814	0.054019064	6.646049438
22.14331916	201.9055291	0.063905595	6.699073655
31.93813996	360.2182952	0.051940474	6.508383966
15.0043239	96.87375415	0.074047196	6.513707409
33.59639437	217.79683	0.049838467	6.525989458
14.5105476	240.3075591	0.091773053	7.059838757
22.64511668	145.2470342	0.060896844	6.690794083
36.63779791	271.9342776	0.046886822	6.692382512
22.53687646	223.8485445	0.063057394	7.192500126
18.45742185	156.6067946	0.068933918	6.692034073
24.22641045	221.2807757	0.060402617	7.10497004
32.98619797	236.2695524	0.048329228	6.86602231
33.16188979	246.4903846	0.057507396	5.281948916
33.71028996	365.4548193	0.052347946	6.156721701
32.19524434	272.9826733	0.049068719	6.78006942
29.52773329	182.1274306	0.053978347	6.859391514
19.62747042	149.2573047	0.063228022	6.920165267
25.12655169	273.3305389	0.066295672	6.585970958
30.80012286	228.6730219	0.052441792	6.601266407
22.23546815	263.9810041	0.062753029	6.913411804
41.54711966	305.1920252	0.044636202	6.57645911
31.44208475	252.7484775	0.050509539	6.586924273
42.35258441	292.0234146	0.043388459	6.759514311
26.89589366	282.3679446	0.054150916	6.737939987
14.14006814	100.8940754	0.080273023	6.655903963
25.21378978	243.1256446	0.055373345	6.856578924
30.77200498	189.4974271	0.053290927	6.44217235
29.39368456	299.1865672	0.062708844	5.637245743
46.99508115	474.0784708	0.052002154	6.455496957
21.9265212	191.2683995	0.061623832	6.948237911
33.08610397	203.8512696	0.054264405	6.363200004
28.73503342	250.3014918	0.053598548	7.049881497
21.0791025	177.3079526	0.064254855	6.371846393
18.06982653	171.1303081	0.086960516	7.097415707
39.53798983	287.3765643	0.044417783	6.563465107
40.41771054	358.7952576	0.04290961	6.786648807
34.47637556	431.3271462	0.054500735	6.729698886
27.47108042	206.4520885	0.053631474	6.587566185
18.77700374	147.1271283	0.063423697	6.395205886
34.91563888	206.3043889	0.049288879	6.736783339
32.42909892	278.9729828	0.050184275	7.19051337
14.09992103	157.6187702	0.084404436	6.863323356
15.00597008	202.8495057	0.082147941	7.035456974
31.2549003	268.5852535	0.052177791	5.95747451
28.92159371	249.787508	0.054946723	6.864250956
39.74467263	360.8274112	0.052204386	6.278998367
38.17219674	215.8526744	0.045524087	7.0462027
35.93824529	228.3139013	0.050554003	6.23118262
28.77095517	197.3187469	0.05319549	6.711034956
32.1140295	250.4049342	0.050735587	6.9857443
37.8249515	225.6775908	0.045554003	6.670346583
20.65581714	161.5106424	0.065230545	6.99478977
17.16439341	253.2048839	0.069800599	7.047529595
18.9988772	191.8125272	0.078724327	7.134489379
43.09970003	350.7311828	0.043482869	6.686562134
27.89051919	223.4395703	0.053207971	6.546223281
38.73787691	181.4032787	0.045267401	6.417424703
32.36859642	270.1995465	0.049088343	6.810938669
23.41393464	245.3988053	0.058097099	6.716285563
54.07015306	359.0714286	0.038044848	6.47530946
37.17948641	340.1886652	0.045371663	6.621191462
29.92180278	333.6697708	0.053033483	6.97054147
21.38387634	250.9586207	0.077384067	5.562552111
22.89995453	158.1751696	0.058866858	6.756736023
17.22956646	222.646092	0.074485596	7.032295242
39.84456883	226.8097686	0.0453407	6.468631446
48.4767705	510.9761598	0.042513318	6.660950231
19.6889725	192.9781421	0.071934068	5.780338741
25.12979592	186.0571429	0.068480726	5.34364168
28.23704721	288.5539623	0.058125739	7.151293025
40.76903956	188.8427762	0.04540603	6.477464751
17.61568775	138.0136986	0.068989089	6.859205617
20.4255463	176.6266504	0.063980129	6.976299114
19.22620028	142.1854978	0.065751822	6.979068068
72.30530082	452.3241107	0.03722914	6.148513364
28.58847426	314.6044408	0.053552686	6.747575945
28.6802829	190.9837398	0.055324212	5.452291083
20.96259425	195.7044855	0.065726359	6.030407786
36.21130167	343.8289963	0.049924069	6.859137469

log.sigma.4.0.mm.3D_gldm_DependenceNonUniformity	log.sigma.4.0.mm.3D_gldm_GrayLevelNonUniformity	log.sigma.4.0.mm.3D_gldm_SmallDependenceEmphasis
162.5212465	33.21246459	0.326285338
280.7640828	134.5686086	0.23097425
93.88479263	26.47465438	0.306310654
542.6675522	130.6037951	0.294362981
85.60167131	16.09192201	0.369285004
140.3685848	38.64852255	0.272734348
56.91691395	26.41839763	0.209901046
262.3572854	40.82634731	0.365887782
452.7431457	90.64502165	0.32967972
507.3111685	194.7191737	0.246600352
194.5066505	48.24546554	0.284828993
282.1079622	76.83535762	0.276454714
393.6422372	116.1494696	0.208112845
252.5760286	59.49373882	0.278216749
188.4796926	51.82766191	0.284029626
553.8351807	820.2247013	0.127666199
171.3323782	34.05157593	0.365662073
448.0308748	113.6157804	0.273563413
143.3367698	25.26460481	0.355454791
371.9689397	207.7625848	0.190744891
664.7936447	414.9869832	0.186070506
366.0093023	92.9127907	0.27423342
497.9022336	174.5261809	0.265526138
105.1122661	24.98336798	0.341222463
193.5431894	89.15282392	0.268236856
155.0662824	34.58789625	0.331179713
368.1534911	318.085401	0.161520859
244.1570133	87.26517795	0.277220349
416.2339943	82.75524079	0.359414118
331.945712	160.2918961	0.214201566
509.4397609	219.1409248	0.248956666
936.022597	358.1875211	0.236367847
428.559272	98.25332022	0.2842963
32.75	5.980769231	0.437032585
89.9939759	17.37951807	0.35790328
432.1168317	99.11881188	0.288327752
525.6736111	155.4576389	0.277484366
535.5802027	212.0667859	0.202680349
154.439521	55.35688623	0.230721115
227.7264061	55.01143947	0.326531732
227.5848033	92.49796472	0.21894345
235.1406086	42.5383001	0.338593862
385.7965895	82.93666261	0.3222999
236.3580488	44.47317073	0.337116609
389.1352503	101.6954207	0.277009039
298.0834829	178.8482944	0.202685829
817.0056259	236.222691	0.259890551
129.6895369	31.06861063	0.328289305
35.11940299	8.402985075	0.327844113
117.5875252	25.84507042	0.364456778
576.4591121	211	0.22872451
183.7811366	44.87666264	0.314849998
679.6637006	204.8000523	0.276770784
109.5820643	37.97461929	0.233927148
714.5600184	567.2434463	0.182533761
230.519909	39.04323094	0.379302199
528.1390789	94.10077519	0.364390228
277.6821346	70.46945089	0.31015844
159.9115479	43.65601966	0.289513522
180.5800227	55.87627696	0.230447468
434.38084	104.4431336	0.296920411
977.0543994	274.909456	0.279325263
404.3016181	260.8097087	0.192325047
302.0377151	224.3460271	0.16953112
62.29032258	11.32258065	0.394937877
292.7256922	85.33226014	0.290010708
93.73096447	20.56852792	0.401861914
628.6984673	145.5405067	0.303955687
115.5964126	22.5470852	0.3749623
419.9955246	106.9761313	0.305108611
573.7657895	154.2361842	0.289900942
260.8547387	51.43046944	0.321213349
757.5673375	337.1114551	0.22346899
893.6804646	468.7808219	0.189848691
1131.896732	722.6893246	0.195475279
276.6326165	48.52688172	0.36818396
245.6445837	59.43330349	0.291762351
155.7868852	27.61311475	0.374095814
365.0725624	86.59183673	0.304888276
542.4539705	165.3443429	0.267244822
147.515873	19.17460317	0.45538966
331.2878449	60.84340045	0.388574486
520.3459885	148.0694842	0.280068831
36.31034483	11.22068966	0.353832317
632.0728399	199.617517	0.249941381
749.47626	407.8831264	0.189963549
191.2262211	35.27506427	0.358309579
420.5554124	65.9806701	0.388088751
35.86338798	13.16393443	0.33131871
25.78095238	7.19047619	0.405690476
619.9444025	231.0498113	0.24814907
171.4645892	32.05665722	0.37276095
142.8336595	70.50684932	0.229107822
646.0841076	261.6787286	0.220570187
849.0310024	381.755081	0.207653202
82.34387352	9.418972332	0.412687747
239.3601974	65.12006579	0.274837935
33.22764228	6.804878049	0.42515131
85.78627968	24.91029024	0.229217358
148.6158612	40.28872367	0.320887019

log.sigma.4.0.mm.3D_gldm_DependenceNonUniformityNormalized	log.sigma.4.0.mm.3D_gldm_DependenceVariance	log.sigma.4.0.mm.3D_gldm_LargeDependenceEmphasis
0.230200066	1.641935976	8.478753541
0.1351777	8.559552556	25.44583534
0.216324407	2.192720168	9.940092166
0.20594594	2.870321212	11.37191651
0.238444767	1.630760159	7.735376045
0.21830262	1.677280632	9.66251944
0.16889292	2.706971092	14.94658754
0.261833618	1.292321545	6.934131737
0.217769671	2.409768451	9.85040885
0.163754412	4.393669087	16.42995481
0.235195466	1.626531409	9.048367594
0.19035625	2.830795456	12.17408907
0.189798571	2.941820901	14.2487946
0.225917736	1.850013281	9.677996422
0.206893186	2.410817415	10.86608123
0.079722928	20.42960279	62.05225277
0.24546186	1.556645676	7.544412607
0.192123017	3.409648444	13.03773585
0.24628311	1.502993588	7.573883162
0.132798622	10.2475925	29.32774009
0.127257589	9.632773786	29.29785605
0.212796106	1.936554895	10.19883721
0.182314989	3.197306486	13.24093739
0.21852862	2.246765877	9.428274428
0.160750157	3.645067383	14.96013289
0.223438447	2.000548132	9.060518732
0.106218549	19.87444831	49.54645124
0.170381726	4.085620132	14.88415911
0.235826626	1.88757345	8.292917847
0.130584466	10.42886679	29.3729347
0.160251576	3.957011059	15.87134319
0.157845295	7.200387318	21.11467116
0.210801413	2.222950437	10.47761928
0.314903846	0.74852071	4.903846154
0.271066192	1.262556249	6.86746988
0.213919224	2.022109597	10.00693069
0.182525559	3.216572145	13.04305556
0.15968402	4.644946591	18.40608229
0.18495751	2.806089856	13.44071856
0.217089043	2.474316181	10.04861773
0.154399459	4.981602411	18.75305292
0.24673726	1.58659956	7.912906611
0.234955292	1.648411595	8.429963459
0.230593218	1.734887329	8.434146341
0.207207269	2.52610066	11.19062833
0.133789714	5.973298866	22.5529623
0.191515618	2.813827714	12.44913268
0.222452036	2.566479056	10.03259005
0.262085097	1.32412564	7.462686567
0.236594618	1.779149748	8.054325956
0.168358386	7.639499083	21.1296729
0.222226284	1.948228544	9.278113664
0.17787587	5.291446921	16.28133996
0.185418044	2.615704834	13.11505922
0.109544691	10.69310558	34.15682968
0.262252456	1.264268917	6.740614334
0.240829493	1.586830293	7.674874601
0.214758031	2.509579513	10.37819026
0.196451533	2.746632941	11.64373464
0.204971649	2.40533343	12.08513053
0.204993318	2.381361381	10.63850873
0.178359693	4.547882126	15.1347207
0.130841948	8.429103172	26.98317152
0.110596014	11.87112396	36.49176126
0.287052178	1.267557179	6.34562212
0.188490465	3.72199374	13.37475853
0.237895849	1.614367801	7.47715736
0.196652633	3.428487314	12.31623397
0.259184782	1.287055843	6.829596413
0.208849092	2.305860285	10.29189458
0.188738747	4.06117339	13.84736842
0.23104937	1.660991605	8.539415412
0.146588107	6.223886545	20.99458204
0.133067371	8.48572378	26.81625968
0.123300298	9.787472767	30.03747277
0.247878689	1.457323262	7.327956989
0.219914578	2.075775293	9.882721576
0.255388336	1.399613007	7.06557377
0.206957235	2.734728585	10.99546485
0.190602238	2.708819914	12.17427969
0.292690224	1.055870496	5.412698413
0.247045373	1.470752346	7.140193885
0.18637034	3.337558292	13.00716332
0.250416171	1.628727705	7.75862069
0.186397181	3.064126675	13.23591861
0.136865643	7.294576613	24.72169467
0.245792058	1.549566815	7.627249357
0.270976425	1.180663128	6.389175258
0.195974798	3.22912001	11.77595628
0.24553288	1.604897959	7.228571429
0.155960856	8.705626771	23.2490566
0.24286769	1.926714764	8.084985836
0.139758962	8.106770424	24.34246575
0.157966774	6.338017109	20.35745721
0.14623338	6.277872713	21.72063383
0.325469856	0.811292162	5.150197628
0.196842268	2.524671053	11.52467105
0.270143433	1.1375504	5.991869919
0.226349023	1.66126663	10.47229551
0.18415844	3.412338445	12.51672862

log.sigma.4.0.mm.3D_gldm_LargeDependenceLowGrayLevelEmphasis	log.sigma.4.0.mm.3D_gldm_SmallDependenceHighGrayLevelEmphasis	log.sigma.4.0.mm.3D_gldm_LargeDependenceHighGrayLevelEmphasis
0.074442935	121.0198586	2144.243626
0.195156141	61.14631584	4764.700048
0.289044786	56.38189179	1030.476959
0.04806135	109.3426987	3984.239848
0.063795154	136.9408757	2876.740947
0.155851091	54.13469107	1483.617418
0.396291284	29.80474496	1349.970326
0.058614569	148.5950773	2183.39521
0.052907986	137.1258199	3178.506975
0.13161755	70.30014392	2892.404132
0.122442217	57.37896585	1843.71584
0.192674241	68.16520382	2035.180837
0.243728252	39.94827522	2108.913211
0.221869961	60.72289793	1874.931127
0.138392598	58.1933955	1736.463227
0.380088759	30.76822173	11339.62185
0.085645433	105.4724293	2120.786533
0.250051601	71.47576569	1808.641938
0.072218192	136.7568474	2734.833333
0.634854772	29.452211	2812.224206
0.196111459	47.45476255	5289.116194
0.168031526	60.80147539	1672.716279
0.150841715	67.70620294	2082.071769
0.050456078	118.3095704	3610.887734
0.537332831	34.91130538	1092.818937
0.147362266	94.91222304	1501.018732
0.242982694	47.37673784	10949.48702
0.343026855	54.92752288	1402.511514
0.086728011	94.9502659	2499.270255
0.179906834	59.15424843	6692.459874
0.240649016	50.01950665	1787.480654
0.187015737	62.04136545	3826.407589
0.1302813	69.88313921	2418.176094
0.057213726	110.6077057	1258.163462
0.037045606	137.5390629	2430.35241
0.073853711	81.79948796	2681.381683
0.19550014	64.9380134	1819.612847
0.330602346	33.77726886	2570.018187
0.14318037	69.21893555	3673.977246
0.125845786	83.63841201	2048.817922
0.106892001	68.34059664	4424.87924
0.08562769	114.6266137	2133.332634
0.085213668	83.54847725	2153.793544
0.127059561	109.5141947	2138.997073
0.064397261	85.86775603	2891.940895
0.543280552	29.40443048	1492.085278
0.08892813	69.22620977	2857.81669
0.305439627	67.11270135	1565.343053
0.111219846	104.3363329	2013.365672
0.053352477	173.4952532	3896.95171
0.191220773	52.22376062	3527.265479
0.226998881	63.24372673	1759.348247
0.117497382	79.75376358	3487.949228
0.806833773	48.49150968	2056.708968
0.321831784	38.71150758	5133.127242
0.073542694	116.0893664	1768.951081
0.044856519	140.0164463	2534.77793
0.036633341	130.1812541	4632.750193
0.143576615	72.11127912	1945.611794
0.303118446	37.50268874	1519.279228
0.197657589	77.57752027	1499.739972
0.097636714	83.48317024	3906.119387
0.251557213	35.84019211	3751.986408
0.223215499	41.142997	6974.659832
0.063838553	114.2861772	1491.460829
0.097297205	86.85524754	2913.371539
0.223761066	173.4197463	2333.055838
0.258500841	76.47199227	2150.821708
0.374674744	91.70353776	1386.838565
0.137484078	73.79679826	1629.699652
0.14102258	87.17469632	2660.945724
0.188358896	85.75029489	1536.418069
0.258604183	47.16017373	2834.622485
0.147115681	58.70965586	6120.774866
0.211912134	41.94940185	5293.750436
0.058167058	140.8075934	2409.600358
0.108061559	73.8109436	1868.354521
0.528822189	84.49585788	901.2
0.101337926	90.58725203	2346.935374
0.097398213	76.92299613	2475.712228
0.063494553	186.0984584	1578.484127
0.048408192	132.3342605	2310.873229
0.057517721	100.0287872	4120.206304
0.050075954	82.95263527	1969.041379
0.234183995	48.40524672	1661.71041
0.155970032	50.52374327	4759.321768
0.168576284	95.69859322	1251.44473
0.029399565	196.6504583	3215.125644
0.104519533	69.23778761	2030.005464
0.169317728	90.81193915	941.7904762
0.121878854	83.53112561	6087.60805
0.370598146	89.78611884	1016.103399
0.303478602	35.25007569	3153.968689
0.203305419	44.92594973	3445.201222
0.323760465	36.39742685	2845.536858
0.088137839	180.5192677	2319.193676
0.067316125	86.99056447	3631.691612
0.298177179	89.97657182	959.3739837
0.256382072	43.57359517	2215.741425
0.063624978	137.6797425	3629.033457

log.sigma.4.0.mm.3D_gldm_SmallDependenceLowGrayLevelEmphasis	log.sigma.4.0.mm.3D_gldm_LowGrayLevelEmphasis	log.sigma.4.0.mm.3D_gldzm_DistanceZoneVariabilityNormalized
0.003531902	0.008584145	0.918515165
0.002340788	0.009298659	0.901792092
0.005546736	0.025827341	0.918277811
0.001804102	0.004997204	0.872271627
0.005606765	0.010544605	0.847222222
0.00488468	0.015106647	0.846576528
0.005236571	0.029815381	0.752839506
0.003813324	0.008996237	0.886728715
0.002104807	0.005912249	0.927840463
0.001864095	0.007805674	0.95988305
0.005217324	0.01541229	0.856225223
0.003445215	0.014353993	0.936950651
0.004299834	0.017974041	0.935187455
0.003255135	0.016389313	0.905938043
0.007010931	0.016743792	0.915826114
0.001163246	0.007835001	0.939954069
0.004975586	0.012827578	0.777678608
0.003014924	0.017012161	0.968999938
0.003888059	0.012180184	0.863071356
0.003593318	0.023615137	0.976354737
0.001560097	0.007460622	0.945987654
0.003886122	0.014560745	0.870339569
0.002721185	0.01037675	0.952730451
0.004052606	0.008033775	0.743295657
0.007290343	0.033861719	0.958024567
0.005054444	0.017550195	0.951586942
0.001282347	0.005950885	0.906910895
0.004604035	0.01873765	0.953904106
0.004523729	0.011563119	0.959408306
0.002073046	0.00919312	0.960442305
0.003034546	0.013568893	0.967888643
0.001995869	0.008866995	0.966376206
0.00506075	0.017231233	0.932760506
0.015159133	0.020239498	0.834710744
0.005261077	0.008542231	0.864088889
0.002987387	0.008541054	0.861738671
0.003257154	0.013843336	0.948971225
0.003529831	0.017557602	0.975004006
0.003412445	0.013655394	0.867346939
0.004638643	0.012744646	0.970880148
0.002115992	0.006903284	0.930756082
0.002922716	0.010550032	0.901212371
0.003693706	0.010730916	0.894294097
0.003762005	0.014994722	0.820758743
0.002324145	0.006671514	0.90685045
0.003112592	0.019833415	0.996131543
0.002300036	0.008008325	0.875378208
0.006958287	0.024807744	0.93529525
0.005391275	0.020731539	0.751404959
0.003682291	0.010703346	0.765700061
0.002690535	0.010599905	0.949187146
0.004516157	0.018787924	0.914544397
0.00254237	0.008618551	0.916698481
0.002934559	0.025691731	0.976051423
0.001794821	0.01077579	0.963409501
0.006652446	0.014222886	0.855763655
0.002660123	0.006280114	0.772957126
0.002576477	0.005225351	0.719103353
0.003441698	0.013987308	0.895061728
0.003660939	0.019637642	0.992000129
0.003882819	0.014163009	0.896513553
0.002384384	0.00766587	0.931673205
0.002346819	0.01079694	0.945051198
0.001651758	0.007397929	0.951612668
0.007712575	0.014479015	0.92866941
0.003151083	0.009352463	0.945792623
0.003283788	0.017191071	0.80715851
0.004160393	0.019232279	0.947406866
0.006063975	0.031890893	0.832547837
0.003713687	0.013024345	0.922004098
0.002194107	0.008731031	0.903647747
0.004937345	0.018269009	0.926195802
0.002264379	0.0128373	0.95240928
0.000958817	0.005790143	0.957641186
0.00281077	0.009840828	0.957081739
0.003484568	0.009524942	0.82612118
0.003649775	0.010835022	0.870399248
0.008115384	0.04302779	0.872651519
0.00273465	0.009488583	0.827427219
0.001781088	0.007609393	0.910054089
0.007879692	0.013848567	0.815910585
0.004050655	0.00763297	0.868503026
0.001897305	0.00530248	0.92737629
0.01128505	0.015041	0.658203125
0.003109865	0.016590531	0.966578
0.001405105	0.006701783	0.8054957
0.004759309	0.015087442	0.948013983
0.002264391	0.004838989	0.800365009
0.009602418	0.014727708	0.869073597
0.012840252	0.022298321	0.896859504
0.001794881	0.007186144	0.90827795
0.007184473	0.02706481	0.951101109
0.004478579	0.016572954	0.916535537
0.003009649	0.012970963	0.908073993
0.003480707	0.017572863	0.946519338
0.005487397	0.017740223	0.708757396
0.002278329	0.008196244	0.942906574
0.008267883	0.034966735	0.855957031
0.004226294	0.022140199	0.718271684
0.003029049	0.006703616	0.899966234

log.sigma.4.0.mm.3D_gldzm_LowIntensityEmphasis	log.sigma.4.0.mm.3D_gldzm_LargeDistanceEmphasis	log.sigma.4.0.mm.3D_gldzm_HighIntensitySmallDistanceEmphasis
0.009902073	1.127659574	360.7384752
0.009969859	1.155357143	262.0325893
0.021942258	1.12804878	177.7134146
0.005920803	1.205696203	356.7428797
0.01413093	1.25	356.8365385
0.016303129	1.251162791	194.8837209
0.031376253	1.433333333	137.0444444
0.010115278	1.180803571	386.4263393
0.006359834	1.112454655	404.6840992
0.00771953	1.061433447	287.4180887
0.018690413	1.233898305	198.6262712
0.012990746	1.097759674	245.5351324
0.021660902	1.100591716	194.852071
0.013351699	1.1484375	216.8574219
0.023198242	1.132075472	203.0581761
0.010206948	1.092948718	246.9919872
0.013417544	1.382165605	267.0175159
0.012206244	1.047244094	265.2486877
0.012678988	1.221789883	370.2675097
0.019486335	1.035897436	161.7111111
0.008529356	1.083333333	258.494213
0.014085536	1.209059233	215.8022648
0.010241767	1.07266436	255.2153979
0.011284468	1.453658537	322.8439024
0.031381495	1.064343164	129.2808311
0.017347406	1.074468085	274.0097518
0.007851536	1.146818923	297.4547308
0.016850119	1.070815451	199.3809013
0.012736693	1.062176166	258.2393135
0.010503903	1.060559006	275.984472
0.012612326	1.048966268	204.3601741
0.008490194	1.051313378	264.957697
0.019746962	1.104499274	247.6850508
0.030356335	1.272727273	249.4090909
0.012921121	1.22	369.8533333
0.010413294	1.224137931	270.0736351
0.011699115	1.07860262	235.1053493
0.01810215	1.037974684	170.4306962
0.017222756	1.214285714	283.9201681
0.013666811	1.044334975	254.9956897
0.009400785	1.107734807	315.8411602
0.009878115	1.156327543	327.4565757
0.012036787	1.167962675	250.726283
0.013533507	1.298578199	314.9478673
0.008037216	1.146919431	305.021327
0.01441259	1.005813953	152.8745155
0.008942281	1.200306984	260.1302763
0.023253806	1.10041841	202.8012552
0.023615568	1.436363636	302.1863636
0.012425375	1.406542056	442.9719626
0.011581728	1.07826087	232.2214674
0.015695376	1.134185304	202.1956869
0.009377756	1.13064133	286.5704671
0.018512901	1.036363636	210.4060606
0.010120803	1.055928412	215.2695749
0.017479224	1.234718826	295.4969438
0.00695394	1.391709845	369.0797927
0.00762034	1.507042254	396.4089537
0.014061718	1.166666667	248.6927083
0.016687593	1.012048193	168.9568273
0.01292393	1.164218959	258.0507343
0.008633018	1.106253459	294.3898727
0.011966734	1.084821429	189.984747
0.009409974	1.074427481	248.9594466
0.018507377	1.111111111	285.875
0.011320684	1.083643123	293.7444238
0.012173602	1.324324324	403.1743243
0.015070357	1.081081081	254.0514385
0.016693575	1.276699029	235.0400485
0.01291803	1.12195122	235.2462737
0.007683124	1.152255639	296.0035244
0.016716463	1.115124153	261.6439052
0.010596696	1.073170732	214.5356326
0.005323491	1.064944134	316.6073673
0.014487463	1.06582155	216.5838615
0.010237465	1.288577154	361.8517034
0.012237679	1.208955224	244.039801
0.023214296	1.205035971	217.2634892
0.0094664	1.286153846	286.2884615
0.007290394	1.141602634	281.4377058
0.016292763	1.307692308	391.5961538
0.009546783	1.212264151	336.3081761
0.006445928	1.113207547	349.5149371
0.027054699	1.65625	228.5898438
0.013892503	1.051	198.547
0.007415221	1.327510917	249.6766376
0.01281919	1.080118694	268.6394659
0.005576548	1.337398374	481.9596883
0.025557343	1.211267606	207.443662
0.028091659	1.163636364	211.8090909
0.007719214	1.14454776	328.4602705
0.019457947	1.07523511	236.9090909
0.019494734	1.130909091	155.1072727
0.014896661	1.144886364	204.3383049
0.01781743	1.082489146	179.08267
0.016144012	1.530769231	413.6211538
0.00910357	1.088235294	311.4810049
0.02815937	1.234375	208.0898438
0.024872696	1.508928571	172.953125
0.00899259	1.158415842	415.1394389

log.sigma.4.0.mm.3D_gldzm_LowIntensityLargeDistanceEmphasis	log.sigma.4.0.mm.3D_gldzm_HighIntensityEmphasis	log.sigma.4.0.mm.3D_gldzm_DistanceZoneVariability	log.sigma.4.0.mm.3D_gldzm_ZonePercentage
0.011046004	366.1241135	259.0212766	0.399433428
0.011135951	268.1625	505.0035714	0.269619644
0.026074481	179.945122	150.597561	0.377880184
0.006845787	371.1170886	826.9135021	0.359772296
0.016274623	371.2115385	132.1666667	0.43454039
0.019577126	201.1209302	182.0139535	0.33437014
0.100697729	139.3444444	67.75555556	0.267062315
0.011313573	396.0290179	397.2544643	0.447105788
0.006922296	412.5695284	767.3240629	0.397787398
0.00875898	289.9078498	843.7372014	0.28373144
0.023524225	205.6813559	252.5864407	0.356711004
0.014170657	249.2362525	460.0427699	0.331309042
0.024701849	198.0118343	474.1400394	0.244455159
0.015438766	221.4921875	347.8802083	0.343470483
0.025287753	208.1289308	291.2327044	0.349066959
0.01103456	251.5128205	879.7970085	0.134734418
0.016536082	282.9872611	244.1910828	0.449856734
0.012861618	266.2388451	738.3779528	0.326758148
0.014295771	382.5680934	221.8093385	0.441580756
0.019940285	162.6290598	571.1675214	0.208853981
0.009367237	262.4462963	1021.666667	0.206738132
0.026686474	223.6916376	499.5749129	0.33372093
0.016225647	257.1107266	826.017301	0.31746613
0.034551628	346.8585366	152.3756098	0.426195426
0.033532974	130.3565684	357.3431635	0.309800664
0.018320392	275.8156028	268.3475177	0.406340058
0.008522127	308.3915171	555.9363785	0.176860935
0.025801531	200.5042918	444.5193133	0.325191905
0.013427385	260.7409326	740.6632124	0.437393768
0.012087698	278.6071429	618.5248447	0.253343824
0.017153406	205.6528836	889.4896627	0.289084618
0.009202564	267.3982896	1581.95785	0.276053963
0.0205418	251.8563135	642.6719884	0.338908018
0.104981335	250.9090909	45.90909091	0.528846154
0.017771636	377.1133333	129.6133333	0.451807229
0.011784222	282.591954	599.7701149	0.344554455
0.013405234	238.6375546	869.2576419	0.318055556
0.018367224	172.0987342	770.2531646	0.235539654
0.021054749	291.5210084	206.4285714	0.28502994
0.014211355	256.1133005	394.1773399	0.387035272
0.011254407	321.058011	336.9337017	0.245590231
0.011054913	334.8263027	363.1885856	0.422875131
0.014263426	255.7371695	575.0311042	0.391595615
0.018965497	325.4123223	346.3601896	0.411707317
0.009653366	312.5545024	574.0363349	0.337060703
0.014531243	152.9457364	514.003876	0.231597846
0.010253648	271.5310821	1140.617805	0.30543835
0.024313219	205.3556485	223.5355649	0.409948542
0.084977899	310.6	41.32727273	0.410447761
0.014292234	469.9579439	163.8598131	0.430583501
0.012953224	234.8913043	873.2521739	0.268691589
0.017002271	205.9840256	286.2523962	0.378476421
0.010703785	292.3056215	1157.790182	0.330541743
0.019933355	210.769697	161.0484848	0.279187817
0.010653656	217.2841163	1291.93214	0.205580254
0.02043049	305.99511	350.007335	0.465301479
0.013202077	386.6569948	745.9036269	0.44003648
0.011714172	422.7706237	357.3943662	0.384377417
0.016585138	253.9583333	257.7777778	0.353808354
0.017169521	169.0321285	247.0080321	0.282633371
0.018714393	263.7022697	671.4886515	0.353468617
0.009537589	301.4947427	1683.533481	0.329864914
0.012700095	193.3541667	635.0744048	0.217475728
0.00984144	252.3931298	498.6450382	0.191871109
0.019593443	289	100.2962963	0.497695853
0.01180055	298.7825279	508.8364312	0.346426272
0.01527524	413.9945946	149.3243243	0.469543147
0.015923333	256.8683522	1086.675676	0.35877385
0.019926166	243.5776699	171.5048544	0.461883408
0.014704459	240.2859079	680.4390244	0.366981601
0.009247241	302.3045113	961.481203	0.35
0.01797591	265.234763	410.3047404	0.39238264
0.01123763	217.1051829	1249.560976	0.253869969
0.005636456	320.6731844	1371.342179	0.213222156
0.014816415	220.8425158	1962.974647	0.223420479
0.012394162	378.5170341	412.2344689	0.447132616
0.017148915	250.2711443	349.9004975	0.359892569
0.027118437	222.3165468	242.5971223	0.455737705
0.021046464	296.7446154	537.8276923	0.368480726
0.008516362	288.1086718	829.0592755	0.320098384
0.018487745	405.3516484	222.7435897	0.541666667
0.016284028	347.6006289	552.3679245	0.474272931
0.0068738	358.7389937	884.7169811	0.341690544
0.097776639	239.875	42.125	0.44137931
0.014449824	199.819	966.578	0.29489826
0.009149454	277.9388646	922.2925764	0.209094229
0.013372068	272.4718101	319.4807122	0.433161954
0.007689353	506.2127371	590.6693767	0.475515464
0.027600024	216.1267606	61.70422535	0.387978142
0.085364387	213.5272727	49.32727273	0.523809524
0.008541626	337.7075232	1074.492815	0.297610063
0.030149642	238.1222571	303.4012539	0.45184136
0.020925621	158.9309091	252.0472727	0.269080235
0.016914108	208.5179924	958.9261364	0.258190709
0.021469255	181.1432706	1308.089725	0.238029625
0.020513689	438.4692308	92.13846154	0.513833992
0.009420075	318.0196078	384.7058824	0.335526316
0.091805204	209.15625	54.78125	0.520325203
0.041952856	186.7678571	80.44642857	0.295514512
0.009741706	424.5478548	272.689769	0.375464684

log.sigma.4.0.mm.3D_gldzm_IntensityVariabilityNormalized	log.sigma.4.0.mm.3D_gldzm_LowIntensitySmallDistanceEmphasis	log.sigma.4.0.mm.3D_gldzm_IntensityVariability
0.043458579	0.009616091	12.25531915
0.05002551	0.009678336	28.01428571
0.057108864	0.020909203	9.365853659
0.046478039	0.005689556	44.06118143
0.041584484	0.013595007	6.487179487
0.063926447	0.01548463	13.74418605
0.064691358	0.014045884	5.822222222
0.038285236	0.009815704	17.15178571
0.040096267	0.006219218	33.15961306
0.051981451	0.007459668	45.69169511
0.055374892	0.01748196	16.33559322
0.046519634	0.012695769	22.84114053
0.048757241	0.020900665	24.7199211
0.054009332	0.012829933	20.73958333
0.048316918	0.022675865	15.36477987
0.053644806	0.010000045	50.21153846
0.04679703	0.01263791	14.69426752
0.045332424	0.012042401	34.54330709
0.041650896	0.012274792	10.70428016
0.060781649	0.019372847	35.55726496
0.0610631	0.008319886	65.94814815
0.050103801	0.010935301	28.75958188
0.056186801	0.008745797	48.71395617
0.045520523	0.005467677	9.331707317
0.064005348	0.030843625	23.87399464
0.046325638	0.01710416	13.06382979
0.054035857	0.007683888	33.12398042
0.056549209	0.014612267	26.35193133
0.045752906	0.01256402	35.32124352
0.048623703	0.010107954	31.3136646
0.062810146	0.011477056	57.72252448
0.052664614	0.008312102	86.21197312
0.044516674	0.019548252	30.67198839
0.049256198	0.011700085	2.709090909
0.045511111	0.011708492	6.826666667
0.046749075	0.010070562	32.53735632
0.048354341	0.011272585	44.29257642
0.057766384	0.018035882	45.63544304
0.051091025	0.016264757	12.15966387
0.047368293	0.013530675	19.23152709
0.051921492	0.00893738	18.79558011
0.044166272	0.009583915	17.79900744
0.048250207	0.011480127	31.02488336
0.042216033	0.01217551	17.81516588
0.04796488	0.007633178	30.36176935
0.070646295	0.014382927	36.45348837
0.04855611	0.00861444	63.2686109
0.05299277	0.022988953	12.66527197
0.055206612	0.008274985	3.036363636
0.037688881	0.01195866	8.065420561
0.053149811	0.011238854	48.89782609
0.055946269	0.015368652	17.51118211
0.046952392	0.009046249	59.30087094
0.059320478	0.018157787	9.787878788
0.055595872	0.00998759	74.55406413
0.04186369	0.016741408	17.12224939
0.042809203	0.005391906	41.31088083
0.043419471	0.006596882	21.57947686
0.052107446	0.013430864	15.00694444
0.071047241	0.016567111	17.69076305
0.046743232	0.011476315	35.01068091
0.046377168	0.008406876	83.80354178
0.060467156	0.011783394	40.63392857
0.056042771	0.009302108	29.36641221
0.051097394	0.018235861	5.518518519
0.044616575	0.011200717	24.00371747
0.045434624	0.011398192	8.405405405
0.044237308	0.014857112	50.7401918
0.048920728	0.015885427	10.0776699
0.04873275	0.012471422	35.96476965
0.047488906	0.007292095	50.52819549
0.045722526	0.016401601	20.25507901
0.055416001	0.010436462	72.70579268
0.060859524	0.005245249	87.15083799
0.049261387	0.014405225	101.0351048
0.043778941	0.009698291	21.84569138
0.052498701	0.01100987	21.10447761
0.047461312	0.022238261	13.1942446
0.047597633	0.006571384	30.93846154
0.054343727	0.006983902	49.50713502
0.038548753	0.015744018	10.52380952
0.045261263	0.007862472	28.78616352
0.044860216	0.00633896	42.7966457
0.055664063	0.009374214	3.5625
0.056194	0.013753172	56.194
0.053799889	0.006981662	61.60087336
0.045795948	0.01268097	15.43323442
0.040830341	0.005048347	30.13279133
0.060900615	0.025046672	4.323943662
0.061157025	0.013773477	3.363636364
0.043876729	0.007513611	51.90617075
0.045371016	0.016785023	14.47335423
0.059570248	0.019137012	16.38181818
0.0537764	0.014392299	56.78787879
0.056866137	0.016904473	78.58900145
0.034556213	0.015051593	4.492307692
0.045259035	0.009024443	18.46568627
0.058105469	0.012247912	3.71875
0.060746173	0.020602656	6.803571429
0.040856561	0.008805311	12.37953795

log.sigma.4.0.mm.3D_gldzm_HighIntensityLargeDistanceEmphasis	log.sigma.4.0.mm.3D_gldzm_SmallDistanceEmphasis	log.sigma.4.0.mm.3D_glcm_SumVariance	log.sigma.4.0.mm.3D_glcm_Homogeneity1
387.6666667	0.968085106	801.3539759	0.303757618
292.6821429	0.961160714	547.1840226	0.403699863
188.8719512	0.967987805	316.3153402	0.339562609
428.6139241	0.948575949	1050.03549	0.321590341
428.7115385	0.9375	992.6459645	0.297256942
226.0697674	0.937209302	400.1325396	0.335593429
148.5444444	0.891666667	225.2446742	0.405267358
434.4397321	0.954799107	902.8816716	0.269659107
444.1112455	0.971886336	1015.977157	0.30567607
299.8668942	0.984641638	600.4913971	0.375971772
233.9016949	0.941525424	483.0531575	0.317237453
264.0407332	0.975560081	538.1571146	0.33870904
210.6508876	0.974852071	401.9096153	0.372329207
240.03125	0.962890625	523.1739258	0.330747959
228.4119497	0.966981132	467.8460436	0.335068415
269.5961538	0.976762821	566.3372467	0.539750757
346.866242	0.904458599	683.3239686	0.292691036
270.1994751	0.988188976	504.6062164	0.337091857
431.770428	0.944552529	956.7679809	0.277438952
166.3008547	0.991025641	256.5148887	0.433837637
278.2546296	0.979166667	543.6786502	0.435547687
255.2491289	0.947735192	465.4594004	0.332072888
264.6920415	0.98183391	522.1846716	0.364549132
442.9170732	0.886585366	1041.023215	0.3128033
134.6595174	0.983914209	203.7324236	0.386018818
283.0390071	0.981382979	524.1309342	0.303658756
352.1386623	0.963295269	688.7593918	0.472187814
204.9978541	0.982296137	333.3423533	0.371557827
270.7474093	0.984455959	733.0382672	0.302093087
289.0978261	0.984860248	594.4908788	0.41459492
210.8237214	0.987758433	377.0256819	0.386547221
277.1606597	0.987171655	598.8245044	0.386724975
268.5413643	0.973875181	625.9828217	0.327101591
256.9090909	0.931818182	608.2912574	0.269709601
406.1533333	0.945	987.276679	0.286230323
332.6652299	0.943965517	738.4659679	0.319286232
252.7663755	0.980349345	461.8744257	0.348539907
178.7708861	0.990506329	366.4074896	0.397591999
321.9243697	0.946428571	740.9386001	0.352724517
260.5837438	0.988916256	576.3199436	0.32203885
341.9254144	0.973066298	728.0282536	0.381887616
364.3052109	0.960918114	786.0373565	0.291222813
275.7807154	0.958009331	643.4236943	0.306980725
367.2701422	0.92535545	755.1596457	0.294275068
342.6872038	0.963270142	772.9008064	0.333864336
153.2306202	0.998546512	212.5330997	0.435000368
317.1343054	0.949923254	667.2831615	0.345403792
215.5732218	0.974895397	433.8550264	0.314470913
344.2545455	0.890909091	750.7209299	0.286918872
577.9018692	0.898364486	1280.852216	0.282697494
245.5706522	0.980434783	492.1083526	0.384652214
221.1373802	0.966453674	476.7847015	0.308835041
315.2462391	0.967339667	667.3727315	0.346505262
212.2242424	0.990909091	420.3293058	0.362316069
225.3422819	0.986017897	449.3574315	0.460556954
347.9877751	0.941320293	747.9964161	0.277103943
456.9658031	0.902072539	940.0905134	0.275836459
528.2173038	0.873239437	1182.282178	0.305048163
275.0208333	0.958333333	502.4208684	0.346512949
169.3333333	0.996987952	340.0448387	0.364455256
286.3084112	0.95894526	503.8634997	0.320479292
329.9142225	0.973436635	772.5546337	0.345327931
206.8318452	0.978794643	401.9540116	0.437975284
266.1278626	0.98139313	543.9936364	0.461996851
301.5	0.972222222	667.9161551	0.287475356
318.9349442	0.979089219	682.6409373	0.342229743
457.2756757	0.918918919	901.4396553	0.284765967
268.136007	0.97972973	543.4757585	0.327265869
277.7281553	0.930825243	527.6699796	0.286357294
260.4444444	0.969512195	507.9694812	0.330689863
327.5084586	0.96193609	665.5168127	0.333123456
279.5981941	0.971218962	548.5581649	0.300904393
227.3833841	0.981707317	402.437045	0.398191952
336.9364525	0.983763966	705.9017567	0.421771963
237.8771331	0.983544612	531.3415155	0.434650118
445.1783567	0.927855711	917.6275252	0.274418932
275.1965174	0.947761194	559.2465993	0.320423757
242.528777	0.948741007	415.4543458	0.287769616
338.5692308	0.928461538	709.3041554	0.309604337
314.7925357	0.964599341	669.6631611	0.345300959
460.3736264	0.923076923	887.5738412	0.248274925
392.7704403	0.946933962	923.2413101	0.284162795
395.6352201	0.971698113	964.9469002	0.341012509
285.015625	0.8359375	614.594513	0.310815844
204.907	0.98725	383.5911526	0.363106299
390.9877729	0.918122271	630.7454543	0.413809724
287.8011869	0.979970326	551.149847	0.296093673
603.2249322	0.915650407	1373.78039	0.263123711
250.8591549	0.947183099	493.1558394	0.366585876
220.4	0.959090909	417.055887	0.308298539
374.6965342	0.96386306	800.0415652	0.373864383
242.9749216	0.981191223	432.660536	0.288901622
174.2254545	0.967272727	330.2227405	0.408697132
225.2367424	0.963778409	448.5080308	0.388728258
189.3856729	0.979377713	339.1650605	0.409143278
537.8615385	0.867307692	1200.036574	0.238230888
344.1740196	0.977941176	902.5496164	0.339870445
213.421875	0.94140625	424.2855795	0.275606529
242.0267857	0.872767857	483.552813	0.360195014
462.1815182	0.96039604	929.3485325	0.331614737

log.sigma.4.0.mm.3D_glcm_Homogeneity2	log.sigma.4.0.mm.3D_glcm_ClusterShade	log.sigma.4.0.mm.3D_glcm_MaximumProbability	log.sigma.4.0.mm.3D_glcm_Idmn	log.sigma.4.0.mm.3D_glcm_SumVariance2
0.210880713	227.6241195	0.012274111	0.976383688	114.6125283
0.328721665	213.1521779	0.031506635	0.982786249	69.21251527
0.247817116	169.0652513	0.020289496	0.968315013	67.15575664
0.231485978	-54.88954146	0.010911686	0.980530634	90.96891448
0.203622796	-124.3669187	0.014035396	0.970245029	153.2862273
0.241711969	-6.740150726	0.013926605	0.967566166	62.59137481
0.324344153	148.1023606	0.027111371	0.972724754	46.86354267
0.175308536	241.9951753	0.009771576	0.973889352	139.9106811
0.214223858	290.3899184	0.010071589	0.978436213	121.526591
0.292333069	220.470207	0.01630416	0.982650549	68.26369471
0.224120376	-108.0151385	0.013472156	0.96561649	73.73442379
0.248433908	308.2074992	0.011456737	0.978508773	95.05653743
0.285637257	415.8026428	0.014105686	0.978900231	86.35898275
0.23739429	-105.725287	0.012382007	0.973599026	88.01618053
0.243190849	205.0509343	0.014402072	0.973059537	81.30007987
0.488638734	85.56324612	0.122831372	0.990680779	43.18210205
0.197676672	-159.4844914	0.012324398	0.969427973	108.2225977
0.249950693	512.5566408	0.012854082	0.975252517	105.8414889
0.18415505	-503.566646	0.012376904	0.969414468	138.7313289
0.35966717	135.0049853	0.043308321	0.981234491	49.74398044
0.361097258	89.19497206	0.032237898	0.988643159	42.50728439
0.239717737	407.5790006	0.010896271	0.98209326	90.74525375
0.27839687	245.751579	0.017550127	0.982047595	72.3838811
0.21785534	-535.0947478	0.016475666	0.973766299	104.7189074
0.300434212	90.45757715	0.0219513	0.973821694	42.5526717
0.212427939	308.8711149	0.015037954	0.97057509	96.84530602
0.404994907	142.1980191	0.086396489	0.989353314	45.61617122
0.286151237	285.9868312	0.016845298	0.97908505	70.42749745
0.209567052	-150.8752981	0.010870152	0.968028423	112.9978054
0.34138072	67.16535142	0.030397823	0.985420262	69.05417852
0.303708233	262.1733202	0.023381003	0.981770665	56.14867355
0.305723931	224.3441028	0.0271488	0.98498123	77.28315227
0.23629707	43.86474543	0.009882072	0.976292448	101.058113
0.173745093	77.91252423	0.025091097	0.951825825	106.8898226
0.191167886	-484.776927	0.019390211	0.966473406	106.8210222
0.227753902	-33.85354763	0.011972958	0.976886945	95.15190033
0.262679135	441.2976417	0.012877943	0.979839232	91.51835445
0.315270457	27.86110028	0.019078526	0.980247021	62.1626437
0.264823908	-343.0016567	0.019305956	0.98031606	80.0663134
0.23087065	-12.1129853	0.014161673	0.97521633	91.03509493
0.299415346	53.86768285	0.020766365	0.986443221	67.12935314
0.197206161	185.491116	0.010636702	0.97366694	125.9245359
0.213444401	-118.5343206	0.009409586	0.970432688	92.58872558
0.201129663	76.09964716	0.011007625	0.971877356	130.3811075
0.2426551	118.1448039	0.010989962	0.978643804	78.96131787
0.360866532	165.5274659	0.028112657	0.980651117	38.54488876
0.255130809	62.56392881	0.01052791	0.980471339	77.84752786
0.222077376	28.9531594	0.014595566	0.966986335	92.38588268
0.193106845	-227.5109274	0.02598293	0.958697811	93.39514407
0.191469255	-491.601147	0.016387649	0.975487769	151.7868688
0.301275886	193.6540751	0.029566038	0.98374655	69.52442478
0.217823538	-37.65378456	0.015022514	0.952639179	94.26198706
0.260692944	266.3816077	0.019053527	0.982313576	82.93700222
0.272395069	-118.3698482	0.020845657	0.974961392	66.45634041
0.396069402	217.6278222	0.06247493	0.983460771	62.02603036
0.182338145	-35.65733194	0.010100718	0.970628216	118.7940925
0.182363219	32.95470677	0.007294585	0.970320796	111.4289742
0.213559851	-366.825032	0.016225233	0.978380338	102.4069692
0.257088887	265.0569145	0.0147003	0.976249764	87.8844655
0.272481836	-33.61159348	0.015543384	0.97308473	58.29180355
0.230341383	457.0044182	0.013396625	0.973319036	103.3184498
0.259069088	395.1561519	0.015110332	0.98462194	101.9503247
0.364894879	145.8073712	0.039340613	0.986595335	44.97080241
0.396295254	161.8528336	0.043896306	0.984040149	43.88874758
0.190795315	-73.29573741	0.018347991	0.965292015	98.08910602
0.256308299	362.8461103	0.016915807	0.979352233	88.34746582
0.19191531	-515.3535732	0.015547846	0.971034803	118.0821411
0.240195391	286.3556502	0.010984403	0.972205566	116.9761398
0.190524688	-195.3773167	0.013295946	0.964612787	109.6734908
0.241383819	337.5993226	0.011147962	0.974487717	91.12332271
0.246186246	271.4272889	0.013596718	0.977593402	96.22581239
0.208100474	216.8522039	0.009165921	0.971109712	113.1160646
0.319762723	276.5533821	0.025028742	0.981757745	64.9726919
0.346964608	128.5151091	0.027853654	0.986514351	53.02333911
0.3630385	306.3313163	0.039525359	0.986709305	60.99757268
0.181386714	71.0676766	0.00982029	0.975650984	122.1285974
0.228740361	113.8638796	0.012624739	0.978255929	82.64288955
0.193400732	474.2767133	0.010034914	0.962660906	120.2115635
0.218582783	136.8818467	0.011166073	0.971191744	95.12854566
0.255419227	120.9634876	0.013289423	0.981595407	72.55584048
0.155792459	-28.45351531	0.01046149	0.962493943	147.9956081
0.189812867	-96.43203935	0.008293579	0.975482028	110.2299995
0.254536728	197.4387025	0.017557184	0.981202457	91.76757346
0.218017709	-185.0376585	0.034697921	0.959189952	68.70536215
0.276018935	222.6523744	0.013433444	0.978100792	68.02388465
0.337187509	235.2683936	0.026744195	0.985262798	56.06692972
0.202857063	458.1817722	0.011577484	0.972348488	120.4746541
0.17035017	-440.8851114	0.007338184	0.970103679	145.0289499
0.279137424	73.4175159	0.029550888	0.970901816	51.64262209
0.214576769	175.0068293	0.032451955	0.960687618	74.2480099
0.294041346	168.4515274	0.033973437	0.98288947	83.24494308
0.195946667	492.2345993	0.012043595	0.969408249	118.3842216
0.329303243	129.2123819	0.033356254	0.975558739	53.41322377
0.306906551	162.3434633	0.023297558	0.981429507	62.36418468
0.331144884	97.79829899	0.024438173	0.980638589	58.10327309
0.147671199	-473.8365687	0.015125361	0.958738462	211.3977061
0.250887909	-34.712154	0.013921475	0.977104023	84.50114196
0.180367973	-10.15726948	0.020757882	0.942460921	83.31757426
0.270159304	-197.9932042	0.020484254	0.975745622	75.21610089
0.244604644	368.266211	0.015858839	0.981767103	102.6138671

log.sigma.4.0.mm.3D_glcm_Contrast	log.sigma.4.0.mm.3D_glcm_DifferenceEntropy	log.sigma.4.0.mm.3D_glcm_InverseVariance	log.sigma.4.0.mm.3D_glcm_Entropy	log.sigma.4.0.mm.3D_glcm_Dissimilarity
29.22330579	3.427469584	0.216464982	8.218148098	4.280372342
18.87953451	3.164816867	0.30658256	7.823835187	3.124460407
20.04460789	3.183148172	0.250173325	7.509075752	3.524190994
26.77224525	3.422020969	0.235418313	8.436313833	4.031265495
31.35287827	3.46326242	0.213660656	8.009709428	4.402033097
18.76159508	3.159064184	0.246343385	7.736933174	3.469205826
12.06299702	2.786201982	0.330822487	6.922810026	2.62281883
40.52927706	3.690977213	0.178921624	8.716564337	5.097123368
31.58305882	3.554667987	0.21881668	8.712041619	4.374133614
17.76431576	3.168500884	0.291257041	7.924752576	3.169243993
23.4533415	3.276707814	0.227556564	7.907799638	3.899285899
22.17916348	3.286820528	0.250114467	8.202904932	3.645715592
16.63145529	3.105141166	0.284700744	7.971623863	3.104249347
20.7659472	3.246264852	0.243253479	8.097343308	3.614993849
21.42739275	3.270006872	0.24818678	7.993121214	3.616587029
10.16724019	2.675698583	0.358259552	6.750156928	1.986667173
30.01048304	3.400988565	0.210813511	8.202839785	4.375077597
25.82878463	3.387739334	0.248725909	8.393662785	3.880558187
38.42086213	3.602758895	0.186633371	8.381830288	4.953947583
12.60209879	2.947673133	0.31808146	7.415072854	2.595479492
11.41950807	2.89850304	0.332618018	7.354807153	2.479203511
21.99518153	3.293939478	0.246399626	8.185554504	3.657089243
18.37421755	3.182602179	0.281776172	7.988651463	3.264590386
25.60846828	3.361540738	0.223697107	7.939573579	3.985535511
13.79911996	3.0051599	0.296089417	7.379997226	2.864734598
33.02803603	3.544883815	0.215356044	8.202883235	4.464478396
10.77178585	2.839111175	0.337265345	7.139601828	2.290829732
17.59452641	3.158961164	0.278594504	7.810259069	3.202008423
29.30500186	3.444439241	0.212563155	8.445289914	4.323280705
17.89592873	3.146919558	0.311742499	7.830967975	3.015353761
15.21755219	3.076095729	0.300661751	7.676282836	2.964047908
18.34919987	3.177603565	0.283610115	8.045926975	3.185673822
24.53801763	3.371199481	0.243561922	8.432386321	3.852459032
36.98637965	3.494611429	0.180842088	6.642322782	4.924332584
33.18640999	3.500610526	0.198660264	7.798864683	4.592655344
25.30670829	3.38248175	0.235045953	8.358033439	3.963797957
23.49365453	3.343209352	0.262583906	8.213005408	3.677788075
13.18533185	2.984736369	0.308344618	7.722958759	2.774367798
20.19309302	3.195215774	0.265617416	7.739205721	3.452833452
25.78141476	3.395570031	0.23940805	8.196760699	3.947396714
18.62826815	3.182847391	0.291774105	7.828399095	3.172172948
32.88383888	3.548290874	0.201685758	8.505379879	4.54834564
27.0475798	3.384713398	0.222569455	8.297895579	4.132864293
33.35693645	3.557364001	0.205013542	8.57380494	4.546692499
21.9903919	3.294327153	0.250148633	8.184922053	3.658231122
11.02131627	2.841274027	0.34108811	7.054612924	2.447687782
20.01974131	3.257767818	0.260624301	8.225275901	3.469459678
26.38066891	3.389950483	0.22108907	7.965613776	4.075715947
36.37371356	3.517616898	0.179450736	6.861840763	4.800979485
40.1423887	3.674569947	0.197190779	8.186164628	4.971874087
16.52217858	3.134706733	0.28333964	7.920962485	3.077457563
28.40630173	3.409511518	0.218910023	7.980428007	4.246746811
24.28344321	3.364271805	0.253233485	8.304520823	3.757947303
16.91164919	3.123881234	0.262614332	7.623318259	3.204672637
16.06600247	3.018297784	0.320125097	7.479567681	2.721149894
36.91681992	3.619345893	0.192139499	8.544469369	4.841308923
39.67788398	3.601912906	0.189728116	8.716315666	4.987242509
31.68170665	3.51967669	0.217406888	8.396937045	4.385282662
20.10021278	3.23372414	0.265568379	7.999265326	3.469223983
14.08279827	2.950191141	0.273209328	7.53978995	2.988215632
27.93519038	3.472872068	0.231049797	8.412370346	4.094288884
24.67579994	3.392503206	0.251224566	8.489073476	3.774394885
11.85692268	2.890052261	0.32742946	7.283178657	2.52369219
12.50739249	2.881539057	0.347000721	7.150450573	2.45158204
29.9873193	3.408696662	0.203004512	7.47009278	4.404922064
25.58937234	3.397983631	0.25378492	8.221461881	3.839593199
36.4808363	3.561210625	0.200474342	7.946375687	4.77151748
29.21980883	3.497031047	0.237312173	8.616491252	4.137974919
30.41348207	3.449360479	0.200160448	8.025405207	4.46782819
24.90083433	3.387308033	0.247623704	8.306721491	3.856552445
27.87597963	3.420155903	0.2388379	8.383094578	4.03252652
30.19017421	3.494157507	0.212824306	8.424373455	4.353954582
17.62198486	3.15841764	0.29687917	7.855847063	3.074036883
14.54811919	3.029516728	0.315480247	7.594155649	2.772580869
14.49635541	3.017270936	0.330753739	7.61443676	2.679594029
39.72093404	3.629992411	0.190368486	8.580908131	5.012566111
25.3297779	3.355435381	0.238523856	8.114943579	3.936322156
32.45064489	3.518820525	0.203629444	8.217967415	4.554124992
30.108123	3.4733201	0.216792128	8.404477897	4.307085067
19.90800548	3.244524313	0.256244474	8.066279062	3.492713637
50.64626076	3.746379521	0.168488917	8.452363172	5.742944083
34.0452893	3.573973391	0.198661124	8.568064269	4.660190405
25.9105397	3.410595071	0.246620396	8.372311301	3.875444945
26.49449354	3.327553063	0.226492799	6.822218113	4.078241684
18.52516993	3.178298357	0.273364612	7.970816034	3.288932817
14.0763345	3.011910487	0.324334724	7.626759868	2.749014992
30.65537245	3.497494718	0.213877193	8.282715072	4.400476007
44.42837798	3.743336513	0.180789223	8.889773518	5.329440069
15.4002983	2.915687036	0.292511308	6.661893992	3.064228408
27.55114014	3.245305434	0.21190219	6.397708961	4.167856757
23.65659039	3.342995411	0.27164994	8.208180948	3.57041963
36.79081774	3.63049423	0.199417679	8.271417567	4.734789716
14.10049529	3.027072992	0.302304759	7.496345176	2.804355545
16.74020938	3.129248111	0.293402496	7.862598861	3.053346466
14.08944345	3.023166524	0.318686918	7.683395812	2.777511913
59.53987664	3.803947213	0.152810313	7.861291532	6.239281319
22.2371569	3.310070756	0.258886687	8.155813458	3.639284224
35.30203335	3.415651367	0.179004766	6.814639955	4.833245719
15.02577993	3.01632416	0.286631327	7.458807864	3.063026569
29.7235616	3.474633957	0.250022147	8.132348386	4.065539486

log.sigma.4.0.mm.3D_glcm_DifferenceVariance	log.sigma.4.0.mm.3D_glcm_Idn	log.sigma.4.0.mm.3D_glcm_Idm	log.sigma.4.0.mm.3D_glcm_Correlation	log.sigma.4.0.mm.3D_glcm_Autocorrelation
9.756089179	0.894434127	0.210880713	0.595888321	270.4801963
8.575570867	0.917208312	0.328721665	0.573658649	193.3198592
7.024199852	0.880183886	0.247817116	0.53886811	119.0959411
9.651196528	0.904865372	0.231485978	0.548342765	344.2893985
10.9324492	0.883430819	0.203622796	0.662823077	326.9664969
6.328579864	0.876838084	0.241711969	0.541068993	146.8361299
4.37074247	0.892197489	0.324344153	0.593971697	89.79817672
13.73815312	0.888280449	0.175308536	0.554068801	299.5425714
11.82253732	0.900369385	0.214223858	0.588843532	335.141166
7.360805894	0.912792527	0.292333069	0.590869097	210.2918805
7.373476023	0.8732873	0.224120376	0.520404902	172.3476752
8.086949563	0.900931101	0.248433908	0.623950856	191.363187
6.542069302	0.903227933	0.285637257	0.674664982	148.8854195
7.283603722	0.888755616	0.23739429	0.619621488	186.5468322
7.79184055	0.889154729	0.243190849	0.586212772	168.6890585
6.024190428	0.946022566	0.488638734	0.620682054	196.2197912
9.269456212	0.880285436	0.197676672	0.56982662	234.1341801
9.92024871	0.896038466	0.249950693	0.609171389	180.0482748
12.20668134	0.880410715	0.18415505	0.571265007	315.0210428
5.630377313	0.912362273	0.35966717	0.595825888	101.2246843
5.096059306	0.929992742	0.361097258	0.577022903	191.483927
8.073242305	0.907994318	0.239717737	0.612251308	168.1718199
7.306495356	0.910240298	0.27839687	0.597956173	186.2930963
9.036711041	0.88965369	0.21785534	0.61209116	340.742753
5.317699602	0.892216751	0.300434212	0.510495099	82.79562428
12.25874537	0.885607393	0.212427939	0.495632308	183.6124097
5.361140709	0.935422578	0.404994907	0.618562853	234.53376
6.896392824	0.903672037	0.286151237	0.60077063	125.9840595
9.586110839	0.878184577	0.209567052	0.588977324	249.6923709
8.449213053	0.923950945	0.34138072	0.590730199	208.3555724
6.112277379	0.909921318	0.303708233	0.573590939	140.1243415
7.609618033	0.919369139	0.305723931	0.618689671	210.419528
9.122871389	0.896099005	0.23629707	0.611188232	218.5426539
11.62637545	0.85136994	0.173745093	0.484035267	203.1046421
11.04598119	0.875476931	0.191167886	0.5321545	322.3613235
8.96208115	0.896027647	0.227753902	0.582192681	251.964942
9.484632779	0.905930602	0.262679135	0.593359296	166.523648
5.243272905	0.90608627	0.315270457	0.650142868	137.8719802
7.401869916	0.905625705	0.264823908	0.600600424	251.8103432
9.666822713	0.893939918	0.23087065	0.561469513	201.8704449
8.201196045	0.923707215	0.299415346	0.569751329	248.3028617
11.35054396	0.888818292	0.197206161	0.587604468	265.478678
8.814265367	0.88276182	0.213444401	0.551480722	222.2384042
11.61045512	0.886296391	0.201129663	0.596825884	256.3464036
8.121748553	0.900427318	0.2426551	0.56651879	262.2420393
4.744679581	0.910252579	0.360866532	0.554163644	85.7228713
7.621012856	0.904978048	0.255130809	0.59257248	231.1930119
9.024519894	0.877170488	0.222077376	0.556907498	156.3261727
12.23026272	0.863577045	0.193106845	0.443904306	246.9487296
14.31576713	0.893575586	0.191469255	0.584897977	410.1277503
6.714867158	0.914851782	0.301275886	0.616824094	177.3066921
9.182411937	0.855371701	0.217823538	0.538289698	168.9321185
9.48376963	0.910940736	0.260692944	0.549650632	230.2416265
6.32770291	0.893302579	0.272395069	0.596563484	153.8097562
8.155821294	0.923517441	0.396069402	0.59516243	162.2888146
12.50910026	0.882721801	0.182338145	0.530133205	252.8911975
12.24359182	0.882873039	0.182363219	0.484257393	310.0031209
11.32755632	0.900119626	0.213559851	0.534825895	381.8160774
7.692485298	0.896499795	0.257088887	0.627461958	180.1050552
4.656433001	0.887269715	0.272481836	0.611632614	128.666231
10.67194955	0.890779492	0.230341383	0.575903604	178.9733848
9.850938021	0.916616399	0.259069088	0.614624338	263.2653343
5.111156135	0.924672764	0.364894879	0.584579498	147.6286966
6.201488043	0.922958515	0.396295254	0.554252625	190.626429
9.92377349	0.872256524	0.190795315	0.534840926	226.3685179
10.21381487	0.90486043	0.256308299	0.553988448	234.5179534
12.36279368	0.884434523	0.19191531	0.52976892	297.0965413
11.49008684	0.890265773	0.240195391	0.60015216	191.830591
9.605582271	0.870553522	0.190524688	0.568353744	185.0342848
9.46091936	0.89331138	0.241383819	0.57310362	181.0207825
10.1729734	0.900661376	0.246186246	0.557389451	229.3927214
10.40043873	0.884267594	0.208100474	0.580754416	192.7294151
7.730118136	0.913271472	0.319762723	0.576778149	148.2651646
6.572900013	0.925102709	0.346964608	0.571351118	240.900892
7.080926546	0.927811111	0.3630385	0.617946438	188.3990874
12.66437519	0.89241752	0.181386714	0.513104098	303.2641441
8.878210705	0.899467174	0.228740361	0.535097075	196.1184548
10.83701213	0.869078689	0.193400732	0.576058622	148.6832118
10.37476892	0.885679868	0.218582783	0.523160311	241.9484475
7.287427246	0.906818987	0.255419227	0.571553745	231.0687825
15.47111487	0.867767409	0.155792459	0.495612003	291.5271042
11.44156974	0.891642152	0.189812867	0.531935369	306.0830134
10.14691215	0.90859726	0.254536728	0.562381575	319.3541316
9.162051998	0.864732416	0.218017709	0.448967226	208.2806831
7.106371384	0.901395746	0.276018935	0.571311633	142.3240006
6.203405136	0.92117353	0.337187509	0.600479302	218.6702133
10.61186046	0.886175031	0.202857063	0.597269074	192.9817045
14.72685735	0.881507924	0.17035017	0.5378525	437.5894057
4.821611605	0.885585724	0.279137424	0.529743183	172.4356361
8.814721598	0.86670241	0.214576769	0.470768835	144.9276995
10.22346562	0.915620938	0.294041346	0.559201382	270.0390515
13.5409457	0.882665263	0.195946667	0.529878313	153.1376285
5.983273251	0.899007442	0.329303243	0.581537884	125.1209407
6.985037161	0.910754058	0.306906551	0.577794626	163.1324433
6.128584042	0.90986117	0.331144884	0.608816864	128.5122376
16.90945905	0.861673103	0.147671199	0.562658988	380.062549
8.53995834	0.898392668	0.250887909	0.586269872	300.9985001
10.39779824	0.838493504	0.180367973	0.416174743	146.938887
5.319300735	0.893238202	0.270159304	0.669179383	174.2315354
12.47295034	0.91150143	0.244604644	0.556383527	307.539211

log.sigma.4.0.mm.3D_glcm_SumEntropy	log.sigma.4.0.mm.3D_glcm_AverageIntensity	log.sigma.4.0.mm.3D_glcm_Energy	log.sigma.4.0.mm.3D_glcm_SumSquares	log.sigma.4.0.mm.3D_glcm_ClusterProminence
5.361647873	15.93940041	0.004211259	36.77297826	37639.56846
5.025147186	13.52251052	0.007636495	22.83169315	16247.20966
4.933618201	10.3568784	0.006951563	21.80009113	11606.03318
5.265921702	18.30118794	0.003728967	29.36012546	23349.95438
5.465813153	17.3115414	0.004659716	46.74720765	57392.29314
4.940937029	11.65551524	0.005547283	20.33824247	9135.450973
4.654019845	9.0034851	0.010718634	14.73163492	6723.402855
5.52586852	16.81559041	0.002913715	45.19729167	52485.07584
5.456681824	17.88360657	0.00316377	38.31740958	44099.64198
5.048795553	14.14762185	0.005889919	22.40390545	13941.76201
5.048723692	12.63842068	0.005059739	24.29694132	12566.72486
5.266587938	13.24607099	0.004355267	30.36843273	25628.33713
5.166528154	11.47550594	0.005249024	25.9610203	21614.00539
5.195996858	13.03451456	0.004408287	27.31167567	18093.26823
5.135885853	12.44258927	0.005084841	26.41821483	19373.47287
4.547427356	13.74230668	0.029542255	13.67362641	9324.152242
5.315151658	14.76003979	0.004158458	35.66795856	28737.53321
5.332340818	12.74543547	0.003929191	34.09486363	31768.29102
5.453754536	17.27187353	0.003703305	44.87257675	49960.63027
4.79762643	9.588117644	0.009248399	15.58651981	8217.329858
4.721165854	13.58973169	0.009691183	13.86602312	6200.806926
5.217644263	12.3474812	0.00440497	28.85207259	25488.07439
5.081291094	13.21300842	0.005776939	23.47973791	16559.31534
5.232111829	18.09697353	0.005145625	33.25052753	29639.07203
4.694789518	8.694818374	0.007896482	14.08794791	5154.11123
5.225152479	13.08076929	0.004333097	33.93422598	26164.80044
4.694501617	15.07907996	0.016611726	14.57063612	8173.042712
5.024733163	10.6343567	0.005986683	22.29776302	14102.95585
5.34951334	15.20364799	0.003487446	36.38358105	26955.0827
5.046774034	14.08431196	0.00760789	22.5204696	15541.84772
4.880875965	11.40564107	0.007219458	18.01068438	11138.24868
5.140075314	14.1037789	0.005812852	24.80964342	18058.70176
5.331321163	14.2179591	0.003644229	32.3770904	26367.85599
4.857152208	13.63404036	0.010864939	36.18554258	24716.01426
5.195558661	17.61071779	0.005571035	35.98120663	31003.78671
5.263188348	15.44778256	0.003761973	31.01882374	21201.84706
5.210773723	12.34385674	0.004572887	30.06246817	23302.93918
4.974114759	11.20801327	0.006276721	18.83699389	9903.87678
5.071589129	15.45280794	0.006823356	25.55653663	22184.80294
5.214430013	13.68422848	0.004405599	29.90146502	22208.1343
5.028149264	15.51872771	0.0064956	22.15728154	14420.2038
5.433781837	15.69314924	0.003390744	40.35187795	41200.50444
5.224924888	14.38763929	0.003841484	30.37912747	19298.97454
5.473398579	15.37749969	0.003302314	41.89434632	44768.33493
5.153033925	15.85381338	0.004430959	26.00708897	17544.40438
4.56762343	8.878804446	0.011031155	12.39155126	4728.640308
5.165542007	14.81155724	0.00435924	25.25728806	16110.74136
5.170698582	11.83657056	0.004885411	29.97023218	19792.821
4.870181045	15.33333465	0.009773731	33.35257526	23459.71339
5.499083677	19.77533904	0.004655763	46.83232789	71712.12719
5.059970982	12.85090037	0.005999351	22.07949556	14353.87329
5.137263763	12.34623057	0.00488536	30.6670722	16839.74315
5.189945412	14.83400075	0.004491641	27.59716675	23040.80124
4.972676919	11.89120558	0.006597846	20.8419974	12610.81127
4.879493686	12.32364397	0.01393267	20.11788929	13574.50547
5.406769317	15.44738979	0.003262035	39.93167565	36343.37814
5.392170425	17.39133231	0.002912354	38.31605164	31518.40696
5.302870951	19.32559553	0.004156945	32.90232476	32245.32872
5.186249656	12.80508199	0.004979152	27.5396908	21200.61199
4.904225091	10.8438245	0.006560568	18.09365045	8151.990374
5.294599281	12.73124139	0.003888429	33.80809481	27722.15797
5.341534952	15.80892303	0.003856252	32.08066067	35715.1546
4.7150839	11.81571107	0.011020019	14.4059748	8465.61117
4.676072441	13.53106171	0.012770626	14.26761512	7097.997421
5.066305297	14.55268987	0.006501669	33.12633352	23821.44778
5.207315855	14.9500275	0.004691956	29.53754489	24585.87597
5.279370982	16.77470082	0.005173955	38.94629712	40351.29098
5.41660226	13.11125699	0.003269212	37.51507832	34613.21514
5.262606006	12.88344398	0.004542684	35.54127232	27592.99051
5.231954408	12.89964753	0.004133812	30.01956757	22425.59643
5.281440693	14.74271327	0.004059435	32.10683958	23964.68581
5.362347234	13.19886332	0.003477188	36.80043475	30350.70446
4.990209871	11.71929239	0.00662113	21.23939379	13623.86162
4.864617858	15.30125942	0.007745229	17.57985161	8401.743722
4.903942086	13.3588002	0.010782388	19.57835329	15102.2457
5.419427355	17.10994056	0.003214687	40.52560057	42008.81026
5.134109463	13.59339779	0.004449071	27.98525314	17944.72227
5.33347329	11.32198842	0.003922806	39.36995962	35848.97836
5.263259943	15.21183642	0.003752303	32.8069069	21745.48899
5.087439288	14.86563764	0.004773494	23.94768097	13688.54938
5.495840348	16.61739931	0.003380898	51.05430382	54962.50099
5.370716148	17.16542429	0.003153743	36.26593544	29501.57814
5.257832137	17.6104392	0.004260549	29.47942125	25521.76644
4.758774905	14.05833286	0.010663301	23.79996392	12843.37027
5.034666073	11.40772206	0.005221326	21.79745235	13213.4281
4.883841476	14.48844248	0.008243551	18.20780731	10304.55794
5.364438934	13.1581015	0.003936498	38.93765637	35993.96626
5.563577283	20.58800864	0.002598001	46.19855531	52313.58641
4.550565702	12.78053157	0.012126689	16.7607301	6084.759952
4.570577365	11.53720855	0.013470561	25.44978751	16526.86687
5.173323451	16.12102101	0.006332222	27.1870004	24769.0784
5.314755673	11.68001317	0.003953687	40.82916601	34471.68058
4.837068435	10.73717668	0.008329386	16.87842976	7978.128659
4.984854514	12.3425722	0.006347184	20.15325597	12755.67133
4.915277769	10.84079355	0.007279281	18.07109866	9634.12165
5.548142325	18.8660009	0.00493417	67.89823082	100730.1264
5.187935195	16.99365057	0.004674924	27.37471011	20126.56243
4.767048825	11.61221756	0.00971744	29.6549019	16075.07828
5.026408559	12.61522569	0.007257755	22.5604702	14282.68209
5.265398992	17.28880543	0.004847729	34.2963762	36218.13472

log.sigma.4.0.mm.3D_glcm_SumAverage	log.sigma.4.0.mm.3D_glcm_Imc2	log.sigma.4.0.mm.3D_glcm_Imc1	log.sigma.4.0.mm.3D_glcm_DifferenceAverage	log.sigma.4.0.mm.3D_glcm_Id
31.56610935	0.914909546	-0.201358463	4.280372342	0.303757618
26.88717783	0.825043269	-0.144209407	3.124460407	0.403699863
20.7137568	0.890487826	-0.195398353	3.524190994	0.339562609
36.23407786	0.765043265	-0.108742509	4.031265495	0.321590341
34.43266416	0.967163149	-0.293556936	4.402033097	0.297256942
23.31103048	0.821403171	-0.140518121	3.469205826	0.335593429
18.0069702	0.901357322	-0.225825287	2.62281883	0.405267358
33.14489235	0.878087655	-0.159518605	5.097123368	0.269659107
35.36325382	0.816048184	-0.124324603	4.374133614	0.30567607
28.11863704	0.77619366	-0.11812714	3.169243993	0.375971772
25.27684136	0.822383903	-0.138863917	3.899285899	0.317237453
26.31572858	0.840388286	-0.148497769	3.645715592	0.33870904
22.92956621	0.830521564	-0.149324551	3.104249347	0.372329207
26.05505523	0.83319091	-0.143202539	3.614993849	0.330747959
24.795983	0.847829926	-0.15260367	3.616587029	0.335068415
27.41991532	0.856163369	-0.188289279	1.986667173	0.539750757
29.29120105	0.896919858	-0.187011526	4.375077597	0.292691036
25.30032688	0.815808907	-0.132630823	3.880558187	0.337091857
34.05127146	0.93074992	-0.218444115	4.953947583	0.277438952
19.17623529	0.779994559	-0.128441729	2.595479492	0.433837637
27.10793425	0.735773573	-0.110245618	2.479203511	0.435547687
24.57371803	0.820636934	-0.135804969	3.657089243	0.332072888
26.2893131	0.78096343	-0.119889918	3.264590386	0.364549132
35.82988028	0.919021132	-0.213176093	3.985535511	0.3128033
17.38963675	0.74325975	-0.111214152	2.864734598	0.386018818
25.89405864	0.882473479	-0.170680965	4.464478396	0.303658756
30.05450613	0.810080758	-0.148027106	2.290829732	0.472187814
21.23716735	0.807563264	-0.136839093	3.202008423	0.371557827
30.24764633	0.818586264	-0.132849885	4.323280705	0.302093087
27.96864858	0.831551651	-0.146486813	3.015353761	0.41459492
22.79346772	0.740961794	-0.108876463	2.964047908	0.386547221
27.97690502	0.782338402	-0.126170272	3.185673822	0.386724975
28.24205688	0.807944817	-0.125543927	3.852459032	0.327101591
27.23878424	0.990374957	-0.460722767	4.924332584	0.269709601
34.8642835	0.953109223	-0.265867337	4.592655344	0.286230323
30.62645646	0.794886937	-0.119994639	3.963797957	0.319286232
24.45505057	0.786152215	-0.119643717	3.677788075	0.348539907
22.41602654	0.784145499	-0.126283808	2.774367798	0.397591999
30.77732581	0.87536512	-0.184388379	3.452833452	0.352724517
27.24228634	0.839363733	-0.142884993	3.947396714	0.32203885
30.7351301	0.828581621	-0.14400719	3.172172948	0.381887616
31.12460966	0.877180355	-0.163672238	4.54834564	0.291222813
28.69297906	0.799478801	-0.123995466	4.132864293	0.306980725
30.4660973	0.879477914	-0.16377042	4.546692499	0.294275068
31.4954597	0.78619916	-0.118183408	3.658231122	0.333864336
17.75760889	0.738484146	-0.117261542	2.447687782	0.435000368
29.44378322	0.744020045	-0.101857579	3.469459678	0.345403792
23.64395501	0.883636659	-0.178206148	4.075715947	0.314470913
30.49457723	0.982872187	-0.398524079	4.800979485	0.286918872
39.09372978	0.9522723	-0.254683642	4.971874087	0.282697494
25.61622206	0.77994006	-0.121351875	3.077457563	0.384652214
24.69246113	0.834565125	-0.14664188	4.246746811	0.308835041
29.36475352	0.75174962	-0.106216477	3.757947303	0.346505262
23.78241116	0.8690044	-0.173256991	3.204672637	0.362316069
24.55956344	0.832010447	-0.156314884	2.721149894	0.460556954
30.4892546	0.865486322	-0.154498555	4.841308923	0.277103943
34.17679986	0.782164583	-0.114466428	4.987242509	0.275836459
38.16188902	0.834383637	-0.139351574	4.385282662	0.305048163
25.54384746	0.87584631	-0.171229599	3.469223983	0.346512949
21.68764899	0.829481474	-0.1554978	2.988215632	0.364455256
25.3068445	0.785229191	-0.114970976	4.094288884	0.320479292
31.2371126	0.78254602	-0.11541002	3.774394885	0.345327931
23.60886122	0.776080509	-0.132028744	2.52369219	0.437975284
27.03892881	0.812097597	-0.150207527	2.45158204	0.461996851
28.93221423	0.970631169	-0.321645078	4.404922064	0.287475356
29.58478583	0.82201697	-0.134356052	3.839593199	0.342229743
33.2595966	0.947573292	-0.254243416	4.77151748	0.284765967
26.06766627	0.788693209	-0.114784732	4.137974919	0.327265869
25.70325764	0.921510183	-0.212989725	4.46782819	0.286357294
25.64835073	0.790859122	-0.117795317	3.856552445	0.330689863
29.14084766	0.783528444	-0.119159996	4.03252652	0.333123456
26.22551	0.851333402	-0.147200387	4.353954582	0.300904393
23.35993106	0.751963953	-0.112560369	3.074036883	0.398191952
30.41570686	0.759040097	-0.117316415	2.772580869	0.421771963
26.5908435	0.776776648	-0.12438499	2.679594029	0.434650118
33.62133989	0.870297745	-0.158417555	5.012566111	0.274418932
26.96384485	0.824825551	-0.137484152	3.936322156	0.320423757
22.51122302	0.902842358	-0.189356146	4.554124992	0.287769616
30.0448769	0.785173471	-0.119803538	4.307085067	0.309604337
29.52299934	0.753730136	-0.107520018	3.492713637	0.345300959
32.68459078	0.945850453	-0.237422576	5.742944083	0.248274925
33.88312238	0.818147931	-0.126610609	4.660190405	0.284162795
34.80716425	0.790819271	-0.120752421	3.875444945	0.341012509
28.11666572	0.969331001	-0.343009291	4.078241684	0.310815844
22.79814393	0.742616118	-0.109461905	3.288932817	0.363106299
28.85602213	0.747523751	-0.11381946	2.749014992	0.413809724
26.11448402	0.890457826	-0.177430291	4.400476007	0.296093673
40.61282271	0.835514266	-0.130571505	5.329440069	0.263123711
25.56106315	0.939782136	-0.283024442	3.064228408	0.366585876
23.07441709	0.984713865	-0.434658708	4.167856757	0.308298539
31.94623623	0.788271498	-0.121327954	3.57041963	0.373864383
23.03968806	0.88831799	-0.173848255	4.734789716	0.288901622
21.47435335	0.808722049	-0.140118974	2.804355545	0.408697132
24.63516295	0.747508391	-0.110888543	3.053346466	0.388728258
21.67946558	0.760982088	-0.116122633	2.777511913	0.409143278
36.98710879	0.987164835	-0.379522851	6.239281319	0.238230888
33.78866756	0.832602569	-0.139676735	3.639284224	0.339870445
23.22443512	0.980820642	-0.389866837	4.833245719	0.275606529
25.23045138	0.917540102	-0.224613885	3.063026569	0.360195014
34.01763082	0.903362729	-0.191537915	4.065539486	0.331614737

log.sigma.4.0.mm.3D_glcm_ClusterTendency	log.sigma.4.0.mm.3D_firstorder_InterquartileRange	log.sigma.4.0.mm.3D_firstorder_Skewness	log.sigma.4.0.mm.3D_firstorder_Uniformity
114.6125283	211.7598228	0.22658924	0.047043151
69.21251527	138.160099	0.409110943	0.064789893
67.15575664	177.1476393	0.23284802	0.061001508
90.96891448	201.1704254	-0.100402351	0.049565008
153.2862273	228.463768	-0.190941384	0.044824295
62.59137481	177.4475441	-0.152513293	0.060106567
46.86354267	107.2952042	0.330472953	0.078392871
139.9106811	258.2319927	-0.018480702	0.040744858
121.526591	215.8842354	0.14169113	0.043600299
68.26369471	152.7299194	0.520330545	0.062853187
73.73442379	200.4367867	-0.338688812	0.058337927
95.05653743	188.4962626	0.404602792	0.05184572
86.35898275	181.8761082	0.436688121	0.056002637
88.01618053	210.2567081	-0.154063647	0.053214435
81.30007987	166.7815876	0.24797771	0.056890957
43.18210205	69.09744358	0.430101485	0.118068908
108.2225977	212.0763865	-0.195099259	0.048784493
105.8414889	211.4145145	0.476189862	0.048720318
138.7313289	225.8026123	-0.378883684	0.043409974
49.74398044	130.5957069	0.378608606	0.074174432
42.50728439	114.9690987	0.393057596	0.07943855
90.74525375	186.1407185	0.412228719	0.054019064
72.3838811	151.842598	0.475015045	0.063905595
104.7189074	201.134697	-0.438798192	0.051940474
42.5526717	132.3649606	0.418678549	0.074047196
96.84530602	203.2917061	0.33650391	0.049838467
45.61617122	96.91094589	0.558433528	0.091773053
70.42749745	160.4303713	0.492004956	0.060896844
112.9978054	259.0603867	-0.170966635	0.046886822
69.05417852	146.9712414	0.263662868	0.063057394
56.14867355	144.405345	0.558309096	0.068933918
77.28315227	168.2642002	0.451848815	0.060402617
101.058113	213.5625381	0.034892936	0.048329228
106.8898226	216.6701584	-0.020340816	0.057507396
106.8210222	205.4095325	-0.494009704	0.052347946
95.15190033	230.0337467	-0.047734665	0.049068719
91.51835445	200.5029831	0.561258469	0.053978347
62.1626437	161.4495106	0.070559404	0.063228022
80.0663134	133.2531261	-0.459424142	0.066295672
91.03509493	194.2253036	0.006699254	0.052441792
67.12935314	143.8351898	0.239413288	0.062753029
125.9245359	239.3237534	1.19E-05	0.044636202
92.58872558	226.462059	-0.215732437	0.050509539
130.3811075	222.593399	0.020858892	0.043388459
78.96131787	185.0671272	0.153838863	0.054150916
38.54488876	132.3041377	0.65077266	0.080273023
77.84752786	174.6844215	0.151193926	0.055373345
92.38588268	220.1944237	-0.117184287	0.053290927
93.39514407	167.3108978	-0.464248027	0.062708844
151.7868688	172.3313828	-0.486463486	0.052002154
69.52442478	165.4875374	0.359332034	0.061623832
94.26198706	258.0408955	-0.200191084	0.054264405
82.93700222	182.1174545	0.375886322	0.053598548
66.45634041	147.8877716	-0.272513417	0.064254855
62.02603036	102.4711914	0.512227337	0.086960516
118.7940925	219.7086754	-0.055187218	0.044417783
111.4289742	250.4804993	-0.015816724	0.04290961
102.4069692	180.6224136	-0.498223631	0.054500735
87.8844655	175.8276043	0.222233704	0.053631474
58.29180355	165.1091232	-0.113195397	0.063423697
103.3184498	232.0415688	0.383790095	0.049288879
101.9503247	197.3994732	0.349897258	0.050184275
44.97080241	105.2955046	0.42119246	0.084404436
43.88874758	108.6191931	0.549395373	0.082147941
98.08910602	195.7438278	-0.208685428	0.052177791
88.34746582	173.9982681	0.492552247	0.054946723
118.0821411	197.6008215	-0.426773594	0.052204386
116.9761398	232.2778702	0.225492843	0.045524087
109.6734908	225.6519251	-0.321086673	0.050554003
91.12332271	191.6060944	0.425414297	0.05319549
96.22581239	216.5231171	0.373217808	0.050735587
113.1160646	246.896759	0.098670639	0.045554003
64.9726919	141.9282217	0.541156503	0.065230545
53.02333911	137.6682081	0.40888343	0.069800599
60.99757268	106.6243086	0.646250519	0.078724327
122.1285974	248.0294533	-0.019022365	0.043482869
82.64288955	205.3528137	0.105990223	0.053207971
120.2115635	239.1580124	0.267794864	0.045267401
95.12854566	226.1913567	0.172990135	0.049088343
72.55584048	177.253603	0.27397744	0.058097099
147.9956081	272.1399486	-0.062376726	0.038044848
110.2299995	238.3573151	-0.135776864	0.045371663
91.76757346	185.4438038	0.210584269	0.053033483
68.70536215	152.9475021	-0.589240404	0.077384067
68.02388465	175.4742203	0.399056517	0.058866858
56.06692972	125.0534306	0.613299044	0.074485596
120.4746541	244.095686	0.294248394	0.0453407
145.0289499	264.8857822	-0.483974932	0.042513318
51.64262209	179.205265	-0.041427903	0.071934068
74.2480099	172.6562538	0.055528882	0.068480726
83.24494308	165.986866	0.252230487	0.058125739
118.3842216	243.1272144	0.372249412	0.04540603
53.41322377	138.4260383	0.283291953	0.068989089
62.36418468	154.914978	0.326761969	0.063980129
58.10327309	155.1549683	0.271205678	0.065751822
211.3977061	356.2540665	-0.261285763	0.03722914
84.50114196	171.3473587	-0.143039751	0.053552686
83.31757426	190.8311996	-0.141084897	0.055324212
75.21610089	149.0263443	-0.433987264	0.065726359
102.6138671	187.9655495	0.431735078	0.049924069

log.sigma.4.0.mm.3D_firstorder_MeanAbsoluteDeviation	log.sigma.4.0.mm.3D_firstorder_Energy	log.sigma.4.0.mm.3D_firstorder_RobustMeanAbsoluteDeviation	log.sigma.4.0.mm.3D_firstorder_Median
122.8710806	17434518.28	85.45775562	-26.95464516
90.58710032	29796875.67	60.07908753	-21.6557312
97.91844987	7839286.338	72.0608983	-67.88574982
114.0451111	52374074.37	82.73195882	21.48507881
134.5072446	10261370.43	95.14647833	-8.029623032
95.91333994	12541983.03	71.46501526	-66.32537079
74.46462671	3111246.301	49.39478868	-1.497210503
143.9520798	30987750.89	106.2094552	-10.84298801
129.3841226	55799200.36	91.53864394	-36.50730515
92.71885788	45601492.3	64.24556083	-44.38386726
105.9825195	15617085.04	80.33268963	-30.88356972
110.6273477	29091436.63	78.63328688	-39.69600105
104.4440095	34123386.62	75.72215656	1.304434657
111.4923338	19393730.56	84.61661509	9.265581131
101.7166689	15356720.8	71.25085367	24.03131866
59.84536799	54234537.42	32.04613722	0.752197027
121.312202	17093271.85	88.72021443	-39.60940933
119.8731116	50621377.5	86.68123248	-31.17998505
136.4992207	16651345.14	96.3984633	34.78820992
78.48795686	29071572.06	53.68629077	-14.61735058
72.61139333	55819262.43	49.12192216	-50.83357811
105.5984148	29640973.31	75.28167567	-4.721551895
92.43518721	39714474.73	62.93064541	-42.17326355
115.7783026	9664398.258	83.99621164	34.15057373
77.94023869	15733997.39	55.36600684	-72.85068512
116.15112	16081861.58	81.53513419	-61.44939232
69.37574498	31859606.97	43.00045252	-16.13225079
94.71219153	25427695.89	66.5752003	-74.60070038
130.1347429	40404217.41	102.1430908	6.969692707
92.61861448	44262417.86	62.74418692	-53.7553997
85.73855838	42491850.12	60.12007954	-57.9917984
97.93689351	90435222.53	68.28862936	-4.05283165
117.8884894	41858092.09	86.51163933	-3.432048798
119.743199	2265919.559	88.00196913	16.65676689
118.0092187	7330946.941	83.59711497	-10.96788502
120.4766659	41831318.68	92.71635293	-19.69441986
112.3599385	55560522.36	82.85233523	-50.03019905
90.63822935	41000704.84	66.33941994	5.024611473
94.73262455	13168138.81	60.0887751	20.81834412
111.4056157	21394464.69	79.34437866	-23.80129433
91.80990954	21276695.06	62.42834618	-27.39855576
132.333813	25623057.23	97.29544679	-15.50272846
119.428078	35009274.54	92.04365208	-23.30849266
131.9931591	27407493.58	94.59739495	-9.308965683
105.454456	31785275.5	75.73110332	-9.787410736
76.70273901	21554604.18	55.4866579	-48.19152832
101.6060716	67337935.48	72.77561096	4.740086794
116.4524257	14229143.72	88.91484336	-55.02017975
103.810102	2468552.53	66.81744665	11.16798353
128.3325664	14576065.7	80.43782766	26.49230385
93.44393877	47235331.71	66.54820217	-12.47480917
126.8190073	23124220.35	104.2952677	-68.51570129
106.2461861	69299181.34	74.10279633	-22.46598625
91.36933845	7831585.719	63.26971478	8.366312027
76.82494069	73290184.48	47.12292081	-4.245641708
127.9545125	23323406.22	92.47071264	-34.50378799
133.1521171	58980865.23	100.3879196	-37.01754379
115.0790292	28026896.19	77.06414759	10.90184879
105.1042419	16133398.24	74.37869427	-59.80194664
89.76618426	11072941	66.55308461	-19.65044785
124.4319649	48777821.8	94.38320009	-47.39910889
113.2383319	110997643.4	79.67478899	0.820100158
70.30014547	27448650.33	45.5753061	11.66179323
73.49236785	26581157.17	47.86094175	-23.72291946
114.1319865	4863907.781	81.81405625	-51.23562241
105.8593029	29674716.07	72.30251521	20.09238625
123.2050553	10399186.98	81.93187588	-19.39112282
126.8425005	76375733.77	93.17030858	-1.140934467
124.0328021	13618253.44	91.65419096	-61.06179237
108.2643623	36311885.74	77.62585784	-24.80406952
117.1533364	61818848.81	86.63815154	-39.23192024
129.0848131	28920744.15	97.92556372	-37.52138519
88.94737346	67648398.38	61.45870365	-19.53922272
82.45236218	76913035.68	57.90377488	-32.73200798
79.4007431	111002339	48.78785844	9.725392818
135.2394998	32618537.76	100.5894476	-35.14015579
110.27307	23786002.75	82.8692813	-60.67239761
128.2802668	17274394.28	94.72345931	-66.23318481
119.7427298	46189222.67	90.31621173	-90.21736908
99.6472552	46004381.77	73.40129636	-52.98469162
151.9193415	25094604.25	112.1390631	-123.8113098
128.024588	32116152.71	97.01666234	-17.06277657
107.9346467	53577403.33	74.96676057	19.02204323
87.11455633	1944852.571	56.90498026	24.46177673
97.83836654	48262149.79	71.55783482	-7.042399883
80.36862703	58795359.69	53.73366293	-18.4778204
131.7271484	21006883.63	99.26540713	-54.15543747
144.6501134	50834959.73	107.2282898	-19.02277946
93.26921075	2264694.793	70.23332859	-26.56039238
98.8114272	1864287.929	65.25234508	-38.22090912
101.8479953	70221808.49	68.21724217	-9.199127197
133.4940739	22801536.93	100.8217917	-93.05289841
83.67965698	12700740.29	58.98061954	-38.07260513
90.76192778	54673679.05	64.19350875	-26.575387
89.15059206	70274733.62	64.53407057	-4.368436337
182.3937984	12791122.16	142.0331498	-48.77031708
106.0100314	21697786.5	73.05638454	17.03014755
110.5073378	2204902.934	79.77079752	18.65061569
91.2252381	5052828.027	63.08858877	36.32481766
116.8122311	20120978.83	79.20490547	-55.67925262

log.sigma.4.0.mm.3D_firstorder_TotalEnergy	log.sigma.4.0.mm.3D_firstorder_Maximum	log.sigma.4.0.mm.3D_firstorder_RootMeanSquared	log.sigma.4.0.mm.3D_firstorder_90Percentile	log.sigma.4.0.mm.3D_firstorder_Minimum
470731993.5	421.914917	157.1457448	174.8441544	-402.1965027
804515643.1	432.4551392	119.7752583	136.5644043	-333.1584473
211660731.1	265.4225769	134.3981836	94.40844574	-322.0983887
1414100008	453.6386414	140.9833645	199.2586395	-415.4534302
277057001.6	320.3009949	169.0656812	221.2085419	-438.7032471
338633541.7	185.4493866	139.6617945	66.23160706	-354.6600952
84003650.14	268.561676	96.08425922	150.9508392	-217.0136566
836669274	482.5621948	175.8576103	188.2226166	-430.2727966
1506578410	436.1005249	163.827477	185.4547394	-450.9510498
1231240292	380.3654175	121.3245868	133.258606	-352.4838257
421661296.1	245.1486511	137.4191421	97.5923111	-366.2868958
785468789.1	423.6565552	140.106564	157.2527679	-347.7656555
921331438.7	389.3192749	128.2689936	176.1266449	-271.7761536
523630725.2	344.372406	131.7072788	159.4093246	-316.8058472
414631461.7	382.8424377	129.8344839	187.7704773	-263.1373291
1464332510	454.2281494	88.35666613	110.372113	-322.9770813
461518339.9	318.1885071	156.4893867	121.6963379	-400.3052979
1366777193	444.8674316	147.3339073	192.4316452	-312.203125
449586318.8	406.6672668	169.1465597	214.8316467	-403.894104
784932445.7	352.3712769	101.8773651	110.9109955	-241.2892151
1507120085	389.7973328	103.3690352	78.43055038	-366.036377
800306279.3	530.1890259	131.2749939	173.6274048	-292.6816406
1072290818	409.7435608	120.5906292	137.024292	-340.4299622
260938753	310.0844116	141.7473247	184.620575	-411.0631409
424817929.5	252.4877777	114.3158088	70.55751343	-271.7012939
434210262.6	405.7419434	152.2258559	154.9442734	-357.2381287
860209388.2	385.1271667	95.8751216	115.4061394	-364.6441345
686547789	368.1972961	133.2080311	110.4929153	-301.4293518
1090913870	344.1499939	151.30072	184.1723236	-364.3545837
1195085282	428.6292114	131.956198	93.90279083	-392.5617676
1147279953	359.2389221	115.6132358	101.7025436	-312.2259521
2441751008	511.055542	123.4927492	186.5400269	-306.4515991
1130168486	401.658783	143.4897983	175.1674072	-345.1845703
61179828.09	319.6350098	147.6065312	238.8757462	-311.4049377
197935567.4	252.2775726	148.5973266	132.3773499	-458.684906
1129445604	381.442688	143.9047379	153.1392075	-398.2819214
1500134104	482.8484802	138.8950973	160.7456879	-306.8727722
1107019031	342.0444946	110.5640993	138.7924805	-273.0508118
355539748	377.7428894	125.5795609	153.5274445	-367.6136169
577650546.7	384.0109253	142.8114301	127.9781525	-351.7331848
574470766.7	489.6490173	120.1443474	136.0944519	-395.4926453
691822545.2	419.2286072	163.9717467	170.9105743	-420.6393738
945250412.7	308.1537781	146.0175238	129.4969131	-392.644989
740002326.6	411.1750488	163.5206963	192.4429169	-386.2348938
858202438.6	365.6648865	130.0963752	153.5549637	-380.147583
581974312.8	305.5325317	98.35862134	108.1484375	-230.9387207
1818124258	423.7833862	125.6375541	173.9027634	-330.4099426
384186880.5	284.6789246	156.2266448	92.49514008	-360.8985291
66650918.31	278.32901	135.7277931	167.3293289	-392.8355713
393553773.8	451.5358582	171.254489	218.6493683	-478.7597961
1275353956	438.0689087	117.4536741	137.8348618	-311.9713135
624353949.4	158.5990601	167.2171414	92.48970642	-396.7535706
1871077896	501.4606018	134.6714491	159.8283539	-368.0584106
211452814.4	304.1792603	115.1147865	145.0190735	-298.5231323
1978834981	428.4388123	105.9983776	140.8635773	-279.1325989
629731967.9	415.7134705	162.8926733	158.9958282	-416.1615906
1592483361	379.7042236	163.9971322	164.4786255	-461.4724731
756726197.2	411.7033386	147.2272635	148.4876129	-494.851593
435601752.6	304.12854	140.7831651	126.2445801	-365.0301514
298969406.9	243.7998199	112.1097899	110.5744858	-283.6326294
1317001189	422.9124451	151.7210093	165.9192383	-332.1856079
2996936371	584.7072754	142.3461958	183.6894379	-369.9382019
741113559	435.6368713	94.24997804	130.3026031	-250.453476
717691243.6	311.9661255	98.65658803	119.0990906	-341.9234314
131325510.1	251.443634	149.7141328	132.677774	-406.5439148
801217334	518.4642334	138.2316711	214.4619476	-318.0152893
280778048.6	351.9502869	162.4619204	142.1414993	-458.8943787
2062144812	425.9709473	154.5632993	200.4065216	-322.1326294
367692842.8	254.4417267	174.7403804	78.07265472	-418.09198
980420915	405.0160217	134.3749657	174.62883	-309.5660706
1669108918	468.9385071	142.6013592	180.166037	-350.0531006
780860091.9	377.7713928	160.0507674	154.6919434	-362.4691162
1826506756	445.6982422	114.4109277	138.5405838	-289.2232971
2076651963	378.0038147	107.0149943	115.5793953	-380.944397
2997063152	475.2948914	109.9625302	161.0845825	-294.937561
880700519.6	477.9228516	170.9622193	152.366272	-473.4378357
642222074.3	411.2724304	145.9264932	102.6661621	-383.8417053
466408645.6	337.4909058	168.2815473	143.5619583	-349.4265137
1247109012	312.5698242	161.8158416	121.565564	-428.9335022
1242118308	377.7166748	127.1399837	130.3503494	-389.005249
677554314.8	319.3652649	223.1387045	103.0116684	-547.0609131
867136123.1	444.8202209	154.7559545	164.3016815	-435.473999
1446589890	487.1491089	138.5265836	203.378038	-399.202301
52511019.41	238.3752899	115.8135413	157.3431793	-340.0705872
1303078044	412.139801	119.2997234	170.2111969	-267.1756592
1587474712	377.2042236	103.6190977	141.0958633	-328.5994568
567185858.1	411.8383484	164.3202233	168.9886551	-357.5032959
1372543913	345.6098633	180.9820001	154.4485535	-567.6958618
61146759.41	204.3970795	111.2446915	145.2320587	-324.361145
50335774.08	257.2612	133.2483513	97.82743683	-337.2227783
1895988829	482.8286743	132.9129932	165.3224823	-381.7200317
615641497.2	430.0982666	179.7130895	137.3200455	-353.3363647
342919987.8	263.4953918	111.4779747	104.6522797	-278.9016113
1476189334	377.6941528	115.6185439	116.2688622	-322.3414612
1897417808	362.086731	110.0173268	134.7027054	-272.8436584
345360298.3	348.6029663	224.8506062	185.2451324	-529.2995605
585840235.6	339.1672363	133.5798429	188.3205948	-395.6442871
59532379.23	264.8712463	133.8881627	183.1973389	-284.1177063
136426356.7	279.444397	115.4642805	161.0698151	-292.933075
543266428.3	497.0333557	157.9020559	148.3581268	-458.300354

log.sigma.4.0.mm.3D_firstorder_Entropy	log.sigma.4.0.mm.3D_firstorder_StandardDeviation	log.sigma.4.0.mm.3D_firstorder_Range	log.sigma.4.0.mm.3D_firstorder_Variance	log.sigma.4.0.mm.3D_firstorder_10Percentile
4.621561944	154.7866324	824.1114197	23958.90158	-235.196701
4.230969172	117.9447698	765.6135864	13910.96872	-173.6189819
4.213293702	118.4707494	587.5209656	14035.31847	-213.1843063
4.503151457	139.4890244	869.0920715	19457.18794	-168.7191315
4.670896138	168.6282271	759.0042419	28435.47897	-247.4117584
4.184526916	114.9135479	540.1094818	13205.12349	-245.0312561
3.922039085	95.92070066	485.5753326	9200.780815	-114.3967712
4.780501873	173.8111249	912.8349915	30210.30714	-264.790274
4.703460405	160.3895469	887.0515747	25724.80676	-242.4775543
4.225944071	116.8136128	732.8492432	13645.42013	-169.1388016
4.254878451	126.5319881	611.4355469	16010.34402	-241.3895386
4.455397311	137.0287544	771.4222107	18776.87952	-202.1390137
4.329351534	128.1773924	661.0954285	16429.44393	-156.6780106
4.369287573	131.6725336	661.1782532	17337.6561	-186.4337204
4.342287811	126.7432339	645.9797668	16063.84735	-140.3435669
3.655185241	88.08283992	777.2052307	7758.586688	-97.72400818
4.527570893	146.6843339	718.4938049	21516.29382	-272.0632172
4.532651462	146.5630637	757.0705566	21480.73165	-191.1750275
4.718268384	168.8352261	810.5613708	28505.33358	-240.7905975
3.996879751	100.8002954	593.6604919	10160.69956	-149.3182678
3.923344915	93.22320925	755.8337097	8690.566742	-156.4610596
4.390923102	131.263364	822.8706665	17230.07072	-165.8866852
4.235650195	117.4759869	750.1735229	13800.6075	-161.9196625
4.456777334	140.9330484	721.1475525	19862.12414	-184.6988525
3.955220167	96.33288614	524.1890717	9280.024953	-179.5742767
4.528300893	144.3995387	762.980072	20851.22677	-231.4890259
3.870696815	95.15611862	749.7713013	9054.686911	-121.7433815
4.22507499	118.452511	669.6266479	14030.99736	-207.4460114
4.527435361	151.2556892	708.5045776	22878.28352	-211.0576172
4.2573395	118.559105	821.190979	14056.26137	-209.7286774
4.094920489	107.0256237	671.4648743	11454.48414	-167.8151245
4.295088479	122.7593579	817.5071411	15069.85994	-135.5261658
4.531359295	143.3601946	746.8433533	20552.14539	-198.3170532
4.338634549	144.9343485	631.0399475	21005.96538	-163.6496857
4.481621264	145.0261462	710.9624786	21032.58307	-234.0645706
4.481791213	141.7702306	779.7246094	20098.79829	-217.8466599
4.394123646	135.9162319	789.7212524	18473.22211	-189.8152756
4.14921883	110.5307033	615.0953064	12217.03637	-155.0750565
4.25069917	125.4302409	745.3565063	15732.74534	-179.4320343
4.451334785	138.2442093	735.7441101	19111.46142	-236.372525
4.249237857	117.7258168	885.1416626	13859.36794	-171.4417831
4.655782397	160.9627162	839.867981	25908.996	-259.1124695
4.43962753	140.2411829	700.7987671	19667.58939	-240.8339249
4.698025121	162.5980973	797.4099426	26438.14125	-243.9658569
4.394577777	129.4514345	745.8124695	16757.6739	-178.2314194
3.84569611	93.78948019	536.4712524	8796.466593	-136.2487595
4.357384829	125.4592904	754.1933289	15740.03355	-158.4797211
4.382232584	138.4569671	645.5774536	19170.33173	-270.1211426
4.242983153	135.7223721	671.1645813	18420.56229	-202.899617
4.652537503	171.2160523	930.2956543	29314.93657	-260.262085
4.231140969	116.8335818	750.0402222	13650.08584	-159.9848175
4.304414121	143.8745759	555.3526306	20699.8936	-288.2090454
4.43693518	133.7882965	869.5190125	17899.30828	-186.5785828
4.181148299	115.1147027	602.7023926	13251.39478	-153.5373383
3.973972953	105.9462648	707.5714111	11224.61102	-143.9445892
4.649132035	156.8942148	831.875061	24615.79465	-263.3553833
4.670038814	158.6877736	841.1766968	25181.8095	-254.0559021
4.508918984	146.4956092	906.5549316	21460.96352	-226.8121643
4.400441201	130.3297382	669.1586914	16985.84065	-220.2604752
4.113362173	108.1988974	527.4324493	11707.00141	-179.9616089
4.503247101	147.5655257	755.098053	21775.58439	-213.1160339
4.527191851	142.2485197	954.6454773	20234.64136	-178.1676407
3.889544888	93.48136959	686.0903473	8738.766461	-111.4072472
3.908405661	96.64239052	653.8895569	9339.751645	-136.7035217
4.451973192	140.3618732	657.9875488	19701.45543	-242.1919434
4.421233604	134.03256	836.4795227	17964.72713	-127.2238815
4.559023114	157.5811202	810.8446655	24831.80943	-264.718457
4.597235881	154.5029833	748.1035767	23871.17184	-213.0100952
4.483051708	149.6831215	672.5337067	22405.03687	-320.0096283
4.415964645	133.7493648	714.5820923	17888.8926	-175.9135895
4.48002505	141.4573866	818.9916077	20010.19221	-188.0606384
4.586954963	153.7225575	740.240509	23630.62468	-252.0190857
4.168975433	113.3188383	734.9215393	12841.15911	-157.7968658
4.05238276	103.1757549	758.9482117	10645.2364	-158.2360992
4.063875789	108.7673438	770.2324524	11830.33509	-116.7214844
4.696256211	163.7635961	951.3606873	26818.51541	-271.0802002
4.381070789	131.8512122	795.1141357	17384.74215	-236.5665009
4.574062214	155.6160854	686.9174194	24216.36603	-270.4795624
4.493783581	142.4197424	741.5033264	20283.38302	-252.4194931
4.279084509	120.679207	766.7219238	14563.47101	-186.1628189
4.851646939	183.8353596	866.426178	33795.43943	-379.1902924
4.589435529	152.3396138	880.29422	23207.35792	-239.2363586
4.466245501	136.520005	886.3514099	18637.71176	-154.3680771
4.025605777	114.611631	578.4458771	13135.82596	-139.5726196
4.255376368	119.2503368	679.3154602	14220.64283	-141.9452667
4.03620969	103.5341289	705.8036804	10719.31585	-122.9258499
4.600652009	157.7163637	769.3416443	24874.45137	-249.2641769
4.735081601	173.9630267	913.3057251	30263.13464	-307.03078
3.999225366	110.8257831	528.7582245	12282.3542	-140.4846436
4.110284206	125.0253947	594.4839783	15631.34932	-217.5599152
4.409244955	132.5973287	864.5487061	17582.05159	-177.6056519
4.58488453	159.3288776	783.4346313	25385.69124	-286.4077301
4.063357665	104.7303121	542.3970032	10968.43828	-175.2456253
4.187220872	112.8607628	700.035614	12737.55177	-164.741037
4.125661819	109.524191	634.9303894	11995.54841	-153.4343109
4.875513523	211.8025537	877.9025269	44860.32176	-379.7275391
4.427795729	133.1694645	734.8115234	17734.10628	-167.1893005
4.268706531	133.7037711	548.9889526	17876.69839	-171.9687744
4.15670227	114.073665	572.3774719	13012.80105	-149.0078003
4.58032316	150.1503054	955.3337097	22545.11421	-242.3190887

log.sigma.4.0.mm.3D_firstorder_Kurtosis	log.sigma.4.0.mm.3D_firstorder_Mean	log.sigma.4.0.mm.3D_glrlm_ShortRunLowGrayLevelEmphasis	log.sigma.4.0.mm.3D_glrlm_GrayLevelVariance
2.79465753	-27.127173	0.008229888	38.49005505
3.319596072	-20.8601002	0.008670713	23.50676291
2.41957459	-63.46300718	0.023860159	22.71363499
2.543982093	20.47244795	0.004815308	31.62177345
2.481288118	-12.15424086	0.010354079	45.70685642
2.215810368	-79.37438722	0.014276381	21.26536319
2.914480477	5.603932098	0.027720795	15.32147869
2.415377363	-26.75055056	0.008697405	48.45052948
2.634542963	-33.38615653	0.005670519	41.85130777
3.077525365	-32.77552793	0.007205138	22.59368599
2.18208699	-53.60668418	0.014895527	25.63051657
2.855241094	-29.20564597	0.013287808	30.43572344
2.742515988	4.846730737	0.016936658	26.64345741
2.205562643	-3.025096	0.014885778	27.6860722
2.884905164	28.16284522	0.016352412	26.36010441
5.422902048	6.95081016	0.007192908	14.40629601
2.440515386	-54.52187019	0.012398001	34.61438619
2.71134127	-15.05153104	0.015561713	34.86599294
2.551491621	10.25792796	0.011816284	45.63479565
3.198106632	-14.77490984	0.021219929	17.01833095
3.43201008	-44.66084067	0.006828145	14.78556877
3.029625586	1.747367541	0.013597515	27.78533334
3.229852428	-27.23035732	0.009588743	22.55337274
2.6790277	15.17168181	0.007944898	32.30946464
2.82378377	-61.54737354	0.031035485	15.31071382
2.721622588	-48.18178531	0.016712645	34.07913683
4.215067756	-11.71972823	0.005482674	16.14007237
2.80737225	-60.9375269	0.01696587	22.89526154
1.996744064	-3.691119765	0.011125001	36.45858366
3.373670032	-57.93251943	0.008782432	23.89775861
3.17871702	-43.72569223	0.012357049	18.76116409
3.003026351	13.43871899	0.008126589	24.91258407
2.482249701	-6.097281415	0.01678937	33.38223046
2.260373938	27.95930413	0.020398113	33.1772725
2.738843183	-32.38182232	0.008537006	34.13410841
2.179607041	-24.69362897	0.008231841	32.29628573
2.768418391	-28.61163984	0.012805945	29.94971722
2.516091563	-2.717291677	0.016060404	19.78482351
3.578531467	6.122155454	0.013009545	25.68934833
2.623009062	-35.82796603	0.01214324	31.10222628
3.255389876	-23.98533478	0.006561904	23.01282052
2.386125311	-31.26879778	0.010054143	41.42112117
2.130726357	-40.66359391	0.010299845	31.40251144
2.519187103	-17.34580287	0.014286966	42.48056155
2.680084179	-12.93804172	0.006418124	27.36060251
2.853408059	-29.63025141	0.01730871	14.54702557
2.669763844	6.690399837	0.007620883	25.62858951
2.179073124	-72.36320061	0.023168395	30.38319395
3.192981624	-1.21306005	0.020147422	29.49210168
3.399262216	3.628143072	0.01052543	48.03833111
2.952614038	-12.05320419	0.009926046	22.6135285
1.723512362	-85.21548431	0.017303674	32.48186948
3.052556661	-15.39775699	0.008192825	29.52229063
2.79836314	0.138896196	0.020183265	21.11004163
3.728302065	-3.323408311	0.009772741	19.3485564
2.515050966	-43.79758393	0.013977206	39.82354739
2.240382377	-41.39142288	0.006062842	40.47890803
3.179778317	-14.65959088	0.005163113	35.14443609
2.626285724	-53.2358799	0.013270393	27.8187361
2.314726767	-29.35308468	0.017963017	18.56869326
2.38512098	-35.26585123	0.013018252	34.98055218
3.172741837	5.272391211	0.007300459	33.03969007
3.960711646	12.01215633	0.010026021	14.93473258
3.481241372	-19.8335755	0.00685025	16.21181769
2.55653384	-52.08518153	0.014338375	31.45401196
3.170368854	33.8122428	0.0090163	29.77901121
2.996810583	-39.52298268	0.015189234	39.97457123
2.444463598	-4.317599373	0.017751606	38.43660765
2.305105201	-90.16187477	0.028599679	35.65189917
2.731848727	-12.95140158	0.012277959	29.0540275
2.475376155	-18.02651998	0.007990996	32.38947655
2.230815959	-44.56033503	0.017026892	37.77244754
3.228869082	-15.77026505	0.011743887	21.39417696
3.034183146	-28.4072633	0.005266691	17.79222896
4.152579444	16.16857972	0.009442786	20.44271766
2.549493876	-49.08732042	0.009252959	43.15208336
2.557186575	-62.52838764	0.010291968	28.02456655
2.390693385	-64.04930228	0.038719395	38.64138028
2.266903768	-76.81785971	0.008986775	32.52825947
2.548726349	-40.01380336	0.007102116	23.59042225
2.307391216	-126.4730881	0.013607926	54.10922092
2.268194969	-27.24054928	0.007447966	37.13476154
2.865398813	23.49260769	0.005097807	30.62015956
3.259966062	16.64182645	0.015401176	21.89669996
2.658379638	3.43237154	0.01530874	23.11941472
3.462156202	-4.19542087	0.00616871	18.03939337
2.337372819	-46.11598866	0.013932582	39.71223307
2.436067425	-49.91342227	0.004696196	48.33909213
2.353140559	-9.645059977	0.014813934	20.01741951
2.86253744	-46.08442049	0.021607956	25.05872918
3.244357722	-9.154899205	0.006853505	29.75518057
2.433358083	-83.13304567	0.024561256	40.623329
2.683912174	-38.19555675	0.015660693	18.29031357
3.109928628	-25.10171148	0.01227235	21.00651772
2.773431224	-10.40498861	0.016349735	19.75191232
2.005088782	-75.48160927	0.017122378	72.18629379
2.78929107	10.46270285	0.007956844	29.2112956
2.342856652	7.02436639	0.032489027	28.49442624
2.861200095	17.86614169	0.020308146	21.10984112
3.436349475	-48.8666047	0.006529649	37.3738951

log.sigma.4.0.mm.3D_glrlm_LowGrayLevelRunEmphasis	log.sigma.4.0.mm.3D_glrlm_GrayLevelNonUniformityNormalized	log.sigma.4.0.mm.3D_glrlm_RunVariance	log.sigma.4.0.mm.3D_glrlm_GrayLevelNonUniformity
0.008611653	0.04663413	0.074054528	30.87771587
0.009414202	0.061433633	0.205222679	112.3849981
0.025498363	0.06012754	0.082892912	24.30898511
0.00505211	0.049213494	0.09574202	120.121483
0.010702479	0.044501736	0.074207428	15.07647984
0.015084227	0.060218313	0.08693781	35.99962387
0.0299633	0.076877503	0.1175601	23.42592849
0.009032186	0.040554748	0.061357407	38.48695989
0.005941152	0.043155472	0.087313345	83.76061389
0.007776737	0.061551556	0.128952581	172.5823096
0.015559983	0.058015668	0.085714323	44.79850093
0.014248604	0.051484874	0.099784131	70.26970324
0.018083199	0.055560374	0.124007098	104.7636118
0.016018028	0.053159947	0.085739587	55.32210801
0.017055958	0.056104802	0.095433112	47.36532584
0.008178219	0.099845086	0.553877338	549.6347817
0.012888679	0.048587384	0.066953884	32.03039648
0.016752414	0.048090406	0.115269428	103.0824113
0.012279403	0.043186925	0.070138436	23.71930966
0.02348963	0.071021989	0.248722288	173.223272
0.007516967	0.076416053	0.223073038	346.5308863
0.014457143	0.053891114	0.101980707	86.02768307
0.010294369	0.06275106	0.111979373	157.0963828
0.008216869	0.051344939	0.075356045	23.10511121
0.033563044	0.072934294	0.121465508	79.8341741
0.017571697	0.049146938	0.077591988	31.93986221
0.006103829	0.079920707	0.380608019	229.8421395
0.018409974	0.060057617	0.120442957	78.51495477
0.011613832	0.04668708	0.076742539	77.55293846
0.009479899	0.059975525	0.223740275	132.8449778
0.013415753	0.067610286	0.132909483	194.6848286
0.008826685	0.058433753	0.171740188	310.1896622
0.017482618	0.048024237	0.094587285	90.60449089
0.020694088	0.056956483	0.039821612	5.687645899
0.008740491	0.051795659	0.061282341	16.29385256
0.008623278	0.048863272	0.091916505	91.77816423
0.013720773	0.053245127	0.116138425	140.7758572
0.017467373	0.062564212	0.153166293	187.983864
0.013818745	0.064861871	0.121241162	49.47979853
0.012763886	0.051784685	0.091950055	50.66542292
0.007022676	0.061038576	0.145587187	80.60981441
0.010530595	0.044390859	0.071823831	39.83950258
0.010767732	0.050376572	0.080679696	77.61761481
0.014958321	0.043190185	0.076885727	41.56742334
0.006741092	0.053576146	0.098754879	93.10420075
0.019338683	0.077860662	0.178118055	153.0165659
0.008063901	0.05479297	0.109365189	214.8400138
0.024579509	0.053250911	0.083148404	28.97519187
0.020939662	0.062153679	0.058932064	7.85428699
0.01088127	0.050330635	0.071601683	23.56759929
0.010674071	0.059900184	0.164658505	184.0266894
0.018443606	0.05396447	0.086859069	41.69778647
0.00870158	0.052427463	0.132194053	182.5199818
0.023545292	0.063450856	0.111560589	34.26686703
0.010866316	0.078490855	0.293335614	437.1079572
0.014401293	0.044232469	0.06277579	36.87634087
0.006310567	0.042879621	0.068369239	88.72389873
0.005351159	0.05318401	0.089196084	64.0152643
0.014054526	0.053122062	0.095380967	39.94550991
0.01931423	0.063796965	0.120213606	51.6279829
0.013929071	0.048697704	0.097215087	95.75751102
0.007740693	0.049545019	0.126571442	247.9041581
0.010943202	0.080222985	0.228832428	216.7182585
0.007557731	0.077031868	0.29184361	178.4360619
0.014710074	0.051819584	0.052566099	10.70271433
0.009490897	0.053907464	0.115647367	76.95891464
0.016475036	0.05174295	0.064572999	19.27587455
0.01899418	0.045156476	0.109814886	133.3699951
0.030746337	0.050490261	0.060412235	21.34623567
0.012989056	0.052675539	0.091082463	98.4948758
0.008604679	0.050099789	0.112459868	139.8359817
0.018050051	0.045500985	0.078124974	48.16124524
0.012791759	0.063369966	0.178417091	291.7964305
0.005789182	0.068053459	0.218914388	399.4311887
0.010187744	0.072861592	0.243853185	579.2983895
0.009596118	0.043418859	0.06623692	45.80297383
0.010845708	0.052970114	0.088483813	55.08527752
0.041388717	0.045259932	0.067066683	26.14234841
0.009500445	0.048649971	0.093417652	79.62994927
0.007579326	0.05753304	0.101478078	150.6596248
0.013966228	0.038047182	0.049495131	18.37219152
0.00770416	0.045413108	0.060381591	57.66146068
0.005370748	0.052011184	0.108982576	133.4515061
0.015635805	0.075421927	0.071682401	10.31952996
0.016444811	0.058353162	0.11712362	181.2063411
0.006736579	0.071649506	0.199867349	344.4896162
0.014774663	0.045121665	0.071495926	33.12624858
0.004860876	0.042422781	0.058886197	62.59580079
0.015272749	0.070235254	0.091267945	11.90753741
0.022440534	0.067859835	0.05565645	6.749424373
0.007326984	0.054373952	0.189170139	192.8753265
0.026259456	0.045158485	0.06841039	30.06519718
0.016909654	0.06633662	0.16790958	59.90806019
0.01316979	0.062566575	0.174376416	228.9452827
0.017719507	0.064030835	0.190144225	329.9279537
0.017718793	0.037039927	0.046726128	8.981865269
0.008335764	0.05274515	0.100816374	59.20981007
0.034277366	0.055324353	0.054197772	6.489789156
0.021864462	0.064942097	0.09830458	22.75235701
0.006834391	0.048857922	0.099964049	36.37548425

log.sigma.4.0.mm.3D_glrlm_LongRunEmphasis	log.sigma.4.0.mm.3D_glrlm_ShortRunHighGrayLevelEmphasis	log.sigma.4.0.mm.3D_glrlm_RunLengthNonUniformity	log.sigma.4.0.mm.3D_glrlm_ShortRunEmphasis
1.212865333	298.6317307	586.8020644	0.953405023
1.500273251	197.0154262	1475.373742	0.916216247
1.239236716	139.7646637	353.753988	0.947980916
1.264002392	347.8650459	2120.752034	0.945524356
1.201305426	355.4491628	304.5066218	0.958442409
1.245179642	166.2190121	521.7281593	0.947660386
1.35103122	104.0862116	252.1680057	0.924751593
1.176943003	342.0703821	855.7429947	0.960453596
1.236251138	358.2506334	1712.01778	0.951691782
1.352402928	214.5855101	2338.096778	0.929803739
1.237268545	193.6191798	681.4921458	0.951057106
1.282307112	200.0967772	1170.848822	0.940581019
1.33822743	154.3597862	1587.993271	0.933189717
1.241265726	197.7849514	910.4927492	0.948612954
1.263487662	177.692851	733.4380527	0.945337854
2.179711409	179.0249427	3830.858459	0.857265226
1.191829045	257.9951039	592.6047157	0.958381815
1.301691596	196.4252243	1846.181703	0.942046369
1.195310518	351.5227882	493.2160789	0.958342681
1.575221204	108.4210496	1944.794673	0.911347737
1.556500291	188.5235332	3555.010728	0.905277941
1.269814222	178.3031206	1396.664413	0.947347673
1.305046158	195.128955	2135.556465	0.938434422
1.220796909	340.5092199	396.3657616	0.950952785
1.335159539	93.74696815	920.2420269	0.932619255
1.220819727	213.1444949	574.4803933	0.952294031
1.844910785	219.071334	2139.065942	0.884585768
1.328326043	142.5044292	1106.580832	0.934915894
1.209774053	258.2861296	1488.581338	0.95687952
1.547171241	206.0829227	1756.7685	0.909696225
1.356190471	151.8291562	2409.995677	0.93083199
1.425112172	210.7302226	4388.306431	0.925724353
1.257351315	224.521265	1645.439887	0.947385188
1.125876172	238.3711582	91.9581838	0.968530957
1.17685337	352.4355124	283.998664	0.960616898
1.250941175	259.6725341	1644.297832	0.948561884
1.306464421	176.8374712	2269.583266	0.940696991
1.403512059	139.6551948	2472.922619	0.924282254
1.332601552	256.323858	648.9229939	0.935126861
1.243997414	220.3026015	862.5912191	0.951302665
1.396628159	248.3504555	1082.950597	0.922890636
1.200392767	295.0632183	801.9788429	0.956967791
1.219156997	241.3660834	1373.007846	0.955223954
1.212733589	282.5413828	856.7091047	0.955228567
1.27029002	269.5309768	1507.912602	0.944895386
1.472675841	98.36724816	1576.688142	0.913076296
1.295156206	230.6464481	3361.777047	0.940696497
1.23398555	182.8886308	479.1323528	0.950460131
1.185679942	288.5205672	112.5465876	0.954947536
1.199393395	453.1630292	418.710761	0.957110023
1.410821654	181.6032721	2550.283251	0.927544753
1.236143497	195.0457345	684.4638662	0.952419059
1.341123965	239.3587882	2953.78577	0.936101919
1.311804141	168.8451027	457.8939382	0.936035396
1.682259498	159.2119639	4300.16001	0.89834729
1.175966239	278.348838	755.3547795	0.96192862
1.19446754	346.2651386	1855.943474	0.957647645
1.247866438	407.616045	1054.229738	0.948342764
1.269134654	199.4880625	648.0394695	0.9428117
1.312473476	140.6046384	698.1943189	0.941591113
1.259797167	202.2864146	1718.46824	0.948087238
1.32898241	264.0686601	4261.593289	0.937607754
1.559396715	146.9736134	2154.958655	0.909685985
1.693593674	185.7258784	1771.09137	0.894340856
1.157398911	261.3530455	187.5645431	0.963166706
1.304505433	239.6131576	1226.158283	0.940888152
1.186266776	351.6235938	335.1246023	0.958909684
1.283820773	209.0138791	2571.052141	0.946154875
1.175216739	221.3838592	382.1593724	0.960970734
1.249708563	191.4740909	1635.908035	0.948394472
1.302479295	242.3691293	2394.997583	0.940271989
1.217768926	219.6740326	939.7850517	0.953921216
1.44580497	154.0772662	3775.91618	0.922311537
1.534533294	237.1160987	4679.285336	0.911520351
1.585982085	176.7957262	6237.493024	0.905469851
1.187245198	338.6919011	949.8925615	0.959471049
1.245502113	215.4424517	912.4593934	0.949076582
1.183957358	179.0754744	522.2642098	0.961187179
1.258958229	260.9098033	1427.134457	0.946387453
1.284854685	235.5962556	2243.984827	0.940413116
1.140383328	352.8802339	445.9463184	0.969089122
1.176811318	327.5857707	1143.346365	0.959829127
1.296689626	315.9987719	2200.078925	0.94032096
1.200973423	239.7473874	122.9987367	0.957744751
1.312703973	152.5120167	2654.135634	0.938893091
1.502412084	208.6686521	3842.743608	0.912759179
1.196571926	222.729454	660.3552105	0.958936964
1.166438778	492.796312	1341.422057	0.963449495
1.264093597	184.3602444	146.941711	0.943380169
1.17244257	183.2013957	89.3838856	0.958671725
1.452651477	272.2491951	2929.511954	0.925252539
1.19433008	186.3897337	596.6051665	0.95774513
1.453623244	127.935911	722.4657759	0.913337867
1.430441386	165.9297865	3023.890533	0.925349715
1.464146468	133.9230253	4202.507211	0.920815388
1.136912286	437.7283016	223.4077616	0.968495008
1.276813609	296.7006126	969.2933738	0.943273601
1.155105421	186.7835589	107.2188011	0.965377967
1.272843041	183.1340509	302.8449622	0.943482812
1.278088246	331.9504644	641.4176625	0.942357982

log.sigma.4.0.mm.3D_glrlm_LongRunHighGrayLevelEmphasis	log.sigma.4.0.mm.3D_glrlm_RunPercentage	log.sigma.4.0.mm.3D_glrlm_LongRunLowGrayLevelEmphasis	log.sigma.4.0.mm.3D_glrlm_RunEntropy
365.2926317	0.937894966	0.010347889	4.934177213
305.2813446	0.880411837	0.013459738	4.818379988
172.2462097	0.931407302	0.032477875	4.543627972
461.6785708	0.926317326	0.006187748	4.896284464
446.072133	0.943432612	0.012223196	4.914516054
212.6834758	0.929776289	0.018848647	4.530221675
143.9922785	0.903903218	0.039600403	4.359490424
409.7635262	0.947105788	0.010499332	5.062539357
452.2791598	0.933547934	0.007221769	5.057635747
291.3665237	0.905025575	0.010612419	4.71934528
252.3028933	0.933680588	0.018690572	4.591407794
260.1112855	0.920896917	0.018510777	4.859687344
215.1844568	0.90913137	0.023709441	4.789796271
255.7320614	0.930851796	0.021029054	4.723294883
230.0348403	0.926623322	0.020433957	4.71696615
425.3696333	0.790324545	0.015627772	4.66330051
321.7860204	0.944346484	0.01515787	4.806443968
250.3069557	0.919118617	0.022597972	4.945676309
436.5203611	0.94369548	0.014240601	4.987594221
172.7992594	0.870458353	0.036809025	4.614027411
305.3541698	0.86790258	0.011376968	4.577266475
231.1593096	0.927906977	0.018599205	4.761655794
254.8478176	0.916570431	0.013682186	4.675033322
442.2955858	0.935391012	0.009389786	4.768974111
124.2333202	0.909085101	0.045695275	4.414551999
258.4004097	0.936266903	0.021245335	4.848550374
430.9452572	0.828953793	0.010395627	4.692646313
182.7652108	0.912072575	0.02563996	4.662852936
332.8928359	0.94112007	0.013774427	4.840446814
344.6419986	0.871058524	0.013259851	4.885225514
203.0155702	0.905703293	0.018679851	4.578854784
304.9881239	0.894992865	0.012648966	4.828070609
295.9256934	0.927957925	0.021424079	4.913109005
277.9318334	0.960059172	0.021877988	4.489041192
429.2782162	0.947405005	0.009602176	4.728936929
340.2211686	0.929779132	0.010471541	4.851847937
229.3305171	0.917895299	0.018320621	4.82429147
206.6147208	0.895784597	0.024778056	4.668562068
364.7032318	0.913035468	0.017706433	4.67886471
280.6435763	0.932609812	0.01575894	4.79656032
361.2403928	0.895731135	0.009330725	4.77182414
362.2455743	0.941722496	0.012675754	4.956597773
308.1601683	0.938302258	0.012956396	4.76599505
349.1170646	0.93891182	0.018339505	5.00716243
354.789223	0.925247809	0.00830677	4.789408376
138.6109835	0.881853335	0.030486259	4.423017235
311.9267238	0.919073894	0.010213787	4.790464988
231.0995991	0.933368518	0.031019307	4.696074954
352.5318905	0.943168772	0.024117838	4.460681254
570.3470889	0.942114224	0.012359391	4.947909791
263.2833802	0.897196262	0.014624547	4.75009302
250.7307072	0.93433169	0.023950516	4.627872098
327.6289473	0.910957663	0.011293686	4.900776404
229.3520312	0.913835741	0.037593769	4.589191205
278.513322	0.852156275	0.017574328	4.706066035
335.0033284	0.948455413	0.016317487	4.918746596
425.3045765	0.943561682	0.007422226	4.978471604
540.870323	0.930572907	0.006217044	4.877352338
255.8393559	0.923738424	0.017477968	4.778765919
189.3816047	0.918798568	0.026587329	4.493456254
250.0839474	0.927941337	0.018312859	4.880819171
366.5768346	0.913317606	0.009951269	4.982186249
239.5759261	0.87279064	0.016274619	4.519433247
336.9645877	0.847618511	0.011979739	4.633884682
307.5793657	0.951790145	0.016257638	4.66020589
318.9783382	0.918965773	0.011802833	4.841973546
422.34711	0.945333854	0.02167559	4.809806596
269.4725749	0.923798754	0.025054392	4.993790703
266.4526891	0.947913073	0.039625252	4.730167865
240.7264746	0.929770876	0.016295159	4.789242719
315.320613	0.918142713	0.011632931	4.901946193
269.0472039	0.937589426	0.022611933	4.90449047
225.2881077	0.890643606	0.018537505	4.721368231
378.937689	0.873791634	0.008837223	4.666916632
297.8784498	0.865384615	0.014540137	4.749104304
413.3077325	0.945271574	0.011130623	4.98473547
273.9127363	0.930996488	0.013433485	4.733994542
208.5320406	0.946910467	0.055670737	4.832214245
332.7347648	0.927917321	0.011879337	4.866985088
308.4916116	0.920130818	0.009831295	4.699482433
403.3803596	0.958180708	0.015490638	5.050314684
398.5213588	0.94688235	0.008829337	4.878655319
429.1242565	0.918861583	0.006706342	4.897947267
302.1880558	0.943236074	0.016704425	4.265520685
201.3478619	0.915795205	0.02202233	4.693231241
324.5460284	0.87790077	0.009957172	4.654974116
263.9305459	0.943642476	0.018729256	4.879166484
595.7417697	0.950683981	0.005586625	5.00418369
240.9724703	0.926019336	0.017397939	4.33404764
212.8677252	0.947252747	0.025801189	4.31973354
410.8070784	0.891785196	0.009963138	4.961418318
218.405415	0.943015908	0.034105302	4.873995227
197.6085444	0.883486377	0.023131112	4.6237243
249.8817067	0.894451759	0.017767088	4.719506664
204.2938601	0.887318159	0.024975821	4.689548072
515.1953407	0.958346002	0.020134293	5.049655615
401.7834751	0.923076923	0.010107268	4.82332021
218.0272012	0.953721076	0.041534131	4.437269054
251.6215047	0.924294703	0.028336118	4.509632825
429.2049336	0.922409684	0.008249588	4.97335608

log.sigma.4.0.mm.3D_glrlm_HighGrayLevelRunEmphasis	log.sigma.4.0.mm.3D_glrlm_RunLengthNonUniformityNormalized	log.sigma.4.0.mm.3D_glszm_GrayLevelVariance
310.7656337	0.885337868	43.27203108
212.3942612	0.805387382	31.49724171
145.3370946	0.873556524	25.06406157
367.4687225	0.867738864	35.2766684
370.7200429	0.897653451	47.90347633
174.4141162	0.872052561	20.81224446
110.9947404	0.824097384	19.56358025
354.3735999	0.901362719	53.20064872
374.2571854	0.881512865	47.51467477
227.581201	0.832967535	28.8225476
203.5482477	0.880860205	26.93805228
210.5689859	0.856605581	36.17139468
164.4656983	0.840765446	32.3287311
207.8658759	0.874280017	26.87515598
186.8562875	0.867471817	33.77268502
206.0670441	0.693691201	27.92086712
269.2106177	0.897577428	36.07708223
205.2581117	0.860327028	36.72827412
366.2488623	0.897029078	48.11942649
117.0246714	0.795948809	21.73013076
205.434339	0.782567357	21.79427641
187.0344673	0.872928307	31.6229437
205.3681282	0.852026961	26.54033783
359.0278037	0.879848663	37.44185604
98.95227051	0.839421082	19.17565712
221.1780967	0.883031995	38.30597052
244.4930695	0.742836977	28.36159449
149.3519364	0.844751493	24.34418114
270.656528	0.894535111	35.50771162
225.1678426	0.792016611	33.37348819
160.3243304	0.835615594	21.03013992
224.9901903	0.825286055	28.0280569
236.6988507	0.871628742	38.71326527
246.2832932	0.920356775	36.15338843
366.1453179	0.902179847	41.73515556
273.4302125	0.874573928	34.80093143
185.5121271	0.857363489	33.04703858
150.3331435	0.821728606	22.64037174
273.6549617	0.84718357	34.98291081
230.3243209	0.880877379	35.37860662
266.9188866	0.818668781	32.33971033
306.9662943	0.893247705	40.57186486
252.7069309	0.890123135	32.97109922
294.1840149	0.889597652	44.25412727
284.013212	0.866510422	33.5642406
104.7610755	0.799551664	15.47465597
244.3241113	0.856697757	31.69724863
191.0422604	0.879208385	28.98835805
300.6636865	0.889106948	31.40628099
473.6178871	0.893732291	62.63501179
193.8529693	0.828973114	27.90678639
204.3897982	0.884276434	25.49810654
253.4356271	0.847329248	35.29115849
178.9527443	0.846748757	25.31482094
174.5586889	0.77058933	26.01583512
288.53738	0.905331208	44.33086842
360.3394389	0.895626212	41.58305887
430.4174849	0.87456018	44.91021785
209.3063976	0.861109941	29.99879437
148.4333703	0.860226879	16.72282705
210.3673508	0.873453858	35.88979699
280.5437186	0.850563097	37.87719895
159.8831763	0.794274082	21.94684488
205.4918934	0.762495325	24.86699493
270.136715	0.907639123	33
252.5821301	0.857664847	40.21851895
363.9490844	0.898550127	45.14699781
218.8168758	0.86969144	38.74609857
229.3942974	0.903082278	33.60912904
199.9195581	0.874114587	32.23069197
254.6734799	0.856586263	33.39383233
228.2734503	0.886865923	37.92638943
164.6910617	0.818320367	25.98298771
257.0636086	0.795777866	21.27686207
193.8579172	0.783412169	33.21529948
352.0399044	0.899606512	42.87113706
225.4429376	0.876089258	30.09574639
184.1704065	0.903526028	35.27648672
273.3099827	0.870193572	33.80591716
248.3047951	0.85601606	26.20898616
361.9414016	0.922620538	54.30406956
340.8701944	0.899898104	38.26111507
335.0629345	0.856338018	38.83914688
250.5919298	0.897073405	29.625
160.6447432	0.853410017	24.494919
226.1245608	0.797886257	26.61253294
229.9725276	0.898613459	37.02691756
511.2109652	0.90864528	47.45206226
194.3366875	0.864299748	26.77365602
188.7868103	0.897489434	26.58380165
291.7773639	0.824684184	39.46659388
192.1020232	0.895456796	39.03811873
139.1824078	0.798693176	22.33856529
178.0622222	0.824713752	26.75346415
143.9577648	0.814601165	23.96933281
452.0393225	0.920712919	71.89402367
314.5800799	0.862586526	37.45710784
192.3536819	0.91332894	28.58984375
194.7583086	0.863107031	25.56855867
348.4778581	0.86059315	50.51480792

log.sigma.4.0.mm.3D_glszm_SmallAreaHighGrayLevelEmphasis	log.sigma.4.0.mm.3D_glszm_GrayLevelNonUniformityNormalized	log.sigma.4.0.mm.3D_glszm_SizeZoneNonUniformityNormalized
236.476787	0.043458579	0.329158493
169.6353365	0.05002551	0.319936224
116.6266921	0.057108864	0.318188578
213.6746773	0.046478039	0.309781641
235.7356104	0.041584484	0.379355687
113.0324226	0.063926447	0.264683613
78.57862163	0.064691358	0.23654321
261.948689	0.038285236	0.344796317
262.4376287	0.040096267	0.341949237
182.3528306	0.051981451	0.319111981
105.4548033	0.055374892	0.268899741
150.9790386	0.046519634	0.303819048
103.6817982	0.048757241	0.225750732
118.5462639	0.054009332	0.271728516
119.0524096	0.048316918	0.295518373
146.2922711	0.053644806	0.294527084
184.7850471	0.04679703	0.353361191
163.1017081	0.045332424	0.306997058
235.8956058	0.041650896	0.336008115
98.06560824	0.060781649	0.286118782
155.6392489	0.0610631	0.289567901
133.2515151	0.050103801	0.281076619
159.2577238	0.056186801	0.305792688
206.2200051	0.045520523	0.344533016
89.9498861	0.064005348	0.360507155
190.4019267	0.046325638	0.333207585
174.612602	0.054035857	0.280829446
132.807568	0.056549209	0.344471256
163.0634538	0.045752906	0.358271363
171.1569279	0.048623703	0.298155935
127.1306663	0.062810146	0.318559583
157.917709	0.052664614	0.297189422
143.158873	0.044516674	0.304336652
167.5487071	0.049256198	0.397024793
228.1326219	0.045511111	0.327644444
162.6439792	0.046749075	0.30058875
155.4761564	0.048354341	0.338885605
89.56504163	0.057766384	0.247537254
163.5055933	0.051091025	0.251218134
163.8937087	0.047368293	0.348382635
189.7177484	0.051921492	0.294389671
205.0928906	0.044166272	0.31817818
152.9247938	0.048250207	0.317100779
203.0355347	0.042216033	0.336290739
178.8126653	0.04796488	0.289064586
90.51415247	0.070646295	0.293860044
155.536679	0.04855611	0.289805862
122.8115426	0.05299277	0.324311549
173.1463895	0.055206612	0.283305785
305.4604412	0.037688881	0.378548345
133.2963908	0.053149811	0.27278828
115.9310254	0.055946269	0.330869969
176.4128986	0.046952392	0.320816791
118.3665482	0.059320478	0.264352617
128.9081286	0.055595872	0.298810364
194.6875405	0.04186369	0.35854042
246.5702225	0.042809203	0.361484067
238.9707691	0.043419471	0.319061249
155.5957046	0.052107446	0.329282407
82.08403719	0.071047241	0.231722069
166.3412796	0.046743232	0.324425447
182.4312888	0.046377168	0.327968556
110.6208445	0.060467156	0.28562128
138.440916	0.056042771	0.269739526
180.5626466	0.051097394	0.365397805
191.2412552	0.044616575	0.328996283
313.2246893	0.045434624	0.431877283
159.8492968	0.044237308	0.339394911
153.340744	0.048920728	0.350362899
155.559969	0.04873275	0.3310456
186.4008418	0.047488906	0.318651422
164.5129931	0.045722526	0.318253851
134.5372105	0.055416001	0.304168138
181.6750358	0.060859524	0.275908211
125.0788552	0.049261387	0.29188958
247.7467868	0.043778941	0.362404167
146.7590582	0.052498701	0.293519962
150.33972	0.047461312	0.361860152
175.8692935	0.047597633	0.317723077
171.91897	0.054343727	0.293082113
291.7270957	0.038548753	0.429483825
216.5470887	0.045261263	0.38101242
210.4152808	0.044860216	0.308020077
134.418117	0.055664063	0.354003906
114.9374881	0.056194	0.27839
156.4859848	0.053799889	0.278187678
172.6663918	0.045795948	0.354964823
310.6947376	0.040830341	0.362247633
139.5440648	0.060900615	0.387026384
148.6571517	0.061157025	0.387107438
204.7575753	0.043876729	0.306702661
165.9916578	0.045371016	0.375163373
93.3379844	0.059570248	0.311709091
117.3002086	0.0537764	0.275643509
104.5792473	0.056866137	0.277008928
259.8147014	0.034556213	0.360118343
177.2222979	0.045259035	0.29495867
143.9665877	0.058105469	0.411132813
87.36181341	0.060746173	0.231664541
299.9746999	0.040856561	0.390626191

log.sigma.4.0.mm.3D_glszm_SizeZoneNonUniformity	log.sigma.4.0.mm.3D_glszm_GrayLevelNonUniformity	log.sigma.4.0.mm.3D_glszm_LargeAreaEmphasis	log.sigma.4.0.mm.3D_glszm_ZoneVariance
92.82269504	12.25531915	15.14893617	8.881193099
179.1642857	28.01428571	148.0946429	134.3384917
52.18292683	9.365853659	17.15853659	10.15541344
293.6729958	44.06118143	27.27109705	19.54527742
59.17948718	6.487179487	11.73717949	6.441280408
56.90697674	13.74418605	20.02325581	11.07898323
21.28888889	5.822222222	30.45555556	16.43469136
154.46875	17.15178571	10.43303571	5.430624203
282.7920193	33.15961306	17.98186215	11.66214037
280.4994312	45.69169511	72.6370876	60.21527203
79.32542373	16.33559322	17.50169492	9.642700373
149.1751527	22.84114053	29.81670061	20.70638499
114.4556213	24.7199211	59.26627219	42.53220203
104.34375	20.73958333	20.02083333	11.54424371
93.97484277	15.36477987	22.08490566	13.87794193
275.6773504	50.21153846	3379.206197	3324.119986
110.955414	14.69426752	9.694267516	4.752850014
233.9317585	34.54330709	45.34645669	35.98060774
86.3540856	10.70428016	9.774319066	4.645944677
167.3794872	35.55726496	197.6974359	174.7721645
312.7333333	65.94814815	440.0333333	416.636406
161.3379791	28.75958188	23.67595819	14.69685197
265.1222607	48.71395617	64.70703576	54.78489907
70.62926829	9.331707317	11.0195122	5.514193932
134.4691689	23.87399464	40.22520107	29.80597862
93.96453901	13.06382979	15.21276596	9.156279865
172.1484502	33.12398042	672.0783034	640.1087796
160.5236052	26.35193133	41.26824034	31.81195546
276.5854922	35.32124352	14.2888601	9.061832197
192.0124224	31.3136646	185.4037267	169.8232996
292.7562568	57.72252448	110.0446137	98.07858521
486.4990837	86.21197312	83.80696396	70.68460155
209.6879536	30.67198839	30.5065312	21.80017737
21.83636364	2.709090909	5.490909091	1.915371901
49.14666667	6.826666667	8.893333333	3.994488889
209.2097701	32.53735632	23.12931034	14.70597173
310.419214	44.29257642	45.51091703	35.62552202
195.5544304	45.63544304	113.1392405	95.11437911
59.78991597	12.15966387	36.86134454	24.55245039
141.4433498	19.23152709	20.81034483	14.13461258
106.5690608	18.79558011	104.5524862	87.97274198
128.2258065	17.79900744	13.70471464	8.112604597
203.8958009	31.02488336	15.59564541	9.074492864
141.9146919	17.81516588	12.81279621	6.913192651
182.9778831	30.36176935	27.21958926	18.41754079
151.6317829	36.45348837	190.7906977	172.1470465
377.6170376	63.2686109	43.67920184	32.9602364
77.51046025	12.66527197	14.23012552	8.27979202
15.58181818	3.036363636	9.381818182	3.445950413
81.00934579	8.065420561	17.31308411	11.91940344
250.9652174	48.89782609	90.52391304	76.67257089
103.5623003	17.51118211	25.15335463	18.17227899
405.1916073	59.30087094	44.56136184	35.4087009
43.61818182	9.787878788	44.39393939	31.56451791
400.704698	74.55406413	936.6122297	912.951006
146.6430318	17.12224939	8.37408313	3.755262104
348.8321244	41.31088083	10.79274611	5.628313243
158.5734406	21.57947686	18.54124748	11.77287467
94.83333333	15.00694444	29.14583333	21.15735918
57.69879518	17.69076305	38.10040161	25.58187771
242.9946595	35.01068091	40.84779706	32.84395928
592.639181	83.80354178	49.19092418	40.00066519
191.9375	40.63392857	265.6547619	244.5111873
141.3435115	29.36641221	421.7270992	394.5639058
39.46296296	5.518518519	7.064814815	3.027692044
177	24.00371747	35.47769517	27.14513688
79.8972973	8.405405405	9.037837838	4.502089116
389.2859634	50.7401918	34.00784656	26.23896614
72.17475728	10.0776699	8.504854369	3.817419172
244.3116531	35.96476965	23.05826558	15.63300982
339.0451128	50.52819549	30.09398496	21.93071966
140.986456	20.25507901	15.48758465	8.992565567
399.0685976	72.70579268	135.4588415	119.9429283
395.1005587	87.15083799	252.1382682	230.1426922
598.665529	101.0351048	1053.099951	1033.06658
180.8396794	21.84569138	11.10220441	6.100393171
117.9950249	21.10447761	20.86318408	13.14252741
100.5971223	13.1942446	10.13669065	5.321981264
206.52	30.93846154	23.79076923	16.42580828
266.9978046	49.50713502	33.01866081	23.25903791
117.2490842	10.52380952	5.648351648	2.240067625
242.3238994	28.78616352	8.495283019	4.049550552
293.851153	42.7966457	41.93710692	33.37197456
22.65625	3.5625	11.078125	5.945068359
278.39	56.194	43.785	32.286119
318.5248908	61.60087336	345.4777293	322.6051097
119.6231454	15.43323442	14.67062315	9.340964524
267.3387534	30.13279133	8.905149051	4.48261984
27.47887324	4.323943662	16.4084507	9.765125967
21.29090909	3.363636364	5.945454545	2.300826446
362.8292477	51.90617075	147.4429417	136.15266
119.677116	14.47335423	13.10344828	8.205343894
85.72	16.38181818	73.6	59.78864132
291.0795455	56.78787879	123.4090909	108.4081404
382.8263386	78.58900145	263.5383502	245.8886238
46.81538462	4.492307692	5.807692308	2.020177515
120.3431373	18.46568627	25.73529412	16.85255671
26.3125	3.71875	6.328125	2.634521484
25.94642857	6.803571429	28.59821429	17.14724171
118.359736	12.37953795	30.58415842	23.49063817

log.sigma.4.0.mm.3D_glszm_ZonePercentage	log.sigma.4.0.mm.3D_glszm_LargeAreaLowGrayLevelEmphasis	log.sigma.4.0.mm.3D_glszm_LargeAreaHighGrayLevelEmphasis	log.sigma.4.0.mm.3D_glszm_HighGrayLevelZoneEmphasis
0.399433428	0.147531509	3528.06383	366.1241135
0.269619644	0.927406294	27941.63929	268.1625
0.377880184	0.411082062	1804.713415	179.945122
0.359772296	0.107350836	9547.746835	371.1170886
0.43454039	0.085391992	4295.871795	371.2115385
0.33437014	0.341996095	2720.306977	201.1209302
0.267062315	0.697150451	2738.177778	139.3444444
0.447105788	0.095667244	2971.424107	396.0290179
0.397787398	0.091501651	5535.748489	412.5695284
0.28373144	0.563125191	11766.57565	289.9078498
0.356711004	0.262731882	3123.59661	205.6813559
0.331309042	0.377399194	4901.04277	249.2362525
0.244455159	1.05288576	6871.412229	198.0118343
0.343470483	0.34517863	3761.882813	221.4921875
0.349066959	0.293593928	3361.320755	208.1289308
0.134734418	19.24098949	606779.4241	251.5128205
0.449856734	0.11423565	2561.436306	282.9872611
0.326758148	0.820182251	5088.463255	266.2388451
0.441580756	0.089663388	3470.610895	382.5680934
0.208853981	4.029400821	17659.91453	162.6290598
0.206738132	2.878983226	72237.21944	262.4462963
0.33372093	0.353767045	3478.972125	223.6916376
0.31746613	0.575649745	8992.611303	257.1107266
0.426195426	0.05562017	4239.107317	346.8585366
0.309800664	1.121643477	2810.922252	130.3565684
0.406340058	0.210999206	2390.776596	275.8156028
0.176860935	3.376921712	140509.1941	308.3915171
0.325191905	0.782330139	3781.263948	200.5042918
0.437393768	0.108073307	4898.702073	260.7409326
0.253343824	1.000134521	40819.78106	278.6071429
0.289084618	1.5449069	9573.652884	205.6528836
0.276053963	0.69306014	13776.82407	267.3982896
0.338908018	0.315147934	6352.592163	251.8563135
0.528846154	0.068324895	1350.018182	250.9090909
0.451807229	0.046528849	3282.86	377.1133333
0.344554455	0.159380986	6205.409483	282.591954
0.318055556	0.677594063	5110.472707	238.6375546
0.235539654	1.545721765	15434.45949	172.0987342
0.28502994	0.279417719	10195.20588	291.5210084
0.387035272	0.259177264	4110.719212	256.1133005
0.245590231	0.555252783	23101.98343	321.058011
0.422875131	0.164383373	3102.590571	334.8263027
0.391595615	0.138916629	3838.791602	255.7371695
0.411707317	0.191745234	3114.559242	325.4123223
0.337060703	0.153083686	6939.676145	312.5545024
0.231597846	4.332550364	10809.43023	152.9457364
0.30543835	0.285225305	9534.893323	271.5310821
0.409948542	0.405059928	2050.506276	205.3556485
0.410447761	0.128759297	2657.036364	310.6
0.430583501	0.082181826	8122.453271	469.9579439
0.268691589	0.837054884	13807.04022	234.8913043
0.378476421	0.725700614	3419.408946	205.9840256
0.330541743	0.315562504	9213.350752	292.3056215
0.279187817	0.819427601	6595.727273	210.769697
0.205580254	6.594258758	146847.3535	217.2841163
0.465301479	0.092505026	2171.095355	305.99511
0.44003648	0.06338439	3499.262176	386.6569948
0.384377417	0.060651398	8235.955734	422.7706237
0.353808354	0.334617727	4283.409722	253.9583333
0.282633371	0.856171052	4226.052209	169.0321285
0.353468617	0.753301196	3842.196262	263.7022697
0.329864914	0.287276271	12019.29275	301.4947427
0.217475728	2.144289493	38657.99405	193.3541667
0.191871109	2.429122674	79752.60305	252.3931298
0.497695853	0.072816859	1657.861111	289
0.346426272	0.221180489	7812.315985	298.7825279
0.469543147	0.145800049	3007.383784	413.9945946
0.35877385	0.608592514	5569.219704	256.8683522
0.461883408	0.470017066	1663.815534	243.5776699
0.366981601	0.286860234	3360.189702	240.2859079
0.35	0.264824365	5490.619361	302.3045113
0.39238264	0.285174882	2541.065463	265.234763
0.253869969	1.574220861	18152.77058	217.1051829
0.213222156	1.340641824	54637.76816	320.6731844
0.223420479	6.374934799	179780.6704	220.8425158
0.447132616	0.082448447	3691.59519	378.5170341
0.359892569	0.204018887	3818.487562	250.2711443
0.455737705	0.753295237	1172.935252	222.3165468
0.368480726	0.194305972	4865.8	296.7446154
0.320098384	0.246259209	6119.456641	288.1086718
0.541666667	0.069604312	1629.604396	405.3516484
0.474272931	0.055518411	2641.092767	347.6006289
0.341690544	0.168834521	12852.93291	358.7389937
0.44137931	0.076174466	2840.046875	239.875
0.29489826	0.726571021	4589.99	199.819
0.209094229	2.098253927	61245.86725	277.9388646
0.433161954	0.345119827	1805.448071	272.4718101
0.475515464	0.034866161	4743.142276	506.2127371
0.387978142	0.160576231	2521.619718	216.1267606
0.523809524	0.111072967	915.3818182	213.5272727
0.297610063	0.633652624	38956.80135	337.7075232
0.45184136	0.655100003	1305.877743	238.1222571
0.269080235	0.900024805	8578.050909	158.9309091
0.258190709	1.125529362	19180.9858	208.5179924
0.238029625	2.766003482	36439.59407	181.1432706
0.513833992	0.092362971	2690.692308	438.4692308
0.335526316	0.133724504	7994.296569	318.0196078
0.520325203	0.213213025	1037.265625	209.15625
0.295514512	0.371584843	5871.839286	186.7678571
0.375464684	0.150751256	8267.722772	424.5478548

log.sigma.4.0.mm.3D_glszm_SmallAreaEmphasis	log.sigma.4.0.mm.3D_glszm_LowGrayLevelZoneEmphasis	log.sigma.4.0.mm.3D_glszm_ZoneEntropy	log.sigma.4.0.mm.3D_glszm_SmallAreaLowGrayLevelEmphasis
0.589614988	0.009902073	6.358317603	0.007233219
0.584129297	0.009969859	6.513554188	0.006320709
0.576982408	0.021942258	5.858369651	0.009118672
0.571681278	0.005920803	6.723906659	0.003844457
0.638251671	0.01413093	5.959074517	0.0113241
0.524854648	0.016303129	6.092691153	0.010862939
0.492701783	0.031376253	5.632030008	0.011089803
0.605080155	0.010115278	6.567479665	0.006926989
0.602780602	0.006359834	6.738937522	0.004002969
0.581259699	0.00771953	6.544072855	0.004499622
0.525164291	0.018690413	6.341489741	0.010645429
0.567150969	0.012990746	6.610396335	0.006370412
0.479574682	0.021660902	6.865394887	0.011154808
0.526468434	0.013351699	6.392639629	0.005330245
0.556218302	0.023198242	6.427882046	0.01644531
0.559355263	0.010206948	6.716961115	0.005156589
0.612479958	0.013417544	6.216805041	0.008528241
0.565503623	0.012206244	6.663323042	0.004792618
0.596475818	0.012678988	6.390029316	0.005897323
0.55225279	0.019486335	6.476325496	0.009666126
0.551993345	0.008529356	6.549649001	0.00517156
0.542075262	0.014085536	6.66150356	0.007858708
0.567790585	0.010241767	6.505405719	0.006156199
0.601899527	0.011284468	6.153118088	0.008281285
0.622897582	0.031381495	5.930015845	0.016032474
0.594471435	0.017347406	6.251473567	0.008320967
0.541683525	0.007851536	6.601321496	0.005144716
0.605032001	0.016850119	6.111214287	0.01018521
0.617783384	0.012736693	6.433028866	0.008148968
0.55661303	0.010503903	6.60498163	0.005171322
0.582989131	0.012612326	6.380766131	0.007209189
0.561015626	0.008490194	6.777198993	0.004762546
0.565307836	0.019746962	6.704333326	0.009629167
0.647858586	0.030356335	5.113089032	0.027213616
0.579197469	0.012921121	5.952711338	0.010625078
0.563247264	0.010413294	6.675201243	0.006532014
0.603833552	0.011699115	6.603993963	0.006571133
0.504640569	0.01810215	6.724132595	0.009554112
0.504851031	0.017222756	6.437363079	0.008249067
0.610240913	0.013666811	6.336794135	0.009417683
0.557612013	0.009400785	6.40887452	0.006547319
0.576072985	0.009878115	6.450281641	0.004244375
0.578561855	0.012036787	6.50199508	0.007034761
0.597702955	0.013533507	6.495085832	0.005095853
0.548786809	0.008037216	6.682750511	0.005215927
0.553891399	0.01441259	6.119573098	0.007980585
0.553541807	0.008942281	6.864718714	0.005199557
0.582523728	0.023253806	6.070930916	0.011268149
0.537338229	0.023615568	5.317545623	0.007232063
0.637396041	0.012425375	6.28692734	0.005775936
0.533484524	0.011581728	6.768671688	0.006885176
0.590522384	0.015695376	6.073130915	0.008018121
0.581489783	0.009377756	6.707373737	0.005484382
0.524953298	0.018512901	6.03060216	0.005740407
0.563360731	0.010120803	6.639417396	0.005295325
0.618999908	0.017479224	6.405938457	0.011866055
0.622320953	0.00695394	6.579750282	0.004883959
0.574605909	0.00762034	6.623851864	0.005778774
0.579682603	0.014061718	6.086550893	0.005994952
0.472588926	0.016687593	6.181825417	0.006485733
0.58762767	0.01292393	6.565558439	0.008001654
0.592491237	0.008633018	6.833862578	0.005367352
0.548026064	0.011966734	6.505791895	0.007170892
0.528499305	0.009409974	6.536951324	0.006145681
0.609885467	0.018507377	5.669400933	0.013423068
0.588711524	0.011320684	6.53680676	0.006816334
0.681106974	0.012173602	5.923881313	0.004847486
0.602221479	0.015070357	6.668666192	0.006833198
0.610445988	0.016693575	5.947258007	0.007151637
0.593136427	0.01291803	6.506466596	0.007161206
0.580579012	0.007683124	6.647551526	0.004296276
0.578433521	0.016716463	6.476220836	0.008593677
0.569320521	0.010596696	6.665607042	0.004909007
0.538474584	0.005323491	6.664645905	0.002445852
0.554306928	0.014487463	6.909241159	0.009489855
0.621530945	0.010237465	6.383032242	0.005676456
0.550612517	0.012237679	6.39021238	0.007748507
0.619154741	0.023214296	6.080322918	0.009701195
0.580336985	0.0094664	6.535744757	0.004799681
0.557305692	0.007290394	6.622053513	0.003398804
0.680800488	0.016292763	6.151306982	0.012858987
0.639309878	0.009546783	6.337326204	0.007597957
0.568875141	0.006445928	6.773494781	0.004327232
0.598176709	0.027054699	5.378524414	0.024396677
0.540410764	0.013892503	6.648384783	0.005429862
0.540426484	0.007415221	6.78696575	0.004346589
0.612356936	0.01281919	6.223483097	0.008328209
0.619898451	0.005576548	6.507822191	0.003990849
0.645459204	0.025557343	5.16046781	0.023200392
0.63787232	0.028091659	5.085638577	0.02273268
0.570019196	0.007719214	6.849350282	0.003965789
0.631056179	0.019457947	6.158781101	0.010862624
0.577433832	0.019494734	6.129476982	0.012222473
0.536231626	0.014896661	6.739959574	0.007516261
0.538842014	0.01781743	6.71998094	0.008606046
0.609600362	0.016144012	6.008616229	0.006517392
0.5562465	0.00910357	6.561640119	0.003973658
0.660563862	0.02815937	4.991729297	0.009572814
0.455002266	0.024872696	5.841167427	0.008976783
0.647390552	0.00899259	6.30579736	0.006942142

log.sigma.4.0.mm.3D_ngtdm_Coarseness	log.sigma.4.0.mm.3D_ngtdm_Complexity	log.sigma.4.0.mm.3D_ngtdm_Strength	log.sigma.4.0.mm.3D_ngtdm_Busyness	log.sigma.4.0.mm.3D_ngtdm_Contrast
0.016223154	1.381964469	5.821439201	0.122296674	0.00021605
0.006495857	0.285539441	1.79129878	0.360102542	4.10E-05
0.023608935	0.934409832	4.230294007	0.201314925	0.000357672
0.004352027	0.401062089	1.701382992	0.343783734	4.19E-05
0.031595393	2.205883808	10.05456452	0.065925641	0.00060927
0.015611613	0.587618147	2.471446909	0.258059239	0.000248553
0.035267286	0.589486649	4.134540203	0.172185344	0.000327894
0.010113982	1.435597143	4.508467282	0.162947485	0.000196261
0.005526673	0.568687453	2.218494825	0.306629605	7.47E-05
0.003789293	0.217641101	1.222188327	0.584417055	2.63E-05
0.009907854	0.661994103	2.100362555	0.309874886	0.000231871
0.008605539	0.471098844	2.629619016	0.318738966	8.16E-05
0.005823139	0.230811323	1.434694632	0.678932484	6.09E-05
0.009648576	0.483683911	2.229531551	0.304464877	0.000136711
0.012655466	0.571679641	2.701782316	0.264871681	0.000156765
0.002129222	0.065707153	0.929102025	0.759818285	3.79E-06
0.014511237	1.110298558	4.109121276	0.158447878	0.000261284
0.005070915	0.326208797	1.609749713	0.638715322	6.49E-05
0.019171181	1.802707613	6.771574075	0.088221983	0.000339858
0.004448735	0.132683055	0.972579492	0.882860443	2.77E-05
0.002345902	0.102929633	0.77121666	0.850038647	8.56E-06
0.006250136	0.510590677	2.463810162	0.406118498	6.48E-05
0.004278457	0.244950674	1.440101445	0.540563091	2.99E-05
0.022712455	1.376531628	6.393534671	0.08712279	0.000335126
0.008123445	0.26892746	1.302556164	0.706704956	9.27E-05
0.015745929	1.245227293	4.434501434	0.179064289	0.000253039
0.003964464	0.141808623	1.39921749	0.425608901	1.10E-05
0.007611289	0.3407014	1.753320747	0.551793307	8.89E-05
0.00517051	0.446150101	1.43785829	0.483325057	0.00012474
0.005306818	0.288517015	1.942102972	0.352252071	2.74E-05
0.003356427	0.173116814	0.946927769	0.900436198	2.61E-05
0.002089742	0.129655017	0.830536314	0.98475805	1.29E-05
0.005787185	0.370675995	1.739916071	0.427058797	7.10E-05
0.067677664	6.364342355	15.09628645	0.047528643	0.002963373
0.026711905	2.363851183	7.868318497	0.073295798	0.00060949
0.005289722	0.429274636	1.796237467	0.386920355	6.71E-05
0.003698865	0.305503892	1.464535332	0.782638409	4.18E-05
0.003477646	0.115840344	0.707438771	1.054878022	3.05E-05
0.011783088	0.874734539	4.075067897	0.14705305	0.00012403
0.010199647	0.781251952	3.099294113	0.227551028	0.00013792
0.008865769	0.506062223	3.286687753	0.194524119	4.69E-05
0.010852112	1.059475926	3.813552312	0.194414456	0.000200691
0.005762081	0.454816277	1.586230765	0.411779859	0.000106558
0.010587498	0.959692712	3.535725417	0.210267711	0.000191623
0.005885987	0.404180652	1.758280891	0.352887869	6.18E-05
0.004524664	0.149488162	0.905646644	1.144857273	3.74E-05
0.002762577	0.167920412	0.833106707	0.793160449	2.37E-05
0.015460317	1.092022848	3.827214771	0.200834271	0.000339923
0.056775998	4.319934033	11.30269907	0.049992634	0.002079799
0.021309027	2.6766117	10.82081509	0.053597656	0.000325022
0.003552149	0.193739183	1.223131962	0.657882087	2.26E-05
0.008906929	0.621833682	1.772849779	0.440485509	0.000388107
0.003027608	0.272154986	1.291334381	0.609739039	2.56E-05
0.01891536	0.688842846	3.597146942	0.168074885	0.000205045
0.002116976	0.07508274	0.605932099	1.148138421	9.63E-06
0.012289578	1.173335687	4.045967141	0.171167421	0.000211656
0.004589327	0.576883976	1.726861007	0.382240322	8.16E-05
0.007988344	0.943904441	3.835089089	0.143905466	8.92E-05
0.014646513	0.674226575	3.431166811	0.216979919	0.000168234
0.011768345	0.346382351	1.750181455	0.388581828	0.000155488
0.004755031	0.40852247	1.584299232	0.650897765	8.07E-05
0.002199773	0.239392252	1.285058772	0.676478115	1.57E-05
0.00404888	0.161106463	1.296251721	0.577838137	1.52E-05
0.004612382	0.155471466	1.05518581	0.518521571	2.22E-05
0.046249846	3.06652207	10.91986219	0.055346068	0.000876398
0.008122809	0.531753094	2.793007451	0.260210261	6.72E-05
0.024028214	2.646018379	8.808028829	0.066373295	0.000483551
0.003462864	0.263971369	1.058696647	0.863682025	5.89E-05
0.020624938	1.433380543	4.932965379	0.13973524	0.000550118
0.005638724	0.355424324	1.60894499	0.535867109	6.71E-05
0.003610271	0.309051986	1.325046147	0.633689862	4.26E-05
0.008742303	0.799318992	2.783089745	0.312974833	0.000174991
0.002268779	0.115247401	0.671460759	1.266573808	1.67E-05
0.001869419	0.097612388	0.668068099	0.925703621	7.62E-06
0.001423792	0.068407432	0.562012545	1.404944861	5.98E-06
0.009085719	1.272862047	4.106238682	0.168834953	0.000152136
0.008931389	0.770814666	3.001088886	0.257192596	0.000115548
0.016155552	1.1866458	4.003746948	0.264514918	0.000425741
0.005690262	0.498880682	1.680771911	0.440220517	9.30E-05
0.003831421	0.260041697	1.242233782	0.576884269	3.28E-05
0.019002417	2.746879542	6.922938357	0.102714515	0.000565594
0.007381417	0.86420674	2.823226574	0.232686062	0.000121913
0.004580245	0.363563999	1.870537894	0.326186303	3.26E-05
0.05274372	3.127504404	10.6350611	0.051339779	0.001257165
0.003294109	0.171387335	0.873536769	1.050310504	3.26E-05
0.002285582	0.098453471	0.710774047	0.902751554	1.04E-05
0.013023239	1.250273252	4.504001124	0.222731876	0.000250977
0.006042041	0.927713321	2.805318512	0.209119429	0.000136317
0.049785903	1.682790403	7.561967728	0.0830638	0.00091464
0.065004431	4.713103322	13.0650688	0.057507637	0.002259385
0.003293881	0.23106838	1.284817237	0.494290703	2.22E-05
0.013258331	1.469844873	4.686957473	0.262266492	0.000332132
0.012093479	0.300022368	1.836829047	0.375719606	0.000104625
0.002836281	0.141818455	0.810469712	0.930300624	2.08E-05
0.001983524	0.075791836	0.450432676	1.787351481	1.65E-05
0.033177035	5.670835066	14.09835203	0.051809637	0.001675732
0.010271464	0.488344822	2.506006766	0.214121825	0.000110748
0.064346227	3.46022292	8.998535481	0.090251841	0.002920531
0.030088718	0.890346606	5.596548248	0.104027645	0.000314713
0.014625241	1.642632266	7.350450692	0.094447638	0.000134517

log.sigma.3.5.mm.3D_gldm_GrayLevelVariance	log.sigma.3.5.mm.3D_gldm_HighGrayLevelEmphasis	log.sigma.3.5.mm.3D_gldm_GrayLevelNonUniformityNormalized	log.sigma.3.5.mm.3D_gldm_DependenceEntropy
35.17070998	309.9036827	0.048559895	6.618006701
22.27008465	212.9147809	0.065663807	7.065927067
24.19718512	135.4170507	0.058039032	6.168095354
32.30743984	332.6948767	0.048621354	6.91222443
43.26670339	339.3175487	0.04632956	6.489785798
20.2656434	161.7387247	0.061257858	6.345732431
13.91075029	123.5074184	0.084855903	6.000722798
46.08855841	354.999002	0.04210541	6.606719629
40.8679641	374.4617605	0.043736802	6.911815907
21.43649405	199.475468	0.065012684	6.820880008
23.91007254	240.0036276	0.060379074	6.251540257
29.09601134	212.7881242	0.053143343	6.842665149
24.40925487	162.0019286	0.058600815	6.76379458
27.09780755	183.0572451	0.053568057	6.511787376
24.55794467	205.7277717	0.057464506	6.52697949
12.35181242	201.0611775	0.121429647	7.083655347
34.06821783	251.7363897	0.048628501	6.448693804
32.24820364	176.0321612	0.050534145	6.93578363
45.04190137	298.9020619	0.042837236	6.543160835
15.8495845	116.7225991	0.07449971	6.980212219
14.25791655	238.9249617	0.079363724	6.90348486
27.77157382	188.1767442	0.05430503	6.644482036
22.07344053	205.8044672	0.064161416	6.718413919
31.01760452	393.2827443	0.053712596	6.462891305
15.61266156	122.6079734	0.07535099	6.456204446
33.28647568	222.6066282	0.050199736	6.574348509
14.56829637	273.9864397	0.093006367	7.042919755
22.34829113	176.6071179	0.063073627	6.620663869
37.22324262	279.5943343	0.048025745	6.652659451
22.08634422	233.4901652	0.065247199	7.149653807
18.24084189	212.2991507	0.069843869	6.684400505
22.88514812	190.3758853	0.062635185	7.084212129
32.38921543	239.7038859	0.048440041	6.871889634
37.25591716	296.9615385	0.058431953	5.369635603
36.85733597	344.2680723	0.05247496	6.254957605
30.73189001	278.1554455	0.050238212	6.759683151
29.54177035	162.3513889	0.055365789	6.867299122
19.35616352	174.5569469	0.063442968	6.849003549
25.06043817	276.4443114	0.064207394	6.545655858
29.65901703	206.8102955	0.052819836	6.615649911
21.84348932	238.2367707	0.062296449	6.896859573
38.88012341	308.2119622	0.046184303	6.605162558
30.79755423	260.7021924	0.051518379	6.563372116
40.51262439	293.9590244	0.044424033	6.718333625
26.04806764	314.3785942	0.054851251	6.710154178
13.8717101	85.79398564	0.080585675	6.539509055
25.85851077	243.6319737	0.054374486	6.823852116
29.24936671	179.0703259	0.052973177	6.409406797
35.10113611	408.6865672	0.065159278	5.423162071
44.44033213	431.5875252	0.053896822	6.393066932
21.5045882	192.4617407	0.061798861	6.916089212
32.36521627	194.2116082	0.057422627	6.37850366
28.19990711	283.1457734	0.053738548	7.117395364
20.21782462	177.5059222	0.065440147	6.365440282
17.93388519	148.1379733	0.0860257	7.113484673
39.42111796	267.2627986	0.044391897	6.578234259
43.12969738	372.4537164	0.041900721	6.775652124
34.43132484	362.9574633	0.053639414	6.717516876
26.49794445	216.7616708	0.055013915	6.639544011
17.46142102	150.3779796	0.066191164	6.438804917
33.53236153	182.9740444	0.051250055	6.745825096
31.89298216	279.8088719	0.050597758	7.137413473
13.91065657	182.2381877	0.084888931	6.852108307
14.94332602	234.6580007	0.084039778	7.010622046
30.10367602	277.6221198	0.053197137	6.064060844
29.77621445	280.3857051	0.055017209	6.825679107
36.81633126	326.8527919	0.053917906	6.383556459
37.2079083	214.7716609	0.045928165	7.041563633
32.07382513	213.3565022	0.053469806	6.157947717
29.4699664	201.1307807	0.052990254	6.793622097
31.31254317	252.7641447	0.052003982	6.944538698
35.35211248	202.720992	0.047909176	6.694281986
20.37297662	162.8306889	0.066155428	6.992496375
17.03969353	257.0451161	0.070539946	7.044658584
18.75249733	247.5527233	0.078907044	7.113718081
42.27802508	360.0501792	0.0432163	6.707777275
27.93478663	236.0483438	0.053656801	6.589048119
35.90098361	161.3409836	0.049782317	6.394806354
32.66164047	284.1825397	0.049689944	6.726102404
24.30541318	224.6184118	0.057632885	6.755867706
51.67393786	348.3472222	0.040084089	6.565244448
40.78045645	353.1551081	0.043787929	6.635557194
30.06112211	333.7217049	0.052592446	6.962823884
26.75414982	356.4896552	0.073293698	5.448249263
22.61011467	157.2686523	0.05918115	6.775964244
16.92866258	224.1130387	0.076030688	6.999339273
37.08339391	203.785347	0.046500486	6.511842323
46.73198533	442.2087629	0.043739704	6.67246101
18.31682045	195.8032787	0.071097972	5.831419189
26.39038549	233.4761905	0.062857143	5.34269058
27.91677962	290.3730818	0.058667743	7.174122268
40.92769383	175.5665722	0.046714122	6.45579125
18.501124	146.7534247	0.068529532	6.86237971
20.19574632	181.0699267	0.064435172	7.014653706
18.63682482	165.7328626	0.065942213	6.930544688
74.0394632	435.3399209	0.037760315	6.32016817
27.20004112	318.3289474	0.055549093	6.732263748
33.3864763	247.5447154	0.055192015	5.577364887
20.76402977	195.8548813	0.066951636	6.212454173
35.23297848	352.929368	0.051929446	6.774467989

log.sigma.3.5.mm.3D_gldm_DependenceNonUniformity	log.sigma.3.5.mm.3D_gldm_GrayLevelNonUniformity	log.sigma.3.5.mm.3D_gldm_SmallDependenceEmphasis
152.6033994	34.28328612	0.363608291
278.1324025	136.3837265	0.245705768
108.3963134	25.18894009	0.344147286
542.3753321	128.1172676	0.310163958
81.67409471	16.63231198	0.357170697
133.6096423	39.38880249	0.293175115
61.04747774	28.59643917	0.215947302
259.8522954	42.18962076	0.415237009
456.3631554	90.92881193	0.333305197
508.2834087	201.4092963	0.254239494
196.6324063	49.93349456	0.303315836
279.6518219	78.75843455	0.292513767
389.4744455	121.5380906	0.228742827
252.5313059	59.88908766	0.281951946
189.4895719	52.35016465	0.282550373
565.1292644	843.5717576	0.137961502
169.7048711	33.94269341	0.350526058
432.5574614	117.8456261	0.275708627
147.3264605	24.93127148	0.352495704
357.1727954	208.673688	0.19811082
683.8300153	414.5960949	0.203224092
365.7790698	93.40465116	0.301045835
495.4372025	175.2248261	0.277758325
103.02079	25.83575884	0.346187658
201.8770764	90.72259136	0.267322059
152.0057637	34.83861671	0.318817521
375.7051356	322.3600692	0.173036626
248.9413817	90.38450803	0.306623702
413.4147309	84.76543909	0.361224785
316.7254131	165.8583792	0.239932939
514.080843	222.0336584	0.255922277
926.3848229	371.4266442	0.238300744
422.2267585	98.47860305	0.29550506
33.78846154	6.076923077	0.404898504
84.48795181	17.42168675	0.404650217
429.5207921	101.4811881	0.277042613
508.5104167	159.4534722	0.284938267
552.9260584	212.7877162	0.233635639
162.4275449	53.61317365	0.260427413
226.6110582	55.40800763	0.319785529
231.6295794	91.82496608	0.241018538
221.6883526	44.01364113	0.35036207
373.4835566	84.59317905	0.329677294
243.7629268	45.53463415	0.325342311
389.1203408	103.0106496	0.29582374
327.7459605	179.5448833	0.212469953
846.1678387	231.9615565	0.289016005
130.4373928	30.88336192	0.338677926
37.88059701	8.731343284	0.385029022
118.7746479	26.78672032	0.341719606
578.6261682	211.5992991	0.233992298
163.5610641	47.4885127	0.306499249
643.0921225	205.3349908	0.282925361
114.2318105	38.6751269	0.241597958
710.4122336	561.1456385	0.19114371
225.6075085	39.02047782	0.371942484
539.6402189	91.88828089	0.403912101
273.6728538	69.35576179	0.330915672
157.9287469	44.78132678	0.313225831
166.2576617	58.31441544	0.229925062
423.2057574	108.5988674	0.296350098
995.2665206	277.1745162	0.291419829
402.2899676	262.3067961	0.198812234
298.7495423	229.5126327	0.176330748
53.21198157	11.5437788	0.411340246
306.7617514	85.44172569	0.300654323
89.80203046	21.24365482	0.36300865
614.3637785	146.8323428	0.30759618
111.0403587	23.84753363	0.358330245
407.0387867	106.5634013	0.322451906
563.9848684	158.0921053	0.297795421
241.8733392	54.0894597	0.319816944
743.8235294	341.8912539	0.231822235
873.9690292	473.7462775	0.205261825
1145.182571	724.3666667	0.207897457
274.390681	48.22939068	0.362918394
242.0241719	59.93464637	0.302279936
138.2721311	30.36721311	0.356149189
376.478458	87.65306122	0.327532662
541.0077301	164.0231904	0.287061954
126.0952381	20.20238095	0.40206537
346.9000746	58.71961223	0.404106363
525.3531519	146.8381089	0.307232896
39.33103448	10.62758621	0.401308429
615.5644353	200.6832793	0.269540178
743.6764061	416.3440467	0.204936995
185.5526992	36.17737789	0.361979648
395.9858247	67.88402062	0.383079612
35.20765027	13.01092896	0.311474891
33.68571429	6.6	0.511507937
612.8616352	233.2042767	0.255564499
169.4050992	32.98016997	0.38933504
152.5733855	70.037182	0.246721601
625.608313	263.5398533	0.229017983
850.6424389	382.8604891	0.213237105
72.8972332	9.553359684	0.455733421
241.1463816	67.54769737	0.293249009
35.34146341	6.788617886	0.438263324
80.68865435	25.37467018	0.253162709
148.9727385	41.9070632	0.332541526

log.sigma.3.5.mm.3D_gldm_DependenceNonUniformityNormalized	log.sigma.3.5.mm.3D_gldm_DependenceVariance	log.sigma.3.5.mm.3D_gldm_LargeDependenceEmphasis
0.216152124	2.394168961	9.424929178
0.133910642	9.539639619	26.76793452
0.249761091	1.378135021	7.479262673
0.205835041	2.526119203	10.60379507
0.227504442	1.718375866	8.21448468
0.207791046	2.123488024	10.30326594
0.181149786	2.482737367	13.70623145
0.259333628	1.325325397	6.502994012
0.219510897	2.051414995	9.201058201
0.16406824	4.043697156	15.6714009
0.237765908	1.504299423	8.484885127
0.188698935	2.694999463	11.71120108
0.187789029	2.842592636	13.44744455
0.225877733	1.739891385	9.438282648
0.208001725	2.484055711	10.96487377
0.081348678	20.57175071	60.53591478
0.243130188	1.450472492	7.650429799
0.185487762	3.489272969	13.34219554
0.253138248	1.373106128	7.309278351
0.127516171	9.88980653	29.11995716
0.130901611	8.292881365	26.17036753
0.21266225	2.033904813	9.834883721
0.181412377	3.676210631	13.74771146
0.214180437	2.514036506	9.852390852
0.16767199	3.961589828	15.04318937
0.219028478	2.183100931	9.631123919
0.108397327	19.0780491	47.38315061
0.173720434	3.524639285	13.33775297
0.234229309	1.905558668	8.322379603
0.124596937	12.09825924	31.52478363
0.161711495	4.132354302	15.89084618
0.156220038	8.120925127	22.59426644
0.207686551	2.259582119	10.39596655
0.324889053	0.721523669	5.115384615
0.254481783	1.504681376	6.939759036
0.212634055	2.106951279	10.39108911
0.176566117	3.582978395	13.68125
0.164855712	4.351315938	16.653548
0.194524006	2.740673384	12.23592814
0.216025794	2.694154222	10.45853194
0.157143541	5.058436693	17.95793758
0.232621566	1.566919068	7.977964323
0.22745649	1.710563601	8.549330085
0.23781749	1.569012731	8.207804878
0.207199329	2.464237554	10.7199148
0.147103214	5.290761614	19.94165171
0.19835158	2.593683656	11.28691983
0.223734808	2.080979379	9.085763293
0.282691022	1.055023391	6.134328358
0.238983195	1.552720751	7.969818913
0.168991287	7.283291322	20.41121495
0.197776377	2.419970494	10.75090689
0.168304664	5.914683116	17.43130071
0.193285635	2.229620277	12.03891709
0.10890882	10.6564961	33.81526905
0.256663832	1.55044322	7.307167235
0.246073971	1.59144224	7.198814409
0.211657273	2.377255362	9.962103635
0.19401566	3.163073728	11.92874693
0.188714713	2.405539572	12.77525539
0.199719565	2.621368811	11.15195847
0.181684286	4.586242211	14.80102227
0.130190928	8.457739655	26.77281553
0.109391996	11.70998883	36.17099963
0.245216505	1.510034191	7.055299539
0.197528494	3.208847798	12.0856407
0.227923935	1.675616481	8.065989848
0.192168839	3.240252704	12.13919299
0.248969414	1.841883006	8.058295964
0.202406159	2.796243733	10.92342118
0.185521338	3.466152614	12.86118421
0.214236793	2.073820001	9.559787422
0.143928702	6.717792842	21.64357585
0.130132375	7.956643057	25.6777844
0.124747557	9.540732624	28.96405229
0.245869786	1.383355173	7.297491039
0.216673386	1.903102456	9.501342883
0.226675625	2.3093362	9.137704918
0.213423162	2.180104997	9.646258503
0.190094072	2.889064752	12.03724526
0.250188964	1.686255984	7.317460317
0.258687602	1.387215902	6.700223714
0.188163736	3.442167962	12.58022923
0.271248514	1.422401902	6.696551724
0.181528881	3.102945756	13.03538779
0.135806502	7.860566408	24.91161432
0.238499613	1.943213434	8.341902314
0.255145506	1.371291782	6.926546392
0.192391532	2.438412613	10.8579235
0.320816327	0.82122449	4.485714286
0.154179028	9.133912994	23.70037736
0.239950565	1.603254982	7.456090652
0.149289027	11.29592411	27.61056751
0.152960468	8.732463101	23.79217604
0.146510926	5.368906861	20.03651395
0.288131357	1.130903467	5.56916996
0.198311169	2.716315357	11.42269737
0.287328971	1.017648225	5.520325203
0.212898824	2.495039717	11.68601583
0.184600667	3.099167907	11.89219331

log.sigma.3.5.mm.3D_gldm_LargeDependenceLowGrayLevelEmphasis	log.sigma.3.5.mm.3D_gldm_SmallDependenceHighGrayLevelEmphasis	log.sigma.3.5.mm.3D_gldm_LargeDependenceHighGrayLevelEmphasis
0.071775952	133.238745	2249.818697
0.182789964	64.40244749	5131.246028
0.286654002	57.47169741	794.0852535
0.0520411	101.6229609	3525.811385
0.078065048	123.6847839	2856.626741
0.167631983	53.31904457	1402.121306
0.26301756	34.8878771	1421.305638
0.050366964	161.9521433	2102.707585
0.046399888	135.0823282	3145.317941
0.161187299	64.99072611	2456.23725
0.068901903	74.31162394	1974.19468
0.137747261	72.98264226	2016.255061
0.20012321	42.46493012	1932.256509
0.177423131	54.92482102	1695.284436
0.103537638	64.605497	1987.183315
0.367781934	33.77295484	10956.41428
0.104733975	88.5802883	1902.919771
0.339854734	62.38712349	1588.818611
0.127887953	109.8542096	2134.128866
0.563841059	32.24403958	2851.71296
0.140720701	57.97421865	5588.390505
0.152700598	63.59331392	1579.301163
0.142060466	71.74577119	2164.755767
0.037610557	128.6498282	4158.334719
0.255984776	43.76098674	1347.564784
0.128157603	91.87506458	1629.727666
0.19863573	56.12085017	12117.18205
0.173807741	70.30579551	1673.381717
0.069513165	94.79144297	2543.087819
0.173461066	70.24307749	7448.90716
0.128649316	68.50924018	2572.298522
0.243672928	55.2826149	3446.484486
0.103593382	73.01312917	2434.332514
0.109177141	122.9057826	1493.019231
0.045449687	140.3449411	2288.231928
0.069048772	77.95882127	2881.111881
0.269219771	60.08483277	1717.701389
0.186329671	45.35326917	2673.774597
0.087870784	74.13305221	3484.116168
0.166260248	77.36810637	1871.906578
0.120114395	64.53623061	3923.293758
0.06763822	119.4705616	2289.643232
0.069809068	87.61044456	2295.523143
0.077410615	108.0704938	2227.800976
0.050493657	99.46818816	3255.865282
0.652388893	26.2482874	1119.156643
0.084288469	78.19197697	2530.822316
0.26873246	72.33983755	1229.715266
0.078573961	152.5479063	2650.641791
0.061291816	149.7479339	3429.832998
0.18346526	53.46233334	3369.783002
0.254712659	61.50013174	1958.927449
0.098237338	91.42028244	4336.846375
0.20402989	48.8773707	2039.553299
0.391345249	35.30108674	4398.555266
0.104303406	108.1643556	1680.62116
0.045446431	162.4592823	2479.763338
0.058884604	119.3724477	3738.980665
0.117576312	78.81921356	2126.609337
0.207119246	37.42670883	1667.442679
0.239982151	70.43346347	1455.688532
0.088126819	90.47187314	3795.347755
0.197709283	42.91765928	4420.734628
0.181525004	47.25742369	8167.882461
0.086250884	125.053702	1670.009217
0.071395814	93.23988688	3073.489375
0.130596531	134.4050684	2532.040609
0.227837267	78.61133374	2088.842352
0.208934549	89.98476651	1303.289238
0.144535523	78.69005211	1719.760318
0.115657608	89.01961812	2651.371382
0.260713756	75.31879348	1563.11426
0.244698741	49.94270058	2988.919698
0.138399186	63.71713264	5870.361674
0.144250977	57.13827784	6748.914597
0.045874355	132.8786762	2565.675627
0.083473217	74.35701171	2084.041182
0.49592834	70.56135821	916.0688525
0.086908787	104.6263544	2303.43424
0.102295124	73.48767568	2307.880534
0.07823535	167.4124718	1883.573413
0.042014425	145.2627716	2411.50261
0.056457354	108.2969829	3993.797636
0.035920537	142.4933563	2305.951724
0.234686496	51.46835068	1672.537305
0.152832402	55.63681414	4809.869065
0.187203623	89.79350234	1178.90617
0.043356714	171.8805515	3007.609536
0.09454325	68.40349547	1887.661202
0.05566321	133.8933862	902.5428571
0.117889291	86.37571755	6288.450063
0.315896966	88.95713296	915.674221
0.294936896	39.38387909	3788.178082
0.200049038	47.46171255	4289.244988
0.212556406	42.5280675	3009.393903
0.089476149	206.9741557	2506.13834
0.06260995	90.59896266	3731.504934
0.235513978	120.3873916	1099.252033
0.188120391	48.66967592	2338.804749
0.05690658	148.1998307	3459.112763

log.sigma.3.5.mm.3D_gldm_SmallDependenceLowGrayLevelEmphasis	log.sigma.3.5.mm.3D_gldm_LowGrayLevelEmphasis	log.sigma.3.5.mm.3D_gldzm_DistanceZoneVariabilityNormalized
0.003356741	0.007603323	0.911860493
0.002947226	0.009274318	0.899794989
0.008568765	0.032551415	0.909750693
0.002322818	0.006202386	0.872420133
0.005485719	0.011717917	0.879292404
0.004973748	0.015678815	0.876574356
0.004600861	0.022894342	0.76446281
0.00373519	0.00779416	0.833227756
0.002104406	0.005550236	0.921699292
0.002036428	0.010053257	0.96771591
0.003659879	0.009182352	0.865329107
0.003506835	0.01145552	0.954048443
0.004720909	0.016638007	0.9278125
0.004754954	0.019238857	0.903630601
0.004085083	0.011444766	0.91090925
0.001058155	0.007382688	0.934836521
0.004576714	0.012864341	0.82106048
0.00368748	0.020973385	0.951560511
0.006559999	0.016634733	0.85947462
0.003127521	0.020521801	0.971297955
0.001263493	0.005713197	0.947196643
0.004573857	0.014243102	0.86963927
0.002379843	0.009121002	0.948200845
0.004416515	0.007105109	0.68119601
0.004524863	0.017319582	0.953989748
0.003839367	0.014065571	0.943709305
0.001118576	0.004974195	0.926349283
0.00324184	0.011303558	0.961479229
0.004323728	0.010134379	0.93960533
0.001964489	0.007893961	0.950308565
0.001768432	0.007392199	0.97328342
0.002615758	0.011929254	0.96583237
0.004193365	0.012957028	0.934629871
0.00742545	0.025421053	0.767011834
0.00642288	0.010592794	0.813165719
0.002622684	0.007671546	0.848030185
0.005168138	0.020364665	0.954663604
0.003370707	0.012194089	0.942603432
0.004531899	0.010644805	0.791056241
0.00507701	0.015048273	0.956855536
0.002415932	0.008180411	0.92654405
0.00300839	0.009180662	0.875269581
0.003564116	0.009453693	0.904174733
0.004013826	0.010655996	0.835075452
0.00215949	0.005656345	0.8972994
0.004609081	0.028648557	0.981885572
0.002536351	0.008082625	0.877970071
0.007050556	0.023155143	0.933412604
0.005636722	0.018892208	0.644970414
0.004676893	0.009741534	0.738617004
0.002276182	0.010293409	0.93957495
0.004599314	0.019663163	0.935762298
0.001919213	0.006312121	0.923257682
0.004277934	0.016122912	0.87962108
0.002809371	0.014765556	0.951904697
0.007335571	0.014909479	0.841031163
0.002972337	0.00660032	0.757043819
0.004508772	0.009262127	0.713113064
0.004078739	0.011244331	0.905891579
0.004656307	0.015612283	0.991379472
0.004871964	0.018917681	0.935885709
0.002209016	0.007098034	0.939084688
0.001926941	0.008552284	0.939971807
0.00145545	0.006121212	0.952582758
0.00716632	0.012762001	0.946942149
0.002457147	0.007647019	0.932057132
0.004111461	0.01651459	0.778007952
0.004291976	0.016256365	0.952489981
0.006167588	0.017786145	0.921699292
0.004636788	0.014208958	0.93474785
0.001964071	0.008403149	0.89844946
0.004658647	0.021205563	0.921013358
0.002441885	0.012173685	0.945268152
0.001109609	0.00553965	0.953660201
0.00161741	0.006297718	0.947268307
0.003382743	0.007308789	0.821938411
0.004700539	0.010888308	0.851319204
0.008567084	0.036775361	0.890878192
0.003789613	0.010495684	0.855536199
0.002459957	0.008445653	0.915225026
0.008671324	0.013757316	0.827050364
0.004034738	0.007876605	0.862539027
0.002023174	0.005467185	0.914826333
0.009206762	0.011891652	0.686728395
0.004055979	0.016488473	0.97761481
0.001487044	0.006666735	0.772434638
0.004384146	0.017691604	0.927274878
0.002632161	0.006090905	0.806284991
0.008475413	0.013887042	0.805175781
0.013899081	0.018468863	0.8828125
0.001848485	0.007026016	0.912395729
0.009471357	0.036856129	0.947406866
0.00706625	0.019951242	0.895061728
0.003445405	0.012205303	0.927438928
0.002429912	0.011456013	0.957379246
0.005022073	0.016138735	0.695874955
0.002691967	0.008067414	0.950032873
0.0079031	0.031302359	0.834710744
0.005858654	0.015928141	0.787752675
0.002930073	0.00630586	0.907625601

log.sigma.3.5.mm.3D_gldzm_LowIntensityEmphasis	log.sigma.3.5.mm.3D_gldzm_LargeDistanceEmphasis	log.sigma.3.5.mm.3D_gldzm_HighIntensitySmallDistanceEmphasis
0.008809599	1.138613861	357.9348185
0.011681384	1.158703072	255.7086177
0.02685716	1.142105263	157.7973684
0.007519548	1.205438066	315.183283
0.015160548	1.193548387	333.4532258
0.016878	1.198237885	178.215859
0.027842759	1.409090909	155.6789773
0.008622948	1.275449102	371.9416168
0.006251321	1.12244898	394.9651861
0.008968191	1.049234136	258.3498359
0.011436666	1.217821782	240.7574257
0.011998724	1.070588235	253.072549
0.021122711	1.1125	185.9214286
0.018759553	1.152284264	192.7652284
0.014011847	1.140186916	227.8123053
0.008547041	1.101156069	247.7723988
0.013036678	1.298013245	244.1374172
0.014019846	1.074509804	231.6686275
0.0163004	1.228136882	304.8346008
0.016728882	1.04368932	166.7144013
0.006059366	1.081414474	291.7263569
0.015210527	1.210280374	205.0673676
0.008568995	1.079822616	260.6757206
0.011550606	1.597014925	343.9067164
0.017195831	1.070680628	161.2859948
0.013331016	1.086956522	280.298913
0.006254998	1.114874816	326.2290133
0.010324767	1.058939096	231.8659136
0.011698225	1.093506494	262.3175325
0.008595289	1.076487252	288.2528329
0.00681893	1.040625	269.2015625
0.010890611	1.052158273	232.532524
0.015131762	1.101488498	245.800406
0.025750822	1.403846154	300.0769231
0.014405704	1.312883436	336.0460123
0.009212462	1.248546512	269.8055959
0.01998219	1.069620253	208.9538502
0.014504777	1.08871851	196.4153888
0.01686535	1.355555556	265.5564815
0.015700803	1.066176471	235.6360294
0.010184087	1.114558473	267.0059666
0.009419127	1.20049505	330.3508663
0.010750947	1.151376147	261.1479358
0.012253414	1.272058824	321.8829657
0.007067662	1.162895928	327.8050528
0.021203307	1.027422303	128.7198355
0.008901028	1.195827725	260.910498
0.021769526	1.103448276	210.6411638
0.020168365	1.692307692	376.5230769
0.013239565	1.463768116	412.1799517
0.010298726	1.093555094	231.549896
0.015910039	1.099667774	205.0398671
0.006729278	1.119905956	319.0815047
0.018006689	1.192982456	198.2266082
0.014949272	1.073966642	188.3078318
0.018624339	1.26119403	278.4235075
0.007167023	1.424501425	380.115622
0.013625912	1.520710059	335.8313609
0.012592432	1.148514851	254.4966997
0.018950105	1.012987013	172.6017316
0.017803284	1.099469496	234.846817
0.007669331	1.094339623	301.5078616
0.009909888	1.092920354	216.3101032
0.008065098	1.072897196	272.9
0.015878672	1.081818182	299.1931818
0.008766246	1.105633803	309.1698944
0.015471344	1.38150289	345.8468208
0.013313013	1.073043478	256.9219565
0.016570922	1.12244898	244.5459184
0.015358259	1.101298701	238.286039
0.007226296	1.160962072	296.1327475
0.01596473	1.123569794	230.9044622
0.01067457	1.084476534	214.7841155
0.005339203	1.071199487	314.9172547
0.007951923	1.081300813	273.1587624
0.008912272	1.296370968	351.7273185
0.014782236	1.242647059	240.216299
0.027255495	1.173745174	194.9816602
0.011494389	1.235123367	309.8555878
0.008568558	1.133064516	253.313256
0.018722205	1.28685259	395.6822709
0.009670212	1.222727273	340.8162879
0.006433911	1.13372093	343.0193798
0.019561899	1.583333333	342.1354167
0.015610894	1.033962264	192.4959906
0.007348441	1.392771084	248.688755
0.014062243	1.113372093	240.7049419
0.006655415	1.325997249	429.0464237
0.025201373	1.328125	207.0117188
0.024038238	1.1875	253.1445313
0.007805673	1.137729549	330.653172
0.030339533	1.081081081	221.6006006
0.028339396	1.166666667	159.2017974
0.01544459	1.113105925	208.731149
0.011557216	1.065354884	203.8009487
0.014202795	1.561151079	419.1834532
0.009561429	1.076923077	306.1713287
0.026270728	1.272727273	265.1022727
0.022725826	1.362068966	176.5043103
0.008547739	1.145631068	434.4231392

log.sigma.3.5.mm.3D_gldzm_LowIntensityLargeDistanceEmphasis	log.sigma.3.5.mm.3D_gldzm_HighIntensityEmphasis	log.sigma.3.5.mm.3D_gldzm_DistanceZoneVariability	log.sigma.3.5.mm.3D_gldzm_ZonePercentage
0.010472483	364.3432343	276.2937294	0.42917847
0.013074315	262.5290102	527.2798635	0.282137699
0.028964155	161.9736842	172.8526316	0.437788018
0.008592698	328.4612286	866.3131923	0.376850095
0.017188834	344.316129	136.2903226	0.431754875
0.019320881	183.1718062	198.9823789	0.353032659
0.090214628	157.8522727	67.27272727	0.261127596
0.010945413	384.4311377	417.4471058	0.5
0.006745552	405.0492197	767.7755102	0.400673401
0.009546671	260.4660832	884.4923414	0.295029051
0.023001554	248.9207921	262.1947195	0.366384522
0.0127825	255.3666667	486.5647059	0.344129555
0.022266244	189.8642857	519.575	0.270009643
0.02056761	197.5888325	356.0304569	0.352415027
0.015165307	233.4174455	292.4018692	0.352360044
0.009076576	253.7630058	970.3603083	0.149417015
0.015484048	256.0033113	247.9602649	0.432664756
0.015554574	232.8196078	727.9437908	0.328044597
0.018674262	313.7148289	226.0418251	0.451890034
0.017358169	168.0226537	600.2621359	0.220635487
0.006824243	295.9136513	1151.791118	0.232771822
0.030329085	211.7928349	558.3084112	0.373255814
0.014631837	262.559867	855.2771619	0.330281948
0.037906302	372.3731343	136.920398	0.417879418
0.025519974	163.7225131	364.4240838	0.317275748
0.015053454	282.1304348	260.4637681	0.397694524
0.006654461	335.8100147	628.9911635	0.195903058
0.011522298	233.6738703	489.3929273	0.355198883
0.013552872	266.0207792	723.4961039	0.436260623
0.009115007	292.1770538	670.917847	0.277734068
0.007454289	271.096875	934.3520833	0.301981755
0.011431988	234.9736211	1611.008393	0.281281619
0.015851519	250.0933694	690.691475	0.363502213
0.090769068	307.4615385	39.88461538	0.5
0.022156915	349.3435583	132.5460123	0.490963855
0.010547479	283.2325581	583.4447674	0.340594059
0.020761106	212.0970464	905.021097	0.329166667
0.01558342	199.3110624	860.5969332	0.272212284
0.020774959	280.4148148	213.5851852	0.323353293
0.016243837	238.4191176	390.3970588	0.388941849
0.011533297	272.5083532	388.221957	0.284260516
0.011201427	339.6163366	353.6089109	0.423924449
0.013397831	265.0321101	591.3302752	0.398294762
0.029400895	329.2818627	340.7107843	0.39804878
0.008803264	336.2745098	594.9095023	0.353035144
0.022079635	129.2394881	537.0914077	0.24551167
0.010931744	270.9925976	1304.663526	0.348335677
0.022884857	213.3922414	216.5517241	0.397941681
0.074747051	396.3692308	41.92307692	0.485074627
0.015735033	439.2705314	152.8937198	0.416498994
0.012863495	234.8087318	903.8711019	0.280957944
0.017407257	207.0730897	281.6644518	0.363966143
0.007773314	324.8628527	1178.076803	0.333943994
0.024658043	202.8538012	150.4152047	0.289340102
0.016004132	190.4670051	1312.676577	0.211405795
0.022707502	289.5	338.0945274	0.457337884
0.018070886	399.6163343	797.1671415	0.480164159
0.028516037	356.964497	361.5483235	0.392111369
0.01476805	258.2046205	274.4851485	0.372235872
0.022196859	172.6147186	229.008658	0.262202043
0.023024817	238.3938992	705.6578249	0.355828221
0.008034191	309.0031447	1791.773585	0.3483023
0.010486752	220.6386431	637.300885	0.219417476
0.008711018	276.0598131	509.6317757	0.195898938
0.016566187	301.6818182	104.1636364	0.506912442
0.009651809	314.6549296	529.4084507	0.365743722
0.018960547	358.4971098	134.5953757	0.439086294
0.014000864	259.2704348	1095.363478	0.35971223
0.018050271	247.5612245	180.6530612	0.439461883
0.02212274	241.1155844	719.7558442	0.382894083
0.011477884	302.8390379	971.2238668	0.355592105
0.017601988	233.8581236	402.4828375	0.387068202
0.011470254	217.6779783	1309.19639	0.267995356
0.005765918	319.0795382	1486.756254	0.232132222
0.008350376	279.7773261	2097.252033	0.241176471
0.011860916	366.9798387	407.6814516	0.444444444
0.020842695	248.1593137	347.3382353	0.3652641
0.034376644	198.7606178	230.7374517	0.424590164
0.02699471	318.7271408	589.4644412	0.390589569
0.010420955	258.9284274	907.9032258	0.348559382
0.020877802	408.5697211	207.5896414	0.498015873
0.016347049	354.9742424	569.2757576	0.492170022
0.006900936	354.6996124	944.1007752	0.369627507
0.07137215	359.875	49.44444444	0.496551724
0.016662172	193.4292453	1036.271698	0.312592156
0.010195587	280.7959839	961.6811245	0.227355734
0.015304911	244.4069767	318.9825581	0.442159383
0.013786383	447.9614856	586.1691884	0.468427835
0.028605068	220.5	51.53125	0.349726776
0.074493967	256.71875	56.5	0.60952381
0.008638887	338.6803005	1093.050083	0.301383648
0.032753043	222.8168168	315.4864865	0.471671388
0.032690072	165.4052288	273.8888889	0.299412916
0.019763224	211.9910233	1033.166966	0.272371638
0.013377882	205.5839775	1362.350668	0.245091285
0.02069804	452.8633094	96.72661871	0.549407115
0.009815184	312.2097902	407.5641026	0.352796053
0.091930451	266.6818182	55.09090909	0.536585366
0.030790238	187.637931	91.37931034	0.306068602
0.009698272	441.0809061	280.4563107	0.382899628

log.sigma.3.5.mm.3D_gldzm_IntensityVariabilityNormalized	log.sigma.3.5.mm.3D_gldzm_LowIntensitySmallDistanceEmphasis	log.sigma.3.5.mm.3D_gldzm_IntensityVariability
0.04580161	0.008393878	13.87788779
0.050029703	0.011333151	29.31740614
0.057285319	0.026330411	10.88421053
0.045554531	0.00725126	45.23564955
0.042580645	0.014653476	6.6
0.064798463	0.01626728	14.7092511
0.064566116	0.012249792	5.681818182
0.041390273	0.008042332	20.73652695
0.04067077	0.006127764	33.8787515
0.052463742	0.008823571	47.95185996
0.058545459	0.008545445	17.73927393
0.046728181	0.01180278	23.83137255
0.051033163	0.020836828	28.57857143
0.053763302	0.018307539	21.18274112
0.052047243	0.013723482	16.70716511
0.055102632	0.008414657	57.19653179
0.050195167	0.012424835	15.1589404
0.04777137	0.013636164	36.54509804
0.044282843	0.015706935	11.64638783
0.060242352	0.01657156	37.22977346
0.06071189	0.005868147	73.82565789
0.051081608	0.011430887	32.79439252
0.05547662	0.007053285	50.03991131
0.048092869	0.004961683	9.666666667
0.062018585	0.015114795	23.69109948
0.045289855	0.012900406	12.5
0.054149197	0.006155132	36.76730486
0.056858666	0.010025384	28.9410609
0.0462169	0.011234564	35.58701299
0.04856792	0.008465359	34.28895184
0.061386719	0.00666009	58.93125
0.053107155	0.010755267	88.58273381
0.046196722	0.014951823	34.13937754
0.048816568	0.009496261	2.538461538
0.045805262	0.012467901	7.466257669
0.04901298	0.008878708	33.72093023
0.049355516	0.019787461	46.78902954
0.057021074	0.014235116	52.06024096
0.049657064	0.015887948	13.40740741
0.0471213	0.015565044	19.2254902
0.051383849	0.009846784	21.52983294
0.04526517	0.008973552	18.28712871
0.048985776	0.010089225	32.03669725
0.04203912	0.007966544	17.15196078
0.048340534	0.006633761	32.04977376
0.069753918	0.020984225	38.15539305
0.048520149	0.00839335	72.10094213
0.053247622	0.021490693	12.35344828
0.046153846	0.006523694	3
0.038437303	0.012615697	7.956521739
0.054492762	0.009657534	52.42203742
0.057979493	0.015535734	17.45182724
0.046568676	0.006468268	59.42163009
0.055983038	0.01634385	9.573099415
0.055333788	0.014685556	76.30529369
0.041434618	0.017603549	16.65671642
0.042370688	0.004441057	44.61633428
0.043233002	0.009903381	21.91913215
0.049178185	0.012048527	14.9009901
0.066921534	0.018138417	15.45887446
0.045240591	0.016497901	34.11140584
0.04601831	0.007578116	87.80293501
0.059014453	0.009765672	40.01179941
0.054638833	0.007903618	29.2317757
0.054214876	0.015706793	5.963636364
0.045508084	0.008544855	25.84859155
0.047479034	0.014599043	8.213872832
0.045618147	0.01314105	52.46086957
0.053727613	0.016201085	10.53061224
0.047923765	0.013667139	36.9012987
0.048991024	0.006163398	52.95929695
0.046557295	0.015555416	20.34553776
0.05425276	0.010475649	75.1400722
0.059963851	0.005232525	93.48364336
0.049348443	0.00785231	109.2574526
0.043314256	0.008175111	21.48387097
0.050497405	0.013267121	20.60294118
0.051445268	0.025475208	13.32432432
0.047423645	0.007619309	32.67489115
0.054591978	0.008105459	54.15524194
0.039888256	0.018183306	10.01195219
0.042479339	0.008001003	28.03636364
0.045242173	0.006317155	46.68992248
0.05632716	0.006609336	4.055555556
0.056078676	0.015348074	59.44339623
0.054362349	0.006636654	67.6811245
0.047187669	0.013751576	16.23255814
0.042924797	0.004872673	31.20632737
0.068847656	0.024350449	4.40625
0.059082031	0.011424306	3.78125
0.044137279	0.00759737	52.87646077
0.043818593	0.029736155	14.59159159
0.054081763	0.027251727	16.54901961
0.053262057	0.014364931	59.33393178
0.057374357	0.01110205	81.64371047
0.037523938	0.012578984	5.215827338
0.046114724	0.00949799	19.78321678
0.058769513	0.009855798	3.878787879
0.061236623	0.020709723	7.103448276
0.040688723	0.008260106	12.57281553

log.sigma.3.5.mm.3D_gldzm_HighIntensityLargeDistanceEmphasis	log.sigma.3.5.mm.3D_gldzm_SmallDistanceEmphasis	log.sigma.3.5.mm.3D_glcm_SumVariance	log.sigma.3.5.mm.3D_glcm_Homogeneity1
389.9768977	0.965346535	812.7980508	0.305489976
289.8105802	0.960324232	559.6691762	0.401046522
178.6789474	0.964473684	292.1923258	0.323285666
381.5730111	0.948640483	939.8629812	0.310353017
387.7677419	0.951612903	894.7331784	0.297183842
202.9955947	0.950440529	370.9730505	0.33584478
166.5454545	0.897727273	267.7042214	0.395229514
434.3892216	0.931137725	915.5572709	0.263419088
445.3853541	0.969387755	1028.390826	0.298325347
268.9310722	0.987691466	521.6002151	0.372603906
281.5742574	0.945544554	600.4658319	0.31629005
264.5431373	0.982352941	553.6292431	0.33557947
205.6357143	0.971875	399.0617021	0.364285729
216.8832487	0.961928934	448.6857575	0.326756454
255.8380062	0.964953271	535.4473366	0.33048139
277.7254335	0.974710983	563.3848167	0.529492876
303.4668874	0.925496689	630.5994273	0.291854181
237.4235294	0.981372549	425.7782232	0.341901151
349.2357414	0.942965779	759.5114639	0.276339769
173.2556634	0.98907767	262.8544356	0.433221653
312.6628289	0.979646382	669.5990497	0.418158663
238.694704	0.947429907	470.902663	0.322164461
270.0964523	0.980044346	532.4399582	0.35918046
486.238806	0.850746269	1144.824925	0.308512678
173.4685864	0.982329843	277.7695909	0.379885253
289.4565217	0.97826087	536.6126804	0.30632244
374.1340206	0.971281296	801.9606289	0.460561544
240.9056974	0.985265226	434.2550139	0.360168703
280.8337662	0.976623377	767.6671749	0.299652409
307.8739377	0.980878187	627.2523577	0.410300305
278.678125	0.98984375	557.5081786	0.378826727
244.7380096	0.986960432	498.8640493	0.390068162
267.2652233	0.974627876	639.1501928	0.322012058
337	0.899038462	758.5652604	0.261077418
402.5337423	0.921779141	916.1194273	0.276647307
336.940407	0.937863372	757.872995	0.320568892
224.6698312	0.982594937	395.5786708	0.349760079
210.8937568	0.977820372	445.1852644	0.381727847
339.8481481	0.911111111	750.4411639	0.335711565
249.5514706	0.983455882	506.9530713	0.322552142
294.5178998	0.971360382	650.6887555	0.374678288
376.6782178	0.949876238	799.7109183	0.29100269
280.5688073	0.962155963	681.7408061	0.304037708
358.877451	0.931985294	757.1081385	0.290730626
370.1523379	0.959276018	881.130408	0.321669751
131.3180987	0.993144424	170.8200507	0.420751135
311.320996	0.951043069	668.2755306	0.329932047
224.3965517	0.974137931	403.7606281	0.313358171
475.7538462	0.826923077	1064.410607	0.267928623
547.6328502	0.884057971	1154.341509	0.284585974
247.8440748	0.976611227	494.0326832	0.378459285
215.2059801	0.975083056	449.3487281	0.3163684
347.9882445	0.970023511	770.9700839	0.3452973
221.3625731	0.951754386	420.9488323	0.356958081
199.1036983	0.981508339	369.6403192	0.460384085
333.8059701	0.934701493	689.7221407	0.275006746
477.6191833	0.893874644	977.7556924	0.263738377
441.4970414	0.869822485	979.8899261	0.298214622
273.0363036	0.962871287	537.5135576	0.34237231
172.6666667	0.996753247	351.6013655	0.37018178
252.5822281	0.975132626	431.5966681	0.321748096
338.9842767	0.976415094	775.6207156	0.339397868
237.9528024	0.976769912	481.3847199	0.433687006
288.6990654	0.981775701	651.9999953	0.456544876
311.6363636	0.979545455	688.2932216	0.290847044
336.5950704	0.973591549	772.0153768	0.328517457
409.0982659	0.904624277	816.0074201	0.287140554
268.6643478	0.98173913	537.3357954	0.326490137
259.622449	0.969387755	489.2809778	0.298568875
252.4337662	0.974675325	519.4842332	0.324332232
329.6641998	0.959759482	672.3197355	0.329745264
245.6727689	0.969107551	478.7897187	0.30698841
229.2534296	0.978880866	404.7052579	0.396919626
335.7286722	0.982200128	720.0213037	0.415490739
306.2515808	0.979674797	717.3558747	0.419907467
427.9899194	0.925907258	955.657431	0.271915688
279.9313725	0.939338235	600.470047	0.312477698
213.8764479	0.956563707	357.5796406	0.301920729
354.2133527	0.941219158	752.957298	0.300753492
281.3891129	0.966733871	603.2599259	0.33765727
460.1195219	0.928286853	862.2355606	0.254796335
411.6060606	0.944318182	954.8260008	0.27054733
401.4205426	0.966569767	964.341069	0.330766965
430.8333333	0.854166667	954.7737226	0.28819852
197.1622642	0.991509434	378.374155	0.358718667
409.2248996	0.901807229	635.8734893	0.406266163
259.2151163	0.971656977	482.7930308	0.300056858
523.6217331	0.918500688	1168.903416	0.263330232
274.453125	0.91796875	495.8339304	0.358493712
271.015625	0.953125	553.0753973	0.281059808
370.7888147	0.965567613	805.2493102	0.368935264
227.6816817	0.97972973	389.823211	0.284609905
190.2189542	0.958333333	355.7619387	0.400510819
225.0305206	0.971723519	461.5076623	0.389207671
212.7160928	0.983661279	411.9368056	0.400887973
587.5827338	0.85971223	1139.867295	0.233613651
336.3636364	0.980769231	923.2711588	0.336064117
273	0.931818182	579.6239268	0.261160027
232.1724138	0.909482759	484.4249642	0.354588491
467.7119741	0.963592233	956.6221756	0.325544975

log.sigma.3.5.mm.3D_glcm_Homogeneity2	log.sigma.3.5.mm.3D_glcm_ClusterShade	log.sigma.3.5.mm.3D_glcm_MaximumProbability	log.sigma.3.5.mm.3D_glcm_Idmn	log.sigma.3.5.mm.3D_glcm_SumVariance2
0.21468837	235.7205983	0.013072684	0.97513021	103.1692039
0.327063111	205.6362448	0.037132633	0.980676087	67.8245241
0.23183576	258.1255939	0.016151818	0.963363626	70.57196469
0.220645005	-93.14427195	0.010154757	0.975962067	91.58651257
0.204390403	17.12913032	0.016311343	0.968445054	141.4625304
0.242465577	33.48604243	0.013990968	0.968942484	58.92531559
0.312939967	97.61245445	0.029502616	0.973396048	42.47228505
0.170434094	262.8517607	0.008074778	0.971830797	131.7749704
0.207887515	320.608924	0.009121444	0.974583435	118.4158437
0.289197318	249.8661932	0.016116154	0.980125422	65.28228555
0.224391771	-107.7775516	0.013624155	0.964607173	68.24063582
0.24644651	323.9648348	0.012520139	0.976785513	89.00533234
0.27715367	372.7622444	0.014380788	0.978723089	77.90508797
0.234134429	-17.15788209	0.01109804	0.971707608	84.2479606
0.23902513	159.7716656	0.014475878	0.970678344	75.90820914
0.476490052	98.18566527	0.131579321	0.990563309	41.63363289
0.1984461	-64.16823954	0.009680291	0.967081996	104.8737212
0.256496596	533.8319266	0.012039142	0.973375061	98.61705903
0.183891223	-274.0259485	0.012097068	0.964115771	138.5878217
0.35985415	125.0845953	0.043604801	0.980005132	47.69587808
0.341738675	91.36303023	0.027466066	0.987965566	41.65285997
0.229387546	409.6948622	0.010159927	0.979739439	88.59347567
0.272975448	263.7137799	0.016271896	0.980274138	70.27385509
0.214863034	-579.6030201	0.018000698	0.972746808	98.01544894
0.295594386	126.4642616	0.025096185	0.975979797	42.54654051
0.217249436	347.2636065	0.012990781	0.968345421	93.88227936
0.392420865	150.3600455	0.082326001	0.988223836	43.90939865
0.274543194	313.6215453	0.01802141	0.979083006	68.02071919
0.209063317	-229.2665794	0.011950956	0.966744013	112.517196
0.337625575	93.95346293	0.036634764	0.986048451	64.36931386
0.295466812	247.5456828	0.022209215	0.981966941	54.84720245
0.309811359	242.3698802	0.031810544	0.98270932	73.45426905
0.232282424	27.5596569	0.009040879	0.973614209	97.45589122
0.167581042	75.23920496	0.026385105	0.949055804	118.9608593
0.183277467	-483.983183	0.018794237	0.960789346	113.4159476
0.229853752	-38.30643499	0.011383442	0.975874804	89.81076016
0.265804756	482.9093054	0.014167324	0.976566339	90.12687562
0.29753128	44.58150466	0.018363516	0.978514754	59.24530484
0.246768065	-260.1883336	0.019339704	0.976862429	77.7979556
0.232809789	19.87459118	0.015249273	0.972179981	86.62318816
0.292059227	41.94647902	0.019970046	0.982645561	65.81349472
0.198154901	204.2775993	0.011165545	0.97409293	114.0522418
0.211699331	-135.7310073	0.010984726	0.970161357	90.76595876
0.198371015	128.4057866	0.010218459	0.971291918	121.1930251
0.229436151	31.67095423	0.012709413	0.977311949	73.3915268
0.344651481	149.0687961	0.0298259	0.976592812	37.15288407
0.238863641	69.29190755	0.010131788	0.97684822	77.78145655
0.22308624	67.14114792	0.012668586	0.965439058	85.81613878
0.177889287	-475.1442062	0.034438959	0.95542338	103.7484853
0.194732277	-428.6748809	0.017112719	0.97363893	138.7444242
0.295096029	202.0440454	0.026346313	0.981971096	66.63506796
0.227542586	-20.08302069	0.016148421	0.950955405	91.7744338
0.26069612	232.9769418	0.01905586	0.980658638	79.21032356
0.267795382	-87.24312973	0.021560158	0.971189441	63.02492109
0.397223641	194.2797629	0.066267602	0.981189257	59.93588167
0.182058803	15.35310302	0.009442284	0.967382356	117.3953935
0.171229058	1.659494819	0.007332455	0.965333625	117.5648545
0.207466241	-450.5581279	0.014488266	0.971507375	100.0038327
0.253957524	230.5001989	0.01593415	0.973842098	82.98685532
0.280010958	-4.726015377	0.015569195	0.974979501	52.99767025
0.233130682	467.5138036	0.015771114	0.971878361	97.81390175
0.254233437	407.7476755	0.015051409	0.983333829	97.95213237
0.361324365	118.4910498	0.04196891	0.98605615	43.94852789
0.390745185	131.7250078	0.053610546	0.983437974	43.45363487
0.194306597	-126.6223675	0.017552862	0.959299732	93.54103803
0.242078707	364.3808433	0.015699422	0.978018257	88.12694079
0.195375318	-496.2578099	0.020164622	0.968158856	106.0187383
0.240416214	298.8604169	0.01073133	0.970536069	112.0233228
0.204784187	-94.1178486	0.012914553	0.963672409	96.75218227
0.235895487	339.9023185	0.011251346	0.9724675	91.00877488
0.243639747	288.2481364	0.011868766	0.975937271	92.53017312
0.215842815	248.0528939	0.010906747	0.970545526	104.7111724
0.31926942	275.5423623	0.026957313	0.980351044	62.90012774
0.340799089	136.8771826	0.028441416	0.984140597	51.93778728
0.346953961	234.101713	0.038600502	0.986633255	56.09563462
0.180139486	31.1829552	0.00861512	0.972180397	118.9656601
0.221013935	93.37353673	0.011465033	0.975528217	81.08190704
0.211354959	573.0800125	0.016177441	0.962387456	108.8458319
0.209881978	111.1989043	0.010742621	0.968207631	93.93655583
0.248964163	169.6013632	0.013947213	0.977424295	74.7626564
0.163650177	133.9075024	0.011622404	0.961428485	137.9295569
0.177664809	-74.29962891	0.008325334	0.971783464	116.3946268
0.244992433	97.04134507	0.0172132	0.977167426	89.2648536
0.196958102	-420.5969964	0.031696687	0.959376432	87.41246548
0.272027014	197.0989261	0.013504162	0.974785909	65.92576428
0.329065397	217.2202619	0.030471948	0.985205105	53.48115483
0.207336807	478.3490015	0.012116275	0.968590668	111.5687729
0.17175857	-428.9388993	0.008414009	0.966876166	137.3051646
0.269621941	64.49557551	0.031063631	0.967470455	47.15274318
0.18590547	-1.984361759	0.026487511	0.957078614	77.42514356
0.28949629	157.0925755	0.03195139	0.98222501	80.10245732
0.192927343	579.3858157	0.012048755	0.964867022	115.3534634
0.321767742	118.3930117	0.050250438	0.974626663	54.71421813
0.308221778	141.9687411	0.032593546	0.979601909	60.14016377
0.321838359	99.62350276	0.021929957	0.980231135	55.25962127
0.145595054	-25.32768434	0.013886272	0.953165387	210.6327956
0.24743162	-68.99615048	0.01493088	0.975377162	78.29235266
0.166295152	-125.0347784	0.021737747	0.941077808	99.43765894
0.263185387	-133.1460996	0.021989508	0.973415426	72.69938398
0.238930117	431.2568663	0.016601351	0.980871042	95.69136132

log.sigma.3.5.mm.3D_glcm_Contrast	log.sigma.3.5.mm.3D_glcm_DifferenceEntropy	log.sigma.3.5.mm.3D_glcm_InverseVariance	log.sigma.3.5.mm.3D_glcm_Entropy	log.sigma.3.5.mm.3D_glcm_Dissimilarity
30.95344204	3.488870824	0.221550551	8.210981081	4.361945185
21.44057492	3.243309175	0.30198631	7.847156735	3.284039871
23.60709377	3.294940852	0.25363814	7.650776583	3.801423491
31.75285385	3.548816696	0.224680598	8.559622836	4.362134104
33.57991706	3.521259268	0.208660772	7.94412572	4.517720469
19.53735421	3.2030821	0.244716185	7.705516175	3.516066904
12.91371002	2.85508915	0.326188867	6.895588511	2.72722318
43.94606695	3.752712021	0.18063832	8.716857431	5.305210757
35.66738772	3.64115141	0.211997365	8.751751844	4.63715175
19.30854591	3.223763589	0.289093041	7.930979593	3.270491451
24.30176809	3.320969037	0.236260781	7.886291481	3.939976766
24.13181727	3.34731215	0.252356426	8.216341499	3.76896125
18.04096524	3.162714896	0.279832076	7.965367731	3.234586123
22.41410458	3.304546014	0.241012394	8.099554255	3.738753008
23.48701043	3.340874494	0.239775126	8.035407197	3.775508107
11.64333409	2.75709186	0.34861207	6.791598466	2.120172302
32.59325331	3.480664118	0.208386888	8.27234165	4.525590531
26.30364255	3.40733222	0.257005042	8.334579754	3.868281474
40.64586278	3.656758018	0.187891881	8.399319032	5.058103002
13.54011744	2.99110105	0.317867955	7.426688392	2.658057667
13.75250286	3.016670252	0.320783134	7.45630012	2.715745165
25.10692136	3.391492962	0.234585063	8.254165905	3.892339578
20.32641479	3.258065089	0.271942179	8.014536936	3.418962168
28.57893058	3.450915843	0.218690479	7.947266063	4.180485811
16.35384535	3.118150477	0.292419651	7.442058867	3.061299556
35.99708814	3.615395387	0.218616154	8.211257786	4.585447846
12.77624353	2.94375033	0.32766652	7.206549537	2.479373238
20.25580844	3.251025988	0.275500164	7.821744214	3.410468268
32.9028054	3.5357064	0.215538454	8.442043722	4.516557653
20.28327459	3.21804277	0.307043734	7.820875349	3.171910637
17.3012476	3.163721709	0.289815524	7.717462929	3.137785869
18.90225485	3.206162969	0.280437707	8.025260547	3.20622815
27.60169829	3.456748102	0.239741328	8.479242706	4.051873899
46.05037	3.622264594	0.166442352	6.582998061	5.432545695
39.55198822	3.617807918	0.189556508	7.829226202	4.981094941
26.58679469	3.427961035	0.23502548	8.355449844	4.020470161
26.08928453	3.414850648	0.262898764	8.231765733	3.808744154
15.61793105	3.098420626	0.293668286	7.81425691	3.013991578
23.93092636	3.292864501	0.249088431	7.890796846	3.778783265
27.41515284	3.445673795	0.239812512	8.20222862	4.03751241
20.3445398	3.240039068	0.289255114	7.889300089	3.306351189
34.30419258	3.58984132	0.206137061	8.446881047	4.61477508
29.30210367	3.453042768	0.222550841	8.322715703	4.272586298
36.30137925	3.626731618	0.201472073	8.569659456	4.714568087
24.95988136	3.39061744	0.233325589	8.222086114	3.91226986
12.37453172	2.914613338	0.334529932	7.116393416	2.60116472
24.01200869	3.381584452	0.244663704	8.34793781	3.794272431
27.8269726	3.435559298	0.232506805	8.000955793	4.151683381
48.83089643	3.689507172	0.184769356	6.876013146	5.485857303
41.30037153	3.689285314	0.20404905	8.157773318	5.004477617
18.47919675	3.211054242	0.276874622	7.966283812	3.230119474
29.74437818	3.458917554	0.220746097	7.929221066	4.27835751
26.74852611	3.43381711	0.249086637	8.330644532	3.905015925
18.18526615	3.17620208	0.265930521	7.649092492	3.307389968
17.32436205	3.052775584	0.324622229	7.5022084	2.786793095
41.46913182	3.709857483	0.187254009	8.591198885	5.085220665
47.07976602	3.731384156	0.178424089	8.846867303	5.418871969
36.12327826	3.611905441	0.209575706	8.455093048	4.650241316
22.36476631	3.307621047	0.262950272	8.015876502	3.625592143
14.28726454	2.984560943	0.277576049	7.492014443	2.974681171
29.67183181	3.52159652	0.232330147	8.381570034	4.178988131
28.39413282	3.484730893	0.249386355	8.534411331	3.993311543
13.25406437	2.959395826	0.325202906	7.330117102	2.64025016
14.00255955	2.950199093	0.340770966	7.193783376	2.576763923
30.96939547	3.454157848	0.198474592	7.455087068	4.432376813
30.78226455	3.519483831	0.241478397	8.329092508	4.180664344
38.22321948	3.610590736	0.199515161	7.917362594	4.840645529
31.22273725	3.545912214	0.237698393	8.623214934	4.241597173
29.25832147	3.453527577	0.21301597	7.92660477	4.319961385
28.961222	3.495811715	0.237212222	8.375226271	4.12117111
30.18581939	3.478575997	0.241138516	8.390824403	4.159498173
30.98445715	3.526458487	0.217169121	8.376148413	4.352778774
19.14419735	3.209278922	0.294884148	7.866135338	3.168792255
16.23077421	3.098399414	0.312629066	7.639271157	2.905920704
17.43108746	3.136661933	0.317277685	7.704055981	2.9343827
43.5951461	3.704681997	0.187833578	8.637696864	5.211763057
28.7924972	3.45399377	0.228829728	8.186146919	4.175091387
32.99130822	3.550750887	0.216223227	8.06068188	4.493616076
33.59776945	3.549550727	0.21352686	8.447130454	4.529698611
23.25422163	3.35516034	0.250297058	8.178922667	3.738689808
52.28698505	3.806352066	0.159029984	8.351627335	5.80629199
41.87761481	3.728830809	0.18644059	8.709310225	5.143681532
30.18468247	3.513915706	0.241410168	8.462169624	4.159767089
36.05275141	3.493353733	0.212606941	6.885736458	4.716477207
20.09595292	3.239084329	0.270507849	8.004259271	3.406748994
16.11908574	3.100684779	0.314231614	7.678602271	2.914742173
31.03034544	3.522927105	0.214155172	8.281211145	4.390889662
47.0304827	3.790902058	0.177150153	8.867358028	5.46302538
17.35855377	3.012069401	0.271099244	6.779603759	3.252277734
32.77324857	3.33090648	0.210693716	6.519719606	4.579000956
26.05710084	3.407043031	0.267530428	8.230040219	3.726206147
40.36871101	3.702952326	0.199521617	8.25236663	4.930686222
17.4401443	3.164367485	0.288415248	7.605616871	3.068575325
18.57430254	3.203670989	0.282887492	7.891650217	3.174877207
15.55536617	3.087585623	0.310501733	7.726354709	2.90984861
68.59714412	3.904502919	0.150155136	7.868795974	6.657606023
24.13892979	3.365536548	0.25719549	8.150343632	3.75864525
43.06239396	3.565897487	0.163049225	6.840894168	5.311152453
16.59787391	3.0914456	0.265244997	7.502414702	3.213241375
34.73729132	3.581448046	0.240590047	8.136060856	4.334432078

log.sigma.3.5.mm.3D_glcm_DifferenceVariance	log.sigma.3.5.mm.3D_glcm_Idn	log.sigma.3.5.mm.3D_glcm_Idm	log.sigma.3.5.mm.3D_glcm_Correlation	log.sigma.3.5.mm.3D_glcm_Autocorrelation
10.86585254	0.893058825	0.21468837	0.540668552	272.6991254
10.09807818	0.913962189	0.327063111	0.522001574	196.2780251
8.571562256	0.87274635	0.23183576	0.497994912	110.1532574
11.82990682	0.8960052	0.220645005	0.490194317	310.7030515
12.0767393	0.881204195	0.204390403	0.618390275	296.3775067
6.782616608	0.880016367	0.242465577	0.504146859	137.1350375
4.751827427	0.892907727	0.312939967	0.538083427	103.2965942
14.9235662	0.884509087	0.170434094	0.503918054	302.1752153
13.48705487	0.892981423	0.207887515	0.538595896	337.5746096
8.258977784	0.908107737	0.289197318	0.546488337	185.3026765
7.930316295	0.872490196	0.224391771	0.478179522	207.789107
9.121869303	0.898372086	0.24644651	0.575765058	195.3974655
7.098152619	0.902826347	0.27715367	0.621349452	147.4202095
8.000091582	0.885738453	0.234134429	0.581154955	162.3961089
8.676713101	0.885244653	0.23902513	0.531365761	189.0736432
6.937194904	0.945845128	0.476490052	0.565634836	194.6487962
10.54602753	0.877204741	0.1984461	0.528924099	217.0350292
10.54364353	0.893824433	0.256496596	0.579131991	154.4000836
13.35804611	0.872435385	0.183891223	0.550156934	254.8506171
6.225173933	0.910868039	0.35985415	0.557778169	102.9859309
6.174323955	0.928238803	0.341738675	0.505689483	228.4985351
9.385551332	0.903039627	0.229387546	0.561268674	169.029804
8.208558865	0.906736568	0.272975448	0.554124118	188.7621099
10.45933249	0.888496405	0.214863034	0.554601884	369.5918143
6.690065928	0.89816023	0.295594386	0.449920124	106.7298818
14.06700169	0.88366639	0.217249436	0.450544134	186.7021724
6.44537152	0.932786792	0.392420865	0.550457784	266.951852
8.177190813	0.904333049	0.274543194	0.542632053	157.3531955
11.4493651	0.87775028	0.209063317	0.54958098	258.8501877
9.839124226	0.926372047	0.337625575	0.524945454	216.9929698
7.123061724	0.911010948	0.295466812	0.52125585	195.3954332
8.074291912	0.914762428	0.309811359	0.591594726	179.0096049
10.58932124	0.891879877	0.232282424	0.560785494	221.5886142
15.30657986	0.849115699	0.167581042	0.444941348	245.0608697
13.57977371	0.867255679	0.183277467	0.489398213	299.8226662
9.784779676	0.895080859	0.229853752	0.545984833	257.1278565
11.089068	0.901053565	0.265804756	0.551817623	144.4281756
6.272479053	0.902578013	0.29753128	0.582858169	162.0700591
8.486884786	0.897991678	0.246768065	0.533774317	253.794092
10.57441436	0.889204753	0.232809789	0.521513754	179.6214656
9.037373746	0.91481294	0.292059227	0.531979677	224.5597545
12.21648097	0.890375701	0.198154901	0.540369935	268.7407726
9.836334404	0.883069589	0.211699331	0.515856444	233.0778688
12.96031034	0.885893401	0.198371015	0.543215248	255.9479685
9.136823137	0.89729317	0.229436151	0.49593996	293.0397342
5.302311206	0.901824465	0.344651481	0.499533678	71.05623243
9.198553482	0.897517839	0.238863641	0.530292473	230.4387615
9.884223931	0.87562947	0.22308624	0.510915096	146.0858627
17.19673609	0.860791878	0.177889287	0.370002856	336.0054806
15.13285485	0.89088707	0.194732277	0.5456073	371.899892
7.696786796	0.911401297	0.295096029	0.566633938	177.1992938
10.2519592	0.855402072	0.227542586	0.511147183	159.566937
10.77737011	0.908220168	0.26069612	0.498394293	260.5185727
6.922322933	0.886815698	0.267795382	0.554693314	153.5143368
9.066669939	0.920004377	0.397223641	0.556396813	136.734196
14.65010598	0.878226428	0.182058803	0.483326612	233.8462114
14.84067942	0.874689313	0.171229058	0.438321014	319.6316824
13.24739663	0.887584988	0.207466241	0.477297708	321.088076
8.852392817	0.892902654	0.253957524	0.57518757	190.1481259
4.987954557	0.89217042	0.280010958	0.576101449	131.9532364
11.70019299	0.889236076	0.233130682	0.535896168	155.3004404
11.83211323	0.914627917	0.254233437	0.55566456	262.963028
5.898425304	0.92412755	0.361324365	0.539123455	171.6955434
7.042742258	0.922222627	0.390745185	0.511270625	222.4563729
10.69335874	0.864085413	0.194306597	0.505363981	232.2970157
12.54799377	0.902762714	0.242078707	0.48713677	260.0644524
13.43116845	0.880470018	0.195375318	0.473537327	270.2752321
12.62224983	0.888298229	0.240416214	0.563834837	189.2495447
9.899983488	0.870881501	0.204784187	0.537643452	172.7426638
11.38226477	0.890591777	0.235895487	0.5202468	183.5861759
11.42585564	0.898290524	0.243639747	0.514934176	230.5424888
11.26321349	0.884760127	0.215842815	0.54504027	170.2297086
8.649249251	0.911272967	0.31926942	0.536658496	148.3998393
7.486991986	0.919981924	0.340799089	0.525908089	244.4418812
8.541367245	0.92776978	0.346953961	0.529936204	243.3925025
14.38768346	0.886661137	0.180139486	0.468137971	313.3418843
10.37008593	0.894494743	0.221013935	0.480941571	208.0047999
12.00757142	0.871455778	0.211354959	0.535769835	129.27623
11.8200478	0.880970368	0.209881978	0.477934183	254.2824542
8.817617449	0.898821066	0.248964163	0.527465635	210.1091506
16.51772898	0.86683768	0.163650177	0.455616379	283.2459634
14.48104013	0.885136166	0.177664809	0.475778098	313.8572434
12.07838568	0.900747506	0.244992433	0.499370279	317.9466196
12.85369398	0.866063078	0.196958102	0.424840957	306.7592091
7.870362087	0.895442078	0.272027014	0.531563833	140.1541254
7.300985577	0.921757572	0.329065397	0.539596276	219.4036831
11.14720064	0.880436519	0.207336807	0.567011564	171.0219225
15.7934944	0.87646448	0.17175857	0.496839979	376.6790645
5.587875575	0.879874499	0.269621941	0.452822212	173.1087139
10.30898142	0.860290525	0.18590547	0.419105391	185.6874685
11.45394574	0.91463892	0.28949629	0.511898874	270.5793526
15.22113495	0.875845566	0.192927343	0.4846035	137.990959
7.740564047	0.898951588	0.321767742	0.516385116	132.5016952
8.051460971	0.908076388	0.308221778	0.528759776	166.5267975
6.830456114	0.90925136	0.321838359	0.559907052	151.1695979
19.99541511	0.854660168	0.145595054	0.512429141	360.4385545
9.573305473	0.895843415	0.24743162	0.532103137	306.0045397
13.0578812	0.837389941	0.166295152	0.407461648	192.4870287
5.944581673	0.888923652	0.263185387	0.63010475	173.8731189
15.24711818	0.910760113	0.238930117	0.4729649	313.8970268

log.sigma.3.5.mm.3D_glcm_SumEntropy	log.sigma.3.5.mm.3D_glcm_AverageIntensity	log.sigma.3.5.mm.3D_glcm_Energy	log.sigma.3.5.mm.3D_glcm_SumSquares	log.sigma.3.5.mm.3D_glcm_ClusterProminence
5.275857186	16.12726482	0.004233569	34.37496771	30980.83345
5.00202341	13.68846795	0.007948779	23.22749129	15151.28575
4.953209985	9.917690634	0.006068011	23.54476461	13642.44454
5.269224295	17.42908076	0.003433405	31.10673111	23477.38737
5.381370094	16.50691013	0.005014936	44.38765693	51397.64731
4.899197527	11.28112473	0.005696341	19.61566745	8104.62817
4.577714808	9.791483102	0.011183432	13.84649877	5932.474319
5.482988578	17.01182195	0.002866221	43.94951065	47038.32436
5.436761804	18.00853075	0.002996864	38.64322077	41469.38497
5.005766791	13.24952345	0.006067632	21.93240025	13507.43542
4.986880471	14.02714015	0.005164946	23.13560098	10694.56418
5.216042989	13.4856524	0.004424966	29.39583861	22982.74895
5.096531406	11.52340225	0.005313746	24.26322315	18346.27584
5.153259949	12.12491699	0.004369881	26.74142066	16081.77787
5.093551877	13.31179463	0.004893431	25.55928644	16108.33815
4.518659247	13.72791625	0.030327725	13.70484873	8791.529736
5.281242278	14.20516913	0.003862334	35.42344126	25756.76372
5.263334639	11.7370934	0.004137101	32.13471892	28348.84623
5.436966298	15.37879166	0.003618191	46.17249583	45597.15226
4.768728931	9.717924243	0.009232524	15.30899888	7429.429969
4.708448364	14.97061598	0.009026027	14.45596963	5854.640049
5.198676627	12.44749091	0.00419548	29.21075773	24533.24365
5.055479653	13.35340635	0.00564254	23.48970837	15495.85374
5.181270518	18.99174556	0.005201605	32.10667992	29214.53441
4.681117591	10.00879799	0.008098738	14.72509647	5527.147256
5.205645475	13.26950382	0.004281768	33.96085011	25335.77088
4.664679869	16.17199287	0.016020769	14.66402239	8110.291247
4.980344259	12.118934	0.006108062	22.88766929	13299.51336
5.319953297	15.60808003	0.003630035	37.4483173	26197.79276
4.978419992	14.51392266	0.008192984	21.9486097	14555.3702
4.85687921	13.68395121	0.007047869	18.5597003	10180.68167
5.093576197	12.9302491	0.006107875	23.86695762	16211.88222
5.299963221	14.39898879	0.003502574	32.30663	24011.00381
4.830385677	15.13019101	0.011520212	42.12679599	32635.11989
5.214863188	16.97139699	0.005469768	39.37944914	33660.20452
5.221955574	15.68517656	0.003784671	30.02718665	18933.55143
5.187110856	11.4113635	0.004748405	30.13624399	22936.24686
4.944305407	12.29639338	0.005925688	18.75221157	9195.124395
5.069168823	15.57938081	0.006006176	26.02187801	20682.2396
5.174202213	12.88339331	0.004353877	29.08732212	19730.15511
5.017960271	14.74699842	0.006224124	22.50539992	13233.3196
5.361518539	15.96893218	0.003558008	37.68722332	35034.78916
5.199658499	14.84819532	0.003819211	30.8330077	18357.46808
5.424016691	15.47782051	0.003301334	40.21574013	39154.00666
5.101071376	16.90428031	0.004345819	25.27219754	15261.34835
4.545335979	8.053209789	0.010637507	12.38185395	4286.828401
5.161425206	14.83653517	0.003973522	26.34071657	15836.11955
5.111281493	11.47734852	0.004662857	28.588874	17111.56652
4.908332199	18.17205703	0.010292505	38.70215594	34061.7448
5.416115666	18.88614724	0.004813347	44.18462883	61770.19701
5.029247665	12.89726896	0.005790756	21.86698551	13509.28508
5.09524659	12.00092369	0.005187855	30.37970299	15623.97735
5.156638595	15.9221938	0.004430974	27.15211229	20868.78643
4.939190518	11.92825002	0.006476118	20.30254681	10905.59179
4.859007084	11.24845199	0.013830783	19.63300872	12483.65011
5.393012664	14.87826626	0.003139222	40.96361143	35060.476
5.426532446	17.71976305	0.002679599	41.55597907	35229.42053
5.271957426	17.7075381	0.004006654	34.30774916	30706.40818
5.136973899	13.26668746	0.005047258	26.93376917	18704.38157
4.835271365	11.0569132	0.00676631	16.8212337	6699.647713
5.247334897	11.82653279	0.004094876	32.793506	25783.14721
5.309435664	15.89130694	0.003770698	32.08347972	34076.72013
4.698965529	12.82746614	0.011015088	14.58744448	7725.824527
4.663333588	14.69668299	0.013280173	14.72014873	6788.94325
5.050454537	14.76670051	0.00654461	31.78134529	21879.84157
5.199740352	15.90306263	0.004468343	30.48003417	26706.34044
5.186389167	16.08197407	0.005418529	36.7180389	32235.50548
5.380743725	13.08037767	0.003266042	36.75187718	31790.46995
5.157240477	12.50228553	0.004864433	31.83935374	20307.77549
5.228966969	13.07340205	0.003983036	31.25008218	22371.00515
5.243765593	14.85020786	0.004075555	31.79423309	22076.55313
5.299961088	12.38809825	0.003687967	34.79072834	26188.59617
4.960750768	11.76599246	0.006775565	21.11476146	12976.13485
4.845888271	15.45456124	0.007700188	17.83686429	8120.684837
4.861311844	15.43349943	0.01011615	19.06705025	13450.21155
5.395226551	17.4675586	0.003048036	40.75730805	37995.4668
5.132928355	14.10331178	0.00427782	28.56805591	17811.85625
5.234495728	10.56094315	0.004708506	36.5724609	31633.36941
5.260297785	15.68394121	0.00365971	33.37064635	22075.75053
5.098677927	14.14261749	0.004529868	25.44083174	14002.79118
5.449097099	16.46519452	0.003681136	49.15029976	46457.36301
5.408403518	17.47513042	0.002915217	39.47347754	33479.57931
5.242258421	17.66407815	0.004053057	30.01439488	23578.46295
4.836968282	17.27310096	0.010202799	31.84225297	22324.85917
5.012448471	11.34984203	0.00511783	21.60862293	12190.87433
4.854270169	14.57729285	0.00821484	18.1349045	9987.792078
5.299897722	12.35490187	0.003930335	36.73130101	30824.12151
5.516914094	19.10736873	0.002650643	45.57669404	46205.39704
4.558084044	12.86906901	0.011090614	16.12782424	5347.946111
4.611065014	13.20469283	0.011999508	27.54959803	16509.99505
5.138148712	16.22458499	0.006334499	26.96789987	23225.82279
5.272540155	11.06606466	0.004078561	40.96897434	33782.19246
4.846629976	11.09854991	0.008722969	18.03859061	8824.636038
4.95663056	12.52129215	0.006586552	20.0531618	11915.4576
4.883172174	11.88944521	0.006897279	17.79405214	8797.964518
5.567081704	18.47574092	0.004890767	69.87748354	97209.35712
5.134699038	17.21312477	0.004767773	26.27523185	17773.00236
4.799489096	13.35209237	0.009519108	35.62501323	24131.1995
4.994925593	12.64150495	0.007132164	22.32431447	13490.54671
5.221593475	17.55209541	0.004934092	33.84689507	33382.39903

log.sigma.3.5.mm.3D_glcm_SumAverage	log.sigma.3.5.mm.3D_glcm_Imc2	log.sigma.3.5.mm.3D_glcm_Imc1	log.sigma.3.5.mm.3D_glcm_DifferenceAverage	log.sigma.3.5.mm.3D_glcm_Id
31.91367185	0.899207232	-0.185858308	4.361945185	0.305489976
27.17906741	0.815860733	-0.13654783	3.284039871	0.401046522
19.83538127	0.891227249	-0.190687355	3.801423491	0.323285666
34.39371538	0.73716318	-0.095107282	4.362134104	0.310353017
32.82222519	0.96754339	-0.296373574	4.517720469	0.297183842
22.56224946	0.803533331	-0.131586511	3.516066904	0.33584478
19.5829662	0.890051956	-0.212530898	2.72722318	0.395229514
33.47664696	0.863200379	-0.148083879	5.305210757	0.263419088
35.60182225	0.794738708	-0.113195571	4.63715175	0.298325347
26.36714483	0.747599393	-0.105344728	3.270491451	0.372603906
28.05428031	0.800427369	-0.126670476	3.939976766	0.31629005
26.77048711	0.818658438	-0.134615749	3.76896125	0.33557947
23.01683884	0.793081474	-0.129027258	3.234586123	0.364285729
24.24204398	0.818254393	-0.134226775	3.738753008	0.326756454
26.52991184	0.825996458	-0.137875047	3.775508107	0.33048139
27.36043451	0.839473008	-0.17353272	2.120172302	0.529492876
28.20478619	0.878238587	-0.169300622	4.525590531	0.291854181
23.34970418	0.794655514	-0.122735378	3.868281474	0.341901151
30.35079186	0.921570391	-0.206546971	5.058103002	0.276339769
19.43584849	0.765761098	-0.121462733	2.658057667	0.433221653
29.76722933	0.689842649	-0.091243485	2.715745165	0.418158663
24.74972099	0.789088577	-0.11824629	3.892339578	0.322164461
26.55312073	0.756153051	-0.107880344	3.418962168	0.35918046
37.53484571	0.909761924	-0.201539283	4.180485811	0.308512678
20.01759597	0.722328337	-0.100719003	3.061299556	0.379885253
26.24515871	0.872193013	-0.162642324	4.585447846	0.30632244
32.19724716	0.777506826	-0.128617713	2.479373238	0.460561544
24.11530017	0.787763519	-0.123867987	3.410468268	0.360168703
30.91292126	0.797868666	-0.122731111	4.516557653	0.299652409
28.70313448	0.81784805	-0.137322393	3.171910637	0.410300305
27.2766753	0.714622685	-0.097142727	3.137785869	0.378826727
25.71848634	0.762885858	-0.116843807	3.20622815	0.390068162
28.57370505	0.782797636	-0.112554452	4.051873899	0.322012058
30.1091736	0.99274123	-0.487888065	5.432545695	0.261077418
33.54267387	0.953762515	-0.26689737	4.981094941	0.276647307
31.06852981	0.769666355	-0.109223073	4.020470161	0.320568892
22.66409805	0.7656859	-0.109858831	3.808744154	0.349760079
24.58899594	0.739487366	-0.10459257	3.013991578	0.381727847
31.00307363	0.854522515	-0.167584516	3.778783265	0.335711565
25.67465312	0.825282387	-0.13420922	4.03751241	0.322552142
29.20094826	0.812168539	-0.133242431	3.306351189	0.374678288
31.54510003	0.86088518	-0.151429376	4.61477508	0.29100269
29.50828661	0.779509788	-0.113467785	4.272586298	0.304037708
30.63876127	0.862861194	-0.1513967	4.714568087	0.290730626
33.5215412	0.744590088	-0.100198582	3.91226986	0.321669751
16.10641958	0.702972189	-0.10118584	2.60116472	0.420751135
29.46143865	0.700375122	-0.084961709	3.794272431	0.329932047
22.93713236	0.860468543	-0.1581647	4.151683381	0.313358171
35.89276454	0.988688008	-0.43382461	5.485857303	0.267928623
37.27841213	0.943884349	-0.239018086	5.004477617	0.284585974
25.70240202	0.750920643	-0.107718013	3.230119474	0.378459285
24.00184737	0.817325928	-0.137605487	4.27835751	0.3163684
31.45770861	0.728280524	-0.096833878	3.905015925	0.3452973
23.85650004	0.851237301	-0.158937679	3.307389968	0.356958081
22.45642162	0.822672869	-0.14999775	2.786793095	0.460384085
29.31530216	0.855649953	-0.145419197	5.085220665	0.275006746
34.75365185	0.777792984	-0.109068904	5.418871969	0.263738377
34.93298425	0.814828414	-0.127589386	4.650241316	0.298214622
26.45435561	0.858373909	-0.158183223	3.625592143	0.34237231
22.11382641	0.808767893	-0.142504212	2.974681171	0.37018178
23.51585204	0.768523466	-0.106806759	4.178988131	0.321748096
31.34110576	0.755160516	-0.102811533	3.993311543	0.339397868
25.61364898	0.764305463	-0.124634518	2.64025016	0.433687006
29.33179711	0.802144913	-0.142494551	2.576763923	0.456544876
29.43379728	0.963839803	-0.301863437	4.432376813	0.290847044
31.35050806	0.812855565	-0.126362091	4.180664344	0.328517457
31.82423851	0.939549486	-0.242417373	4.840645529	0.287140554
26.00289089	0.764850555	-0.104437096	4.241597173	0.326490137
24.96577696	0.899547531	-0.191875385	4.319961385	0.298568875
25.92825306	0.775388783	-0.109031344	4.12117111	0.324332232
29.32210636	0.755071671	-0.10740335	4.159498173	0.329745264
24.63759813	0.829279567	-0.134740879	4.352778774	0.30698841
23.44818319	0.733039059	-0.104446979	3.168792255	0.396919626
30.69282237	0.733986003	-0.105504152	2.905920704	0.415490739
30.57593235	0.726050565	-0.101098044	2.9343827	0.419907467
34.3185336	0.852463678	-0.145689429	5.211763057	0.271915688
27.921898	0.813660102	-0.129518415	4.175091387	0.312477698
21.00196526	0.888911459	-0.180189774	4.493616076	0.301920729
30.93091313	0.768434028	-0.112136534	4.529698611	0.300753492
28.08751014	0.721094814	-0.093851243	3.738689808	0.33765727
32.35626	0.939773078	-0.229365364	5.80629199	0.254796335
34.36314793	0.815138783	-0.121706501	5.143681532	0.27054733
34.82360136	0.758364697	-0.104780912	4.159767089	0.330766965
34.28129692	0.977346746	-0.366925489	4.716477207	0.28819852
22.68810073	0.7142062	-0.098397553	3.406748994	0.358718667
28.98688025	0.715596104	-0.099297122	2.914742173	0.406266163
24.56448319	0.869149942	-0.160129823	4.390889662	0.300056858
37.63163523	0.813028519	-0.118887752	5.46302538	0.263330232
25.73813801	0.932222527	-0.266469914	3.252277734	0.358493712
26.40938566	0.987841213	-0.448336768	4.579000956	0.281059808
32.0665577	0.772915211	-0.113483996	3.726206147	0.368935264
21.83722619	0.88242592	-0.168506111	4.930686222	0.284609905
22.19709981	0.816265939	-0.140492753	3.068575325	0.400510819
24.99055443	0.73038209	-0.102666467	3.174877207	0.389207671
23.7684853	0.72962612	-0.102316308	2.90984861	0.400887973
36.04680423	0.984917938	-0.365543436	6.657606023	0.233613651
34.20265153	0.812539016	-0.128861102	3.75864525	0.336064117
26.70418475	0.987226119	-0.425095313	5.311152453	0.261160027
25.2830099	0.910798931	-0.214464325	3.213241375	0.354588491
34.56257619	0.893608908	-0.180916904	4.334432078	0.325544975

log.sigma.3.5.mm.3D_glcm_ClusterTendency	log.sigma.3.5.mm.3D_firstorder_InterquartileRange	log.sigma.3.5.mm.3D_firstorder_Skewness	log.sigma.3.5.mm.3D_firstorder_Uniformity
103.1692039	200.9754391	0.269930722	0.048559895
67.8245241	137.4669685	0.415678114	0.065663807
70.57196469	183.0674419	0.356099068	0.058039032
91.58651257	207.641407	-0.099376823	0.048621354
141.4625304	216.9063034	-0.117874509	0.04632956
58.92531559	179.4241676	-0.048063059	0.061257858
42.47228505	111.2192039	0.183525959	0.084855903
131.7749704	266.8599052	0.003748377	0.04210541
118.4158437	225.4295006	0.168615949	0.043736802
65.28228555	146.2717037	0.567020846	0.065012684
68.24063582	196.8408432	-0.370316231	0.060379074
89.00533234	184.7789545	0.451989523	0.053143343
77.90508797	177.7191563	0.450578456	0.058600815
84.2479606	208.6576233	-0.060550259	0.053568057
75.90820914	166.8987865	0.180651533	0.057464506
41.63363289	68.04923058	0.536238395	0.121429647
104.8737212	220.0006256	-0.145100204	0.048628501
98.61705903	205.5984097	0.509048369	0.050534145
138.5878217	238.3872719	-0.201737057	0.042837236
47.69587808	130.9765854	0.375771857	0.07449971
41.65285997	115.9912727	0.461366239	0.079363724
88.59347567	182.4758778	0.419044927	0.05430503
70.27385509	155.6279144	0.512348979	0.064161416
98.01544894	195.0110092	-0.519571212	0.053712596
42.54654051	133.8755751	0.537774206	0.07535099
93.88227936	199.0491257	0.397902632	0.050199736
43.90939865	96.58992243	0.612605352	0.093006367
68.02071919	158.2692184	0.573833773	0.063073627
112.517196	265.7579956	-0.190131123	0.048025745
64.36931386	140.8134301	0.356254209	0.065247199
54.84720245	143.8532734	0.580528092	0.069843869
73.45426905	163.0651627	0.479971389	0.062635185
97.45589122	215.0603638	0.041308246	0.048440041
118.9608593	218.6064224	-0.10337624	0.058431953
113.4159476	214.8351898	-0.451818834	0.05247496
89.81076016	227.5308437	-0.045718963	0.050238212
90.12687562	201.0722179	0.57865292	0.055365789
59.24530484	160.0038834	0.10001477	0.063442968
77.7979556	136.8543358	-0.367922813	0.064207394
86.62318816	193.6654358	0.014225659	0.052819836
65.81349472	142.8762474	0.231501065	0.062296449
114.0522418	231.974472	0.053013819	0.046184303
90.76595876	225.0183487	-0.216010818	0.051518379
121.1930251	228.2451019	0.056210702	0.044424033
73.3915268	183.7467651	0.05549243	0.054851251
37.15288407	133.060873	0.617108201	0.080585675
77.78145655	179.5296841	0.149841645	0.054374486
85.81613878	221.4329815	-0.07016174	0.052973177
103.7484853	142.5974588	-0.652632256	0.065159278
138.7444242	160.8141937	-0.445492381	0.053896822
66.63506796	163.041626	0.388964319	0.061798861
91.7744338	266.0951595	-0.178181738	0.057422627
79.21032356	184.3263397	0.358242166	0.053738548
63.02492109	143.9046326	-0.223841356	0.065440147
59.93588167	105.6861382	0.482578147	0.0860257
117.3953935	232.1320877	-0.007427973	0.044391897
117.5648545	258.5569305	-0.054482149	0.041900721
100.0038327	187.0359955	-0.510468234	0.053639414
82.98685532	179.1786299	0.225822612	0.055013915
52.99767025	161.8308144	-0.072244887	0.066191164
97.81390175	226.9367752	0.416676489	0.051250055
97.95213237	197.5645981	0.406337152	0.050597758
43.94852789	104.89991	0.371125253	0.084888931
43.45363487	108.0655041	0.450941954	0.084039778
93.54103803	197.0564842	-0.299436076	0.053197137
88.12694079	171.5769043	0.486352495	0.055017209
106.0187383	186.4183846	-0.418454505	0.053917906
112.0233228	235.4120407	0.239400979	0.045928165
96.75218227	230.6491814	-0.253714575	0.053469806
91.00877488	195.1777267	0.434166602	0.052990254
92.53017312	214.9626751	0.389127486	0.052003982
104.7111724	241.5370026	0.132474874	0.047909176
62.90012774	142.1210508	0.569531422	0.066155428
51.93778728	136.1867943	0.431172663	0.070539946
56.09563462	106.6453495	0.589077542	0.078907044
118.9656601	249.6368675	-0.039319644	0.0432163
81.08190704	207.7635956	0.089834553	0.053656801
108.8458319	231.7914658	0.410539379	0.049782317
93.93655583	223.8775969	0.139176616	0.049689944
74.7626564	186.2009239	0.319406682	0.057632885
137.9295569	286.2264566	0.023121676	0.040084089
116.3946268	247.4178238	-0.124968255	0.043787929
89.2648536	188.5441475	0.134673655	0.052592446
87.41246548	152.1891861	-0.737414119	0.073293698
65.92576428	176.9480515	0.374749804	0.05918115
53.48115483	122.3337822	0.627098737	0.076030688
111.5687729	240.772686	0.340497035	0.046500486
137.3051646	264.2246971	-0.456507956	0.043739704
47.15274318	170.133709	-0.041698284	0.071097972
77.42514356	158.5547276	-0.241221321	0.062857143
80.10245732	166.7222748	0.248986172	0.058667743
115.3534634	248.5850058	0.436408495	0.046714122
54.71421813	142.7090044	0.213950882	0.068529532
60.14016377	152.7109938	0.314902753	0.064435172
55.25962127	153.3635559	0.299135209	0.065942213
210.6327956	373.210556	-0.154123688	0.037760315
78.29235266	165.4491615	-0.194471307	0.055549093
99.43765894	199.3814545	-0.294353114	0.055192015
72.69938398	147.2994118	-0.338331819	0.066951636
95.69136132	180.5895042	0.552462959	0.051929446

log.sigma.3.5.mm.3D_firstorder_MeanAbsoluteDeviation	log.sigma.3.5.mm.3D_firstorder_Energy	log.sigma.3.5.mm.3D_firstorder_RobustMeanAbsoluteDeviation	log.sigma.3.5.mm.3D_firstorder_Median
118.1907403	15899761.1	82.96873259	-23.10014534
90.50453633	29463891.72	60.31736409	-19.55072784
100.5972815	7547682.336	73.07374515	-63.34998512
116.1633797	54157025.4	84.62122414	23.6117382
130.0303125	9694154.881	90.1868837	-0.649273336
93.7937381	10793052.63	70.43984905	-56.01986313
71.66525094	2944041.818	46.43525402	-0.520434499
141.6720223	29472479.96	105.4472097	-8.815971851
129.4666621	54693437.34	92.89530034	-32.23179245
90.93445471	43858121.89	62.29434135	-41.40930939
102.3871092	13943093.04	77.86743494	-20.3847084
108.8218016	27712191.83	77.26661235	-37.1979332
100.0860691	31744894.74	72.08320199	4.422829866
110.8201767	18820522.4	85.68673876	11.28655958
99.188227	14402600.3	70.05071603	18.56779099
59.24543842	53545332.71	31.49354725	-0.640004814
121.9327363	16144904.01	90.90619236	-28.95391464
116.4540188	47352122.02	84.55125538	-31.32149124
137.4252754	16431668.43	99.46647935	28.4193449
77.83285314	28053033.05	53.7310863	-11.55288792
73.2124757	53800812.17	49.28226769	-45.01028442
105.9090775	29764832.77	75.43706288	-2.888058662
92.68192446	39067649.13	63.46429552	-39.05645752
112.9064575	9391087.572	80.79893128	33.93927002
79.46647848	15134739.71	55.96435524	-70.64396667
115.2892722	15691255	80.88304276	-57.15114403
68.86509961	31670696.89	42.3294936	-13.60836124
94.22865386	23936849.44	65.67497986	-68.57215118
132.2632167	40859744.94	105.4125474	15.00448227
91.13519915	41063571.19	61.47622275	-47.82403183
85.24339734	41053488.5	59.63084616	-54.858284
95.25381827	85101564.14	66.58628331	-7.723082542
117.6198287	41056629.47	87.20946965	0.519171417
123.8652373	2485268.446	87.72185071	0.718350357
124.1435453	7775326.102	89.1830762	2.789013565
117.9663069	39499289.76	90.94436404	-13.0568881
112.3428343	54742333.33	83.05052145	-49.47209358
90.12356349	40323390.22	66.21030578	4.73524642
95.45912013	13109140.77	61.44123629	20.9984436
110.6128592	20337762.24	80.09475546	-17.19606781
91.54644479	20600392.27	62.82328755	-24.1593895
128.2595279	23752268.24	94.93551978	-14.91563892
118.7911983	33563535.77	91.89443082	-15.27869606
129.8061074	26138239.45	93.94707047	-6.358855247
104.4246811	30819947.33	75.51271009	-8.986044407
76.1294626	20700853.18	55.210484	-43.79718399
103.4111854	69126181.21	74.47349853	3.818297148
113.6996903	12488787.63	86.84497933	-43.33274078
108.3599057	2916488.693	65.20031786	11.10874557
124.5719606	13773932.53	77.63003208	24.74665833
92.51118806	46114954.65	65.95613805	-11.38094759
126.4654858	20717559.25	105.1818976	-55.1522522
105.8451049	68017813.99	74.61661338	-19.10703659
90.6441945	7545627.98	63.93984708	12.22744179
77.01205605	72626679.13	47.73999349	-4.175802708
129.5430074	22882864.29	94.76768609	-28.9374485
137.9178351	61355241.73	104.5814414	-27.22070122
115.6385304	27780526.89	78.08357673	18.046772
103.717247	14893479.53	73.77417541	-47.24480438
86.95227964	10061849.86	65.20118063	-18.1814003
122.3106071	46555180.38	92.83724708	-46.69099045
112.6827975	109394849.4	79.70537713	1.438794374
70.02069012	27075912.5	45.57040552	10.04157495
72.82246392	26208054.55	47.10449875	-18.36682701
111.1789184	4518428.366	79.5896733	-42.36473083
105.9428932	30590735.41	71.06341218	23.60805321
119.4316569	9546321.704	80.63072918	-13.16117287
126.0575515	74168559.64	93.97191872	-3.290171385
119.7337824	11488018.18	91.8103248	-47.17933846
109.9532458	37135699.93	79.36517013	-22.33327484
116.1980465	60240027.01	86.32282147	-37.30642128
125.4792742	26577071.96	96.09090298	-33.75882339
88.444026	66440335.62	61.37257383	-18.39717007
81.93788496	75371060.07	57.31499436	-30.60664463
78.773162	109611060	48.41678223	11.13140249
135.8948791	31352295.37	102.4248061	-32.78139877
109.8430703	22353057.31	82.45000918	-53.19909286
123.5354981	15720374.64	92.15997023	-60.93788147
119.8492092	43612060.94	90.41776862	-83.83678818
102.1631292	46212472.06	75.78488561	-49.22288322
150.2121614	22008091.04	113.2098615	-112.3798065
133.8431171	34810232.3	100.8651481	-9.926179886
108.5219744	53865458.17	75.8622568	20.29167271
96.77418492	2447777.118	59.83298617	28.17401886
97.99545846	47866766.94	72.15964327	-8.638441086
78.81417021	57656694.31	52.02928298	-15.58317089
128.0974839	19335656.24	97.1525077	-50.0044899
142.9132074	47834972.32	107.3513876	-11.78542948
89.34250805	2082473.329	66.39032468	-19.35981178
102.0211088	2009623.463	68.90604172	-42.70209122
101.7437551	69519223.05	68.59948973	-7.626009941
133.2571629	21665814.95	100.5977306	-83.43078995
85.04429662	12724999.34	58.94575919	-28.107481
90.40933836	53246931.26	64.11318829	-20.21507549
87.70151411	67804878.15	63.5746276	-6.377554417
188.3534733	12726094.97	149.4630961	-34.33631134
103.4438245	20928430.76	70.92889861	21.62358093
118.4412672	2586197.056	83.02712058	19.05496025
90.37470436	5038042.873	61.89702462	30.08002281
114.6536068	19155131.19	76.66810877	-57.20046997

log.sigma.3.5.mm.3D_firstorder_TotalEnergy	log.sigma.3.5.mm.3D_firstorder_Maximum	log.sigma.3.5.mm.3D_firstorder_RootMeanSquared	log.sigma.3.5.mm.3D_firstorder_90Percentile	log.sigma.3.5.mm.3D_firstorder_Minimum
429293549.6	403.8726196	150.0696774	171.4003754	-401.6631165
795525076.4	437.828064	119.104126	145.4237122	-338.625061
203787423.1	298.4096985	131.8748389	118.024157	-292.4881897
1462239686	470.4470215	143.3630037	200.4930725	-379.2749634
261742181.8	342.5074158	164.3265558	227.0431366	-404.4763184
291412421	229.0317383	129.5587237	84.43617096	-336.8908997
79489129.08	269.0066223	93.46672741	133.3375885	-232.9253235
795756959	485.0147705	171.5040893	182.7969299	-445.8373718
1476722808	421.0689697	162.196084	183.1741211	-451.1834106
1184169291	396.5253296	118.9828338	138.8978912	-341.4483337
376463512	220.7605133	129.8454715	100.9165482	-394.9977722
748229179.5	424.6551819	136.7449625	163.6200455	-328.9413147
857112157.9	414.7251587	123.7179086	166.0005081	-273.0422058
508154104.7	362.8363647	129.7462861	163.8859756	-292.3479004
388870208.1	369.0279236	125.7364706	182.8330688	-282.6958008
1445723983	514.4158325	87.79345921	109.2316116	-319.547699
435912408.4	339.0492859	152.0862748	136.0954605	-387.5474854
1278507295	435.8372498	142.496911	185.7548691	-297.4463501
443655047.6	413.4275513	168.0271028	220.7652344	-355.04599
757431892.3	360.6622925	100.0767878	112.3948288	-239.6056061
1452621929	406.6278992	101.4828912	90.46807022	-377.4695129
803650484.8	544.5838623	131.5489845	175.3938095	-299.14151
1054826526	402.677124	119.6045742	141.1591644	-329.8751526
253559364.5	313.7235107	139.728632	174.0332336	-445.8860168
408637972.2	311.821228	112.1177141	81.8134407	-283.9772339
423663884.9	408.0223083	150.3658123	153.5853806	-372.1487732
855108815.9	399.5235901	95.59045541	118.2146339	-388.4485474
646294934.9	376.8725281	129.2440025	129.5856628	-326.9405518
1103213113	352.0997925	152.1512311	189.2178772	-373.1143494
1108716422	511.7611694	127.0985468	103.8755272	-383.2656555
1108444190	372.8220215	113.6396191	108.3591492	-350.2166748
2297742232	483.7572021	119.7957538	176.6264099	-291.2424622
1108528996	407.4346924	142.1094496	174.8838806	-345.9297485
67102248.05	314.7962341	154.5859372	231.5418915	-370.7007751
209933804.8	277.5908203	153.0348226	153.785997	-442.3978882
1066480824	394.8917847	139.8359891	159.2654434	-385.6463928
1478043000	480.5281372	137.8686137	167.7333694	-299.9833069
1088731536	344.1015015	109.6470597	139.7067169	-280.7817078
353946800.8	377.2339783	125.2979241	155.6935669	-362.4506531
549119580.4	385.9680786	139.2399436	133.197879	-342.0080261
556210591.3	445.851532	118.2194687	135.8826355	-368.3245239
641311242.3	436.2225952	157.8723575	164.9345306	-409.1545105
906215465.8	339.9022827	142.9707767	135.8392685	-378.9872742
705732465.1	436.2004089	159.689453	188.6570892	-388.1802368
832138577.8	356.246521	128.105611	146.6504883	-414.6043091
558923035.9	307.378418	96.39100966	110.2191277	-220.0378113
1866406893	401.9526367	127.2948569	177.9411392	-328.6689758
337197265.9	315.0748901	146.3611667	108.269841	-330.1470947
78745194.71	292.4933167	147.5291191	188.0290802	-469.9482422
371896178.4	469.9542236	166.4756738	199.3553192	-457.2107239
1245103775	446.7668457	116.0523703	139.9861618	-314.033905
559374099.9	181.366272	158.2765372	107.3466721	-369.0308838
1836480978	480.763031	133.4205775	160.8037262	-377.0577087
203731955.5	296.1821899	112.9936283	141.9320068	-295.3977051
1960920336	441.0547791	105.5174784	143.1403442	-266.5317993
617837335.7	448.3947144	161.346948	169.7938171	-395.5539246
1656591527	383.29422	167.2655565	176.1896362	-458.7094727
750074226.1	382.9012146	146.5787375	150.0262299	-449.0817871
402123947.3	306.3804321	135.2651393	136.8702652	-364.2460022
271669946.2	251.9352875	106.8688072	109.459549	-277.1587219
1256989870	432.6256714	148.2239998	162.6608154	-321.545166
2953660933	609.6795654	141.3147268	181.4140579	-359.4311829
731049637.5	446.8083496	93.60785881	131.6769745	-286.7453308
707617472.8	314.038208	97.96175161	123.6837234	-359.3283691
121997565.9	221.7228088	144.2991702	130.5517578	-400.550415
825949856	548.0008545	140.3489704	216.1601868	-333.836792
257750686	361.6017761	155.6574537	121.8917587	-441.1673279
2002551110	434.9758606	152.3135724	198.8310242	-324.9848022
310176490.8	271.2255554	160.4926425	85.48153687	-377.3991699
1002663898	426.8105469	135.8907112	179.7609406	-321.2826538
1626480729	457.7546387	140.7685986	183.4218582	-370.4179688
717580942.9	401.3126831	153.4286964	150.0520935	-347.6714478
1793889062	435.9576416	113.3847525	141.265184	-286.3477173
2035018622	359.7675171	105.9368287	122.6341019	-390.9775696
2959498621	520.3554688	109.2712342	157.9745071	-335.6824341
846511974.9	465.7705078	167.6110218	158.6988068	-462.1032104
603532547.4	422.5455017	141.4626793	109.3225037	-376.4461365
424450115.3	365.5327148	160.5338157	136.9853485	-312.7757263
1177525645	301.3792114	157.2367341	135.9888809	-440.4647827
1247736746	395.4104309	127.4272036	140.3012085	-350.6096191
594218458.1	335.0038147	208.9661391	123.0920204	-523.1864624
939876272.2	457.8464661	161.1161486	179.3277893	-446.4527283
1454367371	467.3836365	138.8984733	202.6158463	-397.2217407
66089982.19	262.3301392	129.9277549	182.002063	-401.9523315
1292402707	394.3052673	118.8100429	168.015213	-262.8456726
1556730746	439.5493164	102.610818	138.7282028	-349.6532593
522062718.5	398.254425	157.6484335	164.5819763	-331.3960876
1291544253	353.2432251	175.5605388	158.41418	-505.7018127
56226779.89	207.0786591	106.6753752	134.9022369	-310.471344
54259833.51	232.4588928	138.3447544	79.68290405	-377.7282715
1877019022	506.1181335	132.2464091	168.3025208	-381.7116089
584977003.6	431.1196899	175.180259	160.8914185	-346.5057983
343574982.1	305.8287354	111.5843881	121.1081184	-298.6387329
1437667144	393.9057312	114.0999982	121.2008347	-319.4382935
1830731710	374.6394653	108.066716	131.1496353	-280.0610657
343604564.2	372.2716064	224.278333	214.3937317	-502.568512
565067630.4	332.1399841	131.1902457	186.3400574	-389.1488647
69827320.5	270.4568481	145.0034218	183.6419434	-335.7928467
136027157.6	278.335022	115.2952259	167.0836121	-275.7934875
517188542.2	530.0800781	154.065642	155.3095306	-471.6367188

log.sigma.3.5.mm.3D_firstorder_Entropy	log.sigma.3.5.mm.3D_firstorder_StandardDeviation	log.sigma.3.5.mm.3D_firstorder_Range	log.sigma.3.5.mm.3D_firstorder_Variance	log.sigma.3.5.mm.3D_firstorder_10Percentile
4.563339713	148.2166697	805.5357361	21968.18119	-220.6200714
4.218545776	117.8178189	776.453125	13881.03844	-170.1262207
4.264850509	122.3432797	590.8978882	14967.87808	-203.5516739
4.527258682	141.8028766	849.7219849	20108.05582	-168.8174255
4.647530877	164.1817577	746.9837341	26955.64955	-235.7626953
4.147764995	112.0818997	565.9226379	12562.35224	-214.2477325
3.860008711	93.46375687	501.9319458	8735.473849	-118.3852966
4.738402204	169.9613828	930.8521423	28886.87166	-259.380069
4.685590343	159.1637461	872.2523804	25333.09806	-239.3235809
4.197937222	115.4149013	737.9736633	13320.59944	-160.0537506
4.202179579	121.8391039	615.7582855	14844.76724	-225.3321167
4.429799658	134.7396929	753.5964966	18154.78484	-188.0520004
4.275640833	123.5909654	687.7673645	15274.72674	-146.451152
4.342180787	129.7458544	655.1842651	16833.98673	-181.2541504
4.314614704	123.443774	651.7237244	15238.36535	-139.9850159
3.637095088	87.58038205	833.9635315	7670.323319	-95.29413452
4.512647558	145.704224	726.5967712	21229.72089	-256.8878784
4.475458235	141.9363909	733.2835999	20145.93907	-183.6177567
4.695899853	167.6741681	768.4735413	28114.62666	-232.9166855
3.980114406	99.41113652	600.2678986	9882.574064	-143.9457092
3.928577259	94.25979109	784.0974121	8884.908215	-148.6437683
4.384753181	131.4908928	843.7253723	17289.85488	-166.2128326
4.224082102	117.2921568	732.5522766	13757.45005	-159.2625122
4.436806862	139.1156936	759.6095276	19353.17621	-176.7894745
3.963199931	98.43619216	595.7984619	9689.683927	-169.5812958
4.509793645	143.9606578	780.1710815	20724.671	-223.7288208
3.862337913	95.11715513	787.9721375	9047.2732	-119.257885
4.194504154	118.3616713	703.8130798	14009.48524	-192.9440887
4.50504367	152.1423482	725.2141418	23147.29411	-205.5907654
4.228994666	117.213559	895.026825	13739.01842	-197.8018799
4.078931513	106.6556363	723.0386963	11375.42474	-162.0294891
4.246349814	119.2846345	774.9996643	14228.82403	-132.5401535
4.516086288	142.0870098	753.3644409	20188.71834	-192.9739441
4.340071541	153.742612	685.4970093	23636.79075	-173.5129349
4.502546368	151.0702175	719.9887085	22822.21062	-238.4094284
4.448992743	138.4802813	780.5381775	19176.78831	-207.6246002
4.375352893	135.7270514	780.5114441	18421.83248	-183.1412903
4.146374048	109.6437653	624.8832092	12021.75526	-148.3045578
4.28103917	124.9615277	739.6846313	15615.3834	-166.1464417
4.425107943	135.9789392	727.9761047	18490.27189	-224.0731293
4.246230497	116.5253641	814.1760559	13578.16048	-162.6357407
4.597955849	155.5177468	845.3771057	24185.76956	-239.2124115
4.418367094	138.973949	718.8895569	19313.7585	-229.1658539
4.662197499	159.0594772	824.3806458	25299.91728	-234.7575439
4.372147386	127.4643728	770.8508301	16247.16634	-179.3965164
3.834916053	92.92688741	527.4162292	8635.406404	-134.3647842
4.373507564	127.1243639	730.6216125	16160.60389	-160.2819443
4.358549756	135.007477	645.2219849	18227.01885	-251.336734
4.330798228	147.4886723	762.4415588	21752.90846	-206.8063156
4.600486956	166.402276	927.1649475	27689.71747	-256.0308441
4.220360128	115.5756551	760.8007507	13357.73205	-155.190477
4.244618921	142.0646705	550.3971558	20182.37062	-266.1382629
4.425866874	132.7433647	857.8207397	17620.80086	-180.8734283
4.146547755	112.9865733	591.579895	12765.96576	-156.1233063
3.975888212	105.4941007	707.5865784	11129.00528	-143.1723846
4.640041262	157.4786739	843.9486389	24799.53272	-252.5334259
4.70860597	163.845509	842.0036926	26845.35082	-252.987677
4.510768451	146.2889564	831.9830017	21400.45877	-219.7663452
4.365987274	128.4200574	670.6264343	16491.71114	-206.4505951
4.061064423	104.0744168	529.0940094	10831.48423	-166.4991302
4.465316782	144.7383664	754.1708374	20949.1947	-201.7688477
4.512085252	141.1844137	969.1107483	19933.03866	-172.0304779
3.88324171	92.86561343	733.5536804	8624.022158	-113.1278343
3.900605965	96.49995819	673.3665771	9312.24193	-136.0467529
4.385266926	137.1519093	622.2732239	18810.64624	-238.3240173
4.446593538	136.3325201	881.8376465	18586.55603	-134.0668335
4.515527231	151.2626318	802.769104	22880.38379	-257.3636139
4.576873957	152.2508429	759.9606628	23180.31918	-207.8618042
4.385223437	141.4878817	648.6247253	20018.82067	-292.6106567
4.425880313	135.5280575	748.0932007	18367.85436	-173.47229
4.44686497	139.9220897	828.1726074	19578.19118	-182.0430359
4.521288918	148.4216595	748.9841309	22028.989	-234.0785309
4.152441664	112.4988776	722.3053589	12655.99747	-153.5933365
4.041489878	102.8800473	750.7450867	10584.30414	-152.1595993
4.062554378	108.1255484	856.0379028	11691.13422	-116.6507431
4.67661743	162.3280362	927.8737183	26350.39133	-262.1897125
4.391778097	131.6339456	798.9916382	17327.49564	-221.7064941
4.478125463	149.8266795	678.3084412	22448.03389	-246.2988876
4.487911798	142.7758868	741.8439941	20384.95386	-239.1129135
4.292297038	122.9158611	746.02005	15108.30892	-180.9791107
4.79175946	179.5676258	858.1902771	32244.53224	-340.7454651
4.657583902	159.7877414	904.2991943	25532.12231	-239.2433472
4.471083709	136.9530544	864.6053772	18756.13911	-157.2731247
4.125256979	129.0332764	664.2824707	16649.58642	-165.6636169
4.243871362	118.7775999	657.1509399	14108.11823	-141.4015656
4.024461834	102.5768986	789.2025757	10522.02012	-120.8658295
4.54970816	152.4952479	729.6505127	23254.80065	-234.9407425
4.6918791	170.8195107	858.9450378	29179.30522	-291.5856903
4.007747645	106.5959003	517.5500031	11362.68595	-137.6082581
4.217113074	127.8683202	610.1871643	16350.30732	-240.7061249
4.39937907	132.0288935	887.8297424	17431.62871	-174.6981049
4.571325888	159.6740505	777.6254883	25495.80241	-271.9448547
4.109911767	107.6391826	604.4674683	11586.19362	-167.0286545
4.181745618	112.2360911	713.3440247	12596.94015	-158.4155746
4.112115585	107.6673249	654.700531	11592.25285	-148.643486
4.865391857	215.5313164	874.8401184	46453.74834	-358.4254944
4.395446609	130.4414391	721.2888489	17014.96904	-162.9328995
4.336801748	144.9746822	606.2496948	21017.65848	-193.266629
4.157261794	113.8698668	554.1285095	12966.34657	-144.5322266
4.547595177	148.2235082	1001.716797	21970.20837	-223.7524963

log.sigma.3.5.mm.3D_firstorder_Kurtosis	log.sigma.3.5.mm.3D_firstorder_Mean	log.sigma.3.5.mm.3D_glrlm_ShortRunLowGrayLevelEmphasis	log.sigma.3.5.mm.3D_glrlm_GrayLevelVariance
2.834288173	-23.51014422	0.007283418	35.44454908
3.245286527	-17.45721615	0.008847735	23.66998278
2.546137357	-49.22494334	0.030533201	24.26984073
2.514975279	21.09253413	0.00601635	32.68416115
2.560107402	-6.896911609	0.011401312	43.30000475
2.195819556	-64.98546479	0.01482559	20.21654924
3.165034583	-0.745173709	0.021709268	14.27299662
2.394009712	-22.9517101	0.007540784	46.05999903
2.597390853	-31.2165278	0.005337353	41.2084752
3.215882548	-28.91911659	0.009233599	22.06258066
2.173840992	-44.88963389	0.008883258	23.91276868
2.894384715	-23.3323795	0.010732997	29.57517093
2.861899416	5.603048855	0.015832362	24.70320126
2.130783636	-0.334685821	0.018295426	27.0095104
2.710878089	23.90177187	0.011034028	25.02651499
5.605301412	6.112950183	0.006699467	14.18566818
2.307197559	-43.59488604	0.012237142	33.89229313
2.73314049	-12.62658228	0.018968817	32.65188999
2.350910748	10.88487966	0.015904817	44.88773258
3.14182211	-11.52342811	0.018378179	16.5777294
3.462968959	-37.60144943	0.005225701	15.0363468
3.028429259	3.909021783	0.013409431	27.87356641
3.170317668	-23.40521551	0.008400834	22.48177138
2.953928279	13.07342253	0.007152137	31.50657384
2.977894523	-53.67213331	0.01613631	16.0801255
2.789032774	-43.41896488	0.013346271	33.84543481
4.376838325	-9.500629758	0.004566936	16.15587266
2.877773922	-51.90883284	0.010420469	22.69897463
1.923913617	1.644087205	0.009846471	37.02398344
3.586210135	-49.1428751	0.007470824	23.63748194
3.198579714	-39.22420547	0.006782758	18.64928374
3.015482068	11.05434691	0.010987728	23.67294423
2.426143399	-2.525333957	0.012555788	32.67251658
2.544134831	16.12517404	0.024692519	37.3894443
2.593088411	-24.44271504	0.010576855	37.20337447
2.164918481	-19.42461165	0.007380908	30.78086855
2.766154873	-24.20582882	0.019021564	30.0352058
2.522903352	-0.849964886	0.011465057	19.61003198
3.461398172	9.175313635	0.010482504	25.59210688
2.543683543	-29.95813761	0.01415911	30.01678812
3.115105971	-19.94197361	0.007759999	22.60097872
2.419801736	-27.16453008	0.008820352	38.83009282
2.116835677	-33.56910006	0.009145703	30.81092795
2.498313207	-14.17053735	0.010296256	40.8009097
2.636744448	-12.80161023	0.005466934	26.45481297
2.764209154	-25.60898941	0.025514461	14.24289648
2.604029167	6.586098981	0.007688981	26.21534701
2.156232643	-56.52054742	0.021569286	29.03624706
3.712055871	-3.45434975	0.018460491	35.58133736
3.357746962	4.942924478	0.009514227	45.20261757
3.019544792	-10.50812041	0.009598309	22.15336937
1.659739384	-69.77887646	0.018095201	31.76425305
2.990722633	-13.42570841	0.005943791	28.99821274
2.659084815	1.262647017	0.015008998	20.37194633
3.604260774	-2.221026266	0.013683224	19.26290642
2.455035038	-35.11844127	0.01443156	39.70255476
2.231446343	-33.65138314	0.006373564	43.08186585
3.091606566	-9.212356992	0.009182704	34.98586727
2.592248634	-42.48466499	0.01074657	26.98631558
2.283014643	-24.27875045	0.014617304	17.46134712
2.442241465	-31.95558506	0.017537981	33.73769988
3.308088699	6.06740156	0.006769368	32.70979748
3.851943327	11.76473853	0.007966097	14.7568325
3.434076413	-16.86009631	0.005699787	16.13659103
2.534819277	-44.8509119	0.01238736	29.97058438
3.34331778	33.33582849	0.007384058	30.63613205
2.987876272	-36.72681731	0.015578147	37.2550549
2.391192889	-4.370946201	0.014998243	37.42085775
2.140998963	-75.75663414	0.016511867	31.70106283
2.726900906	-9.921241535	0.013536377	29.84331171
2.435941734	-15.41451204	0.007794472	31.51114686
2.226132698	-38.87641811	0.019432469	35.26324307
3.247615866	-14.14583413	0.011173027	21.1929587
3.032495486	-25.26474901	0.005036238	17.70981344
4.234561675	15.78190106	0.005951357	20.16023768
2.406235619	-41.75000953	0.007119652	42.22224112
2.593518943	-51.80920751	0.010612872	28.12251747
2.535054461	-57.64609336	0.03333142	35.65436054
2.301976151	-65.86681029	0.010098889	32.84403443
2.476312749	-33.60629862	0.00794719	24.50135637
2.218090114	-106.8752313	0.013619133	51.69722892
2.309973977	-20.64681668	0.0077292	40.78589348
2.802593768	23.16563757	0.005267918	30.70621
3.600478926	15.21956264	0.012130594	27.20362153
2.581380412	2.776337916	0.015302309	22.76482884
3.715719105	-2.638152662	0.006160346	17.83052375
2.331178978	-39.9778431	0.016462413	37.0760957
2.339638332	-40.52403682	0.005857582	46.44617273
2.429339143	-4.117004762	0.013913983	18.59190117
2.786808743	-52.81064064	0.018500572	26.53427857
3.211465093	-7.581820777	0.006737587	29.42948422
2.459667674	-72.05775978	0.034604795	41.07732929
2.802086783	-29.40887711	0.01971768	19.57460675
3.106256918	-20.53946069	0.011693563	20.92625187
2.797187045	-9.282363264	0.010608701	19.14963586
1.876961777	-62.02436853	0.015480825	73.52363475
2.859938149	13.99683954	0.007870581	27.80482323
2.591998049	2.886838807	0.029262104	33.04052448
2.812707052	18.07325482	0.015025551	20.96515195
3.685394672	-42.02396547	0.006158874	36.45865547

log.sigma.3.5.mm.3D_glrlm_LowGrayLevelRunEmphasis	log.sigma.3.5.mm.3D_glrlm_GrayLevelNonUniformityNormalized	log.sigma.3.5.mm.3D_glrlm_RunVariance	log.sigma.3.5.mm.3D_glrlm_GrayLevelNonUniformity
0.007631004	0.04822384	0.074745619	31.88328099
0.009521282	0.061421188	0.202595341	112.1661126
0.032152806	0.057859016	0.064720706	23.68947481
0.006288001	0.048260056	0.09074393	118.1586797
0.01185024	0.045916264	0.080943169	15.50704526
0.015670444	0.061299348	0.089949024	36.59340314
0.023165345	0.083199742	0.116633012	25.51478377
0.007814569	0.042034823	0.05590083	40.05166409
0.005581967	0.043447046	0.080407058	84.51106716
0.009985456	0.063526034	0.12471667	178.5724096
0.009266845	0.060120866	0.075391815	46.58300392
0.011432362	0.052562176	0.094518263	71.90378125
0.016802043	0.05804133	0.11648868	109.9368234
0.019248791	0.053538295	0.086379697	55.76948532
0.011586234	0.056733528	0.086522684	47.88375573
0.007634045	0.100913395	0.516885099	558.9381392
0.012832763	0.048680156	0.067992434	32.03361142
0.020527537	0.049908504	0.112469129	106.8155905
0.016630377	0.042845396	0.0621047	23.55905008
0.020402913	0.071412638	0.233348765	174.0402851
0.005734217	0.07625674	0.194242554	348.9776274
0.014192145	0.0541156	0.087335528	86.66798786
0.009031624	0.062945721	0.112324239	157.5515812
0.007342863	0.052921672	0.076092685	23.78449883
0.0173132	0.073196974	0.11899275	80.2509165
0.014067795	0.04959792	0.080571519	32.13566883
0.00508567	0.081365803	0.351801919	235.3851738
0.011182557	0.061963955	0.114357236	81.53435224
0.010229764	0.047705703	0.075446539	79.23440259
0.008105275	0.06121032	0.235446555	135.2792201
0.007331034	0.068279041	0.130744505	196.8044961
0.011937477	0.060230418	0.172101702	318.7103304
0.013132499	0.048211948	0.08747414	91.03150788
0.025485519	0.057999738	0.045199786	5.778057186
0.010827724	0.051788872	0.057935259	16.31784556
0.007746439	0.050025028	0.090590685	93.75341448
0.020332097	0.054411717	0.117245506	143.6005787
0.012278791	0.062641583	0.136664962	189.8450257
0.010942325	0.062856219	0.110046668	48.30430662
0.014976469	0.052079261	0.089731259	50.88098692
0.008298482	0.060815833	0.14370545	80.72784394
0.009212138	0.045961633	0.068365414	41.22262046
0.009531058	0.051254645	0.078471633	78.93463892
0.010727279	0.044206405	0.077582063	42.56441429
0.005725146	0.05430541	0.092886248	94.64711229
0.028128922	0.078164681	0.159585469	155.253073
0.00811867	0.053908349	0.095091173	212.7448069
0.022864414	0.052934323	0.076697805	28.90648389
0.019063783	0.063777023	0.054154785	8.134410673
0.009870895	0.05248597	0.07120271	24.55543732
0.010334639	0.060347513	0.157215825	185.7906249
0.019369144	0.056765341	0.098312442	43.54371401
0.006347702	0.052600699	0.137804403	182.510599
0.01607998	0.064600536	0.101348843	35.05516289
0.015074395	0.07759973	0.283011026	432.8211936
0.014990856	0.044237395	0.060925851	36.79042178
0.006620313	0.04190298	0.062987249	87.05132413
0.009496228	0.052540385	0.083699779	63.36612408
0.011332009	0.054093228	0.093137492	40.71406224
0.015621402	0.066219479	0.112876488	53.35095073
0.018726149	0.050348941	0.094609661	98.80956865
0.007166858	0.049859726	0.119948173	250.0980605
0.008672763	0.080466483	0.226822458	217.649743
0.006270369	0.07762502	0.290743238	179.9518172
0.012846471	0.053313998	0.05863864	10.96564337
0.007751317	0.053832058	0.100391892	77.26139633
0.016421563	0.053218999	0.074027526	19.73901781
0.016041787	0.04562938	0.103318527	134.7564363
0.01748279	0.053370284	0.066056084	22.43501814
0.014261999	0.052458224	0.091323795	97.98762517
0.008332459	0.051477864	0.109696586	144.0647652
0.020804971	0.047721364	0.084049458	50.28232749
0.012164743	0.064103343	0.176016053	294.9391959
0.005530812	0.068531354	0.214101878	403.541923
0.006451565	0.073060764	0.225643989	583.1770645
0.007376817	0.043214728	0.066038563	45.57214891
0.011055021	0.053407225	0.084228549	55.6220192
0.035772367	0.04931569	0.077835911	28.21892453
0.01057853	0.049219312	0.082717961	81.03858042
0.008441217	0.056991554	0.100417755	149.5647652
0.013999483	0.039909303	0.064269605	19.05108809
0.00796817	0.043734881	0.055003566	55.70373683
0.005535095	0.051587191	0.108094548	132.849017
0.012299492	0.070868634	0.060380439	9.777378474
0.016377635	0.058776809	0.114550979	182.8173531
0.006713476	0.072796088	0.191141747	350.6966623
0.017410683	0.046287057	0.075163627	33.89421488
0.006098276	0.043633967	0.060810374	64.1874098
0.014358913	0.069556946	0.089285991	11.80239994
0.018775793	0.062583453	0.0428382	6.340246415
0.007186555	0.054814228	0.1883324	194.4042121
0.036442188	0.046226937	0.062987751	30.85425432
0.020879072	0.064318934	0.185135697	58.06365346
0.01250308	0.062521057	0.184797092	227.4004252
0.011494628	0.06448408	0.168224242	333.6890594
0.016058512	0.037763026	0.048290077	9.147008193
0.008227479	0.054591609	0.09089922	61.40873172
0.030747526	0.055410541	0.045714179	6.521181359
0.015976365	0.065829096	0.099840628	23.0020759
0.006430579	0.050740228	0.095741157	37.85817547

log.sigma.3.5.mm.3D_glrlm_LongRunEmphasis	log.sigma.3.5.mm.3D_glrlm_ShortRunHighGrayLevelEmphasis	log.sigma.3.5.mm.3D_glrlm_RunLengthNonUniformity	log.sigma.3.5.mm.3D_glrlm_ShortRunEmphasis
1.216284935	301.987061	583.3823631	0.952065144
1.502389707	199.9620319	1464.386245	0.914044023
1.191153454	132.4661617	366.5410582	0.956867292
1.251276147	314.9025565	2137.085309	0.947465739
1.215446976	324.9694805	302.7481229	0.957145921
1.251983431	155.5650786	519.793583	0.946702823
1.33260473	117.9137348	257.4907727	0.931026066
1.162399486	344.8692323	865.0775532	0.963115956
1.224142087	359.7702685	1717.966147	0.952272869
1.341796554	192.0432903	2354.390161	0.931525108
1.217692473	229.4253446	685.2892835	0.952319709
1.271980944	205.1971265	1176.54367	0.941561246
1.319259122	153.9311412	1606.135996	0.935910713
1.239986355	174.4337415	913.963705	0.949588533
1.254500141	196.9829168	729.6491038	0.943680134
2.118753802	178.9012499	3873.541433	0.859616907
1.195829795	241.1582555	588.6553964	0.956813489
1.30265628	172.2729428	1835.575972	0.94030681
1.184782017	287.1747993	492.7648607	0.957568099
1.562744603	110.5117106	1932.210893	0.90880171
1.502927544	223.2463401	3628.871301	0.909747565
1.244956611	181.2423095	1403.354805	0.948519265
1.305939119	199.3059805	2134.5491	0.938342437
1.223711978	370.7194796	394.776605	0.9500953
1.328651728	119.03025	923.5002374	0.93355534
1.230719489	217.4826648	569.2520877	0.94990932
1.797801475	248.8035487	2154.566633	0.885455245
1.306161859	172.5459937	1128.704729	0.940055472
1.208287401	265.5801964	1486.60442	0.956693543
1.566760291	215.4607618	1746.971651	0.908385126
1.351503093	204.8117658	2415.115742	0.93131367
1.433152799	181.6412148	4341.901967	0.922942537
1.248488142	227.5876311	1643.468664	0.946584678
1.13600194	287.9024542	91.54317741	0.967752794
1.170370951	331.775479	284.7759603	0.961146287
1.255105466	263.225016	1630.438115	0.946042101
1.312339772	157.5538918	2256.180979	0.938952358
1.364694482	164.5451423	2527.13774	0.929548057
1.299928173	259.6751426	662.0097508	0.940838214
1.244821385	199.7443181	858.0143273	0.949824426
1.382877255	224.296252	1100.849149	0.926971896
1.198601912	296.8705567	798.5024437	0.955524283
1.218092795	248.3109684	1368.963532	0.954198352
1.212652223	283.4995492	858.6674487	0.95574232
1.25671714	299.4690991	1519.278452	0.946933691
1.425786399	83.79014158	1619.827643	0.919918733
1.265421672	232.4719828	3413.183866	0.944180575
1.218200422	174.6719499	483.3329129	0.952948337
1.162151333	391.9992846	116.1901529	0.962957525
1.202596447	412.787611	416.9305038	0.955686578
1.39806376	183.1075983	2560.838303	0.928303006
1.265529426	185.0968523	670.9996961	0.947395001
1.355558076	269.6222931	2924.861597	0.933546379
1.290078677	167.9647491	462.3067834	0.938304584
1.66800968	137.6890146	4303.531662	0.898068553
1.17923051	259.2198575	747.7021089	0.959181957
1.179654144	360.55578	1876.980782	0.960598006
1.235921976	343.7480991	1058.442022	0.949660037
1.264007561	209.1433173	649.4547638	0.943473052
1.31549971	142.995552	685.2115258	0.935897782
1.261853857	178.962506	1704.003411	0.945658722
1.31628892	265.6802659	4283.989924	0.938756375
1.552965792	169.3725018	2162.237911	0.910782669
1.689091809	213.1078918	1771.004604	0.894368416
1.173917052	270.0711178	185.7591936	0.960312449
1.275597905	267.8717126	1241.582639	0.943790507
1.206279218	314.8647077	331.4458029	0.956218343
1.277137409	207.9662386	2559.986883	0.944781412
1.193116192	208.3102414	375.5012094	0.956580196
1.252595247	195.3614128	1630.964241	0.947576004
1.293611573	243.6616031	2413.052619	0.942134931
1.234805109	196.4671683	927.6703896	0.95063165
1.446043548	155.1541308	3761.418106	0.921120954
1.521507432	241.1171838	4714.004596	0.913184615
1.556023349	227.6198765	6291.888911	0.907304893
1.187966598	345.8295758	947.626842	0.958751259
1.237592534	225.8010315	914.8129033	0.94958816
1.216224465	159.4200984	508.391635	0.95443148
1.233563818	274.4803308	1448.411077	0.950248542
1.27891009	215.2848811	2258.297917	0.941989101
1.1821451	342.4631871	431.5605643	0.960600735
1.164427502	339.3615798	1151.682624	0.961544952
1.287445826	316.7774136	2226.85426	0.943436944
1.17360305	344.0165886	125.3664559	0.962409034
1.306681902	151.4050812	2665.874887	0.939896945
1.487742891	210.4176386	3854.625324	0.913350565
1.205155919	200.5393809	655.5261541	0.957439871
1.175310841	425.7521613	1328.623091	0.96089547
1.257847187	187.4171528	147.3161922	0.94470346
1.11891974	229.9797429	94.84831519	0.974195796
1.451317859	273.5048661	2926.795641	0.925045391
1.183128048	173.4695726	599.8628909	0.958967179
1.471825711	136.7163037	728.1871124	0.916445568
1.454606862	169.1217842	2979.38833	0.922034093
1.431168348	156.1583717	4233.352792	0.921964428
1.141487537	421.4308073	223.1160411	0.967911172
1.261406641	299.3264543	970.4884211	0.943252434
1.139569544	242.9542537	107.9253016	0.966620359
1.278870088	184.2919456	300.5340685	0.941791978
1.268361637	341.6389114	645.074043	0.943814801

log.sigma.3.5.mm.3D_glrlm_LongRunHighGrayLevelEmphasis	log.sigma.3.5.mm.3D_glrlm_RunPercentage	log.sigma.3.5.mm.3D_glrlm_LongRunLowGrayLevelEmphasis	log.sigma.3.5.mm.3D_glrlm_RunEntropy
367.7543824	0.936478536	0.009176849	4.881064545
312.2019432	0.878819303	0.013140379	4.819356811
158.0822233	0.943459766	0.039834366	4.53778159
416.9608043	0.929149029	0.007546608	4.908222575
413.2941444	0.940432826	0.01379788	4.894857327
198.9160014	0.928460342	0.019621983	4.503381072
160.4567034	0.909609678	0.029667074	4.27601994
408.9548221	0.950944265	0.009011797	5.002223626
453.797634	0.935619936	0.006717796	5.030591163
258.3283184	0.907309927	0.013578387	4.686423028
291.4038534	0.936843084	0.01097715	4.530280646
264.0201712	0.922973113	0.014567189	4.832005772
210.4261633	0.913211186	0.021508067	4.722669363
225.8068555	0.93174625	0.023879385	4.690617018
254.3680808	0.926454446	0.014024555	4.693407266
410.584309	0.7953184	0.014622907	4.632457976
301.2287651	0.94269341	0.015382581	4.797626818
219.128003	0.91773321	0.028277935	4.898063772
353.7593735	0.944752842	0.01965306	4.961046259
174.8672055	0.869799248	0.031903203	4.605030997
349.9604921	0.875839321	0.008501664	4.55607215
230.2785414	0.931037567	0.017781119	4.747490132
259.5746134	0.916401431	0.012084616	4.663382374
485.1183124	0.93427155	0.00817177	4.762933929
156.2024295	0.910426783	0.022943821	4.428132362
265.8976365	0.933495899	0.01724128	4.842587224
481.4614814	0.833836389	0.008533199	4.670428068
220.7764326	0.917977347	0.015030247	4.61480216
341.5445891	0.941032905	0.011987358	4.821833097
363.6672382	0.868940265	0.011594744	4.862462507
275.9282787	0.906574394	0.010131989	4.564141608
262.7339037	0.892139058	0.016884037	4.794953024
298.3550803	0.928752507	0.01576315	4.896445594
337.2987331	0.957840237	0.028678767	4.491709546
402.3333735	0.948795181	0.011873389	4.743245883
348.7978446	0.927760853	0.009447882	4.829797899
205.5422288	0.916239316	0.026589499	4.813387571
234.6229334	0.90355947	0.016394253	4.639771816
361.168148	0.919944726	0.01302909	4.680043973
252.8252083	0.931289873	0.018860883	4.783145539
324.2872075	0.900323557	0.010952037	4.74600077
366.4244056	0.941076762	0.010912834	4.905163253
318.3968513	0.937880633	0.011320262	4.750939926
352.7219773	0.939362101	0.01273843	4.974317886
393.2421985	0.927951176	0.006934605	4.757068787
114.6105728	0.891244303	0.042201802	4.377498088
304.6284016	0.925060406	0.010131126	4.781268475
213.0764143	0.936667106	0.028820863	4.670265668
477.9696296	0.951779564	0.021507206	4.517774263
518.3606412	0.9410308	0.011368393	4.898714182
261.8180736	0.899105859	0.014164521	4.730383587
245.4696994	0.927448609	0.025380138	4.601251978
375.5815929	0.907937914	0.008430834	4.903400509
227.4178193	0.918000781	0.020727639	4.547315761
238.5108952	0.853370912	0.023437632	4.704919196
311.6209723	0.9461801	0.017376823	4.925521434
435.715199	0.947385036	0.007746723	4.996766131
450.3073159	0.932536141	0.010889721	4.874585399
267.6814407	0.924588925	0.013975343	4.748050275
194.160704	0.914607526	0.020514503	4.477138361
222.762495	0.926126257	0.02418406	4.857264009
363.3291583	0.915536271	0.009141838	4.961928474
276.6719396	0.87386109	0.012869276	4.510390044
391.1844185	0.848238177	0.00986439	4.629310571
321.9380966	0.947890819	0.014760316	4.597665122
352.9221022	0.923869434	0.009481945	4.850269946
392.3060234	0.941233893	0.019898353	4.792499332
267.0363045	0.923726571	0.021241683	4.976590956
249.1800033	0.942566402	0.02195232	4.662047883
245.298937	0.928814597	0.01761388	4.805448486
318.0235221	0.920571862	0.011022099	4.860421052
245.3505691	0.933228861	0.026893418	4.860451226
227.4836748	0.889869612	0.017488982	4.711219425
381.0772257	0.876551977	0.008405456	4.648038275
379.2589193	0.868954248	0.009344101	4.735840357
426.4553685	0.944926937	0.008521869	4.964620784
290.4584079	0.932442669	0.013076078	4.742509802
188.9410292	0.937957125	0.046709827	4.775469283
344.4540478	0.933368219	0.012710551	4.843170251
281.2845916	0.922130926	0.010808884	4.706702113
402.4761091	0.947191697	0.015696764	5.033483189
411.6639129	0.949807836	0.008971794	4.931100134
426.1088734	0.922195283	0.006863665	4.88409382
417.3927438	0.950132626	0.013039822	4.342700346
200.045067	0.917247011	0.021767751	4.673194632
323.5432362	0.879642636	0.009775988	4.640318844
237.8574714	0.941170655	0.021938043	4.844709795
520.0341352	0.947809278	0.007115158	4.975884522
243.00799	0.926860025	0.01641957	4.331565319
258.8069698	0.964835165	0.019937238	4.346995283
413.4544016	0.891669086	0.009654972	4.952902376
201.5675094	0.945412944	0.04437972	4.853742843
212.8457743	0.883110041	0.026687299	4.67123008
261.3806148	0.889204439	0.016761477	4.734179642
232.2818091	0.891160338	0.016217136	4.663779917
496.9135968	0.95743387	0.018583771	5.030616281
402.8939027	0.924974696	0.00978498	4.792346067
278.9459929	0.95684803	0.036700913	4.491234333
251.458667	0.921859143	0.020041379	4.522668069
435.9932905	0.9244114	0.007709683	4.932663965

log.sigma.3.5.mm.3D_glrlm_HighGrayLevelRunEmphasis	log.sigma.3.5.mm.3D_glrlm_RunLengthNonUniformityNormalized	log.sigma.3.5.mm.3D_glszm_GrayLevelVariance
314.0234536	0.881817412	40.34323432
216.1448323	0.80078229	31.03226596
137.0617048	0.893758417	24.34373961
332.480274	0.871954198	36.74996476
339.2814899	0.895223298	46.47533819
163.2279689	0.869922742	19.28669293
125.2568225	0.837057564	21.12177169
356.7305338	0.907552826	47.22132581
376.3784954	0.88259989	46.09912248
203.2789102	0.836705252	28.1204363
240.4875364	0.883221037	24.51445937
215.6969467	0.858734293	35.45521338
163.3989299	0.846635149	30.29325255
183.4471893	0.876646941	26.47056095
207.4091305	0.863286999	30.99812696
205.1706969	0.697512201	26.56111315
251.8482324	0.893469312	32.68888426
180.1723683	0.856526826	33.76992781
299.3644274	0.895049018	42.02917492
119.5091554	0.790908868	21.44814152
242.5541934	0.791609097	21.89681373
189.849068	0.874808893	30.71850283
209.5671929	0.85179679	26.41633645
391.5403156	0.877637866	38.0837603
125.3856811	0.841298315	20.72599435
226.174708	0.877536004	38.95127074
277.9166028	0.743994564	26.95139044
180.5957972	0.856245475	25.15800078
278.344244	0.89367574	34.74383539
235.2840813	0.789320229	33.49584902
216.6670731	0.836612797	21.42395725
194.0978981	0.819220469	27.35800281
240.1106307	0.869654716	36.15821402
297.1870338	0.918533531	42.77366864
344.7236635	0.903252965	40.40362829
278.2367722	0.868853588	31.49374662
165.4545759	0.853634617	33.07942103
175.9767613	0.832754514	23.34923924
276.0740991	0.858856835	34.97037037
208.7905305	0.877458494	34.72745458
240.3569418	0.827689776	30.73925302
309.608445	0.889833071	39.65794407
260.4135323	0.887756642	31.65276258
295.4876516	0.891046046	45.17761798
315.3718643	0.870842315	32.78973267
88.80269604	0.813265653	15.72156586
245.1261485	0.864206168	31.86779117
181.4330178	0.884366686	28.2211467
407.6406773	0.908951088	44.0852071
431.8054025	0.890556917	58.54937105
195.2299876	0.830585578	26.69361733
194.7459795	0.873015268	24.32940034
286.3811587	0.841651	35.622991
178.3096796	0.85126832	24.45169454
151.0900292	0.769979246	26.05472957
268.9362009	0.898404094	45.68964258
374.0763333	0.902337635	43.18488396
362.3476906	0.876859229	44.01653381
219.5276123	0.862432218	31.12060909
151.763024	0.847768487	18.89765934
186.5312415	0.867728024	37.04758529
281.6815371	0.852994119	39.61664447
184.4140326	0.796316507	23.27344219
236.6435396	0.762235695	25.17789851
279.6039636	0.901904934	30.0314876
282.3581025	0.864032329	40.07602658
328.1141448	0.892357868	43.17805473
217.8235587	0.86611385	36.84563251
215.8303516	0.892518983	29.5469596
204.0042965	0.872294972	34.01731995
256.19072	0.860742847	31.61074176
204.9454916	0.87937996	35.02876383
166.0100535	0.815754646	26.85916199
260.9701935	0.799134631	21.77700381
249.5224646	0.7870928	32.81624307
360.4606857	0.897738005	42.68945109
236.9704839	0.877016037	31.9684196
164.555653	0.887849377	32.16129754
286.8238878	0.878485395	34.44502771
226.9128248	0.859541329	26.51963185
352.9050531	0.902631555	51.79632069
353.011833	0.903715307	42.80959366
334.9705334	0.863539801	37.29362343
357.0106149	0.907711666	33.37017747
159.4657276	0.855708179	24.25750356
227.7315137	0.798923659	27.02724537
207.064618	0.894820977	36.17536506
442.5372343	0.902591524	44.21133372
197.2516185	0.866603306	24.5
234.8713948	0.935281991	28.89746094
293.4073622	0.824181289	39.63285568
178.5240097	0.898087462	41.17441766
148.0909846	0.805561509	25.91832201
182.2912182	0.817638362	27.13844041
167.7734784	0.816957261	23.72273695
435.3682986	0.91990936	67.68738678
317.7606561	0.862070338	36.13878429
249.5075671	0.91616214	31.10583104
195.6723294	0.859239481	23.9744352
358.0332015	0.863742509	51.36986416

log.sigma.3.5.mm.3D_glszm_SmallAreaHighGrayLevelEmphasis	log.sigma.3.5.mm.3D_glszm_GrayLevelNonUniformityNormalized	log.sigma.3.5.mm.3D_glszm_SizeZoneNonUniformityNormalized
253.1338568	0.04580161	0.397444695
173.3716965	0.050029703	0.350807814
104.2704985	0.057285319	0.32199446
193.47876	0.045554531	0.330126596
219.8535566	0.042580645	0.372154006
109.9480667	0.064798463	0.31126162
93.4673957	0.064566116	0.24018595
267.6327782	0.041390273	0.405874877
256.0956825	0.04067077	0.35028225
165.3806564	0.052463742	0.32380811
136.6535195	0.058545459	0.296692045
157.5207273	0.046728181	0.334494425
102.9410428	0.051033163	0.255809949
107.8350284	0.053763302	0.272475457
130.5496404	0.052047243	0.299812696
152.6210837	0.055102632	0.307615059
152.6611922	0.050195167	0.339743871
142.639872	0.04777137	0.319984621
184.6589152	0.044282843	0.328658792
107.6591812	0.060242352	0.305636724
172.3742908	0.06071189	0.304790296
125.0267603	0.051081608	0.305257131
165.146355	0.05547662	0.324024956
227.2400645	0.048092869	0.363728621
107.6351653	0.062018585	0.330158165
184.5559611	0.045289855	0.316923966
189.7629804	0.054149197	0.285707779
160.466667	0.056858666	0.387882554
159.6334835	0.0462169	0.364280654
199.163794	0.04856792	0.363822035
168.4808152	0.061386719	0.318678385
141.3596491	0.053107155	0.302336548
142.9095264	0.046196722	0.308805192
188.4889968	0.048816568	0.354289941
226.5885704	0.045805262	0.3953856
153.3944899	0.04901298	0.279179624
142.3271488	0.049355516	0.35333547
112.1084566	0.057021074	0.286235453
155.2875837	0.049657064	0.276241427
151.2278724	0.0471213	0.326136582
160.0734966	0.051383849	0.309482174
220.5999258	0.04526517	0.353752083
162.2980452	0.048985776	0.335470265
205.6327801	0.04203912	0.320321511
200.5476033	0.048340534	0.313709566
76.74092423	0.069753918	0.282929324
163.5809514	0.048520149	0.313294653
140.4652065	0.053247622	0.353411118
237.7782482	0.046153846	0.365680473
266.6889191	0.038437303	0.342808467
131.5303942	0.054492762	0.269092457
124.7290816	0.057979493	0.347225748
205.4551919	0.046568676	0.345236633
116.1792855	0.055983038	0.264594234
118.5909044	0.055333788	0.33118922
183.9198482	0.041434618	0.354520928
274.8051676	0.042370688	0.409618789
225.0519041	0.043233002	0.361219067
163.9326986	0.049178185	0.366554477
89.13406869	0.066921534	0.2705534
153.6196981	0.045240591	0.325971477
194.8896552	0.04601831	0.337249867
134.4769834	0.059014453	0.308442321
158.4646469	0.054638833	0.293688532
206.0302145	0.054214876	0.403471074
190.7355845	0.045508084	0.325623636
248.1262653	0.047479034	0.373383675
168.3452257	0.045618147	0.356068053
162.3299271	0.053727613	0.345949604
161.0736724	0.047923765	0.351327374
192.0652244	0.048991024	0.342986802
149.2083474	0.046557295	0.333912834
139.8400648	0.05425276	0.316301268
191.3832859	0.059963851	0.310911708
164.2942149	0.049348443	0.306160436
225.427323	0.043314256	0.351676314
142.5600066	0.050497405	0.308727413
131.6136618	0.051445268	0.368047584
203.7217187	0.047423645	0.350780774
155.6934909	0.054591978	0.315644104
282.447877	0.039888256	0.387247821
234.3406101	0.042479339	0.389100092
220.5232837	0.045242173	0.34805225
222.6353188	0.05632716	0.392361111
121.464285	0.056078676	0.321354575
168.8098447	0.054362349	0.308571152
162.731718	0.047187669	0.364572066
283.5211375	0.042924797	0.368223882
147.2455006	0.068847656	0.379394531
194.367474	0.059082031	0.471679688
212.9062646	0.044137279	0.329339941
161.7437141	0.043818593	0.399922445
93.15430052	0.054081763	0.309218677
121.2177453	0.053262057	0.290942114
119.4363404	0.057374357	0.295314552
315.7459067	0.037523938	0.427565861
178.4022374	0.046114724	0.320281894
184.1945297	0.058769513	0.404499541
95.59850807	0.061236623	0.252972652
323.336835	0.040688723	0.422440067

log.sigma.3.5.mm.3D_glszm_SizeZoneNonUniformity	log.sigma.3.5.mm.3D_glszm_GrayLevelNonUniformity	log.sigma.3.5.mm.3D_glszm_LargeAreaEmphasis	log.sigma.3.5.mm.3D_glszm_ZoneVariance
120.4257426	13.87788779	14.99669967	9.567645873
205.5733788	29.31740614	144.5819113	132.0193625
61.17894737	10.88421053	9.547368421	4.329750693
327.81571	45.23564955	21.96475327	14.92329286
57.68387097	6.6	13.16774194	7.803288241
70.65638767	14.7092511	21.94273128	13.91911351
21.13636364	5.681818182	35.28409091	20.61867252
203.3433134	20.73652695	7.329341317	3.329341317
291.785114	33.8787515	17.94357743	11.71456818
295.9606127	47.95185996	69.51203501	58.02334701
89.89768977	17.73927393	17.46864686	10.01917023
170.5921569	23.83137255	27.74117647	19.29702422
143.2535714	28.57857143	52.33571429	38.61927296
107.3553299	21.18274112	19.88324873	11.83148239
96.23987539	16.70716511	27.38629283	19.33201347
319.3044316	57.19653179	3388.817919	3344.025977
102.602649	15.1589404	10.29139073	4.9494759
244.7882353	36.54509804	49.39869281	40.10615746
86.43726236	11.64638783	8.72243346	3.825384204
188.8834951	37.22977346	213.5032362	192.9609268
370.625	73.82565789	339.9292763	321.4732081
195.9750779	32.79439252	17.34267913	10.16495376
292.27051	50.03991131	45.59534368	36.42827838
73.10945274	9.666666667	14.00497512	8.278359447
126.1204188	23.69109948	58.54973822	48.61569036
87.47101449	12.5	16.02898551	9.706311699
193.9955817	36.76730486	537.5287187	511.4721294
197.43222	28.9410609	42.34774067	34.42169051
280.4961039	35.58701299	12.27142857	7.017212009
256.8583569	34.28895184	179.2011331	166.2370535
305.93125	58.93125	91.01979167	80.05403537
504.2973621	88.58273381	82.74220624	70.10307294
228.2070365	34.13937754	20.69418133	13.12609843
18.42307692	2.538461538	6.192307692	2.192307692
64.44785276	7.466257669	7.926380368	3.777786142
192.0755814	33.72093023	20.86918605	12.24881693
334.9620253	46.78902954	50.80168776	41.57239759
261.3329682	52.06024096	104.3986857	90.90332294
74.58518519	13.40740741	25.04814815	15.4840192
133.0637255	19.2254902	17.55637255	10.94592825
129.673031	21.52983294	79.3221957	66.94657697
142.9158416	18.28712871	13.13118812	7.566727527
219.3975535	32.03669725	15.85321101	9.549579628
130.6911765	17.15196078	14.29656863	7.985143935
207.9894419	32.04977376	26.13273002	18.10922517
154.76234	38.15539305	143.1480804	126.5577239
465.5558546	72.10094213	31.33243607	23.09097743
81.99137931	12.35344828	15.7112069	9.396384512
23.76923077	3	7.323076923	3.073136095
70.96135266	7.956521739	14.0821256	8.317486989
258.8669439	52.42203742	70.8004158	58.13214414
104.5149502	17.45182724	31.55813953	24.00933765
440.5219436	59.42163009	51.57601881	42.60890407
45.24561404	9.573099415	31.71345029	19.76854417
456.7099347	76.30529369	861.5902828	839.2151189
142.5174129	16.65671642	9.997512438	5.216436474
431.328585	44.61633428	9.320987654	4.983677081
183.1380671	21.91913215	23.04733728	16.54332831
111.0660066	14.9009901	26.71287129	19.4957575
62.4978355	15.45887446	55.05194805	40.50647477
245.7824934	34.11140584	32.72546419	24.82742614
643.4727463	87.80293501	46.49371069	38.25067244
209.1238938	40.01179941	396.4572271	375.6862192
157.1233645	29.2317757	465.9719626	439.9142772
44.38181818	5.963636364	6.5	2.608347107
184.9542254	25.84859155	31.50528169	24.02967851
64.59537572	8.213872832	11.19075145	6.003942664
409.4782609	52.46086957	35.83391304	28.10551304
67.80612245	10.53061224	11.52040816	6.342461474
270.5220779	36.9012987	23.98311688	17.16220105
370.7687327	52.95929695	34.63644773	26.7279175
145.9199085	20.34553776	16.83981693	10.16522053
438.0772563	75.1400722	153.6418773	139.718472
484.7113534	93.48364336	229.5202053	210.9622918
677.8392051	109.2574526	906.8437218	889.6515742
174.4314516	21.48387097	11.09274194	6.030241935
125.9607843	20.60294118	20.06127451	12.56602629
95.32432432	13.32432432	14.64092664	9.093901403
241.6879536	32.67489115	19.54716981	12.99238079
313.1189516	54.15524194	28.52620968	20.29532632
97.19920319	10.01195219	7.61752988	3.585593879
256.8060606	28.03636364	8.213636364	4.08535124
359.1899225	46.68992248	37.23837209	29.91904032
28.25	4.055555556	8.125	4.069251543
340.6358491	59.44339623	45.10283019	34.86886703
384.1710843	67.6811245	390.0240964	370.6782304
125.4127907	16.23255814	12.61627907	7.50131828
267.698762	31.20632737	8.715268226	4.157894836
24.28125	4.40625	21.640625	13.46459961
30.1875	3.78125	3.890625	1.198974609
394.5492487	52.87646077	190.8288815	179.8195581
133.1741742	14.59159159	11.29129129	6.796381968
94.62091503	16.54901961	65.28104575	54.12631894
324.1095153	59.33393178	147.021544	133.5419679
420.2326072	81.64371047	242.9739986	226.3266811
59.43165468	5.215827338	5.330935252	2.01801149
137.4009324	19.78321678	35.97202797	27.93764433
26.6969697	3.878787879	5.5	2.026859504
29.34482759	7.103448276	24.52586207	13.85099584
130.5339806	12.57281553	27.5566343	20.73591605

log.sigma.3.5.mm.3D_glszm_ZonePercentage	log.sigma.3.5.mm.3D_glszm_LargeAreaLowGrayLevelEmphasis	log.sigma.3.5.mm.3D_glszm_LargeAreaHighGrayLevelEmphasis	log.sigma.3.5.mm.3D_glszm_HighGrayLevelZoneEmphasis
0.42917847	0.114839605	3454.970297	364.3432343
0.282137699	0.865718034	28139.36519	262.5290102
0.437788018	0.343586609	962.1526316	161.9736842
0.376850095	0.098364614	7350.015106	328.4612286
0.431754875	0.094220647	4334.574194	344.316129
0.353032659	0.365566674	2516.378855	183.1718062
0.261127596	0.567451151	3666.886364	157.8522727
0.5	0.051532908	2473.471058	384.4311377
0.400673401	0.084793306	5715.920768	405.0492197
0.295029051	0.636664712	9668.685996	260.4660832
0.366384522	0.152537275	3675.633663	248.9207921
0.344129555	0.285910116	4467.121569	255.3666667
0.270009643	0.819235453	6074.214286	189.8642857
0.352415027	0.363049481	3439.086294	197.5888325
0.352360044	0.25758231	4663.158879	233.4174455
0.149417015	19.54054348	597809.2023	253.7630058
0.432664756	0.15186912	2435.708609	256.0033113
0.328044597	1.226262583	4701.397386	232.8196078
0.451890034	0.203876621	2324.498099	313.7148289
0.220635487	4.15668891	18535.28479	168.0226537
0.232771822	1.786449206	68143.84375	295.9136513
0.373255814	0.24484122	2546.579439	211.7928349
0.330281948	0.423540451	6407.68071	262.559867
0.417879418	0.051887933	6165.40796	372.3731343
0.317275748	0.9149621	4699.722513	163.7225131
0.397694524	0.200221777	2548.032609	282.1304348
0.195903058	2.244158494	132895.5685	335.8100147
0.355198883	0.482624911	5032.554028	233.6738703
0.436260623	0.101753299	3665.867532	266.0207792
0.277734068	0.864541244	41915.52125	292.1770538
0.301981755	0.773083026	12128.35938	271.096875
0.281281619	0.818166939	11833.39149	234.9736211
0.363502213	0.198252143	4651.2341	250.0933694
0.5	0.112884275	1703.461538	307.4615385
0.490963855	0.055372655	2680.300613	349.3435583
0.340594059	0.134297858	5688.805233	283.2325581
0.329166667	0.872665924	4960.546414	212.0970464
0.272212284	1.044284599	16145.28149	199.3110624
0.323353293	0.154094664	7107.3	280.4148148
0.388941849	0.27434411	2938.45098	238.4191176
0.284260516	0.459814585	16498.3747	272.5083532
0.423924449	0.125301943	3144.985149	339.6163366
0.398294762	0.145812384	3719.25841	265.0321101
0.39804878	0.133244249	3782.039216	329.2818627
0.353035144	0.11990494	7794.662142	336.2745098
0.24551167	4.210830473	6910.586837	129.2394881
0.348335677	0.200209156	6794.185734	270.9925976
0.397941681	0.535236631	1808.577586	213.3922414
0.485074627	0.084184163	3130.861538	396.3692308
0.416498994	0.076362618	6148.661836	439.2705314
0.280957944	0.577218608	11539.10915	234.8087318
0.363966143	0.900905763	3663.61794	207.0730897
0.333943994	0.276542665	12238.44671	324.8628527
0.289340102	0.420589398	5134.269006	202.8538012
0.211405795	7.771372834	112543.2879	190.4670051
0.457337884	0.117608319	2218.238806	289.5
0.480164159	0.057225059	3094.817664	399.6163343
0.392111369	0.098244405	9174.039448	356.964497
0.372235872	0.242713656	4474.858086	258.2046205
0.262202043	0.881939565	6711.380952	172.6147186
0.355828221	0.642323146	3113.6313	238.3938992
0.3483023	0.245306647	11701.04874	309.0031447
0.219417476	2.587074015	66973.10324	220.6386431
0.195898938	2.163242207	105878.2411	276.0598131
0.506912442	0.069643153	1601.290909	301.6818182
0.365743722	0.162094732	7924.213028	314.6549296
0.439086294	0.124078205	3539.236994	358.4971098
0.35971223	0.721419981	5465.423478	259.2704348
0.439461883	0.306655384	1694.280612	247.5612245
0.382894083	0.302140148	3435.002597	241.1155844
0.355592105	0.301419075	6043.823312	302.8390379
0.387068202	0.394561132	2540.672769	233.8581236
0.267995356	1.529806861	21259.2917	217.6779783
0.232132222	1.197872978	50610.89609	319.0795382
0.241176471	4.03074166	208271.7986	279.7773261
0.444444444	0.064980725	3806.540323	366.9798387
0.3652641	0.165391675	4361.367647	248.1593137
0.424590164	0.820513417	1283.752896	198.7606178
0.390589569	0.158597941	4355.047896	318.7271408
0.348559382	0.237531247	4759.558468	258.9284274
0.498015873	0.08885964	1929.151394	408.5697211
0.492170022	0.048122476	2948.762121	354.9742424
0.369627507	0.148153785	11588.16764	354.6996124
0.496551724	0.046457615	2795.430556	359.875
0.312592156	0.699266437	5108.724528	193.4292453
0.227355734	2.33249834	69037.249	280.7959839
0.442159383	0.279460057	1640.107558	244.4069767
0.468427835	0.051010919	3833.086657	447.9614856
0.349726776	0.188183228	3565.46875	220.5
0.60952381	0.053682909	802.953125	256.71875
0.301383648	0.777337794	51323.6202	338.6803005
0.471671388	0.470374183	1091.045045	222.8168168
0.299412916	0.685522526	8606.405229	165.4052288
0.272371638	1.196302555	23582.95691	211.9910233
0.245091285	1.927062029	39269.01124	205.5839775
0.549407115	0.079116995	2262.683453	452.8633094
0.352796053	0.149096874	12473.48718	312.2097902
0.536585366	0.159639306	1202.909091	266.6818182
0.306068602	0.26001947	5094.801724	187.637931
0.382899628	0.130661576	7356.563107	441.0809061

log.sigma.3.5.mm.3D_glszm_SmallAreaEmphasis	log.sigma.3.5.mm.3D_glszm_LowGrayLevelZoneEmphasis	log.sigma.3.5.mm.3D_glszm_ZoneEntropy	log.sigma.3.5.mm.3D_glszm_SmallAreaLowGrayLevelEmphasis
0.654298314	0.008809599	6.167478305	0.006626725
0.614536237	0.011681384	6.383398537	0.008142905
0.578885507	0.02685716	5.910972469	0.012723359
0.592180871	0.007519548	6.653797889	0.004770936
0.631097811	0.015160548	6.055540274	0.01110606
0.575169228	0.016878	5.910859119	0.010484974
0.500656555	0.027842759	5.692093125	0.009750686
0.661757597	0.008622948	6.339122535	0.006368974
0.611888003	0.006251321	6.674178683	0.004145635
0.587834603	0.008968191	6.556268906	0.004193404
0.558840014	0.011436666	6.163326657	0.007906295
0.597538866	0.011998724	6.499555331	0.007657728
0.513374116	0.021122711	6.716080351	0.012291642
0.528507275	0.018759553	6.426685035	0.007888008
0.548530196	0.014011847	6.257086719	0.008975984
0.570298944	0.008547041	6.593766378	0.003982432
0.601547168	0.013036678	6.179410433	0.00818299
0.579850413	0.014019846	6.53857705	0.005951508
0.580818731	0.0163004	6.295892099	0.011485613
0.570785656	0.016728882	6.370073148	0.007679653
0.566425836	0.006059366	6.503371939	0.003792549
0.566651187	0.015210527	6.493395046	0.009294011
0.585090413	0.008568995	6.438563889	0.005075728
0.624606511	0.011550606	6.078304782	0.009764175
0.592997468	0.017195831	6.079440523	0.010082635
0.575419556	0.013331016	6.326824053	0.006198932
0.545942166	0.006254998	6.594065077	0.00413319
0.646532993	0.010324767	6.076098309	0.006866064
0.623560652	0.011698225	6.362888907	0.007990837
0.625419244	0.008595289	6.397277714	0.005111157
0.582168476	0.00681893	6.38055954	0.004023276
0.565320554	0.010890611	6.73698325	0.006007508
0.568420762	0.015131762	6.628927443	0.008200445
0.610095827	0.025750822	5.147461112	0.008971892
0.651270503	0.014405704	5.902079575	0.011691157
0.538824049	0.009212462	6.677865758	0.00572293
0.615379506	0.01998219	6.513174486	0.010848946
0.550091731	0.014504777	6.60486053	0.008908378
0.528429757	0.01686535	6.431279861	0.011586756
0.586615817	0.015700803	6.391345749	0.010170866
0.571153613	0.010184087	6.409805467	0.006504702
0.615974678	0.009419127	6.366317965	0.005043058
0.597599142	0.010750947	6.430620989	0.006877512
0.579161818	0.012253414	6.538035874	0.007799321
0.576711657	0.007067662	6.566050078	0.004801693
0.544356564	0.021203307	6.211541654	0.010285051
0.576118302	0.008901028	6.727082139	0.005363525
0.615599745	0.021769526	6.046323138	0.013008807
0.606185767	0.020168365	5.275762813	0.007116666
0.602676855	0.013239565	6.264406132	0.009738465
0.530302758	0.010298726	6.738558879	0.005014198
0.609779362	0.015910039	5.972214804	0.008389207
0.608713325	0.006729278	6.6869481	0.004307702
0.5266089	0.018006689	6.070370203	0.010516865
0.595603122	0.014949272	6.569231598	0.008534946
0.612549861	0.018624339	6.426777703	0.014009356
0.665028706	0.007167023	6.433492869	0.005174386
0.62365426	0.013625912	6.538990375	0.009795658
0.620044238	0.012592432	6.044014436	0.008560147
0.533701995	0.018950105	6.124343719	0.013479925
0.587447618	0.017803284	6.587253568	0.009588394
0.600095685	0.007669331	6.79825527	0.004639877
0.572547233	0.009909888	6.42563654	0.005912551
0.555885662	0.008065098	6.479837337	0.00541393
0.659049642	0.015878672	5.537339805	0.013019525
0.585373632	0.008766246	6.600010795	0.004778961
0.632634943	0.015471344	6.049206139	0.006501613
0.617918465	0.013313013	6.584554584	0.008306429
0.603030225	0.016570922	5.928022952	0.010822874
0.613894521	0.015358259	6.517344285	0.008919276
0.605304383	0.007226296	6.511466808	0.003530427
0.597469812	0.01596473	6.413516829	0.007099875
0.581616383	0.01067457	6.626039859	0.00575773
0.573646002	0.005339203	6.514161404	0.003178858
0.568043997	0.007951923	6.840151346	0.004793729
0.612054289	0.008912272	6.440619788	0.00640862
0.571155299	0.014782236	6.440474002	0.01054188
0.628412708	0.027255495	6.018268201	0.013637367
0.613899315	0.011494389	6.446571708	0.007227524
0.577876868	0.008568558	6.46970605	0.005040665
0.642437005	0.018722205	6.172091166	0.016145562
0.645753432	0.009670212	6.36744053	0.007103505
0.6111789	0.006433911	6.646950027	0.004338678
0.635761827	0.019561899	5.290977605	0.0178365
0.58678264	0.015610894	6.494045647	0.00863936
0.570953946	0.007348441	6.602680421	0.004374758
0.621856457	0.014062243	6.180121894	0.006416964
0.626741867	0.006655415	6.461635048	0.004617686
0.638158402	0.025201373	4.951008939	0.022296117
0.713624132	0.024038238	5.052668899	0.021653541
0.590790317	0.007805673	6.769624596	0.004104042
0.652723638	0.030339533	6.09239284	0.012804851
0.569609239	0.028339396	6.24735393	0.018294446
0.551345	0.01544459	6.661942412	0.009194072
0.553947886	0.011557216	6.583893372	0.006374325
0.678278144	0.014202795	5.930936152	0.006107872
0.583417289	0.009561429	6.50158398	0.005150485
0.657673611	0.026270728	5.186376082	0.009301487
0.512598688	0.022725826	5.848693009	0.015789094
0.67298023	0.008547739	6.218036757	0.006635628

log.sigma.3.5.mm.3D_ngtdm_Coarseness	log.sigma.3.5.mm.3D_ngtdm_Complexity	log.sigma.3.5.mm.3D_ngtdm_Strength	log.sigma.3.5.mm.3D_ngtdm_Busyness	log.sigma.3.5.mm.3D_ngtdm_Contrast
0.014783416	1.402284715	5.034031882	0.137098138	0.000225642
0.005995243	0.340457189	1.803324078	0.363231363	4.17E-05
0.022268981	1.040661633	3.8978826	0.244314814	0.000435223
0.003951774	0.414913002	1.414065761	0.424134307	5.17E-05
0.03047456	2.253870919	9.211763049	0.070523932	0.000606041
0.014669091	0.627883143	2.363839056	0.287697968	0.000246138
0.031143339	0.711080682	4.27846759	0.149758271	0.000291209
0.008957957	1.676746259	4.430299883	0.169001784	0.000190474
0.005047934	0.582123718	1.884963461	0.355028634	8.52E-05
0.003476978	0.217090332	1.051293141	0.708627171	2.96E-05
0.009406403	0.640092957	1.860885827	0.32120695	0.000242976
0.007681265	0.517658863	2.355438036	0.354140161	8.63E-05
0.005235181	0.280291029	1.450834188	0.64799059	5.64E-05
0.008810274	0.533044039	2.101985382	0.358560145	0.000142886
0.011425894	0.636194599	2.388305047	0.271183413	0.000163005
0.001944362	0.08523833	1.046791056	0.764188055	3.64E-06
0.013214346	1.23207357	3.786124862	0.180702054	0.000279053
0.004664408	0.325831905	1.437497611	0.776505625	6.80E-05
0.01796903	1.548108927	5.159250805	0.131534519	0.00043318
0.004278745	0.139047708	0.910380673	0.901482337	2.83E-05
0.002136699	0.115947461	0.698528012	0.860275395	9.76E-06
0.005868058	0.510589984	2.082717537	0.457710517	7.51E-05
0.003950284	0.267654014	1.315095454	0.580390651	3.24E-05
0.020559113	1.776482715	6.913900518	0.078309136	0.000310817
0.007468061	0.363734663	1.453574204	0.575407233	8.94E-05
0.014740473	1.3432251	4.172023075	0.18893263	0.000266221
0.0035709	0.159078608	1.25881232	0.439864177	1.24E-05
0.006885379	0.417556721	1.765684382	0.496673717	8.92E-05
0.004684651	0.528815112	1.457194579	0.474686309	0.000126837
0.004934401	0.3413186	2.010627648	0.349305628	2.74E-05
0.003100547	0.204838815	0.920804689	0.763364892	2.61E-05
0.001956067	0.129008722	0.743142241	1.180575507	1.36E-05
0.005195636	0.411963131	1.566989591	0.467920293	7.67E-05
0.061327299	7.163238227	14.36262574	0.048424301	0.00369878
0.022812112	2.929829876	7.508212237	0.083183803	0.000712009
0.005037585	0.447499074	1.708274863	0.393771912	6.63E-05
0.003316472	0.31287981	1.278175851	0.987200107	4.86E-05
0.00312273	0.141862737	0.673431799	1.009937391	3.14E-05
0.010658949	0.989135851	3.559719994	0.165532516	0.00014147
0.009563742	0.71030169	2.468382188	0.291043811	0.000161655
0.008096768	0.481050544	2.532338763	0.252586186	5.76E-05
0.010043405	1.1394036	3.692001475	0.202020329	0.000194273
0.005307474	0.529202101	1.612796453	0.405379455	0.000103384
0.009705894	1.049409777	3.271244716	0.223766029	0.000196577
0.005251162	0.456804259	1.624638738	0.36366028	6.60E-05
0.004143049	0.149844612	0.767782774	1.495170993	4.38E-05
0.002488311	0.19055881	0.73754745	0.896269267	2.76E-05
0.014678411	1.098538058	3.346716127	0.240523922	0.000370877
0.050877441	6.769422365	14.99959095	0.034797858	0.002071708
0.019693651	2.50000031	9.100772877	0.064837805	0.000357656
0.003322121	0.193119298	1.020082606	0.748625171	2.57E-05
0.008026267	0.660196181	1.674094059	0.467464645	0.000395161
0.00284804	0.288356153	1.194441542	0.60283849	2.67E-05
0.016748727	0.687632776	2.959923108	0.201575899	0.000231734
0.001996102	0.082581942	0.592132578	1.316705607	1.03E-05
0.010885542	1.326170805	3.703090523	0.201637356	0.000234036
0.004176174	0.63431553	1.573991882	0.415323563	9.73E-05
0.007518525	0.837018561	2.817493756	0.192812031	0.000116132
0.013238999	0.754166894	3.063009951	0.226184205	0.000178428
0.011127701	0.401810693	1.877898562	0.355476772	0.000134357
0.004393031	0.442596297	1.557224608	0.725843506	8.17E-05
0.002003484	0.280897556	1.271570397	0.707352086	1.61E-05
0.00378751	0.184881696	1.272455597	0.538806633	1.50E-05
0.004234332	0.170050583	0.97932051	0.512875804	2.41E-05
0.043653768	2.592407159	8.357957979	0.071619036	0.001099463
0.007660025	0.664856936	2.939428234	0.229341752	6.80E-05
0.021335394	2.846451278	7.792267366	0.075787408	0.000485009
0.003214262	0.282401627	0.995056385	0.930806143	6.07E-05
0.018477025	1.543899182	4.593022836	0.154723384	0.000502436
0.005074798	0.429494607	1.600911992	0.555793635	7.15E-05
0.003298247	0.332104124	1.219513806	0.679895481	4.43E-05
0.008066082	0.789793784	2.525844937	0.367079348	0.000180401
0.002110172	0.134493739	0.690622871	1.264277354	1.65E-05
0.001736399	0.092229162	0.517961383	1.103545586	9.35E-06
0.001304551	0.085711482	0.565947313	1.197639783	5.80E-06
0.008233259	1.235109801	3.327924661	0.203340783	0.000180447
0.008128742	0.889797751	2.913256124	0.255851308	0.000118683
0.014371566	1.283927764	3.940420906	0.306222753	0.000406422
0.005159458	0.54306774	1.548676497	0.455954209	0.000101829
0.00345016	0.269182511	1.034741363	0.73581978	4.09E-05
0.017150808	2.957890389	6.559559883	0.115181047	0.000562878
0.006771436	1.02496221	2.699792288	0.244453625	0.000144221
0.004171261	0.378906907	1.548685671	0.375801681	3.88E-05
0.049564804	3.948209406	12.00706776	0.045789059	0.001632145
0.00306463	0.166454393	0.729399103	1.222539309	3.69E-05
0.002092367	0.125302224	0.789570881	0.867627856	9.84E-06
0.012197803	1.124350569	3.612908662	0.283387303	0.000277639
0.005529072	0.910411443	2.395974284	0.25073525	0.000145929
0.046118469	1.830610204	6.78024578	0.092434809	0.000933463
0.063351288	5.691855066	13.54501524	0.050307317	0.002443796
0.003024663	0.270716139	1.297259023	0.508651609	2.25E-05
0.012006401	1.724121931	4.671200927	0.282837756	0.000340577
0.0110492	0.413737975	2.036295836	0.334058248	0.00010628
0.002653696	0.152729965	0.739143145	0.978492168	2.23E-05
0.001841713	0.089168448	0.441468629	1.641513592	1.60E-05
0.029787128	6.222723679	12.92020289	0.060206711	0.001882327
0.009277729	0.576330148	2.496605656	0.210509464	0.000106261
0.06054816	5.234002424	11.65106945	0.058267576	0.002820441
0.0276382	0.950931943	5.176079358	0.115058519	0.000336183
0.013009346	1.868985868	6.793408281	0.104420975	0.000147124

log.sigma.3.0.mm.3D_gldm_GrayLevelVariance	log.sigma.3.0.mm.3D_gldm_HighGrayLevelEmphasis	log.sigma.3.0.mm.3D_gldm_GrayLevelNonUniformityNormalized	log.sigma.3.0.mm.3D_gldm_DependenceEntropy
32.16093741	280.2903683	0.050473882	6.589926961
21.68312042	245.5146846	0.067035178	7.053033464
25.26246576	148.656682	0.059217652	6.213656876
33.14398914	299.8060721	0.0480176	6.874859368
38.94690451	308.8579387	0.048145188	6.339903334
19.35419846	151.5863142	0.063352433	6.30141803
13.0723701	141.2255193	0.086511284	5.982084455
43.26661248	325.1177645	0.043914168	6.676509322
38.58109347	308.1630592	0.044622454	6.94362278
20.61497974	203.7462879	0.06781901	6.774214738
22.27891784	315.4461911	0.064692388	6.278799909
27.33677363	191.4601889	0.055262338	6.834068569
22.27681033	160.2656702	0.06122689	6.704284964
25.26720345	183.7388193	0.056336225	6.465772446
23.31413954	199.6410538	0.057869363	6.462186993
11.96027149	199.5993954	0.12854598	6.980194439
32.25458945	234.0329513	0.049827177	6.535864796
29.20364075	153.0694683	0.053523356	6.901533314
42.16510492	265.9810997	0.044047661	6.519621767
15.02832097	118.53588	0.07537689	6.936533053
14.54114854	219.0300536	0.080519085	6.822649614
27.07731713	189.5656977	0.054683613	6.582842599
21.3202469	183.5071402	0.065208026	6.711364211
29.48613638	428.7380457	0.054577046	6.418680154
16.49171298	152.8031561	0.075444807	6.47106167
31.88253577	228.6498559	0.052168027	6.663162119
14.33785526	310.2850548	0.093565421	7.009108506
22.49380542	186.8988137	0.064629029	6.586461976
36.42215442	284.8283286	0.050342752	6.619547735
21.02555463	214.6097561	0.068173335	7.107078504
18.01770565	218.6423404	0.071290528	6.673099901
20.68340863	186.2026981	0.065906344	6.999256459
31.52235386	242.5076242	0.049557365	6.792521214
39.63128698	398.4423077	0.05362426	5.400838608
37.91921904	290.0783133	0.050152417	6.130903292
28.34950961	251.1420792	0.052433585	6.734538214
29.11629581	191.5548611	0.055952932	6.894579526
19.1195654	176.1756112	0.063193531	6.808992526
24.50607767	278.7844311	0.062365807	6.558154092
28.10209733	212.2488084	0.053726778	6.667345134
20.81175702	186.9138399	0.063687361	6.884226302
34.80689357	278.7303253	0.049047081	6.579127617
30.12433554	239.2807552	0.052843966	6.616768618
37.92006282	296.3609756	0.045490065	6.682873135
25.28225935	315.3695421	0.05513819	6.588560089
13.64984154	72.49416517	0.081141277	6.503490924
26.48687987	275.1589311	0.053608939	6.814608221
27.62246498	145.4734134	0.053932313	6.327909796
37.44202495	497.7686567	0.079416351	5.534935997
39.76868049	354.5633803	0.057224636	6.40412007
20.71222301	193.7683995	0.063217687	6.898026115
30.97501641	183.7230955	0.060475576	6.388150602
27.10127773	285.2412981	0.05483923	7.05630907
19.24561027	177.9373942	0.064941981	6.288394813
17.24047032	148.8442434	0.086129814	7.054505286
39.58572998	247.9101251	0.044011384	6.639213621
44.18120709	387.1655267	0.041639973	6.773021268
33.90300799	407.9798917	0.053803304	6.678884334
25.06902094	227.6941032	0.056990987	6.71645643
16.07082293	205.8910329	0.069046242	6.404454985
31.64291882	186.5214724	0.053168027	6.711165037
30.91641394	248.8263965	0.051634664	7.134062494
13.47168505	208.0064725	0.084293629	6.836888761
14.75476556	269.572684	0.088597884	6.98304559
27.52307333	225.8525346	0.055320776	5.95189765
30.25847569	278.6303928	0.055562858	6.763741913
33.44194001	331.0482234	0.057460898	6.261530297
34.84544012	270.0269002	0.047676363	6.992542849
28.27364817	174.8139013	0.059261196	6.087941481
29.70333776	231.9199403	0.052710342	6.792653431
29.6608202	255.2335526	0.053730739	6.921079044
31.90972831	206.6926484	0.050637787	6.669523313
19.53322961	164.370356	0.067370335	6.995868919
16.52319377	261.1922275	0.072798737	6.98910552
18.45156349	345.6397603	0.080278597	7.075635752
40.53704587	302.4489247	0.044626225	6.552026514
27.37040721	249.2390331	0.054451871	6.543154157
32.64947326	143.3819672	0.053754367	6.351439395
31.68082326	299.1071429	0.050542855	6.702343908
24.76057247	205.0614898	0.05737979	6.75470553
47.5845931	340.1031746	0.041611552	6.324056017
44.06583287	366.7785235	0.041931711	6.653386381
30.27063817	298.3760745	0.052457236	6.952625276
30.95286564	394.8413793	0.063115339	5.545199997
21.86863696	180.6762017	0.060142809	6.745707052
16.31107186	254.9108839	0.078401149	6.993179975
33.46670323	182.4447301	0.050283834	6.479472767
43.12949702	415.0824742	0.046045508	6.601181462
16.76198155	174.0382514	0.069963272	5.744785531
27.28562358	264.152381	0.062312925	5.342292066
26.98972819	360.5597484	0.058427246	7.187963959
40.41673756	240.2025496	0.047159515	6.386094747
19.44147732	233.9980431	0.070398398	6.786424165
19.64779987	185.0877751	0.066074689	6.955113636
17.69234106	142.823114	0.067090965	6.900203323
73.69185583	382.3675889	0.039947507	6.260728224
25.56084444	285.4703947	0.057472461	6.616954524
36.1451517	279.0487805	0.055852998	5.44114629
20.12408713	169.6860158	0.068664239	6.162801935
34.23273433	327.2490706	0.05473636	6.759691792

log.sigma.3.0.mm.3D_gldm_DependenceNonUniformity	log.sigma.3.0.mm.3D_gldm_GrayLevelNonUniformity	log.sigma.3.0.mm.3D_gldm_SmallDependenceEmphasis
145.9745042	35.63456091	0.363288297
276.8574868	139.2320655	0.235515998
94.67281106	25.70046083	0.381075252
560.9650854	126.5263757	0.324542706
90.06963788	17.28412256	0.316922562
130.0855365	40.73561431	0.278647382
60.2462908	29.15430267	0.236523404
242.8063872	44.00199601	0.3857464
452.011063	92.77008177	0.337393061
499.6074887	210.1032924	0.261086615
178.5042322	53.50060459	0.287303603
268.1727395	81.89878543	0.314652876
389.1060752	126.9845709	0.237011405
243.862254	62.98389982	0.301503409
200.3962678	52.71899012	0.329112393
580.5710379	893.0089247	0.148321303
158.9971347	34.77936963	0.343625787
409.9957118	124.8164666	0.28451177
141.8041237	25.63573883	0.38293317
352.116744	211.1306676	0.19820398
699.4138591	420.6316998	0.221750718
371.55	94.05581395	0.30103325
475.3119736	178.0831197	0.283337443
104.4802495	26.25155925	0.350311526
198.4900332	90.83554817	0.292693822
136.9884726	36.20461095	0.341878478
374.9774957	324.2977496	0.196270078
255.2372645	92.61339846	0.309221437
411.7286119	88.85495751	0.356646339
312.7773407	173.2966168	0.230016868
513.2409563	226.6325889	0.264628587
913.5969646	390.8246206	0.241140489
422.9498278	100.750123	0.304599839
33.46153846	5.576923077	0.500934829
95.79518072	16.65060241	0.461774154
409.4475248	105.9158416	0.275538182
509.7090278	161.1444444	0.297907962
566.6529517	211.9511032	0.258699625
168.2598802	52.0754491	0.287728197
206.8570067	56.3593899	0.303842472
223.9796472	93.87516961	0.261442141
226.1794334	46.74186779	0.334090649
356.6017052	86.76979294	0.314567655
239.9287805	46.62731707	0.370547741
420.2204473	103.5495208	0.315344387
334.5448833	180.7827648	0.23212046
868.5972808	228.6957337	0.311670879
127.7204117	31.44253859	0.331885871
36.44776119	10.64179104	0.425545191
110.110664	28.44064386	0.375303664
567.4859813	216.4573598	0.262972195
165.9866989	50.01330109	0.29254776
645.7327925	209.5406962	0.278010316
120.5194585	38.38071066	0.268640667
709.6024835	561.8247739	0.188046634
214.8725825	38.68600683	0.407454272
545.49886	91.31646147	0.38929494
274.0564578	69.56767208	0.342025819
147.995086	46.39066339	0.306600991
160.5618615	60.82973893	0.249943462
422.6960831	112.6630486	0.312226857
942.0569551	282.8546915	0.290235303
413.5624595	260.4673139	0.198730006
283.548517	241.9608202	0.183163087
56.06912442	12.00460829	0.418990015
315.7752737	86.28911784	0.319136082
96.30964467	22.63959391	0.367967054
603.148264	152.4213325	0.305457365
100.5381166	26.43049327	0.349136987
403.0039781	106.0004973	0.326383762
553.475	163.3414474	0.301053225
233.1434898	57.170062	0.307489876
713.4075077	348.1698916	0.22915171
882.1131626	488.9163192	0.218901523
1157.155773	736.9575163	0.22362637
285.7491039	49.80286738	0.369089491
246.6222023	60.82273948	0.306527476
131.2918033	32.79016393	0.347065095
382.0839002	89.15759637	0.334643187
535.2839072	163.3028812	0.288608376
139.4801587	20.97222222	0.472523424
360.8001491	56.23042506	0.434475569
529.1504298	146.4606017	0.319951471
41.97931034	9.151724138	0.440243295
618.5358301	203.9442642	0.260201519
718.3115413	429.3246896	0.209665182
169.9742931	39.12082262	0.347142941
397.0335052	71.46262887	0.397669042
38.13661202	12.80327869	0.290262885
33.36190476	6.542857143	0.453291005
596.2593711	232.2483019	0.258518646
171.2011331	33.29461756	0.384195943
163.332681	71.94716243	0.261686659
635.3696822	270.2454768	0.240328217
859.5673441	389.5301412	0.222724123
71.22134387	10.10671937	0.468339921
255.8717105	69.88651316	0.290999438
35.17886179	6.869918699	0.419365402
78.22955145	26.0237467	0.276653916
146.5861214	44.17224287	0.329525752

log.sigma.3.0.mm.3D_gldm_DependenceNonUniformityNormalized	log.sigma.3.0.mm.3D_gldm_DependenceVariance	log.sigma.3.0.mm.3D_gldm_LargeDependenceEmphasis
0.206762754	2.188284955	9.385269122
0.133296816	10.12829743	27.78285989
0.218140118	2.34063157	9.119815668
0.212889975	2.67388195	10.38899431
0.250890356	1.494960467	8.019498607
0.202310321	2.830133825	11.718507
0.178772376	2.658648047	13.33234421
0.242321744	1.777231963	7.726546906
0.217417539	2.585196892	10.01010101
0.16126775	4.038097842	15.62621046
0.215845504	1.915131541	9.805320435
0.180953266	3.568176644	12.95276653
0.187611415	2.735269475	13.06557377
0.218123662	2.078798391	9.787119857
0.219973949	1.914254489	9.035126235
0.083571475	18.88270771	56.13890888
0.227789591	1.853408429	8.607449857
0.175812912	3.517417451	13.59777015
0.243649697	1.831355322	7.817869416
0.125711083	8.751389473	27.77543734
0.133884736	8.966178452	26.08460949
0.216017442	1.876224986	9.522093023
0.174043198	3.736239369	14.05089711
0.217214656	2.017660712	9
0.164858832	4.502762111	15.48504983
0.19738973	3.13360297	11.32276657
0.108187391	16.05380734	41.80957877
0.178113932	3.610278896	13.22330775
0.233274001	2.130085628	8.755240793
0.1230438	12.70786244	33.01809599
0.161447297	4.388665354	16.07392262
0.154063569	7.894704777	22.33726813
0.208042217	2.268401672	10.24840138
0.321745562	0.801775148	4.5
0.288539701	1.147336333	5.542168675
0.202696794	2.18582884	10.7980198
0.176982301	3.747914738	13.67916667
0.168948405	3.90666277	15.08944544
0.201508839	2.638330525	11.28263473
0.197194477	2.72982849	11.26692088
0.151953628	5.416172499	18.38398915
0.237334138	1.903881155	8.615949633
0.217175216	1.927071202	9.331303289
0.234076859	1.906326234	8.237073171
0.223759557	1.956867308	9.227902023
0.150154795	5.279186234	19.01705566
0.203609302	2.575747705	10.71683075
0.219074463	1.853781676	8.931389365
0.271998218	1.524838494	6.671641791
0.221550632	2.07214312	8.674044266
0.165737728	7.018047428	19.5146028
0.20070943	2.503909031	11.03143894
0.168995758	6.154309293	17.79560325
0.203924634	2.14358067	10.88155668
0.108784682	10.30532141	33.38448567
0.244451175	1.959474064	7.759954494
0.24874549	1.526467438	7.198814409
0.21195395	2.467117545	9.890951276
0.181812145	4.815495415	14.92383292
0.182249559	2.671363287	12.82292849
0.199479039	2.421673967	10.5922605
0.171970967	4.864188674	15.64914202
0.133838984	8.132464469	25.89773463
0.103825894	12.32047872	37.82094471
0.258383062	1.409458685	6.612903226
0.203332436	3.000813083	11.27430779
0.244440723	1.736787858	7.873096447
0.188660702	3.154822872	12.17360025
0.225421786	1.84160148	8.582959641
0.20039979	2.888895758	11.07856788
0.182064145	3.596779778	13.15789474
0.20650442	2.265596352	10.22409212
0.138043248	7.207836358	22.98877709
0.131345021	7.800746956	24.85139964
0.126051827	9.270389831	27.74727669
0.256047584	1.325615678	6.998207885
0.220789796	2.083089616	9.651745748
0.215232464	2.254135985	9.429508197
0.21660085	2.364430201	9.725623583
0.188082891	2.92849132	12.14054814
0.276746347	1.472080499	6.115079365
0.269053057	1.419416654	6.367636092
0.189523793	3.520005993	12.47707736
0.289512485	1.384066587	6.103448276
0.18240514	2.810595396	12.79887939
0.131174496	8.414543228	26.0036523
0.218475955	2.127127101	9.151670951
0.255820557	1.317500199	6.692010309
0.208396787	1.985547493	10.27868852
0.317732426	1.016780045	5.20952381
0.150002358	9.025664111	23.68830189
0.242494523	1.571628053	7.410764873
0.159816713	15.06465202	31.77690802
0.155347111	7.870083034	22.13594132
0.148048113	4.833538754	18.78194971
0.281507288	1.519192614	6.193675889
0.210420815	2.15157278	10.23848684
0.286007006	1.160155992	5.943089431
0.206410426	2.219784045	10.72031662
0.181643273	3.348104949	12.40768278

log.sigma.3.0.mm.3D_gldm_LargeDependenceLowGrayLevelEmphasis	log.sigma.3.0.mm.3D_gldm_SmallDependenceHighGrayLevelEmphasis	log.sigma.3.0.mm.3D_gldm_LargeDependenceHighGrayLevelEmphasis
0.073012581	123.7138667	2079.505666
0.146983341	71.01019309	6281.748676
0.259272295	70.86022033	915.983871
0.065644895	99.45544719	2966.101328
0.052922316	91.8959872	2466.406685
0.200471044	50.84917561	1371.595645
0.209904997	38.04086637	1725.344214
0.070146013	140.8108247	2115.560878
0.06851173	113.9981928	2744.78355
0.133688897	67.01725657	2547.398967
0.046191778	90.88681756	3096.614268
0.165362432	74.07895453	1836.384615
0.182320626	42.78456616	1833.140309
0.149200601	60.3021005	1665.449016
0.090721188	68.71714103	1689.032931
0.342878489	36.4282075	9996.076436
0.12074961	85.93272766	1839.416905
0.35548807	55.85625316	1483.319039
0.14454474	110.563922	1725.716495
0.438641714	31.42182231	2802.333809
0.151904448	59.38634085	5106.803025
0.149572558	65.64570619	1517.165698
0.156971152	66.01467723	1955.344562
0.033289802	145.9363109	4007.386694
0.179840784	59.04882226	1746.502492
0.119537513	96.83652628	2037.54611
0.153626086	71.82226048	12081.11079
0.142841393	73.89628027	1775.085834
0.068401864	97.35811623	2619.246459
0.196409554	61.4898498	6990.912667
0.121033675	72.58307517	2707.428122
0.211559761	55.02557096	3348.428331
0.090900636	79.25518036	2451.001476
0.026613022	190.4176683	1953.076923
0.078833744	141.2132397	1508.512048
0.089663236	72.7773896	2635.514356
0.160976133	71.14885309	2077.9375
0.151013005	51.09418463	2493.339595
0.067909927	81.94399712	3244.377246
0.126692859	73.77016649	2070.252622
0.211310567	57.60453089	3091.004749
0.07367055	101.2853672	2117.513116
0.091304675	74.51604454	2325.30877
0.077910019	124.5624019	2190.638049
0.043754177	103.4140802	2895.43344
1.049387061	23.15963191	888.6674147
0.063186822	92.7506557	2687.824191
0.525537566	57.27422341	1019.202401
0.025214482	203.0215485	3428.141791
0.08212128	135.9213731	3027.855131
0.164039512	61.4444683	3234.665596
0.240265896	54.54199866	1859.471584
0.094355915	90.49865386	4486.537294
0.129756183	52.29029486	1736.817259
0.359360374	34.78196148	4371.196535
0.144828589	111.7034003	1533.707622
0.042882882	160.0000284	2634.563611
0.036446311	135.7334713	4274.652746
0.114284622	78.85469772	2878.558968
0.102974039	55.03703167	2337.6958
0.178867011	73.88471703	1447.834356
0.114193868	81.95785511	3504.366375
0.160266877	46.87244957	4881.554045
0.160138803	54.71291535	9900.874039
0.10069244	104.3558948	1314.313364
0.06616463	100.4864925	2903.636188
0.056387749	133.6289305	2561.667513
0.103478997	97.05257255	2699.073506
0.237517421	69.36713406	1128.549327
0.100288841	90.66513349	2064.865738
0.106455717	91.24677859	2655.926974
0.148913576	74.02936447	1793.236492
0.23793941	48.7168789	3194.540248
0.124523151	69.24857784	5874.98913
0.092195095	84.39711665	9112.116231
0.066126806	118.7131671	2105.248208
0.076899856	81.557635	2143.239033
0.43158045	64.52216367	831.6704918
0.0666322	108.2685469	2560.634354
0.116923968	67.0004506	2163.284259
0.052604423	183.6536067	1586.011905
0.041816905	161.9590421	2283.425802
0.075832878	99.4315658	3528.577364
0.029115968	163.3030881	2415.365517
0.153410184	54.45181969	1935.005308
0.131870785	63.47651259	5829.295106
0.212086677	77.25197641	1112.141388
0.035797803	160.6746443	2790.014175
0.097176206	49.61688305	1716.540984
0.041341783	115.3146984	1477.695238
0.084416904	104.1150833	7844.833962
0.096311564	117.5657149	1231.800283
0.16504708	65.49279802	7023.123288
0.180455371	52.01972125	3992.843765
0.260047234	38.83077631	2323.432828
0.070163057	181.4879205	2441.521739
0.060586322	76.11872152	2956.560855
0.565227238	125.6599187	1394.463415
0.196703982	48.71946	1727.511873
0.068452487	138.1859801	3342.451053

log.sigma.3.0.mm.3D_gldm_SmallDependenceLowGrayLevelEmphasis	log.sigma.3.0.mm.3D_gldm_LowGrayLevelEmphasis	log.sigma.3.0.mm.3D_gldzm_DistanceZoneVariabilityNormalized
0.003977189	0.008317533	0.897735573
0.001923937	0.006843913	0.88796283
0.009153155	0.022723757	0.876729625
0.003897118	0.00910291	0.879078778
0.007138383	0.01226628	0.863249403
0.004929646	0.016537579	0.940227833
0.00566818	0.017335457	0.733359475
0.003803655	0.008401617	0.87480557
0.00305456	0.007924016	0.944681552
0.00264425	0.009512237	0.964109146
0.002549615	0.005891713	0.809638526
0.004183348	0.013476113	0.929671266
0.004859005	0.015867633	0.944399098
0.004087655	0.014793491	0.906111873
0.005873642	0.012895615	0.875246647
0.001166859	0.00717067	0.927727797
0.004471356	0.013807929	0.772510247
0.006197297	0.025288262	0.956928374
0.005632777	0.014338232	0.807000446
0.003396937	0.017972321	0.958352196
0.001603257	0.006465294	0.937518647
0.004461073	0.015281395	0.838319459
0.002651909	0.010589785	0.95125
0.003535848	0.005867997	0.72334032
0.003689418	0.011776948	0.916244872
0.003432303	0.012174026	0.907503858
0.001065722	0.004242655	0.911582825
0.003213322	0.010175097	0.962419812
0.003669164	0.008888057	0.943347241
0.002204961	0.009002374	0.941996733
0.001828821	0.0070785	0.973978498
0.002387225	0.010353626	0.951531109
0.003703118	0.010311029	0.945141779
0.012720643	0.014729663	0.758300781
0.0070493	0.017496892	0.847222222
0.002544472	0.008624983	0.863754325
0.004094467	0.012746517	0.958598406
0.004044361	0.012807319	0.947762728
0.003909807	0.008618023	0.837662974
0.005344845	0.013188485	0.952659455
0.002981605	0.01329187	0.884969523
0.003653271	0.009162328	0.91343134
0.005531381	0.01297733	0.894589308
0.002733019	0.009342698	0.836666683
0.002233643	0.005687295	0.885839871
0.007860944	0.04549797	0.986278528
0.002458697	0.006628405	0.857251691
0.010399652	0.043574279	0.917167969
0.010911153	0.012432961	0.652483849
0.005292254	0.011394153	0.743077182
0.003008699	0.009884696	0.932069589
0.005711077	0.018376511	0.945435519
0.001895712	0.006047106	0.912692687
0.005356326	0.013690857	0.886669529
0.002782301	0.013732708	0.966285581
0.009546851	0.021701976	0.831260141
0.002686232	0.006249477	0.750350535
0.00309162	0.00586226	0.706970426
0.004102977	0.009685808	0.902852683
0.003116547	0.008503621	0.961255448
0.003773001	0.015616135	0.932244433
0.002795613	0.008786165	0.933961575
0.001692596	0.006965033	0.949330081
0.001666567	0.005514683	0.971383493
0.008154991	0.017620691	0.963976096
0.002956635	0.007661721	0.900912558
0.004592374	0.009091279	0.779193334
0.002209865	0.007781009	0.945248959
0.010982678	0.024848962	0.871265278
0.003784138	0.009854701	0.928931233
0.002030315	0.007691611	0.900532749
0.004110521	0.013902402	0.931122449
0.002704085	0.011523613	0.940439527
0.001172079	0.005263767	0.960185427
0.001046769	0.003854527	0.941496569
0.004108613	0.011027065	0.81526517
0.003686971	0.009063907	0.86649512
0.012867105	0.035492506	0.88677602
0.00267924	0.007508492	0.854320278
0.00314162	0.009848056	0.880826446
0.005167003	0.009180061	0.855675587
0.004625423	0.008256151	0.810342724
0.002473033	0.007414434	0.919208706
0.010724881	0.012710654	0.692356994
0.002699905	0.010970503	0.969789925
0.001304707	0.005502345	0.758814328
0.006203916	0.018919778	0.945987654
0.002909372	0.006044438	0.769149345
0.009516974	0.01567347	0.761420118
0.01503425	0.018507771	0.82
0.001574713	0.004568362	0.901349726
0.004826319	0.011802043	0.969612102
0.002952269	0.007860797	0.923650273
0.003209594	0.011385851	0.936523277
0.003501992	0.015400881	0.954478853
0.013535464	0.018714156	0.671530612
0.00479734	0.009981998	0.972545367
0.005094017	0.040658623	0.855957031
0.004938097	0.020860765	0.848267285
0.002385602	0.007592518	0.905299059

log.sigma.3.0.mm.3D_gldzm_LowIntensityEmphasis	log.sigma.3.0.mm.3D_gldzm_LargeDistanceEmphasis	log.sigma.3.0.mm.3D_gldzm_HighIntensitySmallDistanceEmphasis
0.010328281	1.162162162	330.8175676
0.008215193	1.178700361	293.2694043
0.023266004	1.197969543	176.4416244
0.011806703	1.19391635	296.1292776
0.019721047	1.22147651	293.7919463
0.017159711	1.092511013	180.5693833
0.023896163	1.475247525	152.9727723
0.009147247	1.201298701	354.2732684
0.008892531	1.085409253	334.2458482
0.010337762	1.05483871	259.7897849
0.008109762	1.319587629	301.1640893
0.014196892	1.109489051	235.0720803
0.020368293	1.085858586	181.1056397
0.014104627	1.148148148	198.7611111
0.016841854	1.200534759	202.1370321
0.008473239	1.112637363	247.8276099
0.013684748	1.39261745	239.8263423
0.022110296	1.066062176	199.9190415
0.014202733	1.324626866	278.6343284
0.016575576	1.063829787	166.1968085
0.007085881	1.096848578	270.7868947
0.016035289	1.266129032	214.2762097
0.009675659	1.075	233.9059783
0.009241588	1.497487437	389.7022613
0.012216762	1.131386861	196.439781
0.011508148	1.145833333	278.3741319
0.005226829	1.139072848	364.8423841
0.010139226	1.057471264	238.8175287
0.010072074	1.087533156	271.5822281
0.010646667	1.089686099	266.7264574
0.006810271	1.039553753	277.0263692
0.009805473	1.074556213	227.5372781
0.01201881	1.084677419	255.6364247
0.021513421	1.421875	377.5859375
0.017695903	1.25	295.7861111
0.009308629	1.220588235	253.2540441
0.013646772	1.063444109	241.2968278
0.016166209	1.080516899	197.8014414
0.01237579	1.267326733	271.9405941
0.016242663	1.07277628	245.6239892
0.013296392	1.18380744	212.8402626
0.010680043	1.136020151	297.0799748
0.01738445	1.167464115	231.3377193
0.00835285	1.269146608	324.4934354
0.006907297	1.182320442	316.8138812
0.036283683	1.020725389	106.1196028
0.007654574	1.232075472	285.1172956
0.033585837	1.12987013	171.0010823
0.022368457	1.671641791	460.2873134
0.013823136	1.45412844	344.8337156
0.011380391	1.105613701	231.4312559
0.017665312	1.084210526	193.3938596
0.006692574	1.137239165	320.9951846
0.018186646	1.180904523	196.1193467
0.014657862	1.051454139	188.0018643
0.024611577	1.279069767	261.2656977
0.006832661	1.438596491	384.1008772
0.008365788	1.534926471	372.2274816
0.012519166	1.153583618	259.5895904
0.011598168	1.059288538	226.5118577
0.013499401	1.105331599	233.4213264
0.009852311	1.102564103	273.1832265
0.008249859	1.078034682	240.9931358
0.008762392	1.043557169	305.2667877
0.019998621	1.055045872	245.4610092
0.009230518	1.15682968	307.2681282
0.011526764	1.379120879	339.7431319
0.007035051	1.084507042	318.0596391
0.028560304	1.207446809	197.8417553
0.011307066	1.110687023	271.1854326
0.007075455	1.157466063	300.400905
0.013964129	1.107142857	236.9952381
0.011578752	1.092172641	215.180139
0.005232112	1.060960591	320.6560961
0.004630676	1.090484282	372.2413976
0.012085608	1.308910891	303.0222772
0.011437157	1.215777262	258.4100928
0.032983337	1.180722892	186.3594378
0.00798043	1.237288136	317.4851695
0.010746382	1.190909091	227.489899
0.010294566	1.234875445	375.2384342
0.010031258	1.31824611	351.4809052
0.008099407	1.126522962	303.7071228
0.020349978	1.569620253	373.6803797
0.010487987	1.046021093	210.3425216
0.00622706	1.420803783	272.0023641
0.017755275	1.083333333	222.7376543
0.00700019	1.399467377	390.5775632
0.028520949	1.415384615	164.5115385
0.027767412	1.3	248.1833333
0.005792004	1.156097561	391.3727642
0.012063842	1.046296296	302.4729938
0.010786646	1.119266055	247.3302752
0.013932393	1.098445596	217.3054836
0.016650036	1.069910898	180.5039411
0.026316124	1.621428571	360.2267857
0.015911817	1.041763341	267.3863109
0.023085624	1.234375	296.5273438
0.02141263	1.248120301	169.0639098
0.00871036	1.149501661	411.3048173

log.sigma.3.0.mm.3D_gldzm_LowIntensityLargeDistanceEmphasis	log.sigma.3.0.mm.3D_gldzm_HighIntensityEmphasis	log.sigma.3.0.mm.3D_gldzm_DistanceZoneVariability	log.sigma.3.0.mm.3D_gldzm_ZonePercentage
0.012640792	336.9290541	265.7297297	0.419263456
0.01022911	300.2454874	491.9314079	0.266730862
0.041544998	182.4720812	172.715736	0.453917051
0.016287937	306.4809886	924.7908745	0.399240987
0.028954725	302.6711409	128.6241611	0.415041783
0.019038178	182.5022026	213.4317181	0.353032659
0.075784133	157.2277228	74.06930693	0.299703264
0.011234963	363.3658009	404.1601732	0.461077844
0.00946742	339.9193357	796.366548	0.405483405
0.010999238	261.7688172	896.6215054	0.300193673
0.020408953	318.3367698	235.604811	0.351874244
0.015185126	239.6076642	509.459854	0.36977058
0.021134268	184.5791246	560.973064	0.286403086
0.023073434	202.7111111	366.9753086	0.362254025
0.027681154	208.6283422	327.342246	0.41053787
0.009899849	253.7527473	1013.078755	0.157190154
0.022160215	252.0302013	230.2080537	0.426934097
0.028645107	200.6282383	738.7487047	0.331046312
0.017832696	291.5522388	216.2761194	0.4604811
0.020045993	167.99509	585.5531915	0.21813638
0.007824562	276.2513451	1219.71176	0.249042879
0.035567324	222.6096774	519.7580645	0.360465116
0.015710506	235.4206522	875.15	0.33687294
0.029068033	418.5226131	143.9447236	0.413721414
0.02052098	202.810219	376.5766423	0.341362126
0.014877157	281.0590278	261.3611111	0.414985591
0.005631405	380.2874172	688.2450331	0.217830352
0.010754271	240.8448276	502.3831418	0.364270761
0.011669557	275.3580902	711.2838196	0.427195467
0.01271628	270.6860987	630.1958146	0.2631786
0.007268065	278.9462475	960.3427992	0.310160428
0.012752897	230.6579882	1608.087574	0.284991568
0.012638903	259.8481183	703.1854839	0.365961633
0.078533481	386.1875	48.53125	0.615384615
0.02589882	303.9611111	152.5	0.542168675
0.010578198	264.4985294	587.3529412	0.336633663
0.017450766	243.6102719	951.8882175	0.344791667
0.017595781	200.8230616	953.4493042	0.29994037
0.017709901	283.6831683	253.8118812	0.362874251
0.016886074	248.2479784	353.4366577	0.353670162
0.015726719	220.6980306	404.4310722	0.310040706
0.013460968	300.8790932	362.6322418	0.416579224
0.027718094	235.3460925	560.907496	0.381851401
0.01869372	333.726477	382.356674	0.445853659
0.009451341	327.3977901	641.3480663	0.385516507
0.038044578	106.3955095	571.0552677	0.259874327
0.009863377	299.6918239	1363.030189	0.372714487
0.035938841	173.4329004	211.8658009	0.396226415
0.084777716	480.0447761	43.71641791	0.5
0.018242027	361.9633028	161.9908257	0.438631791
0.017264117	234.4376784	979.605138	0.306950935
0.018717029	195.4385965	269.4491228	0.344619105
0.007886441	327.570626	1137.215088	0.326092646
0.033502442	198.4522613	176.4472362	0.336717428
0.015215806	189.5242356	1295.788963	0.205580254
0.038321938	271.2162791	357.4418605	0.489192264
0.01770701	406.2163743	769.8596491	0.467852257
0.021776396	397.4283088	384.5919118	0.420726991
0.014769989	264.8703072	264.5358362	0.35995086
0.012479603	228.1541502	243.1976285	0.287173666
0.01662601	236.8660598	716.8959688	0.362907032
0.010507757	280.232906	1748.376068	0.341730559
0.008876858	244.5231214	656.9364162	0.22394822
0.009132914	307.2540835	535.2323049	0.201757598
0.020835543	246.4862385	105.0733945	0.502304147
0.012261976	315.7419899	534.2411467	0.381841597
0.015087861	353.5604396	141.8131868	0.461928934
0.007799372	321.3679577	1073.802817	0.355333125
0.047778647	201.7393617	163.7978723	0.421524664
0.018193708	275.8715013	730.1399491	0.390850323
0.012478883	306.6316742	995.0886878	0.363486842
0.015318309	239.9380952	391.0714286	0.372010629
0.012879826	218.0292612	1285.580834	0.264512384
0.005648402	324.2703202	1559.341133	0.241810602
0.004944461	382.3683093	2216.282923	0.256427015
0.018149649	316.0752475	411.7089109	0.452508961
0.019211695	265.1531323	373.4593968	0.385854969
0.053936117	190.0522088	220.8072289	0.408196721
0.014532451	326.6652542	604.8587571	0.401360544
0.018330315	234.2050505	872.0181818	0.347856641
0.012331642	384.7295374	240.4448399	0.557539683
0.014488786	373.7708628	572.9123055	0.527218494
0.0087651	312.6551078	980.7956888	0.382163324
0.075152294	386.0886076	54.69620253	0.544827586
0.011152528	211.8791946	1011.490892	0.307578885
0.00861493	311.144208	962.9353822	0.231738495
0.018475623	226.3858025	306.5	0.416452442
0.016014195	410.114514	577.6311585	0.483891753
0.040211854	173.5692308	49.49230769	0.355191257
0.083893425	253.6833333	49.2	0.571428571
0.006449594	403.3544715	1108.660163	0.309433962
0.013013075	303.632716	314.154321	0.458923513
0.01196735	252.5504587	302.0336391	0.319960861
0.017759319	220.3825561	1084.493955	0.283129584
0.020200895	182.4105552	1392.584647	0.251291767
0.060196706	384.3285714	94.01428571	0.553359684
0.016099094	269.7424594	419.1670534	0.354440789
0.0797877	298.15625	54.78125	0.520325203
0.027250688	175.0526316	112.8195489	0.350923483
0.010221856	416.5747508	272.4950166	0.372986369

log.sigma.3.0.mm.3D_gldzm_IntensityVariabilityNormalized	log.sigma.3.0.mm.3D_gldzm_LowIntensitySmallDistanceEmphasis	log.sigma.3.0.mm.3D_gldzm_IntensityVariability
0.045676589	0.009750154	13.52027027
0.048065269	0.007711713	26.62815884
0.055064547	0.018696256	10.84771574
0.045209921	0.010686394	47.5608365
0.042205306	0.017412627	6.288590604
0.06425508	0.016690094	14.58590308
0.068326635	0.01092417	6.900990099
0.042137516	0.008625318	19.46753247
0.041782934	0.008748809	35.22301305
0.05207076	0.010172393	48.42580645
0.059812709	0.005034965	17.40549828
0.047178859	0.013949834	25.8540146
0.052511648	0.0201768	31.19191919
0.055107453	0.011862425	22.31851852
0.05158855	0.014132029	19.29411765
0.055424734	0.008116586	60.52380952
0.05033557	0.011565881	15
0.049531531	0.020476593	38.23834197
0.045862107	0.013295242	12.29104478
0.061777934	0.015707971	37.74631751
0.058125335	0.006901211	75.62106072
0.050182102	0.01115228	31.11290323
0.056157845	0.008166948	51.66521739
0.04936744	0.004284977	9.824120603
0.060247098	0.010140708	24.76155718
0.045476466	0.010665895	13.09722222
0.053788869	0.005125685	40.61059603
0.053478369	0.009985465	27.91570881
0.04714731	0.009672703	35.54907162
0.048245937	0.010129263	32.27653214
0.06076141	0.006695823	59.91075051
0.055032387	0.009068617	93.00473373
0.045395421	0.011863786	33.77419355
0.049804688	0.007258406	3.1875
0.045925926	0.015645174	8.266666667
0.051262976	0.008991236	34.85882353
0.04784245	0.012695773	47.50755287
0.056245035	0.015808816	56.58250497
0.05105164	0.011042263	15.46864686
0.050602655	0.016081811	18.77358491
0.053143659	0.01268881	24.28665208
0.045600188	0.009984812	18.10327456
0.049451961	0.014801038	31.00637959
0.04535813	0.005767633	20.72866521
0.050395287	0.006271286	36.48618785
0.071921394	0.03584346	41.64248705
0.047727542	0.007102374	75.88679245
0.053765859	0.032997587	12.41991342
0.047003787	0.006766142	3.149253731
0.043220268	0.012718414	9.422018349
0.052399916	0.009909459	55.07231208
0.058393352	0.017402383	16.64210526
0.047633648	0.006394108	59.35152488
0.05967021	0.014357697	11.87437186
0.056083004	0.014518376	75.20730798
0.04355868	0.021183987	18.73023256
0.041036368	0.004114074	42.10331384
0.043144464	0.005013137	23.47058824
0.052382672	0.011956461	15.34812287
0.066912465	0.011377809	16.92885375
0.047985917	0.012717749	36.90117035
0.045762679	0.00968845	85.66773504
0.062000902	0.008093109	42.90462428
0.054825248	0.008669761	30.20871143
0.0568134	0.019789391	6.19266055
0.044171887	0.008472653	26.19392917
0.046371211	0.01063649	8.43956044
0.046306226	0.006843971	52.60387324
0.057661838	0.023755718	10.84042553
0.047293929	0.009585406	37.17302799
0.048628816	0.005724598	53.73484163
0.048843537	0.013625584	20.51428571
0.053561079	0.011253483	73.21799561
0.059663848	0.005128039	96.89408867
0.049134538	0.00455223	115.6627018
0.041733163	0.010569598	21.07524752
0.049843616	0.009493523	21.48259861
0.051015306	0.027745142	12.70281124
0.048828561	0.006342424	34.57062147
0.052692582	0.008850398	52.16565657
0.041096237	0.009785297	11.5480427
0.040410204	0.008916876	28.57001414
0.0465907	0.007932984	49.71227741
0.052395449	0.006649399	4.139240506
0.057856375	0.010321852	60.34419942
0.054712577	0.005630092	69.43026005
0.049782807	0.017575188	16.12962963
0.043785383	0.004746689	32.8828229
0.070769231	0.025598223	4.6
0.057222222	0.013735909	3.433333333
0.044981162	0.005627606	55.32682927
0.043971955	0.011826534	14.24691358
0.053970392	0.010491469	17.64831804
0.053033489	0.012975661	61.41278066
0.057458586	0.015762321	83.83207676
0.035714286	0.017845979	5
0.048293237	0.015864998	20.81438515
0.053710938	0.008910105	3.4375
0.064729493	0.019953115	8.609022556
0.039436651	0.008332487	11.87043189

log.sigma.3.0.mm.3D_gldzm_HighIntensityLargeDistanceEmphasis	log.sigma.3.0.mm.3D_gldzm_SmallDistanceEmphasis	log.sigma.3.0.mm.3D_glcm_SumVariance	log.sigma.3.0.mm.3D_glcm_Homogeneity1
361.375	0.959459459	728.4668706	0.305984702
328.1498195	0.95532491	665.8054846	0.395909433
206.5939086	0.950507614	330.7462861	0.317850265
347.8878327	0.951520913	832.4830988	0.300692196
338.1879195	0.944630872	801.9825019	0.294681188
190.2334802	0.976872247	345.815498	0.339274458
174.2475248	0.881188119	323.2082118	0.382834215
399.7359307	0.949675325	832.5002242	0.265863377
362.6132859	0.978647687	825.7630724	0.29653578
269.6849462	0.986290323	536.7960932	0.367830776
387.0274914	0.920103093	856.724311	0.320589314
257.75	0.972627737	484.8233259	0.338841277
198.473064	0.978535354	394.2788612	0.357402047
218.5111111	0.962962963	453.0644067	0.326503137
234.5935829	0.94986631	514.5432787	0.317199124
277.4532967	0.971840659	560.8137151	0.517476582
300.8456376	0.901845638	575.7718624	0.295940166
203.4650259	0.983484456	354.0807725	0.3470277
343.2238806	0.918843284	664.9665231	0.279770378
175.188216	0.984042553	268.5165509	0.432967869
298.1091468	0.975787855	600.7807941	0.409368108
255.9435484	0.933467742	470.5568244	0.315966343
241.4793478	0.98125	456.1422959	0.359115585
533.8040201	0.875628141	1253.568138	0.300190274
228.2919708	0.967153285	371.6732119	0.372751653
291.7986111	0.963541667	559.0030069	0.312643608
442.0675497	0.965231788	923.7152317	0.444220799
248.954023	0.985632184	463.3466349	0.354424006
290.4615385	0.978116711	784.3309002	0.30021616
286.5246637	0.977578475	566.6635747	0.410932786
286.6257606	0.990111562	579.7026379	0.373264253
243.1408284	0.981360947	484.498408	0.391147072
276.6948925	0.978830645	651.1498154	0.314004506
420.59375	0.89453125	1039.803928	0.246194256
336.6611111	0.9375	739.0450188	0.264600967
309.4764706	0.944852941	674.4089103	0.326450328
252.8640483	0.984138973	488.0527476	0.339177636
212.9095427	0.979870775	449.8052501	0.367527939
330.6534653	0.933168317	758.469222	0.320206938
258.7439353	0.98180593	524.1601188	0.32485996
252.1291028	0.95404814	479.1147962	0.377057152
316.0755668	0.965994962	720.7885672	0.296711771
251.3795853	0.958133971	614.3439865	0.305290711
370.6586433	0.932713348	765.7320076	0.288664178
369.7334254	0.95441989	894.4481655	0.30699914
107.4991364	0.994818653	135.5211248	0.410686593
357.9899371	0.941981132	768.7359134	0.316568125
183.1601732	0.967532468	307.7478469	0.309191717
559.0746269	0.832089552	1318.197062	0.265963172
430.4816514	0.88646789	930.005993	0.287557538
246.4633682	0.973596575	497.5509406	0.37160037
203.6175439	0.978947368	420.441595	0.322044853
353.8723917	0.965690209	781.0574564	0.345614477
207.7839196	0.954773869	423.5560562	0.347075393
195.6137211	0.987136465	369.5677173	0.459429492
311.0186047	0.930232558	635.2659161	0.270081475
494.6783626	0.890350877	1020.537235	0.259091885
498.2316176	0.866268382	1121.708446	0.290632991
285.9931741	0.961604096	574.5200281	0.345742868
234.7233202	0.985177866	527.7519259	0.373696048
250.6449935	0.9736671	443.2237391	0.319904842
308.4316239	0.974358974	677.2035847	0.339238395
258.6430636	0.980491329	569.0258147	0.426253021
315.2032668	0.989110708	769.3667822	0.455711561
250.587156	0.986238532	523.4209228	0.296930398
349.6374368	0.96079258	762.7068674	0.316698053
408.8296703	0.90521978	841.7393405	0.283768274
334.6012324	0.978873239	714.4252705	0.321870619
217.3297872	0.948138298	382.1555813	0.310396259
294.6157761	0.972328244	615.1165149	0.316912882
331.5547511	0.960633484	684.3922908	0.330411952
251.7095238	0.973214286	492.6608551	0.31431254
229.4257498	0.97695684	406.4121186	0.400813992
338.7272167	0.984759852	734.8495267	0.408926142
422.8759558	0.977378929	1034.722462	0.404925382
368.2871287	0.922772277	791.5984139	0.268693987
292.12529	0.946055684	644.8317037	0.30885865
204.8232932	0.954819277	303.9789726	0.307509907
363.3855932	0.940677966	802.4233368	0.297748187
261.0656566	0.952272727	539.9289088	0.334544726
422.6939502	0.941281139	845.1705599	0.251091546
462.9306931	0.920438472	993.9891029	0.257250575
348.4470478	0.96836926	846.9816485	0.321492649
435.721519	0.857594937	1053.97971	0.277522081
218.0258869	0.988494727	450.9015023	0.356660327
467.7115839	0.894799054	739.2871716	0.39981102
240.9783951	0.979166667	417.812503	0.3080367
488.2623169	0.900133156	1103.575475	0.264306985
209.8	0.896153846	425.9565055	0.354724862
275.6833333	0.925	647.4436348	0.273990769
451.2813008	0.96097561	1030.240858	0.365427462
308.2716049	0.988425926	576.2233952	0.277976171
273.4311927	0.970183486	641.3055563	0.390648707
232.6908463	0.975388601	476.1305602	0.383134035
190.0370117	0.982522276	341.1955479	0.394155969
480.7357143	0.844642857	978.5765366	0.234645177
279.1670534	0.989559165	815.1958093	0.329468402
304.671875	0.94140625	676.3671825	0.257561221
199.0075188	0.937969925	405.8220964	0.342932429
437.654485	0.962624585	879.667656	0.320777351

log.sigma.3.0.mm.3D_glcm_Homogeneity2	log.sigma.3.0.mm.3D_glcm_ClusterShade	log.sigma.3.0.mm.3D_glcm_MaximumProbability	log.sigma.3.0.mm.3D_glcm_Idmn	log.sigma.3.0.mm.3D_glcm_SumVariance2
0.215742272	227.087081	0.011504596	0.970734003	92.89069443
0.322031444	179.798634	0.040547434	0.979270983	63.21073665
0.22742676	337.2387907	0.017132443	0.965205888	72.17453373
0.212246214	-85.55979799	0.008920784	0.969721074	93.02047016
0.201850535	92.50138006	0.015682748	0.967172118	123.1832801
0.249061926	69.98953389	0.017156244	0.966652895	55.02119276
0.297937488	16.29339167	0.027708875	0.972624622	38.74404968
0.175271459	171.4478719	0.009216057	0.966290856	120.4578344
0.206880429	221.4129737	0.009257506	0.97126636	108.0762037
0.284722616	227.5263013	0.018111436	0.980340738	59.69251232
0.230196823	-130.0840005	0.014717534	0.965067105	63.58903255
0.251769362	335.3906437	0.014823459	0.973696682	81.16127788
0.270030414	305.4007994	0.013075059	0.976819083	68.6054366
0.235184636	49.80047991	0.011532425	0.970247352	76.82616848
0.224997134	91.74960133	0.013068596	0.965298879	68.83385439
0.463411587	116.7904191	0.132853133	0.990268744	38.99224512
0.204432454	14.11364625	0.011284683	0.963556333	96.62637485
0.262597637	484.2726252	0.013043657	0.969966914	87.16919878
0.188417593	-3.832363166	0.010766441	0.958691134	129.7009392
0.361217904	110.7119436	0.042269112	0.976966268	44.28098628
0.332415256	116.4710974	0.035233708	0.985075464	40.83296221
0.223940797	361.423515	0.010233228	0.977590306	82.91321187
0.273572117	287.7997791	0.017767279	0.974802952	65.44919196
0.208460912	-489.9371763	0.014510134	0.970247407	89.17795931
0.289578891	166.6455125	0.024947599	0.978036745	43.47777812
0.226524084	342.9918268	0.014695149	0.966596332	87.45682089
0.374853168	154.7181322	0.070847019	0.987531754	40.48604625
0.269200204	333.3004392	0.01929279	0.974409393	63.94691025
0.211729856	-264.5635943	0.012984761	0.96551858	105.8806293
0.338704043	129.4647124	0.047869478	0.985378885	59.30237874
0.290612674	233.0871651	0.021438343	0.979475873	52.16730434
0.311612068	229.7945004	0.034134397	0.980091373	65.20098059
0.225256988	12.76761421	0.009927881	0.972769106	90.82122121
0.154119439	-46.22905288	0.025097012	0.951111743	120.1506458
0.171295369	-359.8267582	0.01390694	0.950415439	114.9358155
0.237625562	-22.02258913	0.010882747	0.972428752	83.59858834
0.255695253	393.6159152	0.015402799	0.976447717	83.99723149
0.282469766	45.65124141	0.015290109	0.97459566	55.39719833
0.230421454	-190.7295751	0.016705268	0.972880262	73.52770902
0.236315762	28.52142245	0.013706568	0.968467117	79.9439346
0.295128314	88.22050187	0.020640869	0.977237952	61.47718454
0.205363645	150.9016859	0.011178862	0.970981422	102.2580176
0.215037157	-109.6877626	0.010630621	0.967304974	88.16055098
0.19780072	139.3401483	0.009458692	0.969088465	109.40747
0.214118681	-40.16716985	0.011675379	0.972622901	69.28303412
0.333714993	137.6853529	0.02837359	0.973602563	35.60406438
0.226219404	63.85360067	0.009324332	0.973493546	75.28395808
0.21792988	85.36985562	0.011905447	0.960699611	78.27402916
0.181070211	-618.8001586	0.032633634	0.952555972	108.7595479
0.199279004	-341.5514987	0.019168126	0.971987512	119.6887116
0.288862544	188.4869694	0.027873493	0.980935744	62.06489525
0.236157603	8.121601582	0.017164186	0.948574106	86.5820569
0.263231306	196.5826356	0.020656602	0.977722893	74.24493903
0.256570182	-28.9445831	0.017062401	0.969022724	58.33772224
0.396573645	175.9984441	0.071297498	0.978501587	56.59408577
0.179224883	48.34685461	0.009546126	0.960734919	115.3765445
0.168987735	22.12150006	0.006536994	0.96448694	118.6772755
0.200609896	-414.0526009	0.013426396	0.969233655	95.33798701
0.259145567	185.9739587	0.019361437	0.971426822	75.35810472
0.287155175	20.45928097	0.015473138	0.977950499	47.17765953
0.232484664	433.9544165	0.016165946	0.970160849	90.36527712
0.255578906	394.764658	0.016547218	0.980158809	91.88472639
0.353025253	91.31492748	0.040299708	0.985339609	41.72489438
0.391333761	87.8680166	0.062988261	0.98222814	41.13509817
0.202963826	-114.906533	0.01830902	0.953788473	81.48714991
0.230690794	347.6144694	0.017176186	0.975433211	86.87704815
0.192935822	-374.2627607	0.01894543	0.965978153	91.54894411
0.236360627	236.678218	0.010423013	0.971424675	98.82513821
0.220038724	-12.87337171	0.017644249	0.959078058	82.26319651
0.230445965	288.9828358	0.011708188	0.97119478	86.91507451
0.245050704	270.9382443	0.012525508	0.971559549	86.67240305
0.225265648	245.1510001	0.012051443	0.97012988	92.73765384
0.325064221	260.5731396	0.028131558	0.977696964	58.1702484
0.334131355	132.6270305	0.03327609	0.982378114	48.86632124
0.33033276	249.8346636	0.03656268	0.987328068	51.78966887
0.177567363	-83.06926071	0.009848378	0.963140502	113.4635894
0.21875947	62.9586717	0.011872055	0.971460179	77.93649371
0.218085789	553.9141699	0.01642851	0.959210176	93.96051851
0.207793356	99.29838305	0.011258145	0.965597911	88.21633743
0.248268166	196.586242	0.0136703	0.973892678	74.14435728
0.160936735	263.7124941	0.010984965	0.962371381	122.8316391
0.165718139	-68.25873786	0.007390507	0.963915032	121.1227869
0.236590696	19.48852183	0.016311479	0.972815425	88.08379467
0.188316264	-549.3199381	0.026905061	0.953812103	98.1616114
0.271064232	172.6572475	0.01504226	0.972763988	62.61479481
0.322257209	193.4725781	0.035224366	0.986446251	48.32025815
0.216899074	462.8017332	0.013407724	0.964341356	97.0803299
0.174179265	-374.062144	0.00903519	0.962446515	124.4777539
0.267179997	20.1630186	0.029447605	0.95783493	43.20228692
0.179626395	-167.5243285	0.025165511	0.946523004	78.50420745
0.286462814	175.6997436	0.031034916	0.98141408	75.19025981
0.18713627	554.6215406	0.012697492	0.964333285	104.9672007
0.312283214	85.8956439	0.069500657	0.97441231	57.01388279
0.303172098	125.8509953	0.03381219	0.978620411	56.85619677
0.31479736	101.3830776	0.017963705	0.978484266	50.62677626
0.148732959	291.5652064	0.015184178	0.947104625	204.4059539
0.239870424	-79.2599803	0.013848952	0.971842202	72.27527629
0.165015846	-280.5938175	0.023502348	0.941279388	110.7177745
0.250909751	-62.73517814	0.022895047	0.967992155	67.43397134
0.235098213	461.6875051	0.018003536	0.977263437	85.26382416

log.sigma.3.0.mm.3D_glcm_Contrast	log.sigma.3.0.mm.3D_glcm_DifferenceEntropy	log.sigma.3.0.mm.3D_glcm_InverseVariance	log.sigma.3.0.mm.3D_glcm_Entropy	log.sigma.3.0.mm.3D_glcm_Dissimilarity
32.81891515	3.548966215	0.220305699	8.175099532	4.443721867
24.64424333	3.32917565	0.291950903	7.860196456	3.485939935
28.28576218	3.443778089	0.233065019	7.648193214	4.121879966
38.60747945	3.691347353	0.213532659	8.691424105	4.763678436
35.09838381	3.560554467	0.20361833	7.989793125	4.611927948
21.20269654	3.278866531	0.248928302	7.68375398	3.61210797
14.59388211	2.961875481	0.304147173	6.949476057	2.913287356
47.95733519	3.829039298	0.176266874	8.696009318	5.500592005
38.5667385	3.702330375	0.206796524	8.758354952	4.793609506
21.78576336	3.303635944	0.28241182	7.92314107	3.435344082
26.0943626	3.39332812	0.233014821	7.842250176	4.018131647
25.97811414	3.403245305	0.255688034	8.185657588	3.84876967
19.80253189	3.234942719	0.271361629	7.953819171	3.384505453
23.7542163	3.35064628	0.243411641	8.048355867	3.814839517
26.17100371	3.419690756	0.234545618	8.0953293	3.996286198
13.50325681	2.835987579	0.348000833	6.807529775	2.271793911
34.17433794	3.539424952	0.209529586	8.219250295	4.588904974
26.28342426	3.411212486	0.26334964	8.241783714	3.823288003
42.08927506	3.695156382	0.19370605	8.372085659	5.078534751
14.62448736	3.027899174	0.323730155	7.420503343	2.721448473
16.26164134	3.12475183	0.308126103	7.515657993	2.920236891
27.97093329	3.472008295	0.227980959	8.298620151	4.094750393
21.63305243	3.306909572	0.266894639	7.997471052	3.50232062
33.57290302	3.563281324	0.215524979	7.972782259	4.494788478
20.17855107	3.249799137	0.284889618	7.500422486	3.319927393
38.4638861	3.654414034	0.226080458	8.162834976	4.656861829
15.31945229	3.054796392	0.321219012	7.265241166	2.712032371
23.68264812	3.356768883	0.266853915	7.849414441	3.629174736
36.80299092	3.621077851	0.219019252	8.419824236	4.691580615
22.58500706	3.282804268	0.29719671	7.791760853	3.294156786
19.919619	3.258192728	0.282812726	7.763386645	3.32862454
19.3489182	3.226183035	0.282804231	7.95278402	3.223350054
32.44225947	3.567807851	0.232952435	8.489902094	4.349148657
57.64354108	3.749975794	0.161264214	6.729850129	6.017531631
45.15846561	3.716052898	0.186856318	7.927913259	5.293346461
27.13507607	3.451797458	0.243172683	8.30414766	4.004251968
31.41336399	3.535787531	0.252808924	8.288119544	4.144916238
18.74837761	3.222350888	0.280822432	7.910021986	3.283863329
28.29813959	3.386926659	0.231915123	8.01961136	4.124597418
29.42343802	3.507567873	0.232356773	8.204056224	4.147619288
21.09150565	3.26792851	0.286645243	7.882607769	3.34063847
34.59392054	3.606648762	0.213186234	8.397934372	4.585159015
32.46956498	3.541450296	0.218781331	8.346997672	4.443768801
39.53540802	3.687640952	0.206875831	8.549324795	4.862853863
28.62809542	3.488063744	0.222198897	8.269946776	4.204455895
14.11885602	2.999780232	0.325856937	7.153817107	2.766948404
29.67608336	3.52806449	0.232092467	8.470381088	4.184004852
29.82346752	3.493671648	0.219869057	7.995889235	4.28329598
59.67515955	3.79041184	0.191470848	6.821166032	5.968870067
41.85777242	3.699445731	0.211850976	8.044485258	5.009116292
20.94356979	3.292233678	0.274394684	8.007209905	3.408162934
31.75289162	3.513568452	0.234218371	7.869212019	4.328099568
29.52312058	3.493012704	0.255863929	8.326225022	4.035236721
19.7406545	3.234161303	0.261056778	7.67734272	3.439700088
18.79964317	3.095716722	0.32301078	7.50450118	2.863015071
48.12317131	3.82895349	0.183369645	8.634995962	5.423742227
54.13314974	3.847319233	0.175504293	8.9385524	5.769093833
41.78887298	3.712815455	0.202212749	8.508896047	4.957835947
24.72142316	3.392348177	0.25375387	7.991971438	3.759522924
14.81757474	3.029050839	0.297277131	7.457123504	2.983346195
31.75252354	3.564141363	0.23710763	8.365111297	4.284135444
32.76473987	3.576285772	0.243590788	8.545409237	4.20092884
14.94770013	3.042638492	0.316061559	7.3777173	2.783037304
16.24691796	3.022949682	0.335022949	7.187203009	2.724978609
30.69967024	3.457015298	0.221042225	7.440041911	4.350880584
36.83364201	3.636804197	0.234481291	8.392255277	4.531577957
41.26691275	3.669570028	0.201820018	7.910339878	4.989896964
34.22467618	3.605512674	0.234555736	8.555113598	4.418472317
28.85627344	3.464585934	0.233633806	7.765930619	4.211262657
34.54522038	3.616217266	0.230945128	8.442921856	4.460023899
32.23369952	3.530811286	0.24116342	8.369424393	4.244193045
31.60627896	3.550176182	0.225962468	8.29768139	4.330699132
20.61170441	3.251910491	0.29503988	7.841300702	3.234694941
18.20226944	3.169642735	0.308554029	7.662505548	3.051310967
20.45915911	3.243184365	0.305549763	7.731644564	3.179257021
47.59360847	3.781619534	0.185295879	8.657354989	5.40447316
32.00613536	3.539049753	0.225585445	8.239878991	4.371770993
33.95924824	3.585240003	0.221245303	7.95785106	4.483055966
36.72923081	3.616573751	0.212244717	8.454180278	4.697770203
27.31452476	3.466590467	0.249142025	8.249448382	3.982374056
54.00314655	3.839759256	0.17371849	8.344206459	5.866137684
51.97965623	3.889233083	0.172485544	8.869882747	5.694708277
36.53937755	3.646579664	0.22847773	8.556273075	4.53699712
44.92118637	3.609381628	0.207951662	6.958505336	5.194464401
21.89927597	3.303481824	0.268194216	8.010630403	3.527904463
18.64324618	3.19815942	0.300487383	7.692263071	3.104401328
31.27174006	3.54140672	0.219601978	8.156105559	4.34674763
48.27122026	3.813384673	0.186178006	8.799652842	5.499119316
19.18036366	3.045809359	0.271711136	6.793641102	3.389088481
38.93331601	3.486379339	0.188272619	6.505658291	4.966202358
28.88986363	3.474404296	0.262702639	8.255925903	3.888428003
46.45358939	3.801819752	0.191876322	8.265176244	5.245788698
22.20108271	3.311425007	0.271066325	7.689358038	3.404575371
20.9984105	3.27637599	0.285357208	7.912902866	3.335373086
17.07635403	3.146644365	0.306860634	7.742594968	3.033027822
79.38193553	4.022435323	0.14921833	7.881901773	7.031923165
26.19539731	3.423163697	0.248677372	8.147553775	3.898800507
50.35669051	3.688640777	0.1549917	6.816918184	5.681287774
18.64566217	3.18269937	0.256105505	7.538642234	3.410470996
40.01976121	3.669490604	0.235340252	8.078000559	4.594520472

log.sigma.3.0.mm.3D_glcm_DifferenceVariance	log.sigma.3.0.mm.3D_glcm_Idn	log.sigma.3.0.mm.3D_glcm_Idm	log.sigma.3.0.mm.3D_glcm_Correlation	log.sigma.3.0.mm.3D_glcm_Autocorrelation
12.13489309	0.886118767	0.215742272	0.480226871	246.1857975
11.89445301	0.912108655	0.322031444	0.443479961	227.113499
10.72186345	0.876927829	0.22742676	0.43755195	121.5600019
15.01689883	0.885705766	0.212246214	0.418542099	277.1934201
12.77161501	0.879230283	0.201850535	0.558251321	268.2287552
7.761192862	0.877897865	0.249061926	0.445519629	128.2063887
5.510795236	0.891006204	0.297937488	0.458491749	120.5064613
16.78567369	0.875902293	0.175271459	0.435642659	275.6993252
14.92903213	0.887579583	0.206880429	0.476573894	276.0681734
9.638801961	0.909574497	0.284722616	0.468725044	188.7669878
9.138833938	0.875156353	0.230196823	0.421742125	283.0961696
10.4230115	0.894204126	0.251769362	0.516044449	172.8836466
7.848396567	0.899070476	0.270030414	0.549462262	145.1005677
8.755170629	0.884123463	0.235184636	0.52884581	163.0011562
9.682335512	0.875855576	0.224997134	0.451745841	181.574413
8.121310509	0.945339912	0.463411587	0.489419179	192.7818054
11.67726917	0.872742683	0.204432454	0.479359955	199.3555758
10.95366906	0.889058622	0.262597637	0.535879965	130.8350589
14.81693154	0.865748103	0.188417593	0.51186394	225.337948
6.964465753	0.906315874	0.361217904	0.50309032	104.3424446
7.527055641	0.921691479	0.332415256	0.431722761	206.9540746
10.60281826	0.898875853	0.223940797	0.498474111	168.0734138
8.948512124	0.89650244	0.273572117	0.504382372	164.2156298
12.68367982	0.884958387	0.208460912	0.460912175	398.8755581
8.842059886	0.904395496	0.289578891	0.366826594	135.6745762
15.88044103	0.882884975	0.226524084	0.394427018	192.7291452
7.76401967	0.931082092	0.374853168	0.453111967	300.7656335
10.08592406	0.89675166	0.269200204	0.460699729	165.1229442
13.72019577	0.877953231	0.211729856	0.487112623	262.3273249
11.36274413	0.925957298	0.338704043	0.452726776	197.3818413
8.512400803	0.906712987	0.290612674	0.448123905	201.0292786
8.464553718	0.909689316	0.311612068	0.54227447	173.7759358
12.90232088	0.891472512	0.225256988	0.47733894	223.5824798
20.09084409	0.853348175	0.154119439	0.360878659	325.4918715
15.84607952	0.852989245	0.171295369	0.441709278	245.9155226
10.51658681	0.890092081	0.237625562	0.511898714	231.3427717
13.67722649	0.90172916	0.255695253	0.459074518	172.4737509
7.694496249	0.895454746	0.282469766	0.494303472	162.4733297
9.729691602	0.890136669	0.230421454	0.449470106	255.0615875
11.71523777	0.883842844	0.236315762	0.463691688	184.207924
9.581888601	0.904330282	0.295128314	0.489766033	171.3546793
12.87238994	0.88585312	0.205363645	0.497240777	244.3570705
11.50093956	0.879714448	0.215037157	0.46432132	211.4707136
14.79126105	0.883353294	0.19780072	0.47358213	257.1892213
10.42005378	0.887830003	0.214118681	0.420350708	295.5552882
6.142955338	0.89677216	0.333714993	0.431016825	58.00683185
11.68810162	0.891945246	0.226219404	0.437529144	259.0212861
10.80351684	0.868603092	0.21792988	0.448094165	114.1188116
22.14721653	0.859275114	0.181070211	0.303651685	405.3384904
15.69975666	0.888563749	0.199279004	0.487920372	304.41215
8.968381204	0.909942944	0.288862544	0.496404626	177.2731101
11.90883826	0.855267064	0.236157603	0.462934742	149.5254973
12.52339634	0.903815062	0.263231306	0.435459051	262.3588857
7.598936835	0.883112117	0.256570182	0.496982948	153.6380611
10.12954488	0.916085177	0.396573645	0.504747454	136.1487592
17.75993578	0.868960123	0.179224883	0.417808878	215.3805204
17.8221959	0.874287484	0.168987735	0.383792031	330.470682
15.77405192	0.884591398	0.200609896	0.401777377	360.8391428
10.22488564	0.890117203	0.259145567	0.50567691	200.5396502
5.554590004	0.899671468	0.287155175	0.522806698	185.9681095
12.87478766	0.887260392	0.232484664	0.481330665	158.3051165
14.48414512	0.909387135	0.255578906	0.47994345	231.6204974
6.822159712	0.922948872	0.353025253	0.475819915	197.5253261
8.454499813	0.921267718	0.391333761	0.434171102	255.8480037
11.2600663	0.857923978	0.202963826	0.455133387	181.4571474
15.45353981	0.898567475	0.230690794	0.411898956	255.4445179
15.09403739	0.877757159	0.192935822	0.384240822	276.4714646
14.05675354	0.890472052	0.236360627	0.487313765	242.6530472
10.55302633	0.866245785	0.220038724	0.481920656	138.1898726
13.9923691	0.889425715	0.230445965	0.436343042	211.6936267
12.85188128	0.891629834	0.245050704	0.464060179	233.0999794
12.20918493	0.885792568	0.225265648	0.493334291	174.1668284
9.714547634	0.907554401	0.325064221	0.479603584	148.1982052
8.587707103	0.916750678	0.334131355	0.459435754	247.772665
10.05979044	0.929505822	0.33033276	0.439382014	334.1277997
16.41340299	0.872358147	0.177567363	0.413669563	262.9548936
11.97250614	0.887762856	0.21875947	0.422306825	220.6047332
13.17148684	0.868598693	0.218085789	0.468175084	110.9422809
13.37624444	0.877700413	0.207793356	0.417751894	267.9071335
10.97894497	0.893866147	0.248268166	0.463060687	189.4208553
17.83228709	0.868996609	0.160936735	0.393073444	276.9775499
18.54800711	0.872496149	0.165718139	0.404149858	323.2391523
15.08991144	0.893573445	0.236590696	0.419742205	281.5376644
16.68690871	0.860046204	0.188316264	0.383002867	333.5872325
8.844509748	0.8926292	0.271064232	0.480012981	162.4350923
8.679565295	0.925559394	0.322257209	0.447869818	248.7363034
11.86267834	0.875094741	0.216899074	0.513974563	149.8054544
16.65538946	0.870147545	0.174179265	0.448280278	356.2960864
6.307277577	0.866258556	0.267179997	0.381298746	151.1343424
12.41844832	0.846625417	0.179626395	0.35091202	212.5396058
13.05183238	0.9136849	0.286462814	0.447496079	335.361488
18.10542729	0.876066273	0.18713627	0.390642366	195.8680116
10.27345047	0.900262799	0.312283214	0.439837469	219.44397
9.413869273	0.907180151	0.303172098	0.461162656	170.141085
7.621495044	0.906140694	0.31479736	0.49486385	128.0071159
25.53092939	0.84933442	0.148732959	0.442772979	308.9350195
10.57954661	0.889647844	0.239870424	0.470988421	272.9032672
16.08420687	0.83929013	0.165015846	0.388111761	220.6517265
6.653965895	0.879046252	0.250909751	0.567643751	148.4033547
18.19888347	0.904618562	0.235098213	0.369623429	288.7028069

log.sigma.3.0.mm.3D_glcm_SumEntropy	log.sigma.3.0.mm.3D_glcm_AverageIntensity	log.sigma.3.0.mm.3D_glcm_Energy	log.sigma.3.0.mm.3D_glcm_SumSquares	log.sigma.3.0.mm.3D_glcm_ClusterProminence
5.197303607	15.35867018	0.004265719	32.57487358	24458.16536
4.94613721	14.91575231	0.008178989	22.92742877	13199.602
4.951497575	10.52663398	0.006214771	25.37408226	15391.63262
5.277812589	16.46475561	0.003131479	33.50429364	23825.4884
5.328036352	15.78946846	0.004806866	40.34233063	41026.55134
4.832999711	10.94218299	0.005971661	19.05597232	7273.400207
4.5309104	10.69756361	0.010786841	13.33448295	4948.969941
5.413811314	16.34217166	0.002955098	42.50905861	37398.63449
5.37798188	16.3157686	0.002967475	37.32805243	33208.35037
4.937209611	13.48512154	0.006310361	21.26404326	11810.22095
4.925972777	16.56795814	0.005394217	22.66012584	9467.967891
5.13415205	12.68207089	0.004666492	27.68463011	19397.08826
5.00961003	11.54246226	0.005347539	22.35791787	14998.60162
5.076104673	12.24039316	0.00459566	25.23501247	13440.6958
5.034263606	13.09311122	0.004644995	24.07588308	12947.51513
4.462896071	13.72272506	0.031251079	13.54955406	8059.028919
5.219836954	13.61549057	0.004038745	33.42812127	21318.57552
5.16676479	10.77855342	0.004499677	28.85507332	22862.87767
5.389926787	14.41680847	0.003587916	44.54438222	37008.86387
4.716209261	9.844734096	0.009296038	14.72636841	6218.384368
4.678340307	14.23794684	0.009241293	14.83438831	5763.960871
5.157775933	12.4976027	0.004098762	28.50953448	21505.60868
4.993527062	12.40664178	0.005781022	22.19915898	13687.0392
5.118213655	19.86990915	0.004975318	30.56223507	25814.75544
4.674139096	11.41280676	0.008186571	16.17033233	6242.103668
5.153009532	13.61478758	0.004496704	33.05673581	22364.1263
4.601326333	17.27915098	0.014775856	14.31051576	7441.223296
4.91956633	12.50305567	0.006304946	22.60469194	12540.80127
5.261534258	15.84981628	0.003808198	36.72408421	23078.23411
4.912915084	13.88163416	0.009056166	21.299412	13000.99409
4.814360919	13.94994179	0.007011139	18.63335123	9181.467797
5.003250368	12.79009578	0.006525981	21.75446572	12979.69057
5.241292479	14.6379875	0.003501263	31.95131728	20816.32193
4.877485865	17.78015325	0.010259395	44.91591846	38614.40074
5.247923721	15.23660942	0.004836245	41.25271654	32260.34412
5.170378277	14.84596026	0.003890056	28.53882656	15986.79797
5.143800648	12.81963214	0.004592915	30.34042205	19792.62585
4.904021799	12.38221056	0.005531323	18.55004121	8128.861393
5.053107893	15.7054694	0.005316362	26.09213271	17672.3186
5.120770837	13.13562448	0.004324865	27.80542183	16412.50842
4.961201245	12.73652151	0.006399038	21.12826902	11542.97545
5.291620607	15.23052224	0.003696548	35.0484633	27308.80483
5.17098052	14.1303801	0.003793234	30.84067064	17141.61236
5.34679328	15.66295997	0.00328771	38.03858396	31721.15417
5.061178979	17.07786781	0.004134469	25.34863951	13200.76472
4.513580908	7.254581143	0.010212517	12.4307301	3895.483319
5.138277861	15.88718916	0.00367724	27.11500235	15162.04595
5.051015846	10.10054136	0.00465037	27.0953815	14256.47361
4.87349437	20.18603491	0.010771044	41.17407846	40779.54451
5.295025847	17.15649506	0.005371317	40.13051663	47063.05632
4.976699051	12.9761792	0.005805558	21.34071541	11953.07823
5.036233299	11.65265255	0.005490218	29.58373713	13641.91385
5.111144078	16.08164074	0.004516468	26.72081413	17716.44799
4.888042838	11.9987392	0.006255747	19.51959419	9116.289182
4.821262751	11.27038963	0.013950498	19.08761536	10877.3853
5.381426258	14.34234713	0.003067082	42.41091779	32889.60221
5.426473812	18.11354724	0.0025057	43.15245425	36198.19619
5.24247563	18.95259328	0.003801942	33.75449999	27908.26349
5.071531985	13.74743598	0.005306334	25.60545065	15378.79172
4.751958736	13.33649132	0.007010218	15.49880857	5322.738085
5.186207102	12.05871358	0.004249677	31.44659764	22449.30485
5.257604376	14.95945483	0.003785659	31.95240617	31054.49183
4.664950518	13.85702127	0.010340104	14.59079277	6521.03136
4.613421971	15.86037376	0.014893275	14.79622837	6138.983928
4.958884784	12.98888832	0.00669113	28.04670504	16684.67006
5.175373726	15.89773816	0.004402546	31.58440461	27739.34713
5.099671937	16.47157272	0.005414793	33.72992451	23458.01667
5.288715848	15.25749024	0.003446384	34.27377404	25239.20544
5.028927736	11.1714593	0.005670056	27.77986749	14239.13783
5.202434402	14.29253906	0.003815558	31.7617395	20523.33624
5.18176618	14.99679467	0.004181331	30.91340901	18839.32019
5.21141259	12.66993667	0.003964006	31.87986498	20777.54389
4.9021372	11.81187365	0.007044433	20.14858859	11368.71238
4.799657057	15.61671796	0.007946616	17.57620564	7214.797374
4.775488638	18.28880761	0.009915724	17.77715678	12786.49721
5.357676809	15.98936205	0.00306835	41.27344968	31256.26748
5.111920127	14.5970707	0.004150313	28.5515768	16074.86419
5.09760629	9.824422234	0.00515972	32.57964952	24775.31124
5.21462591	16.22026288	0.003656448	32.60133642	19822.98104
5.079659592	13.4236825	0.004362556	26.30048002	13411.91719
5.357449092	16.40636267	0.003661941	45.68146035	36535.7477
5.438387054	17.8095738	0.002593952	43.24635079	36725.25091
5.233082032	16.67217679	0.003747656	31.54828926	22099.80571
4.875906834	18.0535688	0.009345344	36.4515423	29968.74063
4.973182946	12.34757988	0.005091649	21.25922121	10679.08201
4.782544239	15.68137364	0.008299125	17.40094148	9309.374927
5.186524907	11.58694306	0.00442382	32.78430404	23967.84246
5.437548019	18.66204256	0.002808065	43.22676339	37024.46575
4.527438296	12.04444445	0.010760942	15.59566265	4589.179809
4.618171686	14.22938084	0.012053262	29.35938086	16241.77406
5.084124422	18.20825726	0.006069369	25.93032189	20692.67177
5.216191995	13.70062257	0.004154554	40.14659001	29994.93933
4.861476314	14.55142651	0.010223671	20.19882413	9704.408979
4.914831889	12.73767017	0.006713695	19.9589441	10667.93872
4.827989623	10.94029402	0.00669486	16.98926772	7502.366543
5.502145147	17.11415541	0.004907743	71.42783642	89926.91071
5.078023281	16.24053114	0.004769003	25.17411856	15297.2081
4.891539095	14.39542485	0.009812498	41.05110879	30617.69316
4.945901108	11.66915914	0.006958708	21.51990838	11675.06726
5.124775354	17.00824603	0.005280347	32.96046699	29084.99222

log.sigma.3.0.mm.3D_glcm_SumAverage	log.sigma.3.0.mm.3D_glcm_Imc2	log.sigma.3.0.mm.3D_glcm_Imc1	log.sigma.3.0.mm.3D_glcm_DifferenceAverage	log.sigma.3.0.mm.3D_glcm_Id
30.40706388	0.880194383	-0.168855737	4.443721867	0.305984702
29.49352701	0.800653566	-0.12730649	3.485939935	0.395909433
21.02715548	0.893259672	-0.190838009	4.121879966	0.317850265
32.47020376	0.71432048	-0.084183959	4.763678436	0.300692196
31.37695418	0.95839135	-0.271883184	4.611927948	0.294681188
21.88436598	0.794738425	-0.125597	3.61210797	0.339274458
21.39512722	0.879193445	-0.19862252	2.913287356	0.382834215
32.09617966	0.838972722	-0.133233466	5.500592005	0.265863377
32.16707583	0.76614887	-0.100119684	4.793609506	0.29653578
26.77970945	0.707141813	-0.089857971	3.435344082	0.367830776
33.08671579	0.77609656	-0.11507983	4.018131647	0.320589314
25.22473192	0.790238018	-0.120271114	3.84876967	0.338841277
23.05569004	0.758115068	-0.112059366	3.384505453	0.357402047
24.47164031	0.788648174	-0.120431143	3.814839517	0.326503137
26.14566919	0.788058625	-0.117066067	3.996286198	0.317199124
27.30619284	0.813702506	-0.154722631	2.271793911	0.517476582
27.10442191	0.864679225	-0.159501968	4.588904974	0.295940166
21.50320047	0.758797155	-0.108350117	3.823288003	0.3470277
28.52120821	0.906580025	-0.190439347	5.078534751	0.279770378
19.68946819	0.742107576	-0.111503552	2.721448473	0.432967869
28.34150123	0.649087348	-0.077178419	2.920236891	0.409368108
24.84468113	0.758338784	-0.103954525	4.094750393	0.315966343
24.75919033	0.716011051	-0.093735032	3.50232062	0.359115585
39.24064111	0.905402758	-0.195016755	4.494788478	0.300190274
22.79002273	0.720156454	-0.097559491	3.319927393	0.372751653
26.86651396	0.867887282	-0.159398833	4.656861829	0.312643608
34.32037885	0.731530373	-0.105764494	2.712032371	0.444220799
24.90272815	0.748543619	-0.105262651	3.629174736	0.354424006
31.30604858	0.761740802	-0.106956839	4.691580615	0.30021616
27.43708207	0.804533803	-0.129556116	3.294156786	0.410932786
27.78228816	0.679503362	-0.083152137	3.32862454	0.373264253
25.47952943	0.731771338	-0.103800282	3.223350054	0.391147072
28.91208868	0.748305391	-0.097131403	4.349148657	0.314004506
35.1945231	0.993986445	-0.49976487	6.017531631	0.246194256
30.22443796	0.953350369	-0.261781665	5.293346461	0.264600967
29.47655637	0.742127732	-0.098667357	4.004251968	0.326450328
25.24454523	0.727614837	-0.09388917	4.144916238	0.339177636
24.76313722	0.680792358	-0.082615116	3.283863329	0.367527939
31.22345499	0.830292023	-0.150622875	4.124597418	0.320206938
26.19607435	0.799509474	-0.120899128	4.147619288	0.32485996
25.39612233	0.78259768	-0.119259589	3.34063847	0.377057152
30.16057671	0.841736814	-0.140224174	4.585159015	0.296711771
28.10831113	0.753779435	-0.102087478	4.443768801	0.305290711
30.96336458	0.840801429	-0.13762501	4.862853863	0.288664178
33.78666823	0.712820351	-0.08682296	4.204455895	0.30699914
14.50916229	0.657304348	-0.083976371	2.766948404	0.410686593
31.4716736	0.644970189	-0.067752603	4.184004852	0.316568125
20.19422843	0.835613358	-0.142634941	4.28329598	0.309191717
39.63968395	0.987147948	-0.420561866	5.968870067	0.265963172
33.75587834	0.935437637	-0.229364368	5.009116292	0.287557538
25.84474292	0.711889049	-0.092275697	3.408162934	0.37160037
23.3053051	0.784659614	-0.1220958	4.328099568	0.322044853
31.69683339	0.707252969	-0.088453163	4.035236721	0.345614477
23.9974784	0.832116362	-0.14529767	3.439700088	0.347075393
22.51121695	0.816411139	-0.14543657	2.863015071	0.459429492
28.18167031	0.849195088	-0.139236198	5.423742227	0.270081475
35.45546039	0.763779027	-0.10111499	5.769093833	0.259091885
37.27784314	0.797357599	-0.1167555	4.957835947	0.290632991
27.41179334	0.842897389	-0.1468776	3.759522924	0.345742868
26.67298265	0.771981861	-0.12338684	2.983346195	0.373696048
23.96986647	0.738336779	-0.094926197	4.284135444	0.319904842
29.45059487	0.723958635	-0.090585559	4.20092884	0.339238395
27.62757182	0.739085638	-0.111305577	2.783037304	0.426253021
31.59883691	0.79066388	-0.134707843	2.724978609	0.455711561
25.97777665	0.958310241	-0.289352533	4.350880584	0.296930398
31.17144232	0.805927495	-0.121434199	4.531577957	0.316698053
32.4831056	0.921055616	-0.214825828	4.989896964	0.283768274
30.09940909	0.731365356	-0.091961243	4.418472317	0.321870619
22.3429186	0.871218566	-0.171125318	4.211262657	0.310396259
28.18484252	0.744825851	-0.095411711	4.460023899	0.316912882
29.62955323	0.724405354	-0.095022194	4.244193045	0.330411952
25.20709881	0.806616804	-0.123458749	4.330699132	0.31431254
23.56284072	0.71094525	-0.095852536	3.234694941	0.400813992
30.99060268	0.699204235	-0.091428761	3.051310967	0.408926142
36.12711107	0.678196327	-0.08307212	3.179257021	0.404925382
31.39710388	0.832251761	-0.132053479	5.40447316	0.268693987
28.92060139	0.792929214	-0.117462838	4.371770993	0.30885865
19.5857542	0.860654871	-0.159424468	4.483055966	0.307509907
31.93863407	0.739316458	-0.100495623	4.697770203	0.297748187
26.66143738	0.687859403	-0.081386472	3.982374056	0.334544726
32.22956413	0.925487186	-0.209332074	5.866137684	0.251091546
34.98181281	0.807099619	-0.114437068	5.694708277	0.257250575
32.78081791	0.735305461	-0.093447579	4.53699712	0.321492649
35.78553696	0.9854987	-0.406913576	5.194464401	0.277522081
24.67781957	0.686700138	-0.08772134	3.527904463	0.356660327
31.06864984	0.669764934	-0.082039635	3.104401328	0.39981102
23.09304633	0.847728962	-0.146995618	4.34674763	0.3080367
36.72502204	0.788914762	-0.108583015	5.499119316	0.264306985
24.0888889	0.928587848	-0.259014225	3.389088481	0.354724862
28.45876168	0.989143682	-0.45776777	4.966202358	0.273990769
35.98789943	0.757080057	-0.104916691	3.888428003	0.365427462
26.92384952	0.876860633	-0.163327694	5.245788698	0.277976171
29.03343361	0.823387247	-0.141317844	3.404575371	0.390648707
25.39079902	0.703511365	-0.091342128	3.335373086	0.383134035
21.87356356	0.685170219	-0.086224958	3.033027822	0.394155969
33.32169238	0.984243383	-0.361383443	7.031923165	0.234645177
32.33417706	0.790663335	-0.117262146	3.898800507	0.329468402
28.66566468	0.98508635	-0.411373143	5.681287774	0.257561221
23.33831829	0.8971981	-0.19811565	3.410470996	0.342932429
33.30961923	0.872914239	-0.164287607	4.594520472	0.320777351

log.sigma.3.0.mm.3D_glcm_ClusterTendency	log.sigma.3.0.mm.3D_firstorder_InterquartileRange	log.sigma.3.0.mm.3D_firstorder_Skewness	log.sigma.3.0.mm.3D_firstorder_Uniformity
92.89069443	198.5257797	0.304346687	0.050473882
63.21073665	134.5972824	0.417173976	0.067035178
72.17453373	183.3689785	0.48190185	0.059217652
93.02047016	210.4008522	-0.072808695	0.0480176
123.1832801	188.8062363	-0.052743114	0.048145188
55.02119276	172.8328514	0.053870616	0.063352433
38.74404968	105.9593887	-0.088359515	0.086511284
120.4578344	262.7015781	0.020311291	0.043914168
108.0762037	223.9527779	0.168893254	0.044622454
59.69251232	136.951107	0.601938048	0.06781901
63.58903255	190.7667217	-0.41133124	0.064692388
81.16127788	178.5905914	0.478965542	0.055262338
68.6054366	167.2675972	0.41090244	0.06122689
76.82616848	212.7516861	0.021945043	0.056336225
68.83385439	169.5843086	0.075176073	0.057869363
38.99224512	66.32031441	0.646643601	0.12854598
96.62637485	226.2622986	-0.11396231	0.049827177
87.16919878	199.2871971	0.52798881	0.053523356
129.7009392	244.6675949	-0.076495859	0.044047661
44.28098628	131.1702271	0.386473723	0.07537689
40.83296221	115.7173486	0.517611348	0.080519085
82.91321187	179.2856979	0.400817818	0.054683613
65.44919196	154.7019711	0.540893247	0.065208026
89.17795931	187.157074	-0.544019132	0.054577046
43.47777812	133.7274365	0.639477742	0.075444807
87.45682089	196.1061487	0.455235257	0.052168027
40.48604625	93.7949872	0.664364282	0.093565421
63.94691025	152.8065681	0.623701576	0.064629029
105.8806293	270.3439178	-0.200455502	0.050342752
59.30237874	133.9455123	0.447132061	0.068173335
52.16730434	139.0325384	0.587464104	0.071290528
65.20098059	155.9246769	0.505242665	0.065906344
90.82122121	222.0477753	0.07185578	0.049557365
120.1506458	190.1335564	-0.256127037	0.05362426
114.9358155	217.808363	-0.374974704	0.050152417
83.59858834	217.1381702	-0.045437042	0.052433585
83.99723149	196.5073109	0.601823855	0.055952932
55.39719833	159.9876957	0.115222574	0.063193531
73.52770902	151.2654572	-0.314254589	0.062365807
79.9439346	200.3394852	0.03881141	0.053726778
61.47718454	148.7446089	0.242478205	0.063687361
102.2580176	224.8216629	0.070784878	0.049047081
88.16055098	224.2753639	-0.199718482	0.052843966
109.40747	230.821785	0.08399913	0.045490065
69.28303412	187.3596134	-0.043581942	0.05513819
35.60406438	133.2949047	0.582404301	0.081141277
75.28395808	183.862196	0.147625442	0.053608939
78.27402916	212.7615147	-0.024316141	0.053932313
108.7595479	114.4912786	-0.849867148	0.079416351
119.6887116	157.7438889	-0.372832296	0.057224636
62.06489525	158.9698524	0.411010475	0.063217687
86.5820569	262.3748093	-0.166349529	0.060475576
74.24493903	186.2031097	0.350716123	0.05483923
58.33772224	153.9317513	-0.132110273	0.064941981
56.59408577	109.1145039	0.464739279	0.086129814
115.3765445	235.684269	0.029221645	0.044011384
118.6772755	264.3607864	-0.080976948	0.041639973
95.33798701	182.3560333	-0.561634069	0.053803304
75.35810472	174.6088543	0.184642345	0.056990987
47.17765953	156.5378113	-0.061630547	0.069046242
90.36527712	223.0013695	0.430811775	0.053168027
91.88472639	193.8584251	0.475782123	0.051634664
41.72489438	106.845336	0.341139966	0.084293629
41.13509817	105.6704035	0.325799295	0.088597884
81.48714991	186.0364304	-0.313908771	0.055320776
86.87704815	167.5711098	0.472630557	0.055562858
91.54894411	190.7375088	-0.388095816	0.057460898
98.82513821	233.2372513	0.256928156	0.047676363
82.26319651	224.3315487	-0.182537956	0.059261196
86.91507451	198.5963516	0.41822842	0.052710342
86.67240305	212.189209	0.403729891	0.053730739
92.73765384	231.3492279	0.157552622	0.050637787
58.1702484	143.8444357	0.59630246	0.067370335
48.86632124	130.0996952	0.463224613	0.072798737
51.78966887	107.3166666	0.540366318	0.080278597
113.4635894	249.0732098	-0.080186612	0.044626225
77.93649371	207.0070572	0.036981089	0.054451871
93.96051851	225.802125	0.497759892	0.053754367
88.21633743	224.5077057	0.114592855	0.050542855
74.14435728	193.6895313	0.33613112	0.05737979
122.8316391	284.2857227	0.087896312	0.041611552
121.1227869	259.7243118	-0.1081889	0.041931711
88.08379467	190.8813725	0.078653139	0.052457236
98.1616114	149.4904404	-0.829472816	0.063115339
62.61479481	175.4013367	0.354069888	0.060142809
48.32025815	119.6474743	0.660215988	0.078401149
97.0803299	229.4984169	0.363902499	0.050283834
124.4777539	264.7875347	-0.427204801	0.046045508
43.20228692	158.597847	-0.12339925	0.069963272
78.50420745	177.2947731	-0.572962159	0.062312925
75.19025981	167.3783798	0.211432354	0.058427246
104.9672007	244.2725554	0.506170812	0.047159515
57.01388279	143.7855978	0.096623646	0.070398398
56.85619677	146.5495615	0.321466088	0.066074689
50.62677626	149.2432318	0.3399463	0.067090965
204.4059539	391.9906769	-0.065728542	0.039947507
72.27527629	162.2456989	-0.256638699	0.057472461
110.7177745	200.5312424	-0.445131106	0.055852998
67.43397134	146.9198418	-0.210749307	0.068664239
85.26382416	179.0210457	0.701004957	0.05473636

log.sigma.3.0.mm.3D_firstorder_MeanAbsoluteDeviation	log.sigma.3.0.mm.3D_firstorder_Energy	log.sigma.3.0.mm.3D_firstorder_RobustMeanAbsoluteDeviation	log.sigma.3.0.mm.3D_firstorder_Median
113.8997177	14328185.49	81.31351294	-19.20932007
89.16116174	28493115.31	59.49415299	-16.61746979
102.4849578	7358189.05	74.06660322	-49.30753899
118.1904203	55670102.96	86.45328604	23.96698761
123.2283427	8734035.633	84.45988268	8.683661461
92.54141593	9355737.053	70.22421582	-46.39082336
68.71049056	2744608.533	43.94444104	-0.759144068
138.9054934	27428827.66	105.5443498	-2.312566638
127.2851206	51589627.35	92.68987579	-29.68364143
88.73707443	41689182.98	60.1483772	-38.64488792
98.75942744	12335439.77	75.36384655	-14.18089485
105.4598935	25618516.27	75.16275246	-33.85287476
95.25740663	28924603.98	68.23522417	3.429697633
107.6425025	17590786.06	84.07427363	8.173342228
96.82470461	13451929.03	69.10312052	17.09539223
57.90469786	51610738.27	30.52193174	-1.798704386
119.9745497	14751911.82	91.4260905	-14.47946167
111.1451927	42654413.96	80.98776941	-29.16521931
134.4340949	15350856.54	99.83055041	28.52567768
76.03356141	26370576.23	52.79556363	-8.711318016
73.42166406	51861770.27	49.08148757	-39.22004318
104.7665767	29143793.55	74.67569853	1.670748711
91.41899855	37126533.92	63.13747594	-34.44245529
109.0970239	8920252.249	77.35865382	25.31532097
80.78574509	14711444.9	56.21032984	-63.16250801
113.156236	14832652.96	79.64258561	-49.35251236
68.12421153	31028065.6	41.81617351	-11.49278545
93.48862995	22544979.49	64.30312933	-60.70106125
132.1988022	40061384.6	106.6800969	29.02217102
88.26067286	37294025.3	59.13421679	-39.77894211
84.19087846	38964311.75	58.58907509	-51.71727371
90.69188655	77038959.63	63.37875989	-8.558350086
117.343251	39972788.31	88.44363059	7.489667892
121.5531698	2573741.389	81.4214792	3.724483132
127.5058212	7910294.148	92.96250913	16.04154205
113.4033532	35990746.54	87.80973092	-8.839571476
111.1208621	53264923.39	81.97549234	-41.52837181
89.46148534	39826589.48	65.59623131	3.032170057
96.02966208	12862970.24	63.84757219	21.55729103
108.994151	19046386.29	80.21283175	-13.9456501
90.13083797	19474537.54	62.29005613	-21.10351276
122.9221789	21206239.76	91.94766625	-12.25798321
117.2448914	31687621.58	91.33035827	-7.520020485
126.6227118	24346686.63	93.14314544	-5.575222015
103.8608378	29794360.61	76.43905729	-5.421930313
75.8735446	19879184.9	55.36224435	-39.26229668
104.6934827	70651461.67	75.52205758	3.520821095
110.2879471	10972823.99	84.324359	-36.56878662
108.0490447	3134180.66	60.26665071	16.51718426
119.2959921	12310643.32	75.19092587	33.13806152
90.6422141	44335668.26	64.30427957	-12.18621111
124.9192117	18300702.41	105.1369882	-31.77444077
104.2903045	64994403.69	74.5239151	-16.1543293
88.54940768	7094753.145	63.22335006	9.372306824
76.24234534	70067333.09	47.6550106	-4.680440426
131.079277	22340246.59	97.86486828	-24.70269585
140.3563923	61879453.33	106.9634774	-17.96134949
114.8573953	27293566.55	77.9238472	23.49733734
100.9491069	13563622.89	72.0786871	-34.33626175
83.5910113	9056147.431	63.13960339	-14.77614689
118.9093173	43243298.5	90.5591988	-43.39527512
111.1200515	105902018.9	78.84558955	0.47984229
69.50863981	26218906.03	45.71731677	8.789849281
71.69866242	25419063.65	46.38532707	-12.2183466
106.6423109	3988519.699	77.05391711	-30.49305534
105.1486495	30743931.01	69.33995297	23.7494297
116.440267	8610716.738	81.40828052	1.277973354
123.1351596	69548767.62	92.94129961	-6.426157475
115.0256063	9491364.091	90.9500086	-29.1050415
111.4135857	37331217.96	81.41651008	-19.68318176
113.3892872	56824709.38	84.58985594	-34.45009232
120.2223668	23593218.05	92.99463068	-28.05837631
86.8949801	63686990.87	60.47312618	-16.94574928
80.00606998	72069916.8	55.50460288	-26.81138802
77.79900631	107246680.9	47.67631086	11.13430166
135.1646079	29458540.62	103.2297915	-20.58603096
109.6122409	20878857.65	82.70072714	-35.70354843
118.7723237	13852233.56	90.18755259	-57.60561562
117.862007	39920488.49	89.19822386	-70.37605286
104.0561243	46041027.23	78.11662654	-46.34214401
146.4120362	18623194.11	112.7954906	-92.4654808
139.0160913	37034041.72	104.7053867	0.225550234
109.2995333	54133731.27	77.0265434	19.21265316
102.414613	2799286.493	62.03635451	34.24332428
96.79392723	46081929.7	71.88331213	-10.71641922
76.3769087	55331242.54	49.83476174	-13.61844158
122.7676329	17095880.06	94.13033954	-48.93367958
139.2637031	43361794.83	106.4069247	2.05803597
85.89551484	1934233.59	63.57696771	-2.206636667
106.2525882	2059671.117	74.71458341	-24.86768341
100.3462065	67021490.96	68.24745568	-6.347349167
131.7861006	20319481.89	98.11668997	-76.58669662
86.19964568	12902306.71	59.00724748	-20.51987076
89.22069746	51026524.25	63.46213716	-16.04197645
85.3295711	64200884.68	61.72995612	-8.550374508
188.37815	12164162.83	151.6309578	-19.05929756
100.4968716	19792462.84	69.19770483	24.17695713
120.807656	2788019.045	81.66451951	21.52355194
87.98260091	4865726.705	60.02859348	26.30660439
112.2500646	18194985.09	74.13751476	-53.85694122

log.sigma.3.0.mm.3D_firstorder_TotalEnergy	log.sigma.3.0.mm.3D_firstorder_Maximum	log.sigma.3.0.mm.3D_firstorder_RootMeanSquared	log.sigma.3.0.mm.3D_firstorder_90Percentile	log.sigma.3.0.mm.3D_firstorder_Minimum
386861008.3	389.4571228	142.4601007	166.6337051	-375.2703857
769314113.3	433.3812561	117.1255706	150.6733582	-363.0543518
198671104.3	364.6506348	130.2088808	135.5354187	-281.4712524
1503092780	455.7863159	145.3518964	199.3696625	-373.3244934
235818962.1	372.7202759	155.9768895	210.1552948	-386.4910889
252604900.4	250.5051422	120.623942	94.96691132	-301.4017029
74104430.38	251.6296997	90.24543739	103.7737274	-261.9385071
740578346.8	461.8536987	165.4511393	188.2438614	-418.9519348
1392919939	437.0761414	157.5266186	180.0650208	-403.6027832
1125607940	446.0679321	116.0034713	140.181514	-334.9717407
333056873.8	182.0520325	122.1306206	101.7723541	-425.3685608
691699939.3	407.9152832	131.4779382	168.0843338	-306.8177795
780964307.4	421.1288147	118.0944084	156.6380005	-261.0873718
474951223.7	356.0329285	125.4358629	158.7645081	-297.5482788
363202083.9	342.8427734	121.5158965	180.9899902	-296.7142334
1393489933	551.9355469	86.19287883	110.8834244	-311.8578796
398301619.2	349.1339417	145.3772477	141.884845	-356.7748108
1151669177	417.598938	135.2439096	174.0729248	-273.7210083
414473126.7	375.7587585	162.4070262	211.6312958	-343.5721436
712005558.1	348.1170349	97.0293798	116.9715729	-238.5902405
1400267797	401.429718	99.63733075	103.5346199	-359.0195618
786882425.8	527.46875	130.1693743	173.1649963	-298.337616
1002416416	371.6616516	116.5953829	142.9627991	-313.7903442
240846810.7	313.901123	136.1808466	170.7784576	-456.2715759
397209012.4	381.9169312	110.5387175	96.42359085	-304.1859131
400481630	419.6791077	146.194045	153.1347473	-360.1247253
837757771.3	421.0501099	94.61567049	118.1734161	-408.76651
608714446.4	364.2230225	125.4301169	141.4446075	-328.1898193
1081657384	381.9503784	150.6574536	188.8008972	-368.8318481
1006938683	568.2888794	121.124461	112.402729	-370.237793
1052036417	353.2926331	110.7103543	115.1484085	-368.0816345
2080051910	433.780304	113.9797914	166.308696	-282.5793457
1079265284	470.0826721	140.2211525	174.9597046	-328.0050354
69491017.49	329.0187683	157.3134239	221.6707596	-441.8709717
213577942	284.9695435	154.3573361	173.7015366	-396.5435486
971750156.4	374.6818848	133.4810895	157.3439682	-372.8541565
1438152932	539.5236206	135.9954597	172.5604446	-320.8730164
1075317916	337.1065674	108.9695179	143.8001083	-297.7510681
347300196.5	379.0187378	124.1158925	158.783136	-374.8517456
514252429.8	357.3292847	134.7468279	139.5273346	-336.1302795
525812513.5	377.3638916	114.9436105	140.4864166	-323.381073
572568473.6	422.4692993	149.1713374	161.0326996	-386.2203674
855565782.7	363.0499573	138.9179114	138.5811691	-369.8986206
657360538.9	446.3733521	154.1196451	187.546344	-394.7714844
804447736.5	331.2658691	125.9561104	143.8698883	-413.1137085
536737992.2	326.6099548	94.45864107	119.5268707	-194.5423889
1907589465	416.643158	128.6915851	180.2616577	-350.1347961
296266247.8	327.8518982	137.1907812	120.3832184	-292.8562927
84622877.82	285.387207	152.9359602	198.829689	-519.1397095
332387369.7	487.5763855	157.3845802	188.7314636	-403.0277405
1197063043	453.0612793	113.7914814	141.3322632	-321.7616882
494118965.1	202.2836456	148.7582719	117.9715942	-341.662384
1754848900	472.0500793	130.4215817	159.6474152	-389.4977722
191558334.9	281.0594177	109.5657723	140.7766113	-277.6347961
1891817993	422.0577393	103.641599	147.6688599	-268.989624
603186657.9	446.5326843	159.4224737	176.4314514	-363.3684998
1670745240	439.4702454	167.9785849	184.2670776	-455.0783386
736926296.7	390.3078918	145.2883804	156.2065704	-455.0286255
366217817.9	313.8818054	129.0849619	144.0992523	-355.2415161
244515980.6	257.0401611	101.3873537	111.1961823	-327.7084656
1167569059	435.2921143	142.854501	163.2773193	-308.0357666
2859354511	603.8311768	139.0404312	177.0694138	-335.935791
707910462.9	445.0463257	92.11450949	131.6960892	-313.5485229
686314718.6	310.567749	96.47591901	128.3422089	-379.1109924
107690031.9	203.707016	135.5738733	134.1643677	-357.7891541
830086137.2	571.3248901	140.6999589	212.6066528	-330.1938477
232489351.9	363.1116943	147.8330511	123.7153183	-432.1942749
1877816726	440.7946167	147.4936765	188.6714752	-352.9858093
256266830.4	262.3097839	145.8803805	94.79065704	-340.9823608
1007942885	458.1038513	136.2479713	185.5278931	-343.825531
1534267153	412.2368774	136.7199276	185.2712387	-357.6522522
637016887.2	400.6759949	144.5594941	144.2307617	-341.3785706
1719548754	405.9259949	111.0105137	139.9520538	-299.7346497
1945887754	353.2569275	103.5909144	126.1838608	-381.041626
2895660385	535.8582764	108.0862849	152.0987946	-402.8782043
795380596.7	417.6200867	162.4701258	160.4651642	-422.2161865
563729156.5	394.5335388	136.7183411	117.9784073	-389.0985107
374010306.2	364.1020508	150.6936615	137.4093414	-283.2835693
1077853189	309.3871155	150.4349109	146.3197327	-441.3829651
1243107735	412.4796143	127.1906113	147.0916214	-345.2749329
502826240.9	375.0434265	192.2258618	135.6895004	-488.6073303
999919126.5	448.6183777	166.1828363	193.7088776	-442.8442078
1461610744	477.4572449	139.2439307	200.938858	-368.1492615
75580735.3	265.68396	138.9439602	186.2899048	-439.2629089
1244212102	370.4486694	116.5739289	164.7576904	-279.6493835
1493943549	511.6288757	100.5202342	134.9028397	-368.4139709
461588761.6	370.5270386	148.2367663	160.6644272	-305.5701904
1170768460	326.5059204	167.1505333	157.6275024	-484.692749
52224306.93	199.6329193	102.8084746	137.1824371	-287.0647583
55611120.15	191.4218292	140.0568292	91.69187622	-410.4309082
1809580256	492.9114075	129.8489601	168.212793	-426.8202515
548626010.9	426.5518494	169.6500392	170.2668839	-384.3748169
348362281	323.572876	112.3590951	128.0204826	-362.4336853
1377716155	400.9478149	111.6956705	125.4927353	-307.6206055
1733423886	395.7558594	105.1554995	130.1694412	-269.4247437
328432396.4	405.9993591	219.2708257	215.5263977	-459.0435181
534396496.6	341.8552856	127.5801568	171.9007187	-374.2167053
75276514.23	281.4660645	150.5550448	172.1877502	-371.9171143
131374621	282.888092	113.3063504	155.0722748	-265.621582
491264597.3	540.3064575	150.1547532	162.9332367	-446.5859985

log.sigma.3.0.mm.3D_firstorder_Entropy	log.sigma.3.0.mm.3D_firstorder_StandardDeviation	log.sigma.3.0.mm.3D_firstorder_Range	log.sigma.3.0.mm.3D_firstorder_Variance	log.sigma.3.0.mm.3D_firstorder_10Percentile
4.493829152	141.241456	764.7275085	19949.14891	-201.7413025
4.195694145	116.3307954	796.4356079	13532.85397	-163.5333466
4.264163233	125.4091137	646.1218872	15727.44581	-191.1331055
4.545841456	143.8755101	829.1108093	20700.1624	-175.3140015
4.607285772	155.9677153	759.2113647	24325.92821	-211.1699066
4.119198672	109.7997733	551.9068451	12055.99021	-192.4666809
3.834354015	90.13753772	513.5682068	8124.775706	-123.1871063
4.677263301	164.4857839	880.8056335	27055.57311	-243.2470459
4.648659922	155.2430903	840.6789246	24100.41709	-232.7164063
4.160497748	113.4711655	781.0396729	12875.7054	-150.6561966
4.119334969	117.0105862	607.4205933	13691.47728	-206.9528992
4.377938869	130.3398266	714.7330627	16988.4704	-172.0912201
4.224702021	117.9275204	682.2161865	13906.90006	-140.4801559
4.277788316	125.4203697	653.5812073	15730.26913	-166.0805542
4.29077844	119.9427321	639.5570068	14386.25899	-139.3282623
3.592572674	86.0402473	863.7934265	7402.924156	-94.4749527
4.461952449	141.8038745	705.9087524	20108.33882	-234.2624039
4.391280868	134.9136618	691.3199463	18201.69615	-173.3996567
4.631483439	161.9940017	719.3309021	26242.0566	-225.5556915
3.949131518	96.6957395	586.7072754	9350.066037	-134.9034882
3.925929263	94.99864475	760.4492798	9024.742505	-139.422821
4.375199722	130.0317526	825.806366	16908.25669	-159.4392395
4.188588861	115.0683055	685.4519958	13240.71493	-153.6702118
4.404543936	135.6774741	770.172699	18408.37697	-163.914505
3.982608445	100.9919662	686.1028442	10199.37724	-157.2703537
4.468733983	141.4765025	779.803833	20015.60076	-212.7205582
3.846599251	94.33283241	829.8166199	8898.68327	-116.4715462
4.182883903	118.185089	692.4128418	13967.71526	-181.2905273
4.450415349	150.5183118	750.7822266	22655.76218	-201.6218079
4.18290297	114.4026775	938.5266724	13087.97262	-181.2104843
4.063904856	105.5366607	721.3742676	11137.98675	-153.8917938
4.171809968	113.6193371	716.3596497	12909.35376	-126.6583023
4.480720411	140.2197062	798.0877075	19661.56602	-188.7249146
4.456745933	157.0090658	770.88974	24651.84674	-192.6250671
4.526833313	153.5783048	681.513092	23586.29571	-232.3209518
4.377839675	132.720431	747.5360413	17614.71281	-193.847998
4.367422292	134.6612177	860.396637	18133.64355	-171.9256912
4.147708037	108.9677659	634.8576355	11873.974	-141.619838
4.297873485	123.6062016	753.8704834	15278.49308	-157.7199707
4.38867821	132.7057915	693.4595642	17610.8271	-208.0033417
4.206646319	113.9264309	700.7449646	12979.23167	-153.5780655
4.5284606	147.5085943	808.6896667	21758.78539	-219.355661
4.381356963	136.5025037	732.9485779	18632.93351	-215.1122055
4.609305001	153.7745258	841.1448364	23646.60478	-219.8486298
4.350186267	125.3938608	744.3795776	15723.62034	-177.2124985
3.810371689	92.17404123	521.1523438	8496.053876	-130.5693954
4.393749049	128.520295	766.7779541	16517.46622	-160.3072586
4.319749672	130.8648896	620.7081909	17125.61933	-224.7455688
4.20227167	152.9202744	804.5269165	23384.61034	-202.1690857
4.524215097	157.254773	890.604126	24729.06363	-239.3126953
4.19799746	113.4800033	774.8229675	12877.71115	-147.3021149
4.176408187	138.7416562	543.9460297	19249.24715	-241.0895477
4.391947273	129.9866764	861.5478516	16896.53604	-174.4021912
4.132637104	109.5345807	558.6942139	11997.82438	-148.8473969
3.958547771	103.637616	691.0473633	10740.75545	-139.1241028
4.641048459	157.1781356	809.9011841	24704.96631	-234.4926361
4.722976343	166.1548724	894.548584	27607.44163	-246.6200165
4.500531628	145.2394553	845.3365173	21094.49937	-219.9546082
4.320608421	125.0690836	669.1233215	15642.27566	-193.421521
3.998684685	99.68443634	584.7486267	9936.986849	-150.0008392
4.42179821	140.3604449	743.3278809	19701.05449	-190.3126343
4.478112986	138.8942874	939.7669678	19291.62306	-167.3479004
3.867598549	91.43216182	758.5948486	8359.840216	-110.877401
3.870645854	95.55550203	689.6787415	9130.853968	-130.4239044
4.332722775	130.8113494	561.49617	17111.60914	-212.9357758
4.451780106	137.1215803	901.5187378	18802.32777	-141.207132
4.436915859	144.4867131	795.3059692	20876.41027	-248.7182663
4.52310018	147.4419838	793.780426	21739.13859	-196.2338715
4.256306038	133.2349304	603.2921448	17751.54669	-248.6138153
4.429575848	136.0799754	801.9293823	18517.7597	-171.3982697
4.39493791	136.1726206	769.8891296	18542.98259	-171.9136581
4.44318291	140.9915031	742.0545654	19878.60395	-218.0534576
4.120487101	110.3841504	705.6606445	12184.66065	-146.5234512
4.015025554	101.355352	734.2985535	10272.90739	-144.3264847
4.050141842	107.0907036	938.7364807	11468.41881	-116.8134117
4.640126251	159.0892202	839.8362732	25309.38	-247.0309296
4.377922417	130.6743457	783.6320496	17075.78462	-211.940213
4.380481112	142.5166003	647.3856201	20310.98136	-222.6675537
4.459871421	140.6267959	750.7700806	19775.89573	-225.6848724
4.290347381	124.3452907	757.7545471	15461.75133	-177.1779785
4.72333672	172.4100002	863.6507568	29725.20816	-309.5244629
4.722912326	165.6307796	891.4625854	27433.55514	-237.1039124
4.469056812	137.500173	845.6065063	18906.29757	-163.2080719
4.273519695	138.0298168	704.9468689	19052.23033	-178.2641357
4.213821098	116.5486486	650.098053	13583.5875	-138.9787445
3.993916066	100.5097639	880.0428467	10102.21265	-113.6006165
4.455266249	144.6258132	676.097229	20916.62584	-212.612114
4.62259236	164.4195529	811.1986694	27033.78938	-269.1973724
3.951875305	102.7912678	486.6976776	10566.04473	-120.8710449
4.222018163	129.8459548	601.8527374	16859.97197	-252.4190002
4.386189008	129.7240477	919.7316589	16828.32855	-168.975827
4.58416412	159.0330799	810.9266663	25291.5205	-251.7539444
4.129183637	110.3217683	686.0065613	12170.89257	-158.778215
4.157075212	110.5659752	708.5684204	12224.83488	-152.4360916
4.081350997	104.8718825	665.180603	10998.11175	-140.1454163
4.839842125	214.0437595	865.0428772	45814.731	-340.5980103
4.352762479	126.6297247	716.071991	16035.08719	-159.0273895
4.315193895	150.5296203	653.3831787	22659.16658	-205.9976227
4.127686936	111.7749647	548.5096741	12493.64273	-137.4146973
4.492282072	146.1696766	986.8924561	21365.57436	-197.4673706

log.sigma.3.0.mm.3D_firstorder_Kurtosis	log.sigma.3.0.mm.3D_firstorder_Mean	log.sigma.3.0.mm.3D_glrlm_ShortRunLowGrayLevelEmphasis	log.sigma.3.0.mm.3D_glrlm_GrayLevelVariance
2.798939473	-18.59385362	0.008027221	32.58081555
3.211558064	-13.62150196	0.00647983	23.13676948
2.748922285	-35.02722995	0.021311745	25.39900273
2.506038598	20.66425414	0.008933675	33.61837215
2.686300482	-1.691702528	0.012355211	39.56761388
2.207664697	-49.94141747	0.015522669	19.3787102
3.410791512	-4.4117188	0.016369818	13.28360967
2.286461167	-17.84674731	0.008050845	43.21722404
2.550228326	-26.7248663	0.007633072	39.05086491
3.341336175	-24.10601487	0.008966282	21.29234872
2.21303496	-34.99158755	0.005746637	22.28979313
2.832656993	-17.26203427	0.012743883	27.9575948
2.925398513	6.276083454	0.015126326	22.58948662
2.122114645	1.971436931	0.013980292	25.19099044
2.612362875	19.48984624	0.012602885	23.67361616
5.840453755	5.127202436	0.006472656	13.69619878
2.186164321	-32.0344398	0.013170326	32.05598308
2.726008213	-9.445576966	0.023499666	29.63589034
2.223841244	11.57521387	0.01352525	41.81150903
3.072013755	-8.03955892	0.016478034	15.78408769
3.486482922	-30.04754854	0.005977955	15.44070774
2.989651283	5.98408922	0.01443154	27.26187736
3.081212726	-18.80873163	0.009802942	21.76987171
3.146959469	11.69811971	0.005857113	29.84226767
3.229377553	-44.93807758	0.011018671	17.06452091
2.838399205	-36.83881158	0.01158999	32.56457868
4.524160088	-7.310392045	0.003900694	15.86174218
2.946630281	-42.01189088	0.009486606	23.01751812
1.852049715	6.473495528	0.008598189	36.26007644
3.836136837	-39.78897373	0.008633566	22.69617265
3.202781459	-33.44840518	0.006526817	18.48455872
3.028548129	9.057543719	0.009557199	21.44956125
2.370594448	0.636868405	0.009920935	31.78574992
3.004700419	9.780931109	0.01504665	39.73323552
2.406841611	-15.48843071	0.017048712	38.14664955
2.127754398	-14.22984356	0.00817105	28.33693821
2.810481776	-19.00319757	0.012059977	29.68213513
2.544247067	0.617928874	0.012322237	19.47226769
3.260625527	11.2366231	0.008465682	25.04141967
2.449242958	-23.36408596	0.012700969	28.5000239
2.956634619	-15.25784839	0.012357519	21.52181615
2.420305023	-22.21041425	0.008837433	34.80817656
2.108375018	-25.79249111	0.012697766	30.11303656
2.459077147	-10.30825979	0.008867311	37.98376349
2.536287628	-11.88786841	0.005512619	25.53611806
2.653418212	-20.64899507	0.040387248	13.9358395
2.602019681	6.637608586	0.006366946	26.9243796
2.13527693	-41.17877027	0.040060683	27.57982836
4.188179958	-2.190336266	0.012865973	38.48314461
3.279196893	6.390810165	0.011061307	40.25424728
3.085501482	-8.413684708	0.009330502	21.45178725
1.600544547	-53.66354736	0.017085026	30.45118577
2.919045541	-10.64203631	0.005710199	27.93142149
2.520751193	2.614205894	0.013192582	19.44759714
3.503969544	-0.908617813	0.01279004	18.50575911
2.361249619	-26.65630925	0.021089155	39.62646866
2.246835524	-24.68528627	0.006034484	44.11907583
3.076981265	-3.770162398	0.005819476	34.62725797
2.544186339	-31.94764024	0.009334308	25.69905904
2.360631044	-18.50428724	0.008103696	16.1298686
2.462583049	-26.57732038	0.014599294	31.91555655
3.459728057	6.37325974	0.008370575	31.77156173
3.683190589	11.19118597	0.006479379	14.22902988
3.408284669	-13.29469749	0.005227156	15.9995259
2.438085701	-35.61833752	0.017117033	27.59444658
3.507603916	31.53015484	0.0074321	31.13837246
2.831778936	-31.27620085	0.008904525	33.75886824
2.363276326	-3.904616395	0.007248138	35.10173097
2.000000663	-59.409921	0.023764208	28.01426314
2.672603888	-6.763873125	0.009448426	30.13222617
2.387173907	-12.22113	0.007169235	29.96215302
2.229587922	-31.91932633	0.013118524	31.94183811
3.249395304	-11.77597109	0.01065825	20.43377684
3.029041895	-21.40490971	0.004820744	17.21748142
4.335969802	14.63646735	0.003605934	19.87645048
2.24667231	-32.97213638	0.010699639	40.66307023
2.505972895	-40.20099705	0.008757239	27.58486284
2.575094828	-48.96527599	0.032974659	32.69960726
2.293350124	-53.43001662	0.007197915	31.77944009
2.449148888	-26.75257482	0.009300983	24.94197837
2.120203864	-85.00337519	0.008956165	47.62845823
2.32375437	-13.53439777	0.00812179	44.19590932
2.732846893	21.96758207	0.007096763	30.90321688
3.857106431	15.91206319	0.013049551	31.6654249
2.484217337	2.427631149	0.010174347	21.96043288
4.122392471	-1.450805711	0.005077454	17.28003436
2.325270485	-32.51942582	0.017613565	33.53593576
2.232940529	-30.09171667	0.005885975	43.02640942
2.47300604	1.880885701	0.015728197	16.9917184
2.779485828	-52.4970802	0.018869581	27.51381061
3.18303209	-5.694198157	0.004374561	28.44725349
2.568804794	-59.072966	0.011241879	40.6370104
2.891601967	-21.29961681	0.007656334	20.73878164
3.091407149	-15.84575394	0.010880184	20.40768916
2.828358388	-7.717986886	0.014381179	18.17999466
1.824856136	-47.59163775	0.01881338	73.60449103
2.913088231	15.54378414	0.009946863	26.07841936
2.865633077	2.766753484	0.035005137	35.93556453
2.764663516	18.56572937	0.019841894	20.26652928
3.891525324	-34.36386993	0.007362417	35.63467722

log.sigma.3.0.mm.3D_glrlm_LowGrayLevelRunEmphasis	log.sigma.3.0.mm.3D_glrlm_GrayLevelNonUniformityNormalized	log.sigma.3.0.mm.3D_glrlm_RunVariance	log.sigma.3.0.mm.3D_glrlm_GrayLevelNonUniformity
0.008385668	0.050060487	0.076716491	33.05737091
0.006996253	0.062295084	0.210046996	113.5004783
0.022514944	0.058598839	0.069777479	23.86311326
0.009282653	0.04762988	0.085353811	116.9255417
0.012649007	0.047426871	0.074426295	16.01360052
0.016504892	0.063078195	0.091123532	37.46660712
0.01747497	0.08554825	0.109853059	26.32411928
0.008398773	0.043730098	0.06543469	41.39266253
0.007985304	0.044252307	0.082165326	85.89848648
0.009582151	0.065922712	0.121966573	185.3523546
0.00598683	0.064205443	0.089133591	49.4082651
0.013521721	0.05422523	0.099460769	73.99264104
0.016041485	0.060618313	0.107432109	115.0235247
0.014760332	0.05610625	0.080755068	58.44237621
0.013093585	0.05730044	0.075352117	48.85328507
0.007365535	0.105935995	0.461878796	593.0277241
0.013805959	0.04990866	0.072610469	32.69316151
0.025119969	0.052787069	0.117319001	112.8015981
0.014214828	0.044091359	0.062822189	24.2306876
0.018083452	0.072320017	0.228706452	176.4616092
0.006515301	0.076641219	0.190384179	352.1370172
0.015256113	0.054372539	0.081727963	87.17426898
0.010525517	0.06381108	0.111714908	159.448189
0.006029338	0.054075484	0.072474026	24.36911016
0.011784658	0.072873632	0.121468383	79.94866523
0.012224447	0.05121451	0.083404452	32.99862681
0.004323202	0.083088541	0.311712854	243.051506
0.010135834	0.063002115	0.105416952	83.00800881
0.008953587	0.049951009	0.079766576	82.8298821
0.009327155	0.06308339	0.241798143	138.8097115
0.007038724	0.069451061	0.12866627	200.2649569
0.010368297	0.063286975	0.166943107	334.9329141
0.010403724	0.049298154	0.085606535	93.18781075
0.015144165	0.0534205	0.040137698	5.359115448
0.017568287	0.049778929	0.048881885	15.8302861
0.008643183	0.052227988	0.093597527	97.65415569
0.01280572	0.054903472	0.113298862	145.0517907
0.013031551	0.062452634	0.123356365	190.5948859
0.008820788	0.061104559	0.098329804	47.24772193
0.013333019	0.053067784	0.09413249	51.55635249
0.013356571	0.062043871	0.138238856	82.32089638
0.009220426	0.04877933	0.069714637	43.64440279
0.013196291	0.05254432	0.080245897	80.56675432
0.009309023	0.045395602	0.069651794	43.81617559
0.00574997	0.054811983	0.080206476	96.21819204
0.044509486	0.07888431	0.154397116	157.4997971
0.006682051	0.053105192	0.090918913	210.4086319
0.042724893	0.053783472	0.077750319	29.35442443
0.012955219	0.075949155	0.054757821	9.683808836
0.011513706	0.055570439	0.071833872	25.96104055
0.009980574	0.061489269	0.147157271	190.0325148
0.018203147	0.059770663	0.097444769	45.78427549
0.006091892	0.053572297	0.137781933	185.7248752
0.013858191	0.064380321	0.091386664	35.18860079
0.014050983	0.077601297	0.275832779	432.9806404
0.021873714	0.043940872	0.063494877	36.53152177
0.006270882	0.041637722	0.062466966	86.45858775
0.006011884	0.05254559	0.079455983	63.44746797
0.009839769	0.055741095	0.11082415	41.57344137
0.008583125	0.068839789	0.108572076	55.54992002
0.01550633	0.052188906	0.091202672	102.6881025
0.008882373	0.050892451	0.126403464	254.3261528
0.007050587	0.080273309	0.208295407	217.6176731
0.005718089	0.079978701	0.303711094	184.5690334
0.017721085	0.055208142	0.055169202	11.39018316
0.007773749	0.054262556	0.093306351	78.21661733
0.009215899	0.056198904	0.066240022	20.88814787
0.007722273	0.047367363	0.106467388	139.7678021
0.024842987	0.058877117	0.075930835	24.64848099
0.009922865	0.052041587	0.09119128	97.17024393
0.007650152	0.053006283	0.108710988	148.1765084
0.013873332	0.05030157	0.086029	52.81216751
0.011583133	0.065078654	0.18527748	298.0079949
0.005271995	0.070173195	0.202635694	414.6957609
0.003917479	0.074314299	0.215140468	595.9542269
0.011098971	0.044395546	0.062242298	46.91412888
0.009158396	0.054079248	0.080791941	56.33648142
0.035018377	0.053005495	0.077013861	30.24677861
0.007541505	0.050070875	0.083227395	82.50150834
0.009868623	0.056746073	0.097461404	148.8595648
0.009228746	0.041427826	0.050501112	19.95262567
0.008351051	0.041808926	0.052644148	53.42342723
0.007480373	0.051453799	0.101086371	132.6630776
0.013163814	0.061953769	0.053106268	8.582873475
0.01089853	0.059751499	0.112233484	185.7683279
0.005545391	0.074757567	0.190285779	359.1082955
0.018684098	0.049707795	0.075046211	36.21910553
0.006087916	0.045834432	0.058956707	67.53178841
0.016221922	0.069268896	0.085432317	11.75993108
0.019051319	0.061877441	0.043234169	6.236749926
0.004666625	0.055166125	0.185309378	195.512967
0.01176233	0.046531051	0.060331722	31.0619937
0.008144315	0.063427708	0.216491047	57.15944076
0.011629117	0.063710861	0.168883084	232.7826833
0.015507911	0.065902158	0.159662237	342.4243526
0.019141627	0.039496831	0.051056528	9.546653257
0.010280553	0.056639678	0.085271085	63.9962131
0.038271301	0.055533658	0.052300761	6.519324743
0.020955667	0.067432024	0.088870734	23.6779906
0.007720605	0.053221484	0.100418355	39.63101727

log.sigma.3.0.mm.3D_glrlm_LongRunEmphasis	log.sigma.3.0.mm.3D_glrlm_ShortRunHighGrayLevelEmphasis	log.sigma.3.0.mm.3D_glrlm_RunLengthNonUniformity	log.sigma.3.0.mm.3D_glrlm_ShortRunEmphasis
1.221755296	272.9713717	581.3218959	0.951019428
1.514945034	229.37824	1459.553154	0.913675398
1.207212067	146.2001926	360.0394971	0.952702621
1.23913903	285.5281137	2148.678019	0.948802538
1.20924467	294.7332892	301.2990279	0.95544948
1.264451678	146.4967732	510.8114795	0.942189628
1.315082121	133.4936411	259.420215	0.933403775
1.186639027	316.2423304	849.7281034	0.958811536
1.230456166	296.2163431	1706.242518	0.95060678
1.337840113	195.7247344	2350.627161	0.931131957
1.247641211	299.3785365	674.3551221	0.948611259
1.281956184	185.971051	1168.75695	0.940227875
1.30624508	152.4124459	1607.250439	0.935478
1.233962553	175.8514514	910.3248042	0.948430497
1.218471534	191.0866502	751.1886547	0.951515876
2.026695055	178.9010861	3950.962456	0.863796893
1.210417572	224.9117323	581.1830902	0.95363054
1.311613591	149.2243125	1829.49736	0.939527224
1.185925051	257.8529538	492.2709843	0.957601103
1.554603425	111.7146673	1931.574152	0.908599467
1.48760746	205.5344132	3672.522833	0.913068868
1.236330133	182.9395428	1404.779716	0.948505862
1.309367541	177.7528586	2121.746181	0.936645821
1.21439449	406.6991124	397.8435813	0.951733712
1.330023644	148.5015019	927.1578212	0.934547794
1.245396485	222.2228327	559.1520784	0.945450202
1.724543002	283.094948	2207.891813	0.890712323
1.292856481	182.2229146	1126.528036	0.939332566
1.216577051	270.9538832	1480.690325	0.955763621
1.583661378	197.7391047	1724.447039	0.90530695
1.347531645	210.7809177	2415.106316	0.931362923
1.427635616	177.6026842	4337.912762	0.922482571
1.244411962	230.4989635	1649.345876	0.947325603
1.116505659	385.5762601	93.66779502	0.973508748
1.139940835	282.5808688	293.2926813	0.968817055
1.263348011	238.1118259	1619.724629	0.944531524
1.3053342	185.4216693	2260.321737	0.939346945
1.333718808	166.2053182	2571.103626	0.93376588
1.276087727	262.8309268	672.3552839	0.944038256
1.261174099	204.2606766	842.5564431	0.945401388
1.377319423	176.4420353	1095.299313	0.925612612
1.205316271	268.5512183	792.4857589	0.953591249
1.229826943	226.5663379	1347.616717	0.950056329
1.198706392	286.6746277	861.6487328	0.956368335
1.22639698	300.5636279	1545.830538	0.951176235
1.405935384	70.89242903	1644.748412	0.924163508
1.25198521	263.2078213	3454.472822	0.947204904
1.220888494	141.4915186	482.655074	0.952444075
1.162035413	477.6938519	115.8368231	0.962938701
1.206987966	339.1732967	415.2604826	0.954502325
1.37759622	184.7237976	2577.588914	0.92976911
1.266780461	175.3113204	667.2111764	0.946127969
1.356301602	271.3677332	2919.223545	0.933207771
1.262980714	169.9350743	472.0151203	0.943307118
1.658729627	137.848278	4295.573039	0.897497561
1.182609423	241.732492	748.0436873	0.959426014
1.17984911	373.5916906	1873.531744	0.960204146
1.229021232	385.1896064	1059.951916	0.949753015
1.303843046	218.4755916	635.1391491	0.938227738
1.304191616	195.4524708	685.847377	0.936918194
1.25212013	182.0755081	1715.334685	0.947351582
1.331586946	236.34914	4239.500099	0.936198206
1.522086951	193.203031	2167.340947	0.911488317
1.715327275	243.1459563	1748.269758	0.891264414
1.163169034	219.7970707	187.2304572	0.962610474
1.257796285	266.5209786	1255.377354	0.946546653
1.192659777	318.2437423	332.3787894	0.956810379
1.282096344	260.7061568	2554.664628	0.944410848
1.212672961	170.4379835	372.5519228	0.954907474
1.253009633	224.6621746	1628.306125	0.947289809
1.295609565	246.3644011	2401.421969	0.940743927
1.243488954	199.2155113	915.9826578	0.947687008
1.467363789	156.3043524	3716.653637	0.918224101
1.500687783	244.6863376	4746.009505	0.914572638
1.531686324	318.1047253	6360.704729	0.909959074
1.178992329	290.8376187	952.0635633	0.959963702
1.23333072	238.8695466	913.3388201	0.948996508
1.221267272	141.8632988	503.2119714	0.951685862
1.232442895	287.626403	1453.020125	0.951014379
1.276696901	196.1091284	2251.51316	0.941128161
1.147288639	334.531254	441.6602497	0.966574211
1.154902583	354.4192979	1164.387258	0.964385737
1.276148629	283.2819706	2227.697594	0.943585698
1.1533228	380.7838277	127.0910171	0.966313497
1.304413286	173.147341	2654.795553	0.938819625
1.493310857	237.9697147	3812.516741	0.910500912
1.216197076	179.5452086	643.536665	0.952407891
1.169542118	399.0821288	1334.371878	0.962083318
1.252139447	164.962984	147.4915864	0.944469862
1.131493599	254.5696693	93.08197803	0.968871069
1.449241716	338.4165237	2916.345028	0.924047747
1.180253302	236.893133	598.6555994	0.958274612
1.509845008	217.7949619	732.0475689	0.918851173
1.426696249	173.4578796	3003.062802	0.923643587
1.412764419	135.3940905	4272.086186	0.923880711
1.148662275	368.8697571	221.5504708	0.966300708
1.245164409	269.3167751	982.8036596	0.946493644
1.152602084	272.7121039	107.3503594	0.965308038
1.258037757	161.1544342	303.9797883	0.943996494
1.27776194	316.4131239	642.098076	0.942606714

log.sigma.3.0.mm.3D_glrlm_LongRunHighGrayLevelEmphasis	log.sigma.3.0.mm.3D_glrlm_RunPercentage	log.sigma.3.0.mm.3D_glrlm_LongRunLowGrayLevelEmphasis	log.sigma.3.0.mm.3D_glrlm_RunEntropy
334.0511565	0.935280017	0.010019781	4.826242796
364.3042726	0.876856413	0.00987402	4.806054381
173.4103387	0.938319745	0.027977245	4.568696165
369.8704384	0.931630419	0.010843743	4.917428626
374.3995062	0.940218556	0.01393517	4.875367536
186.4421766	0.923794712	0.020974733	4.494995644
183.7492715	0.912805296	0.022303732	4.243775212
379.9988655	0.944649163	0.009973646	4.96654479
374.3294292	0.933658934	0.009600867	5.006758707
263.6980354	0.907533396	0.012537074	4.652918889
394.0068267	0.930425077	0.007130083	4.472831361
236.6751169	0.920637392	0.017154454	4.795296303
205.9959059	0.914843113	0.020293683	4.66768078
224.7650131	0.931677446	0.018340832	4.636813048
241.5924782	0.935827071	0.015263907	4.632491792
389.3142641	0.803700546	0.013780396	4.566042819
280.4727189	0.93850562	0.016741249	4.767199142
192.4550294	0.916347803	0.033618809	4.824476705
311.9215024	0.944356331	0.017283207	4.901631426
177.3169379	0.870705517	0.027417176	4.574417637
317.1268577	0.879329132	0.009458332	4.543289086
230.2611456	0.932110912	0.01887087	4.734364936
231.5475943	0.914936766	0.013981666	4.63704358
523.032525	0.936830321	0.006769316	4.718291427
194.547369	0.911001789	0.015642723	4.44550772
277.0408756	0.928397251	0.015033908	4.833644461
523.3473987	0.843268676	0.007044663	4.621955816
232.2959183	0.919211981	0.013217003	4.606215855
348.9803061	0.939463935	0.010635772	4.774977611
336.389207	0.865127398	0.013182859	4.837471518
283.6320145	0.906985748	0.009629628	4.54936903
255.9979001	0.89229472	0.014648413	4.723106313
300.8160197	0.929811949	0.012609868	4.856615795
448.2455544	0.964497041	0.015557236	4.56662686
329.0848617	0.957831325	0.019712579	4.72737186
315.8881197	0.925590251	0.010834198	4.768494238
242.1506322	0.917254274	0.016447886	4.804169749
232.0954638	0.909843585	0.016517486	4.618596369
356.9451127	0.925380009	0.010495552	4.678890535
262.9474099	0.926083449	0.016268607	4.772720478
252.5198223	0.899958251	0.017933237	4.712281404
332.3337883	0.938816692	0.010922877	4.849571566
295.4687174	0.933804928	0.015477457	4.736549995
351.1257514	0.941688555	0.011235348	4.913279565
386.6002121	0.934750553	0.006822341	4.705877757
95.38729376	0.895905262	0.066327085	4.334017747
340.5404009	0.928720834	0.008168607	4.785659932
173.4576229	0.936139332	0.055063731	4.636934474
581.1216109	0.951205511	0.013337052	4.429685162
427.5081702	0.939637827	0.013426007	4.829724685
259.8056897	0.902498203	0.013288848	4.700761277
231.2446351	0.926146405	0.023618465	4.546291716
379.0499124	0.907233306	0.008056719	4.872235602
221.8733601	0.92476897	0.016898885	4.504901015
238.6965012	0.853689312	0.021683886	4.684910372
288.7095158	0.945830052	0.025204807	4.92034626
454.4484078	0.946858887	0.007317988	5.014742059
504.3148081	0.933666488	0.006866406	4.861164216
290.0019823	0.916178416	0.01222956	4.738355307
264.2562294	0.915917227	0.010926996	4.419269044
225.6894381	0.928522162	0.019766933	4.807031361
325.8558527	0.912152105	0.011398097	4.940943126
310.0947751	0.87635051	0.01033797	4.485424021
458.2990965	0.844238515	0.008830739	4.623691628
259.8244863	0.950726693	0.0202279	4.543162207
346.3913178	0.927831988	0.009347522	4.838461352
393.8362855	0.943186255	0.010543189	4.717701155
337.482804	0.922956618	0.010102199	4.926449051
206.3021678	0.938599517	0.03032987	4.548490033
283.2823701	0.928393834	0.012194874	4.814498616
320.698853	0.919534413	0.010077748	4.816205044
252.7356387	0.929958438	0.017321594	4.804529122
232.5812654	0.885672184	0.016662808	4.697275996
383.1966527	0.879644477	0.007839961	4.615558215
522.7356389	0.873135579	0.005723457	4.710574895
356.032369	0.946856907	0.012810459	4.924681106
304.2697749	0.932649267	0.010954186	4.731086408
167.9130674	0.935435057	0.044383108	4.706622528
363.7538829	0.934109541	0.009183056	4.808167633
256.8725233	0.921725499	0.012526392	4.708277304
382.5723933	0.955586081	0.010427704	4.944824252
422.6892752	0.952905409	0.009323711	4.986635481
377.9635742	0.923352436	0.009266742	4.87848767
456.2604904	0.954907162	0.013668218	4.461190015
230.7880234	0.916906744	0.014459966	4.645907019
370.6314226	0.877156262	0.008095114	4.624119553
213.8059394	0.936523631	0.023502146	4.782670069
486.3283059	0.949296193	0.006962624	4.902958032
217.4391788	0.927700715	0.01841021	4.264876514
300.2946028	0.95970696	0.019810898	4.368160078
513.72252	0.891185293	0.006331351	4.941345579
274.9237446	0.9455219	0.014015394	4.871692783
348.2464757	0.881228361	0.0109956	4.691495163
261.3756009	0.893191649	0.015454971	4.700410591
196.4630154	0.894817033	0.021392907	4.618284735
440.5151134	0.955305564	0.020572122	5.028876442
356.502692	0.929086538	0.011745271	4.7305749
318.1587936	0.954346467	0.051379908	4.487342428
212.5203951	0.926324335	0.025981741	4.47868015
406.9212515	0.922695644	0.009375805	4.889303692

log.sigma.3.0.mm.3D_glrlm_HighGrayLevelRunEmphasis	log.sigma.3.0.mm.3D_glrlm_RunLengthNonUniformityNormalized	log.sigma.3.0.mm.3D_glszm_GrayLevelVariance
284.0768361	0.879591562	39.24998859
248.6018952	0.800121602	33.15313636
151.3657848	0.883350033	27.42533948
300.4564075	0.874574113	37.48357284
308.6028483	0.891015571	48.38637899
153.8047266	0.859361259	19.74305731
142.1849567	0.841346788	18.87422802
327.7813468	0.897450312	44.72011863
310.1553568	0.878549027	44.30780878
207.4027353	0.835412523	28.6648734
315.4488813	0.875032582	22.87493062
195.0855535	0.855559576	34.66858583
161.7400427	0.84550938	28.37120929
184.6679986	0.873462387	25.06499619
200.3339532	0.880562591	28.82462324
203.9374939	0.704627172	27.04622697
235.1269933	0.886112242	31.13878879
156.401762	0.854865859	31.66191542
267.7882835	0.89484783	38.88460682
121.0792661	0.790022313	20.31889446
222.4526172	0.798288959	23.7565581
191.4231576	0.874709033	31.01049948
187.0834986	0.847955172	25.01301394
427.8705393	0.881696625	36.8013434
156.3035802	0.843906493	21.96989125
232.2053039	0.867008545	37.46063609
314.5405859	0.75401199	27.59404237
190.8970065	0.853805179	27.36025968
283.9834581	0.891580376	34.49098354
216.5766311	0.782862126	34.24376119
223.0347709	0.836469142	21.80908479
189.8631323	0.818238782	25.43964847
242.9576948	0.87157233	36.5786489
397.0258802	0.933116716	41.62109375
290.9393679	0.921746285	40.62033951
251.6973852	0.865294793	28.74056877
194.8641765	0.854337411	33.87103886
177.2458897	0.841640582	23.98065286
278.4967502	0.866526591	32.72737967
214.3689559	0.866880522	31.49974208
188.9947088	0.824231196	27.68872247
280.3667909	0.885282624	37.49153919
238.7996571	0.877758853	31.13424347
298.2600049	0.892032905	38.14235165
315.6567612	0.879914498	30.16876011
74.93776242	0.821983164	15.00983472
276.7375789	0.871197359	32.70855583
147.240372	0.883459707	27.45210922
496.730639	0.907796214	53.96123858
354.9398386	0.888076511	48.12094942
196.5691421	0.833190667	28.0192522
184.567839	0.869735745	24.15020006
288.4038605	0.840907675	34.76524364
179.1967426	0.862616859	22.72326456
151.5927531	0.768543957	25.4321477
250.1329555	0.899153806	41.23552731
388.2429289	0.901344376	45.43856229
406.6386224	0.876960291	43.44898559
230.6180697	0.851062681	29.72430663
207.4959472	0.848591086	18.76295521
189.7820976	0.871367781	33.51623458
250.9298689	0.847282429	40.08225468
210.3085951	0.796968479	20.99469578
271.3128255	0.756019996	25.85498071
227.2371001	0.906738116	28.58530427
280.3233582	0.870074598	41.68038584
331.8350169	0.893336501	38.59545949
273.5087774	0.865141903	35.23405574
177.0222365	0.889196631	25.04739135
234.9448407	0.87147466	34.75303336
259.1130196	0.857493513	31.46212404
208.6639211	0.871938494	32.98931973
167.6952538	0.809816991	26.59055948
264.7414557	0.801769537	22.0372336
347.9441802	0.792201992	33.7070842
302.7251957	0.90032251	43.98858935
250.6244857	0.875467244	32.14813658
146.5975535	0.881252345	32.74621377
301.0511702	0.88046479	32.11627206
206.9927465	0.857432175	27.39740945
343.5358506	0.916248424	47.13795418
367.1889565	0.910705877	46.81080886
299.4624863	0.863253062	36.07358174
394.4068814	0.916437111	40.31693639
182.798757	0.852787611	22.95509211
258.6100809	0.792691486	27.6106252
185.9322656	0.882760317	32.54034255
414.8640998	0.905253387	42.046968
174.3421456	0.866589248	21.33159763
263.3942148	0.92237297	30.18083333
363.8339155	0.821795883	38.94809968
244.1511435	0.896157743	42.77033798
235.7758099	0.811375621	26.54015281
186.5308873	0.820634307	27.48931739
144.9293539	0.82090329	23.24267187
381.9571869	0.915738762	76.07530612
284.9637405	0.869271188	33.18235798
280.9161434	0.913419435	34.09375
170.2207491	0.86431847	21.14342247
332.0281162	0.861185311	54.31118862

log.sigma.3.0.mm.3D_glszm_SmallAreaHighGrayLevelEmphasis	log.sigma.3.0.mm.3D_glszm_GrayLevelNonUniformityNormalized	log.sigma.3.0.mm.3D_glszm_SizeZoneNonUniformityNormalized
244.448623	0.045676589	0.426383309
199.5701789	0.048065269	0.345058583
131.9080725	0.055064547	0.412198201
183.9527707	0.045209921	0.334389322
148.8885327	0.042205306	0.291473357
105.0083004	0.06425508	0.276096955
88.90895844	0.068326635	0.261052838
244.9774977	0.042137516	0.388692116
215.6556777	0.041782934	0.352408854
169.7928585	0.05207076	0.346111689
173.5255969	0.059812709	0.285483166
159.7521162	0.047178859	0.380940647
97.98816294	0.052511648	0.252383544
119.8881777	0.055107453	0.312031702
125.4822607	0.05158855	0.331765278
161.3456449	0.055424734	0.340489944
153.5392241	0.05033557	0.343678213
130.6308018	0.049531531	0.350704046
192.4896806	0.045862107	0.384272667
105.3535141	0.061777934	0.325486646
173.2646478	0.058125335	0.334977351
134.5271243	0.050182102	0.318324662
152.3300116	0.056157845	0.34164225
264.0345604	0.04936744	0.382086311
140.5594761	0.060247098	0.381213703
193.2379565	0.045476466	0.377290702
238.9369876	0.053788869	0.350309197
163.1426227	0.053478369	0.370480469
168.2718184	0.04714731	0.363282652
179.7605653	0.048245937	0.354376275
177.610184	0.06076141	0.341297434
140.3321003	0.055032387	0.305598543
159.7265413	0.045395421	0.325084547
261.6843533	0.049804688	0.451171875
216.8448945	0.045925926	0.451049383
149.393231	0.051262976	0.28841263
162.1764612	0.04784245	0.36919362
123.9478455	0.056245035	0.328197811
160.3067572	0.05105164	0.296757399
155.0425954	0.050602655	0.346691756
143.2464779	0.053143659	0.347423258
182.2237185	0.045600188	0.318985591
139.214624	0.049451961	0.3239878
227.3578997	0.04535813	0.382711911
196.0320942	0.050395287	0.32350203
64.86163377	0.071921394	0.31782807
185.0712643	0.047727542	0.341243622
113.0316642	0.053765859	0.35327299
319.6170689	0.047003787	0.43773669
242.8203689	0.043220268	0.408719805
149.5199158	0.052399916	0.328333036
108.0765667	0.058393352	0.325109264
206.8853497	0.047633648	0.343987437
107.5456005	0.05967021	0.27986667
121.2156105	0.056083004	0.338323655
187.4837788	0.04355868	0.411162791
272.8243545	0.041036368	0.389968043
243.61318	0.043144464	0.356712262
166.158186	0.052382672	0.367540682
125.50258	0.066912465	0.295911512
160.5723116	0.047985917	0.363079405
183.9109769	0.045762679	0.355798337
139.435262	0.062000902	0.294275619
188.5526821	0.054825248	0.343236682
171.8342973	0.0568134	0.417894117
204.7283961	0.044171887	0.353906879
232.131498	0.046371211	0.358833474
208.9246604	0.046306226	0.361327738
129.3411875	0.057661838	0.369737438
184.5378121	0.047293929	0.363835311
194.9302072	0.048628816	0.345561311
151.7919303	0.048843537	0.32638322
137.1255337	0.053561079	0.32569625
207.8097923	0.059663848	0.345961289
234.4301858	0.049134538	0.33484573
204.147361	0.041733163	0.350447995
153.7138814	0.049843616	0.304999435
128.7139008	0.051015306	0.377913259
204.8696261	0.048828561	0.352572696
142.139009	0.052692582	0.322579329
287.7224034	0.041096237	0.470650068
251.1583304	0.040410204	0.41632373
196.5908743	0.0465907	0.363419409
235.8613959	0.052395449	0.400096138
126.0774729	0.057856375	0.304747258
193.1175087	0.054712577	0.331076696
146.1722721	0.049782807	0.365797897
255.8417823	0.043785383	0.385082651
94.64490084	0.070769231	0.312189349
162.6825231	0.057222222	0.408333333
249.604393	0.044981162	0.333387534
215.1910649	0.043971955	0.391651425
145.9630834	0.053970392	0.311019462
132.9827194	0.053033489	0.307393785
109.5373619	0.057458586	0.314775676
277.1436292	0.035714286	0.462244898
138.0936444	0.048293237	0.299917636
191.5183185	0.053710938	0.375
93.02433927	0.064729493	0.269150319
311.5289445	0.039436651	0.427445613

log.sigma.3.0.mm.3D_glszm_SizeZoneNonUniformity	log.sigma.3.0.mm.3D_glszm_GrayLevelNonUniformity	log.sigma.3.0.mm.3D_glszm_LargeAreaEmphasis	log.sigma.3.0.mm.3D_glszm_ZoneVariance
126.2094595	13.52027027	18.95945946	13.27058985
191.1624549	26.62815884	175.0523466	160.9966147
81.20304569	10.84771574	12.07106599	7.217655698
351.7775665	47.5608365	17.9230038	11.64921695
43.4295302	6.288590604	10.66442953	4.859240575
62.67400881	14.58590308	18.27753304	10.25391527
26.36633663	6.900990099	31.23762376	20.10449956
179.5757576	19.46753247	10.6969697	5.993141058
297.0806643	35.22301305	20.50652432	14.42442049
321.883871	48.42580645	90.7655914	79.66881258
83.07560137	17.40549828	20.6838488	12.60731451
208.7554745	25.8540146	29.85036496	22.53669615
149.9158249	31.19191919	38.50505051	26.31390221
126.3728395	22.31851852	21.79259259	14.17226642
124.0802139	19.29411765	16.34491979	10.41165747
371.8150183	60.52380952	3666.892857	3626.421355
102.4161074	15	12.47651007	6.990225666
270.7435233	38.23834197	50.3238342	41.19905232
102.9850746	12.29104478	9.723880597	5.007852528
198.8723404	37.74631751	230.3649755	209.3492785
435.8055342	75.62106072	325.1099154	308.9866968
197.3612903	31.11290323	24.56774194	16.87159209
314.3108696	51.66521739	47.55978261	38.74791942
76.03517588	9.824120603	15.63316583	9.790863867
156.6788321	24.76155718	52.51581509	43.93419409
108.6597222	13.09722222	16.68055556	10.87379437
264.4834437	40.61059603	529.7615894	508.6868015
193.3908046	27.91570881	29.39655172	21.86036978
273.9151194	35.54907162	14.59814324	9.118570102
237.077728	32.27653214	203.9192825	189.4815545
336.5192698	59.91075051	105.3194726	94.92440722
516.4615385	93.00473373	88.27455621	75.96234726
241.8629032	33.77419355	23.43145161	15.96474845
28.875	3.1875	3.5625	0.921875
81.18888889	8.266666667	6.166666667	2.764691358
196.1205882	34.85882353	23.52058824	14.69619377
366.6092649	47.50755287	44.49144008	36.07968772
330.166998	56.58250497	83.55666004	72.44113055
89.91749175	15.46864686	21.44224422	13.84794519
128.6226415	18.77358491	26.98921833	18.99450019
158.7724289	24.28665208	56.69584245	46.29274739
126.6372796	18.10327456	11.38790932	5.62549093
203.1403509	31.00637959	17.74162679	10.88340977
174.8993435	20.72866521	12.92122538	7.890676996
234.2154696	36.48618785	21.56629834	14.83786362
184.0224525	41.64248705	127.9723661	113.1651558
542.5773585	75.88679245	29.90566038	22.70707013
81.60606061	12.41991342	15.84848485	9.478870336
29.32835821	3.149253731	11.43283582	7.432835821
89.10091743	9.422018349	14.24311927	9.04555593
345.0780209	55.07231208	72.09705043	61.48346598
92.65614035	16.64210526	33.52631579	25.10613727
428.6083467	59.35152488	64.98956661	55.58545033
55.69346734	11.87437186	23.69346734	14.87346279
453.6920209	75.20730798	956.8553318	933.1941082
176.8	18.73023256	9.095348837	4.916652244
400.1072125	42.10331384	10.30116959	5.732575835
194.0514706	23.47058824	20.14889706	14.49953706
107.6894198	15.34812287	28.3890785	20.6709222
74.86561265	16.92885375	46.4229249	34.29711447
279.2080624	36.90117035	41.07542263	33.48249546
666.0544872	85.66773504	55.3482906	46.78516395
203.6387283	42.90462428	277.5578035	257.6187393
189.123412	30.20871143	602.1288566	577.5625311
45.55045872	6.19266055	7.587155963	3.623769043
209.8667791	26.19392917	33.91062395	27.05205475
65.30769231	8.43956044	9.582417582	4.895906292
410.4683099	52.60387324	33.65052817	25.73046565
69.5106383	10.84042553	16.28723404	10.65923495
285.9745547	37.17302799	25.14885496	18.60280902
381.8452489	53.73484163	29.83891403	22.27019103
137.0809524	20.51428571	20.24047619	13.01462018
445.226774	73.21799561	181.6503292	167.3578364
561.841133	96.89408867	192.570197	175.4681016
788.2268479	115.6627018	726.5624469	711.3544349
176.9762376	21.07524752	10.22970297	5.346040584
131.4547564	21.48259861	15.84918794	9.132552043
94.10040161	12.70281124	18.30522088	12.30370478
249.6214689	34.57062147	17.5819209	11.37422197
319.3535354	52.16565657	34.52525253	26.26107948
132.252669	11.5480427	5.914590747	2.697610213
294.3408769	28.57001414	6.352192362	2.754543872
387.7685098	49.71227741	32.83598875	25.98896256
31.60759494	4.139240506	5.405063291	2.036212145
317.8513902	60.34419942	40.83509108	30.26479962
420.1363278	69.43026005	383.9779354	365.3569082
118.5185185	16.12962963	16.87037037	11.10444292
289.1970706	32.8828229	8.021304927	3.750560726
20.29230769	4.6	16.66153846	8.735147929
24.5	3.433333333	4.45	1.3875
410.0666667	55.32682927	157.7439024	147.2999703
126.8950617	14.24691358	12.62345679	7.875361988
101.7033639	17.64831804	64.19571865	54.42770436
355.9620035	61.41278066	155.3471503	142.8724679
459.2577108	83.83207676	186.4167238	170.5807972
64.71428571	5	5.907142857	2.641377551
129.2645012	20.81438515	24.80278422	16.84279262
24	3.4375	5.609375	1.915771484
35.79699248	8.609022556	16.96992481	8.849567528
128.6611296	11.87043189	44.25581395	37.06771448

log.sigma.3.0.mm.3D_glszm_ZonePercentage	log.sigma.3.0.mm.3D_glszm_LargeAreaLowGrayLevelEmphasis	log.sigma.3.0.mm.3D_glszm_LargeAreaHighGrayLevelEmphasis	log.sigma.3.0.mm.3D_glszm_HighGrayLevelZoneEmphasis
0.419263456	0.14310663	4099.476351	336.9290541
0.266730862	0.841395967	40173.213	300.2454874
0.453917051	0.289172103	1193.192893	182.4720812
0.399240987	0.109199095	4997.094106	306.4809886
0.415041783	0.075436827	3138.993289	302.6711409
0.353032659	0.329665118	1984.484581	182.5022026
0.299703264	0.36544581	4049.772277	157.2277228
0.461077844	0.101428578	2650.242424	363.3658009
0.405483405	0.13930908	5016.442467	339.9193357
0.300193673	0.694607436	13930.95914	261.7688172
0.351874244	0.09112201	6755.051546	318.3367698
0.36977058	0.347724449	3962.193431	239.6076642
0.286403086	0.563164472	4698.479798	184.5791246
0.362254025	0.350138235	3323.432099	202.7111111
0.41053787	0.138652389	3143.831551	208.6283422
0.157190154	20.98506159	648962.7555	253.7527473
0.426934097	0.210881953	2256.385906	252.0302013
0.331046312	1.339149416	4214.212435	200.6282383
0.4604811	0.172586224	2077.757463	291.5522388
0.21813638	4.070886317	20559.22259	167.99509
0.249042879	1.977463489	56436.54189	276.2513451
0.360465116	0.363444553	3099.333871	222.6096774
0.33687294	0.505472828	5665.463043	235.4206522
0.413721414	0.050807494	7335.452261	418.5226131
0.341362126	0.592335825	5430.722628	202.810219
0.414985591	0.17600507	2841.850694	281.0590278
0.217830352	1.904575362	150867.8728	380.2874172
0.364270761	0.292128318	3841.072797	240.8448276
0.427195467	0.103381287	4487.477454	275.3580902
0.2631786	1.080285238	43541.24066	270.6860987
0.310160428	0.770320683	15733.19067	278.9462475
0.284991568	0.841497271	12273.46982	230.6579882
0.365961633	0.191776244	5474.52957	259.8481183
0.615384615	0.030762086	1468	386.1875
0.542168675	0.083006786	1651.177778	303.9611111
0.336633663	0.194116617	5225.548529	264.4985294
0.344791667	0.554732169	5531.423968	243.6102719
0.29994037	0.824469615	12289.42644	200.8230616
0.362874251	0.116733807	6115.052805	283.6831683
0.353670162	0.295043667	4804.002695	248.2479784
0.310040706	0.541207244	8701.849015	220.6980306
0.416579224	0.092357573	2870.493703	300.8790932
0.381851401	0.160662857	4520.556619	235.3460925
0.445853659	0.12076637	3258.708972	333.726477
0.385516507	0.090732701	6938.030387	327.3977901
0.259874327	5.204721991	5064.056995	106.3955095
0.372714487	0.162724639	7194.208805	299.6918239
0.396226415	1.004012931	1639.554113	173.4329004
0.5	0.044818182	5767.179104	480.0447761
0.438631791	0.102934894	5167.385321	361.9633028
0.306950935	0.546481034	11662.10561	234.4376784
0.344619105	0.911682634	3780.178947	195.4385965
0.326092646	0.332989203	15645.82745	327.570626
0.336717428	0.28688629	3474.527638	198.4522613
0.205580254	8.483391421	122644.6197	189.5242356
0.489192264	0.149161998	1820.574419	271.2162791
0.467852257	0.058245337	3706.235867	406.2163743
0.420726991	0.063114985	8867.727941	397.4283088
0.35995086	0.230064771	5068.303754	264.8703072
0.287173666	0.41841875	7004.047431	228.1541502
0.362907032	0.697826631	3949.564369	236.8660598
0.341730559	0.34848074	11922.30609	280.232906
0.22394822	1.608870471	52249.99422	244.5231214
0.201757598	2.410413693	157875.3666	307.2540835
0.502304147	0.099324191	1481.706422	246.4862385
0.381841597	0.169971379	8548.703204	315.7419899
0.461928934	0.06154099	3220.005495	353.5604396
0.355333125	0.294640555	6765.697183	321.3679577
0.421524664	0.540587961	1688.25	201.7393617
0.390850323	0.240252513	3988.301527	275.8715013
0.363486842	0.231418775	5503.127602	306.6316742
0.372010629	0.276710098	3320.32381	239.9380952
0.264512384	1.662425463	25546.35991	218.0292612
0.241810602	0.985092678	42497.43042	324.2703202
0.256427015	2.231000541	240073.0234	382.3683093
0.452508961	0.085925546	3068.706931	316.0752475
0.385854969	0.120910464	3468.691415	265.1531323
0.408196721	0.861046639	1227.096386	190.0522088
0.401360544	0.116792445	4252.360169	326.6652542
0.347856641	0.323309647	5083.431313	234.2050505
0.557539683	0.051550724	1560.298932	384.7295374
0.527218494	0.041694915	2226.398868	373.7708628
0.382163324	0.168437157	8965.645736	312.6551078
0.544827586	0.035051977	2120.227848	386.0886076
0.307578885	0.482759688	5399.040268	211.8791946
0.231738495	1.940368743	79930.52325	311.144208
0.416452442	0.367501547	1770.722222	226.3858025
0.483891753	0.044992803	3138.005326	410.114514
0.355191257	0.161274002	2854.384615	173.5692308
0.571428571	0.046656444	1261.7	253.6833333
0.309433962	0.501173305	53356.53577	403.3544715
0.458923513	0.171767748	1742.848765	303.632716
0.319960861	0.338654759	13938.58716	252.5504587
0.283129584	1.104688963	27034.46891	220.3825561
0.251291767	2.037333282	23662.25017	182.4105552
0.553359684	0.067312401	2304.964286	384.3285714
0.354440789	0.126881792	7417.276102	269.7424594
0.520325203	0.295560347	1395.484375	298.15625
0.350923483	0.279677295	2725.706767	175.0526316
0.372986369	0.20861104	11286.44518	416.5747508

log.sigma.3.0.mm.3D_glszm_SmallAreaEmphasis	log.sigma.3.0.mm.3D_glszm_LowGrayLevelZoneEmphasis	log.sigma.3.0.mm.3D_glszm_ZoneEntropy	log.sigma.3.0.mm.3D_glszm_SmallAreaLowGrayLevelEmphasis
0.678961231	0.010328281	6.135432985	0.008319
0.608072455	0.008215193	6.412734762	0.005362991
0.667025754	0.023266004	5.720112155	0.016991346
0.596137249	0.011806703	6.624444796	0.007938387
0.534075769	0.019721047	6.079480349	0.015349631
0.533168141	0.017159711	6.039445543	0.010290624
0.514691847	0.023896163	5.734605418	0.015477302
0.645995375	0.009147247	6.329856389	0.006947913
0.613578617	0.008892531	6.675344778	0.005940245
0.608342353	0.010337762	6.449010512	0.006189772
0.545390209	0.008109762	6.126330452	0.00595337
0.638944995	0.014196892	6.331655828	0.008417897
0.513148765	0.020368293	6.778049682	0.012449202
0.574926346	0.014104627	6.265909461	0.007338243
0.590311135	0.016841854	6.230800553	0.012098346
0.604423063	0.008473239	6.51965746	0.004691566
0.599749737	0.013684748	6.161807187	0.007552086
0.613274644	0.022110296	6.441014421	0.012559782
0.643577576	0.014202733	6.071356915	0.009837347
0.589197671	0.016575576	6.254783229	0.00988416
0.598025467	0.007085881	6.420150011	0.004634281
0.581882563	0.016035289	6.535110328	0.008845928
0.602925809	0.009675659	6.356591029	0.005379514
0.640211403	0.009241588	5.966970881	0.00780156
0.640299756	0.012216762	5.981921714	0.008474509
0.63570419	0.011508148	6.164115179	0.005527441
0.612344865	0.005226829	6.422626874	0.003740407
0.631623229	0.010139226	6.137354486	0.006756135
0.62396112	0.010072074	6.374906959	0.006831274
0.615822847	0.010646667	6.453392972	0.005681092
0.602833386	0.006810271	6.268209589	0.004234036
0.568850652	0.009805473	6.644394444	0.005616373
0.588310307	0.01201881	6.619732498	0.007784559
0.692491319	0.021513421	5.283214016	0.020152856
0.69765675	0.017695903	5.800958876	0.009859704
0.549927664	0.009308629	6.584357329	0.005453204
0.629443006	0.013646772	6.471482383	0.009050043
0.591371865	0.016166209	6.398206542	0.009972312
0.554412509	0.01237579	6.369446286	0.009184809
0.609805094	0.016242663	6.288978738	0.012429732
0.610619311	0.013296392	6.281656235	0.006534077
0.578328311	0.010680043	6.468927716	0.006943678
0.586572839	0.01738445	6.510926212	0.011845786
0.640298986	0.00835285	6.261051045	0.00412861
0.58192848	0.006907297	6.478676773	0.004560788
0.582446625	0.036283683	6.104276162	0.017306123
0.602057096	0.007654574	6.621518309	0.005163022
0.61422791	0.033585837	5.956597649	0.017091774
0.681671489	0.022368457	5.453857101	0.02135421
0.664051757	0.013823136	6.090367998	0.010240698
0.59327743	0.011380391	6.584345965	0.007383576
0.587667691	0.017665312	6.037389704	0.012635986
0.607000521	0.006692574	6.647837981	0.004438848
0.53485716	0.018186646	6.018457846	0.013053097
0.602194115	0.014657862	6.516352916	0.009035474
0.665913621	0.024611577	6.20596737	0.016166192
0.646091878	0.006832661	6.529745341	0.004688228
0.615839229	0.008365788	6.464482932	0.006485524
0.628964523	0.012519166	6.063864351	0.00974596
0.559247728	0.011598168	6.061869191	0.008761096
0.623615442	0.013499401	6.40639592	0.006385146
0.618799698	0.009852311	6.749019189	0.006183959
0.558813723	0.008249859	6.446199443	0.00536819
0.605966144	0.008762392	6.285675341	0.006790855
0.669086531	0.019998621	5.371139086	0.013817612
0.612134495	0.009230518	6.535228211	0.00614124
0.609482281	0.011526764	6.033030479	0.008652948
0.623367199	0.007035051	6.553143104	0.004507303
0.628369597	0.028560304	5.716336594	0.022426008
0.622773855	0.011307066	6.417368909	0.007804625
0.607372922	0.007075455	6.508038193	0.003839141
0.587982216	0.013964129	6.400005074	0.007778496
0.588364776	0.011578752	6.57200932	0.00714376
0.609732531	0.005232112	6.428848572	0.003376136
0.597992567	0.004630676	6.741733562	0.003044034
0.609837999	0.012085608	6.425356609	0.006757041
0.562475644	0.011437157	6.417309276	0.007825656
0.63791903	0.032983337	5.977752633	0.025602112
0.613760527	0.00798043	6.400337457	0.005081324
0.584711752	0.010746382	6.500412312	0.00683918
0.713216669	0.010294566	5.922890198	0.008310357
0.669475429	0.010031258	6.396675384	0.007809415
0.624482901	0.008099407	6.562533505	0.004968101
0.652267932	0.020349978	5.349551913	0.019080231
0.569514392	0.010487987	6.552114531	0.00596276
0.594327557	0.00622706	6.559397583	0.003972985
0.625623439	0.017755275	6.086218144	0.011720413
0.643297207	0.00700019	6.412552707	0.005129136
0.573759848	0.028520949	5.261076375	0.024823661
0.648583333	0.027767412	5.142318879	0.025339167
0.594487902	0.005792004	6.73901628	0.004084453
0.648753398	0.012063842	6.181378344	0.008655441
0.571115776	0.010786646	6.243815415	0.007530212
0.57054665	0.013932393	6.62299684	0.008204361
0.577472943	0.016650036	6.528766899	0.009179549
0.706617438	0.026316124	5.868843843	0.023135503
0.560781211	0.015911817	6.522308182	0.011409359
0.629741621	0.023085624	5.220714016	0.005058527
0.52733896	0.02141263	5.728818974	0.008329241
0.679202931	0.00871036	6.210699031	0.004509668

log.sigma.3.0.mm.3D_ngtdm_Coarseness	log.sigma.3.0.mm.3D_ngtdm_Complexity	log.sigma.3.0.mm.3D_ngtdm_Strength	log.sigma.3.0.mm.3D_ngtdm_Busyness	log.sigma.3.0.mm.3D_ngtdm_Contrast
0.013134636	1.433331873	4.167677132	0.167679527	0.000235666
0.005496517	0.374990788	1.644042115	0.362703051	4.44E-05
0.018670767	1.667201814	4.684368256	0.206562522	0.000415339
0.003492635	0.474048152	1.240253275	0.511634251	6.10E-05
0.028468094	2.353412467	8.335910059	0.077858644	0.00057583
0.013293357	0.671302185	2.181684171	0.330224908	0.000252207
0.026894368	0.894138915	4.23653348	0.138663926	0.000276119
0.007925142	1.622339777	3.524666121	0.215297567	0.000215343
0.004571558	0.637298607	1.73667279	0.416851595	8.65E-05
0.003106051	0.261526807	1.055617296	0.717128316	2.93E-05
0.008321612	0.650827944	1.691837519	0.322523407	0.000261145
0.00670928	0.539815071	1.96296649	0.447149127	9.58E-05
0.004644721	0.310871574	1.291933712	0.683951109	5.65E-05
0.007733108	0.582658117	1.909987032	0.400475276	0.000143168
0.009877582	0.656273509	1.902738055	0.333788331	0.000188065
0.001713773	0.110853041	1.160347243	0.801029955	3.49E-06
0.011862216	1.24633732	3.209298346	0.219971199	0.000303202
0.004224915	0.295920918	1.13549047	1.012956042	7.41E-05
0.015940176	1.586796403	4.340647701	0.170110592	0.000464283
0.003983924	0.13417005	0.727434845	1.022753812	3.13E-05
0.001892203	0.131684353	0.599193656	1.02183475	1.14E-05
0.005430712	0.50842471	1.709062873	0.520592791	8.56E-05
0.003588463	0.230141334	0.902527934	0.824511062	4.21E-05
0.018072132	1.873831725	5.541025001	0.090251011	0.000358355
0.006836429	0.542960099	1.775877459	0.449553621	8.38E-05
0.013439267	1.452581872	3.964203383	0.200098115	0.000270577
0.003102548	0.198629263	1.213834717	0.44934686	1.31E-05
0.006160216	0.462312519	1.491833945	0.537053961	0.000101558
0.00412238	0.590587029	1.347096218	0.511884887	0.000134544
0.004510926	0.383077089	1.941830031	0.393409052	2.84E-05
0.002820682	0.243369057	0.870809263	0.7646995	2.67E-05
0.001801465	0.116020843	0.564878402	1.398344886	1.47E-05
0.004475006	0.516264653	1.520975604	0.491865793	7.88E-05
0.060971558	9.539530614	17.45490496	0.037921595	0.003721263
0.021626415	2.734254079	5.548104849	0.113503657	0.000965213
0.004530779	0.41502599	1.345711545	0.502151756	7.38E-05
0.002967855	0.378847991	1.226925085	0.937948626	5.02E-05
0.002797155	0.161572771	0.584233606	1.134042727	3.55E-05
0.009609837	1.128686063	3.105854017	0.185853301	0.000157693
0.00850129	0.776823582	2.201769079	0.316418352	0.000165942
0.007182542	0.455089218	1.871231409	0.360867937	6.97E-05
0.009068787	1.106548852	3.154354953	0.233633882	0.000192645
0.004737234	0.540065103	1.374388192	0.490063108	0.00011782
0.008648084	1.0892089	2.79305451	0.259846723	0.000212089
0.004574857	0.515541529	1.41246023	0.40536301	7.20E-05
0.003702172	0.175462619	0.753720248	1.800022875	4.76E-05
0.002225728	0.234058396	0.691044662	0.899560656	3.05E-05
0.013466732	1.102736724	2.810682443	0.310514056	0.000411505
0.042144929	8.44264053	17.2809897	0.032824421	0.002209696
0.017330587	2.687279566	8.121352156	0.078557634	0.000338264
0.002992995	0.234747238	1.021379344	0.767497822	2.56E-05
0.007157766	0.719825052	1.534418816	0.513779988	0.000403048
0.002581399	0.308767247	1.048368259	0.652157885	2.77E-05
0.015579079	0.752747826	2.690407288	0.223021376	0.000239253
0.001873919	0.074015676	0.433549999	1.565776285	1.23E-05
0.009639411	1.377667637	3.059334965	0.259790833	0.000281424
0.003802429	0.799012171	1.648573433	0.393433008	9.81E-05
0.006899809	0.99924156	2.771052013	0.186430035	0.000118554
0.011976274	0.772110045	2.538830988	0.254701923	0.000198943
0.010305673	0.439080541	1.823652053	0.323152999	0.000129383
0.003948704	0.481021048	1.408185607	0.763710316	8.29E-05
0.001797258	0.298643139	1.106083969	0.869733959	1.81E-05
0.003493167	0.190591607	1.074801648	0.572969097	1.70E-05
0.003814708	0.187293311	0.900831463	0.517772224	2.62E-05
0.039722367	2.466333144	6.897949569	0.087172356	0.001148631
0.006969278	0.799794339	2.893640778	0.237233801	7.33E-05
0.018179932	2.974158457	6.524779992	0.090363537	0.000510111
0.002866956	0.355983897	1.025183156	0.774240421	5.33E-05
0.015544286	1.409312813	3.528115732	0.21954071	0.000544377
0.004539911	0.520590928	1.537311505	0.544050227	7.54E-05
0.002981595	0.311115161	0.933439684	0.832978029	5.12E-05
0.00731639	0.841810232	2.328110883	0.377506508	0.000169467
0.001940322	0.132801728	0.564788138	1.441418185	1.82E-05
0.001568287	0.109024306	0.505133363	1.129096449	9.35E-06
0.001168849	0.123098923	0.651714635	0.932582482	5.15E-06
0.007235351	1.248846776	2.72800537	0.259424118	0.000200325
0.007311547	0.96678645	2.552131565	0.2725172	0.000127763
0.012539711	1.298291821	3.380831149	0.377623511	0.000420867
0.004686103	0.597569508	1.42369407	0.479546061	0.000106607
0.003040119	0.336339825	1.023640066	0.818894943	4.43E-05
0.015292364	3.060027376	5.683173576	0.134501157	0.000583718
0.006111977	1.232100129	2.485609518	0.263757709	0.00017156
0.003699825	0.409090356	1.291776551	0.469058302	4.73E-05
0.053801573	5.45158608	14.67418659	0.034766363	0.001635949
0.002734896	0.181563358	0.636763286	1.254564324	3.88E-05
0.001874012	0.171133821	0.935732916	0.767357526	8.69E-06
0.010794002	1.017126308	2.812352783	0.366215467	0.000302854
0.004939288	0.909052785	2.062287141	0.29337734	0.000150043
0.042358145	1.425727747	4.446534359	0.156725767	0.001274212
0.053746367	6.022342592	11.39748384	0.057398649	0.003105136
0.002728885	0.335418243	1.278509671	0.451645486	2.15E-05
0.011232348	1.934811225	4.109092078	0.251969029	0.000349237
0.009624043	0.579340757	2.053517112	0.257693436	0.000113241
0.002387256	0.184787355	0.737028455	0.997434881	2.23E-05
0.001677905	0.099651149	0.422674251	1.931474178	1.65E-05
0.024986621	8.014958979	12.94022108	0.069193889	0.001855376
0.008300906	0.58241081	2.047388348	0.254474941	0.000116781
0.054351926	5.927079128	11.41196192	0.058801363	0.003314536
0.024725738	0.949342748	4.129189745	0.150565374	0.000395902
0.011377681	1.88948708	5.504436476	0.135114975	0.000177798

log.sigma.2.5.mm.3D_gldm_GrayLevelVariance	log.sigma.2.5.mm.3D_gldm_HighGrayLevelEmphasis	log.sigma.2.5.mm.3D_gldm_GrayLevelNonUniformityNormalized	log.sigma.2.5.mm.3D_gldm_DependenceEntropy
29.65155005	225.7308782	0.052708873	6.511988156
20.69402904	249.1593645	0.070909605	6.985537042
26.76809871	186.6682028	0.059153943	6.257147991
34.27558318	367.4523719	0.047041108	6.883933801
33.03455125	308.9275766	0.053374819	6.388950893
18.72621291	141.4774495	0.065021321	6.25429869
12.57501607	216.462908	0.095422166	5.856713999
39.96716746	236.8063872	0.046575512	6.611751962
35.57317882	281.5906686	0.046482137	6.869885825
20.23794272	238.1672046	0.06925666	6.739747003
20.46987918	261.1511487	0.066944083	6.302294954
25.55425384	195.8373819	0.057834819	6.746435384
20.04946116	158.6393443	0.063782292	6.574046567
22.69131243	158.2325581	0.060584484	6.452000556
22.03060773	220.9923161	0.059737011	6.401994997
11.28780502	171.3592918	0.137881464	6.823016252
29.16348798	216.2808023	0.053776242	6.459508608
25.44432006	152.5960549	0.057883235	6.906146074
37.28492814	232.8041237	0.046692883	6.561038431
13.87254845	120.2841842	0.07815985	6.892299677
14.89918156	200.0706355	0.082462055	6.774603008
25.92803002	216.6569767	0.056047188	6.559725654
19.77976931	162.7096302	0.067379817	6.64729767
28.43632246	350.045738	0.057282775	6.40557875
17.37030151	163.4709302	0.075335813	6.494739072
30.5075119	236.4755043	0.053326579	6.639958908
13.95427188	313.1364686	0.093817811	6.919830672
22.52931961	172.4508025	0.067507058	6.48141596
34.80070171	256.8866856	0.052414513	6.618305996
19.60656811	223.9323367	0.073296084	6.991589256
17.54581878	198.2406417	0.074259248	6.60198239
17.89062392	157.1266442	0.071766392	6.902987969
30.8793215	244.4589277	0.050894863	6.7934489
38.02727441	432.2788462	0.057507396	5.469294526
37.82271556	270.936747	0.047212948	6.317167092
25.10843937	285.2118812	0.055607784	6.655834485
28.57171103	223.6006944	0.057070795	6.900672556
19.2001807	203.2659511	0.063470881	6.736094931
23.20352254	278.9353293	0.062431783	6.434388588
27.04493544	219.5109628	0.054195698	6.674628396
19.95293914	166.5698779	0.064314237	6.791318378
30.26281616	221.706191	0.053475577	6.442045424
28.27472068	277.5761267	0.056282926	6.5424019
34.80932207	298.9590244	0.048324569	6.714234653
24.60676563	252.4270501	0.056029628	6.515856979
13.70848476	93.07675045	0.08049623	6.542274049
27.45219778	308.749414	0.053160447	6.833517563
25.83594056	156.3361921	0.054944408	6.317023811
35.0663845	500.2089552	0.091779906	5.497043672
34.20515852	252.7927565	0.062503795	6.286426793
19.88195058	223.3525117	0.064758999	6.814379368
29.15093818	172.8029021	0.064590038	6.269144521
25.36261384	356.7817325	0.056626485	7.018331067
18.0338295	154.0659898	0.065629107	6.321091847
16.21020552	148.8183351	0.088696234	6.954510967
39.12791322	287.14562	0.044658515	6.587351673
43.60944048	363.8937528	0.042453406	6.878997576
32.91852195	376.4717711	0.056328652	6.603549684
23.44975822	209.6683047	0.058901653	6.688762718
14.8615094	338.984109	0.073282476	6.280801549
29.06376052	217.2156678	0.055051255	6.643159337
29.51450586	246.8212851	0.053320854	7.045520031
13.04424388	179.6546926	0.085818121	6.752608149
14.22717082	504.3526181	0.092164351	6.955952222
24.44311835	181.2119816	0.059610525	5.866795634
29.66355628	273.5099807	0.057944472	6.691854186
31.4412443	272.9517766	0.056713649	6.173828142
31.49278653	333.6130748	0.050282419	6.927508066
25.48189185	216.367713	0.0601661	6.151163156
29.35038393	235.3749378	0.052943272	6.77194104
27.39560671	227.7391447	0.055796399	6.809071017
27.99443294	185.4313552	0.055316752	6.596049243
18.61933029	219.1420279	0.069740541	6.995769397
15.73910975	207.043925	0.076853175	6.871586792
18.23975293	418.1308279	0.080657534	6.988151428
38.46839953	312.7625448	0.046468121	6.587571018
26.36712354	233.0367055	0.055298235	6.623116117
29.25794141	172.4721311	0.056237571	6.306659808
29.75237523	282.7318594	0.052093135	6.66813273
25.13259737	213.4662684	0.058155125	6.725342759
43.26341254	268.9186508	0.044028723	6.335687934
46.20488344	379.01566	0.041762661	6.636992367
31.23584153	485.0082378	0.052108819	6.80122162
31.18478002	400.0896552	0.065969084	5.732408597
20.37882225	178.8743733	0.062574697	6.690501008
15.33490026	320.7545654	0.082741291	6.909183901
29.49034998	186.6413882	0.055339312	6.442048127
38.42836604	388.7596649	0.050403869	6.613576699
15.92570695	155.7595628	0.073546538	5.749470405
29.05705215	274.8	0.083718821	5.412642751
25.66060115	525.7539623	0.060704751	7.101733515
40.63402523	288.7634561	0.047877762	6.541995721
20.7003305	307.5088063	0.070628176	6.793738477
18.96793186	164.2794621	0.066809261	6.950186596
16.71361755	167.538753	0.069007687	6.871036455
69.45845116	399.0316206	0.038041525	6.311142799
24.0501151	350.9876645	0.060241192	6.550496091
35.64439157	314.2357724	0.05651398	5.560162628
18.68767274	144.9683377	0.071254029	6.222305499
33.07180203	242.4064436	0.058476865	6.717199216

log.sigma.2.5.mm.3D_gldm_DependenceNonUniformity	log.sigma.2.5.mm.3D_gldm_GrayLevelNonUniformity	log.sigma.2.5.mm.3D_gldm_SmallDependenceEmphasis
151.6855524	37.21246459	0.348012883
267.1887338	147.2792489	0.253655321
100.3870968	25.67281106	0.427118777
568.0208729	123.9533207	0.357232892
82.67688022	19.16155989	0.381866265
128.6734059	41.80870918	0.321815909
66.35311573	32.15727003	0.251452955
236.0359281	46.66866267	0.381210646
441.6993747	96.63636364	0.348313296
507.1142673	214.5571336	0.282110104
172.5018138	55.36275695	0.29212931
269.6126856	85.71120108	0.31711201
399.1465767	132.2844744	0.277609503
227.706619	67.73345259	0.313294773
209.1405049	54.42041712	0.357822376
597.3526702	957.8625306	0.156924894
154.1432665	37.53581662	0.352222297
379.3190395	134.983705	0.274785108
127.6632302	27.17525773	0.338554914
343.5947876	218.9257408	0.199263692
708.0371363	430.7817764	0.246284648
383.827907	96.40116279	0.328947631
473.5536434	184.0142805	0.303558203
106.5301455	27.55301455	0.368499788
205.5332226	90.70431894	0.315052916
133.1700288	37.00864553	0.322366604
385.694172	325.1725332	0.200507955
260.5408234	96.7376134	0.345405157
376.1240793	92.51161473	0.351219267
316.3367427	186.3186467	0.246709546
531.846178	236.0701478	0.296133589
892.2694772	425.5747049	0.247312576
405.2744712	103.4692573	0.315640543
31.94230769	5.980769231	0.467799145
82.57831325	15.6746988	0.40520164
401.3138614	112.3277228	0.286746139
486.1270833	164.3638889	0.313463093
582.1520572	212.8813357	0.280657588
178.7796407	52.13053892	0.294549388
211.8770257	56.85128694	0.347891085
228.734057	94.79918589	0.278925564
226.2088143	50.96222455	0.346808073
343.1985384	92.41656516	0.304539351
215.6185366	49.53268293	0.342903124
430.1384452	105.2236422	0.356500799
334.6651706	179.3456014	0.244490073
864.9540553	226.782466	0.335900235
130.9897084	32.03259005	0.349581805
29.86567164	12.29850746	0.354728187
99.76056338	31.06438632	0.333293286
572.8726636	221.7348131	0.282287488
163.0362757	53.41596131	0.292168527
621.565297	216.3697985	0.267975191
117.1962775	38.78680203	0.297784758
735.7919669	578.5655373	0.191254216
223.4527873	39.25483504	0.421025939
507.5517556	93.1003192	0.389957123
284.8391338	72.83294664	0.357732455
143.4889435	47.94594595	0.304867366
163.8217934	64.56186152	0.234109915
427.6833412	116.6536102	0.321918948
968.5808689	292.0916393	0.30144637
416.2323625	265.1779935	0.214270512
280.1270597	251.7008422	0.185478137
47.80184332	12.93548387	0.375969786
318.5016098	89.98776561	0.331019433
104.5482234	22.34517766	0.367820079
582.7366281	160.7528933	0.307047058
95.97757848	26.83408072	0.352463864
396.6240676	106.4689209	0.337203685
567.6493421	169.6210526	0.309547735
225.0832595	62.45261293	0.307888838
695.3618421	360.4191176	0.233569111
897.450268	516.1459202	0.223769039
1210.421133	740.4361656	0.239331449
277.5179211	51.85842294	0.384861112
223.7126231	61.76812892	0.312459817
133.1278689	34.30491803	0.333428437
370.2891156	91.89229025	0.336613381
521.7919888	165.509487	0.307919262
140.4880952	22.19047619	0.434971397
367.7964206	56.00372856	0.447269218
583.972063	145.4878223	0.350736006
33.63448276	9.565517241	0.374606263
608.3329401	212.1907992	0.274468343
708.0909423	453.0913075	0.214529401
161.5089974	43.05398458	0.369812437
363.0811856	78.22680412	0.361132552
35.75409836	13.45901639	0.283029186
29.38095238	8.79047619	0.435039683
587.4538365	241.3013836	0.261257196
162.2492918	33.80169972	0.392205965
161.6849315	72.18199609	0.29108307
626.1281174	273.2498778	0.253767236
841.556321	400.658629	0.235094325
71.65612648	9.624505929	0.496346069
252.1990132	73.25328947	0.32075757
39.29268293	6.951219512	0.480126468
74.62005277	27.00527704	0.273813317
143.5848823	47.19083024	0.347330798

log.sigma.2.5.mm.3D_gldm_DependenceNonUniformityNormalized	log.sigma.2.5.mm.3D_gldm_DependenceVariance	log.sigma.2.5.mm.3D_gldm_LargeDependenceEmphasis
0.214852057	2.21015336	9.376770538
0.128641663	10.50370324	28.36928262
0.231306675	2.304869502	8.451612903
0.215567694	2.535567262	9.69601518
0.230297716	2.424686339	8.977715877
0.200114162	2.626848777	10.73561431
0.196893518	2.261990508	11.69436202
0.235564799	1.752267919	7.868263473
0.212457612	2.365323451	9.649350649
0.163690855	4.048945966	15.06843125
0.208587441	2.118728698	10.2140266
0.181924889	3.207300926	12.34547908
0.192452544	2.465691312	11.51783992
0.203673183	2.22137986	10.16994633
0.229572453	2.30746059	9.024149286
0.085987141	17.34777691	52.3171153
0.220835625	1.850543099	8.604584527
0.16265825	4.025584823	15.25900515
0.219352629	2.142027137	9.271477663
0.122668614	8.193546932	27.35487326
0.13553544	9.936018441	26.71401225
0.22315576	1.878766901	8.919767442
0.173399357	3.563221464	13.44965214
0.221476394	2.475343727	9.37006237
0.170708657	4.975256896	15.5
0.19188765	4.382986322	13.4870317
0.111279334	13.02950865	36.39526832
0.18181495	3.684529111	12.72644801
0.213101461	2.272235232	9.450991501
0.124444037	11.72191618	31.06530291
0.167299836	4.400035385	15.16231519
0.150467028	7.506429309	21.85430017
0.199347994	2.682618739	10.94294147
0.307137574	0.922707101	5
0.248729859	1.522499637	7.042168675
0.198670228	2.196466033	10.75643564
0.168794126	4.590516493	15.08541667
0.173569486	3.529315027	13.79069767
0.214107354	2.216199935	10.23832335
0.201980005	2.966947504	10.8379409
0.155179143	4.566915638	16.51017639
0.237364968	1.893914286	8.44386149
0.209012508	1.949507523	9.729598051
0.210359548	2.398454253	9.823414634
0.229040706	1.778003473	8.272630458
0.150208784	5.148961802	18.50359066
0.202755287	2.843689975	10.7721519
0.224682176	1.883497259	8.636363636
0.222878147	2.582535086	9.880597015
0.20072548	2.246541624	9.989939638
0.167310941	6.372548571	18.20502336
0.197141809	2.421371224	11.03385732
0.162670845	6.098198217	18.36927506
0.198301654	2.400497021	10.84094755
0.112799627	9.385236861	31.05488272
0.2542125	1.757294785	7.19112628
0.231441749	1.873789858	8.015959872
0.22029322	2.212159112	9.11446249
0.176276343	6.605593755	17.62407862
0.185949822	2.584293723	13.02724177
0.201832629	2.313454685	10.2005663
0.176812864	4.602902566	14.85505659
0.13470303	7.645464124	24.4368932
0.102573072	14.1693128	40.79128524
0.220284992	2.393934889	9.221198157
0.205087965	2.431530855	10.18737927
0.265350821	1.259527429	6.807106599
0.18227608	3.280131935	12.57147326
0.215196364	2.195740916	9.434977578
0.197227284	2.716986955	10.74241671
0.186726757	3.257797351	12.31710526
0.199365155	2.343342165	10.59433127
0.13455144	7.616848953	23.84365325
0.133628688	7.192544646	23.54466945
0.131854154	7.881534832	24.43703704
0.248671972	1.55960869	7.292114695
0.200279877	2.196085723	10.22470904
0.218242408	1.990862671	9.131147541
0.209914465	2.120666029	9.48185941
0.183342231	2.971027626	12.04708363
0.278746221	1.507353867	6.392857143
0.274270261	1.423547265	6.233407905
0.209159048	2.837911327	10.42406877
0.23196195	1.71196195	7.910344828
0.179396326	2.93320837	12.77292834
0.129308061	9.003573866	26.97151205
0.207595112	2.678028826	10.03341902
0.233944063	1.740653895	8.081185567
0.195377587	2.557257607	11.49180328
0.279818594	1.36	6.2
0.147787129	8.237782113	22.69308176
0.229814861	1.992159475	8.206798867
0.158204434	14.61154407	30.8630137
0.153087559	7.134905937	20.89193154
0.144945973	4.924401794	18.79572856
0.283225796	1.49091534	5.996047431
0.207400504	2.43572271	10.28125
0.319452707	0.803225593	4.642276423
0.196886683	2.748644189	11.90765172
0.177924266	4.151511941	13.66418835

log.sigma.2.5.mm.3D_gldm_LargeDependenceLowGrayLevelEmphasis	log.sigma.2.5.mm.3D_gldm_SmallDependenceHighGrayLevelEmphasis	log.sigma.2.5.mm.3D_gldm_LargeDependenceHighGrayLevelEmphasis
0.091793955	95.17127351	1663.226629
0.141710028	75.59532928	6591.867116
0.111095468	100.3678109	1108.958525
0.042554782	136.9319336	3333.075142
0.046719727	120.2984801	2669.406685
0.196288312	52.45461436	1148.637636
0.07008744	51.09218797	2643.489614
0.127770566	98.32680172	1702.850299
0.073041527	107.4866586	2479.252044
0.095869535	81.96584022	2966.917366
0.063107863	76.13180347	2582.948005
0.125904945	73.69388138	1896.370445
0.15236224	47.50853869	1638.070395
0.180520751	55.93070149	1384.31127
0.062507668	81.64520442	1907.646542
0.376598807	33.52514083	7917.260256
0.1068764	81.44457339	1686.653295
0.28473695	54.28627703	1742.714408
0.175946904	86.22476659	1814.718213
0.386521331	30.45807075	2795.254552
0.171344753	60.51677597	4706.406394
0.084955074	81.6787918	1644.05814
0.164953869	61.72488409	1669.446357
0.042385733	130.3798619	3457.64657
0.157946346	64.27071647	1958.180233
0.108352948	96.52391856	2639.435159
0.132458265	74.69878626	10584.59059
0.143457703	77.62013615	1546.396371
0.082750781	89.36727268	2654.121813
0.168640131	67.98977452	6710.650669
0.125731923	73.16780966	2355.120164
0.244464855	48.09731456	2748.539798
0.094469193	83.36593036	2429.030497
0.026927917	189.7078098	2377.413462
0.096331371	111.0846796	1839.960843
0.063062218	83.45230934	2909.492079
0.122965507	87.42620804	2698.819444
0.113961331	64.46747934	2455.598092
0.057210584	78.48194243	2842.588024
0.108584597	88.34054335	2034.868446
0.207439995	55.42393387	2365.297151
0.101778217	85.80249734	1627.410283
0.061723687	88.25740067	2682.805116
0.072475844	114.3537021	2563.187317
0.077918623	91.17504305	2094.87114
0.459579316	29.81457944	1217.835278
0.053560947	112.8332888	2990.661275
0.245276257	63.30567255	1078.140652
0.032120901	161.5101885	5014.985075
0.199641938	88.34719411	2523.203219
0.117118156	75.65164349	3548.743283
0.206089981	50.80371329	1980.120919
0.068603293	109.4355799	5918.745616
0.169275143	52.95913404	1387.324873
0.324618117	35.59691803	4015.563084
0.057337928	130.7380788	1788.240046
0.057938584	149.6246832	2738.462836
0.039574764	126.5667002	3748.502707
0.140488808	76.79551033	3217.855037
0.051890072	83.10036385	3969.942111
0.109575481	80.90164933	1752.368098
0.1000716	85.33346805	3255.333881
0.183676389	45.57298747	3857.051456
0.085802937	96.81754851	20241.52289
0.211732955	74.75215728	1329.258065
0.065862729	106.1542125	2503.736639
0.059179006	106.443351	1790.317259
0.064071712	115.6010662	3554.744135
0.10900794	82.85418442	1670.300448
0.088470326	88.65497265	2155.810542
0.107437985	84.28131766	2246.963487
0.163876238	65.25080581	1560.786537
0.150622672	63.05988045	4618.182276
0.1521615	55.56424064	4360.820578
0.065604511	106.9616275	9751.613617
0.069723732	120.032443	2192.006272
0.091942847	79.21824122	2183.68487
0.164251942	73.15938837	1102.491803
0.064942838	102.8954879	2428.013039
0.105041291	75.82970715	2185.244202
0.078121426	135.8037904	1265.736111
0.039618115	172.5685971	2253.971663
0.027765703	176.8284492	4909.983166
0.03235376	143.6978563	3463.110345
0.141285923	56.82992521	1907.971395
0.101334484	79.20233396	7787.449963
0.161484584	83.52779689	1261.888175
0.040277693	137.1547358	3142.098582
0.111842012	41.72053845	1981.879781
0.104866028	106.6775397	1956.514286
0.050093111	143.6977261	11314.8795
0.062109812	136.9917325	1783.026912
0.117060659	94.48352059	8928.565558
0.204890913	48.44058794	3184.476039
0.18446039	48.3791657	2663.696521
0.051576641	212.2874583	2125.521739
0.038462394	107.965416	3725.120888
0.093666739	147.5457882	1562.105691
0.268393854	42.25928374	1552.741425
0.113168479	105.2911376	2660.674102

log.sigma.2.5.mm.3D_gldm_SmallDependenceLowGrayLevelEmphasis	log.sigma.2.5.mm.3D_gldm_LowGrayLevelEmphasis	log.sigma.2.5.mm.3D_gldzm_DistanceZoneVariabilityNormalized
0.005084092	0.010999462	0.914644371
0.002018538	0.0065475	0.903009267
0.006344288	0.013227696	0.91011036
0.002379388	0.005291745	0.872624303
0.006333823	0.009824502	0.839950754
0.00635193	0.017925014	0.916824197
0.005001684	0.009498306	0.620146098
0.007537902	0.018354327	0.846610646
0.00314441	0.008852804	0.947824126
0.002206619	0.006754949	0.954748699
0.003173196	0.007233817	0.848598721
0.004710018	0.011494653	0.935208015
0.004363482	0.014265502	0.926136513
0.005391028	0.018368816	0.909501752
0.005341534	0.009902466	0.867346939
0.001744349	0.008596595	0.915726975
0.007040715	0.014063511	0.732857012
0.005297316	0.018122589	0.962227238
0.00904431	0.020638355	0.81027905
0.003890221	0.016724748	0.946748401
0.002292809	0.007601529	0.914700255
0.003597156	0.009737564	0.875722804
0.004038934	0.012502728	0.931973208
0.006245783	0.009669343	0.737139551
0.003881379	0.011046129	0.930222559
0.003901635	0.010122283	0.94779877
0.001088129	0.004181475	0.908739161
0.004842227	0.012193101	0.938523558
0.004086163	0.010385069	0.930592
0.002201211	0.007534405	0.942358592
0.002531034	0.008686401	0.95820089
0.003175521	0.012807748	0.952981976
0.003127865	0.009586132	0.917240526
0.011784568	0.014012724	0.700555556
0.008028419	0.01570405	0.849847712
0.002067969	0.006072214	0.896766416
0.002804582	0.008639212	0.95791421
0.003849516	0.010573324	0.937341739
0.003912949	0.007979352	0.783085232
0.004041281	0.010540721	0.932210455
0.005694549	0.014925734	0.880251499
0.007221265	0.014737304	0.904774467
0.002673194	0.007303501	0.895393226
0.002853515	0.007589191	0.867054756
0.003212406	0.009520453	0.874282127
0.005550222	0.023651228	0.990322809
0.002337143	0.005704892	0.842956835
0.007106046	0.022095529	0.945141779
0.010747188	0.012395855	0.600475624
0.005699855	0.018831414	0.779359249
0.00218822	0.007317944	0.942228125
0.006018073	0.019873234	0.972323792
0.001270641	0.00417402	0.912326067
0.005436495	0.016074366	0.89176072
0.002795861	0.013003267	0.963569405
0.005616665	0.01023762	0.835384206
0.003046284	0.008107402	0.733650208
0.004792204	0.007971441	0.691193547
0.004313751	0.011649347	0.902221278
0.002116102	0.004846995	0.975810079
0.003071172	0.010113036	0.922388625
0.002746389	0.00808128	0.918877211
0.002208579	0.00846059	0.935967721
0.000818998	0.00262	0.961418454
0.0099611	0.021076312	0.907625601
0.003068614	0.007944402	0.894412223
0.006567333	0.011803044	0.777098765
0.001617507	0.005050529	0.940278777
0.005088712	0.011252922	0.939756772
0.00468736	0.010127972	0.91004668
0.002657544	0.008483614	0.885211319
0.005256499	0.01515617	0.919573236
0.001631828	0.006947919	0.951699857
0.00176926	0.007130282	0.95905828
0.000909927	0.00306894	0.944877334
0.003757954	0.010138143	0.811634349
0.004459168	0.010557342	0.857988166
0.005115062	0.015734836	0.92260666
0.002923523	0.007573224	0.832215239
0.00375348	0.009853495	0.874315262
0.008485401	0.014906198	0.847222222
0.005380996	0.008518508	0.848583305
0.001410413	0.003212553	0.894722983
0.010379243	0.012644065	0.68
0.002926017	0.01044597	0.957086852
0.001042145	0.004065651	0.762245482
0.005754365	0.014825344	0.937387543
0.002490175	0.005634674	0.792387543
0.009997308	0.01874862	0.755102041
0.009140693	0.026145002	0.802469136
0.001085874	0.00279603	0.893172564
0.004566249	0.008762271	0.981425781
0.002668397	0.005720462	0.909297052
0.003975096	0.01378199	0.921917253
0.002494305	0.010284177	0.946400893
0.008915483	0.013172351	0.67513536
0.002599393	0.005405904	0.978634971
0.009506757	0.025425609	0.763557891
0.007213635	0.02453919	0.872035429
0.005779895	0.014309736	0.908468935

log.sigma.2.5.mm.3D_gldzm_LowIntensityEmphasis	log.sigma.2.5.mm.3D_gldzm_LargeDistanceEmphasis	log.sigma.2.5.mm.3D_gldzm_HighIntensitySmallDistanceEmphasis
0.013390952	1.134020619	274.3264605
0.007884257	1.153321976	294.673339
0.014380372	1.141509434	224.2995283
0.006364649	1.20508326	366.4252848
0.014657641	1.263157895	307.2383041
0.019981471	1.130434783	161.4110672
0.017988467	1.764705882	187.7181373
0.020302465	1.251101322	252.4818282
0.009493995	1.08041958	303.5460373
0.007534095	1.069486405	293.2716516
0.009823054	1.247524752	252.9323432
0.014321813	1.100558659	233.3743017
0.01631399	1.115220484	170.4142959
0.018939343	1.142517815	176.5124703
0.013716758	1.214285714	223.028699
0.011163737	1.132238548	215.0901037
0.018220686	1.476351351	217.2871622
0.018170731	1.057771664	201.8008941
0.026180204	1.318367347	246.7734694
0.019211214	1.082125604	158.8768116
0.009078992	1.133928571	245.178228
0.010587985	1.199710564	240.7825615
0.013157913	1.105769231	206.1359509
0.015447249	1.466981132	323.0919811
0.012172474	1.108597285	200.9213801
0.011773365	1.08045977	296.8831418
0.005264353	1.143782383	364.0556995
0.013763207	1.095238095	221.4188713
0.011412853	1.108	249.703
0.009060034	1.089108911	272.3613861
0.008807673	1.064066852	249.5601207
0.01315949	1.07226792	195.3119859
0.010265198	1.129750983	260.3355177
0.021443791	1.55	397.575
0.018696782	1.245283019	270.7264151
0.006967819	1.163793103	285.8559626
0.00875027	1.064516129	279.2751711
0.013992367	1.097132285	230.5305273
0.011890857	1.371335505	258.9250814
0.011593909	1.105386417	246.5620609
0.020164852	1.191897655	191.5074627
0.019671209	1.15037594	242.9392231
0.008793405	1.166107383	280.1740772
0.008103458	1.214797136	326.5817422
0.00960818	1.202205882	247.8973652
0.021796046	1.01458671	128.8209076
0.006757871	1.257701422	317.5405806
0.019959118	1.084677419	179.797379
0.025373602	1.827586207	446.737069
0.018305262	1.378787879	249.8270202
0.007759768	1.089316988	263.408275
0.020081528	1.042105263	177.6394737
0.004617148	1.137844612	399.8807435
0.018235428	1.172248804	177.6244019
0.01491734	1.055679287	190.5740535
0.012605614	1.271493213	299.3099548
0.008201352	1.474609375	355.6767578
0.012175419	1.572438163	330.0834806
0.014497639	1.154639175	248.8780069
0.008031705	1.036734694	366.0540816
0.009867619	1.121323529	250.8008578
0.009114946	1.127066116	274.678719
0.010093597	1.099337748	212.486755
0.00419752	1.059033989	525.9955277
0.024601559	1.145631068	199.2621359
0.00935042	1.167763158	306.3963816
0.015932145	1.383333333	274.0361111
0.005075381	1.092429577	378.6445863
0.012953985	1.093264249	237.1398964
0.013099096	1.141614907	261.6409938
0.008321889	1.183394161	269.3106752
0.016658321	1.125925926	215.0537037
0.006899949	1.074289876	273.5579024
0.007681422	1.062724014	255.5198626
0.003739418	1.085097883	441.3921294
0.01006846	1.315789474	304.6281676
0.013609846	1.230769231	243.405649
0.015013473	1.120967742	214.8366935
0.008658214	1.277310924	294.6960784
0.011947755	1.202148438	240.2832031
0.018260129	1.25	300.1136364
0.011067165	1.247552448	366.8552448
0.003835561	1.167238422	483.2671527
0.024023162	1.6	369.2576923
0.010475323	1.065813528	208.3002742
0.004767318	1.417047184	328.0881744
0.01518943	1.097058824	224.1860294
0.006597967	1.352941176	367.6301471
0.032944504	1.428571429	140.2063492
0.02796295	1.333333333	241.0787037
0.003920321	1.169858451	534.8345129
0.010703544	1.028125	355.2078125
0.008451415	1.142857143	318.6064426
0.01659653	1.122093023	191.5473422
0.010631888	1.082677165	209.9675197
0.016915002	1.612244898	383.9863946
0.007674592	1.032397408	341.3650108
0.024647938	1.410958904	303.0171233
0.030331018	1.20610687	152.139313
0.018974291	1.144230769	304.380609

log.sigma.2.5.mm.3D_gldzm_LowIntensityLargeDistanceEmphasis	log.sigma.2.5.mm.3D_gldzm_HighIntensityEmphasis	log.sigma.2.5.mm.3D_gldzm_DistanceZoneVariability	log.sigma.2.5.mm.3D_gldzm_ZonePercentage
0.016147006	277.0996564	266.161512	0.412181303
0.009823176	301.1499148	530.0664395	0.282619162
0.031014632	230.3349057	192.9433962	0.488479263
0.008501859	382.0464505	995.6643295	0.433017078
0.036924733	315.3918129	143.6315789	0.47632312
0.037574582	163.972332	231.9565217	0.393468118
0.059992767	203.4019608	63.25490196	0.302670623
0.040545537	259.4647577	384.3612335	0.453093812
0.009967524	308.4918415	813.2331002	0.412698413
0.009227905	296.12286	948.0654582	0.320529374
0.022880744	262.4224422	257.1254125	0.366384522
0.016445742	237.8435754	502.2067039	0.362348178
0.018568697	173.2916074	651.0739687	0.338958534
0.029795514	179.3182898	382.9002375	0.376565295
0.02719342	229.7806122	340	0.430296378
0.013964311	221.3422645	1059.496111	0.166546711
0.043715524	229.9358108	216.9256757	0.424068768
0.028674634	202.4662999	699.5392022	0.311749571
0.04965225	255.2653061	198.5183673	0.420962199
0.022317509	160.9855072	587.9307568	0.221706533
0.010712132	251.6325549	1331.803571	0.278713629
0.020085125	247.3198263	605.1244573	0.401744186
0.020003041	208.4925214	872.3269231	0.3427316
0.037016961	353.5660377	156.2735849	0.440748441
0.020706143	204.9208145	411.158371	0.367109635
0.013815093	298.2337165	247.3754789	0.376080692
0.005717297	379.3782383	701.5466321	0.222735141
0.015629551	224.7654321	532.1428571	0.395673412
0.013806479	254.168	697.944	0.424929178
0.010102768	276.950495	666.2475248	0.278127459
0.0094831	252.1652739	1031.982358	0.338785782
0.018393112	198.6010576	1621.975323	0.287015177
0.011818543	266.9960682	699.8545216	0.375307427
0.079339895	410.25	42.03333333	0.576923077
0.023031899	275.9622642	135.1257862	0.478915663
0.007780255	294.8505747	624.1494253	0.344554455
0.010028875	282.1974585	979.9462366	0.355208333
0.015401459	235.2719704	1013.26642	0.322301729
0.022421008	272.4299674	240.4071661	0.367664671
0.013292786	250.440281	398.0538642	0.407054337
0.030332143	198.108742	412.8379531	0.318181818
0.035610783	245.7192982	361.0050125	0.418677859
0.017638625	285.488255	533.6543624	0.362971985
0.019022032	333.7899761	363.2959427	0.408780488
0.012672969	256.7107843	713.4142157	0.434504792
0.023011605	128.8865478	611.0291734	0.276929982
0.009029125	336.1563981	1422.911137	0.395686826
0.021703704	181.0766129	234.3951613	0.425385935
0.09731658	475.7931034	34.82758621	0.432835821
0.025470391	260.8232323	154.3131313	0.398390342
0.009952058	266.290718	1076.024518	0.333528037
0.02067135	178.4631579	277.1122807	0.344619105
0.005327956	410.2898914	1092.054302	0.313268778
0.045133232	179.2535885	186.3779904	0.353637902
0.016451099	192.2093541	1297.927988	0.206500077
0.026565077	310.1289593	369.239819	0.502844141
0.019128416	380.9833984	751.2578125	0.466940264
0.036148821	355.6060071	391.2155477	0.437741686
0.019809385	252.2852234	262.5463918	0.357493857
0.008331025	367.355102	239.0734694	0.278093076
0.012750086	255.1188725	752.6691176	0.385087305
0.011921142	283.018595	1778.946281	0.353413655
0.011185799	216.3072848	706.6556291	0.24433657
0.004389899	531.1019678	537.4329159	0.204686928
0.057107379	200.4563107	93.48543689	0.474654378
0.016794768	317.2121711	543.8026316	0.391500322
0.030567899	285.2111111	139.8777778	0.456852792
0.005620259	384.1355634	1068.15669	0.355333125
0.014981205	239.6735751	181.373057	0.432735426
0.024427454	265.5130435	732.5875776	0.400298359
0.012700759	275.6760949	970.1916058	0.360526316
0.0191844	218.2518519	372.4271605	0.358724535
0.007442077	277.1631464	1306.683904	0.265673375
0.008500926	258.3178017	1605.46356	0.249255509
0.004041013	451.4846185	2365.027966	0.272657952
0.021600787	318.0448343	416.3684211	0.459677419
0.025697397	253.0745192	356.9230769	0.372426141
0.030868255	216.9233871	228.8064516	0.406557377
0.016332242	305.7464986	594.2016807	0.404761905
0.019917748	247.7832031	895.2988281	0.359803233
0.027338033	306.9545455	223.6666667	0.523809524
0.013116206	387.2951049	606.7370629	0.533184191
0.004408016	503.7590051	1043.246998	0.417621777
0.08993234	382.0769231	44.2	0.448275862
0.011855469	210.547532	1047.053016	0.322618697
0.006481693	376.1430746	1001.590563	0.239956172
0.016774189	228.1588235	318.7117647	0.437017995
0.014671255	384.0838235	538.8235294	0.43814433
0.059026538	146.3968254	47.57142857	0.344262295
0.037582817	246.7592593	43.33333333	0.514285714
0.004364183	555.3755204	1072.70025	0.302138365
0.011741961	355.5875	314.05625	0.45325779
0.010552372	326.2408964	324.6190476	0.349315068
0.021702913	195.9526578	1109.988372	0.294376528
0.012072565	213.1496063	1442.314961	0.262487082
0.031234671	413.2108844	99.24489796	0.581027668
0.007826006	343.1533477	453.1079914	0.380756579
0.088337023	306.9726027	55.73972603	0.593495935
0.041365422	155.0763359	114.2366412	0.345646438
0.022211674	306.7532051	283.4423077	0.3866171

log.sigma.2.5.mm.3D_gldzm_IntensityVariabilityNormalized	log.sigma.2.5.mm.3D_gldzm_LowIntensitySmallDistanceEmphasis	log.sigma.2.5.mm.3D_gldzm_IntensityVariability
0.047554941	0.012701938	13.83848797
0.04938053	0.007399528	28.98637138
0.052732289	0.010221807	11.17924528
0.043414874	0.005830346	49.5363716
0.04360316	0.009090868	7.456140351
0.063319221	0.015583193	16.01976285
0.063052672	0.007487392	6.431372549
0.043548293	0.015241697	19.77092511
0.044090719	0.009375613	37.82983683
0.052026826	0.007110643	51.66263847
0.061224934	0.006558631	18.55115512
0.048365116	0.013790831	25.97206704
0.057249059	0.015750314	40.24608819
0.056042338	0.0162253	23.59382423
0.051098501	0.010347592	20.03061224
0.059124535	0.010463593	68.40708729
0.051976808	0.011846976	15.38513514
0.051968766	0.015544755	37.78129298
0.045897543	0.020312193	11.24489796
0.06164023	0.01843464	38.27858293
0.056245094	0.008670707	81.89285714
0.05158111	0.0082137	35.64254703
0.056482395	0.011446631	52.86752137
0.050195799	0.010054822	10.64150943
0.057953359	0.010039057	25.61538462
0.047459667	0.011262932	12.38697318
0.054333673	0.005151117	41.94559585
0.050897542	0.01329662	28.85890653
0.048359111	0.010814447	36.26933333
0.048600677	0.008799351	34.36067893
0.059185519	0.008638817	63.74280409
0.058167553	0.011851085	99.00117509
0.04667545	0.009876862	35.61336828
0.050555556	0.006969765	3.033333333
0.045923816	0.017613003	7.301886792
0.054292179	0.006764711	37.78735632
0.046507072	0.008430619	47.57673509
0.054532883	0.013640095	58.95004625
0.052615943	0.009258319	16.15309446
0.049004821	0.011169189	20.92505855
0.051777361	0.017623029	24.28358209
0.050144157	0.015686316	20.0075188
0.050966173	0.0065821	30.37583893
0.046325779	0.005373815	19.41050119
0.051668829	0.008841983	42.16176471
0.069571225	0.021492156	42.92544571
0.046015504	0.006190057	77.67417062
0.055183403	0.019522972	13.68548387
0.049346017	0.007387858	2.862068966
0.049280686	0.016513979	9.757575758
0.053770845	0.007211695	61.40630473
0.059526008	0.019934072	16.96491228
0.048618483	0.004439445	58.19632414
0.064490282	0.011510976	13.4784689
0.056019894	0.0145339	75.45879733
0.04182961	0.009115748	18.48868778
0.04136467	0.005469586	42.35742188
0.045824021	0.006182069	25.93639576
0.049704184	0.013169702	14.46391753
0.073752603	0.007956875	18.06938776
0.050167003	0.009147003	40.93627451
0.045118375	0.008413397	87.34917355
0.060942941	0.009820546	46.01192053
0.057811515	0.004149426	32.31663685
0.058346687	0.016475104	6.009708738
0.044034583	0.007489333	26.77302632
0.048580247	0.012273207	8.744444444
0.048113284	0.004939162	54.65669014
0.060431153	0.01244718	11.66321244
0.046666409	0.010267006	37.56645963
0.049608064	0.007227171	54.37043796
0.052607834	0.016026801	21.30617284
0.054207961	0.006764416	74.42753095
0.058508156	0.007476546	97.94265233
0.049483249	0.003664019	123.8565721
0.043914747	0.007185378	22.52826511
0.052283654	0.010587959	21.75
0.053167274	0.011049778	13.18548387
0.049670849	0.006739707	35.46498599
0.051025391	0.009955257	52.25
0.042412764	0.015990653	11.1969697
0.039589222	0.010554905	28.30629371
0.044027609	0.003692447	51.33619211
0.050887574	0.007545867	3.307692308
0.058621231	0.010130286	64.13162706
0.05574205	0.004339803	73.24505327
0.054567474	0.014793241	18.55294118
0.045432526	0.004579646	30.89411765
0.068783069	0.026423996	4.333333333
0.060356653	0.025557983	3.259259259
0.045777145	0.003809356	54.97835137
0.041757813	0.01044394	13.3625
0.04991016	0.007926176	17.81792717
0.053495546	0.015319934	64.40863787
0.057703171	0.010271718	87.93963255
0.035587024	0.013335085	5.231292517
0.048248581	0.007636738	22.33909287
0.054231563	0.008725666	3.95890411
0.06310821	0.027572417	8.267175573
0.040495562	0.018164946	12.63461538

log.sigma.2.5.mm.3D_gldzm_HighIntensityLargeDistanceEmphasis	log.sigma.2.5.mm.3D_gldzm_SmallDistanceEmphasis	log.sigma.2.5.mm.3D_glcm_SumVariance	log.sigma.2.5.mm.3D_glcm_Homogeneity1
288.1924399	0.966494845	558.3014699	0.306946056
327.0562181	0.961669506	679.2994753	0.394977237
254.4764151	0.964622642	448.5081487	0.302973205
444.5311131	0.948729185	1039.51884	0.286515866
348.005848	0.934210526	812.0397276	0.295590015
174.2173913	0.967391304	319.4717431	0.329169741
266.1372549	0.808823529	565.2684194	0.363035545
287.3964758	0.93722467	580.474986	0.273390882
328.2750583	0.979895105	749.2319819	0.294321498
307.5276939	0.982628399	650.6948234	0.354677317
300.3828383	0.938118812	684.5566011	0.322000788
255.7206704	0.974860335	497.5689171	0.336011204
184.8008535	0.971194879	388.3310974	0.344041863
190.5415677	0.964370546	377.9496465	0.327740796
256.7882653	0.946428571	589.0289744	0.307056025
246.3509075	0.966940363	470.4725479	0.509503985
280.5304054	0.880912162	525.6489478	0.298196887
205.127923	0.985557084	354.0830189	0.355151779
289.2326531	0.920408163	563.4924339	0.290979661
169.4202899	0.979468599	275.5069747	0.433101265
277.4498626	0.966517857	534.7118539	0.402625005
273.4688857	0.950072359	554.0566597	0.306858923
217.9188034	0.973557692	389.8904263	0.356175845
475.4622642	0.883254717	1005.16314	0.291060578
220.918552	0.972850679	410.1390992	0.363041873
303.6360153	0.979885057	589.0067626	0.32040236
440.6683938	0.964054404	936.1548555	0.42661783
238.1516755	0.976190476	417.4773483	0.347493726
272.028	0.973	691.9708159	0.305143054
295.3069307	0.977722772	599.9417551	0.405039894
262.5858867	0.983983287	512.55407	0.36473745
211.7573443	0.98193302	390.2756975	0.393881599
293.63827	0.967562254	657.1976772	0.307426892
460.95	0.8625	1138.852606	0.253244945
296.9056604	0.938679245	675.3357578	0.265063891
330.829023	0.959051724	797.6082341	0.323976129
293.886608	0.983870968	582.7418278	0.333357527
254.2377428	0.975716929	538.1748029	0.352175304
326.4495114	0.907166124	763.6376088	0.309892355
265.9531616	0.973653396	549.8058361	0.318156843
224.5138593	0.952025586	411.0945169	0.370313364
256.839599	0.962406015	544.9582357	0.302961476
306.7449664	0.958473154	750.3288936	0.30415633
362.6229117	0.946300716	782.7888703	0.296471152
291.9644608	0.949448529	683.2572612	0.298587097
129.1491086	0.996353323	191.5112859	0.400157429
410.6196682	0.935574645	872.3787563	0.30213926
186.1935484	0.978830645	341.5198796	0.304203088
592.0172414	0.793103448	1342.722008	0.285205813
304.8080808	0.90530303	639.5280193	0.299862633
277.8204904	0.977670753	596.1622954	0.35945848
181.7578947	0.989473684	390.650772	0.324332208
451.9264829	0.965538847	1009.689508	0.347585606
185.7703349	0.956937799	354.7578262	0.341026556
198.7505568	0.986080178	368.491175	0.4525934
353.4049774	0.932126697	761.1546284	0.260967284
482.2099609	0.881347656	949.6565533	0.259081245
457.6961131	0.856890459	1035.405135	0.284318539
265.9140893	0.961340206	517.6420132	0.348841912
372.5591837	0.990816327	987.2449355	0.368074471
272.3909314	0.969669118	543.0472028	0.314664569
316.3780992	0.968233471	671.5101293	0.332800273
231.589404	0.975165563	472.816177	0.420131773
551.5277281	0.985241503	1563.686767	0.449726219
205.2330097	0.963592233	399.0934257	0.310786764
360.4753289	0.958059211	745.432522	0.305064038
329.9111111	0.904166667	675.5327767	0.278841956
406.0994718	0.976892606	913.6336733	0.317540525
249.8082902	0.976683938	514.6385634	0.311835284
281.0012422	0.964596273	624.7882848	0.311335155
301.1377737	0.95415146	594.5204139	0.33089207
231.0444444	0.968518519	429.8287035	0.320367886
291.5841224	0.981427531	586.1501701	0.396763716
269.5095579	0.984318996	551.2955288	0.410077216
491.8545745	0.978725529	1287.283482	0.392720567
371.711501	0.921052632	836.8510555	0.264832426
291.75	0.942307692	589.554615	0.310294061
225.2701613	0.969758065	388.7076537	0.305231319
349.9481793	0.930672269	757.2985138	0.297166971
277.7832031	0.949462891	567.9075224	0.325716491
334.3181818	0.9375	646.5386729	0.256244069
469.0545455	0.938111888	1025.922925	0.245731055
585.7264151	0.958190395	1455.166125	0.303250752
433.3538462	0.85	1074.445253	0.285702586
219.5365631	0.983546618	446.963756	0.353308465
571.0144597	0.896583798	956.1683954	0.397069539
244.05	0.975735294	429.0481171	0.311734253
449.8985294	0.911764706	1045.276106	0.271014556
171.1587302	0.892857143	370.1620463	0.351800957
269.4814815	0.916666667	684.3204365	0.275493251
637.5395504	0.957535387	1593.207518	0.366247249
357.10625	0.99296875	718.1015569	0.274148119
356.7787115	0.964285714	888.8946461	0.37102402
213.5739203	0.969476744	408.8101746	0.378430873
225.8779528	0.979330709	419.5024258	0.388678507
530.1088435	0.846938776	1041.31361	0.233547025
350.3066955	0.991900648	1049.978586	0.317351458
322.7945205	0.897260274	786.3144389	0.251818341
166.8244275	0.948473282	334.6967436	0.343111046
316.2435897	0.963942308	616.5404874	0.327524612

log.sigma.2.5.mm.3D_glcm_Homogeneity2	log.sigma.2.5.mm.3D_glcm_ClusterShade	log.sigma.2.5.mm.3D_glcm_MaximumProbability	log.sigma.2.5.mm.3D_glcm_Idmn	log.sigma.2.5.mm.3D_glcm_SumVariance2
0.21886634	262.96488	0.013770403	0.961448303	82.91404847
0.323006831	152.2872092	0.049294526	0.976605233	57.73568638
0.214687799	372.8575767	0.016286842	0.967367544	71.45604995
0.19964547	17.82933567	0.009903556	0.967757749	89.18423929
0.203717734	89.64783237	0.018541227	0.96863166	98.91427164
0.239084763	77.35230667	0.017255323	0.95612405	50.70865769
0.276847177	-58.62800798	0.033124771	0.97049487	35.17486978
0.185594695	129.4558079	0.011133862	0.953464951	113.5521635
0.206171197	168.5243634	0.008738111	0.966973476	96.58753944
0.271840904	191.8897477	0.019117472	0.980024834	53.12556224
0.233310371	-107.7486268	0.014520716	0.960028705	55.60146917
0.250797478	284.9914147	0.01513507	0.966109733	69.93562992
0.255615058	204.3987465	0.013092635	0.972435195	57.83495855
0.238325541	75.42895947	0.013077547	0.966240128	65.62575396
0.215180651	32.27423251	0.014335687	0.960369918	61.88737719
0.454695567	126.6198799	0.138255452	0.98778345	35.4869914
0.208095925	24.8320526	0.012188354	0.959949659	83.50198832
0.272478697	379.2649405	0.015098324	0.969454545	73.19837292
0.2015468	106.5060602	0.011735774	0.955591922	110.6328435
0.363085602	94.43425315	0.044751882	0.972901278	39.25871897
0.326426748	128.7669741	0.044160839	0.978717839	38.78442932
0.215549931	305.4829935	0.010653273	0.971749036	74.23876898
0.271476222	244.1251494	0.020838064	0.969092923	56.25572004
0.199658336	-438.591336	0.015709046	0.958326965	82.96806241
0.280462269	179.0036202	0.024968453	0.977808571	43.42748518
0.236494423	323.061867	0.018416082	0.964162661	78.86962618
0.35598577	143.1821203	0.06010519	0.985955676	36.47538132
0.263813145	328.8650175	0.023708273	0.96989369	58.69530229
0.219677623	-213.5197732	0.012955496	0.958742728	98.96739512
0.333576108	136.9358079	0.053777225	0.983547512	51.7408034
0.282345191	210.9654104	0.022308804	0.974810236	47.18326724
0.315249518	192.4199926	0.037090793	0.975928193	53.30528895
0.221353505	31.18895583	0.010608635	0.97020666	83.01898252
0.166245237	-185.6400283	0.030604791	0.946925192	110.262621
0.172663193	-201.2483637	0.013256121	0.94016999	106.0609752
0.235663714	-43.50469163	0.012529427	0.972325532	69.82000401
0.251697399	355.233435	0.016876617	0.975541669	75.42376267
0.26730871	50.76495276	0.014702907	0.970663251	52.05190962
0.221087212	-164.8134043	0.015391235	0.966985945	67.00605264
0.231448651	55.59770717	0.013083967	0.964145825	73.66270791
0.289669548	96.15714851	0.019800289	0.968292969	53.87468055
0.213515885	156.2944422	0.011971859	0.963096617	88.07024144
0.216703363	-90.21923685	0.014828057	0.963433665	79.46311013
0.209430791	136.8333241	0.012021201	0.962212502	95.7467777
0.207113315	-47.93694721	0.010225289	0.964640673	66.50810565
0.322148582	128.0098163	0.026582536	0.971282134	34.06385423
0.213255793	74.23559364	0.010189951	0.971651592	69.95966444
0.213954321	106.159554	0.01224238	0.957191809	69.61072844
0.204310833	-787.4532891	0.042012112	0.945802205	101.6870402
0.213542077	-262.5873066	0.020679971	0.964480327	103.6519955
0.276228223	161.5101828	0.028519868	0.976068638	56.30627418
0.241028488	16.68740541	0.017882578	0.943780562	78.75971174
0.267446194	219.8030684	0.021826453	0.978855296	65.9409655
0.251460017	20.87126092	0.016944194	0.959754652	51.96017495
0.389732773	150.4553218	0.074549699	0.976237801	51.22215267
0.171962092	67.16625709	0.009682311	0.951310907	106.5455331
0.171463932	38.96609679	0.006929236	0.963713585	112.8694788
0.195679401	-465.9178305	0.013444421	0.963059667	89.63679094
0.26492362	107.5207043	0.032334478	0.965832934	64.8709926
0.280445953	8.671017274	0.019351943	0.981438771	41.0242534
0.227320472	339.6968824	0.013799398	0.967580789	79.38805303
0.250424362	369.5780731	0.017275925	0.97698967	82.49389465
0.347080827	84.41015483	0.040714176	0.981232562	38.06394414
0.385278868	58.44222032	0.064504717	0.985409684	36.95149262
0.218359303	-37.35559802	0.022613441	0.950363305	69.6745256
0.219135924	286.431189	0.018637548	0.97404711	81.73990575
0.188822177	-240.7333445	0.013763494	0.955169998	85.22092799
0.233005852	227.8957779	0.01106901	0.97173774	82.90195708
0.22285393	35.85089505	0.017539763	0.956301833	70.44888326
0.227061275	235.790327	0.012236434	0.966463813	81.19258999
0.24655433	271.4095385	0.012854616	0.964321337	78.21333015
0.232812205	226.3973022	0.013596992	0.963521471	78.67983302
0.322062368	220.3260953	0.030065682	0.975641699	53.05983834
0.336614157	153.311179	0.035826959	0.977453396	44.61616848
0.318058284	214.3254176	0.035148157	0.985345087	50.41902109
0.175547122	-123.8003966	0.010492066	0.956642715	102.1470075
0.222306461	52.4082954	0.011316683	0.962282204	72.24112422
0.215856052	419.2432519	0.017784213	0.953976792	78.72973434
0.208688341	100.4102053	0.010502295	0.960929654	80.61502102
0.241405627	164.8693702	0.014930574	0.971438349	69.70383155
0.166592815	213.1460001	0.012109753	0.951841192	107.9779733
0.156341155	-38.81248142	0.007292979	0.955767505	118.2471909
0.21892528	83.77223662	0.013350035	0.973132529	90.07251726
0.197557439	-555.9386459	0.029117639	0.951314122	96.50375172
0.268575798	146.6397243	0.015315505	0.970291738	55.85968222
0.320304932	200.8487275	0.041797707	0.988157462	42.39823631
0.222195231	353.0748963	0.017989379	0.96078397	80.19036567
0.18260506	-286.0544087	0.010715309	0.957029448	106.749988
0.266056253	-29.73564347	0.031479473	0.952112979	41.06833414
0.188406631	-330.1994059	0.035766156	0.924619768	79.68865931
0.289967114	130.8736657	0.035262491	0.979718128	73.87545122
0.185583597	549.4441256	0.011332932	0.964220394	93.85576154
0.292763555	9.68874078	0.069434147	0.969512954	54.83509874
0.299604311	126.2646018	0.026412664	0.97424636	51.4846998
0.30961591	103.324964	0.019542389	0.977118258	45.34810408
0.148815085	499.4275938	0.013970041	0.944489198	181.1856471
0.22750524	-129.4123792	0.017416818	0.971308512	61.36275819
0.161359596	-402.7651329	0.021207528	0.940916076	108.476435
0.251580594	12.35449471	0.023750912	0.965369375	58.59401158
0.24598646	472.3439126	0.021138545	0.968842083	78.35894199

log.sigma.2.5.mm.3D_glcm_Contrast	log.sigma.2.5.mm.3D_glcm_DifferenceEntropy	log.sigma.2.5.mm.3D_glcm_InverseVariance	log.sigma.2.5.mm.3D_glcm_Entropy	log.sigma.2.5.mm.3D_glcm_Dissimilarity
36.88742904	3.640364387	0.223665149	8.200789846	4.618904877
28.24105692	3.396853514	0.293589994	7.817019354	3.663989053
37.25843635	3.606639934	0.228088296	7.736387787	4.647755703
49.09229097	3.859104755	0.203814507	8.821831783	5.324600997
35.59207859	3.583066478	0.207261606	7.85221963	4.631854537
24.29440095	3.368617753	0.243828369	7.715560863	3.840774152
18.91777669	3.150488823	0.284779115	6.967277152	3.288683724
51.40354664	3.883407317	0.189826653	8.656390369	5.564648661
42.49902505	3.770319006	0.206801051	8.734148786	4.98789845
26.52534466	3.434414481	0.272593618	7.966274718	3.767021912
28.16558736	3.461892506	0.234845266	7.810551244	4.119490525
30.20730057	3.502562912	0.257560748	8.147659583	4.065229304
22.14573143	3.314709862	0.262470776	7.944733569	3.598628754
25.39922264	3.402446903	0.247102407	7.960832483	3.899884021
30.49646362	3.530950036	0.22472358	8.119594583	4.285959859
15.48444981	2.898846803	0.34731563	6.76872553	2.396551197
35.53431431	3.594094697	0.215723627	8.131738684	4.631091407
26.94057149	3.433665524	0.265328939	8.11115279	3.810207274
43.15929793	3.736303091	0.19528692	8.294594033	5.04593648
16.18364105	3.073804201	0.326949673	7.381650802	2.811265124
19.62975757	3.24068315	0.29835352	7.553802604	3.158074072
31.85939786	3.564026174	0.223465192	8.316998059	4.352044144
23.44430694	3.358441443	0.268581754	7.936101064	3.611193799
40.25925746	3.701496692	0.196537862	7.999388881	4.886600825
25.12703714	3.372212756	0.278111515	7.567887874	3.620244964
42.19019929	3.706092162	0.23048724	8.101147109	4.742233046
18.43389442	3.16808025	0.31595039	7.327774471	2.971016314
28.62557458	3.471102534	0.267417962	7.854474589	3.903355049
42.65404972	3.723090031	0.221697306	8.409830591	4.910318402
25.7323934	3.349728818	0.299728364	7.727686515	3.467605745
23.40347922	3.358202606	0.2789888	7.778076481	3.553082941
19.28499461	3.229708173	0.287843915	7.812461853	3.201357855
40.37195279	3.719386834	0.225390784	8.509066545	4.781559915
63.73026958	3.80571093	0.179502858	6.628696247	6.210641933
52.71253529	3.835580205	0.164086665	7.987466471	5.641744553
29.22886801	3.505285544	0.244233543	8.20068181	4.11119669
38.66839896	3.671596433	0.241429559	8.315218464	4.535780458
23.76224149	3.383116486	0.26790367	8.026215401	3.657949727
32.79837894	3.47693546	0.224744438	8.023213823	4.434611759
33.98156972	3.597854606	0.236003015	8.238782361	4.409687914
24.57327653	3.353551239	0.288572146	7.882703876	3.55496616
35.08156959	3.623713697	0.225946174	8.30596952	4.546038115
36.84742905	3.634521875	0.220888163	8.308219132	4.675073795
44.18468166	3.768816624	0.212088705	8.4919057	5.021317031
33.17503148	3.597792785	0.217729992	8.350638896	4.498933527
16.97052777	3.122266952	0.308615415	7.23406813	3.00581127
38.33615096	3.706347237	0.218206426	8.5822161	4.704386728
32.95735821	3.568207371	0.220523694	7.979826975	4.466598226
61.98750919	3.829367646	0.195880868	6.645829746	5.943684866
43.48864358	3.743619944	0.210047858	8.013655792	5.005718763
25.16941603	3.415461241	0.261548659	8.042125099	3.706647337
35.69455393	3.583594414	0.242815906	7.827806836	4.484257193
33.04052412	3.560967102	0.258698669	8.271852922	4.187905551
22.2408315	3.317407979	0.256638199	7.697822134	3.626982694
21.16529399	3.152183444	0.32648458	7.496729289	2.9964958
57.85988107	3.95601176	0.177959805	8.675665744	5.898029118
61.76416939	3.963762616	0.173517313	8.975691961	6.087101275
48.66402819	3.816009186	0.201192699	8.484569224	5.276430022
28.30130973	3.482670495	0.251547635	7.98454531	3.933726058
16.75358575	3.118193087	0.283666226	7.395502875	3.125852534
34.89195861	3.627618166	0.233943489	8.318421737	4.456759677
38.75047296	3.675802603	0.243329619	8.547288782	4.489464013
17.09955925	3.122663853	0.313208912	7.41325818	2.934596515
19.22360118	3.112782808	0.326822135	7.163449284	2.915577473
30.86836391	3.479160995	0.216906667	7.341088705	4.285610887
43.55535733	3.748306024	0.223750297	8.425934845	4.901217822
46.83278975	3.765603381	0.199980576	7.965391059	5.24710039
38.03527028	3.681045329	0.228873936	8.498257668	4.638035812
31.32846838	3.525443084	0.231294009	7.752100236	4.32822924
41.0134293	3.723959683	0.230060492	8.47485156	4.793968295
34.36003232	3.578555698	0.247196824	8.341737094	4.315288091
32.34358388	3.567149243	0.233527157	8.186116444	4.314495575
24.40140554	3.347691865	0.28832007	7.853469742	3.458856605
19.5613407	3.205951545	0.314099421	7.62536005	3.103403724
25.1822302	3.364220107	0.298217528	7.85057189	3.481014461
53.90511028	3.886772077	0.183565295	8.650737587	5.705080126
35.91505253	3.631776022	0.221338876	8.262005669	4.566746251
36.29338315	3.634800042	0.218517759	7.927101949	4.598488031
39.75243967	3.680423752	0.214141376	8.444940535	4.840110529
34.30346929	3.610718026	0.243211428	8.300294389	4.38505932
56.74005059	3.898045405	0.177020984	8.300851297	5.903428756
65.48304257	4.059691543	0.1596234	8.941210692	6.338564249
47.29829076	3.817062927	0.219291495	8.705509766	5.110373192
47.79815159	3.674636053	0.191815656	6.927277692	5.304143025
24.16225581	3.374379207	0.266518428	7.981810142	3.670024756
21.09146163	3.269848011	0.297469652	7.622515392	3.244351544
32.45398224	3.574478788	0.22373031	8.034731251	4.381780824
49.82001505	3.848460665	0.187099171	8.652919033	5.530844796
22.25428487	3.131167438	0.263609422	6.806972652	3.605609787
49.60741294	3.659502826	0.191881062	6.4060902	5.501425012
33.63307678	3.551245288	0.267761634	8.276608236	4.093656293
55.49969081	3.925350468	0.186242332	8.296037883	5.649511993
30.9847952	3.520881406	0.249205215	7.806680201	3.978466825
24.20283349	3.363013809	0.284359852	7.931056933	3.524886883
19.7099252	3.239849441	0.298186247	7.761395746	3.217998708
89.56339855	4.113030269	0.154706153	7.924378557	7.333716583
30.59435617	3.529460976	0.237475314	8.102353441	4.199801668
54.85877198	3.72965115	0.173081877	6.944225078	5.871111796
20.42018528	3.244738545	0.245138585	7.440841597	3.528279566
46.35989342	3.731473239	0.245218439	8.02004829	4.800884124

log.sigma.2.5.mm.3D_glcm_DifferenceVariance	log.sigma.2.5.mm.3D_glcm_Idn	log.sigma.2.5.mm.3D_glcm_Idm	log.sigma.2.5.mm.3D_glcm_Correlation	log.sigma.2.5.mm.3D_glcm_Autocorrelation
14.78808234	0.873455772	0.21886634	0.384076104	192.7269529
14.21857088	0.908907631	0.323006831	0.348112453	229.4117572
15.03828257	0.882522846	0.214687799	0.316881338	156.6765054
19.8570858	0.883521046	0.19964547	0.297525517	335.4057303
13.19160231	0.882033398	0.203717734	0.472616047	269.6894877
9.157607715	0.86262785	0.239084763	0.35355642	118.7456198
7.572279152	0.888169757	0.276847177	0.30830772	193.1145636
19.57647627	0.85986729	0.185594695	0.379061098	197.1920089
17.0080815	0.881393572	0.206171197	0.391623369	251.3065057
11.99776178	0.909552085	0.271840904	0.339042544	221.2967936
10.47982265	0.869102381	0.233310371	0.330717985	230.5640709
13.05381426	0.883645257	0.250797478	0.395682214	175.0304404
8.692840098	0.890322099	0.255615058	0.444386072	142.1124619
9.751857095	0.878738898	0.238325541	0.442391425	138.1574714
11.65812725	0.86886546	0.215180651	0.343029246	202.7399644
9.535083265	0.940040216	0.454695567	0.396392819	164.1578259
12.87769204	0.86858662	0.208095925	0.403828312	182.5869188
11.80430151	0.889941898	0.272478697	0.460457535	130.3430124
16.49875771	0.863686195	0.2015468	0.438760757	193.1935971
8.024258942	0.900873191	0.363085602	0.415228603	105.7746354
9.462848338	0.90935226	0.326426748	0.327770976	185.5936989
12.32196455	0.888174594	0.215549931	0.402713494	192.6438431
10.00368142	0.887461466	0.271476222	0.411846001	142.0907289
15.71251023	0.866912869	0.199658336	0.352927803	324.5875872
11.67924576	0.905927603	0.280462269	0.268062618	146.2688369
18.93370126	0.882271399	0.236494423	0.307658165	200.425228
9.407571987	0.927282423	0.35598577	0.332469529	302.4687312
12.98222898	0.891072198	0.263813145	0.344777126	148.9993691
17.41227176	0.871274483	0.219677623	0.399849317	232.4288241
13.35647577	0.922908467	0.333576108	0.340769832	205.5698802
10.47942384	0.899030772	0.282345191	0.336755974	178.9306112
8.63363022	0.902035108	0.315249518	0.467534947	143.1773962
16.88817002	0.88875181	0.221353505	0.351062208	222.7744063
23.66126372	0.850837562	0.166245237	0.279968212	351.2111591
19.46068864	0.841917264	0.172663193	0.341141768	224.4354094
11.80033851	0.891020046	0.235663714	0.413380755	266.3666809
17.49016569	0.901524255	0.251697399	0.327666748	199.5719015
10.11709974	0.889548294	0.26730871	0.372691963	188.1511067
11.28346226	0.880245112	0.221087212	0.3488422	254.7842259
14.0562059	0.878375414	0.231448651	0.369551019	190.7175714
11.60605976	0.890290043	0.289669548	0.373255602	148.5493405
13.85174598	0.874617613	0.213515885	0.43113241	189.6294558
13.75854468	0.875198802	0.216703363	0.370233477	250.769706
17.93495144	0.875212613	0.209430791	0.372573591	260.2773519
12.42070669	0.874952172	0.207113315	0.337400768	231.3125875
7.616443526	0.893775651	0.322148582	0.333353484	76.76612172
15.7002747	0.88980757	0.213255793	0.297728512	287.0256862
12.41155152	0.864485647	0.213954321	0.356509866	124.2553405
24.67334007	0.854168832	0.204310833	0.25732398	410.3552494
17.52844257	0.878481749	0.213542077	0.413454178	215.9396706
11.06320222	0.900924803	0.276228223	0.383406532	205.7677327
14.57467586	0.85261732	0.241028488	0.374878468	138.782751
14.84074208	0.90792635	0.267446194	0.335759048	327.4354619
8.805597273	0.869323755	0.251460017	0.403091867	130.6934691
11.73543103	0.913214754	0.389732773	0.417953651	134.8336005
22.16258617	0.856898494	0.171962092	0.303710801	251.0614384
21.76574195	0.874748267	0.171463932	0.302730043	307.0766052
19.30821379	0.876224866	0.195679401	0.307995535	333.0225847
12.479758	0.88337417	0.26492362	0.392303265	181.3311317
6.671575633	0.908302118	0.280445953	0.422081076	319.4011247
14.52213314	0.883831272	0.227320472	0.390963374	188.3285267
17.95820047	0.904633863	0.250424362	0.367167551	227.6579442
8.133046236	0.914944163	0.347080827	0.383148046	167.2057937
10.3468489	0.92926245	0.385278868	0.31837133	479.2184418
12.07502671	0.855805978	0.218359303	0.387456782	142.4822864
18.66037965	0.896523499	0.219135924	0.313462424	248.0925235
18.20088667	0.863152776	0.188822177	0.295202225	225.02022
15.90169629	0.891576656	0.233005852	0.375202042	300.0898029
12.12919098	0.863925613	0.22285393	0.384985022	178.8747769
17.33784782	0.883230854	0.227061275	0.334397676	212.59747
14.63423812	0.881530534	0.24655433	0.391861415	204.4013475
13.21500426	0.876898564	0.232812205	0.417878507	153.5203485
11.99653577	0.90549088	0.322062368	0.374192943	202.1247721
9.676272692	0.908444233	0.336614157	0.388928022	191.5686274
12.77677215	0.925646899	0.318058284	0.338527069	405.0754658
19.61301497	0.863854573	0.175547122	0.315010442	274.188246
14.26330898	0.874546102	0.222306461	0.338786099	202.3039102
14.52689551	0.862202658	0.215856052	0.367607939	138.1342562
15.13813043	0.871780345	0.208688341	0.344669836	252.8531239
14.55835623	0.891459549	0.241405627	0.343500572	195.9029523
20.51811991	0.856246524	0.166592815	0.31329008	215.3539622
24.33674756	0.861418272	0.156341155	0.292634141	329.1539874
20.2620477	0.895162754	0.21892528	0.317844706	455.7195688
18.40483158	0.858347962	0.197557439	0.348942682	338.2204452
10.15354688	0.889413724	0.268575798	0.393338237	159.9805234
10.27383841	0.931206095	0.320304932	0.340608268	309.5823325
12.80639832	0.871012774	0.222195231	0.423742313	152.585281
17.98673627	0.863543603	0.18260506	0.370998769	337.3126248
7.52258462	0.86023114	0.266056253	0.29368982	132.5052761
17.43015951	0.824881516	0.188406631	0.242879617	220.7986075
16.22009501	0.912429079	0.289967114	0.375413581	495.1484199
22.79830303	0.877746364	0.185583597	0.261045089	236.6840949
14.74256375	0.893309678	0.292763555	0.281892688	290.2268753
11.34589635	0.900463302	0.299604311	0.359328425	147.73079
9.116578899	0.904673253	0.30961591	0.393128877	151.6167891
32.11771116	0.848701946	0.148815085	0.343038602	325.2216138
12.56622607	0.88905442	0.22750524	0.340420952	338.8416078
18.57613051	0.840351564	0.161359596	0.341388673	252.9295762
7.640643737	0.876099109	0.251580594	0.482863342	124.7003311
22.6114919	0.893163687	0.24598646	0.265423782	207.5369513

log.sigma.2.5.mm.3D_glcm_SumEntropy	log.sigma.2.5.mm.3D_glcm_AverageIntensity	log.sigma.2.5.mm.3D_glcm_Energy	log.sigma.2.5.mm.3D_glcm_SumSquares	log.sigma.2.5.mm.3D_glcm_ClusterProminence
5.119658473	13.54501833	0.00429274	30.92006096	19125.70397
4.870781571	15.09990757	0.009134621	22.42548707	11027.77283
4.922967721	12.28674121	0.005869303	28.40161095	15840.61945
5.249011114	18.38115215	0.002914659	34.17406027	22733.73337
5.160877816	16.0547769	0.005368134	34.25007195	28572.41665
4.784842802	10.58893625	0.00592906	18.75076466	6213.222932
4.474500559	13.7485857	0.011047848	13.52316162	4257.681696
5.347123629	13.61869107	0.003078058	42.51224604	29439.25116
5.293488525	15.6391261	0.002976912	35.59448165	25852.23343
4.856519446	14.84403255	0.00623232	20.91404651	9779.946116
4.834012517	14.95568008	0.005538191	20.94176413	7312.037601
5.018141007	12.90154195	0.004949694	25.74148351	14312.99847
4.901687453	11.54884244	0.00529715	20.14428014	10795.54124
4.963354322	11.31863227	0.005012917	22.780419	10058.31295
4.960933783	13.99656024	0.004597392	23.54705805	10459.54995
4.37519072	12.67219475	0.033507814	13.21237882	6840.001414
5.093983163	13.08869911	0.00434185	30.13879159	15557.87653
5.036882856	10.92178253	0.005022667	25.43234856	16095.76041
5.276408266	13.36963074	0.003889042	39.54039198	27105.97095
4.630482692	9.999865785	0.009935062	13.86059	4840.367701
4.623319313	13.48176666	0.009907295	14.97455229	5475.649397
5.080105869	13.58735444	0.004095999	27.41610007	17311.9141
4.875959413	11.58034969	0.006111381	20.08027002	10140.59913
5.067144448	17.91243967	0.00493928	31.71104819	22432.63653
4.65734993	11.96821453	0.008065484	17.83265762	6629.363798
5.072287845	14.034142	0.004900156	31.84659228	18807.20302
4.528308907	17.43564775	0.013463707	14.07819516	6340.009618
4.847903807	11.94176703	0.006885949	22.45507419	11598.10416
5.201621205	14.93123996	0.003910884	36.29005734	19735.02222
4.804812116	14.29755002	0.01006188	20.17840081	10450.90185
4.732287481	13.19397823	0.007311759	18.12281179	7831.259143
4.852486103	11.61353338	0.007352518	18.30224569	9181.190747
5.16590494	14.83108854	0.003514372	31.92954624	17449.63029
4.782127864	18.65647912	0.011273092	43.45070406	37695.99039
5.197996497	14.64066418	0.004587789	40.78237612	26487.06655
5.0358602	16.19121688	0.004189212	25.64728941	11192.83983
5.072135819	14.08608076	0.004653195	29.74951346	17217.24687
4.864847283	13.46669608	0.005231141	19.09554948	7457.983681
4.988852123	15.78722825	0.005116589	25.56296866	13770.02512
5.071150311	13.49844403	0.004183925	27.49178907	13512.35267
4.8669892	11.89072678	0.006398064	19.71340479	8950.020919
5.186389263	13.31126652	0.004019385	31.20530696	20076.13858
5.089555699	15.64957586	0.004141624	29.98610808	13994.84672
5.245160824	15.91156797	0.003513726	35.8072079	24163.64684
5.0303399	15.00846972	0.003796689	25.57602529	11519.21241
4.480199514	8.513925594	0.009787545	12.7585955	3591.113534
5.086631364	16.9870589	0.003526553	27.63453957	14465.30102
4.965847748	10.73076779	0.004722706	25.72901734	11453.53599
4.793511423	20.36501172	0.01278399	39.85596914	38417.35018
5.19845288	14.27873752	0.005390233	37.64090772	32334.9551
4.906395838	14.15978804	0.005762933	21.14478077	9924.17269
4.968164935	11.31311339	0.005795334	28.61356642	11373.37381
5.012212873	18.13548752	0.004806818	24.84152535	14633.93818
4.803385445	11.10169913	0.00604402	18.55025161	7129.67796
4.755084019	11.2997206	0.014251276	18.33260168	8886.225503
5.326545791	15.77472884	0.002997941	42.49554541	27875.31631
5.381433829	17.57570954	0.002480659	43.47439067	33396.11457
5.17798157	18.27680148	0.003932218	34.14200588	25546.00432
4.965333085	13.14068251	0.005883686	23.56638922	11687.58976
4.641608575	17.76568731	0.00741254	14.8192665	4241.239541
5.090588911	13.42212076	0.004305207	29.58303321	17066.02935
5.173278996	14.99816528	0.003855643	31.11682345	26304.69213
4.602019301	12.73927891	0.010446493	13.97927398	5324.355831
4.505751293	21.94142377	0.015727065	13.55198138	5283.698591
4.895646568	11.52134186	0.007324905	25.13572238	11743.70952
5.126984538	15.80896735	0.004506488	32.00907048	25509.74654
5.057108789	14.76846669	0.004894023	33.58933369	18144.63551
5.170078646	17.31357153	0.003610012	30.47751543	18890.75696
4.931157765	13.00258265	0.005764192	25.44433791	10287.02333
5.148887937	14.47342779	0.003799671	31.87708363	17625.30691
5.093402932	14.00773203	0.00433948	29.07412009	15449.37782
5.08795971	11.93589137	0.004406013	28.13562623	15464.26274
4.836807374	14.05733685	0.007312029	20.22478168	9326.072527
4.716000776	13.63903068	0.008883707	16.36423582	6486.929881
4.769129428	20.18721848	0.009315237	18.55653887	11743.83722
5.274170154	16.53532159	0.003138533	39.94947903	25002.96343
5.055673551	13.9746667	0.004090332	27.72660377	13326.61471
4.978500162	11.31544175	0.005357358	29.15652149	16776.31605
5.140107614	15.81728763	0.003722728	31.45766187	16366.53183
5.032854462	13.85758499	0.004394944	27.15585362	12060.2853
5.255849218	14.4883966	0.003812797	43.11425127	27044.20514
5.422611569	18.16897646	0.002499498	45.7066009	35500.37218
5.243919908	21.41404923	0.003425241	33.5697865	21910.84897
4.840376366	18.22791932	0.009766443	36.68307543	30178.96917
4.885611599	12.33735282	0.005248468	20.09341556	8391.645016
4.65725099	17.62089991	0.009214329	15.92738715	8464.626035
5.041079572	11.88062526	0.005095396	28.52425666	16189.8013
5.313034892	18.30269996	0.003185555	39.55488146	26078.2373
4.465280132	11.30138454	0.011114682	15.83065475	4366.867125
4.619319293	14.59808511	0.013685266	32.32401806	16056.24415
5.070744202	22.19393227	0.006283393	26.39534697	18427.24543
5.154064357	15.4272025	0.004060067	39.33974429	27812.03853
4.840012728	17.02805071	0.009854912	22.08494573	9272.422438
4.837985233	11.89625365	0.006552423	19.2506192	8958.227114
4.757277865	12.05884659	0.00669757	16.39131065	6158.690102
5.44527611	17.93364124	0.004687883	68.09318745	74964.10122
4.952638106	18.41410508	0.005045321	23.32809537	11946.12119
4.917483029	15.55528757	0.008902952	41.69835999	30999.59609
4.845250908	10.7297462	0.007513809	19.75354922	9142.559023
5.052763093	14.45397232	0.00569345	33.53046412	25276.60761

log.sigma.2.5.mm.3D_glcm_SumAverage	log.sigma.2.5.mm.3D_glcm_Imc2	log.sigma.2.5.mm.3D_glcm_Imc1	log.sigma.2.5.mm.3D_glcm_DifferenceAverage	log.sigma.2.5.mm.3D_glcm_Id
26.92220694	0.846601084	-0.144181851	4.618904877	0.306946056
29.80158765	0.792943316	-0.122767884	3.663989053	0.394977237
24.33754057	0.891844485	-0.18593825	4.647755703	0.302973205
36.07610644	0.685358355	-0.072361227	5.324600997	0.286515866
31.86205715	0.950310297	-0.259500786	4.631854537	0.295590015
21.17787251	0.759996415	-0.107911487	3.840774152	0.329169741
27.4971714	0.853839306	-0.174376865	3.288683724	0.363035545
26.95325811	0.812513345	-0.118497314	5.564648661	0.273390882
30.83998643	0.727076136	-0.085824685	4.98789845	0.294321498
29.30153525	0.646572857	-0.069303665	3.767021912	0.354677317
29.91136015	0.734930099	-0.098398718	4.119490525	0.322000788
25.69680327	0.749353289	-0.101422159	4.065229304	0.336011204
23.08103276	0.701268254	-0.089271802	3.598628754	0.344041863
22.63486194	0.741231611	-0.102092932	3.899884021	0.327740796
27.92023934	0.763560289	-0.104191502	4.285959859	0.307056025
25.23138404	0.788972428	-0.139293822	2.396551197	0.509503985
26.11693383	0.833042662	-0.13981569	4.631091407	0.298196887
21.79577998	0.720106473	-0.093929965	3.810207274	0.355151779
26.5526853	0.89559332	-0.180922783	5.04593648	0.290979661
19.99973157	0.725856049	-0.104369989	2.811265124	0.433101265
26.89268203	0.612825322	-0.065130126	3.158074072	0.402625005
26.98352109	0.728101306	-0.090946189	4.352044144	0.306858923
23.14140341	0.659855214	-0.076771859	3.611193799	0.356175845
35.43436262	0.892866077	-0.181860043	4.886600825	0.291060578
23.80685965	0.716588438	-0.093544624	3.620244964	0.363041873
27.65727829	0.869684345	-0.160998364	4.742233046	0.32040236
34.52285401	0.686005858	-0.086747656	2.971016314	0.42661783
23.78807515	0.73072807	-0.096023392	3.903355049	0.347493726
29.55072118	0.722388706	-0.092817635	4.910318402	0.305143054
28.21802851	0.78233502	-0.117790731	3.467605745	0.405039894
26.30458424	0.628418793	-0.067012379	3.553082941	0.36473745
23.20891479	0.67697717	-0.085123659	3.201357855	0.393881599
29.12757383	0.708536049	-0.081930864	4.781559915	0.307426892
36.84207807	0.994106808	-0.504638938	6.210641933	0.253244945
29.05264086	0.945543743	-0.246357149	5.641744553	0.265063891
32.01315331	0.679951844	-0.078094976	4.11119669	0.323976129
27.59552635	0.694356563	-0.08083003	4.535780458	0.333357527
26.91250112	0.623374636	-0.064143081	3.657949727	0.352175304
31.38173999	0.808846385	-0.138402373	4.434611759	0.309892355
26.89096898	0.765481467	-0.103874705	4.409687914	0.318156843
23.76638835	0.748759084	-0.10331789	3.55496616	0.370313364
26.56036258	0.810811046	-0.123830708	4.546038115	0.302961476
30.9898983	0.725820009	-0.090536364	4.675073795	0.30415633
31.45468694	0.821223632	-0.126690328	5.021317031	0.296471152
29.86459047	0.66680043	-0.071495799	4.498933527	0.298587097
17.02785119	0.629019799	-0.072256456	3.00581127	0.400157429
33.41354967	0.592411506	-0.052854669	4.704386728	0.30213926
21.4521406	0.815505413	-0.130641297	4.466598226	0.304203088
40.00961641	0.97886525	-0.382943417	5.943684866	0.285205813
28.34277611	0.908085471	-0.196181805	5.005718763	0.299862633
28.14107886	0.666338405	-0.075967189	3.706647337	0.35945848
22.62622679	0.74916999	-0.105736031	4.484257193	0.324332208
35.73251902	0.686860721	-0.080662259	4.187905551	0.347585606
22.20339826	0.796012492	-0.124694008	3.626982694	0.341026556
22.56622323	0.801415686	-0.13531402	2.9964958	0.4525934
30.91125882	0.83652658	-0.130130555	5.898029118	0.260967284
34.3069245	0.739550265	-0.09067882	6.087101275	0.259081245
35.92999182	0.774484035	-0.105559997	5.276430022	0.284318539
26.24231824	0.819797798	-0.133031745	3.933726058	0.348841912
35.40178419	0.73595524	-0.105596086	3.125852534	0.368074471
26.62286149	0.702495612	-0.082037243	4.456759677	0.314664569
29.44262171	0.685603028	-0.077296944	4.489464013	0.332800273
25.45236109	0.701542874	-0.094916204	2.934596515	0.420131773
43.57901812	0.784685444	-0.129056339	2.915577473	0.449726219
23.04268372	0.945226773	-0.265194848	4.285610887	0.310786764
30.8884588	0.795773963	-0.114243877	4.901217822	0.305064038
29.34786201	0.914932393	-0.20443689	5.24710039	0.278841956
33.99207443	0.680441307	-0.075515351	4.638035812	0.317540525
26.00516531	0.843037195	-0.150316454	4.32822924	0.311835284
28.46387267	0.718032368	-0.084869105	4.793968295	0.311335155
27.81509119	0.691924904	-0.082310047	4.315288091	0.33089207
23.82533383	0.765666397	-0.10572966	4.314495575	0.320367886
27.92525473	0.685300927	-0.084975522	3.458856605	0.396763716
27.22519367	0.674048733	-0.082243049	3.103403724	0.410077216
39.93811974	0.650636181	-0.072101145	3.481014461	0.392720567
32.37832159	0.800348263	-0.114115526	5.705080126	0.264832426
27.79951255	0.763393852	-0.103485221	4.566746251	0.310294061
22.58258307	0.825282064	-0.136401206	4.598488031	0.305231319
31.15279305	0.706871797	-0.087518022	4.840110529	0.297166971
27.35308895	0.64978221	-0.068397709	4.38505932	0.325716491
28.45863302	0.909349883	-0.192256447	5.903428756	0.256244069
35.5494366	0.7891766	-0.104913174	6.338564249	0.245731055
42.19051784	0.723466037	-0.086033474	5.110373192	0.303250752
36.10595634	0.985209818	-0.407589285	5.304143025	0.285702586
24.66179343	0.634877463	-0.070949623	3.670024756	0.353308465
34.88580013	0.632009642	-0.070030768	3.244351544	0.397069539
23.71713439	0.806903134	-0.125118189	4.381780824	0.311734253
35.94608674	0.742029566	-0.091637113	5.530844796	0.271014556
22.60276908	0.932113487	-0.264940821	3.605609787	0.351800957
29.19617022	0.981432161	-0.418692855	5.501425012	0.275493251
44.04922919	0.763334202	-0.105098831	4.093656293	0.366247249
30.13852124	0.866177914	-0.15424068	5.649511993	0.274148119
33.72006035	0.809194841	-0.130288133	3.978466825	0.37102402
23.74084436	0.667900122	-0.077984112	3.524886883	0.378430873
24.10001485	0.634235156	-0.069853964	3.217998708	0.388678507
34.76933712	0.985229681	-0.365722097	7.333716583	0.233547025
36.39460593	0.749143638	-0.098749437	4.199801668	0.317351458
30.94441675	0.991228146	-0.452910169	5.871111796	0.251818341
21.4594924	0.883671188	-0.187680161	3.528279566	0.343111046
28.25100204	0.865838114	-0.159052011	4.800884124	0.327524612

log.sigma.2.5.mm.3D_glcm_ClusterTendency	log.sigma.2.5.mm.3D_firstorder_InterquartileRange	log.sigma.2.5.mm.3D_firstorder_Skewness	log.sigma.2.5.mm.3D_firstorder_Uniformity
82.91404847	198.5801525	0.338366828	0.052708873
57.73568638	131.3199196	0.410453552	0.070909605
71.45604995	192.0229244	0.611653487	0.059153943
89.18423929	215.6613235	-0.01805539	0.047041108
98.91427164	175.7034569	-0.042055519	0.053374819
50.70865769	171.8659	0.103465082	0.065021321
35.17486978	95.34950638	-0.539102226	0.095422166
113.5521635	260.5724754	0.020588839	0.046575512
96.58753944	220.7206573	0.135153484	0.046482137
53.12556224	137.0725613	0.609331572	0.06925666
55.60146917	189.1699905	-0.407939827	0.066944083
69.93562992	172.3507195	0.477257096	0.057834819
57.83495855	160.1186981	0.318221131	0.063782292
65.62575396	200.6708832	0.089056168	0.060584484
61.88737719	163.8919907	-0.024290271	0.059737011
35.4869914	59.09825993	0.760461525	0.137881464
83.50198832	218.6703987	-0.106623938	0.053776242
73.19837292	188.5605793	0.522582052	0.057883235
110.6328435	240.238409	-0.065053648	0.046692883
39.25871897	126.5623322	0.387766612	0.07815985
38.78442932	113.6478953	0.558413029	0.082462055
74.23876898	177.9913654	0.386358591	0.056047188
56.25572004	149.3898602	0.529500603	0.067379817
82.96806241	181.8370132	-0.48570844	0.057282775
43.42748518	133.9128523	0.728833944	0.075335813
78.86962618	192.3992729	0.465091857	0.053326579
36.47538132	92.59931898	0.686164082	0.093817811
58.69530229	147.4731941	0.658629141	0.067507058
98.96739512	262.3833847	-0.196882027	0.052414513
51.7408034	124.1992803	0.530037914	0.073296084
47.18326724	137.7247887	0.595821971	0.074259248
53.30528895	144.1253433	0.52331403	0.071766392
83.01898252	220.834198	0.122223947	0.050894863
110.262621	152.993001	-0.488762841	0.057507396
106.0609752	234.527174	-0.28496201	0.047212948
69.82000401	204.2669659	-0.044245743	0.055607784
75.42376267	197.4964581	0.61540743	0.057070795
52.05190962	157.8757477	0.111615112	0.063470881
67.00605264	156.9489861	-0.317760935	0.062431783
73.66270791	196.9397888	0.080968734	0.054195698
53.87468055	146.4267607	0.256989559	0.064314237
88.07024144	213.5682297	0.052187945	0.053475577
79.46311013	228.4642315	-0.168730431	0.056282926
95.7467777	223.315033	0.086988576	0.048324569
66.50810565	192.4326305	-0.117846091	0.056029628
34.06385423	135.5668221	0.548968903	0.08049623
69.95966444	185.6546383	0.150805892	0.053160447
69.61072844	208.8730392	0.035597903	0.054944408
101.6870402	92.07659912	-1.068904073	0.091779906
103.6519955	162.1770935	-0.291599425	0.062503795
56.30627418	154.3738823	0.403672851	0.064758999
78.75971174	256.4778214	-0.175887788	0.064590038
65.9409655	185.0040207	0.341058156	0.056626485
51.96017495	155.4194603	-0.025848639	0.065629107
51.22215267	104.9167919	0.468270886	0.088696234
106.5455331	240.9450684	0.050042207	0.044658515
112.8694788	264.4224548	-0.078803202	0.042453406
89.63679094	176.1536026	-0.658241438	0.056328652
64.8709926	169.2465868	0.086864017	0.058901653
41.0242534	148.8503952	-0.144511174	0.073282476
79.38805303	212.6459541	0.400801376	0.055051255
82.49389465	190.4817638	0.51851843	0.053320854
38.06394414	108.8330755	0.329210555	0.085818121
36.95149262	102.1994762	0.123503718	0.092164351
69.6745256	178.8028793	-0.223710113	0.059610525
81.73990575	159.4961472	0.428251925	0.057944472
85.22092799	203.2982445	-0.351428686	0.056713649
82.90195708	226.712822	0.257579438	0.050282419
70.44888326	222.342103	-0.141854363	0.0601661
81.19258999	199.657814	0.364532638	0.052943272
78.21333015	202.2424641	0.41716647	0.055796399
78.67983302	214.5658722	0.156484226	0.055316752
53.05983834	139.1230602	0.611707266	0.069740541
44.61616848	122.2970915	0.495157393	0.076853175
50.41902109	105.499403	0.495447182	0.080657534
102.1470075	256.3633633	-0.128506973	0.046468121
72.24112422	202.6510925	-0.003160904	0.055298235
78.72973434	223.8133202	0.44291763	0.056237571
80.61502102	216.8158569	0.104895605	0.052093135
69.70383155	196.2854977	0.336094236	0.058155125
107.9779733	283.3621197	0.09033053	0.044028723
118.2471909	267.8412857	-0.104906159	0.041762661
90.07251726	196.1460781	0.037318817	0.052108819
96.50375172	154.8357353	-0.842574044	0.065969084
55.85968222	173.4365387	0.332674763	0.062574697
42.39823631	114.2628136	0.724045979	0.082741291
80.19036567	224.7263851	0.333322879	0.055339312
106.749988	260.2001438	-0.400202297	0.050403869
41.06833414	146.5712051	-0.259106039	0.073546538
79.68865931	180.2725716	-0.866157014	0.083718821
73.87545122	167.2649002	0.077523498	0.060704751
93.85576154	236.0814705	0.554696797	0.047877762
54.83509874	156.0353632	-0.018852511	0.070628176
51.4846998	146.4063911	0.339417716	0.066809261
45.34810408	144.822711	0.374793123	0.069007687
181.1856471	357.6787262	-0.024207753	0.038041525
61.36275819	156.6125822	-0.340825512	0.060241192
108.476435	178.0675201	-0.560527804	0.05651398
58.59401158	138.0979919	-0.122889921	0.071254029
78.35894199	164.8285542	0.847637441	0.058476865

log.sigma.2.5.mm.3D_firstorder_MeanAbsoluteDeviation	log.sigma.2.5.mm.3D_firstorder_Energy	log.sigma.2.5.mm.3D_firstorder_RobustMeanAbsoluteDeviation	log.sigma.2.5.mm.3D_firstorder_Median
111.5064761	13104426.05	81.44381115	-13.8582387
86.43106039	26820731.91	57.27171711	-14.90921879
104.4986626	7434712.9	75.18845062	-44.71576309
120.0543389	57275360.44	87.82839508	22.61924744
112.5175487	7412855.022	75.92800714	10.9218235
91.59324193	8282020.698	70.05281821	-28.85847282
66.63551804	2665148.17	41.72372762	6.827986717
135.1445633	25061760.41	104.6906614	4.9419384
122.7963668	46908522.45	90.54720666	-21.38686752
87.50119367	39978254.08	59.11721063	-35.50225258
96.15572341	11025649.49	74.21064852	-1.716737151
102.3653961	23910709.36	72.7747046	-31.12221527
91.05650137	26027542.06	65.95280971	6.783992529
102.1432613	15803820.58	80.03656242	10.35583496
94.45150822	12704172.46	67.25428006	20.18841362
55.59684055	48665758.13	28.83328058	-2.812831879
115.3316667	12972415.72	89.63151538	0.004750147
103.8526769	36984866.05	75.96629135	-26.46100712
127.4193864	13598677.53	95.94304688	23.83413792
73.0443534	24203282.18	50.8432006	-5.923105717
73.78145294	50917183.68	48.67312016	-33.19821358
102.9495837	27970311.79	73.50805646	0.298611693
88.91036493	34093438.74	62.27001858	-32.80426407
106.9476371	8609817.987	74.22245911	24.15733719
81.14475895	14501955.74	54.86357961	-52.64126778
110.9174454	13757685.79	78.74279012	-44.00499916
67.39847314	30151981.25	41.43389301	-10.52275133
93.19401822	21594754.84	63.1232247	-53.57873917
129.7324545	38406875.94	105.1901617	31.20385933
84.27926164	33362847.21	55.94196108	-28.76881123
82.71658881	37013415.01	56.85700655	-44.40602493
83.98162209	66437889.36	58.27565831	-8.740665436
116.8372257	39135451.58	89.13275447	11.43070793
116.7913405	2484701.9	73.29142826	19.03251457
129.1756877	7893022.587	95.39013384	17.64030933
107.2169199	31648564.1	83.57075906	-2.474123001
109.2792664	51853135.25	80.12380352	-36.26464462
89.3005252	40136354.43	64.75486104	0.746542513
95.23482956	12205180.05	65.55518196	24.16675186
107.3963855	17935572.17	80.04286609	-9.145194054
88.72378739	18453754.54	61.5892202	-18.51292992
116.5145608	18319329.31	88.69792066	-5.146178246
114.9146316	29398064.69	90.60996067	-0.998590052
123.1651328	22321986.43	92.03600078	1.110691071
103.9287808	29027678.75	77.99001899	0.635365665
76.26753373	19464762.53	56.13538428	-33.25906754
105.8667704	73077380.55	75.96977835	1.91572994
107.7681095	9805122.715	83.11296576	-24.33673859
103.5225728	2923279.405	55.51293413	21.58437824
113.7345076	10593386.02	75.08067818	38.49808502
88.56035078	42412442.64	62.38498268	-12.07446766
122.0017075	16135259.81	103.2953804	-9.877625465
101.6323228	60730268.37	73.40865468	-13.22870159
86.24779696	6573014.013	62.87435918	6.144016266
73.98036505	65748010.79	46.27256741	-4.639205456
132.2380997	21915765.02	99.92047658	-14.16979694
139.7537386	60130233.11	106.8538216	-5.968500137
112.8025722	26572614.8	75.91896806	28.75768089
97.19979304	12330188.17	68.99792942	-24.62186432
80.43189018	8348995.553	60.63240295	-8.688201904
114.4696565	39073074.09	87.80657126	-41.08408356
108.2333346	100914323.7	76.72361879	-2.189120531
68.5919383	25245396.01	45.38396144	6.253448486
70.03435548	24465811.19	45.02622625	-8.457544327
101.4361445	3413634.466	74.57415917	-23.41552925
103.4593635	29917973.53	67.96812165	22.86702347
116.0365807	7980581.111	84.39231294	4.051864624
117.8351187	62778699.86	89.51660733	-7.984398842
109.7743508	7912698.221	87.32941023	-16.5588522
111.8030856	36868377.7	82.54656595	-17.6635704
108.6753088	52061866.25	80.98182826	-30.84878731
113.4381912	20241009.52	88.63454595	-23.67069054
84.53676384	60238098.65	58.4677362	-14.73540783
77.00209343	67642203.18	52.37748803	-24.10402584
77.1220729	105635996.1	46.94296129	9.257752895
132.7987698	27470335.77	102.4889989	-6.285346985
108.5511949	19317830.02	82.32868863	-21.62577248
115.3052605	11977951.91	89.01826373	-62.37801933
114.475466	35372867.5	87.26350717	-57.19018364
105.0091051	45626504.98	79.20079106	-39.88191032
141.4100237	15576495.5	110.2624136	-73.71199036
144.3140056	38780361.49	110.2163414	13.99094772
110.7004789	55498907.25	77.75623869	17.91955376
103.0755447	2846114.694	61.92412261	34.88682556
94.35776516	43181012.18	70.62710233	-11.08082294
73.4282635	52264832.55	47.53409227	-11.9135251
115.9431915	14648290.99	90.0642803	-45.24918365
133.3218959	37880342.14	103.9003754	10.92930222
84.02139924	1837534.674	63.08531752	18.89027596
112.463567	2090075.182	82.17059173	10.96888828
97.70234475	63637661.39	66.58442571	-6.117517948
131.7677912	19262051.28	96.83150512	-76.58654022
88.37225002	13411729.54	60.26423809	-12.59772539
87.64302619	48857289.19	62.41950697	-12.3721242
82.68006209	60527167.13	59.35465263	-12.64421988
183.1762199	11291521.26	146.4457788	-6.874035358
97.36756109	18528801.16	67.41852286	29.86811733
118.155044	2735813.894	77.75716994	34.72352982
85.57743832	4558822.203	58.77790119	28.85103035
109.2744705	17226688.99	70.50150462	-51.07072449

log.sigma.2.5.mm.3D_firstorder_TotalEnergy	log.sigma.2.5.mm.3D_firstorder_Maximum	log.sigma.2.5.mm.3D_firstorder_RootMeanSquared	log.sigma.2.5.mm.3D_firstorder_90Percentile	log.sigma.2.5.mm.3D_firstorder_Minimum
353819503.4	370.342926	136.2406327	165.8438873	-344.4629211
724159761.5	439.4565735	113.6362947	152.6061646	-350.2727051
200737248.3	455.5950623	130.8842041	158.4013885	-314.1792603
1546434732	490.573822	147.4326276	201.7903961	-408.5057373
200147085.6	377.5541077	143.6962812	184.4049774	-396.6568909
223614558.8	240.9631653	113.4913258	105.1106735	-278.7085876
71959000.6	227.1971283	88.92947511	86.70670471	-326.6932678
676667531	402.8895264	158.1509941	183.1542404	-349.5354919
1266530106	432.6713257	150.209926	170.8937897	-385.6841431
1079412860	482.4882507	113.598136	144.084137	-365.8652344
297692536.3	205.0581512	115.4647278	108.3259567	-398.7411194
645589152.8	360.7912903	127.0200059	171.3997498	-322.6229858
702743635.6	391.78479	112.0242906	145.0667191	-260.2476196
426703155.6	351.9909668	118.8940561	153.5624649	-271.5505676
343012656.5	321.4362488	118.0902391	167.2671661	-304.8279724
1313975470	539.3448486	83.69761779	105.5064285	-295.40271
350255224.4	330.6973877	136.3272636	141.9580139	-341.2585449
998591383.4	400.8877563	125.9353786	166.5513672	-264.6266174
367164293.4	390.4538269	152.8575327	205.2341049	-322.8192749
653488618.7	323.6492615	92.9566743	118.9274673	-249.3747101
1374763959	368.9558716	98.72578665	116.5544418	-335.988678
755198418.2	460.0144653	127.5217988	173.2218628	-310.43396
920522846	345.6599426	111.7312233	144.9204712	-281.0852966
232465085.6	290.9222412	133.7902444	165.0116577	-422.0794067
391552804.9	457.7076416	109.7488667	117.6565735	-308.3278503
371457516.2	404.4634399	140.7968563	163.1304688	-362.3818054
814103493.7	426.7346802	93.27035891	116.5851059	-424.335144
583058380.7	389.1439514	122.7583504	151.9979767	-302.2788391
1036985650	379.5055542	147.5136253	185.5926239	-325.2300415
900796874.7	568.4447021	114.5628424	118.466938	-356.0941162
999362205.2	374.8093262	107.9031975	123.4863251	-347.0235901
1793823013	398.3338318	105.8474907	155.0557175	-274.2567749
1056657193	511.663269	138.7447266	176.5778229	-348.8345947
67086951.3	324.4610291	154.5683164	207.1951035	-471.0613098
213111609.8	292.8249207	154.1887299	185.0135452	-363.0621033
854511230.8	351.286377	125.1703079	150.4609863	-381.8804016
1400034652	582.7625732	134.1810749	178.5019089	-331.73349
1083681570	332.9886169	109.392471	150.4018341	-313.6530151
329539861.3	352.5349426	120.9007117	155.9568787	-359.8476257
484260448.6	365.8306274	130.7584831	146.920578	-340.1594238
498251372.5	347.8742371	111.8906065	146.3611801	-295.3947449
494621891.3	367.9535522	138.646316	148.1066345	-331.0906677
793747746.8	338.4337158	133.8051392	138.4246002	-380.9173889
602693633.6	386.3051453	147.5721781	178.0949554	-397.5590515
783747326.4	326.5047913	124.3249691	141.2594315	-370.8552856
525548588.4	345.0484619	93.46886341	123.5335419	-218.5570831
1973089275	458.3229675	130.8823428	181.2758331	-377.8041992
264738313.3	328.4962463	129.6857444	136.5184937	-281.1339417
78928543.94	241.6381226	147.7007717	183.1412567	-505.9125671
286021422.7	467.0424805	145.9954109	166.5436005	-333.7768555
1145135951	420.5357971	111.2960535	141.5499649	-330.1949158
435652015	233.1394653	139.6803217	124.2869659	-312.8016052
1639717246	477.5634155	126.0706759	160.3841705	-435.1495972
177471378.4	270.3852539	105.4601874	144.243103	-260.4098511
1775196291	418.6398315	100.3962797	145.6357269	-259.916748
591725655.5	382.0305786	157.9006363	190.1722412	-384.8323059
1623516294	523.5181885	165.5873393	189.1242645	-445.8825378
717460599.7	391.3504639	143.3566632	158.88974	-446.9373779
332915080.6	318.7522278	123.075795	143.4704391	-349.8394165
225422879.9	263.2419434	97.34847682	112.0743103	-426.9316406
1054973000	420.7926636	135.7917271	163.5632507	-328.6585083
2724686740	612.7379761	135.7267322	171.6832993	-325.4385376
681625692.3	409.7819824	90.38822419	128.2299118	-285.190979
660576902.2	313.7712097	94.64963856	130.0241699	-528.9921875
92168130.57	229.6944733	125.4234205	139.756189	-311.5490723
807785285.3	612.5117188	138.7970894	203.7561859	-329.1846313
215475690	348.8269348	142.3210526	131.182402	-384.1815186
1695024896	433.6109009	140.1311998	174.8936432	-416.5336304
213642852	234.7645111	133.1971315	110.3468208	-366.290802
995446198	457.1208801	135.4007216	187.6361084	-328.7217712
1405670389	364.0983582	130.8648689	179.4539856	-342.944519
546507257.1	350.6598816	133.8964657	139.971283	-307.5348816
1626428663	383.8860168	107.9628586	138.8240555	-326.7263489
1826339486	338.7112427	100.3583537	129.3046875	-328.9534607
2852171895	515.0124512	107.2715671	145.2381531	-464.6225586
741699065.9	380.6167908	156.8916703	166.3689575	-410.493103
521581410.5	345.7218933	131.5081243	131.7865479	-373.4073792
323404701.6	318.7301636	140.1284651	149.0893707	-302.0865784
955067422.5	321.1436157	141.6073655	154.3463821	-408.4886475
1231915635	462.7808533	126.6167476	150.7988663	-337.635376
420565378.4	367.2106323	175.8002987	153.5383041	-423.056366
1047069760	449.7953796	170.0558348	206.3710632	-436.0155029
1498470496	497.7666931	140.9887659	202.5731354	-493.9676819
76845096.75	269.1703186	140.1013105	183.3816437	-432.4517517
1165887329	357.1982117	112.8450502	163.562851	-287.3373108
1411150479	593.3013916	97.69516302	126.0015106	-417.0692444
395503856.6	347.5917969	137.2156606	156.3076996	-313.9093628
1022769238	317.3756104	156.2287968	153.1955948	-473.1691284
49613436.19	213.8949127	100.2056539	140.067865	-269.0405579
56432029.91	137.3712158	141.0867744	91.35574493	-423.6697998
1718216857	412.3717346	126.5285524	163.7764648	-527.8508911
520075384.5	484.4847717	165.17675	171.2713165	-403.0429688
362116697.6	305.4728699	114.5557639	138.2005463	-415.7792969
1319146808	408.3638611	109.2956883	131.3670303	-284.4359741
1634233512	384.3813782	102.1025664	131.936615	-284.2613831
304871074	443.0446472	211.2593638	218.1478271	-459.7372437
500277631.2	330.5808716	123.4402728	163.2216415	-423.2108765
73866975.15	299.6274719	149.1388259	171.0830078	-382.1488647
123088199.5	300.2646484	109.6747675	149.710675	-241.6482849
465120602.7	507.3866272	146.1046839	181.9885193	-365.4795837

log.sigma.2.5.mm.3D_firstorder_Entropy	log.sigma.2.5.mm.3D_firstorder_StandardDeviation	log.sigma.2.5.mm.3D_firstorder_Range	log.sigma.2.5.mm.3D_firstorder_Variance	log.sigma.2.5.mm.3D_firstorder_10Percentile
4.427774368	135.6486898	714.8058472	18400.56704	-178.8882599
4.146196915	113.2507643	789.7292786	12825.73561	-152.7328888
4.28304027	129.1488638	769.7743225	16679.42902	-175.9462936
4.576281878	146.221479	899.0795593	21380.72093	-179.0520416
4.497386856	143.6745134	774.2109985	20642.36581	-201.9255585
4.08654866	107.9005508	519.6717529	11642.52885	-170.3844513
3.760962116	88.75570192	553.8903961	7877.574623	-132.0176117
4.585718592	157.7132099	752.4250183	24873.45657	-225.2041916
4.584951563	148.7921132	818.3554688	22139.09294	-219.5265015
4.140301166	112.1071882	848.3534851	12568.02164	-142.9099152
4.066927435	112.8008504	603.7992706	12724.03186	-188.0197021
4.318511819	126.5039531	683.4142761	16003.25015	-164.8442993
4.164566492	111.8163521	652.0324097	12502.89659	-134.7317505
4.192184478	118.8390675	623.5415344	14122.72397	-151.583342
4.255725123	117.1267158	626.2642212	13718.66756	-139.4406586
3.522408761	83.60383742	834.7475586	6989.601631	-93.49076996
4.363354821	134.7422597	671.9559326	18155.47655	-208.524324
4.283187496	125.8148746	665.5143738	15829.38267	-155.725975
4.552834775	152.3845347	713.2731018	23221.04641	-201.6037231
3.90101525	92.84673581	573.0239716	8620.516351	-121.1547546
3.922579	96.20224365	704.9445496	9254.871683	-132.5618561
4.351887734	127.2847474	770.4484253	16201.40691	-152.4637482
4.130975709	110.9065193	626.7452393	12300.25602	-145.9950867
4.354284075	133.3398225	713.0016479	17779.50828	-158.1615601
4.003604904	103.9001494	766.0354919	10795.24104	-150.2090347
4.434634022	137.8715101	766.8452454	19008.55331	-195.0924957
3.830647972	93.12540698	851.0698242	8672.341426	-114.2664604
4.174293018	118.6444179	691.4227905	14076.49789	-166.7586029
4.397834258	147.1602534	704.7355957	21656.14017	-193.1346863
4.113561877	110.5612147	924.5388184	12223.7822	-165.3094421
4.033284096	104.6472748	721.8329163	10951.05213	-141.019577
4.062126524	105.5850967	672.5906067	11148.21264	-117.4695908
4.443848238	138.7120347	860.4978638	19241.02858	-178.9467773
4.380895974	154.3127122	795.5223389	23812.41316	-180.9948227
4.542902766	154.0689998	655.8870239	23737.25669	-228.2081085
4.277690213	124.8126641	733.1667786	15578.20113	-177.8688782
4.347755009	133.5343801	914.4960632	17831.43066	-165.3923889
4.1584423	109.3804126	646.6416321	11964.07466	-136.8270676
4.256954265	120.3140494	712.3825684	14475.47049	-163.5237274
4.357972548	129.7480424	705.9900513	16834.5545	-193.5171631
4.175040064	111.4222639	643.2689819	12414.92089	-148.0637527
4.415412493	137.6520487	699.04422	18948.08651	-199.0632813
4.323449937	132.5961998	719.3511047	17581.7522	-197.7252411
4.536352419	147.4545263	783.8641968	21742.83732	-206.3297729
4.315492485	123.9009423	697.3600769	15351.44351	-177.5310532
3.821811456	92.28754575	563.605545	8516.991101	-123.3960449
4.422293251	130.7036151	836.1271667	17083.43499	-160.9984207
4.284030397	126.837973	609.630188	16087.87139	-199.6004761
3.967604324	147.6983425	747.5506897	21814.80038	-216.9760727
4.376076702	145.8135503	800.8193359	21261.59145	-225.2966797
4.167078104	111.1436145	750.7307129	12352.90305	-141.1539047
4.114162893	134.5287209	545.9410706	18097.97674	-222.1784363
4.337846974	125.8617661	912.7130127	15841.18416	-164.0044861
4.093616068	105.3824843	530.795105	11105.468	-135.775116
3.920778452	100.3951903	678.5565796	10079.19424	-131.9106354
4.625415828	156.7872344	766.8628845	24582.23686	-231.1468689
4.704403842	164.8857654	969.4007263	27187.31564	-237.9984924
4.444955491	143.3524196	838.2878418	20549.91621	-211.6160339
4.28377078	121.1121646	668.5916443	14668.15642	-177.2841293
3.924699911	96.57261203	690.173584	9326.269395	-133.0159149
4.362997564	134.3762449	749.4511719	18056.97521	-177.900351
4.43631757	135.5928195	938.1765137	18385.41269	-163.707605
3.848965951	89.81156104	694.9729614	8066.116497	-105.4977234
3.832456355	94.20139773	842.7633972	8873.903334	-124.741272
4.221619637	122.928247	541.2435455	15111.35392	-198.1555664
4.426386952	135.9878225	941.6963501	18492.68787	-142.0220795
4.39893005	140.3285594	733.0084534	19692.10459	-234.8149124
4.455941165	140.1019294	850.1445313	19628.55061	-180.2949677
4.197150983	126.3311739	601.0553131	15959.56549	-217.3581009
4.420347314	135.3521158	785.8426514	18320.19524	-165.3066559
4.339735078	130.5785574	707.0428772	17050.75964	-163.4417496
4.333138691	131.7637628	658.1947632	17361.68918	-192.7070801
4.085315666	107.6274964	710.6123657	11583.67797	-136.0819138
3.970875355	98.94133207	667.6647034	9789.387192	-135.3992615
4.044923079	106.5370462	979.6350098	11350.14221	-116.5255272
4.581048217	155.1262821	791.1098938	24064.16341	-227.1208954
4.337523086	128.4354647	719.1292725	16495.6686	-198.491214
4.306401049	134.8351068	620.8167419	18180.50604	-197.8277878
4.413965295	135.9036598	729.6322632	18469.80476	-205.2922989
4.288840112	125.1086754	800.4162292	15652.18066	-169.8246155
4.637977928	164.5735389	790.2669983	27084.44969	-275.7652649
4.737837163	169.9367653	885.8108826	28878.5042	-235.7807922
4.489246428	139.5963334	991.734375	19487.13629	-168.1974014
4.254818352	138.9626768	701.6220703	19310.62553	-175.414505
4.155062	112.820292	644.5355225	12728.41828	-134.8942261
3.935048421	97.69275199	1010.370636	9543.873791	-106.7587624
4.346007277	135.1440328	661.5011597	18263.90961	-184.5018951
4.503599386	155.001277	790.5447388	24025.39588	-248.6902802
3.932276888	99.92964447	482.9354706	9985.933843	-116.5106003
3.949603479	133.4778143	561.0410156	17816.32692	-232.3206726
4.337267316	126.4784276	940.2226257	15996.79264	-159.5005005
4.583918379	159.200072	887.5277405	25344.66293	-227.9473801
4.150818369	113.6956879	721.2521667	12926.70945	-158.3729324
4.133570279	108.7289148	692.7998352	11821.97691	-143.3727921
4.047914077	101.9507019	668.6427612	10393.94562	-131.2022934
4.866161569	208.5757632	902.7818909	43503.84898	-306.6196594
4.29923736	122.5654584	753.791748	15022.29159	-152.2835922
4.412799955	149.0120659	681.7763367	22204.5958	-199.6974182
4.070624663	108.0854406	541.9129333	11682.46248	-135.8902374
4.421269519	143.8052758	872.8662109	20679.95734	-183.1341583

log.sigma.2.5.mm.3D_firstorder_Kurtosis	log.sigma.2.5.mm.3D_firstorder_Mean	log.sigma.2.5.mm.3D_glrlm_ShortRunLowGrayLevelEmphasis	log.sigma.2.5.mm.3D_glrlm_GrayLevelVariance
2.673946701	-12.68632936	0.01063786	30.13125545
3.230750174	-9.352638917	0.006227381	22.18034236
3.08954995	-21.24254837	0.012711063	27.14140752
2.520370855	18.85891682	0.005141563	34.77841772
2.879998227	2.501084419	0.009906694	33.94193289
2.198746677	-35.18170221	0.017005015	18.78034763
3.820667505	-5.556700532	0.009458358	12.94885321
2.111762207	-11.75926725	0.01774961	40.01535199
2.496915914	-20.58953428	0.008522665	35.97330706
3.405479982	-18.34434132	0.006370797	20.96450361
2.194955938	-24.65910613	0.007026076	20.54380546
2.760333138	-11.43817043	0.011030483	26.13296812
2.88412801	6.822396994	0.013558449	20.29333677
2.162145342	3.615606418	0.017456555	22.74948168
2.651105791	15.05446808	0.009785	22.48510712
6.17604768	3.961009124	0.007844188	12.88675
2.120076798	-20.72790993	0.013697791	29.11613642
2.682801145	-5.507894884	0.016843093	25.88278502
2.184324383	12.01577736	0.01995191	37.050143
2.993979457	-4.519617921	0.015565609	14.59951719
3.497707797	-22.17902762	0.007135337	15.94068145
2.912983602	7.771889648	0.009313539	26.17023487
2.925036804	-13.55028528	0.011778449	20.2452731
3.136897691	10.96910293	0.009806744	28.97702513
3.701851988	-35.34929558	0.010450402	18.03395886
2.844395337	-28.55173244	0.009731877	31.26919593
4.636417948	-5.197925207	0.003858639	15.36647448
3.069718706	-31.51372237	0.011628859	23.28366738
1.815814668	10.20438553	0.010032043	34.6593144
4.027291675	-30.01437414	0.007185245	21.25840274
3.270746196	-26.30680335	0.008159465	18.1086503
3.071437898	7.448399516	0.011947331	18.61295404
2.384401029	3.011738754	0.009149853	31.26534227
3.553401	8.885452542	0.01433724	38.5201764
2.225872799	-6.075173425	0.015339984	37.87121893
2.082213749	-9.455414339	0.005780084	25.07653164
2.858136634	-13.15789452	0.008135359	29.38899148
2.632089702	1.624207286	0.010225793	19.68683867
2.997671779	11.89586486	0.007869158	23.62141537
2.383997509	-16.22425359	0.010060578	27.42125645
2.826716065	-10.22677578	0.014268267	20.62244944
2.360349584	-16.57451179	0.014409932	30.35001469
2.069813148	-17.94611622	0.007034101	28.35619341
2.411332744	-5.891554266	0.007234078	34.9869498
2.388387369	-10.25935852	0.00905587	24.85214938
2.561234055	-14.81341708	0.021617388	14.09564498
2.734707847	6.837592335	0.005501052	28.0198063
2.0913823	-27.02815052	0.020526863	25.76030089
4.47391208	0.847091967	0.012994303	36.62104486
3.087935635	7.284816831	0.017639814	34.40689507
3.083406511	-5.823097691	0.00690235	20.62521864
1.581656007	-37.58477808	0.018840716	28.80085095
2.872266444	-7.254734493	0.003931418	26.25930401
2.425166595	4.047607287	0.01531099	18.0927406
3.507677066	0.467679494	0.012132545	17.36000651
2.264792725	-18.71828206	0.010073845	39.48033382
2.286799718	-15.22666419	0.007813952	43.64126436
3.151381362	1.103025018	0.008007868	33.51724955
2.566310271	-21.89737175	0.011300062	24.3724182
2.891988136	-12.26607289	0.004709291	14.97516103
2.396616907	-19.55550938	0.009535709	29.21993926
3.533188205	6.027698775	0.007722171	30.42873893
3.534458847	10.19384984	0.007887079	13.79024022
3.760727566	-9.200583977	0.002472731	15.58857483
2.29003381	-24.8933824	0.020126598	24.47526897
3.560347983	27.78388299	0.00768511	30.45254425
2.5218412	-23.73135945	0.011673663	31.66419864
2.381509595	-2.864010483	0.004753392	31.87206576
1.93733554	-42.21267991	0.010789449	25.27837039
2.588018778	-3.627692931	0.009838322	29.81073634
2.374311935	-8.651835455	0.007958499	27.76479056
2.216712945	-23.80282219	0.01436058	28.11006256
3.270735944	-8.502991748	0.00642524	19.58554999
3.104329218	-16.8051174	0.006612866	16.54197981
4.458669196	12.53183576	0.002879767	19.55585807
2.157778224	-23.46982747	0.009750956	38.49147765
2.368662074	-28.26160242	0.010208122	26.48528732
2.316873504	-38.15076279	0.01479918	29.35693246
2.226381257	-39.7849371	0.0072911	29.83376207
2.520822424	-19.48384227	0.00944917	25.49746515
2.048402693	-61.81662672	0.014673259	43.39808604
2.263012944	-6.362604756	0.008442586	46.3786199
2.762621587	19.76602687	0.003115915	31.8879579
3.823055311	17.82559037	0.01309464	31.84973359
2.386378372	2.363700411	0.009750509	20.53781433
4.831070488	-0.686356769	0.003755668	16.36604677
2.258670131	-23.7534819	0.014012327	29.58814632
2.113517239	-19.54587085	0.005468169	38.36803741
2.424257769	7.432310357	0.018888313	16.17691611
2.841463356	-45.70723136	0.025707072	29.35779621
3.338328834	-3.56116806	0.002689881	27.05311238
2.725812846	-44.03062366	0.008550606	41.26342963
2.895969509	-14.01119477	0.005631281	22.23510424
3.080656539	-11.11623006	0.013225386	19.7530733
2.863883849	-5.566725579	0.009544258	17.28262554
1.937696569	-33.56590276	0.013145401	69.53260059
2.999909024	14.67001578	0.005332797	24.58978432
3.032345831	6.147649156	0.024890576	35.95324135
2.791462047	18.603552	0.023406568	18.80533201
4.050258559	-25.81901102	0.014168736	34.57548141

log.sigma.2.5.mm.3D_glrlm_LowGrayLevelRunEmphasis	log.sigma.2.5.mm.3D_glrlm_GrayLevelNonUniformityNormalized	log.sigma.2.5.mm.3D_glrlm_RunVariance	log.sigma.2.5.mm.3D_glrlm_GrayLevelNonUniformity
0.011104866	0.052038102	0.074479484	34.3730763
0.006711866	0.064870287	0.210738316	118.0803617
0.013258438	0.058288614	0.067012674	23.858856
0.005353005	0.046670944	0.07875281	115.0538834
0.010136459	0.052097601	0.073164087	17.58355523
0.017947229	0.064488506	0.085321525	38.5174695
0.009819494	0.092702408	0.099897386	28.7555678
0.018463343	0.046163199	0.066893779	43.63553944
0.008916833	0.046136057	0.077603972	89.65063337
0.006792154	0.067176567	0.11731519	189.5542193
0.007337659	0.066597498	0.091246258	51.17200349
0.011619543	0.056793469	0.096556222	77.61842675
0.014347212	0.063227646	0.093315575	121.0068027
0.018406014	0.060113428	0.084324244	62.50429292
0.010103861	0.058977294	0.070735752	50.44281219
0.00885697	0.113107114	0.416327751	638.8679952
0.014229091	0.053605247	0.073087921	35.11375223
0.01807792	0.057028623	0.122569888	120.9665488
0.020830864	0.046665872	0.075928496	25.41190751
0.016975105	0.074825788	0.221371842	182.4451537
0.007716032	0.077211978	0.192450512	355.4124456
0.00976298	0.055637093	0.078500209	89.61363877
0.01252077	0.06567458	0.10979001	164.5630519
0.010019988	0.056331799	0.072235142	25.40315434
0.011109418	0.072732602	0.118223584	80.02989065
0.010240691	0.052203435	0.099520525	33.41909855
0.004256871	0.08450391	0.273659985	249.8921784
0.012272988	0.064901691	0.104391399	85.85292697
0.010468396	0.051832986	0.08288457	85.58304388
0.007759452	0.066929213	0.239077724	147.9849011
0.008706826	0.071812441	0.120632578	208.2883239
0.012900696	0.068705635	0.164545175	363.8199867
0.009637207	0.0505153	0.089001631	95.29420731
0.014443628	0.056839369	0.04380824	5.680683397
0.015859253	0.047032934	0.059143243	14.80554928
0.006098118	0.055534462	0.093898058	103.8699563
0.00865996	0.055629251	0.129708918	146.4495625
0.01076501	0.062527825	0.112324849	191.9601499
0.008168853	0.061588858	0.087531524	47.81120973
0.010578639	0.053604889	0.085735939	52.32591218
0.01516464	0.062803657	0.129451964	83.84102858
0.014934563	0.05309371	0.071427844	47.56475338
0.007371581	0.055643717	0.082822449	85.08514672
0.007605086	0.047980011	0.077309667	45.9180098
0.009505054	0.055647547	0.070290183	98.28501151
0.02350036	0.07822826	0.145151218	156.5314475
0.005760134	0.052490712	0.087387046	208.2900886
0.021784883	0.054957116	0.071981742	30.069217
0.013111611	0.086433095	0.077323731	10.82599373
0.018717512	0.060548506	0.079551808	28.03467903
0.00737067	0.062869144	0.142764904	195.0753768
0.019922775	0.063412148	0.095501027	48.5508314
0.004205553	0.055201267	0.140214902	190.6299113
0.01614092	0.065449743	0.089102697	35.84654278
0.013285791	0.080024013	0.257380673	449.4355223
0.010374357	0.044362133	0.056940541	36.997531
0.008141829	0.04234496	0.069415288	87.58065757
0.008217841	0.055148156	0.074129577	66.8503134
0.011907107	0.056806011	0.121134309	42.11825511
0.004950244	0.072918779	0.10804262	58.72867761
0.010078011	0.054223066	0.085990207	106.9060055
0.008170034	0.052311919	0.118371163	262.3241739
0.008561146	0.081412837	0.195416033	221.8620383
0.002712231	0.081896129	0.313908612	188.0711965
0.021107015	0.059089529	0.074147966	12.02798551
0.008034155	0.05655431	0.086157326	81.82024544
0.012004388	0.05611548	0.057358099	20.95918451
0.0050457	0.04983956	0.106714473	146.7882176
0.01129644	0.059703889	0.079270955	24.89881173
0.010275748	0.052281869	0.086544384	97.73615592
0.008459417	0.054932654	0.102213518	154.0973311
0.015170477	0.054736257	0.087219802	57.34982633
0.006986349	0.067070689	0.192559002	306.3871801
0.007182488	0.073489411	0.195095776	435.8994673
0.003114429	0.075235041	0.182514076	609.3355982
0.010152682	0.046203553	0.064036718	48.79816123
0.010686993	0.055060786	0.081290248	57.16425218
0.01562115	0.055395713	0.076765579	31.62078543
0.0076267	0.051718805	0.082605692	85.20871242
0.009949281	0.057180263	0.100192967	150.1400403
0.015073186	0.043708545	0.056494344	21.00597638
0.008646874	0.041603518	0.051210319	53.22921519
0.003250619	0.051022472	0.087190476	132.861231
0.013231359	0.06453939	0.065074162	8.823331911
0.010408142	0.061941968	0.107557402	192.7765345
0.004102348	0.078192963	0.193249288	374.9025743
0.014778007	0.054343588	0.079989976	39.49580472
0.005681618	0.049952449	0.071319448	73.00373129
0.019499244	0.072634248	0.093737444	12.28036793
0.026440539	0.081851106	0.055512341	8.201614828
0.002862993	0.057182434	0.176371885	202.9005857
0.008855902	0.047095472	0.069098206	31.34212766
0.005954357	0.062464384	0.210740343	56.43942148
0.014092226	0.064683792	0.15937354	237.019777
0.010314144	0.067585356	0.155007289	351.3232088
0.013407923	0.037800187	0.049521787	9.150480559
0.005528239	0.059103312	0.084749177	66.8957009
0.025540981	0.056191082	0.036808975	6.657024379
0.024752952	0.070639554	0.09256879	24.68567509
0.01474394	0.056128964	0.10353869	41.66841206

log.sigma.2.5.mm.3D_glrlm_LongRunEmphasis	log.sigma.2.5.mm.3D_glrlm_ShortRunHighGrayLevelEmphasis	log.sigma.2.5.mm.3D_glrlm_RunLengthNonUniformity	log.sigma.2.5.mm.3D_glrlm_ShortRunEmphasis
1.218547575	219.9684842	581.1164939	0.950881763
1.518831903	231.7042957	1454.468744	0.912685108
1.193463446	184.0495991	366.3314033	0.957138138
1.222680787	351.9455386	2174.707281	0.951868571
1.207434089	296.0077231	300.5536825	0.955053734
1.245885025	137.2162041	519.8367846	0.946411109
1.284296577	202.4589916	265.5242558	0.939719989
1.191678748	228.7854518	846.5875587	0.957703514
1.223120824	270.2348427	1708.446341	0.950827926
1.324461309	228.2538718	2375.770784	0.933804144
1.253302041	247.9539284	670.7560543	0.947201362
1.275109184	189.3693093	1173.943356	0.941249256
1.270605097	151.0880248	1644.107433	0.941223942
1.242161488	152.0262366	906.0446786	0.947072806
1.206597807	211.7149089	757.8394368	0.953680398
1.950482462	154.5494031	4020.3295	0.867498905
1.210793381	208.1001323	581.3860882	0.953795599
1.333977403	147.6981126	1784.407994	0.933133694
1.220068391	224.6776978	479.9419908	0.95127332
1.548261687	112.9088461	1922.543664	0.907378869
1.48425812	188.5821665	3699.143656	0.915272553
1.221121171	209.6256879	1424.312645	0.952588446
1.299713057	157.788034	2141.508616	0.939397501
1.211653459	332.4684841	398.5669061	0.95260101
1.31936477	158.3375728	934.2208159	0.936592973
1.276266172	229.0364132	551.8142553	0.942845707
1.655500481	286.8195974	2257.246059	0.89517593
1.282867482	169.058535	1142.697244	0.94306043
1.229320907	242.7586095	1460.473522	0.952301128
1.567467384	207.3334296	1749.629953	0.909119334
1.324544065	191.5227205	2458.195175	0.936004769
1.423218873	150.0905857	4340.97359	0.922779828
1.251925508	233.6503425	1638.450027	0.945862757
1.128087533	416.697155	92.56990359	0.970594548
1.174104539	261.0134298	284.4952106	0.960667551
1.262167679	270.8899	1619.670357	0.944624233
1.330658458	216.2531452	2247.909194	0.938502081
1.308718346	193.7460241	2609.766561	0.937158207
1.252149012	263.6347317	678.3233983	0.946078807
1.241841095	211.8447065	853.2740253	0.94834886
1.352526142	158.630288	1115.809559	0.930464388
1.203922312	214.4295499	797.1997745	0.955322909
1.238429365	263.6492225	1336.745256	0.948045211
1.226114229	287.9141767	839.4412145	0.949597502
1.202019288	241.3456986	1572.201076	0.955443524
1.390521987	90.21165144	1650.413983	0.924867565
1.244548797	295.8630565	3465.219366	0.947974416
1.209501305	151.9248019	485.1155515	0.953458793
1.225916611	473.9049562	110.424215	0.950446348
1.233652921	240.0076726	404.0002504	0.947776549
1.362880621	212.7790193	2605.462331	0.932501881
1.266188566	162.9644678	664.1222803	0.944669467
1.367799962	337.6590488	2883.859779	0.930129734
1.254580573	148.3059855	474.3468529	0.944857219
1.621326905	138.4298883	4363.427201	0.901074086
1.168465138	278.9038761	753.1008453	0.961083445
1.196628078	350.4501064	1854.543318	0.957501111
1.214174532	355.3394303	1071.638793	0.952551668
1.328115543	200.6567648	626.7211739	0.935207531
1.307538192	320.2402113	680.7091534	0.934996408
1.24267075	210.3595445	1723.354675	0.948160916
1.314548762	235.3029905	4275.201232	0.938369475
1.494873462	168.3152602	2192.229013	0.914113149
1.73962469	450.6667898	1728.474869	0.888762973
1.213616822	175.8270008	180.4849298	0.953377486
1.24156914	263.0995132	1268.205727	0.949039985
1.172874175	263.0107709	336.7616795	0.959858678
1.28675994	320.5456149	2538.7517	0.942746299
1.226170936	208.9454633	367.8066713	0.951167533
1.2459105	226.6966708	1629.80732	0.947186535
1.279550719	220.158858	2421.826522	0.943114731
1.250004944	179.2040045	910.1549878	0.945896103
1.480465902	206.3875028	3692.995269	0.916823713
1.482608105	194.5752557	4789.829371	0.916815452
1.471457805	386.0447476	6496.965591	0.914639703
1.182168632	300.9202025	951.8214984	0.95991968
1.241022925	222.608839	901.9901981	0.945810814
1.221253894	169.284555	503.7276062	0.951652162
1.231859321	271.4495879	1452.142101	0.950824121
1.276790995	204.9619283	2262.877489	0.94271731
1.158088292	264.7242039	439.5927097	0.965582611
1.150388565	367.0408259	1168.690178	0.965435827
1.23910942	462.7234525	2289.597492	0.950393083
1.19241337	379.3571484	122.0987137	0.95640644
1.297276786	171.5279009	2660.786085	0.939281744
1.501766342	297.8100619	3792.341931	0.909120846
1.227518207	183.5354244	639.6426379	0.950827276
1.200876902	371.5776355	1306.65614	0.9567827
1.27395308	144.8756768	145.6612749	0.940715744
1.156684212	262.4122673	91.69354986	0.965725037
1.436733864	489.9379724	2915.04459	0.923660146
1.195699074	283.0300745	595.512919	0.957410501
1.496531218	286.2221469	736.0980437	0.920351827
1.409100221	154.6251616	3018.429498	0.924746695
1.40608106	159.0582104	4268.644128	0.923696479
1.143514498	389.4741179	222.6190986	0.967576105
1.240360519	331.5079091	988.5125693	0.948120582
1.115935814	303.6590438	109.814429	0.971016047
1.271989658	137.6476589	299.2824354	0.939989006
1.287656276	234.6689616	636.3160849	0.940459257

log.sigma.2.5.mm.3D_glrlm_LongRunHighGrayLevelEmphasis	log.sigma.2.5.mm.3D_glrlm_RunPercentage	log.sigma.2.5.mm.3D_glrlm_LongRunLowGrayLevelEmphasis	log.sigma.2.5.mm.3D_glrlm_RunEntropy
268.285298	0.93549793	0.013170678	4.762412057
371.5639266	0.875893485	0.009448988	4.764990908
215.8363767	0.943105282	0.015709083	4.574508423
445.951775	0.935542257	0.006338397	4.930981037
372.6244007	0.940004285	0.011151104	4.783180241
171.3037595	0.928938868	0.022293242	4.446504346
280.114425	0.920337822	0.011441944	4.158654121
279.4526694	0.943344081	0.021590964	4.88547927
340.6971713	0.934657935	0.010673996	4.942458764
306.6298025	0.910786115	0.008863925	4.620727201
326.5428095	0.929029858	0.008831303	4.428425818
242.598299	0.92219454	0.014387904	4.726970892
198.9767119	0.92274312	0.017921987	4.57617523
193.3475173	0.930026146	0.022738701	4.562055924
265.3365043	0.938782403	0.011506865	4.588400476
321.1447362	0.811019699	0.015859968	4.475290721
259.5932645	0.93850562	0.016673843	4.676232524
195.4019534	0.909552711	0.024297746	4.749754862
280.0156512	0.935765266	0.024747898	4.859646983
179.4112079	0.870101337	0.025160348	4.534077799
288.0117808	0.880919425	0.010948949	4.536639382
260.2495714	0.936404293	0.011812159	4.696299761
204.064415	0.917528096	0.016236713	4.572634539
425.8627716	0.937470014	0.010953663	4.676362461
207.7622113	0.913685152	0.014423867	4.454190074
293.7142218	0.922411882	0.01261844	4.819946026
507.533966	0.852545608	0.006682825	4.57241302
211.6359459	0.922808524	0.015440692	4.588701021
318.3252151	0.935410765	0.012583324	4.744543591
346.3074255	0.869303395	0.011058141	4.761020441
253.4939616	0.912284947	0.011435657	4.504738462
215.0801354	0.892774679	0.017887719	4.616197289
302.3475476	0.927920088	0.011868896	4.829701749
491.9224794	0.960798817	0.014888446	4.524934008
317.6288144	0.948100093	0.018068902	4.782183909
358.1327989	0.925932978	0.007606179	4.666155017
287.4793952	0.913862179	0.011403003	4.797133824
260.9247199	0.915256181	0.013376587	4.609985711
349.1816432	0.929525564	0.00956638	4.619571185
267.704095	0.930556574	0.013001848	4.721543994
219.9925598	0.905542219	0.019564733	4.655391144
263.0844945	0.940027444	0.017298988	4.733451817
343.5619515	0.931181486	0.008875428	4.693007301
360.9846861	0.933658537	0.009274134	4.880151325
303.7488292	0.940444008	0.011427726	4.646894478
122.7189203	0.897907748	0.033268636	4.344208516
379.4355814	0.930163367	0.006977157	4.810870431
185.0951071	0.938514316	0.02737245	4.592808456
615.3011713	0.934557979	0.013634301	4.279717229
311.6483146	0.931434762	0.02342111	4.720940702
297.3080701	0.906160137	0.00979406	4.654684656
220.2396532	0.925588317	0.024973135	4.491841114
479.3785723	0.903730397	0.005618007	4.835605113
189.2067601	0.926721333	0.020058395	4.455475078
233.1195806	0.859420512	0.020120775	4.626867133
331.8258962	0.948805461	0.011673998	4.900950312
432.9137679	0.943140763	0.009599044	5.014523709
461.7220792	0.93741448	0.009123094	4.788715008
272.1558233	0.910791911	0.014787301	4.728284313
437.0635947	0.91417096	0.006119107	4.356451009
263.0643834	0.930446147	0.012669584	4.739013732
318.3997625	0.915311596	0.01037434	4.890875849
261.4946599	0.88085636	0.012337057	4.453489635
872.6417654	0.84001352	0.00428858	4.60635087
215.6028749	0.937965261	0.025441363	4.488809767
334.4535092	0.931348754	0.009639213	4.800475011
319.1354811	0.947871925	0.013414807	4.665330034
420.0441822	0.921224225	0.006511571	4.871064008
260.0738036	0.934977578	0.013750191	4.508721776
288.0752882	0.929503117	0.012271986	4.801888882
283.1894098	0.922697368	0.010903242	4.753207764
226.6464664	0.927982558	0.018909007	4.708402005
315.326625	0.883528816	0.010152284	4.672223721
299.4681966	0.88293169	0.010384215	4.567309667
609.2661338	0.881967488	0.004395658	4.671984816
368.3894483	0.946374414	0.0119369	4.867552935
286.4790342	0.929481441	0.012828097	4.707339168
203.745655	0.935687264	0.01952576	4.632259259
344.8324915	0.934109541	0.009207426	4.763183856
266.8309706	0.92259041	0.012361709	4.707708887
304.3598765	0.953449328	0.016879451	4.864240821
434.4109348	0.954110021	0.009522811	4.99550669
598.6965845	0.932527	0.003888335	4.861801283
481.8520455	0.94270557	0.01381731	4.497857624
226.8192841	0.917814123	0.013644716	4.588188748
471.041412	0.875428443	0.006009307	4.574959338
221.1549687	0.934150682	0.01829141	4.682969602
467.6570882	0.941613799	0.006652463	4.821805276
201.8607091	0.923497268	0.022130865	4.24834729
322.6676221	0.953846154	0.02942508	4.139441123
747.969903	0.892230285	0.003835323	4.891500835
335.8978667	0.942580083	0.010284141	4.888047503
454.1219055	0.883411109	0.007886929	4.716929166
227.4343698	0.895805906	0.018501999	4.669933497
228.4641789	0.8952145	0.014365895	4.58800168
452.6290906	0.956825783	0.014545524	5.049406016
436.9296204	0.930731275	0.006437542	4.672909879
352.3457336	0.963101939	0.028142602	4.555282912
182.4461931	0.922062107	0.030645195	4.429884836
302.3396664	0.91983605	0.017372479	4.839281716

log.sigma.2.5.mm.3D_glrlm_HighGrayLevelRunEmphasis	log.sigma.2.5.mm.3D_glrlm_RunLengthNonUniformityNormalized	log.sigma.2.5.mm.3D_glszm_GrayLevelVariance
228.8585094	0.87920102	36.05002303
251.8125539	0.798116636	31.95517879
189.8381171	0.894032825	31.28539961
368.790909	0.881564282	40.81716071
309.4240358	0.889465955	44.58725762
143.513552	0.869477912	19.87745473
215.8029947	0.854705414	21.15196078
238.0012887	0.895123282	41.21945409
283.1139735	0.878851195	40.26532675
241.7540855	0.841339601	29.24638228
261.493532	0.871647835	23.04129225
198.865411	0.857870338	32.87929701
159.7149201	0.857881997	24.33357547
159.5402115	0.870637375	24.22410165
221.5657646	0.885575525	29.3270252
175.2747643	0.710959138	25.60837979
217.3472241	0.886461462	29.84713979
155.8573364	0.84023892	28.49491702
234.6552753	0.880315098	38.39460225
122.694514	0.787117238	20.21358725
203.4798732	0.80286728	24.8777732
218.579359	0.883472996	29.85337218
165.7269132	0.853855034	24.53662498
349.4099795	0.883205301	34.97721609
166.6705762	0.848018891	24.49878688
240.0758763	0.86142153	36.19080753
317.1932019	0.762468365	27.86431179
176.3602916	0.862590216	30.04728
255.7691124	0.883308354	32.67423289
226.1509146	0.790637859	33.55518967
201.9071444	0.846704229	22.77076442
160.3190077	0.818625754	23.04634245
245.9484161	0.867873645	35.94013079
430.5100552	0.925887508	46.07638889
271.3093369	0.902630282	39.30564456
286.0737155	0.865183241	25.43797067
227.5534613	0.852804121	35.12699983
205.2850079	0.849093903	25.97325086
278.822038	0.87086256	29.80114378
221.6953747	0.873582254	32.2036648
168.7886435	0.834734939	28.77595574
223.3066918	0.889486718	32.80804769
277.8179925	0.873203096	30.40019312
301.376029	0.876380801	36.37155177
252.4586127	0.889713761	28.16753172
95.64304868	0.823172888	16.29277967
310.6868802	0.872689727	35.64349268
158.0586019	0.885597322	25.93351652
499.2964939	0.879993707	56.24970273
253.001814	0.872055256	39.59516886
226.117432	0.838940518	26.98986631
172.5345728	0.866175826	23.48249923
360.3103361	0.834043408	33.63162013
155.7425446	0.865559247	19.21293011
151.6002335	0.775558326	25.57518068
288.8639791	0.902627474	44.40945619
365.0699049	0.895339565	44.66934872
374.4945211	0.883218954	40.51214586
212.4104227	0.84478977	31.59428916
341.1299914	0.844046797	18.50418992
219.9383971	0.87335643	31.03705155
249.1229412	0.851747051	39.61991582
182.2355239	0.802188268	21.49224332
505.9384303	0.751252087	24.48964897
183.0611394	0.885574205	26.37006315
275.6611306	0.875716993	41.53202638
273.5116791	0.900507647	35.57111111
337.3116381	0.861349877	33.16700245
218.350465	0.880702582	22.98907353
237.6246976	0.871088416	35.15229505
231.0348812	0.862327693	30.10774998
187.7078448	0.86803469	29.91858558
222.5520756	0.806755025	26.51867114
209.9744161	0.806346663	23.09676135
420.3844781	0.801379799	33.48389795
313.2107018	0.900453108	39.55184691
234.3149792	0.867788658	28.57904378
175.5515891	0.881513581	28.63630008
284.2779332	0.879944268	31.64462059
215.7592794	0.861103759	30.10545349
271.9500435	0.914037821	44.12167126
379.6999758	0.913080085	49.86646584
486.0526047	0.878591557	39.44151841
398.0771849	0.892274087	42.95053254
181.0201194	0.853861899	22.02509366
324.6791831	0.789942647	27.69863014
190.2262461	0.879402308	28.68474048
388.5153253	0.893339071	38.39729888
154.6571144	0.858611672	19.74502394
272.935375	0.914583113	33.27469136
528.4779502	0.820629854	39.12869029
292.7335853	0.894424993	48.68089844
309.574347	0.814207185	31.20975449
165.9313051	0.822651998	26.84466714
170.2227081	0.820174299	23.20188618
400.9618108	0.918779372	70.33134342
350.2445372	0.872887702	32.99788682
313.3963817	0.92638205	39.89641584
145.8013991	0.855059221	23.08956355
246.4689236	0.85628325	50.65063075

log.sigma.2.5.mm.3D_glszm_SmallAreaHighGrayLevelEmphasis	log.sigma.2.5.mm.3D_glszm_GrayLevelNonUniformityNormalized	log.sigma.2.5.mm.3D_glszm_SizeZoneNonUniformityNormalized
186.8759702	0.047554941	0.382718674
210.2744561	0.04938053	0.402116847
180.6660649	0.052732289	0.482778569
246.0044566	0.043414874	0.375608062
202.8266888	0.04360316	0.381142916
102.5039736	0.063319221	0.347747973
104.3762913	0.063052672	0.280084583
174.2803971	0.043548293	0.395845058
205.3591691	0.044090719	0.377961976
199.8178372	0.052026826	0.381866418
143.5129331	0.061224934	0.295232494
159.9186981	0.048365116	0.39653014
99.3520863	0.057249059	0.300886872
114.1506943	0.056042338	0.345805993
144.1192526	0.051098501	0.364379425
143.8359635	0.059124535	0.35651093
149.4852518	0.051976808	0.375456538
136.0549104	0.051968766	0.373150764
157.134892	0.045897543	0.337609329
99.68811206	0.06164023	0.329334796
166.0476467	0.056245094	0.370713078
156.9239814	0.05158111	0.340074265
143.3840685	0.056482395	0.399465355
231.8096112	0.050195799	0.387815949
143.2278492	0.057953359	0.407444565
208.1881862	0.047459667	0.374818338
241.5106612	0.054333673	0.341616553
166.2206386	0.050897542	0.442344839
162.8855318	0.048359111	0.370816
194.1125323	0.048600677	0.395721508
171.7201634	0.059185519	0.390224747
124.8352705	0.058167553	0.329946382
167.1139308	0.04667545	0.351683102
266.322838	0.050555556	0.422777778
181.9899659	0.045923816	0.406906372
169.007378	0.054292179	0.318775598
199.8290254	0.046507072	0.414244612
148.805714	0.054532883	0.360052235
143.9614692	0.052615943	0.303568208
174.1145276	0.049004821	0.394928947
135.7371689	0.051777361	0.387627807
157.4618001	0.050144157	0.353948782
177.4608069	0.050966173	0.333892617
218.5521465	0.046325779	0.370873941
159.9952882	0.051668829	0.359804522
80.44964991	0.069571225	0.344194342
221.8288497	0.046015504	0.381496232
118.0894265	0.055183403	0.358090531
270.9844349	0.049346017	0.382282996
173.3004865	0.049280686	0.378175696
176.1423944	0.053770845	0.345898215
104.2944603	0.059526008	0.32981225
260.0631879	0.048618483	0.340992414
112.2477143	0.064490282	0.338110391
124.0253659	0.056019894	0.343469967
215.6908604	0.04182961	0.424059704
259.1227512	0.04136467	0.406484604
218.4279368	0.045824021	0.369014471
170.5876127	0.049704184	0.372137788
188.5413818	0.073752603	0.263273636
162.8862265	0.050167003	0.357966287
187.8007332	0.045118375	0.367472445
132.863517	0.060942941	0.320373668
322.3931875	0.057811515	0.349202031
129.6734448	0.058346687	0.375247431
217.6416538	0.044034583	0.374345351
178.5221778	0.048580247	0.344320988
247.6552183	0.048113284	0.376481601
153.0314072	0.060431153	0.378614191
174.8651765	0.046666409	0.386431079
183.0065961	0.049608064	0.373265091
137.2795807	0.052607834	0.356171315
178.7808897	0.054207961	0.350711808
161.9086635	0.058508156	0.34987346
284.7270968	0.049483249	0.35896788
200.8970196	0.043914747	0.392382841
159.6585571	0.052283654	0.346789479
144.22774	0.053167274	0.345213319
196.2767529	0.049670849	0.364989133
164.5424399	0.051025391	0.375822067
218.2117372	0.042412764	0.416637971
265.1885227	0.039589222	0.433177172
329.3354695	0.044027609	0.383801771
245.2099961	0.050887574	0.386035503
128.7828589	0.058621231	0.326273608
233.572316	0.05574205	0.335007147
157.5187913	0.054567474	0.41700692
235.1412588	0.045432526	0.363927336
82.75754531	0.068783069	0.302595112
154.1488182	0.060356653	0.414266118
345.4858162	0.045777145	0.359327954
255.4531602	0.041757813	0.433222656
204.3792938	0.04991016	0.358786652
121.8577143	0.053495546	0.340692984
135.8112715	0.057703171	0.347232556
317.7445885	0.035587024	0.498542274
203.0302358	0.048248581	0.35097892
207.2016743	0.054231563	0.428410584
85.70801487	0.06310821	0.278014102
230.5226802	0.040495562	0.467085799

log.sigma.2.5.mm.3D_glszm_SizeZoneNonUniformity	log.sigma.2.5.mm.3D_glszm_GrayLevelNonUniformity	log.sigma.2.5.mm.3D_glszm_LargeAreaEmphasis	log.sigma.2.5.mm.3D_glszm_ZoneVariance
111.371134	13.83848797	15.91752577	10.03148286
236.0425894	28.98637138	201.5417376	189.021955
102.3490566	11.17924528	10.21698113	6.026076896
428.5687993	49.5363716	16.00613497	10.67290943
65.1754386	7.456140351	9.16374269	4.756198488
87.98023715	16.01976285	20.13833992	13.67910763
28.56862745	6.431372549	32.32352941	21.40763168
179.7136564	19.77092511	11.92951542	7.058452522
324.2913753	37.82983683	14.77972028	8.908418505
379.1933535	51.66263847	98.71097684	88.97758225
89.45544554	18.55115512	16.98019802	9.530721389
212.9366853	25.97206704	35.30726257	27.69089604
211.5234708	40.24608819	29.11237553	20.4086166
145.584323	23.59382423	20.96912114	13.91700566
142.8367347	20.03061224	13.06887755	7.667996408
412.4831461	68.40708729	3859.190147	3823.13827
111.1351351	15.38513514	14.16216216	8.601488313
271.2806052	37.78129298	64.52269601	54.23333819
82.71428571	11.24489796	12.79183673	7.148788005
204.5169082	38.27858293	232.5136876	212.1693751
539.7582418	81.89285714	492.6387363	479.7656231
234.9913169	35.64254703	17.17800289	10.98215426
373.8995726	52.86752137	58.40918803	49.89601025
82.21698113	10.64150943	12.58962264	7.441860983
180.0904977	25.61538462	53.5520362	46.13195881
97.82758621	12.38697318	19.79310345	12.72280207
263.7279793	41.94559585	527.642487	507.4856439
250.8095238	28.85890653	35.39506173	29.00763012
278.112	36.26933333	14.46266667	8.924488889
279.7751061	34.36067893	254.9222065	241.9947744
420.272052	63.74280409	106.461467	97.74882945
561.5687427	99.00117509	102.0070505	89.8678447
268.3342071	35.61336828	24.6802097	17.58074374
25.36666667	3.033333333	4.366666667	1.362222222
64.69811321	7.301886792	8.27672956	3.916775444
221.8678161	37.78735632	26.54022989	18.11689127
423.7722385	47.57673509	56.40469208	48.47906365
389.2164662	58.95004625	73.04902868	63.42238873
93.19543974	16.15309446	18.96091205	11.56322083
168.6346604	20.92505855	20.98594848	14.95069901
181.7974414	24.28358209	78.15778252	68.2802315
141.2255639	20.0075188	17.55137845	11.84658388
199	30.37583893	26.97315436	19.38294446
155.3961814	19.41050119	16.25059666	10.26620947
293.6004902	42.16176471	12.20588235	6.909115484
212.3679092	42.92544571	127.7666126	114.7271395
643.9656398	77.67417062	30.7535545	24.36655595
88.80645161	13.68548387	12.81854839	7.292257414
22.17241379	2.862068966	16.13793103	10.80023781
74.87878788	9.757575758	22.41919192	16.11858484
395.0157618	61.40630473	58.36252189	49.37302671
93.99649123	16.96491228	26.50175439	18.08157587
408.1679198	58.19632414	62.23308271	52.04328002
70.66507177	13.4784689	23.15311005	15.15693322
462.654046	75.45879733	1099.871566	1076.420664
187.4343891	18.48868778	9.57239819	5.617519093
416.2402344	42.35742188	12.27050781	7.68405056
208.8621908	25.93639576	16.57420495	11.35548265
108.2920962	14.46391753	35.46391753	27.63930516
64.50204082	18.06938776	55.96326531	43.0326364
292.1004902	40.93627451	34.30514706	27.56170553
711.4266529	87.34917355	66.52272727	58.51639979
241.8821192	46.01192053	181.3456954	164.5953774
195.2039356	32.31663685	594.6243292	570.7561228
38.65048544	6.009708738	9.427184466	4.988594589
227.6019737	26.77302632	31.94243421	25.41810671
61.97777778	8.744444444	9.4	4.608765432
427.6830986	54.65669014	35.26320423	27.34314171
73.07253886	11.66321244	14.42487047	9.08470026
311.0770186	37.56645963	26.59503106	20.35434435
409.0985401	54.37043796	32.45620438	24.76266716
144.2493827	21.30617284	29.51358025	21.74256363
481.5273125	74.42753095	170.5841224	156.4162728
585.688172	97.94265233	325.9139785	309.8182563
898.4966041	123.8565721	828.2357171	814.7844356
201.2923977	22.52826511	13.66081871	8.928285626
144.2644231	21.75	20.57451923	13.36477788
85.61290323	13.18548387	17.16129032	11.11127732
260.6022409	35.46498599	25.16526611	19.06145988
384.8417969	52.25	42.32617188	34.60168076
109.9924242	11.1969697	7.590909091	3.946280992
309.7216783	28.30629371	6.351048951	2.833456893
447.5128645	51.33619211	26.03945111	20.30576747
25.09230769	3.307692308	10.16923077	5.192899408
356.9433272	64.13162706	42.71572212	33.1079889
440.1993912	73.24505327	413.2404871	395.8730334
141.7823529	18.55294118	25.24705882	20.01103806
247.4705882	30.89411765	11.69411765	6.484982699
19.06349206	4.333333333	17.6031746	9.16553288
22.37037037	3.259259259	6.240740741	2.459876543
431.5528726	54.97835137	193.7843464	182.8299551
138.63125	13.3625	14.3	9.432460938
128.0868347	17.81792717	69.34453782	61.14922832
410.1943522	64.40863787	156.3754153	144.8357386
529.1824147	87.93963255	212.9566929	198.4427928
73.28571429	5.231292517	5.149659864	2.187514462
162.5032397	22.33909287	25.13606911	18.23835536
31.2739726	3.95890411	4.123287671	1.284293488
36.41984733	8.267175573	17.29007634	8.919876464
145.7307692	12.63461538	37.83012821	31.13994699

log.sigma.2.5.mm.3D_glszm_ZonePercentage	log.sigma.2.5.mm.3D_glszm_LargeAreaLowGrayLevelEmphasis	log.sigma.2.5.mm.3D_glszm_LargeAreaHighGrayLevelEmphasis	log.sigma.2.5.mm.3D_glszm_HighGrayLevelZoneEmphasis
0.412181303	0.173706076	2420.831615	277.0996564
0.282619162	0.949580492	45994.83135	301.1499148
0.488479263	0.132174955	1356.226415	230.3349057
0.433017078	0.066386981	5393.890447	382.0464505
0.47632312	0.053081998	2698.918129	315.3918129
0.393468118	0.356116711	1851.422925	163.972332
0.302670623	0.170074189	7474.215686	203.4019608
0.453093812	0.196453297	2232.343612	259.4647577
0.412698413	0.116658387	3550.224942	308.4918415
0.320529374	0.631656992	17051.39577	296.12286
0.366384522	0.10015772	4331.841584	262.4224422
0.362348178	0.342141343	4951.964618	237.8435754
0.338958534	0.380125145	3670.853485	173.2916074
0.376565295	0.40541397	2462.503563	179.3182898
0.430296378	0.093054926	2678.280612	229.7806122
0.166546711	26.1776386	575819.9283	221.3422645
0.424068768	0.195594506	2553.506757	229.9358108
0.311749571	1.291504399	5827.072902	202.4662999
0.420962199	0.249678457	2167.812245	255.2653061
0.221706533	3.270285953	22590.1256	160.9855072
0.278713629	3.104179058	81035.76168	251.6325549
0.401744186	0.161044989	2918.767004	247.3198263
0.3427316	0.706716982	5980.803419	208.4925214
0.440748441	0.062050083	4519.471698	353.5660377
0.367109635	0.531490334	6225.71267	204.9208145
0.376080692	0.181985434	3502.689655	298.2337165
0.222735141	1.850811739	153752.8122	379.3782383
0.395673412	0.358199972	4197.961199	224.7654321
0.424929178	0.113772813	4100.404	254.168
0.278127459	1.306187482	54108.66195	276.950495
0.338785782	0.814169092	15196.64345	252.1652739
0.287015177	1.168887926	11235.75969	198.6010576
0.375307427	0.199707374	5287.547837	266.9960682
0.576923077	0.032575428	2016.083333	410.25
0.478915663	0.119759071	2070.798742	275.9622642
0.344554455	0.168565958	6366.45977	294.8505747
0.355208333	0.529803317	7933.397849	282.1974585
0.322301729	0.550218163	12366.21647	235.2719704
0.367664671	0.099588339	5367.677524	272.4299674
0.407054337	0.186749119	3954.920375	250.440281
0.318181818	0.727454047	10902.56716	198.108742
0.418677859	0.21476117	2774.586466	245.7192982
0.362971985	0.157530064	7721.097315	285.488255
0.408780488	0.11052831	4364.059666	333.7899761
0.434504792	0.095147238	2973.463235	256.7107843
0.276929982	3.017739764	6735.440843	128.8865478
0.395686826	0.141549177	8212.799171	336.1563981
0.425385935	0.376384605	1403.758065	181.0766129
0.432835821	0.05811281	8127.448276	475.7931034
0.398390342	0.288427716	6029.929293	260.8232323
0.333528037	0.350322597	11226.95009	266.290718
0.344619105	0.607302722	3950.8	178.4631579
0.313268778	0.232979417	19455.76859	410.2898914
0.353637902	0.360318111	2899.334928	179.2535885
0.206500077	9.408620652	141992.8344	192.2093541
0.502844141	0.076857504	2194.196833	310.1289593
0.466940264	0.080061469	4290.834961	380.9833984
0.437741686	0.064283051	6844.791519	355.6060071
0.357493857	0.279330379	6143.938144	252.2852234
0.278093076	0.240651817	14756.38776	367.355102
0.385087305	0.410089016	4100.066176	255.1188725
0.353413655	0.402736397	13536.8311	283.018595
0.24433657	1.315792413	27648.10199	216.3072848
0.204686928	1.230400618	295212.7996	531.1019678
0.474654378	0.1668564	1366.805825	200.4563107
0.391500322	0.167545437	7765.912829	317.2121711
0.456852792	0.08120426	2515.838889	285.2111111
0.355333125	0.18802352	9082.935739	384.1355634
0.432735426	0.199086551	2043.388601	239.6735751
0.400298359	0.227806165	4469.198758	265.5130435
0.360526316	0.312648441	5019.27281	275.6760949
0.358724535	0.471432216	3384.94321	218.2518519
0.265673375	1.056649332	32232.34086	277.1631464
0.249255509	2.081870696	55018.13321	258.3178017
0.272657952	2.062047807	335826.5461	451.4846185
0.459677419	0.113804523	3607.448343	318.0448343
0.372426141	0.18542708	3947.331731	253.0745192
0.406557377	0.337981737	1701.185484	216.9233871
0.404761905	0.163084977	5389.239496	305.7464986
0.359803233	0.361865525	6642.31543	247.7832031
0.523809524	0.090904196	1539.291667	306.9545455
0.533184191	0.040800665	2259.798601	387.2951049
0.417621777	0.063969129	12241.37993	503.7590051
0.448275862	0.050026682	4296.476923	382.0769231
0.322618697	0.477315194	5355.920475	210.547532
0.239956172	1.565258875	112732.8858	376.1430746
0.437017995	0.416754968	2318.214706	228.1588235
0.43814433	0.060143753	4355.725	384.0838235
0.344262295	0.184675825	2952.380952	146.3968254
0.514285714	0.105691235	1911.888889	246.7592593
0.302138365	0.399953751	97567.30891	555.3755204
0.45325779	0.118085242	2651.728125	355.5875
0.349315068	0.266455223	19572.73389	326.2408964
0.294376528	1.31548821	22971.49252	195.9526578
0.262487082	1.781476464	30554.54462	213.1496063
0.581027668	0.044750539	2094.360544	413.2108844
0.380756579	0.087414996	8899.090713	343.1533477
0.593495935	0.082489378	1342.479452	306.9726027
0.345646438	0.302899939	2343.396947	155.0763359
0.3866171	0.266361247	7021.288462	306.7532051

log.sigma.2.5.mm.3D_glszm_SmallAreaEmphasis	log.sigma.2.5.mm.3D_glszm_LowGrayLevelZoneEmphasis	log.sigma.2.5.mm.3D_glszm_ZoneEntropy	log.sigma.2.5.mm.3D_glszm_SmallAreaLowGrayLevelEmphasis
0.641453069	0.013390952	6.132307088	0.010567062
0.658818121	0.007884257	6.20889269	0.005572889
0.721032236	0.014380372	5.719962831	0.011269603
0.63476103	0.006364649	6.545304696	0.004625495
0.636600593	0.014657641	6.038899448	0.012268532
0.60773856	0.019981471	5.790675698	0.012831666
0.539039781	0.017988467	5.783493632	0.014784234
0.652913564	0.020302465	6.261279362	0.013354814
0.638586875	0.009493995	6.531677618	0.00573513
0.641048688	0.007534095	6.324459286	0.005385231
0.553369277	0.009823054	6.084045809	0.007277416
0.653968249	0.014321813	6.217830711	0.010846957
0.564724084	0.01631399	6.464021074	0.009371667
0.607803467	0.018939343	6.161068208	0.009977563
0.624601757	0.013716758	6.153239085	0.010917228
0.619640085	0.011163737	6.431184943	0.007595559
0.63337336	0.018220686	6.041379856	0.014594201
0.634277183	0.018170731	6.326357694	0.013034913
0.599376016	0.026180204	6.214794038	0.018167494
0.594624729	0.019211214	6.24565332	0.012524221
0.630736214	0.009078992	6.299566854	0.006448231
0.599474685	0.010587985	6.364349444	0.006980901
0.657133537	0.013157913	6.174293577	0.00909454
0.645793448	0.015447249	5.956523535	0.012975703
0.661203975	0.012172474	5.939711694	0.008555235
0.635546013	0.011773365	6.088816407	0.008403829
0.605867671	0.005264353	6.460622393	0.003745891
0.691052183	0.013763207	5.994342981	0.01021923
0.631779627	0.011412853	6.309411271	0.007672062
0.651678127	0.009060034	6.287669917	0.006050817
0.647813926	0.008807673	6.134313183	0.005482353
0.594453064	0.01315949	6.509584449	0.007609307
0.61481889	0.010265198	6.523859697	0.006172975
0.670662037	0.021443791	5.277316637	0.019808191
0.661850932	0.018696782	5.794032566	0.014242858
0.582366921	0.006967819	6.417608005	0.004669039
0.669302232	0.00875027	6.360943343	0.006131927
0.620518242	0.013992367	6.342628313	0.009454182
0.560979854	0.011890857	6.298068537	0.009252258
0.652622961	0.011593909	6.146240417	0.00800602
0.646920149	0.020164852	6.196192298	0.015005411
0.610790809	0.019671209	6.218906872	0.014935224
0.5958597	0.008793405	6.361892922	0.005709945
0.630743406	0.008103458	6.205104012	0.005537373
0.620580039	0.00960818	6.339550905	0.005618024
0.608004631	0.021796046	6.00569332	0.014036068
0.639709287	0.006757871	6.539805871	0.004831437
0.620013654	0.019959118	5.957680975	0.012917457
0.627936089	0.025373602	5.25419565	0.024291021
0.635792714	0.018305262	5.936939396	0.010835716
0.610018907	0.007759768	6.509312261	0.004866011
0.593125024	0.020081528	5.924294657	0.012655578
0.603264511	0.004617148	6.599735748	0.003062468
0.597594063	0.018235428	5.831638019	0.011736175
0.60661665	0.01491734	6.50243204	0.009180767
0.675602967	0.012605614	6.214681498	0.009840056
0.662208556	0.008201352	6.48871724	0.004919206
0.627908225	0.012175419	6.388112736	0.009939286
0.632985047	0.014497639	6.140472988	0.009583571
0.52485884	0.008031705	6.071284937	0.006531281
0.618985725	0.009867619	6.388369223	0.005785658
0.628215391	0.009114946	6.669011905	0.006012603
0.584881931	0.010093597	6.372186814	0.00669068
0.611505856	0.00419752	6.24007885	0.003329825
0.630366317	0.024601559	5.404412057	0.018382079
0.633599813	0.00935042	6.467534278	0.00624749
0.599993103	0.015932145	5.968696172	0.01265611
0.637192741	0.005075381	6.469664031	0.003493166
0.637747273	0.012953985	5.662975998	0.010206695
0.645446541	0.013099096	6.405547589	0.010175742
0.634118811	0.008321889	6.417451164	0.005610998
0.619161398	0.016658321	6.223780706	0.011435878
0.613844992	0.006899949	6.503296407	0.004308376
0.611649273	0.007681422	6.397044762	0.005154922
0.620445564	0.003739418	6.632321977	0.002578623
0.647588101	0.01006846	6.240596442	0.00597032
0.610414275	0.013609846	6.280181963	0.010008854
0.606269853	0.015013473	5.998407566	0.00955272
0.62454027	0.008658214	6.295413025	0.005742073
0.635869352	0.011947755	6.358749378	0.008534068
0.667962223	0.018260129	5.988284261	0.014442152
0.683623038	0.011067165	6.348798469	0.009272538
0.642050126	0.003835561	6.488356159	0.002813934
0.640835603	0.024023162	5.255070844	0.022369979
0.591147169	0.010475323	6.428047314	0.006597422
0.598823345	0.004767318	6.547010471	0.003210983
0.670678773	0.01518943	5.871287002	0.010901653
0.624644835	0.006597967	6.418423987	0.004695923
0.56491778	0.032944504	5.078403884	0.025488055
0.667089763	0.02796295	5.129434956	0.012291042
0.621369097	0.003920321	6.667779166	0.00302287
0.684190074	0.010703544	6.16554657	0.00885154
0.619333208	0.008451415	6.226285784	0.006682746
0.601154367	0.01659653	6.450153525	0.009682749
0.610270963	0.010631888	6.429242919	0.006640655
0.733172609	0.016915002	5.815052917	0.014326712
0.612276304	0.007674592	6.334413098	0.005796315
0.674015411	0.024647938	5.309267368	0.011571033
0.539366348	0.030331018	5.803402513	0.014782241
0.710240529	0.018974291	6.070124908	0.011873847

log.sigma.2.5.mm.3D_ngtdm_Coarseness	log.sigma.2.5.mm.3D_ngtdm_Complexity	log.sigma.2.5.mm.3D_ngtdm_Strength	log.sigma.2.5.mm.3D_ngtdm_Busyness	log.sigma.2.5.mm.3D_ngtdm_Contrast
0.011072098	1.303978024	2.824705097	0.268113751	0.000302805
0.00491965	0.390868152	1.366335728	0.412698993	5.05E-05
0.016306634	2.223842113	4.754495906	0.204736563	0.000484728
0.003096451	0.633414556	1.217961603	0.482064594	6.64E-05
0.025662679	2.760533721	8.1347216	0.073642026	0.000480958
0.011526228	0.679069977	1.675917081	0.433133119	0.000304505
0.021332346	1.09928901	3.681746566	0.129739545	0.000318869
0.006780772	1.289311222	2.208561043	0.382243612	0.000297637
0.003987305	0.673022344	1.446258898	0.503464419	9.32E-05
0.002698605	0.302885752	0.907536189	0.746171929	3.30E-05
0.007235688	0.71004237	1.428432176	0.387455047	0.000263291
0.005732463	0.52746875	1.334766449	0.573692419	0.000119982
0.003899787	0.327534836	0.983589671	0.818054699	6.25E-05
0.006637891	0.602608293	1.564350956	0.521658994	0.000152986
0.008733666	0.738406411	1.671211601	0.339017842	0.000199876
0.001478482	0.103584705	0.803631819	1.119505384	4.44E-06
0.010093891	1.276232015	2.659918486	0.267441475	0.000317015
0.003658238	0.331318469	1.010268609	1.030550274	6.94E-05
0.014016525	1.609173175	3.591298614	0.206464142	0.000474291
0.003582819	0.132501367	0.561983034	1.193749471	3.47E-05
0.001665982	0.140081975	0.460400489	1.292930822	1.47E-05
0.004764747	0.602065081	1.511652309	0.508142642	8.65E-05
0.00312853	0.218722342	0.65521937	1.139320671	5.08E-05
0.015129452	1.88016985	3.905153952	0.131634209	0.00046992
0.006250651	0.639265542	1.754370863	0.477112711	0.000100917
0.011965511	1.59878703	3.372026466	0.21551095	0.000286352
0.002672743	0.267363328	1.200787799	0.470943557	1.32E-05
0.005376402	0.571003343	1.394803783	0.580919415	0.000109572
0.003587114	0.619930584	1.092997869	0.634597556	0.000154503
0.003899776	0.45915481	1.802028973	0.413293981	2.80E-05
0.002454726	0.236340502	0.635885663	1.028578937	3.44E-05
0.001597519	0.09740378	0.389828521	1.960447561	1.67E-05
0.003730928	0.578985776	1.245455145	0.624297801	9.68E-05
0.055711331	10.2211554	15.77071064	0.035960196	0.003779443
0.019445803	2.935349903	4.386868902	0.147752465	0.001204568
0.003918726	0.439360554	1.125586321	0.556885891	7.66E-05
0.002591864	0.537265101	1.312399063	0.790123282	4.73E-05
0.002453608	0.205872773	0.537518326	1.132014706	3.91E-05
0.008464355	1.197075878	2.532210994	0.218872669	0.000178308
0.007341521	0.835034739	1.787646871	0.388824294	0.000195177
0.006073257	0.409618801	1.170860735	0.56450522	9.81E-05
0.007790959	0.95060866	2.203628069	0.339619062	0.00021951
0.004036221	0.601849452	1.214165126	0.523177036	0.000122531
0.007471453	1.11949234	2.243572048	0.302388551	0.000229034
0.003866637	0.509341947	1.020273969	0.584750113	9.24E-05
0.003287123	0.215004525	0.67309935	1.588163357	5.02E-05
0.001948048	0.339963153	0.741671613	0.826306589	3.13E-05
0.01169624	1.253453225	2.401456501	0.348898793	0.000435189
0.035309685	7.633630234	13.98054946	0.044183309	0.002916033
0.013924413	2.271020485	5.062966672	0.141294585	0.00043713
0.002567771	0.250647305	0.769881032	0.865032216	3.06E-05
0.006174605	0.808769228	1.341254433	0.579557283	0.000422658
0.00225873	0.352074576	0.948029277	0.645760869	2.88E-05
0.013435471	0.697377832	1.878571369	0.338093829	0.000305262
0.001674647	0.090908057	0.428260647	1.597677301	1.19E-05
0.008232117	1.570448033	2.54155504	0.280503213	0.000321231
0.003364714	1.031617639	1.714926196	0.405825637	9.72E-05
0.005883688	1.003999482	2.12012452	0.242100148	0.000147258
0.010794651	0.857939492	2.191311248	0.272730211	0.000209314
0.009108755	0.627941651	2.27181767	0.232699461	0.000110518
0.003481491	0.528636865	1.177558607	0.753533779	8.38E-05
0.001573709	0.319434026	0.887662749	1.024397271	2.08E-05
0.003117624	0.19442514	0.825654326	0.727806078	2.01E-05
0.003468264	0.282429199	1.202046766	0.340075152	2.26E-05
0.033721651	2.403367537	5.509782696	0.117590727	0.001177029
0.005950935	0.925243465	2.545019205	0.274181287	8.26E-05
0.016005824	2.980568392	4.754699539	0.125391162	0.000624918
0.002507363	0.397441799	0.929106178	0.743358683	5.25E-05
0.014311861	1.303841289	2.621472444	0.259578871	0.0006359
0.004010889	0.547006293	1.218947495	0.642396237	9.08E-05
0.002664939	0.293941393	0.694272248	1.104652298	5.98E-05
0.006385261	0.767462381	1.741345729	0.480638524	0.000181352
0.001749876	0.147265496	0.483036267	1.327005755	1.93E-05
0.00139666	0.095831801	0.342108154	1.668264071	1.23E-05
0.00102979	0.15317463	0.601145485	0.916332519	5.66E-06
0.006158402	1.245428367	2.042296256	0.323386058	0.00024338
0.006394242	0.866630504	1.725220424	0.380313034	0.000169314
0.010921283	1.202577419	2.296614104	0.459176633	0.000492029
0.004139981	0.615800702	1.175855657	0.571837745	0.00011758
0.002603147	0.425902555	0.96404747	0.856907339	4.95E-05
0.013023206	2.729442226	4.058025954	0.200158003	0.000701095
0.005189568	1.45465743	2.118944249	0.308115598	0.000213674
0.003195149	0.559862656	1.268241356	0.381978781	5.22E-05
0.048564505	5.879550632	13.83675196	0.037435676	0.001766002
0.002367366	0.207258925	0.560720122	1.386752881	4.04E-05
0.001630457	0.236635133	1.099576063	0.672865864	7.75E-06
0.009103714	1.028291195	2.249773037	0.407727214	0.000307519
0.004104317	0.823031722	1.495450168	0.406640695	0.000174232
0.035658487	2.014668474	4.661146356	0.127105658	0.001093952
0.032845434	6.5653219	8.459765021	0.083068063	0.004703684
0.002333614	0.416190804	1.221423103	0.403212885	2.21E-05
0.009877466	2.534020781	4.021944038	0.217249106	0.000357701
0.008389844	0.838264355	2.071093394	0.22520769	0.000126641
0.002105061	0.197098515	0.600857759	1.273936687	2.63E-05
0.00148394	0.12140868	0.391198012	1.851102121	1.63E-05
0.024405851	8.907898133	11.40338611	0.071955678	0.001971025
0.007014888	0.736679761	1.98565009	0.24384581	0.000115889
0.051237929	8.222626644	14.31628309	0.040333436	0.002576926
0.021855622	1.086524228	3.677026299	0.179521677	0.000407014
0.010222861	1.843364897	4.287375178	0.19236756	0.00021182

log.sigma.2.0.mm.3D_gldm_GrayLevelVariance	log.sigma.2.0.mm.3D_gldm_HighGrayLevelEmphasis	log.sigma.2.0.mm.3D_gldm_GrayLevelNonUniformityNormalized	log.sigma.2.0.mm.3D_gldm_DependenceEntropy
31.15519144	263.9985836	0.050995514	6.449655971
20.21897393	286.4256139	0.074968086	6.879417426
31.17609739	203.8502304	0.058941579	6.207034477
38.68971033	485.1096774	0.044974634	6.840314027
27.56626656	341.1225627	0.060435596	6.170988107
19.41748559	154.9968896	0.064939086	6.205311165
14.85794539	349.8456973	0.096020921	5.806514811
38.56073183	271.6257485	0.047434074	6.497358226
33.76061631	320.3290043	0.047460334	6.72649368
22.16427183	346.8386056	0.067733989	6.669266655
20.71165867	186.8004837	0.063741997	6.233592616
28.70143021	398.9392713	0.056846804	6.673003627
19.43656387	183.0766635	0.064297464	6.438551155
20.9473512	157.2602862	0.062298188	6.460379061
23.2668989	310.3666301	0.059467106	6.288748503
11.32066754	254.6034259	0.148961966	6.592927269
26.3714522	197.2765043	0.057056182	6.414802334
22.61650053	177.9245283	0.060601402	6.718854554
33.70027515	321.766323	0.049916746	6.451403976
13.402483	222.3241699	0.084129055	6.757379339
16.97860505	270.4123277	0.083568681	6.619851771
26.46911303	218.9686047	0.055571255	6.478934369
19.02134021	192.7667521	0.070146377	6.514547081
31.39675226	428.1600832	0.056496125	6.276117168
19.56227511	201.7001661	0.074423847	6.43772027
31.06361651	381.9510086	0.054161234	6.566358506
14.79808312	352.4307559	0.09060217	6.772705663
24.81377694	239.4270761	0.067051248	6.476285035
35.48368288	324.0407932	0.04893226	6.585577797
19.37287914	206.6707317	0.080060801	6.841860032
19.13844521	268.3293489	0.074163265	6.481436045
15.43171458	153.2664418	0.078464662	6.680908491
33.12422791	381.5809149	0.049213312	6.789264517
37.24029216	397.875	0.07137574	5.415355675
41.25695856	316.996988	0.045162578	6.149407665
22.94030267	256.2886139	0.05831095	6.502551528
30.46811523	232.196875	0.055624035	6.82702489
21.9439021	367.6222421	0.06087659	6.718534871
21.87747714	246.302994	0.063275126	6.401982386
28.67457227	288.8093422	0.052763493	6.578269329
21.80789773	320.3894166	0.063267602	6.76384426
27.735693	255.2927597	0.055611649	6.483966858
27.9306244	255.0182704	0.055988434	6.560032188
33.7017718	272.035122	0.049872219	6.650331208
26.41182642	256.6496273	0.054357898	6.507993566
15.4419005	118.8182226	0.075978085	6.474639492
31.90711739	597.2006564	0.050633461	6.834976429
26.59932507	192.8456261	0.055150358	6.143163135
29.25289597	341.2761194	0.087770105	5.411129352
31.22666785	312.750503	0.05992899	6.235275442
20.81224343	257.526285	0.064094026	6.719719003
29.31864565	214.2720677	0.057858345	6.276048568
25.21417755	525.0601937	0.05711073	6.881068228
17.77864241	178.9526227	0.065657737	6.230106815
15.87870813	258.3699218	0.096209126	6.745776044
42.51394891	367.8668942	0.042639466	6.518133302
43.79961778	538.8367533	0.042719144	6.811633036
34.12907027	421.6372776	0.056865782	6.478591504
24.30340207	280.5	0.061892918	6.60617776
16.32565924	557.5085131	0.072849576	6.221554293
27.86874324	253.5630014	0.05520537	6.523649133
29.88181889	458.4428624	0.05385357	6.954139112
13.83453294	297.838835	0.086052723	6.628223609
14.88104183	1260.627243	0.097227125	6.846776411
22.20268003	166.4239631	0.061861581	5.959528897
30.45064597	266.992273	0.055239449	6.609413978
34.29480275	255.6548223	0.053299492	6.214208983
29.14684992	447.8145136	0.05214822	6.769469784
25.90004323	235.7600897	0.056858171	6.062990771
30.85745802	301.968175	0.051774168	6.696246777
26.0664386	398.6707237	0.057590244	6.706589796
25.16677245	218.3250664	0.057381647	6.513543939
19.04691136	285.4224071	0.07069418	6.844251049
15.90204923	272.8463371	0.082229471	6.745746945
20.22516623	498.9242919	0.077493034	6.847169925
40.31179183	361.8969534	0.044521846	6.558454879
26.61961274	217.5640107	0.054721168	6.584831213
28.52438861	209.0081967	0.05628057	6.264507478
28.55492664	237.803288	0.05279886	6.595559221
27.17563579	283.4005622	0.05631111	6.691146536
42.62609836	329.8075397	0.045784518	6.25382952
52.01390328	559.2207308	0.039488267	6.661909725
36.77869435	1203.715616	0.050092723	6.802543929
29.26487515	294.3931034	0.066159334	5.512278315
20.17968583	205.2391625	0.064068408	6.540750884
15.57714158	521.3670562	0.084479669	6.790989001
26.95035058	193.9511568	0.056353712	6.279766139
35.26277326	399.7725515	0.052236389	6.462036748
16.55546597	211.3333333	0.07044104	5.776529828
34.56235828	293.6190476	0.084263039	5.066313913
26.40980062	835.429434	0.061108026	6.939117724
45.40634304	422.184136	0.045157252	6.481443169
24.58401278	393.0587084	0.066063243	6.759226181
20.45864169	317.5447433	0.064050071	6.84370594
17.45282103	286.1846366	0.07064348	6.702850983
69.69719883	629.1225296	0.0403537	6.160282953
24.45958965	383.8675987	0.06169792	6.476159026
34.64934893	289.6097561	0.055456408	5.46936316
18.07635703	192.121372	0.069624968	6.056349517
33.95893122	352.5836431	0.060261129	6.670270787

log.sigma.2.0.mm.3D_gldm_DependenceNonUniformity	log.sigma.2.0.mm.3D_gldm_GrayLevelNonUniformity	log.sigma.2.0.mm.3D_gldm_SmallDependenceEmphasis
166.9716714	36.00283286	0.39298465
276.403948	155.7087145	0.256473154
102.2073733	25.58064516	0.436409073
619.5874763	118.5081594	0.403383866
85.8189415	21.69637883	0.366637264
137.251944	41.75583204	0.369182049
68.66765579	32.35905045	0.316935219
248.2694611	47.52894212	0.40814983
486.5921116	98.67003367	0.379293773
544.8231117	209.8398967	0.323731348
185.3700121	52.7146312	0.343123041
291.1133603	84.24696356	0.351776019
441.7782064	133.3529412	0.315419678
219.2576029	69.64937388	0.32695593
226.4621295	54.17453348	0.413237612
632.4340003	1034.838779	0.179788082
151.9283668	39.8252149	0.373264633
428.0883362	141.32247	0.284383179
139.8900344	29.05154639	0.383233776
359.2513388	235.6454838	0.214846098
768.7787136	436.5627871	0.295295902
408.4604651	95.58255814	0.38277326
503.0314903	191.5697547	0.316706643
125.045738	27.17463617	0.441678794
217.2774086	89.60631229	0.330673706
141.7463977	37.58789625	0.365226247
443.1534911	314.0271206	0.240015422
278.3984648	96.08443824	0.362997593
427.9042493	86.36543909	0.392858682
338.5074744	203.5145555	0.272314275
567.9298522	235.7650204	0.324130815
950.8037099	465.2954469	0.263868865
435.6797836	100.050664	0.376966294
33.25	7.423076923	0.564855769
95.71084337	14.9939759	0.473355872
425.8415842	117.7881188	0.307535683
546.4652778	160.1972222	0.334164141
627.4818128	204.1800835	0.324253673
173.6299401	52.83473054	0.333763942
221.3012393	55.34890372	0.371979796
246.2550882	93.25644505	0.304334878
209.7072403	52.99790136	0.357249249
352.6199756	91.93300853	0.337491994
227.3160976	51.11902439	0.354426899
448.7912673	102.0841321	0.394350352
370.6588869	169.2791741	0.294537636
929.9915612	216.0023441	0.380153446
151.2538593	32.15265866	0.392771584
32.02985075	11.76119403	0.406069588
124.110664	29.78470825	0.418906392
614.9199766	219.4579439	0.31670729
190.8645707	47.84885127	0.347579197
699.1015441	218.2200995	0.313180101
124.2893401	38.8037225	0.332609882
811.41392	627.5721294	0.230463489
242.3447099	37.48009101	0.452014748
535.2909257	93.68308254	0.412673156
307.3480278	73.52745553	0.396033128
149.1474201	50.38083538	0.346045304
168.7139614	64.18047673	0.325332758
457.7947145	116.9801793	0.348592031
1020.112815	295.0098576	0.331153918
446.2330097	265.9029126	0.255648427
296.5496155	265.5272794	0.213077904
45.57142857	13.42396313	0.366343368
342.9381842	85.78686413	0.376058734
105.5837563	21	0.45081269
650.2599312	166.7178605	0.327659785
115.5336323	25.35874439	0.39847011
433.0696171	104.1178518	0.371927471
595.5078947	175.0743421	0.343352819
232.3764393	64.78387954	0.325654196
775.3989938	365.3475232	0.270626949
943.9937463	552.2531269	0.252695199
1363.390196	711.3860566	0.285279123
291.5806452	49.68637993	0.425481849
233.1951656	61.12354521	0.364796069
141.9967213	34.33114754	0.391450558
384.1043084	93.13718821	0.366848316
570.4223472	160.2614195	0.343023686
140.8174603	23.07539683	0.477232694
384.7032066	52.95376585	0.481984715
620.8273639	139.8588825	0.398036227
37.34482759	9.593103448	0.382819923
659.0171041	217.2559717	0.313275621
741.4010226	462.6106647	0.240454259
173.2750643	43.84318766	0.391304048
382.8402062	81.07087629	0.381290009
38.08196721	12.89071038	0.345134575
30.94285714	8.847619048	0.461079365
675.7677987	242.9044025	0.307364552
190.1246459	31.88101983	0.456186815
172.8747554	67.51663405	0.329985796
685.3897311	261.9647922	0.302776452
921.748536	410.1560455	0.272999789
75.93280632	10.20948617	0.497044049
247.3486842	75.02467105	0.319166522
39.42276423	6.821138211	0.478504968
86.79419525	26.3878628	0.321997903
149.3320942	48.6307311	0.357618989

log.sigma.2.0.mm.3D_gldm_DependenceNonUniformityNormalized	log.sigma.2.0.mm.3D_gldm_DependenceVariance	log.sigma.2.0.mm.3D_gldm_LargeDependenceEmphasis
0.236503784	2.019813978	8.135977337
0.133078454	10.94881024	28.77371208
0.23550086	2.400157149	8.433179724
0.235137562	2.630990066	8.910436433
0.239049976	1.401090929	7.423398329
0.213455589	2.30881197	9.32348367
0.20376159	2.605270805	10.70326409
0.247773913	1.727451285	7.369261477
0.23405104	1.847721124	8.034151034
0.175862851	3.632590801	13.17107811
0.224147536	1.903481209	8.731559855
0.196432767	2.608658832	10.48043185
0.213007814	2.165564564	9.787849566
0.196115924	2.568821784	10.71019678
0.248586311	1.704147744	7.248079034
0.091036994	14.16788865	44.50424644
0.217662417	2.297362091	9.126074499
0.183571328	3.423437505	13.09948542
0.240360884	1.686246029	7.639175258
0.128258243	8.987781306	27.26561942
0.147162847	9.299407376	23.81049005
0.237477015	1.965932937	8.123255814
0.184193149	3.424462023	12.57890882
0.259970349	1.4938905	6.563409563
0.180462964	3.927026192	13.35049834
0.20424553	3.817364151	11.80979827
0.127857326	10.95173025	29.47201385
0.194276668	3.075917134	11.12281926
0.242438668	1.746122351	7.601699717
0.133165804	11.06088059	28.54130606
0.178650473	3.777345649	13.23214847
0.160337894	6.036962539	18.59460371
0.214303878	2.625399066	9.657156911
0.319711538	1.557692308	5.557692308
0.288285673	1.314813471	5.734939759
0.210812665	1.955522988	9.646534653
0.189744888	4.495756173	13.66319444
0.187084619	4.434958712	13.81454979
0.207940048	2.100291871	9.536526946
0.210964003	2.416762617	9.4957102
0.167065867	4.708089011	15.53459973
0.22004957	1.883399085	8.639034627
0.21475029	2.029707392	9.21680877
0.221771802	2.063862939	8.884878049
0.238972986	1.906319573	7.890308839
0.166363953	4.391764357	15.24506284
0.218000835	3.023597217	10.13220816
0.259440582	1.26038795	6.598627787
0.239028737	2.220093562	8.432835821
0.249719646	1.773400969	7.30583501
0.179591115	4.990967756	15.03971963
0.2307915	1.809857456	8.412333736
0.182962979	5.262125223	15.41821513
0.210303452	2.190665968	9.639593909
0.124392752	8.559834493	26.68787368
0.275705017	1.307259128	5.994311718
0.244090709	1.984252011	7.756953944
0.237701491	1.725049332	7.618716164
0.183227789	7.19872743	17.68550369
0.191502794	2.693229884	11.15664018
0.21604281	1.929467563	8.896177442
0.186219937	4.605170458	13.99707923
0.144411977	6.502855647	20.6394822
0.108586458	15.3672213	40.43317466
0.210006583	2.38442099	9.552995392
0.220823042	2.209237133	8.893110109
0.267979077	1.607719859	6.573604061
0.203396913	2.817857569	10.81169847
0.259044019	1.581954192	7.040358744
0.215350381	2.464835251	9.439582297
0.195890755	3.01736669	11.14736842
0.205825013	2.164038345	9.8255093
0.150038505	7.153467486	20.84404025
0.140558926	7.051473622	21.88475283
0.148517451	5.940689051	18.95250545
0.26127298	1.512631518	6.684587814
0.208769172	2.421066468	9.639212175
0.23278151	2.102456329	8.344262295
0.217746207	2.313792864	9.22675737
0.200429497	2.987058806	10.9360506
0.279399723	1.348954397	5.873015873
0.286877857	1.378824555	5.797912006
0.222359371	2.455729017	9.032234957
0.257550535	1.290083234	6.820689655
0.194342997	2.628224607	11.04010616
0.135390983	7.38243486	23.17275383
0.222718592	1.930637519	8.303341902
0.246675391	1.624641301	7.469072165
0.208098181	2.472334199	10.0273224
0.294693878	1.014603175	5.285714286
0.170004478	6.566466453	18.01383648
0.269298365	1.582116862	6.464589235
0.169153381	11.35441424	24.77495108
0.167576951	5.973061137	17.20293399
0.158757929	4.855576607	16.74061316
0.300129669	1.592526051	5.964426877
0.203411747	2.372316482	10.30098684
0.320510278	0.869323815	4.772357724
0.229008431	1.740770393	8.688654354
0.185045966	4.309488383	13.41387856

log.sigma.2.0.mm.3D_gldm_LargeDependenceLowGrayLevelEmphasis	log.sigma.2.0.mm.3D_gldm_SmallDependenceHighGrayLevelEmphasis	log.sigma.2.0.mm.3D_gldm_LargeDependenceHighGrayLevelEmphasis
0.060437302	118.0326864	1755.191218
0.120228658	85.44403817	7581.497352
0.087127003	110.0522055	1218.052995
0.026410656	208.6589822	3962.916129
0.034176365	126.3138796	2507.779944
0.125283495	62.54202095	1217.454121
0.034890007	100.6418755	3992.605341
0.063864633	116.7493982	1866.312375
0.045841054	126.7048337	2399.92304
0.050306933	130.3873222	3947.457069
0.08853352	67.62182638	1569.475212
0.036808636	159.9477245	3644.511471
0.088777882	61.05781348	1607.720829
0.149883448	58.71128798	1455.276386
0.032938283	133.7719769	2123.767289
0.198720484	55.497292	10379.79646
0.109362774	79.13550103	1651.540115
0.143830824	61.6983269	1868.698113
0.047925118	128.8192883	2261.694158
0.15346601	57.06991533	5429.260264
0.10546496	94.87169594	5769.788285
0.068839754	97.15242382	1489.055814
0.10678663	71.56865128	1967.096302
0.021951087	188.132202	2816.621622
0.102431411	80.36237023	2231.756645
0.043511895	160.5793798	4002.365994
0.094809338	96.82038316	9663.466532
0.078180025	104.7631241	2143.43545
0.044526426	128.2939219	2377.623796
0.168216205	68.16450944	5539.289142
0.068863569	106.966114	2940.568418
0.18665647	49.06994893	2367.36425
0.040186615	155.2657463	3419.530743
0.027990929	208.2975775	2460.759615
0.046327152	160.616507	1597.171687
0.058253468	82.13471277	2390.538119
0.098203044	94.21304907	2695.879514
0.047996838	129.486139	4562.842278
0.064941483	83.36377242	2377.34012
0.054476616	120.5437607	2457.907531
0.060094851	106.3342731	4695.404342
0.061610114	94.89099073	2088.010493
0.066813409	89.09144502	2294.107186
0.072008561	104.6047442	2224.138537
0.051516753	102.4362598	1996.218317
0.234069306	44.23051704	1387.117145
0.021078335	241.7288291	5633.694796
0.085196446	80.97003383	1127.164666
0.093121153	138.6210778	3007.783582
0.044127716	131.1261169	2243.736419
0.078415693	92.73384827	3471.745327
0.087499	75.85129934	1827.616687
0.034654631	176.0729601	7638.117509
0.09215661	65.03383874	1519.588832
0.124796937	68.72619551	6280.849456
0.030011297	177.7520065	2074.715586
0.023880522	237.0023083	3652.026904
0.028950908	163.3013651	3294.525135
0.082906961	107.5239663	4548.224816
0.033506548	198.4232586	5526.818388
0.063138468	100.2802785	1940.853233
0.038078619	163.5253425	5950.525374
0.082225772	85.71240864	5614.689968
0.03309633	270.2562788	50474.17173
0.179641361	76.3657119	1144.894009
0.067250503	112.8809108	2043.113329
0.076729186	116.8665999	1702.675127
0.032669955	160.893271	4433.52299
0.062327496	100.6126058	1480.015695
0.05143411	123.9403714	2514.583789
0.038946029	152.0003006	3878.688487
0.084870309	77.03858945	1918.968113
0.090465889	89.28803093	5373.762964
0.095665561	78.09317489	5530.346933
0.042251574	151.2055845	9037.708824
0.037623313	155.6531753	2339.006272
0.093875335	84.32190646	1927.38675
0.092249365	95.26736545	1268.296721
0.076333851	95.37361498	1914.144558
0.058415275	110.4372084	2752.565706
0.037901181	173.9154597	1804.295635
0.017215959	277.8399016	3132.404922
0.008808994	498.0856548	10402.87536
0.042594798	96.18712452	2200.434483
0.088333497	72.75800559	1940.569154
0.04985874	139.9217436	11272.82798
0.097279555	90.89520209	1258.658098
0.031218491	149.6386155	2947.717139
0.06635273	73.45855705	2251.639344
0.111843956	121.130291	1787.885714
0.024237548	262.835418	14566.73711
0.027219432	222.9576414	2231.94051
0.073536495	139.9475454	9133.32681
0.065433154	106.2509914	5168.148166
0.075858537	90.52050759	4204.955219
0.020151748	324.8165357	3559.557312
0.035488069	117.214952	4065.681743
0.101228452	139.5133808	1454.195122
0.066505187	59.69649703	1705.85752
0.052590955	152.4278839	4114.690211

log.sigma.2.0.mm.3D_gldm_SmallDependenceLowGrayLevelEmphasis	log.sigma.2.0.mm.3D_gldm_LowGrayLevelEmphasis	log.sigma.2.0.mm.3D_gldzm_DistanceZoneVariabilityNormalized
0.005988859	0.010046175	0.918039073
0.0019017	0.005489879	0.906185076
0.008060315	0.01346108	0.911694102
0.00181916	0.003640432	0.868724058
0.004687653	0.007434479	0.891922593
0.00783569	0.016035593	0.873494898
0.004508446	0.006673219	0.63554402
0.005028359	0.01050919	0.839891712
0.003097264	0.006662422	0.92686068
0.00161714	0.004209728	0.933644857
0.005424808	0.012950136	0.875889605
0.002117944	0.004329141	0.938628248
0.004532556	0.010635005	0.917328765
0.008044228	0.017246514	0.924893645
0.003854985	0.00642697	0.85650027
0.001081564	0.004970261	0.903384847
0.007595605	0.015188122	0.760536819
0.003924922	0.011856626	0.950342813
0.00446856	0.008505663	0.82677882
0.001644436	0.006327037	0.947175529
0.001781728	0.005177446	0.896401463
0.004641495	0.009826706	0.866107996
0.003333022	0.009156209	0.943296936
0.004125021	0.005901463	0.7312
0.003180031	0.009495675	0.907586899
0.003313568	0.005764721	0.920403252
0.001409731	0.004015919	0.891861566
0.003233442	0.008480611	0.937565036
0.003891342	0.00748914	0.932640274
0.003139814	0.008569667	0.951683611
0.001909461	0.005479452	0.951453886
0.003631065	0.011721373	0.94639269
0.002018768	0.005195168	0.916912615
0.016030563	0.017186767	0.687235464
0.008652189	0.012577796	0.881078357
0.002857075	0.006967805	0.885569931
0.003694239	0.00923615	0.968206183
0.001662982	0.00405186	0.927179683
0.005052009	0.009803103	0.801962487
0.004155366	0.007798088	0.94786352
0.002394859	0.005292186	0.825694401
0.004010118	0.008642952	0.880670896
0.004269252	0.009347784	0.881329211
0.006162165	0.011647327	0.833663956
0.004757338	0.009020091	0.864037524
0.006041662	0.017853427	0.973549727
0.001180444	0.002486517	0.826330213
0.006355574	0.013493686	0.929173875
0.008659389	0.022529826	0.650364204
0.005235328	0.008473661	0.766897126
0.002350219	0.006214797	0.933210712
0.005507809	0.012939242	0.966111772
0.00102042	0.002625676	0.929941389
0.005418838	0.01165455	0.86418698
0.001417777	0.005350295	0.966775068
0.004029793	0.00686514	0.83982686
0.001713702	0.003390328	0.753219375
0.003594209	0.006058075	0.704969012
0.003887483	0.007797699	0.897153184
0.001212847	0.004294163	0.981652949
0.004718898	0.009050423	0.919783667
0.001279924	0.003202465	0.917013434
0.001718052	0.004721327	0.953719509
0.000557342	0.001205115	0.935357418
0.010027391	0.019602726	0.89721172
0.004201533	0.009887167	0.868584838
0.010108765	0.019268438	0.76795403
0.00130245	0.003367375	0.950471647
0.008507398	0.012944405	0.86963927
0.003488153	0.007154287	0.899516954
0.001431425	0.003665714	0.887348537
0.004200655	0.00980687	0.941362899
0.001645918	0.005023978	0.947992541
0.001514556	0.005010671	0.951097932
0.000937361	0.002591132	0.940999552
0.004818539	0.007990629	0.787938232
0.005536262	0.012850374	0.856345706
0.006180489	0.012093249	0.911152221
0.005231942	0.011139166	0.823603217
0.002579817	0.006314745	0.870947556
0.008593304	0.011544105	0.866021973
0.002450415	0.003958515	0.850689405
0.000746529	0.001312612	0.887989884
0.01234959	0.016142751	0.617539027
0.00317021	0.008613833	0.951288276
0.000706715	0.002352789	0.778644641
0.009322952	0.015822331	0.911791239
0.00240718	0.004970286	0.82958665
0.008321308	0.012808795	0.811634349
0.021533775	0.038317774	0.762187872
0.001321974	0.002247296	0.904490245
0.003223362	0.005345073	0.968512
0.002287943	0.004537708	0.881986435
0.001707746	0.004594327	0.919045205
0.001584963	0.004908753	0.935130934
0.005731944	0.00733793	0.7207333
0.003726813	0.006230795	0.96105434
0.007737854	0.024852227	0.78125
0.006937045	0.012528162	0.796658145
0.002857701	0.005571435	0.929712401

log.sigma.2.0.mm.3D_gldzm_LowIntensityEmphasis	log.sigma.2.0.mm.3D_gldzm_LargeDistanceEmphasis	log.sigma.2.0.mm.3D_gldzm_HighIntensitySmallDistanceEmphasis
0.013846512	1.128440367	298.6269113
0.007191351	1.148026316	327.754523
0.017065622	1.138888889	249.744213
0.004330977	1.21187801	490.9067014
0.011553942	1.171974522	338.0541401
0.019977485	1.203571429	170.2607143
0.013093601	1.719008264	289.7438017
0.012218542	1.263265306	278.4765306
0.007911933	1.114044351	327.3093981
0.004885496	1.103074141	403.5818264
0.015872422	1.199421965	190.6697977
0.005794473	1.095070423	452.9854754
0.013577285	1.129606099	191.2658831
0.022928763	1.117241379	177.845977
0.008676764	1.233409611	308.6270023
0.006012893	1.152694611	302.6659805
0.019581205	1.417218543	196.3807947
0.013376149	1.076433121	217.3449045
0.011260099	1.287356322	331.8362069
0.007376924	1.081447964	271.107089
0.005947662	1.164407778	315.4151444
0.011436535	1.216455696	244.9996835
0.010345693	1.087613293	229.5163646
0.008485302	1.48	392.201
0.010630828	1.145695364	237.6418322
0.0084905	1.124567474	435.3555363
0.005712823	1.172077922	388.5892857
0.009461363	1.096774194	288.1816638
0.009367536	1.104693141	322.1856197
0.011418117	1.074315515	249.672425
0.005820783	1.074678112	326.9497854
0.013626022	1.082690187	184.1878445
0.005706987	1.130289532	400.219098
0.025253223	1.582089552	361.6977612
0.016406491	1.19047619	327.3465608
0.008705036	1.182781457	257.1672185
0.010707142	1.048473968	278.798474
0.00494346	1.11352657	394.5595813
0.013807144	1.33431085	234.6986804
0.01049098	1.080357143	318.0580357
0.007563766	1.289370079	330.4183071
0.010776492	1.191176471	261.3069853
0.012181456	1.190045249	256.4611614
0.016621641	1.274647887	285.5346244
0.01144622	1.220090293	249.868228
0.020861542	1.040214477	151.3746649
0.002972643	1.288188976	590.2048556
0.015412901	1.110294118	204.9503676
0.027607862	1.677419355	323.9677419
0.011587479	1.404081633	297.0408163
0.007153901	1.103773585	290.0023585
0.01550224	1.051724138	216.6393678
0.003128511	1.109052031	550.7601568
0.015046192	1.219827586	198.8200431
0.006113432	1.050694025	300.7432106
0.008445842	1.263383298	374.2392934
0.003936563	1.432532348	533.8830869
0.008448337	1.53960396	383.9863861
0.010945671	1.163141994	302.6714502
0.005039083	1.027777778	611.6057099
0.012504941	1.125581395	286.5232558
0.003760656	1.130123928	478.7185415
0.006397452	1.071106095	335.1044018
0.002483096	1.100318471	1258.741242
0.025614616	1.163043478	202.5597826
0.011605431	1.212121212	288.4361472
0.023406435	1.401913876	250.8397129
0.003825684	1.076229508	488.4821721
0.01885527	1.210280374	247.3925234
0.009116609	1.15916955	326.2099193
0.004017426	1.179746835	436.7888186
0.012069604	1.090697674	239.9622093
0.005888442	1.080152672	332.3562341
0.005872569	1.075240128	312.6938367
0.003213761	1.09127789	517.9601927
0.010491	1.361702128	345.5226064
0.015795719	1.233684211	223.6905263
0.014546248	1.139784946	245.2894265
0.01415912	1.29326288	252.1733818
0.007386031	1.208	309.7404444
0.016270011	1.216606498	357.215704
0.004787885	1.243774574	547.0285059
0.001744399	1.178654292	1190.889791
0.028969491	1.772727273	241.9886364
0.009768461	1.074939565	232.8722804
0.002862469	1.380222841	520.0388231
0.022050268	1.138728324	231.2637283
0.005922516	1.282157676	385.3443983
0.021230227	1.315789474	201.5921053
0.048552579	1.413793103	255.9439655
0.003903067	1.150849858	835.305772
0.006611704	1.048	476.6106667
0.006490623	1.188916877	401.1782116
0.005510973	1.126790831	344.2020057
0.005618368	1.100682594	333.4236348
0.010354608	1.503355705	609.147651
0.010700217	1.059602649	362.3752759
0.022550658	1.375	279.2569444
0.0196699	1.344594595	180.2668919
0.007552128	1.109422492	418.7363222

log.sigma.2.0.mm.3D_gldzm_LowIntensityLargeDistanceEmphasis	log.sigma.2.0.mm.3D_gldzm_HighIntensityEmphasis	log.sigma.2.0.mm.3D_gldzm_DistanceZoneVariability	log.sigma.2.0.mm.3D_gldzm_ZonePercentage
0.01618451	302.9663609	300.1987768	0.463172805
0.010306444	334.8980263	550.9605263	0.292729899
0.039872217	252.3935185	196.9259259	0.497695853
0.005515998	515.5609952	1082.430177	0.472865275
0.033013148	344.4840764	140.0318471	0.437325905
0.037079816	173.3892857	244.5785714	0.435458787
0.044976197	316.6198347	76.90082645	0.359050445
0.026349383	286.0285714	411.5469388	0.489021956
0.009113805	334.3864836	877.7370644	0.455507456
0.0056562	409.3607595	1032.611212	0.357004519
0.030034523	195.7919075	303.0578035	0.418379686
0.006265904	460.3679577	533.1408451	0.383265857
0.016621658	196.1994917	721.9377382	0.379459981
0.034204698	180.708046	402.3287356	0.389087657
0.018471043	324.2036613	374.2906178	0.479692645
0.006839578	314.0681138	1206.922156	0.192313229
0.041667224	209.5629139	229.6821192	0.432664756
0.021054106	218.2955414	746.0191083	0.336620926
0.018106567	341.5862069	215.789272	0.448453608
0.008251306	276.0030166	627.9773756	0.236701178
0.007101605	326.8520919	1521.193282	0.324846861
0.021033503	253.5924051	684.2253165	0.459302326
0.012352365	231.7995972	936.693857	0.363603076
0.013419819	428.66	182.8	0.51975052
0.01290226	244.0110375	411.1368653	0.376245847
0.00994259	439.017301	265.9965398	0.416426513
0.006310528	406.6363636	824.0800866	0.266589729
0.0107324	293.0305603	552.2258065	0.41102582
0.011200056	327.8002407	775.0240674	0.47082153
0.01371405	252.6166884	729.9413299	0.301730921
0.006498292	330.4223176	1108.443777	0.366467443
0.01710379	187.8555678	1716.75634	0.305902192
0.00646886	412.1213808	823.3875278	0.441711756
0.093272816	373.8656716	46.04477612	0.644230769
0.020437679	336.7830688	166.5238095	0.569277108
0.010183693	267.9761589	668.605298	0.373762376
0.011916488	280.927289	1078.581688	0.386805556
0.005456894	404.9098229	1151.557166	0.370304114
0.037072571	247.4486804	273.4692082	0.408383234
0.01141954	322.8727679	424.6428571	0.427073403
0.015270106	350.4940945	419.4527559	0.344640434
0.015467934	267.9117647	359.3137255	0.428121721
0.026611748	262.8989442	584.321267	0.403775883
0.041063723	292.4342723	355.1408451	0.415609756
0.015053938	261.3137698	765.537246	0.471778488
0.022283681	152.3217158	726.2680965	0.334829443
0.003762905	631.8635171	1574.159055	0.446554149
0.018383804	207.25	252.7352941	0.466552316
0.103628625	335.7258065	40.32258065	0.462686567
0.020837566	311.8816327	187.8897959	0.492957746
0.008911041	293.745283	1187.044025	0.371495327
0.017162285	217.8333333	336.2068966	0.420798065
0.003479604	562.6179615	1304.707769	0.367181366
0.037340347	201.2672414	200.4913793	0.392554992
0.006797794	302.8479179	1601.946288	0.254024222
0.01263327	390.117773	392.1991435	0.531285552
0.006648873	572.4380776	814.9833641	0.493388053
0.024271727	411.6930693	427.2112211	0.468677494
0.024209441	307.6223565	296.9577039	0.406633907
0.005126531	613.9783951	318.0555556	0.367763905
0.014597429	290.7093023	791.0139535	0.405851817
0.004287815	494.4556721	1923.894185	0.382986491
0.006821143	339.6264108	844.9954853	0.286731392
0.002598117	1281.176752	587.4044586	0.229952398
0.076835197	203.75	82.54347826	0.423963134
0.021963252	300.4978355	601.9292929	0.446233097
0.053117054	258.9138756	160.5023923	0.530456853
0.00412119	495.1098361	1159.57541	0.381607757
0.02302911	252.9439252	186.1028037	0.479820628
0.011392951	334.4209919	779.8811995	0.431128792
0.005003238	449.6109705	1051.508017	0.389802632
0.014261274	242.3186047	404.7860465	0.380868025
0.006585033	336.8829517	1490.244275	0.304179567
0.00653584	316.6467449	1782.357524	0.27903514
0.003545585	530.9939148	2783.476673	0.322222222
0.026760706	367.4680851	444.3971631	0.505376344
0.03496036	232.6463158	406.7642105	0.425246195
0.019020527	248.437276	254.2114695	0.457377049
0.030818367	262.4306473	623.4676354	0.429138322
0.010073457	322.8524444	979.816	0.395291637
0.023684303	363.6137184	239.8880866	0.549603175
0.006015321	576.6854522	649.0760157	0.568978374
0.001931096	1248.561485	1148.17092	0.463108883
0.106717337	253.0909091	40.75757576	0.455172414
0.010766791	236.4246575	1180.548751	0.365968741
0.003664216	589.2137883	1118.133705	0.262235208
0.025558481	235.8265896	315.4797688	0.444730077
0.008783016	398.9294606	599.791148	0.465850515
0.029523695	211.1052632	61.68421053	0.415300546
0.071598556	265.4482759	44.20689655	0.552380952
0.004170915	861.325779	1277.140227	0.355220126
0.006877802	479.9786667	363.192	0.531161473
0.007627786	419.7052897	350.1486146	0.388454012
0.006504298	353.1439828	1282.987106	0.341320293
0.006305968	339.6774744	1643.960182	0.302790217
0.012804155	648.3892617	107.3892617	0.588932806
0.010917445	368.1103753	435.3576159	0.372532895
0.078196157	283.0694444	56.25	0.585365854
0.028378488	188.2837838	117.9054054	0.390501319
0.008321516	423.7082067	305.8753799	0.407682776

log.sigma.2.0.mm.3D_gldzm_IntensityVariabilityNormalized	log.sigma.2.0.mm.3D_gldzm_LowIntensitySmallDistanceEmphasis	log.sigma.2.0.mm.3D_gldzm_IntensityVariability
0.045516184	0.013262013	14.88379205
0.048081501	0.006412578	29.23355263
0.0468107	0.011363973	10.11111111
0.040030505	0.004034722	49.87800963
0.049210921	0.006189141	7.72611465
0.059693878	0.015701902	16.71428571
0.060310088	0.005122952	7.297520661
0.042748855	0.008685832	20.94693878
0.043189798	0.007611465	40.90073918
0.048113365	0.004692821	53.21338156
0.057469344	0.012331897	19.88439306
0.042997421	0.005676615	24.42253521
0.054925255	0.012816192	43.22617535
0.05589642	0.020109779	24.31494253
0.050243757	0.006228194	21.95652174
0.056204955	0.005806222	75.08982036
0.051993334	0.014059701	15.70198675
0.051317295	0.01145666	40.28407643
0.045844894	0.009548482	11.96551724
0.060789091	0.007158329	40.30316742
0.051382922	0.005659176	87.19681791
0.048431341	0.009037293	38.26075949
0.05429649	0.009844025	53.9164149
0.04576	0.007251672	11.44
0.052302774	0.01006297	23.69315673
0.044958753	0.008127478	12.99307958
0.053020933	0.005563397	48.99134199
0.047437313	0.009143604	27.94057725
0.044956202	0.008909406	37.35860409
0.047811535	0.010844133	36.6714472
0.052725967	0.005651406	61.42575107
0.05746497	0.01275658	104.2414553
0.04319175	0.005516519	38.78619154
0.050568055	0.008248324	3.388059701
0.044427648	0.015398694	8.396825397
0.051645103	0.008335372	38.99205298
0.042823667	0.010404805	47.70556553
0.049162304	0.004815102	61.05958132
0.054944488	0.007990787	18.73607038
0.046635842	0.01025884	20.89285714
0.047538595	0.00563718	24.1496063
0.049247885	0.009603632	20.09313725
0.049409763	0.008573883	32.7586727
0.043201305	0.010511121	18.40375587
0.048392603	0.010544291	42.8758465
0.059854523	0.020506007	44.65147453
0.042380254	0.002775078	80.7343832
0.053984645	0.014670176	14.68382353
0.055671176	0.008602671	3.451612903
0.048263224	0.009274957	11.8244898
0.050262549	0.006714616	63.93396226
0.051674594	0.015087229	17.98275862
0.046912506	0.003040738	65.81824661
0.061422414	0.009472653	14.25
0.052573727	0.005942341	87.11466506
0.039502222	0.007398985	18.44753747
0.040006013	0.003258486	43.28650647
0.042985982	0.004492489	26.04950495
0.04644901	0.007629729	15.37462236
0.06544353	0.005017221	21.2037037
0.047352623	0.011981819	40.72325581
0.043326933	0.003628867	90.89990467
0.056655066	0.006291529	50.19638826
0.053364234	0.00245434	33.51273885
0.061672968	0.012809471	5.673913043
0.044333252	0.009015976	30.72294372
0.045626245	0.01597878	9.535885167
0.047229239	0.003751807	57.61967213
0.05367281	0.01781181	11.48598131
0.044128622	0.008547523	38.25951557
0.04844594	0.003770973	57.40843882
0.052082207	0.011521687	22.39534884
0.049764809	0.005714295	78.2302799
0.05454122	0.005706751	102.2102455
0.046930409	0.003130805	138.8201487
0.041125949	0.006423573	23.19503546
0.050176177	0.011004559	23.83368421
0.051451035	0.013427678	14.35483871
0.046765634	0.009994308	35.4015852
0.046178765	0.006714174	51.95111111
0.04033677	0.014416438	11.1732852
0.036589032	0.004481026	27.91743119
0.039782899	0.001697725	51.43928848
0.048209366	0.009532529	3.181818182
0.05455752	0.009518879	67.70588235
0.053779261	0.002662033	77.2270195
0.051271342	0.021173215	17.73988439
0.047242453	0.005207391	34.15629322
0.07132964	0.01915686	5.421052632
0.064803805	0.042791084	3.75862069
0.044394867	0.003836105	62.68555241
0.038307556	0.00654518	14.36533333
0.045727084	0.006206332	18.15365239
0.049261706	0.005262642	68.76934097
0.053521623	0.005446468	94.09101251
0.034998423	0.009742221	5.214765101
0.046464824	0.01064591	21.04856512
0.049382716	0.008639283	3.555555556
0.056519357	0.017492754	8.364864865
0.040566883	0.007359781	13.34650456

log.sigma.2.0.mm.3D_gldzm_HighIntensityLargeDistanceEmphasis	log.sigma.2.0.mm.3D_gldzm_SmallDistanceEmphasis	log.sigma.2.0.mm.3D_glcm_SumVariance	log.sigma.2.0.mm.3D_glcm_Homogeneity1
320.324159	0.967889908	685.0342448	0.275066118
363.4720395	0.962993421	803.6777793	0.384204566
262.9907407	0.965277778	498.4912875	0.282716748
614.1781701	0.947030498	1430.214073	0.264368129
370.2038217	0.957006369	935.1833397	0.290184065
185.9035714	0.949107143	362.387709	0.310500769
424.1239669	0.820247934	1025.167816	0.331467279
316.2367347	0.934183673	696.3832026	0.26060568
362.6948258	0.971488912	878.5238115	0.276573571
432.4764919	0.974231465	993.2762055	0.327984025
216.2803468	0.950144509	452.1501225	0.303242184
489.8978873	0.976232394	1145.795217	0.301924104
215.9339263	0.967598475	465.7828287	0.320646322
192.1563218	0.970689655	378.0821871	0.321479471
386.5102975	0.941647597	898.3668864	0.273679992
359.6766467	0.961826347	754.8926387	0.478679969
262.2913907	0.895695364	474.7747619	0.29554305
222.0980892	0.98089172	434.1399739	0.340599276
380.5862069	0.92816092	857.9538593	0.272874486
295.586727	0.979638009	604.1407756	0.419107686
372.5998821	0.958898055	771.1594327	0.368020002
287.9632911	0.945886076	559.3339426	0.287158584
240.9325277	0.978096677	487.7125207	0.344944906
574.496	0.88	1233.761103	0.253348705
269.4878587	0.963576159	534.6220474	0.341815369
453.6643599	0.968858131	1051.830143	0.3054218
478.8246753	0.956980519	1065.397278	0.3939454
312.426146	0.975806452	634.8083855	0.319362407
350.2587244	0.973826715	906.5429549	0.274905047
264.3937419	0.981421121	548.9692141	0.390489784
344.3124464	0.981330472	742.9066412	0.333084609
202.5264609	0.979327453	381.3102471	0.381567095
459.7305122	0.967427617	1084.369724	0.281764015
422.5373134	0.854477612	1039.234531	0.245936429
374.5291005	0.952380952	818.0676186	0.240442288
311.2119205	0.954304636	699.1348902	0.313729794
289.4425494	0.987881508	603.8181086	0.309805661
446.310789	0.971618357	1086.20852	0.313431039
298.4486804	0.916422287	657.5982327	0.300365108
342.1316964	0.979910714	780.6824746	0.290751624
430.7972441	0.92765748	924.1191783	0.337028914
294.3308824	0.952205882	669.7829928	0.29216648
288.6500754	0.952488688	674.2290721	0.291534432
320.0328638	0.931338028	703.9756995	0.282973629
307.0959368	0.944977427	700.0833735	0.27791703
156.1099196	0.989946381	263.3298183	0.368546389
798.4981627	0.927952756	1861.849687	0.28238368
216.4485294	0.972426471	452.7575885	0.280793904
382.7580645	0.830645161	895.1293122	0.289509336
371.244898	0.898979592	840.2910068	0.267252419
308.7169811	0.974056604	708.5333295	0.332182177
222.6091954	0.987068966	514.6176482	0.293236476
610.0491803	0.972736992	1587.466778	0.32947665
211.0560345	0.945043103	434.2344837	0.321903211
311.2667471	0.987326494	730.8512192	0.414905525
453.6316916	0.934154176	1000.359978	0.236884781
726.6580407	0.891866913	1552.45109	0.251359177
522.519802	0.86509901	1178.676736	0.262474033
327.4259819	0.959214502	752.2611506	0.324527409
623.4691358	0.993055556	1723.626972	0.338356573
307.4534884	0.968604651	666.2126966	0.294942301
557.4041945	0.967469018	1364.215152	0.316360492
357.714447	0.982223476	874.8244636	0.387491204
1370.91879	0.974920382	4361.089833	0.438741501
208.5108696	0.95923913	364.9505718	0.310429515
348.7445887	0.946969697	723.9835396	0.284979882
291.2105263	0.899521531	625.9475035	0.256768324
521.6204918	0.980942623	1282.193031	0.303320196
275.1495327	0.947429907	583.8131953	0.280866833
367.2652826	0.960207612	827.0575302	0.287316946
500.8995781	0.955063291	1151.783383	0.307422895
251.744186	0.977325581	538.0327409	0.312181238
354.9898219	0.979961832	804.9865233	0.365798307
332.4583778	0.981189968	776.6665802	0.38214917
583.1288032	0.977180527	1578.601156	0.364664871
455.25	0.909574468	990.8000957	0.243735092
268.4694737	0.941578947	548.366675	0.293708518
261.0286738	0.965053763	501.9425223	0.287942621
303.4597094	0.92668428	609.0615642	0.289906619
375.3004444	0.948	777.2068434	0.299194379
389.2057762	0.945848375	836.0179108	0.241850692
695.3132372	0.939056356	1612.199126	0.227010465
1479.24826	0.955336427	4049.000388	0.272740995
297.5	0.806818182	753.3062859	0.282009236
250.634166	0.981265109	534.1912256	0.332825505
865.913649	0.90494429	1688.027968	0.381135445
254.0780347	0.965317919	453.659971	0.298263284
453.2697095	0.929460581	1095.602276	0.260657539
249.1578947	0.921052632	546.4905385	0.327157366
303.4655172	0.896551724	748.6361308	0.261567347
965.4058074	0.962287535	2717.69251	0.335440095
493.4506667	0.988	1119.891807	0.250142553
493.813602	0.952770781	1160.022188	0.33903917
388.9118911	0.968302292	911.1962683	0.336163459
364.6928328	0.974829352	815.6264152	0.353133401
805.3557047	0.874161074	1805.803084	0.222890824
391.0507726	0.985099338	1155.21414	0.30427915
298.3194444	0.90625	709.7108241	0.249012259
220.3513514	0.913851351	480.1531502	0.321547986
443.5957447	0.972644377	971.841484	0.317236192

log.sigma.2.0.mm.3D_glcm_Homogeneity2	log.sigma.2.0.mm.3D_glcm_ClusterShade	log.sigma.2.0.mm.3D_glcm_MaximumProbability	log.sigma.2.0.mm.3D_glcm_Idmn	log.sigma.2.0.mm.3D_glcm_SumVariance2
0.187325962	185.8019324	0.012263393	0.961139047	73.2587982
0.311716757	124.1823982	0.054249413	0.971527818	51.21211361
0.19716407	411.850078	0.019995758	0.960153281	71.78838414
0.178943852	141.5891955	0.010385638	0.964481128	97.82493709
0.198947192	21.98518515	0.019483479	0.968670775	72.86740391
0.220445807	65.73472068	0.017010906	0.94957224	47.73627399
0.245312625	-168.1427728	0.030052368	0.96304402	38.45891349
0.174569293	60.54374224	0.010320573	0.943670803	98.93551563
0.188025679	76.77376094	0.00800334	0.962608552	81.48743945
0.243634094	219.7503796	0.02298863	0.978082983	49.33815759
0.214438495	-63.01723052	0.015332192	0.943944239	51.11966421
0.216135212	267.9133386	0.015711203	0.972965516	63.20996471
0.231062832	130.5790742	0.012041808	0.972868468	50.13982525
0.232937446	82.77172301	0.014112833	0.960884762	55.16102058
0.180887293	-49.61842048	0.012333161	0.964380199	55.34816569
0.420564417	119.8582821	0.122990403	0.987443585	30.64372857
0.205995905	10.34988722	0.011990778	0.956518354	69.51961689
0.255916879	269.841603	0.01502973	0.965675023	60.42324771
0.182492861	-7.527692232	0.012782911	0.95450423	87.39308512
0.347714813	86.48611503	0.053683216	0.974714298	35.2826925
0.290138917	111.5540806	0.050822642	0.976356992	38.07573007
0.195630928	270.1224452	0.009628828	0.96243848	67.73124953
0.260750985	191.9310209	0.019530339	0.967945996	48.96802685
0.163019814	-220.6086418	0.015815194	0.955212106	75.11079648
0.259107778	164.2874573	0.023518632	0.976333707	42.61555715
0.222693026	245.2248496	0.018654493	0.964670493	67.44969109
0.31969207	134.7163453	0.056249916	0.98188834	34.27284774
0.234913267	289.0617581	0.02072908	0.967502911	55.7708234
0.188041836	-186.8432966	0.012099859	0.948216215	87.15879806
0.317931988	138.8925701	0.060453083	0.976637218	47.23806646
0.248701425	174.7176899	0.023360333	0.977202149	43.38743864
0.30159711	139.7179669	0.037811991	0.977144984	42.32377514
0.196214326	152.6344292	0.008902758	0.966154824	79.03440269
0.159750816	-440.5042789	0.040723571	0.93929267	100.299151
0.151837421	-146.2311427	0.013749883	0.932996948	96.29707882
0.224530647	-6.615976954	0.009832013	0.964319253	60.37665972
0.228311046	323.1996546	0.023407921	0.96555738	72.39481285
0.227849284	37.47296541	0.016800309	0.969714755	49.25108755
0.211433154	-126.7786578	0.014810007	0.958195144	58.79789021
0.205281862	55.69933212	0.010836188	0.960560478	67.36106515
0.256522423	61.75951201	0.020291517	0.96822205	49.99898758
0.202748651	49.40047958	0.012188193	0.961365891	73.34065738
0.203922536	-40.81173807	0.013476787	0.953125288	73.19670597
0.195939279	84.24548616	0.012277426	0.956942596	84.00761902
0.187275508	-67.04670682	0.009845815	0.953342479	64.79842014
0.287225508	132.0790859	0.026251733	0.970764453	35.01117371
0.196095394	96.31357296	0.012087598	0.973368576	76.37542982
0.189326977	117.7639977	0.011878451	0.946735489	64.80806882
0.206027527	-758.2573401	0.037487831	0.951287079	86.43543871
0.177707482	-153.7741294	0.019737613	0.961395419	79.05376286
0.249095645	113.1364909	0.02650786	0.968653924	52.02562831
0.206564201	-2.465826256	0.01549248	0.938503731	72.69023486
0.24851396	181.6655882	0.021587653	0.978206323	65.63223781
0.232680728	39.96294282	0.016357825	0.962838725	45.67215158
0.34764997	104.3166869	0.073588776	0.978020192	44.96650885
0.149763891	130.8304618	0.008656597	0.950455365	99.6236853
0.165716576	9.696871184	0.008044158	0.968106829	110.0027207
0.174322051	-428.7490632	0.012674425	0.955329102	84.77799289
0.240665339	-1.855340321	0.039096543	0.968344679	56.85027076
0.248169779	30.44812911	0.018686272	0.981864928	39.11383877
0.206612372	214.5822585	0.012549343	0.960477309	69.4121304
0.23436191	368.2759439	0.021833457	0.974352396	78.52917494
0.311514249	56.63946886	0.040935562	0.977946312	35.38296488
0.373987337	-46.15966228	0.080489609	0.989065475	37.68449854
0.219460201	41.18619941	0.02053325	0.946953279	57.58196643
0.199999511	165.1176461	0.015469033	0.962993584	77.25551397
0.168932588	-173.1527329	0.015127659	0.937784551	83.124249
0.218008935	242.3496857	0.012850399	0.97425007	73.26638183
0.188402301	48.75881499	0.013782592	0.948859508	64.38851575
0.203192359	191.5948282	0.011234784	0.962036487	74.24108143
0.221519968	216.0433386	0.013097423	0.968876651	62.19197699
0.224915034	150.4458563	0.013831587	0.961261801	65.39603312
0.288303275	163.2548006	0.033602865	0.972680364	46.78757994
0.306550055	115.896143	0.042360539	0.978107411	39.42692823
0.288512912	165.1013958	0.029142313	0.98104338	52.14955467
0.156602721	-116.506755	0.008502841	0.943998647	94.77881388
0.206032425	34.71189918	0.011767544	0.961445772	66.97411334
0.199306254	273.5427825	0.01586394	0.947788961	68.36664994
0.20181496	114.436606	0.011690041	0.953636177	71.96388857
0.215065114	176.4090693	0.01289887	0.971145831	63.33208966
0.152664633	122.4419082	0.011688454	0.948446835	93.50173815
0.140533456	9.510242319	0.006771728	0.955177063	127.0877586
0.19012894	-44.35390915	0.013776892	0.976649473	94.31518615
0.191922035	-374.7005176	0.025446576	0.948560541	87.54217301
0.24692238	120.4805572	0.01534537	0.965550384	51.21747504
0.304483273	173.3451985	0.038136993	0.988687292	40.72073471
0.208692787	230.2838546	0.018113673	0.955165633	66.04622172
0.172336145	-171.1832074	0.009987939	0.95140018	89.40705546
0.238689655	-59.48830849	0.029083162	0.950307239	39.76483948
0.180088459	-449.7971899	0.035756377	0.900417576	83.70333621
0.257408618	-45.27367221	0.034585142	0.979199643	64.35548457
0.162733022	650.8949399	0.013802724	0.960794881	94.166025
0.261802237	7.844959356	0.057523371	0.962743192	54.02025243
0.253792377	104.3596742	0.022432382	0.97584886	46.16767478
0.271056632	81.18767731	0.024156658	0.976853566	41.03982863
0.13849825	391.6607405	0.017541245	0.948734146	164.0767084
0.216458607	-155.6164117	0.017092008	0.967520456	55.69774647
0.160201736	-322.7790676	0.023887927	0.934389207	99.31008994
0.229977363	18.85430846	0.018832123	0.964221636	49.88004131
0.23494947	551.7023029	0.023370201	0.970128092	71.23636774

log.sigma.2.0.mm.3D_glcm_Contrast	log.sigma.2.0.mm.3D_glcm_DifferenceEntropy	log.sigma.2.0.mm.3D_glcm_InverseVariance	log.sigma.2.0.mm.3D_glcm_Entropy	log.sigma.2.0.mm.3D_glcm_Dissimilarity
53.97704895	3.903080085	0.192936605	8.30009325	5.611667073
35.20153296	3.537165222	0.274766394	7.838294512	4.054008271
56.09761934	3.879241815	0.204402026	7.795881329	5.608159546
70.67979108	4.109612436	0.180634882	9.078788002	6.324163871
37.83269274	3.633993121	0.211465409	7.716045511	4.761799199
31.23522364	3.553723959	0.22774786	7.812771637	4.324788456
31.14265836	3.477813468	0.252196784	7.123342733	4.12844074
64.34148243	4.039342148	0.181490525	8.671538626	6.175410238
51.83568202	3.907636032	0.195381232	8.758030095	5.491397573
36.45657646	3.648178208	0.248760592	8.077418205	4.399257059
35.25448374	3.624423079	0.222035781	7.967599883	4.589270932
48.11491749	3.810143208	0.225112367	8.297822055	5.074433693
28.61281944	3.499137638	0.239959703	8.041440603	4.09887394
30.05942525	3.522914717	0.234093948	7.992639728	4.194952012
43.74653735	3.779660309	0.193201005	8.232044108	5.134062757
21.08628011	3.08367268	0.341863468	6.817285697	2.796943638
39.15166913	3.682802863	0.210369823	8.093397895	4.816382803
31.00005549	3.52569897	0.256321359	8.118000702	4.06043382
51.12240529	3.854472753	0.187389779	8.279067679	5.469406453
20.84386101	3.224866736	0.309269493	7.414400532	3.1343945
30.1657523	3.524612876	0.267995595	7.769639154	3.914798471
41.20136854	3.743419258	0.202795155	8.420403357	4.920351574
28.45735722	3.480891986	0.261845395	7.917862609	3.929409514
59.71885163	3.967858262	0.170606133	8.116706369	6.002958313
34.27575167	3.571619588	0.260191402	7.698516064	4.183319714
53.05063036	3.839199179	0.229104781	8.082896936	5.248712146
25.63195269	3.385014098	0.29359574	7.510218246	3.511691091
43.05999388	3.732365232	0.236531853	8.000406205	4.733611415
59.61054155	3.951078345	0.194060975	8.61476357	5.791917452
32.22214141	3.484384946	0.288317121	7.713027421	3.829765753
34.25643072	3.613695232	0.246774322	7.943222757	4.28334254
21.08842122	3.293081519	0.286477	7.732689258	3.348476183
57.81541704	3.963624386	0.200895531	8.703183834	5.691403765
74.59654675	3.840963549	0.17261153	6.522406168	6.671696653
74.55711426	4.065841827	0.156912109	8.050193278	6.682272446
34.09334587	3.619018239	0.229810496	8.233303935	4.403048761
53.60502873	3.894796999	0.218841184	8.504875999	5.323349365
38.92099059	3.73261538	0.221895439	8.298975896	4.69293976
37.16245045	3.600852039	0.212179731	8.05322295	4.718994166
48.83656766	3.842175757	0.212862544	8.348168283	5.288125505
39.60091869	3.667126411	0.257734555	8.066053398	4.485097974
42.34043861	3.761041407	0.20735621	8.27199752	4.960761102
45.91394941	3.80535963	0.202526616	8.434913819	5.161479742
54.86503921	3.919979265	0.197215743	8.511525033	5.539854839
45.47615275	3.827298038	0.191867043	8.523856063	5.229555346
23.9190613	3.360458717	0.279517667	7.512452931	3.565176789
56.90889008	3.973348854	0.198724018	8.869939546	5.622323142
42.50299542	3.747456893	0.19951641	8.070758812	5.059177169
55.35917545	3.736439466	0.21371864	6.678583774	5.546533245
53.65610219	3.884791782	0.189885337	8.046502122	5.630148573
36.17051152	3.654169325	0.241683754	8.236126136	4.415127695
47.02303267	3.792561856	0.210853503	8.110447615	5.182240054
41.87011942	3.714482434	0.247550656	8.399557345	4.666897756
28.39017926	3.490581237	0.240740884	7.761717261	4.089422026
30.14608393	3.385257408	0.30355856	7.641874958	3.620450003
78.87569758	4.178824414	0.156551975	8.817489952	6.901030964
74.83701346	4.115273864	0.172978219	9.032069128	6.604469577
64.07685666	4.013899979	0.181533207	8.602237036	6.046593411
41.49427911	3.731842013	0.22442246	8.010327707	4.698584081
24.27264158	3.344176436	0.256580111	7.54773976	3.684965546
43.59541653	3.785974798	0.212856979	8.416172328	4.9853297
50.60899234	3.856272048	0.226067736	8.675536935	5.094111512
24.92404714	3.362408294	0.290482338	7.605413723	3.519359077
26.01231453	3.257091028	0.313627989	7.245127987	3.27213623
33.42102719	3.540521286	0.213650467	7.292497126	4.428629939
58.05661534	3.949131144	0.206119212	8.609548382	5.644693587
63.6586037	3.988162725	0.175680571	8.088505199	6.152163448
45.08401131	3.797596734	0.221338997	8.519012602	5.009033138
40.69903257	3.718926156	0.198057858	7.928297709	4.96661711
56.12529436	3.940613944	0.206769164	8.622923023	5.598106996
42.76072899	3.745876486	0.225008135	8.35737314	4.855874064
37.31245399	3.670009774	0.228470462	8.186074506	4.587548509
33.520359	3.564050218	0.264510947	8.00281021	4.069491342
27.91767912	3.442622324	0.286072206	7.744623009	3.698791052
36.75804513	3.593639621	0.279660958	8.110629139	4.152417382
72.16763178	4.119072141	0.161446338	8.820704904	6.620296835
44.99954423	3.788668465	0.207163151	8.346374614	5.084632615
45.14714053	3.788040033	0.204318392	8.030329172	5.13344824
45.37116862	3.797012527	0.204996318	8.492921572	5.135142283
48.44558618	3.847401483	0.217519845	8.462110923	5.206388916
69.74955263	4.062858062	0.159961819	8.305746715	6.509838432
91.14860663	4.290865848	0.143092451	9.131806525	7.400068087
74.25274108	4.134112485	0.189626963	9.008245273	6.363614338
47.61406787	3.651711939	0.192905244	6.95350123	5.278212401
30.70122441	3.54223494	0.249186943	8.080834027	4.122701176
27.73719377	3.428738104	0.290142063	7.727954653	3.640796207
37.90319366	3.683650441	0.217232595	8.062667958	4.717202825
57.32286111	3.960023434	0.176336835	8.626130764	5.919085705
28.10675858	3.2444418	0.237326833	6.867210965	4.069658854
71.09365146	3.811157242	0.195218268	6.382062943	6.46415953
46.24519488	3.758475086	0.2535234	8.361588616	4.788507778
75.86348583	4.123590226	0.172737432	8.428382648	6.560417929
47.16232938	3.785342912	0.236923221	7.977559195	4.877430284
36.51392605	3.64713323	0.248380888	8.148012457	4.378259987
29.42423552	3.511329942	0.263397911	7.94430779	3.935111109
111.6216892	4.292793536	0.140816371	7.890627622	8.061118738
40.00992546	3.716904837	0.222176309	8.155591169	4.74497899
62.28148585	3.841913071	0.169242437	6.91810587	6.198198177
25.03439889	3.387328254	0.242569107	7.542198182	3.902875308
54.98569712	3.823293928	0.237871866	7.948073196	5.151603876

log.sigma.2.0.mm.3D_glcm_DifferenceVariance	log.sigma.2.0.mm.3D_glcm_Idn	log.sigma.2.0.mm.3D_glcm_Idm	log.sigma.2.0.mm.3D_glcm_Correlation	log.sigma.2.0.mm.3D_glcm_Autocorrelation
21.83866354	0.872625575	0.187325962	0.15591269	226.4356859
18.2381328	0.901325665	0.311716757	0.191513645	263.3214517
24.02453199	0.873569822	0.19716407	0.127753922	167.4366115
29.9325774	0.879572456	0.178943852	0.167884838	443.8869451
14.47104668	0.882441251	0.198947192	0.321224935	303.326792
12.24316919	0.854704717	0.220445807	0.209257812	130.4234076
13.46272911	0.878579529	0.245312625	0.116678972	323.890291
25.4661079	0.848471474	0.174569293	0.215084885	229.1171593
21.17122082	0.874847223	0.188025679	0.227778958	286.5238537
16.81800926	0.905993242	0.243634094	0.155459465	318.2245083
13.58053888	0.847964645	0.214438495	0.185009697	157.9438121
21.77954841	0.896203818	0.216135212	0.138351721	361.8705875
11.36000843	0.891001924	0.231062832	0.273361062	164.1365363
12.03883999	0.871859977	0.232937446	0.29410881	136.4467695
16.96045632	0.87478776	0.180887293	0.126646209	290.574297
13.05910162	0.939108325	0.420564417	0.192950133	245.1412841
15.03697323	0.864848717	0.205995905	0.279453825	165.1511912
14.00040027	0.88424085	0.255916879	0.320068523	154.3040297
20.27265131	0.862249834	0.182492861	0.266845369	279.7252055
10.7340357	0.905494416	0.347714813	0.255400122	205.2281281
14.65076952	0.905068176	0.290138917	0.11940146	252.9672535
16.37304103	0.873286149	0.195630928	0.246598077	191.4045952
12.61653586	0.886966632	0.260750985	0.263608749	170.4458111
23.05096279	0.861450076	0.163019814	0.123983057	385.5740028
16.44680918	0.904094328	0.259107778	0.111567586	181.9612528
24.85951965	0.884286697	0.222693026	0.124157034	333.5213498
13.12431747	0.918031354	0.31969207	0.148753081	336.7293276
20.22703268	0.88845532	0.234913267	0.132953919	211.8180972
24.61857362	0.857141241	0.188041836	0.195446416	292.2527795
17.27296762	0.910520538	0.317931988	0.192817051	187.7143646
15.59991711	0.903789568	0.248701425	0.122653538	244.5987933
9.56497988	0.904132214	0.30159711	0.334118471	138.7087265
24.84726318	0.882804866	0.196214326	0.162011194	344.7682767
28.64550639	0.842891751	0.159750816	0.160517135	317.8837596
28.26786988	0.834348225	0.151837421	0.136209037	261.5447766
14.326529	0.878521585	0.224530647	0.278980234	234.8282004
24.65624795	0.885344131	0.228311046	0.154608745	202.2227275
16.64922387	0.887788498	0.227849284	0.123176875	345.4029548
13.28005966	0.86685539	0.211433154	0.22918569	220.9609216
20.3976532	0.873167522	0.205281862	0.164935841	256.1215617
19.08774075	0.891132811	0.256522423	0.123259989	298.1072314
17.26715238	0.872805202	0.202748651	0.270152809	224.9720061
18.28240963	0.861732519	0.203922536	0.23024584	225.2722555
23.26784224	0.868733698	0.195939279	0.214292619	233.0757716
17.64491345	0.859246463	0.187275508	0.178664758	233.0085155
10.91630585	0.892869492	0.287225508	0.185401484	98.99589638
24.8246627	0.894978067	0.196095394	0.150700873	566.6084616
16.45840715	0.850562271	0.189326977	0.205890943	157.182704
23.27745888	0.86236017	0.206027527	0.233032498	281.6354204
21.16973473	0.871922061	0.177707482	0.200969025	272.4921866
16.28879182	0.88871281	0.249095645	0.182883882	236.2033437
19.22311129	0.844940565	0.206564201	0.21259553	175.3095275
19.49141413	0.907540544	0.24851396	0.222082686	490.3511883
11.46113471	0.87429522	0.232680728	0.235101867	153.5842656
16.37805309	0.915291722	0.34764997	0.211472395	241.8803806
30.47540827	0.855426517	0.149763891	0.125332547	316.6456581
28.81914283	0.883563805	0.165716576	0.199901359	477.8810522
26.0375726	0.865194607	0.174322051	0.153125515	370.3421566
19.07589685	0.888768464	0.240665339	0.160743345	247.9164141
10.47031377	0.910483749	0.248169779	0.23432852	524.4271627
18.31243784	0.872750382	0.206612372	0.230864339	223.248467
24.05410863	0.900644929	0.23436191	0.222543985	427.0583761
12.15746688	0.908906525	0.311514249	0.181065409	283.8447443
14.94201798	0.939892414	0.373987337	0.184923057	1236.024044
13.52272132	0.852495396	0.219460201	0.265169575	130.2617327
25.41053167	0.878879198	0.199999511	0.151920521	238.0053767
24.84082706	0.840717579	0.168932588	0.137239886	205.7962625
19.48842124	0.896767687	0.218008935	0.241431202	403.9068297
15.6969911	0.852410204	0.188402301	0.225105008	197.543151
24.06939217	0.876705501	0.203192359	0.147353531	269.1071418
18.23671654	0.887759476	0.221519968	0.191804612	364.9499967
15.86886147	0.874692858	0.224915034	0.273454326	185.3768691
16.50139949	0.899956671	0.288303275	0.17233622	264.1244089
13.96932528	0.909941179	0.306550055	0.175300181	255.4345982
19.24214609	0.917388821	0.288512912	0.176753663	485.0781762
27.01707035	0.84680928	0.156602721	0.142211108	314.9129519
18.49791463	0.873843153	0.206032425	0.199125037	187.0577154
18.25084212	0.854161288	0.199306254	0.202871457	171.5645542
18.10968107	0.862216475	0.20181496	0.227859119	205.9828093
20.83735545	0.891086453	0.215065114	0.139792987	254.9369505
26.3465865	0.852201921	0.152664633	0.14735125	268.8585393
35.46719353	0.861831703	0.140533456	0.171531857	492.8899173
32.76881612	0.902156341	0.19012894	0.128737108	1166.425408
18.78295286	0.855073052	0.191922035	0.303823265	244.0670456
13.27483991	0.882073388	0.24692238	0.247533268	184.6906451
14.24550189	0.934020086	0.304483273	0.1947331	514.5023191
15.31111667	0.863334888	0.208692787	0.268942803	158.4386943
21.33004918	0.856121532	0.172336145	0.225553515	348.6546458
9.436488273	0.857076719	0.238689655	0.170002869	184.4810243
27.58248063	0.805031503	0.180088459	0.087667482	232.5259553
22.7247126	0.911432402	0.257408618	0.166919056	801.4706968
32.01961116	0.873313391	0.162733022	0.110550361	349.9927983
22.91171735	0.883830224	0.261802237	0.074856514	363.8427547
16.89981995	0.902335635	0.253792377	0.1232467	294.3790328
13.68960438	0.90408677	0.271056632	0.169541242	267.4136431
44.11286254	0.855916059	0.13849825	0.19800674	539.9835327
17.17797924	0.88393814	0.216458607	0.171967853	365.4483327
22.44020713	0.834261072	0.160201736	0.238876125	227.2271748
9.540576616	0.874336344	0.229977363	0.331639258	168.7235942
27.91183297	0.89664148	0.23494947	0.136798278	309.1160467

log.sigma.2.0.mm.3D_glcm_SumEntropy	log.sigma.2.0.mm.3D_glcm_AverageIntensity	log.sigma.2.0.mm.3D_glcm_Energy	log.sigma.2.0.mm.3D_glcm_SumSquares	log.sigma.2.0.mm.3D_glcm_ClusterProminence
5.038948773	15.24473231	0.0039695	33.06018933	14981.32227
4.775697405	16.36616177	0.009968492	22.3821783	9225.323071
4.916584704	13.09560226	0.005894361	34.12489915	18329.701
5.311783318	21.32542477	0.002459264	41.6934176	26931.42065
4.96015512	17.37126837	0.0060564	28.08013756	17145.45736
4.737642662	11.23768457	0.005627372	19.74287441	5533.011766
4.479942461	17.98104097	0.010029017	17.61615733	5620.687647
5.253099026	15.10176183	0.003096205	42.27381648	22001.02995
5.181197071	17.0399949	0.002937736	33.73340842	19022.76011
4.773195726	18.05771917	0.006194081	21.63148159	9681.554377
4.790900221	12.40764986	0.004974251	21.59353699	6325.758998
4.943706606	19.28524099	0.004689485	27.79160768	13521.69595
4.811992279	12.62835564	0.004912345	19.99233933	8243.917491
4.848074984	11.411088	0.005071234	21.3444875	7664.678144
4.887056125	17.29248637	0.004249951	25.46052458	8973.936534
4.249237574	15.75148621	0.032475994	13.40481143	6059.871708
4.97241549	12.57243546	0.004492111	27.45633037	11174.16719
4.912074468	12.16266745	0.005079288	23.38373377	11186.77129
5.143362123	16.72613956	0.003982751	35.63985023	18692.37337
4.54779801	14.22297872	0.011106334	14.26510471	4149.564065
4.609862051	16.05218855	0.009538953	17.93058206	5453.742516
5.013655828	13.72141918	0.003795786	28.36545137	14731.96397
4.768612205	12.88142877	0.006420058	19.66291121	7633.288235
5.03636783	19.95196297	0.004594609	33.11280578	18342.59831
4.642577302	13.59369605	0.00766456	20.42757878	7172.938653
4.952149684	18.51626024	0.0051515	30.68974565	16250.53511
4.47139112	18.51296173	0.01191343	15.08264463	5957.998561
4.825777497	14.70955093	0.006321952	26.22578627	11644.41067
5.160319968	17.25669431	0.003410248	37.43539187	17606.4824
4.727212356	13.76036399	0.011274581	20.97032242	8870.004339
4.684601856	15.82335245	0.006706553	20.17545245	7293.016642
4.687852232	11.56164544	0.007896127	16.02119316	5995.57923
5.141812298	18.83765766	0.003118576	34.04857371	17818.64803
4.653834915	17.94252699	0.012924702	43.16466299	34694.19407
5.139842776	16.35955793	0.004436008	44.11851164	22611.62461
4.942403508	15.19816012	0.004092604	24.16159418	8503.441046
5.056118087	14.42199169	0.004325858	32.84114946	16698.80349
4.829040955	18.84139225	0.004507944	22.15588189	7612.53537
4.891800077	14.70358159	0.004853331	24.25905416	9896.328441
5.008519794	16.19395935	0.003872617	29.95240844	12163.27758
4.814883256	17.49976128	0.005762423	23.13573652	8225.938388
5.054301948	14.88652802	0.004058589	29.72635143	13258.51245
5.043488913	14.88755881	0.003741792	30.52460459	12193.2146
5.15276255	15.28747135	0.003582368	35.58038731	19515.38972
5.006134731	15.24004795	0.003384534	28.44771836	10796.37131
4.50798449	9.810829751	0.008507926	14.76750951	4093.784048
5.147229799	24.01987374	0.003013704	33.8592986	18078.66037
4.928485997	12.32828212	0.004468784	27.0188627	10252.59689
4.661154962	16.70963449	0.012088245	35.96952491	27539.60181
5.036647224	16.6710463	0.005112592	33.20299829	18814.3355
4.85634005	15.43429549	0.00509634	23.01435703	8548.431806
4.968846876	12.99493003	0.004583098	29.92831688	10724.98871
5.007793705	22.24717617	0.004577295	26.97890302	13322.7653
4.722385043	12.21740637	0.005721256	18.52688171	5635.537801
4.676478314	15.67216992	0.013796589	19.47437909	7332.689004
5.28382659	18.1582681	0.002716961	44.64031099	26527.95784
5.335932617	22.16118704	0.002462097	46.26082501	30674.94515
5.143651572	19.5451411	0.003651946	35.60367341	23963.04498
4.875031267	15.87143742	0.006450979	25.13921115	9932.656656
4.595356949	22.94092893	0.006925544	15.11713888	5178.06719
5.018059784	14.93014154	0.003985532	29.49342643	12814.00085
5.135168979	20.80460597	0.003819593	32.16933969	22702.08205
4.566453467	16.93129593	0.009421458	15.6438282	4504.319645
4.478633287	35.27202544	0.017727533	15.82864229	7429.550751
4.758823324	11.14426617	0.007627571	22.7507484	8283.595612
5.110717451	15.70510809	0.003775937	34.65029744	21546.16862
5.050158727	14.2709023	0.004480217	37.31176376	16822.08462
5.073032343	20.26193413	0.003707615	28.93336999	15495.4041
4.89400616	13.86169811	0.004919023	26.46179116	9302.448715
5.094080794	16.65944856	0.003456647	33.17855312	16125.67074
4.942874858	19.31091627	0.004346798	26.40324585	11333.483
4.969320539	13.39463844	0.004460836	26.09984826	10928.88744
4.763603556	16.39190139	0.006883827	21.02472444	7910.006997
4.632171523	16.09946702	0.00900636	17.5912107	5514.338932
4.804052474	22.16019605	0.008051301	22.37124748	12271.82423
5.236987132	18.05455649	0.002730941	41.94714954	22629.74847
5.007816873	13.6251123	0.003867052	28.99893722	11497.95156
4.92623847	12.90775525	0.004974534	28.82417016	12009.53607
5.061306724	14.25457172	0.00361263	30.36231351	13155.52535
4.980974537	16.21756589	0.003960977	28.51319472	12147.85974
5.179381675	16.57964166	0.003847715	42.2880392	22762.43393
5.458148402	22.52521748	0.002198529	53.58484519	38459.10729
5.272577892	34.59934179	0.002942362	41.95062808	27541.32754
4.797259739	15.38046768	0.009405194	34.51651712	23453.10664
4.823234232	13.42268411	0.005115384	20.73499523	7078.583988
4.634998743	22.81159039	0.008873992	18.05248639	8516.181427
4.920760123	12.3217657	0.004986812	26.24392244	11068.9135
5.191355771	18.83938629	0.003187753	36.73289654	18251.20801
4.434794361	13.47116773	0.010423055	16.96789951	4206.638561
4.503930171	15.13909605	0.014043017	38.69924692	18005.63674
4.949879836	28.5683527	0.006236291	27.29132653	17422.15076
5.143413538	19.06057593	0.003811047	43.68994881	31374.4055
4.802531228	19.36250822	0.009166081	25.25556911	10304.21523
4.762611936	17.41812497	0.005388894	20.9849489	7952.396227
4.69621032	16.49360701	0.006246885	18.30642582	5354.01053
5.388048347	23.62222495	0.00498648	68.65450747	65897.50155
4.868616669	19.35464645	0.004942012	23.40475631	11445.97905
4.819942419	14.82365246	0.009131704	40.9667973	25514.96522
4.752591594	12.74729179	0.006719127	18.72861005	7096.47551
4.921490315	17.90180536	0.00627165	33.41673228	25019.36759

log.sigma.2.0.mm.3D_glcm_SumAverage	log.sigma.2.0.mm.3D_glcm_Imc2	log.sigma.2.0.mm.3D_glcm_Imc1	log.sigma.2.0.mm.3D_glcm_DifferenceAverage	log.sigma.2.0.mm.3D_glcm_Id
29.77248862	0.828891863	-0.13134953	5.611667073	0.275066118
32.20651997	0.789784497	-0.118422737	4.054008271	0.384204566
25.57128991	0.911397898	-0.203542569	5.608159546	0.282716748
41.81333063	0.705937402	-0.074220386	6.324163871	0.264368129
34.32376075	0.914507576	-0.210766971	4.761799199	0.290184065
22.47536914	0.73952036	-0.097183996	4.324788456	0.310500769
35.8907647	0.862680041	-0.174782504	4.12844074	0.331467279
29.69450426	0.779929727	-0.103298875	6.175410238	0.26060568
33.41278677	0.688931061	-0.0723914	5.491397573	0.276573571
35.49656468	0.620835352	-0.059638751	4.399257059	0.327984025
24.81529973	0.706434227	-0.08455621	4.589270932	0.303242184
37.84611784	0.74920907	-0.096129463	5.074433693	0.301924104
25.19912243	0.63270803	-0.06497242	4.09887394	0.320646322
22.81745915	0.704751534	-0.08740979	4.194952012	0.321479471
33.92164988	0.754000442	-0.097062424	5.134062757	0.273679992
31.16096603	0.739382156	-0.111440977	2.796943638	0.478679969
25.09977639	0.793273537	-0.118402407	4.816382803	0.29554305
24.24340061	0.680130452	-0.077816429	4.06043382	0.340599276
32.90044283	0.878203996	-0.164920682	5.469406453	0.272874486
28.39804995	0.706558221	-0.095187761	3.1343945	0.419107686
31.68527867	0.568851574	-0.050358919	3.914798471	0.368020002
27.18488609	0.686456036	-0.07550437	4.920351574	0.287158584
25.71467678	0.623711419	-0.065854964	3.929409514	0.344944906
39.07503766	0.902123024	-0.187391202	6.002958313	0.253348705
26.8235416	0.727517929	-0.093897052	4.183319714	0.341815369
36.32637518	0.87218214	-0.162623383	5.248712146	0.3054218
36.58240345	0.659070619	-0.074320193	3.511691091	0.3939454
28.88780701	0.738952162	-0.09460503	4.733611415	0.319362407
33.7836064	0.730509856	-0.090644799	5.791917452	0.274905047
27.12620341	0.755896619	-0.105178585	3.829765753	0.390489784
31.13292946	0.595451412	-0.054996884	4.28334254	0.333084609
23.09928416	0.59483751	-0.06068349	3.348476183	0.381567095
36.84866276	0.71089806	-0.078675563	5.691403765	0.281764015
35.28196015	0.992532962	-0.487798276	6.671696653	0.245936429
32.00177295	0.951948198	-0.256903585	6.682272446	0.240442288
30.2158875	0.635275069	-0.063036387	4.403048761	0.313729794
28.10840173	0.688555738	-0.074865513	5.323349365	0.309805661
37.03063148	0.572891855	-0.04788832	4.69293976	0.313431039
29.36135924	0.751575345	-0.107361479	4.718994166	0.300365108
31.71630977	0.773428163	-0.104069001	5.288125505	0.290751624
34.38006796	0.745046683	-0.096791455	4.485097974	0.337028914
29.47600905	0.770584111	-0.103757649	4.960761102	0.29216648
29.55900151	0.695644598	-0.076976265	5.161479742	0.291534432
30.05017052	0.819631911	-0.12401603	5.539854839	0.282973629
30.21083429	0.639770181	-0.060769601	5.229555346	0.27791703
19.61802745	0.617935025	-0.064285246	3.565176789	0.368546389
47.40198872	0.614287837	-0.053422066	5.622323142	0.28238368
24.62306317	0.802663541	-0.121309326	5.059177169	0.280793904
33.08634644	0.981028184	-0.395373263	5.546533245	0.289509336
32.624831	0.88672256	-0.174829936	5.630148573	0.267252419
30.47868748	0.621484087	-0.060069101	4.415127695	0.332182177
25.98986006	0.754010569	-0.101256119	5.182240054	0.293236476
44.01838773	0.691629139	-0.077899743	4.666897756	0.32947665
24.43379116	0.762553238	-0.106794262	4.089422026	0.321903211
30.86557982	0.764778945	-0.111714229	3.620450003	0.414905525
35.29564164	0.843070216	-0.131530202	6.901030964	0.236884781
43.31376712	0.74654486	-0.089736088	6.604469577	0.251359177
38.21657086	0.769438456	-0.099355793	6.046593411	0.262474033
31.24502011	0.823414946	-0.132939669	4.698584081	0.324527409
45.63788021	0.715291289	-0.092082134	3.684965546	0.338356573
29.44732948	0.661055016	-0.067399367	4.9853297	0.294942301
40.99140998	0.677669471	-0.071508606	5.094111512	0.316360492
33.53940963	0.668914352	-0.078343934	3.519359077	0.387491204
70.23110641	0.788015839	-0.127868982	3.27213623	0.438741501
22.28853234	0.938156906	-0.254325027	4.428629939	0.310429515
30.54105752	0.778632571	-0.103060823	5.644693587	0.284979882
28.34484918	0.914181236	-0.20104009	6.152163448	0.256768324
39.8424429	0.667606465	-0.069432971	5.009033138	0.303320196
27.684102	0.853806959	-0.152491174	4.96661711	0.280866833
32.53148879	0.7099448	-0.079331908	5.598106996	0.287316946
37.95161598	0.635551336	-0.061940964	4.855874064	0.307422895
26.70875429	0.734918281	-0.091958988	4.587548509	0.312181238
32.29882716	0.653747621	-0.070530203	4.069491342	0.365798307
31.7840486	0.619869812	-0.06292168	3.698791052	0.38214917
43.87382117	0.629993992	-0.062351276	4.152417382	0.364664871
35.16982263	0.789092776	-0.105401648	6.620296835	0.243735092
26.94790648	0.744292994	-0.093219969	5.084632615	0.293708518
25.74760251	0.805834969	-0.123422529	5.13344824	0.287942621
28.23623062	0.670697008	-0.07243474	5.135142283	0.289906619
31.69930391	0.625417916	-0.058594688	5.206388916	0.299194379
32.42686407	0.901145724	-0.182536302	6.509838432	0.241850692
43.99415646	0.811299104	-0.111520328	7.400068087	0.227010465
68.15875806	0.737071519	-0.085092839	6.363614338	0.272740995
30.59117159	0.984430202	-0.401089496	5.278212401	0.282009236
26.79981041	0.595009537	-0.056287061	4.122701176	0.332825505
45.2219351	0.61524167	-0.061874908	3.640796207	0.381135445
24.60754461	0.788915891	-0.114580408	4.717202825	0.298263284
36.91034885	0.686439115	-0.072414677	5.919085705	0.260657539
26.94233546	0.937687737	-0.272397131	4.069658854	0.327157366
30.27819211	0.979224097	-0.411369269	6.46415953	0.261567347
56.46029737	0.724398058	-0.086968878	4.788507778	0.335440095
37.16995306	0.882750146	-0.164469519	6.560417929	0.250142553
38.05908646	0.832435802	-0.139063343	4.877430284	0.33903917
34.1738657	0.622095587	-0.060171927	4.378259987	0.336163459
32.52739579	0.563637183	-0.049188665	3.935111109	0.353133401
45.90374221	0.985955878	-0.370095259	8.061118738	0.222890824
38.02726457	0.734097535	-0.091040043	4.74497899	0.30427915
29.51753589	0.99038985	-0.446846878	6.198198177	0.249012259
25.49458359	0.862901688	-0.167262655	3.902875308	0.321547986
34.93114237	0.86604133	-0.159768774	5.151603876	0.317236192

log.sigma.2.0.mm.3D_glcm_ClusterTendency	log.sigma.2.0.mm.3D_firstorder_InterquartileRange	log.sigma.2.0.mm.3D_firstorder_Skewness	log.sigma.2.0.mm.3D_firstorder_Uniformity
73.2587982	202.2702713	0.33397051	0.050995514
51.21211361	126.3041267	0.391201714	0.074968086
71.78838414	190.3970604	0.780551089	0.058941579
97.82493709	221.144783	0.090629275	0.044974634
72.86740391	158.7627754	-0.121083926	0.060435596
47.73627399	177.1343651	0.076037032	0.064939086
38.45891349	101.67202	-1.050951276	0.096020921
98.93551563	253.2533302	-0.009094242	0.047434074
81.48743945	212.1145744	0.070664226	0.047460334
49.33815759	148.1534328	0.546779001	0.067733989
51.11966421	180.6201363	-0.284403838	0.063741997
63.20996471	171.4369888	0.417703033	0.056846804
50.13982525	161.2699947	0.235190211	0.064297464
55.16102058	178.9640522	0.124089593	0.062298188
55.34816569	159.9250374	-0.103301038	0.059467106
30.64372857	50.28318787	0.908361276	0.148961966
69.51961689	211.4607391	-0.08955235	0.057056182
60.42324771	170.4899273	0.481981439	0.060601402
87.39308512	228.5152283	-0.158535892	0.049916746
35.2826925	116.1472206	0.335336098	0.084129055
38.07573007	112.2909575	0.574499641	0.083568681
67.73124953	179.7318058	0.418698088	0.055571255
48.96802685	150.9925537	0.450920308	0.070146377
75.11079648	185.405304	-0.359631961	0.056496125
42.61555715	130.4385138	0.788456651	0.074423847
67.44969109	192.3943634	0.310773119	0.054161234
34.27284774	96.78810692	0.607762842	0.09060217
55.7708234	142.8509674	0.70634291	0.067051248
87.15879806	245.7219162	-0.176847394	0.04893226
47.23806646	113.7791243	0.594071745	0.080060801
43.38743864	133.7858734	0.669885175	0.074163265
42.32377514	127.3602619	0.519820157	0.078464662
79.03440269	220.2710037	0.148756002	0.049213312
100.299151	131.2790709	-0.747144524	0.07137574
96.29707882	243.177948	-0.249811974	0.045162578
60.37665972	192.1306362	-0.056413759	0.05831095
72.39481285	192.6683617	0.577408729	0.055624035
49.25108755	164.9026184	0.092492682	0.06087659
58.79789021	159.0620575	-0.36889135	0.063275126
67.36106515	207.0378342	0.122364822	0.052763493
49.99898758	146.8139553	0.222302743	0.063267602
73.34065738	205.4348297	0.000697069	0.055611649
73.19670597	216.8971882	-0.1572616	0.055988434
84.00761902	214.980545	0.03886471	0.049872219
64.79842014	200.4534817	-0.146798197	0.054357898
35.01117371	138.6187601	0.529266286	0.075978085
76.37542982	187.9872532	0.125635309	0.050633461
64.80806882	207.2311134	0.087672859	0.055150358
86.43543871	99.91311884	-1.124758236	0.087770105
79.05376286	173.2987595	-0.250997878	0.05992899
52.02562831	150.8799992	0.34512313	0.064094026
72.69023486	232.1768684	-0.19728717	0.057858345
65.63223781	174.5020599	0.291206029	0.05711073
45.67215158	154.912365	0.049712751	0.065657737
44.96650885	91.55189896	0.487891298	0.096209126
99.6236853	247.842392	0.058674863	0.042639466
110.0027207	262.6930466	-0.022318195	0.042719144
84.77799289	174.5838165	-0.70588919	0.056865782
56.85027076	165.8902378	-0.031109046	0.061892918
39.11383877	143.5959396	-0.309274979	0.072849576
69.4121304	205.5681343	0.278028824	0.05520537
78.52917494	187.1721439	0.468495096	0.05385357
35.38296488	109.4207735	0.269843261	0.086052723
37.68449854	90.79669952	-0.414831642	0.097227125
57.58196643	170.2752838	-0.077482052	0.061861581
77.25551397	169.6826172	0.314644388	0.055239449
83.124249	226.6601009	-0.359077255	0.053299492
73.26638183	208.961647	0.184955459	0.05214822
64.38851575	209.785017	-0.167442988	0.056858171
74.24108143	205.3849754	0.266503359	0.051774168
62.19197699	191.2397614	0.40850053	0.057590244
65.39603312	200.317627	0.091506406	0.057381647
46.78757994	132.6163359	0.578610997	0.07069418
39.42692823	112.6358328	0.49764829	0.082229471
52.14955467	110.23806	0.419554031	0.077493034
94.77881388	251.7048855	-0.194770606	0.044521846
66.97411334	203.739769	0.019003908	0.054721168
68.36664994	224.0508862	0.285388345	0.05628057
71.96388857	214.1604481	0.09183053	0.05279886
63.33208966	195.1315689	0.34840431	0.05631111
93.50173815	267.0593224	0.030202831	0.045784518
127.0877586	279.9817963	-0.145167929	0.039488267
94.31518615	201.5228443	-0.023293772	0.050092723
87.54217301	135.8056183	-0.653158089	0.066159334
51.21747504	169.3372231	0.297456498	0.064068408
40.72073471	112.3628941	0.784534478	0.084479669
66.04622172	211.6625767	0.216998497	0.056353712
89.40705546	248.5631542	-0.373756368	0.052236389
39.76483948	160.4846153	-0.362639711	0.07044104
83.70333621	199.2605286	-1.042441673	0.084263039
64.35548457	161.8925629	-0.235699118	0.061108026
94.166025	243.2255726	0.526630937	0.045157252
54.02025243	160.1831074	-0.017914736	0.066063243
46.16767478	152.0506458	0.336050627	0.064050071
41.03982863	140.3194008	0.372310778	0.07064348
164.0767084	325.0945129	-0.067499181	0.0403537
55.69774647	158.9001369	-0.507924122	0.06169792
99.31008994	179.3738213	-0.577251324	0.055456408
49.88004131	144.7844296	-0.160797744	0.069624968
71.23636774	149.1295738	0.913861317	0.060261129

log.sigma.2.0.mm.3D_firstorder_MeanAbsoluteDeviation	log.sigma.2.0.mm.3D_firstorder_Energy	log.sigma.2.0.mm.3D_firstorder_RobustMeanAbsoluteDeviation	log.sigma.2.0.mm.3D_firstorder_Median
114.8904037	13714365.89	84.38485163	-16.84065247
84.25793358	26181586.75	54.2715107	-11.13979912
111.0004425	8523797.427	77.19850828	-36.17207336
125.9418313	64368249.34	90.30898292	13.97071552
101.9144973	6163923.079	68.30999768	15.14780331
93.75123081	8137201.422	71.49753346	-23.51622772
70.97483478	3118838.782	42.81286917	14.35919857
134.2223553	24186029.49	104.3076182	4.788377762
119.1477675	43941644.49	87.2622771	-12.39908981
91.26189751	43152758	61.03511311	-31.51489544
96.82797486	10829379.26	74.3036915	0.789562106
105.7649504	26669222.31	72.70977004	-24.85098171
90.49260359	25301635.1	66.0912418	6.188875198
96.77551274	14568405.8	73.67254571	7.409549713
96.53475152	13342866.98	68.0976873	16.6017704
53.84482207	48700765.86	26.64810904	-3.49053216
109.4926029	11534641.01	84.49928602	6.604681969
96.52332212	32718413.36	69.58624356	-17.36831379
119.8161226	12234003.6	88.47174352	30.33103085
70.15277863	23393355.98	46.95786684	-4.842360973
76.85534433	56266268.61	48.85565033	-25.54556179
103.9023781	28621308.64	73.93332424	-0.375304461
87.06037512	32453509.81	60.92759634	-26.80164909
112.1077372	9437304.745	78.01880491	35.81947708
84.78943145	15458177.44	56.1322142	-45.50614738
111.1579553	13711535.86	78.29697271	-33.06297874
69.45171922	31944682.45	42.73320453	-10.14874649
95.05109106	22737978.02	61.81156432	-40.73760605
128.3492712	39121893.69	100.6082505	36.46257019
80.75910323	31689344.13	51.08697202	-19.65563583
84.44025708	38913287.2	56.34483713	-37.03041077
76.74431781	56924403.57	52.16996473	-8.325203419
119.1485195	42006342.64	88.84895693	7.5179286
113.8095752	2412558.138	68.0693086	42.95842361
135.3269793	8532942.132	100.9367346	28.86904144
102.0394288	28870659.8	78.73078142	2.745867133
109.8824789	54883290.71	77.59965114	-25.59373474
93.69410807	45813007.55	66.32151817	-4.984143019
94.86645196	11591262.92	67.04025496	29.69552994
110.9533363	18834403.46	82.75817011	-9.245306969
91.14491562	19946550.98	62.03134976	-12.89441824
111.7666257	16505820.04	85.46759208	-3.696082115
113.8998593	28731710.51	88.98088358	2.276381969
120.7110289	21525549.97	89.28654999	10.46185303
107.4583033	30975145.88	80.56701281	6.60014987
79.27086382	21590045.7	56.6222134	-24.73581696
112.1711129	85173013.63	78.5048824	-0.641317487
109.2168778	9731430.126	83.57058517	-12.40642071
96.87899467	2434638.518	53.30135438	36.50459099
111.3806513	9749091.378	77.49714596	36.10433197
89.56345577	44397013.59	61.50503672	-11.54369211
119.8532827	15446214.69	97.67198352	0.312865794
100.0663252	59998193.17	70.98569298	-10.7472353
86.94981212	6624596.444	63.64849192	2.604707003
71.54561652	64451717.46	42.88084182	-5.403566837
136.4618291	23433923.39	102.5487378	-15.28577328
139.4814454	60123393.1	106.2129365	5.274323463
113.8648181	27579233.89	74.99499089	35.14412689
96.20647361	12464821.76	65.81286698	-13.27707195
81.42782005	9009257.084	59.49238099	-12.50407887
111.2143351	37081042.29	84.16296727	-32.01608276
107.4394644	102014486.7	74.4683454	-3.773471832
70.03233657	26896076.4	45.36146226	1.020049512
68.5485358	25289973.76	41.77818226	-4.144472599
97.49116696	3087939.17	72.24273009	-12.03724289
105.9657265	30351339.1	70.43032476	16.33638382
122.2040017	8519675.509	90.89149598	14.40423965
112.3529673	58016908.97	84.08832627	-10.68592262
107.6300568	7413737.643	83.15033423	-15.49532032
113.1856228	38809843.06	81.73404808	-16.42289352
104.2827413	49557784.07	75.82606818	-26.03292179
106.9706765	17961155.77	82.35790455	-18.33128929
83.60276583	61462019.18	55.62031987	-13.18540287
75.14646984	67438742.63	48.92232104	-20.15971565
80.59073093	116327591.1	48.43954987	5.957009315
134.0916845	28175113.66	101.9006973	2.362899065
108.305388	18829088.75	81.70171314	-15.35409164
115.1967263	11295537.21	89.88693747	-52.87628174
112.0802155	32584265.23	84.8495428	-36.4825573
107.2869901	48673316.92	79.0523067	-32.9751873
138.1250113	14085305.4	106.2323074	-36.54566383
152.2838013	43406492.98	115.6403173	12.72023392
117.5082248	64911062.39	79.74284977	11.89819956
100.7760103	2704708.324	61.77025849	40.38646698
93.21786902	42666669.62	68.93570534	-15.14329433
72.51643181	53177604.25	46.2154205	-9.760742664
110.5126091	13217783.84	84.68628858	-35.0194912
128.2206089	34211724.76	100.5068906	25.54094505
86.03293326	1938529.938	64.50278842	25.5066185
123.2053705	2409867.015	88.99690132	43.01110458
97.06252376	65265360.35	64.68629499	-3.547105312
136.3633616	20497520.31	97.64757908	-54.95772552
94.98154348	15788116.17	63.251655	-9.655562401
89.22455772	52176714.04	62.37336225	-9.803514481
82.66593312	63073204.63	57.60240041	-15.30398417
179.3188732	11122755.75	137.5561175	-22.21004295
97.06754875	18803759.39	66.23635747	25.74496841
116.584014	2635539.172	78.55035765	42.08930588
84.61330253	4435916.443	58.3603063	29.75474167
107.8061408	17259357.89	67.56493685	-37.3457756

log.sigma.2.0.mm.3D_firstorder_TotalEnergy	log.sigma.2.0.mm.3D_firstorder_Maximum	log.sigma.2.0.mm.3D_firstorder_RootMeanSquared	log.sigma.2.0.mm.3D_firstorder_90Percentile	log.sigma.2.0.mm.3D_firstorder_Minimum
370287879.1	492.8893433	139.3752038	178.4472885	-373.5688782
706902842.3	411.9223633	112.2741391	153.7628571	-394.3044739
230142530.5	546.0161133	140.143092	194.2965057	-318.8163452
1737942732	535.3439331	156.2951634	213.1914246	-498.8410034
166425923.1	393.9523621	131.0332102	156.9976532	-415.1544495
219704438.4	263.2397156	112.4946961	118.5569748	-286.0191345
84208647.11	224.1868439	96.20142663	88.56531677	-426.5862732
653022796.2	391.6911621	155.3632968	186.7499634	-359.5439148
1186424401	431.3444519	145.3820901	169.2445038	-401.0517578
1165124466	502.4283142	118.0221637	152.1333954	-446.6446228
292393239.9	242.7849426	114.4324054	119.1386826	-314.7623291
720069002.2	501.0018311	134.1470337	181.067308	-453.1353455
683144147.7	469.7522888	110.4510676	144.2653458	-289.6276855
393346956.6	354.6858826	114.1524175	153.690773	-254.9894409
360257408.4	423.2086487	121.0222965	161.1290436	-398.8941956
1314920678	591.5078125	83.72771633	100.5472839	-359.6410522
311435307.3	351.5987854	128.5506657	139.2236969	-318.6429138
883397160.8	381.3308105	118.4491216	161.4160187	-288.7756653
330318097.2	363.5354309	144.9849121	198.437883	-376.8910217
631620611.5	310.1383362	91.38811308	123.3465652	-331.3687439
1519189252	432.5672302	103.7821046	136.1906235	-394.6949463
772775333.2	433.5527039	128.9972702	178.9431183	-318.5031128
876244765	354.2356262	109.0109153	152.4508514	-303.1495667
254807228.1	368.0626526	140.0720393	172.1766663	-464.287384
417370790.9	544.309021	113.3093898	131.9755203	-330.6287842
370211468.1	402.8935852	140.5605075	172.5736435	-455.9385986
862506426.1	446.8258972	96.00304508	121.6169052	-432.6981201
613925406.5	469.2563477	125.9658513	161.6657532	-367.2747498
1056291130	367.2651978	148.880418	198.2407349	-391.2413025
855612291.4	507.9544678	111.652603	124.8103714	-326.728302
1050658754	508.6187134	110.6378419	139.8969666	-395.7707825
1536958896	428.484375	97.97649473	142.1814087	-259.9104004
1134171251	522.5444336	143.7436757	185.5367065	-431.9915161
65139069.72	339.4348755	152.3078278	172.7363327	-441.5001526
230389437.6	333.9630127	160.3172883	202.437262	-377.1213989
779507814.6	331.5793762	119.5508504	146.2523453	-354.659729
1481848849	563.6079102	138.0460002	193.6867386	-331.6300659
1236951204	395.8071594	116.8726449	157.8267563	-432.811615
312964098.7	347.7041321	117.820847	141.3996887	-340.3761292
508528893.5	406.7660828	133.9948758	153.8357819	-392.8659668
538556876.5	376.7317505	116.3282417	162.5789841	-409.0065918
445657141.1	387.9660339	131.6049131	149.6396149	-352.1186523
775756183.8	330.2596741	132.2799934	147.8510117	-373.6331177
581189849.2	433.2763062	144.9156187	182.3570557	-364.431427
836328938.7	327.7383423	128.4277536	150.3023544	-368.1236572
582931233.9	419.1419983	98.43945204	134.4280441	-248.8076782
2299671368	523.1633301	141.2994858	191.3909836	-556.0782471
262748613.4	323.5221252	129.1974842	151.994458	-303.9407959
65735239.98	338.6977844	134.7922259	138.9618256	-407.6397705
263225467.2	456.8572083	140.0566957	155.3529053	-394.6781616
1198719367	400.6031494	113.8701784	148.1458298	-359.0019226
417047796.6	252.8942871	136.6653062	139.803067	-325.1935425
1619951216	480.2546997	125.3085103	162.4693146	-538.3151855
178864104	347.4099731	105.8731838	147.2443542	-283.5158997
1740196372	489.6340332	99.4016419	140.451062	-357.3665771
632715931.4	451.1877136	163.2781479	202.518515	-436.2893066
1623331614	583.5360718	165.577921	195.7055145	-540.9663086
744639315	387.1213989	146.0467297	166.2731354	-455.3841248
336550187.6	425.6521606	123.7459044	147.5900146	-386.1238708
243249941.3	286.0875549	101.1245346	122.6604691	-569.8294067
1001188142	397.9699097	132.2849654	170.0978027	-365.3925781
2754391141	547.5995483	136.4645698	175.7460464	-493.2356262
726194062.8	394.4510498	93.29647284	128.8853363	-383.3152466
682829291.5	297.5874023	96.23063228	126.3351517	-860.480896
83374357.58	256.1752625	119.2901271	138.9515991	-291.1013794
819486155.7	560.3156128	139.7987218	199.4249603	-349.1214294
230031238.7	327.2090759	147.0494539	151.8106308	-364.8696594
1566456542	488.3146362	134.7119042	170.5720215	-482.4758911
200170916.4	263.6795349	128.9291671	128.6438255	-363.0588379
1047865763	493.8965454	138.9200427	190.4590607	-395.9830017
1338060170	387.4971924	127.6789045	178.7165054	-474.8619385
484951205.8	354.4762878	126.1305149	142.051413	-329.427063
1659474518	415.0460815	109.0541405	143.1146301	-379.9538879
1820846051	432.7550049	100.2073064	132.165863	-383.0524902
3140844960	521.8344727	112.5693159	145.9732208	-508.5236511
760728068.8	360.8299255	158.8915278	181.2178879	-434.4848938
508385396.2	433.9620056	129.8338886	143.4007874	-339.6237183
304979504.7	319.7515259	136.0781912	164.4757034	-329.5210266
879775161.2	334.0122375	135.9110212	154.0974854	-355.8464661
1314179557	567.4248047	130.7759922	165.3879166	-376.9030457
380303245.7	375.9095764	167.1736657	172.6198395	-431.170929
1171975311	486.6280823	179.9131719	227.481369	-545.578125
1752598685	509.796936	152.4760566	214.1722473	-823.961853
73027124.74	320.2820435	136.5765751	177.8546143	-351.624115
1152000080	365.4870605	112.1709706	159.6162872	-310.60849
1435795315	639.6209106	98.54456213	122.9517746	-534.0587769
356880163.6	333.0527039	130.3435437	160.8812195	-322.2049561
923716568.5	322.8837891	148.4709975	159.6345963	-458.1195679
52340308.34	218.0591736	102.9225912	137.159964	-309.0754395
65066409.41	136.0791473	151.4962522	101.3582977	-418.9855347
1762164730	415.9634705	128.1364842	162.7020569	-684.5469971
553433048.5	515.3703003	170.3916515	216.9843292	-493.1727295
426279136.6	342.2135925	124.2910076	159.4588409	-450.2199402
1408771279	486.3234863	112.9475223	138.149617	-406.4559326
1702976525	436.9952393	104.2278869	138.7423859	-390.0055237
300314405.3	455.5267334	209.6746562	240.9451294	-575.6443481
507701503.5	369.309845	124.3527962	158.0834579	-436.0197449
71159557.64	313.0819397	146.380148	173.2933868	-364.7818604
119769744	300.6835327	108.1862515	143.537381	-293.1362305
466002663	527.0333252	146.2431557	195.6730896	-438.4146729

log.sigma.2.0.mm.3D_firstorder_Entropy	log.sigma.2.0.mm.3D_firstorder_StandardDeviation	log.sigma.2.0.mm.3D_firstorder_Range	log.sigma.2.0.mm.3D_firstorder_Variance	log.sigma.2.0.mm.3D_firstorder_10Percentile
4.457178616	139.2332747	866.4582214	19385.90479	-173.0931702
4.119020761	112.1691686	806.2268372	12581.92239	-148.1308899
4.351718977	139.8727604	864.8324585	19564.38911	-166.2531631
4.667860116	155.5008834	1034.184937	24180.52474	-187.6259003
4.329643089	130.9389366	809.1068115	17145.00512	-165.8699738
4.105092374	110.3860939	549.2588501	12185.08973	-161.944455
3.78684357	96.08177283	650.7731171	9231.70707	-136.9877716
4.553098675	155.268789	751.2350769	24108.39685	-212.8374374
4.560074959	144.7193947	832.3962097	20943.7032	-207.6993042
4.205653864	117.4162753	949.072937	13786.58172	-142.7203964
4.124563462	113.4410243	557.5472717	12868.866	-170.9519135
4.407139544	133.9866008	954.1371765	17952.4092	-157.4487305
4.150323885	110.219265	759.3799744	12148.28637	-133.1440948
4.173706473	114.0638363	609.6753235	13010.55876	-142.0162216
4.292566004	120.5344357	822.1028442	14528.5502	-142.4497986
3.462459163	83.68501312	951.1488647	7003.181421	-90.79609833
4.294076296	128.1002727	670.2416992	16409.67987	-190.8375244
4.233043074	118.4442946	670.1064758	14029.05093	-145.4683365
4.504864844	144.4913543	740.4264526	20877.75147	-177.1464905
3.863980322	91.38007566	641.5070801	8350.318228	-114.8797607
3.978455257	102.7781367	827.2621765	10563.34538	-130.044223
4.369398377	128.6782551	752.0558167	16558.09332	-149.9964798
4.093802088	108.7168574	657.3851929	11819.35509	-134.5358887
4.435961202	139.6839932	832.3500366	19511.61797	-184.2021484
4.073540758	110.4274617	874.9378052	12194.2243	-145.3215485
4.455543973	139.2519264	858.8321838	19391.09901	-175.30289
3.879252564	95.94632998	879.5240173	9205.698237	-112.4511681
4.239847672	124.1781494	836.5310974	15420.2128	-162.2504791
4.503508872	148.3739274	758.5065002	22014.82233	-187.8276672
4.057999112	109.7900032	834.6827698	12053.8448	-159.248967
4.077258542	109.1426833	904.3894958	11912.12532	-138.48703
3.961086128	97.7811117	688.3947754	9561.145806	-107.5396019
4.522069938	143.6773009	954.5359497	20643.16678	-179.6837097
4.2420803	151.8491595	780.9350281	23058.16724	-201.3329483
4.624481865	160.2905008	711.0844116	25693.04466	-223.9483475
4.233957057	119.4263869	686.2391052	14262.66188	-165.7804565
4.410122367	137.8591742	895.2379761	19005.15191	-167.8332428
4.255874821	116.8511699	828.6187744	13654.1959	-138.8255463
4.197902954	117.2920932	688.0802612	13757.43512	-161.3439911
4.412430052	133.6915347	799.6320496	17873.42644	-183.5997711
4.229784033	116.2064384	785.7383423	13503.93633	-148.7986816
4.347115925	131.1755714	740.0846863	17207.03053	-182.6563873
4.336684908	131.8404871	703.8927917	17381.91404	-189.3671432
4.528362478	144.9085148	797.7077332	20998.47767	-190.9235931
4.362156002	128.1650321	695.8619995	16426.27546	-181.9826874
3.937667633	98.08523648	667.9496765	9620.713616	-124.5404381
4.531625818	141.1223266	1079.241577	19915.51106	-166.2772522
4.303763187	128.3221766	627.4629211	16466.58101	-187.2739105
4.01486554	134.745721	746.3375549	18156.40934	-180.1168381
4.379180481	139.8705786	851.5353699	19563.77877	-207.2888458
4.209970626	113.8321082	759.605072	12957.74887	-141.9207108
4.259929619	134.7676499	578.0878296	18162.31947	-209.0771393
4.341602457	125.2553162	1018.569885	15688.89425	-162.7807007
4.084863543	105.7415519	630.9258728	11181.27581	-128.3526154
3.88727201	99.38763682	847.0006104	9877.902352	-124.8988403
4.700583099	162.8506458	887.4770203	26520.33283	-223.875058
4.714722092	165.4576291	1124.50238	27376.22701	-222.4615265
4.467956437	145.965715	842.5055237	21305.98996	-212.8715851
4.294850075	123.0897917	811.7760315	15151.09683	-175.1053314
3.979028909	100.9367701	855.9169617	10188.23156	-121.7177505
4.365505016	131.7609157	763.3624878	17360.93891	-170.6528076
4.453532399	136.3701697	1040.835175	18596.82317	-160.2694611
3.885872819	92.87214192	777.7662964	8625.234745	-101.765065
3.828441538	96.1064018	1158.068298	9236.440467	-123.1425323
4.177743177	118.5440001	547.2766418	14052.67995	-183.512915
4.463752885	138.0643528	909.4370422	19061.76552	-150.0998901
4.452396022	146.2664384	692.0787354	21393.87099	-231.0161652
4.433680216	134.7051467	970.7905273	18145.47655	-169.5352966
4.287344319	126.3618873	626.7383728	15967.32655	-195.0356293
4.476280661	138.917678	889.8795471	19298.12126	-163.7147675
4.32037076	127.576884	862.3591309	16275.86133	-156.2458099
4.287687488	125.212968	683.9033508	15678.28736	-180.2808502
4.11416273	108.9622011	794.9999695	11872.76127	-133.0506226
3.957342322	99.52052861	815.8074951	9904.335614	-128.0331726
4.116181945	112.1708642	1030.358124	12582.30278	-123.6055046
4.641480829	158.2499384	795.3148193	25043.04299	-226.5178757
4.353675371	128.7285934	773.5857239	16571.05075	-185.0767456
4.312834019	133.5666246	649.2725525	17840.04321	-182.8243637
4.394400749	133.4323882	689.8587036	17804.20221	-194.5448608
4.355458913	130.2202762	944.3278503	16957.32034	-170.8798523
4.621351341	162.5733089	807.0805054	26430.08076	-249.1969208
4.826698879	179.9128878	1032.206207	32368.64719	-242.116333
4.605524618	151.5590594	1333.758789	22970.1485	-175.6638458
4.253344844	135.1309908	671.9061584	18260.38468	-180.6044586
4.153871859	112.1419973	676.0955505	12575.82756	-131.2619019
3.928972359	98.54427451	1173.679688	9710.974039	-106.4309883
4.32480312	129.5752468	655.2576599	16789.74459	-167.1070023
4.443410648	148.1276617	781.0033569	21941.80417	-218.4125793
3.969236027	102.2876545	527.134613	10462.76426	-124.8013382
3.941766637	147.5913912	555.064682	21783.21875	-258.6622925
4.349220057	128.1292621	1100.510468	16417.10781	-155.5321503
4.685839085	168.1479633	1008.54303	28273.73756	-218.9629135
4.269472451	124.0439948	792.4335327	15386.91265	-165.6531982
4.202599751	112.7560319	892.7794189	12713.92274	-143.8199219
4.074186539	104.1908069	827.0007629	10855.72425	-126.4863625
4.871665504	208.5742468	1031.171082	43503.21643	-286.0021912
4.292626714	123.8210774	805.3295898	15331.6592	-155.8539276
4.405158672	145.9341941	677.8638	21296.789	-223.2238556
4.089483955	106.7379445	593.8197632	11392.98879	-130.447818
4.410804972	145.310601	965.447998	21115.17076	-178.3233368

log.sigma.2.0.mm.3D_firstorder_Kurtosis	log.sigma.2.0.mm.3D_firstorder_Mean	log.sigma.2.0.mm.3D_glrlm_ShortRunLowGrayLevelEmphasis	log.sigma.2.0.mm.3D_glrlm_GrayLevelVariance
2.690582131	-6.288294906	0.009950755	31.69374068
3.43636179	-4.85385551	0.005253932	21.92145532
3.679318266	-8.70040935	0.013265958	32.04124408
2.787044335	15.73700666	0.003555629	39.44588382
3.228177422	4.969612693	0.00744776	28.01746159
2.270429919	-21.67872029	0.015621243	19.65932773
4.740017837	-4.796604613	0.006792449	15.43861363
2.11081275	-5.41822258	0.010265506	38.81686362
2.555664131	-13.86538606	0.006482603	34.16391519
3.5504102	-11.94359249	0.004017162	23.04349528
2.154327299	-15.03028291	0.012627139	20.947126
3.195037959	-6.558769286	0.004218878	29.59789536
2.914925694	7.152060198	0.010309199	19.81011836
2.34664088	4.496182928	0.01677351	21.21350371
2.981198102	10.85569184	0.00637475	23.75452596
7.156859034	2.673772711	0.004495308	12.96246485
2.208399466	-10.75145511	0.014877293	26.53555169
2.79783483	-1.069334004	0.011260377	23.14164333
2.394049263	11.95296065	0.008394951	33.97305386
3.177511332	-1.212016721	0.005886541	14.30195368
3.665787751	-14.40068924	0.004894386	18.29432543
2.978052039	9.066553676	0.009552982	26.96714877
2.809801407	-8.001534769	0.008720578	19.57570307
3.10637839	10.41912732	0.005940625	32.03138034
4.386571108	-25.39278457	0.00912259	20.42096913
3.024038541	-19.13523617	0.005730298	32.13148451
4.800252238	-3.299458691	0.003825699	16.13427513
3.580140882	-21.14669925	0.0081771	25.65554938
2.070306374	12.27014801	0.0073576	35.6425603
4.201559337	-20.30908531	0.008315795	21.08906195
3.792013008	-18.12751307	0.005191008	19.94768912
3.257329358	6.18447359	0.011110231	16.11525103
2.689432998	4.367780382	0.00503512	33.80730379
3.749391167	11.81131484	0.017715355	38.27525384
2.258668599	2.930575073	0.01257559	41.59572626
2.176610191	-5.453802524	0.006741715	23.19071045
2.95772856	-7.179572016	0.008936753	31.55964436
2.968005838	2.240363473	0.003898152	22.76575691
2.759690673	11.14974791	0.009672581	22.18451902
2.58681543	-9.011121634	0.007676087	29.26419492
3.076125474	-5.321982305	0.00517009	22.86516951
2.324599492	-10.62179904	0.008417427	27.97472253
2.132093098	-10.77416453	0.009133507	28.14259082
2.521328963	-1.434881995	0.011517519	34.18584957
2.363292333	-8.210507927	0.008896596	26.77736316
2.814230966	-8.343386769	0.017018864	16.05602543
3.168173269	7.073444953	0.002416917	32.78318338
2.154313734	-15.01362433	0.013075482	26.63413404
4.43292802	3.540455736	0.022426845	30.27083783
2.938650804	7.217981249	0.008414818	31.61047695
3.107359831	-2.944257848	0.005956682	21.6465211
1.787265358	-22.695516	0.012607601	29.40516824
3.046936159	-3.650823797	0.002517792	26.17048164
2.521337892	5.277805444	0.011344223	18.06840781
4.024677529	1.668550051	0.004999282	17.15430346
2.346937884	-11.80765608	0.006795474	42.99066674
2.440878028	-6.310381431	0.003298889	44.14491587
3.291936266	4.863875636	0.006044968	34.8374363
2.978032732	-12.72603688	0.007742353	25.65425668
4.687667037	-6.159540128	0.004186172	16.63725515
2.402671522	-11.7632126	0.008889201	28.2471041
3.528611452	5.075001319	0.003080681	30.96452715
4.001775761	8.888031269	0.004497988	14.71596538
7.068779458	-4.888161317	0.001190392	16.59229853
2.263057195	-13.32120375	0.018969716	22.29209258
3.393749783	21.95261011	0.009639539	31.20348234
2.351241728	-15.15489716	0.019128036	34.72669541
2.600094927	-1.349294959	0.003234191	29.69209034
2.159442753	-25.60085133	0.012933174	26.07221276
2.739081953	-0.810551529	0.007009125	31.51252614
2.60108394	-5.103070492	0.003498805	26.59058309
2.298121001	-15.18615943	0.009480911	25.44598761
3.565512241	-4.477086029	0.004757922	20.17665664
3.557339922	-11.71190199	0.004717699	16.85816973
4.757426246	9.462985729	0.002464518	21.56964004
2.260078037	-14.26445306	0.007931889	40.58889314
2.442898527	-16.90526183	0.012535125	26.9546235
2.073055828	-26.02366055	0.011739783	28.78299391
2.228783456	-25.83802376	0.010913885	29.0183471
2.871935344	-12.04324636	0.006098907	27.84149168
2.185064567	-38.94809052	0.01163783	43.03441179
2.314973256	0.319744608	0.003916531	52.45191517
3.438833156	16.69728572	0.001295216	37.75453826
3.407885749	19.81858156	0.016556057	29.90354259
2.488984797	2.549328695	0.00823419	20.5559078
5.949521878	-0.238089348	0.002197033	16.68591951
2.25376526	-14.13134049	0.015650037	27.31733137
2.105254459	-10.09122991	0.004854499	35.36578464
2.545324334	11.41470603	0.012957873	16.80919876
3.003877036	-34.1744887	0.038312994	35.01483791
4.331557884	-1.360429724	0.002256613	27.81666451
2.901742459	-27.56043057	0.005272264	46.18418608
3.080287299	-7.832108421	0.004490969	26.27942415
3.40432781	-6.574196738	0.004401316	21.41835565
3.248347055	-2.779955431	0.004630269	18.24072496
2.320627854	-21.45332225	0.007448682	70.54277293
3.491526434	11.48732861	0.006274012	25.21234434
2.873101134	11.41747521	0.024150705	34.83963846
2.895912194	17.64302225	0.012510487	18.48367151
4.209590919	-16.48908195	0.005476955	35.62416328

log.sigma.2.0.mm.3D_glrlm_LowGrayLevelRunEmphasis	log.sigma.2.0.mm.3D_glrlm_GrayLevelNonUniformityNormalized	log.sigma.2.0.mm.3D_glrlm_RunVariance	log.sigma.2.0.mm.3D_glrlm_GrayLevelNonUniformity
0.010233827	0.050461638	0.071744655	33.6084532
0.005645643	0.067189115	0.208729041	122.3483339
0.013674559	0.057232676	0.068254714	23.45179833
0.003682399	0.044429282	0.071491496	110.293493
0.007625583	0.059645961	0.063424122	20.21550598
0.016240464	0.064100435	0.07420184	38.60222105
0.006955401	0.092554042	0.086440355	28.98277048
0.010611739	0.046919595	0.061997166	44.52763974
0.006724625	0.047173419	0.067252394	92.45949765
0.004244167	0.06523094	0.103873765	185.8663012
0.01312068	0.062947425	0.072126168	48.83197262
0.004397457	0.055527372	0.083299711	76.57581259
0.010762327	0.063559387	0.080642866	122.8940438
0.01751553	0.061482655	0.085297412	63.83552117
0.006543342	0.058697673	0.059971886	50.68815916
0.005061321	0.123753761	0.340612695	712.1897735
0.015421498	0.056611909	0.073991389	37.06679979
0.011922402	0.059571279	0.108090531	127.6499812
0.008646907	0.049500566	0.068958474	27.21307587
0.006402877	0.078360411	0.217586266	191.9465542
0.005246567	0.076204775	0.175030075	355.1642771
0.009914058	0.054913682	0.067582222	89.07277608
0.009209809	0.068108899	0.103155083	171.5220949
0.006050712	0.055636494	0.053823298	25.47703284
0.009612897	0.071273046	0.101078241	78.98944482
0.005923647	0.052683182	0.087926362	33.99763415
0.004123802	0.082292545	0.218283125	249.1070571
0.008567847	0.064524361	0.08849598	85.94453579
0.007591308	0.048524646	0.06513056	80.96482509
0.008874381	0.071546196	0.222240591	159.7508084
0.005498953	0.071089293	0.10795217	207.9969992
0.011859692	0.074919819	0.142669961	400.9023617
0.005248559	0.048694974	0.075675497	92.70986309
0.017786344	0.068981669	0.044774201	6.90291573
0.012815028	0.044896925	0.045179011	14.27411774
0.007043614	0.057876561	0.081319917	108.93661
0.009367043	0.053775939	0.112478335	142.8231184
0.004100027	0.059299071	0.108256083	183.124817
0.010022301	0.062442076	0.075792886	48.6743576
0.007944889	0.052181014	0.078240872	51.24402666
0.005425013	0.061213315	0.12084839	82.286791
0.008748123	0.055117624	0.070927547	49.29870627
0.00949299	0.055237915	0.076087119	84.84298548
0.011889619	0.049255357	0.074245901	47.35768238
0.009170992	0.0538535	0.064129291	95.51328774
0.018057185	0.073256794	0.118747177	148.8632042
0.002515462	0.049708106	0.078423906	198.4761739
0.0135523	0.054952427	0.057286954	30.42119839
0.023066264	0.083349225	0.065702033	10.53409401
0.008632022	0.058818849	0.059733755	27.71392979
0.006288337	0.061899776	0.116091716	194.2712806
0.013091772	0.05702399	0.069691077	44.31587728
0.002657156	0.055496635	0.117915471	194.2299131
0.011816847	0.065086196	0.077054307	35.90740606
0.005426611	0.085595336	0.218857011	489.2193446
0.006960087	0.042340124	0.051322986	35.55041981
0.00341704	0.04246884	0.063523963	88.10993167
0.006195688	0.055591251	0.063856874	67.94331095
0.008061901	0.057907843	0.118196652	43.08191619
0.004372553	0.072018483	0.09450779	58.79595645
0.009204715	0.054335442	0.074881077	107.876004
0.003239851	0.052336875	0.111939623	263.9650138
0.004807916	0.08117775	0.160957636	224.3805771
0.001277147	0.083421266	0.296975376	192.9070119
0.019779883	0.061331394	0.078404725	12.45296277
0.010012271	0.053973925	0.072333777	78.72378091
0.019595881	0.052515704	0.054529206	19.71435918
0.003391927	0.051576701	0.088713929	153.3043545
0.013231469	0.056383276	0.058969823	23.8564589
0.00726718	0.050947207	0.077343519	95.99298604
0.003681185	0.056485999	0.090163216	159.4908463
0.009906499	0.056645774	0.08372202	59.60533099
0.005087933	0.067314636	0.170192686	311.8888361
0.005073663	0.077198413	0.174122191	461.7279395
0.002629992	0.072809906	0.140359456	601.4894586
0.008129	0.044221739	0.055417063	46.93294138
0.013040722	0.054155881	0.07832267	56.56897841
0.012193215	0.055266172	0.064968641	31.77089021
0.011319277	0.052119949	0.074836686	86.18062
0.006380924	0.055229294	0.088100449	146.1807903
0.011819735	0.045233644	0.048115593	21.81186274
0.004008378	0.039186193	0.045906682	50.32202786
0.001336773	0.048812471	0.07543168	128.0992792
0.016760075	0.064669948	0.057533176	8.892759965
0.008664512	0.062996198	0.093334703	197.9966955
0.002377828	0.079659935	0.166525137	386.3753124
0.01612795	0.055284016	0.070061446	40.48920209
0.005022181	0.051674261	0.062113674	75.83035781
0.013296272	0.069591045	0.079830659	11.88401974
0.039026232	0.081803346	0.047094857	8.239250994
0.002342385	0.057679359	0.137925077	208.2938921
0.005411167	0.044515522	0.053065264	29.96568279
0.004706968	0.059197793	0.175103225	54.33160016
0.004659369	0.062115503	0.132654233	231.1050894
0.004943724	0.06827863	0.132562587	359.1529795
0.00753793	0.039417235	0.047586753	9.555593674
0.006449115	0.060259044	0.085424736	68.16119209
0.024867609	0.055027304	0.041410992	6.515213369
0.012871079	0.068428098	0.071740157	24.30499648
0.005704845	0.057816395	0.102324991	43.04525089

log.sigma.2.0.mm.3D_glrlm_LongRunEmphasis	log.sigma.2.0.mm.3D_glrlm_ShortRunHighGrayLevelEmphasis	log.sigma.2.0.mm.3D_glrlm_RunLengthNonUniformity	log.sigma.2.0.mm.3D_glrlm_ShortRunEmphasis
1.196332077	257.397292	598.8328427	0.959080806
1.515861357	266.7121949	1454.926551	0.912752132
1.192156574	201.1863348	368.296425	0.958732185
1.199034402	467.9866951	2221.259924	0.957431562
1.186465889	326.9017861	303.4109793	0.957574293
1.215475174	149.8083512	531.2926635	0.952000128
1.247774621	328.6280596	272.8954505	0.946721346
1.177446472	262.4962131	856.1074223	0.960637376
1.192915882	308.389344	1752.304035	0.957241015
1.287526182	332.4911469	2440.68722	0.940554792
1.211044462	178.8670181	686.8200509	0.952919748
1.239560891	383.873315	1203.33721	0.947658653
1.232258114	175.4601713	1692.022686	0.948949442
1.246115719	151.2989374	902.4393116	0.946150768
1.173370402	299.7927168	779.2553088	0.960892851
1.815112295	229.3734871	4187.092265	0.876277037
1.213513726	189.2760035	580.1126999	0.953045886
1.295313537	171.7259845	1838.005884	0.940492693
1.190606951	310.7629785	494.6633781	0.959432448
1.532622893	207.5845349	1952.266453	0.9113506
1.43405726	255.9700876	3834.91961	0.924495243
1.193487906	213.0929752	1450.294908	0.957099049
1.281141781	186.2244513	2171.431794	0.942846059
1.159430276	412.367608	416.4559187	0.963432096
1.2833658	194.419288	950.483301	0.940831963
1.245936611	367.8036865	563.1899035	0.947886683
1.534148764	326.2318639	2397.824306	0.909860716
1.248302534	232.5416983	1162.446462	0.947592935
1.187627288	311.4837813	1503.006803	0.959020679
1.525174862	192.8625814	1795.448727	0.915527869
1.290639041	259.0272418	2514.689736	0.941703136
1.37441828	146.2070624	4454.904779	0.928995032
1.217243295	366.217584	1680.692251	0.952156544
1.128685725	383.1376613	92.9616683	0.971428481
1.136958633	309.3381504	292.0788595	0.967380958
1.234618442	244.0456216	1646.655102	0.948612623
1.29104281	224.0404418	2300.024082	0.944391432
1.289207351	351.1004227	2659.323338	0.942524228
1.226918308	233.4863107	683.5849014	0.948718661
1.220252067	278.655433	867.8228082	0.952656574
1.325556466	302.6631099	1137.823408	0.935683847
1.207495042	244.5270697	791.8509474	0.953367887
1.221110835	243.3622234	1353.337599	0.951142959
1.211418974	261.536968	851.622198	0.953693453
1.186109152	246.1981903	1589.722383	0.958214262
1.325556783	114.9539479	1718.938913	0.934844181
1.221163062	572.849437	3526.216472	0.95235235
1.166825232	187.4171583	501.2184608	0.962139476
1.195008627	325.0095589	112.9913184	0.956120483
1.17320288	300.9043634	425.4526711	0.961015067
1.30824528	245.6253523	2680.711931	0.939233189
1.203915532	204.1775985	689.7505946	0.954177886
1.312406041	497.2245059	2990.461406	0.93909585
1.225391642	172.1210845	483.5766318	0.949653327
1.532962754	240.3192537	4562.669924	0.91215258
1.148192624	357.5939918	769.4436346	0.966554593
1.182407091	522.0211122	1867.595505	0.95952795
1.18493776	403.1605015	1097.468142	0.958653575
1.316818048	266.2041592	632.6353617	0.937486929
1.26233244	533.6392408	709.3633085	0.945588667
1.214633146	244.9942565	1754.443103	0.952785819
1.293693969	436.2498986	4350.359861	0.942935411
1.419546466	279.3563219	2270.71545	0.92292634
1.700654365	1128.563853	1758.00712	0.893376323
1.224767023	163.0299618	179.0441442	0.951223822
1.208121564	258.567567	1294.592664	0.954177767
1.157304705	246.7345261	342.3529449	0.964600516
1.246899102	428.9502587	2597.382063	0.948152021
1.171583325	228.4926459	382.5515274	0.961338763
1.218061809	291.3633502	1668.352343	0.953162303
1.251352252	383.2227054	2462.372911	0.94719219
1.235996843	209.5838705	922.129325	0.949358655
1.419830827	269.6453136	3840.75317	0.927013894
1.438489814	255.5316607	4886.005692	0.921716851
1.377609418	465.8029228	6826.760542	0.926506377
1.162083896	349.216516	963.7015472	0.963064323
1.22357552	208.7768351	920.2653036	0.951225827
1.19210762	204.7670802	512.3140658	0.955992679
1.215543387	229.4511033	1462.957259	0.952612176
1.245391904	272.4410605	2313.877054	0.948366152
1.142146538	321.3338752	442.7149134	0.96720053
1.136889948	542.800171	1179.849421	0.967788951
1.209160088	1155.662225	2338.825285	0.955694864
1.172361947	279.6549778	123.983787	0.960142513
1.258702417	197.3206672	2729.060678	0.945733091
1.44363883	484.3715487	3905.344198	0.916342014
1.199961009	189.5921253	653.610924	0.956265208
1.182015676	383.3694979	1318.206888	0.958828219
1.237617333	199.2688225	149.9855663	0.947609472
1.136545259	281.289128	92.98101509	0.968921144
1.351951677	783.9871618	3048.558364	0.934567698
1.153520004	414.0038371	614.4193946	0.965207459
1.419467672	369.3400734	766.9987332	0.930223966
1.343889671	300.0456532	3145.89876	0.93511333
1.353002475	271.9667137	4403.957381	0.931522301
1.138272469	611.6105671	223.4586952	0.968633424
1.242281516	362.2647528	987.4137675	0.947913298
1.121812366	280.8081141	109.9752631	0.971757451
1.211708411	182.3591094	313.361041	0.952064968
1.279562798	339.6985867	642.7927168	0.943350547

log.sigma.2.0.mm.3D_glrlm_LongRunHighGrayLevelEmphasis	log.sigma.2.0.mm.3D_glrlm_RunPercentage	log.sigma.2.0.mm.3D_glrlm_LongRunLowGrayLevelEmphasis	log.sigma.2.0.mm.3D_glrlm_RunEntropy
309.3841244	0.943342776	0.011598949	4.750934906
425.6227178	0.876078664	0.007876468	4.745129485
235.3688149	0.943991492	0.015571858	4.643148129
576.590029	0.942081448	0.004268693	4.994946875
404.3007326	0.944075423	0.008417641	4.607322069
185.21991	0.936595287	0.019070499	4.438914239
440.4416315	0.929011641	0.007696336	4.158165402
317.7306485	0.947105788	0.012137709	4.842241024
379.897561	0.942797943	0.007808458	4.880666111
437.4792961	0.919675225	0.005341477	4.651479584
225.1824437	0.93795926	0.015300945	4.454775767
486.3328447	0.93055123	0.005218271	4.788748268
223.0024088	0.932275054	0.012830394	4.525615402
192.6913586	0.928718866	0.02089798	4.551401488
361.9308398	0.947901714	0.007293615	4.587649013
449.7996665	0.826621342	0.008743436	4.368473501
236.9161028	0.937954596	0.017947002	4.614775343
223.7706007	0.918821744	0.015205266	4.663249427
380.9661064	0.944620671	0.009793182	4.786451662
331.9138682	0.874028506	0.009485004	4.489569477
378.2081985	0.891948404	0.007226494	4.556527342
256.6803881	0.943023256	0.011528923	4.691410842
240.4116935	0.922091091	0.01159448	4.519237047
496.9441074	0.951863106	0.006523481	4.692256031
252.2515846	0.92039356	0.011893225	4.506879503
468.1350721	0.929727333	0.006819291	4.819866837
531.2175988	0.872941542	0.005861299	4.537384684
291.0101694	0.929357453	0.010368379	4.628793443
383.7588475	0.945391153	0.008677354	4.804366087
309.5112896	0.877655389	0.012140651	4.685218852
337.0243919	0.92019745	0.007018282	4.521159352
204.4005985	0.902166299	0.015744525	4.483390522
460.4700122	0.9364713	0.006197659	4.874621608
453.3991476	0.961538462	0.018102365	4.424325818
357.2693732	0.957599629	0.013802779	4.82863063
314.6810753	0.931797411	0.008451566	4.609099064
292.1918342	0.922008547	0.011587789	4.828241781
467.0322706	0.920668777	0.005106557	4.687534317
302.3531509	0.933578996	0.011549947	4.547584739
347.719352	0.936129647	0.009184574	4.757027006
420.6749972	0.911908987	0.006691583	4.689311553
306.4546086	0.938493825	0.01020423	4.677046273
310.64862	0.935350885	0.01108307	4.690870286
326.4482009	0.938011257	0.013574404	4.861422901
304.31787	0.944376178	0.010360142	4.678239496
151.720679	0.91175252	0.023149192	4.406642139
723.4594364	0.935915468	0.002971907	4.897181114
223.2753945	0.949597572	0.015592617	4.567662851
409.6752403	0.942594719	0.025676521	4.298519859
366.5936799	0.947995666	0.009604403	4.650257699
331.1394656	0.91653936	0.007941327	4.662342841
258.020954	0.939633522	0.015254292	4.580819077
682.0788779	0.91588992	0.00337076	4.790128262
216.2345304	0.933489522	0.01393088	4.429834017
387.4251855	0.874703711	0.007980893	4.54526523
419.9688413	0.955193839	0.007671838	4.94965117
630.4060366	0.946052124	0.003951661	5.01658666
500.9876347	0.94508894	0.006888823	4.777528171
364.2780854	0.913910414	0.009622087	4.736136059
693.6512536	0.926569458	0.005220352	4.36190768
302.7370217	0.936944132	0.010671433	4.71845423
586.4304864	0.920591457	0.004048998	4.886683007
415.6002646	0.893851133	0.006480619	4.445485485
2137.844143	0.845900346	0.001847008	4.591345798
197.2301829	0.935483871	0.023716366	4.457475179
317.5781751	0.939026202	0.011644766	4.803543023
296.3535896	0.952752831	0.021530104	4.690901671
551.6911592	0.929717764	0.004143983	4.818101152
274.2215484	0.948602967	0.014544965	4.55329281
362.5632536	0.936885591	0.008460261	4.825533443
490.663	0.928795547	0.004554056	4.712398569
266.3952495	0.932002453	0.011907511	4.652003826
397.0300792	0.896150869	0.006947505	4.6543197
386.2859574	0.890330783	0.007026961	4.537459293
681.7584731	0.899723479	0.003475432	4.669504176
419.5229625	0.950992556	0.008989047	4.910750038
263.8120896	0.935128435	0.015266603	4.700034626
242.2986608	0.942370744	0.014235401	4.616162261
285.0640071	0.937336473	0.01313046	4.741109211
347.5974078	0.930023244	0.0077132	4.745749063
373.7032663	0.956654457	0.012601108	4.84962724
633.8206092	0.957609132	0.004394984	5.070805099
1447.840064	0.93982808	0.001533028	4.94998087
349.3915585	0.94801061	0.017613258	4.493536689
253.2342409	0.9269106	0.010739708	4.55631261
742.0484802	0.885626791	0.003335316	4.533545673
227.2810386	0.9413684	0.018320721	4.639124526
472.3339489	0.945479778	0.005765177	4.748063708
263.7690064	0.932744851	0.014755186	4.258979659
338.2189655	0.958974359	0.041909758	4.126424089
1123.280168	0.908330914	0.002803344	4.840700726
478.4829019	0.953475703	0.006024641	4.938294009
550.8601184	0.897561343	0.005982052	4.778530365
421.9949633	0.909573068	0.006004593	4.685911184
378.7634691	0.90586661	0.006557253	4.5795275
713.032448	0.958041958	0.007922841	5.062467561
478.2802613	0.930161943	0.007253907	4.67447036
325.8582042	0.962476548	0.027761877	4.550616867
233.2917274	0.937081388	0.01443618	4.417902242
441.1394184	0.922409684	0.006816475	4.817797851

log.sigma.2.0.mm.3D_glrlm_HighGrayLevelRunEmphasis	log.sigma.2.0.mm.3D_glrlm_RunLengthNonUniformityNormalized	log.sigma.2.0.mm.3D_glszm_GrayLevelVariance
266.5957167	0.898755173	39.47503484
289.6940979	0.798241041	34.03530774
207.2772249	0.898123005	42.00923783
487.2445925	0.894454884	48.81375329
341.2940288	0.894661059	36.33624082
156.3549793	0.88165069	23.23927296
348.2530631	0.870515756	25.36752954
272.5461241	0.901810312	42.41381091
321.362775	0.893711196	40.73646005
350.6403463	0.856092214	34.52175132
187.3003913	0.884361259	24.37872966
402.4420674	0.871921906	43.79123376
184.1357122	0.87462611	26.07437247
158.7472128	0.868506082	25.23294491
311.3062476	0.902166189	31.45704277
258.6574164	0.726834215	29.10035711
198.1049937	0.884825184	29.36340511
180.622896	0.856701234	28.94391821
322.9315468	0.899310624	39.27937053
225.4828957	0.795671449	21.92999506
274.1221182	0.822346311	30.73634119
221.0583621	0.893532048	33.65224163
195.4916967	0.861545517	25.60387973
428.0350727	0.908716626	40.885136
204.5101362	0.856969766	31.03148497
385.3747935	0.872406323	43.98448294
355.9271284	0.791582691	29.16697588
242.8302687	0.872079018	35.45258431
324.4853203	0.899211719	37.69449187
208.9167971	0.803725554	34.9868755
272.1839284	0.858960263	29.2434239
155.6463983	0.831604508	23.35443914
383.3172381	0.882177436	42.05175694
395.8884529	0.92855393	49.32813544
318.5393502	0.917988627	45.43971333
256.8912555	0.874153275	28.56714355
235.1513056	0.865399431	41.62249757
370.4370433	0.860738322	32.74918642
246.2034833	0.875200963	26.39020992
290.9089517	0.883342909	36.67398258
322.1305464	0.84576036	35.71106005
256.0029064	0.884802882	32.34945694
255.4605457	0.880225042	31.4736253
273.2961361	0.885479737	42.63105314
256.7820824	0.896011821	31.89043511
121.1857762	0.844600774	22.49615824
599.6740429	0.88268516	43.39996445
193.8683226	0.905119518	27.37456747
340.4900521	0.89276207	43.97736733
312.9441649	0.902647272	38.75541858
259.8298086	0.853538172	30.09142983
214.135272	0.886862543	30.74124719
527.6640072	0.85358212	35.38697191
180.2690922	0.876166454	22.26129608
261.350484	0.797554641	28.55275164
369.031704	0.915985861	49.33194246
541.8071995	0.899454476	49.62689413
421.0268251	0.897373013	44.77461905
282.5050923	0.849819775	38.00794078
561.5064867	0.867836433	22.3178155
255.666452	0.883385957	34.93250946
460.934621	0.861971843	42.13320053
300.620321	0.819811189	25.82478
1262.265284	0.759564525	30.96876902
169.2515354	0.880607376	24.71396503
269.2472301	0.886978665	41.20883958
255.6611536	0.911425601	40.4683043
450.4980931	0.873298256	35.46435367
236.8069344	0.903602894	29.90226221
304.1342311	0.88482199	39.66714426
402.0825606	0.871253448	32.1760715
219.739887	0.875855518	30.17599243
288.5736761	0.827954933	31.38948132
275.3295958	0.816212195	26.52078376
501.1153346	0.825837165	37.93774506
362.3244507	0.907553699	44.87081887
218.623038	0.880250691	31.72315125
211.9809507	0.890708225	30.79546768
239.5482191	0.883661446	34.92702369
285.6279085	0.873708946	36.385536
331.1865577	0.917546848	49.54366667
560.1478008	0.918514861	58.36235183
1206.912526	0.890663286	50.53432098
292.5698566	0.900935705	38.8677686
207.2314394	0.867651272	25.41450352
525.4897677	0.804549865	31.28701816
196.3950907	0.891815779	32.47569247
399.819225	0.897716647	36.61429536
210.6320312	0.874854244	19.50138504
291.6967088	0.922302502	40.44827586
837.5179089	0.843611632	42.60401536
426.0350497	0.912503065	56.82633956
395.468895	0.835252153	37.734203
319.4541068	0.844796817	32.35946082
289.5046351	0.836682546	27.98829113
630.4620229	0.921281824	79.45470925
382.9865946	0.872512911	37.23989689
289.2206025	0.928496275	37.18962191
191.8206549	0.881772376	24.85865595
356.8610981	0.862787929	53.29196885

log.sigma.2.0.mm.3D_glszm_SmallAreaHighGrayLevelEmphasis	log.sigma.2.0.mm.3D_glszm_GrayLevelNonUniformityNormalized	log.sigma.2.0.mm.3D_glszm_SizeZoneNonUniformityNormalized
209.5185389	0.045516184	0.417164661
224.3924883	0.048081501	0.378630324
195.3099756	0.0468107	0.495498971
360.4190459	0.040030505	0.429290262
220.1974038	0.049210921	0.376364153
115.5796686	0.059693878	0.410688776
188.3285965	0.060310088	0.368895567
193.7488901	0.042748855	0.413785923
220.2242379	0.043189798	0.391326358
294.0718098	0.048113365	0.447176179
123.1424922	0.057469344	0.353737178
338.0225902	0.042997421	0.467584557
117.0973728	0.054925255	0.33657319
118.772672	0.05589642	0.374247589
224.8927036	0.050243757	0.434651697
214.7053584	0.056204955	0.386370971
145.9752721	0.051993334	0.412920486
138.410741	0.051317295	0.328550448
224.6270001	0.045844894	0.40109511
173.3670955	0.060789091	0.348288892
234.8221003	0.051382922	0.445338215
174.2422292	0.048431341	0.396276238
153.8341431	0.05429649	0.394204548
297.1869706	0.04576	0.451904
174.5876534	0.052302774	0.425600242
316.3838102	0.044958753	0.433842986
272.6420016	0.053020933	0.381901108
214.341895	0.047437313	0.454129326
213.9745621	0.044956202	0.398462989
182.116491	0.047811535	0.422151358
238.2224964	0.052725967	0.420635488
122.0860462	0.05746497	0.343782099
285.8048516	0.04319175	0.418633836
284.7588029	0.050568055	0.579416351
241.0152833	0.044427648	0.451359144
159.7117537	0.051645103	0.324141924
199.167924	0.042823667	0.402039974
268.4940759	0.049162304	0.399090086
153.3381548	0.054944488	0.344536081
229.8691645	0.046635842	0.431959503
238.4183466	0.047538595	0.40496156
173.1110981	0.049247885	0.38208862
166.5213159	0.049409763	0.359545373
195.7406931	0.043201305	0.385097754
172.9873217	0.048392603	0.403951103
104.6297774	0.059854523	0.389296983
437.1802239	0.042380254	0.418360579
135.6984358	0.053984645	0.38535359
244.5265977	0.055671176	0.470863684
212.7132083	0.048263224	0.42910454
197.7495435	0.050262549	0.386667606
137.8942203	0.051674594	0.357543929
365.7245451	0.046912506	0.374633016
127.4311337	0.061422414	0.37039239
201.6969752	0.052573727	0.381004637
280.7956857	0.039502222	0.451939346
394.5398556	0.040006013	0.421619442
272.651034	0.042985982	0.412955157
216.9917441	0.04644901	0.416288643
421.8242521	0.06544353	0.416380887
196.223161	0.047352623	0.392771769
334.6190986	0.043326933	0.402869499
223.2597717	0.056655066	0.377145871
832.6291261	0.053364234	0.398150026
149.9709303	0.061672968	0.42084121
206.4101875	0.044333252	0.407399078
183.0252004	0.045626245	0.456880566
322.6277853	0.047229239	0.372112335
169.6010823	0.05367281	0.398943139
231.6937475	0.044128622	0.419355611
308.2734184	0.04844594	0.420386334
154.5446863	0.052082207	0.378518118
224.6873408	0.049764809	0.386932742
209.355363	0.05454122	0.387324609
360.6564741	0.046930409	0.410582274
247.9907304	0.041125949	0.421765756
157.7913578	0.050176177	0.415561219
171.0329222	0.051451035	0.421898485
176.191395	0.046765634	0.405244578
224.1288282	0.046178765	0.408437728
275.7594167	0.04033677	0.488615778
415.823592	0.036589032	0.471586429
875.9237189	0.039782899	0.441925676
145.7440918	0.048209366	0.380165289
152.1954194	0.05455752	0.367641053
392.0430192	0.053779261	0.385532972
171.9023921	0.051271342	0.455678439
245.3664044	0.047242453	0.374995457
137.6956395	0.07132964	0.376731302
174.0581883	0.064803805	0.440546968
559.149076	0.044394867	0.390490454
361.816526	0.038307556	0.463239111
292.4501243	0.045727084	0.420604153
241.3867638	0.049261706	0.403311549
226.3030151	0.053521623	0.391329867
476.1387083	0.034998423	0.481464799
225.0388433	0.046464824	0.361987047
196.3636806	0.049382716	0.427083333
106.6401724	0.056519357	0.325876552
317.0374521	0.040566883	0.455437404

log.sigma.2.0.mm.3D_glszm_SizeZoneNonUniformity	log.sigma.2.0.mm.3D_glszm_GrayLevelNonUniformity	log.sigma.2.0.mm.3D_glszm_LargeAreaEmphasis	log.sigma.2.0.mm.3D_glszm_ZoneVariance
136.412844	14.88379205	12.95412844	8.292755006
230.2072368	29.23355263	221.4786184	209.8087534
107.0277778	10.11111111	9.981481481	5.944358711
534.8956661	49.87800963	17.50481541	13.03257487
59.08917197	7.72611465	11.47133758	6.24268733
114.9928571	16.71428571	16.79642857	11.52284439
44.63636364	7.297520661	22.66942149	14.91250598
202.755102	20.94693878	9.857142857	5.675535194
370.5860612	40.90073918	11.85955649	7.039978412
494.5768535	53.21338156	98.15370705	90.30762993
122.3930636	19.88439306	13.61560694	7.902678005
265.5880282	24.42253521	40.99295775	34.1852683
264.8831004	43.22617535	27.8043202	20.85938754
162.7977011	24.31494253	25.57701149	18.97152068
189.9427918	21.95652174	9.974828375	5.628986904
516.1916168	75.08982036	3790.09506	3763.056617
124.7019868	15.70198675	13.08609272	7.744177887
257.9121019	40.28407643	35.4955414	26.67047913
104.6858238	11.96551724	12.4137931	7.441405734
230.9155354	40.30316742	338.4027149	320.5543203
755.7389511	87.19681791	497.7371833	488.2607992
313.0582278	38.26075949	11.98481013	7.244544144
391.4451158	53.9164149	65.42497482	57.86109007
112.976	11.44	7.46	3.758224
192.7969095	23.69315673	59.00662252	51.94252689
125.3806228	12.99307958	21.83391003	16.06726452
352.8766234	48.99134199	422.8225108	408.7518928
267.4821732	27.94057725	39.11544992	33.19626659
331.1227437	37.35860409	10.90132371	6.390172628
323.7900913	36.6714472	277.5932203	266.6092244
490.0403433	61.42575107	90.60343348	83.15732764
623.6207277	104.2414553	121.2392503	110.5527667
375.9331849	38.78619154	17.85857461	12.73324165
38.82089552	3.388059701	4	1.590554689
85.30687831	8.396825397	5.619047619	2.533355729
244.7271523	38.99205298	18.18543046	11.02714793
447.8725314	47.70556553	33.502693	26.81902923
495.6698873	61.05958132	55.73268921	48.44008028
117.4868035	18.73607038	13.23460411	7.23856864
193.5178571	20.89285714	19.55133929	14.06863341
205.7204724	24.1496063	69.49606299	61.07692665
155.8921569	20.09313725	14.77696078	9.321072424
238.3785822	32.7586727	16.05429864	9.920644995
164.0516432	18.40375587	16.06338028	10.27404726
357.9006772	42.8758465	10.86004515	6.367176393
290.4155496	44.65147453	105.5924933	96.67274256
796.9769029	80.7343832	25.51076115	20.49598281
104.8161765	14.68382353	9.606617647	5.012529736
29.19354839	3.451612903	15.83870968	11.16753382
105.1306122	11.8244898	8.069387755	3.954285714
491.841195	63.93396226	55.28144654	48.03553064
124.4252874	17.98275862	14.13505747	8.487605694
525.6101212	65.81824661	57.28795438	49.87077584
85.93103448	14.25	20.59913793	14.1098209
631.3246832	87.11466506	1123.195534	1107.698459
211.0556745	18.44753747	8.764453961	5.221675554
456.1922366	43.28650647	10.87523105	6.767303822
250.2508251	26.04950495	10.90924092	6.356721563
137.7915408	15.37462236	37.02114804	30.97341207
134.9074074	21.2037037	38.99691358	31.60321407
337.7837209	40.72325581	24.57093023	18.49986344
845.2202097	90.89990467	83.14680648	76.32918182
334.1512415	50.19638826	241.006772	228.8435253
250.0382166	33.51273885	698.6576433	679.7462245
38.7173913	5.673913043	13.72826087	8.16481569
282.3275613	30.72294372	17.45887446	12.43687754
95.48803828	9.535885167	7.253588517	3.699732149
453.9770492	57.61967213	28.60409836	21.73712107
85.37383178	11.48598131	9.046728972	4.70320552
363.5813149	38.25951557	19.16839677	13.7883513
498.1578059	57.40843882	31.4278481	24.84656661
162.7627907	22.39534884	29.14186047	22.2481828
608.2582697	78.2302799	152.851145	142.0432797
725.846318	102.2102455	288.5688367	275.7253719
1214.502366	138.8201487	519.5280595	509.8966683
237.8758865	23.19503546	7.65248227	3.737135959
197.3915789	23.83368421	18.71368421	13.18376066
117.7096774	14.35483871	17.21146953	12.43121234
306.7701453	35.4015852	20.73976222	15.30969254
459.4924444	51.95111111	32.42133333	26.02155773
135.3465704	11.1732852	7.119133574	3.80857303
359.8204456	27.91743119	5.80733945	2.718406511
571.4098995	51.43928848	18.18406806	13.52140773
25.09090909	3.181818182	9.651515152	4.824839302
456.2425463	67.70588235	35.31103948	27.84462635
553.6253482	77.2270195	514.8662953	500.3245009
157.6647399	17.73988439	26.75722543	21.70122624
271.1217151	34.15629322	10.29875519	5.690814935
28.63157895	5.421052632	13.51315789	7.715200831
25.55172414	3.75862069	6.431034483	3.153686088
551.3725212	62.68555241	156.1494334	148.2243312
173.7146667	14.36533333	6.922666667	3.378232889
166.9798489	18.15365239	57.5163728	50.8893147
563.0229226	68.76934097	96.06446991	87.48074523
687.9579067	94.09101251	192.3105802	181.4033037
71.73825503	5.214765101	5.577181208	2.694022792
163.9801325	21.04856512	26.1192053	18.91359541
30.75	3.555555556	4.125	1.206597222
48.22972973	8.364864865	15.56081081	9.003058802
149.8389058	13.34650456	30.29483283	24.27817555

log.sigma.2.0.mm.3D_glszm_ZonePercentage	log.sigma.2.0.mm.3D_glszm_LargeAreaLowGrayLevelEmphasis	log.sigma.2.0.mm.3D_glszm_LargeAreaHighGrayLevelEmphasis	log.sigma.2.0.mm.3D_glszm_HighGrayLevelZoneEmphasis
0.463172805	0.10289781	2471.538226	302.9663609
0.292729899	0.875756745	58004.34211	334.8980263
0.497695853	0.110765309	1395.615741	252.3935185
0.472865275	0.04955508	7454.043339	515.5609952
0.437325905	0.051961847	3915.56051	344.4840764
0.435458787	0.233739422	1883.639286	173.3892857
0.359050445	0.072961768	8584.380165	316.6198347
0.489021956	0.087976292	2231.083673	286.0285714
0.455507456	0.066231303	3414.92925	334.3864836
0.357004519	0.374020579	26968.39241	409.3607595
0.418379686	0.138740483	2345.289017	195.7919075
0.383265857	0.138033383	13624.76056	460.3679577
0.379459981	0.260009422	3925.94028	196.1994917
0.389087657	0.424549829	2566.949425	180.708046
0.479692645	0.046613368	2893.089245	324.2036613
0.192313229	16.4395558	878620.7635	314.0681138
0.432664756	0.152885768	2439.294702	209.5629139
0.336620926	0.387238189	4686.040764	218.2955414
0.448453608	0.082551388	3259.509579	341.5862069
0.236701178	1.844395966	66114.14781	276.0030166
0.324846861	2.200250228	114600.1544	326.8520919
0.459302326	0.100602843	2117.392405	253.5924051
0.363603076	0.522079043	9099.617321	231.7995972
0.51975052	0.026945549	3168.804	428.66
0.376245847	0.402965242	9524.399558	244.0110375
0.416426513	0.090418951	6597.33564	439.017301
0.266589729	1.329332449	136950.9556	406.6363636
0.41102582	0.24622448	7041.142615	293.0305603
0.47082153	0.060207662	3524.649819	327.8002407
0.301730921	1.478716956	54179.89179	252.6166884
0.366467443	0.440823787	19416.35451	330.4223176
0.305902192	1.175903266	13925.05954	187.8555678
0.441711756	0.070124554	6071.325167	412.1213808
0.644230769	0.032527541	1718.402985	373.8656716
0.569277108	0.050046553	1492.84127	336.7830688
0.373762376	0.113286771	4307.484768	267.9761589
0.386805556	0.237463596	6152.239677	280.927289
0.370304114	0.190680171	17568.94928	404.9098229
0.408383234	0.086292709	3420.73607	247.4486804
0.427073403	0.123884563	4798.779018	322.8727679
0.344640434	0.250620043	20768.05118	350.4940945
0.428121721	0.105130105	3184.294118	267.9117647
0.403775883	0.106849559	4192.874811	262.8989442
0.415609756	0.134828418	3709.666667	292.4342723
0.471778488	0.072914892	2616.097065	261.3137698
0.334829443	1.529861422	8288.4437	152.3217158
0.446554149	0.05113333	13912.89764	631.8635171
0.466552316	0.117472735	1569.136029	207.25
0.462686567	0.12308152	5718.854839	335.7258065
0.492957746	0.047272018	2566.24898	311.8816327
0.371495327	0.272010073	12383.82547	293.745283
0.420798065	0.141262264	3035.307471	217.8333333
0.367181366	0.12560527	27773.07128	562.6179615
0.392554992	0.217568994	2775.581897	201.2672414
0.254024222	4.935826036	258770.1141	302.8479179
0.531285552	0.045401075	2704.053533	390.117773
0.493388053	0.036020536	4664.914048	572.4380776
0.468677494	0.040550932	4749.737624	411.6930693
0.406633907	0.168400584	9348.861027	307.6223565
0.367763905	0.099118389	18289.66358	613.9783951
0.405851817	0.187096211	4319.402326	290.7093023
0.382986491	0.214442936	33839.94948	494.4556721
0.286731392	0.927310428	64218.15124	339.6264108
0.229952398	0.561200623	875487.8455	1281.176752
0.423963134	0.229559933	1763.543478	203.75
0.446233097	0.111406769	3958.408369	300.4978355
0.530456853	0.084601232	1875.07177	258.9138756
0.381607757	0.086310763	11144.64508	495.1098361
0.479820628	0.083606265	1837.817757	252.9439252
0.431128792	0.106036974	4625.303345	334.4209919
0.389802632	0.113341713	10068.05992	449.6109705
0.380868025	0.270673218	4502.732558	242.3186047
0.304179567	0.644122367	38549.23601	336.8829517
0.27903514	1.20351518	71407.64888	316.6467449
0.322222222	1.067971137	254960.2708	530.9939148
0.505376344	0.045233534	2605.005319	367.4680851
0.425246195	0.180901614	3165.968421	232.6463158
0.457377049	0.196350448	2176.62724	248.437276
0.429138322	0.16853282	3631.433289	262.4306473
0.395291637	0.173848511	7166.083556	322.8524444
0.549603175	0.052787706	1972.068592	363.6137184
0.568978374	0.018363109	2966.454784	576.6854522
0.463108883	0.017390058	20882.19644	1248.561485
0.455172414	0.065116825	3117.287879	253.0909091
0.365968741	0.285641789	5355.382756	236.4246575
0.262235208	1.09734393	244676.2702	589.2137883
0.444730077	0.327818121	2900.109827	235.8265896
0.465850515	0.04763752	3684.897649	398.9294606
0.415300546	0.096429679	3028.552632	211.1052632
0.552380952	0.11928345	2235.275862	265.4482759
0.355220126	0.198267811	126172.4207	861.325779
0.531161473	0.029834777	2414.074667	479.9786667
0.388454012	0.167777113	21039.84383	419.7052897
0.341320293	0.348490372	28323.15473	353.1439828
0.302790217	0.825917158	47035.41638	339.6774744
0.588932806	0.022393412	3200.926174	648.3892617
0.372532895	0.081495926	10786.11258	368.1103753
0.585365854	0.08588588	1274.111111	283.0694444
0.390501319	0.116776586	3012.135135	188.2837838
0.407682776	0.113730252	9247.644377	423.7082067

log.sigma.2.0.mm.3D_glszm_SmallAreaEmphasis	log.sigma.2.0.mm.3D_glszm_LowGrayLevelZoneEmphasis	log.sigma.2.0.mm.3D_glszm_ZoneEntropy	log.sigma.2.0.mm.3D_glszm_SmallAreaLowGrayLevelEmphasis
0.668760399	0.013846512	6.099021868	0.011790578
0.638925999	0.007191351	6.302441351	0.005272908
0.730250827	0.017065622	5.738687819	0.014799938
0.681085102	0.004330977	6.498197448	0.003314023
0.636592983	0.011553942	6.00764551	0.0098482
0.664851812	0.019977485	5.740330026	0.015601797
0.629715561	0.013093601	5.596307197	0.011744879
0.666510286	0.012218542	6.164536227	0.00871359
0.648602288	0.007911933	6.463025763	0.005751724
0.695526734	0.004885496	6.252203792	0.003723358
0.612380159	0.015872422	6.001939962	0.010505832
0.710588	0.005794473	6.234636309	0.004817846
0.597906932	0.013577285	6.353660727	0.009874096
0.634178647	0.022928763	6.096690387	0.017969045
0.68525796	0.008676764	6.058471882	0.007255613
0.645300448	0.006012893	6.365085417	0.004162453
0.667895001	0.019581205	5.980954921	0.015175968
0.592469107	0.013376149	6.504029446	0.008961943
0.657275742	0.011260099	6.053803984	0.008644746
0.609014062	0.007376924	6.177113043	0.005172669
0.693870172	0.005947662	6.119067325	0.004433369
0.650377788	0.011436535	6.226018176	0.008565944
0.650936235	0.010345693	6.154277758	0.007331326
0.697764228	0.008485302	5.93459626	0.007429856
0.676978804	0.010630828	6.009632784	0.006352567
0.684794915	0.0084905	6.064703526	0.007203663
0.641436645	0.005712823	6.301080948	0.004366549
0.700892281	0.009461363	6.098813728	0.006246738
0.654681331	0.009367536	6.330254754	0.007174993
0.675711078	0.011418117	6.233917191	0.008463531
0.673956572	0.005820783	6.200666747	0.004108558
0.60716098	0.013626022	6.434991718	0.008946251
0.672770145	0.005706987	6.416176559	0.003561602
0.78778296	0.025253223	5.166616616	0.024635672
0.697064163	0.016406491	5.780910467	0.014137346
0.587012082	0.008705036	6.452095943	0.006241879
0.658753835	0.010707142	6.502304966	0.007762008
0.655432216	0.00494346	6.349502284	0.003714764
0.60753292	0.013807144	6.17841877	0.010797077
0.683343123	0.01049098	6.151995984	0.008632739
0.660927455	0.007563766	6.197470177	0.005979685
0.641348214	0.010776492	6.202264753	0.008009609
0.621965626	0.012181456	6.395251676	0.008915058
0.642490613	0.016621641	6.320926521	0.013070864
0.65945484	0.01144622	6.22312793	0.008758941
0.64799253	0.020861542	6.026178355	0.014010734
0.67204199	0.002972643	6.531795165	0.002261058
0.643850327	0.015412901	5.852690758	0.011407136
0.708875349	0.027607862	5.097431545	0.013772017
0.681215	0.011587479	5.980006779	0.009701761
0.645259849	0.007153901	6.393156268	0.005131708
0.616697981	0.01550224	6.058309965	0.010536782
0.634278444	0.003128511	6.500439453	0.002264504
0.629071346	0.015046192	5.783399034	0.011742262
0.641383352	0.006113432	6.450956758	0.004140028
0.698185071	0.008445842	6.204847514	0.006763735
0.674123005	0.003936563	6.489648884	0.003013916
0.667907612	0.008448337	6.369278436	0.006890747
0.670548678	0.010945671	6.098892555	0.008331229
0.670991971	0.005039083	5.824270192	0.002105795
0.651051756	0.012504941	6.339383283	0.01027206
0.659644142	0.003760656	6.592633392	0.002741407
0.63803236	0.006397452	6.311846768	0.004975387
0.656032329	0.002483096	6.228391806	0.002197335
0.671847203	0.025614616	5.286254413	0.021153373
0.662139405	0.011605431	6.333118715	0.007700262
0.701428429	0.023406435	5.85511054	0.016310518
0.633135973	0.003825684	6.51329893	0.002720558
0.65171241	0.01885527	5.808898019	0.016413381
0.673397107	0.009116609	6.381895287	0.006963727
0.674034195	0.004017426	6.292294468	0.002983979
0.635026362	0.012069604	6.137897532	0.009210623
0.64641631	0.005888442	6.461079508	0.004269846
0.645929906	0.005872569	6.352143928	0.004177841
0.665850097	0.003213761	6.523629946	0.002383753
0.674735984	0.010491	6.299046523	0.008586993
0.669860434	0.015795719	6.097922852	0.010730656
0.674105045	0.014546248	5.838098174	0.011826895
0.660920428	0.01415912	6.275110439	0.0103154
0.663924995	0.007386031	6.352777108	0.005328326
0.726505766	0.016270011	5.917553997	0.014790843
0.71387544	0.004787885	6.347678384	0.003926201
0.691465827	0.001744399	6.513935362	0.001454162
0.638260459	0.028969491	5.309101316	0.025822145
0.628910021	0.009768461	6.326985156	0.006897876
0.644588726	0.002862469	6.409270129	0.002107251
0.70166379	0.022050268	5.895460297	0.019120997
0.631679124	0.005922516	6.273764739	0.004374973
0.631618001	0.021230227	5.064396765	0.018522539
0.686466963	0.048552579	4.926272374	0.033919771
0.64870936	0.003903067	6.573587675	0.003427572
0.70749389	0.006611704	6.225252877	0.005500678
0.670314529	0.006490623	6.116304893	0.005240143
0.660381164	0.005510973	6.413467394	0.00403109
0.648588963	0.005618368	6.350569398	0.004095912
0.719656765	0.010354608	5.809295199	0.009311815
0.623741065	0.010700217	6.334729433	0.009046675
0.676481481	0.022550658	5.372923918	0.008459663
0.584483803	0.0196699	5.823138988	0.015591674
0.701481885	0.007552128	6.112693529	0.00622827

log.sigma.2.0.mm.3D_ngtdm_Coarseness	log.sigma.2.0.mm.3D_ngtdm_Complexity	log.sigma.2.0.mm.3D_ngtdm_Strength	log.sigma.2.0.mm.3D_ngtdm_Busyness	log.sigma.2.0.mm.3D_ngtdm_Contrast
0.009143109	1.867733677	2.595718877	0.290368822	0.000379009
0.004334549	0.484341913	1.237353444	0.401928955	5.38E-05
0.013986168	3.166931387	4.525078754	0.204803907	0.000624088
0.002750859	0.936395795	1.245373927	0.434105985	8.05E-05
0.019658825	2.884874463	6.066541378	0.091359668	0.000516226
0.00943303	0.925305926	1.524653623	0.449908502	0.000355888
0.016491476	1.872104576	3.99564722	0.111785294	0.000424686
0.005857692	1.396992691	1.70054635	0.425157777	0.000361102
0.0034913	0.834005131	1.292956691	0.486926703	9.73E-05
0.002312423	0.497906774	1.010400626	0.586549123	3.58E-05
0.006809066	0.793425515	1.138193272	0.517255447	0.000316856
0.004787043	1.308492801	2.10287584	0.268986011	0.000101067
0.003259236	0.484385366	0.99735597	0.772440021	6.40E-05
0.005963717	0.690537476	1.308915817	0.572212082	0.000164966
0.007410956	1.397704217	2.207092657	0.238195674	0.000181177
0.00126057	0.155823039	0.906171382	0.905373444	4.22E-06
0.00887037	1.326319106	2.153269387	0.323860116	0.000344061
0.003255528	0.365966337	0.807436031	0.987150807	7.02E-05
0.012162079	2.008762632	3.335632587	0.175349789	0.000459488
0.003139391	0.208338646	0.623729124	0.75146745	3.10E-05
0.001458142	0.217550802	0.494944234	1.097885305	1.73E-05
0.004088455	0.71886395	1.229261278	0.601081326	0.000107708
0.002664389	0.312220357	0.653861042	0.995173071	4.97E-05
0.012980915	3.139077094	4.212857336	0.113709141	0.000506772
0.005603851	1.058759633	2.231027836	0.376769837	0.000103574
0.010221248	2.511657749	3.796650449	0.148533813	0.000271638
0.002334482	0.295958193	0.884781029	0.56330074	1.99E-05
0.004862593	0.992034865	1.786553635	0.395411673	0.000105049
0.003492733	0.821482964	1.058376373	0.562473247	0.000167112
0.003369134	0.438418767	1.202287478	0.565268467	3.92E-05
0.002203151	0.459594952	0.993730144	0.702643522	3.08E-05
0.001395582	0.122147681	0.390738504	1.954554868	1.49E-05
0.003342945	1.09647383	1.596895888	0.392025755	8.60E-05
0.040766014	13.01274505	15.77598167	0.041005958	0.003907321
0.017397434	4.510905516	4.629953884	0.130852546	0.001309056
0.003398249	0.500336869	0.915376026	0.644965861	8.16E-05
0.002408941	0.602171339	1.000730623	0.884024709	6.61E-05
0.002154276	0.385532214	0.685912836	0.725252299	4.05E-05
0.007350217	1.006215447	1.601883564	0.336497061	0.00025463
0.006179927	1.29442532	1.896994095	0.335039541	0.000208796
0.00519168	0.791224946	1.489929374	0.335531224	9.50E-05
0.006736286	1.145172449	1.879714994	0.345763539	0.000233423
0.003636412	0.700520667	1.003835577	0.616142789	0.000144382
0.006668591	1.499860386	2.227374043	0.300065668	0.000228281
0.003358277	0.611195536	0.846113076	0.684470664	0.00012194
0.003049223	0.281313928	0.621080874	1.420783962	6.42E-05
0.001690577	0.652700093	0.977486926	0.512603512	3.25E-05
0.009988948	1.458188806	1.948692175	0.386018849	0.000540381
0.03386056	7.84055537	12.52915941	0.049055515	0.002114869
0.012447958	2.68934543	4.091332344	0.142756824	0.000479752
0.002231951	0.351244337	0.697722929	0.828054472	3.52E-05
0.006099201	1.124886955	1.320092099	0.498644963	0.000442063
0.001984518	0.510561216	0.97698187	0.511586742	2.92E-05
0.01122424	0.980938577	1.796287551	0.346891038	0.000334809
0.001419038	0.15696529	0.565832384	1.054464295	9.89E-06
0.007232119	2.549299767	2.780158254	0.231201311	0.000343065
0.002904414	1.50944609	1.842921407	0.328435051	9.48E-05
0.005043681	1.380126462	2.019243532	0.2429947	0.000165287
0.009536563	1.439976502	2.635996355	0.195284422	0.000199436
0.007872822	0.929746348	2.318700282	0.187302013	0.000132103
0.003139948	0.601600744	0.956190879	0.733442163	9.42E-05
0.001390729	0.410930943	0.754294743	0.760542768	2.31E-05
0.002727059	0.31062046	0.929111509	0.522836329	2.00E-05
0.003206699	0.380175848	1.568184018	0.230299014	2.78E-05
0.02952472	2.693433673	4.736262815	0.139137008	0.001198295
0.005200022	1.147358926	2.097137204	0.320674576	0.000104901
0.013236551	3.449774514	3.567990307	0.172269264	0.000922653
0.002205897	0.630924891	1.107989226	0.530353807	4.24E-05
0.013012631	2.00819771	2.831999132	0.218885417	0.000611478
0.003613547	0.802198707	1.246266388	0.51402556	9.67E-05
0.002335795	0.410179389	0.712690291	0.761297819	5.84E-05
0.005685737	0.881842301	1.473600408	0.469618851	0.000186327
0.001587315	0.229195318	0.536358394	0.997655707	1.83E-05
0.001208625	0.166145478	0.45156526	1.236912269	1.07E-05
0.000893082	0.211924931	0.538747511	0.914858447	7.26E-06
0.005535955	1.59298808	1.811246096	0.32345892	0.000294518
0.005721568	1.124271094	1.707532267	0.417358718	0.0001873
0.009045127	1.753436288	2.116407225	0.378738119	0.000498793
0.003666628	0.646646056	0.930350784	0.725845069	0.000139276
0.002309426	0.67803169	1.069311161	0.682841727	5.23E-05
0.01114573	3.46734907	3.698089662	0.18857797	0.000761646
0.004523482	2.320160079	2.277523632	0.250394914	0.000245279
0.002847601	1.127343065	1.792326359	0.200348702	5.33E-05
0.044482591	6.208686763	11.29356675	0.046861734	0.001834124
0.002043355	0.265994742	0.501569748	1.325466147	4.47E-05
0.001422254	0.333218339	1.13025448	0.53391185	8.14E-06
0.008280147	1.17015924	1.870902558	0.407535177	0.000325044
0.00359836	0.886805509	1.182195045	0.477066885	0.000196417
0.031616456	2.473579264	4.24398744	0.129337796	0.001357663
0.027735579	9.214825968	8.181368274	0.090794107	0.007031853
0.002012753	0.630538437	1.374343431	0.325973111	2.42E-05
0.008709354	3.759167292	4.131673056	0.175009424	0.000423976
0.007655095	1.105568633	1.851498209	0.216456193	0.000181172
0.001836638	0.369246471	0.751736093	0.762376322	2.44E-05
0.001291264	0.208640354	0.475660578	1.194687523	1.49E-05
0.021153474	12.07680568	10.81522944	0.059540266	0.002167042
0.005990123	1.090776009	2.1216564	0.225974279	0.000115936
0.043586638	8.624483645	11.04171309	0.053994447	0.003105658
0.019434922	1.562291437	3.727801513	0.149021694	0.000394484
0.009099122	2.427803917	4.13117952	0.156078203	0.000225466

log.sigma.1.5.mm.3D_gldm_GrayLevelVariance	log.sigma.1.5.mm.3D_gldm_HighGrayLevelEmphasis	log.sigma.1.5.mm.3D_gldm_GrayLevelNonUniformityNormalized	log.sigma.1.5.mm.3D_gldm_DependenceEntropy
32.12966359	303.1657224	0.051830125	6.422947213
18.46360939	395.9677419	0.088234878	6.702778347
33.15591752	214.2258065	0.058389433	6.201996792
40.85255684	614.4686907	0.04631234	6.73109449
20.75730325	237.1504178	0.070413793	6.049426502
19.54741697	277.7418351	0.063584626	6.239539136
15.68577693	390.6884273	0.093625901	5.800549843
34.67954809	374.7175649	0.049019725	6.426059083
28.97905924	397.1611352	0.052924166	6.522590363
23.18467245	433.1752744	0.069045358	6.504005426
18.71002984	220.0616687	0.065581369	6.17577523
32.81198767	781.9163293	0.057470574	6.586721874
17.52850068	206.6639344	0.068890771	6.322557061
17.20409881	177.7996422	0.068823384	6.324682956
22.94972895	376.1503842	0.061438378	6.250282348
10.5029258	284.959119	0.171672402	6.339899042
21.02171575	172.5444126	0.060869779	6.252927653
17.94819559	260.7572899	0.069577862	6.508818133
26.61783635	311.5429553	0.057450904	6.287271533
11.59636005	223.0542663	0.103162651	6.558769318
17.67915355	385.8870597	0.089132441	6.497518257
23.75951731	416.8593023	0.060361682	6.386152483
15.92867039	251.7777371	0.080934806	6.434239949
31.62582285	386.1767152	0.055744054	6.228999288
18.80742693	184.0921927	0.07594287	6.363918486
28.08711973	392.4899135	0.058355272	6.454297053
15.15648444	518.7484132	0.095696585	6.588474293
23.53177933	385.9385904	0.072245335	6.43756426
31.22192907	352.7586402	0.050224623	6.52052124
17.64560467	274.5149489	0.096235641	6.628016001
18.83027666	246.5278389	0.079122279	6.354599657
11.78481182	172.6895447	0.093591906	6.417565187
32.78102407	419.7845548	0.050494195	6.689112158
33.99029216	322.6634615	0.074149408	5.287622862
38.76128611	353.7168675	0.047539556	6.147729227
19.78201034	288.2064356	0.062776198	6.379948471
29.57453692	331.9600694	0.063430507	6.697099005
23.82233524	730.5989863	0.062496955	6.569283607
17.90954713	209.0443114	0.068682276	6.240655754
28.74172415	443.3241182	0.054184793	6.485882056
22.80460639	362.2489824	0.069769262	6.638010428
23.76336504	289.1490031	0.058263021	6.37478402
25.35863694	359.6114495	0.055556709	6.420929573
29.64973611	304.2712195	0.053696609	6.458420156
26.43060361	479.0686901	0.05576197	6.463316359
16.68683924	196.220377	0.077636028	6.334963397
34.74018092	919.6980778	0.051147122	6.699979417
25.5435451	321.6432247	0.056586121	6.334301063
22.21792159	237.6940299	0.095121408	5.422104538
27.36445231	497.3903421	0.059750859	6.208916952
20.40242111	361.734521	0.068678555	6.58624612
25.63996848	252.0205562	0.056416675	6.28139559
22.89332283	671.5226381	0.065048379	6.684181529
17.63103633	179.1489002	0.06810276	6.167695409
14.55344617	289.539629	0.116836146	6.430471982
42.82274174	452.2059158	0.042836194	6.515727591
39.24589556	591.5280438	0.044679949	6.636575528
32.18714848	548.5978345	0.057883816	6.309205028
23.9699968	289.3316953	0.069206575	6.406719803
16.34401368	662	0.076346274	6.150261025
25.40543112	478.3780085	0.057024904	6.451190629
27.14142427	684.664111	0.061535398	6.723618659
13.8091038	487.9773463	0.0962359	6.448387286
13.88943952	2358.562431	0.11547832	6.559997309
19.91815498	149.0599078	0.067255622	5.957860929
29.00427024	392.7990985	0.058082958	6.538417794
33.98529336	329.1446701	0.051882295	6.090101181
24.83864692	530.8423522	0.057885731	6.580436206
24.92560176	349.6524664	0.05728046	6.094648448
30.27277522	451.8796619	0.055535183	6.580637075
22.37782624	480.4082237	0.063728575	6.488593902
21.13856215	316.619132	0.061148982	6.41512169
17.39363201	395.7894737	0.084699829	6.597433337
14.64617832	311.5412448	0.093864279	6.475859451
21.21740569	584.1214597	0.077888466	6.648499437
39.68121234	489.2275986	0.04539221	6.485925233
23.93347381	285.2318711	0.057705887	6.361246191
26.89904864	193.0131148	0.056404192	6.201347465
24.70867463	311.7460317	0.056690628	6.470245852
26.26627946	324.4898103	0.059606781	6.578893886
37.88047603	466.3869048	0.047264739	6.297292912
51.11378811	658.1610738	0.039400405	6.47913155
40.40122092	2247.819126	0.052358714	6.650449651
24.11681332	287.2275862	0.076337693	5.545089567
18.58583196	261.9127101	0.069545115	6.429376296
14.90009193	665.364317	0.089827328	6.582388278
23.58882938	356.4871465	0.059367173	6.222528779
29.84967144	443.5998711	0.052719637	6.360140278
18.04323808	341.2568306	0.071217415	5.670605905
38.25487528	423.1619048	0.070839002	5.258113584
24.91262428	1285.457358	0.067339045	6.715946078
45.09757722	619.1954674	0.045871486	6.54468921
26.02557435	439.8414873	0.068845478	6.66510649
20.78705986	396.3506112	0.068167574	6.663681308
17.33624795	436.9862212	0.078439115	6.495647914
63.09484604	638.9051383	0.040291209	6.15208519
24.83958405	590.3092105	0.064933291	6.46833578
29.78623835	287.3495935	0.063784784	5.385286409
17.75403958	245.055409	0.072743854	5.977334888
30.83467921	361.3804213	0.065239256	6.617025276

log.sigma.1.5.mm.3D_gldm_DependenceNonUniformity	log.sigma.1.5.mm.3D_gldm_GrayLevelNonUniformity	log.sigma.1.5.mm.3D_gldm_SmallDependenceEmphasis
172.6147309	36.59206799	0.450037584
281.4207992	183.2638421	0.27518316
108.4009217	25.34101382	0.448648578
694.1347249	122.0330171	0.456410008
80.97214485	25.27855153	0.372634732
137.5847589	40.88491446	0.372149303
68.88130564	31.55192878	0.355579358
263.8622754	49.11776447	0.438151346
495.5675806	110.029341	0.391592493
603.7023886	213.9025178	0.369149973
185.1233374	54.23579202	0.364562772
322.4952767	85.17139001	0.401908686
437.6692382	142.87946	0.357110248
222.4168157	76.94454383	0.34757154
229.3995609	55.97036224	0.423258287
646.8946308	1192.608176	0.202142316
154.6189112	42.48710602	0.357409813
429.8164666	162.2555746	0.295773178
142.4054983	33.43642612	0.405352422
355.931096	288.9585862	0.235425359
810.0700613	465.6278714	0.321134304
400.2593023	103.822093	0.384358291
464.9589894	221.032955	0.323298041
125.7941788	26.81288981	0.489842272
224.6312292	91.43521595	0.359574326
138.5360231	40.49855908	0.38030975
488.3150606	331.6843624	0.289131473
275.213538	103.5275645	0.388423853
454.8470255	88.64645892	0.430145127
337.2415421	244.6309992	0.286971118
587.6121422	251.5297263	0.359607268
933.6785835	555	0.265346544
468.3556321	102.6546975	0.404963065
30.76923077	7.711538462	0.492705662
96.45180723	15.78313253	0.506844043
442.2079208	126.8079208	0.343014176
568.9444444	182.6798611	0.378730557
696.4329159	209.6147883	0.382738534
181.754491	57.3497006	0.364112746
241.171592	56.83984747	0.403450256
261.394844	102.8398915	0.353209507
211.8037775	55.52465897	0.359051229
384.9208283	91.22411693	0.377927225
243.3629268	55.03902439	0.394010282
456.4206603	104.7209798	0.403926769
414.1238779	172.97307	0.34273464
1027.992968	218.193624	0.429017269
137.974271	32.9897084	0.433092394
33.28358209	12.74626866	0.424012167
126.3360161	29.69617706	0.393569421
658.4129673	235.1553738	0.348134055
205.0943168	46.65659008	0.400766297
742.9199163	248.5498561	0.35307477
131.9813875	40.24873096	0.380299091
869.6730032	762.122183	0.27031187
252.4175199	37.65301479	0.496202885
574.4062927	97.98312813	0.441197999
342.7648879	74.84377417	0.45399746
159.6879607	56.33415233	0.380257623
174.185017	67.26106697	0.34686332
485.7333648	120.8357716	0.385236043
1086.289157	337.0909091	0.364132503
479.8614887	297.368932	0.296098318
314.755401	315.3712926	0.227293169
52.25345622	14.59447005	0.430093087
342.689633	90.20283323	0.385614133
116.035533	20.44162437	0.501033559
690.1385674	185.0606819	0.364095545
113.2780269	25.5470852	0.438289736
466.2391845	111.6812531	0.414607295
634.0197368	193.7348684	0.379342067
244.2807795	69.03720106	0.359954354
787.1160991	437.7287152	0.29393106
1027.978856	630.3924955	0.282698363
1558.275817	715.016122	0.334513268
316.4892473	50.65770609	0.486226471
260.4270367	64.45747538	0.400540311
147.4131148	34.40655738	0.428060014
397.5260771	100.0022676	0.373820969
600.7470134	169.6408995	0.376001044
141.6111111	23.82142857	0.465269218
435.5428784	52.83594333	0.536497639
700.6618911	146.1855301	0.441278468
34.33793103	11.06896552	0.429708812
688.7133589	235.8274845	0.346350648
812.9897736	491.8944485	0.28165536
189.8560411	46.18766067	0.395244597
406.2654639	81.82087629	0.414513358
43.78688525	13.03278689	0.406597842
33.55238095	7.438095238	0.564137566
732.7162264	267.6727044	0.345090582
183.407932	32.38526912	0.454999313
190.1722114	70.36007828	0.399308637
746.7833741	278.805379	0.338072583
1003.849811	455.4174991	0.313699469
80.2173913	10.19367589	0.545916776
252.6726974	78.95888158	0.36602571
38.44715447	7.845528455	0.548992773
81.22163588	27.56992084	0.335687093
146.2565056	52.64807931	0.366349952

log.sigma.1.5.mm.3D_gldm_DependenceNonUniformityNormalized	log.sigma.1.5.mm.3D_gldm_DependenceVariance	log.sigma.1.5.mm.3D_gldm_LargeDependenceEmphasis
0.244496786	2.790199745	8.739376771
0.135493885	9.981686764	26.51372171
0.249771709	2.41799571	8.138248848
0.263428738	2.562842771	7.9943074
0.22554915	1.963501214	8.459610028
0.213973187	2.331015434	9.279937792
0.204395566	2.48087066	10.0148368
0.263335604	1.553515723	6.622754491
0.238368245	2.051766665	8.142857143
0.194868428	3.878834759	12.19883796
0.223849259	1.797154383	8.325272068
0.217608149	2.973319055	9.955465587
0.211026634	2.26951121	9.425265188
0.198941696	3.070100262	11.11985689
0.251810715	1.662100369	7.072447859
0.093118559	13.17617535	41.8426659
0.221517065	1.889417985	8.598853868
0.184312378	3.241550183	12.65265866
0.244682987	2.146302004	8.082474227
0.127072865	9.137884274	27.10567654
0.15506701	7.890468277	20.9161562
0.232708897	1.91384397	8.111627907
0.170252285	4.349009119	14.55108019
0.261526359	2.084888983	7.324324324
0.186570788	4.847377512	14.07807309
0.19961963	3.940519396	12.129683
0.140887207	9.289409045	24.70398153
0.192054109	3.952513016	12.45708304
0.257703697	1.658938279	6.932011331
0.132667798	10.20280592	27.14791503
0.184841819	3.663972074	12.44636678
0.157450014	5.376410113	17.76728499
0.230376602	2.766118997	9.200688637
0.295857988	1.152366864	5.307692308
0.290517492	1.420598055	5.765060241
0.218914812	2.040519557	9.06039604
0.197550154	5.596201775	14.70486111
0.207642491	3.959136716	11.78711986
0.217670049	2.226239736	9.136526946
0.229906189	2.37346749	8.731172545
0.177337072	6.866242367	17.62143826
0.222249504	2.073954343	8.840503673
0.234421942	1.684016254	7.791717418
0.237427246	1.653063653	7.530731707
0.243035495	1.828976513	7.616613419
0.185872477	3.572883394	12.41202873
0.240973504	2.842522863	8.977027661
0.236662557	2.138339281	8.02915952
0.248384941	1.628425039	7.119402985
0.254197215	1.617981531	7.245472837
0.192293507	4.809251559	13.9182243
0.247997965	1.808588318	7.546553809
0.194430755	5.628591591	15.03506935
0.223318761	1.970642549	8.438240271
0.133324085	7.458531398	23.05273647
0.287164414	1.528839396	5.992036405
0.261927174	1.657631368	6.779297766
0.265092721	1.463659697	6.35962877
0.196176856	4.780306552	13.42997543
0.197712846	2.600272366	10.4415437
0.229227638	1.860168934	8.08541765
0.198300321	4.668618515	13.37531946
0.15529498	6.055613159	18.40776699
0.115252802	13.1357742	35.52727938
0.240799337	2.103676018	7.912442396
0.220662996	2.406184735	9.130070831
0.294506429	1.346543328	5.593908629
0.215870681	2.683853695	9.844541758
0.253986607	1.556958716	6.766816143
0.231844448	2.370286151	8.584286425
0.208559124	2.477991257	9.601973684
0.216369158	2.02811929	8.897254207
0.152305747	6.731207137	19.6873065
0.153064154	5.832366397	18.47528291
0.169746821	4.988564465	15.4204793
0.283592516	1.314300947	5.725806452
0.233148645	1.895305641	7.925693823
0.241660844	1.91841978	7.6
0.225354919	2.379763319	9.068027211
0.211084685	3.806609022	11.42234715
0.280974427	1.456207483	6.099206349
0.324789619	0.943021697	4.539149888
0.250953399	2.22593975	7.817335244
0.236813317	1.816123662	7.675862069
0.203100371	2.859481892	10.6437629
0.148464166	6.776554416	20.23886048
0.244030901	1.709062192	7.498714653
0.261768984	1.462706916	6.682989691
0.239272597	1.928394398	7.841530055
0.319546485	1.495147392	5.533333333
0.184331126	5.614426391	15.46691824
0.259784606	1.85052444	7.050991501
0.186078485	8.272731799	19.30136986
0.182587622	4.958609525	14.64303178
0.172898693	4.447614563	14.65105064
0.317064788	1.194988205	4.992094862
0.207790047	2.990951242	10.54440789
0.312578492	1.292616829	5.260162602
0.214305108	1.898232399	9.042216359
0.181234827	4.896228631	14.53159851

log.sigma.1.5.mm.3D_gldm_LargeDependenceLowGrayLevelEmphasis	log.sigma.1.5.mm.3D_gldm_SmallDependenceHighGrayLevelEmphasis	log.sigma.1.5.mm.3D_gldm_LargeDependenceHighGrayLevelEmphasis
0.048491365	157.5417747	2205.607649
0.075147978	120.5043337	9833.492056
0.074377875	119.4533747	1293.152074
0.017107499	294.3065784	4488.473245
0.052967384	94.21366866	1983.86351
0.046719666	110.180476	2362.609642
0.030640182	135.0477907	4044.219585
0.028853195	175.7013901	2237.284431
0.027282709	162.9268803	3061.328523
0.033866614	175.4790872	4818.124274
0.05898663	88.16369163	1672.453446
0.015110971	332.3005687	7328.152497
0.066228157	80.31879824	1752.635969
0.103376444	68.14937923	1694.270125
0.026370975	165.8946633	2573.384193
0.161903738	68.20146758	11085.2165
0.093607018	67.61891696	1390.04298
0.064323028	85.79109121	2953.601201
0.041074467	123.9299304	2293.085911
0.142035116	58.40012606	5554.313816
0.061166727	141.5650201	7472.988132
0.025211541	175.6359368	3080.45814
0.073060304	92.88903661	3260.198828
0.028696578	191.4985494	2805.656965
0.108270227	79.59243942	2207.127076
0.040151379	163.4655461	4341.170029
0.051579287	162.8944641	12205.75476
0.045152346	170.6698572	4263.337055
0.031169065	158.3085063	2310.377904
0.110711285	88.74986708	7074.156963
0.066988434	106.0636075	2643.659012
0.131241718	51.9789284	2710.273356
0.029930339	184.4125374	3548.780128
0.040123131	151.1481704	1871.913462
0.032971935	192.0485576	1863.084337
0.040922068	101.5468425	2519.372772
0.059509747	144.5331476	4339.224653
0.018568576	296.1373349	8005.55486
0.066491276	74.65142587	1891.372455
0.025467608	187.7225983	3628.530982
0.056763438	137.7158784	5970.459294
0.045688294	113.1475254	2270.739769
0.028732584	135.3672819	2808.689403
0.041049518	125.091311	2175.960976
0.01937065	197.5535477	3643.386049
0.08531778	78.26417838	2125.742819
0.011255462	408.1878812	7857.93624
0.036545507	152.0173576	2376.744425
0.183369421	108.8762576	1639.30597
0.019616801	197.7433949	3592.641851
0.046250885	137.6707169	4620.007886
0.049757941	107.3683429	1814.110036
0.024759162	248.4018383	9680.780424
0.081352874	75.69190458	1303.094755
0.091234812	87.80918485	6163.586233
0.021188593	234.8398338	2526.194539
0.016443378	280.2027502	3600.94984
0.015390405	247.5348672	3537.524362
0.062020641	119.6392269	3576.002457
0.019389239	243.6994152	6366.215664
0.021500678	198.0733224	3572.553563
0.022046788	261.6812173	8661.741877
0.04232165	156.3014693	8365.280906
0.015636535	539.504814	82996.64152
0.114941799	70.53109037	1052.175115
0.030654794	162.1851366	3314.09208
0.03203142	162.276795	1748.200508
0.022021369	204.0970976	4986.473256
0.027273402	160.4354628	2261.307175
0.025358508	204.9927023	3562.024863
0.024289575	195.7700184	4257.094408
0.038565829	120.7523056	2639.747564
0.056548677	131.4833991	7246.309598
0.067431266	98.36293225	5406.183889
0.028942609	206.2171639	8629.667429
0.017669211	240.4016483	2799.976703
0.040953905	123.0026566	2078.63205
0.077594596	92.06802004	1227.072131
0.043322361	126.1679881	2506.914966
0.046095775	136.444206	3339.354884
0.020255561	232.502744	2616.763889
0.010281457	348.6247162	3030.614467
0.004037105	1018.388207	17147.16655
0.051239278	135.4357989	1981.37931
0.054139102	100.1296225	2505.07166
0.032690908	199.940558	12914.50968
0.02958972	152.5907129	2411.520566
0.020773175	184.6598364	2871.210696
0.035012592	145.3812614	2506.721311
0.03605314	223.7435873	2590.133333
0.01289051	447.7422756	19449.72855
0.016189165	308.5413823	3951.994334
0.051824597	191.1993304	7823.90998
0.043777695	145.0871428	5443.022494
0.039167522	151.9615616	5850.377885
0.016053888	367.9284102	2864.083004
0.021874325	210.4948156	6218.794408
0.052413036	164.4726874	1367.772358
0.04891216	81.40007168	2187.335092
0.051714818	156.8742125	4662.609665

log.sigma.1.5.mm.3D_gldm_SmallDependenceLowGrayLevelEmphasis	log.sigma.1.5.mm.3D_gldm_LowGrayLevelEmphasis	log.sigma.1.5.mm.3D_gldzm_DistanceZoneVariabilityNormalized
0.005971619	0.009031453	0.889563372
0.001445655	0.003640078	0.90685045
0.008249104	0.013564891	0.955969483
0.00164799	0.002816325	0.870429988
0.007360592	0.011245502	0.836620644
0.003703417	0.00668693	0.884789323
0.004415935	0.006278599	0.678277522
0.003482389	0.005804537	0.85107563
0.00235489	0.004430351	0.919577983
0.001746447	0.003512089	0.935850498
0.004554761	0.009009445	0.893389511
0.001387354	0.002259807	0.8972994
0.004705071	0.009141233	0.907977149
0.004810967	0.011744412	0.898168071
0.005266695	0.00716673	0.85654321
0.001123557	0.004357555	0.892006197
0.0073517	0.0146936	0.779333481
0.00223197	0.005834042	0.957253124
0.004385672	0.007253746	0.803086735
0.002400591	0.006626354	0.926835945
0.001300214	0.003395458	0.890793129
0.001795329	0.003667776	0.86827752
0.002221026	0.005729075	0.943791061
0.007148754	0.008837707	0.714625446
0.004927315	0.010392116	0.911658726
0.003597885	0.005683275	0.916824197
0.001036914	0.002556606	0.880115415
0.001739747	0.004790051	0.937074493
0.003955709	0.006601948	0.933697077
0.002164562	0.005413988	0.925413651
0.002598695	0.006227402	0.945610258
0.003073377	0.008652386	0.931272522
0.002597603	0.0045722	0.917904536
0.015583379	0.019234381	0.748454716
0.006744487	0.009740617	0.846495436
0.002566766	0.005566771	0.887500913
0.002394435	0.005585063	0.96061481
0.000929911	0.001888535	0.914024793
0.006732157	0.011207869	0.810243126
0.002411561	0.004106143	0.940295738
0.00257922	0.004786512	0.83316563
0.00338404	0.006609155	0.879012896
0.002503667	0.004802342	0.897605109
0.005157243	0.008353796	0.869176809
0.001795996	0.003338408	0.880613486
0.003589251	0.008356685	0.956293426
0.000887961	0.001592616	0.836052268
0.003956633	0.006467115	0.966896802
0.009642271	0.031771926	0.74604589
0.003149269	0.004662579	0.826053465
0.001709924	0.003964868	0.947630017
0.005008885	0.009221035	0.934911004
0.000915166	0.002007028	0.917791749
0.00957733	0.015194996	0.89956382
0.001554078	0.004624229	0.958075512
0.003396927	0.005080513	0.809801455
0.001600627	0.002875988	0.7547416
0.002268031	0.003582241	0.688998628
0.007205238	0.010161844	0.863184401
0.001996715	0.003082059	0.988372491
0.001588206	0.003178988	0.917243052
0.000867381	0.001936149	0.920876946
0.001083896	0.002703433	0.950176401
0.000470887	0.00080392	0.963133583
0.016059846	0.024728881	0.91011036
0.002384751	0.004538232	0.881872891
0.007657407	0.009889714	0.755102041
0.001265829	0.002705222	0.957745659
0.004475497	0.006563947	0.868431357
0.001935209	0.003734566	0.897786645
0.001337483	0.002886578	0.886464176
0.002690507	0.005426602	0.925269045
0.001178974	0.003301422	0.934294205
0.001485194	0.004249064	0.952924247
0.000922262	0.00220014	0.929539579
0.003019766	0.004594277	0.823045185
0.003533225	0.00654284	0.846952587
0.009641129	0.015021608	0.897735573
0.003265681	0.006173613	0.845055474
0.002548534	0.005193169	0.867346939
0.003847071	0.005433805	0.858935537
0.002527626	0.003448829	0.808538249
0.00057314	0.000839001	0.880592969
0.010165854	0.01311811	0.760802469
0.002751802	0.006127687	0.960513477
0.000666237	0.001834035	0.764225322
0.002944932	0.005189713	0.930011548
0.002335396	0.004083246	0.816280303
0.006897472	0.009142352	0.936909263
0.024073777	0.025271545	0.836934729
0.000611764	0.001148444	0.901843236
0.002547058	0.003711854	0.962878013
0.002659961	0.004361896	0.855727788
0.001791633	0.003918179	0.913328831
0.001181414	0.003022366	0.943208558
0.00578787	0.006853499	0.733616408
0.003129352	0.004373256	0.953424582
0.012151452	0.015525865	0.854570637
0.006257562	0.009571572	0.785089546
0.002769578	0.005268024	0.912209718

log.sigma.1.5.mm.3D_gldzm_LowIntensityEmphasis	log.sigma.1.5.mm.3D_gldzm_LargeDistanceEmphasis	log.sigma.1.5.mm.3D_gldzm_HighIntensitySmallDistanceEmphasis
0.012414711	1.175977654	339.7115922
0.005093741	1.146919431	430.2322275
0.017297958	1.067567568	263.1362613
0.00342816	1.208901651	611.5355348
0.018156022	1.269230769	240.1185897
0.009227792	1.184115523	290.2355596
0.011177803	1.604316547	342.4856115
0.00743411	1.243083004	391.4629447
0.005704851	1.125918153	401.5603358
0.004553527	1.099526066	469.5831359
0.011853439	1.169491525	232.8036723
0.003249222	1.162895928	803.9487179
0.012484807	1.14504717	219.3469929
0.01420133	1.161434978	195.9742152
0.011169061	1.233333333	368.7338889
0.005589668	1.171832884	326.0815364
0.019832972	1.37883959	178.309727
0.007310066	1.065552699	292.5838689
0.01004404	1.332142857	296.5098214
0.009951589	1.114084507	248.6514085
0.003981307	1.173889491	424.9849675
0.004401472	1.212658228	436.8360759
0.006698896	1.086826347	285.2664671
0.013541651	1.517241379	349.4750958
0.013497905	1.138947368	215.0857895
0.008771645	1.130434783	431.8971572
0.003474878	1.192131747	537.0240165
0.005187719	1.097560976	428.4186992
0.00862266	1.102990033	357.790144
0.007405759	1.116395494	305.8651439
0.007206859	1.083932854	288.97502
0.011225192	1.106900452	194.6814197
0.006042541	1.128661088	439.7241109
0.028269092	1.442622951	298.6516393
0.012733669	1.251308901	361.2945026
0.007091584	1.179487179	287.6959707
0.006541555	1.060289389	373.4085611
0.002324457	1.135041551	752.6757271
0.016818828	1.318435754	197.9706704
0.005568604	1.092402464	460.7931211
0.006998955	1.275563258	366.5381282
0.008820014	1.194029851	306.5211443
0.006209431	1.162381597	353.3660352
0.012123072	1.21108742	314.1561834
0.004206839	1.191275168	461.7290268
0.010268982	1.067058824	227.7597059
0.00196337	1.27027027	892.6543385
0.008373405	1.050505051	349.5496633
0.026437078	1.447761194	245.4067164
0.007049159	1.288702929	478.5868201
0.004751629	1.080727273	388.8196364
0.011950359	1.101036269	264.2927461
0.002473777	1.128846154	687.2285256
0.022963214	1.159090909	195.5681818
0.005748935	1.064263323	323.1968391
0.006415913	1.319277108	445.8669679
0.003423664	1.429328622	581.2791519
0.004712524	1.577777778	485.8325926
0.017419213	1.221590909	308.3167614
0.005296783	1.01754386	708.3304094
0.003903686	1.129746835	505.3662975
0.00230474	1.123792801	696.1479368
0.003527283	1.076696165	523.0243363
0.001904077	1.056358382	2350.515896
0.03512417	1.141509434	164.2853774
0.005876119	1.189111748	403.967765
0.013743564	1.428571429	306.9837662
0.00332864	1.064780343	555.6332837
0.009367818	1.212389381	356.2245575
0.004522824	1.162076271	474.9237288
0.003412141	1.181254841	501.9455848
0.007125939	1.11663067	331.8790497
0.00393355	1.10202864	441.2775955
0.005189844	1.0723589	346.095152
0.002701041	1.109702153	598.3714981
0.005843482	1.294303797	467.4707278
0.008346116	1.250485437	291.9616505
0.020771552	1.162162162	216.4146959
0.008229018	1.25390625	325.3037109
0.006494755	1.214285714	343.0635146
0.007473118	1.229090909	486.6572727
0.004348954	1.321686747	609.9129518
0.001188037	1.191310441	2184.179047
0.021375889	1.416666667	294.7604167
0.007664663	1.060447761	285.1078358
0.002315286	1.409580838	626.7327844
0.00689695	1.108938547	380.8310056
0.00520868	1.306994819	431.4501295
0.014399441	1.097826087	353.3451087
0.038079384	1.268656716	389.7164179
0.001657345	1.155271565	1259.279233
0.005156904	1.056756757	664.5358108
0.006295758	1.234782609	443.586413
0.005152865	1.136189258	417.1015026
0.003646217	1.08775409	482.2029003
0.009578992	1.474683544	623.8560127
0.007854032	1.071570577	567.2415507
0.0203341	1.236842105	299.1052632
0.016869663	1.367346939	234.4353741
0.007407057	1.13803681	415.2384969

log.sigma.1.5.mm.3D_gldzm_LowIntensityLargeDistanceEmphasis	log.sigma.1.5.mm.3D_gldzm_HighIntensityEmphasis	log.sigma.1.5.mm.3D_gldzm_DistanceZoneVariability	log.sigma.1.5.mm.3D_gldzm_ZonePercentage
0.01471787	347.3687151	318.4636872	0.507082153
0.006349195	440.1990521	574.0363349	0.30476649
0.035785195	263.4504505	212.2252252	0.511520737
0.004301181	644.22972	1212.508973	0.528652751
0.045201564	245.6474359	130.5128205	0.43454039
0.011728599	297.0938628	245.0866426	0.430793157
0.037847971	380.3309353	94.28057554	0.412462908
0.011823281	404.4841897	430.6442688	0.50499002
0.006347783	413.4847849	876.3578174	0.458393458
0.004987576	478.0221169	1184.78673	0.408650742
0.01426858	239.3333333	316.259887	0.428053204
0.003584699	828.8989442	594.9095023	0.447368421
0.021650932	226.2429245	769.9646226	0.408871745
0.018389534	200.706278	400.5829596	0.398926655
0.026100215	389.5955556	385.4444444	0.493962678
0.006429626	340.430593	1323.737197	0.213617389
0.037444763	186.5494881	228.3447099	0.419770774
0.008435366	293.9421594	744.7429306	0.333619211
0.015703365	309.7178571	224.8642857	0.481099656
0.011015841	255.4647887	658.0535211	0.2534809
0.00473682	441.3472373	1644.404117	0.353369066
0.00557267	452.7949367	685.9392405	0.459302326
0.007520985	289.4011976	945.6786427	0.366898572
0.021178003	389.7509579	186.5172414	0.542619543
0.015018001	221.8168421	433.0378947	0.394518272
0.009921649	436.3444816	274.1304348	0.430835735
0.003913889	565.9130833	961.9661482	0.315349106
0.005654506	438.7479675	576.300813	0.429169574
0.013560826	366.1273533	843.1284607	0.511614731
0.008784551	311.0100125	739.4055069	0.314319434
0.007959739	293.9552358	1182.958433	0.393519975
0.015288716	199.2794118	1646.489819	0.298145025
0.006868881	452.292887	877.5167364	0.470241023
0.098776493	307.8852459	45.6557377	0.586538462
0.016034581	376.2984293	161.6806283	0.575301205
0.009033431	297.8608059	726.8632479	0.405445545
0.007094263	377.6511254	1195.004823	0.431944444
0.002560872	778.5387812	1319.851801	0.43053071
0.046855127	207.9972067	290.0670391	0.428742515
0.006157413	467.7556468	457.9240246	0.464251668
0.013805443	390.8214905	480.7365685	0.391451832
0.012294256	314.0248756	353.3631841	0.421825813
0.008274709	360.8903924	663.3301759	0.450060901
0.01785106	320.4264392	407.6439232	0.457560976
0.004958251	486.0536913	787.2684564	0.476038339
0.011219151	229.9435294	812.8494118	0.381508079
0.00240825	950.4167852	1763.234234	0.494374121
0.008817076	351.4208754	287.1683502	0.509433962
0.094714086	251.3731343	49.98507463	0.5
0.008679982	499.539749	197.4267782	0.480885312
0.005371147	394.0254545	1302.991273	0.401577103
0.021550121	267.0906736	360.8756477	0.466747279
0.002795178	704.3544872	1431.755128	0.408270086
0.041647912	198.3636364	237.4848485	0.446700508
0.006387852	326.7575758	1833.756531	0.293423272
0.009077708	474.87751	403.2811245	0.566552901
0.0053497	631.5106007	854.3674912	0.51618787
0.012951592	543.277037	465.0740741	0.522041763
0.040246184	314.3465909	303.8409091	0.432432432
0.005333941	710.4707602	338.0233918	0.388195233
0.004504117	515.7816456	869.5464135	0.44738084
0.002553684	719.6290606	2097.757682	0.415845199
0.003767169	531.7315634	966.3294002	0.329126214
0.001935692	2376.015896	666.4884393	0.253387038
0.071196846	165.1698113	96.47169811	0.488479263
0.007525966	422.0515759	615.5472779	0.449452672
0.036722236	326.4025974	174.4285714	0.586294416
0.003624348	560.9664929	1286.25242	0.420081326
0.011343207	364.119469	196.2654867	0.506726457
0.0054721	490.5497881	847.5105932	0.4694182
0.004146841	518.8830364	1144.425252	0.424671053
0.008370606	337.6198704	428.399568	0.410097431
0.00446216	449.7983294	1565.877088	0.324303406
0.005771338	350.4544139	1975.411963	0.308665873
0.003002355	617.0524919	3152.068711	0.369389978
0.013626413	492.8686709	520.164557	0.566308244
0.017388193	305.3495146	436.1805825	0.461056401
0.030611245	218.9763514	265.7297297	0.485245902
0.011536727	339.3476563	649.0026042	0.43537415
0.008423585	359.9902597	1068.571429	0.432888264
0.009292037	498.0545455	236.2072727	0.545634921
0.005649266	656.1409639	671.086747	0.618941089
0.001290865	2295.934828	1256.606167	0.511103152
0.073820827	303.125	54.77777778	0.496551724
0.008072578	289.3828358	1287.08806	0.395163669
0.003005932	711.1706587	1276.256287	0.304967129
0.00785789	387.547486	332.9441341	0.460154242
0.007531592	448.9430052	630.1683938	0.49742268
0.015370708	357.4782609	86.19565217	0.50273224
0.041376	400.7761194	56.07462687	0.638095238
0.001825537	1301.083706	1411.384665	0.393710692
0.005372543	669.5243243	356.2648649	0.52407932
0.007175043	473.5847826	393.6347826	0.450097847
0.005829367	429.6227621	1428.446292	0.382396088
0.003973368	490.7764006	1902.451661	0.347399242
0.011402296	668.3860759	115.9113924	0.624505929
0.007995398	579.4652087	479.5725646	0.413651316
0.069662164	300.7236842	64.94736842	0.617886179
0.022513922	245.3333333	115.4081633	0.387862797
0.008248868	422.9110429	297.3803681	0.403965304

log.sigma.1.5.mm.3D_gldzm_IntensityVariabilityNormalized	log.sigma.1.5.mm.3D_gldzm_LowIntensitySmallDistanceEmphasis	log.sigma.1.5.mm.3D_gldzm_IntensityVariability
0.044474267	0.011838921	15.92178771
0.047306015	0.004779877	29.94470774
0.044233423	0.012676149	9.81981982
0.038514282	0.003209904	53.65039483
0.054076266	0.011394637	8.435897436
0.055428847	0.00860259	15.35379061
0.066093887	0.004510261	9.18705036
0.042494024	0.006336817	21.50197628
0.043409612	0.005544119	41.36935992
0.04648493	0.004445015	58.84992101
0.057055763	0.011249654	20.19774011
0.039732101	0.003165353	26.34238311
0.05489053	0.010193276	46.54716981
0.058004384	0.013154279	25.86995516
0.05091358	0.007436272	22.91111111
0.055335983	0.005379678	82.11859838
0.051986628	0.015430024	15.23208191
0.051430403	0.007028741	40.01285347
0.051760204	0.008629208	14.49285714
0.059638961	0.009685527	42.34366197
0.048091449	0.003792429	88.77681473
0.049139561	0.004108673	38.82025316
0.055039621	0.006493373	55.1497006
0.042644706	0.011632562	11.1302682
0.052622715	0.013117881	24.99578947
0.044820528	0.008484144	13.40133779
0.050730382	0.003365126	55.44830741
0.045864234	0.005071022	28.20650407
0.044202357	0.007388119	39.91472868
0.046655629	0.007061061	37.27784731
0.050744377	0.007018639	63.48121503
0.060512684	0.010209312	106.9864253
0.04109697	0.005835956	39.28870293
0.057242677	0.010642242	3.491803279
0.043831035	0.011908441	8.371727749
0.052655119	0.006606122	43.12454212
0.041034264	0.006403378	51.04662379
0.046003714	0.002265353	66.42936288
0.058893293	0.009309753	21.08379888
0.044369205	0.005421401	21.60780287
0.044949524	0.005297333	25.93587522
0.049800748	0.007951453	20.0199005
0.04878223	0.005693111	36.05006766
0.045567169	0.010691074	21.37100213
0.047450315	0.004018986	42.42058166
0.053940484	0.01003144	45.84941176
0.039725208	0.00185215	83.78046468
0.049189992	0.008262487	14.60942761
0.070617064	0.009367826	4.731343284
0.04711052	0.006641453	11.25941423
0.048458579	0.00459675	66.63054545
0.051571854	0.009550418	19.90673575
0.046493261	0.002393427	72.52948718
0.057219927	0.018292039	15.10606061
0.051962168	0.005589206	99.45559039
0.038636474	0.005750464	19.24096386
0.039960232	0.002942154	45.23498233
0.044714403	0.002652757	30.18222222
0.045196281	0.01171247	15.90909091
0.060582743	0.005287493	20.71929825
0.046346739	0.003753578	43.93670886
0.042882217	0.002242504	97.6856892
0.057170675	0.003467312	58.1425762
0.055673427	0.001896173	38.52601156
0.05749377	0.026106001	6.094339623
0.042364184	0.005463658	29.57020057
0.045857461	0.007998896	10.59307359
0.046771295	0.003254712	62.81384959
0.049768972	0.00887397	11.24778761
0.042292086	0.004285505	39.92372881
0.04770139	0.003228466	61.58249419
0.05432222	0.006814772	25.1511879
0.049225768	0.003801397	82.50238663
0.053776893	0.005044471	111.4794983
0.045173178	0.002625713	153.1822471
0.040573426	0.003897749	25.64240506
0.048641719	0.006085597	25.05048544
0.048644083	0.018311629	14.39864865
0.048065186	0.00740209	36.9140625
0.044098657	0.006012548	54.32954545
0.041560331	0.007018388	11.42909091
0.035950065	0.004023877	29.83855422
0.038181944	0.00116233	54.4856342
0.05632716	0.008264654	4.055555556
0.052307864	0.007562684	70.09253731
0.053660583	0.002142625	89.61317365
0.050731875	0.006656715	18.16201117
0.046887165	0.004627952	36.19689119
0.066398866	0.014156624	6.108695652
0.051013589	0.037255229	3.417910448
0.044672498	0.001615297	69.91246006
0.036230825	0.005102994	13.40540541
0.042873346	0.006075937	19.72173913
0.048851721	0.00498374	76.40409207
0.052022115	0.003564429	104.9286068
0.035731453	0.009123166	5.64556962
0.047480524	0.00781869	23.88270378
0.052977839	0.008002084	4.026315789
0.05502337	0.015458598	8.088435374
0.040987617	0.007196605	13.36196319

log.sigma.1.5.mm.3D_gldzm_HighIntensityLargeDistanceEmphasis	log.sigma.1.5.mm.3D_gldzm_SmallDistanceEmphasis	log.sigma.1.5.mm.3D_glcm_SumVariance	log.sigma.1.5.mm.3D_glcm_Homogeneity1
377.9972067	0.956005587	810.1146395	0.257422574
480.0663507	0.963270142	1175.394137	0.372553826
264.7072072	0.983108108	534.3358555	0.272473123
775.0064609	0.947774587	1905.576283	0.244980987
267.7628205	0.932692308	624.0692227	0.299732546
324.5270758	0.953971119	779.873921	0.293054602
531.7122302	0.848920863	1185.653304	0.307106714
456.56917	0.939229249	1029.214307	0.243194346
461.1825813	0.968520462	1143.777622	0.271371354
511.7780411	0.975118483	1290.026452	0.308663777
265.4519774	0.957627119	573.0164667	0.294548397
928.6998492	0.959276018	2504.760284	0.277508429
253.8266509	0.963738208	552.1623097	0.307846702
219.6345291	0.959641256	452.5219004	0.322142947
473.0422222	0.941666667	1107.880445	0.263560203
397.8268194	0.957041779	866.1343971	0.471891862
219.5085324	0.905290102	413.7955984	0.295660957
299.3753213	0.983611825	724.6612643	0.332610385
362.55	0.916964286	854.8705484	0.271179301
282.7183099	0.971478873	619.7424218	0.414977674
506.7963164	0.956527627	1150.860429	0.349484831
516.6303797	0.946835443	1206.829496	0.278276874
305.9401198	0.978293413	702.6565003	0.346847556
550.8544061	0.870689655	1093.423024	0.248762506
248.7410526	0.965263158	483.9709804	0.33590094
454.1337793	0.967391304	1111.779706	0.299971282
681.4693504	0.951967063	1655.596775	0.37901709
480.0650407	0.975609756	1120.182128	0.316655316
399.4761905	0.974252492	999.2480614	0.262150641
331.5894869	0.970901126	783.8911493	0.382750721
313.8760991	0.979016787	678.8732541	0.321949985
217.6713801	0.973274887	455.6832326	0.383900978
502.5679916	0.967834728	1214.945495	0.265544859
344.8196721	0.889344262	837.149318	0.243140015
436.3141361	0.937172775	945.1503882	0.23415607
338.5201465	0.955128205	827.9254035	0.298255134
394.6213826	0.984927653	925.3781282	0.297973573
881.9909972	0.966239612	2394.080175	0.290816826
248.103352	0.920391061	547.8176632	0.299244607
495.6057495	0.976899384	1276.848577	0.271569456
487.9549393	0.931109185	1064.50603	0.324223817
344.039801	0.951492537	802.9488375	0.286013308
390.9878214	0.959404601	1028.443116	0.267794794
345.5074627	0.947228145	822.5686682	0.268031816
583.352349	0.952181208	1439.817604	0.263547284
238.6788235	0.983235294	512.5082607	0.339755656
1181.466572	0.932432432	2996.761648	0.261955028
358.9057239	0.987373737	895.0910988	0.26681805
275.238806	0.888059701	596.0176791	0.287648583
583.3514644	0.927824268	1459.682264	0.25816468
414.8487273	0.979818182	1049.115938	0.311167954
278.2823834	0.974740933	662.7173684	0.270337142
772.8583333	0.967788462	2144.675998	0.310278197
209.5454545	0.960227273	444.0523781	0.302618517
341.0005225	0.983934169	844.1341933	0.393005354
590.9196787	0.920180723	1281.57118	0.224774698
832.4363958	0.892667845	1775.960199	0.242095257
773.0548148	0.855555556	1626.26988	0.250658958
338.4659091	0.944602273	792.4526211	0.309119418
719.0321637	0.995614035	2147.592352	0.327103249
557.443038	0.967563291	1397.330712	0.278346716
813.5535558	0.9690518	2203.426619	0.301430441
566.560472	0.980825959	1516.648504	0.369866938
2478.015896	0.985910405	8524.485486	0.423179456
168.7075472	0.964622642	327.7929973	0.298226498
494.3868195	0.952722063	1133.79493	0.278330946
404.0779221	0.892857143	892.9840073	0.231519595
582.2993299	0.983804914	1615.417972	0.295286971
395.699115	0.946902655	996.3230193	0.261138803
553.0540254	0.959480932	1334.076049	0.273142463
586.6328428	0.95468629	1433.613918	0.298790319
360.5831533	0.970842333	886.194325	0.290106049
483.8812649	0.97449284	1170.406881	0.354825185
367.8914616	0.981910275	902.2545503	0.363587307
691.7764671	0.972574462	1899.059444	0.333967186
594.460443	0.926424051	1407.608329	0.227566388
358.9009709	0.937378641	788.9122337	0.272883966
229.222973	0.959459459	462.8793563	0.275128746
395.5234375	0.936523438	871.4405445	0.27974143
427.6972403	0.946428571	905.7216197	0.288302318
543.6436364	0.942727273	1287.883854	0.241467053
841.053012	0.919578313	1970.33817	0.210737594
2742.957954	0.95217239	7925.526451	0.251610461
336.5833333	0.895833333	763.5536867	0.293667462
306.4828358	0.98488806	740.8102636	0.310452487
1048.922156	0.89760479	2206.980533	0.360302079
414.4134078	0.972765363	1008.013884	0.278876652
518.9145078	0.923251295	1265.806201	0.250459508
374.0108696	0.975543478	1000.965771	0.298148922
445.0149254	0.932835821	1193.8912	0.238668141
1468.301597	0.961182109	4390.713464	0.314780175
689.4783784	0.985810811	1824.992884	0.244727488
593.5782609	0.941304348	1304.577675	0.31984705
479.7078005	0.965952685	1167.13637	0.320318887
525.0704016	0.978061477	1330.323035	0.339481921
846.5063291	0.881329114	1888.964295	0.208596871
628.359841	0.982107356	1890.330775	0.299657343
307.1973684	0.940789474	731.409132	0.246185687
288.9251701	0.908163265	665.0295518	0.31138274
453.601227	0.965490798	1014.20402	0.317461811

log.sigma.1.5.mm.3D_glcm_Homogeneity2	log.sigma.1.5.mm.3D_glcm_ClusterShade	log.sigma.1.5.mm.3D_glcm_MaximumProbability	log.sigma.1.5.mm.3D_glcm_Idmn	log.sigma.1.5.mm.3D_glcm_SumVariance2
0.171205111	175.665541	0.016809108	0.959199173	65.52915015
0.299168825	98.47400915	0.064261586	0.973836938	41.50485267
0.187827273	439.645076	0.019487154	0.953270673	68.19068149
0.160527685	129.7024892	0.01167774	0.966277058	91.52081255
0.208762596	12.82316588	0.019931318	0.967442766	51.79131228
0.202700577	26.97063636	0.014757998	0.961833278	44.06860873
0.218922819	-186.4526613	0.031867822	0.959287239	37.10417758
0.156175924	51.74853373	0.009113042	0.951421039	76.07298559
0.182483263	51.67715136	0.009798237	0.962860091	63.37618496
0.222631112	190.0406984	0.025365129	0.977511888	50.86381542
0.202873004	-3.813470295	0.014128361	0.953231739	41.37447239
0.191254267	187.6468019	0.016858255	0.977945382	66.06079031
0.217266153	70.8436372	0.013340855	0.974245148	39.73444739
0.233221628	56.56709369	0.0187218	0.9587844	40.27904116
0.170922406	-16.63890424	0.013387953	0.965489438	48.81646055
0.414064703	111.8117742	0.125617323	0.987388703	25.7456058
0.204526532	9.621031418	0.012385086	0.953377293	49.35183409
0.246693208	114.7516354	0.021361862	0.970319388	43.53699612
0.179695474	-29.94729704	0.015787581	0.956241128	62.77472455
0.343190116	54.07267164	0.06508364	0.975076234	28.00757505
0.269193756	118.3112911	0.0547603	0.977967721	34.58110566
0.186248532	226.3874303	0.012713897	0.975311401	52.90004187
0.264006993	102.7793382	0.032284129	0.97027163	36.91394055
0.160704806	-120.6989213	0.01481581	0.953997994	67.10038873
0.252624645	129.2215561	0.029413243	0.977338088	37.25892363
0.216312986	105.9047341	0.023375041	0.962340505	53.37983302
0.303927006	91.91758932	0.057194716	0.983086787	34.52872333
0.232159146	271.3930331	0.024832233	0.974454554	47.12404358
0.174483741	-70.88966557	0.009200607	0.95227601	69.06015535
0.31074109	105.4933866	0.065782245	0.977019715	37.52077651
0.23639792	161.4284847	0.027435071	0.976856017	38.87751113
0.303059652	81.39095133	0.041966216	0.978484887	29.40010715
0.178960282	151.6940625	0.011394455	0.96729624	73.94482672
0.155723641	-360.0826903	0.034207999	0.942356644	81.64784636
0.146673518	-84.21068784	0.013176081	0.935293127	78.86311109
0.208424602	-13.57446162	0.01199588	0.962652664	46.92540641
0.216774179	281.1417383	0.035890091	0.96658348	62.48084752
0.204946848	7.606195024	0.020906116	0.975460363	49.85205214
0.208550921	-80.1851794	0.016562047	0.955075121	42.90190738
0.18425631	146.960658	0.012807486	0.967755316	61.67925082
0.244580031	62.19744051	0.03764423	0.966167239	45.53901949
0.196053289	9.037268811	0.013874427	0.959106868	55.48066488
0.177990831	-29.31641223	0.009014934	0.959740961	56.33481602
0.179093721	41.44662476	0.013006102	0.956578302	65.4002081
0.172715797	3.589211371	0.010145364	0.968982138	59.13732805
0.255732769	124.2427113	0.03083169	0.972781221	35.84824844
0.175727343	121.7169387	0.012772361	0.975111865	74.13518633
0.176357294	55.11871686	0.013293877	0.958717255	52.64087123
0.198861016	-364.8739293	0.035194267	0.952835764	62.50179232
0.168328613	-24.06958751	0.014766308	0.965250428	65.8072062
0.226103206	86.77047824	0.02790198	0.97194342	42.836741
0.180519966	-30.86501499	0.012753292	0.950204696	59.12942843
0.227352331	78.48873325	0.027273511	0.981115664	50.21437861
0.211176005	43.47189596	0.017076639	0.962283466	40.11659324
0.32239013	78.0822342	0.074547831	0.978234206	36.57506865
0.138248336	144.7342526	0.00892148	0.948788101	93.55000355
0.155359707	2.141026329	0.007579247	0.965154153	90.62707974
0.162525188	-288.8961236	0.011111375	0.957437945	77.97278143
0.226300422	-20.37731706	0.032033278	0.968997267	48.75891661
0.238301053	13.77701751	0.02454491	0.982219995	35.53303964
0.188559153	166.4560694	0.011635452	0.970846318	56.98630363
0.218638424	200.6060949	0.028715456	0.977976411	61.44138679
0.291082479	57.01557541	0.047433789	0.980853093	32.22218577
0.356130806	-178.9275277	0.082575486	0.992555048	33.03641651
0.205997634	53.95594983	0.022034367	0.939969508	46.59953927
0.194605181	127.1023076	0.016186605	0.96217106	66.94735713
0.142138196	-164.1001417	0.013492947	0.936142286	71.90984112
0.208747773	86.36662793	0.013399618	0.97724318	60.83735801
0.168580188	-22.49933786	0.013998326	0.952661358	52.99282838
0.187821062	174.0169294	0.013072317	0.971276539	71.78926547
0.210994335	159.3935131	0.014558261	0.971633739	50.66037673
0.199308683	45.17111712	0.012435659	0.966318882	47.18971303
0.275126118	141.6508402	0.045926531	0.97775604	37.99193802
0.28457736	100.8913907	0.043722782	0.984960556	31.96619616
0.254089977	69.97492565	0.028079338	0.980921898	48.08501172
0.139144531	-34.62691597	0.007228926	0.958505462	89.16457593
0.181802286	8.038081104	0.011458788	0.96094534	52.76166693
0.185940656	161.5508346	0.014478238	0.946060064	57.82636392
0.191263464	39.04174655	0.011192332	0.958395739	53.40117323
0.202502854	182.5695475	0.021131895	0.973799782	55.06286662
0.151456979	136.7627865	0.013354699	0.958043819	75.12740455
0.125164423	-121.207382	0.0068519	0.958240958	109.8159519
0.169335775	-44.37965215	0.015820504	0.981931527	97.42571096
0.206422591	-156.3184815	0.031294343	0.951401659	64.85531053
0.222912389	56.34544097	0.019052996	0.969473942	41.85649658
0.280146162	128.758246	0.043525461	0.988520773	32.07524148
0.187988145	82.46841567	0.013356002	0.960456133	49.34277898
0.160123042	-66.75393084	0.008812563	0.952950079	67.10060575
0.208539867	-52.22282604	0.029373568	0.957015276	37.94989592
0.153514215	-450.7211309	0.038005405	0.922832312	78.76645883
0.233843825	-138.5362697	0.028080525	0.982450006	56.68595971
0.159311139	373.1099101	0.011351485	0.963911282	89.7022451
0.242265254	91.07949207	0.046867766	0.967327884	55.29444223
0.236561539	124.5996609	0.022817898	0.977742441	45.00412562
0.255895675	99.39602974	0.032189341	0.984264009	39.37513145
0.124055948	213.0555298	0.013603905	0.948863654	132.850819
0.212157287	-256.6917505	0.015515637	0.97553568	57.96316309
0.155880832	-159.7742715	0.024919553	0.9300766	78.78013474
0.219911671	8.157932549	0.018004612	0.969358306	42.45228718
0.235473153	426.8406467	0.024328488	0.969893921	59.19562205

log.sigma.1.5.mm.3D_glcm_Contrast	log.sigma.1.5.mm.3D_glcm_DifferenceEntropy	log.sigma.1.5.mm.3D_glcm_InverseVariance	log.sigma.1.5.mm.3D_glcm_Entropy	log.sigma.1.5.mm.3D_glcm_Dissimilarity
66.93650826	4.072905801	0.170085881	8.349959375	6.311179441
38.17321281	3.618879273	0.269436489	7.730913426	4.247640631
68.02951252	4.026551995	0.194035815	7.850875232	6.111489905
87.3185448	4.274112836	0.165533354	9.169565988	7.05995223
34.88077295	3.603051652	0.214516966	7.565563651	4.565186773
38.71597626	3.71862995	0.202367856	7.941402761	4.828643659
40.14657319	3.659445268	0.22062112	7.213700233	4.71255255
72.65254069	4.135427012	0.160913585	8.665285765	6.654891067
54.52848059	3.958721604	0.186618126	8.639094224	5.642552584
45.74159263	3.812813686	0.221639453	8.24289882	4.924313513
36.60785595	3.677570278	0.202852792	7.934380474	4.705399711
65.43883893	4.02925663	0.194938853	8.449051461	5.90611675
32.51108937	3.603614184	0.224893798	8.009735207	4.381248692
29.51336082	3.520972709	0.235297827	7.848992436	4.155682745
50.46039868	3.885501224	0.177515026	8.214975748	5.501568049
23.51159248	3.149652521	0.340399543	6.648986369	2.943729669
36.50943918	3.659526724	0.207306389	7.981048982	4.689782018
32.18668948	3.568717408	0.248370553	7.990546719	4.170261249
48.60929727	3.840308108	0.182044474	8.136154329	5.396570081
22.19212277	3.270290397	0.303903365	7.259598472	3.223105142
34.93985978	3.632001015	0.254295799	7.753042265	4.231172516
45.47023474	3.81819551	0.190328728	8.290975554	5.168259647
30.02771491	3.523209414	0.257978212	7.744445071	4.015441894
68.93446298	4.077107913	0.163303136	8.164821256	6.425060703
36.35231023	3.624140811	0.250686197	7.655847578	4.326062601
53.75165347	3.886043777	0.217517542	7.971439385	5.340898853
30.92911869	3.504300181	0.284582446	7.613822929	3.83835072
45.80521859	3.782644799	0.232217663	7.904780717	4.848104391
64.99012086	4.027835954	0.17927318	8.664248857	6.109796866
35.39515803	3.551412629	0.288456536	7.569314504	4.013669284
38.97886946	3.703734509	0.234710516	7.930919376	4.544705146
19.83674415	3.26100349	0.289823436	7.444448749	3.245639181
67.83915105	4.093838931	0.179591707	8.802115873	6.186813624
74.90164819	3.902447174	0.158574033	6.496713642	6.709737333
80.92039761	4.137333376	0.151106373	8.029415439	6.990323644
38.16541727	3.711900595	0.216181439	8.220464275	4.718745812
60.5756521	4.006737704	0.205098908	8.476746812	5.699404234
49.47031682	3.901996749	0.20391715	8.422977034	5.308234143
34.75535977	3.613249077	0.211877467	7.88785833	4.59672033
57.46522404	3.979198369	0.187096865	8.392652983	5.778132139
47.67626036	3.807385454	0.243237518	8.033090403	4.938437983
45.21036416	3.819908328	0.197189986	8.226885871	5.132357921
52.84982337	3.92336285	0.179922987	8.469073825	5.633499552
58.53631177	3.977221908	0.184457721	8.456744561	5.792403545
55.13889247	3.967558611	0.173473381	8.562246261	5.726335485
31.10979396	3.551809743	0.255629495	7.732019158	4.090959927
71.23418769	4.136632507	0.181751014	8.979328716	6.2954952
51.04303028	3.891972859	0.177939693	8.114292187	5.576640758
46.74286831	3.666251059	0.204316507	6.599139624	5.172922184
59.07754801	3.969377923	0.168348019	8.101912462	5.962816621
42.77315309	3.78441812	0.222572214	8.218959397	4.842734561
52.44477082	3.899514039	0.181767008	8.241752597	5.592665657
46.69759183	3.835661193	0.223237095	8.354434049	5.039174679
33.4705449	3.607212777	0.217949297	7.781607748	4.469134358
33.24142322	3.497629936	0.290928546	7.562592285	3.880265865
91.26514954	4.294052256	0.14099918	8.861323741	7.439820042
79.06176153	4.183437313	0.163207648	9.02575398	6.818369313
71.44906822	4.103728602	0.174786579	8.651094758	6.403173962
48.3040679	3.822172054	0.231573903	7.960761024	5.049853849
29.52446035	3.488111985	0.247662156	7.548529702	4.033621475
49.09247836	3.883044424	0.195468575	8.461677151	5.339110041
55.53005529	3.952095967	0.211017913	8.622503855	5.428248289
28.71406082	3.471244707	0.275350625	7.594164769	3.786167611
29.14122996	3.322051336	0.311315679	7.133457468	3.419056125
35.29000787	3.579471551	0.212574562	7.211257747	4.579595881
66.0280825	4.052803892	0.197167809	8.60133736	6.0171272
73.68357795	4.106140799	0.146945704	8.130949414	6.78870241
47.8803784	3.860234787	0.210420655	8.534967425	5.170549638
49.2821041	3.860851199	0.175358574	7.975398491	5.520942475
64.63690043	4.049925272	0.191630338	8.683729861	5.998549183
43.1341269	3.772330432	0.21982693	8.286689104	4.913126532
41.13610561	3.75672004	0.203907607	8.141404464	4.915022696
35.56150614	3.622463973	0.254679288	7.859759964	4.207951177
29.87697189	3.501503332	0.280666855	7.635826963	3.859559487
46.07443136	3.776583904	0.251535769	8.258322888	4.743001848
83.44051996	4.235461473	0.142802087	8.898869903	7.122008287
48.36927936	3.860122536	0.18672025	8.297631299	5.373085977
50.35187494	3.878052875	0.190599321	8.069469895	5.457562417
48.7005318	3.870933004	0.195305606	8.379378859	5.364796218
53.23682579	3.932266424	0.202130895	8.437832985	5.460734653
69.49417731	4.074292348	0.157583769	8.2494737	6.466173803
105.2008894	4.402520382	0.133831705	9.176967415	8.010340311
95.11627521	4.313632242	0.172413903	9.148138114	7.193562007
44.98069592	3.638637674	0.21206862	6.797482701	5.075152601
37.00700615	3.691298136	0.224699418	8.11638887	4.593810989
30.6523587	3.50228194	0.281246993	7.614960522	3.863212944
43.19071785	3.778407191	0.199648715	8.049542019	5.103051879
58.82324949	4.003449066	0.165010217	8.626988098	6.042993972
35.87286381	3.430961293	0.22075837	6.896316012	4.636855333
89.24166903	3.956329209	0.158522885	6.490208972	7.20988663
53.54238106	3.891957714	0.226550127	8.404617422	5.217046142
86.98170616	4.237299373	0.160709573	8.513585101	7.022940022
56.92563532	3.916546584	0.228812533	8.067349687	5.369942749
43.24433651	3.770364404	0.233007109	8.225356025	4.753905259
34.24095799	3.61312573	0.252979626	7.981859464	4.211554249
121.776546	4.354489785	0.125718416	7.931065562	8.517774189
48.41848296	3.826661504	0.211765648	8.176078064	5.070530487
62.55540809	3.859856919	0.158416468	6.86466152	6.230690491
30.56557371	3.52534057	0.22174914	7.491786657	4.26866117
55.56284326	3.858953809	0.230710115	7.844757815	5.194228527

log.sigma.1.5.mm.3D_glcm_DifferenceVariance	log.sigma.1.5.mm.3D_glcm_Idn	log.sigma.1.5.mm.3D_glcm_Idm	log.sigma.1.5.mm.3D_glcm_Correlation	log.sigma.1.5.mm.3D_glcm_Autocorrelation
26.77607722	0.868514112	0.171205111	-0.004688833	259.7708323
19.8561612	0.90428365	0.299168825	0.046876009	367.1925981
30.37768584	0.865115273	0.187827273	0.005632127	175.0880523
37.18600194	0.881607422	0.160527685	0.030591601	573.6103384
13.72293896	0.880512849	0.208762596	0.195606186	209.4440832
15.26828381	0.87146819	0.202700577	0.066126798	254.5544917
17.46375659	0.872213738	0.218922819	-0.026546988	366.4445764
28.03504373	0.856237629	0.156175924	0.029877637	324.4811707
22.4835869	0.874826676	0.182483263	0.080607163	360.8191004
21.35908209	0.904768086	0.222631112	0.055767446	401.3680875
14.21407649	0.858695312	0.202873004	0.060813932	193.3841612
30.1647851	0.905349579	0.191254267	0.007853453	738.6947868
13.07028649	0.893217602	0.217266153	0.102240144	188.063836
11.93039929	0.868752338	0.233221628	0.153346141	158.3700628
20.03220961	0.876643792	0.170922406	-0.009024752	348.1940548
14.71733373	0.939087821	0.414064703	0.051632297	273.5607351
14.09501797	0.859172752	0.204526532	0.148736883	145.4922249
14.49695748	0.890818117	0.246693208	0.151365315	240.1265057
19.00419069	0.863115166	0.179695474	0.131882013	277.0997477
11.58493313	0.90620619	0.343190116	0.115010292	207.2807851
16.96858281	0.907531002	0.269193756	-0.001403398	359.2625032
18.35185568	0.895164575	0.186248532	0.080296513	378.1238571
13.59608145	0.891483724	0.264006993	0.103521392	232.4421257
27.28374414	0.860525626	0.160704806	-0.005284219	341.7478145
17.46414071	0.905613014	0.252624645	0.014241951	165.1735117
24.90995488	0.879485915	0.216312986	0.001298587	348.2086302
16.13135087	0.920951273	0.303927006	0.056232571	501.4427215
22.10111309	0.900638363	0.232159146	0.018594791	350.5908608
26.43068439	0.86082381	0.174483741	0.040803977	316.8663663
19.15252233	0.911154845	0.31074109	0.033677384	254.0720715
18.17721297	0.903340346	0.23639792	0.00330834	223.4320535
9.142471226	0.90657502	0.303059652	0.193597145	159.561683
29.26570935	0.883938906	0.178960282	0.049490525	379.5538211
29.27515368	0.845525835	0.155723641	0.052684331	259.0830543
31.12991047	0.836552685	0.146673518	-0.003928686	296.1531239
15.747244	0.874460604	0.208424602	0.105212349	270.056058
27.75626983	0.885915302	0.216774179	0.021545732	294.8468825
21.21674509	0.897586513	0.204946848	0.009684633	709.1841312
12.89711821	0.861250872	0.208550921	0.107872929	186.2731949
23.88370627	0.883004201	0.18425631	0.040417446	397.2181999
23.08052729	0.887682159	0.244580031	-0.016227952	335.0285559
18.67434328	0.869197165	0.196053289	0.104878257	262.0626813
20.56587901	0.868483619	0.177990831	0.03794796	326.295287
24.48334627	0.866545666	0.179093721	0.059932209	265.9157969
22.09426686	0.883327422	0.172715797	0.04039166	444.2163173
14.24191701	0.895420272	0.255732769	0.068136396	175.0303422
31.4067163	0.897881987	0.175727343	0.026250077	876.2781648
19.76832024	0.866045807	0.176357294	0.019982681	286.7569613
19.49739784	0.86169016	0.198861016	0.151240073	194.3411705
23.1436558	0.876693368	0.168328613	0.060771442	448.5068559
19.11476534	0.893078613	0.226103206	0.007882168	331.8664532
20.60643108	0.855781421	0.180519966	0.061088702	218.5299798
21.0139022	0.911295796	0.227352331	0.040522468	640.679362
13.40513111	0.872460566	0.211176005	0.091335585	154.5896121
17.74533166	0.913721652	0.32239013	0.059601653	271.5915887
35.57721628	0.852646156	0.138248336	0.01974122	394.805744
31.16581056	0.878103556	0.155359707	0.075495812	538.4094348
29.5139645	0.867673665	0.162525188	0.052986411	495.5294547
22.6515681	0.889971806	0.226300422	0.009959297	256.4393485
13.15011955	0.912027987	0.238301053	0.090756419	638.5489523
20.4020591	0.887996001	0.188559153	0.078120783	432.6964579
25.75023543	0.905812032	0.218638424	0.057655563	658.0438856
14.15161157	0.914148497	0.291082479	0.062923416	463.4242052
17.24382529	0.949613258	0.356130806	0.065654217	2328.707348
14.19852855	0.842894628	0.205997634	0.136776985	117.1177432
29.4118531	0.877832245	0.194605181	0.015649358	355.0917
27.09237808	0.835100249	0.142138196	-0.004135809	282.6700545
20.9308129	0.902012019	0.208747773	0.120351182	496.0731543
18.66536604	0.856149849	0.168580188	0.041026719	316.2823454
28.22825773	0.891208185	0.187821062	0.059511045	413.8407219
18.54245157	0.891426137	0.210994335	0.08373835	442.8333757
16.79383104	0.879753871	0.199308683	0.073931236	286.0388495
17.62563858	0.90831596	0.275126118	0.038184783	366.1129513
14.86170087	0.92326213	0.28457736	0.039131871	288.7454732
23.45168169	0.915460779	0.254089977	0.025901533	571.1569069
32.07059781	0.865665631	0.139144531	0.038716732	432.3624669
19.20027892	0.870387009	0.181802286	0.049191453	256.8108284
20.25963385	0.850878004	0.185940656	0.067583116	157.8172417
19.49142902	0.867646975	0.191263464	0.051208691	281.2806656
23.17968577	0.895408213	0.202502854	0.02339435	290.204342
27.23021607	0.865492538	0.151456979	0.039841244	398.4015146
40.58915344	0.865037539	0.125164423	0.02987072	589.4144672
42.79153295	0.912843436	0.169335775	0.022601111	2198.566439
18.76293698	0.860003851	0.206422591	0.186284056	246.287619
15.64843621	0.88681813	0.222912389	0.064614794	243.6129325
15.62624749	0.932757484	0.280146162	0.029147778	652.385604
17.02014837	0.869009766	0.187988145	0.070545319	321.0408776
21.90671114	0.856730215	0.160123042	0.072242571	395.0104789
12.5915615	0.864718033	0.208539867	0.033610454	314.3201319
36.14211588	0.825840896	0.153514215	-0.05134068	357.4432135
26.04664054	0.916730277	0.233843825	0.031046132	1250.281784
37.26057957	0.878034982	0.159311139	0.017770041	548.6195829
27.84618441	0.890110359	0.242265254	-0.009246197	402.8238313
20.42179466	0.90591062	0.236561539	0.023869873	366.0453851
16.40549814	0.920038144	0.255895675	0.071310034	412.9538165
47.88555186	0.854053394	0.124055948	0.051772369	560.0151067
22.5517292	0.900314712	0.212157287	0.092033101	570.6776711
23.25821003	0.828657138	0.155880832	0.119545771	233.5969934
12.21694601	0.8838815	0.219911671	0.163228292	221.4304342
28.33093688	0.895808836	0.235473153	0.03798451	319.0991365

log.sigma.1.5.mm.3D_glcm_SumEntropy	log.sigma.1.5.mm.3D_glcm_AverageIntensity	log.sigma.1.5.mm.3D_glcm_Energy	log.sigma.1.5.mm.3D_glcm_SumSquares	log.sigma.1.5.mm.3D_glcm_ClusterProminence
4.969455352	16.58452165	0.004042153	33.76012499	13517.38757
4.607787526	19.432645	0.01190314	19.82518093	7126.821265
4.870010133	13.61999619	0.005818566	36.66391764	18905.14732
5.264671143	24.47303507	0.00247385	45.11931536	28188.22554
4.728112486	14.39462501	0.006582317	22.3229649	8936.716232
4.699688846	16.0813269	0.005042488	21.62395133	5012.039015
4.435144323	19.37397366	0.009216562	19.27806926	5645.246302
5.105842884	18.46748639	0.003101219	37.10523462	15159.33013
5.004274084	19.29696514	0.003320428	29.12769294	12606.93967
4.802574442	20.28375048	0.005726722	24.17583002	10496.5691
4.668734982	13.86972085	0.00509699	19.56382031	4532.972061
4.968691739	27.64584395	0.00444614	32.97632913	17593.81006
4.658291026	13.76088	0.005137991	18.64818212	5614.601924
4.650266125	12.47685294	0.005815008	17.44810049	4597.69303
4.798672727	19.04686835	0.004356981	24.42955711	8022.407697
4.056171055	16.69117604	0.038023909	12.65168624	5028.513775
4.76480277	11.93096229	0.004831467	21.52367998	6178.834378
4.709702039	15.57148493	0.005828181	19.88068437	6047.376646
4.934300278	16.80442414	0.004534019	28.8443071	10653.92986
4.36785319	14.37846364	0.014605531	12.80980934	3011.053335
4.502397368	19.22407378	0.010662292	17.21130344	5214.379165
4.825347155	19.68778463	0.004313603	24.24516111	10458.6172
4.57675052	15.32501426	0.007949578	17.53576546	4449.657487
4.961153336	18.96664711	0.004398875	33.21238244	14730.25777
4.550898216	13.04811011	0.008027697	19.61949024	5870.796354
4.792368832	19.05732095	0.005954929	27.09193551	11111.67293
4.483148953	22.49818762	0.012355511	16.4509109	5675.600777
4.673043965	19.06720409	0.007307297	23.569373	10341.44036
5.04517043	18.19169063	0.003216399	33.5204825	13476.05214
4.526262049	16.18546098	0.014311016	19.03925074	6670.125101
4.598862448	15.23112329	0.007235895	20.42869118	6901.056708
4.426800275	12.55374076	0.010062159	12.50970205	3248.823054
5.107029897	19.89470471	0.003049025	35.04367975	16936.80226
4.601896854	16.37580119	0.012929984	38.97918927	23374.81442
5.014381573	17.66143149	0.004497739	40.46707645	16926.70269
4.786644989	16.57356534	0.004301053	22.08622457	5773.277492
4.939269354	17.60667166	0.005626309	31.23470248	14497.30679
4.840244882	27.0092741	0.004469287	25.40180932	8354.038433
4.675721558	13.57352118	0.005386515	19.42415762	5199.112227
4.94834493	20.30967752	0.003881114	29.16858687	12003.33181
4.715326725	18.69659354	0.007564484	23.63727901	7942.020303
4.8776335	16.34793685	0.004254526	26.04981838	8313.9807
4.900004623	18.42260795	0.003525872	27.27383985	8610.288556
4.991227103	16.58913119	0.003715063	31.53216526	13370.38454
4.947910057	21.3866876	0.00340195	27.95367789	10258.30825
4.537140717	13.26792839	0.007930106	17.45132312	4622.172925
5.118083548	30.05471	0.002975265	36.10014382	19413.50413
4.81897809	17.29391984	0.004418899	26.81912787	7758.386598
4.500022497	13.81764939	0.012524453	27.61670077	14022.89594
4.941400297	21.51112368	0.004588771	29.83247873	12292.41709
4.711597198	18.5811396	0.005644766	21.38020018	6724.924491
4.883686158	14.90565738	0.004076391	28.72989199	8465.973273
4.823203891	25.6534819	0.005282925	24.61147208	9429.240109
4.634545096	12.36962161	0.00577299	18.45607331	4848.280238
4.49194208	16.72976736	0.016062139	17.89178711	5939.687388
5.242819622	20.42169277	0.002658511	45.51946729	24897.88304
5.228664297	23.633655	0.002463152	42.94934749	22931.2613
5.098697835	22.61384108	0.003493861	35.35443004	20256.87208
4.749604541	16.34916494	0.006975566	24.50957852	9176.310634
4.522272928	25.34807488	0.00749967	16.43566101	5210.72679
4.896897248	21.05909507	0.003875623	25.86442168	9821.199013
4.96558198	26.01834682	0.004581461	29.69673741	15488.31605
4.485455621	21.67024452	0.010410552	14.61126646	4148.790613
4.344157912	48.46109958	0.020128341	14.98851877	11365.20466
4.612117191	10.68991666	0.008180103	20.47238678	5969.792567
5.01264569	19.32061407	0.003973815	33.00736678	16005.7925
4.997081392	17.22837868	0.004311136	37.07895943	13394.30215
4.971491418	22.43702347	0.003771328	27.2149018	11155.94345
4.802581791	18.09722985	0.004827445	26.49889045	7217.870616
5.069433852	20.70042354	0.003489184	34.14847683	16017.39808
4.809368593	21.29003918	0.004819343	22.81647848	8267.824151
4.769966833	17.19613142	0.004502368	22.46885441	6668.586991
4.584921992	19.39032339	0.00900913	18.26854177	6326.97658
4.453687534	17.2528205	0.01048483	15.38758917	4524.395281
4.753789474	24.20705126	0.007220497	24.13792474	11090.91094
5.206869361	21.24957257	0.002548807	41.79506598	21363.82439
4.849574137	16.31844336	0.00401956	26.25671477	7986.32964
4.849340003	12.52642657	0.004747507	27.48000262	9107.591526
4.871040776	17.08004695	0.003967992	26.38420854	7867.563907
4.877785987	17.4334479	0.004561662	27.02946832	11077.79918
5.023909537	20.33789115	0.004124207	35.80465691	17679.24883
5.37425265	24.88031385	0.002117548	52.88661296	32683.57145
5.288861449	47.52683214	0.002884133	47.70636878	35046.02354
4.635896424	15.64399093	0.010762272	28.28265278	13317.27669
4.700626249	15.81963087	0.005283611	20.57505766	5126.985041
4.433942258	25.83175414	0.010263822	16.10728953	6361.152607
4.786599997	18.19419209	0.004793795	23.8776441	6793.556315
5.022736313	20.2500115	0.003128934	30.89924414	11474.60376
4.393945522	17.87165517	0.010162782	19.05853357	4306.446784
4.556722189	19.44553286	0.012985661	41.10151466	18690.2866
4.863137583	35.71378179	0.005733186	27.06423356	16186.57357
5.159359603	23.90340434	0.003455002	45.8916917	29023.68576
4.815889	20.44520243	0.008163228	27.38968303	11725.87338
4.743242254	19.42010761	0.005470712	21.70690476	8404.753766
4.649556721	20.47207175	0.007079232	18.56686901	5832.841676
5.305526939	24.28187417	0.004678757	63.96515357	50365.80704
4.871604534	24.08417881	0.00483985	25.65688362	16095.68801
4.753683221	15.22771527	0.009556146	36.00391775	16131.53059
4.609065705	14.82526282	0.006917843	18.59635513	6289.140613
4.772606503	18.26100717	0.007109754	30.0606028	19350.29406

log.sigma.1.5.mm.3D_glcm_SumAverage	log.sigma.1.5.mm.3D_glcm_Imc2	log.sigma.1.5.mm.3D_glcm_Imc1	log.sigma.1.5.mm.3D_glcm_DifferenceAverage	log.sigma.1.5.mm.3D_glcm_Id
32.25612817	0.843778853	-0.138379869	6.311179441	0.257422574
38.28094423	0.746849713	-0.100628395	4.247640631	0.372553826
26.45909011	0.911330513	-0.201410648	6.111489905	0.272473123
47.85624688	0.709566948	-0.073585591	7.05995223	0.244980987
28.64904072	0.886723864	-0.184800381	4.565186773	0.299732546
31.82497381	0.750707346	-0.098863899	4.828643659	0.293054602
38.3244401	0.865248202	-0.174940622	4.71255255	0.307106714
35.97819725	0.776104642	-0.100989998	6.654891067	0.243194346
37.87357293	0.652584494	-0.062441471	5.642552584	0.271371354
40.00425321	0.634384962	-0.060784147	4.924313513	0.308663777
27.72547061	0.694945744	-0.080024507	4.705399711	0.294548397
54.3515434	0.784512715	-0.107430858	5.90611675	0.277508429
27.29499117	0.575184084	-0.049204252	4.381248692	0.307846702
24.95370589	0.65420407	-0.073080365	4.155682745	0.322142947
37.34177599	0.741390519	-0.092803389	5.501568049	0.263560203
33.0455267	0.693885846	-0.095479267	2.943729669	0.471891862
23.85337381	0.749488291	-0.099154953	4.689782018	0.295660957
30.80788784	0.636229724	-0.064039737	4.170261249	0.332610385
33.0773701	0.843995444	-0.142628586	5.396570081	0.271179301
28.69317292	0.657707644	-0.079780499	3.223105142	0.414977674
37.91313706	0.552160797	-0.046279153	4.231172516	0.349484831
38.79463109	0.664006827	-0.068942793	5.168259647	0.278276874
30.37866633	0.584914776	-0.056510845	4.015441894	0.346847556
36.99709331	0.899946109	-0.184131245	6.425060703	0.248762506
25.68626603	0.717707578	-0.090321525	4.326062601	0.33590094
37.32500986	0.864368458	-0.158544554	5.340898853	0.299971282
44.74558601	0.634731895	-0.065899707	3.83835072	0.37901709
37.42990759	0.728347523	-0.091560016	4.848104391	0.316655316
35.54315493	0.735366821	-0.089459918	6.109796866	0.262150641
31.8458502	0.711419136	-0.088477031	4.013669284	0.382750721
29.89691059	0.580957667	-0.050471726	4.544705146	0.321949985
25.07334887	0.51017522	-0.042717989	3.245639181	0.383900978
38.88564783	0.727549405	-0.0825998	6.186813624	0.265544859
32.07658271	0.986450148	-0.440736874	6.709737333	0.243140015
34.44527667	0.949922374	-0.25443952	6.990323644	0.23415607
32.7328982	0.590077616	-0.050839317	4.718745812	0.298255134
34.31391012	0.693545766	-0.074954946	5.699404234	0.297973573
53.25736282	0.580352901	-0.047559862	5.308234143	0.290816826
27.14572979	0.700727383	-0.08447503	4.59672033	0.299244607
39.8073988	0.779901044	-0.105930824	5.778132139	0.271569456
36.63643146	0.74927998	-0.097766715	4.938437983	0.324223817
32.21731473	0.745249472	-0.093684016	5.132357921	0.286013308
36.07854105	0.665799003	-0.067303336	5.633499552	0.267794794
32.50716717	0.79369942	-0.111267881	5.792403545	0.268031816
42.10534573	0.670121034	-0.067378527	5.726335485	0.263547284
26.36947577	0.62355773	-0.061967795	4.090959927	0.339755656
59.17929862	0.610987128	-0.05094196	6.2954952	0.261955028
33.84326877	0.814107708	-0.125213358	5.576640758	0.26681805
27.58881962	0.959445321	-0.32261142	5.172922184	0.287648583
42.27503308	0.872535044	-0.161967921	5.962816621	0.25816468
36.43346229	0.586365881	-0.05066169	4.842734561	0.311167954
29.451129	0.772328279	-0.105122494	5.592665657	0.270337142
50.58843332	0.619891553	-0.057122769	5.039174679	0.310278197
24.73238254	0.756825429	-0.103866017	4.469134358	0.302618517
32.90931405	0.669848949	-0.076307242	3.880265865	0.393005354
39.71010527	0.856980588	-0.139546742	7.439820042	0.224774698
46.28052639	0.725193686	-0.080847289	6.818369313	0.242095257
44.44622776	0.772976739	-0.099735009	6.403173962	0.250658958
32.01985883	0.811588202	-0.126073363	5.049853849	0.309119418
50.47909612	0.719927783	-0.092600548	4.033621475	0.327103249
41.50752506	0.644657923	-0.061745769	5.339110041	0.278346716
51.24700072	0.623332513	-0.056247314	5.428248289	0.301430441
43.01360593	0.628875078	-0.065256852	3.786167611	0.369866938
96.49311361	0.728079165	-0.101749261	3.419056125	0.423179456
21.37983333	0.920403433	-0.231637743	4.579595881	0.298226498
37.67507174	0.769041434	-0.098982916	6.0171272	0.278330946
33.64944356	0.907894207	-0.193708369	6.78870241	0.231519595
44.39958963	0.634093024	-0.05919905	5.170549638	0.295286971
35.51593684	0.855270052	-0.152466422	5.520942475	0.261138803
40.59795601	0.72838173	-0.083797274	5.998549183	0.273142463
41.99724124	0.610668217	-0.055603234	4.913126532	0.298790319
33.73535763	0.698823858	-0.079404622	4.915022696	0.290106049
38.23615723	0.609724251	-0.058616768	4.207951177	0.354825185
33.95421305	0.563969451	-0.049536976	3.859559487	0.363587307
47.77669061	0.574493796	-0.047480236	4.743001848	0.333967186
41.51665193	0.807323337	-0.111778533	7.122008287	0.227566388
31.9813828	0.721516026	-0.084714108	5.373085977	0.272883966
24.97456474	0.8118489	-0.125211296	5.457562417	0.275128746
33.47228406	0.648938086	-0.064052794	5.364796218	0.27974143
34.04379498	0.644619332	-0.06228103	5.460734653	0.288302318
39.84765288	0.904951524	-0.188094909	6.466173803	0.241467053
48.5074207	0.817819401	-0.113385208	8.010340311	0.210737594
93.7653062	0.753112343	-0.0879255	7.193562007	0.251610461
31.06384406	0.975161899	-0.364206666	5.075152601	0.293667462
31.13831137	0.546438997	-0.043456227	4.593810989	0.310452487
51.06974563	0.555954002	-0.047553807	3.863212944	0.360302079
35.74862518	0.756728562	-0.100121749	5.103051879	0.278876652
39.64418928	0.66173765	-0.064741923	6.042993972	0.250459508
35.42394631	0.942966311	-0.278205069	4.636855333	0.298148922
37.94391921	0.991903724	-0.486868039	7.20988663	0.238668141
70.69639162	0.66850756	-0.068390969	5.217046142	0.314780175
46.81581487	0.899478822	-0.176525581	7.022940022	0.244727488
40.16094863	0.842439102	-0.142260355	5.369942749	0.31984705
38.24145788	0.616757281	-0.056995088	4.753905259	0.320318887
40.57928116	0.530662503	-0.041343256	4.211554249	0.339481921
47.21003571	0.982244759	-0.350605777	8.517774189	0.208596871
47.67722111	0.763554094	-0.101760247	5.070530487	0.299657343
30.29232628	0.986082065	-0.416908134	6.230690491	0.246185687
29.56013116	0.851195712	-0.158614095	4.26866117	0.31138274
35.67542928	0.862997851	-0.159831818	5.194228527	0.317461811

log.sigma.1.5.mm.3D_glcm_ClusterTendency	log.sigma.1.5.mm.3D_firstorder_InterquartileRange	log.sigma.1.5.mm.3D_firstorder_Skewness	log.sigma.1.5.mm.3D_firstorder_Uniformity
65.52915015	197.8685207	0.267015818	0.051830125
41.50485267	98.79780197	0.349766853	0.088234878
68.19068149	169.6250134	0.921568223	0.058389433
91.52081255	205.3676376	0.20791668	0.04631234
51.79131228	144.5504074	-0.138775685	0.070413793
44.06860873	158.809948	0.006445079	0.063584626
37.10417758	102.2261086	-1.155296895	0.093625901
76.07298559	224.8846779	-0.055566548	0.049019725
63.37618496	185.9077873	0.042518667	0.052924166
50.86381542	140.5749378	0.428138585	0.069045358
41.37447239	164.3378754	-0.124406685	0.065581369
66.06079031	164.3770542	0.199620305	0.057470574
39.73444739	145.3311787	0.255804045	0.068890771
40.27904116	149.926363	0.120773637	0.068823384
48.81646055	155.8574982	-0.170543531	0.061438378
25.7456058	39.95360661	1.014829346	0.171672402
49.35183409	176.2165508	-0.005788747	0.060869779
43.53699612	138.8483467	0.388509952	0.069577862
62.77472455	183.5449028	-0.175088285	0.057450904
28.00757505	87.89531708	0.258948497	0.103162651
34.58110566	101.8539762	0.540174828	0.089132441
52.90004187	158.7002735	0.426381591	0.060361682
36.91394055	127.6901245	0.383982185	0.080934806
67.10038873	178.065712	-0.24020677	0.055744054
37.25892363	122.6055927	0.755830107	0.07594287
53.37983302	171.9471664	0.080694985	0.058355272
34.52872333	88.72593498	0.376742026	0.095696585
47.12404358	130.5709915	0.692787988	0.072245335
69.06015535	201.4868164	-0.132862289	0.050224623
37.52077651	90.18672323	0.603167517	0.096235641
38.87751113	118.4094429	0.762494563	0.079122279
29.40010715	100.6470089	0.515169576	0.093591906
73.94482672	191.9366302	0.116293164	0.050494195
81.64784636	153.3322792	-0.630687515	0.074149408
78.86311109	230.8792	-0.26091821	0.047539556
46.92540641	159.5452938	-0.07988444	0.062776198
62.48084752	163.7826271	0.502894843	0.063430507
49.85205214	155.9933014	0.052716786	0.062496955
42.90190738	147.6790123	-0.360050777	0.068682276
61.67925082	190.8965073	0.119870981	0.054184793
45.53901949	135.5301905	0.179102356	0.069769262
55.48066488	177.6065979	-0.021184034	0.058263021
56.33481602	186.1687164	-0.175352391	0.055556709
65.4002081	186.0353546	0.019539604	0.053696609
59.13732805	181.4006519	-0.110637856	0.05576197
35.84824844	123.872139	0.538108423	0.077636028
74.13518633	185.1694279	0.017352613	0.051147122
52.64087123	190.3794632	0.066096299	0.056586121
62.50179232	106.6163769	-0.883184791	0.095121408
65.8072062	175.5578384	-0.307116415	0.059750859
42.836741	135.0849342	0.28727289	0.068678555
59.12942843	192.9328384	-0.189533233	0.056416675
50.21437861	146.0613251	0.18135429	0.065048379
40.11659324	146.378582	0.066881888	0.06810276
36.57506865	67.61594009	0.455798602	0.116836146
93.55000355	232.2883759	-0.001691561	0.042836194
90.62707974	233.7238998	0.04637084	0.044679949
77.97278143	154.4190712	-0.59629685	0.057883816
48.75891661	139.1707764	-0.030880775	0.069206575
35.53303964	130.8631363	-0.319124898	0.076346274
56.98630363	176.6565323	0.119181671	0.057024904
61.44138679	158.8146629	0.381018473	0.061535398
32.22218577	94.83612919	0.135562335	0.0962359
33.03641651	69.75389481	-1.477860642	0.11547832
46.59953927	159.9332733	-0.013772387	0.067255622
66.94735713	157.8591995	0.219033459	0.058082958
71.90984112	223.2036209	-0.390050432	0.051882295
60.83735801	169.381958	0.067980052	0.057885731
52.99282838	184.956789	-0.249714333	0.05728046
71.78926547	176.5237503	0.219956334	0.055535183
50.66037673	153.3157978	0.342645698	0.063728575
47.18971303	163.8576508	-0.007891783	0.061148982
37.99193802	111.4774733	0.495950698	0.084699829
31.96619616	92.41208172	0.454665527	0.093864279
48.08501172	108.0842237	0.255578014	0.077888466
89.16457593	224.4244289	-0.228867488	0.04539221
52.76166693	177.7607574	0.082673752	0.057705887
57.82636392	206.2915001	0.169803158	0.056404192
53.40117323	188.3467121	0.035437978	0.056690628
55.06286662	173.5968342	0.362151312	0.059606781
75.12740455	226.2088871	-0.009876527	0.047264739
109.8159519	265.1077576	-0.189875498	0.039400405
97.42571096	176.1456699	-0.119615861	0.052358714
64.85531053	121.8991737	-0.410046265	0.076337693
41.85649658	145.7661552	0.253696884	0.069545115
32.07524148	100.1729317	0.664620701	0.089827328
49.34277898	184.5111446	0.113135467	0.059367173
67.10060575	204.0055885	-0.343271363	0.052719637
37.94989592	136.5474987	-0.423560805	0.071217415
78.76645883	183.3174744	-1.12257421	0.070839002
56.68595971	134.2933693	-0.52774853	0.067339045
89.7022451	212.4548378	0.393299686	0.045871486
55.29444223	145.6569786	0.065874038	0.068845478
45.00412562	135.6751471	0.300789515	0.068167574
39.37513145	117.4192772	0.358532478	0.078439115
132.850819	285.182869	-0.019494599	0.040291209
57.96316309	146.483551	-0.772091662	0.064933291
78.78013474	179.5576363	-0.474701933	0.063784784
42.45228718	130.2196674	-0.185958467	0.072743854
59.19562205	131.8449421	0.885182423	0.065239256

log.sigma.1.5.mm.3D_firstorder_MeanAbsoluteDeviation	log.sigma.1.5.mm.3D_firstorder_Energy	log.sigma.1.5.mm.3D_firstorder_RobustMeanAbsoluteDeviation	log.sigma.1.5.mm.3D_firstorder_Median
112.8543878	14034600.89	79.66529043	-13.44828796
76.71308127	23954033.28	45.26173902	-7.732925415
109.0823887	8893736.827	70.50970709	-28.35186958
124.5891356	67434728.98	84.51720627	0.262867391
88.32302272	4604633.084	60.18167804	19.16345787
90.42514306	7838263.244	65.99704125	-8.97549057
72.30516934	3320460.249	42.92160019	14.21153641
123.5345971	21666109.91	91.94369682	7.230230331
108.3062224	37650398.78	76.67911014	-7.739180088
89.98955818	44819970.72	57.46493389	-19.25100231
89.40231354	9599962.126	66.36994008	-1.128772259
107.2554862	30331348.87	69.49194692	-17.58112907
83.85298158	22743566.86	59.18308281	2.807612419
85.10349152	12022949.48	61.61891919	0.130934263
93.34828754	13008527.22	64.08180612	7.300168514
49.36072774	45170928.02	22.31360976	-3.712430239
94.94325377	9167356.68	70.39416457	-0.620630801
82.9259513	26088452.53	56.70947608	-7.922209024
105.1629056	9839723.134	74.62131076	26.87979221
61.58599048	20128332.36	37.83784017	-3.440692186
75.99014899	57699262.86	46.1663122	-16.56090069
95.64069286	25520006.12	66.01305162	0.459815025
77.53662013	27074644.23	52.38718738	-17.08479309
108.9198552	9443232.331	73.65676457	21.84441185
81.51149589	14433685.52	52.97387018	-28.53053474
102.1352126	12146649.62	69.19044211	-16.28013039
68.38641347	32509120	40.20502934	-7.82446456
89.39362821	21248219.62	55.33043996	-26.618927
114.2950822	34450498	83.06651714	17.41170692
72.97910205	28234964.42	42.32177189	-11.95871258
80.28292026	37555008.55	50.79930224	-23.06641388
64.99826673	43470678.15	42.35770356	-5.768797874
113.1418427	41827826.65	78.52765837	2.020337582
110.5706806	2236338.247	68.77836366	49.45978165
128.9398382	8097815.089	93.22594541	22.77039528
90.67825715	24864141.17	65.51935978	-0.962546349
102.450902	52969582.57	66.9383768	-13.38809109
94.78210358	49763318.25	64.52361639	-8.932673454
86.19469635	9426347.735	61.83732898	23.46245956
106.9752257	18772517.01	75.97463703	-7.553629875
88.86940834	20976112.34	56.26359145	-7.534105301
101.0272325	14149182.44	74.356579	-3.664666653
103.8538148	25921964.21	76.48245201	4.389392614
108.854115	18929373.56	76.24782275	8.817716599
103.8309552	30944982.99	74.45891939	4.426675797
78.01728598	23196916.39	51.81723742	-12.13532019
113.0812299	92307578.11	75.74187649	-0.91868031
104.1190292	9316264.076	76.52339345	-9.465143204
85.92434818	1836149.246	49.49018214	28.62036037
103.9842389	8507923.869	72.40244137	26.63554001
85.79649658	43355496.92	56.18261362	-8.283165932
106.6180402	13314024.88	80.96266007	2.928744793
91.14143282	54553773.12	60.51188393	-7.021806717
83.92884248	6507763.343	59.28241901	3.811033487
64.48276274	58863441.16	34.75154937	-4.281490326
132.2757612	23440919.57	94.83262858	-6.118835926
128.6985081	53775419.87	94.34264831	1.323242188
108.2960804	26041977.35	69.95807994	25.90699005
90.89518531	12316259.43	57.45163857	-5.261254311
78.77292384	8959569.141	55.01603256	-12.2185955
101.1100687	33636799.36	71.69233895	-16.48397636
98.35619293	92996912.08	64.05297946	-3.980248451
67.11614617	26630958.72	41.04143581	-2.904860973
61.63113646	23613323.13	34.74376561	-1.663783908
90.32273023	2686784.364	64.23972696	-7.397643566
101.8288635	28341673.65	65.62498374	7.970613956
121.3461265	8384482.91	89.50272141	16.20114803
98.51377039	49352333.88	69.06944614	-6.565014362
102.5080494	6976484.865	75.08856814	-11.82358122
106.1556152	37778117.95	71.66517643	-10.996665
92.74169558	42460046.42	63.25488569	-15.49412489
93.41292679	14954939.65	67.51249504	-5.782476425
75.63375397	55803897.1	46.5667566	-8.17250967
68.88655976	61469203.04	41.94814227	-12.24023008
81.42590248	121449053	47.88762008	1.476020753
128.3629196	27852830.78	92.28275756	3.115666747
99.71022409	16773516.43	72.22339705	-9.706572533
108.6117112	10378873.08	82.09960013	-29.7094059
101.6220637	27529988.63	74.23997495	-20.52452183
99.89813126	46548634.76	68.94718779	-16.11054325
124.6559193	12064255.07	90.1961201	-20.92284775
147.1016758	42848583.66	107.8729166	10.36084843
117.9234505	70819035.44	75.55310404	6.172631502
91.08385002	2237754.912	54.93477748	30.80579376
85.40839168	39155331.14	59.22220747	-11.19570637
68.83691749	50793033.18	42.41354859	-7.211806059
99.27725984	11412353.82	72.85373783	-15.5481782
113.8803744	28789815.03	85.02013854	17.39769936
83.19834568	2076561.928	56.98178743	21.78529167
120.1978769	2525667.157	76.72095445	28.13780022
90.5885397	61581241.71	57.24522599	-0.316879749
130.6378368	19995257.74	89.11715918	-23.38002777
94.39819365	16607486.02	60.0131718	-6.662675619
85.65893465	52744976.9	56.19241435	-7.520417929
77.88715732	62318062.2	50.32387016	-11.71461296
163.2265351	10065233.42	118.6672105	-22.80246735
93.19407925	18856076.78	61.06162433	18.88799572
109.5690301	2292594.94	75.3027401	44.954422
81.28610081	4236553.717	55.00400419	24.15991402
100.3773015	15602240.6	60.28704382	-23.75662994

log.sigma.1.5.mm.3D_firstorder_TotalEnergy	log.sigma.1.5.mm.3D_firstorder_Maximum	log.sigma.1.5.mm.3D_firstorder_RootMeanSquared	log.sigma.1.5.mm.3D_firstorder_90Percentile	log.sigma.1.5.mm.3D_firstorder_Minimum
378934224	548.6444092	140.9930427	188.2714691	-397.5913391
646758898.7	424.2545166	107.3917887	140.6968231	-456.6477966
240130894.3	540.6573486	143.1519513	190.6081772	-309.7500916
1820737683	615.1563721	159.9747715	215.5277435	-574.9212646
124325093.3	409.6469727	113.2531501	119.7939423	-343.6558838
211633107.6	336.1830139	110.4089897	129.2437439	-385.368103
89652426.71	244.8308563	99.26226761	97.7168457	-472.3294983
584984967.6	445.2237244	147.0471495	179.6578094	-430.0306396
1016560767	419.4113159	134.5728796	161.5891083	-452.4830933
1210139209	556.6009521	120.2804592	153.7997925	-496.303833
259198977.4	277.385437	107.7412488	125.0109116	-326.6747742
818946419.6	601.9699707	143.061166	187.7751373	-652.1460571
614076305.2	519.1832275	104.7188615	132.1713287	-323.2597046
324619636.1	321.662384	103.7013976	141.4243317	-294.78302
351230234.9	440.7718201	119.4964151	159.7644958	-447.0220032
1219615057	601.3790283	80.63634923	92.7744751	-397.6672058
247518630.4	346.7459412	114.602569	133.8763184	-278.0727539
704388218.4	384.76828	105.7693625	144.1819443	-350.9222107
265672524.6	362.2128296	130.0259235	172.6225662	-378.0758057
543464973.8	339.2271423	84.77100717	114.8614426	-347.8688965
1557880097	470.6703186	105.0953615	140.5778473	-463.7990112
689040165.4	540.8240967	121.8080985	167.9351761	-469.3050537
731015394.3	351.422699	99.56817094	142.9964905	-364.8086548
254967272.9	440.4619751	140.1160221	166.9713593	-448.5886536
389709509	606.628479	109.490232	137.2392044	-301.5415649
327959539.7	383.0473022	132.2965494	163.703009	-474.9568481
877746239.9	456.7665405	96.84748063	122.6788177	-534.3618774
573701929.9	521.003479	121.7694034	150.7024017	-469.2723389
930163446	405.1917725	139.7093259	187.1666656	-420.6776428
762344039.4	506.843811	105.391564	121.186792	-391.6451111
1013985231	592.1546631	108.6897697	136.2743256	-354.4502869
1173708310	424.2622375	85.61913994	121.6942589	-291.9622803
1129351320	577.0905762	143.4379139	186.8933716	-468.2554016
60381132.68	418.0873108	146.6398731	154.5312241	-393.7287598
218641007.4	366.6212769	156.1762124	196.0467514	-414.3544922
671331811.6	349.3604126	110.9458462	138.8754181	-391.5556335
1430178730	551.6245117	135.6179051	186.7689896	-420.1958313
1343609593	405.6445923	121.8072495	165.7497772	-645.2178955
254511388.8	302.7845459	106.2498904	134.7276123	-315.1099854
506857959.3	492.1988525	133.7745532	160.7512573	-476.5101318
566355033.1	402.4176941	119.2926698	160.8575836	-446.0144958
382027925.9	359.4451294	121.8482294	143.0236053	-380.6743164
699893033.5	386.5418701	125.6456267	149.9751404	-426.7398071
511093086.1	425.0353088	135.8958481	174.1928833	-399.6000366
835514540.7	457.2218933	128.3652084	146.6443527	-513.1040039
626316742.5	465.0146179	102.0369657	136.4981873	-305.8586731
2492304609	549.8262329	147.0985029	191.5226974	-709.3766479
251539130.1	382.7341309	126.4115096	150.9781708	-408.1653748
49576029.63	352.5107117	117.0581322	110.2080498	-349.6468506
229713944.5	449.8097534	130.8379115	149.0238373	-501.4210815
1170598417	462.9481201	112.5266024	146.7369781	-426.5800476
359478671.7	357.2359619	126.8825585	142.0643829	-353.0359497
1472951874	551.348938	119.487886	154.4788361	-621.7017212
175709610.3	375.1418762	104.9354273	149.4984131	-294.7407837
1589312911	523.7248535	94.99464726	127.06866	-376.0647278
632904828.5	454.4717712	163.3025193	196.53591	-475.7036438
1451936337	544.6760864	156.5930883	196.7753845	-569.5432739
703133388.4	382.3879089	141.9180764	168.0007507	-529.8852539
332539004.6	515.1732788	123.0062593	145.6363525	-394.8799744
241908366.8	327.9934692	100.8452875	124.5305328	-609.2998657
908193582.6	433.0587463	125.9916762	165.0784546	-518.7775269
2510916626	552.791748	130.2936499	169.2411804	-605.0505371
719035885.3	377.6118164	92.83551713	128.4571854	-505.4577332
637559724.5	299.2799072	92.98602976	113.0093613	-1189.220947
72543177.83	261.4502563	111.2721653	141.6821045	-262.1440125
765225188.6	506.6498718	135.0911873	195.1885132	-439.8507996
226381038.6	346.7383118	145.8780774	166.635202	-411.0783386
1332513015	545.5215454	124.2460325	161.8040985	-537.0612793
188365091.3	282.3848877	125.0693517	140.9821243	-448.9916687
1020009185	622.2571411	137.0610719	188.8464203	-494.150238
1146421253	387.3053284	118.1825729	163.6599823	-505.2660522
403783370.6	393.8359985	115.0920595	134.9034271	-402.8722839
1506705222	463.9074402	103.9132701	137.3378479	-453.5136108
1659668482	646.3430786	95.66948751	125.6600342	-421.2084045
3279124431	583.6596069	115.0206257	142.3678436	-556.4119873
752026431.2	520.4624634	157.9801686	183.1417236	-513.8118286
452884943.7	424.5255432	122.5421435	142.5107513	-395.0916138
280229573.3	355.6532593	130.4398177	160.197966	-302.1672363
743309692.9	355.3122559	124.9262563	150.1469818	-413.0910034
1256813139	631.1842041	127.8898347	164.6616898	-402.4212646
325734886.9	415.4595642	154.7159139	169.6893555	-507.8383789
1156911759	572.4083862	178.7532088	223.8690338	-593.4066162
1912113957	596.0682373	159.2638818	210.5673828	-1141.107056
60419382.62	304.7235413	124.2287909	159.4642517	-363.3070679
1057193941	426.1616516	107.4562191	150.729126	-362.6901245
1371411896	614.6654053	96.30977191	116.3215294	-610.2844849
308133553.2	321.4172058	121.1149671	152.732515	-429.8435974
777325005.7	315.6578064	136.1988933	159.847934	-492.254364
56067172.05	226.5784454	106.5238609	156.2852539	-406.5795593
68193013.24	249.2284241	155.0934329	126.3094772	-487.014801
1662693526	426.4389954	124.4674105	155.0526215	-855.0222168
539871959	541.9429321	168.2911014	226.397049	-597.4005127
448402122.5	455.4045105	127.4754342	166.758168	-493.2531738
1424114376	564.592041	113.5609192	139.5012772	-468.4729004
1682587679	616.1664429	103.6020752	137.7362289	-480.6637268
271761302.3	514.1221313	199.4580941	245.8958771	-596.6520386
509114072.9	450.5599365	124.5256684	140.0918198	-564.5618286
61900063.39	286.6264954	136.5246617	175.316217	-356.9538269
114386950.4	380.5547485	105.7272056	126.8153702	-339.624939
421260496.1	538.5031128	139.0454295	182.1233337	-432.6759644

log.sigma.1.5.mm.3D_firstorder_Entropy	log.sigma.1.5.mm.3D_firstorder_StandardDeviation	log.sigma.1.5.mm.3D_firstorder_Range	log.sigma.1.5.mm.3D_firstorder_Variance	log.sigma.1.5.mm.3D_firstorder_10Percentile
4.487504696	140.9873621	946.2357483	19877.43627	-177.0909042
4.008920885	107.3832632	880.9023132	11531.16523	-134.7231262
4.410356039	143.1462009	850.4074402	20490.83483	-159.9857056
4.701832833	159.5747031	1190.077637	25464.08588	-187.1686005
4.132561687	113.1407399	753.3028564	12800.82703	-145.850766
4.148527466	109.8486981	721.5511169	12066.73648	-154.0986053
3.810226498	99.21384461	717.1603546	9843.386962	-131.0400787
4.537456244	147.0411934	875.254364	21621.11254	-186.1511551
4.454329393	134.3459434	871.8944092	18048.8325	-181.429953
4.231278455	120.1272257	1052.904785	14430.55035	-140.5505768
4.108104422	107.4378229	604.0602112	11542.88579	-147.8243042
4.476930055	143.0169387	1254.116028	20453.84475	-160.7096436
4.084648309	104.5446331	842.4429321	10929.58031	-120.6896439
4.068999409	103.6181349	616.4454041	10736.71787	-120.5611038
4.259674804	119.2895213	887.7938232	14229.9899	-140.2736969
3.307636619	80.61974036	999.0462341	6499.542536	-80.18015289
4.196723644	114.5294155	624.8186951	13116.98702	-159.9807404
4.099357017	105.7524185	735.6904907	11183.57401	-125.976609
4.357341177	129.6574194	740.2886353	16811.04641	-165.5637009
3.713490293	84.76577871	687.0960388	7185.23724	-101.2484131
3.974702632	104.809639	934.4693298	10985.06043	-126.6596901
4.290458496	121.5369263	1010.12915	14771.22446	-135.7217102
3.958995717	99.50718544	716.2313538	9901.679954	-120.8844299
4.458111892	139.881562	889.0506287	19566.8514	-181.0908661
4.053336805	108.3422664	908.1700439	11738.04669	-137.1575714
4.388431829	131.8940615	858.0041504	17396.04347	-162.6726974
3.872394974	96.82992379	991.128418	9376.034141	-113.3195801
4.187479344	121.1739496	990.2758179	14683.12605	-143.1981628
4.499977207	139.2886079	825.8694153	19401.3163	-164.452829
3.929069895	104.751586	898.4889221	10972.89478	-139.5880234
4.046256814	108.1955087	946.60495	11706.26809	-132.0318665
3.763129335	85.48756748	716.2245178	7308.124193	-94.24452972
4.5464224	143.3770707	1045.345978	20556.9844	-175.0114319
4.117514165	146.1597804	811.8160706	21362.68142	-183.6665543
4.590696511	156.0100392	780.975769	24339.13232	-203.3966492
4.178139256	110.9144506	740.9160461	12302.01534	-152.5815155
4.376908808	135.5915918	971.820343	18385.07977	-164.8472443
4.287723703	121.7882336	1050.862488	14832.37385	-138.7116196
4.074157083	105.89936	617.8945313	11214.67444	-141.5524719
4.426310152	133.7313065	968.7089844	17884.06234	-171.650531
4.224769298	119.2779566	848.4321899	14227.23094	-146.3031525
4.29176275	121.7337023	740.1194458	14819.09428	-164.4796417
4.343663732	125.5359438	813.2816772	15759.27318	-176.7361343
4.454728778	135.8895257	824.6353455	18465.96321	-169.9130981
4.382175791	128.2310113	970.3258972	16443.19225	-175.6247574
4.004766824	102.0065173	770.873291	10405.32957	-123.4181992
4.577509802	146.9756571	1259.202881	21601.84379	-171.7526627
4.329393257	126.2143207	790.8995056	15930.05475	-165.1608459
3.802285576	116.9930004	702.1575623	13687.36214	-128.1611877
4.327713828	130.7315578	951.230835	17090.74021	-184.2717468
4.18466088	112.5241839	889.5281677	12661.69195	-138.9259262
4.302997587	126.3656595	710.2719116	15968.2799	-190.1137817
4.25854721	119.4841478	1173.050659	14276.46157	-152.0708771
4.079671548	104.804256	669.8826599	10983.93208	-115.8097687
3.736667795	94.96945339	899.7895813	9019.197076	-110.7637573
4.727790211	163.1756291	930.175415	26626.28594	-223.6161987
4.667539544	156.5928102	1114.21936	24521.3082	-198.4989166
4.447972042	141.8013282	912.2731628	20107.61667	-197.0890137
4.241831208	122.8809747	910.0532532	15099.73395	-160.1106812
3.974284493	100.8231607	937.293335	10165.30973	-110.5557709
4.351592871	125.8783579	951.8362732	15845.36098	-159.585434
4.368558697	130.2444885	1157.842285	16963.62678	-155.3873199
3.821869972	92.58446291	883.0695496	8571.882772	-97.81746063
3.680143014	92.96961283	1488.500854	8643.348909	-110.2142563
4.086542245	111.2161315	523.5942688	12369.0279	-152.8880249
4.426515165	134.3189279	946.5006714	18041.5744	-148.042514
4.469372176	145.6585814	757.8166504	21216.42233	-212.4324615
4.341916649	124.2459891	1082.582825	15437.0658	-151.8876892
4.310327587	124.4764464	731.3765564	15494.38571	-178.8554611
4.454684257	137.0589182	1116.407379	18785.14706	-161.783783
4.240710765	118.1576764	892.5713806	13961.23648	-142.7065414
4.221570909	114.8279337	796.7082825	13185.45437	-155.7305115
3.998027906	103.9051385	917.421051	10796.27782	-121.6358414
3.864584136	95.42609448	1067.551483	9106.139507	-119.8072739
4.139504808	114.8783967	1140.071594	13197.04603	-126.8962753
4.662579835	157.8219668	1034.274292	24907.77321	-221.4959488
4.308119537	122.2659385	819.617157	14948.95971	-161.2746826
4.318560892	129.6293784	657.8204956	16803.77574	-175.6903687
4.3251525	124.1418319	768.4032593	15411.19443	-172.3533813
4.349287096	127.756708	1033.605469	16321.77644	-162.254097
4.608549581	153.4187761	923.2979431	23537.32087	-207.8925476
4.847080379	178.7118426	1165.815002	31937.9227	-233.6793365
4.635623418	158.7990505	1737.175293	25217.13845	-182.2828781
4.077112211	122.9599421	668.0306091	15119.14736	-169.6439148
4.112218636	107.4299726	788.8517761	11541.19901	-123.4240341
3.87784967	96.30974699	1224.94989	9275.567366	-105.0434151
4.269838305	120.9655331	751.2608032	14632.6602	-148.6048981
4.422124248	136.1386929	807.9121704	18533.74371	-194.7096298
3.994573626	105.8912631	633.1580048	11212.9596	-132.3262909
4.225112643	153.6226948	736.2432251	23599.93236	-246.7217346
4.27159547	124.4672406	1281.461212	15492.09397	-151.0339264
4.708177423	167.7559994	1139.343445	28142.07533	-209.2298279
4.289269638	127.4223689	948.6576843	16236.46009	-164.123793
4.191816793	113.5229248	1033.064941	12887.45445	-141.179068
4.034590013	103.601637	1096.83017	10733.29919	-116.140789
4.842830727	199.0941066	1110.77417	39638.46329	-240.5372284
4.259087456	124.3282745	1015.121765	15457.51983	-143.0906525
4.262294843	135.9166423	643.5803223	18473.33365	-165.9848083
4.042297651	104.772292	720.1796875	10977.23318	-120.6728516
4.349800599	138.8302585	971.1790771	19273.84068	-158.5162048

log.sigma.1.5.mm.3D_firstorder_Kurtosis	log.sigma.1.5.mm.3D_firstorder_Mean	log.sigma.1.5.mm.3D_glrlm_ShortRunLowGrayLevelEmphasis	log.sigma.1.5.mm.3D_glrlm_GrayLevelVariance
2.974423346	-1.265630379	0.009032359	33.0656584
4.123061599	-1.353166304	0.003503496	20.13457387
4.222416774	-1.283096515	0.013469987	34.16431699
3.424049905	11.30670724	0.002777446	41.92170551
3.673156896	5.04469728	0.011325675	21.34389239
2.724718282	-11.10893934	0.00659274	20.00942884
5.45446507	-3.100130399	0.006391548	16.29949703
2.487127587	-1.323495401	0.005742289	35.19669852
2.915736554	-7.812004787	0.004359476	29.72274784
4.209620634	-6.069474801	0.003425815	24.3146037
2.471694458	-8.080278819	0.008843358	19.0325173
4.221092083	-3.557031087	0.002247524	34.22221262
3.466971099	6.038181747	0.008971364	18.01650346
2.712320194	4.154754752	0.011408328	17.69323098
3.570659978	7.028749482	0.007258069	23.51198563
8.709640056	1.636545871	0.00398274	12.14480874
2.475089015	-4.094118262	0.014459343	21.45358838
3.275878453	1.893151165	0.005601979	18.61750252
2.739492523	9.782350034	0.00720463	27.03937274
3.963960114	0.941497527	0.006341319	12.59491876
4.062430557	-7.744325218	0.003229549	19.08498826
3.452798647	8.123324787	0.003576834	24.36179302
3.088816231	-3.484352063	0.005472965	16.67442765
3.267949973	8.102360704	0.009014808	32.52571978
4.748545932	-15.81341845	0.010132479	19.76738561
3.419399108	-10.31181374	0.005706718	29.39013797
5.103278805	-1.844007543	0.002447761	16.52843826
4.366713821	-12.02753276	0.00464051	24.70612917
2.675786771	10.83417957	0.006561974	31.7889225
4.845102916	-11.59685207	0.005220515	19.40674366
4.682647213	-10.35364433	0.005988979	19.76326317
3.915774482	4.744779383	0.008257937	12.45174242
3.347891108	4.17740907	0.004520659	33.75913913
3.697979881	11.85626239	0.019624975	34.62457243
2.539990875	7.202567716	0.009766795	39.43401731
2.636426407	-2.639210911	0.005427332	20.25328976
3.406843038	-2.671404968	0.005445217	31.13016775
3.46337106	2.15225143	0.001834958	24.80829215
2.746137217	8.623500607	0.011167894	18.24486846
3.207999768	-3.401285843	0.004065317	29.60302815
3.639923289	-1.873534397	0.004735975	24.29503342
2.621755686	-5.28173642	0.006481817	24.31160719
2.608627082	-5.248841034	0.004723191	25.7359348
2.998744506	1.31084406	0.008332332	30.17139393
3.01820984	-5.868089691	0.003284803	27.00040111
3.61206785	-2.492548223	0.0080851	17.4679897
3.849486089	6.010470718	0.001563606	35.92669641
2.71179697	-7.057975504	0.006409748	26.08056523
4.393626446	3.904378075	0.03071616	22.53740779
3.284197882	5.274361082	0.004686978	27.92842425
3.549790433	-0.737766761	0.00382688	21.37917856
2.296216494	-11.44131765	0.009110562	26.02094971
3.666355562	-0.945165861	0.001943572	24.03285349
2.923682283	5.245170464	0.015197891	18.05258756
4.975159798	2.187677018	0.004378509	15.83150073
2.683633662	-6.436372158	0.005055841	43.55254779
2.763269572	-0.295108709	0.002820205	39.85701551
3.535964944	5.755322497	0.003564706	32.83028857
3.945089906	-5.550305461	0.010386	25.29496462
5.583583157	-2.112411357	0.003097844	16.93322375
2.963079007	-5.342424075	0.003104262	26.04074596
3.922719151	3.578886209	0.001871285	28.44393166
4.964145986	6.822790454	0.002587305	14.72866125
19.03303413	-1.74723278	0.00082339	15.57572783
2.527841709	-3.530845197	0.024766033	20.37634933
3.537788398	14.42409363	0.004465622	29.99968104
2.473700244	-7.999446451	0.009968913	34.43667708
3.284858965	-0.10390898	0.002632267	25.57981783
2.729943351	-12.16375929	0.006582573	25.47343341
3.490536298	0.768347948	0.003658036	31.19146728
3.10365223	-2.425706409	0.002794405	23.08514923
2.897769586	-7.792803595	0.005308259	21.54186948
4.231120377	-1.299956708	0.003137708	18.63583777
4.554252894	-6.819921743	0.004042737	15.62717678
5.032590797	5.718243086	0.002112218	22.578766
2.836287603	-7.068272135	0.004577496	40.27158636
2.877514552	-8.222968904	0.006425379	24.43535325
2.299071578	-14.51793029	0.014951945	27.39520595
2.619704027	-13.97766392	0.006067384	25.27578464
3.492571385	-5.833814196	0.005073396	27.30686412
2.847601643	-19.9923275	0.005453133	38.6545802
2.689908263	3.845381729	0.003451616	51.64688733
4.815943826	12.15917739	0.000841008	41.65503983
3.552418627	17.71002935	0.013342401	24.65731362
3.061613612	2.374873041	0.005953165	19.18719312
6.805394089	0.069274488	0.001738565	16.01193657
2.749750238	-6.014569494	0.005121052	24.02324667
2.470102675	-4.049054127	0.004032476	30.27706982
3.458621803	11.59195138	0.009292483	18.33161726
3.879986413	-21.30822763	0.02612505	39.22151467
5.884959619	0.205677899	0.001130298	26.34371646
3.369975979	-13.40967905	0.003717165	46.31690592
3.705838403	-3.677801839	0.004354944	27.85923463
4.294567433	-2.937331628	0.003817723	21.86204689
4.335506396	-0.301309997	0.002891199	18.25779037
2.701752795	-12.04441845	0.006962478	64.11132949
5.22595941	7.008726986	0.004459047	25.83475878
2.837462755	12.87049355	0.015580633	29.90263076
3.690829941	14.17775792	0.009651224	18.33997286
4.584569044	-7.73245068	0.005197713	32.69042585

log.sigma.1.5.mm.3D_glrlm_LowGrayLevelRunEmphasis	log.sigma.1.5.mm.3D_glrlm_GrayLevelNonUniformityNormalized	log.sigma.1.5.mm.3D_glrlm_RunVariance	log.sigma.1.5.mm.3D_glrlm_GrayLevelNonUniformity
0.009248793	0.05052686	0.069714551	33.69924215
0.003743067	0.077598411	0.195293912	142.2374406
0.013823657	0.056597164	0.06545991	23.25273083
0.002855557	0.045291152	0.063384626	113.2399428
0.011590772	0.06922539	0.068020557	23.37289831
0.006817643	0.062692543	0.073360295	37.77531373
0.006532953	0.090117208	0.082002771	28.34567986
0.005890556	0.048491821	0.05625801	46.25129122
0.004499402	0.052093426	0.064734468	102.1878931
0.003571607	0.065582933	0.094414613	188.4713229
0.009146804	0.064855854	0.072075334	50.42975877
0.002314824	0.055304582	0.078372915	76.79127722
0.009303158	0.067666682	0.073983541	131.2986088
0.01191041	0.067223564	0.090702869	69.84293552
0.007380078	0.06058539	0.058535859	52.37848009
0.004449388	0.143894746	0.318772898	833.5503081
0.014952542	0.06019717	0.071755903	39.44936866
0.005899836	0.067616198	0.102822997	145.1522234
0.007401395	0.056691579	0.065497741	31.17287404
0.006813167	0.093283843	0.207758845	228.8673996
0.003441907	0.079928038	0.15386371	375.7435015
0.003706533	0.059382489	0.068998176	96.28987892
0.005789856	0.076852693	0.113121834	192.2076313
0.009133333	0.05432675	0.058613925	24.83781073
0.010600904	0.072198299	0.099753526	80.11832682
0.005874364	0.055914901	0.093522667	36.02911643
0.002615592	0.086104001	0.176403718	264.9283046
0.004858113	0.068525429	0.097437828	90.9944159
0.006722772	0.049711293	0.059964172	83.36577544
0.005579254	0.083850686	0.199063226	187.7257865
0.006291002	0.075091246	0.098541526	220.7195232
0.008784245	0.088707326	0.137431556	475.1728276
0.004659303	0.049561074	0.074430474	94.80644496
0.019803077	0.072340957	0.04137575	7.224948379
0.009935276	0.047035232	0.045531129	14.96518445
0.00564205	0.061926398	0.073203028	117.1593304
0.005688687	0.058591408	0.111456389	155.6781236
0.001913291	0.06007865	0.089742467	187.5849102
0.011499241	0.067639526	0.070278112	52.94326792
0.004187227	0.053144993	0.070834061	52.48723281
0.004937169	0.064479031	0.127002751	86.72547842
0.00671558	0.057483132	0.071322174	51.40927716
0.004875929	0.055088303	0.065890665	85.33671793
0.008543263	0.053003227	0.062886069	51.35333711
0.003386886	0.0550202	0.06195815	97.74472013
0.008472909	0.074136376	0.100034634	152.670036
0.001615014	0.049764204	0.068018143	200.2435684
0.006590894	0.055847225	0.061654243	30.77232949
0.031916683	0.092688859	0.063949358	11.78447887
0.004787473	0.058725159	0.056197575	27.64885536
0.00401657	0.065494558	0.106527636	206.8657008
0.009374076	0.055831679	0.060934749	43.69641185
0.002037742	0.061607029	0.11154783	216.7034445
0.015592619	0.067206648	0.071330249	37.36470404
0.004703204	0.103726625	0.179475677	600.4363026
0.005160818	0.042474581	0.049111066	35.73878265
0.002904466	0.044275723	0.056488777	92.38132821
0.003646038	0.056674956	0.052656062	69.86565262
0.010629925	0.064778484	0.095517485	48.803015
0.003185516	0.074276679	0.085431436	60.91745329
0.003214997	0.055987696	0.068245646	111.8166462
0.001959544	0.058278639	0.105915463	295.3568742
0.002748346	0.089589765	0.140481742	250.2713189
0.000866992	0.098517613	0.249560061	230.5751056
0.025346983	0.06610814	0.062388089	13.56941326
0.004618571	0.056297262	0.072659315	82.09117311
0.010112153	0.051457516	0.044821575	19.44695087
0.002738715	0.0567301	0.079013653	169.6785156
0.006716645	0.056578084	0.056446977	23.98944742
0.003782873	0.054183388	0.071239577	102.7146347
0.002913586	0.062093662	0.078113302	176.6533003
0.005507028	0.060560242	0.071681259	64.10771079
0.003344469	0.077886854	0.158647355	362.5318005
0.004307746	0.087025811	0.143290788	527.1150477
0.002230432	0.073216246	0.115191497	614.5495844
0.004670179	0.044942268	0.047137653	48.03459393
0.006635813	0.05707899	0.064571917	60.18937626
0.015332724	0.055361503	0.060784538	31.97504854
0.006276261	0.055811808	0.074609829	92.44551586
0.005274265	0.05732196	0.08905099	152.1140411
0.005550069	0.046749618	0.050406028	22.51636934
0.00350237	0.039151533	0.037287179	50.69313333
0.000859432	0.050554456	0.061608183	133.7548396
0.013569289	0.074385019	0.060006785	10.198557
0.006207412	0.067543831	0.088549899	213.2819543
0.001859306	0.084058244	0.14623301	413.1022717
0.005273298	0.058488769	0.062322118	43.04543263
0.004142299	0.052240504	0.055251741	77.07374674
0.009459945	0.070354308	0.060134188	12.166575
0.026200823	0.068183205	0.040241857	6.884409639
0.001178443	0.063468098	0.115455329	231.560036
0.003795837	0.044841764	0.054181096	30.1000151
0.004528067	0.06189194	0.129398472	57.6261166
0.003996912	0.065251583	0.110605432	245.2179867
0.003055037	0.074435633	0.114810812	395.7324152
0.007025921	0.039637663	0.040222636	9.663428917
0.004553952	0.063406357	0.079409854	71.91985493
0.015814053	0.062393224	0.039363375	7.384064491
0.009894122	0.07157241	0.071831591	25.38242545
0.005415377	0.06150624	0.102601817	45.62553394

log.sigma.1.5.mm.3D_glrlm_LongRunEmphasis	log.sigma.1.5.mm.3D_glrlm_ShortRunHighGrayLevelEmphasis	log.sigma.1.5.mm.3D_glrlm_RunLengthNonUniformity	log.sigma.1.5.mm.3D_glrlm_ShortRunEmphasis
1.190852129	296.1000618	600.6938865	0.959899861
1.48288626	368.094763	1480.486542	0.917291612
1.182840098	211.2351489	371.0161738	0.960849215
1.174569468	595.7317523	2268.254576	0.962793966
1.199904753	227.1389613	300.1467606	0.954895403
1.212913873	266.4528281	531.9785235	0.952417941
1.233623008	369.8268929	276.2522444	0.949781749
1.160336258	364.1653441	867.8169784	0.964070704
1.188239235	382.1736575	1753.637245	0.95742817
1.257563492	415.0440585	2503.850037	0.94715986
1.2044204	212.1400567	692.430353	0.955510812
1.218533468	750.5579834	1229.588724	0.953493792
1.217154724	198.5815047	1706.363409	0.951008498
1.250038265	171.2027748	908.3936519	0.948171858
1.169282862	362.8375316	782.0038967	0.961816205
1.768622131	256.9369855	4260.795278	0.880987791
1.207632138	165.8509353	581.2968289	0.953985085
1.285007455	248.8036624	1842.939269	0.941177408
1.186653236	300.1164971	493.2466748	0.958549521
1.517394072	207.0032425	1955.949872	0.911844424
1.390462131	364.2131191	3911.963506	0.929203829
1.195519896	402.7335993	1450.526659	0.95725025
1.308240793	240.0922679	2127.981719	0.937728454
1.166806473	372.2396629	415.2363144	0.963054317
1.278011708	177.4240939	953.2735619	0.94186164
1.254501736	376.5180053	562.7528303	0.94805635
1.447361204	482.0979567	2499.615891	0.920011291
1.265024253	371.0411071	1154.455467	0.946162071
1.170006179	341.2587356	1524.125875	0.962861279
1.493138652	254.8096176	1800.337193	0.915740049
1.269869531	237.6387915	2546.710216	0.944842121
1.36601572	163.4192722	4459.739507	0.929282878
1.204758315	405.0293829	1708.460075	0.956729386
1.127515996	310.4777238	92.09933234	0.968984335
1.135469947	344.9049955	292.9023761	0.968326127
1.214171658	275.3321942	1667.97493	0.951902342
1.288135775	318.9108729	2304.83812	0.945168343
1.244313418	699.5699144	2737.112896	0.949628012
1.210191267	198.9766206	690.430519	0.951810285
1.199573817	426.8223763	881.5471537	0.956629634
1.329897809	343.1490486	1145.871355	0.938068097
1.207464996	278.8049787	791.6801628	0.95352816
1.190529719	344.2303289	1386.111455	0.957443499
1.18303611	293.1575805	869.6497101	0.958704244
1.179928813	459.90059	1595.635903	0.959187399
1.272996647	188.6301803	1783.56696	0.944518466
1.192416191	884.9645253	3602.734599	0.957897571
1.181842589	310.9462394	494.1699	0.95842178
1.178992032	229.8218746	115.1644742	0.961647038
1.171786879	477.27764	422.8120119	0.958989453
1.283018271	345.2031986	2728.921008	0.943811578
1.178314504	242.8842144	703.914665	0.959475658
1.292815316	637.160579	3034.851321	0.943184896
1.202272931	173.5501355	495.4787601	0.955956582
1.455535949	270.2965652	4702.342532	0.919391404
1.141029464	440.3112867	774.3403312	0.96826579
1.162316427	574.8305449	1897.574246	0.963614871
1.153425065	528.8823795	1123.940072	0.964827173
1.264388181	276.3277673	652.3894321	0.944877936
1.241543237	633.3065742	716.9939463	0.948377522
1.194358825	461.4067459	1784.771799	0.957167729
1.275984976	652.4003895	4410.72108	0.946525749
1.370402637	458.9764373	2331.329106	0.929730878
1.616250117	2123.118784	1800.841689	0.898489973
1.182040331	144.5102338	184.6124484	0.959173727
1.208842995	377.8030787	1293.657038	0.954059796
1.132136922	319.7127464	348.5646291	0.969248997
1.222108697	508.2574199	2639.073982	0.952198514
1.163378449	338.1783097	385.0403219	0.96319802
1.19757383	436.8067224	1698.771836	0.957970404
1.221309839	461.4084399	2511.834265	0.952201465
1.209783738	303.5873851	935.4571689	0.952805887
1.396380963	373.0734593	3881.550683	0.929629875
1.374498177	292.877156	5035.450476	0.928992345
1.312351436	551.0231293	7123.993973	0.937174417
1.137977208	473.8622759	983.0970831	0.968163806
1.187154613	275.301352	943.8716721	0.957829797
1.177018528	188.108586	520.2089488	0.960064102
1.209891248	300.9124991	1472.358042	0.954817233
1.240382718	312.4675965	2337.213083	0.951203118
1.146470089	454.084188	441.5613949	0.966707884
1.110161021	640.3144295	1208.919022	0.974018874
1.176050184	2165.078336	2388.127263	0.96092828
1.181245835	277.8266846	122.988767	0.957841115
1.243000535	251.5174565	2768.859088	0.949524791
1.389336382	620.3467613	4046.143753	0.925240536
1.180659374	345.0695907	661.3527834	0.95925829
1.162372944	427.7722194	1337.043238	0.962599235
1.185170667	328.8633419	155.1840369	0.956805975
1.124000855	407.0253887	93.32313677	0.969854487
1.303903294	1212.417854	3128.437161	0.940862033
1.161285546	601.55836	608.292836	0.962458578
1.336313022	417.0073223	787.8712253	0.935852061
1.296353949	376.5752306	3225.684	0.941244861
1.308433672	415.5100945	4531.518231	0.938704948
1.117859649	625.7799431	226.9012689	0.972649965
1.229177984	559.6264916	992.7366262	0.949270796
1.121277853	280.7171841	109.509544	0.97041105
1.215282902	233.011304	311.6588482	0.950552799
1.287399348	346.1208503	634.4117233	0.94009399

log.sigma.1.5.mm.3D_glrlm_LongRunHighGrayLevelEmphasis	log.sigma.1.5.mm.3D_glrlm_RunPercentage	log.sigma.1.5.mm.3D_glrlm_LongRunLowGrayLevelEmphasis	log.sigma.1.5.mm.3D_glrlm_RunEntropy
354.0940315	0.944650251	0.010257359	4.778423468
578.7023622	0.882078442	0.00512238	4.631898052
245.8909204	0.946472882	0.015468352	4.693349416
715.747203	0.948824989	0.003219483	5.001127441
283.9281185	0.940432826	0.012747926	4.441832135
333.9990748	0.937073813	0.007832646	4.483131054
484.1907903	0.932892034	0.007176279	4.159605108
430.8770299	0.9518655	0.006546282	4.808752902
469.4285587	0.943537944	0.005115345	4.77898426
537.8677956	0.927521478	0.004279046	4.648577849
262.2582025	0.94019161	0.010559576	4.432800469
945.9619042	0.936831724	0.002630802	4.837659676
248.6377399	0.935575996	0.010784797	4.452064548
218.2747317	0.929338104	0.014282368	4.447792955
438.1012079	0.948999409	0.00792409	4.549966069
492.8492042	0.832534243	0.007442004	4.204388287
206.5568292	0.938836235	0.017187222	4.522714071
329.9833938	0.920471038	0.007348501	4.532066246
367.5953071	0.944752842	0.008283251	4.639604008
332.1522387	0.875429105	0.00955511	4.358950166
527.8886202	0.899649546	0.004599069	4.532567134
493.3767525	0.942710197	0.004299527	4.617886271
323.4829993	0.915612765	0.007349336	4.425712598
449.838936	0.950423797	0.009672004	4.731788657
230.0174115	0.921607462	0.012784114	4.480725402
485.6948254	0.928397251	0.006689618	4.766834664
742.8410965	0.887456168	0.003549832	4.481977726
479.9562302	0.926297708	0.00588164	4.598286925
410.7885075	0.950141643	0.007461762	4.784202814
404.3459187	0.880136779	0.007633798	4.572472391
306.4491137	0.924480364	0.007757261	4.477847925
231.1323471	0.903074329	0.011500814	4.292938687
500.7575197	0.940898256	0.005309599	4.883176414
366.5456023	0.960059172	0.020523315	4.286141125
398.3888828	0.958294717	0.010651382	4.79501536
348.4997932	0.936557502	0.006596877	4.535579792
419.563426	0.922382479	0.006925354	4.811990396
900.4915868	0.930851796	0.002293377	4.681450674
252.9953457	0.937356057	0.012946433	4.414071516
528.0760628	0.941482731	0.004745801	4.752690055
476.4331736	0.912326479	0.005995568	4.696402626
345.4739546	0.938413108	0.007744558	4.627475233
428.2755628	0.943408601	0.005557496	4.662796624
358.0366762	0.94521576	0.009475248	4.75863037
564.7555325	0.945932662	0.003834041	4.695901638
245.0021494	0.924112692	0.010361323	4.43057486
1090.957201	0.94320026	0.00185006	4.915555904
376.7576046	0.945111492	0.007378897	4.626417512
279.3270053	0.948335247	0.036814318	4.041152489
582.8770135	0.947221792	0.005203216	4.61412345
458.4609062	0.922380482	0.004960148	4.618780198
295.2653477	0.946330574	0.010544031	4.594373656
862.4227508	0.92050007	0.00252039	4.70010786
212.213299	0.940648184	0.017389877	4.396776131
414.5048852	0.886578851	0.006525233	4.365488467
512.7597692	0.957206616	0.005623066	4.968140218
681.013787	0.95141885	0.003282298	4.948756675
634.0155053	0.95335832	0.00399225	4.721178952
361.6054274	0.925344925	0.011779209	4.65190028
814.164583	0.930760499	0.003597337	4.350486184
566.3680002	0.942498276	0.003719053	4.683489258
866.5289926	0.924972618	0.002414114	4.794390778
660.4513095	0.903286034	0.003595167	4.353022081
3803.189453	0.856462834	0.00113194	4.425476816
174.0281183	0.945763914	0.027879988	4.348543393
470.6502145	0.938729011	0.005308664	4.775794714
371.822287	0.959195627	0.010732164	4.682263905
644.965168	0.935540531	0.003230575	4.708573552
405.126521	0.950672646	0.007307955	4.571547536
535.8112895	0.942585013	0.004361144	4.784641189
581.1382104	0.935804656	0.003465919	4.611128851
380.3535663	0.937657559	0.006393678	4.564568201
544.6045031	0.900020838	0.004493777	4.542730642
422.9756885	0.901704311	0.005691191	4.410612899
761.261881	0.914236635	0.002816118	4.631821462
556.7142792	0.957678522	0.005064643	4.905928051
335.8256365	0.94401212	0.007566861	4.620140038
223.7957759	0.946784363	0.017011963	4.605097061
372.6833522	0.938993546	0.007240443	4.665604335
397.0582928	0.932320666	0.006248517	4.734441891
530.7852895	0.955586081	0.005986173	4.845715068
731.4247021	0.965525153	0.00371716	5.053351336
2636.653448	0.947514878	0.000942403	4.95213395
335.7986177	0.94535809	0.014553331	4.323529637
321.0724605	0.931152599	0.007440526	4.50363094
917.0788581	0.897342249	0.002487108	4.438656217
416.8204033	0.945916551	0.005947824	4.569514612
514.2499204	0.950584853	0.004623534	4.707511724
401.8730961	0.944934847	0.010164841	4.240907935
479.623577	0.961172161	0.026510981	4.39825924
1670.505554	0.917735849	0.001426433	4.732331733
711.9885597	0.950751798	0.004134609	4.979765781
579.7249712	0.910733103	0.005419969	4.765942866
508.515177	0.918769983	0.004890515	4.644249244
563.8879221	0.915604547	0.003876963	4.509961188
708.729528	0.963514746	0.007299009	5.020098459
725.9900456	0.932755567	0.004982697	4.635430725
319.5961989	0.961851157	0.016864616	4.430915767
297.5694908	0.935660645	0.010949354	4.376275707
456.7257141	0.919073492	0.006442682	4.786637112

log.sigma.1.5.mm.3D_glrlm_HighGrayLevelRunEmphasis	log.sigma.1.5.mm.3D_glrlm_RunLengthNonUniformityNormalized	log.sigma.1.5.mm.3D_glszm_GrayLevelVariance
306.2021959	0.900441282	43.36319091
399.0415154	0.807312521	36.01756474
217.4776036	0.902755996	44.09033358
616.9693273	0.907062323	54.15482293
237.5220881	0.888460281	32.22530408
278.9176354	0.882518596	27.87329432
389.8893093	0.87753425	26.1331194
376.4432054	0.909690894	43.409087
398.2930699	0.893854265	40.74767152
436.0628598	0.871033958	39.60599867
221.1947559	0.890031053	24.55354464
784.9091883	0.885295741	54.66622078
207.8977989	0.879066456	26.19392466
179.4094905	0.873637316	24.45981721
376.8138261	0.904352277	32.63751111
288.7399331	0.735218996	30.22924991
173.303701	0.886447178	29.40229939
262.8375747	0.857769327	28.85521012
312.4423638	0.896703392	32.37641582
225.3472334	0.796291382	23.99642134
389.3552374	0.832003935	34.09909541
419.0583067	0.894020219	33.11562891
254.2083375	0.850143588	25.54948387
386.3871328	0.907748743	45.15788083
186.4731456	0.858777815	31.60919224
395.2032635	0.873130811	45.25748034
521.7725778	0.812285866	31.85070929
389.4775373	0.868971846	38.09729658
353.6736385	0.907794114	39.69089623
276.6463534	0.803906934	37.20899873
249.3638878	0.866002495	31.98639106
174.5209924	0.831933475	21.52174403
421.8586141	0.892828372	47.2562105
321.3867152	0.921722142	42.88954582
355.1498969	0.920115528	47.94885009
288.8049955	0.881258546	28.0029638
335.1458237	0.867156079	46.37601193
734.1334811	0.876487735	36.99497587
209.0475155	0.881292621	23.32580912
444.9608846	0.892357968	41.97318368
364.3279964	0.851561388	40.47652202
290.9359704	0.884968322	32.75359521
359.5608478	0.894312742	32.39316195
305.133799	0.897184321	39.70320193
479.2938422	0.89801153	35.60767033
198.2232806	0.865435264	28.16986021
922.1118001	0.895228652	50.95724458
323.2133877	0.89649617	32.97264451
238.3484137	0.905015836	26.82067276
497.5106953	0.89758549	37.37970274
364.040565	0.863621996	32.63844179
252.6949186	0.899043147	31.6351647
673.851712	0.862444623	35.96627178
180.525194	0.890920267	24.60330579
292.1171548	0.811825216	30.82410965
453.6601687	0.920051852	53.80381365
594.3971484	0.908937625	49.13261809
548.2704662	0.911496562	42.44579204
291.1129363	0.865710691	41.53380036
665.2994008	0.873682348	26.99994015
480.5027472	0.893481557	36.45805849
687.3796904	0.86999388	43.2866116
491.2399028	0.833676233	25.90183015
2361.254628	0.769019878	32.86834467
149.990798	0.898766057	26.10786757
394.6265473	0.886713849	43.64318027
329.54215	0.922102896	41.80131557
532.5056924	0.882116713	36.37573275
350.4564984	0.907867185	34.19886052
454.215302	0.895832112	44.14988397
482.8034927	0.882376072	33.72355118
317.7757789	0.883328091	28.83223787
398.9483388	0.833438288	33.25848138
313.6574557	0.831079786	28.36187315
586.3126981	0.84854705	40.9250888
489.3253117	0.919568782	48.7397227
286.4730601	0.89490415	32.87519653
194.6384917	0.90040417	34.62234067
313.6869019	0.888413913	34.05393982
326.7909848	0.880471215	40.47463474
468.2514978	0.91635064	50.61667438
657.7496181	0.93359108	60.25168965
2250.852402	0.902440821	58.25368298
288.8259867	0.895993807	34.984375
263.6635501	0.876417037	27.78842225
668.2826575	0.823006238	31.70995339
358.3164618	0.898339374	30.78081208
444.0945678	0.906062278	36.93714596
342.5839249	0.894847219	21.63279773
421.2140862	0.923947054	49.35353085
1287.387555	0.857156661	43.62225765
622.4851796	0.90583272	62.76759679
443.1152164	0.846055847	43.02228261
398.5039434	0.857967024	34.97557741
440.2250761	0.852130841	30.90038982
641.4557735	0.930541711	76.78821503
589.9402253	0.875078842	41.09605587
288.4783768	0.925049443	32.3675554
245.117749	0.878260023	30.22222222
365.180904	0.855006064	54.05898792

log.sigma.1.5.mm.3D_glszm_SmallAreaHighGrayLevelEmphasis	log.sigma.1.5.mm.3D_glszm_GrayLevelNonUniformityNormalized	log.sigma.1.5.mm.3D_glszm_SizeZoneNonUniformityNormalized
270.683004	0.044474267	0.508504728
309.038123	0.047306015	0.427765674
207.1742147	0.044233423	0.495049103
469.4727617	0.038514282	0.477055053
171.5299238	0.054076266	0.402449047
205.2521587	0.055428847	0.421991685
251.5071102	0.066093887	0.4133844
289.2553847	0.042494024	0.456810761
283.543713	0.043409612	0.416154211
352.2212542	0.04648493	0.478742865
163.3338255	0.057055763	0.392623448
612.8359665	0.039732101	0.490992313
155.1073397	0.05489053	0.414290562
135.375882	0.058004384	0.415823765
275.6563576	0.05091358	0.442024691
247.9728479	0.055335983	0.442956677
126.9145983	0.051986628	0.386970145
191.5169122	0.051430403	0.375463419
199.7425243	0.051760204	0.415714286
167.6536932	0.059638961	0.394945447
329.2484249	0.048091449	0.473270073
310.8447311	0.049139561	0.401406826
205.2754518	0.055039621	0.434311019
306.7323905	0.042644706	0.567769117
169.6336652	0.052622715	0.485597784
315.4515939	0.044820528	0.471907473
404.8906241	0.050730382	0.447715605
339.4660935	0.045864234	0.513649283
255.2645126	0.044202357	0.433754104
228.5291571	0.046655629	0.466169383
226.7765332	0.050744377	0.488995854
128.9521231	0.060512684	0.365874086
324.467437	0.04109697	0.444650391
216.3343454	0.057242677	0.473797366
293.3038254	0.043831035	0.531975549
187.8864592	0.052655119	0.365550472
282.0556826	0.041034264	0.460804789
558.3285945	0.046003714	0.465488102
132.1126458	0.058893293	0.391701258
328.499102	0.044369205	0.447575358
287.4748685	0.044949524	0.479732916
210.0983298	0.049800748	0.390708151
232.9648341	0.04878223	0.393357882
217.3402573	0.045567169	0.420179032
332.4528059	0.047450315	0.4191628
167.4506517	0.053940484	0.447008997
683.7587522	0.039725208	0.468019041
257.802933	0.049189992	0.466857123
183.4405058	0.070617064	0.447092894
323.457167	0.04711052	0.38800091
275.1723691	0.048458579	0.42019914
184.7198034	0.051571854	0.418185723
481.9954276	0.046493261	0.421203156
137.6973932	0.057219927	0.414543159
233.6190158	0.051962168	0.442005178
358.279259	0.038636474	0.517273915
453.1458468	0.039960232	0.451393138
389.9053369	0.044714403	0.475129767
230.2658229	0.045196281	0.470299587
490.9028287	0.060582743	0.434561062
356.5613556	0.046346739	0.424168581
501.6551694	0.042882217	0.437013661
366.4413497	0.057170675	0.424141608
1513.236666	0.055673427	0.3765495
121.946931	0.05749377	0.477750089
294.0706049	0.042364184	0.432730437
233.0953001	0.045857461	0.492550739
382.6054057	0.046771295	0.413171853
263.0998389	0.049768972	0.459981204
365.0470329	0.042292086	0.474493231
375.4531344	0.04770139	0.467882576
231.144926	0.05432222	0.42042926
319.7445174	0.049225768	0.431459009
245.0860307	0.053776893	0.420978938
446.8179672	0.045173178	0.4689038
359.5236372	0.040573426	0.485789537
216.7646128	0.048641719	0.436425676
160.4514601	0.048644083	0.481122169
230.7635631	0.048065186	0.412685818
259.2252985	0.044098657	0.435921424
360.3228859	0.041560331	0.458221488
485.8279135	0.035950065	0.519448396
1659.778716	0.038181944	0.469391243
233.1314517	0.05632716	0.488040123
199.5757758	0.052307864	0.409824014
498.0439802	0.053660583	0.432285847
266.4398143	0.050731875	0.416825318
293.3657907	0.046887165	0.403245725
238.7727121	0.066398866	0.40831758
310.5597056	0.051013589	0.594118957
888.9685468	0.044672498	0.433918893
503.1783916	0.036230825	0.480759679
367.4275691	0.042873346	0.523941399
301.4515738	0.048851721	0.431125352
342.4160659	0.052022115	0.42929374
523.0617187	0.035731453	0.549431181
390.8122048	0.047480524	0.436929121
237.1113749	0.052977839	0.578947368
154.5365134	0.05502337	0.383867833
332.44667	0.040987617	0.502408822

log.sigma.1.5.mm.3D_glszm_SizeZoneNonUniformity	log.sigma.1.5.mm.3D_glszm_GrayLevelNonUniformity	log.sigma.1.5.mm.3D_glszm_LargeAreaEmphasis	log.sigma.1.5.mm.3D_glszm_ZoneVariance
182.0446927	15.92178771	16.87709497	12.98804657
270.7756714	29.94470774	321.7045814	310.9383038
109.9009009	9.81981982	12.33333333	8.511484457
664.5376884	53.65039483	17.17516152	13.5970077
62.78205128	8.435897436	14.00641026	8.710511177
116.8916968	15.35379061	15.75812274	10.36969073
57.46043165	9.18705036	17.96402878	12.08602039
231.1462451	21.50197628	9.95256917	6.031229983
396.5949633	41.36935992	15.97376705	11.21468517
606.0884676	58.84992101	88.82622433	82.83803648
138.9887006	20.19774011	15.67514124	10.21750614
325.5279035	26.34238311	38.07541478	33.07887499
351.3183962	46.54716981	22.56132075	16.57960462
185.4573991	25.86995516	40.73991031	34.45623278
198.9111111	22.91111111	9.491111111	5.392735802
657.3477089	82.11859838	4265.37938	4243.465121
113.3822526	15.23208191	14.54607509	8.870947827
292.1105398	40.01285347	62.07969152	53.09510907
116.4	14.49285714	10.97142857	6.650969388
280.4112676	42.34366197	580.2183099	564.6547292
873.6565547	88.77681473	538.2957746	530.2874265
317.1113924	38.82025316	12.35443038	7.614164397
435.1796407	55.1497006	169.9151697	162.4865528
148.1877395	11.1302682	11.77394636	8.377622172
230.6589474	24.99578947	53.23368421	46.80879335
141.1003344	13.40133779	27.26421405	21.87684702
489.3531565	55.44830741	403.5901189	393.5343151
315.8943089	28.20650407	45.61788618	40.18860731
391.6799557	39.91472868	8.450719823	4.630275113
372.4693367	37.27784731	442.3429287	432.2211337
611.7338129	63.48121503	112.9808153	106.5232853
646.8653846	106.9864253	135.9536199	124.7038183
425.0857741	39.28870293	22.39853556	17.87623969
28.90163934	3.491803279	4.721311475	1.814565977
101.6073298	8.371727749	6.345549738	3.324141334
299.3858364	43.12454212	15.64835165	9.565111865
573.2411576	51.04662379	60.46141479	55.10166872
672.1648199	66.42936288	62.20498615	56.80998266
140.2290503	21.08379888	15.85195531	10.41185512
217.9691992	21.60780287	21.10677618	16.46704249
276.8058925	25.93587522	112.9774697	106.4515257
157.0646766	20.0199005	17.55472637	11.93476028
290.691475	36.05006766	10.93640054	5.99946532
197.0639659	21.37100213	12.56929638	7.792881465
374.7315436	42.42058166	12.86129754	8.448478297
379.9576471	45.84941176	134.7294118	127.8588457
987.0521574	83.78046468	31.29634898	27.20479257
138.6565657	14.60942761	9.087542088	5.234318494
29.95522388	4.731343284	8.925373134	4.925373134
92.73221757	11.25941423	9.527196653	5.202885104
577.7738182	66.63054545	80.976	74.77499451
161.4196891	19.90673575	11.52072539	6.930474644
657.0769231	72.52948718	72.64294872	66.64358933
109.4393939	15.10606061	11.67045455	6.658961777
845.9979101	99.45559039	1341.463427	1329.84865
257.6024096	19.24096386	7.572289157	4.456851502
510.9770318	45.23498233	8.785335689	5.032285176
320.7125926	30.18222222	8.567407407	4.898054321
165.5454545	15.90909091	44	38.65234375
148.619883	20.71929825	44.15497076	37.51907425
402.1118143	43.93670886	21.5221519	16.5258895
995.5171203	97.6856892	138.0807726	132.2979933
431.3520157	58.1425762	211.2330383	202.0014793
260.5722543	38.52601156	822.1719653	806.5968521
50.64150943	6.094339623	10.51886792	6.327963688
302.0458453	29.57020057	22.27936963	17.32906339
113.7792208	10.59307359	5.272727273	2.363561403
554.889799	62.81384959	33.24274013	27.57600065
103.9557522	11.24778761	8.539823009	4.645312867
447.9216102	39.92372881	20.7404661	16.20230245
604.0364059	61.58249419	35.79550736	30.25059504
194.6587473	25.1511879	26.92224622	20.97623257
723.1252983	82.50238663	425.1551313	415.6469603
872.6893391	111.4794983	586.0434153	575.5474407
1590.052787	153.1822471	482.2365084	474.9077605
307.0189873	25.64240506	5.683544304	2.565414196
224.7592233	25.05048544	11.85436893	7.150102743
142.4121622	14.39864865	15.85135135	11.60441015
316.9427083	36.9140625	20.296875	15.02124023
537.0551948	54.32954545	27.68993506	22.35353506
126.0109091	11.42909091	6.894545455	3.535656198
431.1421687	29.83855422	4.398795181	1.78842938
669.8213034	54.4856342	21.25297828	17.42488174
35.13888889	4.055555556	9.041666667	4.98591821
549.1641791	70.09253731	39.81567164	33.41175039
721.9173653	89.61317365	519.8646707	509.1125548
149.2234637	18.16201117	12.43575419	7.713023938
311.3056995	36.19689119	8.621761658	4.580203495
37.56521739	6.108695652	7.402173913	3.445534026
39.80597015	3.417910448	3.925373134	1.46936957
679.0830671	69.91246006	149.5980831	143.1468077
177.8810811	13.40540541	10.02162162	6.380745069
241.0130435	19.72173913	54.32608696	49.38996219
674.2800512	76.40409207	133.0102302	126.171537
865.8854735	104.9286068	277.1462568	268.8603076
86.81012658	5.64556962	4.803797468	2.239745233
219.7753479	23.88270378	20.79125249	14.94697027
44	4.026315789	4.381578947	1.762292244
56.42857143	8.088435374	19.72108844	13.07381184
163.7852761	13.36196319	47.75766871	41.62976589

log.sigma.1.5.mm.3D_glszm_ZonePercentage	log.sigma.1.5.mm.3D_glszm_LargeAreaLowGrayLevelEmphasis	log.sigma.1.5.mm.3D_glszm_LargeAreaHighGrayLevelEmphasis	log.sigma.1.5.mm.3D_glszm_HighGrayLevelZoneEmphasis
0.507082153	0.089714712	4104.53352	347.3687151
0.30476649	0.889847901	117802.0079	440.1990521
0.511520737	0.115900716	1839.936937	263.4504505
0.528652751	0.035309952	9335.592965	644.22972
0.43454039	0.083714813	3420.108974	245.6474359
0.430793157	0.082576527	3799.234657	297.0938628
0.412462908	0.055230139	7269.05036	380.3309353
0.50499002	0.044471333	3126.227273	404.4841897
0.458393458	0.05187058	5952.585519	413.4847849
0.408650742	0.238758118	33946.06872	478.0221169
0.428053204	0.11109318	2980.641243	239.3333333
0.447368421	0.055623593	27532.10558	828.8989442
0.408871745	0.164932465	3773.704009	226.2429245
0.398926655	0.376527223	5253.345291	200.706278
0.493962678	0.037953637	3392.748889	389.5955556
0.213617389	16.18008788	1128779.114	340.430593
0.419770774	0.155423917	2285.249147	186.5494881
0.333619211	0.299223475	14033.8946	293.9421594
0.481099656	0.053296366	3239.45	309.7178571
0.2534809	2.935553413	116379.3451	255.4647887
0.353369066	1.517667388	191895.8364	441.3472373
0.459302326	0.038438864	4629.432911	452.7949367
0.366898572	0.786156675	37439.97904	289.4011976
0.542619543	0.044496998	4549.045977	389.7509579
0.394518272	0.391354855	7948.334737	221.8168421
0.430835735	0.092343872	9187.826087	436.3444816
0.315349106	0.821422687	199615.7877	565.9130833
0.429169574	0.145400851	15568.8748	438.7479675
0.511614731	0.039218882	2770.723145	366.1273533
0.314319434	1.70579154	116081.2278	311.0100125
0.393519975	0.562403422	23499.77618	293.9552358
0.298145025	1.008485436	19220.98925	199.2794118
0.470241023	0.068825015	8344.106695	452.292887
0.586538462	0.046407488	1620.147541	307.8852459
0.575301205	0.037683093	2050.198953	376.2984293
0.405445545	0.07000236	4297.739927	297.8608059
0.431944444	0.223344549	17430.62942	377.6511254
0.43053071	0.094417862	42003.95222	778.5387812
0.428742515	0.109248508	3309.413408	207.9972067
0.464251668	0.059789973	8414.071869	467.7556468
0.391451832	0.352341303	37357.23917	390.8214905
0.421825813	0.090571199	4279.798507	314.0248756
0.450060901	0.042594216	3737.277402	360.8903924
0.457560976	0.071613575	3261.573561	320.4264392
0.476038339	0.031575919	6203.797539	486.0536913
0.381508079	0.850705192	22025.53059	229.9435294
0.494374121	0.03824202	26699.4642	950.4167852
0.509433962	0.044864041	2581.804714	351.4208754
0.5	0.192954986	2142.104478	251.3731343
0.480885312	0.025870884	4865.543933	499.539749
0.401577103	0.25749017	26293.49455	394.0254545
0.466747279	0.079949085	2517.619171	267.0906736
0.408270086	0.117221509	46025.85385	704.3544872
0.446700508	0.113915602	1822.147727	198.3636364
0.293423272	5.15509861	350571.848	326.7575758
0.566552901	0.027475969	3088.190763	474.87751
0.51618787	0.022319502	4496.847173	631.5106007
0.522041763	0.020508439	4791.127407	543.277037
0.432432432	0.187844342	11604.6875	314.3465909
0.388195233	0.082882274	25170.16959	710.4707602
0.44738084	0.057568338	8854.661392	515.7816456
0.415845199	0.220917447	87297.33187	719.6290606
0.329126214	0.482222094	93519.85447	531.7315634
0.253387038	0.352616119	1926564.777	2376.015896
0.488479263	0.157037031	1290.037736	165.1698113
0.449452672	0.071995675	7799.967049	422.0515759
0.586294416	0.033827438	1645.458874	326.4025974
0.420081326	0.072124447	16353.61653	560.9664929
0.506726457	0.036750148	2782.69469	364.119469
0.4694182	0.058347926	8344.961864	490.5497881
0.424671053	0.088392486	15321.27266	518.8830364
0.410097431	0.123862432	6883.933045	337.6198704
0.324303406	1.175760756	154851.6993	449.7983294
0.308665873	2.03797165	169625.3222	350.4544139
0.369389978	0.871276255	268332.4255	617.0524919
0.566308244	0.018977946	2689.974684	492.8686709
0.461056401	0.062506101	3003.6	305.3495146
0.485245902	0.16627778	2150.702703	218.9763514
0.43537415	0.099646161	5074.398438	339.3476563
0.432888264	0.106748361	8009.332792	359.9902597
0.545634921	0.025227821	2809.058182	498.0545455
0.618941089	0.011247178	2781.939759	656.1409639
0.511103152	0.010656248	46012.92852	2295.934828
0.496551724	0.056387415	2624.666667	303.125
0.395163669	0.197548976	8847.092537	289.3828358
0.304967129	0.835898419	324943.2892	711.1706587
0.460154242	0.050295793	3811.326816	387.547486
0.49742268	0.028415934	3494.304404	448.9430052
0.50273224	0.038600522	2393.804348	357.4782609
0.638095238	0.045346731	1794.507463	400.7761194
0.393710692	0.120506893	187845.3591	1301.083706
0.52407932	0.022801506	5710.508108	669.5243243
0.450097847	0.139433799	22248.47391	473.5847826
0.382396088	0.37289998	48649.34655	429.6227621
0.347399242	0.713691488	108803.9782	490.7764006
0.624505929	0.018430618	2708.867089	668.3860759
0.413651316	0.043181026	12353.14513	579.4652087
0.617886179	0.048049452	1156.315789	300.7236842
0.387862797	0.106576549	4702.408163	245.3333333
0.403965304	0.156486008	15747.73006	422.9110429

log.sigma.1.5.mm.3D_glszm_SmallAreaEmphasis	log.sigma.1.5.mm.3D_glszm_LowGrayLevelZoneEmphasis	log.sigma.1.5.mm.3D_glszm_ZoneEntropy	log.sigma.1.5.mm.3D_glszm_SmallAreaLowGrayLevelEmphasis
0.740940112	0.012414711	5.944465448	0.010927753
0.680115105	0.005093741	6.204439247	0.004023652
0.730903955	0.017297958	5.740481625	0.014593249
0.71799146	0.00342816	6.391684823	0.002799802
0.658618216	0.018156022	5.799749178	0.015695208
0.674520063	0.009227792	5.822417402	0.007676549
0.668333648	0.011177803	5.570756788	0.010133225
0.702579167	0.00743411	6.152252506	0.006200306
0.669383948	0.005704851	6.329370388	0.004488174
0.71941635	0.004553527	6.197598506	0.003751776
0.651121337	0.011853439	5.927934477	0.00923898
0.728227241	0.003249222	6.263481708	0.002851247
0.668806175	0.012484807	6.126579987	0.010084701
0.669957187	0.01420133	5.906137042	0.00974718
0.69080925	0.011169061	6.001622556	0.010103573
0.692206397	0.005589668	6.161597815	0.004151056
0.646132515	0.019832972	5.956686963	0.015132005
0.636691798	0.007310066	6.381918061	0.005428329
0.669002486	0.01004404	5.886428991	0.008292353
0.65321819	0.009951589	6.119367978	0.007906676
0.715355217	0.003981307	6.113490582	0.003067534
0.656871142	0.004401472	6.239604892	0.003356378
0.685193964	0.006698896	6.029739711	0.00502555
0.780170104	0.013541651	5.73934648	0.012754986
0.723567211	0.013497905	5.82042272	0.010799014
0.714049475	0.008771645	5.950606982	0.007766348
0.696006008	0.003474878	6.188857986	0.002804684
0.743925445	0.005187719	5.946176836	0.003106545
0.683453509	0.00862266	6.2410491	0.00697068
0.70982121	0.007405759	6.158116763	0.005879873
0.726794271	0.007206859	6.031988226	0.005519765
0.627921955	0.011225192	6.303984039	0.008272655
0.693083538	0.006042541	6.361049635	0.004956407
0.71377163	0.028269092	5.102387747	0.025572081
0.756652208	0.012733669	5.725941978	0.010902687
0.627289395	0.007091584	6.329671539	0.005309275
0.705933351	0.006541555	6.301400755	0.004567019
0.709481725	0.002324457	6.199969959	0.001882935
0.649368736	0.016818828	5.903153664	0.014235185
0.695142418	0.005568604	6.125986295	0.004696933
0.720089669	0.006998955	6.04705601	0.005939034
0.648804697	0.008820014	6.148962132	0.007005173
0.651036116	0.006209431	6.268426958	0.004850442
0.673653013	0.012123072	6.228657071	0.010272215
0.672796835	0.004206839	6.292778102	0.003307505
0.695266494	0.010268982	5.984261306	0.007877403
0.711451265	0.00196337	6.42850583	0.001602092
0.710408572	0.008373405	5.822261336	0.007120393
0.693830846	0.026437078	5.102964937	0.010835536
0.640087643	0.007049159	6.031231954	0.006099194
0.673898664	0.004751629	6.290264438	0.003572788
0.671973475	0.011950359	6.010597171	0.009394324
0.673612933	0.002473777	6.320361672	0.001914164
0.668596382	0.022963214	5.803588507	0.019768299
0.691409611	0.005748935	6.223873984	0.004270723
0.74673552	0.006415913	6.076018711	0.0055616
0.69823399	0.003423664	6.398977541	0.002750981
0.71679124	0.004712524	6.135988198	0.003961718
0.713148913	0.017419213	6.018205419	0.015821472
0.685533773	0.005296783	5.784043093	0.004804261
0.676346507	0.003903686	6.249932508	0.003076786
0.687389642	0.00230474	6.462961887	0.001765939
0.677344608	0.003527283	6.134047322	0.002780905
0.637035716	0.001904077	6.224879714	0.001745896
0.718343607	0.03512417	5.345660965	0.030299999
0.682263811	0.005876119	6.285961377	0.004657658
0.729718081	0.013743564	5.773184353	0.012544824
0.667269092	0.00332864	6.371372492	0.00255569
0.704991917	0.009367818	5.805186782	0.008224445
0.716264844	0.004522824	6.250727072	0.003588666
0.71125622	0.003412141	6.169292214	0.002696595
0.673691448	0.007125939	6.055231713	0.005683399
0.68325496	0.00393355	6.322139979	0.002949986
0.674726876	0.005189844	6.247643635	0.00385203
0.712044126	0.002701041	6.369317021	0.002117434
0.724713447	0.005843482	6.201763683	0.004919285
0.687106817	0.008346116	6.093609438	0.006752713
0.721089458	0.020771552	5.743949627	0.018340424
0.66623838	0.008229018	6.210996798	0.00660934
0.686292368	0.006494755	6.338149235	0.00509662
0.703742867	0.007473118	6.035967141	0.006604564
0.749053433	0.004348954	6.255660072	0.003845465
0.712286562	0.001188037	6.45186902	0.001048951
0.722665697	0.021375889	5.227226349	0.019773714
0.665399256	0.007664663	6.227650615	0.005855726
0.683901071	0.002315286	6.271350816	0.00179334
0.670043008	0.00689695	5.99645355	0.005698338
0.659185123	0.00520868	6.22950408	0.004172939
0.66102506	0.014399441	5.172685055	0.013174823
0.79371476	0.038079384	5.060356683	0.037450513
0.684739921	0.001657345	6.392241959	0.001396359
0.720720345	0.005156904	6.233215559	0.004541098
0.751598366	0.006295758	5.988503936	0.005509019
0.682617618	0.005152865	6.320459118	0.004010549
0.681124755	0.003646217	6.251480026	0.002803844
0.769234012	0.009578992	5.851001913	0.00902508
0.687088258	0.007854032	6.218049957	0.007163529
0.78508578	0.0203341	4.973008395	0.018962083
0.641018336	0.016869663	5.69126389	0.014997016
0.736185765	0.007407057	6.004402238	0.006121847

log.sigma.1.5.mm.3D_ngtdm_Coarseness	log.sigma.1.5.mm.3D_ngtdm_Complexity	log.sigma.1.5.mm.3D_ngtdm_Strength	log.sigma.1.5.mm.3D_ngtdm_Busyness	log.sigma.1.5.mm.3D_ngtdm_Contrast
0.008719803	2.607110896	2.824959214	0.219075728	0.0003703
0.003856412	0.551584086	1.210681326	0.338803797	4.96E-05
0.014139549	4.291369236	5.236045949	0.168759889	0.000616003
0.00263737	1.664172302	1.826547247	0.278268552	6.39E-05
0.016491875	2.895266722	4.977359234	0.121768409	0.000438144
0.009395383	1.430294188	1.976230435	0.2637826	0.000313858
0.016934536	2.275977944	4.117524265	0.098266576	0.000456212
0.005706	1.88556781	1.94556901	0.296777818	0.000289504
0.00323213	0.830708851	1.121330365	0.455619464	9.37E-05
0.002236744	0.829683169	1.393969745	0.399209243	2.95E-05
0.006781425	1.100941187	1.405398998	0.388217527	0.000234943
0.004674683	2.245590542	2.97599312	0.143750021	9.63E-05
0.00307394	0.623449193	1.11798204	0.653193239	5.49E-05
0.005787924	0.635410296	1.050559351	0.556486136	0.000151349
0.006934445	1.563169839	2.103651826	0.227861614	0.000195857
0.001068174	0.19584988	0.988911824	0.888976165	3.62E-06
0.008814821	1.249436919	1.782095111	0.360265047	0.000304832
0.003152742	0.442677863	0.880048529	0.610491275	4.94E-05
0.011102532	1.958201647	2.780319989	0.185682766	0.000401314
0.002799627	0.255778767	0.695984103	0.679436328	2.42E-05
0.001367312	0.315036506	0.603458813	0.802379866	1.60E-05
0.003826201	0.922897618	1.410474336	0.373887176	9.09E-05
0.002480401	0.304673312	0.565726447	0.884803379	4.65E-05
0.013268058	3.608125343	4.136800595	0.113144738	0.000524487
0.00540714	1.135061137	2.104114027	0.400189322	0.000105957
0.009802123	2.643235462	3.466660165	0.137931889	0.000259798
0.002210101	0.436636122	1.062553453	0.404787993	1.84E-05
0.004838082	1.146139355	1.900947951	0.276926508	9.91E-05
0.003683623	1.027765956	1.219294878	0.432335404	0.000127191
0.002989545	0.559691439	1.340129919	0.450739504	3.18E-05
0.00214336	0.596200298	1.142977488	0.643950485	2.77E-05
0.001280419	0.133458895	0.393080851	1.660784343	1.06E-05
0.003329913	1.476603532	1.83843406	0.310336578	7.75E-05
0.038459993	11.01010481	11.93537373	0.059485869	0.005162875
0.01648654	5.19580971	4.572114257	0.119304467	0.001234391
0.003290583	0.537409953	0.823026854	0.597010988	7.65E-05
0.002352805	0.74039127	1.009199974	0.597536783	5.91E-05
0.001998804	0.58803765	0.862964768	0.445465963	4.03E-05
0.007009842	0.93741389	1.349839476	0.384883059	0.000226847
0.006024249	2.014556666	2.544373141	0.206516794	0.000170974
0.005022742	1.126917164	1.751499147	0.257884396	8.81E-05
0.006675146	1.301284117	1.808266113	0.295845272	0.000199003
0.003762493	0.939339061	1.193245654	0.42161254	0.000115153
0.006351863	1.702570391	2.15546614	0.262108937	0.000201171
0.00329279	1.174944465	1.475605803	0.328206226	8.15E-05
0.003049497	0.506618309	0.961917767	0.701463657	4.99E-05
0.001609975	1.051932533	1.329445139	0.335372565	2.99E-05
0.009792565	2.389310907	2.736090748	0.203035781	0.000392469
0.033509862	5.626624654	8.392210703	0.072149661	0.002118785
0.011842924	2.855587094	3.545413525	0.123582763	0.000491911
0.002123333	0.474978274	0.795896217	0.599239748	3.21E-05
0.006599872	1.499212691	1.636597713	0.355248722	0.000322016
0.00187372	0.590925413	1.008206152	0.42774107	2.71E-05
0.010531623	1.326807667	1.97470883	0.304750214	0.000315136
0.001173654	0.19071464	0.571260346	1.044123155	8.82E-06
0.007126354	2.930586862	2.68986561	0.199030844	0.00036489
0.002873924	1.5958877	1.688736997	0.31257912	9.04E-05
0.004942143	1.501921209	1.898652695	0.220841494	0.000170315
0.008770505	1.716536173	2.744823749	0.188499405	0.000204639
0.007147835	1.285170311	2.679323081	0.162552032	0.000131704
0.003186208	0.868192309	1.215215151	0.384580649	7.26E-05
0.001337242	0.540830487	0.892215517	0.516192917	1.85E-05
0.002433633	0.430623156	1.108486315	0.386987224	1.86E-05
0.002790257	0.528253231	2.435431557	0.159322938	2.24E-05
0.027908022	2.343857802	3.485035281	0.186678722	0.001406197
0.004924974	1.295708994	1.877635765	0.253697384	0.000104512
0.012389449	4.183438213	3.603570213	0.153684186	0.000943661
0.002153784	0.671320752	1.063011272	0.458080873	3.81E-05
0.013204876	2.379158759	2.894455682	0.165750945	0.000606646
0.003537307	1.21702511	1.657960789	0.308191154	7.92E-05
0.002282129	0.460052114	0.726177995	0.59519022	4.91E-05
0.005696434	1.074203464	1.617412987	0.319681415	0.000141582
0.001425453	0.281669038	0.589971843	0.794282517	1.59E-05
0.001116898	0.235588145	0.616990898	1.003530276	8.39E-06
0.000846473	0.294807543	0.607630726	0.779241015	7.27E-06
0.005413469	2.758141606	2.620519499	0.197243397	0.000218728
0.005496932	1.250900699	1.62546366	0.345752449	0.000167782
0.008682926	1.898911394	1.964659859	0.375338282	0.000514518
0.003565299	0.787207701	1.02545479	0.530731523	0.00010914
0.002299046	0.933113615	1.339272502	0.51812525	4.44E-05
0.011603114	4.544209017	4.596588788	0.116034577	0.000548433
0.004395549	3.316554142	2.768629277	0.190906374	0.000209399
0.002708142	1.786414651	2.443945096	0.120912449	5.01E-05
0.04216592	5.345880081	9.065059461	0.054764875	0.001763012
0.001971736	0.395927912	0.655538576	0.871267151	3.42E-05
0.001352589	0.368834699	1.045646474	0.484801392	8.56E-06
0.007787382	1.526350916	2.063046055	0.255284831	0.000262793
0.003788166	1.002927717	1.211746617	0.409859245	0.00016354
0.032322188	2.834306476	4.246517192	0.106801203	0.001681499
0.03753191	13.34981562	11.59663975	0.046661208	0.005445612
0.001832136	0.878834299	1.752271602	0.241223296	2.09E-05
0.008823832	4.437179844	4.334860668	0.121316396	0.000407931
0.00721537	1.903694278	2.700550913	0.15961325	0.00014943
0.001774573	0.547161313	0.982891693	0.553226201	2.11E-05
0.001229639	0.374125069	0.812506968	0.695341918	1.05E-05
0.019890009	13.32758463	9.68960852	0.063604224	0.002131954
0.005593441	1.813488338	3.213124676	0.146339254	9.31E-05
0.038873289	7.988570941	8.790118166	0.068292463	0.003281531
0.016515944	2.06922521	3.87552691	0.139413866	0.000414424
0.00912736	2.356020361	3.73981476	0.153772478	0.000225378

log.sigma.1.0.mm.3D_gldm_GrayLevelVariance	log.sigma.1.0.mm.3D_gldm_HighGrayLevelEmphasis	log.sigma.1.0.mm.3D_gldm_GrayLevelNonUniformityNormalized	log.sigma.1.0.mm.3D_gldm_DependenceEntropy
12.91855524	123.1685552	0.085479379	6.104313138
7.150239608	163.1535869	0.150521949	6.267764905
13.11631167	85.48387097	0.093886046	5.899375008
16.72551934	330.7077799	0.075957642	6.41209765
7.022152218	81.25069638	0.118209821	5.723320707
7.646829476	135.6920684	0.104837598	5.864736013
6.229499247	161.1839763	0.143181678	5.507902127
13.00249401	223.2235529	0.078184947	6.164435865
10.6328208	189.2453102	0.088819434	6.262399675
9.416955003	246.7937379	0.110982699	6.170016186
6.73167244	113.7678356	0.107960037	5.842387797
13.89501367	391.6491228	0.094034396	6.23289852
6.440753825	119.246866	0.115964846	5.906044026
5.825022961	79.61896243	0.117965572	6.040128863
8.735544708	168.8649835	0.100914424	5.94870322
3.960126031	114.6988628	0.240307886	5.875701847
7.278068325	62.6504298	0.104018029	5.910773792
6.163153816	117.6865352	0.122403564	6.179682755
9.171092099	103.314433	0.094407246	5.926925024
4.03307535	94.96608354	0.173649284	6.062402681
7.016858667	160.0769525	0.135141891	6.142874702
8.241532923	194.9610465	0.103695241	6.053678985
5.397168359	114.4393995	0.138445296	6.072935993
11.81034833	171.5883576	0.088999442	6.032172273
6.848800648	74.11877076	0.124566782	6.071430502
9.899012948	162.425072	0.097779236	6.158944653
6.221902471	276.9506636	0.150379967	6.243142216
8.585766956	185.0509421	0.11941968	6.112141508
10.55589051	147.6249292	0.087719828	6.131982292
6.661940609	157.5979544	0.155096967	6.234839331
7.176039757	113.767537	0.13174051	6.013997178
3.987991435	77.92478921	0.164393756	5.979644225
12.92511194	197.5400885	0.085112854	6.272250731
13.85465976	131.0769231	0.095968935	5.213714993
14.44943932	150.6777108	0.076208448	5.836618862
7.381220224	138.7559406	0.105007352	6.02185308
11.35134537	166.6565972	0.108431231	6.27132316
9.828010254	431.7176506	0.100260496	6.204388644
6.236402883	81.47904192	0.114065044	5.758454741
11.32680541	220.1830315	0.087667132	6.167130366
9.397585742	165.2069199	0.114716598	6.334448166
8.95176771	139.5057712	0.095785221	6.073847848
9.31930884	163.8081608	0.093521759	6.105599633
10.87791172	144.2409756	0.089008923	6.144655233
10.40206936	246.8541001	0.091269801	6.159518913
7.079362867	97.15888689	0.123268729	6.077702554
14.18385564	439.3980309	0.082932262	6.394958995
9.996851913	163.4082333	0.091638741	6.002964599
7.681944754	98.5	0.130875473	5.237194276
9.780906769	194.1830986	0.096875013	5.823191224
7.914126984	190.0852804	0.113401518	6.215001558
9.259490386	136.8101572	0.092791796	6.021268591
8.629941519	388.5686993	0.11421235	6.295315225
7.141046893	81.5499154	0.110710288	5.878677854
5.561943071	139.156983	0.188067017	5.985308505
16.97788753	170.298066	0.071089937	6.250760428
14.24440656	225.4277246	0.074736201	6.296762564
12.00181715	226.4555298	0.093864338	6.081382859
9.623650007	163.7616708	0.11329679	6.166847203
6.243836008	245.246311	0.123779731	5.843284776
9.762903825	247.6705993	0.095506375	6.12243741
9.996235631	284.2499087	0.105840002	6.323211249
5.372153727	219.5634304	0.16036447	6.004496466
5.313680848	1194.187111	0.182696574	6.117679834
7.512667502	63.7281106	0.105990783	5.697032614
11.09653128	200.9079202	0.097040437	6.214396008
13.11446443	165.9568528	0.080136051	5.845921576
9.025010643	281.5899281	0.101521416	6.203311889
9.765333105	161.0112108	0.091717107	5.919818521
11.65151636	195.0377921	0.096536924	6.198974354
7.95469356	163.1210526	0.109735111	6.156541666
7.647543112	161.108946	0.104467846	5.941231775
6.318407356	162.0222523	0.141777304	6.144968179
5.45265471	135.068642	0.154237558	6.143177579
8.670553633	251.8470588	0.124845454	6.302289465
15.87325044	252.6084229	0.073293637	6.094717144
8.929071267	138.1593554	0.096051981	6.075754454
10.14963988	96.15245902	0.087890352	5.950684058
9.00270592	185.0011338	0.095729789	6.212204742
9.821235337	139.4177793	0.101438124	6.222791829
13.87394495	215.1309524	0.080892542	6.09760895
19.64708296	295.3407905	0.064544418	6.191703526
16.46740675	1089.053367	0.086476096	6.342303526
8.698216409	126.6827586	0.11705113	5.193438223
7.05868858	140.6652905	0.116215917	5.987496876
5.797836605	278.1442659	0.146365894	6.219808156
8.76076189	163.0886889	0.096460504	5.861114774
10.86746566	219.3646907	0.086282914	6.017989701
7.362059184	170.726776	0.118934576	5.462551977
14.62276644	186.8571429	0.117823129	5.051935104
9.462893778	610.3849057	0.114900708	6.346975315
17.21455312	316.1713881	0.076595591	6.221074397
10.28326044	218.721135	0.107760387	6.303512767
8.282538005	189.2738386	0.115618032	6.264332833
6.754020987	247.5275577	0.131444018	6.095194824
23.20839257	226.8063241	0.062100642	5.960280522
10.05069942	320.3725329	0.106510236	6.149395754
11.14693635	149.9268293	0.09220702	5.38192505
7.147945225	103.4881266	0.117856322	5.723999995
11.53197932	119.7744734	0.108380973	6.237313285

log.sigma.1.0.mm.3D_gldm_DependenceNonUniformity	log.sigma.1.0.mm.3D_gldm_GrayLevelNonUniformity	log.sigma.1.0.mm.3D_gldm_SmallDependenceEmphasis
128.9716714	60.34844193	0.318637268
211.3225806	312.6340876	0.198369511
80.92165899	40.74654378	0.382938207
512.1666034	200.1483871	0.347688703
56.86072423	42.43732591	0.24254948
106.4494557	67.41057543	0.288215018
51.65875371	48.25222552	0.24039091
193.4471058	78.34131737	0.331185384
348.8566619	184.6556037	0.274432413
445.2207876	343.8244028	0.265605074
141.4401451	89.28295042	0.265288587
239.5775978	139.3589744	0.299382063
330.3375121	240.5110897	0.231685545
150.5742397	131.8855098	0.21795132
163.428101	91.93304061	0.294382567
551.1350223	1669.418886	0.131687692
112.7277937	72.60458453	0.242701486
291.4571184	285.4451115	0.186152128
105.2268041	54.94501718	0.250333947
263.7583006	486.3916458	0.153684679
610.0995406	705.9812404	0.230383793
280.9	178.355814	0.266754313
321.3507873	378.0941047	0.209425978
90.71309771	42.80873181	0.327976144
155.6910299	149.9784053	0.25343733
101.2132565	67.85878963	0.277832398
354.253895	521.2169648	0.197308605
195.9406839	171.128402	0.271336019
329.3308782	154.8254958	0.283462496
244.9716758	394.256491	0.211534631
416.9276502	418.8030827	0.246517417
659.0182125	974.8549747	0.16609288
365.8086572	173.0344319	0.305227407
25.98076923	9.980769231	0.375316425
76.74698795	25.30120482	0.39354261
326.0970297	212.1148515	0.233323011
414.6125	312.2819444	0.281123793
525.4341085	336.273703	0.267380402
149.1317365	95.24431138	0.224082947
180.8417541	91.96282173	0.325854671
189.192673	169.0922659	0.253604792
158.9601259	91.28331584	0.267149657
290.9330085	153.5627284	0.270387501
178.777561	91.23414634	0.283348872
338.9840256	171.4046858	0.286632486
298.9021544	274.6427289	0.257974679
756.5930614	353.7890295	0.309746829
101.9090909	53.42538593	0.287050965
24.67164179	17.53731343	0.363273873
93.08853119	48.14688129	0.288047996
489.5408879	388.2867991	0.242076691
148.2648126	76.73881499	0.286279067
534.625229	436.4053913	0.239196484
96.24534687	65.42978003	0.263069254
656.9745516	1226.761153	0.183558726
182.2036405	62.48805461	0.37222263
425.3903329	163.8964888	0.312208477
229.2072699	121.3665893	0.293986419
112.031941	92.22358722	0.290704795
121.9148695	109.0499432	0.241264752
357.3397829	202.3780085	0.267407485
768.8419131	579.7915298	0.256975176
348.0330097	495.5262136	0.203679019
232.7846943	498.9443427	0.149772947
37.15668203	23	0.281138508
246.8531874	150.7037991	0.282652703
87.91878173	31.57360406	0.379542158
491.7475759	324.5639662	0.248694787
83.69955157	40.9058296	0.299841092
337.6494281	194.1357534	0.287192851
445.8973684	333.5947368	0.249189293
196.5146147	117.9441984	0.233097963
592.1164861	732.7051084	0.202716728
726.5908279	1035.85944	0.185785285
1146.725054	1146.081264	0.24084211
244.1487455	81.79569892	0.360028882
188.7385855	107.2900627	0.277487257
112.4262295	53.61311475	0.292438917
276.0181406	168.8673469	0.260687327
434.1672523	288.6929023	0.262076951
96.84126984	40.76984127	0.368997353
325.945563	86.55406413	0.414814971
505.2901146	241.4412607	0.324293146
29.12413793	16.97241379	0.277061185
506.4762607	394.0881746	0.251291867
573.463477	801.4996348	0.182463713
140.9640103	75.04627249	0.271003877
312.7860825	133.9110825	0.287389603
32.14754098	21.76502732	0.275299374
24.2	12.37142857	0.428059443
537.9831447	456.7303145	0.251769913
133.8470255	54.07648725	0.359515245
141.0900196	110.1311155	0.306010179
528.9515892	472.8777506	0.233800916
721.3988977	763.1639683	0.222616313
65.07114625	15.71146245	0.443223939
179.4358553	129.5164474	0.263250851
26.88617886	11.34146341	0.354649955
60.13456464	44.66754617	0.253379273
104.9306072	87.46344486	0.293936885

log.sigma.1.0.mm.3D_gldm_DependenceNonUniformityNormalized	log.sigma.1.0.mm.3D_gldm_DependenceVariance	log.sigma.1.0.mm.3D_gldm_LargeDependenceEmphasis
0.182679421	4.300628366	13.94050992
0.101744141	13.65865224	40.3721714
0.186455435	5.37773153	14.96774194
0.194370627	4.734494417	13.64364326
0.158386418	3.871074868	16.05292479
0.165551253	3.315997862	13.87091757
0.15329007	3.959407937	16.74480712
0.193060984	2.586368182	10.79041916
0.167800222	3.769116067	14.57768158
0.143712327	6.096567852	19.90961911
0.171027987	3.010493779	13.55139057
0.161658298	5.28927426	16.82726046
0.15927556	3.713408142	16.10896818
0.134681789	5.569506626	21.29874776
0.179394183	3.167352073	12.82436883
0.079334248	13.17236485	52.87850871
0.161501137	3.275202995	15.11747851
0.124981612	6.440280503	25.03516295
0.180802069	2.66953626	12.88316151
0.094165762	11.04864522	40.35380221
0.116787814	9.845313859	29.84456355
0.163313953	3.5180841	14.61627907
0.117667809	8.956606954	28.94873673
0.188592719	3.162149195	11.88981289
0.129311487	8.359488306	25.13953488
0.145840427	7.031916219	20.95965418
0.102208279	12.52485791	37.9711483
0.136734601	7.465920684	22.62316818
0.186589733	2.946824066	12.32464589
0.09636966	14.47007535	42.39103068
0.131150566	7.115716036	23.16860648
0.111132919	8.418013531	31.34806071
0.179935395	4.097254136	13.89326119
0.249815089	1.532544379	7.403846154
0.231165626	2.189432429	8.530120482
0.161434173	3.47139006	15.58316832
0.143962674	8.124442515	22.95763889
0.156658947	5.042997459	17.35778175
0.178600882	2.612751981	13.88143713
0.172394427	4.063853086	14.01429933
0.128353238	10.51311102	28.87381275
0.166799712	3.289844078	14.09758657
0.1771821	2.920586433	12.93422655
0.174417133	2.841590482	12.72878049
0.180502676	3.100021435	12.76357827
0.134157161	6.779033131	22.06193896
0.17735421	4.723263502	14.90154712
0.174801185	3.603305785	13.72727273
0.18411673	3.371129427	12.10447761
0.187300868	2.426300256	11.52313883
0.14297339	6.328161507	20.86331776
0.179280305	2.949779875	12.56952842
0.139917621	8.050452735	23.98979325
0.162851687	3.629536104	14.95600677
0.100716626	9.860800245	34.86080025
0.207285143	3.493951266	11.21843003
0.19397644	2.617193662	11.04651163
0.177267803	2.924386114	12.48027842
0.137631377	9.77144444	25.71253071
0.138382372	4.999581272	19.415437
0.168636047	3.583881736	14.4374705
0.140350842	6.275923169	20.70427163
0.112632042	8.729005771	29.66925566
0.085237896	14.35322613	49.27608934
0.171228949	4.980483765	16.14285714
0.158952471	4.571580917	16.15260786
0.223144116	2.039037337	8.558375635
0.153815319	4.099040105	16.55020332
0.187667156	2.834080717	11.83408072
0.167901257	4.165439165	14.93336648
0.146676766	5.184347299	18.65131579
0.174060775	2.930690288	14.07528787
0.114573623	8.529572315	29.00309598
0.108188033	9.800081659	32.80464562
0.124915583	8.428678381	26.14379085
0.218771277	1.842785293	8.586021505
0.16896919	3.481478157	14.03670546
0.184305294	3.271969901	12.7704918
0.156472869	4.611615016	16.80612245
0.152553497	5.950458016	19.02810963
0.192145377	3.12244898	11.28571429
0.243061568	1.73365008	7.381804623
0.180977835	3.728969795	13.06733524
0.200856124	2.036623068	10.62758621
0.149358968	4.698645546	17.68711295
0.10472306	10.08811383	34.20489408
0.181187674	2.674962497	12.39845758
0.201537424	2.525487565	11.14304124
0.175669623	5.169816955	16.75956284
0.23047619	2.249795918	8.428571429
0.135341672	8.807549955	25.2309434
0.189585022	3.567992681	12.18130312
0.138052857	10.187618	26.56555773
0.129328017	7.038184851	23.57066015
0.124250585	7.432848222	25.16672408
0.257198207	2.763111437	8.557312253
0.147562381	5.808774996	19.37006579
0.21858682	1.827615837	8.723577236
0.158666397	3.40968108	15.4116095
0.130025536	9.267886784	26.16232962

log.sigma.1.0.mm.3D_gldm_LargeDependenceLowGrayLevelEmphasis	log.sigma.1.0.mm.3D_gldm_SmallDependenceHighGrayLevelEmphasis	log.sigma.1.0.mm.3D_gldm_LargeDependenceHighGrayLevelEmphasis
0.164536824	44.80960304	1507.508499
0.276651072	36.86799222	6125.749639
0.305803507	45.25597264	944.3640553
0.048760254	123.226037	4159.396964
0.264565591	21.1908715	1273.272981
0.126875363	43.33783681	1747.306376
0.108259904	35.53050348	2848.192878
0.059682294	75.68529758	2311.818363
0.091697905	55.63486869	2641.127946
0.091387586	72.67897291	4573.016462
0.153667232	33.38635648	1419.376058
0.047764268	122.9907069	6269.238866
0.164451649	30.74211421	1771.419479
0.353721995	18.79267138	1499.527728
0.093408334	52.71255345	2023.418222
0.510793965	18.89981852	5621.18051
0.404290596	17.5327636	847.3123209
0.256139919	24.87150112	2680.419811
0.182771695	27.43163618	1217.345361
0.488444967	16.52361161	3515.77865
0.210135299	42.19664019	4416.417305
0.090193578	58.22412356	2599.062209
0.301055479	27.78284846	2964.858294
0.084224328	57.34972658	2015.126819
0.453755105	22.76909724	1588.226744
0.155117458	52.27351445	3102.572046
0.145391807	58.88685349	10121.93566
0.141945079	57.90544542	3804.904396
0.11074007	43.73314549	1687.278187
0.289492471	39.13730804	6404.697876
0.249399139	34.80513824	2312.27776
0.485407931	15.32997724	2180.375042
0.087038592	65.70135469	2543.551894
0.11774335	53.99992679	939.7692308
0.087007877	61.39982899	1163.894578
0.133186129	33.97284718	2075.557921
0.167010473	54.65313705	3445.967014
0.043890355	121.0695823	7138.863745
0.223366371	18.33370983	1123.720958
0.074787965	74.57205686	2897.471878
0.199748602	46.55101186	4436.761872
0.130582094	39.7143896	1821.736621
0.095486074	45.28134898	2060.897077
0.119598858	44.84358268	1695.06439
0.056925022	70.88908629	3178.71885
0.284565875	29.85289596	1897.836625
0.037307827	141.8270964	6288.132208
0.105592486	50.59500385	2065.181818
0.147041944	34.63078223	1249.932836
0.070878804	58.56937612	2260.947686
0.123457275	50.19879141	3752.104556
0.116695428	40.79085801	1647.049577
0.065528289	96.86853813	9044.572363
0.251905736	24.24984494	1121.062606
0.280929066	28.95710482	4498.234708
0.093705001	66.80187354	1799.817975
0.065029648	76.44471328	2251.094847
0.063407434	66.34423665	2835.566125
0.179025369	50.0466708	3945.980344
0.088874939	63.16059051	4470.792281
0.06598874	70.45720839	3419.549788
0.081095499	79.57128415	5551.114458
0.148541368	48.40385463	6092.503883
0.04211393	177.8880745	58359.06518
0.407555504	20.04429148	915.5069124
0.095429362	62.2633808	2999.627817
0.073341296	62.94662549	1388.667513
0.064986532	73.04927663	4490.736628
0.091078946	49.06992121	1848.639013
0.093407042	62.70370275	2663.027847
0.138013535	46.19748686	2747.059868
0.103203977	40.14484912	2174.519043
0.202324872	38.27221256	4350.223491
0.269625458	27.68104171	4170.237493
0.112402264	64.19951403	6314.425926
0.041430293	92.54620739	2175.769713
0.12908664	41.89921981	1817.636526
0.216230454	32.06360221	1033.204918
0.110890839	52.4915243	2845.157596
0.166807948	41.91503598	2415.764933
0.06842477	88.10728459	2221.869048
0.032582704	124.933819	2118.059657
0.012854286	363.0035672	13956.67085
0.119817182	40.24120267	1209.082759
0.14917331	38.74353383	2284.751991
0.130764254	54.5357701	9142.251644
0.095090197	48.31719456	1851.579692
0.061639076	64.52891753	2333.5
0.117644519	47.70310221	2762.038251
0.085783946	78.51044709	1510.304762
0.043103599	154.0993652	15099.91547
0.04657912	122.6833738	3558.791785
0.136229122	73.13015292	5429.423679
0.140492501	48.2255992	4245.282641
0.112683112	61.54608671	5795.654323
0.057404716	108.4106732	1744.158103
0.067360446	83.32270521	6170.04523
0.091059677	54.26437218	1276.138211
0.182461186	26.89764579	1555.07124
0.270549412	43.74558707	2815.77943

log.sigma.1.0.mm.3D_gldm_SmallDependenceLowGrayLevelEmphasis	log.sigma.1.0.mm.3D_gldm_LowGrayLevelEmphasis	log.sigma.1.0.mm.3D_gldzm_DistanceZoneVariabilityNormalized
0.011347824	0.020175897	0.931384649
0.002380428	0.00817729	0.911888164
0.017231814	0.032847648	0.927187728
0.001934506	0.004308682	0.866407433
0.012472425	0.026983151	0.844273427
0.004821621	0.011342502	0.886134068
0.005673966	0.01085063	0.648760331
0.003395492	0.007081541	0.850986121
0.003026237	0.007784657	0.931762492
0.001782662	0.005262219	0.946469152
0.005044586	0.013248449	0.903476013
0.001769637	0.003770307	0.913732402
0.003665792	0.011566403	0.917136834
0.006660981	0.019497176	0.909297052
0.005389033	0.010357397	0.853497923
0.00191466	0.010594329	0.90714813
0.018561578	0.039067078	0.794878056
0.002879067	0.011358828	0.9552807
0.008107266	0.018923951	0.832887952
0.00325467	0.013746304	0.946706699
0.002306885	0.007925522	0.898315701
0.002265397	0.006741513	0.889273356
0.002817492	0.01118543	0.958016099
0.00611141	0.011079524	0.730899545
0.009572555	0.02422577	0.922283363
0.004875902	0.01056614	0.943176681
0.001235126	0.004363049	0.877371027
0.002780051	0.007463668	0.940286606
0.004588412	0.011191353	0.926772907
0.002298294	0.008109193	0.942326031
0.003716355	0.012204537	0.958012334
0.003544602	0.016656996	0.923508652
0.004178526	0.008671351	0.948238056
0.015948438	0.027014651	0.809868719
0.010670741	0.01661676	0.8872
0.003273818	0.01001522	0.896927425
0.003133962	0.009225423	0.967293671
0.001013796	0.002856004	0.912966254
0.010140233	0.02304225	0.79375918
0.00317447	0.006771191	0.926807656
0.003445876	0.009084519	0.864670467
0.004408446	0.011170645	0.917678344
0.003387641	0.008945724	0.901838492
0.006135961	0.012853272	0.844162048
0.002265731	0.00556223	0.872277715
0.005538101	0.015788939	0.971113752
0.001253528	0.002995792	0.843361874
0.004810812	0.010214904	0.956777876
0.025137724	0.032727698	0.654320988
0.004080456	0.008651232	0.838487422
0.002088493	0.006734455	0.946883481
0.00507936	0.011925723	0.956385346
0.000984739	0.003095256	0.935805983
0.010138714	0.023615621	0.866614077
0.002053295	0.0089184	0.972913475
0.012641809	0.017963152	0.811858385
0.002784905	0.006729883	0.781980045
0.003351779	0.007137625	0.711716765
0.006008191	0.011051745	0.88408044
0.002558674	0.005922498	0.982144306
0.001956291	0.005349374	0.909438323
0.001378115	0.004346791	0.91734396
0.001508848	0.005474408	0.951471645
0.000504993	0.001223944	0.952522185
0.018589907	0.04091876	0.944489796
0.003051798	0.007396959	0.888850635
0.007411582	0.012526728	0.7888
0.00145535	0.004436051	0.949226208
0.005672816	0.011140249	0.874780415
0.003501665	0.008077294	0.911041023
0.002828733	0.008411913	0.901328063
0.003061481	0.008738931	0.9232
0.002155015	0.007882609	0.937229491
0.002334686	0.009265234	0.957075894
0.001697946	0.00508971	0.934232684
0.003835684	0.006968801	0.824358633
0.005113223	0.011860704	0.834710744
0.010650178	0.022728891	0.889273356
0.00319186	0.007991711	0.843842951
0.004108689	0.010988666	0.883478898
0.004695093	0.008583534	0.872742415
0.00408614	0.006553177	0.82428426
0.000684621	0.001337359	0.880888554
0.012060228	0.019478829	0.816743024
0.003299813	0.009687443	0.957056154
0.0009794	0.004124102	0.773808985
0.004051185	0.009488408	0.964456092
0.002652293	0.006562251	0.856953705
0.0074691	0.012419273	0.905
0.032562704	0.036141425	0.893200427
0.000776368	0.002050252	0.902272318
0.003202856	0.005599457	0.939655895
0.003132546	0.006795298	0.845550878
0.002358068	0.007377315	0.924168975
0.001289149	0.004771803	0.950215657
0.008407014	0.012142742	0.806502013
0.00234294	0.005310301	0.954310728
0.011723069	0.017911688	0.857988166
0.012694565	0.021591598	0.7738
0.006829855	0.015175059	0.93085824

log.sigma.1.0.mm.3D_gldzm_LowIntensityEmphasis	log.sigma.1.0.mm.3D_gldzm_LargeDistanceEmphasis	log.sigma.1.0.mm.3D_gldzm_HighIntensitySmallDistanceEmphasis
0.033606385	1.106719368	140.3498024
0.011876681	1.138568129	185.8152425
0.044247359	1.113513514	114.077027
0.005371176	1.215930902	335.5403071
0.052359967	1.255319149	84.93617021
0.016281371	1.181818182	149.4356061
0.022620825	1.681818182	133.1676136
0.009838845	1.243243243	224.4135135
0.010840373	1.106109325	198.8561093
0.006670265	1.082568807	272.9747706
0.018323821	1.152542373	125.8072034
0.005676092	1.135523614	402.698152
0.015734604	1.12992126	131.5684055
0.030346211	1.142857143	87.49900794
0.017650391	1.238754325	168.400519
0.015251446	1.146424518	142.7988082
0.075593455	1.348066298	70.68370166
0.015621755	1.068649886	139.1618993
0.031490044	1.27607362	109.9171779
0.021319193	1.082191781	109.341895
0.009946276	1.161186848	180.9851644
0.008408944	1.176470588	213.3615619
0.013321048	1.064356436	135.1212871
0.017504438	1.480662983	157.8522099
0.037793645	1.121495327	89.70327103
0.017122386	1.087804878	189.7646341
0.006205856	1.196861626	290.0356633
0.009993525	1.092417062	210.6042654
0.015429602	1.114186851	154.2045848
0.010824867	1.089160839	182.3662587
0.014965294	1.064362336	140.7240763
0.022042967	1.119496855	94.04979036
0.01323306	1.079763663	212.8408419
0.040454954	1.319148936	134.9574468
0.025309511	1.18	152.2666667
0.014173585	1.163522013	143.3485325
0.011459796	1.049889135	193.4506652
0.003694649	1.136787565	443.1831606
0.044114569	1.350253807	80.03680203
0.00940744	1.114130435	230.2119565
0.013392218	1.218978102	175.6922141
0.016073552	1.129032258	150.5008961
0.01238506	1.155279503	165.1718427
0.020856479	1.255520505	155.1269716
0.007680697	1.205685619	233.7077759
0.02114005	1.043973941	117.6547231
0.003912296	1.256968049	432.7826309
0.015925767	1.066298343	181.4875691
0.064344	1.666666667	90.63888889
0.013718617	1.265822785	189.8243671
0.008625499	1.081911263	205.4584755
0.016993265	1.066914498	143.0213755
0.004019426	1.099597586	399.9657948
0.038054996	1.215568862	90.94011976
0.011529435	1.041195477	159.0040388
0.031248154	1.315363881	169.3665768
0.008565381	1.373540856	230.4422827
0.011137364	1.523923445	204.0849282
0.019591009	1.185328185	172.6640927
0.010291823	1.027027027	266.3085586
0.007163545	1.142622951	262.3016393
0.005291314	1.129581152	300.2501636
0.007352144	1.074650078	240.3685848
0.003357386	1.072992701	1180.539538
0.063666448	1.085714286	74.51785714
0.010336767	1.177189409	214.7316701
0.018070074	1.36	159.1514286
0.005801087	1.078199052	292.2959123
0.017987992	1.201342282	163.2583893
0.01173315	1.139968896	214.5307154
0.011167272	1.156133829	184.3550186
0.012792775	1.12	176.0209091
0.010571589	1.097312326	190.8294717
0.012604272	1.065830721	151.9198668
0.007016525	1.10212766	261.4558511
0.010307468	1.291855204	243.5820136
0.018086833	1.272727273	145.5386364
0.034418231	1.176470588	109.7585784
0.011946814	1.256097561	194.1996951
0.015359954	1.1863581	154.5130938
0.0122416	1.204878049	232.4621951
0.009156853	1.291993721	283.8316327
0.001985408	1.190802348	1065.553327
0.037899375	1.306122449	140.1122449
0.012954737	1.06586169	154.5403403
0.005442025	1.394523327	266.0821783
0.014606928	1.054298643	180.2047511
0.008868806	1.232604374	221.4428429
0.023858054	1.15	169.45
0.065571475	1.169811321	180.3254717
0.002979951	1.154553818	596.1711132
0.0083036	1.093425606	337.4982699
0.01001655	1.253012048	223.1114458
0.010475669	1.118421053	205.5450405
0.005731917	1.07663411	279.0155898
0.017432494	1.325581395	235.0096899
0.009075745	1.070175439	310.6717836
0.02962621	1.230769231	147.3365385
0.049170183	1.39	101.605
0.02307652	1.107569721	148.6085657

log.sigma.1.0.mm.3D_gldzm_LowIntensityLargeDistanceEmphasis	log.sigma.1.0.mm.3D_gldzm_HighIntensityEmphasis	log.sigma.1.0.mm.3D_gldzm_DistanceZoneVariability	log.sigma.1.0.mm.3D_gldzm_ZonePercentage
0.040452409	142.3833992	235.6403162	0.358356941
0.015329686	189.7228637	394.8475751	0.20847376
0.068170782	114.8432432	171.5297297	0.426267281
0.006784619	354.1564299	902.7965451	0.39544592
0.081952165	85.79787234	79.36170213	0.26183844
0.020501635	152.9090909	175.4545455	0.307931571
0.073616997	146.3181818	57.09090909	0.261127596
0.013503009	232.0351351	314.8648649	0.369261477
0.011825688	204.1061093	579.5562701	0.299182299
0.007247034	277.440367	825.3211009	0.281471917
0.021590051	129.1567797	213.220339	0.285368803
0.006190664	414.2731006	444.9876797	0.328609987
0.02078112	135.3996063	465.9055118	0.244937319
0.041656636	89.91269841	229.1428571	0.225402504
0.034687635	179.3391003	246.6608997	0.317233809
0.017217295	148.3348468	799.1975028	0.126817331
0.14151502	72.83425414	143.8729282	0.259312321
0.018085406	139.6956522	417.4576659	0.187392796
0.052747988	112.208589	135.7607362	0.280068729
0.023019355	111.5045662	414.6575342	0.156372724
0.011627922	187.9318364	1120.199679	0.238705972
0.010272725	220.5679513	438.4117647	0.286627907
0.014550815	136.529703	580.5577558	0.221896741
0.026537766	173.6685083	132.2928177	0.376299376
0.041007359	92.75700935	296.0529595	0.266611296
0.018942034	190.9170732	193.3512195	0.295389049
0.007096122	304.7617689	615.0370899	0.202250433
0.010788879	215.8649289	396.8009479	0.29448709
0.023950123	157.532872	535.6747405	0.327478754
0.012280411	185.5	539.0104895	0.22501967
0.015957549	143.2252682	803.772348	0.263919472
0.029671973	96.5639413	881.0272537	0.160876897
0.0142369	217.6366322	641.957164	0.333005411
0.116491203	137.3829787	38.06382979	0.451923077
0.035989426	156.7666667	133.08	0.451807229
0.017553703	149.21174	427.8343816	0.236138614
0.012338755	195.2749446	872.4988914	0.313194444
0.004050929	459.8455959	881.0124352	0.28771616
0.115655455	84.03045685	156.3705584	0.235928144
0.010781113	234.2554348	341.0652174	0.350810296
0.017594882	185.323601	355.379562	0.278833107
0.021505062	151.4229391	256.0322581	0.292759706
0.015772514	169.3519669	435.5879917	0.294153471
0.029615664	159.9747634	267.5993691	0.309268293
0.009184795	247.9816054	521.6220736	0.318423855
0.022581038	118.2752443	596.2638436	0.275583483
0.0047688	459.874915	1240.585316	0.344819503
0.017135766	182.9337017	173.1767956	0.310463122
0.228290287	93.88888889	35.33333333	0.402985075
0.016868992	200.2531646	132.4810127	0.317907445
0.009559537	208.7042093	832.3105802	0.25671729
0.029725607	144.267658	257.267658	0.325272068
0.004440954	407.6619718	930.1911469	0.260141324
0.053658148	93.83233533	144.7245509	0.282571912
0.012635231	159.8521809	1204.466882	0.189789974
0.048855712	179.3530997	301.1994609	0.422070535
0.013821564	247.5590143	602.9066148	0.351573187
0.028643895	225.3755981	297.4976077	0.323279196
0.025654829	175.5482625	228.976834	0.318181818
0.010515187	267.1261261	218.036036	0.251986379
0.008364139	268.1590164	554.757377	0.287871638
0.005880396	311.7028796	1401.701571	0.278933917
0.007856876	244.6749611	611.7962675	0.208090615
0.003440656	1196.6691	391.486618	0.150494324
0.109202162	74.7	66.11428571	0.322580645
0.013222006	222.9633401	436.4256619	0.316162267
0.048262072	166.9085714	138.04	0.444162437
0.006399156	295.8957346	801.1469194	0.263997498
0.022188618	165.9261745	130.3422819	0.334080717
0.01425681	219.6360809	585.7993779	0.319741422
0.013605272	189.7100372	727.3717472	0.265460526
0.015295957	178.4945455	253.88	0.243578388
0.012003562	194.0296571	1011.270621	0.20878483
0.014148006	153.3487461	1221.22884	0.189994044
0.007626224	269.7489362	2195.446809	0.255991285
0.021859896	256.6561086	364.3665158	0.396057348
0.042046854	152.5727273	275.4545455	0.295434199
0.059637736	112.0784314	181.4117647	0.33442623
0.015848056	203.2560976	415.1707317	0.278911565
0.020453198	160.5030451	725.3361754	0.288475053
0.014762877	238.7804878	178.9121951	0.406746032
0.011832319	303.2574568	525.0690738	0.475018643
0.002209963	1117.089041	900.2681018	0.366045845
0.122406178	141.3673469	40.02040816	0.337931034
0.013718593	157.053787	871.8781559	0.268652315
0.007057556	302.6713996	762.9756592	0.180058437
0.016279405	182.8687783	213.1447964	0.284061697
0.01250305	227.1908549	431.0477137	0.324097938
0.025942256	174.35	54.3	0.327868852
0.071016472	183.4528302	47.33962264	0.504761905
0.003330429	616.3597056	980.7700092	0.273459119
0.008876375	341.9515571	271.5605536	0.409348442
0.011928819	238.6716867	280.7228916	0.324853229
0.011875919	210.9423077	913.0789474	0.241564792
0.006199536	283.4800902	1264.73704	0.229245608
0.02314065	243.5503876	104.0387597	0.509881423
0.009319228	317.0994152	326.374269	0.28125
0.094834543	148.4615385	44.61538462	0.422764228
0.062135256	108.13	77.38	0.263852243
0.026132324	150.2191235	233.6454183	0.311028501

log.sigma.1.0.mm.3D_gldzm_IntensityVariabilityNormalized	log.sigma.1.0.mm.3D_gldzm_LowIntensitySmallDistanceEmphasis	log.sigma.1.0.mm.3D_gldzm_IntensityVariability
0.06069459	0.031894879	15.35573123
0.067315949	0.011013429	29.147806
0.06477721	0.038266503	11.98378378
0.053201248	0.005017816	55.43570058
0.081484835	0.044961917	7.659574468
0.078512397	0.015226304	15.54545455
0.090909091	0.009871783	8
0.061636231	0.008922804	22.80540541
0.062613083	0.010594044	38.94533762
0.067794693	0.006526072	59.11697248
0.084314852	0.017507264	19.89830508
0.059480792	0.005547449	28.96714579
0.078949408	0.014472975	40.10629921
0.087899975	0.027518604	22.15079365
0.069359802	0.01339108	20.0449827
0.076799788	0.014759984	67.66061294
0.078782699	0.059113064	14.25966851
0.075881426	0.015005842	33.16018307
0.072151756	0.026175558	11.7607362
0.088446863	0.020894153	38.73972603
0.072819514	0.009525865	90.80593424
0.075379862	0.007942999	37.16227181
0.087638467	0.013013606	53.10891089
0.066084674	0.015246105	11.96132597
0.078871517	0.036990217	25.31775701
0.069839381	0.016667475	14.31707317
0.07257006	0.00598329	50.87161198
0.069124683	0.009794687	29.17061611
0.067066965	0.013299472	38.76470588
0.067142648	0.01046098	38.40559441
0.073245435	0.01471723	61.45292014
0.091522399	0.020135716	87.31236897
0.058427461	0.0129821	39.55539143
0.065640561	0.021445891	3.085106383
0.0624	0.022639532	9.36
0.074526764	0.013328555	35.54926625
0.059697347	0.011240056	53.84700665
0.065216247	0.003605579	62.93367876
0.080986369	0.026229347	15.95431472
0.063327032	0.009064022	23.30434783
0.062544029	0.012341552	25.70559611
0.075936846	0.014715675	21.18637993
0.069677524	0.011538196	33.65424431
0.064673745	0.018666683	20.50157729
0.065150278	0.007304673	38.95986622
0.074610871	0.020779804	45.81107492
0.055075673	0.00369817	81.01631543
0.069137084	0.015623268	12.51381215
0.0781893	0.023357429	4.222222222
0.07867329	0.012931023	12.43037975
0.067277041	0.00839199	59.13651877
0.073921035	0.013810179	19.88475836
0.067485395	0.003914045	67.0804829
0.075190935	0.034154209	12.55688623
0.074959351	0.011252985	92.7996769
0.052491627	0.026846265	19.47439353
0.058466694	0.007251335	45.07782101
0.0596827	0.006760731	24.94736842
0.062357448	0.018075054	16.15057915
0.081852122	0.010235982	18.17117117
0.066358506	0.006863397	40.47868852
0.061692216	0.005144044	94.26570681
0.081840807	0.007225961	52.62363919
0.083476892	0.003336569	34.30900243
0.088979592	0.05228252	6.228571429
0.05973511	0.009615457	29.3299389
0.072653061	0.010522074	12.71428571
0.066089598	0.00565157	55.77962085
0.071213008	0.016937835	10.61073826
0.059709904	0.011102235	38.39346812
0.069799723	0.010557773	56.3283767
0.074618182	0.01216698	20.52
0.074590227	0.010213596	80.48285449
0.076902006	0.012218338	98.12695925
0.062593753	0.0068641	147.0953191
0.05597756	0.007419361	24.74208145
0.069311295	0.012096828	22.87272727
0.071703191	0.028113355	14.62745098
0.069799722	0.010971503	34.34146341
0.063056995	0.014086643	51.76979294
0.061415824	0.011611281	12.5902439
0.052712257	0.008487987	33.57770801
0.053009907	0.00192927	54.17612524
0.076218242	0.016772674	3.734693878
0.075413202	0.012763773	68.701427
0.074499381	0.00504047	73.45638945
0.073360496	0.014188809	16.21266968
0.068009438	0.007960245	34.20874751
0.094444444	0.023337004	5.666666667
0.077251691	0.064210226	4.094339623
0.064190073	0.002892332	69.77460902
0.052813065	0.008160407	15.26297578
0.060005081	0.009538483	19.92168675
0.065475995	0.010125607	64.6902834
0.074806908	0.005615012	99.56799399
0.050898384	0.016005455	6.565891473
0.06730276	0.009014874	23.01754386
0.082100592	0.013324127	4.269230769
0.0792	0.045928915	7.92
0.060618085	0.022312569	15.21513944

log.sigma.1.0.mm.3D_gldzm_HighIntensityLargeDistanceEmphasis	log.sigma.1.0.mm.3D_gldzm_SmallDistanceEmphasis	log.sigma.1.0.mm.3D_glcm_SumVariance	log.sigma.1.0.mm.3D_glcm_Homogeneity1
150.5177866	0.973320158	290.168337	0.341877762
205.3533487	0.965357968	449.4088268	0.467028423
117.9081081	0.971621622	178.4208927	0.35718023
428.6209213	0.946017274	990.7992955	0.311437788
89.24468085	0.936170213	180.010136	0.394634748
166.8030303	0.954545455	351.957907	0.375841755
198.9204545	0.829545455	446.7756453	0.396417696
262.5216216	0.939189189	611.122828	0.328491004
225.1061093	0.973472669	517.5445143	0.365039802
295.3027523	0.979357798	727.250769	0.392513576
142.5550847	0.961864407	285.2725633	0.380147857
460.5728953	0.966119097	1211.633271	0.347799237
150.7244094	0.967519685	307.7243204	0.398723606
99.56746032	0.964285714	185.7698196	0.420738673
223.0934256	0.940311419	464.1144895	0.359937447
170.4790011	0.963393871	319.5176002	0.55241215
81.43646409	0.912983425	126.5165859	0.384011975
141.8306636	0.982837529	298.6858054	0.437028641
121.3742331	0.930981595	236.8784212	0.364003753
120.1552511	0.979452055	242.2663172	0.504386088
215.7185245	0.959703288	443.256432	0.436889004
249.3935091	0.955882353	543.8139392	0.37799288
142.1633663	0.983910891	295.1442642	0.453496594
236.9337017	0.879834254	455.0726192	0.341800794
104.9719626	0.969626168	167.7919766	0.434452133
195.5268293	0.97804878	425.7356973	0.396103043
363.6661912	0.950784593	859.3007024	0.461160211
236.9075829	0.976895735	516.5080365	0.420000818
170.8460208	0.971453287	381.918387	0.359674139
198.034965	0.97770979	437.3666105	0.477554925
153.2300358	0.983909416	286.7602325	0.424381469
106.6205451	0.970125786	189.1708868	0.48437251
236.8197932	0.980059084	543.3217771	0.344915312
147.0851064	0.920212766	301.5988786	0.312497868
174.7666667	0.955	365.013348	0.315186332
172.6645702	0.959119497	366.2373301	0.386350407
202.5720621	0.987527716	441.0686655	0.388357617
526.4953368	0.965803109	1376.971758	0.36239783
100.0050761	0.912436548	188.313916	0.38516793
250.4293478	0.971467391	615.7850352	0.353788524
223.8491484	0.945255474	448.3860385	0.413371254
155.1111111	0.967741935	355.6119947	0.372764756
186.0724638	0.961180124	436.1586582	0.358581467
179.3659306	0.936119874	362.1012686	0.355264087
305.0769231	0.948578595	723.495111	0.350312202
120.757329	0.989006515	232.1173591	0.423148506
568.2440517	0.935757988	1361.468662	0.334171722
188.718232	0.983425414	428.7171635	0.358805867
106.8888889	0.833333333	224.3572728	0.368860857
241.9683544	0.933544304	535.2187152	0.346663739
221.6871445	0.979522184	532.687017	0.401817095
149.2527881	0.983271375	343.2929555	0.354525695
438.4466801	0.975100604	1217.31315	0.387471953
105.4011976	0.946107784	182.8345069	0.384291722
163.2447496	0.989701131	380.0465465	0.486687602
219.2991914	0.92115903	432.6087723	0.310222604
316.0259403	0.906614786	616.0280749	0.325053826
310.5382775	0.869019139	633.592747	0.341434624
187.0849421	0.953667954	434.8688721	0.404172221
270.3963964	0.993243243	736.0018852	0.418827009
291.5885246	0.964344262	712.04033	0.365685618
357.5137435	0.967604712	861.8799872	0.384778804
261.9004666	0.981337481	655.6818756	0.463924733
1261.187348	0.981751825	4255.077386	0.514150771
75.42857143	0.978571429	123.7032723	0.386539419
255.8900204	0.955702648	558.4356963	0.373296237
197.9371429	0.91	421.8761352	0.307708002
310.2950237	0.980450237	838.8802299	0.380194782
176.5973154	0.94966443	420.0308021	0.349378935
240.0575428	0.965007776	535.5676536	0.35995758
211.1301115	0.960966543	441.9667745	0.398163005
188.3890909	0.97	428.7507136	0.382996942
206.8303985	0.975671918	447.9158094	0.449707103
159.0642633	0.98354232	366.6857838	0.467570472
302.9212766	0.974468085	764.8463519	0.41779899
308.9524887	0.927036199	720.8940621	0.298076249
180.7090909	0.931818182	352.0125837	0.36581295
121.3578431	0.955882353	214.2536675	0.35403432
239.4817073	0.93597561	504.7481169	0.377308263
184.4628502	0.953410475	359.4177939	0.382103533
264.0536585	0.948780488	572.5524329	0.335089649
380.9607535	0.92700157	849.2989695	0.280218034
1323.231898	0.952299413	3729.303855	0.325260941
146.3877551	0.923469388	308.0739437	0.374475344
167.1075741	0.983534577	374.1102652	0.401541146
451.2099391	0.902496056	875.2861337	0.451559465
193.5248869	0.986425339	427.7556916	0.370482518
250.1829026	0.941848907	605.6695582	0.340027704
193.95	0.9625	470.5521339	0.394995956
195.9622642	0.95754717	494.1520493	0.321582317
697.1140754	0.961361546	2013.616883	0.404242145
359.7647059	0.976643599	914.7551427	0.321362525
300.9126506	0.936746988	628.7865018	0.402739055
232.5313765	0.970394737	534.0213212	0.410704167
301.3380917	0.980841473	744.702093	0.426741382
277.7131783	0.918604651	607.0096312	0.284259987
342.8099415	0.98245614	1006.411106	0.381200037
152.9615385	0.942307692	365.7833931	0.325825014
134.23	0.9025	251.8224088	0.395829938
156.6613546	0.97310757	287.4725681	0.416234557

log.sigma.1.0.mm.3D_glcm_Homogeneity2	log.sigma.1.0.mm.3D_glcm_ClusterShade	log.sigma.1.0.mm.3D_glcm_MaximumProbability	log.sigma.1.0.mm.3D_glcm_Idmn	log.sigma.1.0.mm.3D_glcm_SumVariance2
0.255678003	44.48255112	0.038140959	0.960532299	26.88040418
0.403977891	20.71064507	0.105869024	0.976369339	15.99653613
0.272781847	112.4600706	0.04773512	0.956699291	25.39053805
0.224221653	38.64239732	0.028238765	0.970758673	34.88344422
0.310550437	2.999807984	0.03607485	0.968559421	15.77590834
0.290077386	5.997557964	0.027594483	0.969118427	16.02424893
0.315576386	-40.22299799	0.062877106	0.963054322	14.87925717
0.239623916	7.54920357	0.016898229	0.965216294	29.09175731
0.279453682	5.193623679	0.021159931	0.971688255	23.20528915
0.313089754	37.90889521	0.054352438	0.980843123	19.89818286
0.293662541	1.435806729	0.026171558	0.962924608	14.14326454
0.263271866	20.0004533	0.045488912	0.977302236	25.63652832
0.31577121	14.81433831	0.032274941	0.980491965	13.64592821
0.342194285	11.52748472	0.038936857	0.963450556	12.91063751
0.271176587	-7.596907877	0.02505619	0.969361945	18.91965008
0.506297038	22.38900733	0.167953636	0.988871435	9.304353388
0.29820097	5.012065097	0.026108477	0.949021308	16.16492433
0.363656676	20.79804932	0.049400271	0.976957498	14.0665746
0.275256553	1.687786938	0.024265141	0.959054191	20.86883637
0.444994169	9.0520296	0.111528517	0.978086322	9.198327662
0.365750957	25.07692888	0.087779043	0.981582455	13.95353672
0.292381474	35.93431162	0.026747482	0.981444415	17.85241795
0.38257361	17.40211477	0.089442808	0.974967886	11.53041324
0.254047568	-23.61561243	0.030793228	0.963282173	25.10031577
0.361265699	24.51338938	0.067577418	0.978797096	12.76861115
0.317562791	18.71418182	0.050028566	0.967765796	19.17720258
0.39543806	15.01126881	0.093267614	0.984111748	13.18286556
0.34713847	47.55988577	0.069577186	0.978585482	17.01439587
0.274631358	-7.224971961	0.023821326	0.958491787	23.84475719
0.416535284	26.09762064	0.107518715	0.982869699	13.9116651
0.349898881	37.39057288	0.074612419	0.979705062	14.51670342
0.419429439	13.6953437	0.09462609	0.983466312	9.259418168
0.259186834	25.16529948	0.028735346	0.97344591	28.49455259
0.22128771	-41.36391869	0.037136093	0.952176122	31.84956128
0.224925961	-4.366858867	0.019429064	0.945254216	29.71563756
0.302048365	1.055471592	0.027872372	0.975180825	16.53195945
0.310445668	61.32011224	0.073217613	0.972360212	24.63056407
0.278739793	8.032164773	0.043747346	0.980914409	18.88452349
0.299758736	-11.24021172	0.030012132	0.956923474	14.02543971
0.266779844	27.68183042	0.029572084	0.974406799	24.00883869
0.340036132	15.33179728	0.065851821	0.969224936	19.22304682
0.287928082	9.51341206	0.025505446	0.962466151	19.93916827
0.271105334	-5.259440542	0.022144524	0.967967893	20.99521328
0.268323601	13.82722163	0.02194569	0.967482987	23.83521149
0.261983728	-4.076492407	0.020629014	0.976495146	22.71299102
0.347572709	27.98245453	0.067161209	0.977529854	14.02507241
0.247562168	23.15251215	0.026558981	0.978748338	28.18280942
0.270635025	16.54994726	0.024844928	0.967693334	20.35097399
0.282372038	-64.96807507	0.053951209	0.95029218	20.04283567
0.257416755	-19.78940547	0.026253338	0.967941045	22.09049456
0.323051733	16.18417273	0.051155645	0.978253636	17.08048271
0.265707996	-1.297279321	0.022174752	0.967638535	20.3550421
0.307131577	14.38334658	0.056698735	0.984756793	17.99015055
0.299766018	9.769964017	0.035145493	0.96135135	15.28291054
0.42681701	17.15200631	0.123575738	0.980980186	13.81902237
0.220885107	4.266124784	0.022907788	0.952838778	36.10994002
0.236374254	19.12710357	0.014883568	0.96944714	31.68037547
0.253946197	-66.11649892	0.025427059	0.964384253	27.47973932
0.327589337	0.896918447	0.062713995	0.978593801	19.54322905
0.33969031	2.638631828	0.049423019	0.980678769	12.35105788
0.279579059	24.98259248	0.031958905	0.976157801	21.13568922
0.305427681	37.56462652	0.054361559	0.980682132	22.75463836
0.396961298	11.24376506	0.098188754	0.982793375	11.63108874
0.459387562	-65.53046889	0.119491981	0.994442232	12.12610551
0.302430625	9.70453745	0.047291147	0.939712714	16.81597386
0.29441573	18.92277983	0.036770911	0.970749242	25.86010483
0.21496305	-21.74805677	0.020412974	0.945814819	28.06644985
0.297798334	13.67657504	0.035570855	0.981157447	21.07886049
0.259219673	-2.557563387	0.024076139	0.962136001	20.10377639
0.276594407	36.86665443	0.041639284	0.97529751	26.26692672
0.318299588	21.72961867	0.042471347	0.974036536	17.33835774
0.298667459	11.70118959	0.027172546	0.974890439	16.98285928
0.380394609	23.27825781	0.085394875	0.982806993	13.81037421
0.401966756	18.43009095	0.094984387	0.988635398	11.98140614
0.345726476	16.60006715	0.059389726	0.981160784	19.12274449
0.205472586	-32.08953385	0.014554311	0.971655234	33.75027991
0.279110749	9.842006254	0.024044627	0.967520761	19.2802779
0.266102091	22.36347314	0.026775589	0.953363309	20.7217229
0.293528571	12.46899855	0.028522748	0.970765664	19.14744152
0.300436975	36.00728647	0.052896699	0.975940264	20.84041442
0.245693639	11.80969388	0.025709367	0.96503422	27.04070214
0.189137965	-31.95886005	0.012400105	0.963606622	40.79535296
0.242180707	-15.91309547	0.031217872	0.98402915	41.01782565
0.291302826	-31.13049935	0.052898546	0.955669746	21.53280802
0.321126615	14.50990957	0.052003729	0.978035858	15.21729103
0.382774906	24.92074861	0.075507451	0.989221348	12.8021449
0.284302298	17.74377437	0.025947578	0.970454069	18.02816017
0.249819671	-12.31982112	0.019408216	0.967033845	24.56713867
0.312967365	-8.711709749	0.062254181	0.965755483	14.29385951
0.234866161	-113.7964615	0.048621599	0.93955439	31.93531553
0.329379238	-38.55892453	0.058080538	0.985492878	21.51773358
0.23394446	57.79658482	0.025401749	0.970193777	32.77558513
0.328898152	21.13377441	0.071801995	0.973952072	21.40286486
0.334318795	23.97341053	0.05377393	0.981796368	17.37841002
0.353027317	22.26129021	0.071025007	0.987562144	14.42335493
0.193642438	88.45313883	0.021254284	0.948119715	48.78391786
0.298625194	-77.21786194	0.035847225	0.981542885	21.21461937
0.234273192	-37.76871391	0.031165818	0.936998164	27.84519533
0.312637435	3.632797815	0.03746798	0.970970487	15.81490908
0.342839237	80.2255454	0.058573803	0.972964006	21.68834703

log.sigma.1.0.mm.3D_glcm_Contrast	log.sigma.1.0.mm.3D_glcm_DifferenceEntropy	log.sigma.1.0.mm.3D_glcm_InverseVariance	log.sigma.1.0.mm.3D_glcm_Entropy	log.sigma.1.0.mm.3D_glcm_Dissimilarity
25.91437373	3.448567526	0.246492951	7.363927373	3.873247791
14.06834727	2.974904391	0.344633897	6.398559486	2.53603082
24.93728096	3.384837281	0.266467503	6.941134427	3.669138283
37.52354298	3.709989078	0.220661096	8.021534814	4.623034135
12.6035514	2.957787783	0.307344807	6.409555264	2.738029896
15.10265803	3.087470882	0.29923399	6.801691346	2.989341925
15.43353488	3.063633782	0.308740596	6.302474782	2.906991315
26.41982064	3.463831153	0.246072498	7.592629989	3.94550406
19.64070727	3.275565845	0.277183871	7.353926779	3.337703186
18.86575764	3.217253595	0.303602549	7.02792541	3.118575838
13.54153373	3.016550744	0.303718755	6.699815357	2.858069703
28.99932491	3.493552641	0.257984659	7.364973999	3.915686439
12.04959435	2.944372635	0.323504072	6.643898876	2.654541256
10.57470341	2.851019423	0.333842444	6.470303935	2.473830255
18.15947904	3.210193329	0.275983974	7.033468884	3.251929515
8.266195516	2.545027805	0.400915285	5.4024096	1.771538756
13.50015125	3.014451128	0.300699448	6.747902969	2.848416354
11.18750137	2.888783294	0.344831452	6.548033494	2.441317879
16.88503893	3.152970673	0.274254563	7.005199763	3.177307475
7.927602667	2.63974885	0.378771682	5.863725965	1.942459355
13.50718881	3.012462915	0.334900133	6.536287597	2.618514964
15.70360568	3.102584274	0.298985132	6.935806119	2.993674592
9.980335629	2.818601433	0.347239622	6.267473543	2.30075026
23.96383852	3.377633426	0.257760993	7.224561792	3.725410916
12.36303896	2.93985663	0.340744038	6.370985478	2.526771782
17.83875453	3.173992808	0.304785807	6.861194252	3.069203556
12.60911477	2.932943101	0.350937679	6.348671817	2.443241204
15.99780803	3.091115633	0.333327663	6.659207542	2.826904753
21.26004456	3.292506567	0.275171836	7.35993989	3.467510123
12.76077465	2.905836328	0.361127674	6.232895361	2.383481776
13.98955235	3.023997483	0.332507283	6.567675152	2.673283478
7.102404764	2.604420221	0.384368758	5.929881619	1.934983009
26.44125352	3.462427616	0.259580502	7.622263935	3.82681108
29.47154607	3.374628896	0.227937221	6.307226505	4.210206777
28.79560697	3.477068607	0.239216675	7.273842056	4.145467993
14.32028543	3.06814835	0.303169861	6.917785229	2.888175682
22.55188917	3.354424442	0.277148228	7.224184763	3.424807365
20.94772255	3.3201726	0.280477308	7.201305719	3.444822504
12.58875983	2.957449417	0.312569236	6.628167655	2.771301797
22.03516405	3.343539079	0.263886625	7.322597145	3.539562382
18.55900612	3.205457993	0.308554971	6.90671262	3.057344752
17.20846063	3.176910228	0.293609002	7.080499636	3.138153735
18.90133077	3.238441415	0.274899198	7.229962647	3.32961133
21.08449078	3.306958137	0.270983899	7.322931449	3.468147633
21.63644366	3.331069563	0.264364304	7.363597981	3.517019499
13.03851969	2.994754923	0.327506116	6.555577949	2.64004068
30.03942229	3.554489585	0.246388645	7.751827115	4.06770266
19.0814759	3.243948293	0.265027749	7.043356255	3.350493829
16.99392024	3.079295056	0.286236209	6.152333794	3.152579198
20.50689265	3.281008246	0.260298974	7.053772893	3.502724047
16.08078876	3.136151832	0.307167537	6.946116176	2.937395886
19.03989305	3.242699274	0.265235207	7.101472232	3.371173816
18.69874081	3.242651772	0.286136493	7.031774143	3.197404771
14.23526464	3.048537159	0.303468188	6.706899932	2.896909476
12.0948248	2.874796642	0.354580955	6.186469418	2.328994553
34.55274269	3.646945141	0.217410295	7.846264654	4.492572495
28.57582595	3.505595828	0.242772917	7.775142102	4.070058888
25.37659112	3.430803513	0.253939012	7.455798163	3.79428731
18.61298494	3.193736261	0.305411274	6.822369452	3.065822703
11.12511542	2.876800747	0.335182054	6.349558962	2.491427079
19.01505007	3.251844327	0.278338931	7.237962839	3.289969555
21.46701184	3.325240983	0.283021348	7.30689363	3.358464877
10.76107577	2.851363047	0.353989397	6.238251944	2.309490324
11.3041545	2.704533422	0.380304232	5.791001942	2.058672692
14.32195413	3.019728388	0.29813418	6.442069075	2.90811164
24.01825662	3.374731181	0.288876472	7.387092873	3.549340702
27.95118189	3.47513609	0.222450003	7.327893596	4.1808547
18.23009417	3.225521237	0.290257308	7.212353212	3.181112687
18.85365315	3.235611896	0.262608301	7.028317534	3.38434558
24.55032362	3.408598919	0.267256014	7.451179171	3.650397892
15.15570142	3.094045294	0.312139811	6.916198115	2.892858757
14.55539131	3.064124992	0.308627014	6.880025897	2.906000713
12.65045483	2.954398437	0.341040146	6.498764898	2.494951211
10.86571109	2.848414368	0.360931824	6.273510853	2.289788487
18.56595921	3.18658941	0.322987683	6.968363493	2.997438144
34.07036034	3.626787274	0.214162026	7.844014172	4.508020984
17.6447731	3.195924809	0.282265346	7.078330968	3.208048316
19.61804447	3.259940227	0.269416802	7.08518194	3.40619946
17.17360799	3.183330644	0.290348814	7.081235173	3.132570824
19.31244137	3.261367949	0.284635309	7.175413623	3.241079073
24.76525848	3.393495309	0.252053459	7.270739514	3.788836571
41.93623348	3.776296906	0.201016545	8.122955574	5.035931031
41.02320432	3.746070804	0.230771358	8.058441022	4.666160451
16.85239692	3.061383777	0.309555582	6.229842051	3.096193499
13.73914838	3.036960487	0.316798362	6.785925108	2.778314336
12.52005855	2.926522814	0.352120067	6.39025334	2.444860811
15.85062239	3.114203465	0.297424752	6.897121155	3.059212042
21.10438573	3.320091047	0.257323814	7.416421894	3.571476045
13.90997913	2.879782047	0.305631027	5.991388115	2.839522676
33.03852878	3.408578642	0.240223304	6.055719467	4.319083102
20.31203992	3.251174032	0.30600803	7.088078518	3.162671778
34.25161617	3.610092718	0.232612151	7.553246024	4.364088159
21.3586196	3.279586218	0.30424277	6.967352883	3.255097398
16.71295797	3.141046814	0.313401288	6.917609504	2.915820565
13.52214425	3.005227929	0.33811554	6.625750667	2.634215704
45.10385528	3.752678394	0.196821508	7.440338813	5.131986279
19.61071825	3.223745394	0.29029786	6.98408664	3.201171973
24.65242757	3.296776827	0.228847744	6.481868791	3.899157365
12.89628525	2.967919346	0.31620237	6.450790048	2.732070105
19.42179704	3.184694437	0.315794592	6.718447201	3.033237561

log.sigma.1.0.mm.3D_glcm_DifferenceVariance	log.sigma.1.0.mm.3D_glcm_Idn	log.sigma.1.0.mm.3D_glcm_Idm	log.sigma.1.0.mm.3D_glcm_Correlation	log.sigma.1.0.mm.3D_glcm_Autocorrelation
10.84643174	0.872126748	0.255678003	0.017769193	106.0586551
7.587860492	0.909922553	0.403977891	0.062377971	153.3127983
11.44916516	0.870170274	0.272781847	0.008105864	68.78374907
16.108609	0.888999816	0.224221653	-0.031660493	314.3193452
5.049836898	0.882749883	0.310550437	0.110760908	70.95746094
6.145275453	0.884009913	0.290077386	0.029309932	124.9138672
6.891866983	0.878513234	0.315576386	-0.013814354	151.4683155
10.81078936	0.877872742	0.239623916	0.04819853	204.5530129
8.478075915	0.890696393	0.279453682	0.083365387	176.8105913
9.117782199	0.912556067	0.313089754	0.026406648	236.2061066
5.347718132	0.872777865	0.293662541	0.021502573	104.0928795
13.53541499	0.904321456	0.263271866	-0.05761681	374.1417073
4.96295824	0.906260326	0.31577121	0.062789495	111.1443624
4.362572137	0.876464059	0.342194285	0.098470303	72.7752596
7.572646911	0.884826548	0.271176587	0.020478276	159.3000211
5.102187682	0.941520628	0.506297038	0.059481068	110.4223615
5.30673801	0.85341919	0.29820097	0.0889226	53.30049546
5.168143846	0.903525531	0.363656676	0.112469758	108.6343358
6.689093533	0.867571337	0.275256553	0.105145251	90.09871748
4.088806137	0.910887591	0.444994169	0.073566179	89.05018838
6.644906236	0.915155413	0.365750957	0.016148204	151.2381084
6.654339392	0.909165153	0.292381474	0.063533284	182.8312227
4.616510568	0.900325479	0.38257361	0.070741829	106.3811268
10.02006089	0.875987073	0.254047568	0.023325812	156.879883
5.949847232	0.90837788	0.361265699	0.015333664	66.12945074
8.367435914	0.887994033	0.317562791	0.033803927	147.3619806
6.63026592	0.923246922	0.39543806	0.022641541	270.4005052
7.979708057	0.909643171	0.34713847	0.029433286	173.4004347
8.929064149	0.870190576	0.274631358	0.059940474	135.4325419
7.067723453	0.923065586	0.416535284	0.042502534	148.7714665
6.823879869	0.910550376	0.349898881	0.018663007	104.0209566
3.320482836	0.917573954	0.419429439	0.131298399	72.90761748
11.74061715	0.895145199	0.259186834	0.037545237	184.4092836
11.66498061	0.857540992	0.22128771	0.040023434	106.1500004
11.4603016	0.849095029	0.224925961	0.017226807	128.9563717
5.963997927	0.895725217	0.302048365	0.071353582	129.9943612
10.77436755	0.896745339	0.310445668	0.043242373	152.8411814
9.076371345	0.908837711	0.278739793	-0.04632512	419.3943032
4.802776609	0.863356608	0.299758736	0.055588055	73.52689969
9.480560643	0.89551134	0.266779844	0.042645478	205.2670558
9.174785446	0.893287566	0.340036132	0.017005703	154.1180658
7.338890482	0.875073519	0.287928082	0.073044799	127.1005082
7.701331653	0.882555351	0.271105334	0.052548632	151.6642129
8.96975991	0.883222587	0.268323601	0.060374553	129.2766084
9.217470524	0.898932362	0.261983728	0.024877884	236.4987935
6.043225332	0.904513651	0.347572709	0.033919482	87.1579365
13.46673292	0.905304985	0.247562168	-0.028366146	418.4627859
7.829053683	0.881895138	0.270635025	0.03148715	148.7763752
6.987996417	0.856818233	0.282372038	0.085437418	83.70754186
8.186905338	0.881405158	0.257416755	0.038371944	180.684987
7.418004407	0.905715974	0.323051733	0.030306558	179.3479441
7.57730937	0.881033483	0.265707996	0.033498823	122.8624738
8.419937926	0.919212364	0.307131577	-0.015280964	374.4335373
5.825948282	0.871923364	0.299766018	0.035755572	71.71688715
6.595780713	0.919266735	0.42681701	0.067435223	131.5848066
14.31678031	0.860369006	0.220885107	0.022128949	150.4243155
11.69081785	0.885722083	0.236374254	0.051282327	206.2963029
10.78345099	0.878576003	0.253946197	0.041594894	210.5083657
9.189986057	0.908916702	0.327589337	0.024577704	149.9503674
4.904113623	0.908413891	0.33969031	0.050648783	235.8919115
8.157353634	0.898629647	0.279579059	0.052968264	233.0359045
10.13205101	0.911690707	0.305427681	0.032063665	276.3139427
5.383162383	0.918626609	0.396961298	0.040332286	212.2382876
7.037044571	0.957094546	0.459387562	0.033859021	1180.840264
5.844515985	0.843532024	0.302430625	0.07912042	51.76206894
11.3571972	0.893484388	0.29441573	0.036855428	188.2703238
10.37373541	0.846795864	0.21496305	0.003055023	146.4757921
8.080034624	0.910235432	0.297798334	0.072353997	269.9035505
7.38653415	0.87107658	0.259219673	0.031761923	146.0187108
11.13608866	0.899487944	0.276594407	0.034927563	181.6110018
6.716710955	0.896439522	0.318299588	0.065534348	152.6908206
6.085644814	0.895207072	0.298667459	0.076516334	148.7679735
6.391310538	0.918882005	0.380394609	0.043583705	152.7911351
5.607884219	0.93360086	0.401966756	0.047881471	127.7866287
9.561010835	0.91627047	0.345726476	0.015059822	246.4876414
13.60119854	0.887668532	0.205472586	-0.002072693	236.2704332
7.302811627	0.881917766	0.279110749	0.044416702	125.6862399
7.952357142	0.860330244	0.266102091	0.026753311	82.00274025
7.299455128	0.888783299	0.293528571	0.053749109	171.8274969
8.775071618	0.900597681	0.300436975	0.038036053	128.0134209
10.3413191	0.878255439	0.245693639	0.041266084	192.4324769
16.50540147	0.873614317	0.189137965	-0.009795015	273.88288
19.11362237	0.918615396	0.242180707	0.004341735	1069.400627
7.206096196	0.86571343	0.291302826	0.122271525	110.7267755
5.97958309	0.903129637	0.321126615	0.050152279	131.7272617
6.526256112	0.935342387	0.382774906	0.013263837	275.0752968
6.481855418	0.886221134	0.284302298	0.063628092	148.5117186
8.289111638	0.879296664	0.249819671	0.075555403	202.9280053
5.352467512	0.879744986	0.312967365	0.018587524	157.9873692
14.14895219	0.845974249	0.234866161	-0.015064114	162.8730829
10.27386995	0.924751553	0.329379238	0.026769451	595.78537
15.15303859	0.889084895	0.23394446	-0.019692106	291.4913321
10.72757186	0.901695601	0.328898152	0.000907745	207.1697792
8.174017704	0.915165084	0.334318795	0.019394481	179.5115996
6.569441068	0.928539389	0.353027317	0.032619974	239.5232829
18.44349604	0.854041818	0.193642438	0.040571035	200.8480408
9.32794651	0.912753177	0.298625194	0.03945351	315.9527049
9.364765579	0.836910512	0.234273192	0.062824486	127.7430955
5.40842092	0.888228459	0.312637435	0.100809645	93.7712488
10.18165977	0.901793282	0.342839237	0.053581199	104.4261124

log.sigma.1.0.mm.3D_glcm_SumEntropy	log.sigma.1.0.mm.3D_glcm_AverageIntensity	log.sigma.1.0.mm.3D_glcm_Energy	log.sigma.1.0.mm.3D_glcm_SumSquares	log.sigma.1.0.mm.3D_glcm_ClusterProminence
4.347468119	10.28662773	0.00909313	13.19869448	2277.785116
3.906489693	12.36243835	0.031361322	7.51622085	1153.833068
4.202857654	8.286402071	0.012777441	12.58195475	2928.270492
4.57749305	18.04298971	0.006052603	18.53544412	4495.296205
3.937270669	8.376080628	0.015469622	7.094864936	869.5644571
4.003942742	11.16608883	0.011849886	7.78172674	755.8847072
3.844019675	12.31272661	0.019149522	7.578198012	886.1314316
4.432246514	14.28481382	0.006689525	13.93540105	2382.520884
4.293161619	13.26341305	0.008656885	10.7114991	1773.228699
4.125088137	15.421266	0.014376079	10.09914356	1811.445389
3.9242462	10.19502377	0.012378789	6.921199568	575.8231727
4.290674311	19.6214625	0.010808608	13.63970763	3051.069532
3.898457638	10.52351158	0.014104678	6.42388064	739.4386997
3.845634551	8.496450178	0.015606038	5.871335231	516.8624403
4.128138754	12.61385324	0.010512367	9.26978228	1237.126757
3.378975301	10.49644891	0.067868847	4.401577211	678.2336958
4.004371349	7.254320923	0.011777555	7.416268895	709.1967167
3.905668115	10.38809888	0.017201378	6.313518992	713.3004463
4.181201881	9.439032177	0.010006687	9.438468825	1220.886761
3.573364993	9.419761492	0.035236472	4.281482582	351.8754438
3.867088115	12.29505168	0.023322463	6.886131013	835.6100261
4.069316917	13.50772385	0.011674301	8.4589093	1274.685015
3.749794751	10.29526309	0.023324868	5.377687217	475.9593156
4.291908577	12.51371827	0.009248121	12.26603857	2051.468823
3.800906787	8.125750237	0.020800452	6.282912528	717.5644709
4.087669719	12.12533605	0.013891147	9.253989278	1395.052212
3.790910831	16.45874673	0.028681246	6.573344355	915.7667011
3.967730374	13.16181644	0.01949536	8.289145481	1345.547578
4.297089236	11.60948691	0.008451543	11.27620044	1693.360375
3.792818425	12.18816255	0.034876931	6.703178412	1029.615661
3.885397833	10.19258141	0.020738566	7.126563944	1011.307978
3.600902272	8.506926699	0.029038331	4.090455733	353.1759797
4.431838753	13.60438448	0.007825884	14.10379171	2760.653217
4.125650018	10.27174444	0.014846307	15.33027684	3567.168808
4.380166567	11.34524167	0.007949464	14.62781113	2486.791777
4.053993379	11.37718159	0.011224652	7.713061221	796.9538492
4.276902524	12.3483514	0.015148731	11.87520019	2298.688231
4.131233377	20.73094056	0.011395469	9.864934692	1336.927125
3.905573074	8.553631619	0.01322842	6.653549886	580.5626836
4.293360113	14.32879258	0.008775257	11.71797389	1946.565546
4.099253136	12.40764072	0.018130991	9.445513236	1438.762511
4.165696789	11.24349864	0.010018245	9.286907227	1238.216511
4.212179074	12.29382166	0.008840435	9.974136013	1276.773305
4.287255579	11.33946631	0.008541641	11.22992557	1858.268474
4.267302788	15.41127799	0.008517116	11.39468663	1748.250692
3.877213919	9.322514035	0.019291305	6.765898025	786.5081282
4.42120454	20.68658287	0.007559961	14.00176177	3132.163718
4.160689538	12.18416942	0.009912192	9.858112473	1292.373632
3.922624965	9.105899348	0.018563384	9.259188976	1455.929055
4.201987914	13.42691889	0.009900342	10.6493468	1423.599407
4.05856559	13.3853746	0.014077111	8.315815411	1089.811231
4.168321633	11.06931697	0.009183194	9.848733787	1161.788759
4.078469644	19.55574239	0.014153125	9.095370424	1321.852258
3.962076112	8.453024434	0.013357935	7.379543795	752.9172087
3.767537143	11.4521929	0.039875667	6.478461795	896.2197564
4.585454238	12.24872217	0.005980234	17.66567068	3976.384586
4.497999115	14.37562697	0.006139646	15.38231108	3089.195608
4.362115495	14.49055147	0.008718328	13.21408261	2715.848415
4.092390184	12.24121616	0.017534416	9.601722532	1717.251913
3.79677935	15.34871957	0.018368353	5.869043325	622.4065738
4.211234245	15.26390028	0.009823188	10.16938703	1494.514648
4.258436267	16.75557881	0.011945676	11.59128602	2038.527605
3.743709692	14.56090522	0.027951082	5.598041128	573.8880812
3.582854093	34.3971807	0.044625703	5.481029241	2463.821104
3.963456632	7.150807138	0.015087093	7.784481997	830.8248195
4.331116439	13.71781053	0.01063927	12.59943804	2434.120994
4.358389461	12.10109376	0.007606543	14.00440794	2066.909846
4.216887244	16.47255757	0.010412032	10.13628823	1503.816378
4.143560596	12.07078827	0.009628209	9.739357385	1139.384531
4.353159296	13.49969424	0.009746235	13.00726706	2497.847854
4.062786968	12.33459526	0.01342887	8.12351479	1015.221678
4.052157525	12.17206368	0.011471674	7.884562648	945.2801817
3.863086025	12.34995995	0.023028334	6.625321168	875.6890588
3.75039093	11.29509412	0.028024762	5.749540087	672.6195952
4.083082294	15.77080315	0.018635428	9.839831124	1752.383629
4.53384378	15.57069037	0.005593045	17.56771307	3588.378602
4.144374633	11.19259379	0.009820403	9.23126275	1189.965572
4.168858289	9.040049736	0.009792456	10.08494184	1254.355983
4.142508091	13.08938185	0.010527821	9.080262379	1125.271615
4.194293854	11.30233711	0.011872459	10.10490214	1695.534083
4.346449805	13.8511725	0.008921845	12.95149015	2444.305207
4.681842876	16.81412859	0.004560446	21.2651619	4741.401483
4.672228816	32.95888295	0.006844434	20.48799957	6505.164016
4.006615281	10.46587421	0.018010222	9.596301236	1568.637761
3.977815675	11.46111423	0.014431202	7.239109854	774.5462211
3.798508314	16.68997821	0.023664049	6.734175392	949.5996311
4.086519232	12.16406222	0.011222426	8.46969564	1006.032327
4.323542589	14.21459995	0.007588749	11.4178811	1655.013812
3.769739756	12.56426422	0.021700572	7.050959661	663.837066
4.047110088	12.77132307	0.018384046	16.24346108	3222.407312
4.172177626	24.44401781	0.015779441	10.0826629	2421.233935
4.469658382	17.32542355	0.007837136	17.75267123	4139.048602
4.140849982	14.41476352	0.017635709	10.89123331	1922.815006
4.054164025	13.40967236	0.015312701	8.684625415	1392.453832
3.914876203	15.53011756	0.019428385	7.305957944	866.2330914
4.652070889	14.22205385	0.007021205	24.19795007	7347.265081
4.139777434	17.91033963	0.012156076	10.43294412	2951.853451
4.150408473	11.26538786	0.012930929	13.12440573	2153.012593
3.929200054	9.645697018	0.015273205	7.177798584	1017.734885
4.079411993	10.19103153	0.01880302	10.27753602	2650.637536

log.sigma.1.0.mm.3D_glcm_SumAverage	log.sigma.1.0.mm.3D_glcm_Imc2	log.sigma.1.0.mm.3D_glcm_Imc1	log.sigma.1.0.mm.3D_glcm_DifferenceAverage	log.sigma.1.0.mm.3D_glcm_Id
20.57325546	0.68204355	-0.081885094	3.873247791	0.341877762
24.7248767	0.606652462	-0.07063299	2.53603082	0.467028423
16.57280414	0.761757727	-0.118503445	3.669138283	0.35718023
35.49524401	0.549592853	-0.043737672	4.623034135	0.311437788
16.75216126	0.691935011	-0.097754839	2.738029896	0.394634748
22.33217766	0.59198425	-0.061741358	2.989341925	0.375841755
24.62545322	0.706669438	-0.104942193	2.906991315	0.396417696
28.5572564	0.624705412	-0.063237241	3.94550406	0.328491004
26.5268261	0.495761076	-0.037833438	3.337703186	0.365039802
30.72114018	0.500316306	-0.040270546	3.118575838	0.392513576
20.39004755	0.515797423	-0.045290056	2.858069703	0.380147857
38.72875225	0.651859822	-0.072855491	3.915686439	0.347799237
21.04702317	0.411502421	-0.027972111	2.654541256	0.398723606
16.99290036	0.486417711	-0.045916712	2.473830255	0.420738673
25.22770648	0.572962388	-0.055142197	3.251929515	0.359937447
20.991685	0.569351417	-0.071468639	1.771538756	0.55241215
14.50864185	0.542522952	-0.050989824	2.848416354	0.384011975
20.77619777	0.502756748	-0.045030345	2.441317879	0.437028641
18.87806435	0.63969787	-0.073782477	3.177307475	0.364003753
18.83952298	0.508487361	-0.055144965	1.942459355	0.504386088
24.58668972	0.440425184	-0.033062031	2.618514964	0.436889004
27.00308474	0.493731824	-0.041260683	2.993674592	0.37799288
20.59052619	0.449604733	-0.039995931	2.30075026	0.453496594
25.02743654	0.743542179	-0.106058758	3.725410916	0.341800794
16.25150047	0.549887323	-0.055437428	2.526771782	0.434452133
24.2506721	0.707253938	-0.097128037	3.069203556	0.396103043
32.87898363	0.516737443	-0.0479688	2.443241204	0.461160211
26.31688096	0.573080601	-0.058702329	2.826904753	0.420000818
23.21897381	0.559373893	-0.055144491	3.467510123	0.359674139
24.37075074	0.58249243	-0.064755323	2.383481776	0.477554925
20.38516281	0.458605092	-0.035911083	2.673283478	0.424381469
17.0138534	0.387852599	-0.029946343	1.934983009	0.48437251
27.12155264	0.557545574	-0.047966304	3.82681108	0.344915312
20.54348888	0.962287747	-0.344101981	4.210206777	0.312497868
22.69048333	0.82073499	-0.143992517	4.145467993	0.315186332
22.75436318	0.425380735	-0.028723009	2.888175682	0.386350407
24.6836246	0.557181447	-0.051314235	3.424807365	0.388357617
40.98337878	0.440032618	-0.029594573	3.444822504	0.36239783
17.10726324	0.508493032	-0.045319491	2.771301797	0.38516793
28.61968232	0.613298234	-0.062559168	3.539562382	0.353788524
24.81528143	0.596350532	-0.062185775	3.057344752	0.413371254
22.48699729	0.571636442	-0.054550562	3.138153735	0.372764756
24.58764331	0.509309596	-0.041183949	3.32961133	0.358581467
22.67893262	0.604307843	-0.060446238	3.468147633	0.355264087
30.73948692	0.521303773	-0.042271075	3.517019499	0.350312202
18.64502807	0.487852942	-0.040990493	2.64004068	0.423148506
40.93532818	0.484321033	-0.033798033	4.06770266	0.334171722
24.36833884	0.641477159	-0.072991078	3.350493829	0.358805867
18.2117987	0.883390396	-0.221441561	3.152579198	0.368860857
26.85383778	0.680679678	-0.084836249	3.502724047	0.346663739
26.76546473	0.456622619	-0.033757824	2.937395886	0.401817095
22.13863395	0.603440066	-0.062533154	3.371173816	0.354525695
38.70967021	0.48733241	-0.038390275	3.197404771	0.387471953
16.90604887	0.601180119	-0.065203221	2.896909476	0.384291722
22.9043858	0.556994505	-0.060046663	2.328994553	0.486687602
24.49744434	0.693675643	-0.080558214	4.492572495	0.310222604
28.67106561	0.566214878	-0.049804621	4.070058888	0.325053826
28.98110294	0.599149823	-0.058265532	3.79428731	0.341434624
24.47167926	0.670662854	-0.084516392	3.065822703	0.404172221
30.69743913	0.521276405	-0.049132966	2.491427079	0.418827009
30.49620115	0.491717111	-0.037677018	3.289969555	0.365685618
33.22596729	0.495083879	-0.038102468	3.358464877	0.384778804
29.12181043	0.47608117	-0.041534421	2.309490324	0.463924733
68.72070611	0.595144395	-0.073824736	2.058672692	0.514150771
14.30161428	0.785405882	-0.139370532	2.90811164	0.386539419
27.40858881	0.631439272	-0.067013414	3.549340702	0.373296237
24.20218753	0.76609584	-0.114070756	4.1808547	0.307708002
32.81404518	0.477739314	-0.035447085	3.181112687	0.380194782
24.14157654	0.666217609	-0.080375589	3.38434558	0.349378935
26.92068625	0.574247402	-0.052652511	3.650397892	0.35995758
24.66919051	0.448292492	-0.032936275	2.892858757	0.398163005
24.34412735	0.531923093	-0.04738984	2.906000713	0.382996942
24.69819905	0.479382134	-0.040705603	2.494951211	0.449707103
22.58383822	0.470517022	-0.039839182	2.289788487	0.467570472
31.39095883	0.470613704	-0.035451746	2.997438144	0.41779899
30.74701545	0.645924741	-0.066356932	4.508020984	0.298076249
22.38518758	0.542147606	-0.048178044	3.208048316	0.36581295
18.08009947	0.642891952	-0.073159016	3.40619946	0.35403432
26.17876369	0.496749132	-0.04038287	3.132570824	0.377308263
22.5947381	0.503202111	-0.040555051	3.241079073	0.382103533
27.702345	0.749679417	-0.107940353	3.788836571	0.335089649
33.11571842	0.645976964	-0.064158038	5.035931031	0.280218034
65.40337491	0.628875371	-0.060900882	4.666160451	0.325260941
20.93174841	0.904841992	-0.24478074	3.096193499	0.374475344
22.92222847	0.420904363	-0.029231721	2.778314336	0.401541146
33.16653156	0.460546893	-0.036694712	2.444860811	0.451559465
24.32812445	0.590800303	-0.060662324	3.059212042	0.370482518
28.42919991	0.499427993	-0.038252674	3.571476045	0.340027704
25.12852843	0.838712205	-0.187176491	2.839522676	0.394995956
25.54264613	0.947823899	-0.32394428	4.319083102	0.321582317
48.80506291	0.537642267	-0.047250887	3.162671778	0.404242145
34.16767106	0.761436008	-0.107917173	4.364088159	0.321362525
28.78593666	0.714078776	-0.097571038	3.255097398	0.402739055
26.78391006	0.485696853	-0.038613006	2.915820565	0.410704167
30.93850112	0.408754319	-0.02753499	2.634215704	0.426741382
28.27782189	0.926473056	-0.232941925	5.131986279	0.284259987
35.52737795	0.614061428	-0.065530612	3.201171973	0.381200037
22.53077573	0.924514551	-0.263400248	3.899157365	0.325825014
19.29139404	0.693589708	-0.09719204	2.732070105	0.395829938
20.38206305	0.716758955	-0.103113222	3.033237561	0.416234557

log.sigma.1.0.mm.3D_glcm_ClusterTendency	log.sigma.1.0.mm.3D_firstorder_InterquartileRange	log.sigma.1.0.mm.3D_firstorder_Skewness	log.sigma.1.0.mm.3D_firstorder_Uniformity
26.88040418	121.069849	0.236246115	0.085479379
15.99653613	51.51155281	0.308101321	0.150521949
25.39053805	99.20015335	0.898201243	0.093886046
34.88344422	118.257618	0.235568794	0.075957642
15.77590834	87.11909866	-0.031750379	0.118209821
16.02424893	91.67256546	-0.03941447	0.104837598
14.87925717	59.65102577	-0.973380988	0.143181678
29.09175731	127.7588282	-0.08258856	0.078184947
23.20528915	106.6480694	0.037091869	0.088819434
19.89818286	80.31727695	0.34882191	0.110982699
14.14326454	91.70326996	-0.069731754	0.107960037
25.63652832	95.90919399	0.00209243	0.094034396
13.64592821	82.08664417	0.264629028	0.115964846
12.91063751	80.90867901	0.122114958	0.117965572
18.91965008	95.76539993	-0.179495756	0.100914424
9.304353388	23.95371914	1.006693436	0.240307886
16.16492433	95.70459938	0.060350331	0.104018029
14.0665746	73.31873322	0.320496959	0.122403564
20.86883637	100.6444912	-0.137083787	0.094407246
9.198327662	44.57583046	0.220625195	0.173649284
13.95353672	61.92035341	0.499150478	0.135141891
17.85241795	89.89701366	0.391334647	0.103695241
11.53041324	70.03728485	0.355582712	0.138445296
25.10031577	106.2642365	-0.176900285	0.088999442
12.76861115	70.8786068	0.678238627	0.124566782
19.17720258	89.9753027	-0.018250536	0.097779236
13.18286556	52.96883345	0.190659693	0.150379967
17.01439587	69.99102592	0.603218493	0.11941968
23.84475719	109.5201035	-0.085762777	0.087719828
13.9116651	50.36243749	0.545527269	0.155096967
14.51670342	63.89605999	0.757179272	0.13174051
9.259418168	53.86926556	0.53772422	0.164393756
28.49455259	109.4179459	0.098109728	0.085112854
31.84956128	93.57752514	-0.259359278	0.095968935
29.71563756	132.6395731	-0.236776365	0.076208448
16.53195945	88.25011253	-0.077018953	0.105007352
24.63056407	91.03408527	0.473529667	0.108431231
18.88452349	93.81795502	0.017820707	0.100260496
14.02543971	82.33216286	-0.242964025	0.114065044
24.00883869	110.5730858	0.090596994	0.087667132
19.22304682	76.66738796	0.163247548	0.114716598
19.93916827	98.23617935	0.010807294	0.095785221
20.99521328	101.5751181	-0.174636311	0.093521759
23.83521149	102.5531883	0.042890816	0.089008923
22.71299102	104.3384066	-0.080488799	0.091269801
14.02507241	73.21897507	0.518145381	0.123268729
28.18280942	110.8664455	-0.055553091	0.082932262
20.35097399	107.6575375	0.046041867	0.091638741
20.04283567	75.25961781	-0.864938481	0.130875473
22.09049456	100.5933037	-0.360070282	0.096875013
17.08048271	78.91108131	0.239771889	0.113401518
20.3550421	104.9014835	-0.146702647	0.092791796
17.99015055	78.9883728	0.105632184	0.11421235
15.28291054	82.80972672	0.105188584	0.110710288
13.81902237	36.58287525	0.412755887	0.188067017
36.10994002	136.1093483	-0.085147047	0.071089937
31.68037547	131.4935913	0.06395464	0.074736201
27.47973932	93.85624695	-0.506132569	0.093864338
19.54322905	77.16687202	0.013477278	0.11329679
12.35105788	77.82530975	-0.227206116	0.123779731
21.13568922	98.86021423	0.059902128	0.095506375
22.75463836	87.83608818	0.349508593	0.105840002
11.63108874	52.31823397	0.109507763	0.16036447
12.12610551	37.62743473	-2.173209618	0.182696574
16.81597386	90.12425995	0.007969377	0.105990783
25.86010483	89.16825104	0.183243405	0.097040437
28.06644985	130.419405	-0.350387617	0.080136051
21.07886049	92.39042282	0.024943191	0.101521416
20.10377639	108.9111023	-0.306181311	0.091717107
26.26692672	96.37868881	0.235830418	0.096536924
17.33835774	82.70676899	0.282825067	0.109735111
16.98285928	91.23344803	-0.058283603	0.104467846
13.81037421	60.29098272	0.438376045	0.141777304
11.98140614	51.86003494	0.420906651	0.154237558
19.12274449	66.37227106	0.126447159	0.124845454
33.75027991	131.909193	-0.18521491	0.073293637
19.2802779	99.22463226	0.109533341	0.096051981
20.7217229	118.4742804	0.082134725	0.087890352
19.14744152	103.3343353	-0.014729685	0.095729789
20.84041442	94.32499313	0.342820283	0.101438124
27.04070214	122.004735	-0.005857091	0.080892542
40.79535296	157.3333359	-0.190966313	0.064544418
41.01782565	101.7827711	-0.153373318	0.086476096
21.53280802	78.2519455	-0.457955009	0.11705113
15.21729103	81.32749748	0.208463393	0.116215917
12.8021449	58.12575626	0.508937879	0.146365894
18.02816017	102.3969498	0.076747697	0.096460504
24.56713867	111.3451347	-0.294580802	0.086282914
14.29385951	74.56673622	-0.458663329	0.118934576
31.93531553	85.06760025	-1.183620403	0.117823129
21.51773358	75.38916397	-0.594560917	0.114900708
32.77558513	126.5245619	0.292091098	0.076595591
21.40286486	84.65668201	0.126210641	0.107760387
17.37841002	75.72820568	0.271076573	0.115618032
14.42335493	65.94449568	0.343392099	0.131444018
48.78391786	165.0420609	0.124407806	0.062100642
21.21461937	86.39519978	-0.926168702	0.106510236
27.84519533	99.28464699	-0.409467824	0.09220702
15.81490908	79.64340973	-0.097312054	0.117856322
21.68834703	74.13803864	0.832115804	0.108380973

log.sigma.1.0.mm.3D_firstorder_MeanAbsoluteDeviation	log.sigma.1.0.mm.3D_firstorder_Energy	log.sigma.1.0.mm.3D_firstorder_RobustMeanAbsoluteDeviation	log.sigma.1.0.mm.3D_firstorder_Median
68.7847252	5569046.681	46.74277807	-3.515545964
45.52821453	9210302.866	25.15116946	-2.59798193
67.01476029	3533801.9	41.9124698	-13.42527199
77.20806362	27421695.67	50.0367126	-0.903671086
51.14772274	1552448.084	35.16598885	7.865713596
55.51774637	3091643.881	38.99972274	0.171034127
45.25787449	1318108.607	27.13293125	7.170198441
72.7238145	8083604.532	51.95357933	2.216866255
63.90487089	13730876.02	43.8881072	-3.333771944
55.46593027	18168701.54	33.96032515	-7.105432749
52.36117623	3465468.081	37.42237402	-2.851959705
67.08510268	12786015.29	41.36249328	-6.567647696
49.46161684	8304079.847	33.81655243	-0.089673281
48.18261513	4034244.436	33.80775323	-1.2708354
57.61651806	4968557.782	39.45162321	3.192606211
29.41194206	16746249.53	12.79709967	-1.973119497
54.38470077	3160808.005	38.88602105	-0.124927361
47.01636841	8874184.143	30.82829008	-1.822628796
60.09988614	3278263.004	41.96355296	11.46801138
34.86486163	6884736.544	20.3151048	-1.582080245
46.97504068	22652622.56	28.01473206	-7.032027006
55.31867683	8796411.837	37.3807179	-0.886147976
43.79062005	9056041.75	28.71727242	-6.647801876
66.38358918	3549973.191	44.37240349	11.49244499
48.54765312	5221895.126	30.87343763	-12.26765823
59.35489915	4283512.503	38.86967301	-6.363702536
42.82137527	13292426.42	24.36878805	-3.855844259
52.77176808	7684735.382	31.73126955	-12.00756359
64.1280072	11608366.53	44.37245345	4.621932507
43.3749735	10524107.86	23.8972571	-4.743706703
48.08494793	14178600.78	29.21001644	-10.35641098
36.4800904	14477208.89	22.90294519	-2.684626341
68.06631518	16365749.04	44.90854098	0.739631712
68.92363354	888900.8555	42.17445452	18.60025215
76.40172857	2997190.564	53.67652566	15.23779583
53.33189656	9188194.513	36.81853793	-1.842839539
60.80522437	20273555.8	37.36623384	-5.222795486
59.22466508	20480393.66	38.70140374	-4.737778902
49.82491637	3237651.532	35.23278338	9.558925629
64.8279685	7355917.331	44.03976579	-2.024813175
55.04620087	8592712.079	33.14197755	-2.65598619
59.20555759	5259310.212	41.34252068	-1.478240252
60.57857025	9517582.19	42.26072585	1.62615478
63.99630905	6965268.533	43.11239614	2.521220922
63.1604283	12178546.41	43.49281881	1.539634109
48.98306551	9690727.898	31.1027089	-4.725713015
70.48857213	37512795.52	45.87313852	-0.497693107
63.05733078	3621070.545	44.38413352	-4.152022839
51.24206441	647757.9007	30.04498254	17.3940897
61.81763704	3075780.884	42.60966292	12.46114922
52.04775078	16726708.42	33.08457221	-2.17212081
60.98156165	4733345.69	43.73516279	1.117473841
53.58896791	20310958.24	33.77257955	-2.026995897
51.61625684	2598327.093	34.91407925	4.134244919
38.40252538	22322613.15	19.39492894	-1.758311987
80.56798402	9267527.425	54.99880785	-0.547315359
75.4208609	19368113.47	53.55772918	0.733781159
65.21032163	9648727.87	41.68799246	13.02968121
55.84916684	4931673.124	33.60534941	-1.119470477
47.72173284	3385367.286	32.44440048	-7.232517719
59.91813443	12804412.42	40.21212441	-5.224727631
57.56356081	33942420.97	35.80982563	-2.399381995
40.69207558	10282628.54	23.90764969	-2.430134296
35.85354645	8834284.417	19.20649456	-0.760728419
54.17153556	996183.0012	37.39510222	-0.008989785
61.31924002	10700200.03	38.45807443	3.00950098
73.81450928	3213748.093	53.53583918	6.041554689
56.92077491	17874364.58	37.73821479	-1.935112476
62.60960074	2721105.537	44.11493172	-0.664885953
63.07639786	14517925.85	40.22175888	-4.297996521
53.61533191	15026059.86	35.09870285	-6.566716433
53.92148473	5323810.316	37.18506265	-2.148670197
43.89138636	20092587.46	25.89459473	-3.422459483
40.84305812	22655969.53	23.99949802	-5.188551426
51.37718023	49239169.43	29.74410842	-0.283059776
78.20391085	10946601.37	54.29714391	4.574894667
59.0351902	6139146.198	41.38464586	-3.254758835
64.64772851	3856572.689	46.91382583	-7.866613388
59.01750882	9830750.083	41.27138181	-7.77640605
58.5746307	17319984.59	38.16540509	-5.387197733
72.46804452	4383323.456	49.32225682	-8.778933525
88.91663347	16363029.52	63.19410009	6.372058868
72.83067917	28757494.06	44.51458885	2.861073375
53.02821777	776218.2015	32.08099497	13.11985588
50.63213439	14818669.83	33.46733576	-4.689043522
41.87537367	19536948.47	25.08930783	-3.199166656
58.71160479	4201439.258	41.516907	-6.696150303
66.10553559	10390037.17	47.02379952	5.206068993
50.83653205	851288.4135	32.55910042	5.516653061
71.15473238	968715.5474	41.65506834	12.4649992
54.39492346	23259301.79	33.2031347	0.624271333
78.06153639	7599094.91	50.88581784	-8.290821552
57.87144519	6469070.958	35.78421962	-3.110012174
51.81813144	20882193.54	32.19906362	-3.191678166
46.78286699	24146401.73	28.67202846	-5.0368433
95.9201582	3688939.995	66.4255025	-10.44027138
57.23770386	7569262.674	36.27857438	9.455837727
65.75393276	832197.9038	45.1238	21.03912735
49.94088631	1687633.705	32.88412354	10.87495422
59.70123353	5761046.107	34.83100342	-8.140844345

log.sigma.1.0.mm.3D_firstorder_TotalEnergy	log.sigma.1.0.mm.3D_firstorder_Maximum	log.sigma.1.0.mm.3D_firstorder_RootMeanSquared	log.sigma.1.0.mm.3D_firstorder_90Percentile	log.sigma.1.0.mm.3D_firstorder_Minimum
150364260.4	326.4738159	88.81535962	120.6937637	-236.6236267
248678177.4	259.5122375	66.59148617	86.83833466	-287.6069641
95412651.3	338.8181763	90.23525193	116.2835968	-189.839386
740385783.1	421.9231262	102.013311	136.5555603	-401.5019531
41916098.27	257.7886353	65.75992641	71.85844574	-193.7762909
83474384.77	227.1506805	69.34086339	80.92660522	-263.7724609
35588932.38	173.5493164	62.54039897	71.23403778	-295.3369141
218257322.4	283.7646179	89.8190937	113.1858879	-330.4884033
370733652.6	288.2227173	81.26843157	95.83958435	-306.8320313
490554941.7	368.3067322	76.58103626	95.86722183	-351.170166
93567638.18	189.7084808	64.733367	81.5347641	-226.9589539
345222412.9	374.8396301	92.88455568	119.9622475	-473.4546204
224210155.9	333.2625732	63.27634455	80.3700737	-227.7536316
108924599.8	188.7028351	60.07035556	82.42950134	-182.9682922
134151060.1	259.9291382	73.85093258	92.40156555	-295.3594971
452148737.4	377.4447327	49.09758489	54.19236069	-242.6296997
85341816.15	198.3512115	67.29322575	81.72988205	-164.2218323
239602971.9	255.1088257	61.68789418	83.07784805	-243.4195099
88513101.12	225.6975098	75.05167755	102.6247704	-216.8082275
185887886.7	211.0540619	49.57778372	65.63038635	-207.12146
611620809.1	342.3471985	65.8502852	87.26931076	-288.8267822
237503119.6	360.4595032	71.51358562	94.72451019	-322.6191406
244513127.2	213.5836639	57.58486565	79.64279175	-231.0940094
95849276.15	282.9072571	85.90926402	107.8473597	-289.1957397
140991168.4	367.6116638	65.85683106	82.30544662	-196.0982819
115654837.6	225.4608002	78.56340253	97.28634109	-284.5653992
358895513.3	261.0152283	61.92811391	77.09740829	-383.2192993
207487855.3	323.8140259	73.23039278	92.48640137	-305.2008057
313425896.2	249.5481567	81.0985709	110.1845474	-261.9757385
284150912.3	333.0279236	64.34352834	71.70883713	-277.7818604
382822221	374.0120239	66.78384558	84.3072113	-239.9323578
390884639.9	289.9638062	49.410025	66.84995346	-187.588501
441875224	412.6047974	89.7220637	116.1272507	-317.8501892
24000323.1	303.9506531	92.450655	97.97199402	-248.8558502
80924145.24	245.3605347	95.01411697	113.6593216	-270.1303711
248081251.8	289.1698608	67.44339215	85.55717239	-273.1610107
547386006.5	374.0306091	83.90130569	114.3738441	-296.7173462
552970628.8	295.2103882	78.14255748	107.5314423	-489.9978333
87416591.37	192.9204102	62.26898895	80.45292664	-192.5306549
198609767.9	325.1104736	83.73956023	102.3526672	-336.7580566
232003226.1	255.5259552	76.35129159	101.9132454	-291.4646606
142001375.7	224.2409821	74.28787637	90.93886108	-250.3433685
256974719.1	255.5943756	76.13366583	91.92648697	-298.5834961
188062250.4	321.9518127	82.43411875	102.7765411	-271.8645935
328820753.2	339.7957764	80.52856012	91.66882706	-353.7709656
261649653.3	338.9518127	65.95088176	87.01549911	-213.3900757
1012845479	389.7598572	93.77331936	120.4232521	-496.1364136
97768904.71	257.6603088	78.81052407	99.09143677	-293.4429016
17489463.32	189.8556671	69.52707519	70.80809784	-205.6280212
83046083.86	260.5482788	78.66825237	91.29398499	-322.6687012
451621127.3	304.8114014	69.89375368	88.10516357	-307.7781372
127800333.6	286.156311	75.65390625	92.44640198	-252.3696899
548395872.5	362.1268311	72.9082523	94.68926239	-452.410614
70154831.5	225.4571533	66.30605208	91.69522858	-190.7958527
602710555	313.1870117	58.49905186	74.50881348	-253.0995331
250223240.5	312.6438904	102.6803883	119.0152573	-285.99823
522939063.6	356.1984253	93.97759885	119.983638	-349.598999
260515652.5	250.4084473	86.38448859	100.7429626	-341.0227051
133155174.3	393.4310608	77.83679394	92.52784042	-293.821106
91404916.73	180.5582123	61.98904453	74.24197388	-359.7368469
345719135.4	278.2150879	77.73459652	100.0590759	-370.9819336
916445366.2	381.6216736	78.71552454	101.08479	-391.5099487
277630970.7	230.8786621	57.68631989	80.31548386	-342.4170532
238525679.3	222.0974426	56.87544904	67.34324646	-835.5252686
26896941.03	169.4927063	67.75474208	80.87118378	-165.9848938
288905400.7	332.3505554	83.00614034	113.4771469	-302.2910767
86771198.52	204.8514404	90.3145672	107.1534622	-279.6328125
482607843.5	339.5298157	74.77285921	97.40058746	-386.8225098
73469849.5	201.9100189	78.10975286	89.88027573	-293.684906
391983997.9	401.9299316	84.96621099	110.934288	-316.1313171
405703616.2	263.9413452	70.30492763	96.48260345	-292.192627
143742878.5	265.0117188	68.66956808	80.96649475	-276.5820923
542499861.4	348.805481	62.35290346	80.69449539	-296.0680847
611711177.3	460.5213013	58.08125371	72.39007187	-250.2889404
1329457575	364.7881165	73.23758561	91.37892914	-368.916687
295558236.9	430.7009583	99.03929819	113.3289185	-354.9748535
165756947.4	257.6763611	74.1357015	87.12454834	-255.8598938
104127462.6	238.6211395	79.51257957	98.83145523	-215.7497406
265430252.3	237.1099091	74.65244521	88.83230896	-304.702301
467639583.8	381.0135498	78.01108222	98.39716339	-260.2354736
118349733.3	266.0545044	93.25808464	104.9161621	-347.4962158
441801797.1	381.166626	110.4631613	138.7572021	-385.2685547
776452339.7	411.505188	101.4887287	130.2460648	-798.3779907
20957891.44	175.8780823	73.16576369	91.43540039	-235.3846588
400104085.3	300.2729187	66.10597513	90.17012024	-258.2454529
527497608.7	419.2432556	59.73056816	71.67055893	-398.9732971
113438860	228.3544617	73.48678473	94.48077698	-293.1192017
280531003.5	228.1306763	81.82060607	97.09305038	-340.8970337
22984787.16	153.7989807	68.20446653	101.567453	-294.8937683
26155319.78	191.2843933	96.0513527	86.13004608	-319.1911926
628001148.4	299.3508301	76.49442252	94.13664398	-575.2572021
205175562.6	366.1729431	103.7477254	132.1421814	-411.5518799
174664915.9	314.1782227	79.56013466	101.2237915	-327.081665
563819225.5	388.1687317	71.45397674	87.51284637	-319.4147949
651952846.7	421.0682068	64.48930479	84.42108536	-354.4888306
99601379.86	322.7593384	120.7509441	150.9944153	-337.0453491
204370092.2	330.5661316	78.89691118	88.70155334	-415.4638977
22469343.4	172.217926	82.25470574	109.922261	-252.6218872
45566110.03	263.0257874	66.72974883	78.78020172	-217.8908997
155548244.9	355.5436096	84.49167273	106.9049988	-245.7881622

log.sigma.1.0.mm.3D_firstorder_Entropy	log.sigma.1.0.mm.3D_firstorder_StandardDeviation	log.sigma.1.0.mm.3D_firstorder_Range	log.sigma.1.0.mm.3D_firstorder_Variance	log.sigma.1.0.mm.3D_firstorder_10Percentile
3.832806931	88.81514372	563.0974426	7888.129754	-106.8805008
3.277881896	66.59135063	547.1192017	4434.407978	-78.35632324
3.755384336	90.23524222	528.6575623	8142.398938	-104.1279427
4.041073134	101.8584391	823.4250793	10375.14161	-117.1728165
3.359885852	65.70273079	451.5649261	4316.848833	-82.14906921
3.493587084	69.18524072	490.9231415	4786.597533	-94.48829651
3.186195907	62.52631023	468.8862305	3909.539471	-73.23211517
3.879658922	89.81848911	614.2530212	8067.360987	-118.1456451
3.742473229	81.19867445	595.0547485	6593.224732	-106.0687103
3.574028543	76.54534142	719.4768982	5859.189293	-87.71339874
3.410808791	64.62506495	416.6674347	4176.399019	-85.63696899
3.82916261	92.8671249	848.2942505	8624.302887	-103.9575958
3.364935141	63.20247695	561.0162048	3994.553092	-72.80164795
3.309727144	60.02875734	371.6711273	3603.451708	-70.76535034
3.574400068	73.77421289	555.2886353	5442.634488	-85.54203033
2.685242412	49.09063325	620.0744324	2409.890273	-46.37813568
3.452382985	67.27819003	362.5730438	4526.354853	-90.23644791
3.330974288	61.66823819	498.5283356	3802.971602	-73.3247757
3.620348691	74.88990555	442.5057373	5608.497954	-94.35860214
2.956734557	49.56890674	418.1755219	2457.076515	-57.69933701
3.336034805	65.76958888	631.1739807	4325.638821	-78.51089401
3.536488304	71.38948306	683.0786438	5096.458291	-78.98914261
3.199734353	57.572029	444.6776733	3314.538523	-69.28206635
3.785842588	85.82056132	572.1029968	7365.168746	-109.3288193
3.349931409	65.44474907	563.7099457	4283.015181	-82.72473984
3.658422345	78.45015251	510.0261993	6154.426429	-101.5129768
3.220777089	61.92250403	644.2345276	3834.396506	-71.13327408
3.481308591	73.04269493	629.0148315	5335.235283	-87.40528564
3.738612705	80.90199953	511.5238953	6545.133528	-94.95523376
3.227138762	64.145915	610.8097839	4114.698411	-79.98978729
3.34940423	66.63180483	613.9443817	4439.797415	-81.21821136
2.976918054	49.34605208	477.5523071	2435.032856	-54.43654289
3.859361485	89.6936647	730.4549866	8044.953487	-109.7862427
3.73554495	92.24370284	552.8065033	8508.900713	-125.2269264
3.917039614	94.91174646	515.4909058	9008.239617	-114.1382027
3.477670203	67.43478995	562.3308716	4547.450895	-89.59985046
3.670010729	83.89748375	670.7479553	7038.78778	-99.372155
3.643114904	78.13389109	785.2082214	6104.904936	-90.04369431
3.352320338	62.12025682	385.4510651	3858.926307	-76.71163025
3.773371158	83.73381996	661.8685303	7011.352605	-105.6760437
3.563541654	76.3489223	546.9906158	5829.157936	-93.79880905
3.614262859	74.26259695	474.5843506	5514.933305	-96.26398163
3.641806726	76.10507429	554.1778717	5791.982333	-104.3642067
3.751559007	82.42509987	593.8164063	6793.897088	-102.9849655
3.714796011	80.47330042	693.5667419	6475.95208	-108.0118553
3.38507079	65.95085061	552.3418884	4349.514696	-78.09243774
3.919942967	93.72233404	885.8962708	8783.875897	-108.3297195
3.667843436	78.7496437	551.1032104	6201.506382	-98.02556763
3.329351422	69.50200801	395.4836884	4830.529118	-92.55568085
3.620594486	78.63612542	583.21698	6183.640222	-110.4848206
3.491764949	69.89372724	612.5895386	4885.133108	-87.1159462
3.622235989	75.49983626	538.526001	5700.225276	-107.2367233
3.524827014	72.90821074	814.5374451	5315.607194	-91.75318909
3.443302636	66.23917961	416.253006	4387.628915	-83.04242706
3.026228252	58.48196955	566.2865448	3420.140762	-64.55400391
4.058086348	102.6424945	598.6421204	10535.48168	-139.6866974
3.955900565	93.97407198	705.7974243	8831.126204	-119.0723007
3.760386939	86.32726978	591.4311523	7452.397507	-119.3375839
3.57064993	77.81376115	687.2521667	6054.981424	-95.04199295
3.295409562	61.9845682	540.2950592	3842.086695	-71.36431885
3.67011373	77.70836502	649.1970215	6038.589994	-98.47554779
3.632076964	78.69729907	773.1316223	6193.264881	-93.97139969
3.116927194	57.58108427	573.2957153	3315.581266	-60.17010651
2.968848286	56.87268488	1057.622711	3234.502285	-64.00563812
3.452593282	67.75465611	335.4776001	4590.693425	-89.25941315
3.725693403	82.73637244	634.6416321	6845.307324	-92.04859467
3.840720945	90.24785753	484.4842529	8144.675789	-126.9765648
3.603188355	74.77262214	726.3523254	5590.945021	-92.08353424
3.655834756	77.97251959	495.5949249	6079.713812	-109.7754974
3.748898918	84.96363988	718.0612488	7218.820102	-100.800827
3.498561057	70.29787075	556.1339722	4941.790632	-83.62852478
3.498500972	68.59703015	541.593811	4705.552545	-91.4966507
3.26643888	62.35249497	644.8735657	3887.83363	-71.31642761
3.153494622	58.00364686	710.8102417	3364.423049	-71.15678787
3.482354052	73.19724094	733.7048035	5357.836082	-81.89497299
4.008086549	98.99709099	785.6758118	9800.424025	-130.5530701
3.607699293	74.06065582	513.5362549	5484.98074	-98.20083771
3.689638688	79.27854955	454.3708801	6285.088419	-107.247036
3.615309083	74.41133192	541.8122101	5537.046319	-97.24811783
3.643889737	77.97766621	641.2490234	6080.516428	-99.72520447
3.895702639	92.90313153	613.5507202	8630.991848	-118.4428467
4.16705649	110.4342769	766.4351807	12195.72951	-141.0613403
3.959462231	101.3097604	1209.883179	10263.66755	-116.3662811
3.471557115	72.58570654	411.2627411	5268.684794	-83.67915955
3.418134849	66.09242926	558.5183716	4368.209206	-76.55786133
3.192421604	59.73044938	818.2165527	3567.726584	-64.90154266
3.591095107	73.4601017	521.4736633	5396.386542	-89.70581818
3.742429347	81.80282205	569.02771	6691.701695	-114.5950005
3.336057466	67.89501743	448.692749	4609.733392	-82.38104706
3.501469964	95.55308904	510.4755859	9130.392825	-122.0407898
3.551474743	76.49326208	874.6080322	5851.219144	-90.42140503
4.032037051	103.6186114	777.724823	10736.81663	-124.3575935
3.638150054	79.54493159	641.2598877	6327.396141	-101.1320778
3.500779608	71.44655991	707.5835266	5104.610923	-86.11803055
3.33681357	64.48823009	775.5570374	4158.73182	-72.06774902
4.235437211	120.6315019	659.8046875	14551.95926	-146.8494141
3.583448374	78.83895906	746.0300293	6215.581465	-89.10480118
3.65089031	81.92854306	424.8398132	6712.286168	-94.76726074
3.376442588	66.32380241	480.916687	4398.846766	-73.20426941
3.646411415	84.45166803	601.3317719	7132.084234	-100.0917816

log.sigma.1.0.mm.3D_firstorder_Kurtosis	log.sigma.1.0.mm.3D_firstorder_Mean	log.sigma.1.0.mm.3D_glrlm_ShortRunLowGrayLevelEmphasis	log.sigma.1.0.mm.3D_glrlm_GrayLevelVariance
3.069435186	0.195832653	0.020206067	13.5270555
4.736607294	-0.134360297	0.007620716	8.106309927
4.368947031	-0.04187834	0.032547691	13.91897892
3.802342689	5.619075959	0.004172148	17.52880845
3.908531817	2.742095626	0.026845455	7.372841402
3.112019301	-4.643037996	0.010970373	8.005617643
5.330733172	-1.327415894	0.010861762	6.613958275
2.94031132	-0.329554606	0.006918818	13.42334804
3.326635977	-3.36648752	0.007509538	11.13413432
4.8646267	-2.337909517	0.004985316	10.13435756
2.854778124	-3.742964634	0.012728753	6.980844652
4.902205764	-1.799387862	0.003678486	14.83880682
3.98602792	3.056581004	0.010978519	6.783545109
2.920993826	2.235152903	0.018492103	6.161642902
3.790848745	3.36537596	0.010222254	9.125047787
9.408994015	0.826177134	0.009368651	4.708408449
2.694001741	-1.422455006	0.038539558	7.56775065
3.647985865	1.557140268	0.010553916	6.636625244
2.836879304	4.925073532	0.018478097	9.493489289
4.452547047	0.938148656	0.012566987	4.525438268
4.372958956	-3.259024311	0.007360228	7.763201287
3.942252124	4.21125128	0.006389171	8.621444735
3.396297613	-1.21582455	0.010272676	5.859998487
3.392853746	3.902934504	0.011011687	12.31431314
4.813633219	-7.355747194	0.023376487	7.422081369
3.650504305	-4.21684575	0.010354171	10.64604816
5.5400107	-0.833538524	0.00402882	7.025892787
4.612980057	-5.239765678	0.007095486	9.306410817
3.025149521	5.643108559	0.010859887	10.94370009
5.424901906	-5.038970902	0.007510355	7.668517449
5.130326929	-4.503844605	0.011451811	7.804398402
4.636465528	2.513506502	0.015187448	4.365683872
3.846762224	2.25726112	0.008516427	13.58896734
3.892670224	6.182466935	0.02703254	14.22299385
2.839963746	4.409399745	0.01665654	14.9021288
3.125664644	-1.077148866	0.009556227	7.724046627
3.88442404	-0.800821934	0.008836959	12.29883113
3.877829395	1.163766988	0.002717305	10.42566503
2.973814306	4.30124142	0.022641438	6.463350965
3.629516277	-0.980480628	0.006585641	11.92120754
3.953369873	-0.601491224	0.008732056	10.34582763
3.071925298	-1.937851905	0.010759083	9.31300375
3.005062414	-2.086322044	0.00863107	9.663201772
3.444582959	1.219362994	0.012599364	11.3183304
3.640625525	-2.982769724	0.005386964	10.83346656
4.110713745	-0.06410625	0.015040952	7.636445542
4.347844454	3.091848282	0.002887938	14.97408754
3.259725237	-3.097147503	0.009982812	10.46198228
4.185692922	1.866833354	0.03390263	8.148710258
3.473765075	2.24804566	0.00850533	10.07826683
3.957469217	-0.060797247	0.006353932	8.476693818
2.881508479	-4.825790644	0.011593882	9.600176793
4.26427042	-0.077843942	0.002916303	9.313287294
3.255563801	2.977184447	0.02309085	7.502019591
5.376726862	1.41361487	0.008174616	6.259395619
3.017917155	-2.789348525	0.018315859	17.69448068
3.014343964	0.814175677	0.006491638	14.68766561
3.711981522	3.143622228	0.007010233	12.56498362
4.853095569	-1.893427185	0.011023104	10.55790745
5.12091123	-0.744947147	0.005735554	6.683554359
3.541793008	-2.019282517	0.005128954	10.25968194
4.447977824	1.693789174	0.004091579	10.73276195
5.332917546	3.48284894	0.005055365	5.92514737
30.2944552	-0.56073025	0.001197802	6.196066925
2.78773078	0.107935136	0.040494364	7.846443928
3.93555705	6.686703965	0.007147384	11.79034413
2.64448451	-3.470628165	0.012438817	13.36719856
3.853727141	0.18828841	0.004210194	9.522718802
3.060885858	-4.628139972	0.011007089	10.12740036
4.057168963	0.660990939	0.007851748	12.32917406
3.495810718	-0.996100749	0.007965416	8.449977428
3.425140177	-3.155476943	0.008384046	7.9744098
4.87787338	-0.225699751	0.007335597	6.961746662
5.414144664	-3.001496772	0.00855439	6.054431557
5.147736258	2.430609947	0.004809047	9.520942385
3.487649762	-2.891117704	0.006904096	16.36042298
3.260799904	-3.334890847	0.011537766	9.303995179
2.595184805	-6.096055339	0.022242578	10.52990179
3.173582993	-5.995102744	0.007682958	9.450246849
3.947050734	-2.283094562	0.010570389	10.48985567
3.481276689	-8.128868452	0.008472521	14.43141388
3.031762011	2.525964711	0.006524643	20.1399382
5.643082258	6.024492552	0.001314845	17.33538574
3.986035842	9.194791017	0.019650944	9.066334145
3.555216739	1.338186264	0.009209699	7.495475815
7.128067505	0.119118159	0.00375836	6.489294134
3.177805567	-1.980148274	0.009210251	9.117939986
2.870817768	-1.705837817	0.006347006	11.20539426
4.37370978	6.489673528	0.012523513	7.684778374
4.621235877	-9.770851097	0.037671463	15.13952244
6.498709152	0.421347261	0.001955709	10.30347841
3.981613566	-5.174350106	0.005551631	18.12324572
4.096836576	-1.555276841	0.006586854	11.29659361
4.940738646	-1.029499537	0.007023174	8.988579774
5.085503147	0.372306778	0.004411691	7.368447305
2.923983695	-5.36947281	0.012212183	23.92990178
6.689910339	3.023429869	0.005245419	10.76208549
3.043112402	7.317817164	0.01800217	11.38166417
4.500550664	7.349327425	0.021832106	7.576822016
4.962322261	-2.5997166	0.014816731	12.69409723

log.sigma.1.0.mm.3D_glrlm_LowGrayLevelRunEmphasis	log.sigma.1.0.mm.3D_glrlm_GrayLevelNonUniformityNormalized	log.sigma.1.0.mm.3D_glrlm_RunVariance	log.sigma.1.0.mm.3D_glrlm_GrayLevelNonUniformity
0.020914381	0.081391728	0.105460969	52.81288533
0.008412192	0.129387319	0.293286423	225.7412295
0.03384684	0.087790293	0.105920187	35.0358833
0.004370446	0.072626442	0.102831444	176.7738965
0.028069665	0.114468953	0.121074967	37.16168627
0.01156093	0.102050489	0.10773734	59.94871343
0.011346992	0.135139789	0.130366519	41.07136931
0.007209695	0.076797937	0.088186412	71.43533421
0.007925893	0.086323152	0.112372199	163.6826675
0.005337865	0.103198087	0.14773291	286.3376704
0.013446886	0.105866352	0.112338164	79.99344405
0.003868765	0.087136174	0.124072176	117.2443087
0.011718904	0.112224782	0.124520093	210.1959826
0.019897298	0.113499587	0.156867967	112.4131784
0.010653697	0.098440662	0.096984806	82.41314928
0.010825177	0.207204405	0.393174417	1147.129281
0.040420565	0.101875422	0.117732536	64.43684528
0.011518176	0.115872308	0.191298872	235.8422419
0.019382198	0.092633594	0.103185455	49.36000639
0.014069208	0.155364079	0.308383216	361.5827168
0.008048661	0.120139529	0.210870247	543.9125972
0.006802995	0.100715818	0.115744361	157.7166093
0.011295206	0.126003529	0.212122885	298.2850111
0.011414558	0.08604576	0.088200373	38.27995877
0.025002047	0.11541616	0.178501123	122.4370661
0.01093061	0.091672535	0.152439291	56.94557247
0.004459161	0.132101408	0.264309566	386.7129857
0.007609545	0.109430288	0.16050285	139.4068423
0.011386141	0.085682874	0.102005662	139.2293838
0.008334661	0.132433462	0.29676978	281.3158152
0.012373623	0.120393436	0.17339486	338.577438
0.016853679	0.151410488	0.234722796	767.3989521
0.008890372	0.081674634	0.109987036	152.455893
0.027692856	0.093506413	0.06090981	9.196075819
0.017066826	0.0750222	0.070576783	23.45437763
0.010169393	0.102068625	0.123348833	186.5177918
0.009427886	0.096837884	0.176370947	248.37186
0.002892586	0.094632442	0.139564273	286.7945389
0.023750707	0.11171155	0.112003264	84.82358424
0.006907633	0.084507426	0.107787982	81.30892384
0.009378625	0.102503347	0.197517583	132.0302515
0.011367845	0.093495292	0.1077447	81.26597731
0.009090971	0.091624368	0.104683174	137.9291872
0.01318316	0.087083843	0.096861328	81.89984057
0.005659341	0.08905258	0.101191187	153.687182
0.016124707	0.114247407	0.162714377	226.1235993
0.003042425	0.079221927	0.112041037	309.5054739
0.01046101	0.089297046	0.100221851	47.69436887
0.034517225	0.12359589	0.090869266	15.31853871
0.00886672	0.095023687	0.091093057	43.57017336
0.006827987	0.10653777	0.155970429	325.3575063
0.012163416	0.091008429	0.095580493	69.18561346
0.003142341	0.104835337	0.17705368	354.5032669
0.024274272	0.106864378	0.114229305	57.4204492
0.009067277	0.165399546	0.274194395	913.9804819
0.018714175	0.068918501	0.085433089	56.43836193
0.006808101	0.073506364	0.090081388	149.409594
0.007320208	0.090412324	0.09695775	107.5047054
0.01159281	0.102605607	0.174166378	73.92311637
0.006098431	0.117818976	0.14185259	92.6565807
0.005426462	0.091977465	0.11582692	177.7098422
0.004397241	0.098288684	0.160376513	480.563537
0.005552354	0.144581049	0.22625501	385.7229309
0.001312395	0.157351419	0.358073887	348.7031769
0.042327816	0.102204707	0.111907929	20.18363804
0.007550118	0.091988763	0.124976993	129.7024878
0.012810263	0.079204847	0.066598928	29.34392112
0.00449218	0.097573619	0.130525428	281.6196268
0.01144289	0.089860029	0.092454273	36.99465892
0.008255868	0.091260321	0.114749923	167.4509084
0.00852999	0.104262861	0.139196007	284.4553689
0.008882533	0.102178679	0.11211624	104.9965439
0.00801671	0.128444278	0.235452353	574.2181773
0.009431532	0.138650692	0.24580387	795.3768532
0.005197626	0.113288835	0.189124746	911.7712329
0.007120554	0.072094787	0.072221134	75.52359295
0.012124732	0.093759799	0.112641441	95.68176682
0.023258701	0.085827364	0.099089566	48.17080538
0.00815014	0.092793917	0.125283324	148.0166887
0.011222893	0.094900061	0.145541609	242.9335551
0.008788548	0.078683426	0.090460412	36.82814949
0.006688319	0.063660781	0.062011937	80.85106453
0.00137012	0.081945339	0.105327348	210.7398551
0.020222571	0.112979319	0.089818816	15.17232966
0.009830512	0.109963409	0.137791266	335.5936401
0.004178064	0.133297146	0.245815473	620.236134
0.009666667	0.094198251	0.099387587	67.31967629
0.006652873	0.084785774	0.089295608	121.7975654
0.013009013	0.113721895	0.117802623	18.89342108
0.037960986	0.114139881	0.061540811	11.30500363
0.002097532	0.105164318	0.186163189	369.0101901
0.005752177	0.073260051	0.098998754	47.87671496
0.007017127	0.096379823	0.183373393	86.97590852
0.007550882	0.107353615	0.17583559	387.3624031
0.004813625	0.120499812	0.187585562	613.313258
0.012476298	0.060627009	0.060335105	14.50975099
0.00551354	0.101069151	0.142502988	110.2310656
0.018472266	0.090270216	0.067556399	10.40998512
0.02266546	0.113905846	0.113064477	39.08481615
0.015773025	0.097920232	0.173883927	69.58703977

log.sigma.1.0.mm.3D_glrlm_LongRunEmphasis	log.sigma.1.0.mm.3D_glrlm_ShortRunHighGrayLevelEmphasis	log.sigma.1.0.mm.3D_glrlm_RunLengthNonUniformity	log.sigma.1.0.mm.3D_glrlm_ShortRunEmphasis
1.289710372	117.7281695	555.2457346	0.94043204
1.714479837	147.7791799	1303.206508	0.886943782
1.290097745	83.83074612	342.0177837	0.940752026
1.275611203	315.2897275	2105.91048	0.944516423
1.346330455	75.57958512	268.2031296	0.926626058
1.30712432	128.2231761	495.3541365	0.934668492
1.366465702	147.6703237	250.2024398	0.9249406
1.249461022	212.2477321	809.1702599	0.946614521
1.315187744	178.0842147	1597.198854	0.934393869
1.3957635	230.81161	2273.293058	0.92343706
1.312135509	107.6436487	639.3467569	0.935794726
1.338835683	367.8779361	1128.546973	0.932353437
1.351833768	111.858586	1547.689882	0.926947076
1.434459028	74.17081019	794.1529484	0.914427486
1.281575558	159.763198	712.3804491	0.938486296
1.97927155	100.349712	3759.939534	0.849950232
1.338441073	59.102704	525.3877239	0.928443756
1.507780411	108.6573601	1596.500909	0.906127785
1.297449036	97.76358397	451.0740381	0.936107117
1.767575263	85.36855214	1698.905933	0.877310974
1.543941346	147.4686955	3527.265146	0.903842035
1.325142963	184.3734128	1314.893371	0.932637071
1.548730898	105.7868422	1842.761444	0.902631856
1.258405517	162.1460225	382.8142223	0.942600754
1.469125649	69.87476908	847.4682038	0.913478131
1.402440146	152.7466784	509.5288873	0.923718759
1.667816352	248.7254958	2191.104932	0.888269231
1.429350853	173.2018007	1031.120298	0.918509101
1.28615456	140.0493132	1397.343873	0.940973413
1.733346972	141.3724136	1567.932608	0.88261639
1.454064004	107.0112064	2262.354453	0.916053398
1.610573689	71.34125284	3843.550348	0.89262217
1.297141271	187.58576	1597.014279	0.940206179
1.182061522	126.2225572	88.18090128	0.958085143
1.199392359	145.2722067	279.3193953	0.956817993
1.346136622	129.587183	1516.104681	0.928502407
1.439539216	156.939721	2102.520967	0.92314346
1.365153828	404.5628586	2526.497385	0.930252449
1.323610861	75.99148478	635.1562551	0.931193315
1.297545844	208.3048849	820.2623919	0.938873039
1.509780463	152.6894449	1019.167672	0.909463629
1.310840698	131.5095135	730.2823317	0.933394097
1.296582714	154.0495739	1280.717355	0.937651271
1.286241224	136.5785987	797.4427923	0.936702755
1.286302426	231.7322232	1472.543116	0.939288016
1.433650028	91.21415578	1599.298444	0.917633196
1.304883785	414.4009979	3321.922591	0.938030762
1.293034582	154.6436826	452.0901683	0.936094286
1.260864485	92.44807253	107.0278174	0.943795801
1.267259927	182.6980218	392.7164453	0.940802048
1.414377203	176.7644946	2485.415893	0.920810468
1.280933367	129.0426925	647.4949694	0.938286888
1.455428806	358.354018	2729.707585	0.917408441
1.3257642	76.93203618	449.3388914	0.931446516
1.676812481	126.1989213	4166.426066	0.890171648
1.238846467	162.635239	717.4509386	0.949282491
1.256328064	215.3576662	1762.411695	0.945061666
1.280453719	212.5784855	1013.764706	0.939041257
1.452498295	152.0671705	582.3110868	0.917772104
1.399725277	227.6718815	636.7715774	0.918799676
1.319560677	232.9398197	1628.840421	0.934603138
1.417665768	264.8013486	3994.464662	0.922164486
1.577005227	200.6729714	2064.408888	0.900345734
1.882337291	1034.225157	1562.097495	0.864419521
1.321121754	60.34514391	165.5467364	0.932228319
1.340858147	189.4399305	1180.766454	0.931806576
1.198750051	158.6532614	328.6903224	0.954293338
1.358489316	262.8322077	2393.734243	0.928183474
1.266435631	152.1911553	353.910508	0.942233892
1.317468629	184.7605056	1549.588585	0.935182784
1.383738186	153.2202177	2237.298221	0.923494189
1.320711907	151.1060509	859.8697047	0.931837857
1.578155264	149.102093	3484.480459	0.90339453
1.619042915	122.6457807	4365.406132	0.894593729
1.491417698	231.2686666	6374.262856	0.910388625
1.208288411	241.0184827	928.3811211	0.953836796
1.312266923	130.4466963	863.3552827	0.935784683
1.282712229	92.19273457	479.7340239	0.939654748
1.351468811	173.9341249	1324.360397	0.928181373
1.382951177	131.2870617	2118.737219	0.92721683
1.252562474	206.0718269	408.3989762	0.947156021
1.177446531	284.4534922	1144.990114	0.960491177
1.285689704	1028.746115	2212.963506	0.942257512
1.258460692	121.1546467	116.2391109	0.944361736
1.374395788	131.9323682	2518.677743	0.92607004
1.632510724	250.0581917	3497.918132	0.89014191
1.285657796	154.6978304	608.5477201	0.938484843
1.257383889	207.941446	1240.132113	0.943840335
1.344079198	159.4574479	139.4028707	0.92903385
1.189048822	178.951866	88.25449499	0.955392065
1.471336002	560.8681096	2824.317057	0.916312042
1.267359767	302.2793058	568.1189751	0.946120042
1.467788014	203.1866129	726.6667243	0.916426057
1.462824453	174.8375349	2885.814644	0.913927636
1.490375336	228.7078329	4029.660807	0.910154093
1.179193081	219.7563911	214.9462689	0.958790891
1.386718554	295.8413155	894.7117279	0.924028329
1.206899826	143.0269251	101.5606231	0.95126759
1.33502316	96.20000625	283.2085057	0.926450598
1.465449925	112.400784	566.5136197	0.912675449

log.sigma.1.0.mm.3D_glrlm_LongRunHighGrayLevelEmphasis	log.sigma.1.0.mm.3D_glrlm_RunPercentage	log.sigma.1.0.mm.3D_glrlm_LongRunLowGrayLevelEmphasis	log.sigma.1.0.mm.3D_glrlm_RunEntropy
155.9037469	0.919045544	0.024314524	4.265188987
274.0590451	0.839672605	0.013352991	4.09589257
105.5727922	0.919354839	0.039979721	4.180101361
417.3951781	0.923660779	0.005349406	4.470022889
108.6884137	0.904221127	0.033927817	3.840033281
175.3039621	0.9135064	0.014339662	3.949025967
222.5669645	0.900935859	0.013665337	3.693963809
277.5490457	0.928297252	0.008559075	4.271458395
247.203706	0.912013912	0.009913198	4.220840339
339.9319242	0.895515717	0.007165761	4.141537318
147.505113	0.913589434	0.016923029	3.86260765
520.3285558	0.907816879	0.004816951	4.334836621
159.1076655	0.903048735	0.015294225	3.886118292
111.9279339	0.885922664	0.027033409	3.88696923
214.7028908	0.918939458	0.01262315	4.013047588
221.1917009	0.796104572	0.02032558	3.719020752
82.53502407	0.906105356	0.049191172	3.934071081
173.8925944	0.872608524	0.016735377	3.981388639
132.7156827	0.91554322	0.023496421	4.055077688
164.0718573	0.830252932	0.023383566	3.799270985
242.5028702	0.866459536	0.011913182	4.038975885
254.9418002	0.910330948	0.008787641	4.019512787
172.8945621	0.866490156	0.017031048	3.881492997
215.6429482	0.924836079	0.013206447	4.18276593
105.5691656	0.880909788	0.033581356	3.96031183
223.9816967	0.89492352	0.013909656	4.19284054
457.1307823	0.844444938	0.007020554	4.020920792
259.456275	0.888721885	0.010316477	4.070946298
188.1695258	0.920679887	0.013964086	4.16538831
269.3107033	0.83522968	0.013417749	4.070863583
161.0569921	0.884361313	0.017305676	3.968629977
122.2395398	0.854287197	0.026328682	3.719499392
253.3872752	0.918082409	0.010731706	4.311713789
154.1264867	0.945266272	0.030456603	3.982994724
179.2486843	0.941612604	0.018923257	4.219946823
185.6230838	0.904607768	0.013127173	3.985412837
234.6856394	0.890331197	0.012726064	4.261708276
584.4525193	0.903490666	0.003810672	4.167177364
107.8148618	0.90935053	0.028885863	3.825267753
283.1983417	0.9171372	0.00847454	4.21952082
245.5981047	0.873656195	0.012946429	4.220549383
181.0215345	0.912018726	0.014203974	4.078752217
211.6270818	0.916752553	0.011269619	4.092537036
183.6142022	0.917523452	0.015812266	4.195001108
317.768316	0.918898992	0.006932718	4.161781262
135.9235515	0.888102472	0.0218153	3.977334878
569.9625958	0.915756068	0.003800305	4.392616118
208.8684351	0.916083916	0.012680597	4.104505957
125.0661587	0.924799082	0.03730223	3.719970027
246.0032873	0.922457824	0.010494761	4.023603804
266.0748769	0.891826923	0.009293354	4.071561545
174.2057444	0.91917031	0.014760571	4.048720177
562.1417603	0.884907294	0.004386771	4.136946838
106.7065894	0.909019914	0.029782463	3.919985899
228.7688399	0.846153846	0.014548131	3.836280996
209.5389317	0.93156559	0.02055931	4.430902874
280.0710173	0.926795047	0.00827879	4.360844514
290.0421542	0.919566899	0.008732073	4.205023573
234.8489922	0.884898885	0.01470803	4.166868698
339.7628395	0.892429931	0.007887661	3.849846233
324.3239586	0.911750826	0.006883671	4.152037657
398.240259	0.892366669	0.006040013	4.214255857
340.8054469	0.862459547	0.008434231	3.833707976
2242.846522	0.811170887	0.002059266	3.904759265
82.67196663	0.909961007	0.050843489	3.87822292
265.8688735	0.907573431	0.0095592	4.226955795
198.6330869	0.940257712	0.014411568	4.152197853
380.4448896	0.902745362	0.005893316	4.128448744
203.2356271	0.923076923	0.013438761	4.056683867
253.4565382	0.912251846	0.010230002	4.234679275
221.7202631	0.897317814	0.011366721	4.048673091
211.4653739	0.910063364	0.011214904	3.976760101
250.6825696	0.864432008	0.012077642	3.972163821
215.3736855	0.85398818	0.014552734	3.910349275
372.1912319	0.876579521	0.007318293	4.147724484
305.3847357	0.938585608	0.008085732	4.351703312
179.6805967	0.91350458	0.014947257	4.066865988
120.8390354	0.919924338	0.027939132	4.106846748
246.7388613	0.904151404	0.010439248	4.120302907
189.588313	0.899372939	0.014612854	4.186762115
266.2926305	0.928571429	0.010281384	4.268971962
346.6266845	0.947054437	0.007423103	4.47554998
1395.911835	0.920927926	0.001643783	4.410018872
157.2482322	0.925729443	0.022876407	3.823102894
190.609663	0.899848014	0.012979557	3.958798294
449.5566871	0.84958139	0.00659406	3.964355538
206.9600872	0.918528772	0.011819033	4.026745245
274.4864735	0.925555115	0.008051799	4.151570823
228.1129228	0.90752417	0.015355731	3.753938211
221.5559864	0.942857143	0.039197344	3.7746548
894.6523332	0.88259313	0.002897705	4.17669511
396.3252919	0.925582916	0.006739696	4.434859061
316.4994041	0.882808972	0.009409502	4.246777452
273.9558992	0.882076359	0.010349225	4.127331933
363.2040301	0.876493813	0.007008878	3.992278632
264.6124273	0.945880207	0.013606066	4.509109394
444.1730412	0.896824393	0.00688875	4.128772185
180.4507711	0.937460913	0.020438697	3.932625015
137.4338606	0.905216156	0.026496277	3.862244702
171.0191962	0.880373654	0.020736836	4.261643248

log.sigma.1.0.mm.3D_glrlm_HighGrayLevelRunEmphasis	log.sigma.1.0.mm.3D_glrlm_RunLengthNonUniformityNormalized	log.sigma.1.0.mm.3D_glszm_GrayLevelVariance
124.3387122	0.855600232	21.38339921
165.1200487	0.746489586	18.13101569
87.52713358	0.856718128	21.85110299
332.5071873	0.864987903	27.69881208
81.45376338	0.825394333	14.26946582
136.5446015	0.842842383	14.66850321
160.2907756	0.822627369	13.02066116
223.7970899	0.86954824	20.84354273
189.990587	0.84215026	19.89749899
248.5933008	0.819022352	20.99981062
114.4927388	0.845605892	12.13205616
393.3098666	0.838305256	28.19498332
120.0693762	0.825962048	14.09626682
80.47650419	0.800638654	12.30498866
169.6285437	0.850794152	16.93097544
116.5735198	0.678364075	16.20230081
63.21569694	0.829670044	14.11293917
118.9728699	0.783562928	15.24809786
103.9343983	0.846027826	16.57698822
96.28232772	0.728600252	11.18467609
161.8252849	0.778943642	17.25971747
196.4719588	0.838821818	15.37540167
116.0200251	0.777296848	11.31877321
171.8008041	0.860100578	19.68920363
75.45088878	0.798491452	14.64665521
164.0537232	0.81992402	20.00928019
278.6751877	0.748328644	17.3601234
187.0507623	0.808907621	18.33589205
148.3203809	0.858244545	17.40953772
159.027572	0.737863244	18.67477627
115.4914536	0.804011121	16.30718504
79.06662303	0.757185309	11.0886832
198.6808658	0.855347605	24.60316498
131.4532627	0.896071823	21.01856043
151.3328208	0.893087325	22.36128889
139.2177671	0.829390616	15.29298683
168.6007995	0.819412268	22.27371055
433.5686275	0.833576115	19.71563263
81.53621789	0.835609079	12.16825994
221.162012	0.852271755	20.84144612
166.7149471	0.79076349	20.6603797
140.2910963	0.839875046	16.14686348
164.0941617	0.849943406	16.52503976
145.1091198	0.847254672	20.3324543
246.7670705	0.852961099	18.89971309
98.44353977	0.807222587	14.75953326
440.7700984	0.850193389	26.80495101
164.4357467	0.84594428	18.7823937
98.16881909	0.863332624	14.87654321
194.1936816	0.856165681	16.11857074
191.1581476	0.813528936	17.29588256
137.2306068	0.851023567	15.37341938
389.7710932	0.806849785	18.37398637
82.1415275	0.835875843	14.01362544
140.6678158	0.753173096	15.75523409
170.8965403	0.875697702	27.48567651
226.8066668	0.866278163	22.67573064
226.4148542	0.852243759	22.82413979
164.8854486	0.80795414	21.65417928
246.6727003	0.809128891	14.57389822
248.5973601	0.842773601	19.70907014
285.9520097	0.816628778	21.18781009
221.4775149	0.772846956	13.99413712
1195.575695	0.704145544	18.89354195
64.32003718	0.837815086	11.38387755
202.2932727	0.837071472	22.08612043
166.1159988	0.886767451	17.78958367
282.473958	0.829059416	18.61799488
161.3015069	0.859469661	16.56168641
196.4829496	0.844262225	23.20038022
164.7126412	0.819274678	16.33261011
161.6910794	0.836450696	15.76973223
163.7585531	0.778732587	16.21892546
136.2289742	0.760584967	14.95454238
253.1030992	0.791869441	21.10300842
252.6230694	0.885881983	26.33084908
138.8550196	0.845578017	16.53231405
97.25291401	0.854099618	16.7289504
186.3561509	0.829326223	17.25528786
140.6667001	0.827345218	20.42241347
216.5172159	0.871600703	22.61877454
295.8152465	0.901318473	28.17674588
1090.667414	0.860150736	30.21911394
127.6083836	0.865129548	16.74885464
141.7092235	0.824778949	14.8920102
279.7290196	0.75156888	18.3420298
164.1284708	0.851148532	16.79715813
219.9112562	0.862983515	18.00610255
171.2607729	0.8347217	11.7875
187.120323	0.890143844	23.16767533
611.4336462	0.804585093	21.61908445
318.0671578	0.868951509	30.77707403
220.4360607	0.804991473	22.17414538
190.4198148	0.799341767	20.20607923
249.6735301	0.791495082	16.74321573
228.1459965	0.897593022	32.06213569
320.2891443	0.820140596	21.70182278
150.1631224	0.880018746	15.32544379
103.7113814	0.824828896	16.5451
121.652291	0.796943316	25.50984905

log.sigma.1.0.mm.3D_glszm_SmallAreaHighGrayLevelEmphasis	log.sigma.1.0.mm.3D_glszm_GrayLevelNonUniformityNormalized	log.sigma.1.0.mm.3D_glszm_SizeZoneNonUniformityNormalized
98.66300502	0.06069459	0.410426659
126.484338	0.067315949	0.391324291
95.41113021	0.06477721	0.508137327
245.7760489	0.053201248	0.42446609
57.48099217	0.081484835	0.378225441
108.29406	0.078512397	0.443475156
89.64601472	0.090909091	0.369318182
155.5019286	0.061636231	0.425697589
135.0723235	0.062613083	0.387568367
195.2178136	0.067794693	0.433480978
84.53565542	0.084314852	0.377693192
283.7842818	0.059480792	0.429470125
86.49906627	0.078949408	0.356764214
56.24960903	0.087899975	0.381519274
121.172767	0.069359802	0.404197747
106.5357939	0.076799788	0.418819034
48.30726891	0.078782699	0.364854553
87.57550099	0.075881426	0.35889071
63.98678166	0.072151756	0.311904852
69.41433912	0.088446863	0.36277392
132.3538124	0.072819514	0.439870766
148.2238191	0.075379862	0.393291065
89.41666236	0.087638467	0.380627172
115.9188921	0.066084674	0.409114496
66.50394965	0.078871517	0.455556526
136.4311739	0.069839381	0.432575848
205.3817388	0.07257006	0.410571
154.3768462	0.069124683	0.438040475
94.47205338	0.067066965	0.343141246
136.0296523	0.067142648	0.4407795
102.6165986	0.073245435	0.413800696
63.29237757	0.091522399	0.374147911
148.7703257	0.058427461	0.410065695
93.50038766	0.065640561	0.368039837
107.8606568	0.0624	0.437511111
95.22008379	0.074526764	0.376035582
137.2749529	0.059697347	0.4104675
303.1059169	0.065216247	0.40188891
47.78254849	0.080986369	0.317787111
165.8175287	0.063327032	0.476710184
127.5553745	0.062544029	0.416105754
96.92660648	0.075936846	0.379118973
106.0660637	0.069677524	0.363390473
107.3456058	0.064673745	0.393943616
157.8359009	0.065150278	0.377719489
84.24997954	0.074610871	0.430572208
308.4736546	0.055075673	0.406857528
119.0931646	0.069137084	0.396172278
69.22451036	0.0781893	0.502743484
136.180797	0.07867329	0.390161833
140.0885376	0.067277041	0.389835642
90.3440859	0.073921035	0.362805931
261.8506345	0.067485395	0.370585687
64.01329111	0.075190935	0.402703575
108.5654029	0.074959351	0.398636604
127.7429378	0.052491627	0.448463757
162.8838786	0.058466694	0.384672322
148.0871648	0.0596827	0.39993361
123.3291354	0.062357448	0.468284611
180.4800023	0.081852122	0.418513108
175.0412339	0.066358506	0.386750873
213.4449876	0.061692216	0.410025938
161.179928	0.081840807	0.406645076
748.8604083	0.083476892	0.373944033
46.78596619	0.088979592	0.369795918
149.3316045	0.05973511	0.395837913
111.2553683	0.072653061	0.42484898
193.9136766	0.066089598	0.387395836
105.1250572	0.071213008	0.380568443
147.6068499	0.059709904	0.384935022
127.1219268	0.069799723	0.394904253
107.6713099	0.074618182	0.332919008
133.036539	0.074590227	0.40504758
98.90303279	0.076902006	0.379318943
183.2003306	0.062593753	0.41951435
179.6825704	0.05597756	0.441575725
104.7010555	0.069311295	0.4071809
71.60456742	0.071703191	0.361687812
135.0219096	0.069799722	0.394862516
108.9884522	0.063056995	0.382565749
179.3245136	0.061415824	0.487971446
211.8898786	0.052712257	0.453097698
760.1847588	0.053009907	0.415826379
84.96199434	0.076218242	0.286963765
104.5732679	0.075413202	0.398455998
205.3332047	0.074499381	0.41020535
119.2995214	0.073360496	0.387318032
136.7167185	0.068009438	0.340395006
100.8665138	0.094444444	0.333888889
128.7765003	0.077251691	0.470986116
403.1966084	0.064190073	0.403804602
243.5236844	0.052813065	0.455322613
181.0933946	0.060005081	0.503175352
145.3137618	0.065475995	0.426519038
193.9928579	0.074806908	0.417882873
176.877205	0.050898384	0.465777297
214.3731416	0.06730276	0.428730208
98.60805425	0.082100592	0.389792899
72.16336266	0.0792	0.4208
118.7416562	0.060618085	0.523150426

log.sigma.1.0.mm.3D_glszm_SizeZoneNonUniformity	log.sigma.1.0.mm.3D_glszm_GrayLevelNonUniformity	log.sigma.1.0.mm.3D_glszm_LargeAreaEmphasis	log.sigma.1.0.mm.3D_glszm_ZoneVariance
103.8379447	15.35573123	62.3715415	54.58457404
169.443418	29.147806	1106.796767	1083.787796
94.00540541	11.98378378	38.8	33.29653762
442.293666	55.43570058	65.31190019	58.91711735
35.55319149	7.659574468	88.13829787	73.55239928
87.80808081	15.54545455	79.91414141	69.36804918
32.5	8	106.0113636	91.34594525
157.5081081	22.80540541	38.37837838	31.04452885
241.0675241	38.94533762	107.1720257	96.00009564
377.9954128	59.11697248	656.5963303	643.9742814
89.13559322	19.89830508	75.86016949	63.58049052
209.1519507	28.96714579	196.8829569	187.6223705
181.2362205	40.10629921	293.0787402	276.4104873
96.14285714	22.15079365	275.7857143	256.1031116
116.8131488	20.0449827	63.23529412	53.29861951
368.9795687	67.66061294	11437.09989	11374.92102
66.03867403	14.25966851	88.27624309	73.40478007
156.8352403	33.16018307	790.9748284	762.4978295
50.8404908	11.7607362	58.23312883	45.4842862
158.8949772	38.73972603	2195.10274	2154.207007
548.5188452	90.80593424	1754.073777	1736.523926
193.8924949	37.16227181	129.4929006	117.3208695
230.660066	53.10891089	839.3019802	818.9925307
74.04972376	11.96132597	38.21546961	31.1533836
146.2336449	25.31775701	311.1401869	297.0718452
88.67804878	14.31707317	95.46341463	84.00271267
287.810271	50.87161198	1808.524964	1784.078217
184.8530806	29.17061611	240.1398104	228.608797
198.3356401	38.76470588	57.06055363	47.7358778
252.1258741	38.40559441	1231.195804	1211.446171
347.1787843	61.45292014	742.6305125	728.2737296
356.9371069	87.31236897	2966.719078	2928.081256
277.6144756	39.55539143	134.0103397	124.9926057
17.29787234	3.085106383	8.382978723	3.486645541
65.62666667	9.36	12.97333333	8.074488889
179.3689727	35.54926625	277.9706499	260.0371118
370.2416851	53.84700665	455.6274945	445.4328543
387.8227979	62.93367876	461.1129534	449.0328267
62.60406091	15.95431472	84.06598985	66.10044062
175.4293478	23.30434783	71.42663043	63.30103231
171.0194647	25.70559611	323.9270073	311.0649238
105.7741935	21.18637993	103.2437276	91.57623874
175.5175983	33.65424431	82.67908903	71.12190459
124.8801262	20.50157729	64.55205047	54.09692603
225.8762542	38.95986622	66.23076923	56.36822854
264.3713355	45.81107492	457.0716612	443.9044552
598.4874235	81.01631543	126.2637661	117.8533719
71.70718232	12.51381215	58.09392265	47.71911724
27.14814815	4.222222222	27.25925926	21.10150892
61.64556962	12.43037975	46.86708861	36.97248037
342.665529	59.13651877	558.960182	543.7865425
97.59479554	19.88475836	49.02230483	39.57068034
368.362173	67.0804829	744.5583501	729.7815191
67.25149701	12.55688623	113.251497	100.727527
493.5121163	92.7996769	5143.732633	5115.97046
166.3800539	19.47439353	29.90566038	24.29220944
296.5823606	45.07782101	39.45654994	31.36617764
167.1722488	24.94736842	85.78229665	76.21378288
121.2857143	16.15057915	137.7760618	127.8985108
92.90990991	18.17117117	225.2117117	209.4629697
235.9180328	40.47868852	235.1786885	223.1116071
626.5196335	94.26570681	655.5183246	642.6655365
261.4727838	52.62363919	1517.682737	1494.588958
153.6909976	34.30900243	2638.625304	2594.472351
25.88571429	6.228571429	38.9	29.29
194.3564155	29.3299389	132.7107943	122.7066505
74.34857143	12.71428571	18.4	13.33106939
326.9620853	55.77962085	351.8708531	337.5225554
56.70469799	10.61073826	32.73825503	23.77847845
247.5132193	38.39346812	204.8009331	195.0195066
318.6877323	56.3283767	406.3667906	392.1762122
91.55272727	20.52	156.0254545	139.1706975
437.0463392	80.48285449	1963.277108	1940.336649
484.0109718	98.12695925	3326.932602	3299.230034
985.8587234	147.0953191	2105.634043	2090.374215
195.1764706	24.74208145	28.46606335	22.0910096
134.369697	22.87272727	104.930303	93.47310376
73.78431373	14.62745098	51.29411765	42.35284506
194.2723577	34.34146341	150.5487805	137.6939322
314.0864799	51.76979294	343.4177832	331.4011314
100.0341463	12.5902439	32.91707317	26.87267103
288.6232339	33.57770801	14.62637363	10.19458855
424.9745597	54.17612524	129.5714286	122.1081606
14.06122449	3.734693878	19.53061224	10.77384423
362.9934138	68.701427	560.8594951	547.0041028
404.4624746	73.45638945	3075.249493	3044.405326
85.59728507	16.21266968	101.6832579	89.29030937
171.2186879	34.20874751	54.92246521	45.4022347
20.03333333	5.666666667	31.01666667	21.71416667
24.96226415	4.094339623	9.41509434	5.490210039
438.9356026	69.77460902	621.2134315	607.8408523
131.5882353	15.26297578	37.75778547	31.78999294
167.0542169	19.92168675	169.4698795	159.993867
421.4008097	64.6902834	978.7692308	961.6323124
556.2021037	99.56799399	1843.134485	1824.106275
60.08527132	6.565891473	8.612403101	4.765939547
146.625731	23.01754386	158.6432749	146.0012995
20.26923077	4.269230769	14.86538462	9.270340237
42.08	7.92	87.87	73.5059
131.310757	15.21513944	164.2031873	153.8660656

log.sigma.1.0.mm.3D_glszm_ZonePercentage	log.sigma.1.0.mm.3D_glszm_LargeAreaLowGrayLevelEmphasis	log.sigma.1.0.mm.3D_glszm_LargeAreaHighGrayLevelEmphasis	log.sigma.1.0.mm.3D_glszm_HighGrayLevelZoneEmphasis
0.358356941	0.681538383	6477.071146	142.3833992
0.20847376	7.319401461	169207.0208	189.7228637
0.426267281	0.733852666	2412.356757	114.8432432
0.39544592	0.227124675	19421.76488	354.1564299
0.26183844	1.243161968	7642.691489	85.79787234
0.307931571	0.751246504	9286.757576	152.9090909
0.261127596	0.656108829	17940.125	146.3181818
0.369261477	0.216218484	7605.475676	232.0351351
0.299182299	0.694442779	17905.45177	204.1061093
0.281471917	3.004606788	144874.7856	277.440367
0.285368803	0.930515682	6838.050847	129.1567797
0.328609987	0.547756076	71693.74949	414.2731006
0.244937319	3.011773171	29453.36614	135.3996063
0.225402504	4.720060769	17389.34921	89.91269841
0.317233809	0.458016272	9570.048443	179.3391003
0.126817331	108.1767822	1220196.401	148.3348468
0.259312321	2.051580327	4818.895028	72.83425414
0.187392796	7.962697866	80847.12357	139.6956522
0.280068729	0.814737982	5033.822086	112.208589
0.156372724	26.03132779	188204.5388	111.5045662
0.238705972	12.07057468	256977.8011	187.9318364
0.286627907	0.80006861	21794.97972	220.5679513
0.221896741	8.683439604	82267.72937	136.529703
0.376299376	0.247948071	6652.519337	173.6685083
0.266611296	5.475500082	18725.80374	92.75700935
0.295389049	0.705112047	13681.69268	190.9170732
0.202250433	6.933309877	474588.0528	304.7617689
0.29448709	1.492883972	39338.65403	215.8649289
0.327478754	0.495763944	7375.730104	157.532872
0.22501967	8.242352881	185511.6224	185.5
0.263919472	7.741778978	72232.51132	143.2252682
0.160876897	46.84209262	190807.2642	96.5639413
0.333005411	0.791713557	23530.10783	217.6366322
0.451923077	0.133765249	1111.106383	137.3829787
0.451807229	0.126336297	1882.113333	156.7666667
0.236138614	2.429375992	33027.8218	149.21174
0.313194444	3.160420642	66356.12306	195.2749446
0.28771616	1.15343909	185520.486	459.8455959
0.235928144	1.227078185	6915.781726	84.03045685
0.350810296	0.379823329	14108.30435	234.2554348
0.278833107	2.169645281	49368.94647	185.323601
0.292759706	0.908377294	12704.79211	151.4229391
0.294153471	0.554415609	13249.67909	169.3519669
0.309268293	0.586469472	7959.454259	159.9747634
0.318423855	0.294103457	15937.39465	247.9816054
0.275583483	5.536258886	38565.81433	118.2752443
0.344819503	0.311615798	52084.07614	459.874915
0.310463122	0.445713332	8526.370166	182.9337017
0.402985075	0.320388917	2905.092593	93.88888889
0.317907445	0.264050375	9484.905063	200.2531646
0.25671729	3.228428667	97964.54835	208.7042093
0.325272068	0.434932836	6186.126394	144.267658
0.260141324	2.024808611	275174.8159	407.6619718
0.282571912	1.767992263	8022.107784	93.83233533
0.189789974	40.20686644	663221.9006	159.8521809
0.422070535	0.241086332	4592.932615	179.3530997
0.351573187	0.256107332	7026.237354	247.5590143
0.323279196	0.396647436	19534.20813	225.3755981
0.318181818	0.935142298	21008.50193	175.5482625
0.251986379	1.057738536	48860.03604	267.1261261
0.287871638	1.082583641	52009.87213	268.1590164
0.278933917	2.522815973	171422.7533	311.7028796
0.208090615	7.630669655	303379.2364	244.6749611
0.150494324	2.232879966	3125005.555	1196.6691
0.322580645	0.977139821	2183.928571	74.7
0.316162267	0.763805955	23764.2444	222.9633401
0.444162437	0.142509039	3089.937143	166.9085714
0.263997498	1.377826257	91031.38033	295.8957346
0.334080717	0.25222407	4955.107383	165.9261745
0.319741422	1.223319434	34932.26905	219.6360809
0.265460526	2.960886128	57009.24164	189.7100372
0.243578388	1.182040913	21716.45091	178.4945455
0.20878483	13.52598821	287264.4699	194.0296571
0.189994044	26.85757045	415336.4992	153.3487461
0.255991285	8.983928746	496667.2894	269.7489362
0.396057348	0.134908678	6786.830317	256.6561086
0.295434199	0.952020876	12600.25455	152.5727273
0.33442623	0.851261086	3734.122549	112.0784314
0.278911565	0.991135477	24233.16667	203.2560976
0.288475053	2.876193916	41767.80146	160.5030451
0.406746032	0.197212897	6161.365854	238.7804878
0.475018643	0.068234205	3846.094192	303.2574568
0.366045845	0.125666764	135274.3043	1117.089041
0.337931034	0.212819178	2338.918367	141.3673469
0.268652315	4.752324531	67013.42261	157.053787
0.180058437	11.70820133	813642.1034	302.6713996
0.284061697	0.786692159	14106.69231	182.8687783
0.324097938	0.302903181	10892.18688	227.1908549
0.327868852	0.221879048	5109.783333	174.35
0.504761905	0.114152123	1795.207547	183.4528302
0.273459119	1.04270412	371722.5097	616.3597056
0.409348442	0.137938288	11068.6263	341.9515571
0.324853229	0.843550435	34766.33434	238.6716867
0.241564792	5.674278191	170865.1883	210.9423077
0.229245608	8.386984124	407431.6251	283.4800902
0.509881423	0.064976904	1755.937984	243.5503876
0.28125	0.496648437	52669.20175	317.0994152
0.422764228	0.136101033	2387.75	148.4615385
0.263852243	0.95881885	9055.52	108.13
0.311028501	1.601984563	17632.7012	150.2191235

log.sigma.1.0.mm.3D_glszm_SmallAreaEmphasis	log.sigma.1.0.mm.3D_glszm_LowGrayLevelZoneEmphasis	log.sigma.1.0.mm.3D_glszm_ZoneEntropy	log.sigma.1.0.mm.3D_glszm_SmallAreaLowGrayLevelEmphasis
0.664401952	0.033606385	5.708670443	0.028456787
0.650103333	0.011876681	5.840655301	0.009127743
0.739845663	0.044247359	5.254067866	0.035699759
0.677252118	0.005371176	6.174677185	0.004146822
0.626711906	0.052359967	5.050993529	0.041094033
0.691987348	0.016281371	5.30162796	0.013396419
0.628579446	0.022620825	5.186078068	0.019558255
0.677511102	0.009838845	5.763264838	0.007979848
0.647071014	0.010840373	6.004455773	0.008288274
0.684701862	0.006670265	5.816870823	0.005052866
0.638911567	0.018323821	5.51496311	0.014372838
0.681051797	0.005676092	5.940379919	0.004718773
0.618028944	0.015734604	5.78951887	0.011523786
0.640819251	0.030346211	5.389975399	0.024176673
0.660879273	0.017650391	5.701864066	0.015099712
0.672843097	0.015251446	5.727320639	0.011098226
0.627336042	0.075593455	5.481352735	0.062900192
0.621929441	0.015621755	5.80481871	0.011400194
0.577688078	0.031490044	5.677574933	0.024409847
0.624352737	0.021319193	5.588101864	0.016300163
0.689680889	0.009946276	5.720343199	0.007622672
0.650887718	0.008408944	5.746972014	0.006224094
0.640496221	0.013321048	5.584555765	0.009504935
0.661554461	0.017504438	5.52071222	0.014623336
0.701342697	0.037793645	5.313907372	0.030745152
0.682672406	0.017122386	5.527200917	0.014372411
0.666185056	0.006205856	5.848574746	0.004872808
0.687785204	0.009993525	5.591460737	0.007907753
0.604248482	0.015429602	5.993040021	0.011542946
0.690461168	0.010824867	5.67724823	0.008224381
0.66889383	0.014965294	5.74955441	0.01098168
0.635658891	0.022042967	5.705898286	0.015613455
0.665635061	0.01323306	6.046984093	0.010866292
0.625218167	0.040454954	5.096934064	0.031756218
0.685550577	0.025309511	5.44867556	0.021757067
0.637638061	0.014173585	5.770687393	0.010676614
0.666372348	0.011459796	6.032589967	0.007822824
0.658808895	0.003694649	5.910504602	0.002844715
0.584429704	0.044114569	5.688715794	0.03657335
0.716792138	0.00940744	5.621126365	0.007886601
0.670541432	0.013392218	5.803377361	0.01042265
0.638590913	0.016073552	5.659438758	0.012495595
0.62623491	0.01238506	5.945291745	0.009193944
0.652641776	0.020856479	5.882856136	0.01732308
0.638083789	0.007680697	6.017620041	0.005873275
0.682311494	0.02114005	5.640362508	0.016321886
0.66302173	0.003912296	6.238777071	0.003035307
0.65350457	0.015925767	5.572649469	0.013383987
0.732243029	0.064344	4.759064585	0.060161814
0.64726159	0.013718617	5.331168796	0.011079911
0.648936113	0.008625499	5.934394607	0.006279041
0.62456217	0.016993265	5.717718442	0.013072642
0.632024407	0.004019426	6.046119979	0.002968552
0.657236042	0.038054996	5.335039877	0.030462625
0.655945387	0.011529435	5.835550739	0.00801634
0.695517708	0.031248154	5.844817719	0.028240441
0.643907372	0.008565381	6.147169256	0.006519104
0.65741617	0.011137364	5.94327525	0.008930024
0.711004506	0.019591009	5.521065153	0.017403288
0.672446467	0.010291823	5.421594748	0.008889967
0.64568844	0.007163545	5.906394749	0.005468702
0.666091069	0.005291314	6.123645454	0.003899938
0.663098145	0.007352144	5.632309216	0.005687277
0.635643127	0.003357386	5.710055007	0.00305094
0.624473591	0.063666448	4.862553953	0.048876346
0.653292166	0.010336767	5.958948074	0.00812342
0.67487897	0.018070074	5.379025542	0.015208256
0.647226724	0.005801087	6.019426871	0.004322168
0.636723131	0.017987992	5.446021496	0.014858277
0.644721241	0.01173315	6.087610358	0.009273906
0.652818395	0.011167272	5.833047495	0.008527222
0.597489137	0.012792775	5.863071716	0.009833264
0.66159722	0.010571589	5.837816039	0.008050197
0.638231623	0.012604272	5.865362704	0.009287939
0.673384075	0.007016525	6.065128811	0.005386177
0.690632994	0.010307468	5.917326021	0.008575868
0.662619259	0.018086833	5.656234324	0.014623936
0.622079769	0.034418231	5.557999978	0.027455089
0.651917613	0.011946814	5.815571885	0.009633675
0.642316464	0.015359954	6.066117758	0.011658407
0.725037731	0.0122416	5.479508106	0.01039727
0.699969953	0.009156853	5.949698887	0.007863586
0.670503978	0.001985408	6.213439849	0.00165973
0.545927306	0.037899375	5.068691732	0.032894172
0.655892936	0.012954737	5.784759791	0.009858688
0.665759569	0.005442025	5.836479078	0.004088914
0.646272662	0.014606928	5.634542388	0.011986894
0.60464706	0.008868806	5.996057894	0.006632649
0.586978251	0.023858054	4.764735179	0.021107739
0.712012906	0.065571475	4.722557954	0.063711095
0.660896161	0.002979951	6.059360949	0.002394534
0.699671571	0.0083036	5.804328365	0.007161452
0.736015152	0.01001655	5.574601055	0.008512094
0.679333295	0.010475669	5.919651088	0.007697606
0.672310931	0.005731917	5.825050418	0.004286648
0.709119294	0.017432494	5.509197221	0.015395861
0.679147499	0.009075745	5.777024993	0.00710995
0.646707439	0.02962621	4.796217603	0.025940024
0.672067143	0.049170183	5.148019751	0.044471913
0.749650195	0.02307652	5.459408188	0.019472486

log.sigma.1.0.mm.3D_ngtdm_Coarseness	log.sigma.1.0.mm.3D_ngtdm_Complexity	log.sigma.1.0.mm.3D_ngtdm_Strength	log.sigma.1.0.mm.3D_ngtdm_Busyness	log.sigma.1.0.mm.3D_ngtdm_Contrast
0.009015266	0.750100065	1.257228276	0.470006909	0.00022163
0.003625087	0.170462879	0.570592867	0.760193497	2.68E-05
0.014831597	1.088045964	2.06015948	0.393410221	0.000394649
0.002659575	0.490690762	0.827698725	0.539204807	3.91E-05
0.015840262	0.638170702	1.685287195	0.372538739	0.000277276
0.009723021	0.549602972	1.216768241	0.388160678	0.000134122
0.018916195	0.684993819	1.988309433	0.198522646	0.000253614
0.006183599	0.655514409	1.100528117	0.429485823	0.00013081
0.003267137	0.294788816	0.635974982	0.762540345	4.05E-05
0.002326392	0.239633625	0.636690214	0.731790663	1.77E-05
0.007356318	0.33170109	0.72794937	0.659883347	0.000109326
0.004882777	0.683576624	1.407873341	0.270915928	5.83E-05
0.003148075	0.206807943	0.628388564	1.058725624	2.54E-05
0.005889115	0.161514319	0.433551651	1.178380317	7.76E-05
0.006855735	0.45830527	1.005026798	0.472955073	0.000104038
0.000957786	0.051512534	0.36376578	2.506589278	2.38E-06
0.009082799	0.23980817	0.558979586	1.061268255	0.000200487
0.003132822	0.137338396	0.45249473	1.132676438	2.16E-05
0.01121851	0.536215954	1.164186377	0.455154808	0.000202901
0.002669056	0.066640693	0.289676482	1.619268281	1.29E-05
0.001406697	0.108881122	0.334245294	1.574491656	7.94E-06
0.003825199	0.261106747	0.67335558	0.734153332	4.39E-05
0.002567146	0.083855409	0.261369996	1.712139188	2.17E-05
0.0136257	1.147662829	2.061616021	0.220025791	0.000257353
0.005568155	0.285088295	0.838141738	0.901956387	5.86E-05
0.010073574	0.644614413	1.376682043	0.315910469	0.000144663
0.002149808	0.157599401	0.592798483	0.744736684	9.72E-06
0.00501679	0.361544279	0.961911841	0.487754603	4.56E-05
0.003820688	0.235987019	0.468440735	0.998440917	6.90E-05
0.002969052	0.156355181	0.585947977	0.867857344	1.81E-05
0.002259347	0.169596951	0.537511372	1.25580916	1.46E-05
0.00124019	0.04083667	0.206664565	3.352679165	4.72E-06
0.003340784	0.431468769	0.845433462	0.635167285	4.50E-05
0.050065262	4.262913714	6.866007827	0.083586378	0.002014122
0.017214985	1.49786059	2.140849455	0.241056994	0.000631528
0.003258609	0.172894482	0.424989237	1.145948607	3.92E-05
0.002413889	0.235972801	0.517542422	1.03781896	2.95E-05
0.002039213	0.212354409	0.490760725	0.720557712	2.13E-05
0.007281034	0.233043513	0.543192441	0.906788233	0.00012083
0.006315402	0.642344124	1.284725343	0.3712769	9.16E-05
0.005083201	0.317139581	0.75122484	0.556941133	5.61E-05
0.007035733	0.324507436	0.726487907	0.627612867	0.000121136
0.003998594	0.282427957	0.586866886	0.78040482	5.67E-05
0.006540374	0.560754628	1.137982766	0.467212256	9.45E-05
0.003439792	0.411120891	0.869429553	0.543309671	3.87E-05
0.003187527	0.162893106	0.47396723	1.274691271	2.91E-05
0.00162378	0.358498869	0.721732004	0.626821272	1.57E-05
0.01028948	0.698236642	1.33286748	0.379498018	0.00022311
0.043117954	1.476003827	3.631719632	0.127607546	0.000969544
0.011601159	0.80949214	1.564940479	0.281553042	0.000252246
0.002082727	0.163436147	0.438103375	1.073380526	1.59E-05
0.007226412	0.490207145	0.928250847	0.55571293	0.000141864
0.001836331	0.183887023	0.51302222	0.7620586	1.37E-05
0.011070003	0.445584759	1.029146147	0.524332358	0.000162768
0.001058107	0.054663322	0.237881962	2.461088306	5.25E-06
0.007223016	0.865073376	1.223418778	0.426847847	0.000207628
0.002908662	0.460719662	0.781899605	0.686812037	4.57E-05
0.005114836	0.50408127	1.003566299	0.426000874	7.95E-05
0.009035587	0.601451066	1.566621169	0.309700744	0.000103927
0.007318263	0.322075761	1.056105737	0.419455616	8.55E-05
0.003278814	0.288475335	0.654963213	0.640548865	3.55E-05
0.001325349	0.160831232	0.425789358	1.104555762	9.38E-06
0.002316469	0.123785451	0.491259858	0.89624664	1.11E-05
0.002595976	0.191881105	1.604436213	0.309470288	1.04E-05
0.028606326	0.650848962	1.474462231	0.383156615	0.000791139
0.004842957	0.423759551	0.958351158	0.462180431	5.25E-05
0.013305822	1.338629193	1.821885026	0.275688021	0.000478898
0.002154334	0.185740598	0.480059087	0.883934953	2.04E-05
0.013195864	0.903046836	1.654749964	0.282449473	0.000285511
0.003601122	0.348328142	0.773022246	0.643415606	4.42E-05
0.002269698	0.151910534	0.383874943	1.230871733	2.13E-05
0.005702737	0.343782977	0.833676605	0.562116283	6.69E-05
0.001404059	0.093313844	0.326287792	1.612542936	7.21E-06
0.001101669	0.071508851	0.318693342	2.180748336	4.40E-06
0.000827656	0.087128426	0.264382841	1.783012687	4.49E-06
0.005596358	1.059001415	1.652482717	0.314498645	0.000101026
0.005600388	0.409764987	0.842139514	0.612773655	7.96E-05
0.00976026	0.587234863	0.9950484	0.595925978	0.00024779
0.003725422	0.25816645	0.564072354	0.794917007	4.97E-05
0.00241625	0.263704182	0.627756315	1.034661054	2.30E-05
0.012710911	1.245028292	2.156340822	0.212747217	0.000278926
0.004473601	0.882909022	1.172215864	0.42359837	0.000125064
0.002696881	0.530945956	1.161211392	0.252168953	3.04E-05
0.047219584	1.743315266	4.598772999	0.094852652	0.000793137
0.002017296	0.133482905	0.359276399	1.436927132	1.62E-05
0.001328789	0.118205642	0.527071452	1.030345041	4.35E-06
0.007949907	0.590876804	1.251423144	0.400220744	0.000109848
0.004035027	0.322719266	0.616384789	0.702955325	7.57E-05
0.033702853	1.073226161	2.510142001	0.153627415	0.000797119
0.041321995	3.538026788	5.243086653	0.09610513	0.003026463
0.001785309	0.279227905	0.9153224	0.487890686	1.06E-05
0.009614777	1.483417047	2.48930489	0.18115857	0.00018517
0.007152239	0.648857692	1.44170722	0.289039232	7.38E-05
0.001773825	0.174765674	0.502888867	1.068872993	1.12E-05
0.001241726	0.122628145	0.442825433	1.212033232	5.30E-06
0.02302026	3.675591605	4.428255768	0.130868289	0.001054521
0.00572513	0.589169705	1.716472013	0.266924189	5.15E-05
0.042389146	2.127007119	3.611103637	0.142701201	0.001984948
0.016655862	0.744019168	2.066413163	0.263976232	0.000209326
0.009218406	0.733991392	1.864628921	0.342480994	0.000111222

log.sigma.0.5.mm.3D_gldm_GrayLevelVariance	log.sigma.0.5.mm.3D_gldm_HighGrayLevelEmphasis	log.sigma.0.5.mm.3D_gldm_GrayLevelNonUniformityNormalized	log.sigma.0.5.mm.3D_gldm_DependenceEntropy
0.89305949	13.14305949	0.302430001	5.285923144
0.57410727	12.68464131	0.384433077	5.284443385
0.945592389	7.115207373	0.327029667	5.206377537
1.130641165	31.8857685	0.280105427	5.521042325
0.485967676	7.038997214	0.41515041	5.083446432
0.563774492	12.67807154	0.37586498	5.153434
0.517042503	12.73590504	0.419084433	4.776084114
0.878574189	21.24550898	0.300429082	5.435911795
0.74592879	20.65752766	0.332447219	5.382018978
0.697896267	20.54034861	0.363820595	5.317315798
0.489890032	12.43288996	0.397586592	5.033550276
0.974952694	30.86234818	0.318331904	5.430060507
0.499457628	12.87126326	0.40007802	5.165167849
0.443173345	6.720035778	0.413169121	5.248955748
0.609631519	13.00219539	0.366872269	5.093533112
0.412950434	12.42680294	0.444399533	5.078622096
0.52773171	6.706303725	0.380477172	5.350492377
0.481529816	12.77058319	0.405411399	5.134095494
0.644362372	13.3556701	0.348082805	5.314335725
0.373819928	12.48054266	0.448379289	5.100834588
0.55354894	12.33116386	0.402382235	5.155267468
0.603178745	21.2	0.372457409	5.200280301
0.42021243	12.22702307	0.441309693	5.235016005
0.794567797	13.50727651	0.326446549	5.448503813
0.526905746	6.316445183	0.416043973	5.208025936
0.73691543	12.60662824	0.339688063	5.170110065
0.51087258	20.46335834	0.407935766	5.181372633
0.651743447	20.14584787	0.388117492	5.254853492
0.721981237	13.34560907	0.330537602	5.49982211
0.544833887	12.2368214	0.402067918	5.195851888
0.55412619	12.23938345	0.411452813	5.190076893
0.363712253	6.529679595	0.453508299	5.159271754
0.902881598	21.31923266	0.305721701	5.443132533
0.974852071	13.76923077	0.337093195	4.56612707
0.99545471	13.52108434	0.285146611	5.29532188
0.562535781	12.68465347	0.372411528	5.224211326
0.816384549	12.84861111	0.335465856	5.357281745
0.703695496	30.84883721	0.347073369	5.294746247
0.450752626	6.97005988	0.413870702	5.093997363
0.796685935	20.84556721	0.322854123	5.26384972
0.711163561	12.87109905	0.343268854	5.452083177
0.627454694	12.75655824	0.358755529	5.323744036
0.663838327	20.76065773	0.346941803	5.332957257
0.7767605	13.08487805	0.328150387	5.413847871
0.742090752	20.71033014	0.338587263	5.309701953
0.545954016	12.67998205	0.392554448	5.11649831
0.978065808	43.51500234	0.30203548	5.471312966
0.712997479	12.88507719	0.342738365	5.199778726
0.592392515	13.05223881	0.420249499	4.544321636
0.696452356	21.08249497	0.359667057	5.196486016
0.601499039	20.74649533	0.370277631	5.217225931
0.673353521	12.59129383	0.338808561	5.166772129
0.657585551	30.76812353	0.355174688	5.313985754
0.531600631	7.042301184	0.385440376	4.985236623
0.496029886	12.65338035	0.403429824	5.078172726
1.134187805	13.17406143	0.267926501	5.50357309
0.976677232	21.34792522	0.284010456	5.600886226
0.845412474	21.46249033	0.332908654	5.282386257
0.716226781	12.79484029	0.346539973	5.239665658
0.473623385	20.42338252	0.418094957	5.052891225
0.707692219	20.6720151	0.340809828	5.24144522
0.730421703	20.91967871	0.345985889	5.496926813
0.461001561	20.69935275	0.421578743	5.08111803
0.462110911	90.40278286	0.430941999	5.156274461
0.590583788	6.737327189	0.359213404	4.961286788
0.800221742	21.76046362	0.324771572	5.329931984
0.909119019	12.96446701	0.295730372	5.134843376
0.657867102	20.87269315	0.35389635	5.302039554
0.683162742	12.6367713	0.339299001	5.096937439
0.844728929	21.09696668	0.322400096	5.330506285
0.591619525	12.68782895	0.375555532	5.360400819
0.564334585	12.59521701	0.377595731	5.213636549
0.512331782	12.51973684	0.410679272	5.190409692
0.455238213	12.32980941	0.426413406	5.149831862
0.670813742	21.08191721	0.363663287	5.289527748
1.054897162	20.9516129	0.282354415	5.319896516
0.643214775	12.70904208	0.354892125	5.333577411
0.710370868	12.46065574	0.329174953	5.287582463
0.63108832	20.28854875	0.360358467	5.248759248
0.71404232	12.566409	0.347583792	5.379072799
0.928177753	20.36507937	0.301634543	5.435789047
1.303384732	21.8463833	0.247074289	5.482970693
1.148495805	92.50429799	0.282507687	5.460240291
0.590535077	13.8	0.393198573	4.793688955
0.542870563	12.68062518	0.385736056	5.258057941
0.471418511	30.53889701	0.42475353	5.19712714
0.591479372	12.69794344	0.368689739	5.173073099
0.755675666	20.75708763	0.325619088	5.272550425
0.645226791	13.57377049	0.349099704	5.072584999
1.123446712	20.9047619	0.361088435	4.5633354
0.705845497	42.98037736	0.355193861	5.283535379
1.164474476	30.77903683	0.278426919	5.348209957
0.761479352	20.69569472	0.333770551	5.352877037
0.627432643	20.66454768	0.371360047	5.333813671
0.532294	20.57819497	0.402396454	5.090093748
1.574309863	21.27667984	0.225577653	5.466748802
0.710801566	31.36924342	0.371778155	5.216038592
0.727477031	13.69918699	0.330689404	4.883112731
0.513864426	13.27704485	0.420597183	4.955039707
0.846904947	12.96282528	0.333634293	5.43803936

log.sigma.0.5.mm.3D_gldm_DependenceNonUniformity	log.sigma.0.5.mm.3D_gldm_GrayLevelNonUniformity	log.sigma.0.5.mm.3D_gldm_SmallDependenceEmphasis
49.14730878	213.5155807	0.075914433
128.8334136	798.4675012	0.046017824
29.13824885	141.9308756	0.105339503
194.1149905	738.0777989	0.077822511
23.18941504	149.0389972	0.048883343
39.17418351	241.681182	0.06591878
25.89614243	141.231454	0.049994741
68.40319361	301.0299401	0.057308414
136.0976431	691.1577682	0.056910802
182.9632021	1127.116204	0.060010332
54.61305925	328.8041112	0.056008766
96.21322537	471.7678812	0.069625466
137.924783	829.7618129	0.048378004
63.93023256	461.9230769	0.039638454
66.27332602	334.2206367	0.064898676
479.9074421	3087.243558	0.032055278
40.14899713	265.5730659	0.047874471
143.2898799	945.4193825	0.037948451
35.62542955	202.5841924	0.045496068
171.7118886	1255.910389	0.030811752
290.6068147	2102.044793	0.057590194
113.7988372	640.6267442	0.052457989
140.0750641	1205.21677	0.047102086
31.58627859	157.02079	0.075152276
65.93023256	500.9169435	0.062389291
41.38616715	235.7435159	0.070046845
215.5845355	1413.905366	0.042805466
79.76482903	556.1723657	0.066252493
115.2164306	583.3988669	0.064965154
147.8182533	1022.056648	0.048921314
170.9471532	1308.008493	0.06683401
369.7001686	2689.304216	0.036728709
138.9557304	621.5322184	0.065910007
10.21153846	35.05769231	0.08475413
25.25301205	94.6686747	0.099061247
136.8811881	752.2712871	0.048431046
182.9868056	966.1416667	0.067465911
213.0316041	1164.084079	0.056073317
56.04670659	345.5820359	0.046754201
68.07530982	338.6739752	0.076117465
91.70420624	505.9782904	0.056851169
56.11857293	341.8940189	0.06196868
112.8258222	569.6784409	0.052271565
67.33756098	336.3541463	0.059388046
131.7784878	635.8668797	0.062488922
133.4165171	874.6113106	0.065881976
294.03188	1288.483357	0.075329011
35.46998285	199.8164666	0.070977253
11.98507463	56.31343284	0.054796359
34.60160966	178.7545272	0.06992657
233.5706776	1267.830607	0.053866301
57.1668682	280.1946796	0.062640246
248.8958388	1357.122481	0.053052696
42.36379019	227.7952623	0.050601064
500.2098728	2631.572743	0.04242949
66.80432309	235.5073948	0.082888914
149.4304606	622.8349293	0.063849704
90.87780356	430.4508894	0.06905462
53.35380835	282.0835381	0.057359382
49.21566402	368.3416572	0.060579938
132.4148183	722.1760264	0.057771824
351.1569916	1895.310697	0.061603521
212.2647249	1302.678317	0.052772473
178.9831564	1176.9026	0.035164273
16.92626728	77.94930876	0.046325423
119.1867354	504.3702511	0.057590777
33.21319797	116.5177665	0.08381346
208.3956835	1131.406631	0.055764243
29.4529148	151.3273543	0.062566625
139.4604674	648.3465937	0.067517747
178.6914474	1141.688816	0.055447344
71.2976085	426.3055802	0.054239774
309.0178019	2122.39048	0.048477482
398.2980941	2863.792436	0.045020413
653.9152505	3338.428976	0.051113475
90.33512545	315.1075269	0.072087762
69.8343778	396.4145031	0.06140411
38.29836066	200.7967213	0.066305262
100.3276644	635.6723356	0.064327572
168.7385805	989.2234715	0.06767719
31.86904762	152.0238095	0.094978807
117.4280388	331.3266219	0.098128518
243.2313754	788.7614613	0.082536244
11.4	57.0137931	0.04955144
211.4458862	1308.030964	0.056018925
355.9273192	2325.950329	0.043268734
45.15167095	286.840617	0.065648409
108.8608247	505.3608247	0.053258783
13.84153005	63.8852459	0.064782396
11.43809524	37.91428571	0.121995807
279.4724528	1411.895597	0.054885647
50.89518414	196.5694051	0.100868063
65.52446184	341.1135029	0.072646321
253.7144254	1518.862592	0.059480638
347.8629004	2336.313813	0.051855302
22.4229249	57.07114625	0.120298636
79.32730263	452.0822368	0.057493361
11.73170732	40.67479675	0.063325868
27.45382586	159.4063325	0.047517422
49.72366791	269.2428748	0.070545323

log.sigma.0.5.mm.3D_gldm_DependenceNonUniformityNormalized	log.sigma.0.5.mm.3D_gldm_DependenceVariance	log.sigma.0.5.mm.3D_gldm_LargeDependenceEmphasis
0.069613752	18.10973525	76.5694051
0.062028605	19.22788159	111.8117477
0.067138822	28.31914035	89.33179724
0.073667928	14.61474805	68.53548387
0.064594471	18.80205771	107.7075209
0.06092408	20.42895738	104.3094868
0.076843153	13.32368868	85.1958457
0.06826666	17.68393353	82.51696607
0.065463032	17.81335145	95.23472823
0.05905849	21.72667262	104.8553906
0.066037557	17.92100057	107.2200726
0.064921205	20.25160587	87.48717949
0.066501824	18.02236721	114.2401157
0.057182677	23.30214637	140.1663685
0.072747888	14.45539515	83.81448957
0.06908125	15.66324398	108.8759177
0.057520053	23.74284283	120.7851003
0.06144506	19.86621441	127.787307
0.061212078	20.45781226	104.0137457
0.06130378	19.69603935	134.9842913
0.055629176	24.01161605	117.631317
0.066162115	17.87906842	102.4976744
0.051290759	28.12403744	141.5609667
0.065667939	19.92194017	85.09147609
0.054759329	27.51155892	121.717608
0.059634247	22.85966996	100.8789625
0.062199808	19.03216973	116.6803231
0.055662826	27.92408725	113.1995813
0.065278431	18.7275956	88.67025496
0.058150375	22.08970615	119.9299764
0.053773876	27.59560801	119.7713117
0.062344042	19.25852893	131.3541315
0.068350089	16.12902069	81.18002951
0.09818787	8.847263314	46.48076923
0.076063289	15.19444041	61.86144578
0.067762964	17.76023037	109.6732673
0.063537085	20.14684414	88.53055556
0.063515684	18.82153119	94.57483602
0.067121804	16.6525727	110.405988
0.064895434	19.11991901	87.44613918
0.062214523	20.95274583	99.33649932
0.058886226	21.25538285	108.9223505
0.068712437	16.84515334	95.19244823
0.065695181	17.77984152	91.87414634
0.070169589	15.34127802	87.29286475
0.05988174	21.43075159	100.4874327
0.068924491	15.66310247	77.82372246
0.060840451	21.8200236	95.22641509
0.089440855	10.35041212	71.28358209
0.069620945	15.60596577	85.22937626
0.068215735	16.1367283	94.00292056
0.069125596	17.46228629	89.00483676
0.065138927	17.45087935	96.6613452
0.07168154	15.42950232	92.22165821
0.076684022	13.01667278	85.05702897
0.076000368	15.90265337	65.79863481
0.068139745	17.82538687	81.35202918
0.070284458	15.04839133	78.51740139
0.065545219	18.33884298	91.36363636
0.05586341	24.1913907	125.9012486
0.062489296	19.54231886	96.53279849
0.064103138	18.34034082	93.22380431
0.068694086	16.32428022	101.9915858
0.065537589	17.8596689	125.9937752
0.078001232	16.09921638	84.98156682
0.076746127	12.86556274	74.42691565
0.084297457	11.2595532	57.87309645
0.065184762	17.71500006	97.25774163
0.066037926	18.288745	86.59192825
0.069348815	16.80103983	78.50671308
0.058780081	22.20128116	107.2776316
0.063151115	20.62482064	109.9264836
0.059794466	20.4538334	114.9361455
0.059305851	20.84038978	119.9312091
0.071232598	14.62578965	91.18257081
0.080945453	11.63348364	62.91756272
0.062519586	19.88794964	100.308863
0.062784198	21.02042462	93.40983607
0.056875093	23.6320039	113.7097506
0.059289733	22.45247661	102.7582572
0.063232237	21.05152431	84.92460317
0.087567516	11.19497898	50.72259508
0.087117255	10.33429939	54.35315186
0.07862069	13.41070155	81.33103448
0.062355024	19.28846189	105.7384252
0.064997684	18.46827981	127.4945215
0.058035567	22.52048956	106.5089974
0.070142284	16.38904075	84.64304124
0.075636776	14.18435904	70.31147541
0.10893424	11.05705215	47.17142857
0.070307535	15.15079979	89.88301887
0.072089496	19.26053495	72.05949008
0.064113955	19.17109692	86.34050881
0.062032867	19.47232238	107.5383863
0.059914382	20.90300601	117.7657596
0.088628162	12.78092143	49.68774704
0.065236269	18.42977136	103.1726974
0.095379734	9.207350122	55.29268293
0.072437535	15.67899137	106.5567282
0.06161545	21.1223357	93.27757125

log.sigma.0.5.mm.3D_gldm_LargeDependenceLowGrayLevelEmphasis	log.sigma.0.5.mm.3D_gldm_SmallDependenceHighGrayLevelEmphasis	log.sigma.0.5.mm.3D_gldm_LargeDependenceHighGrayLevelEmphasis
7.805870896	1.219791744	835.3215297
10.86471353	0.735892481	1255.077516
21.78336598	1.271109007	412.4884793
2.456815877	2.804983076	2021.553321
19.00634169	0.437390932	733.5793872
9.659999136	1.020209686	1230.348367
6.564000165	0.653683219	1205.115727
4.510179132	1.336928226	1630.037924
5.145002051	1.348353279	1882.743146
6.05575856	1.478824173	1902.464816
10.14165827	0.832437257	1233.422007
3.269952803	2.270259481	2434.487179
10.47012158	0.751765462	1359.191418
27.92628826	0.30678809	828.1583184
7.426818514	0.929738471	1043.090011
10.45005019	0.514219402	1226.160213
23.8930078	0.358601638	737.4942693
12.18714992	0.582805485	1451.522727
9.452751527	0.675915118	1267.78866
12.97051242	0.405270554	1511.961442
12.13628306	0.926129877	1208.299387
5.593754796	1.201699135	1981.262791
14.42076	0.721179217	1468.991578
6.972785285	1.01290208	1169.04158
28.40152293	0.632803222	581.1303987
10.25227225	1.175856468	1075.920749
6.529395629	1.021331643	2184.69071
6.803309741	1.610960587	1941.534543
7.9028305	0.932900064	1119.120113
12.11600401	0.809445713	1271.086546
12.59569052	1.045832872	1194.92073
26.60209186	0.322360689	754.3192243
4.294240214	1.546908086	1654.593212
4.031558412	1.818840966	653.6346154
5.768231258	1.558956081	767.7078313
9.953536166	0.67644303	1330.436139
9.099103871	1.167702962	939.8493056
3.456337793	1.844606122	2683.055754
18.88668496	0.395584754	774.3712575
4.847272413	1.775877266	1673.960915
9.552269524	0.895736857	1140.964722
10.26817477	0.865147573	1274.494229
4.984725554	1.182412106	1943.772838
8.682526143	0.932197136	1098.757073
4.518694861	1.409700591	1807.01065
10.07113635	1.014817307	1071.146768
1.943895036	3.426981539	3226.765823
9.284762722	0.927788932	1064.349914
5.592398425	0.645155902	1021.619403
4.156847422	1.589571324	1872.321932
5.133102103	1.297452864	1820.757593
8.275116217	1.014236877	1071.4474
3.473031655	1.814016723	2796.791154
17.95872438	0.516428917	570.3519459
8.188003836	0.636244964	967.2831519
6.5736969	1.337844224	783.3174061
4.548361178	1.514874358	1591.498404
3.789530412	1.623777898	1765.960557
8.756882595	0.743575206	1051.802211
7.177222853	1.42145848	2298.84109
5.438570634	1.334140952	1807.406324
5.050389406	1.55624177	1833.047828
5.611370354	1.183573073	1944.049515
1.442241276	3.109249307	11132.80044
19.07302867	0.442873995	479.3824885
3.943834771	1.479563048	1515.047006
5.563680203	1.110994244	727.7106599
5.339786301	1.310958069	1877.697842
8.244023418	0.962663438	1005.188341
4.418698896	1.693125438	1485.23819
10.71027814	0.817907846	1164.750329
10.52713193	0.871660515	1256.701506
11.41430031	0.78829092	1243.352167
11.91830828	0.65226431	1291.704437
4.723384233	1.193247161	1873.939869
3.33716001	1.436594144	1309.767025
9.521150652	1.012023793	1170.499552
9.564862022	1.000141577	1005.581967
6.489042079	1.537798978	2087.705782
10.205605	1.08143053	1126.962052
4.967623839	2.703566731	1555.103175
2.793788179	2.133444153	1041.134974
0.612253997	8.040694437	4936.315186
6.34639272	0.676902208	1137.593103
10.20163713	0.82091989	1190.929519
4.526965074	1.434072927	3710.701059
10.55296237	0.924193542	1159.070694
4.512908832	1.141548683	1712.446521
6.165983607	0.761021346	902.7213115
2.244796296	1.82254531	1091.761905
2.214772482	2.298313445	3758.073711
2.739873749	3.704313697	1972.963173
4.854138207	1.651260831	1626.241683
5.880210167	1.371557495	2076.234719
6.714139796	1.256315715	2152.52308
2.994931984	3.124717593	934.6205534
3.297527365	1.683752614	3349.564967
5.103827913	0.877411491	699.2926829
8.54408971	0.641270978	1443.519789
9.029574268	1.385452197	1062.135068

log.sigma.0.5.mm.3D_gldm_SmallDependenceLowGrayLevelEmphasis	log.sigma.0.5.mm.3D_gldm_LowGrayLevelEmphasis	log.sigma.0.5.mm.3D_gldzm_DistanceZoneVariabilityNormalized
0.015610729	0.108212728	0.948753463
0.008154903	0.098963783	0.942906574
0.037373382	0.267930748	1
0.003983405	0.037088411	0.797846944
0.018850653	0.211530486	0.889273356
0.010099675	0.099110938	0.870200108
0.011756054	0.10354517	0.555555556
0.005077194	0.058037157	0.948753463
0.004702778	0.057742155	0.977780583
0.004566734	0.057426158	0.948753463
0.008550745	0.09802331	0.953514739
0.004508005	0.038540336	0.926035503
0.007010272	0.093455874	0.896147808
0.016667037	0.210426605	0.953514739
0.012098036	0.099426759	0.831961591
0.003531918	0.092706535	0.915657439
0.022192187	0.23463865	0.8828125
0.005069339	0.092866757	0.972227733
0.006488369	0.095299255	1
0.004232291	0.091252231	0.938523558
0.007928668	0.099243499	0.91322314
0.004065122	0.053687138	0.862825789
0.005154649	0.094433866	0.966676242
0.019633319	0.105924578	0.698216735
0.020666428	0.236495247	0.971434573
0.012872476	0.110779299	1
0.003068071	0.054960242	0.850618018
0.004703628	0.057173303	0.906111873
0.010610767	0.100796821	0.906766628
0.007485641	0.10039557	0.95791421
0.008415732	0.098934269	0.978725864
0.010964986	0.197157345	0.874851279
0.006342604	0.058201971	0.958781163
0.011580952	0.11930948	1
0.020982114	0.119335676	0.871581451
0.007960205	0.09842258	0.888038549
0.010891793	0.105789442	0.98601468
0.00271544	0.036037713	0.915657439
0.016887574	0.208541251	0.702479339
0.00678708	0.057964391	0.94035503
0.011953183	0.104325155	0.87359954
0.011326512	0.10099452	0.891272189
0.004034399	0.056434375	0.867346939
0.01290396	0.106497959	0.792387543
0.006029747	0.05806739	0.837662974
0.008426724	0.095338179	0.970809328
0.002766585	0.025766218	0.901033854
0.01810647	0.105965314	0.948753463
0.018604855	0.10622927	0.625
0.006932119	0.057193946	0.81450654
0.003690401	0.05486435	0.972419189
0.009138721	0.104934166	0.942906574
0.00243753	0.035860331	0.961305402
0.016847022	0.214975559	0.91322314
0.005030802	0.094172436	0.987578125
0.019758779	0.131108731	0.875555556
0.00539846	0.058637848	0.717519239
0.006542774	0.058308536	0.683396152
0.015110746	0.109546116	0.78125
0.004399668	0.055804011	1
0.004613995	0.056836887	0.9608
0.004872319	0.05643304	0.939453125
0.003972531	0.053473734	0.925485247
0.000829242	0.011668284	1
0.011214366	0.249231951	1
0.004548056	0.054857654	0.869498307
0.024430972	0.119020023	0.698216735
0.004000641	0.055544229	0.955247363
0.012240582	0.106367713	1
0.005564127	0.057565684	0.92737629
0.007831823	0.098307931	0.985075471
0.007904887	0.099263606	0.961553249
0.006455896	0.096816116	0.92866941
0.006331579	0.09571596	0.9561624
0.004470898	0.055841335	0.955945249
0.010382456	0.064180398	0.865123967
0.009014428	0.101575898	0.898596939
0.010498293	0.106461293	0.825182102
0.005410408	0.057564376	0.894139226
0.011411914	0.105195234	0.8828125
0.007597297	0.062426529	0.957466919
0.013637068	0.066283905	0.906766628
0.001354921	0.011730819	0.852385307
0.008684757	0.097886973	0.78125
0.007810081	0.09605045	0.950032873
0.001933822	0.035038819	0.7738
0.011471848	0.099432662	0.96001666
0.004886115	0.057972761	0.837372449
0.012687584	0.095892532	1
0.038770768	0.084875661	0.857988166
0.002434918	0.025628026	0.881429258
0.006296827	0.039709137	0.970156107
0.006820568	0.058060577	0.842449465
0.004657859	0.05592422	0.880670896
0.00386893	0.054595445	0.946195066
0.012945457	0.070610598	0.931122449
0.003685182	0.036784503	1
0.013202411	0.099771906	0.78125
0.01228265	0.095360598	0.889273356
0.010778454	0.10467312	0.961553249

log.sigma.0.5.mm.3D_gldzm_LowIntensityEmphasis	log.sigma.0.5.mm.3D_gldzm_LargeDistanceEmphasis	log.sigma.0.5.mm.3D_gldzm_HighIntensitySmallDistanceEmphasis
0.239177408	1.078947368	18.26973684
0.21877451	1.088235294	17.53308824
0.367745478	1	13.58139535
0.057287645	1.342281879	36.56711409
0.404624183	1.176470588	10.60294118
0.166576227	1.209302326	15.20930233
0.294240741	2	11.33333333
0.10354741	1.078947368	26.63157895
0.086520102	1.033707865	26.61797753
0.085982702	1.078947368	25.66118421
0.162943122	1.071428571	15.69047619
0.07782688	1.115384615	33.02564103
0.161317062	1.164835165	16.06318681
0.523478836	1.071428571	7.547619048
0.206239712	1.277777778	14.25925926
0.134917926	1.132352941	18.06985294
0.567925347	1.1875	7.390625
0.159847418	1.042253521	15.61971831
0.161615226	1	14.62962963
0.170992063	1.095238095	13.42857143
0.15273408	1.136363636	16.39867424
0.082933936	1.222222222	23.12962963
0.123206215	1.050847458	15.84745763
0.318878601	1.555555556	12.2037037
0.400193237	1.043478261	10.60869565
0.210852273	1	18.18181818
0.084941513	1.243902439	23.17073171
0.07589144	1.148148148	25.01851852
0.189362745	1.147058824	14.16911765
0.178984285	1.064516129	17.5188172
0.134309152	1.032258065	16.5672043
0.377511856	1.201219512	9.714939024
0.120830098	1.063157895	23.49736842
0.163179705	1	21.5
0.233917625	1.206896552	15.42241379
0.181848545	1.178571429	14.25595238
0.193646203	1.021126761	19.30457746
0.055138951	1.132352941	31.59742647
0.393518519	1.545454545	8.537878788
0.095838697	1.092307692	24.98846154
0.273964218	1.203389831	14.65677966
0.202879274	1.173076923	14.15865385
0.089362144	1.214285714	24.02678571
0.24664199	1.352941176	16.11764706
0.105201135	1.267326733	21.55445545
0.140007139	1.044444444	16.00740741
0.041502919	1.156626506	43.93072289
0.275921053	1.078947368	13.39473684
0.317951389	1.75	13.0625
0.104783603	1.310344828	23.52586207
0.07234675	1.041958042	26.27622378
0.140776144	1.088235294	20.68382353
0.049766391	1.059210526	35.73190789
0.411073232	1.136363636	10.875
0.142950468	1.01875	16.9140625
0.275997543	1.2	17.75833333
0.104168823	1.510638298	22.53723404
0.100360576	1.591549296	23.75
0.316767149	1.375	13.74375
0.087807329	1	23.57446809
0.086483787	1.06	24.755
0.086908122	1.09375	26.65722656
0.085927684	1.11627907	21.90310078
0.027718377	1	89.09722222
0.215277778	1	10.875
0.091722908	1.210526316	28.09210526
0.330915638	1.555555556	13.23148148
0.083379734	1.06870229	24.08015267
0.205277778	1	17.60869565
0.094251475	1.113207547	25.57311321
0.173310359	1.022556391	14.77443609
0.156830065	1.058823529	17.33823529
0.163523618	1.111111111	17.12433862
0.170519667	1.067264574	14.97309417
0.105291431	1.067605634	23.60704225
0.187775307	1.218181818	19.95454545
0.14999504	1.160714286	18.66964286
0.174202509	1.290322581	15.33870968
0.088732649	1.168224299	23.37149533
0.194142149	1.1875	15.815625
0.085131002	1.065217391	29.2173913
0.172434849	1.147058824	21.49264706
0.018814311	1.240740741	94.84567901
0.224201389	1.375	13.53125
0.15242783	1.076923077	15.375
0.047504114	1.39	29.83875
0.195391156	1.06122449	13.65816327
0.098417355	1.267857143	26.35714286
0.192152778	1	13
0.313440171	1.230769231	15.84615385
0.052102853	1.189873418	40.37816456
0.066623453	1.045454545	36.70075758
0.107672438	1.25862069	21.7887931
0.086651279	1.191176471	23.36519608
0.082346586	1.083003953	24.70158103
0.114993218	1.107142857	26.4375
0.071724634	1	30.51666667
0.206840278	1.375	14.15625
0.275245098	1.176470588	13.30882353
0.159574496	1.058823529	21.76470588

log.sigma.0.5.mm.3D_gldzm_LowIntensityLargeDistanceEmphasis	log.sigma.0.5.mm.3D_gldzm_HighIntensityEmphasis	log.sigma.0.5.mm.3D_gldzm_DistanceZoneVariability	log.sigma.0.5.mm.3D_gldzm_ZonePercentage
0.318124776	18.28947368	36.05263158	0.053824363
0.273921569	17.58823529	64.11764706	0.032739528
0.367745478	13.58139535	43	0.099078341
0.082603019	39.69798658	118.8791946	0.05654649
0.581094771	10.64705882	15.11764706	0.04735376
0.218901809	15.41860465	37.41860465	0.066874028
0.952240741	12.93333333	8.333333333	0.044510386
0.123284252	26.71052632	36.05263158	0.037924152
0.087456432	26.92134831	87.02247191	0.042809043
0.089674969	26.30263158	144.2105263	0.049063912
0.180800265	15.76190476	40.04761905	0.050785973
0.085038419	33.48717949	72.23076923	0.052631579
0.245053325	16.35164835	81.54945055	0.043876567
0.527943122	7.833333333	40.04761905	0.037567084
0.376671811	15.14814815	44.92592593	0.059275521
0.148937533	18.78676471	124.5294118	0.019576796
0.755425347	7.4375	28.25	0.045845272
0.170410798	15.66197183	69.02816901	0.030445969
0.161615226	14.62962963	27	0.046391753
0.194801587	13.52380952	59.12698413	0.022491967
0.172367918	16.92424242	241.0909091	0.050535988
0.104265926	24	69.88888889	0.047093023
0.135918079	15.89830508	114.0677966	0.043207616
0.599742798	13.48148148	18.85185185	0.056133056
0.402910628	10.7826087	67.02898551	0.05730897
0.210852273	18.18181818	44	0.063400576
0.10355221	24.53658537	104.6260163	0.035487594
0.085729403	26.11111111	73.39506173	0.056524773
0.235114379	14.76470588	92.49019608	0.057790368
0.212138407	17.8172043	89.08602151	0.036585366
0.135283477	16.8655914	182.0430108	0.058508965
0.509310637	10.12195122	143.4756098	0.027655987
0.129369303	23.91578947	91.08421053	0.046728972
0.163179705	21.5	10	0.096153846
0.340239464	16.37931034	25.27586207	0.087349398
0.237840608	14.88095238	74.5952381	0.041584158
0.214772963	19.30985915	140.0140845	0.049305556
0.058346484	33.36764706	124.5294118	0.040548599
0.683291246	9.696969697	23.18181818	0.039520958
0.112505364	25.13846154	61.12307692	0.061963775
0.330291902	15.76271186	51.54237288	0.040027137
0.289417735	14.28846154	46.34615385	0.054564533
0.116147858	24.80357143	48.57142857	0.03410475
0.453724823	17	40.41176471	0.049756098
0.131192286	23.84158416	84.6039604	0.053780618
0.15111825	16.05185185	131.0592593	0.06059246
0.046864458	45.81927711	224.3574297	0.058368495
0.295657895	13.47368421	36.05263158	0.065180103
1.067951389	13.25	5	0.059701493
0.145013488	24.79310345	23.62068966	0.058350101
0.077008755	26.37062937	139.0559441	0.041764019
0.229011438	20.70588235	32.05882353	0.041112455
0.053501314	36.17105263	146.1184211	0.039780162
0.547436869	10.90909091	20.09090909	0.037225042
0.161700468	16.91875	158.0125	0.024528591
0.378775321	18.68333333	52.53333333	0.068259386
0.19092387	24.91489362	67.44680851	0.042863657
0.244966306	27.05633803	48.52112676	0.05491106
0.579267149	13.95	31.25	0.049140049
0.087807329	23.57446809	47	0.053348468
0.093150454	24.89	96.08	0.047192072
0.090000985	27.65625	240.5	0.046732384
0.093033627	22.63565891	119.3875969	0.041747573
0.027718377	89.09722222	72	0.026363969
0.215277778	10.875	8	0.036866359
0.1205748	29.31578947	49.56140351	0.036703155
0.886471193	13.37037037	18.85185185	0.068527919
0.089104925	24.38931298	125.1374046	0.040975915
0.205277778	17.60869565	23	0.051569507
0.108193844	26.0754717	98.30188679	0.052710094
0.178949457	14.79699248	131.0150376	0.04375
0.159183007	17.70588235	49.03921569	0.045172719
0.196345487	17.43386243	175.5185185	0.036571207
0.187335811	15.04035874	213.2242152	0.033204288
0.110595278	24.25352113	339.3605634	0.038671024
0.324138943	20.09090909	47.58181818	0.049283154
0.258625992	19.17857143	50.32142857	0.050134288
0.299041219	16.06451613	25.58064516	0.050819672
0.107789804	24.42990654	95.6728972	0.060657596
0.267225482	16.40625	141.25	0.056219255
0.10143535	29.2826087	44.04347826	0.091269841
0.224722431	21.85294118	92.49019608	0.07606264
0.02246894	100.1975309	138.0864198	0.058022923
0.599201389	13.625	6.25	0.055172414
0.159308172	15.80769231	148.2051282	0.046004129
0.059443962	34.635	154.76	0.036523009
0.256615646	13.67346939	47.04081633	0.062982005
0.165381641	26.625	46.89285714	0.036082474
0.192152778	13	12	0.06557377
0.371132479	16.07692308	11.15384615	0.123809524
0.059039239	42.19620253	139.2658228	0.039748428
0.071673958	36.8030303	64.03030303	0.093484419
0.134590102	23.17241379	48.86206897	0.056751468
0.104816878	24.26960784	179.6568627	0.049877751
0.090581106	24.96837945	239.3873518	0.043575611
0.117179806	27.75	26.07142857	0.110671937
0.071724634	30.51666667	60	0.049342105
0.581840278	14.25	6.25	0.06504065
0.282303922	14.41176471	15.11764706	0.044854881
0.174280379	21.82352941	49.03921569	0.063197026

log.sigma.0.5.mm.3D_gldzm_IntensityVariabilityNormalized	log.sigma.0.5.mm.3D_gldzm_LowIntensitySmallDistanceEmphasis	log.sigma.0.5.mm.3D_gldzm_IntensityVariability
0.191135734	0.219440566	7.263157895
0.23399654	0.204987745	15.91176471
0.269875608	0.367745478	11.60465116
0.178325301	0.050958802	26.5704698
0.314878893	0.360506536	5.352941176
0.342347215	0.153494832	14.72093023
0.28	0.129740741	4.2
0.209141274	0.0986132	7.947368421
0.275849009	0.08628602	24.5505618
0.261599723	0.085059636	39.76315789
0.430839002	0.158478836	18.0952381
0.175542406	0.076023996	13.69230769
0.398864871	0.140382996	36.2967033
0.416099773	0.522362765	17.47619048
0.307270233	0.163631687	16.59259259
0.304606401	0.131413024	41.42647059
0.44921875	0.521050347	14.375
0.35766713	0.157206573	25.3943662
0.319615912	0.161615226	8.62962963
0.36558327	0.165039683	23.03174603
0.333161157	0.14782562	87.95454545
0.342173449	0.077600939	27.71604938
0.325050273	0.120028249	38.3559322
0.207133059	0.248662551	5.592592593
0.304767906	0.399513889	21.02898551
0.297520661	0.210852273	13.09090909
0.337960209	0.080288839	41.56910569
0.273281512	0.073431949	22.13580247
0.250096117	0.177924837	25.50980392
0.25586773	0.170695755	23.79569892
0.315932478	0.134065571	58.76344086
0.346966092	0.344562161	56.90243902
0.177617729	0.118695296	16.87368421
0.22	0.163179705	2.2
0.227110583	0.207337165	6.586206897
0.37585034	0.167850529	31.57142857
0.210870859	0.188364512	29.94366197
0.269896194	0.054337068	36.70588235
0.417814509	0.321075337	13.78787879
0.223195266	0.09167203	14.50769231
0.239299052	0.259882298	14.11864407
0.279585799	0.181244658	14.53846154
0.286989796	0.082665715	16.07142857
0.212610534	0.194871282	10.84313725
0.252230174	0.098703347	25.47524752
0.331687243	0.137229361	44.77777778
0.197496815	0.040162534	49.17670683
0.257617729	0.270986842	9.789473684
0.25	0.130451389	2
0.248513674	0.094726132	7.206896552
0.291896914	0.071181249	41.74125874
0.519031142	0.11871732	17.64705882
0.263331025	0.04883266	40.02631579
0.32231405	0.376982323	7.090909091
0.28625	0.138262968	45.8
0.213333333	0.250303099	12.8
0.177229516	0.082480061	16.65957447
0.275143821	0.064209144	19.53521127
0.255	0.251142149	10.2
0.383431417	0.087807329	18.0212766
0.2868	0.08481712	28.68
0.216186523	0.086134907	55.34375
0.320593714	0.084151198	41.35658915
0.305555556	0.027718377	22
0.34375	0.215277778	2.75
0.19975377	0.084509936	11.38596491
0.251028807	0.192026749	6.777777778
0.260649146	0.081948436	34.14503817
0.372400756	0.205277778	8.565217391
0.193663225	0.090765883	20.52830189
0.314263101	0.171900585	41.79699248
0.347943099	0.15624183	17.74509804
0.286694102	0.15531815	54.18518519
0.309859438	0.166315631	69.09865471
0.223312835	0.103965469	79.27605634
0.208595041	0.153684398	11.47272727
0.357142857	0.122837302	20
0.321540062	0.142992832	9.967741935
0.320814045	0.08396836	34.3271028
0.241015625	0.175871315	38.5625
0.253308129	0.081054915	11.65217391
0.178969627	0.159362954	18.25490196
0.14700503	0.017900653	23.81481481
0.25	0.130451389	2
0.347715319	0.150707745	54.24358974
0.32645	0.044519152	65.29
0.346105789	0.180085034	16.95918367
0.442602041	0.081676284	24.78571429
0.25	0.192152778	3
0.218934911	0.299017094	2.846153846
0.21126422	0.050368756	33.37974684
0.173553719	0.065360827	11.45454545
0.223543401	0.100943022	12.96551724
0.285082661	0.082109879	58.15686275
0.308066053	0.080287957	77.94071146
0.160714286	0.11444657	4.5
0.262777778	0.071724634	15.76666667
0.25	0.113090278	2
0.224913495	0.273480392	3.823529412
0.210303729	0.155898026	10.7254902

log.sigma.0.5.mm.3D_gldzm_HighIntensityLargeDistanceEmphasis	log.sigma.0.5.mm.3D_gldzm_SmallDistanceEmphasis	log.sigma.0.5.mm.3D_glcm_SumVariance	log.sigma.0.5.mm.3D_glcm_Homogeneity1
18.36842105	0.980263158	21.4888215	0.627440109
17.80882353	0.977941176	23.34962789	0.696375466
13.58139535	1	7.978671602	0.646607915
52.22147651	0.91442953	72.90938685	0.603817828
10.82352941	0.955882353	9.758619002	0.706135585
16.25581395	0.947674419	23.5678712	0.683656648
19.33333333	0.75	24.08589884	0.675029882
27.02631579	0.980263158	42.90673459	0.631388731
28.13483146	0.991573034	43.63275486	0.665943318
28.86842105	0.980263158	44.95957247	0.678832418
16.04761905	0.982142857	23.97691898	0.698358266
35.33333333	0.971153846	73.01772945	0.638476136
17.50549451	0.958791209	25.23343839	0.704555055
8.976190476	0.982142857	9.753856343	0.734288254
18.7037037	0.930555556	24.27995944	0.658628806
21.65441176	0.966911765	25.83656612	0.725226414
7.625	0.953125	8.729182304	0.700641876
15.83098592	0.98943662	24.5692275	0.722011
14.62962963	1	23.48220641	0.681111222
13.9047619	0.976190476	26.18851823	0.750314586
19.02651515	0.965909091	23.62138733	0.698561392
27.48148148	0.944444444	47.55703035	0.683162365
16.10169492	0.987288136	24.97085907	0.746121391
18.59259259	0.861111111	23.5362626	0.642054647
11.47826087	0.989130435	8.726788622	0.716267101
18.18181818	1	21.6495468	0.672502216
30	0.93902439	48.20025474	0.700514712
30.48148148	0.962962963	44.94973164	0.700060067
17.14705882	0.963235294	23.5294298	0.668632495
19.01075269	0.983870968	23.08269109	0.704790772
18.05913978	0.991935484	23.27359917	0.70763066
11.75	0.949695122	10.2232002	0.751220496
25.58947368	0.984210526	44.08180231	0.633604817
21.5	1	21.42799004	0.602676401
20.20689655	0.948275862	21.17644893	0.607111549
17.38095238	0.955357143	23.5572733	0.692095907
19.33098592	0.99471831	21.62625525	0.654185011
40.44852941	0.966911765	76.56512832	0.662827514
14.33333333	0.863636364	10.19999865	0.698757496
25.73846154	0.976923077	43.58335787	0.64974276
20.18644068	0.949152542	22.81305478	0.664233886
14.80769231	0.956730769	22.5950353	0.686048411
27.91071429	0.946428571	44.75593876	0.665811045
20.52941176	0.911764706	21.9585549	0.655891259
32.99009901	0.933168317	44.50414441	0.652711654
16.22962963	0.988888889	24.27192506	0.698522163
53.37349398	0.960843373	112.1916868	0.624760229
13.78947368	0.980263158	22.35946185	0.663405358
14	0.8125	22.64476298	0.667339899
29.86206897	0.922413793	45.25333164	0.65167379
26.74825175	0.98951049	46.29706074	0.676551972
20.79411765	0.977941176	21.51674158	0.658127993
37.92763158	0.985197368	75.84972538	0.671278587
11.04545455	0.965909091	9.431838766	0.679728006
16.9375	0.9953125	24.18932955	0.697096529
22.38333333	0.95	20.07477358	0.595650714
34.42553191	0.872340426	42.32067972	0.62169738
40.28169014	0.852112676	44.43133918	0.627851569
14.775	0.90625	22.32953163	0.661206241
23.57446809	1	48.65439586	0.723314426
25.43	0.985	43.96538922	0.664130305
31.65234375	0.9765625	45.26001483	0.669513562
25.56589147	0.970930233	49.63825753	0.714165575
89.09722222	1	288.8153622	0.730419983
10.875	1	7.859833015	0.672789106
34.21052632	0.947368421	46.01208752	0.643280714
13.92592593	0.861111111	20.53202203	0.59837316
25.6259542	0.982824427	45.01182683	0.67688207
17.60869565	1	21.86029781	0.654486576
28.08490566	0.971698113	43.82977292	0.641277942
14.88721805	0.994360902	23.38016694	0.688167088
19.17647059	0.985294118	22.87890152	0.691779793
18.67195767	0.972222222	24.06019565	0.714924973
15.30941704	0.983183857	24.58645225	0.720724164
26.83943662	0.983098592	47.47167166	0.670806784
20.63636364	0.945454545	41.25552897	0.601238581
21.21428571	0.959821429	22.4848782	0.670193507
18.96774194	0.927419355	21.18228311	0.658874168
28.6635514	0.957943925	44.35877812	0.684584153
18.76875	0.953125	22.01800377	0.673192798
29.54347826	0.983695652	40.23393344	0.638918826
23.29411765	0.963235294	41.26046195	0.57298165
121.6049383	0.939814815	270.3421105	0.59570827
14	0.90625	24.93626959	0.683580605
17.53846154	0.980769231	24.09577495	0.698332656
53.82	0.9025	81.34751909	0.716000485
13.73469388	0.984693878	22.98678395	0.689679796
27.69642857	0.933035714	42.88676507	0.643835151
13	1	25.24745149	0.655173164
17	0.942307692	40.53244376	0.596922822
49.46835443	0.952531646	114.6612842	0.663807979
37.21212121	0.988636364	68.22928834	0.614160292
28.70689655	0.935344828	44.61926055	0.6492267
27.8872549	0.952205882	46.29655619	0.685571857
26.03557312	0.979249012	47.73420484	0.70573954
33	0.973214286	38.51340588	0.562617113
30.51666667	1	79.29940982	0.680892008
14.625	0.90625	22.94218014	0.628983301
18.82352941	0.955882353	25.83957117	0.703834416
22.05882353	0.985294118	22.18444262	0.664167171

log.sigma.0.5.mm.3D_glcm_Homogeneity2	log.sigma.0.5.mm.3D_glcm_ClusterShade	log.sigma.0.5.mm.3D_glcm_MaximumProbability	log.sigma.0.5.mm.3D_glcm_Idmn	log.sigma.0.5.mm.3D_glcm_SumVariance2
0.594628716	0.841106635	0.212395376	0.967689174	1.859558729
0.679015174	0.389844034	0.268054393	0.972413912	1.26953727
0.616493165	2.165705999	0.290058537	0.957776754	1.85538826
0.56541652	1.016812809	0.162811246	0.978882474	2.438710375
0.693217272	0.09539275	0.253842143	0.968080697	1.065143256
0.664306976	0.071602089	0.22193636	0.97191703	1.175558441
0.656164187	-0.749615977	0.271872649	0.960085899	1.198181485
0.598246062	0.151166464	0.172419455	0.967788784	1.941694043
0.64187418	0.033239823	0.202638593	0.974510879	1.633605607
0.657255591	0.667110322	0.280325879	0.981023716	1.427919607
0.68347582	0.017048557	0.247871253	0.965710014	1.014775838
0.609366818	0.467845386	0.2351269	0.979282714	1.88922397
0.690490559	0.236381166	0.241925973	0.982080071	1.059305952
0.722982096	0.24614648	0.280416768	0.957851525	0.999026271
0.637510302	-0.023177484	0.18541732	0.968549886	1.302390168
0.716259665	0.466229606	0.299177899	0.984394261	0.902860261
0.684242176	0.014465678	0.233110685	0.948266178	1.158207206
0.709760519	0.334070968	0.285188815	0.977848463	1.077628338
0.660751052	0.140089096	0.204940996	0.971050834	1.466462433
0.742667864	0.261638217	0.325357597	0.982156674	0.829392722
0.681696179	0.544305536	0.323491016	0.98009602	1.103837311
0.665261708	0.517890534	0.237067389	0.98344879	1.269734369
0.736089311	0.459072225	0.375223012	0.980800772	0.895512312
0.612122044	-0.527579348	0.217045887	0.960750535	1.698407232
0.701635626	0.558766952	0.354174545	0.97567607	1.005786838
0.650139918	0.405841137	0.264863082	0.967214697	1.442296892
0.685868349	0.313038288	0.279665476	0.981059808	1.051849912
0.680728222	1.053953438	0.350147649	0.982632891	1.308165274
0.643226345	-0.127442911	0.196213823	0.965435012	1.641607353
0.68941393	0.524145536	0.312380962	0.980455329	1.126318318
0.690958496	0.782949382	0.355860359	0.980101357	1.118436151
0.744938186	0.274415227	0.315692892	0.976408277	0.815321716
0.602814007	0.277244121	0.165405165	0.979674009	1.947619191
0.56544224	-0.764094152	0.210605618	0.962768673	2.181757623
0.57138263	-0.116814143	0.154948774	0.952973782	2.03280985
0.673873749	0.036494653	0.221489292	0.972900426	1.258844828
0.626774061	1.13293702	0.260344257	0.971009988	1.757869655
0.638400452	0.290416515	0.241999157	0.980112578	1.448772517
0.684942199	-0.163415068	0.249726425	0.951253056	0.975520944
0.622560117	0.356349335	0.208019721	0.978078779	1.65791291
0.640210151	0.311441984	0.227965036	0.965868728	1.466602397
0.665977463	0.227431283	0.227067812	0.970588296	1.396484921
0.642081586	-0.104450414	0.193514644	0.975273254	1.486748442
0.629236742	0.295839613	0.187778835	0.972208018	1.692549309
0.626953866	-0.135465586	0.189095215	0.978587205	1.575512917
0.68354149	0.543146796	0.296481822	0.981134194	1.081014533
0.593279771	0.221270449	0.17458992	0.982422276	1.953874026
0.638791819	0.297072127	0.232488356	0.966525199	1.473911479
0.645217693	-1.393721563	0.282820227	0.956442571	1.52954314
0.625090401	-0.433240207	0.233707176	0.973183389	1.540233565
0.656631057	0.291183965	0.229121826	0.982336502	1.282349431
0.632599212	-0.020118385	0.191866182	0.966308408	1.488553069
0.64971771	0.291702362	0.227872682	0.985133738	1.428493111
0.662933847	0.170165859	0.206914037	0.962816692	1.141216462
0.681974794	0.362401647	0.259823966	0.980680247	1.147451783
0.555390029	0.29615021	0.139825848	0.959061264	2.415015162
0.587484589	0.174311013	0.162348966	0.972917612	2.119116297
0.596033999	-0.990019295	0.224089804	0.967822823	1.90584656
0.638464743	0.030738788	0.214585489	0.974463825	1.441405752
0.711905679	0.166726996	0.316905444	0.978532551	0.945952183
0.640159841	0.429915209	0.237786	0.974809587	1.508179778
0.645725382	0.724714398	0.223774143	0.979638446	1.610139828
0.701612624	0.268890884	0.282459835	0.982903349	0.97258934
0.721173199	-0.66896933	0.274991097	0.994224912	0.990914713
0.6525821	0.110610301	0.214032592	0.941003365	1.307540141
0.614573124	0.266672369	0.175755659	0.976128372	1.818755444
0.559993313	-0.392353428	0.151420997	0.952948578	1.948462833
0.655292432	0.282456757	0.232385035	0.981746687	1.473935535
0.630241785	0.061736531	0.195820778	0.9669948	1.411586518
0.611996806	0.682032469	0.216791094	0.980722023	1.845445612
0.668970039	0.418066512	0.279486607	0.971785592	1.279979369
0.674377626	0.268415395	0.241373469	0.973212702	1.233747754
0.700612242	0.507559862	0.313854641	0.981568096	1.102578218
0.708862515	0.419726632	0.320615584	0.987083421	0.966542252
0.649668325	0.232854209	0.194964911	0.980701131	1.416789354
0.564402473	-0.406172235	0.150449992	0.976176405	2.225267462
0.647581706	0.187974964	0.212643732	0.968483749	1.373219311
0.633153285	0.349583136	0.235215486	0.965777508	1.471115139
0.663792402	0.2190472	0.269046244	0.977499923	1.324191747
0.649368209	0.689266258	0.260767144	0.974866858	1.504022908
0.608755788	0.184650699	0.208408771	0.969523055	1.810374209
0.527533454	-0.255029305	0.115423633	0.963781473	2.724095166
0.55634199	-0.044668409	0.136476993	0.987660912	2.637507787
0.665049718	-0.822412335	0.270929303	0.960187698	1.421326115
0.681533569	0.34568052	0.271022513	0.980399118	1.173512156
0.70442017	0.454920004	0.271827256	0.99124047	0.994118889
0.672163057	0.365395367	0.272771574	0.973073311	1.215246955
0.616271613	-0.200492871	0.178986083	0.972444111	1.705322507
0.633399737	-0.221306793	0.229255948	0.956489007	1.2961778
0.556353483	-2.624885679	0.23333419	0.943099055	2.397073559
0.640799524	-0.428671785	0.197271782	0.986598429	1.527066357
0.579772536	1.010719147	0.20943626	0.976340869	2.227530162
0.62348687	0.301070819	0.219084114	0.977914154	1.525779576
0.66596499	0.363500971	0.245303259	0.982324813	1.298053976
0.690815105	0.470436172	0.30289047	0.988324554	1.093893822
0.514203781	1.286831299	0.132636774	0.959534043	3.199730761
0.660543687	-1.063208339	0.260473275	0.985039668	1.505397522
0.596996192	-0.40255256	0.180382503	0.945927312	1.755138012
0.690067794	-0.138292532	0.27571735	0.976111578	1.128178397
0.641822077	1.307147489	0.236618169	0.973501934	1.616949382

log.sigma.0.5.mm.3D_glcm_Contrast	log.sigma.0.5.mm.3D_glcm_DifferenceEntropy	log.sigma.0.5.mm.3D_glcm_InverseVariance	log.sigma.0.5.mm.3D_glcm_Entropy	log.sigma.0.5.mm.3D_glcm_Dissimilarity
1.759193348	1.780143989	0.465502863	3.86289804	0.966430187
1.108071235	1.527472084	0.460634869	3.263576031	0.718503264
1.791019111	1.800595856	0.445114683	3.652797607	0.932046567
2.284925869	1.935658276	0.455753826	4.263836638	1.093569722
0.885632909	1.389805701	0.481318232	2.954807502	0.658683123
1.108761554	1.525735268	0.472154895	3.255110699	0.744021408
1.138700371	1.519879303	0.492502381	3.106227994	0.762546545
1.757758785	1.788768416	0.45509893	3.929976511	0.959468644
1.369000481	1.650045691	0.464549656	3.639146875	0.823650761
1.313554096	1.625858425	0.458573935	3.432458626	0.787488616
0.959921752	1.441800968	0.480155091	3.050844922	0.687418662
1.833654325	1.792302221	0.468697429	3.810718577	0.947828536
0.933853028	1.430417817	0.474307395	3.048358834	0.671375151
0.776870456	1.338644999	0.438830357	2.858978604	0.591174916
1.258518784	1.577595381	0.505552004	3.421816136	0.812944092
0.820307053	1.342060517	0.473502318	2.770762941	0.608466718
0.978795289	1.457615099	0.463499421	3.144720131	0.689395588
0.866672813	1.395300622	0.454007708	2.990877707	0.627937234
1.146361155	1.538627364	0.468562633	3.414827168	0.756253492
0.688044744	1.27119374	0.435919699	2.619717752	0.543428932
1.050733766	1.507310833	0.461810143	3.099485347	0.704962284
1.12989851	1.52287477	0.483406471	3.276789769	0.744558568
0.742730406	1.322310367	0.424955511	2.695554939	0.563604837
1.609357615	1.733560349	0.461868355	3.752015786	0.913122556
0.966203957	1.461352156	0.444737733	2.927258331	0.657528432
1.337069342	1.627282689	0.464780133	3.48038663	0.804203317
1.000427368	1.466725966	0.476135121	3.054336411	0.689155327
1.197476878	1.581902602	0.435213416	3.219104003	0.7299417
1.409453933	1.651394805	0.44758898	3.621936726	0.828106453
1.038274097	1.493640589	0.460401079	3.114464076	0.689648707
1.059495805	1.513806977	0.444223176	3.057559879	0.690348981
0.638588738	1.229943866	0.44593601	2.572954126	0.531522524
1.772384608	1.788325646	0.467517283	3.941047346	0.953188906
2.059185845	1.821764692	0.473637513	3.829251885	1.062720097
1.969127268	1.826583779	0.47855426	4.015702276	1.038727653
1.069939364	1.514371329	0.464411351	3.285207034	0.721622071
1.583340513	1.732808028	0.456571526	3.708769426	0.882929938
1.365628791	1.64896683	0.468532789	3.524959042	0.82883573
0.902469263	1.391920829	0.489340481	2.960179984	0.675507878
1.515522797	1.702121888	0.469843774	3.707690907	0.879591167
1.392566334	1.655170393	0.46762473	3.553339899	0.829939928
1.178240622	1.567867626	0.459334391	3.41409058	0.752390082
1.319349979	1.62017634	0.469122661	3.533797576	0.815402327
1.505564438	1.698616023	0.461350933	3.70991191	0.86676126
1.484607755	1.685253517	0.475575804	3.655378579	0.86622803
0.98971447	1.460549059	0.478044862	3.060606867	0.691915894
1.88520288	1.818091173	0.477540478	3.977770618	0.984117733
1.352859826	1.638938063	0.466421	3.537953198	0.826103694
1.259989395	1.575574871	0.4799416	3.26861876	0.800705794
1.439122069	1.665578006	0.473067187	3.56194522	0.862973729
1.20687863	1.570680119	0.479186486	3.367132533	0.772246576
1.350004322	1.626344433	0.470543523	3.538968697	0.836827748
1.272344091	1.601261331	0.475631287	3.477432217	0.794498628
1.043846685	1.474377428	0.501550857	3.191099612	0.735709277
1.017614166	1.469709067	0.478912374	3.147270084	0.699148062
2.298113162	1.936838956	0.457521378	4.274947099	1.115991586
1.901000233	1.815108234	0.462157625	4.027462869	1.000123189
1.762574709	1.775462608	0.472205077	3.847174196	0.963087388
1.38275112	1.637363815	0.484053248	3.512239685	0.830046882
0.839133811	1.374692586	0.458645098	2.829355375	0.619361012
1.349338809	1.638645276	0.469791854	3.563364934	0.823210095
1.41475367	1.668475299	0.45788905	3.617193276	0.823154618
0.893420397	1.408280927	0.46709788	2.916662431	0.645769402
0.881150311	1.347677231	0.460335192	2.787007569	0.603929929
1.127801929	1.518579799	0.487621996	3.308402476	0.766996821
1.669568302	1.752768593	0.466567534	3.836317132	0.916914466
1.937089075	1.807016795	0.477889143	3.952176134	1.054605588
1.248686908	1.597945852	0.464602534	3.493815577	0.781536713
1.3182915	1.611697304	0.488772428	3.505303145	0.835394915
1.679483134	1.756460818	0.46634129	3.833395252	0.922092593
1.120495768	1.535818513	0.463500102	3.281429199	0.738089524
1.059585438	1.502592999	0.471575655	3.251572568	0.718725628
0.976007053	1.464112286	0.4495504	3.049781434	0.660693034
0.870512697	1.399090167	0.459489096	2.895691155	0.629718365
1.335595256	1.6252235	0.477560565	3.48226295	0.804024126
2.099750105	1.866461802	0.478818685	4.130100809	1.067863241
1.263547268	1.597802165	0.470342269	3.452210038	0.797066718
1.376933016	1.64548932	0.466555448	3.54691964	0.840394689
1.19790622	1.575376026	0.456424797	3.370347222	0.75890407
1.354821254	1.649587851	0.452007541	3.532260608	0.808112822
1.663799403	1.746778013	0.465093145	3.812643851	0.925784897
2.611266256	2.005735557	0.455837965	4.466094608	1.209481901
2.548197159	1.989086917	0.457955894	4.381001743	1.143628731
1.147459649	1.524663923	0.476910271	3.239483883	0.749097831
1.029720414	1.489819947	0.463494558	3.176087388	0.702061867
0.912392866	1.409124872	0.470015789	2.916457135	0.643115528
1.063586949	1.502729129	0.474901066	3.231478024	0.723232678
1.47459555	1.675254173	0.480594641	3.709439302	0.884363071
1.242625543	1.479186898	0.508327354	3.281204	0.817978124
2.512593941	1.937966204	0.445830134	3.796108374	1.144078773
1.421792024	1.656972212	0.475700817	3.560953264	0.830958706
2.104123211	1.876181827	0.470541458	4.07883325	1.040235717
1.540319717	1.701427406	0.481048149	3.643253904	0.880340173
1.215775087	1.580881181	0.462620703	3.357234817	0.757417028
0.991259078	1.469819231	0.463557886	3.063283122	0.679502477
2.951416647	2.062179486	0.443547214	4.568367624	1.284062968
1.303334338	1.605693724	0.464480576	3.409611074	0.777711495
1.586904389	1.674110453	0.474643332	3.667688988	0.934858154
0.934339333	1.426678213	0.477991838	3.008267782	0.672100621
1.451970453	1.659182026	0.478386344	3.542943383	0.835002942

log.sigma.0.5.mm.3D_glcm_DifferenceVariance	log.sigma.0.5.mm.3D_glcm_Idn	log.sigma.0.5.mm.3D_glcm_Idm	log.sigma.0.5.mm.3D_glcm_Correlation	log.sigma.0.5.mm.3D_glcm_Autocorrelation
0.821211369	0.889453065	0.594628716	0.027134318	11.89875548
0.588750964	0.903846251	0.679015174	0.066832756	11.90037209
0.920458695	0.880615851	0.616493165	0.016479835	5.912714359
1.086596501	0.908835401	0.56541652	0.032245948	30.64196226
0.448266118	0.895449222	0.693217272	0.091710408	6.301114363
0.553521404	0.899975234	0.664306976	0.029140101	11.84893915
0.551535884	0.881400583	0.656164187	0.028873096	11.898652
0.834376427	0.890372297	0.598246062	0.04980764	19.87490896
0.689266	0.904158238	0.64187418	0.088176042	19.72958258
0.692067659	0.917945351	0.657255591	0.041055885	19.636146
0.485617113	0.891676205	0.68347582	0.027470724	11.69608438
0.928214403	0.912532617	0.609366818	0.012134091	29.67764298
0.480322917	0.919605049	0.690490559	0.063285905	12.22581948
0.422487318	0.887832177	0.722982096	0.124466798	6.207265008
0.597175978	0.891365882	0.637510302	0.017056449	12.33179825
0.448016133	0.926669616	0.716259665	0.048074919	11.98767315
0.499029509	0.87143684	0.684242176	0.083384965	6.001998232
0.46797676	0.914371533	0.709760519	0.107534789	12.01089887
0.568187002	0.898650273	0.660751052	0.121931444	12.29116354
0.388444365	0.924848132	0.742667864	0.092336376	11.93645498
0.553514204	0.916451878	0.681696179	0.024514012	11.64808217
0.569512456	0.921317089	0.665261708	0.058201448	20.23907705
0.420986374	0.922591065	0.736089311	0.092173563	11.61617041
0.771995736	0.881124654	0.612122044	0.027078135	12.53443825
0.532091024	0.911257767	0.701635626	0.019831156	5.701944602
0.687161352	0.89391712	0.650139918	0.035692334	11.48549406
0.524995354	0.917933072	0.685868349	0.025123943	19.88349575
0.663359272	0.923755556	0.680728222	0.043131797	19.22548288
0.706597988	0.891374244	0.643226345	0.078392428	12.49977203
0.561647558	0.918336191	0.68941393	0.040436176	11.52119218
0.581599554	0.918521454	0.690958496	0.027033064	11.46662242
0.352843543	0.913900171	0.744938186	0.12105578	6.104604565
0.85960695	0.911607281	0.602814007	0.047150886	20.32471928
0.91979401	0.879846664	0.56544224	0.029891422	11.84264756
0.879820163	0.866784102	0.57138263	0.017618997	11.92365782
0.548029882	0.902881083	0.673873749	0.080812786	12.02710609
0.801470912	0.898610639	0.626774061	0.051647989	11.84165437
0.678409561	0.913556537	0.638400452	0.029452289	30.06438474
0.440010286	0.872313465	0.684942199	0.039819924	6.378894806
0.740397005	0.908908769	0.622560117	0.044609402	19.698116
0.701167439	0.890752877	0.640210151	0.025545416	12.00777734
0.610525837	0.899690469	0.665977463	0.084639724	11.86906053
0.647727686	0.904708454	0.642081586	0.059913685	19.81213424
0.74763595	0.899920262	0.629236742	0.057993017	11.96987028
0.730713281	0.910166823	0.626953866	0.030111352	19.87052549
0.509167805	0.917467259	0.68354149	0.042477703	11.85231786
0.915560028	0.91682285	0.593279771	0.017816304	42.35572884
0.668987944	0.890822426	0.638791819	0.042104845	11.83826022
0.61310137	0.876835806	0.645217693	0.098380666	11.71811818
0.691008395	0.899673607	0.625090401	0.034728248	20.00129586
0.608466285	0.918772498	0.656631057	0.030453061	19.91526199
0.642626524	0.889178516	0.632599212	0.048942884	11.53509347
0.638556538	0.924677681	0.64971771	0.056852343	29.8169918
0.501496521	0.884447714	0.662933847	0.044886969	6.292582844
0.524881845	0.916808169	0.681974794	0.060449597	12.00850295
1.049716837	0.87534039	0.555390029	0.024707517	11.83567732
0.879655365	0.898142933	0.587484589	0.053963091	19.88765157
0.82385688	0.889858466	0.596033999	0.040199467	20.2534459
0.692172632	0.903339858	0.638464743	0.020683031	11.76793801
0.45407981	0.91525725	0.711905679	0.058712622	19.69482216
0.669672266	0.903984853	0.640159841	0.055640745	19.57302084
0.734554837	0.914646647	0.645725382	0.064723521	20.16240968
0.473730655	0.92257048	0.701612624	0.043152838	20.14352002
0.515044263	0.954879821	0.721173199	0.057749211	89.528424
0.53662966	0.858187853	0.6525821	0.073139713	5.724059066
0.825139071	0.905861338	0.614573124	0.042855288	20.79273193
0.819216144	0.864049305	0.559993313	0.003838012	11.58763147
0.635721486	0.918092007	0.655292432	0.082155064	19.8941452
0.619420258	0.888874025	0.630241785	0.034046611	11.58112582
0.824106938	0.914194363	0.611996806	0.047475258	20.03225824
0.571164919	0.901068952	0.668970039	0.065214167	11.92215416
0.541363324	0.903129739	0.674377626	0.075443504	11.70673943
0.537712133	0.921534269	0.700612242	0.060741412	11.81808237
0.473119811	0.932588617	0.708862515	0.051850162	11.73935288
0.687939678	0.916156398	0.649668325	0.029046727	20.53399415
0.949233618	0.901835422	0.564402473	0.029096845	19.61000419
0.624466807	0.894015446	0.647581706	0.041769941	11.76632059
0.666655839	0.8890215	0.633153285	0.032187925	11.37197206
0.618028087	0.910911909	0.663792402	0.049836857	19.31694793
0.7000291	0.906082559	0.649368209	0.052260373	11.72915341
0.802966098	0.893761831	0.608755788	0.040649678	18.73968832
1.144631772	0.879655099	0.527533454	0.021495738	20.11903847
1.233819441	0.9291227	0.55634199	0.017674526	91.09643843
0.581770305	0.884034189	0.665049718	0.107448324	12.5421799
0.533813325	0.916612885	0.681533569	0.064500392	12.01624429
0.497660816	0.943458988	0.70442017	0.042883204	30.0763822
0.539912896	0.902491614	0.672163057	0.066096482	11.70476866
0.688460399	0.897225526	0.616271613	0.072357361	19.56656748
0.533420852	0.87330524	0.633399737	0.024645525	12.50438228
1.181004453	0.857880425	0.556353483	-0.021380295	18.81385484
0.729325914	0.928621703	0.640799524	0.034453965	42.01784686
1.019706162	0.904744929	0.579772536	0.02610764	28.69776284
0.763244484	0.908967129	0.62348687	-0.004649668	19.75939537
0.639637295	0.920616869	0.66596499	0.032611836	19.90191656
0.528671857	0.934755503	0.690815105	0.048994195	19.84190806
1.283773761	0.873846239	0.514203781	0.041095297	19.31545194
0.696558505	0.926620715	0.660543687	0.06905018	30.82430645
0.708661944	0.858804311	0.596996192	0.052040688	12.20460194
0.480707323	0.908482905	0.690067794	0.093173476	12.47210344
0.75176644	0.903423438	0.641822077	0.052634309	11.82698879

log.sigma.0.5.mm.3D_glcm_SumEntropy	log.sigma.0.5.mm.3D_glcm_AverageIntensity	log.sigma.0.5.mm.3D_glcm_Energy	log.sigma.0.5.mm.3D_glcm_SumSquares	log.sigma.0.5.mm.3D_glcm_ClusterProminence
2.461781524	3.445794875	0.098021309	0.904688019	10.47978366
2.189301464	3.443825241	0.154146168	0.594402126	5.796277606
2.382415148	2.428203074	0.1301484	0.911601843	14.52689134
2.669574866	5.532034089	0.07841839	1.180909061	21.40594554
2.054408641	2.501185464	0.170790358	0.487694041	3.933907646
2.147885636	3.439782767	0.142064097	0.571079999	3.869977971
2.110707739	3.447235093	0.161761604	0.584220464	4.693056313
2.505533762	4.452922768	0.089467465	0.924863207	10.54732538
2.388061205	4.434335413	0.115486588	0.750651522	8.550695128
2.258405089	4.42803648	0.146162108	0.685368426	8.393404732
2.044066821	3.417910456	0.161510032	0.493674397	2.985048028
2.459731233	5.446419094	0.113732697	0.930719574	14.71378483
2.067728704	3.492045348	0.164659051	0.498289745	3.915189743
2.002229934	2.48023909	0.182037284	0.443974182	2.782777227
2.22690053	3.510097091	0.130627253	0.640227238	5.540101151
1.925537156	3.459337343	0.2006026	0.430791828	3.597801021
2.130085332	2.440603988	0.149996765	0.534250624	3.63771658
2.069653022	3.458035242	0.175870853	0.486075288	3.717689257
2.296933439	3.494371719	0.124906427	0.653205897	5.951522924
1.864256024	3.449788971	0.214030884	0.379359366	2.086708257
2.076738851	3.410981915	0.176986879	0.538642769	4.377281554
2.186513553	4.494881152	0.147463249	0.59990822	6.031039355
1.898891652	3.402632291	0.220321062	0.40956068	2.564109734
2.401674852	3.537233022	0.106427926	0.826941212	8.832575038
1.993537693	2.385796825	0.198849941	0.492997699	3.839147131
2.27569365	3.385111427	0.135636801	0.694841558	7.394216306
2.04890565	4.457647886	0.172654073	0.51306932	4.406577652
2.157027702	4.381520213	0.17922525	0.626410538	7.21787324
2.377013275	3.527218598	0.112924935	0.762765321	7.488553403
2.096500918	3.391040033	0.174665862	0.541148104	5.008981064
2.061278338	3.384059667	0.19059006	0.544482989	5.063659526
1.856763444	2.461782737	0.215784116	0.363477613	2.03014541
2.515907299	4.503422875	0.094468032	0.93000095	12.19130205
2.487761065	3.436426417	0.103047017	1.060235867	16.17947171
2.526378135	3.450639239	0.083430959	1.00048428	11.57502028
2.200318513	3.461184586	0.139872623	0.582196048	4.463828222
2.412461047	3.434813351	0.120552509	0.835302542	10.5510601
2.295499906	5.481198841	0.1267932	0.703600327	6.878016984
2.007375568	2.52199678	0.169192559	0.469497552	2.813218743
2.393600117	4.434232547	0.109601957	0.793358927	8.705522752
2.3050969	3.462524451	0.125493259	0.714792183	7.054805238
2.270312923	3.437206578	0.133020602	0.643681386	6.11768127
2.315054395	4.446358816	0.117825278	0.701524605	6.349030188
2.404694696	3.45293304	0.109522769	0.799528437	9.145636941
2.358435322	4.45508032	0.114603412	0.765030168	8.242490893
2.063377904	3.439387499	0.173144199	0.517682251	4.114403813
2.514233607	6.506800288	0.095708438	0.959769226	14.27243922
2.302722715	3.436226765	0.124460682	0.706692826	6.780242809
2.233095823	3.413030371	0.150549741	0.697383134	7.913384983
2.327349683	4.469397044	0.120210981	0.744838908	6.964168968
2.211967827	4.460533824	0.138002545	0.622307015	5.528982676
2.307324132	3.391192116	0.11337384	0.709639348	5.857178282
2.287258669	5.456911676	0.128525691	0.675209301	6.958308969
2.128092823	2.503617538	0.146186179	0.546265787	4.00671611
2.121489226	3.460627886	0.159025353	0.541266487	4.586112027
2.669982271	3.436019629	0.072281377	1.178282081	17.53797236
2.566687322	4.453349164	0.085312084	1.005029133	13.68719391
2.472121335	4.496354686	0.105239127	0.917105317	12.31518274
2.286495326	3.428283191	0.127911886	0.706039218	7.860173853
1.96269083	4.434855472	0.19335694	0.446271498	3.118683111
2.323532506	4.41964045	0.122090876	0.714379647	7.102396718
2.36301939	4.484810045	0.121929248	0.756223374	9.6988278
1.995919752	4.48594473	0.181981798	0.466502434	3.124596403
1.955724836	9.460493451	0.193746866	0.468016256	8.834723415
2.207270421	2.383031082	0.128863733	0.608835518	4.819589032
2.46396738	4.555795231	0.102573502	0.872080937	10.98403903
2.496554536	3.403533331	0.080465218	0.971387977	9.826176441
2.309807544	4.453962068	0.128093468	0.680655611	6.963325298
2.277526342	3.399651352	0.116092584	0.682469504	5.565441534
2.462997783	4.471093907	0.105264337	0.881232187	11.59831893
2.193366423	3.44704409	0.149935312	0.600118784	5.109830045
2.177926343	3.415120148	0.146070297	0.573333298	4.962531336
2.074983919	3.433126995	0.180169581	0.519646318	4.552308586
1.985661451	3.422766075	0.19213234	0.459263737	3.423992037
2.272229724	4.529200785	0.134744479	0.688096152	8.236551917
2.602583991	4.424741546	0.078665185	1.081254392	15.48949549
2.257861814	3.426181396	0.127812455	0.659191645	5.917312582
2.298094166	3.368699331	0.118384696	0.712012039	6.272076696
2.22302369	4.391497773	0.142512377	0.630524492	5.5141565
2.309496969	3.419326295	0.129688715	0.714711041	7.972241218
2.450840381	4.324644409	0.103189789	0.868543403	10.61881645
2.756922953	4.482247286	0.06084385	1.333840356	21.51846919
2.725024478	9.543270716	0.073080633	1.296426237	27.07831306
2.214590383	3.531625187	0.151191992	0.642196441	6.923611972
2.135119735	3.461251335	0.155461206	0.550808142	4.225058956
2.00074452	5.482330274	0.184513375	0.476627939	4.44647746
2.165436541	3.415645614	0.150818593	0.569708476	4.519726178
2.416570247	4.416828263	0.101680229	0.794979514	8.06402083
2.175239633	3.533854612	0.135734557	0.634700836	5.161879355
2.507143552	4.340429775	0.109843995	1.227416875	18.37530492
2.323841243	6.480083032	0.12545308	0.737214595	10.1310649
2.584375269	5.354127178	0.093392056	1.082913343	18.945152
2.326703049	4.445554001	0.12001771	0.766524823	8.536605447
2.209753815	4.458844505	0.144587257	0.628457266	6.407518952
2.073829641	4.451542818	0.174781173	0.521288225	4.320565797
2.833647897	4.387733872	0.057440403	1.537786852	30.76547722
2.274993847	5.547397957	0.143068232	0.702182965	12.24864769
2.372491591	3.487193062	0.098093102	0.8355106	8.517501591
2.078681787	3.524695593	0.17478847	0.515629432	4.919394616
2.331412692	3.433013258	0.131184328	0.767229959	12.13309568

log.sigma.0.5.mm.3D_glcm_SumAverage	log.sigma.0.5.mm.3D_glcm_Imc2	log.sigma.0.5.mm.3D_glcm_Imc1	log.sigma.0.5.mm.3D_glcm_DifferenceAverage	log.sigma.0.5.mm.3D_glcm_Id
6.891589749	0.282960843	-0.022470882	0.966430187	0.627440109
6.887650482	0.268315832	-0.024479846	0.718503264	0.696375466
4.856406147	0.295477904	-0.025372049	0.932046567	0.646607915
11.06406818	0.25742905	-0.016935312	1.093569722	0.603817828
5.002370928	0.283868194	-0.029440486	0.658683123	0.706135585
6.879565533	0.228459156	-0.01677559	0.744021408	0.683656648
6.894470187	0.270383803	-0.025211359	0.762546545	0.675029882
8.905845536	0.283649849	-0.022479125	0.959468644	0.631388731
8.868670826	0.254417731	-0.018976821	0.823650761	0.665943318
8.856072961	0.242837759	-0.018541934	0.787488616	0.678832418
6.835820912	0.20025478	-0.014192623	0.687418662	0.698358266
10.89283819	0.329903487	-0.031760887	0.947828536	0.638476136
6.984090697	0.218775272	-0.017678264	0.671375151	0.704555055
4.960478181	0.270670753	-0.035343709	0.591174916	0.734288254
7.020194182	0.185234419	-0.01080308	0.812944092	0.658628806
6.918674685	0.234036067	-0.021935766	0.608466718	0.725226414
4.881207977	0.244093924	-0.021326424	0.689395588	0.700641876
6.916070483	0.256836116	-0.026975103	0.627937234	0.722011
6.988743438	0.304026815	-0.032887017	0.756253492	0.681111222
6.899577941	0.246786814	-0.032187231	0.543428932	0.750314586
6.821963831	0.16288788	-0.009019864	0.704962284	0.698561392
8.989762304	0.243856439	-0.023516977	0.744558568	0.683162365
6.805264582	0.222911261	-0.028039047	0.563604837	0.746121391
7.074466045	0.275247955	-0.021822919	0.913122556	0.642054647
4.771593651	0.227136417	-0.020151793	0.657528432	0.716267101
6.770222854	0.310002037	-0.031088515	0.804203317	0.672502216
8.915295772	0.204783625	-0.014576762	0.689155327	0.700514712
8.763040426	0.257712181	-0.02257231	0.7299417	0.700060067
7.054437196	0.31434283	-0.04170227	0.828106453	0.668632495
6.782080065	0.228276996	-0.017601087	0.689648707	0.704790772
6.768119334	0.20570873	-0.015171964	0.690348981	0.70763066
4.923565475	0.209125262	-0.022597285	0.531522524	0.751220496
9.00684575	0.257991997	-0.018803302	0.953188906	0.633604817
6.872852834	0.518325676	-0.083096296	1.062720097	0.602676401
6.901278477	0.338078707	-0.031273553	1.038727653	0.607111549
6.922369172	0.22065193	-0.01629757	0.721622071	0.692095907
6.869626703	0.237543358	-0.017385846	0.882929938	0.654185011
10.96239768	0.190648253	-0.010793754	0.82883573	0.662827514
5.043993561	0.20473951	-0.01739052	0.675507878	0.698757496
8.868465095	0.254427295	-0.019308337	0.879591167	0.64974276
6.925048903	0.238918341	-0.017496066	0.829939928	0.664233886
6.874413155	0.276494283	-0.023975668	0.752390082	0.686048411
8.892717632	0.269983543	-0.022776889	0.815402327	0.665811045
6.905866079	0.305896299	-0.027237574	0.86676126	0.655891259
8.91016064	0.238899619	-0.017211498	0.86622803	0.652711654
6.878774997	0.202156852	-0.014344491	0.691915894	0.698522163
13.01360058	0.220334987	-0.013060976	0.984117733	0.624760229
6.872453529	0.257916527	-0.020040418	0.826103694	0.663405358
6.826060742	0.380409213	-0.04935039	0.800705794	0.667339899
8.938794087	0.269874547	-0.02162557	0.862973729	0.65167379
8.921067648	0.200369355	-0.01382721	0.772246576	0.676551972
6.782384232	0.276198818	-0.025557739	0.836827748	0.658127993
10.91382335	0.23644678	-0.017940538	0.794498628	0.671278587
5.007235075	0.202816646	-0.013762641	0.735709277	0.679728006
6.921255773	0.256173487	-0.023834303	0.699148062	0.697096529
6.872039259	0.27332538	-0.018462656	1.115991586	0.595650714
8.906698328	0.330816274	-0.031899371	1.000123189	0.62169738
8.992709371	0.27552647	-0.021702446	0.963087388	0.627851569
6.856566382	0.277072041	-0.023406084	0.830046882	0.661206241
8.869710945	0.233559253	-0.021360395	0.619361012	0.723314426
8.8392809	0.23055411	-0.016503918	0.823210095	0.664130305
8.969620091	0.261620702	-0.021020638	0.823154618	0.669513562
8.971889461	0.197574661	-0.015112231	0.645769402	0.714165575
18.9209869	0.243411313	-0.022639436	0.603929929	0.730419983
4.766062165	0.278628703	-0.026989649	0.766996821	0.672789106
9.111590462	0.283214483	-0.022975509	0.916914466	0.643280714
6.807066661	0.276740608	-0.020825589	1.054605588	0.59837316
8.907924136	0.246440317	-0.019491543	0.781536713	0.67688207
6.799302704	0.247956905	-0.018429806	0.835394915	0.654486576
8.942187814	0.273498269	-0.021968114	0.922092593	0.641277942
6.89408818	0.229517707	-0.018380121	0.738089524	0.688167088
6.830240295	0.245401975	-0.020434161	0.718725628	0.691779793
6.866253991	0.228274142	-0.019252837	0.660693034	0.714924973
6.845532149	0.186723369	-0.013429165	0.629718365	0.720724164
9.058401569	0.230928252	-0.016384887	0.804024126	0.670806784
8.849483092	0.304767594	-0.024087186	1.067863241	0.601238581
6.852362793	0.238090642	-0.0179682	0.797066718	0.670193507
6.737398661	0.269834622	-0.023680622	0.840394689	0.658874168
8.782995545	0.241088421	-0.02115073	0.75890407	0.684584153
6.838652591	0.241834335	-0.018634751	0.808112822	0.673192798
8.649288818	0.308186826	-0.028302599	0.925784897	0.638918826
8.964494571	0.248298088	-0.014639425	1.209481901	0.57298165
19.08654143	0.29259034	-0.021348125	1.143628731	0.59570827
7.063250374	0.396845515	-0.056153075	0.749097831	0.683580605
6.92250267	0.231958214	-0.019724739	0.702061867	0.698332656
10.96466055	0.19760771	-0.014727259	0.643115528	0.716000485
6.831291228	0.228758845	-0.017577779	0.723232678	0.689679796
8.833656526	0.244338508	-0.017765259	0.884363071	0.643835151
7.067709224	0.468213289	-0.079399754	0.817978124	0.655173164
8.68085955	0.547557223	-0.094484868	1.144078773	0.596922822
12.96016606	0.230163501	-0.016055442	0.830958706	0.663807979
10.70825436	0.321452041	-0.027478281	1.040235717	0.614160292
8.891108002	0.27736926	-0.022390336	0.880340173	0.6492267
8.917689009	0.237888887	-0.018269982	0.757417028	0.685571857
8.903085636	0.184077677	-0.012391833	0.679502477	0.70573954
8.775467743	0.459724585	-0.052339767	1.284062968	0.562617113
11.09479591	0.310806681	-0.031043282	0.777711495	0.680892008
6.974386125	0.450967493	-0.064675864	0.934858154	0.628983301
7.049391186	0.306154524	-0.03363732	0.672100621	0.703834416
6.866026516	0.32203784	-0.031856579	0.835002942	0.664167171

log.sigma.0.5.mm.3D_glcm_ClusterTendency	log.sigma.0.5.mm.3D_firstorder_InterquartileRange	log.sigma.0.5.mm.3D_firstorder_Skewness	log.sigma.0.5.mm.3D_firstorder_Uniformity
1.859558729	30.00631046	0.238609843	0.302430001
1.26953727	13.05400038	0.307576917	0.384433077
1.85538826	24.88069963	0.892288474	0.327029667
2.438710375	29.44358778	0.234711586	0.280105427
1.065143256	22.38276243	-0.020286147	0.41515041
1.175558441	23.04025602	-0.040852229	0.37586498
1.198181485	14.97912025	-0.957523294	0.419084433
1.941694043	31.68336344	-0.084394816	0.300429082
1.633605607	26.71618223	0.036774551	0.332447219
1.427919607	19.99123025	0.34469221	0.363820595
1.014775838	22.79398251	-0.068726052	0.397586592
1.88922397	23.94654369	-0.011097034	0.318331904
1.059305952	20.44040442	0.262548906	0.40007802
0.999026271	20.04032898	0.123268046	0.413169121
1.302390168	23.87397003	-0.17784517	0.366872269
0.902860261	5.974271178	1.001789075	0.444399533
1.158207206	23.78325891	0.061638026	0.380477172
1.077628338	18.27718067	0.318104444	0.405411399
1.466462433	25.21041989	-0.137020239	0.348082805
0.829392722	11.04817104	0.220924191	0.448379289
1.103837311	15.50840867	0.49621069	0.402382235
1.269734369	22.61740232	0.388459834	0.372457409
0.895512312	17.52665567	0.356224612	0.441309693
1.698407232	26.5413475	-0.17514986	0.326446549
1.005786838	17.75772011	0.672828596	0.416043973
1.442296892	22.69755507	-0.018284749	0.339688063
1.051849912	13.2106446	0.179285297	0.407935766
1.308165274	17.35031462	0.594422053	0.388117492
1.641607353	26.95852184	-0.082527906	0.330537602
1.126318318	12.68180364	0.539620711	0.402067918
1.118436151	15.94250488	0.755108623	0.411452813
0.815321716	13.42618108	0.541127889	0.453508299
1.947619191	27.20463753	0.096944629	0.305721701
2.181757623	23.74924874	-0.226933382	0.337093195
2.03280985	32.88648868	-0.230111647	0.285146611
1.258844828	22.18504596	-0.075655825	0.372411528
1.757869655	22.59836245	0.474494751	0.335465856
1.448772517	23.42123866	0.014633906	0.347073369
0.975520944	20.80901432	-0.231540458	0.413870702
1.65791291	27.61809158	0.087361058	0.322854123
1.466602397	19.17250967	0.163504907	0.343268854
1.396484921	24.40718651	0.01492684	0.358755529
1.486748442	25.42468119	-0.17433873	0.346941803
1.692549309	25.33907127	0.046838184	0.328150387
1.575512917	26.00336003	-0.080602428	0.338587263
1.081014533	18.25338721	0.517665199	0.392554448
1.953874026	27.64339828	-0.058904933	0.30203548
1.473911479	26.83434916	0.047859326	0.342738365
1.52954314	18.84204638	-0.880802161	0.420249499
1.540233565	25.02546024	-0.362576482	0.359667057
1.282349431	19.72358918	0.235498598	0.370277631
1.488553069	26.25596333	-0.141447609	0.338808561
1.428493111	19.54944992	0.101892727	0.355174688
1.141216462	20.82349491	0.110917755	0.385440376
1.147451783	9.191750288	0.410817517	0.403429824
2.415015162	33.66734123	-0.092690962	0.267926501
2.119116297	32.88012505	0.063409552	0.284010456
1.90584656	23.29042816	-0.505493913	0.332908654
1.441405752	19.12567449	0.014816354	0.346539973
0.945952183	19.15379047	-0.219508452	0.418094957
1.508179778	24.79474688	0.060088106	0.340809828
1.610139828	21.9203949	0.348971692	0.345985889
0.97258934	13.14432132	0.11279374	0.421578743
0.990914713	9.337600946	-2.196153459	0.430941999
1.307540141	22.8060112	0.01156021	0.359213404
1.818755444	22.24994278	0.180807313	0.324771572
1.948462833	32.24158001	-0.344067844	0.295730372
1.473935535	22.98022652	0.026381978	0.35389635
1.411586518	27.16109681	-0.307902849	0.339299001
1.845445612	23.95607042	0.239580809	0.322400096
1.279979369	20.52642918	0.280676358	0.375555532
1.233747754	22.74537373	-0.059390869	0.377595731
1.102578218	14.89491165	0.435963198	0.410679272
0.966542252	12.94402874	0.41896087	0.426413406
1.416789354	16.57671571	0.11851107	0.363663287
2.225267462	32.70837831	-0.181901938	0.282354415
1.373219311	24.60926342	0.109272921	0.354892125
1.471115139	29.68078351	0.0759179	0.329174953
1.324191747	25.78036547	-0.017088962	0.360358467
1.504022908	23.57519388	0.342224259	0.347583792
1.810374209	30.29232621	-0.005323275	0.301634543
2.724095166	39.51546288	-0.189979038	0.247074289
2.637507787	25.2647109	-0.153188652	0.282507687
1.421326115	20.04352856	-0.477699536	0.393198573
1.173512156	20.23705912	0.205365967	0.385736056
0.994118889	14.58319581	0.498678056	0.42475353
1.215246955	25.42429328	0.076948506	0.368689739
1.705322507	27.81408334	-0.29054581	0.325619088
1.2961778	18.42946792	-0.467621583	0.349099704
2.397073559	20.83372211	-1.189016895	0.361088435
1.527066357	18.81710434	-0.593461233	0.355193861
2.227530162	31.5267446	0.286081357	0.278426919
1.525779576	20.79229188	0.131846877	0.333770551
1.298053976	18.91771579	0.270347224	0.371360047
1.093893822	16.55948341	0.343265136	0.402396454
3.199730761	40.52250862	0.140081282	0.225577653
1.505397522	21.33374906	-0.935903184	0.371778155
1.755138012	25.11964178	-0.405549503	0.330689404
1.128178397	19.92245626	-0.093055209	0.420597183
1.616949382	18.09829712	0.830890458	0.333634293

log.sigma.0.5.mm.3D_firstorder_MeanAbsoluteDeviation	log.sigma.0.5.mm.3D_firstorder_Energy	log.sigma.0.5.mm.3D_firstorder_RobustMeanAbsoluteDeviation	log.sigma.0.5.mm.3D_firstorder_Median
17.2185869	349846.5912	11.68473904	-0.860026866
11.40564712	580079.8551	6.285825434	-0.639401138
16.79455245	222205.0514	10.49506258	-3.249117136
19.35301381	1727418.445	12.51439199	-0.242499709
12.78999247	96960.18239	8.800270811	2.261800528
13.91735404	194766.9808	9.761086181	0.047515232
11.33527817	82765.82276	6.807099882	1.659275174
18.19301573	507534.9643	12.97278515	0.561032653
15.99588448	862100.7604	10.96999482	-0.904836595
13.9029808	1145449.473	8.491536695	-1.796426356
13.06633664	216385.8012	9.321952701	-0.635275006
16.81296149	805441.2051	10.34624242	-1.62563616
12.35005252	518736.1106	8.433256227	-0.115234088
12.01128433	250756.9374	8.425095315	-0.329400912
14.43190161	311409.0671	9.884963339	1.048364282
7.357116599	1049177.435	3.198738379	-0.498872727
13.59402387	197747.2192	9.706861816	-0.091699049
11.74597207	554614.63	7.693382327	-0.420343429
15.00278631	203873.4203	10.48671091	2.777632833
8.691849369	428165.3749	5.058150434	-0.387313366
11.7682859	1422749.088	7.01554808	-1.769535244
13.78197409	546676.7135	9.303458247	-0.122432876
10.91120221	562618.6853	7.153112743	-1.637457728
16.59371786	221968.1228	11.09309475	2.735159397
12.12550998	326198.9455	7.703447509	-2.979354143
14.81508806	267204.2463	9.693723532	-1.59068507
10.74519773	838948.3532	6.106549621	-0.97274211
13.17660401	479793.7204	7.911879853	-2.910140276
15.95367622	719293.4363	11.02936377	1.091647863
10.85697031	660925.9121	5.971163373	-1.185233831
12.01917567	888352.9354	7.28515479	-2.595018625
9.108155818	904360.9583	5.711162241	-0.692706853
17.04223192	1029037.513	11.21889863	0.180970892
17.262543	56128.25117	10.53176402	4.400357723
19.07983072	187400.4114	13.38216989	3.648613691
13.34484369	576873.6017	9.195506027	-0.39777191
15.20569524	1272495.247	9.31604928	-1.305360615
14.84641554	1290506.002	9.679320611	-1.15230304
12.42234112	201576.3258	8.770378564	2.278425455
16.22809087	462524.4985	10.99711944	-0.519415021
13.79557054	541480.7339	8.284006977	-0.572138518
14.8236437	330719.8717	10.32996409	-0.268647432
15.13682816	595874.5967	10.5377968	0.354583398
16.0225737	437525.1	10.78075725	0.650548995
15.8194433	765897.0297	10.87167599	0.411753058
12.30059754	612371.6928	7.794458803	-1.125423431
17.6612039	2359671.988	11.47955947	-0.183096699
15.77907795	227547.5545	11.07504951	-1.04622674
12.86992511	40658.38045	7.549691029	4.338979959
15.45479421	192198.3401	10.65156153	3.074907541
13.03219758	1049907.118	8.279758972	-0.560903758
15.20624414	295671.0867	10.87462499	-0.010174964
13.40051667	1273575.406	8.427434639	-0.554328561
12.93085119	163760.1449	8.720074027	0.960980475
9.621997941	1403593.321	4.8499992	-0.464892358
20.18311428	583292.4144	13.74546863	-0.008646803
18.84722316	1211459.983	13.37075706	0.229784176
16.30009941	603661.3874	10.41396945	3.314912081
14.00103183	310831.4454	8.409459757	-0.256450221
11.9314482	211674.9144	8.105779258	-1.672688484
15.00085507	804621.3042	10.05099729	-1.252036333
14.37731276	2122296.822	8.930526884	-0.604732305
10.17845183	644604.8843	5.971556964	-0.596603662
8.959277275	553987.7832	4.792803272	-0.205661312
13.57185904	62673.02196	9.361891933	0.017644167
15.3396411	671018.3704	9.608440709	0.722633362
18.44974031	201474.3724	13.35644403	1.474810243
14.22821413	1120771.479	9.410000247	-0.54604423
15.69846663	171316.9075	11.05163856	-0.263669699
15.77345282	911033.869	10.03382891	-0.968011618
13.38989758	938825.0869	8.752386431	-1.623731494
13.4719379	333105.8702	9.274109223	-0.509844303
10.95621717	1254951.021	6.45552433	-0.864143342
10.21253355	1419157.224	5.992623677	-1.282108843
12.89162111	3102060.513	7.458993373	-0.058173245
19.58695128	688198.4681	13.58923511	1.152128279
14.75467818	384486.1112	10.32407839	-0.754151583
16.16741766	241422.7064	11.72005846	-1.762944102
14.73418684	614913.9927	10.27806122	-2.026227236
14.62601475	1084294.56	9.50172049	-1.314074576
18.10957223	274946.6277	12.28274189	-2.03085196
22.25279412	1026457.074	15.79530691	1.586394072
18.238964	1808488.722	11.12263794	0.697672427
13.25385827	48408.66578	8.040448526	3.046047449
12.67358297	930906.5142	8.366529234	-1.09781754
10.48909745	1227779.211	6.275929661	-0.749872684
14.68992896	263445.4289	10.37808641	-1.554274797
16.52405731	651226.2686	11.7275582	1.24481529
12.71179604	53464.6625	8.110095591	1.349030495
17.770595	60645.16602	10.34090754	3.197638273
13.60856734	1458437.454	8.29697503	0.138707086
19.50289171	476149.256	12.67428222	-2.196517825
14.48258622	405666.4494	8.945238502	-0.777200758
12.9682382	1312636.452	8.035289665	-0.784623951
11.70227982	1515219.571	7.158459684	-1.242022872
23.95760211	230710.7071	16.54574727	-2.875930786
14.34588643	476747.5619	9.082676533	2.374964714
16.45372061	52154.1432	11.29340581	5.380783081
12.51373849	106388.8064	8.21605463	2.761484861
14.93946818	361645.5662	8.696097582	-2.043223143

log.sigma.0.5.mm.3D_firstorder_TotalEnergy	log.sigma.0.5.mm.3D_firstorder_Maximum	log.sigma.0.5.mm.3D_firstorder_RootMeanSquared	log.sigma.0.5.mm.3D_firstorder_90Percentile	log.sigma.0.5.mm.3D_firstorder_Minimum
9445857.963	81.13062286	22.26057985	30.31360435	-58.52385712
15662156.09	64.94612122	16.71189289	21.72540054	-71.88651276
5999536.389	85.23310852	22.62726687	29.08291473	-47.81594467
46640298.02	105.8319092	25.6040382	34.22403336	-101.5278244
2617924.925	64.31390381	16.4342348	18.14422798	-48.45856857
5258708.483	57.23005676	17.40412443	19.99002876	-66.29355621
2234677.214	43.56546021	15.67150003	17.79631462	-73.83895874
13703444.04	72.01486206	22.50604187	28.24456463	-83.6219635
23276720.53	72.64805603	20.36346926	24.27232285	-77.62229919
30927135.76	92.16703033	19.22858213	23.75427647	-88.96614075
5842416.633	47.91329575	16.17564567	20.50438919	-57.2791481
21746912.54	93.29656982	23.31271315	30.14088917	-119.4449844
14005874.99	83.28890991	15.81498793	20.0570179	-57.24197388
6770437.31	47.1584816	14.97633606	20.48619633	-45.5357666
8408044.811	64.81364441	18.48870267	23.06924057	-73.94306946
28327790.73	94.11368561	12.28926251	13.55495224	-60.78105164
5339174.919	49.21616364	16.8316806	20.55472202	-40.98455048
14974595.01	64.13062286	15.42166936	20.67466621	-61.08520126
5504582.347	56.01098633	18.7162489	25.13233318	-53.88799286
11560465.12	52.30479431	12.3637216	16.39935303	-51.67661285
38414225.38	86.4174881	16.50298762	21.80306702	-72.05196381
14760271.27	90.24645996	17.8279359	23.54006195	-80.88961029
15190704.5	53.3263588	14.35311702	19.77529144	-57.49433899
5993139.315	70.86416626	21.48190375	27.38541412	-72.506073
8807371.529	91.63431549	16.45993186	20.39922047	-49.4400177
7214514.65	56.07378769	19.62193996	24.28129883	-71.80076599
22651605.54	66.01006317	15.55798447	19.42000675	-96.85905457
12954430.45	80.42755127	18.29802365	23.21426506	-76.29851532
19420922.78	62.28901672	20.1874142	27.30957108	-65.03394318
17844999.63	83.46396637	16.12458762	17.89626904	-69.84564972
23985529.26	93.37017059	16.71658292	21.15522728	-60.30437851
24417745.88	73.31259155	12.34933455	16.67771378	-46.8509903
27784012.86	103.9607925	22.4981555	29.22166748	-80.55606079
1515462.781	77.18039703	23.23133063	24.69889164	-62.39176559
5059811.107	62.01934433	23.75834738	28.38111458	-67.50418091
15575587.25	73.58194733	16.89914172	21.42960644	-68.72607422
34357371.66	93.83043671	21.01995783	28.66396313	-74.63368225
34843662.06	74.7700882	19.61545946	26.93517151	-124.2391968
5442560.796	49.11553955	15.53733482	20.01034164	-48.16469955
12488161.46	81.68490601	20.99808195	25.8495079	-85.06654358
14619979.82	63.9926033	19.16649776	25.39045658	-74.43384552
8929436.535	56.33895493	18.62874917	22.66477966	-62.87542725
16088614.11	63.69972992	19.04981924	23.04997311	-75.02350616
11813177.7	81.44208527	20.6604394	25.71263618	-68.3532486
20679219.8	85.46380615	20.19469966	22.99078827	-88.55526733
16534035.71	85.32654572	16.57868043	21.95543518	-53.78623199
63711143.68	98.300354	23.51881267	30.19469452	-125.3987122
6143783.971	64.73183441	19.75612698	24.92877808	-73.75885773
1097776.272	46.60812378	17.41897671	17.63629875	-51.7174263
5189355.182	64.86058044	19.66512095	22.84902573	-80.4817276
28347492.19	76.95682526	17.5109038	21.94796295	-77.76686859
7983119.342	72.61736298	18.90826472	23.19141769	-63.41841125
34386535.95	90.65126801	18.25676448	23.77750587	-114.5437775
4421523.913	56.29354858	16.64601825	22.8760376	-47.88760376
37897019.66	77.99381256	14.66888005	18.55696487	-63.66487122
15748895.19	78.56971741	25.76017012	29.77038078	-72.67342377
32709419.54	89.51168823	23.5036451	29.97417641	-87.49837494
16298857.46	62.80005646	21.60714749	25.1143795	-85.35222626
8392449.027	99.63430023	19.54115689	23.2196394	-74.568367
5715222.69	44.40570068	15.50053693	18.71219635	-89.42987061
21724775.21	69.77724457	19.48634042	25.07175713	-93.5448761
57302014.19	96.03843689	19.68303424	25.47161121	-98.03502655
17404331.88	58.85342026	14.44333739	20.14339943	-85.71231079
14957670.15	56.68120956	14.24259889	16.72921562	-210.2838287
1692171.593	43.04096222	16.99458059	20.26636581	-41.81493378
18117496	83.38166046	20.78650515	28.40413551	-76.23825836
5439808.055	50.8964653	22.61318808	26.99607906	-70.58345032
30260829.92	84.99375916	18.72350785	24.25757751	-97.55940247
4625556.503	50.78414536	19.59894453	22.401124	-73.62727356
24597914.46	100.4499741	21.28439089	27.7990284	-80.29840088
25348277.35	66.90291595	17.57339016	24.08030968	-72.54030609
8993858.496	66.3436203	17.17687567	20.03075867	-69.49563599
33883677.57	87.65601349	15.58303821	20.11622162	-73.95893097
38317245.05	115.7342758	14.536502	18.106287	-63.25128937
83755633.85	90.82828522	18.38246688	22.87111702	-93.57741547
18581358.64	109.5753479	24.8327464	28.40931702	-89.59329224
10381125	65.0719223	18.55298279	21.9388237	-64.36395264
6518413.073	59.46563339	19.8940928	24.63182468	-54.50226212
16602677.8	60.0240593	18.67058378	22.14605274	-77.16628265
29275953.12	94.92162323	19.51893832	24.3588295	-65.04833984
7423558.947	67.11363983	23.35656274	26.15377407	-87.69189453
27714341	95.59765625	27.66661376	34.65264893	-97.23490143
48829195.49	103.4457779	25.45072717	32.57182236	-200.8304901
1307033.976	44.07619476	18.27164107	22.95656853	-59.25199509
25134475.88	75.61521912	16.56872691	22.52511215	-65.34409332
33150038.69	105.4680939	14.97367553	17.97528267	-100.3467102
7113026.58	57.47863007	18.40159784	23.85190792	-73.51063538
17583109.25	57.72363281	20.48425138	24.30905952	-86.07955933
1443545.888	38.71733856	17.09258979	25.49455719	-74.5050354
1637419.482	48.15541077	24.03274869	21.64021568	-80.07722473
39377811.27	75.74828339	19.15469927	23.63285904	-144.3312531
12856029.91	92.47563171	25.96983585	32.94396591	-103.9125748
10952994.13	79.0572052	19.92320013	25.51657562	-81.55809784
35441184.21	97.90415192	17.91474275	21.99871426	-80.59906769
40910928.41	105.659729	16.15471385	21.0860281	-89.76885223
6229189.09	81.97799683	30.19768249	37.51422882	-83.36745453
12872184.17	83.26795959	19.80055908	22.42246437	-105.119957
1408161.866	43.12979507	20.59168338	27.29309082	-63.62779617
2872497.773	65.90842438	16.75438008	19.93877563	-55.05989838
9764430.288	89.42459869	21.16921751	26.50758858	-61.17582321

log.sigma.0.5.mm.3D_firstorder_Entropy	log.sigma.0.5.mm.3D_firstorder_StandardDeviation	log.sigma.0.5.mm.3D_firstorder_Range	log.sigma.0.5.mm.3D_firstorder_Variance	log.sigma.0.5.mm.3D_firstorder_10Percentile
1.954095041	22.26053495	139.65448	495.5314163	-27.06592178
1.62891482	16.71185753	136.832634	279.286182	-19.46140213
1.90623886	22.62723538	133.0490532	511.9917809	-26.27025719
2.116923191	25.5658417	207.3597336	653.612262	-29.30871391
1.49673086	16.42016737	112.7724724	269.6218963	-20.42662163
1.630727303	17.36491976	123.523613	301.5404381	-23.75305824
1.498907913	15.66807711	117.4044189	245.4886404	-18.17741356
1.952209465	22.50583711	155.6368256	506.5127041	-29.65803108
1.832142906	20.3461712	150.2703552	413.9666824	-26.60556755
1.751256194	19.2197873	181.1331711	369.400224	-22.04063263
1.530182264	16.14793362	105.1924438	260.7557603	-21.39930573
1.969788867	23.30822707	212.7415543	543.2734489	-26.12748108
1.536143639	15.79708696	140.5308838	249.5479565	-18.27594433
1.456378438	14.96611537	92.6942482	223.9846093	-17.64897671
1.684836254	18.46964284	138.7567139	341.1277066	-21.52389336
1.373056376	12.28748301	154.8947372	150.9822388	-11.58536739
1.580582484	16.82770349	90.20071411	283.1716048	-22.56227837
1.511849389	15.41693542	125.2158241	237.6818977	-18.41071987
1.727880697	18.67675963	109.8989792	348.8213503	-23.58110046
1.324553506	12.36151207	103.9814072	152.8069806	-14.45614815
1.582751121	16.48287266	158.4694519	271.6850913	-19.62281399
1.665587578	17.7977462	171.1360703	316.7597698	-19.69875546
1.393671544	14.34985682	110.8206978	205.9183908	-17.32282257
1.868571843	21.46037966	143.3702393	460.5478953	-26.90705109
1.536681781	16.35726029	141.0743332	267.5599642	-20.65033817
1.816679787	19.5937608	127.8745537	383.9154621	-25.35078945
1.535473433	15.55658047	162.8691177	242.007196	-17.77392673
1.6785863	18.25147499	156.7260666	333.1163394	-21.8718544
1.810730314	20.13908811	127.3229599	405.5828697	-23.59007759
1.580343955	16.07567201	153.3096161	258.4272306	-19.89382534
1.565026484	16.67872521	153.6745491	278.1798746	-20.3329834
1.300442984	12.33335051	120.1635818	152.1115349	-13.58463364
1.966216524	22.4912188	184.5168533	505.8549232	-27.49824944
1.929835985	23.18225378	139.5721626	537.4168902	-31.0866457
2.033552383	23.73407716	129.5235252	563.3064187	-28.86806183
1.631599446	16.89696839	142.3080215	285.5075409	-22.29402142
1.861005794	21.01897952	168.464119	441.7974999	-24.95297031
1.774219237	19.61334849	199.009285	384.6834392	-22.62278328
1.46611631	15.50092878	97.28023911	240.278793	-19.20895805
1.876909668	20.99661352	166.7514496	440.8577795	-26.445858
1.791496862	19.1658537	138.4264488	367.329948	-23.38226204
1.708188828	18.62256632	119.2143822	346.7999764	-24.09572029
1.745948265	19.04266381	138.7232361	362.6230448	-26.20301418
1.857943244	20.65820452	149.7953339	426.7614142	-26.06617661
1.817585232	20.18095909	174.0190735	407.27111	-27.09847851
1.587524613	16.57867879	139.1127777	274.8525906	-19.58551579
2.00907406	23.50643957	223.6990662	552.5527014	-27.06815052
1.79570203	19.74033818	138.4906921	389.6809516	-24.39582863
1.554201134	17.41314507	98.32555008	303.2176213	-23.88741531
1.744653337	19.65761019	145.342308	386.4216382	-28.07688446
1.670898797	17.51089461	154.7236938	306.6314302	-21.92506161
1.752599182	18.86928633	136.0357742	356.0499667	-26.65694809
1.735201456	18.25675169	205.1950455	333.3089822	-22.94212151
1.586626741	16.62949436	104.1811523	276.5400827	-20.42740059
1.529165859	14.66455965	141.6586838	215.0493099	-16.02021179
2.130193707	25.75078276	151.2431412	663.1028129	-35.19486389
2.028391765	23.50281416	177.0100632	552.3822736	-29.72616768
1.883566066	21.59342602	148.1522827	466.2760472	-29.69845047
1.786140479	19.53539969	174.2026672	381.6318411	-24.01250858
1.47174569	15.49928355	133.8355713	240.2277905	-17.83544731
1.792217679	19.4796467	163.3221207	379.4566354	-24.51717567
1.797750597	19.67858742	194.0734634	387.2468028	-23.48541851
1.457660904	14.41746565	144.565731	207.8633158	-15.08846331
1.411769532	14.24184744	266.9650383	202.8302186	-15.93081474
1.655945152	16.99453955	84.855896	288.8143745	-22.47249069
1.877714764	20.7198418	159.6199188	429.311844	-22.81855469
1.949461259	22.59628959	121.4799156	510.5923034	-31.75435066
1.739717739	18.72344802	182.5531616	350.5675059	-23.12419167
1.768345969	19.5646866	124.4114189	382.7769617	-27.79995346
1.906052844	21.28376806	180.7483749	452.9987829	-25.23385048
1.648072437	17.57158986	139.443222	308.76077	-20.83634911
1.629602778	17.15876154	135.8392563	294.4230975	-23.01161156
1.533017312	15.58291897	161.6149445	242.8273636	-17.77792587
1.455673536	14.51723203	178.9855652	210.7500257	-17.70372391
1.733388851	18.37263807	184.4057007	337.5538295	-20.60174122
2.065937693	24.82212816	199.1686401	616.1380466	-32.66645622
1.724083114	18.53398901	129.4358749	343.5087484	-24.50073433
1.796275241	19.83623955	113.9678955	393.4763997	-26.94030209
1.7089895	18.61014156	137.1903419	346.3373689	-24.27852688
1.783694976	19.51046845	159.9699631	380.6583791	-24.89599419
1.983887768	23.26800369	154.8055344	541.3999959	-29.75456829
2.232555035	27.65981296	192.8325577	765.065253	-35.25646973
2.115239148	25.40666609	304.276268	645.498682	-29.22593403
1.618642784	18.13083029	103.3281898	328.7270069	-21.03141632
1.59466775	16.56536553	140.9593124	274.4113352	-19.20063972
1.462750228	14.97364424	205.8148041	224.2100217	-16.22936916
1.662936866	18.39482248	130.9892654	338.3694942	-22.65792599
1.829642462	20.47944761	143.8031921	419.4077745	-28.63528919
1.720106076	17.01656333	113.222374	289.5634276	-20.50022812
1.875386674	23.90902623	128.2326355	571.6415353	-30.76960144
1.760319297	19.15441112	220.0795364	366.8914652	-22.54400673
2.131729406	25.93739933	196.3882065	672.7486842	-30.76359367
1.841569061	19.91930226	160.615303	396.7786027	-25.64975147
1.691132389	17.91290099	178.5032196	320.8720218	-21.68829937
1.560515454	16.15443977	195.4285812	260.9659243	-18.05870724
2.368117775	30.16792547	165.3454514	910.1037273	-36.74106674
1.731663919	19.78634088	188.3879166	391.4992854	-22.34192181
1.796940068	20.51215331	106.7575912	420.7484334	-23.61224518
1.518278633	16.6545246	120.9683228	277.3731898	-18.38662605
1.875053738	21.15944852	150.6004219	447.7222616	-25.06129189

log.sigma.0.5.mm.3D_firstorder_Kurtosis	log.sigma.0.5.mm.3D_firstorder_Mean	log.sigma.0.5.mm.3D_glrlm_ShortRunLowGrayLevelEmphasis	log.sigma.0.5.mm.3D_glrlm_GrayLevelVariance
3.067530909	0.044710603	0.09239004	1.03594508
4.766822333	-0.034380248	0.076957692	0.690330528
4.369533143	-0.037751351	0.232168929	1.137127069
3.813326287	1.398038034	0.031080766	1.314710787
3.911307774	0.67983614	0.179604587	0.580395279
3.128624212	-1.167522619	0.08074042	0.684580916
5.301518699	-0.327525325	0.093094823	0.595258071
2.966974835	-0.096002147	0.048428952	1.023898117
3.355164261	-0.839165079	0.046838287	0.897677122
4.904042928	-0.581503845	0.044695053	0.855467947
2.876482191	-0.946442134	0.077723904	0.58790835
4.937870284	-0.4573244	0.031834512	1.176456182
4.007503148	0.752254328	0.071849433	0.601918812
2.923966867	0.553201908	0.165231624	0.533637528
3.783771387	0.839297121	0.084409825	0.719451602
9.405850243	0.209127228	0.067709781	0.475967873
2.701064646	-0.365878253	0.2003173	0.638763096
3.654039143	0.382084059	0.06766173	0.581631119
2.826011095	1.215163631	0.076173867	0.76538341
4.447326433	0.23373337	0.063254248	0.433358272
4.386878109	-0.814560672	0.074213889	0.68128014
3.973587383	1.037077017	0.041068263	0.72171809
3.405035832	-0.305904265	0.065073929	0.509221296
3.399801053	0.961401794	0.095566627	0.938609955
4.808517238	-1.835590506	0.18509149	0.652721825
3.654103109	-1.051221035	0.093167134	0.902268514
5.587955598	-0.209009437	0.040656986	0.619001472
4.601091329	-1.30435044	0.042928779	0.804790791
3.029919342	1.396002229	0.085436421	0.844002011
5.445527209	-1.255027948	0.075606412	0.675381262
5.142980983	-1.124397589	0.072968313	0.685214105
4.675170672	0.628115387	0.141794412	0.419959279
3.867641981	0.558639349	0.049163714	1.068033634
3.932877632	1.509249016	0.110235389	1.095220748
2.85606188	1.073616135	0.109665423	1.144657457
3.154452796	-0.271016724	0.078940675	0.67826726
3.911883341	-0.202799137	0.087196193	0.970930781
3.908615644	0.287768499	0.028248544	0.839732925
2.995993446	1.063005367	0.175236492	0.533458095
3.649756332	-0.248326783	0.048001235	0.959278212
3.966830795	-0.157125046	0.086999718	0.852266738
3.096559445	-0.479915796	0.082146308	0.76055418
3.018191716	-0.522080749	0.045730502	0.784804797
3.467893761	0.303878199	0.090451688	0.923472204
3.660185213	-0.744838536	0.048374579	0.885545986
4.130509909	-0.007370871	0.073377511	0.651117582
4.37629356	0.762789695	0.021174245	1.160134042
3.284101789	-0.789684432	0.090706519	0.864956082
4.164421682	0.450697627	0.096201915	0.670818847
3.463474253	0.543455452	0.048531848	0.8161524
3.974792035	-0.017933	0.042811309	0.714128276
2.920701485	-1.213469475	0.088342081	0.793808066
4.303394482	-0.021612528	0.027946507	0.783727948
3.278831823	0.741512638	0.179753108	0.621347608
5.381349291	0.355994327	0.073802134	0.560886439
3.04083567	-0.695378758	0.120979877	1.304054585
3.026232977	0.197634423	0.048452054	1.132428687
3.722176609	0.769919021	0.050426999	0.98480357
4.915121445	-0.474311545	0.094593182	0.861640159
5.061060434	-0.197115952	0.041513853	0.581839424
3.568067086	-0.510712911	0.045195639	0.854065019
4.475510102	0.418370629	0.045595584	0.871540006
5.335589179	0.864105978	0.040342787	0.540474807
30.81458625	-0.146303335	0.008676348	0.565201916
2.803230536	0.037348871	0.205499235	0.670193871
3.9537719	1.663415798	0.045144167	0.919579011
2.656521371	-0.874054772	0.107615486	1.011143695
3.880738664	0.047331668	0.043896387	0.784904138
3.068809089	-1.158302615	0.091265522	0.808311163
4.081500338	0.162826616	0.047507933	0.991419215
3.517390693	-0.251538652	0.075776548	0.704303141
3.442140153	-0.78864458	0.078826232	0.680712146
4.913043933	-0.060961429	0.072877679	0.617640739
5.453631602	-0.748241039	0.070564326	0.546015042
5.150183702	0.601048321	0.045403131	0.801466434
3.524136838	-0.726118032	0.057390378	1.208355896
3.280320738	-0.839298418	0.082829619	0.77067825
2.605161256	-1.516089988	0.086486037	0.841836854
3.210730583	-1.501109566	0.044839352	0.785812021
3.975861099	-0.574955532	0.086218386	0.874727739
3.520546357	-2.032000808	0.051953152	1.113783906
3.046648441	0.61340345	0.060463177	1.454868478
5.67532148	1.496940754	0.010033251	1.297600006
4.010318871	2.264036348	0.086106567	0.700496482
3.579974357	0.333730619	0.074294255	0.646680703
7.136948833	0.030612747	0.025128011	0.578581138
3.190472886	-0.499308555	0.079006203	0.713871989
2.893706955	-0.443598967	0.048269219	0.879986497
4.448134562	1.610340974	0.081153813	0.728735936
4.656687343	-2.43546185	0.086142778	1.274773787
6.509402701	0.10506697	0.020670238	0.84661573
4.027673218	-1.297570682	0.033805363	1.38412704
4.10781247	-0.394082213	0.047810893	0.90855149
4.973975321	-0.256877457	0.043860802	0.770154391
5.119523615	0.09410295	0.040178577	0.651251226
2.933658417	-1.340261392	0.063727284	1.764748469
6.770908134	0.750236208	0.030539935	0.8832522
3.055325175	1.80803512	0.085480837	0.795516358
4.529080005	1.826488982	0.080489279	0.628476658
4.991124484	-0.643045986	0.086204238	1.033597014

log.sigma.0.5.mm.3D_glrlm_LowGrayLevelRunEmphasis	log.sigma.0.5.mm.3D_glrlm_GrayLevelNonUniformityNormalized	log.sigma.0.5.mm.3D_glrlm_RunVariance	log.sigma.0.5.mm.3D_glrlm_GrayLevelNonUniformity
0.111038048	0.272790789	0.552620313	143.4197826
0.100804985	0.347941141	0.87270347	483.1437381
0.276683367	0.276218406	0.651194318	88.57964319
0.037557832	0.253607384	0.511493242	505.4758552
0.2263376	0.382946041	0.724156985	92.9791879
0.102646351	0.341792573	0.735361941	150.9621655
0.112343543	0.382778914	0.623088004	92.02490035
0.05935642	0.275172709	0.633836667	201.0847096
0.05926268	0.298501632	0.711124018	434.6886317
0.057799264	0.317654914	0.801866405	677.3129441
0.100558659	0.364869873	0.867250396	204.0009528
0.039240066	0.277313267	0.66087577	297.2591609
0.09518742	0.366119013	0.945611805	502.5503078
0.220781833	0.379004192	1.117649915	263.9439941
0.10361402	0.336201897	0.586020352	220.0148534
0.091996423	0.416695773	0.852046484	1932.047377
0.253135258	0.34523018	0.915145704	159.2339328
0.092948418	0.369677	1.086756731	551.6042094
0.09788529	0.317798276	0.727822186	127.1116883
0.090118149	0.420027979	1.18961419	736.1852849
0.09880373	0.345897074	0.926813621	1204.657757
0.05389073	0.337742447	0.817068452	398.1460385
0.092139899	0.390589635	1.185574689	671.4654156
0.113671827	0.291951483	0.592874337	102.3401809
0.24108302	0.35869426	0.95762641	287.6005836
0.115580095	0.30021778	0.73501905	145.7255812
0.05501062	0.367670195	0.890903353	839.36773
0.056490585	0.326344421	0.886508475	320.1296375
0.104965535	0.30199133	0.72884534	382.3876093
0.101195933	0.351760125	0.961941904	588.9033463
0.097382398	0.348498286	0.991190503	742.4432209
0.197207878	0.426367846	1.154731998	1597.796176
0.060053001	0.276694753	0.619157256	410.0332314
0.125432491	0.307086143	0.31128825	25.67912473
0.126262492	0.262414602	0.41532707	67.69010514
0.102118396	0.337073173	0.861932343	456.4696249
0.107571795	0.292195893	0.711819038	606.6254409
0.03609493	0.308908556	0.750459203	729.5834551
0.223910079	0.380316537	0.848940885	211.9697237
0.059221107	0.289399452	0.660441309	218.937187
0.108380006	0.307533523	0.736838018	316.632857
0.10475524	0.324443585	0.820559821	210.013658
0.057990675	0.316378014	0.729834627	363.1887302
0.111432289	0.29874313	0.6978073	216.7566278
0.060253137	0.306413991	0.637831951	410.2718067
0.094789129	0.35217998	0.810768735	547.4172295
0.026108751	0.271705005	0.569777194	852.4540039
0.11066927	0.307860588	0.697379949	127.4110563
0.114401693	0.38117924	0.469387052	37.77338371
0.059814144	0.323222074	0.601526276	115.3746962
0.055226596	0.337326711	0.717378072	807.8741336
0.10910026	0.30975081	0.674847017	182.9889949
0.036019083	0.321412535	0.768140424	855.4186072
0.224331404	0.357323046	0.652889244	148.251509
0.094073403	0.378766896	0.718011615	1760.251145
0.139379483	0.245644783	0.457198486	165.6528934
0.059697209	0.260333823	0.632533493	418.3613001
0.061132527	0.294333736	0.548154358	278.8549886
0.115492967	0.313183378	0.639261787	181.0308681
0.056067348	0.37773466	0.985479309	216.8605854
0.057473319	0.303319341	0.741891932	450.8668814
0.057409272	0.311893608	0.742793938	1206.428897
0.053330179	0.390482562	0.843685189	824.6844276
0.011853934	0.402877605	1.074088548	703.038701
0.257310216	0.338798754	0.521649212	52.89070046
0.055658806	0.300352372	0.561046057	343.9436705
0.124992742	0.277049361	0.395662073	84.78913761
0.056093744	0.320305514	0.800637234	712.7842127
0.110990623	0.310791376	0.596833464	99.94286479
0.058329746	0.290137684	0.601012688	429.8731907
0.098792245	0.333922978	0.857534424	695.0437987
0.101685134	0.345264815	0.867498746	263.474699
0.096883501	0.368674454	1.020222468	1267.805702
0.095269754	0.385363141	1.049264131	1697.996874
0.057384843	0.332260739	0.711272331	2141.691535
0.067329267	0.260233034	0.451051287	221.8505244
0.104675574	0.322131349	0.785868844	250.0214653
0.108594886	0.298739685	0.671454088	129.761946
0.058426216	0.31838376	0.940800793	379.2350612
0.108297102	0.30400233	0.813389036	600.9057018
0.063870793	0.270918582	0.597673683	100.0225314
0.069010778	0.231822999	0.364791605	247.7224512
0.011859527	0.26381458	0.41340933	577.1879274
0.104715279	0.355350508	0.504439477	37.25003764
0.096787136	0.348178862	0.891220246	804.6424403
0.035006696	0.389128123	1.023825425	1357.897647
0.101139064	0.33101303	0.798590227	176.7897
0.059642398	0.298809962	0.63050466	334.7401481
0.098828493	0.326334086	0.465691271	44.90466165
0.093662489	0.322760391	0.334222645	27.49787583
0.026114046	0.32446995	0.713649424	911.1882811
0.040430339	0.245415507	0.49805954	131.5553064
0.059134515	0.299842672	0.624160127	221.7291899
0.056821931	0.332130416	0.914233583	921.3298197
0.054142614	0.357265801	0.992704802	1369.87943
0.072975311	0.209675284	0.308863901	42.70555319
0.038671132	0.326725133	0.729542628	272.1632342
0.102004234	0.315632624	0.352016901	30.20492034
0.102317033	0.382080475	0.679383876	97.40232085
0.10760297	0.292271429	0.652313541	168.0123267

log.sigma.0.5.mm.3D_glrlm_LongRunEmphasis	log.sigma.0.5.mm.3D_glrlm_ShortRunHighGrayLevelEmphasis	log.sigma.0.5.mm.3D_glrlm_RunLengthNonUniformity	log.sigma.0.5.mm.3D_glrlm_ShortRunEmphasis
2.367269666	11.35380454	322.7897725	0.810726543
3.131674485	10.11541148	723.9975006	0.74665881
2.501596161	6.78121004	199.8158272	0.816059156
2.269879493	26.83147034	1245.121115	0.817417975
2.956699001	5.304141366	125.6771828	0.740368553
2.882018595	9.921977143	237.6444358	0.75813403
2.646900358	9.327207286	135.9104927	0.774219927
2.530928107	17.47489204	437.7878038	0.800192873
2.76459872	16.2949794	808.6516046	0.771724365
2.93229098	16.73570829	1163.874519	0.765150715
3.102642022	9.680249892	295.5075526	0.751924898
2.591552055	25.62554144	636.0001809	0.796394134
3.268101739	9.931485236	700.3947364	0.738124221
3.780603803	5.080358047	328.9125765	0.704732723
2.531649646	10.37368755	373.6370276	0.783188045
3.137102736	9.711284534	2381.322184	0.738013146
3.271129623	5.156771653	235.2882047	0.73604433
3.598296565	9.899125523	737.1047163	0.722425962
2.885922735	10.50620743	216.0451958	0.756851164
3.839789894	9.418200325	835.2177374	0.705185966
3.191104136	10.21281402	1816.090042	0.748821764
3.014469	16.77322574	635.7166798	0.756393723
3.80354868	9.707688506	837.6940938	0.715915089
2.500718629	10.68862474	206.360832	0.79345959
3.253142875	5.489436892	422.3556378	0.750017297
2.809542087	10.48628504	271.9330392	0.773373678
3.212291608	15.83273454	1150.849476	0.734798704
3.061047049	16.81359708	534.3758514	0.765069678
2.781591996	10.75617263	748.0622677	0.785665096
3.295683971	9.828710535	850.7101161	0.737398992
3.255487735	10.36797638	1136.109558	0.755862565
3.744093471	5.071859552	1815.460317	0.712792584
2.52627832	17.30929174	879.9828644	0.796192253
1.887186361	11.70584079	56.36272584	0.843951757
2.10029095	11.52309241	167.4487049	0.829775783
3.115332379	9.672206836	701.7817565	0.744646367
2.65592611	11.09741067	1229.013439	0.796341702
2.78012249	24.94821146	1333.528799	0.77886586
3.168004603	5.148648989	283.4419662	0.734710994
2.601548356	17.1212519	444.463381	0.793386831
2.810154648	10.47148403	575.2515071	0.773628756
3.019381592	10.01267	345.3735474	0.755478484
2.821124942	16.09411025	638.2916333	0.769241879
2.721700929	10.5962661	411.6410846	0.778199987
2.629658789	16.16142915	756.1418501	0.777557834
2.914418038	10.61830578	871.1624022	0.772974473
2.426038723	35.52568496	1872.554476	0.800486029
2.706526001	10.70636812	238.5115136	0.786105634
2.328540034	9.954310809	58.24075154	0.791766643
2.561409177	16.23354347	203.7649191	0.78131594
2.783347688	16.50334104	1322.489221	0.768555742
2.684805676	10.08928942	340.9611996	0.783180443
2.850845484	24.43977483	1477.731516	0.771006602
2.702727798	5.727065696	228.5121513	0.767378601
2.720434541	10.36654439	2679.596515	0.782317219
2.167003947	11.25303469	430.1155633	0.825428528
2.544310978	17.68615737	969.1969276	0.799011037
2.43587164	16.76384527	558.1504056	0.792932833
2.636877049	10.4173136	328.5841184	0.779867448
3.398571155	15.94611273	288.0855062	0.732407289
2.793166142	16.76821494	833.7579279	0.775912416
2.770588532	16.85052705	2197.927547	0.780204794
3.049321932	16.29906425	1136.583803	0.754807727
3.559604676	65.77800475	846.0275007	0.71834213
2.47268856	5.589123655	87.69821183	0.773502516
2.425745579	17.81658755	684.6814624	0.798495846
2.077894141	10.81980191	196.595226	0.826009684
2.889339423	16.76446945	1243.915246	0.773496557
2.532319186	10.25033534	187.009261	0.789496777
2.462250869	17.66181932	895.527485	0.803244324
3.043102693	10.40098992	1126.158694	0.759497804
3.097157059	9.977400077	408.3206317	0.756106007
3.324019577	10.15752748	1803.425931	0.747514487
3.402313519	9.851650777	2253.554101	0.739322028
2.750660419	16.51775583	3573.849134	0.771068048
2.188567766	17.13530193	536.1406964	0.818051728
2.901205366	10.21318973	430.8791313	0.770253697
2.676712485	10.36243663	247.9689681	0.78062677
3.206366581	16.10327876	635.0730299	0.754386378
2.91103534	10.39459096	1102.637859	0.773541376
2.505338388	17.06879888	220.3266843	0.797231298
1.947089677	18.82853067	722.3909665	0.847913688
2.05445158	78.01474508	1442.11002	0.837722092
2.462817425	10.65141251	59.56809795	0.77518697
3.08459512	10.21517257	1246.280376	0.758094789
3.511741475	22.61014428	1674.281788	0.715959956
2.942136377	10.38977805	290.8050995	0.763757696
2.57925294	16.50126454	653.0293083	0.789307454
2.342914878	11.02865466	85.47775929	0.798107056
1.904017576	17.22534601	58.70634798	0.851450069
2.738093454	33.72840384	1582.370097	0.776694913
2.243919754	26.42251318	339.2886642	0.822200039
2.546236165	17.04244834	433.9805977	0.793450196
3.119951742	16.26424463	1491.831671	0.758763806
3.309807113	16.38754886	1976.922187	0.743455964
1.859910741	18.63625869	137.8385568	0.848180715
2.880731518	23.11651267	441.1118625	0.753047349
2.020746895	11.58763741	60.78323763	0.822433756
2.921982073	9.5325786	128.5966573	0.732004582
2.642133528	10.90166663	327.5247282	0.781159752

log.sigma.0.5.mm.3D_glrlm_LongRunHighGrayLevelEmphasis	log.sigma.0.5.mm.3D_glrlm_RunPercentage	log.sigma.0.5.mm.3D_glrlm_LongRunLowGrayLevelEmphasis	log.sigma.0.5.mm.3D_glrlm_RunEntropy
29.12786267	0.744388756	0.249658898	3.072535222
37.67507576	0.668382652	0.306086996	3.061720511
15.21035388	0.738036157	0.644622366	3.029402717
70.36406773	0.756035615	0.083028807	3.23139281
20.49100016	0.675808871	0.585907137	2.873949318
35.42064169	0.686206484	0.276388482	2.973212114
35.19918682	0.71239443	0.244413722	2.744624888
52.36570688	0.728773223	0.142876476	3.135101933
56.13093429	0.7000407	0.154785844	3.149812092
57.10739113	0.687788648	0.16843068	3.120189507
37.28392321	0.675006976	0.29779198	2.915125536
76.91957157	0.723087304	0.098181329	3.203612397
40.50104161	0.661189823	0.301887123	2.993618328
23.78505349	0.622677859	0.774897186	3.0263722
32.29116447	0.71814574	0.240291495	2.928811035
37.24618609	0.667128035	0.295695706	2.812915291
21.01688286	0.659576813	0.706826686	3.00484372
43.37592269	0.638903549	0.338095136	3.042802488
37.18581533	0.686888713	0.268227478	3.050285036
45.33671494	0.625490896	0.359849978	2.893133731
36.16213003	0.666318176	0.322226041	3.024124788
61.63108823	0.684660107	0.162055552	3.036378299
42.74704316	0.6288201	0.374047759	2.954298566
34.04654403	0.727970574	0.23934221	3.063531717
18.15548593	0.665154613	0.760743149	2.968096934
33.18805897	0.698736422	0.297868666	3.090776317
63.04075277	0.658395845	0.177900774	3.016992362
57.41218931	0.683289495	0.178831632	3.042584586
36.26694042	0.716801046	0.266931751	3.042692612
37.67553137	0.658022151	0.331238875	3.057359178
36.22859311	0.669199313	0.331295282	2.986722472
22.85415034	0.631249189	0.748642209	2.861408435
52.81310351	0.728253055	0.141510626	3.179699308
25.96808058	0.802514793	0.207862677	2.780663459
27.78739168	0.775718258	0.230136509	3.024873963
38.62754088	0.669725819	0.295499731	3.049330387
31.62186444	0.720405983	0.277071247	3.099623038
82.7121137	0.703706252	0.100708923	3.075767853
22.18615572	0.66605251	0.602491589	2.882641137
52.42629711	0.720539708	0.147552658	3.10039527
34.6257676	0.697943847	0.281167459	3.112902574
37.04036796	0.678343692	0.294677878	3.089115961
58.16062271	0.698023049	0.154039858	3.061107318
34.32351076	0.70739212	0.275251284	3.133750815
54.49813659	0.712214303	0.145319705	3.094667919
34.32983893	0.696485292	0.283364854	2.897929504
103.661325	0.735223052	0.061635319	3.20909055
33.18206013	0.708932577	0.273404659	3.043894981
31.49711973	0.738231917	0.22192633	2.683826292
54.84941047	0.717535985	0.137611732	2.986485909
56.067071	0.699069914	0.152291607	2.994822394
33.23640296	0.713142963	0.267554051	2.983038986
85.46821952	0.69615284	0.102161448	3.067453127
18.06022894	0.701418717	0.556249273	2.87342477
32.96858344	0.712013113	0.258689026	2.790612586
27.621332	0.766780432	0.259663102	3.175055879
52.74174518	0.731909222	0.145635533	3.204716926
53.13898649	0.732048307	0.132687992	3.081828494
32.46339451	0.70979021	0.271726518	3.05329249
66.00364714	0.650571903	0.190621149	2.932592567
55.43583716	0.700983773	0.157703925	3.09101287
56.25864337	0.705633724	0.153858084	3.108358266
60.78244109	0.682474483	0.165023776	2.841574944
318.0252934	0.638509422	0.041062404	2.952490011
15.7142777	0.719248493	0.594553576	2.807866339
51.35657001	0.736688296	0.131392577	3.057543776
26.67328537	0.775868801	0.23042855	2.944036
58.2717959	0.695435625	0.159476389	3.068821681
30.99820816	0.720593308	0.256933965	2.962316719
49.82132348	0.736334774	0.140199704	3.086954607
36.05271835	0.683704453	0.30065672	3.015930923
37.43449514	0.675001703	0.300842178	3.021279548
38.79099054	0.664607645	0.325346128	2.983671519
39.30453098	0.655598571	0.331187846	2.932624047
57.34061949	0.701935646	0.148595251	3.066291577
45.79811674	0.763027295	0.13130768	3.136483301
35.6257885	0.693547276	0.284566241	3.049782779
31.57689942	0.711223203	0.279217794	3.00949847
62.35724493	0.673425781	0.182643655	3.097072918
34.47044767	0.693794259	0.297511619	3.121939298
48.99804032	0.731074481	0.151987921	3.14276677
41.84781891	0.796650032	0.121407946	3.171495442
188.6544604	0.783281904	0.023771164	3.128621956
34.38731419	0.721485411	0.220486224	2.785591518
36.98879613	0.680852029	0.296959556	2.973955976
104.6621643	0.636862954	0.123804115	3.036120363
35.01148124	0.68597983	0.290559985	2.992761993
53.06193082	0.720707772	0.144274943	3.050370558
31.40220166	0.750315259	0.216552606	2.766519269
41.05283611	0.807326007	0.13756868	2.726095986
116.3789589	0.705970005	0.068825946	3.068829168
66.48824729	0.758988886	0.08719244	3.201515742
50.9143669	0.723242511	0.145146829	3.06445606
62.41345527	0.67752492	0.172385123	3.103836414
64.35079391	0.659927396	0.184376968	3.026004864
38.40404858	0.804803892	0.125313405	3.233976311
91.54900159	0.684400304	0.099887203	3.118136545
27.2177916	0.777360851	0.195164798	2.740995756
39.05264941	0.671808403	0.258394336	2.929817683
32.43272802	0.711752931	0.267502777	3.147941231

log.sigma.0.5.mm.3D_glrlm_HighGrayLevelRunEmphasis	log.sigma.0.5.mm.3D_glrlm_RunLengthNonUniformityNormalized	log.sigma.0.5.mm.3D_glszm_GrayLevelVariance
13.6485194	0.612817286	3.729224377
13.1614633	0.519245844	3.192906574
7.804151901	0.622054954	2.981070849
32.42401602	0.623588287	5.211927391
7.113102669	0.51341632	2.339100346
12.92096065	0.535970105	2.425094646
12.35155666	0.560662736	2.693333333
21.5857908	0.597302476	3.518698061
20.90715005	0.554025935	3.360686782
21.28717015	0.544306173	3.489439058
12.69054829	0.526301838	2.317460317
31.62208394	0.59104202	5.042735043
13.19790903	0.508077991	2.555729984
7.00508703	0.467110573	2.050453515
13.14546665	0.570213018	2.768175583
12.8270327	0.510273037	2.727887111
6.8875627	0.506279819	2.08984375
13.28751308	0.489186852	2.661376711
13.66194727	0.535813476	2.249657064
12.9621006	0.470489485	2.200050391
13.05660108	0.520705509	2.576216713
21.74432362	0.534533602	2.908093278
12.9719249	0.481588989	1.867566791
13.47092712	0.586784887	3.336076818
6.870742334	0.524197106	2.381012392
13.1475613	0.557569612	2.900826446
21.06165692	0.502899878	2.910304713
21.16027266	0.543613323	2.809632678
13.53973752	0.581380315	2.445982314
12.83170517	0.506654306	3.081512314
13.06000984	0.531308576	2.417418199
6.848839027	0.478489065	1.978584176
21.56206174	0.591325861	4.555789474
13.81258307	0.671778558	3.01
13.72675697	0.645980242	3.269916766
12.86114913	0.516491484	2.463718821
13.46632156	0.59037984	3.422336838
31.53366556	0.563831572	3.440311419
6.979429436	0.50557305	1.924701561
21.2764363	0.586167452	3.551715976
13.25134907	0.556673657	3.807526573
13.0531606	0.531493016	2.702292899
20.85566363	0.552147778	2.747130102
13.38751355	0.564420942	3.555555556
20.73668206	0.562944269	3.636114106
13.28223972	0.556673461	2.389245542
44.00788848	0.596215935	4.787277625
13.3146452	0.574856255	3.166204986
12.76398814	0.585616295	2.6875
20.89512516	0.569251963	3.122473246
21.15080723	0.550169944	2.88542227
12.751495	0.572916779	1.757785467
31.29266669	0.553192555	3.259695291
7.281314387	0.549090301	2.446280992
12.99279596	0.572259291	2.431210938
13.4391029	0.636732256	3.860833333
21.78419148	0.59746335	3.989135355
21.26978233	0.58649952	3.444951399
13.11670801	0.566700212	3.3875
21.17133986	0.499509545	2.453598914
21.20933299	0.559616494	3.0811
21.31000936	0.566359057	3.354431152
21.2060535	0.532851222	2.698395529
91.06833884	0.482560763	4.303819444
7.005076888	0.559466105	1.109375
22.08983662	0.595163083	3.963681133
13.04272694	0.639467311	2.987654321
21.33253106	0.556940113	2.919177204
12.85686492	0.580406671	2.635160681
21.61241609	0.602057936	3.825560698
13.25471248	0.537419422	2.520662559
12.9402656	0.532237845	2.480584391
13.11340278	0.521331995	2.719128804
12.88145871	0.509406402	2.553801605
21.2639342	0.553337772	3.889275937
20.95668235	0.626066936	4.809917355
13.01373483	0.552472765	2.30994898
12.91571277	0.568388589	2.541103018
20.88325389	0.52990787	2.768276705
13.06051414	0.556283589	2.946523438
20.93985381	0.593521981	3.183837429
22.05352642	0.674780612	4.817089581
92.86805223	0.657454468	5.795915257
13.74031924	0.564045457	2.234375
13.13030508	0.535942651	2.363247863
31.10304934	0.478466875	3.106775
13.22226288	0.542863137	2.471470221
20.85271536	0.580628106	3.206313776
13.70670019	0.605712645	1.888888889
20.51585603	0.68532865	4.094674556
43.24771076	0.561599844	4.428176574
31.54282516	0.631713836	4.863406795
21.14842709	0.585840205	3.690844233
21.09754714	0.535601876	3.172698001
21.39045369	0.514089915	2.956334265
21.66844468	0.675440956	4.503826531
31.02575763	0.528246515	3.649722222
13.89483243	0.633227389	2
13.16555083	0.502170427	3.169550173
13.49329466	0.568180171	3.384083045

log.sigma.0.5.mm.3D_glszm_SmallAreaHighGrayLevelEmphasis	log.sigma.0.5.mm.3D_glszm_GrayLevelNonUniformityNormalized	log.sigma.0.5.mm.3D_glszm_SizeZoneNonUniformityNormalized
11.92168837	0.191135734	0.414127424
11.74520296	0.23399654	0.385380623
8.904168558	0.269875608	0.366143862
26.25390834	0.178325301	0.44056574
5.53795776	0.314878893	0.280276817
9.896091338	0.342347215	0.302325581
8.677492624	0.28	0.28
13.29803468	0.209141274	0.27700831
16.75212627	0.275849009	0.381643732
15.99970357	0.261599723	0.337603878
10.44805351	0.430839002	0.357142857
19.90378398	0.175542406	0.380013149
9.608401904	0.398864871	0.3082961
4.333733062	0.416099773	0.27324263
8.879040482	0.307270233	0.341563786
10.45656669	0.304606401	0.264705882
3.709217469	0.44921875	0.2421875
9.029003358	0.35766713	0.281888514
5.288356904	0.319615912	0.135802469
5.710923372	0.36558327	0.272360796
10.2811806	0.333161157	0.333993343
12.3488749	0.342173449	0.289132754
8.889349653	0.325050273	0.290002873
9.206424887	0.207133059	0.459533608
7.459220239	0.304767906	0.358958202
12.47492052	0.297520661	0.358471074
14.00289896	0.337960209	0.286271399
14.50168648	0.273281512	0.318396586
7.807456926	0.250096117	0.273164168
12.09139742	0.25586773	0.355763672
10.11475238	0.315932478	0.371892704
6.165065574	0.346966092	0.361392029
16.68066933	0.177617729	0.399889197
12.42249318	0.22	0.18
10.53293283	0.227110583	0.346016647
7.725308063	0.37585034	0.277210884
12.34504446	0.210870859	0.343979369
19.28021096	0.269896194	0.334883218
5.805655688	0.417814509	0.359044995
15.35457779	0.223195266	0.363313609
12.03658145	0.239299052	0.426601551
8.835188589	0.279585799	0.37056213
15.45286332	0.286989796	0.343112245
9.616209277	0.212610534	0.304882737
14.29927851	0.252230174	0.338888344
9.27648538	0.331687243	0.320384088
32.00855795	0.197496815	0.462008677
6.325645435	0.257617729	0.289473684
4.942088633	0.25	0.25
16.54098789	0.248513674	0.455410226
15.28680275	0.291896914	0.30842584
12.95609557	0.519031142	0.346020761
21.37150388	0.263331025	0.318386427
8.231137638	0.32231405	0.376033058
9.92011249	0.28625	0.34234375
10.79023162	0.213333333	0.305
15.21769139	0.177229516	0.36713445
16.85741561	0.275143821	0.366395556
5.456965283	0.255	0.24625
17.1204316	0.383431417	0.466727026
12.19269957	0.2868	0.2512
18.72462282	0.216186523	0.394012451
15.10080197	0.320593714	0.452076197
46.42646646	0.305555556	0.302083333
5.207115075	0.34375	0.25
19.79581227	0.19975377	0.418898122
8.618218959	0.251028807	0.418381344
15.23491593	0.260649146	0.34421071
10.16432047	0.372400756	0.270321361
16.0766838	0.193663225	0.284442862
8.526045056	0.314263101	0.321951495
11.37303222	0.347943099	0.334871203
11.32094291	0.286694102	0.363287702
8.823774765	0.309859438	0.335840254
15.33276715	0.223312835	0.345177544
12.54021694	0.208595041	0.430743802
12.00599568	0.357142857	0.359056122
10.39075652	0.321540062	0.396462019
15.36781842	0.320814045	0.335313128
10.63970198	0.241015625	0.33796875
21.56955757	0.253308129	0.392249527
13.98218682	0.178969627	0.434832757
72.43532003	0.14700503	0.480185947
3.915148502	0.25	0.15625
9.024509009	0.347715319	0.344345825
20.89523411	0.32645	0.375
8.526688071	0.346105789	0.335276968
11.17477375	0.442602041	0.262117347
2.25777171	0.25	0.180555556
7.566332353	0.218934911	0.408284024
26.47049993	0.21126422	0.410110559
24.9863254	0.173553719	0.375114784
15.29629505	0.223543401	0.396551724
15.17039283	0.285082661	0.381007305
14.55267914	0.308066053	0.319095752
18.96539911	0.160714286	0.392857143
16.77493538	0.262777778	0.327222222
4.536783033	0.25	0.21875
7.921227336	0.224913495	0.266435986
13.74552652	0.210303729	0.284890427

log.sigma.0.5.mm.3D_glszm_SizeZoneNonUniformity	log.sigma.0.5.mm.3D_glszm_GrayLevelNonUniformity	log.sigma.0.5.mm.3D_glszm_LargeAreaEmphasis	log.sigma.0.5.mm.3D_glszm_ZoneVariance
15.73684211	7.263157895	3694.578947	3349.401662
26.20588235	15.91176471	23635.95588	22703.01276
15.74418605	11.60465116	1300.418605	1198.549486
65.6442953	26.5704698	12228.47651	11915.73271
4.764705882	5.352941176	3124.882353	2678.927336
13	14.72093023	3502.534884	3278.928069
4.2	4.2	3142.066667	2637.315556
10.52631579	7.947368421	7520.263158	6824.969529
33.96629213	24.5505618	15539.22472	14993.55612
51.31578947	39.76315789	22249.31579	21833.90703
15	18.0952381	6379.357143	5991.64229
29.64102564	13.69230769	8531	8170
28.05494505	36.2967033	18634.28571	18114.84652
11.47619048	17.47619048	12085.95238	11377.37868
18.44444444	16.59259259	5462.574074	5177.964678
36	41.42647059	156355.1544	153745.8979
7.75	14.375	5665.875	5190.089844
20.01408451	25.3943662	30643.52113	29564.72248
3.666666667	8.62962963	4139.333333	3674.691358
17.15873016	23.03174603	55434.90476	53458.18493
88.17424242	87.95454545	40282.68182	39891.12167
23.41975309	27.71604938	13277.87654	12826.96967
34.22033898	38.3559322	25393.63559	24857.98772
12.40740741	5.592592593	2694.037037	2376.66941
24.76811594	21.02898551	8476.173913	8171.696702
15.77272727	13.09090909	3562.090909	3313.311983
35.21138211	41.56910569	39312.03252	38517.98427
25.79012346	22.13580247	9526.234568	9213.250419
27.8627451	25.50980392	9022.794118	8723.368416
33.08602151	23.79569892	27278.66667	26531.55556
69.17204301	58.76344086	21376.19892	21084.08304
59.26829268	56.90243902	95628.89024	94321.45048
37.98947368	16.87368421	12496.95789	12038.99789
1.8	2.2	359	250.84
10.03448276	6.586206897	997.5862069	866.5231867
23.28571429	31.57142857	17562.16667	16983.87868
48.84507042	29.94366197	18268.21127	17856.86431
45.54411765	36.70588235	27353.61765	26745.41501
11.84848485	13.78787879	8613.545455	7973.302112
23.61538462	14.50769231	5118.015385	4857.565444
25.16949153	14.11864407	12148.64407	11524.49124
19.26923077	14.53846154	6015.634615	5679.758506
19.21428571	16.07142857	16023.42857	15163.6824
15.54901961	10.84313725	6471.588235	6067.657055
34.22772277	25.47524752	11328.0198	10982.28076
43.25185185	44.77777778	13814.42963	13542.05739
115.0401606	49.17670683	20619.23695	20325.71336
11	9.789473684	2930.921053	2695.540859
2	2	941	660.4375
13.20689655	7.206896552	2991.206897	2697.498216
44.1048951	41.74125874	29411.3007	28837.98288
11.76470588	17.64705882	6625.147059	6033.512976
48.39473684	40.02631579	32751.21711	32119.29012
8.272727273	7.090909091	6045.681818	5324.02686
54.775	45.8	104975.8688	103313.7778
18.3	12.8	3173.216667	2958.594167
34.5106383	16.65957447	13369.94681	12825.66784
26.01408451	19.53521127	7555.704225	7224.053958
9.85	10.2	5540.35	5126.2275
21.93617021	18.0212766	6809.680851	6458.317791
25.12	28.68	14540.31	14091.2939
100.8671875	55.34375	38285.72656	37827.83344
58.31782946	41.35658915	30696.35659	30122.58698
21.75	22	44230.51389	42791.78684
2	2.75	2071.875	1336.109375
23.87719298	11.38596491	13265.03509	12522.7116
11.2962963	6.777777778	1556.37037	1343.426612
45.09160305	34.14503817	26476.09924	25880.5157
6.217391304	8.565217391	2832.521739	2456.499055
30.1509434	20.52830189	11341.25472	10981.32939
42.81954887	41.79699248	24838.4812	24316.03222
17.07843137	17.74509804	9175.431373	8685.373318
68.66137566	54.18518519	56892.99471	56145.305
74.89237668	69.09865471	84235.13004	83328.12094
122.5380282	79.27605634	83670.51268	83001.81678
23.69090909	11.47272727	5834.945455	5423.224463
20.10714286	20	7605.267857	7207.407844
12.29032258	9.967741935	3748.258065	3361.057232
35.87850467	34.3271028	10009.15888	9737.371299
54.075	38.5625	16445.4375	16129.04234
18.04347826	11.65217391	1527.434783	1407.389414
44.35294118	18.25490196	3694.029412	3521.184256
77.79012346	23.81481481	12868.16049	12571.13016
1.25	2	1020.875	692.359375
53.71794872	54.24358974	27402.8141	26930.30913
75	65.29	63093.01	62343.3456
16.42857143	16.95918367	4405.469388	4153.372761
14.67857143	24.78571429	13630.46429	12862.38265
2.166666667	3	913.75	681.1875
5.307692308	2.846153846	302.0769231	236.8402367
64.79746835	33.37974684	34489.46203	33856.5256
24.75757576	11.45454545	1859.727273	1745.302112
23	12.96551724	5770.206897	5459.718193
77.7254902	58.15686275	29560.07843	29158.11524
80.7312253	77.94071146	52476.89328	51950.25428
11	4.5	472.4642857	390.8201531
19.63333333	15.76666667	8950.166667	8539.428889
1.75	2	599.375	362.984375
4.529411765	3.823529412	3531.941176	3034.913495
14.52941176	10.7254902	4045.745098	3795.361015

log.sigma.0.5.mm.3D_glszm_ZonePercentage	log.sigma.0.5.mm.3D_glszm_LargeAreaLowGrayLevelEmphasis	log.sigma.0.5.mm.3D_glszm_LargeAreaHighGrayLevelEmphasis	log.sigma.0.5.mm.3D_glszm_HighGrayLevelZoneEmphasis
0.053824363	357.6139142	42975.07895	18.28947368
0.032739528	2183.83308	278225.0294	17.58823529
0.099078341	290.5806137	6753.534884	13.58139535
0.05654649	431.1107667	364022.0336	39.69798658
0.04735376	512.1898693	22456.64706	10.64705882
0.066874028	300.5256202	44392.60465	15.41860465
0.044510386	244.8897963	43525.6	12.93333333
0.037924152	399.9360816	153519.6316	26.71052632
0.042809043	817.5384268	313621.3371	26.92134831
0.049063912	1226.814937	422501.4079	26.30263158
0.050785973	564.2201653	78407.90476	15.76190476
0.052631579	309.234141	244288.7949	33.48717949
0.043876567	1638.43232	230112.9121	16.35164835
0.037567084	2225.403935	77227.78571	7.833333333
0.059275521	469.2906996	69263.83333	15.14814815
0.019576796	14581.2	1809588.419	18.78676471
0.045845272	1022.107639	37187.59375	7.4375
0.030445969	2748.180974	370689.8451	15.66197183
0.046391753	342.7249486	54437.51852	14.62962963
0.022491967	5071.558964	655705.9683	13.52380952
0.050535988	3960.95444	436946.447	16.92424242
0.047093023	694.6311999	266923.679	24
0.043207616	2450.301718	282071.2373	15.89830508
0.056133056	218.7578498	36915.22222	13.48148148
0.05730897	1814.812572	45071.43478	10.7826087
0.063400576	330.3365846	41992.93182	18.18181818
0.035487594	2134.224773	758425.3171	24.53658537
0.056524773	546.9892001	172389.963	26.11111111
0.057790368	760.5597794	116766.7941	14.76470588
0.036585366	2629.947819	303833.0968	17.8172043
0.058508965	2136.777605	226971.9731	16.8655914
0.027655987	18546.59973	575584.4695	10.12195122
0.046728972	647.3662788	257496.9474	23.91578947
0.096153846	29.02981859	5102.8	21.5
0.087349398	86.19923372	13195.55172	16.37931034
0.041584158	1538.472285	218083.5119	14.88095238
0.049305556	1787.588257	200844.4507	19.30985915
0.040548599	971.4371179	795165.3897	33.36764706
0.039520958	1426.646675	60958.78788	9.696969697
0.061963775	269.0980584	102858.4462	25.13846154
0.040027137	1109.3417	145146.6102	15.76271186
0.054564533	524.5084081	75321.59615	14.28846154
0.03410475	815.1995031	334269.7679	24.80357143
0.049756098	568.1246594	82175.13725	17
0.053780618	571.965398	237381.4653	23.84158416
0.06059246	1297.984548	158552.2889	16.05185185
0.058368495	504.4315231	865915.4578	45.81927711
0.065180103	260.7616667	35934	13.47368421
0.059701493	71.17086806	13582.875	13.25
0.058350101	144.3953966	65944.41379	24.79310345
0.041764019	1552.143995	586111.4266	26.37062937
0.041112455	604.8299183	82682.32353	20.70588235
0.039780162	1140.055121	973568.3618	36.17105263
0.037225042	1065.10899	40766	10.90909091
0.024528591	9597.433067	1244824.138	16.91875
0.068259386	305.4302151	39253.26667	18.68333333
0.042863657	719.0525096	271111.1702	24.91489362
0.05491106	362.1901182	168124.5211	27.05633803
0.049140049	490.9872144	68316.225	13.95
0.053348468	366.927234	132526.6596	23.57446809
0.047192072	778.6257282	285607.78	24.89
0.046732384	2052.946295	750168.5781	27.65625
0.041747573	1646.83354	599946.3333	22.63565891
0.026363969	498.433814	3968711.278	89.09722222
0.036866359	410.0069444	13562.75	10.875
0.036703155	685.5044994	273868.9474	29.31578947
0.068527919	139.8792181	20613.40741	13.37037037
0.040975915	1389.751543	531010.5496	24.38931298
0.051569507	249.5340097	35696.78261	17.60869565
0.052710094	608.0747287	222492.5849	26.0754717
0.04375	2348.322765	283433.1504	14.79699248
0.045172719	808.2993519	113597.8824	17.70588235
0.036571207	5380.252773	648050.3704	17.43386243
0.033204288	7985.065516	956353.6906	15.04035874
0.038671024	4337.742934	1696990.642	24.25352113
0.049283154	293.44329	124527.5273	20.09090909
0.050134288	674.1928323	94160.17857	19.17857143
0.050819672	348.7462455	44695.90323	16.06451613
0.060657596	535.4006511	196476.215	24.42990654
0.056219255	1554.9835	187726.225	16.40625
0.091269841	81.66911047	30420.95652	29.2826087
0.07606264	189.8551798	78472.70588	21.85294118
0.058022923	143.1995279	1176734.136	100.1975309
0.055172414	77.77045139	14510.625	13.625
0.046004129	2539.371577	319867.4744	15.80769231
0.036523009	2206.869708	1862624.745	34.635
0.062982005	401.6587925	52455.36735	13.67346939
0.036082474	705.5763509	284966.3036	26.625
0.06557377	74.93962963	12284.83333	13
0.123809524	13.90985043	7045	16.07692308
0.039748428	832.8168376	1465472.652	42.19620253
0.093484419	66.94642723	53721.48485	36.8030303
0.056751468	306.5097386	114807.2931	23.17241379
0.049877751	1570.324097	584582.7647	24.26960784
0.043575611	2868.929259	1004202.32	24.96837945
0.110671937	27.64288032	9282.75	27.75
0.049342105	286.6783187	288382.25	30.51666667
0.06504065	49.30795139	8216.625	14.25
0.044854881	276.0981699	48672.82353	14.41176471
0.063197026	386.0097456	46099.62745	21.82352941

log.sigma.0.5.mm.3D_glszm_SmallAreaEmphasis	log.sigma.0.5.mm.3D_glszm_LowGrayLevelZoneEmphasis	log.sigma.0.5.mm.3D_glszm_ZoneEntropy	log.sigma.0.5.mm.3D_glszm_SmallAreaLowGrayLevelEmphasis
0.660739514	0.239177408	3.83935416	0.206479891
0.64146303	0.21877451	3.973766235	0.166692848
0.624921777	0.367745478	3.561594767	0.239073121
0.689181372	0.057287645	4.2464279	0.044254166
0.531807143	0.404624183	3.219528282	0.258626199
0.564787047	0.166576227	3.660496132	0.099226506
0.509986167	0.294240741	2.866247897	0.169120506
0.534439387	0.10354741	4.198603751	0.072863193
0.640409771	0.086520102	3.938705796	0.065170411
0.600710945	0.085982702	4.207456917	0.053490281
0.616025498	0.162943122	3.148154485	0.108452546
0.636668046	0.07782688	4.281062073	0.058418229
0.566275795	0.161317062	3.653004201	0.102065652
0.493700689	0.523478836	3.095996661	0.247002148
0.600348208	0.206239712	3.686750452	0.145804383
0.524949987	0.134917926	4.468746983	0.075334232
0.496190112	0.567925347	3.405639062	0.295375572
0.53402113	0.159847418	3.920480486	0.092213025
0.323030986	0.161615226	4.236368984	0.064252097
0.52078101	0.170992063	3.752343569	0.111465759
0.596781017	0.15273408	4.049545124	0.100786296
0.543236594	0.082933936	3.912650079	0.054351972
0.545882498	0.123206215	4.019763974	0.068253384
0.691867079	0.318878601	3.457867131	0.275346561
0.619671447	0.400193237	3.789086997	0.217503537
0.617061565	0.210852273	3.684301702	0.131629859
0.545662641	0.084941513	4.105431097	0.047146153
0.580484088	0.07589144	4.054082513	0.05160756
0.536968267	0.189362745	4.517014354	0.114280908
0.618506513	0.178984285	4.19295448	0.127620144
0.632828441	0.134309152	3.893375474	0.097042435
0.622368753	0.377511856	3.774584893	0.238039756
0.653845828	0.120830098	4.298480547	0.087006617
0.368361714	0.163179705	3.121928095	0.02240387
0.601759167	0.233917625	3.620936065	0.158449369
0.533919814	0.181848545	3.919736735	0.120103504
0.607915452	0.193646203	4.408981903	0.135364707
0.599563676	0.055138951	4.353323603	0.038694604
0.615260305	0.393518519	3.116344031	0.256769461
0.62295149	0.095838697	4.20549156	0.07286606
0.669483358	0.273964218	3.718880186	0.208036941
0.629669455	0.202879274	3.865325834	0.152895399
0.606168236	0.089362144	3.936956272	0.059339666
0.567706626	0.24664199	4.078755982	0.183573405
0.602900806	0.105201135	4.169253232	0.076618918
0.582865792	0.140007139	3.95447848	0.089504221
0.706006367	0.041502919	4.282234681	0.032246696
0.548265638	0.275921053	3.83977554	0.219916311
0.438475703	0.317951389	2.5	0.25772795
0.69583464	0.104783603	3.486448667	0.089688359
0.574513747	0.07234675	4.308278877	0.046431208
0.605991884	0.140776144	3.085761182	0.110100258
0.584586544	0.049766391	4.484918269	0.033725688
0.62057229	0.411073232	3.083765141	0.218547651
0.605441785	0.142950468	4.258954149	0.097306263
0.567709914	0.275997543	4.215224664	0.167402491
0.628052971	0.104168823	4.382489397	0.073255391
0.626525577	0.100360576	3.86811287	0.074037955
0.504637653	0.316767149	4.190399972	0.226486115
0.705768603	0.087807329	2.996803229	0.054108235
0.505559775	0.086483787	4.429737898	0.053845156
0.651857033	0.086908122	4.526764908	0.06358084
0.697763452	0.085927684	3.597218904	0.066401016
0.563374736	0.027718377	4.181045831	0.022806941
0.380416771	0.215277778	2.5	0.030121983
0.66698195	0.091722908	4.020348387	0.070825674
0.660667436	0.330915638	3.151073839	0.293847213
0.608043624	0.083379734	4.296553801	0.052533954
0.523292647	0.205277778	3.288502306	0.148275698
0.551551971	0.094251475	4.785787803	0.060452969
0.584585419	0.173310359	4.093277762	0.109914684
0.598324355	0.156830065	3.620872587	0.106211348
0.626062852	0.163523618	4.212526182	0.102936868
0.600195971	0.170519667	4.249825831	0.116288779
0.608628547	0.105291431	4.555211868	0.068546125
0.677839353	0.187775307	3.971039142	0.155922961
0.620870944	0.14999504	3.687522373	0.111585417
0.639715287	0.174202509	3.486139052	0.131502252
0.601050222	0.088732649	4.128548089	0.06118708
0.601865156	0.194142149	4.512611124	0.133841743
0.646913224	0.085131002	3.767414048	0.056867634
0.684606287	0.172434849	4.174801616	0.140450696
0.719233716	0.018814311	4.393095116	0.015315037
0.294568004	0.224201389	3	0.068766562
0.608355977	0.15242783	3.938112653	0.108724438
0.635861887	0.047504114	3.972147385	0.033898893
0.595446961	0.195391156	3.63117006	0.132730168
0.523084404	0.098417355	3.76388549	0.076989673
0.377573947	0.192152778	3.251629167	0.137656765
0.640227893	0.313440171	2.81507241	0.29348345
0.66438138	0.052102853	4.335232762	0.042642602
0.633524333	0.066623453	4.134330259	0.050498727
0.653119478	0.107672438	3.923553031	0.082623877
0.640237705	0.086651279	4.056796681	0.059582929
0.584197208	0.082346586	4.233954996	0.056325212
0.639419129	0.114993218	4.039148672	0.080090081
0.589814622	0.071724634	3.985774332	0.048536128
0.382406563	0.206840278	3	0.144408867
0.517651589	0.275245098	3.410417253	0.205154682
0.548207547	0.159574496	4.221130981	0.10494672

log.sigma.0.5.mm.3D_ngtdm_Coarseness	log.sigma.0.5.mm.3D_ngtdm_Complexity	log.sigma.0.5.mm.3D_ngtdm_Strength	log.sigma.0.5.mm.3D_ngtdm_Busyness	log.sigma.0.5.mm.3D_ngtdm_Contrast
0.008717686	0.02143627	0.137053861	4.193496107	4.48E-05
0.002769024	0.00410371	0.034515106	14.9228836	1.08E-05
0.013695528	0.025564732	0.178764832	5.306291941	0.000109968
0.002413713	0.013419235	0.087512088	5.651390087	7.76E-06
0.014583393	0.015793019	0.13611775	4.627263457	7.42E-05
0.008741509	0.014062667	0.107036673	4.543927315	3.55E-05
0.013885574	0.018134369	0.136572824	3.463155239	9.21E-05
0.006481314	0.01466136	0.100708955	4.219845855	2.98E-05
0.003093975	0.006377134	0.052418062	8.536832652	1.09E-05
0.001894273	0.005749568	0.047647252	11.18949245	5.19E-06
0.006502038	0.007223249	0.05637437	8.246297247	3.43E-05
0.004081466	0.017906667	0.125627442	3.747057068	1.39E-05
0.002587126	0.006031589	0.056454058	11.77390749	6.49E-06
0.005139893	0.002560317	0.024026417	21.47228669	3.33E-05
0.005865661	0.01038276	0.071587821	6.84731117	2.90E-05
0.000697204	0.001741458	0.017584756	43.94422754	1.52E-06
0.008440254	0.004299779	0.036267183	13.80616652	7.08E-05
0.002511481	0.003408674	0.033703477	15.55179798	7.14E-06
0.011165283	0.01478171	0.126431911	3.614329683	4.29E-05
0.001887857	0.002759314	0.029142846	19.9560583	4.30E-06
0.001036648	0.00251143	0.021603794	31.05782447	3.05E-06
0.003230635	0.010148444	0.089213432	6.285749749	7.65E-06
0.002054345	0.002878754	0.029344176	18.96834104	5.13E-06
0.01267227	0.020125144	0.131742279	3.304622382	7.75E-05
0.004530385	0.008072305	0.079124623	13.04925196	1.71E-05
0.008995573	0.013186732	0.095147645	4.96902358	4.70E-05
0.001462233	0.003806609	0.032006056	16.75678442	4.19E-06
0.004150743	0.012074952	0.107314601	5.036314466	9.99E-06
0.003930273	0.004918406	0.040578713	10.73066328	1.66E-05
0.002239028	0.004931535	0.04626386	14.45155403	5.93E-06
0.001800663	0.003968352	0.036841881	17.85891791	4.85E-06
0.000882869	0.00087483	0.009400073	79.37532662	2.89E-06
0.003203137	0.012570697	0.096579458	5.730734798	8.82E-06
0.051343853	0.146535124	0.890999796	0.630622328	0.000347564
0.018278322	0.031730037	0.176550376	2.557532081	0.000160158
0.00302585	0.004227223	0.036364574	13.27885191	1.06E-05
0.002208758	0.004795747	0.036444043	16.1054849	9.18E-06
0.001701171	0.006090904	0.049284037	10.18432531	5.03E-06
0.006047702	0.003863527	0.028737451	16.72347113	5.11E-05
0.006081097	0.017777722	0.135739549	3.571317285	1.84E-05
0.00394187	0.006425934	0.0438186	11.16488152	2.17E-05
0.006583736	0.008957625	0.075972159	6.271148159	2.58E-05
0.003745104	0.008118146	0.067045516	6.719990799	1.20E-05
0.006105683	0.013410792	0.102733261	5.456201104	2.41E-05
0.003114743	0.009996271	0.07530674	6.731935559	9.57E-06
0.002442923	0.005774153	0.051279536	13.18561005	6.88E-06
0.001409193	0.00841945	0.061972932	7.903549131	3.96E-06
0.010367019	0.015719589	0.113438293	4.153325898	5.37E-05
0.035103673	0.045960915	0.346229815	1.359424645	0.000273774
0.011042906	0.028293646	0.208858945	2.208788213	4.47E-05
0.00163025	0.005216097	0.043816737	12.61496636	3.92E-06
0.007469842	0.01132195	0.082331137	5.630752171	3.44E-05
0.001560931	0.006125555	0.05685901	9.256352482	2.99E-06
0.00901295	0.010243412	0.076816384	8.08735977	5.41E-05
0.000821196	0.001921815	0.018018612	37.99089751	2.05E-06
0.007326698	0.018383551	0.103643508	5.212556188	5.11E-05
0.002956232	0.009253063	0.060772368	7.705807934	1.19E-05
0.004482035	0.011318752	0.077208514	5.651693756	2.24E-05
0.007057959	0.01732504	0.127321374	4.665485273	2.83E-05
0.006061096	0.010900189	0.107382291	5.066338508	1.97E-05
0.003008995	0.006356698	0.051128471	8.803653372	1.01E-05
0.001115112	0.003249028	0.027148787	19.22494629	3.06E-06
0.001657716	0.004039853	0.038514185	14.44566028	3.94E-06
0.001887629	0.011829668	0.23285574	3.619286873	1.99E-06
0.025978734	0.014790195	0.110901267	4.724539556	0.000277601
0.003873299	0.012298635	0.089764097	5.482254689	1.28E-05
0.013851276	0.028851892	0.145315915	3.25607069	0.000123371
0.001965405	0.005325629	0.049060982	10.5779599	4.43E-06
0.013159668	0.021618402	0.146548122	3.20577293	6.75E-05
0.003133548	0.012382845	0.09986919	5.901826267	8.12E-06
0.001924014	0.002914899	0.023256107	21.97232626	7.73E-06
0.005097131	0.007680607	0.063485682	7.907223088	1.94E-05
0.00108338	0.002327375	0.023458169	29.29771669	2.62E-06
0.000786725	0.002525984	0.02975753	32.65720118	1.32E-06
0.000591565	0.001995646	0.015662459	34.76229689	1.72E-06
0.005506045	0.025550378	0.161677483	3.430477032	2.10E-05
0.005203763	0.008258057	0.060158895	8.047853611	2.44E-05
0.010136146	0.01591801	0.107338422	4.424398018	5.17E-05
0.003537373	0.00734171	0.063153446	7.309379918	1.03E-05
0.002232722	0.004668651	0.038735682	15.47314126	7.62E-06
0.01305266	0.02858538	0.195381314	2.200411436	6.35E-05
0.00465314	0.017065587	0.087565301	5.27014225	3.10E-05
0.002287011	0.017475159	0.14825839	2.975227886	6.09E-06
0.039381864	0.039748596	0.35691598	1.250644642	0.00023554
0.001697387	0.003724423	0.034515921	18.82279904	4.39E-06
0.000915028	0.004083844	0.048557921	15.04054824	1.34E-06
0.007638572	0.011310991	0.09124846	5.449518351	3.01E-05
0.00396024	0.009055149	0.067767869	6.523761466	1.54E-05
0.029909375	0.037155924	0.243625696	1.85184109	0.000237251
0.041101927	0.117290502	0.65465808	0.758949722	0.000614906
0.001406114	0.007917668	0.075609032	7.425754499	2.62E-06
0.009570207	0.039300136	0.253444203	1.715435999	3.70E-05
0.005549185	0.019433341	0.130869577	3.902097136	1.91E-05
0.001395148	0.004284258	0.0368384	14.86606709	3.42E-06
0.000941978	0.003737717	0.040132597	18.12490126	1.47E-06
0.025807673	0.094144341	0.444827448	1.077047471	0.000215733
0.004861089	0.018457719	0.182605065	2.831926047	1.01E-05
0.046759359	0.055282413	0.368397088	1.226907392	0.000403247
0.013861076	0.02161698	0.197159744	2.603080203	4.99E-05
0.007528124	0.017302986	0.127073929	4.797620314	3.49E-05

original_gldm_GrayLevelVariance	original_gldm_HighGrayLevelEmphasis	original_gldm_GrayLevelNonUniformityNormalized	original_gldm_DependenceEntropy	original_gldm_DependenceNonUniformity
161.0092449	994.631728	0.059076792	6.580311688	171.8016997
146.8414747	991.0279249	0.074975504	7.042731135	284.4405392
125.1872465	982.4354839	0.040667672	6.51946347	121.0967742
206.3707326	955.0732448	0.039984733	7.139046071	644.7237192
87.5070181	304.0640669	0.037111754	6.30629952	119.6740947
105.486713	510.9797823	0.029732809	6.620767757	214.2348367
33.48410218	132.4777448	0.070521005	5.931115865	81.07121662
172.2371265	573.0359281	0.035661213	6.671183947	277.9081836
143.5596951	929.8239538	0.033154329	7.134911234	525.9158249
113.2046974	1143.805358	0.060051238	6.981925802	599.6791478
116.5039529	488.662636	0.03474191	6.701488054	225.1112455
150.23557	1221.582996	0.051505151	7.002192017	303.7894737
97.9709774	502.3027965	0.03021056	6.977767986	569.8379942
102.4555069	532.5912343	0.02993622	6.750476784	352.4740608
106.5674805	594.4390779	0.029028064	6.842885431	294.3874863
101.9638134	1358.0321	0.129665439	6.923150991	676.4688355
121.4255876	504.4183381	0.029884812	6.623869318	227.025788
156.2398333	837.266295	0.043294281	7.050188504	494.8181818
117.5190421	411.8006873	0.031441528	6.692233037	178.4295533
114.3511463	951.6486969	0.094954995	6.91059364	409.5598001
87.61422744	996.3527948	0.100326066	6.728146543	833.3273354
102.3916817	1019.671512	0.0305841	6.863925418	534.3348837
101.7803212	803.1863786	0.055573202	6.886513977	560.6968144
159.7196589	505.7879418	0.032939865	6.642435726	137.3659044
86.94636028	1160.597176	0.054818655	6.701334553	272.2358804
153.9146575	794.573487	0.049514571	6.774745844	153.9423631
87.33293233	871.559146	0.080510234	7.034924187	508.1061743
111.5425649	931.1479414	0.063560603	6.692067278	292.3482205
197.2095884	663.0600567	0.034688024	6.952600208	481.8226629
138.4853672	1190.724626	0.089688512	6.892354605	365.4421715
102.0910323	966.6804026	0.068534751	6.710687736	619.3195974
116.3797726	1017.138786	0.04667899	7.132020094	1256.283642
202.4037271	867.3738318	0.050736628	7.043875515	423.4849975
68.18112056	160.4519231	0.070821006	5.406425032	32.75
150.3271883	650.3614458	0.031481347	6.447499102	111.1566265
152.8039447	636.629703	0.033889325	6.971446883	513.2990099
191.5870944	1232.142361	0.089819155	6.827841215	501.4902778
125.0566872	869.1067382	0.046506116	7.042018961	734.6577221
109.1121948	489.7125749	0.031345692	6.882864383	242.6550898
178.0456397	691.9818875	0.059528299	6.6792963	230.5843661
154.2939495	1224.879919	0.075677183	7.060005691	238.5970149
134.7049677	524.1563484	0.035116366	6.659280107	275.7345226
147.8156759	650.045067	0.033230174	6.822374249	466.043849
149.0209123	466.8341463	0.03505818	6.821830578	279.4917073
118.1547451	606.1458999	0.028987061	6.861111137	627.6964856
89.42001485	732.752693	0.062404714	6.694029377	432.7405745
146.1961642	1080.798172	0.032419688	7.243312827	1100.033755
144.6592741	644.6380789	0.034055236	6.62214666	175.9262436
76.63293607	177.380597	0.057473825	5.653713213	42.29850746
128.1850378	481.0503018	0.040536984	6.53484649	143.4828974
129.5217267	761.3011098	0.047609405	6.956886254	740.8294393
176.1618735	580.1378476	0.045576368	6.560039451	201.1185006
165.0506535	837.8563203	0.066723854	7.035383898	655.3643025
97.65639127	654.9678511	0.036941603	6.604297647	151.7715736
130.0173583	1167.690786	0.122012091	6.685741053	789.2618427
223.1482953	781.4300341	0.030087712	6.917089226	259.2684869
177.8299468	801.3488372	0.028632121	7.124611962	625.2352941
123.4110832	344.1392111	0.0388821	6.765059418	373.7115236
168.7170161	965.8427518	0.03859969	6.887626575	189.5061425
91.62999172	804.0533485	0.03663544	6.690503884	242.723042
148.9988671	819.3176026	0.039186791	6.919091732	529.4945729
185.4266509	1017.556407	0.069484416	7.013597522	1007.572107
95.89383689	907.3877023	0.06523811	6.894252846	543.5145631
112.2250218	936.1735628	0.108256895	7.019621515	290.1622116
133.1894498	596.5299539	0.039011234	6.253087726	58.91705069
194.7030723	990.9021249	0.043016259	7.049197265	339.9066323
118.2825891	551.2182741	0.033986962	6.527777083	125.3401015
179.517131	910.5961839	0.054268796	6.889820677	690.2668126
134.9668855	462.2847534	0.035019807	6.434086854	140.9686099
166.9839013	946.4236698	0.052812218	6.856955808	438.1009448
151.2970291	962.4973684	0.039821243	7.015186857	730.8592105
146.508289	667.3100089	0.035069482	6.852240174	283.8255093
127.3747088	925.3825464	0.085564506	6.932869607	804.5952012
115.1647119	1013.941185	0.082594311	6.857726254	1051.504765
149.78564	1344.75305	0.05234098	7.311477353	1732.430937
174.2852931	697.8584229	0.027390771	6.899852623	358.9390681
136.0678727	585.8343778	0.030141325	6.792958444	349.1378693
126.745899	655.8213115	0.037678044	6.520844914	173.052459
164.7241385	836.1626984	0.034296409	6.972169238	468.0725624
142.8899882	886.4581869	0.061028066	6.885956679	536.4722417
227.5658856	1009.345238	0.023833144	6.743567877	196.1269841
180.2592454	788.5197614	0.025250225	6.920941287	492.9970172
246.4778079	1114.00394	0.045389354	6.954546454	694.0451289
72.76309156	219.0896552	0.056551724	5.697772087	43.33103448
107.4965644	724.2813329	0.047652724	6.956390724	741.9997051
111.5174869	1089.541454	0.058083657	7.220061139	919.4134405
128.519044	715.6156812	0.040232354	6.721856031	205.9922879
168.4864558	618.5167526	0.031150361	6.888253165	464.1301546
156.368957	649.3060109	0.076592314	5.989625251	29.99453552
84.79854875	231.3238095	0.069569161	5.230526947	37.3047619
172.2571851	949.4674214	0.056262268	7.215346887	724.2558491
195.0135383	1289.254958	0.038291777	6.812097392	207.4957507
152.3420225	858.6428571	0.049124735	7.025761932	202.0176125
136.7422594	1101.520538	0.059404116	7.160318942	750.7090465
107.2877246	1031.831037	0.063793925	6.867420026	1072.432656
239.9779406	999.3833992	0.029011545	6.575721617	78.00395257
186.8263876	645.7935855	0.033932165	7.062237158	305.6776316
114.8888889	320.3333333	0.046995836	5.654680131	43.01626016
56.43504292	207.939314	0.045300437	6.335634831	99.9762533
135.3851599	1411.477076	0.049945566	6.923728619	160.2391574

original_gldm_GrayLevelNonUniformity	original_gldm_SmallDependenceEmphasis	original_gldm_DependenceNonUniformityNormalized	original_gldm_DependenceVariance	original_gldm_LargeDependenceEmphasis
41.7082153	0.498778127	0.243345184	11.37197955	22.66005666
155.7241213	0.331316237	0.13694778	11.74790128	29.98988926
17.64976959	0.528576106	0.279024825	3.437851728	9.092165899
105.3597723	0.478683785	0.244676933	5.653804392	12.89981025
13.32311978	0.5392394	0.333354024	0.858807737	4.331476323
19.11819596	0.561839671	0.33318015	1.242944112	4.872472784
23.76557864	0.419466187	0.240567408	2.038214654	7.973293769
35.73253493	0.503163977	0.277353477	3.13281222	8.502994012
68.92784993	0.465088838	0.252965765	2.875025247	8.661375661
186.0387347	0.407555919	0.19356977	8.568974507	19.5029051
28.73155985	0.476114906	0.272202232	2.523206356	7.696493349
76.33063428	0.433659236	0.20498615	5.981317875	15.18488529
62.65670203	0.464358083	0.274753131	1.503139852	6.227579556
33.4686941	0.513861257	0.315271969	1.244693277	5.166368515
26.44456641	0.529833211	0.323147625	1.05573422	4.758507135
900.7858068	0.228030554	0.097375678	14.18216193	41.3339571
20.85959885	0.518117824	0.325251845	1.029252633	4.747851003
100.9622642	0.425457311	0.212186184	5.484262509	13.87735849
18.29896907	0.526422045	0.306579988	1.329849671	5.357388316
265.9689397	0.345539004	0.146219136	20.01494137	41.75830061
524.1033691	0.371940969	0.159519015	17.99816549	37.19410413
52.60465116	0.53794142	0.310659816	1.575417793	5.626744186
151.7704138	0.427330805	0.205308244	8.227759691	18.38777005
15.84407484	0.506932732	0.285584001	1.637760902	6.143451143
66.00166113	0.452951145	0.226109535	4.991901304	12.54983389
34.36311239	0.447822101	0.221818967	6.61906502	15.48126801
279.0484709	0.330714727	0.146597281	9.365285192	24.28101558
91.08234473	0.419396526	0.204011319	6.696330002	16.25750174
61.22436261	0.515170758	0.272987344	2.892338756	8.29631728
227.9881983	0.347417987	0.143761673	13.12952872	30.92053501
217.8719723	0.410774956	0.194815853	6.408692979	16.36520919
276.8064081	0.410344428	0.211852216	5.674341858	14.18145025
103.1475652	0.429739941	0.208305459	7.496051984	17.13871126
7.365384615	0.533103632	0.314903846	1.054363905	4.826923077
10.45180723	0.579215885	0.334809116	2.618703731	6.789156627
68.45643564	0.472373456	0.254108421	2.778380551	8.50990099
258.6791667	0.404158011	0.174128569	13.60193673	29.22638889
155.9815146	0.433269614	0.219039273	5.374479302	13.57722123
26.17365269	0.471583792	0.290604898	1.287595826	5.68502994
62.44518589	0.461518164	0.219813504	7.942608195	17.98951382
111.5481682	0.369268814	0.161870431	14.12663646	30.69606513
33.46589717	0.518312002	0.289333182	2.808681702	7.78069255
54.56394641	0.492677834	0.283826948	1.970936486	6.666260658
35.93463415	0.497070283	0.272674836	2.720095181	7.954146341
54.43769968	0.543364446	0.33423668	0.972224774	4.481363152
139.037702	0.403967318	0.194228265	5.424551731	14.83303411
138.302391	0.473474302	0.257860702	3.109613646	8.941397093
19.8542024	0.539240237	0.301760281	3.241740686	8.313893654
7.701492537	0.519939884	0.315660503	0.921808866	4.686567164
20.14688129	0.501652765	0.288697983	1.69509613	6.167002012
163.0146028	0.4231614	0.216363738	4.958456729	13.00058411
37.69165659	0.470332241	0.243190448	3.864041443	10.51632406
254.9518451	0.378783436	0.171516436	8.585490822	20.80162261
21.83248731	0.477869618	0.256804693	2.810247337	8.461928934
795.8848689	0.284080956	0.120996757	9.520400379	28.34692626
26.44709898	0.534628743	0.294958461	4.517337289	10.32878271
62.79024168	0.507336792	0.285105013	1.825958273	6.417236662
50.2745553	0.484970213	0.2890267	1.426840582	5.855375097
31.42014742	0.456684796	0.232808529	4.663457069	11.96805897
32.27582293	0.480539604	0.275508561	1.600227272	6.305334847
83.03680982	0.473128183	0.249879459	3.421213627	9.622935347
380.6356334	0.397316971	0.183930651	10.88485963	23.66046002
201.5857605	0.363738756	0.175894681	5.336288895	15.49773463
295.6495789	0.275864087	0.106247606	16.44287237	42.79348224
8.465437788	0.504067379	0.271507146	5.557221432	12.33640553
66.80424984	0.427241797	0.21887098	3.412649177	10.49517064
13.39086294	0.535763536	0.318122085	1.123193074	4.883248731
173.4973413	0.445225634	0.215910795	5.790574699	14.40444166
15.61883408	0.541360869	0.316073116	2.157875686	6.524663677
106.2053705	0.439672338	0.217852285	4.536977504	12.21133764
121.0565789	0.451169949	0.240414214	2.700099983	8.776973684
39.59344553	0.470876097	0.251395491	2.991782	8.929140833
442.1973684	0.365027123	0.155687926	12.28666346	28.84249226
554.7033949	0.351126611	0.156567118	7.868187887	20.97051817
480.4901961	0.390532167	0.188717967	4.609956807	13.6583878
30.56810036	0.540327178	0.321629989	1.205964723	4.965949821
33.66786034	0.53924982	0.312567475	1.449398047	5.431512981
22.98360656	0.513053136	0.283692556	3.104208546	8.462295082
60.49886621	0.495463137	0.265347258	3.21961914	8.904761905
173.6858749	0.413348487	0.188500436	9.108218115	20.9170766
12.01190476	0.636270792	0.389140842	1.996582892	5.46031746
33.86055183	0.598299155	0.367633868	0.931399487	4.046979866
126.7270774	0.474664514	0.248583499	3.069596617	9.095988539
8.2	0.484243295	0.298834721	1.095362663	5.234482759
161.5903863	0.437601812	0.218814422	4.501735604	12.24329106
318.0661066	0.354263509	0.167898729	7.362057742	19.18882396
31.30077121	0.512479674	0.264771578	4.478016931	11.02056555
48.34536082	0.51205095	0.299052935	1.485472686	5.704896907
14.01639344	0.35580474	0.163904566	5.630804145	17.29508197
7.304761905	0.599555556	0.355283447	1.164263039	4.542857143
223.6425157	0.386844196	0.182202729	6.824708137	17.33861635
27.03399433	0.549930787	0.29390333	3.904059899	9.566572238
50.20547945	0.405150512	0.197668897	8.108463126	18.38356164
242.9628362	0.398838501	0.183547444	8.245750803	19.83569682
370.3875301	0.388851589	0.184711102	4.997957498	14.53909749
7.339920949	0.557908639	0.308316018	1.986283179	6.391304348
41.26151316	0.460267971	0.251379631	3.396335591	9.442434211
5.780487805	0.576899277	0.349725692	1.20853989	4.674796748
17.16886544	0.460899984	0.263789587	1.789140983	6.926121372
40.30607187	0.416465654	0.198561533	5.437397984	14.46716233

original_gldm_LargeDependenceLowGrayLevelEmphasis	original_gldm_SmallDependenceHighGrayLevelEmphasis	original_gldm_LargeDependenceHighGrayLevelEmphasis	original_gldm_SmallDependenceLowGrayLevelEmphasis
0.021391799	349.6803913	38873.13031	0.00341825
0.090917239	220.8620839	41043.03178	0.003471677
0.014754118	395.6255644	13425.34793	0.004754211
0.034831993	375.1848662	19818.83264	0.004189515
0.110507418	167.2543911	1306.793872	0.012917212
0.031828306	243.1635133	3504.618974	0.007385324
0.215219091	82.39028066	590.7210682	0.01137987
0.157983855	237.0891444	8795.896208	0.009799633
0.020191658	374.9759655	10941.04954	0.003184347
0.018343745	362.6712904	30547.86152	0.001914806
0.051949317	183.7735825	6620.469166	0.006292436
0.015829432	428.4972473	24806.78138	0.002479948
0.055293427	211.7187994	3870.452266	0.005790113
0.044515066	236.3183971	3397.655635	0.005244404
0.027583907	324.279571	2780.164654	0.003964115
0.043045023	217.0687869	68075.75083	0.001083824
0.045361683	239.4825247	3090.091691	0.006256666
0.040939101	264.7264373	18197.31261	0.003579423
0.184530551	191.9269119	2739.628866	0.011324689
0.045535293	217.0599401	58150.27633	0.002101416
0.030862245	259.4961447	52060.55647	0.001525472
0.008757124	495.5506219	7493.955233	0.001817523
0.035258774	247.9057577	22724.31344	0.002722567
0.147317761	257.9331757	3672.577963	0.007409296
0.010821486	411.5271665	19476.25664	0.001942462
0.098346448	263.6045012	19247.27089	0.010039962
0.03299171	232.1011736	26478.99885	0.00231622
0.034312761	278.9596051	21547.61549	0.003688374
0.10807886	308.3014064	8208.840227	0.007138332
0.034878311	273.5398339	49936.18725	0.0022507
0.021428634	285.470326	22173.97137	0.002431202
0.018138705	326.8358803	20840.93609	0.001457215
0.068548136	296.8006628	23604.71471	0.004900476
0.449853026	118.4918643	358.7788462	0.025155805
0.036302501	333.9771714	7775.186747	0.006865568
0.063084237	255.7611917	8380.543564	0.004088858
0.030847093	329.5305332	54022.58681	0.002755506
0.026440589	300.2353116	17366.26118	0.002639534
0.049746624	227.5293143	2786.632335	0.007057007
0.212385949	235.6474086	20838.17064	0.016801368
0.031972263	353.4023485	51280.32361	0.002410872
0.057181981	231.5602663	7416.466946	0.005967204
0.035246575	293.1595822	6150.054811	0.00323678
0.26138598	210.0391733	5716.949268	0.010737439
0.025254388	331.9781907	2667.27689	0.003478526
0.025624542	213.2874378	15312.51391	0.003504479
0.01436112	453.4491852	13165.83943	0.001562911
0.05218441	273.0139808	9428.662093	0.006762129
0.515902981	102.5913661	792.7686567	0.043825503
0.061377625	237.4987438	4038.060362	0.008468184
0.036283219	241.1197706	15135.37646	0.003432058
0.105071116	209.9272818	10947.23579	0.009271936
0.078865945	236.0697127	25468.10285	0.005197206
0.023565152	251.6724568	8221.522843	0.004538772
0.048833783	233.3602723	41448.83719	0.002076779
0.801032922	347.3413124	11389.35722	0.015781768
0.039832548	361.3418505	6267.589603	0.003470899
0.200156445	215.2038206	1247.834493	0.011444152
0.030616002	340.7936561	17743.67813	0.003444437
0.043208148	351.9969282	6180.141884	0.002023868
0.026249234	296.6065239	11993.44785	0.003815987
0.043782613	301.0250795	39270.13545	0.001992783
0.021318039	256.3113936	18547.09126	0.002240544
0.085851609	222.3168694	47250.57488	0.002632356
0.079677003	245.3344021	12140.6682	0.014304711
0.044485162	314.2877674	15283.5499	0.005013588
0.050881873	272.5467752	2760.426396	0.004871532
0.03486553	295.7083738	20967.39756	0.003045675
0.086025553	193.432683	5563.7287	0.019415947
0.029492331	312.406536	17279.29388	0.002995828
0.022655049	341.606177	11580.41053	0.003292283
0.048277654	243.6689936	9268.914969	0.00534159
0.049675974	224.7746144	38917.98394	0.002601427
0.032731713	247.2041745	28536.26608	0.00259072
0.017041514	457.9230211	22138.41362	0.001705944
0.038540442	365.3559764	4179.521505	0.004838657
0.046224085	272.0603658	4487.214861	0.006951876
0.031975733	259.9519541	9087.827869	0.00559389
0.042007473	330.1643694	11546.30215	0.004521204
0.034011361	267.208317	29419.3577	0.002290044
0.021043355	563.4221759	8231.753968	0.005841533
0.022096267	451.417861	3690.922446	0.004199505
0.052592996	420.6063818	14549.21096	0.006365743
0.181392123	140.0866858	694.9655172	0.012988854
0.074327831	244.527342	12992.18815	0.003639855
0.019930849	313.6422828	27715.03451	0.00127385
0.034676554	275.305	13152.32776	0.00570395
0.04775211	285.5408657	4612.064433	0.004934633
0.101101126	192.2762648	15254.04918	0.011853085
0.150612895	194.2444259	462.2190476	0.022785328
0.04982645	317.4922927	22038.99044	0.003924629
0.012893465	560.0721635	17466.93201	0.005120174
0.044073335	251.4290242	24509.99315	0.005194281
0.024618776	352.389393	30026.41638	0.001409359
0.01653762	306.3898134	19850.11591	0.001452941
0.037808174	479.0126694	8207.233202	0.011559294
0.057684311	312.5452211	8778.244243	0.004928943
0.15778045	177.7720438	1512.609756	0.033748827
0.306507819	122.2881067	992.8575198	0.011065193
0.013038972	433.5970412	25712.43618	0.003425657

original_gldm_LowGrayLevelEmphasis	original_gldzm_DistanceZoneVariabilityNormalized	original_gldzm_LowIntensityEmphasis	original_gldzm_LargeDistanceEmphasis	original_gldzm_HighIntensitySmallDistanceEmphasis
0.004488113	0.850867575	0.006684073	1.243455497	612.8789267
0.007606453	0.862825789	0.010737443	1.222222222	589.0671296
0.005689637	0.880636815	0.008534019	1.19123506	685.12251
0.007071507	0.824633117	0.008749917	1.291344124	712.1289585
0.024217642	0.84537284	0.023761718	1.253333333	272.2677778
0.010315311	0.846881056	0.012599672	1.250614251	377.4404177
0.026779518	0.622006097	0.027051721	1.759036145	109.2123494
0.023893724	0.813939009	0.021223073	1.311418685	395.5601211
0.004743376	0.898828043	0.006492434	1.160326087	761.2361866
0.002908122	0.944073704	0.004570205	1.086376404	854.1917135
0.011197432	0.830519849	0.01306845	1.280434783	337.2086957
0.003583243	0.888688752	0.005677024	1.177464789	915.5496479
0.010219419	0.90201079	0.011825225	1.154991243	436.8476357
0.009068595	0.872992044	0.009901309	1.204444444	433.2518519
0.006688618	0.821135758	0.007317673	1.29787234	552.7367021
0.00277228	0.899219474	0.005243432	1.159668835	855.6788882
0.011447269	0.737139551	0.012164905	1.466981132	369.0123821
0.006841636	0.958352196	0.008483092	1.063829787	589.1482713
0.025895703	0.780716066	0.02254529	1.376068376	309.3539886
0.004070254	0.931664798	0.00618947	1.10626703	588.167802
0.002515864	0.87880192	0.004072751	1.194393382	642.7657399
0.002620855	0.825772492	0.00321469	1.289224953	835.4834594
0.005215727	0.91869228	0.006695199	1.127369219	546.5507961
0.018413241	0.657830663	0.015676489	1.657243816	371.110424
0.002524051	0.886491599	0.004102535	1.181208054	850.0604027
0.018698762	0.940756173	0.023428988	1.091666667	562.5729167
0.004040843	0.868996953	0.007140545	1.211401425	642.1633017
0.006530452	0.94816358	0.009027503	1.079881657	643.5835799
0.014894868	0.912007481	0.014269697	1.138370952	556.0500491
0.004627547	0.917575118	0.007346771	1.129201681	721.9952731
0.003872049	0.951739824	0.005844314	1.074226804	670.2274914
0.00275656	0.932888884	0.003537426	1.104292302	764.0390209
0.009742351	0.893166592	0.011399291	1.169868554	646.213094
0.063957171	0.73964497	0.048644144	1.461538462	127.1153846
0.010949049	0.787387777	0.012157901	1.362790698	492.0627907
0.009254305	0.820876512	0.009001826	1.298357664	483.1181569
0.004666613	0.93536204	0.006905336	1.100311042	768.0651244
0.004908495	0.900390833	0.006360727	1.157703927	646.0904834
0.011187039	0.780845178	0.01387709	1.375809935	413.8191145
0.032965817	0.903818874	0.038070101	1.151969981	451.8597561
0.004602974	0.811768834	0.007031081	1.315533981	852.4466019
0.011278188	0.835764743	0.011718913	1.27079646	384.3225664
0.006339921	0.860517613	0.006471965	1.226293103	535.8332435
0.03042962	0.817526314	0.023339014	1.30464716	358.2870052
0.00581392	0.856225223	0.006193702	1.233898305	568.1625
0.005269363	0.970795123	0.008458467	1.044466403	510.8589427
0.002707625	0.804813409	0.003273169	1.328820961	851.5762009
0.010204763	0.889273356	0.012070811	1.176470588	453.1442577
0.100371373	0.701070791	0.091115042	1.548780488	100.277439
0.013488783	0.746698435	0.016144203	1.446366782	375.7404844
0.006365209	0.917974949	0.008144065	1.128545564	533.0173506
0.019666352	0.89236046	0.020876425	1.171232877	386.8487443
0.010336482	0.895123344	0.014219246	1.166562695	558.639582
0.006450618	0.850703125	0.009104034	1.24375	460.1773438
0.004693911	0.949663251	0.007655545	1.077507599	766.4822695
0.041924929	0.808673469	0.030754345	1.321428571	574.8641917
0.007210893	0.716684191	0.007110821	1.512539185	569.6935737
0.027608004	0.682611027	0.024109304	1.593495935	315.2842141
0.006972209	0.841556104	0.008509601	1.260240964	654.1331325
0.006236017	0.943596012	0.005449857	1.087136929	703.560166
0.006029635	0.914314158	0.007841513	1.134564644	586.1521548
0.004797553	0.898207208	0.005293017	1.161369193	702.101467
0.003733223	0.921166086	0.006156486	1.123320158	657.7711462
0.006719132	0.951603667	0.010220127	1.074441687	768.5657568
0.019610288	0.90997582	0.026216614	1.141732283	459.519685
0.009286109	0.87371629	0.01237255	1.203187251	672.7642762
0.011243888	0.72588014	0.010616954	1.491803279	393.272541
0.005735781	0.928236247	0.006976437	1.111813013	618.1997789
0.02869511	0.858935537	0.035052861	1.229090909	305.0781818
0.005699803	0.88548483	0.007386512	1.182926829	640.2533028
0.005506647	0.884723816	0.007415474	1.184227537	700.2895928
0.009119275	0.921268547	0.011125661	1.123152709	485.0775862
0.005428205	0.911726171	0.007669072	1.138835878	566.0320849
0.004597591	0.950918922	0.00746692	1.075522851	661.3062548
0.002912094	0.926698698	0.004415637	1.114307343	1118.719909
0.00834238	0.776190932	0.008789893	1.385164051	569.5549215
0.012180832	0.82212428	0.013360555	1.296023564	440.7945508
0.008674303	0.893952784	0.010730451	1.168539326	467.6882022
0.007858896	0.82486685	0.009039238	1.290909091	574.1292929
0.004651028	0.836860309	0.005918601	1.268792711	563.6025057
0.007946354	0.813738796	0.00901894	1.311797753	772.7514045
0.006397506	0.790130491	0.006865459	1.357377049	672.2281421
0.0112289	0.874467665	0.013573822	1.201884253	842.0893338
0.036477634	0.688779213	0.033007812	1.578313253	169.0391566
0.00978707	0.945296712	0.009463907	1.084431138	533.9465569
0.002345263	0.731448469	0.003531265	1.479452055	767.2549087
0.009420584	0.8972994	0.011810495	1.162895928	493.9066742
0.009171995	0.782882798	0.009624577	1.37173913	455.8184783
0.020711865	0.768888889	0.032991708	1.4	462.9833333
0.036643865	0.731989078	0.038097918	1.47826087	236.1521739
0.007800521	0.875892528	0.010349617	1.19941691	744.9408163
0.005891401	0.958158665	0.008870493	1.064133017	992.5593824
0.008895917	0.86106762	0.012868687	1.225321888	559.9420601
0.002796771	0.894139226	0.0036306	1.168224299	819.3826278
0.002514529	0.90732216	0.003764261	1.146135266	731.3993559
0.014279851	0.745	0.019046033	1.45	707.50625
0.009949842	0.925706327	0.010918555	1.115919629	634.3249614
0.043592438	0.821978357	0.053517446	1.296296296	253.4567901
0.029847963	0.75115468	0.025148832	1.436893204	215.6541262
0.004089011	0.953029826	0.007666394	1.072192513	1033.326872

original_gldzm_LowIntensityLargeDistanceEmphasis	original_gldzm_HighIntensityEmphasis	original_gldzm_DistanceZoneVariability	original_gldzm_ZonePercentage	original_gldzm_IntensityVariabilityNormalized
0.006925867	684.0602094	325.0314136	0.541076487	0.02831611
0.011104421	636.5701058	652.2962963	0.363986519	0.027676297
0.008786108	739.752988	221.0398406	0.578341014	0.028396375
0.010418063	768.2744546	1171.803659	0.539278937	0.023648985
0.024413499	309.1377778	190.2088889	0.626740947	0.035614815
0.012966341	428.5601966	344.6805897	0.632970451	0.03063707
0.029925283	186.4578313	103.253012	0.492581602	0.048265351
0.02180128	460.733564	470.4567474	0.576846307	0.029597347
0.006761132	794.7871377	992.3061594	0.531024531	0.026704605
0.004655402	878.2401685	1344.360955	0.459651388	0.028124408
0.013610837	382.7695652	382.0391304	0.556227328	0.032967864
0.005867835	969.1661972	630.9690141	0.479082321	0.024157905
0.012276575	454.6716287	1030.096322	0.550626808	0.03044249
0.010418739	463.7185185	589.2696296	0.603756708	0.030637037
0.007954523	605	463.1205674	0.61909989	0.027614305
0.005462219	894.9432289	1520.58013	0.243414423	0.031366181
0.013015689	456.6509434	312.5471698	0.607449857	0.029770381
0.008554168	605.3643617	1081.021277	0.483704974	0.02755929
0.023733856	368.9373219	274.031339	0.603092784	0.0314202
0.006321564	611.5349682	1025.762943	0.393073902	0.029622975
0.004332454	686.0206801	1912.272978	0.416539051	0.032392139
0.006439115	918.8412098	873.6672968	0.615116279	0.029159416
0.00687324	573.0742987	1211.755118	0.48297327	0.031600318
0.017726	491.335689	186.1660777	0.588357588	0.028855398
0.004309121	899.8926174	528.3489933	0.495016611	0.031608937
0.023516849	587.875	338.6722222	0.518731988	0.028225309
0.00759015	676.7339667	1097.543151	0.364396999	0.029190011
0.009195954	661.0754438	640.9585799	0.471737613	0.030386716
0.014556109	583.703631	929.3356232	0.57733711	0.026288545
0.007501861	754.5367647	873.5315126	0.374508261	0.027454364
0.005966558	686.8151203	1384.781443	0.457691098	0.029955716
0.003669798	788.203973	2629.813764	0.475379427	0.027974542
0.012173213	675.7815976	883.3417594	0.486473192	0.025507069
0.049414295	210.0769231	48.07692308	0.625	0.053254438
0.012801521	578.3023256	169.2883721	0.647590361	0.028491076
0.009639193	531.2755474	899.6806569	0.542574257	0.027913381
0.007024383	796.0046656	1202.875583	0.446527778	0.024853126
0.006885008	673.0193353	1490.146828	0.493440668	0.027653271
0.014940997	477.6177106	361.5313175	0.554491018	0.029990344
0.03830907	481.2645403	481.7354597	0.508102955	0.027783547
0.007410278	936.2135922	501.6731392	0.4192673	0.023863386
0.012221651	433.8902655	472.2070796	0.592864638	0.03113478
0.006773344	588.7478448	798.5603448	0.565164434	0.02855139
0.023987054	416.4578313	474.9827883	0.566829268	0.030077527
0.006992554	608.7686441	1010.345763	0.628328009	0.027550991
0.008525871	519.0059289	982.444664	0.454219031	0.032688763
0.003842534	932.5244541	1843.022707	0.536802625	0.024908755
0.012259077	497.0560224	317.4705882	0.612349914	0.030153238
0.092138129	195.2439024	57.48780488	0.611940299	0.051160024
0.017008098	459.7716263	215.7958478	0.581488934	0.033584368
0.008395728	557.092939	1521.08449	0.483936916	0.028806668
0.021183761	422.1210046	390.8538813	0.529625151	0.031848794
0.014512727	589.9875234	1434.88272	0.419523685	0.027383544
0.009399389	514.409375	272.225	0.541455161	0.032128906
0.00776428	783.1752786	1874.635258	0.302621493	0.029780767
0.031824442	644.3139098	430.2142857	0.60523322	0.0238849
0.007974521	711.4153605	914.4890282	0.581851345	0.024729513
0.025494699	430.7222222	503.7669377	0.570765661	0.030423543
0.008809965	722.8240964	349.2457831	0.50982801	0.025786036
0.005565796	722.4564315	454.813278	0.547105562	0.031929547
0.008014224	617.4582234	1039.575198	0.536573856	0.027178096
0.005665802	729.299511	2204.200489	0.447973713	0.026792908
0.006381131	679.1849802	1165.275099	0.409385113	0.03075505
0.010300713	786.5397022	766.9925558	0.295129989	0.026279332
0.026371643	492.1889764	115.5669291	0.585253456	0.032550065
0.013800391	711.3559097	657.9083665	0.484867997	0.025038403
0.01126149	522.1557377	177.1147541	0.61928934	0.032585327
0.007152526	643.1857233	1469.397979	0.495151705	0.027051078
0.035435103	346.5163636	236.2072727	0.616591928	0.033626446
0.007686993	680.0193089	871.3170732	0.489308802	0.027348139
0.007676817	743.6354234	1368.667744	0.508881579	0.024935975
0.011334901	508.8965517	561.0525452	0.539415412	0.028189635
0.007912569	592.3454198	1910.978053	0.405572755	0.028613663
0.007574152	678.4566228	2455.272657	0.384455033	0.028935051
0.00474026	1140.700984	3672.506939	0.431699346	0.02186316
0.009362712	667.2582026	544.1098431	0.628136201	0.024857499
0.013965663	498.5876289	558.2223859	0.607878245	0.029144913
0.011018266	498.4213483	318.247191	0.583606557	0.030299205
0.009361553	652.8747475	816.6181818	0.56122449	0.025679012
0.006335008	620.0273349	1102.145027	0.462754743	0.029803014
0.009268525	883.6629213	289.6910112	0.706349206	0.021793334
0.007788292	755.0535519	722.9693989	0.682326622	0.023617307
0.014357466	869.1736205	1299.45895	0.532234957	0.023379265
0.033987444	277.373494	57.1686747	0.572413793	0.045434751
0.009606175	548.8329341	1578.645509	0.492480094	0.030403385
0.007053524	859.3497717	1601.872146	0.399926954	0.027888076
0.012076168	523.6945701	396.6063348	0.568123393	0.029268442
0.010098884	547.4097826	720.2521739	0.592783505	0.028388469
0.033972762	524.8933333	57.66666667	0.409836066	0.045688889
0.039299413	308.2608696	50.50724638	0.657142857	0.043058181
0.010706756	786.3172012	1502.155685	0.431446541	0.02365987
0.00891066	1020.04038	403.3847981	0.59631728	0.021896739
0.013439997	597.776824	401.2575107	0.455968689	0.027675956
0.003839639	860.6190214	1626.439252	0.444743276	0.026014269
0.003983339	764.0394525	2253.788245	0.427833276	0.029215968
0.01939671	871.775	119.2	0.632411067	0.0240625
0.011467734	644.8423493	598.9319938	0.532072368	0.026327671
0.053925689	315.6419753	66.58024691	0.658536585	0.03947569
0.027214041	253.6165049	154.7378641	0.54353562	0.039494769
0.007707823	1065.510695	356.4331551	0.463444857	0.026566387

original_gldzm_LowIntensitySmallDistanceEmphasis	original_gldzm_IntensityVariability	original_gldzm_HighIntensityLargeDistanceEmphasis	original_gldzm_SmallDistanceEmphasis	original_glcm_SumVariance
0.006623624	10.81675393	968.7853403	0.939136126	3293.385824
0.010645699	20.92328042	826.5820106	0.944444444	3288.885071
0.008470997	7.12749004	958.2749004	0.952191235	3293.373354
0.008332881	33.6052076	992.8564391	0.927163969	2954.569759
0.023598772	8.013333333	456.6177778	0.936666667	1008.118281
0.012508005	12.46928747	633.039312	0.937346437	1692.749511
0.02633333	8.012048193	495.439759	0.810240964	390.4128551
0.021078521	17.10726644	721.4273356	0.922145329	1854.75777
0.006425259	29.48188406	928.990942	0.959918478	2995.086428
0.004548906	40.0491573	974.4339888	0.978405899	3756.188104
0.012932853	15.16521739	565.0130435	0.929891304	1594.098522
0.005629322	17.15211268	1183.632394	0.955633803	4035.745131
0.011712388	34.76532399	525.9676007	0.961252189	1555.306937
0.009771951	20.68	585.5851852	0.948888889	1668.304394
0.007158461	15.57446809	814.0531915	0.925531915	1815.193177
0.005188736	53.04021289	1052.000591	0.960082791	4452.459524
0.011952209	12.62264151	807.2051887	0.883254717	1676.377857
0.008465323	31.08687943	670.2287234	0.984042553	2713.392657
0.022248148	11.02849003	607.2706553	0.905982906	1372.126574
0.006156446	32.61489555	705.0036331	0.973433243	3046.530001
0.004007825	70.48529412	859.0404412	0.951401654	3289.490601
0.002408584	30.85066163	1252.272212	0.927693762	3402.978329
0.006650689	41.6808188	679.1683093	0.968157695	2571.850609
0.015164112	8.166077739	972.2367491	0.835689046	1542.433819
0.004050889	18.83892617	1099.221477	0.954697987	3885.978072
0.023407023	10.16111111	689.0833333	0.977083333	2589.118152
0.007028143	36.86698337	815.0166271	0.947149644	2770.252127
0.00898539	20.54142012	731.0428994	0.980029586	3090.009377
0.014198093	26.78802748	694.3179588	0.965407262	1996.500397
0.007307998	26.13655462	884.7027311	0.96769958	4010.023716
0.005813753	43.58556701	753.1656357	0.981443299	3128.986085
0.003504333	78.86023413	884.8637815	0.973926924	3233.011897
0.011205811	25.22649141	794.0556117	0.957532861	2714.512867
0.048451607	3.461538462	541.9230769	0.884615385	558.831397
0.011996997	6.125581395	923.2604651	0.909302326	2166.54574
0.008842484	30.59306569	723.9051095	0.925410584	1986.456852
0.006875574	31.96111975	907.7628305	0.97492224	3982.50475
0.006229657	45.76616314	780.7347432	0.960574018	2718.380865
0.013611113	13.88552916	732.812095	0.906047516	1537.875307
0.038010358	14.80863039	598.8836773	0.962007505	2155.401816
0.006936282	14.74757282	1271.281553	0.921116505	4108.617915
0.011593229	17.59115044	632.1610619	0.932300885	1704.188142
0.00639662	26.49568966	800.40625	0.943426724	2065.516791
0.023177003	17.47504303	649.141136	0.92383821	1462.219546
0.00599399	32.51016949	771.1932203	0.941525424	1857.90731
0.008441616	33.08102767	551.5938735	0.988883399	2423.395014
0.003130827	57.04104803	1256.317467	0.91779476	3428.936916
0.012023744	10.76470588	672.7030812	0.955882353	2156.128799
0.09085927	4.195121951	575.1097561	0.862804878	657.5369703
0.015928229	9.705882353	795.8961938	0.888408304	1557.056819
0.00808115	47.73264937	653.3952927	0.967863609	2336.807437
0.020799591	13.94977169	563.2100457	0.957191781	1897.643422
0.014145876	43.89582034	715.3792888	0.958359326	2645.819988
0.009030195	10.28125	731.3375	0.9390625	2127.637552
0.007628362	58.78723404	849.9473151	0.9806231	3696.542845
0.03048682	12.70676692	922.112782	0.919642857	2554.624696
0.006894896	31.55485893	1278.302508	0.871865204	2627.602801
0.023762955	22.45257453	892.4742547	0.851626016	1091.06894
0.00843451	10.70120482	997.5879518	0.934939759	3249.24448
0.005420872	15.39004149	798.0414938	0.978215768	2620.906254
0.007798335	30.90149516	742.6824978	0.966358839	2639.43521
0.005199821	65.74979625	838.091687	0.959657702	3076.725749
0.006100324	38.90513834	764.8403162	0.96916996	2763.430726
0.010199981	21.18114144	858.4354839	0.981389578	3030.821794
0.026177857	4.133858268	622.8661417	0.964566929	2050.625035
0.01201559	18.85391766	865.7224436	0.949203187	3009.425196
0.010455819	7.950819672	1037.688525	0.87704918	1901.154127
0.006932414	42.82185723	743.1295009	0.972046747	2901.129323
0.034957301	9.247272727	512.2690909	0.942727273	1562.552029
0.007311392	26.91056911	839.0833333	0.954268293	2895.428887
0.007350138	38.57595346	917.018746	0.953943116	3028.269396
0.011073351	17.16748768	604.1724138	0.969211823	2208.770158
0.007608198	59.97423664	697.5987595	0.965291031	2893.9404
0.007440112	74.71030209	747.0580945	0.981119287	3243.116839
0.004334481	86.64370426	1228.625284	0.971423164	4349.006474
0.008646689	17.42510699	1058.071327	0.903708987	2281.649739
0.013209278	19.78939617	729.7599411	0.925994109	1914.015779
0.010658497	10.78651685	621.3539326	0.957865169	2202.842994
0.008958659	25.42222222	967.8565657	0.927272727	2736.977555
0.0058145	39.25056948	845.7266515	0.932801822	2813.794277
0.008956543	7.758426966	1327.308989	0.922050562	3540.74737
0.006634751	21.60983607	1086.355191	0.910655738	2513.08031
0.013377911	34.74158816	977.5107672	0.949528937	3165.710761
0.032762904	3.771084337	710.7108434	0.855421687	760.3098945
0.00942834	50.77365269	608.3784431	0.978892216	2232.213641
0.002650701	61.07488584	1227.729224	0.880136986	3522.875642
0.011744077	12.93665158	642.8461538	0.959276018	2387.892616
0.009506	26.1173913	913.775	0.907065217	2104.383987
0.032746444	3.426666667	772.5333333	0.9	2171.905093
0.037797544	2.971014493	596.6956522	0.880434783	771.906961
0.010260333	40.57667638	951.8227405	0.950145773	2977.630368
0.008860451	9.218527316	1129.964371	0.983966746	4489.526239
0.01272586	12.89699571	749.1158798	0.943669528	2794.707266
0.00357834	47.31995602	1025.564596	0.957943925	3569.40643
0.003709492	72.57246377	894.599839	0.963466184	3424.458512
0.018958364	3.85	1528.85	0.8875	3360.918785
0.010781261	17.03400309	686.9119011	0.971020093	1900.427308
0.053415385	3.197530864	564.382716	0.925925926	1091.409987
0.02463253	8.13592233	405.4660194	0.890776699	620.4223672
0.007656037	9.935828877	1194.245989	0.981951872	4915.268024

original_glcm_Homogeneity1	original_glcm_Homogeneity2	original_glcm_ClusterShade	original_glcm_MaximumProbability	original_glcm_Idmn	original_glcm_SumVariance2	original_glcm_Contrast	original_glcm_DifferenceEntropy
0.26493643	0.201582764	-1897.885425	0.06371064	0.928127725	427.6670081	175.2832024	4.593711975
0.346384008	0.282980458	-8331.761992	0.054279351	0.963342458	416.3504437	117.8266231	4.168042814
0.239036645	0.166288779	-3161.468971	0.024302853	0.934251202	321.7784289	151.8559492	4.519746748
0.227853214	0.157273951	-1556.991419	0.034755161	0.970400835	574.6568148	198.3246934	4.774741676
0.205137601	0.120534196	2013.876381	0.010037862	0.935699641	257.6725044	112.6315663	4.330155095
0.201274068	0.119709353	7.546100785	0.007515032	0.930101813	285.127338	122.8379741	4.459048344
0.273072985	0.187878275	733.185413	0.022291817	0.952724331	96.84768274	69.83207582	3.984907904
0.206776942	0.137089904	-28.53590452	0.015451755	0.901828793	450.2239657	208.1509428	4.788993812
0.22823698	0.150795429	-2140.335899	0.011943217	0.970758447	388.4241671	140.1498231	4.567986752
0.287730756	0.216689358	-3882.928583	0.051520267	0.980331483	299.1433299	113.2776098	4.370055021
0.20896705	0.131404496	975.8053716	0.016345009	0.921144068	304.7943692	141.354666	4.539682366
0.271394975	0.200799924	-3246.998863	0.033778199	0.978732054	406.9801293	140.1425619	4.462402224
0.225756137	0.141112767	195.340317	0.007646808	0.944062869	273.7195253	95.55205875	4.319065762
0.214941589	0.130786727	-354.4725673	0.006907026	0.942453891	283.7300252	103.9315801	4.34954524
0.200532909	0.116361747	911.6351414	0.006331582	0.953007935	282.7997334	119.8791911	4.467087274
0.435270127	0.381590969	-9842.237517	0.095364056	0.979147503	358.9236587	70.33847671	3.642857716
0.196423034	0.115187059	919.4524442	0.00706573	0.932543476	336.6152644	136.8504883	4.486387404
0.2582371	0.186232601	-3311.136076	0.029475354	0.955084458	432.7296336	136.5504332	4.47564003
0.201032655	0.121597921	1543.750281	0.008278093	0.924967184	334.2340002	142.0616916	4.54265002
0.361131627	0.299238999	-4225.500005	0.124731061	0.959855971	332.6237142	87.29005829	4.079851811
0.334842058	0.269983964	-2893.384421	0.113633391	0.951989876	229.4541128	92.65126241	4.197031517
0.207764074	0.12552025	-1444.630258	0.008147258	0.957700863	279.7895047	115.5178788	4.44888235
0.27541118	0.202008955	-2541.037838	0.051781542	0.948193093	269.5344934	103.8060839	4.341676214
0.210062856	0.133668273	5802.970444	0.010633531	0.933681131	464.9745107	158.8742204	4.605835272
0.27244111	0.196298519	-2602.328026	0.032577318	0.957510825	224.1345228	100.6059188	4.289666629
0.263918229	0.196287398	-4259.423603	0.042670119	0.929659908	395.5441372	166.6862804	4.548741686
0.335120992	0.265137925	-2672.754672	0.057663062	0.98304908	255.7588355	75.0343444	4.030265454
0.285961127	0.216047869	-4533.150071	0.041740424	0.941466685	278.8920666	123.285148	4.385481046
0.215015205	0.145399818	2100.475714	0.016123302	0.912487312	524.0010758	223.1489498	4.801909565
0.352145759	0.292232123	-8276.87565	0.085165428	0.950894203	384.8397296	119.8245113	4.163040139
0.282957864	0.213745909	-3613.083556	0.050262565	0.943988209	264.2895189	114.9980312	4.382039438
0.274390821	0.196760515	-3068.473167	0.03666537	0.969724744	335.2868925	87.50396789	4.233109281
0.256558734	0.188986729	-2077.398291	0.049350127	0.966120343	576.4882303	180.8502307	4.655422088
0.207080307	0.130817369	2162.246605	0.031387123	0.905966801	196.4159935	159.9216043	4.221755751
0.188562636	0.115990665	1763.806871	0.016888181	0.9200963	401.5358134	203.2870182	4.722002465
0.220611415	0.14573263	614.3874789	0.018482832	0.931642356	415.1460504	148.6850262	4.602454963
0.307979571	0.249613847	-7495.839862	0.093194566	0.957880657	528.3894546	183.8748696	4.539119313
0.261558675	0.18570363	-2348.282356	0.034808708	0.981689829	347.161603	106.8797773	4.394336481
0.217573797	0.135470063	1712.948029	0.006793149	0.943278234	308.815696	112.8029061	4.346582841
0.258940592	0.19358256	-2191.466842	0.055259632	0.96374952	471.7677671	166.1026406	4.598495658
0.329245464	0.266166724	-6753.576546	0.073165149	0.973098926	443.8287425	112.3125788	4.227683579
0.209155163	0.132725493	1268.819596	0.019433067	0.923838544	366.7673892	152.5065724	4.61339386
0.208886215	0.134975396	1400.957355	0.010785175	0.928246515	389.5232252	172.0456515	4.654471226
0.213415862	0.141296317	1264.854106	0.011480386	0.934194883	400.2853428	176.7172712	4.673925233
0.193175197	0.111670019	1230.119858	0.004943273	0.942736811	311.1524666	135.9283002	4.561014199
0.295004628	0.223143853	-2680.025253	0.036512088	0.947824993	227.5620908	96.25703844	4.194656401
0.2309523	0.154242149	-690.3876444	0.01223301	0.980583016	402.6761643	139.7815594	4.579792876
0.209898993	0.136900242	-56.93811569	0.019232932	0.920900625	385.2195571	166.8644731	4.637479989
0.216211807	0.136779936	3394.600493	0.019875549	0.91793638	247.5171132	145.9787666	4.241410101
0.208180675	0.131946716	4072.66536	0.011678748	0.932288346	359.9605633	161.2076205	4.614187804
0.265097849	0.19239265	-2511.764407	0.035194702	0.951042068	358.7141587	117.2133449	4.411608011
0.227832937	0.161551895	-332.5210107	0.026447939	0.905804926	461.1324397	199.273175	4.680693207
0.293087138	0.228242337	-4260.209957	0.049879679	0.975733261	459.5562092	145.2173343	4.435438106
0.247474155	0.165982593	-1359.236334	0.020262339	0.963855938	271.5407574	93.71315608	4.262640895
0.389518064	0.33434309	-9673.480825	0.091152115	0.968077887	424.4621499	110.8205939	3.95976584
0.206711881	0.138536698	-2038.098213	0.017284472	0.915708586	622.9678322	230.7250269	4.872586825
0.196257823	0.12260726	349.0995606	0.007643764	0.936316605	470.7805506	209.2128008	4.786632875
0.203969397	0.125961301	5325.97455	0.007812941	0.937253669	355.6638773	170.7453247	4.67698213
0.260645675	0.188390464	-4165.116922	0.022250239	0.958861397	474.7905706	139.4947609	4.459077676
0.235373524	0.149515393	-774.9232802	0.014396472	0.984777664	240.3169053	85.84832401	4.188435669
0.228554587	0.156751612	-1932.643578	0.019368032	0.934230558	384.7767382	162.7616426	4.64304845
0.287032666	0.223256553	-2031.163524	0.074735367	0.956314643	536.3466614	160.9345289	4.562866299
0.304692176	0.231569436	-3016.114517	0.040232777	0.980861487	286.8589418	84.40869002	4.124433339
0.401188589	0.344432852	-5751.507024	0.100845321	0.988444259	339.6937327	93.52900815	3.875451658
0.243333019	0.166931851	-2588.632821	0.033636138	0.940381552	349.95995	134.3699572	4.384888808
0.261398566	0.189200857	-2913.899545	0.022231756	0.947327365	584.0723526	155.3593383	4.531793593
0.187797031	0.110778739	2219.031617	0.010227368	0.924831028	317.703981	180.0506006	4.659296929
0.259283588	0.192755386	-3348.348748	0.0427688	0.935605883	493.7789127	169.733011	4.609868188
0.20182603	0.126601831	677.9291689	0.018399376	0.923412582	363.9769761	159.5862206	4.602815116
0.250820158	0.183279133	-2639.628166	0.031518917	0.952889985	449.2345381	172.2168768	4.625149508
0.240356097	0.165878536	-4195.2177	0.014758863	0.959957958	420.6935119	149.0960296	4.538427486
0.228000268	0.155333253	-1518.397701	0.019159296	0.926843886	386.4406019	153.4626369	4.590388896
0.322351268	0.258821305	-4458.810755	0.074609519	0.967328483	354.3594932	116.4313299	4.307163286
0.326264371	0.261137489	-5888.376795	0.055826504	0.958543771	331.2747198	104.4949882	4.196323274
0.299702454	0.224023579	-7846.688761	0.021272825	0.983609629	490.6926426	97.2099994	4.213876292
0.185209576	0.110463427	1164.305921	0.006145463	0.929401434	467.9624483	209.0413175	4.814244665
0.206865503	0.128802062	-749.8707522	0.007811456	0.927741677	361.4108629	151.0808059	4.580412519
0.218844009	0.14492972	-1687.203941	0.020852848	0.918554123	321.430718	157.6357055	4.594576554
0.21940364	0.147123197	-2830.988825	0.01789199	0.932182244	427.2216108	168.3892444	4.654238774
0.279822833	0.215248181	-2445.13805	0.04546008	0.933227415	382.0511841	147.0657606	4.511417723
0.175136164	0.101343994	-1947.07139	0.009799666	0.944312525	596.418495	235.8822621	4.849379201
0.173825141	0.099402984	2196.44465	0.005762428	0.940211466	486.3459321	231.932365	4.922286146
0.234620921	0.165325789	-2271.632614	0.020575744	0.977602746	717.8212169	214.5248189	4.779355992
0.230314161	0.150464572	2471.841534	0.019261239	0.920216874	224.9858155	126.2623964	4.192613544
0.256784629	0.182495346	-2394.11076	0.026819465	0.948286858	282.0216456	113.7143904	4.406372714
0.31244769	0.239228558	-3127.891529	0.040287245	0.987572394	339.4057757	80.07354659	4.106384116
0.231053874	0.159089838	-1513.223937	0.024909781	0.92981915	333.9212267	145.2784262	4.561873085
0.194467205	0.120300991	2683.56418	0.006997312	0.923557504	473.9087286	192.9968074	4.761762697
0.339717362	0.275937377	-4869.323459	0.055912112	0.972111331	421.7398337	116.8121953	3.755626431
0.20110795	0.129022893	1599.860127	0.033177407	0.900244725	211.7175427	180.0653023	4.259761585
0.285927652	0.218407497	-4613.894813	0.036520593	0.978792602	505.2311115	155.9503643	4.475010338
0.22097443	0.151211238	-5803.648626	0.021036731	0.959412944	485.5766261	215.7976185	4.779083118
0.287181863	0.217487613	-4882.408299	0.031231422	0.958174249	439.8456869	127.4705337	4.352179981
0.288546204	0.219551915	-3310.247101	0.043007505	0.976907951	380.0506708	116.3468227	4.372559987
0.294969391	0.221747858	-3934.053788	0.042493328	0.980834718	294.2841643	92.81716536	4.206791644
0.201045108	0.131286469	-1203.204388	0.013838028	0.920421019	644.6094614	276.1140306	4.753143951
0.236083793	0.158573864	8984.787901	0.013124296	0.97642598	544.3683513	137.8608981	4.514832299
0.201558925	0.126280285	2559.846268	0.022230288	0.899074191	314.0504131	174.506418	4.383752589
0.247132732	0.160104241	634.5273807	0.014092097	0.9470743	158.7199537	67.40043672	4.042254046
0.291566375	0.218588749	-7395.29149	0.027751592	0.953369751	324.5296303	140.7219434	4.339979592

original_glcm_InverseVariance	original_glcm_Entropy	original_glcm_Dissimilarity	original_glcm_DifferenceVariance	original_glcm_Idn	original_glcm_Idm	original_glcm_Correlation	original_glcm_Autocorrelation
0.157828741	8.352594704	9.731762982	76.18065879	0.839964708	0.201582764	0.422813977	943.2667013
0.237295114	8.25138078	6.997604084	66.88519202	0.896449254	0.282980458	0.558358513	950.5245429
0.165912448	8.226705543	9.102404058	66.68906483	0.844304638	0.166288779	0.362032795	949.0692598
0.143026224	9.687836093	10.4107369	86.18819036	0.891681039	0.157273951	0.49401499	851.5363587
0.1274461	8.394142307	8.337680707	39.63355838	0.835210495	0.120534196	0.406098197	310.1220539
0.127770433	9.026659685	8.772968467	44.08095804	0.828377286	0.119709353	0.406654173	511.5852147
0.192990542	7.672888982	6.125057517	31.04496553	0.865330191	0.187878275	0.177530101	129.5859182
0.131970635	9.214580463	11.09073567	81.90984676	0.807047867	0.137089904	0.375654014	535.3445183
0.149210212	9.575782727	8.872999852	59.74195143	0.890443565	0.150795429	0.477705982	878.1528999
0.193460476	8.93939674	7.531373059	55.1048629	0.913706284	0.216689358	0.457110194	1088.068668
0.119974104	9.065864332	9.350610051	49.95176904	0.8208095	0.131404496	0.375317068	478.200462
0.191591434	8.892372534	8.379335568	67.28076929	0.910764436	0.200799924	0.491297711	1161.528306
0.142106403	9.60100003	7.615048507	35.73123808	0.846968025	0.141112767	0.486869506	480.3104217
0.140871292	9.347994573	7.971404907	38.55027245	0.844345608	0.130786727	0.470665375	510.4491813
0.119518078	9.372039832	8.608984528	44.21267387	0.856709146	0.116361747	0.411993558	549.4575126
0.305957273	7.678093003	4.836233757	45.47620776	0.928159257	0.381590969	0.677799457	1268.318716
0.118173102	9.141252769	9.240480248	46.13066442	0.831696436	0.115187059	0.431806045	503.5817191
0.176634279	9.229180601	8.503440798	60.54748488	0.871242558	0.186232601	0.527953844	796.2732529
0.123585233	8.994648353	9.325525578	51.68301882	0.824712612	0.121597921	0.413174637	412.6735302
0.208345026	8.301576019	6.35243532	45.4517047	0.886243141	0.299238999	0.586402911	892.1819763
0.202301532	8.303537335	6.698601834	46.92446789	0.873272549	0.269983964	0.430399851	954.8819847
0.131588867	9.586392845	8.442340669	42.23900619	0.863632601	0.12552025	0.423958178	997.2676563
0.164670688	8.939036848	7.536277526	44.85555156	0.860100225	0.202008955	0.447917447	762.2783693
0.133261046	8.759931057	9.61207985	64.28921326	0.838012216	0.133668273	0.495991313	454.515933
0.189394924	8.566104933	7.274773235	46.26686655	0.874354817	0.196298519	0.386938777	1122.115535
0.180356988	8.460641249	9.243875205	78.09351126	0.845100818	0.196287398	0.413653631	750.3344268
0.241977194	8.482734029	5.875739132	39.97706493	0.92275799	0.265137925	0.550620934	821.4575933
0.192230656	8.441980843	7.840850512	60.0955046	0.858682467	0.216047869	0.390217591	897.0345476
0.134577268	9.527289501	11.19061062	90.58367389	0.820837532	0.145399818	0.411742571	572.3078863
0.250654835	8.134252282	7.045987435	68.94922025	0.88175335	0.292232123	0.527074593	1143.414945
0.192992038	8.764195211	7.736628331	53.58299856	0.858858731	0.213745909	0.401119718	911.4405247
0.179081325	9.41091514	6.921027623	37.55011721	0.890054998	0.196760515	0.588356874	954.6879204
0.165026887	9.236753887	9.706006563	83.27498605	0.887988446	0.188986729	0.529512513	785.7547083
0.131435882	6.607330983	9.792605815	60.32876678	0.80865699	0.130817369	0.122105885	152.2140607
0.112304024	8.311375534	11.14468572	74.44832486	0.82033078	0.115990665	0.34304182	623.4434796
0.139451911	9.626625407	9.32799308	58.17408223	0.835967648	0.14573263	0.478394505	590.1812028
0.196892527	8.644481731	9.282838635	94.22408101	0.883363274	0.249613847	0.489775028	1129.034365
0.173283428	9.37194249	7.633020137	47.74109098	0.912650927	0.18570363	0.532221756	806.8525295
0.140455003	9.193610117	8.252998885	39.83184495	0.846680223	0.135470063	0.471829735	469.5103005
0.163036255	8.635952765	9.519671626	72.80979333	0.881903687	0.19358256	0.484388459	628.8076794
0.232812133	8.29117287	7.123103938	60.02515029	0.906047505	0.266166724	0.598696026	1180.212566
0.128343925	9.179176187	9.558927245	58.77669993	0.825803838	0.132725493	0.422046257	506.9297776
0.138581913	9.568877962	10.05051076	64.81833795	0.832465872	0.134975396	0.39613641	607.0487368
0.1401619	9.238557846	10.16590448	68.14070396	0.839397782	0.141296317	0.397430705	429.208493
0.117474031	9.806598888	9.24481433	48.51985431	0.842943972	0.111670019	0.401284312	558.2780132
0.217100931	8.469268668	6.956172836	44.88596976	0.865838919	0.223143853	0.416920817	716.8933032
0.157252235	9.943342917	8.787163446	61.18616967	0.909569966	0.154242149	0.492159432	1001.265066
0.132796359	8.782690208	10.01314959	63.28788932	0.823033437	0.136900242	0.403983921	632.0338234
0.141599455	7.06340081	9.140151057	59.17400719	0.823340608	0.136779936	0.274145128	187.0065542
0.133971269	8.640466125	9.734452575	63.59122504	0.836243536	0.131946716	0.390720949	459.6488344
0.182583448	9.297607271	7.9318212	52.45406499	0.865157426	0.19239265	0.512173232	694.981968
0.153934491	8.768811891	10.65547155	79.48986301	0.81443725	0.161551895	0.404065799	547.1072867
0.206067924	8.930526822	8.400228707	71.68002725	0.907127789	0.228242337	0.523960089	771.8627595
0.166241963	8.62391596	7.316792222	38.83860662	0.878227198	0.165982593	0.490540852	641.1388423
0.282807158	8.003481209	6.378680489	68.23809059	0.908895553	0.33434309	0.589706143	1056.329174
0.129463387	9.296799484	11.54340025	94.30750606	0.821213366	0.138536698	0.467203485	729.0846282
0.122054723	9.929082362	11.0399962	77.83363887	0.841631296	0.12260726	0.394050899	759.176823
0.126477092	9.454310695	9.893353978	68.06631673	0.842922746	0.125961301	0.364195462	320.2993454
0.189423422	8.74506322	8.456465784	66.19466467	0.878200674	0.188390464	0.550412994	943.0774595
0.157666062	8.83978432	7.087187201	33.25441386	0.916193471	0.149515393	0.484811249	782.2705831
0.15217803	9.467345475	9.628798284	67.44458928	0.84170528	0.156751612	0.413023363	771.0399783
0.1803347	9.225215679	8.957173587	78.06925953	0.877002734	0.223256553	0.544532876	888.5983461
0.216603156	8.811769364	6.491053047	39.63644045	0.915002543	0.231569436	0.55332303	819.9331626
0.27007655	7.747474115	5.870688003	57.64190911	0.942495795	0.344432852	0.570496175	883.2580922
0.148182582	7.601290453	8.607349919	58.94495178	0.850372359	0.166931851	0.449694541	603.2697893
0.182828606	9.242062732	8.775676264	75.39224067	0.865678219	0.189200857	0.579995494	873.1041463
0.108197841	8.579020507	10.52262711	65.45310549	0.824635096	0.110778739	0.290268986	555.125588
0.179390176	9.27801294	9.421339198	77.91832179	0.849883414	0.192755386	0.492211943	839.1619118
0.127549252	8.565752291	9.888289068	59.69881339	0.823798487	0.126601831	0.399566199	461.5271877
0.171720396	9.214988141	9.601939854	76.51802075	0.868279481	0.183279133	0.451835591	838.0569203
0.167361993	9.548140569	8.95685222	63.72849549	0.876720459	0.165878536	0.482438877	883.929823
0.152141279	9.21233415	9.399348626	61.88278246	0.832883999	0.155333253	0.437890386	651.5015146
0.20219601	8.71784705	7.451505967	58.8441226	0.89459823	0.258821305	0.511430461	845.6753027
0.236060454	8.63873257	6.909438061	55.11818009	0.884905945	0.261137489	0.525420075	942.6712861
0.221126043	9.456907955	6.672115401	52.01387735	0.923921305	0.224023579	0.671109818	1259.871115
0.113466226	9.666923733	11.18868667	78.07333122	0.83158519	0.110463427	0.391453986	660.2460125
0.139200974	9.412929324	9.439042599	58.36013172	0.831170109	0.128802062	0.41755791	568.7649917
0.142467145	8.689115016	9.612713491	62.6539138	0.822923804	0.14492972	0.349375609	646.4278817
0.143943749	9.502695095	9.886636879	65.93627891	0.83776978	0.147123197	0.438167556	797.8243447
0.179790708	8.971072777	8.785950875	67.70827396	0.847514643	0.215248181	0.449698114	819.4815893
0.103770094	8.961876988	11.92948324	88.24853804	0.848214376	0.101343994	0.443697966	1007.048116
0.105760767	9.865402488	11.82382264	89.059888	0.843149091	0.099402984	0.365363564	721.3197027
0.162779946	9.715264971	10.52262981	99.52247376	0.907205186	0.165325789	0.545413996	902.9122166
0.157569263	7.164546486	8.436801942	51.38900027	0.827205122	0.150464572	0.300642088	224.9066872
0.169175858	9.269860751	7.942075927	47.97058039	0.859128967	0.182495346	0.429470586	666.909297
0.231322755	9.020806076	6.176729109	40.87536536	0.931616789	0.239228558	0.621348533	1032.735157
0.147429738	8.802120141	9.223050242	58.22624344	0.834562092	0.159089838	0.401018	702.6345533
0.122836688	9.701611218	10.76338225	72.50452634	0.825727173	0.120300991	0.431585221	612.7178453
0.270511549	6.592262396	6.959756978	58.55469335	0.909395694	0.275937377	0.560189293	624.2597945
0.136586759	6.529619286	10.47077236	65.63930332	0.802595286	0.129022893	0.095675968	211.4419406
0.205782205	9.258484382	8.547733098	79.83576379	0.913882446	0.218407497	0.532364043	865.4582593
0.153392192	8.806677615	10.78137816	97.04710066	0.875797996	0.151211238	0.392894048	1270.130708
0.207818561	8.59781136	7.891144509	63.41561928	0.880608566	0.217487613	0.557409452	817.5551708
0.195524136	9.159313643	7.683344188	54.88143048	0.906734306	0.219551915	0.534641163	1041.683991
0.22338706	8.818668958	6.769065091	45.88935552	0.915405996	0.221747858	0.523943834	998.006994
0.133855035	8.050418745	12.36039551	114.9736762	0.830148285	0.131286469	0.401585774	937.0214958
0.15661952	9.281949703	8.609153104	61.82136683	0.902278198	0.158573864	0.601762914	563.3164937
0.128004008	7.017539309	10.24512001	63.69367692	0.802300734	0.126280285	0.297658647	310.0694681
0.161445287	8.231117485	6.443708072	25.01178605	0.849560657	0.160104241	0.406809512	204.2892702
0.210792705	8.306601577	7.824231682	77.90046871	0.880191463	0.218588749	0.394900435	1391.929302

original_glcm_SumEntropy	original_glcm_AverageIntensity	original_glcm_Energy	original_glcm_SumSquares	original_glcm_ClusterProminence	original_glcm_SumAverage	original_glcm_Imc2	original_glcm_Imc1
5.803808248	30.36821527	0.009205986	144.5531349	329549.3722	59.32932957	0.954841414	-0.254984979
5.595789151	30.13517898	0.011369784	128.5244645	477785.0914	59.18644435	0.880432823	-0.167603006
5.70919408	30.73047117	0.004898785	112.0004903	247280.7497	60.20956606	0.978218852	-0.319040615
6.259907484	28.52909226	0.003157961	189.1083012	614107.5817	55.04009158	0.870036937	-0.137977459
5.707451213	17.18440456	0.003390285	95.57119405	167673.8311	33.08662847	0.981823601	-0.328988554
5.88906194	22.394327	0.002268958	101.3589778	164851.9886	43.40038073	0.952402723	-0.232654405
5.03595359	11.43078274	0.006337183	44.97954723	30218.4874	22.15644482	0.937753897	-0.240367905
6.10557884	22.70158361	0.002525003	163.8400628	373382.7766	43.57309196	0.919868616	-0.184868275
6.079319643	29.42312315	0.002028702	128.5209847	305168.1585	57.13241843	0.861879215	-0.133105726
5.751288785	32.92629436	0.006935815	97.40919069	295290.9949	64.54704772	0.821374473	-0.119351399
5.921140969	21.67403906	0.002508015	112.0850515	191449.613	41.81576659	0.925762572	-0.194032611
5.93723895	33.7612982	0.005644328	131.2033969	464441.5732	66.17316056	0.924787023	-0.197052708
5.951565578	21.41936054	0.00158189	92.29275471	155169.1052	41.74868435	0.824666074	-0.115040477
5.941618265	22.20454453	0.001876431	96.31111817	163413.5619	43.14946264	0.898062214	-0.164164946
5.965248891	23.27597966	0.001805841	101.2067751	176424.3418	45.10655199	0.938137521	-0.203298885
5.191487808	34.99870791	0.024760912	100.4261499	515775.6867	69.16914111	0.853475022	-0.161463402
5.998792735	22.07006879	0.002082519	118.7086675	226482.6164	42.58458038	0.951983301	-0.230176723
5.99263008	27.6606612	0.004057408	137.3927847	350089.7916	53.74694051	0.871712842	-0.146632825
5.977461252	19.82930085	0.002307579	120.5219683	236708.5023	38.17935757	0.964873194	-0.258816748
5.555278291	29.21580989	0.021116791	101.7567856	262420.3916	57.64355419	0.869506021	-0.161666433
5.367905797	30.91461034	0.019090841	75.11788538	146592.6149	60.68512422	0.70249188	-0.081239622
5.959841175	31.63575012	0.001718915	96.29172149	186005.3105	61.84398845	0.866149464	-0.136249775
5.715155461	27.41524508	0.006580745	89.63858064	172914.0908	53.69564486	0.797027225	-0.112327208
6.061442036	20.01509291	0.00274925	157.7115441	506922.1503	38.87292558	0.980956537	-0.31364117
5.554681166	33.67518529	0.00530565	74.23018601	142115.8671	66.06709041	0.886729315	-0.165418293
5.819321672	27.09129634	0.006387889	133.7104284	335226.5924	52.65097538	0.957822506	-0.256700436
5.579233778	28.36302474	0.01086494	78.16323291	211620.1172	55.72052078	0.846864814	-0.139526632
5.568311107	29.81428685	0.007952356	95.6184696	245755.6362	58.58591152	0.872014902	-0.156661431
6.219144848	23.16996328	0.002195586	186.4165981	509571.0175	44.58481785	0.896877737	-0.163579624
5.43205486	33.33365087	0.015684953	118.010056	453517.659	65.63466662	0.853927025	-0.150983446
5.608236203	30.25522928	0.00772388	88.34891847	200950.1738	59.13043761	0.765652891	-0.097579942
5.928140139	30.45161627	0.004025786	102.6938647	249408.7439	59.7535069	0.80265026	-0.111272537
6.176807719	27.04494107	0.005207099	185.1224296	622440.1737	52.41202438	0.894272487	-0.162282551
4.901387433	12.60090328	0.01161027	94.38360503	122113.5093	23.88703921	0.992710979	-0.484434207
5.903390676	25.07745957	0.003782769	148.4226928	333523.6734	47.89668212	0.991380216	-0.393087985
6.12430106	23.60915043	0.002076542	141.2348132	324712.8032	45.75917698	0.852835867	-0.129174057
5.816814163	33.0330194	0.015179885	170.5722601	604869.9193	64.58453242	0.860642069	-0.147710671
5.959940029	27.88066966	0.004203989	110.7795341	287409.3208	54.65284485	0.819575213	-0.113886889
5.9574073	21.08753446	0.002087495	106.1977878	198770.4524	41.00479317	0.940108544	-0.213415562
5.974188181	24.24159021	0.007639659	155.4680237	431311.2366	47.00249175	0.929433276	-0.207497867
5.714697588	33.67822611	0.015303611	133.1271103	470867.9754	66.25088406	0.931079781	-0.218321274
6.014931849	22.10562444	0.002526934	130.0720713	257391.3665	42.58043421	0.922265752	-0.188646131
6.080972709	24.34457081	0.001845646	139.7013964	295594.05	47.01262973	0.869066555	-0.139524983
6.057636391	20.16542096	0.002286771	144.5167947	305520.624	38.6357031	0.921136735	-0.187204333
6.035849939	23.49573303	0.001373986	111.3728114	201106.8783	45.36197542	0.841946792	-0.119381779
5.45009037	26.90968954	0.007667876	75.09414275	133171.6301	52.30788765	0.80265434	-0.114586108
6.162021774	31.3892526	0.001911468	131.8979846	364813.042	61.17186076	0.812540535	-0.10460829
5.980622295	24.88696509	0.003181702	135.4160563	284693.7122	48.04906133	0.967548083	-0.270366915
5.186375356	13.34352124	0.008280285	104.5144107	191902.2922	25.39092502	0.993166005	-0.4679673339
5.898039076	20.97958196	0.00313501	132.3125292	304146.2378	40.4839159	0.96962501	-0.279443945
5.907335374	25.78036603	0.004308312	115.1086365	251538.7656	50.38127408	0.791386461	-0.104499186
5.992375835	22.81807096	0.004196279	165.0541066	371583.0501	43.88453719	0.9326096	-0.210007541
5.903241594	27.09006959	0.007996909	146.3084949	450775.5423	52.65709054	0.827441852	-0.125032468
5.773206836	24.87846606	0.003629296	88.81728034	155089.1859	48.84882033	0.951709274	-0.240490684
5.342415178	31.69870318	0.020502085	127.5962741	533869.9517	62.5394171	0.814221054	-0.131302413
6.290924777	26.15695845	0.002529959	211.062077	724741.9959	50.23433595	0.963856421	-0.248472952
6.24133778	27.329875	0.001453617	168.1200921	418065.87	52.6682502	0.877656096	-0.14126916
5.994979492	17.37723761	0.001962263	137.73892	365780.9411	33.09982553	0.909762956	-0.170171446
5.953703735	30.01622645	0.004231631	147.9070188	424677.3276	58.62232097	0.96579102	-0.266683173
5.749287443	27.85218408	0.003020725	78.97292432	202142.927	54.53881842	0.930613325	-0.204498268
6.016170555	27.56185482	0.002899321	132.1156744	283251.9769	53.49653691	0.84417563	-0.12488317
5.980643238	28.99527481	0.009215595	169.6270858	487251.7997	56.38167989	0.800564823	-0.10975336
5.708741761	28.34633032	0.006271856	87.44408328	195079.8699	55.47064638	0.852930259	-0.141782255
5.455703061	29.17205863	0.02023252	102.4890922	422000.3144	57.32577493	0.891467916	-0.189865854
5.640832872	24.00077223	0.006847644	118.3571287	249488.6645	46.86657111	0.991380485	-0.41943094
6.103476677	28.08373822	0.003659866	181.0412586	566675.9214	55.34772125	0.933894686	-0.201345399
5.84904509	23.70630618	0.003034298	124.6075022	233368.426	45.62394083	0.983596562	-0.332641653
6.004554235	28.20577119	0.005560459	161.9285595	441984.6984	55.06669734	0.809753195	-0.112797572
5.899390913	21.08232786	0.003457702	130.7846428	247320.2558	40.50960902	0.974276942	-0.296088858
5.995676687	28.48798892	0.004373495	150.6058727	368999.4501	55.45373256	0.862767304	-0.139618987
6.068867761	29.36140299	0.002677611	136.2327305	367337.2737	57.12912643	0.847307539	-0.127516338
6.026158031	25.06626671	0.002770749	134.0870505	287698.2057	48.70813044	0.913954646	-0.178816781
5.684686698	28.72626919	0.012435346	111.7764561	285284.8313	56.07586402	0.778318004	-0.106142085
5.56943186	30.26371931	0.01059137	102.682564	321561.4001	59.52990906	0.752841485	-0.096456967
6.04650569	34.47249873	0.004807496	143.1925371	627633.0982	68.15992691	0.853778	-0.131661979
6.221390517	25.38217059	0.0015258	169.4244722	411948.5546	48.79887138	0.940331382	-0.201417981
6.038485852	23.51273174	0.001849338	126.4567418	247459.823	45.43158625	0.911173443	-0.17290265
5.838971462	25.41164466	0.003681721	117.5140624	209115.6722	49.20730712	0.949369135	-0.233226261
6.093653999	27.84424111	0.002592577	144.6396878	370464.2751	54.14714374	0.888298398	-0.154441061
5.850526438	28.31944557	0.007253142	127.8428692	273958.0247	55.16054906	0.793913405	-0.108545961
6.302732341	31.5492848	0.002374821	199.6794604	725468.8739	60.54768221	0.991833054	-0.37248222
6.273770963	26.78805454	0.0013205	179.1014104	468242.414	51.28814675	0.934638145	-0.189510451
6.277909861	28.73520466	0.003019336	227.2529288	878051.158	55.7442503	0.896167877	-0.157250988
5.169194804	14.70797549	0.007743985	92.36155494	136744.2417	28.27321171	0.992358228	-0.450235777
5.832959782	25.67284938	0.003746485	96.08029938	187380.284	49.99173831	0.773128269	-0.098198752
5.80021105	31.5902318	0.006843729	101.1598758	276240.3866	62.22019427	0.84560364	-0.134326489
5.884801049	26.35423189	0.003926667	117.0334081	220329.1761	51.19952435	0.94085249	-0.218306944
6.206090725	24.24706538	0.00157969	166.5254976	430674.3835	46.57100704	0.896473818	-0.15584338
4.986805203	23.79446354	0.016116284	133.1018593	354706.3472	46.80937649	0.977489948	-0.389402602
4.87842745	15.17198394	0.012316225	103.0384456	112063.2525	28.49396871	0.995817735	-0.539120781
6.068027402	28.67790421	0.005721173	160.3321545	574878.2076	55.78696602	0.8693062	-0.145240141
6.084093234	35.78962829	0.003714599	165.7312555	560784.5594	69.35345222	0.976459398	-0.295903242
5.859793521	27.9862296	0.006085638	135.7992404	385701.235	54.38302826	0.94063704	-0.222861952
6.000109929	31.86762251	0.006608945	119.5775003	357931.8384	62.47266103	0.847343307	-0.132889844
5.619754131	31.31494218	0.007707145	92.64248966	234100.6873	61.56661786	0.760638014	-0.096736915
6.018731157	30.07519955	0.004332334	223.685113	743862.7269	58.1221767	0.996762596	-0.476738888
6.150749819	22.18410129	0.002362019	171.1570541	867942.7471	42.96471705	0.946491432	-0.217780401
5.304195514	17.14764849	0.008438563	123.6816201	216659.9847	33.14807526	0.996466197	-0.52233261
5.457616086	13.75380306	0.004016202	58.85252805	58378.85315	26.93236475	0.954008519	-0.255634516
5.620750622	37.17628478	0.005514723	108.7872175	412145.2882	73.37309392	0.943988859	-0.234531805

original_glcm_DifferenceAverage	original_glcm_Id	original_glcm_ClusterTendency	original_firstorder_InterquartileRange	original_firstorder_Skewness	original_firstorder_Uniformity	original_firstorder_MeanAbsoluteDeviation
9.731762982	0.26493643	427.6670081	646.25	-0.250933633	0.059076792	290.6760748
6.997604084	0.346384008	416.3504437	469	-0.946755781	0.074975504	262.2572907
9.102404058	0.239036645	321.7784289	479.25	-0.58242919	0.040667672	243.2868398
10.4107369	0.227853214	574.6568148	722.5	-0.12373555	0.039984733	327.0295881
8.337680707	0.205137601	257.6725044	334.5	0.736232382	0.037111754	193.4545356
8.772968467	0.201274068	285.127338	456.5	0.136164694	0.029732809	224.1757944
6.125057517	0.273072985	96.84768274	164	1.371704361	0.070521005	111.610281
11.09073567	0.206776942	450.2239657	679	0.166559693	0.035661213	298.0227708
8.872999852	0.22823698	388.4241671	566.5	-0.245515532	0.033154329	266.3954699
7.531373059	0.287730756	299.1433299	390.75	-0.726361087	0.060051238	220.4318203
9.350610051	0.20896705	304.7943692	475	0.3519083	0.03474191	232.8791
8.379335568	0.271394975	406.9801293	473.5	-0.362463377	0.051505151	258.6648855
7.615048507	0.225756137	273.7195253	433.75	0.129655814	0.03021056	214.8215138
7.971404907	0.214941589	283.7300252	455	0.014390202	0.02993622	221.8771829
8.608984528	0.200532909	282.7997334	421.5	0.259654457	0.029028064	222.0013664
4.836233757	0.435270127	358.9236587	165	-1.738677761	0.129665439	190.4709685
9.240480248	0.196423034	336.6152644	501.75	0.329068412	0.029884812	239.77868
8.503440798	0.2582371	432.7296336	623	-0.337480326	0.043294281	284.8067251
9.325525578	0.201032655	334.2340002	450.25	0.463770059	0.031441528	232.6781805
6.35243532	0.361131627	332.6237142	453	-0.80227672	0.094954995	235.3029477
6.698601834	0.334842058	229.4541128	363.25	-0.958456548	0.100326066	200.2977419
8.442340669	0.207764074	279.7895047	443	-0.220502741	0.0305841	217.5767523
7.536277526	0.27541118	269.5344934	438	-0.606016209	0.055573202	220.4144954
9.61207985	0.210062856	464.9745107	512	0.627259454	0.032939865	271.3077226
7.274773235	0.27244111	224.1345228	355.25	-0.920034155	0.054818655	197.3478328
9.243875205	0.263918229	395.5441372	596.5	-0.520892185	0.049514571	279.5140064
5.875739132	0.335120992	255.7588355	280	-0.809516624	0.080510234	187.9469761
7.840850512	0.285961127	278.8920666	385	-0.96811916	0.063560603	222.7402893
11.19061062	0.215015205	524.0010758	709	0.243235094	0.034688024	319.8678747
7.045987435	0.352145759	384.8397296	382	-1.158117738	0.089688512	246.6071144
7.736628331	0.282957864	264.2895189	398	-0.892829791	0.068534751	215.82688
6.921027623	0.274390821	335.2868925	478	-0.573492017	0.04667899	236.2101215
9.706006563	0.256558734	576.4882303	717	-0.160219846	0.050736628	325.3031541
9.792605815	0.207080307	196.4159935	247.25	1.386935406	0.070821006	161.977071
11.14468572	0.188562636	401.5358134	548.75	0.31763828	0.031481347	268.9469988
9.32799308	0.220611415	415.1460504	615.25	0.174179779	0.033889325	274.0748554
9.282838635	0.307979571	528.3894546	619.25	-0.686149234	0.089819155	309.9682369
7.633020137	0.261558675	347.161603	484	-0.361152918	0.046506116	242.4481766
8.252998885	0.217573797	308.815696	449	0.408369775	0.031345692	224.8421241
9.519671626	0.258940592	471.7677671	654	-0.146122117	0.059528299	304.2397362
7.123103938	0.329245464	443.8287425	508.75	-0.746373389	0.075677183	270.3683844
9.558927245	0.209155163	366.7673892	547	0.338625725	0.035116366	256.7647249
10.05051076	0.208886215	389.5232252	590.25	0.292592659	0.033230174	272.4830181
10.16590448	0.213415862	400.2853428	606	0.327775799	0.03505818	272.9519838
9.24481433	0.193175197	311.1524666	485	0.289442147	0.028987061	238.0029125
6.956172836	0.295004628	227.5620908	403.25	-0.738218792	0.062404714	207.9554261
8.787163446	0.2309523	402.6761643	554	-0.098505613	0.032419688	264.3214831
10.01314959	0.209898993	385.2195571	585	0.107212379	0.034055236	268.691667
9.140151057	0.216211807	247.5171132	206.25	1.463507148	0.057473825	165.6089329
9.734452575	0.208180675	359.9605633	417	0.770054066	0.040536984	237.5212725
7.9318212	0.265097849	358.7141587	532.25	-0.43738966	0.047609405	255.6834817
10.65547155	0.227832937	461.1324397	713.5	0.169064394	0.045576368	306.3592507
8.400228707	0.293087138	459.5562092	599	-0.437621972	0.066723854	290.2968594
7.316792222	0.247474155	271.5407574	444.5	-0.23871613	0.036941603	215.5352281
6.378680489	0.389518064	424.4621499	261.5	-1.382794912	0.122012091	231.3953939
11.54340025	0.206711881	622.9678322	737.5	-0.031370396	0.030087712	334.1369303
11.0399962	0.196257823	470.7805506	671	0.145244884	0.028632121	300.3792754
9.893353978	0.203969397	355.6638773	360	1.065120535	0.0388821	225.1143375
8.456465784	0.260645675	474.7905706	624	-0.341233081	0.03859969	295.3490392
7.087187201	0.235373524	240.3169053	378	0.050737336	0.03663544	196.2501311
9.628798284	0.228554587	384.7767382	603	-0.214343623	0.039186791	277.9224019
8.957173587	0.287032666	536.3466614	686	-0.297014204	0.069484416	314.0237673
6.491053047	0.304692176	286.8589418	367	-0.824508516	0.06523811	209.0320558
5.870688003	0.401188589	339.6937327	138	-0.971188565	0.108256895	198.9208978
8.607349919	0.243333019	349.95995	561	-0.17124809	0.039011234	261.1349147
8.775676264	0.261398566	584.0723526	670	-0.357394026	0.043016259	321.0413362
10.52262711	0.187797031	317.703981	431.75	0.563519358	0.033986962	230.4189492
9.421339198	0.259283588	493.7789127	676	-0.301853443	0.054268796	307.7442144
9.888289068	0.20182603	363.9769761	546	0.303175952	0.035019807	258.5849504
9.601939854	0.250820158	449.2345381	645	-0.376368566	0.052812218	294.7048627
8.95685222	0.240356097	420.6935119	555.25	-0.55050523	0.039821243	274.5562574
9.399348626	0.228000268	386.4406019	614	-0.021122265	0.035069482	273.6612599
7.451505967	0.322351268	354.3594932	472	-0.787136585	0.085564506	248.0926073
6.909438061	0.326264371	331.2747198	364	-1.113259391	0.082594311	225.2766593
6.672115401	0.299702454	490.6926426	400.25	-0.82880063	0.05234098	252.4994412
11.18868667	0.185209576	467.9624483	626.25	0.27142808	0.027390771	295.2518114
9.439042599	0.206865503	361.4108629	565	0.049429584	0.030141325	262.9897579
9.612713491	0.218844009	321.430718	562.5	-0.145138344	0.037678044	252.688014
9.886636879	0.21940364	427.2216108	632	-0.264431326	0.034296409	286.5928445
8.785950875	0.279822833	382.0511841	595	-0.354272347	0.061028066	272.8400645
11.92948324	0.175136164	596.418495	719.75	-0.001809339	0.023833144	336.9827176
11.82382264	0.173825141	486.3459321	623	0.274637115	0.025250225	297.3881957
10.52262981	0.234620921	717.8212169	759.25	-0.350169311	0.045389354	359.7016381
8.436801942	0.230314161	224.9858155	169	1.247565262	0.056551724	163.4138407
7.942075927	0.256784629	282.0216456	471	-0.493881694	0.047652724	229.8567655
6.176729109	0.31244769	339.4057757	419	-0.595696152	0.058083657	226.8039029
9.223050242	0.231053874	333.9212267	569.75	-0.132619769	0.040232354	253.8047925
10.76338225	0.194467205	473.9087286	609.5	0.437042343	0.031150361	287.3428267
6.959756978	0.339717362	421.7398337	610	-0.3035797	0.076592314	286.809639
10.47077236	0.20110795	211.7175427	336	1.021367348	0.069569161	194.4903401
8.547733098	0.285927652	505.2311115	574.5	-0.432147932	0.056262268	288.9679302
10.78137816	0.22097443	485.5766261	607.5	-0.478081126	0.038291777	309.4512114
7.891144509	0.287181863	439.8456869	581	-0.525583863	0.049124735	278.0927769
7.683344188	0.288546204	380.0506708	487.5	-0.49888389	0.059404116	253.9754444
6.769065091	0.294969391	294.2841643	395.75	-0.74376778	0.063793925	222.141432
12.36039551	0.201045108	644.6094614	758	-0.0042535	0.029011545	356.0236529
8.609153104	0.236083793	544.3683513	633	0.727717538	0.033932165	296.5435409
10.24512001	0.201558925	314.0504131	401.5	0.726917058	0.046995836	232.8784454
6.443708072	0.247132732	158.7199537	288	0.660711789	0.045300437	155.1775468
7.824231682	0.291566375	324.5296303	312.5	-1.263128331	0.049945566	232.9842318

original_firstorder_Energy	original_firstorder_RobustMeanAbsoluteDeviation	original_firstorder_Median	original_firstorder_TotalEnergy	original_firstorder_Maximum	original_firstorder_RootMeanSquared
141563586	255.3899786	-273	3822216822	67	447.7890065
283494146	204.1223408	-54	7654341942	371	369.448408
62558705	196.543153	-169.5	1689085035	82	379.6636427
670240668	284.9501878	-323	18096498036	915	504.3419324
107707820	140.8316478	-558	2908111140	91	547.742442
137342084	181.5541116	-401	3708236268	69	462.1642306
156064590	72.17379528	-713	4213743930	-45	680.5142714
278598917	260.6737708	-455	7522170759	90	527.2976686
388648815	224.2751216	-253	10493518005	638	432.3658948
325393198	163.3801823	-81	8785616346	849	324.0884311
196353857	189.2470534	-452	5301554139	65	487.2669592
195034836	199.1353152	-72	5265940572	945	362.7705245
396329681	172.8607817	-386	10700901387	80	437.143397
203959111	181.3245274	-355	5506895997	106	427.1207332
233689845	173.8384312	-482	6309625815	188	506.4781705
514262102	106.3868104	22	13885076754	356	272.0781155
178597242	194.6405557	-480	4822125534	108	505.8359163
417678602	247.2639818	-190.5	11277322254	334	423.2108957
165525694	182.907274	-515	4469193738	94	533.299549
300714600	189.9478803	-44	8119294200	163	327.6579446
418487877	154.7753593	-45	11299172679	95	283.034813
270630678	173.5675987	-294	7307028306	165	396.6653721
312885343	179.2104768	-153	8447904261	142	338.4790236
206031196	213.3775672	-671	5562842292	58	654.476344
99668830	149.3648522	-70.5	2691058410	131	287.7176051
112050038	236.5993444	-133.5	3025351026	188	401.8151144
233280650	128.6091879	-8	6298577550	795	259.4329172
155614634	168.0239637	-82	4201595118	112	329.5354361
613906933	279.3315681	-557	16575487191	85	589.7648895
299373559	179.6669154	-15.5	8083086093	113	343.1776114
303209607	167.6301484	-69	8186659389	101	308.8347882
766415542	192.68217	-160	20693219634	278	359.5048923
463359831	285.4965113	-245	12510715437	765	477.4088896
44879597	106.1811422	-701.5	1211749119	29	656.9129216
99699769	220.0339761	-534	2691893763	123	547.9968148
538077967	233.3100007	-450	14528105109	81	516.1155212
534444255	258.8824303	-48	14429994885	470	430.7794353
441223608	196.2815344	-162	11913037416	944	362.7002389
225974555	177.7087363	-505	6101312985	77	520.219386
208305520	264.889406	-231	5624249040	730	445.617918
198014156	211.1223184	-23	5346382212	514	366.5214346
248034282	214.6890095	-494	6696925614	80	510.1635254
452000138	232.0047835	-495	12204003726	101	524.6657413
296041179	232.9322282	-510	7993111833	286	537.4203778
495370900	193.48418	-477.5	13375014300	130	513.5910553
177280204	167.5621521	-42	4786565508	180	282.0801515
652965068	217.9458306	-219	17630056836	1030	391.2321611
136616024	227.4006428	-417	3688632648	87	484.0793352
60841801	100.2680669	-711	1642728627	34	673.8273451
173382144	182.1883033	-628	4681317888	109	590.6415432
508058977	217.3740594	-172	13717592379	233	385.2034665
230466233	274.5767982	-480	6222588291	84	527.8990699
612498469	248.5412346	-68	16537458663	953	400.3722649
79985993	175.8228392	-257	2159621811	325	367.8859737
644845087	153.7022516	20	17410817349	406	314.415544
298889226	287.5726749	-456	8070009102	119	583.1236997
575060934	257.1378208	-438	15526645218	278	512.0797673
673873588	157.876265	-767	18194586876	138	721.9214651
129749089	252.8616874	-153.5	3503225403	401	399.2454323
84196012	148.6692822	-173	2273292324	965	309.1418535
346723845	240.5642639	-231	9361543815	204	404.5073122
1184574131	278.8495447	-242	31983501537	351	465.0183685
233817201	160.2210316	-3.5	6313064427	757	275.0799831
231006656	116.5690621	-14	6237179712	1345	290.8381655
37497095	222.8161315	-249	1012421565	249	415.6893946
360473801	279.4053975	-210	9732792627	180	481.7826156
124870597	177.1438246	-565.5	3371506119	89	562.965762
621149417	271.7047705	-201	16771034259	176	440.7849146
95422016	219.4315687	-449.5	2576394432	201	462.5480608
378976676	257.6390901	-192	10232370252	402	434.1104155
410277794	227.7733855	-73	11077500438	502	367.368753
217212193	237.3967353	-334	5864729211	134	438.6267654
631835252	200.4816657	-53	17059551804	491	349.6557495
662693690	165.7722251	-12	17892729630	225	314.1239674
1000299269	180.1172789	13	27008080263	864	330.098564
314714397	248.9222888	-482	8497288719	233	531.0387442
258448519	224.257541	-394	6978110013	116	481.0170133
108054689	216.4269105	-291	2917476603	85	420.8786457
385672324	243.347733	-293	10413152748	138	467.5843286
479665514	239.164259	-208	12950968878	91	410.5365231
121350591	281.2256875	-319.5	3276465957	492	490.6882739
389055718	247.1859037	-487	10504504386	388	538.6312077
683631473	314.9961298	-173.5	18458049771	1311	494.8269187
48151165	102.2697921	-607	1300081455	34	576.2612255
352806661	189.9887081	-126	9525779847	256	322.5555308
560682818	177.7369178	-60	15138436086	988	319.9829987
124485411	216.3192171	-281.5	3361106097	136	400.0086937
488477204	239.2564685	-551.5	13188884508	143	561.0173495
35242681	250.1598098	-116	951552387	655	438.8427466
35715248	141.8763246	-653	964311696	80	583.2197005
613758640	234.2476968	-48	16571483280	1147	392.943621
117364146	253.64497	-64.5	3168831942	658	407.7231501
160279857	235.3605699	-107	4327556139	350	396.0171785
532521678	202.7753019	-85	14378085306	710	360.8336172
524172216	172.3687032	-27	14152649832	772	300.4681776
64588334	308.3961783	-302	1743885018	288	505.2621673
364111437	243.8091235	-544.5	9831008799	866	547.2053979
34294311	181.96793	-581	925946397	91	528.0298633
130603259	116.2638504	-589	3526287993	-31	587.026088
75703893	151.8702132	26	2044005111	249	306.28261

original_firstorder_90Percentile	original_firstorder_Minimum	original_firstorder_Entropy	original_firstorder_StandardDeviation	original_firstorder_Range	original_firstorder_Variance	original_firstorder_10Percentile
36	-1010	4.783281649	317.5808973	1077	100857.6263	-761
39	-909	4.528934022	302.8763932	1280	91734.10955	-744.4
45	-960	4.983216645	279.8724511	1042	78328.58889	-696.7
44	-1006	5.186235762	359.0694001	1921	128930.8341	-838.6
-141.8	-828	4.952250685	234.4845288	919	54982.99423	-766.4
-8.2	-858	5.130034376	256.5204915	927	65802.76256	-720.8
-452	-880	4.197924972	144.6581409	835	20925.97774	-799.4
39	-898	5.049901371	328.1875119	988	107707.043	-807.9
30	-992	5.157049995	299.3308675	1630	89598.96824	-742
43	-955	4.790231854	265.9046511	1804	70705.28346	-612.6
20	-859	5.012013588	269.9023781	924	72847.2937	-725
58	-977	5.020458001	306.2237595	1922	93772.99089	-659.9
-6	-844	5.11067979	247.1574732	924	61086.81658	-676
5.3	-837	5.12323724	252.9057397	943	63961.31315	-682.3
-57	-954	5.243027724	257.9422379	1142	66534.19812	-756
61	-967	3.927356843	252.2867804	1323	63648.61955	-582.4
-8.7	-883	5.160668936	275.5604683	991	75933.57169	-755.2
49	-914	4.991327009	312.175695	1248	97453.66456	-733
-17.4	-872	5.123488417	271.1092283	966	73500.2137	-780
46	-895	4.435018937	267.4681289	1058	71539.19996	-623
44	-882	4.313616057	233.9121718	977	54714.90412	-536
20	-1038	5.169782438	252.5427793	1203	63777.85535	-648.1
35	-868	4.744237175	252.0225848	1010	63515.38327	-611
-25	-1015	5.161417514	316.142299	1073	99945.95319	-912
50	-956	4.692419135	233.0129736	1087	54295.04587	-547.4
55	-868	4.908602341	310.461409	1056	96386.28647	-735
56	-784	4.533362827	233.5320556	1579	54537.22099	-511.5
38	-881	4.656590772	263.7708976	993	69575.08644	-648
26.6	-984	5.135421646	351.1787592	1069	123326.5209	-879
51	-971	4.399972035	293.9514691	1084	86407.46617	-715.9
52	-876	4.599225951	252.4315454	977	63721.68513	-584.2
44	-965	4.933647071	269.6770682	1243	72725.72114	-654
48	-929	4.993701524	355.6745878	1694	126504.4124	-811
-328	-829	4.235864654	205.7633732	858	42338.56574	-798.7
15.9	-987	5.189445364	306.5694213	1110	93984.81009	-813.7
27	-942	5.11122507	309.1271079	1023	95559.56881	-801
44	-1032	4.603805498	346.0228557	1502	119731.8167	-806.1
50	-897	4.971509204	279.5521546	1841	78149.40713	-656
-46.6	-909	5.141117946	260.6562863	986	67941.69959	-748.2
41	-849	4.831759191	333.3882413	1579	111147.7194	-755.2
49	-996	4.738481109	310.5476732	1510	96439.85732	-707.4
23.8	-895	5.040392263	289.9717005	975	84083.58709	-756
19.9	-959	5.100414802	303.9371534	1060	92377.7932	-780.9
11	-873	5.046031465	305.2959418	1159	93205.61208	-793
-32.7	-975	5.212440233	271.537661	1105	73732.70132	-763
69	-764	4.583915008	236.4216961	944	55895.21837	-536.3
85	-991	5.256393026	302.0358049	2021	91225.62743	-675.5
34.6	-910	5.09988439	300.5128884	997	90307.9961	-750.8
-307.9	-868	4.423690054	218.7815513	902	47865.36718	-832.7
-4	-969	4.960085522	282.3766282	1078	79736.56014	-797.8
43	-853	4.861644394	284.4739956	1086	80925.45417	-689
27	-876	4.858630772	331.5079789	960	109897.54	-801
50	-872	4.723005845	320.9102446	1825	102983.3851	-726
27	-838	4.956690218	246.9507517	1163	60984.67377	-630
62	-906	4.046493587	285.0878063	1312	81275.05731	-685
40	-1025	5.30959151	373.8492386	1144	139763.2532	-967.2
55.8	-998	5.337040868	333.3412798	1276	111116.4088	-793
-185.6	-1017	5.04395885	277.6194712	1155	77072.57078	-932
116	-912	5.098665214	324.8961734	1313	105557.5235	-702.7
56	-838	5.01580465	239.1723082	1803	57203.39302	-518
84	-893	5.048373975	304.9292443	1097	92981.84405	-680.2
41	-1004	4.731663287	340.3010582	1355	115804.8102	-792
84	-824	4.606342551	244.5955573	1581	59826.98664	-531
37	-806	4.233140963	264.6571644	2151	70043.41469	-635
33.4	-813	4.962853847	288.6034013	1062	83291.92325	-695.8
36	-1007	5.048716557	348.721715	1187	121606.8345	-831
-52.1	-995	5.117764057	271.6963043	1084	73818.88176	-780
71	-931	4.855697484	334.7137006	1107	112033.2614	-767
67	-786	5.025861652	289.8361735	987	84005.00744	-705
44	-968	4.847429269	322.7502815	1370	104167.7442	-761
106	-893	5.081913117	307.4555409	1395	94528.9096	-675
66	-860	5.078813169	302.5524242	994	91537.96941	-717
40	-895	4.482001472	281.9503083	1386	79495.97632	-677.3
53	-880	4.447274381	268.127251	1105	71892.22274	-638
115.1	-959	5.01152653	305.9877546	1823	93628.50593	-649
55.5	-962	5.344319708	330.0439372	1195	108929.0005	-804.5
10.4	-900	5.162796683	291.1704745	1016	84780.24523	-756
30	-872	5.004973238	281.3330454	957	79148.28242	-708
35	-959	5.170456911	320.7468525	1097	102878.5434	-804.7
33	-947	4.701983989	298.6597723	1038	89197.65959	-713
138.7	-977	5.568985609	377.1868522	1469	142269.9215	-813.7
62	-1008	5.450254852	335.5843875	1396	112616.8811	-815
95	-1023	5.080102316	392.3518605	2334	153939.9824	-877
-119.2	-811	4.485715816	213.1979797	845	45453.37855	-725.2
82	-793	4.863148496	259.1193627	1049	67142.84414	-576
52	-931	4.807775834	263.8002276	1919	69590.5601	-599.5
62.3	-858	4.993842973	283.1331662	994	80164.38983	-662.6
45.9	-959	5.207484275	324.4756401	1102	105284.441	-814.9
-42.2	-826	4.181882026	312.195979	1481	97466.3293	-745.6
-170.8	-804	4.362137553	229.8451645	884	52828.79964	-743.8
53	-888	4.926363388	328.2168471	2035	107726.2987	-726
122	-1011	5.265706683	348.9928839	1669	121796.033	-739
55	-887	4.884741614	308.0081871	1237	94869.04331	-719.8
43	-971	4.898502431	292.277492	1681	85426.13232	-665
71	-883	4.679859587	258.7412046	1655	66947.01098	-579
170.6	-985	5.349329833	387.226951	1273	149944.7116	-798.6
20	-932	5.195735665	341.3718431	1798	116534.7353	-791.5
-0.6	-776	4.718732975	268.8191991	867	72263.76178	-724
-277.8	-828	4.676067612	188.0450163	797	35360.92815	-772
171	-958	4.865748035	291.2553085	1207	84829.65472	-642.8

original_firstorder_Kurtosis	original_firstorder_Mean	original_glrlm_ShortRunLowGrayLevelEmphasis	original_glrlm_GrayLevelVariance	original_glrlm_LowGrayLevelRunEmphasis	original_glrlm_GrayLevelNonUniformityNormalized
1.508034792	-315.6855524	0.004750789	157.1648797	0.004824853	0.046423506
2.340490511	-211.5609052	0.007717465	150.6686681	0.008116193	0.061480902
2.049119573	-256.5460829	0.005871415	124.5844009	0.005929682	0.037855711
1.617562099	-354.1609108	0.007135082	203.8234944	0.007314233	0.034897086
2.630373211	-495.0139276	0.023521758	87.03677509	0.024166477	0.036978404
1.754356915	-384.4385692	0.010304442	104.0093662	0.010467837	0.029615531
4.704954204	-664.9614243	0.025666388	34.31417511	0.026780813	0.068246262
1.455251499	-412.7175649	0.022970996	167.1537428	0.023904894	0.034179244
1.773662711	-311.995671	0.004776303	142.4862141	0.004876034	0.031917162
3.080965221	-185.278244	0.003011297	115.9535703	0.003084727	0.050391914
1.799449106	-405.6868198	0.011084999	112.923561	0.011375135	0.033770085
2.802338989	-194.4979757	0.00371891	154.4785214	0.00378904	0.04459857
1.79240286	-360.5655738	0.009996134	96.7654745	0.010294411	0.030055103
1.716372977	-344.1958855	0.008866368	101.1737187	0.00910048	0.029812724
1.987909239	-435.8737651	0.006576963	106.2459122	0.006726047	0.028884061
4.718659678	-101.8718871	0.002868744	109.8597292	0.00304502	0.107297958
1.790658482	-424.1891117	0.011242498	119.8284195	0.01150727	0.029802217
1.561267825	-285.7512864	0.006860339	154.6193977	0.007077845	0.038740456
1.983687588	-459.2474227	0.024652131	115.3360984	0.025591007	0.031338333
2.20539558	-189.2631203	0.004330287	116.7625286	0.004489451	0.065729018
2.678651405	-159.3543262	0.002659631	90.24100707	0.002748632	0.071183169
1.999262919	-305.8848837	0.00262272	101.4568953	0.002666204	0.030149106
2.063206821	-225.9483706	0.005282909	101.0059419	0.005452695	0.046759114
2.098729716	-573.0561331	0.017357556	156.5149539	0.018158783	0.032288427
2.707084108	-168.7790698	0.00260615	88.1381294	0.002646826	0.049862396
1.716950502	-255.0864553	0.0188737	153.2620298	0.019458025	0.042368737
3.382490343	-112.9965378	0.00420113	92.03621084	0.004339276	0.067258088
2.647368375	-197.5310537	0.006672768	113.244064	0.006849397	0.055122569
1.48221808	-473.8101983	0.0143674	192.0388432	0.014910446	0.033131782
2.875293739	-177.0971676	0.00489178	144.9785101	0.005050002	0.071067166
2.53089294	-177.9248191	0.003983894	103.5589226	0.004082471	0.058630576
2.021763416	-237.7352445	0.002765958	115.9644119	0.002852454	0.041250784
1.628975418	-318.4569602	0.009760301	200.5056354	0.010107559	0.042288637
4.162887999	-623.8557692	0.060385809	68.92118723	0.062766092	0.069011704
1.748676312	-454.2198795	0.010933661	146.5409162	0.011133309	0.030595532
1.58802852	-413.2985149	0.008970218	148.6827952	0.00930049	0.032143576
1.913095583	-256.5913194	0.004938924	195.2885713	0.005071537	0.066694623
2.327528067	-231.0888491	0.004956926	125.0816465	0.005095118	0.04095688
1.931515129	-450.2071856	0.011063802	108.2795988	0.01132199	0.031161971
1.677815812	-295.6816015	0.03320175	177.0632702	0.034362705	0.048593359
2.227667513	-194.6743555	0.004873788	160.7319881	0.00501585	0.057889206
1.669186924	-419.7418678	0.011078013	130.4700885	0.011398575	0.033697791
1.590494588	-427.6638246	0.006183258	144.7736443	0.006370415	0.03245574
1.605247058	-442.2839024	0.028632711	145.0787899	0.030046999	0.034116795
1.786641611	-435.9392971	0.005694017	117.5623672	0.005829215	0.028856935
2.116964874	-153.8635548	0.005441082	90.50441847	0.005559048	0.055037962
2.237955063	-248.6704173	0.0026882	145.6784366	0.002761294	0.030779292
1.57373218	-379.5060034	0.010157545	140.5611729	0.01038253	0.032427452
4.329138675	-637.3208955	0.096440672	76.45285295	0.099757217	0.057011444
2.317623124	-518.7686117	0.013306598	125.7042562	0.013642144	0.039782922
1.68781631	-259.7234229	0.006397266	128.4715384	0.006584821	0.04247101
1.377100129	-410.828295	0.019489006	170.9570782	0.020093532	0.041923612
1.839720178	-239.4046061	0.010506037	166.5027073	0.010886725	0.055992122
1.855720316	-272.6818951	0.006534113	96.65099063	0.006651462	0.035298762
3.368625465	-132.5974245	0.004838921	137.1734108	0.005060524	0.102735355
1.537902141	-447.5153584	0.03742446	215.7185253	0.040196581	0.027920737
1.545894141	-388.7277702	0.007016033	175.5181944	0.007237756	0.027955624
3.085348842	-666.4068059	0.026256742	124.2956517	0.027318145	0.038068303
1.598398077	-232.0331695	0.007031421	167.8915417	0.00721314	0.03520784
3.992715097	-195.8706016	0.00588403	92.06156423	0.006160563	0.035772331
1.522443598	-265.7899953	0.006083429	147.3426313	0.006211233	0.036249969
1.468887895	-316.9184009	0.004811392	182.0407535	0.005020752	0.053993723
2.604256818	-125.8650485	0.003833561	97.91893231	0.003935222	0.058447882
4.159902867	-120.5961186	0.007047132	123.5423779	0.007424191	0.084781859
1.581761639	-299.1751152	0.020022551	130.9678047	0.020386914	0.035985686
1.535971439	-332.4269156	0.00931232	193.8320438	0.009569504	0.039653885
2.140888133	-493.0634518	0.010912524	117.1134977	0.0112339	0.03372271
1.471564688	-286.8066938	0.005762958	177.2393416	0.005943616	0.046656382
1.632125898	-360.4798206	0.028885556	131.516102	0.029322655	0.034018718
1.544376155	-290.3172551	0.005731446	165.8310737	0.005892844	0.047223072
1.829211599	-201.0743421	0.005541232	151.4834861	0.00566542	0.037457963
1.499333184	-317.5775022	0.009077322	143.43507	0.009313987	0.033100968
2.134904628	-206.7925697	0.005588289	129.950063	0.005822512	0.067280365
2.853059315	-163.6509827	0.004743142	118.6197773	0.004894284	0.070508468
2.859957564	-123.8408497	0.002954035	154.4602934	0.003037888	0.04767998
1.624140851	-416.0206093	0.008153902	172.1718742	0.008366698	0.027086426
1.538011305	-382.8800358	0.012036277	134.447212	0.012300588	0.029826731
1.564912919	-313.0344262	0.008730146	124.4884506	0.008903154	0.035162692
1.61316511	-340.2301587	0.007787707	162.0301558	0.007996033	0.031989456
1.533281197	-281.6781448	0.004731353	140.6908297	0.004896843	0.050401511
1.642289553	-313.8551587	0.007978966	226.1052474	0.008076863	0.023241975
1.695976536	-421.3154362	0.00631153	178.9409705	0.006430153	0.025057674
1.615150934	-301.5189828	0.011206238	245.4806914	0.011490678	0.041720706
3.463510267	-535.3724138	0.035234283	73.78682053	0.03640471	0.055524467
1.892333217	-192.0917134	0.00950626	106.4678682	0.009929046	0.043194598
2.543767924	-181.1037253	0.002376836	113.5464198	0.002463116	0.050101964
1.592696447	-282.5642674	0.009567209	125.9036116	0.00975969	0.036668936
1.715857036	-457.6636598	0.008938445	165.7338247	0.009198934	0.030781802
1.875164986	-308.4098361	0.021508948	159.9933582	0.022084906	0.070539997
2.891905044	-536.0190476	0.035972463	85.97096498	0.036764004	0.066635719
2.223806224	-216.0518239	0.007919944	176.3296543	0.008170232	0.04887803
1.928334359	-210.8130312	0.00611814	197.3928952	0.006161705	0.034787632
1.738045761	-248.9187867	0.009176304	152.2958926	0.009404765	0.043210856
2.262607643	-211.600489	0.002818796	138.9458484	0.002925361	0.049575329
2.50161423	-152.755081	0.002546858	109.3345436	0.002623671	0.056463097
1.450599654	-324.56917	0.014522619	237.915997	0.01468903	0.0282268
2.923734369	-427.6669408	0.009782201	185.3819392	0.010102439	0.032709419
2.098327184	-454.4796748	0.043393544	113.9265051	0.044120954	0.046232355
2.5512171	-556.0923483	0.027710019	56.54903064	0.02923221	0.044471962
3.431185492	-94.75960347	0.004320047	140.7865388	0.004366322	0.045905716

original_glrlm_RunVariance	original_glrlm_GrayLevelNonUniformity	original_glrlm_LongRunEmphasis	original_glrlm_ShortRunHighGrayLevelEmphasis	original_glrlm_RunLengthNonUniformity	original_glrlm_ShortRunEmphasis
0.150051519	29.82961878	1.361166197	835.7943668	550.7647221	0.940014931
0.211392798	111.7660558	1.524274371	831.2898725	1450.845536	0.912219725
0.070768982	15.56802438	1.187364497	903.0743424	372.3983655	0.961879291
0.092808361	86.01125998	1.237862105	862.377779	2187.941318	0.954251496
0.035399352	12.8344791	1.105857085	296.6380863	324.8459329	0.974783924
0.036996712	18.38010853	1.110721029	485.5974101	578.8117256	0.973563619
0.064327822	21.73533331	1.187161329	132.8062228	285.8895083	0.958185146
0.06436906	32.5202684	1.175007565	522.9639633	865.1996445	0.963452502
0.067079417	62.77593407	1.185293142	868.7477561	1773.5508	0.960438234
0.132503041	142.3535408	1.338270752	1016.269248	2403.079252	0.937632833
0.058738472	26.56278329	1.165739105	448.035477	715.8432528	0.963760904
0.110456602	60.96316881	1.288748395	1104.004164	1181.865103	0.944133061
0.051565813	59.52158901	1.148804529	478.2733033	1813.186044	0.96635721
0.04237606	32.07412411	1.122580982	509.4937621	999.1334544	0.971875842
0.040052682	25.37833425	1.115473016	579.6659106	819.4675115	0.973485091
0.302582802	625.8990679	1.734199837	1127.927908	4335.37652	0.884842196
0.039847646	20.05940215	1.115967573	483.1771619	627.4700107	0.973228772
0.10341533	83.80641658	1.26897405	745.3962015	1891.656224	0.948211804
0.046181762	17.53248235	1.129354495	393.4569911	519.3432597	0.971411097
0.312188769	158.6473047	1.672290806	770.8908914	1913.523777	0.909671012
0.261481298	323.9811453	1.585558724	830.192754	3657.589699	0.915141648
0.047075129	49.84010325	1.130710653	972.866066	1535.063052	0.971536884
0.135939367	117.0999994	1.330581587	704.2510378	2156.116246	0.942185876
0.046461605	14.8615623	1.139647828	483.7547233	422.097941	0.966983993
0.091875762	56.0120893	1.24231339	1063.4337	991.9631062	0.952172372
0.109122916	27.19129073	1.282286396	701.7512439	557.7959863	0.946149969
0.171146654	207.5851335	1.435616835	766.6368749	2517.485912	0.921575249
0.118683385	72.71015905	1.304045361	828.44471	1137.685617	0.943074013
0.067454236	55.53621195	1.180364391	618.6877525	1524.852386	0.963347109
0.218489601	158.4845876	1.524492775	1004.62431	1789.885413	0.914797785
0.115329696	171.0769502	1.305943989	858.0481331	2499.728726	0.940565474
0.108419253	226.8024526	1.276290814	918.1211582	4807.708405	0.948188815
0.125303875	79.06654938	1.310782688	765.1424848	1618.413337	0.944240248
0.044088817	6.919540724	1.122786278	162.2846803	93.62524861	0.973316236
0.048764083	9.751664929	1.134616108	607.2327184	295.4624257	0.970966524
0.071270507	61.46565274	1.188937321	588.6557079	1731.023975	0.961677442
0.203284252	170.6058521	1.47950818	1027.918488	2099.600624	0.923474538
0.102570865	127.579999	1.264057604	783.8423122	2730.857308	0.949405683
0.046914165	24.92273149	1.138159769	473.787948	736.3047134	0.968159699
0.136789865	46.75600958	1.328499327	597.4699598	832.7381853	0.944144347
0.203986962	75.337729	1.492030672	1048.367822	1056.940337	0.919538206
0.060477301	30.59984387	1.163053477	481.6592369	830.6378495	0.96580523
0.054375207	50.90904479	1.151878207	614.1912675	1440.485621	0.967131945
0.058767444	33.24391179	1.166825576	435.9018153	885.7454026	0.963363468
0.036941337	52.37312267	1.107943408	591.4948098	1698.574097	0.974839285
0.108775764	112.9352354	1.290839733	653.350373	1768.030041	0.942576798
0.067650326	124.1770458	1.186629736	1009.261239	3637.146232	0.960376434
0.058888689	17.99781526	1.163349456	588.4011768	506.709216	0.96506632
0.036061136	7.363012568	1.113814421	173.3599764	119.9366524	0.971546395
0.046341088	18.92603131	1.138775129	458.2065205	436.6804598	0.967303012
0.096616621	135.2288479	1.25563543	682.071512	2789.663843	0.9492708
0.080938792	32.58694116	1.216521392	516.7337218	694.3320225	0.956379286
0.14605071	193.5085252	1.37116798	727.9508401	2899.79618	0.932314091
0.062470076	19.75977314	1.178329174	604.972871	505.1061512	0.960666318
0.21872251	585.2438463	1.541329873	1001.209267	4510.038535	0.909242734
0.070061798	23.21899969	1.18916216	723.1065906	751.3876207	0.960985883
0.04993077	58.62570302	1.145197268	762.2945284	1923.804468	0.966950155
0.046383115	47.13693391	1.137850572	343.0921927	1137.497464	0.967675841
0.086588162	26.78840943	1.232579649	871.5382433	673.1252887	0.952977874
0.053542202	30.10680181	1.15191152	765.0773726	772.154986	0.966603316
0.072945771	72.43380028	1.199084821	748.6071835	1792.427501	0.958375471
0.178876277	266.9256042	1.413245724	864.7893101	4167.417904	0.934019998
0.118869229	165.6518192	1.315465267	815.3069431	2422.886744	0.939126671
0.307032625	195.0289686	1.726442229	794.6051279	1734.950612	0.890355792
0.077561595	7.331656495	1.215250265	537.3368595	180.9287542	0.954385393
0.07986267	57.6629589	1.22230151	900.184415	1286.581175	0.952977054
0.03890033	12.80704825	1.115748963	533.6944407	353.3113193	0.972542893
0.110251657	138.1446845	1.278641807	805.308518	2586.410913	0.947997116
0.04830454	14.53692143	1.138018782	424.1405222	394.0828124	0.969098692
0.090428081	88.54031544	1.242880273	853.3168531	1649.301314	0.950868846
0.070255713	107.4874344	1.194675106	891.1797805	2577.333491	0.958696011
0.068288283	35.31587797	1.189816387	610.1591376	960.2166821	0.95953536
0.218946993	307.4584826	1.509804286	774.0355443	3717.439277	0.920080909
0.150767695	426.0889228	1.389365015	885.6262616	5013.740261	0.928028987
0.102052245	404.019305	1.277404722	1236.983178	7317.007602	0.943739538
0.039223159	29.13841413	1.116171199	672.1804387	1001.461141	0.972722485
0.044566254	32.04217343	1.126329953	555.2339796	997.7185766	0.971761902
0.059223914	20.36727597	1.168925419	599.4944675	524.9098066	0.96252907
0.068548923	53.43924292	1.185603124	768.9954164	1512.256481	0.961646222
0.144477482	130.1324272	1.364240901	758.7675871	2180.794588	0.93478103
0.038183928	11.32676221	1.108284565	958.0431237	457.0929577	0.975606367
0.03081866	32.61478602	1.092727368	764.9587974	1226.957809	0.977612325
0.070507747	109.9946877	1.193334981	1028.277277	2371.046556	0.95934005
0.042982751	7.731757944	1.128454581	219.0295429	128.6701196	0.969722375
0.093718371	136.5128541	1.248166571	655.0775278	2782.294628	0.950836973
0.136706116	248.7729624	1.356208263	969.7432323	4169.114432	0.932785967
0.079544477	26.83049513	1.211905536	643.1426496	654.8879266	0.957428037
0.046072772	45.8401071	1.133109113	589.6790153	1375.768405	0.969715863
0.119459291	11.72982641	1.343871293	571.1566029	138.8618483	0.928766076
0.036786787	6.773389744	1.105395444	234.7609329	95.5373319	0.976215749
0.129040878	177.6558761	1.32866111	854.2826922	3105.249203	0.939252058
0.067738095	23.26818426	1.18389503	1192.107861	605.7703209	0.961996338
0.120521823	40.44314897	1.316177368	749.2778772	800.0104696	0.939629911
0.143919186	184.2344155	1.360796255	975.0760565	3147.27801	0.93573173
0.11118077	301.6143827	1.294732555	930.7551916	4596.587491	0.942270097
0.045417858	6.841426038	1.136827997	945.8272692	222.668951	0.967624731
0.071571121	37.54784861	1.194765249	606.1534141	1030.987466	0.958930605
0.034395468	5.499383468	1.104888711	311.5246709	111.2425574	0.974508336
0.056733456	16.03658649	1.162687202	205.9858693	327.8024641	0.963543832
0.103860856	34.21128724	1.279915773	1282.795136	643.6807476	0.943753683

original_glrlm_LongRunHighGrayLevelEmphasis	original_glrlm_RunPercentage	original_glrlm_LongRunLowGrayLevelEmphasis	original_glrlm_RunEntropy	original_glrlm_HighGrayLevelRunEmphasis
1528.368499	0.909239486	0.005206348	5.261324571	931.1848517
1642.717251	0.874189845	0.010076617	5.208744995	944.3240208
1229.856929	0.947004608	0.006183936	5.249237346	956.8703652
1266.399941	0.934929207	0.008104662	5.56336089	924.1385669
335.3227867	0.966788087	0.026897751	5.11072054	304.2864476
582.0939154	0.965187223	0.011135722	5.303536878	504.0694509
149.6490757	0.94476147	0.031806252	4.487508872	135.9389463
720.1711284	0.949332105	0.027787581	5.313646352	554.8364688
1139.787639	0.945942946	0.005311078	5.462183623	914.8522435
1623.500368	0.911282713	0.003438229	5.300204656	1108.59131
602.2064019	0.950981304	0.012636183	5.258556787	474.8014923
1652.373319	0.9217793	0.004107502	5.471277188	1191.018679
587.4373553	0.954862399	0.011552682	5.363029359	497.9567189
608.6210772	0.962295308	0.01010696	5.327881675	527.7765236
661.3632351	0.964451575	0.00738437	5.429187004	595.068265
2499.012396	0.838048521	0.003967404	4.824396878	1310.188008
572.5512432	0.964293586	0.012639927	5.3410931	499.9142955
1141.255301	0.927035229	0.008043149	5.394300304	807.0090755
474.4321537	0.961274121	0.029945293	5.303711185	407.905094
1802.419257	0.85911625	0.005403338	5.211887953	888.3332898
1746.282979	0.869949346	0.003280188	5.050406286	942.7621132
1178.294101	0.961046512	0.002861591	5.38683975	1008.971425
1162.729714	0.915866265	0.006253591	5.210854035	769.8128045
583.5452094	0.956820726	0.021485506	5.373289896	502.6067157
1504.292068	0.932724252	0.00284364	5.072833117	1135.107408
1100.151134	0.923963644	0.021858814	5.310971517	763.3655311
1313.146675	0.889919659	0.005010257	5.165522862	848.5631995
1294.032871	0.91953406	0.007695575	5.110776257	900.304119
818.0336982	0.949052081	0.017493741	5.414459119	650.3095534
1968.543463	0.87623313	0.005847348	5.096912474	1136.68122
1340.331138	0.917100201	0.004535643	5.085207063	935.2020254
1377.378477	0.92628097	0.003259136	5.357976037	987.8996057
1235.068698	0.91902834	0.011693859	5.452474425	832.785306
171.5917738	0.963757396	0.075168004	4.398887191	164.1214815
773.4423513	0.959916589	0.011980487	5.364021365	635.6143144
794.313048	0.946382331	0.010873982	5.406041164	622.0117324
2026.834614	0.886431624	0.005729763	5.271364725	1161.504557
1167.718521	0.928306041	0.005707161	5.385019007	843.1643825
557.722111	0.957807462	0.012452249	5.360475404	489.5265315
1022.028989	0.916550561	0.039397435	5.298478071	656.0015675
1973.281996	0.88190168	0.005689977	5.400494707	1174.450153
650.8042234	0.952699976	0.012764822	5.28689278	508.7461538
770.4261008	0.955120397	0.007232431	5.348383743	641.2028986
565.9102063	0.950469043	0.036214171	5.30388026	458.2929042
671.056005	0.966412714	0.006411023	5.409301791	606.4531074
1002.773892	0.920487502	0.006095928	5.03781889	709.8053095
1326.218717	0.945580439	0.003082819	5.576153411	1063.115443
796.1315691	0.951840612	0.011520212	5.335048732	624.3881047
196.3959277	0.963834673	0.113023396	4.582301917	177.9671667
558.8333836	0.95712738	0.015022805	5.180835177	476.6101397
1019.760997	0.929389827	0.007433039	5.268057829	736.1903791
763.6390936	0.939261464	0.022823676	5.182844501	556.6768868
1239.537666	0.904032372	0.012670233	5.279209122	804.3222675
804.9443817	0.947025901	0.007149889	5.222634702	640.4509248
1908.162148	0.871578674	0.006181649	4.788297411	1129.144368
970.3355537	0.94574254	0.053889258	5.584712232	764.4623369
934.9229361	0.956048967	0.008190512	5.585359063	793.8351682
378.526313	0.957522756	0.032158225	5.288717293	350.0829349
1265.42025	0.934511435	0.007981818	5.447812865	935.6965994
942.505568	0.955033616	0.007291304	5.254148122	796.835913
1035.668016	0.942679784	0.00681268	5.375590304	797.1180031
1612.67856	0.90098857	0.006030953	5.30503898	961.4240761
1252.593633	0.915857605	0.004403656	5.085412319	884.466073
1695.790893	0.841027519	0.009377964	5.110827335	910.5841292
772.838068	0.938319745	0.022055006	5.217028792	577.0096562
1275.807541	0.936103819	0.010660273	5.41119781	964.0229282
617.5441879	0.963881296	0.012545642	5.280599323	549.8698783
1263.158438	0.925579269	0.006750663	5.291377714	874.1014785
557.6823172	0.958088996	0.031331623	5.223458131	448.0911718
1247.482906	0.93191294	0.006595253	5.244948248	917.7841346
1194.817604	0.943648785	0.006201437	5.412737814	943.4349972
836.0068911	0.944743476	0.010412144	5.373211686	649.1884569
1546.794862	0.881965944	0.006937513	5.155172925	875.3845829
1497.965544	0.899241765	0.005614675	5.038790929	979.5280162
1766.679715	0.922766885	0.003413709	5.481231499	1325.002269
788.8387983	0.963881996	0.009251513	5.542510134	693.7628978
677.7047158	0.961710626	0.013448106	5.367039698	577.5552683
810.2177956	0.949432535	0.009614146	5.266334917	636.7689074
1043.020251	0.946755625	0.009012923	5.466187887	814.7580844
1330.319558	0.906292232	0.005663211	5.228081012	842.0433841
1152.703625	0.966880342	0.008546369	5.72673229	993.6310113
867.9271394	0.970573051	0.006925819	5.619088969	785.2036155
1389.166304	0.944043421	0.012777929	5.421123191	1089.342794
238.9488301	0.960212202	0.041272293	4.668213166	222.9457178
957.4402237	0.931447497	0.011733934	5.252680438	703.4346696
1556.146857	0.906192055	0.002874718	5.346584654	1060.200274
932.2899273	0.940083053	0.010570003	5.307430054	689.5990438
715.7709828	0.95945678	0.010331484	5.435308446	612.3108798
919.4516986	0.907103825	0.02459106	4.623843241	629.8979308
244.2751488	0.967765568	0.040232633	4.520678415	236.5887981
1326.318547	0.913749395	0.009385879	5.43199667	925.7602063
1593.896557	0.946938331	0.006355529	5.560389076	1260.209633
1222.304385	0.915173867	0.010407665	5.334649714	822.7764958
1595.039942	0.907523039	0.003449896	5.431301206	1066.256764
1400.138527	0.919658708	0.002983807	5.159346029	1006.243749
1161.394772	0.957737914	0.015379431	5.534747558	987.1342835
798.9965294	0.94388917	0.011464782	5.499184687	637.0839703
354.2427472	0.966854284	0.047066672	4.849195346	320.0209489
233.8079618	0.951288817	0.03605418	4.913693482	211.3257508
1876.995603	0.922886283	0.004582071	5.282168563	1381.726166

original_glrlm_RunLengthNonUniformityNormalized	original_glszm_GrayLevelVariance	original_glszm_SmallAreaHighGrayLevelEmphasis	original_glszm_GrayLevelNonUniformityNormalized
0.857436638	118.1932581	574.5255181	0.02831611
0.797647079	127.2798406	474.9998816	0.027676297
0.905449663	106.9599213	578.8822061	0.028396375
0.887719796	174.5068578	594.2099796	0.023648985
0.935633409	81.59731358	231.1761049	0.035614815
0.932476648	85.9009834	325.2102885	0.03063707
0.89674564	43.32399477	153.31093	0.048265351
0.90907033	124.7915973	353.207608	0.029597347
0.901520098	126.8174942	583.2994364	0.026704605
0.850622595	123.5343233	637.8473989	0.028124408
0.90958414	83.93069943	268.5071858	0.032967864
0.864628164	176.1488673	749.4000959	0.024157905
0.915329074	84.73255281	312.0179867	0.03044249
0.928530263	85.5744834	320.4988663	0.030637037
0.932485934	104.1609954	456.2319958	0.027614305
0.742852808	122.0637724	627.486367	0.031366181
0.931915285	100.1181693	331.2298848	0.029770381
0.874228108	120.8571367	439.8666942	0.02755929
0.927800055	92.77163335	267.3359385	0.0314202
0.793802715	91.64526675	441.0947045	0.029622975
0.804128654	81.95904161	504.9426051	0.032392139
0.928204914	91.38172748	693.1710602	0.029159416
0.861145785	81.87562889	415.2704863	0.031600318
0.916690686	128.5879959	389.6656534	0.028855398
0.882917929	85.92022882	670.9713719	0.031608937
0.869295435	130.984375	422.7901374	0.028225309
0.815660383	116.9226308	500.1387008	0.029190011
0.86255855	105.1343856	463.7700035	0.030386716
0.909253903	146.7746631	470.5651316	0.026288545
0.802585099	137.4422787	565.430088	0.027454364
0.856791732	93.93445802	495.3563881	0.029955716
0.874353071	99.84433557	543.7285928	0.027974542
0.865634935	169.1902863	510.5138888	0.025507069
0.93307299	75.15976331	178.4963414	0.053254438
0.926824642	125.1075392	460.9063811	0.028491076
0.904964683	111.7733896	398.1427945	0.027913381
0.821319675	163.5955898	613.0278236	0.024853126
0.876660142	115.1177897	500.6717028	0.027653271
0.919996734	97.32559279	335.915612	0.029990344
0.86571879	149.6556572	408.4571226	0.027783547
0.812485053	170.7691478	693.8753692	0.023863386
0.914476301	99.87420785	340.5308594	0.03113478
0.917846781	113.9963399	435.7066495	0.02855139
0.908617797	111.5016427	316.9577404	0.030077527
0.935735197	110.6089421	458.4432723	0.027550991
0.861321129	82.39416723	370.7319748	0.032688763
0.901407038	138.589246	708.1898257	0.024908755
0.912771122	108.071558	383.713386	0.030153238
0.927877727	78.49851279	151.3658401	0.051160024
0.917537662	108.0469104	354.7319006	0.033584368
0.876124377	105.5368512	397.6195496	0.028806668
0.892967033	120.1708732	338.918903	0.031848794
0.838922518	146.7576374	457.2625457	0.027383544
0.902060599	83.19819336	382.5198497	0.032128906
0.791374723	145.7023145	576.6739464	0.029780767
0.903355096	167.4826552	497.9909667	0.0238849
0.916966165	147.7622689	524.2999006	0.024729513
0.918353624	132.4814907	342.1930581	0.030423543
0.884535644	143.1169459	554.9372878	0.025786036
0.916742711	98.28603158	531.2601175	0.031929547
0.896922263	118.9554004	455.2807603	0.027178096
0.843592201	134.9055736	557.7147726	0.026792908
0.854420231	100.6398825	483.7371946	0.03075505
0.753952781	179.5837315	617.9680622	0.026279332
0.887789441	115.1565503	360.9470149	0.032550065
0.884421702	163.0000829	509.0070696	0.025038403
0.93005629	102.9133298	370.9603628	0.032585327
0.87362939	134.5592834	496.7466358	0.027051078
0.922064716	99.05486281	263.8237154	0.033626446
0.879653157	133.6286839	517.0929626	0.027348139
0.897775739	136.7631862	533.9630262	0.024935975
0.89971181	109.0279038	373.0456726	0.028189635
0.814124404	107.5473812	447.081659	0.028613663
0.829514807	112.8016953	492.0778414	0.028935051
0.863379605	189.7964599	862.8949776	0.02186316
0.930660361	151.4953124	509.6638235	0.024857499
0.928494805	115.3161866	378.7756221	0.029144913
0.906187899	95.26909481	374.567948	0.030299205
0.904892888	127.2149128	493.8534351	0.025679012
0.844730661	102.0415327	485.0688571	0.029803014
0.937801628	204.7384169	720.6144613	0.021793334
0.942495217	164.3776356	587.1947342	0.023617307
0.899191796	214.1443459	649.6597941	0.023379265
0.923639757	85.40078386	221.3574504	0.045434751
0.880076144	83.74626017	403.544391	0.030403385
0.839592129	115.779053	616.6503133	0.027888076
0.895054118	95.80381749	416.1814752	0.029268442
0.923516132	133.6853391	404.1570166	0.028388469
0.833055885	173.9555556	397.9997218	0.045688889
0.93971569	96.53728208	285.8589726	0.043058181
0.854218882	194.5938464	611.6103336	0.02365987
0.905698755	199.5785174	813.5363243	0.021896739
0.854798999	124.8543351	434.6789959	0.027675956
0.846988886	134.7920318	659.0109823	0.026014269
0.860385398	109.5531064	563.8377769	0.029215968
0.918362528	209.3560938	661.0450733	0.0240625
0.897911679	188.2572663	517.7228238	0.026327671
0.934985258	105.2129249	235.9924005	0.03947569
0.908737437	59.6500377	202.0736456	0.039494769
0.863571659	168.2359018	719.691774	0.026566387

original_glszm_SizeZoneNonUniformityNormalized	original_glszm_SizeZoneNonUniformity	original_glszm_GrayLevelNonUniformity	original_glszm_LargeAreaEmphasis	original_glszm_ZoneVariance	original_glszm_ZonePercentage
0.658246759	251.4502618	10.81675393	56.44502618	53.02930292	0.541076487
0.547248117	413.7195767	20.92328042	286.702381	279.1544242	0.363986519
0.625942445	157.1115538	7.12749004	14.77290837	11.78317804	0.578341014
0.556736417	791.122449	33.6052076	62.11893033	58.68039823	0.539278937
0.508464198	114.4044444	8.013333333	3.782222222	1.236424691	0.626740947
0.569209594	231.6683047	12.46928747	5.83046683	3.334532656	0.632970451
0.453258818	75.24096386	8.012048193	8.620481928	4.499092757	0.492581602
0.535242634	309.3702422	17.10726644	14.46020761	11.45496342	0.576846307
0.511673624	564.8876812	29.48188406	17.10960145	13.56333934	0.531024531
0.526439804	749.6502809	40.0491573	191.8047753	187.0717061	0.459651388
0.48926276	225.0608696	15.16521739	13.31086957	10.07869093	0.556227328
0.570902599	405.3408451	17.15211268	69.54084507	65.18392382	0.479082321
0.457654099	522.6409807	34.76532399	7.802101576	4.503838474	0.550626808
0.495716872	334.6088889	20.68	6.444444444	3.701127023	0.603756708
0.505602082	285.1595745	15.57446809	4.179078014	1.57005118	0.61909989
0.487646185	824.6096984	53.04021289	2583.449438	2566.571967	0.243414423
0.493391776	209.1981132	12.62264151	4.830188679	2.120127269	0.607449857
0.516230949	582.3085106	31.08687943	84.94326241	80.66921936	0.483704974
0.528851227	185.6267806	11.02849003	4.894586895	2.145226094	0.603092784
0.514374266	566.3260672	32.61489555	507.715713	501.2435182	0.393073902
0.544249855	1184.287684	70.48529412	968.4981618	962.7346318	0.416539051
0.544496696	576.0775047	30.85066163	6.344045369	3.701115991	0.615116279
0.53626044	707.3275208	41.6808188	173.4230478	169.1360445	0.48297327
0.516887463	146.2791519	8.166077739	5.692579505	2.803780794	0.588357588
0.573391964	341.7416107	18.83892617	30.17449664	26.09355434	0.495016611
0.524274691	188.7388889	10.16111111	34.01111111	30.29478395	0.518731988
0.509393676	643.3642122	36.86698337	469.5851148	462.0541535	0.364396999
0.532124225	359.7159763	20.54142012	69.24112426	64.74747689	0.471737613
0.579028873	590.030422	26.78802748	15.01766438	12.01752762	0.57733711
0.572651119	545.1638655	26.13655462	418.3214286	411.1916311	0.374508261
0.542987919	790.0474227	43.58556701	228.5491409	223.7754415	0.457691098
0.478853586	1349.888258	78.86023413	54.0297978	49.60473707	0.475379427
0.530843321	525.0040445	25.22649141	118.2113246	113.985785	0.486473192
0.513846154	33.4	3.461538462	3.938461538	1.378461538	0.625
0.599091401	128.8046512	6.125581395	5.088372093	2.703861547	0.647590361
0.517572194	567.2591241	30.59306569	33.89233577	30.49544462	0.542574257
0.587996343	756.163297	31.96111975	399.1244168	394.1090461	0.446527778
0.518626519	858.3268882	45.76616314	143.4706949	139.3636436	0.493440668
0.458475806	212.2742981	13.88552916	5.933045356	2.68059281	0.554491018
0.607763764	323.9380863	14.80863039	90.55347092	86.68003337	0.508102955
0.520328652	321.5631068	14.74757282	196.368932	190.6801667	0.4192673
0.544446707	307.6123894	17.59115044	14.20353982	11.35849636	0.592864638
0.50897137	472.325431	26.49568966	10.50431034	7.373546188	0.565164434
0.530775178	308.3803787	17.47504303	9.788296041	6.675895616	0.566829268
0.519619362	613.1508475	32.51016949	4.138983051	1.606029876	0.628328009
0.526101798	532.4150198	33.08102767	171.5197628	166.6728038	0.454219031
0.525021643	1202.299563	57.04104803	47.12751092	43.65718121	0.536802625
0.572785977	204.4845938	10.76470588	12.00840336	9.341540538	0.612349914
0.497620464	40.80487805	4.195121951	3.804878049	1.134443783	0.611940299
0.512673459	148.1626298	9.705882353	6.633217993	3.675770166	0.581488934
0.503537415	834.3614967	47.73264937	125.4628847	121.1929376	0.483936916
0.551166573	241.4109589	13.94977169	35.54109589	31.9760691	0.529625151
0.538781192	863.6662508	43.89582034	379.6887087	374.0068944	0.419523685
0.535488281	171.35625	10.28125	16.903125	13.49217773	0.541455161
0.536376645	1058.807497	58.78723404	1642.363222	1631.44378	0.302621493
0.581060263	309.1240602	12.70676692	15.08458647	12.35463777	0.60523322
0.530528641	676.9545455	31.55485893	8.210815047	5.257050221	0.581851345
0.47995755	354.2086721	22.45257453	6.277777778	3.208159826	0.570765661
0.551574975	228.9036145	10.70120482	32.28433735	28.43706779	0.50982801
0.505896937	243.8423237	15.39004149	8.952282158	5.611426284	0.547105562
0.53945059	613.355321	30.90149516	50.41600704	46.94271753	0.536573856
0.525164245	1288.753056	65.74979625	489.5183374	484.5352909	0.447973713
0.489255573	618.9083004	38.90513834	149.2031621	143.2364386	0.409385113
0.559408653	450.8833747	21.18114144	667.5	656.0191692	0.295129989
0.542935086	68.95275591	4.133858268	8.275590551	5.356066712	0.585253456
0.510110069	384.1128818	18.85391766	46.9309429	42.67737902	0.484867997
0.523078474	127.6311475	7.950819672	4.262295082	1.654864284	0.61928934
0.565773264	895.6190777	42.82185723	181.4725205	177.3938048	0.495151705
0.552581818	151.96	9.247272727	8.458181818	5.827887603	0.616591928
0.551317007	542.495935	26.91056911	85.31199187	81.1352855	0.489308802
0.520598493	805.3658694	38.57595346	26.12281836	22.26122499	0.508881579
0.522768759	318.3661741	17.16748768	21.72577997	18.28898757	0.539415412
0.549965219	1152.727099	59.97423664	820.9780534	814.8986292	0.405572755
0.53718078	1387.000775	74.71030209	899.468629	892.7029888	0.384455033
0.528139326	2093.016149	86.64370426	310.0893263	304.7234925	0.431699346
0.525755951	368.5549215	17.42510699	4.47360913	1.939108793	0.628136201
0.556959142	378.1752577	19.78939617	6.328424153	3.622181108	0.607878245
0.554412322	197.3707865	10.78651685	11.37078652	8.434762025	0.583606557
0.550629528	545.1232323	25.42222222	35.2969697	32.12209366	0.56122449
0.555029752	730.9741838	39.25056948	180.1442673	175.4744677	0.462754743
0.645720237	229.8764045	7.758426966	4.191011236	2.186718849	0.706349206
0.573887545	525.1071038	21.60983607	3.404371585	1.256465108	0.682326622
0.550266371	817.6958277	34.74158816	26.60296097	23.07281057	0.532234957
0.466686021	38.73493976	3.771084337	5.21686747	2.164900566	0.572413793
0.531950948	888.3580838	50.77365269	74.75389222	70.6308039	0.492480094
0.482772252	1057.271233	61.07488584	408.0767123	401.824429	0.399926954
0.607675928	268.5927602	12.93665158	27.760181	24.66194386	0.568123393
0.512844991	471.8173913	26.1173913	5.795652174	2.949829868	0.592783505
0.497244444	37.29333333	3.426666667	25.13333333	19.17973333	0.409836066
0.631590002	43.57971014	2.971014493	4.594202899	2.278512917	0.657142857
0.522916472	896.8017493	40.57667638	206.7504373	201.3783135	0.431446541
0.654865409	275.6983373	9.218527316	14.08551069	11.27331712	0.59631728
0.514579381	239.7939914	12.89699571	54.51502146	49.7051889	0.455968689
0.543015044	987.744365	47.31995602	307.0610225	302.0053234	0.444743276
0.537000734	1333.909823	72.57246377	432.8204509	427.3572033	0.427833276
0.589140625	94.2625	3.85	4.53125	2.030898438	0.632411067
0.496718895	321.3771252	17.03400309	13.68160742	10.14929923	0.532072368
0.563481177	45.64197531	3.197530864	3.666666667	1.360768176	0.658536585
0.472099161	97.25242718	8.13592233	7.208737864	3.823852389	0.54353562
0.54153679	202.5347594	9.935828877	25.18983957	20.5339372	0.463444857

original_glszm_LargeAreaLowGrayLevelEmphasis	original_glszm_LargeAreaHighGrayLevelEmphasis	original_glszm_HighGrayLevelZoneEmphasis	original_glszm_SmallAreaEmphasis	original_glszm_LowGrayLevelZoneEmphasis
0.042337398	99869.37696	684.0602094	0.837465082	0.006684073
0.287221608	416351.0384	636.5701058	0.76744038	0.010737443
0.020422395	22766.85657	739.752988	0.817754471	0.008534019
0.064451691	110419.1217	768.2744546	0.774323775	0.008749917
0.093491133	1115.64	309.1377778	0.741290123	0.023761718
0.031321523	5095.022113	428.5601966	0.782417845	0.012599672
0.234782346	666.1506024	186.4578313	0.699660161	0.027051721
0.135194925	17572.16955	460.733564	0.759978121	0.021223073
0.026883281	24945.10326	794.7871377	0.743390009	0.006492434
0.123017426	318548.1819	878.2401685	0.753728621	0.004570205
0.05346003	14282.71739	382.7695652	0.726839356	0.01306845
0.049331078	119321.6507	969.1661972	0.783470571	0.005677024
0.052306902	6059.562172	454.6716287	0.703341395	0.011825225
0.039835642	5403.127407	463.7185185	0.730757199	0.009901309
0.02410185	2431.85461	605	0.738890292	0.007317673
1.591126696	4366248.04	894.9432289	0.726011603	0.005243432
0.04278108	3314.542453	456.6509434	0.728768692	0.012164905
0.093082038	126206.9122	605.3643617	0.746680773	0.008483092
0.135803858	2511.207977	368.9373219	0.755267769	0.02254529
0.374967085	729959.7875	611.5349682	0.744536185	0.00618947
0.680099218	1392144.961	686.0206801	0.766090198	0.004072751
0.009313758	9113.932892	918.8412098	0.766314047	0.00321469
0.149756474	231766.6444	573.0742987	0.760802209	0.006695199
0.119273656	3681.922261	491.335689	0.747008238	0.015676489
0.023155555	48378.91443	899.8926174	0.784792851	0.004102535
0.096327791	45005.15556	587.875	0.752079107	0.023428988
0.429404491	533421.791	676.7339667	0.741866796	0.007140545
0.074620107	94568.30917	661.0754438	0.757513039	0.009027503
0.109357554	20025.78705	583.703631	0.78836739	0.014269697
0.274511412	697145.7773	754.5367647	0.784645147	0.007346771
0.171272281	327719.8976	686.8151203	0.765313631	0.005844314
0.043640035	86723.8989	788.203973	0.719027749	0.003537426
0.141183135	184794.4338	675.7815976	0.756775116	0.011399291
0.376234784	365.8615385	210.0769231	0.744920809	0.048644144
0.031681135	5381.702326	578.3023256	0.799753441	0.012157901
0.080807674	48605.06022	531.2755474	0.747629839	0.009001826
0.226813928	768270.9596	796.0046656	0.794902954	0.006905336
0.11701806	206168.3408	673.0193353	0.748216414	0.006360727
0.052604772	2989.343413	477.6177106	0.703614522	0.01387709
0.25108729	113135.9531	481.2645403	0.807385266	0.038070101
0.132179026	337251.2508	936.2135922	0.749202104	0.007031081
0.059290503	16769.99115	433.8902655	0.766306738	0.011718913
0.047525439	11440.52155	588.7478448	0.741455554	0.006471965
0.23310894	8511.333907	416.4578313	0.756698298	0.023339014
0.023285846	2516.266102	608.7686441	0.749210918	0.006193702
0.168716952	191453.4812	519.0059289	0.753408907	0.008458467
0.034555217	84674.43799	932.5244541	0.752910075	0.003273169
0.046229842	15408.31373	497.0560224	0.785176629	0.012070811
0.391575911	600.402439	195.2439024	0.728702575	0.091115042
0.059542729	4649.550173	459.7716263	0.743237811	0.016144203
0.121867768	168114.2263	557.092939	0.737050273	0.008144065
0.139385072	45333.03653	422.1210046	0.770792478	0.020876425
0.368391904	509738.6781	589.9875234	0.762455782	0.014219246
0.030895742	18819.2375	514.409375	0.759811643	0.009104034
1.164903911	2502636.882	783.1752786	0.760355535	0.007655545
0.62457574	21545.15226	644.3139098	0.790402378	0.030754345
0.047716693	8568.241379	711.4153605	0.756699675	0.007110821
0.206832086	1236.474255	430.7222222	0.719078952	0.024109304
0.044707431	51726.6747	722.8240964	0.771049674	0.008509601
0.033450102	9500.458506	722.4564315	0.739290131	0.005449857
0.054932092	75167.31926	617.4582234	0.762716553	0.007841513
0.307732375	898398.3839	729.299511	0.752887548	0.005293017
0.128249557	191367.5921	679.1849802	0.727069047	0.006156486
0.682316287	761074.6675	786.5397022	0.774981268	0.010220127
0.064819091	7781.755906	492.1889764	0.763658804	0.026216614
0.06355143	81301.50996	711.3559097	0.742405517	0.01237255
0.04236132	2394.557377	522.1557377	0.751568878	0.010616954
0.139257931	301036.2097	643.1857233	0.780560531	0.006976437
0.083776734	8169.945455	346.5163636	0.771874489	0.035052861
0.074022043	137643.9024	680.0193089	0.770886943	0.007386512
0.035263149	38288.41888	743.6354234	0.749624663	0.007415474
0.05399623	27860.22824	508.8965517	0.75098789	0.011125661
0.608046416	1163004.614	592.3454198	0.769938675	0.007669072
0.660791464	1282343.175	678.4566228	0.761291513	0.00746692
0.185784138	551614.8082	1140.700984	0.754929719	0.004415637
0.032071113	3946.827389	667.2582026	0.753299143	0.008789893
0.045939802	5652.455081	498.5876289	0.774544375	0.013360555
0.033968826	13011.63764	498.4213483	0.772573353	0.010730451
0.056501547	54964.59293	652.8747475	0.770606534	0.009039238
0.14059775	278197.8679	620.0273349	0.773448751	0.005918601
0.018206812	6138.252809	883.6629213	0.830570824	0.00901894
0.018643055	3163.375956	755.0535519	0.786227248	0.006865459
0.064877141	48944.22275	869.1736205	0.770136386	0.013573822
0.168146885	735.686747	277.373494	0.708973839	0.033007812
0.147025886	90755.9988	548.8329341	0.757663341	0.009463907
0.269849613	641048.6479	859.3497717	0.722430917	0.003531265
0.046988024	36614.46833	523.6945701	0.807396128	0.011810495
0.041292335	5407.330435	547.4097826	0.744171078	0.009624577
0.147414529	22265.34667	524.8933333	0.730792212	0.032991708
0.162013852	511.0869565	308.2608696	0.820268728	0.038097918
0.198598583	291226.9079	786.3172012	0.751356702	0.010349617
0.017992057	26173.90499	1020.04038	0.834950105	0.008870493
0.077171682	76504.72747	597.776824	0.745374881	0.012868687
0.206113492	493960.5327	860.6190214	0.765343976	0.0036306
0.29758886	647511.7983	764.0394525	0.761130297	0.003764261
0.034980966	5876.6375	871.775	0.79325825	0.019046033
0.062401447	15109.11128	644.8423493	0.732490874	0.010918555
0.133793995	1077.37037	315.6419753	0.778166152	0.053517446
0.265239018	1071.878641	253.6165049	0.713418561	0.025148832
0.022749962	44627.36898	1065.510695	0.763209915	0.007666394

original_glszm_ZoneEntropy	original_glszm_SmallAreaLowGrayLevelEmphasis	original_ngtdm_Coarseness	original_ngtdm_Complexity	original_ngtdm_Strength	original_ngtdm_Busyness	original_ngtdm_Contrast
5.998991163	0.00602867	0.004458057	7.256683767	6.442296774	0.161495375	0.001660059
6.394750243	0.008290915	0.001973481	2.058503921	3.496998508	0.271327719	0.000316984
5.991025081	0.007990866	0.010765113	10.22027374	10.41440034	0.06635998	0.001996454
6.709535333	0.006976275	0.001498979	5.064190457	4.457652652	0.299005316	0.000257148
5.969458489	0.017774217	0.016592562	7.931233664	10.17549014	0.216202018	0.001663798
6.103060331	0.010944085	0.009692978	4.491751539	5.003250838	0.201464691	0.001246705
5.828302462	0.01910362	0.014423851	4.079735328	6.813928831	0.320847002	0.000889017
6.299257865	0.013806934	0.003658475	5.194824224	3.41271953	0.378659728	0.001576183
6.656822886	0.005572032	0.002584584	4.005220278	4.25158987	0.205354014	0.000252446
6.624559381	0.003881365	0.001479568	3.127710428	4.470396211	0.221712693	8.72E-05
6.266587196	0.010056522	0.005784359	4.245318304	3.740348118	0.309749664	0.001165878
6.637359074	0.004836566	0.003198299	8.415159167	10.51999326	0.097713297	0.0002288
6.610620903	0.009435734	0.003471165	1.195834717	1.733603519	0.542473992	0.00031047
6.345645228	0.007810502	0.006081855	2.505759705	3.318428632	0.261728437	0.000597171
6.495264426	0.005730288	0.007773566	4.945922668	5.69722584	0.166500953	0.00057664
6.665377324	0.003791235	0.000807543	0.40487794	2.050747413	0.462445898	3.59E-05
6.277620467	0.00889776	0.008583814	5.244477147	5.620272739	0.2146216	0.00116835
6.562356331	0.006450245	0.002152968	2.586524307	3.220667386	0.276630268	0.000266127
6.208428034	0.01510346	0.010017041	5.671993143	6.199877089	0.252025665	0.001486078
6.429228521	0.004684256	0.001646705	1.082026554	2.595959256	0.35347143	0.000169218
6.304137374	0.003357141	0.000674551	0.568449631	1.052928172	0.923081899	9.61E-05
6.428707255	0.002753331	0.003828477	2.877941216	3.466731992	0.184826825	0.000271535
6.31166434	0.004932135	0.001789043	1.151940773	1.742822191	0.431457644	0.000218593
6.253695958	0.010270317	0.010758297	10.03151544	10.37119868	0.170504451	0.002081483
6.198348968	0.003756677	0.004144567	3.075666484	4.624461418	0.1387562	0.000394796
6.311874418	0.01754028	0.006114014	6.895637184	6.525975241	0.121603144	0.001487141
6.680393189	0.005757485	0.001429441	1.707767935	3.751450936	0.287349552	5.37E-05
6.284903497	0.006984609	0.002786916	2.686278868	3.395639434	0.22909105	0.000495478
6.438265663	0.010637355	0.002232852	3.473936481	2.565951741	0.530062804	0.00082459
6.407597541	0.005066564	0.001612587	1.558693875	3.15753453	0.297156184	0.000251611
6.384955616	0.004847946	0.00127108	1.133491579	1.677087812	0.49366331	0.000188366
6.728408183	0.002673265	0.00114568	0.661273829	1.414331005	0.48980858	6.84E-05
6.675933123	0.008718886	0.001696921	6.249452156	6.120536176	0.272534051	0.000314062
5.241920798	0.032147455	0.023841852	25.19099504	18.38126279	0.29799681	0.010275488
5.997117913	0.009525938	0.013293536	17.2056523	11.51290151	0.091266553	0.003444775
6.49562935	0.006219894	0.002487104	2.107115697	2.252470339	0.515540981	0.000486544
6.525796514	0.00553181	0.000943281	3.351531878	3.25950597	0.384663337	0.000240709
6.623971828	0.004676341	0.001543389	2.845698668	3.898625614	0.274669829	9.78E-05
6.509939981	0.011623947	0.008042137	3.790345822	4.935361295	0.261289597	0.000796957
6.231356701	0.028623762	0.002847886	8.562702255	7.756270539	0.231346811	0.00078851
6.683894582	0.004988076	0.002870138	4.179764355	6.730593325	0.147717504	0.000331779
6.23361035	0.008764256	0.004919022	4.177248916	3.901397691	0.32865974	0.001047089
6.482252902	0.004896866	0.002818274	3.037519931	2.581631747	0.470630959	0.000614968
6.325726321	0.014268773	0.004186326	4.478515562	3.649259463	0.41392898	0.001189565
6.559017098	0.004971089	0.003155855	2.48469322	2.383372295	0.483830426	0.000367568
6.290202884	0.007164136	0.00213533	1.305641562	1.938762963	0.366144733	0.000239482
6.788258485	0.002602357	0.001416544	3.573173664	4.364582473	0.237700531	7.17E-05
6.074135265	0.010303274	0.007856079	7.500527225	6.437632407	0.151859403	0.001894183
5.354639432	0.057974682	0.027338748	20.26097141	19.70015095	0.180519597	0.006474
6.191964012	0.013524738	0.008847437	9.588215344	8.952029022	0.178218474	0.00166465
6.548017823	0.006199933	0.001460485	1.041323942	1.443457217	0.561563175	0.00022204
6.162720872	0.014419002	0.003699852	6.457958595	4.400485134	0.323675418	0.001858481
6.608552131	0.010752566	0.000918486	2.748631841	3.228331034	0.474634278	0.000126092
6.104592574	0.007886068	0.010741132	4.61982641	6.85653486	0.106103991	0.001063422
6.604405654	0.005761879	0.000631878	0.664134174	1.674404648	0.614064595	5.79E-05
6.365855863	0.021857503	0.005046206	7.547121064	5.710474063	0.19236787	0.001795623
6.672267466	0.004991756	0.002173784	3.661823209	2.743802482	0.400394319	0.000428518
6.567684266	0.016219233	0.003900432	4.454263615	4.85307602	0.820808884	0.000517162
6.36486949	0.005647083	0.007491163	7.156340178	9.657422705	0.077335087	0.00084359
6.380843576	0.002898084	0.00805608	6.327878561	11.70302833	0.077667553	0.000354271
6.483360268	0.006460238	0.002139089	2.454649953	2.316926406	0.370008314	0.000424484
6.63565658	0.00358724	0.000596608	1.053935265	1.264866571	0.980181766	0.000170597
6.594132077	0.00496322	0.001914024	1.582884829	3.742629927	0.24439879	8.41E-05
6.634862926	0.007322494	0.001480962	2.633191009	6.2947766	0.245206448	7.93E-05
5.699017711	0.023362144	0.021367744	18.38514508	18.71835509	0.052557597	0.004322664
6.657536678	0.009138045	0.003449282	3.390650432	4.687610823	0.174865552	0.000588151
6.044948687	0.006255327	0.011456359	13.2249681	9.372993169	0.137790391	0.002135522
6.468532932	0.005377005	0.001081016	1.76647937	1.704048686	0.637367232	0.000335967
6.005861465	0.028918751	0.010334971	8.998601038	7.707246319	0.158483607	0.002479094
6.465334326	0.0053391	0.001875497	3.005956646	2.869837011	0.336770614	0.000479761
6.71402197	0.005791546	0.001745327	2.021664634	2.358782754	0.32126316	0.000212001
6.418936018	0.008858707	0.00426336	3.686855431	3.584489945	0.266492427	0.000929356
6.465299465	0.00559578	0.000756845	1.04331933	1.541231204	0.635212319	9.07E-05
6.519278812	0.006046661	0.000649435	0.51489037	1.040307326	0.83279994	7.83E-05
7.084252175	0.00357477	0.000676698	1.100572383	2.258389826	0.411690291	2.64E-05
6.596779299	0.006909474	0.004475604	5.972584095	4.511300456	0.287190974	0.00097648
6.296432507	0.010041853	0.004809934	3.600980715	3.439317062	0.289667671	0.000875509
6.084010917	0.008771334	0.006926862	6.563467043	5.299378307	0.164978641	0.001775524
6.488439942	0.00719565	0.00284464	2.965852979	2.796059599	0.285292314	0.000588415
6.338406495	0.004254384	0.001190105	1.550958969	1.608019211	0.643371617	0.000343511
6.335182667	0.007791988	0.010760213	20.30952944	14.95382676	0.069341309	0.002166972
6.574403	0.005663958	0.003857868	6.805257293	4.584441679	0.257448528	0.000717263
6.728354721	0.010592068	0.001613721	3.461733215	3.845018126	0.28967415	0.000355456
5.525288528	0.016212773	0.029894293	16.8587519	19.92666058	0.109096358	0.004538986
6.421017555	0.005755467	0.001350831	1.098125111	1.337168095	0.59023663	0.000181688
6.83811077	0.002839833	0.001132095	1.543268756	3.687335314	0.281329689	3.34E-05
6.096337044	0.009041981	0.00574157	5.240652852	5.100855337	0.171702769	0.001161169
6.528295268	0.007289371	0.003030193	3.657502377	3.001297616	0.4862942	0.000721102
5.373632484	0.025663302	0.018697235	17.14847781	26.45638358	0.059809974	0.008593014
5.195197645	0.031513297	0.021660518	31.95097	19.07298496	0.168504226	0.011496897
6.911411076	0.007781815	0.001121821	3.171737939	4.383030458	0.329478736	9.66E-05
6.394277211	0.008385719	0.006410742	15.23875898	11.604002	0.068198102	0.001182371
6.437929008	0.010072557	0.004984084	4.573155072	6.170330122	0.128497355	0.000723686
6.685652227	0.002761562	0.001032736	2.556453162	3.488538922	0.331469766	7.49E-05
6.582827773	0.003064113	0.000811871	1.515276857	2.368307863	0.419657635	4.01E-05
6.118074174	0.017576421	0.016205265	36.63942827	22.11040323	0.045018945	0.006212513
6.698140927	0.00791739	0.005069923	8.597180268	14.78574123	0.138561846	0.000418682
5.320969447	0.049450951	0.02650117	29.33450635	20.28472405	0.098847904	0.010690953
5.912256338	0.016524422	0.016541969	4.621375209	7.589619506	0.241386274	0.001094283
6.456908612	0.007189765	0.006730328	6.848887039	10.05187217	0.063124769	0.00077616

wavelet.LHL_gldm_GrayLevelVariance	wavelet.LHL_gldm_HighGrayLevelEmphasis	wavelet.LHL_gldm_GrayLevelNonUniformityNormalized	wavelet.LHL_gldm_DependenceEntropy	wavelet.LHL_gldm_DependenceNonUniformity
75.34886124	1004.562323	0.038291777	6.693746429	209.7790368
33.6805163	494.3480982	0.061185754	6.898813859	390.7717862
57.65444159	597.5207373	0.042738219	6.251593508	147.1474654
98.73048965	1704.169639	0.03403044	6.985436433	837.9085389
41.35819865	640.5849582	0.050628099	6.135132592	105.8579387
40.96153093	390.7620529	0.045260721	6.334439297	187.9642302
45.39911419	962.9317507	0.046606028	6.143289559	112.4599407
97.39499942	1497.513972	0.02911343	6.73621366	369.3173653
76.85343645	1948.133718	0.036973644	6.824310547	650.017797
53.88248202	1263.678179	0.044237499	6.785773667	836.9593286
48.21590253	727.0483676	0.042655012	6.573530762	232.2091898
83.45139469	1353.397436	0.033967666	6.978553662	422.6531714
126.1612646	2879.121504	0.033287644	7.113007375	591.8505304
27.6266597	346.7826476	0.054843334	6.503790221	263.8050089
77.40487589	3439.877058	0.035787744	6.711524489	288.3260154
34.16548276	2094.962142	0.103142801	6.752156873	975.3166835
60.19441753	889.3538682	0.038837941	6.589267422	208.7851003
84.45820455	1643.59048	0.036484705	7.104417362	608.5154374
51.936928	471.4123711	0.039867267	6.416173288	183.2714777
32.35163068	800.2806141	0.061458262	6.849578392	551.4227062
41.18256441	819.8638974	0.059080015	6.850501726	1107.578101
117.5128444	1631.763372	0.03194159	7.03040346	539.5581395
41.73200546	876.8806298	0.052357214	6.757481307	657.8202124
119.8279053	2409.480249	0.029343753	6.640877812	181.952183
39.33231697	530.7425249	0.051493637	6.613330975	300.2724252
57.479634	799.8458213	0.040698785	6.632188807	202.0115274
28.10057349	541.5427005	0.061873166	6.653475019	727.6497403
38.18197517	720.2191207	0.049736327	6.62625943	359.4256804
68.00787551	722.7008499	0.037338234	6.728983395	540.9660057
29.76295018	427.1864673	0.082255557	6.830628302	383.0810386
44.94918178	761.5681032	0.044953607	6.730934195	864.4759358
23.09224337	484.2986509	0.074850604	6.811571892	982.4097808
51.53887323	642.2464338	0.041067831	6.788483231	559.8548942
100.7669194	1153.894231	0.049001479	5.700063756	33.23076923
54.73191864	378.8403614	0.039029612	6.278902706	106.0180723
41.12296123	727.0183168	0.051318498	6.683547035	491.0891089
52.43249409	770.2072917	0.041646653	6.843022575	761.0590278
35.5452904	1160.533691	0.05632058	6.669015437	793.0113298
23.73315644	375.239521	0.062500627	6.283066123	201.2778443
50.07825147	731.392755	0.044666444	6.640318676	295.7302193
48.63246108	982.8595658	0.046989059	6.621349145	395.1275441
37.57718763	480.4742917	0.046937434	6.511769724	259.4868835
64.11192643	599.5749086	0.03783301	6.753615401	500.1084044
43.74798239	560.4341463	0.045305413	6.566742084	278.8419512
103.863448	1529.911076	0.028907103	6.928824809	666.3471778
24.67071187	357.241921	0.071651722	6.470702776	451.4236984
107.5836015	4124.189639	0.036004879	7.089749889	1284.088608
52.85224882	500.8679245	0.041410578	6.427368998	178.3241852
44.99069949	390.0074627	0.048785921	5.48743952	47.07462687
39.32068062	656.6981891	0.047905137	6.252064255	145.1207243
33.42158422	627.9751752	0.051967045	6.78891611	787.1559579
58.82987562	651.7847642	0.036461387	6.715112076	228.0979444
53.1820849	818.2886679	0.043456933	6.902072072	991.8987176
30.08206	297.4128596	0.052476373	6.273106508	150.7123519
30.10388876	611.2738004	0.105384128	6.707047846	922.7675916
56.82595669	940.5858931	0.040516877	6.528824195	275.3094425
118.2610686	1594.425445	0.027263092	7.070339406	752.2603739
47.03999943	792.6720804	0.046167447	6.617363862	347.6372776
36.79445092	694.3955774	0.058965041	6.60521525	176.0540541
90.16129128	2472.842225	0.055683035	6.695246833	205.6696935
55.11423755	788.1717791	0.041213444	6.771681977	597.1444077
69.21845529	1864.422052	0.037420951	6.907183844	1642.526104
30.97206104	1125.061489	0.06853154	6.656220663	641.1365696
19.68975275	1465.889052	0.094899	6.814390863	349.0402783
42.50419419	280.8294931	0.043895602	6.013949203	67.44239631
101.9091586	2006.925306	0.033915206	6.881245055	519.1841597
83.04875802	834.2817259	0.031745214	6.427406192	145.6954315
44.84975035	629.2126994	0.047437243	6.858864542	779.8476697
44.29783929	473.4192825	0.044340325	6.154320865	140.2466368
64.89392973	1160.69816	0.039320535	6.72870945	627.9766285
51.91471942	630.9661184	0.048194252	6.973711205	703.6973684
38.57572446	449.8857396	0.04711052	6.670258725	282.2010629
50.84249705	1668.974071	0.068456293	6.943291892	962.756192
29.8047383	764.4600953	0.075467819	6.857175889	1153.607207
31.4608811	1020.227669	0.060960386	6.713548984	1990.666013
38.5731459	485.5394265	0.049116147	6.541535694	292.7150538
41.10816878	665.4816473	0.046985266	6.474651504	316.4341987
100.3652889	1449.301639	0.03011556	6.82239394	200.347541
69.61150447	710.0113379	0.035039413	6.753133504	568.3854875
57.33359602	887.6278988	0.039910288	6.858517793	817.2354181
43.8518361	821.4206349	0.048603238	6.453159374	134.3333333
61.33486257	934.4690529	0.038110284	6.599583283	449.541387
79.24987634	1584.923352	0.034177675	6.776375944	982.4383954
53.48318668	386.4827586	0.047705113	5.665846636	55.96551724
58.63207107	826.8911825	0.04528745	6.847830762	882.7994692
55.28114297	1062.949416	0.041537387	6.979369274	1405.542367
63.01039677	1386.829049	0.03949551	6.649041665	222.5115681
70.64796339	980.1056701	0.034957388	6.746205674	506.0657216
17.68240318	351.3934426	0.085012989	5.787878341	35.55737705
96.41360544	1301.495238	0.0430839	5.508253227	42.18095238
33.76467614	1242.826918	0.061707369	6.87795097	770.6110692
97.69768636	836.9830028	0.030282724	6.756144523	234.8895184
58.83771221	1055.654599	0.038453437	6.681472942	322.0665362
68.67734584	1312.213936	0.040945116	7.150567058	992.6440098
26.07190573	876.2473303	0.065447043	6.693284092	1140.028247
259.896827	1797.280632	0.020950179	6.43952385	121.0632411
104.3843429	1495.073191	0.031059286	7.034237787	365.3815789
61.90653711	414.9186992	0.040782603	5.526960571	49.11382114
86.50395082	2022.736148	0.040371482	6.417621085	119.5488127
80.05004537	1380.872367	0.041325207	6.756280346	220.6208178

wavelet.LHL_gldm_GrayLevelNonUniformity	wavelet.LHL_gldm_SmallDependenceEmphasis	wavelet.LHL_gldm_DependenceNonUniformityNormalized	wavelet.LHL_gldm_DependenceVariance
27.03399433	0.51371325	0.297137446	1.511696587
127.0828117	0.360492435	0.18814241	5.196655763
18.5483871	0.553428059	0.33904946	0.907749156
89.67020873	0.547965909	0.317991855	1.70282196
18.17548747	0.481693748	0.29486891	1.196452541
29.10264386	0.497460459	0.292323842	1.343469207
15.70623145	0.532251072	0.333709023	1.068689519
29.17165669	0.569911011	0.368580205	0.741100633
76.86820587	0.523941244	0.312658873	1.25738847
137.0477728	0.462284643	0.270161178	1.502940109
35.27569528	0.474201085	0.280784994	1.281285046
50.34008097	0.48233278	0.285191074	1.496389422
69.03857281	0.483808182	0.285366697	1.335938641
61.31484794	0.401235971	0.235961546	1.818187346
32.60263447	0.54184227	0.31649398	1.376853942
716.5330358	0.312123414	0.140393937	9.021392368
27.10888252	0.52562095	0.299119055	1.61493748
85.08233276	0.460977245	0.26094144	1.92249455
23.20274914	0.508428312	0.314899446	0.98425857
172.1445912	0.382676743	0.196866371	6.328340684
308.6339969	0.422963711	0.212017248	5.339036582
54.93953488	0.527617157	0.313696593	1.190561114
142.9875503	0.4505764	0.240871553	3.321384685
14.11434511	0.617235776	0.378278967	0.882335398
61.99833887	0.453740462	0.249395702	2.488970872
28.24495677	0.506938016	0.291082892	1.415135081
214.4523947	0.380916795	0.209939337	3.318770854
71.27215632	0.451801731	0.250820433	2.069007431
65.901983	0.513463951	0.30649632	1.331810704
209.0936271	0.319181147	0.150700645	11.06734446
142.9075181	0.460074255	0.271933292	1.541758044
443.8640809	0.313541229	0.165667754	5.088160182
83.49090015	0.482977261	0.275383617	1.535142844
5.096153846	0.528782051	0.319526627	1.190828402
12.95783133	0.542912483	0.319331543	1.085135724
103.6633663	0.410535127	0.24311342	1.873263406
119.9423611	0.450756484	0.264256607	1.518192515
188.8992248	0.432198214	0.236437487	3.189842654
52.18802395	0.418429101	0.241051311	1.931256051
46.8551001	0.49095749	0.28191632	1.695600059
69.26187246	0.480687816	0.26806482	1.811756562
44.73137461	0.454301395	0.272284243	1.539055438
62.12180268	0.511509619	0.304572719	1.264163456
46.43804878	0.475544403	0.272040928	1.537307793
54.28753994	0.578980585	0.354817454	0.990284909
159.6400359	0.379633489	0.202613868	3.122304987
153.596812	0.527681996	0.301005299	1.857068692
24.14236707	0.520557938	0.305873388	1.1473687
6.537313433	0.570688226	0.351303186	1.017821341
23.80885312	0.498779343	0.291993409	1.254140537
177.9351636	0.411218557	0.229893679	3.262690792
30.15356711	0.464920469	0.275813718	1.396466592
166.0489401	0.452442865	0.259591394	2.101458893
31.01353638	0.440631698	0.25501244	1.510445744
687.4206653	0.296204685	0.141463681	8.149993294
35.61433447	0.515006636	0.313207557	1.179375674
59.7879617	0.559539943	0.343027986	0.975536929
59.69450889	0.444415315	0.268861004	1.449078236
47.997543	0.414397523	0.216282622	5.961521048
49.05675369	0.444738577	0.233450276	3.052433709
87.33128834	0.484676373	0.281804817	1.590487735
204.9919679	0.513945055	0.299840472	1.347976659
211.7624595	0.402797882	0.207487563	4.720719724
259.1691688	0.262818758	0.127806766	10.08280103
9.525345622	0.522802099	0.310794453	1.127991675
52.67031552	0.557505731	0.33431047	1.338869703
12.50761421	0.601175268	0.369785359	0.811152052
151.6568658	0.429659588	0.243931082	2.666342361
19.77578475	0.513905082	0.314454343	0.938808341
79.07359523	0.531174979	0.312270825	1.295993864
146.5105263	0.433204353	0.231479398	3.429499654
53.18777679	0.433231701	0.249956654	1.977681559
353.7821207	0.381687712	0.186291833	6.055495715
506.8418702	0.341052153	0.171769983	6.686916756
559.6163399	0.401345576	0.21684815	3.078578325
54.81362007	0.440641197	0.262289475	1.771675595
52.48254252	0.489230811	0.283289345	1.442825897
18.3704918	0.562321707	0.328438592	1.342800322
61.80952381	0.534840296	0.322213995	1.239543966
113.5846803	0.502127333	0.28715229	1.762813412
24.49603175	0.469679733	0.266534392	1.642841396
51.10589113	0.562156972	0.335228477	1.079942456
95.42406877	0.581251884	0.351876216	0.938953703
6.917241379	0.634963602	0.385969084	1.079667063
153.5697434	0.462121595	0.260336027	1.962805424
227.458729	0.446871079	0.256673186	1.908623912
30.72750643	0.493615162	0.286004586	1.566378758
54.25386598	0.540783085	0.326073274	1.030356573
15.55737705	0.316183692	0.194302607	2.749798441
4.523809524	0.517592593	0.401723356	0.50521542
245.2867925	0.355596348	0.19386442	4.31326014
21.3796034	0.544246329	0.3327047	0.944089111
39.29941292	0.539718307	0.315133597	1.39890702
167.4655257	0.444543046	0.242700247	3.22637263
379.9855322	0.351819562	0.19635347	3.263100201
5.300395257	0.70079271	0.478510834	0.550297614
37.76809211	0.518959029	0.300478272	1.710688625
5.016260163	0.59733514	0.399299359	0.596205962
15.30079156	0.511553408	0.315432223	1.067035178
33.34944238	0.493711812	0.273383913	2.476803803

wavelet.LHL_gldm_LargeDependenceEmphasis	wavelet.LHL_gldm_LargeDependenceLowGrayLevelEmphasis	wavelet.LHL_gldm_SmallDependenceHighGrayLevelEmphasis	wavelet.LHL_gldm_LargeDependenceHighGrayLevelEmphasis
5.764872521	0.009092997	526.5098679	5741.060907
14.62927299	0.036254966	190.6774203	6709.446798
4.322580645	0.017719939	358.0948515	2382.073733
5.722580645	0.004472467	966.8681438	9174.962808
5.456824513	0.013226597	298.4089748	3590.133705
5.628304821	0.025913598	214.2971974	1930.342146
4.709198813	0.008629216	502.0181166	4585.709199
3.826347305	0.004193747	834.9505755	5890.783433
5.175084175	0.007040659	1042.828976	9856.720539
6.306649451	0.006026216	590.8410707	7840.782117
5.764207981	0.011532099	347.7817612	4068.385732
6.002699055	0.00642226	676.7502019	7521.976383
5.7512054	0.002874317	1478.578403	15274.30183
7.745974955	0.031492352	141.3524879	2641.414132
5.267837541	0.002790099	1922.524487	17243.47969
24.32776738	0.013211973	676.2064683	50139.99223
5.871060172	0.010283975	453.6084451	5228.517192
7.09948542	0.005577596	807.3170846	10956.46012
4.807560137	0.022750848	241.7062772	2254.659794
15.69617994	0.021960955	318.1001756	11959.41307
13.54862175	0.01875845	362.4498156	10713.40716
5.047674419	0.00478459	887.7291755	7788.14593
9.592456975	0.01260276	406.5950796	8167.265837
3.881496881	0.003995631	1510.07912	9139.808732
8.171096346	0.026115053	256.8953738	4024.149502
5.720461095	0.011090831	424.0235505	4433.364553
10.79976919	0.023425142	214.8772438	5627.353433
7.517794836	0.012991241	335.2037139	5303.184927
5.388668555	0.012075154	405.4290328	3373.603966
26.35169158	0.068963398	145.0554786	10853.40755
6.355772255	0.011412174	358.1271052	4672.726958
16.03946037	0.038561623	168.746014	7250.465261
6.187407772	0.013711066	323.263771	3834.030005
5.038461538	0.084645911	689.5740946	5323.490385
4.801204819	0.038942615	208.8668081	1835.725904
7.623762376	0.013188327	307.6594733	5330.84505
6.475	0.011434708	360.9366989	4840.028125
9.55515802	0.009206281	504.155819	10785.72153
7.697005988	0.027008296	155.5316057	2848.161677
6.300285987	0.011945353	367.2689711	4376.256435
6.727272727	0.009205395	479.8734221	6389.984396
6.380902413	0.018295795	211.2932332	3220.604407
5.313032887	0.021954725	313.9563504	3028.032887
6.266341463	0.016356052	277.0787997	3328.549268
4.264110756	0.004135603	865.4042144	6898.048456
10.78635548	0.037717656	144.8047863	3597.868492
6.209563994	0.002024809	2212.280366	25017.43483
5.09948542	0.017565355	278.0849886	2316.58319
4.388059701	0.040216964	217.2871061	1848.977612
5.490945674	0.012430242	326.9817796	3605.273642
9.886098131	0.018755314	265.6543843	5945.770736
6.044740024	0.01581797	300.9082112	3938.681983
7.426066475	0.011545556	377.4575927	5808.556137
6.736040609	0.037871432	138.6342973	1910.282572
23.1162042	0.043953415	190.3213878	12959.06132
5.102389078	0.008587056	498.461637	4638.460751
4.374373005	0.004393028	889.2789397	6890.216598
6.351894818	0.010644075	352.2907686	5061.304718
14.26781327	0.023129973	281.2403121	9932.766585
9.476730988	0.005702344	1138.766461	22378.26107
6.178857952	0.010680004	396.9091581	4655.647003
5.479006937	0.003532923	981.5718926	9897.419861
12.78705502	0.012647498	462.5269361	13718.56505
27.97839619	0.020161808	381.5958664	40026.58989
5	0.051823644	142.9459242	1336.654378
4.99871217	0.008236244	1135.65748	9733.01159
3.842639594	0.010683883	525.4482995	2966.337563
8.602126994	0.017590206	280.1673485	5179.128245
4.726457399	0.018658014	245.6971518	2241.221973
5.218796619	0.005870535	631.2612207	5886.195922
10.07368421	0.023302929	282.6041326	5937.972697
7.49955713	0.025275856	205.0221519	3169.434012
15.90595975	0.010261003	650.4140752	26038.8185
17.75967838	0.024867724	270.1244672	13389.58189
10.09934641	0.01074793	419.5980302	10104.91983
6.935483871	0.019930144	219.9594258	3268.03405
5.925693823	0.013204125	334.943991	3789.957923
5.072131148	0.008010909	833.5079525	6824.137705
5.020408163	0.011943145	407.216492	3273.750567
6.281799016	0.009183757	462.1420696	5395.865425
6.563492063	0.011803056	411.8380622	5018.603175
4.597315436	0.007546854	535.2660844	4213.703952
4.202722063	0.003532204	923.3289538	6640.590974
4.172413793	0.03355821	239.5318027	1609.227586
7.149808316	0.011643201	401.9583624	5498.567974
7.206355004	0.008296797	495.9904922	7342.384405
6.064267352	0.006302528	704.9144431	8160.281491
4.648195876	0.007084106	525.4142158	4569.890464
11.36065574	0.079718864	118.6401021	3931.087432
3.60952381	0.013243725	648.8351852	4899.971429
13.10616352	0.011485007	440.5657406	15917.34667
4.450424929	0.01093871	488.8116687	3497.334278
5.328767123	0.007011939	583.0281865	5501.860078
9.414180929	0.008731131	616.8384023	11607.80954
11.40613159	0.014378235	316.8507968	9704.316914
2.770750988	0.007136714	1286.464561	4614.806324
5.991776316	0.006360053	790.5020842	8754.699836
3.37398374	0.107402538	255.3796296	1282.934959
4.910290237	0.005682011	1056.637253	9613.92876
7.602230483	0.010333201	699.1559795	10218.92193

wavelet.LHL_gldm_SmallDependenceLowGrayLevelEmphasis	wavelet.LHL_gldm_LowGrayLevelEmphasis	wavelet.LHL_gldzm_DistanceZoneVariabilityNormalized	wavelet.LHL_gldzm_LowIntensityEmphasis
0.002642789	0.003330933	0.935407066	0.004690173
0.001845152	0.003524126	0.934832342	0.004839824
0.004175154	0.006257366	0.911454082	0.007360713
0.000910323	0.001230333	0.875204405	0.001547076
0.004500509	0.005455683	0.899367547	0.008139218
0.004835623	0.007063639	0.874310707	0.009101441
0.003901939	0.004439149	0.769816491	0.006409884
0.00160577	0.00196031	0.844914756	0.00255225
0.00073868	0.001744912	0.932597632	0.001700154
0.000862863	0.001353027	0.939382618	0.001743769
0.002332204	0.003254872	0.855727788	0.004451689
0.001171774	0.001659192	0.877486252	0.002207907
0.000695596	0.000905953	0.915130892	0.001288899
0.003544654	0.00603023	0.900980174	0.008198212
0.001271043	0.001417657	0.860729172	0.002071195
0.000391664	0.000950913	0.921385805	0.001397158
0.003000317	0.003678336	0.758572777	0.00524826
0.000837483	0.00123376	0.951516679	0.001673064
0.004794769	0.007145316	0.838962346	0.008851275
0.001070782	0.001944608	0.937759122	0.002619289
0.001023024	0.001917789	0.889357517	0.002389725
0.001312834	0.001682114	0.856257861	0.00225749
0.001109417	0.001842247	0.920792039	0.002340526
0.002472005	0.002654573	0.741242067	0.003614829
0.001799396	0.004376719	0.885963927	0.004600245
0.003008514	0.003816941	0.910574608	0.005391241
0.001428133	0.00284297	0.88417821	0.003624973
0.00186363	0.002779031	0.955345271	0.003832385
0.001913338	0.00292554	0.950262828	0.003493861
0.002547069	0.004587614	0.949507625	0.007513524
0.001552429	0.002514365	0.951469934	0.003163317
0.001227506	0.002947573	0.949847374	0.003790766
0.002191552	0.003325924	0.939817137	0.004287976
0.005552855	0.020962016	0.807810651	0.017011487
0.009863204	0.014089242	0.828395589	0.017292523
0.001468646	0.002477954	0.886687903	0.003360331
0.001389036	0.002366923	0.947474192	0.00292666
0.000783348	0.001317963	0.916631979	0.001712107
0.004153185	0.006023463	0.836934729	0.009100703
0.002121725	0.003005939	0.941134484	0.003989213
0.002089855	0.002686288	0.840414109	0.003942262
0.003030973	0.004597075	0.896415838	0.006163913
0.004151334	0.006641218	0.901619495	0.008039944
0.002442682	0.003742528	0.821978357	0.004792913
0.001257142	0.001618691	0.870620827	0.002021937
0.003099944	0.005347213	0.959159752	0.007758698
0.000568352	0.000690725	0.834535686	0.000981799
0.003868196	0.005365874	0.917053694	0.006943602
0.010556754	0.013491707	0.798553719	0.01680142
0.003192405	0.004115893	0.834710744	0.005819833
0.001278339	0.002429319	0.943310778	0.002975642
0.002592731	0.003927317	0.944012075	0.005137915
0.001338404	0.002186711	0.919948983	0.002790794
0.00707828	0.009967645	0.936171835	0.014620774
0.001083789	0.002497348	0.952135259	0.003515463
0.002115404	0.002843943	0.842020465	0.003720738
0.001219612	0.001610184	0.718487461	0.002011174
0.001852653	0.002704456	0.768320714	0.00375086
0.002481784	0.003411338	0.847583066	0.00545536
0.001597371	0.001877127	0.977631498	0.003259216
0.001725803	0.002593761	0.924614201	0.003322
0.000545368	0.000841297	0.929072709	0.001004722
0.000793213	0.001363853	0.956096276	0.001840002
0.000568234	0.001087682	0.944220785	0.002014403
0.014092456	0.017750979	0.872933884	0.024299793
0.000879868	0.002106615	0.902649296	0.001957464
0.003917561	0.0047739	0.789708176	0.006011402
0.001654694	0.002865524	0.965400103	0.003649451
0.005133281	0.006681279	0.901673525	0.008955359
0.0012094	0.001693296	0.925055482	0.002118571
0.002978205	0.004416644	0.868642123	0.0064256
0.003889179	0.005659137	0.939146405	0.008148543
0.000499276	0.000906307	0.941026814	0.001241615
0.000812623	0.001804423	0.95757963	0.002323695
0.000609005	0.001256445	0.950529225	0.001472542
0.003110734	0.004659384	0.827926243	0.006380756
0.003165732	0.00411215	0.873182383	0.005850152
0.003903662	0.00430775	0.894308154	0.006179108
0.0023987	0.003420496	0.858588867	0.004219367
0.001224143	0.001956963	0.895780349	0.002325702
0.002823872	0.003620505	0.870493365	0.005421499
0.002237327	0.002847742	0.825424425	0.003675393
0.000956354	0.001261244	0.907388584	0.00153647
0.020282397	0.02180286	0.824677161	0.029020909
0.001390997	0.002279769	0.968469216	0.002863633
0.000812366	0.001448043	0.82	0.001731036
0.001804817	0.002225155	0.943526067	0.003263712
0.001737922	0.002359852	0.809422222	0.002972434
0.003865712	0.01437273	0.917959184	0.017929074
0.010316796	0.010807393	0.915657439	0.016148257
0.000622716	0.00118104	0.917018221	0.001637389
0.003143221	0.00410127	0.926672565	0.005287058
0.001730552	0.002267621	0.86988204	0.002969499
0.000785436	0.001302665	0.936840975	0.001665609
0.000723349	0.001538768	0.941622202	0.001966424
0.00513728	0.005446682	0.689827569	0.006674023
0.001899666	0.002338334	0.935899393	0.003322938
0.007343621	0.023390194	0.812601406	0.017463009
0.003240742	0.003512507	0.837372449	0.005557852
0.003589762	0.004220849	0.884969523	0.006574955

wavelet.LHL_gldzm_LargeDistanceEmphasis	wavelet.LHL_gldzm_HighIntensitySmallDistanceEmphasis	wavelet.LHL_gldzm_LowIntensityLargeDistanceEmphasis	wavelet.LHL_gldzm_HighIntensityEmphasis
1.100238663	992.1264916	0.004899012	1013.033413
1.101162791	515.1569767	0.005505706	530.2093023
1.139285714	612.8830357	0.007734449	631.25
1.200607903	1669.11155	0.001783717	1756.240122
1.15942029	610.4154589	0.009549815	624.3285024
1.202156334	402.6219677	0.019005712	423.9433962
1.398104265	846.1421801	0.006980373	948.4407583
1.254160363	1380.328669	0.002832024	1473.406959
1.104761905	1931.040873	0.001789137	1983.20873
1.093862816	1246.182912	0.001832519	1277.034898
1.234782609	683.363587	0.004898567	738.1673913
1.196662694	1335.972884	0.002453286	1403.479142
1.133219471	2919.230145	0.001335833	3035.529462
1.156716418	341.9295709	0.00954217	352.9402985
1.22591944	3337.588004	0.002147751	3524.33275
1.122961104	2108.376725	0.00147739	2164.376412
1.421307506	794.4364407	0.014443906	870.1210654
1.07457899	1687.625702	0.001714997	1730.757017
1.264957265	441.4786325	0.01972299	475.8119658
1.096463023	801.4895498	0.002832417	829.7315113
1.176327523	813.1673085	0.002683079	853.7612485
1.233840304	1570.86193	0.002440968	1678.196768
1.123931624	877.8586182	0.002654398	900.1972934
1.458083832	2134.802395	0.003830302	2437.748503
1.182108626	530.5910543	0.005098703	561.0319489
1.140740741	799.545679	0.005671671	826.2716049
1.185160867	534.3557124	0.004285543	562.6769534
1.068548387	724.4909274	0.003968646	738.4637097
1.076559546	765.6517013	0.003824882	775.8185255
1.07775378	448.1862851	0.007921667	455.1274298
1.074652778	763.19864	0.003305303	777.7893519
1.077216397	526.9380362	0.004138256	536.7287893
1.093167702	649.8349601	0.005090903	660.9432121
1.323076923	1101.080769	0.017201802	1244.169231
1.28436019	365.7239336	0.042154255	383.2654028
1.180873181	700.0974532	0.003699057	748.8669439
1.080974325	778.6319947	0.003267732	795.4825543
1.130750605	1132.534504	0.001848116	1172.745763
1.268656716	350.2307214	0.010995703	372.5572139
1.091062395	725.0004216	0.004169203	748.4249578
1.262315271	943.3094212	0.004494877	1001.817734
1.164383562	452.3762231	0.007635432	467.0313112
1.155645982	590.3512208	0.00913346	614.1169888
1.296296296	538.787037	0.006170623	580.3597884
1.208569119	1425.024454	0.002475731	1499.590946
1.062565172	377.1551095	0.008091291	382.472367
1.273048254	3879.174382	0.001056437	4184.997254
1.130057803	508.732659	0.007266436	531.3063584
1.340909091	351.6477273	0.020469829	385.2272727
1.272727273	602.4938811	0.006665453	653.1818182
1.087591241	626.3955292	0.003180497	643.2354015
1.086474501	631.1502217	0.005318213	649.9135255
1.125310791	803.1310293	0.003444472	832.7722526
1.099009901	307.9339934	0.015624143	314.0033003
1.073602908	633.1089278	0.003710465	649.1281236
1.259398496	904.2142857	0.01005737	961.3157895
1.508438819	1421.804501	0.003294317	1596.604079
1.401162791	713.8735465	0.005530799	790.6046512
1.249350649	653.1253247	0.006318418	689.1584416
1.033936652	2537.930995	0.00327597	2556.165158
1.11769616	786.6260434	0.00373274	812.3038397
1.110457921	1851.784421	0.001091116	1903.356436
1.067368421	1130.518772	0.001902125	1154.710877
1.086142322	1426.539014	0.002083847	1460.601748
1.204545455	263.7064394	0.031929522	275.780303
1.153923541	1966.529678	0.002099003	2032.857143
1.358208955	798.9654851	0.007658854	865.4440299
1.052830189	638.0256289	0.003748916	649.8138365
1.155555556	456.7824074	0.009532905	478.162963
1.116978067	1143.960804	0.002256861	1182.779854
1.212021136	609.1917107	0.009120998	654.1268164
1.094240838	456.3054101	0.00865759	472.6614311
1.091234347	1656.636628	0.001312217	1699.392665
1.06504065	777.2080914	0.002444686	789.7684863
1.07613852	1024.135259	0.001612253	1042.938093
1.285229202	459.8348896	0.010380458	500.6112054
1.204113924	644.1095728	0.006584133	683.8607595
1.167938931	1413.253817	0.00660131	1484.076336
1.229704797	706.8380996	0.004940069	747.9261993
1.165454545	875.6401515	0.002617749	913.3206061
1.208791209	802.9075092	0.005770102	868.9047619
1.289871944	875.8448778	0.00499146	951.2037253
1.146024878	1525.329232	0.003288062	1587.595457
1.291262136	368.868932	0.068735523	381.9902913
1.048066298	853.1895028	0.002992741	866.6762431
1.3	1014.704283	0.002145924	1102.38007
1.087248322	1387.373602	0.003356238	1419.454139
1.32	892.5184615	0.003692478	972.2646154
1.128571429	362.0928571	0.018650219	368.5857143
1.132352941	1232.625	0.016363969	1271.470588
1.130116066	1206.876145	0.001793558	1247.311546
1.114349776	856.0403587	0.005478767	886.3766816
1.209856916	1017.66256	0.003270528	1071.785374
1.09793567	1346.68591	0.001774333	1383.25012
1.090283749	883.8301806	0.002107903	902.9084265
1.575757576	1586.910354	0.007333683	1835.217172
1.099447514	1485.272445	0.003419297	1520.941989
1.313953488	386.3488372	0.021390719	418.0232558
1.267857143	1912.552455	0.00570529	2066.700893
1.18380744	1349.022429	0.006725096	1412.463895

wavelet.LHL_gldzm_DistanceZoneVariability	wavelet.LHL_gldzm_ZonePercentage	wavelet.LHL_gldzm_IntensityVariabilityNormalized	wavelet.LHL_gldzm_LowIntensitySmallDistanceEmphasis	wavelet.LHL_gldzm_IntensityVariability
391.9355609	0.593484419	0.03078702	0.004637964	12.89976134
803.955814	0.414058739	0.039277988	0.004673354	33.77906977
255.2071429	0.64516129	0.035280612	0.007267279	9.878571429
1439.711246	0.624288425	0.026494582	0.001487916	43.58358663
186.1690821	0.576601671	0.043618287	0.007786569	9.028985507
324.3692722	0.576982893	0.039021803	0.006625374	14.47708895
162.4312796	0.62611276	0.040857124	0.006267262	8.620853081
558.4886536	0.659680639	0.027567913	0.002482306	18.22239032
1175.073016	0.606060606	0.030894432	0.001677909	38.92698413
1561.253911	0.536475145	0.034805035	0.001721581	57.84596871
393.6347826	0.556227328	0.038345936	0.004339969	17.63913043
736.2109654	0.566126856	0.028848408	0.002146563	24.20381406
1071.618275	0.56460945	0.026818772	0.001277166	31.40478224
482.9253731	0.479427549	0.046203219	0.007862223	24.76492537
491.4763573	0.626783754	0.030618849	0.002052057	17.48336252
2203.033459	0.344177343	0.037786163	0.0013771	90.34671685
313.2905569	0.591690544	0.033986246	0.002949348	14.03631961
1186.541299	0.534734134	0.028345174	0.001662581	35.34643144
294.4757835	0.603092784	0.037507812	0.006133346	13.16524217
1166.572347	0.444127097	0.039873709	0.002566007	49.60289389
2194.044994	0.472243492	0.035320713	0.002316386	87.13619781
900.78327	0.611627907	0.026292487	0.002211621	27.65969582
1292.792023	0.5140974	0.03575458	0.002262059	50.1994302
247.5748503	0.694386694	0.025404281	0.003560961	8.48502994
554.6134185	0.519933555	0.040119834	0.00447563	25.11501597
368.782716	0.583573487	0.034403292	0.005321134	13.93333333
1346.603414	0.439411425	0.042866892	0.00345983	65.28627708
710.7768817	0.519190509	0.039776997	0.00379832	29.59408602
1005.378072	0.599433428	0.030606666	0.003411106	32.38185255
879.2440605	0.364280094	0.039681577	0.007411488	36.74514039
1644.140046	0.543567159	0.037929205	0.00312782	65.54166667
1992.77979	0.353794266	0.045447523	0.003703894	95.34890372
1059.173913	0.554353173	0.034688602	0.004087244	39.09405501
52.50769231	0.625	0.039526627	0.016963908	2.569230769
174.7914692	0.635542169	0.037443004	0.01107709	7.900473934
852.993763	0.476237624	0.037839135	0.003275649	36.4012474
1439.213298	0.527430556	0.034932982	0.002841393	53.06319947
1514.276029	0.492546213	0.039768364	0.001678105	65.69733656
336.4477612	0.481437126	0.051025965	0.008626953	20.51243781
558.0927487	0.565300286	0.035606528	0.003944215	21.11467116
682.4162562	0.550881954	0.035872018	0.003804108	29.12807882
458.0684932	0.536201469	0.041808204	0.005796033	21.36399217
886.2919634	0.598660171	0.032188093	0.007766565	31.64089522
466.0617284	0.553170732	0.03817549	0.004448486	21.64550265
1076.957963	0.658679446	0.026159856	0.001908488	32.35974131
919.8342023	0.43043088	0.044818801	0.00767555	42.98123045
2127.231463	0.597515237	0.026708372	0.00096314	68.07963907
317.300578	0.59348199	0.035818103	0.006862894	12.39306358
70.27272727	0.656716418	0.046229339	0.015884318	4.068181818
238.7272727	0.575452716	0.040466526	0.005608427	11.57342657
1550.80292	0.480140187	0.041347731	0.002924428	67.9756691
425.7494457	0.545344619	0.035225982	0.005092841	15.88691796
1850.017404	0.526302015	0.033938401	0.002627375	68.25012432
283.660066	0.512690355	0.045670904	0.014369932	13.83828383
2095.649705	0.337421432	0.041652415	0.003466713	91.67696502
447.9548872	0.60523322	0.034859235	0.002136581	18.54511278
1021.68917	0.648426813	0.024644871	0.001690388	35.04500703
528.6046512	0.532095901	0.038944193	0.003305876	26.79360465
326.3194805	0.472972973	0.038205431	0.005239596	14.70909091
432.1131222	0.501702611	0.032227022	0.003255027	14.24434389
1107.687813	0.565361019	0.034202803	0.003219315	40.97495826
3002.762995	0.589996349	0.030700307	0.000983123	99.22339109
1362.437193	0.461165049	0.042754817	0.001824472	60.92561404
756.3208489	0.293299158	0.047778916	0.001997042	38.27091136
115.2272727	0.608294931	0.04476584	0.02239236	5.909090909
897.2334004	0.640051513	0.027055694	0.001922079	26.89336016
211.641791	0.680203046	0.031243039	0.005599539	8.373134328
1534.986164	0.497341257	0.037048376	0.003624585	58.90691824
243.4518519	0.605381166	0.039561043	0.008810972	10.68148148
1138.743298	0.612133267	0.033027774	0.002083998	40.65718928
1315.124174	0.498026316	0.033545124	0.005751751	50.78731836
538.1308901	0.507528787	0.039152801	0.008021282	22.43455497
2104.135957	0.432662539	0.032675587	0.001223965	73.06261181
2473.428184	0.384603931	0.040599446	0.002293448	104.8683701
4007.431214	0.459259259	0.041905509	0.001437614	176.6736243
487.6485569	0.527777778	0.039527731	0.005380831	23.28183362
551.8512658	0.565801253	0.039231493	0.005666657	24.7943038
351.4631043	0.644262295	0.026824389	0.006073558	10.54198473
930.7103321	0.614512472	0.03028962	0.004039191	32.83394834
1478.037576	0.579761068	0.033501561	0.00225269	55.27757576
237.6446886	0.541666667	0.040615063	0.005334348	11.08791209
709.0395809	0.640566741	0.032689553	0.003346376	28.08032596
1677.761493	0.662249284	0.030298634	0.001098573	56.02217415
84.94174757	0.710344828	0.040625884	0.019092256	4.184466019
1752.929282	0.533765851	0.032942828	0.002831355	59.62651934
2345.2	0.522279036	0.034004108	0.001627314	97.25174825
421.7561521	0.574550129	0.031985546	0.003240581	14.29753915
789.1866667	0.628221649	0.031653912	0.002792423	30.8625641
64.25714286	0.382513661	0.063673469	0.017748788	4.457142857
62.26470588	0.647619048	0.03633218	0.016094329	2.470588235
1501.158827	0.411823899	0.039438101	0.001598346	64.56017104
413.2959641	0.631728045	0.02690583	0.005239131	12
547.1558029	0.615459883	0.03325995	0.002894242	20.92050874
1951.43975	0.509290954	0.030400639	0.001638427	63.32453193
2190.213242	0.400620048	0.044252086	0.001931054	102.9303525
136.5858586	0.782608696	0.019385777	0.006509108	3.838383838
677.5911602	0.595394737	0.025636427	0.003298848	18.56077348
69.88372093	0.699186992	0.043537047	0.016481082	3.744186047
187.5714286	0.591029024	0.03260523	0.005520992	7.303571429
404.4310722	0.566294919	0.029471053	0.00653742	13.46827133

wavelet.LHL_gldzm_HighIntensityLargeDistanceEmphasis	wavelet.LHL_gldzm_SmallDistanceEmphasis	wavelet.LHL_glcm_SumVariance	wavelet.LHL_glcm_Homogeneity1	wavelet.LHL_glcm_Homogeneity2
1096.661098	0.974940334	3225.525175	0.217458061	0.131856394
590.4186047	0.974709302	1507.804537	0.3130268	0.231823088
704.7178571	0.965178571	1827.592446	0.206642629	0.118836335
2104.754407	0.949848024	5769.389415	0.200423549	0.119934425
679.9806763	0.960144928	1957.455393	0.244492335	0.153935067
509.2291105	0.949460916	1108.655374	0.227757599	0.137209281
1357.635071	0.900473934	3176.016526	0.213526967	0.125136893
1845.720121	0.936459909	4964.895404	0.185912036	0.102326221
2191.880159	0.973809524	6643.286264	0.209506996	0.123724112
1400.44284	0.976534296	4256.901603	0.245142723	0.1550961
957.3826087	0.941304348	2313.86841	0.243734223	0.154124954
1673.504172	0.950834327	4551.671173	0.217671663	0.130413192
3500.726729	0.966695132	10296.26463	0.221483809	0.134544516
396.983209	0.960820896	1001.531117	0.2764045	0.183688867
4271.311734	0.94352014	12395.87553	0.204679627	0.119895918
2388.375157	0.969259724	7375.539112	0.371659857	0.300620486
1172.859564	0.894673123	2785.893537	0.22086904	0.134657534
1903.282277	0.981355253	5634.481358	0.243229592	0.15694284
613.1452991	0.933760684	1389.741632	0.216986422	0.12949148
942.6993569	0.975884244	2631.695434	0.304956671	0.224192999
1016.137009	0.955918119	2648.281329	0.285668883	0.203757493
2107.536122	0.941539924	5618.475878	0.207265851	0.123476316
989.5519943	0.969017094	2796.845544	0.270618607	0.185103698
3649.532934	0.885479042	8320.428823	0.169242673	0.090291899
682.7955272	0.954472843	1621.491197	0.267979034	0.179509352
933.1753086	0.964814815	2539.231729	0.228584543	0.141325075
675.9619173	0.953709783	1701.032343	0.291591104	0.2034732
794.3548387	0.982862903	2296.078638	0.27410823	0.183316707
816.4858223	0.980860113	2152.008681	0.224687185	0.138146349
482.8920086	0.980561555	1287.616448	0.360273645	0.286550321
836.1521991	0.981336806	2458.406899	0.250294562	0.160305148
575.8918017	0.980695901	1495.700672	0.351163092	0.26889581
705.3762201	0.976708075	1959.765545	0.230882772	0.143544523
1816.523077	0.919230769	3748.766529	0.212164609	0.13152488
453.4312796	0.928909953	1037.027715	0.220639816	0.131459684
943.9449064	0.954781705	2322.509807	0.262041095	0.173718015
862.8847926	0.979756419	2390.410844	0.248346388	0.159593137
1333.590799	0.967312349	3932.507169	0.269889094	0.182692025
461.8631841	0.932835821	1091.758399	0.272405767	0.178866161
842.1231029	0.977234401	2283.444532	0.233851002	0.144898232
1235.850985	0.934421182	3201.40361	0.24090112	0.151602036
525.6516634	0.95890411	1390.940161	0.247931673	0.157738623
709.180061	0.961088505	1780.683239	0.213927414	0.128377703
746.6507937	0.925925926	1653.471027	0.235869654	0.146314078
1797.856912	0.94785772	5081.373325	0.190177822	0.106397897
403.7413973	0.984358707	1037.950945	0.314232275	0.227131893
5408.288741	0.931737936	14973.05894	0.210983147	0.129118352
621.6011561	0.967485549	1482.633437	0.217902811	0.129539319
519.5454545	0.914772727	1180.700244	0.225849676	0.13345482
855.9335664	0.931818182	2044.86626	0.234379649	0.143071437
710.5948905	0.97810219	2003.041135	0.278968421	0.191467853
724.9667406	0.978381375	1990.663018	0.245885261	0.156158555
951.3371457	0.968672302	2637.204711	0.248530485	0.161172008
338.2805281	0.975247525	856.5529814	0.254767496	0.16278643
713.2049069	0.981599273	2035.505749	0.372174777	0.302375607
1189.721805	0.935150376	2996.403929	0.217923984	0.13107334
2295.802391	0.872890295	5217.864203	0.18077273	0.101458229
1097.52907	0.899709302	2536.181684	0.234909984	0.145773
833.2909091	0.937662338	2181.588036	0.29158169	0.209187619
2629.10181	0.991515837	8717.740729	0.265905737	0.184157444
915.015025	0.97057596	2475.268	0.233335401	0.146023375
2109.644493	0.97238552	6432.505589	0.221744091	0.13490934
1251.479298	0.983157895	3838.012825	0.300926098	0.218395941
1596.852684	0.978464419	5150.275474	0.400748937	0.330317393
324.0757576	0.948863636	774.6608132	0.244088529	0.153046463
2298.167002	0.961519115	6795.317882	0.198078061	0.11738273
1131.358209	0.910447761	2577.579132	0.1934221	0.108285859
696.9666667	0.986792453	1962.579441	0.263992422	0.177050819
563.6851852	0.961111111	1378.658397	0.222452515	0.132872718
1338.056052	0.970755483	3823.954715	0.216498701	0.130036616
833.8672391	0.946994716	1977.322427	0.267302106	0.184074157
538.0855148	0.976439791	1311.073111	0.259500708	0.170658304
1870.416816	0.977191413	5779.335148	0.301910477	0.224266475
840.0100658	0.983739837	2458.771946	0.33080239	0.251732979
1118.149431	0.98096537	3408.98777	0.286603489	0.199747146
663.7164686	0.928692699	1436.257229	0.246414794	0.155844219
842.8655063	0.948971519	2078.889843	0.232397075	0.14146733
1767.366412	0.958015267	4842.97989	0.198286826	0.114271194
912.2785978	0.942573801	2145.52328	0.213583176	0.126437427
1064.042424	0.958636364	2839.825641	0.231579933	0.144509753
1132.893773	0.947802198	2644.216978	0.24883841	0.15914066
1252.639115	0.927532014	3001.409015	0.206684369	0.118964629
1836.660357	0.96349378	5359.913473	0.19602287	0.11192413
434.4757282	0.927184466	1090.347434	0.204895852	0.116747671
920.6232044	0.987983425	2655.064562	0.248054987	0.163985525
1453.083217	0.925	3491.661244	0.245559628	0.156937008
1547.776286	0.978187919	4633.082801	0.231488349	0.143111003
1291.249231	0.92	3155.164479	0.200337546	0.113422812
394.5571429	0.967857143	1106.000624	0.342714691	0.254875165
1426.852941	0.966911765	4337.95672	0.207883746	0.123371211
1409.053146	0.967470984	4238.606154	0.308918205	0.225479593
1007.721973	0.971412556	2609.805466	0.201643372	0.116832313
1288.27663	0.947535771	3449.455081	0.216841234	0.130239778
1529.506961	0.975516083	4436.623431	0.254152288	0.1695918
979.2214101	0.977429063	2893.177272	0.307194406	0.219869268
2828.444444	0.856060606	5687.592186	0.132175158	0.06363268
1663.620166	0.975138122	4910.815796	0.216060521	0.131654918
544.7209302	0.921511628	1187.835445	0.204929312	0.118193191
2683.294643	0.933035714	7121.360492	0.213901513	0.12681239
1666.229759	0.95404814	4651.403238	0.237016041	0.153933478

wavelet.LHL_glcm_ClusterShade	wavelet.LHL_glcm_MaximumProbability	wavelet.LHL_glcm_Idmn	wavelet.LHL_glcm_SumVariance2	wavelet.LHL_glcm_Contrast	wavelet.LHL_glcm_DifferenceEntropy
3.087189139	0.011595584	0.9691961	178.0742078	125.0555785	4.394608067
297.0555553	0.024832172	0.974072033	93.40469159	56.7982563	3.900741676
443.1503225	0.013291371	0.96648586	131.3450918	106.3250023	4.320575852
1559.527663	0.007280157	0.976938264	241.0127106	187.0024389	4.719229293
-659.0733579	0.014405335	0.964992657	115.7821405	70.66892118	4.005638004
230.1228524	0.011038224	0.959836999	95.22294507	76.51732778	4.110573291
-241.8205657	0.012588875	0.965293792	112.9088184	89.43739829	4.161733455
-634.1222922	0.005438397	0.96771151	271.5875627	153.9183248	4.604803986
-813.4452844	0.006972087	0.97820179	199.3585401	137.7299692	4.503317372
251.6647932	0.008161642	0.983922256	142.4046863	78.31084211	4.14957757
309.8079725	0.009933864	0.969735523	126.86701	79.21953515	4.136825783
2314.731811	0.006550899	0.979241159	254.0821921	104.4229729	4.353788384
9477.18052	0.006445697	0.987947917	396.3001788	154.1768867	4.451098232
17.5805848	0.011234395	0.966899099	67.01801757	46.158452	3.781006484
376.2494372	0.008650394	0.98161232	175.0512816	137.9362318	4.457266439
62.9930535	0.060235808	0.992508155	83.03378856	60.15558851	3.738067479
-782.6855708	0.008921385	0.955822057	148.5839001	110.5664863	4.300479651
2639.07808	0.008473812	0.984977518	270.5836033	104.9362612	4.290573544
186.3225013	0.008692438	0.949414215	139.9425167	95.0106573	4.210427341
159.1807637	0.027057238	0.980181185	77.01861916	69.13866253	4.015245173
242.5300393	0.029959497	0.980624107	96.81679925	74.3533552	4.096483109
3103.373543	0.007121034	0.978090794	296.2083189	190.2437832	4.631384477
14.44326753	0.019582896	0.979856812	102.8887491	71.77532987	4.070758966
-2192.32828	0.008585067	0.967955371	243.9322068	215.7222914	4.767349547
405.2913795	0.013881128	0.980996873	89.0105274	62.17556681	4.002669314
275.3250767	0.010018704	0.969236164	140.8796348	98.31412523	4.290471695
119.6958828	0.016827496	0.982024292	68.77798387	50.74027197	3.874600648
88.03832877	0.012330288	0.980268319	98.02614396	53.99834932	3.897356013
1539.075678	0.007879384	0.974624156	171.7930014	101.0820026	4.320391527
534.4673764	0.055961772	0.984132949	79.1775271	49.35926452	3.739479274
-126.7759342	0.008543802	0.977026106	122.4179911	68.07836638	4.081699108
272.9603409	0.026540074	0.987404563	65.76564371	33.78055132	3.564067487
194.0436232	0.008244864	0.967189015	127.4627648	92.89998446	4.286978956
-4133.014186	0.025665988	0.951151938	269.2427383	181.0835456	4.258720885
-135.6017958	0.011255806	0.94571987	148.1114735	82.95396909	4.163160977
318.293938	0.010436597	0.977419054	105.3327696	76.92094872	4.077766684
222.2871717	0.007370003	0.980221228	141.0677971	77.95175605	4.157935144
-53.85784865	0.02088839	0.981055045	81.81720862	67.34824301	4.043956728
-50.21873972	0.013990943	0.97033721	53.68168101	45.6900041	3.785047689
229.5677867	0.011686612	0.970933365	124.909126	88.8883614	4.221403894
91.62899029	0.011155218	0.979105995	112.1703184	92.33222337	4.229388916
-79.29500218	0.008641898	0.955877916	92.50736741	66.65065333	4.028978911
203.1239855	0.007382344	0.961115897	161.9546707	117.7002928	4.389141731
55.59490158	0.00979221	0.969878189	101.732855	80.9624101	4.180519488
-1659.017897	0.004815274	0.972399183	274.4123744	155.3962534	4.623463421
141.7202908	0.020830393	0.980395804	58.79602501	43.93076571	3.736229396
357.1949109	0.007296079	0.98361041	235.1968348	207.4878765	4.705713852
648.2547761	0.010554279	0.963161425	127.0138963	92.03347567	4.265021438
-837.8793751	0.029546055	0.941458961	129.3245156	71.71645615	3.870950219
-254.1437548	0.009885799	0.962321252	103.1146923	71.66715478	4.077042244
16.78716926	0.01396097	0.975201408	85.26825553	61.35471134	3.97615923
86.8075106	0.008254661	0.963659571	179.6784091	69.43048445	4.070425614
453.9956274	0.009603165	0.981385831	142.1171342	83.01238489	4.202972115
34.07657171	0.01254159	0.956608238	67.51097823	54.06762083	3.919883831
298.043791	0.065613639	0.979230754	90.2406706	60.07181494	3.782081464
-562.6062087	0.007367614	0.966254135	142.5477892	104.0233525	4.331013157
-2043.671343	0.004557519	0.957779899	303.9858178	210.5708762	4.760174533
-137.1895619	0.01033168	0.981044111	112.5243915	94.27199513	4.235369666
-286.8044374	0.029828177	0.968559809	87.72552895	66.74906318	4.00326006
-2218.107468	0.024897768	0.976478583	182.265915	187.86139	4.331147431
536.1141469	0.008330084	0.977157874	139.1367556	93.46987776	4.265675171
601.5566927	0.007029215	0.981874101	181.3004887	114.2299221	4.415949618
212.6936914	0.027920481	0.985347569	73.23743229	65.88388799	3.981268167
88.13746111	0.058522064	0.989481677	54.32120013	34.49539416	3.435619975
-90.66762136	0.015612691	0.937600109	116.1047426	65.15874648	3.895300555
1192.569947	0.008353273	0.978166209	258.7574425	192.708821	4.714905524
-507.5862256	0.009222691	0.958364864	224.2650343	125.2353768	4.406377064
317.0501275	0.015656775	0.97627187	122.0622357	71.10654255	4.083570523
49.93802116	0.010234681	0.951119645	102.9030938	83.05115494	4.111321113
732.0119003	0.006799092	0.978389322	157.4870942	116.9943359	4.421583309
362.7387495	0.017031369	0.970920966	143.3625513	93.78327328	4.199935262
247.0333383	0.009836357	0.967636845	105.0540676	64.55888955	3.995192084
55.37592671	0.034701501	0.98227322	131.9841725	102.0344173	4.173727962
44.80169178	0.037922141	0.986197309	80.22650783	53.78599227	3.844103657
78.71648572	0.016292717	0.985627747	76.95616138	59.29256399	3.968794691
134.0366784	0.010543413	0.965163649	99.2777781	69.97772098	4.090895852
69.76432718	0.010279745	0.974623632	98.9656078	75.74702923	4.140185015
-240.7455643	0.007691138	0.968874378	287.8754814	144.1705998	4.514673679
645.7300289	0.006814789	0.965669066	176.2396036	110.0984769	4.386784626
513.9173716	0.008581584	0.979169313	148.5340148	89.68684922	4.274231997
402.4885346	0.011882402	0.976373595	109.8944206	76.66566377	4.077152258
-134.974995	0.006763783	0.968856798	146.603926	109.2936627	4.385496458
299.3062416	0.005291288	0.981522361	205.1863745	140.3675106	4.586368054
15.32344199	0.021647448	0.94949521	151.7433671	98.62966743	4.101760379
728.8089407	0.011725538	0.969333557	152.1851376	107.8030231	4.311391982
450.3847549	0.006614568	0.984296255	152.8493232	83.16723758	4.193977408
146.20335	0.010762336	0.980825939	146.4138887	98.03389227	4.223891952
-321.0496662	0.005850523	0.969332496	199.6502624	115.0603006	4.443153144
-127.7699173	0.034563059	0.968223285	37.88481839	31.48553887	3.351034351
-2714.035902	0.025579198	0.958300171	229.7189028	135.6535579	4.075499502
-72.57015321	0.023370998	0.982970895	90.30692873	54.41153274	3.873682415
1695.914372	0.008479526	0.964032234	269.3684672	128.458763	4.469075913
-155.8427295	0.008202926	0.972263917	139.6347623	103.3727575	4.350312724
2052.266655	0.01329348	0.984163201	199.2313999	99.53765291	4.280967284
158.7194033	0.019722542	0.983592895	68.31239077	44.47408015	3.769497897
5086.669151	0.009283489	0.935505076	574.3724731	436.1980753	5.097452032
-587.2428766	0.00681035	0.978916367	300.1831436	131.2093269	4.462029963
-267.7736641	0.018650363	0.927501174	197.4225375	106.0086949	4.091194135
-176.8664883	0.012427417	0.978119371	197.0940984	151.4231995	4.323172027
-473.5468482	0.013713864	0.974833165	181.4203303	133.7062261	4.409189113

wavelet.LHL_glcm_InverseVariance	wavelet.LHL_glcm_Entropy	wavelet.LHL_glcm_Dissimilarity	wavelet.LHL_glcm_DifferenceVariance	wavelet.LHL_glcm_Idn	wavelet.LHL_glcm_Idm	wavelet.LHL_glcm_Correlation
0.13332747	8.882666238	8.197262681	55.244336	0.887607202	0.131856394	0.183373889
0.225030852	8.5992538	5.31083929	27.58944531	0.901482337	0.231823088	0.252182009
0.121890225	8.351788659	7.991428617	41.43490161	0.877887012	0.118836335	0.113045719
0.124276107	9.913944603	9.891728952	85.00705265	0.903475885	0.119934425	0.130101166
0.16673977	8.053187355	6.369126305	28.48736819	0.87824685	0.153935067	0.249349584
0.138178125	8.569693269	6.874256246	27.22479435	0.866466566	0.137209281	0.116587528
0.131590301	8.127709028	7.46757817	32.02573528	0.874066668	0.125136893	0.128531386
0.109398014	9.542696326	9.513268289	60.44944361	0.881247669	0.102326221	0.282736545
0.1293402	9.583703988	8.553907804	62.04000684	0.904785918	0.123724112	0.188122228
0.161772285	9.26255699	6.548484843	34.23017222	0.916251933	0.1550961	0.291603628
0.165092523	8.791879622	6.627397326	34.34242803	0.887839156	0.154124954	0.238289302
0.130085762	9.506446941	7.782815959	41.44876105	0.903485709	0.130413192	0.424271787
0.13918455	9.635986457	8.362758452	80.34480824	0.932383489	0.134544516	0.431619673
0.191578056	8.383063183	5.172320709	18.5567743	0.880557566	0.183688867	0.193388755
0.123600714	9.111825643	8.776135973	55.47974542	0.910211545	0.119895918	0.125550451
0.279705006	7.975363057	4.722678145	36.99666671	0.951652906	0.300620486	0.150987548
0.13816517	8.859906696	7.91308357	42.60932568	0.865661485	0.134657534	0.158767988
0.16450745	9.57088662	7.259542438	49.57718081	0.922292723	0.15694284	0.444464632
0.137074889	8.700622281	7.591959229	32.79308525	0.853128525	0.12949148	0.207643938
0.211175311	8.626107406	5.760057881	34.58980201	0.914183903	0.224192999	0.063694585
0.198529286	8.90682232	6.128828234	35.25813828	0.912825155	0.203757493	0.133979074
0.131735403	9.663325982	9.516814081	93.77372459	0.909496104	0.123476316	0.217227762
0.186892443	8.929723658	6.172096026	31.84330627	0.90907515	0.185103698	0.183170319
0.092107295	8.840038581	11.20378773	86.16844592	0.881952708	0.090291899	0.064951325
0.183496023	8.560890301	5.885303153	26.75260717	0.909410944	0.179509352	0.174881586
0.147875272	8.746223273	7.413040578	42.38775872	0.886479192	0.141325075	0.183999963
0.206205167	8.586262345	5.226707522	22.89699408	0.913062274	0.2034732	0.156608521
0.191533439	8.641800548	5.467395626	23.4309415	0.907714457	0.183316707	0.295718474
0.144257918	9.293578159	7.590888602	41.06216322	0.895275318	0.138146349	0.271118466
0.258473362	8.045209476	4.666158103	26.66236052	0.925668391	0.286550321	0.233238445
0.167944937	9.18295819	6.24585283	27.96023039	0.899657027	0.160305148	0.288780645
0.259145788	8.215990894	4.076602723	16.65642008	0.929438789	0.26889581	0.32577527
0.147712425	9.2893977	7.29099213	38.1227447	0.882196533	0.143544523	0.164649307
0.129009594	6.699145858	9.727792419	83.4985189	0.864895335	0.13152488	0.205842781
0.133992818	8.24802058	7.194912635	30.18609301	0.846220813	0.131459684	0.286732367
0.181472764	8.879972042	6.290593321	35.18766751	0.905075778	0.173718015	0.161615025
0.164352472	9.295644014	6.560507793	33.86837514	0.907758672	0.159593137	0.295680195
0.18874972	8.801762546	5.996926008	30.17500899	0.911161137	0.182692025	0.10469484
0.187924279	8.105358572	5.181407902	18.40209783	0.885434201	0.178866161	0.088745961
0.148650147	8.883827293	7.067798349	37.31023036	0.889014715	0.144898232	0.175317877
0.15740083	8.905177659	6.963106901	42.92138581	0.907491562	0.151602036	0.104257154
0.163485287	8.701917678	6.272790273	25.53233352	0.863313049	0.157738623	0.169231838
0.134137334	9.404191015	8.182099913	45.88103773	0.872774933	0.128377703	0.166585303
0.14828624	8.842529052	6.856092398	32.62673312	0.885841581	0.146314078	0.120561165
0.112261436	9.895784123	9.440752105	62.64333848	0.890694161	0.106397897	0.281501807
0.226225029	8.162002782	4.737816278	20.8151022	0.911624146	0.227131893	0.148596537
0.135384472	9.920608005	9.761582029	107.2887141	0.922120859	0.129118352	0.065434718
0.133282727	8.61819324	7.467062049	35.16844955	0.872509563	0.129539319	0.167224317
0.139763213	7.022839574	6.696307616	25.0091146	0.840495763	0.13345482	0.286847879
0.147536834	8.408158592	6.600800192	27.16623831	0.871084337	0.143071437	0.185493086
0.192655268	8.872570075	5.720703579	27.16662666	0.899577101	0.191467853	0.174078598
0.164315334	8.913158453	6.381991056	27.41797305	0.875299586	0.156158555	0.448171509
0.166916384	9.372555123	6.72333167	36.43769968	0.911044517	0.161172008	0.26788905
0.164992369	8.232723908	5.786022038	19.98007063	0.861534412	0.16278643	0.117176599
0.264218147	8.159607719	4.899895908	34.29153421	0.919840051	0.302375607	0.212052178
0.139973099	9.011559494	7.74603621	42.07285058	0.879897375	0.13107334	0.166703047
0.105228691	10.11666777	10.85975207	81.18615869	0.868958234	0.101458229	0.19053025
0.149844177	8.942384196	7.095569646	42.65899557	0.910521793	0.145773	0.099970678
0.203432915	8.2933314	5.865972279	31.23598989	0.890423567	0.209187619	0.140496846
0.191332244	8.363147568	8.120519097	117.817916	0.917806529	0.184157444	-0.063535308
0.154012128	9.299770987	7.227331725	39.10590771	0.901165056	0.146023375	0.202191887
0.141077035	9.787610107	7.861668951	50.79755672	0.912238027	0.13490934	0.233714018
0.213978082	8.50546977	5.618799075	33.34324426	0.924939914	0.218395941	0.062087129
0.30576969	7.637111953	3.74432929	19.86600906	0.941061776	0.330317393	0.231736272
0.169070557	7.69381597	6.215748721	24.55234288	0.839103065	0.153046463	0.288530229
0.121056648	9.631209544	10.04989974	86.48779268	0.905882106	0.11738273	0.150849601
0.118213171	8.612515192	8.717234472	46.83054406	0.864606307	0.108285859	0.289697569
0.18179936	9.133992849	6.214815545	30.77032041	0.900865858	0.177050819	0.267471981
0.140132971	8.381196054	7.111245029	29.27536151	0.854488078	0.132872718	0.117370841
0.137078304	9.388784195	8.021454422	50.89969749	0.904037066	0.130036616	0.157465516
0.184296322	9.184604693	6.839795484	43.32204	0.895794509	0.184074157	0.220488611
0.176987186	8.765194777	6.064226431	25.87016671	0.883246523	0.170658304	0.248976399
0.207007856	8.937105385	6.556603366	56.18849458	0.922959265	0.224266475	0.126286005
0.237513933	8.509968767	5.029520292	27.54603892	0.928143779	0.251732979	0.201838861
0.204301286	8.785857485	5.55481345	27.74777993	0.92289772	0.199747146	0.137431488
0.156291082	8.808766018	6.380822348	28.36543244	0.877821353	0.155844219	0.178256617
0.145981208	8.797456161	6.702742412	29.8437244	0.892942651	0.14146733	0.139192356
0.113988347	9.021774229	9.088998622	57.47201511	0.884612344	0.114271194	0.340819422
0.134996425	9.504317634	7.952583071	44.48910992	0.87927777	0.126437427	0.239038684
0.152369895	9.44580178	7.18212627	36.75237878	0.903966985	0.144509753	0.254067459
0.164517627	8.316155483	6.484285483	33.39003798	0.900244323	0.15914066	0.186707907
0.122694342	9.265608207	8.06867391	41.97910809	0.882552982	0.118964629	0.157722903
0.118227007	9.834553099	9.024174643	57.32920505	0.908574702	0.11192413	0.197651163
0.12249688	7.216981817	7.878125645	33.88748205	0.850674439	0.116747671	0.216220562
0.171315681	9.374046109	7.375151091	50.18431729	0.892163316	0.163985525	0.179124299
0.16392159	9.499516971	6.705246545	36.73432736	0.917858124	0.156937008	0.302120157
0.148803584	8.798808333	7.268781684	42.61376096	0.909720254	0.143111003	0.193300159
0.116809013	9.574525155	8.33275513	44.24795105	0.882580195	0.113422812	0.277398315
0.249578048	6.689989192	3.976741443	14.9407588	0.890145593	0.254875165	0.113829912
0.126676484	6.651999838	8.65213014	58.01987575	0.869474742	0.123371211	0.236882412
0.227090712	8.661082636	5.186560761	26.5909094	0.918665874	0.225479593	0.253200995
0.120966578	9.132478539	8.69171438	50.46477599	0.875476785	0.116832313	0.363019331
0.137728374	9.11888963	7.783195927	41.24714999	0.889574104	0.130239778	0.155796973
0.171012698	9.539139459	7.083991045	46.90791487	0.920502508	0.1695918	0.334921028
0.221704703	8.511214883	4.837823883	20.3820066	0.917467051	0.219869268	0.21378386
0.065639178	8.266027589	16.12315912	156.1505742	0.837117505	0.06363268	0.149847243
0.137605045	9.484436702	8.412934868	56.9744308	0.90601934	0.131654918	0.402090085
0.132141808	7.068433232	8.0350298	38.18831653	0.825854025	0.118193191	0.3061604
0.134234784	8.296948543	8.403708951	78.61038876	0.908547101	0.12681239	0.127158044
0.158277293	8.787379094	8.096163356	65.14870707	0.902569138	0.153933478	0.158968229

wavelet.LHL_glcm_Autocorrelation	wavelet.LHL_glcm_SumEntropy	wavelet.LHL_glcm_AverageIntensity	wavelet.LHL_glcm_Energy	wavelet.LHL_glcm_SumSquares	wavelet.LHL_glcm_ClusterProminence	wavelet.LHL_glcm_SumAverage
938.1785438	5.622240769	31.10487262	0.002716602	74.91921372	116308.2133	60.82405807
467.692891	5.218681528	21.72461268	0.00521282	37.56654567	32622.65178	42.82695736
547.0451473	5.324714995	23.91287377	0.003690845	60.26984036	69721.77796	46.50917392
1622.916687	5.881960717	41.05078658	0.001548517	107.1612979	261695.498	80.23482925
590.645311	5.2255192	24.47355451	0.004527028	44.9657599	41906.14323	48.13882883
347.6427396	5.204411825	18.96978361	0.003258822	42.16018408	28035.27166	37.03845965
923.1695628	5.229292978	30.88399636	0.004167361	48.47524929	41835.9187	60.57352037
1415.729258	5.958573185	38.07302155	0.001595824	105.2733388	200003.0946	74.46600913
1865.46802	5.750795353	43.81041671	0.001809504	82.20375666	190605.2574	86.02421413
1230.158221	5.545221988	35.35233527	0.002424999	54.78143595	84296.48828	69.68857217
692.589808	5.414083538	26.57443791	0.002883614	51.03178329	49403.43743	52.17912747
1313.988595	5.902262787	36.44591472	0.001795698	90.60579249	223024.5218	71.45724462
2844.951899	6.036343328	53.56338526	0.001783838	140.7564405	903318.5763	105.5343646
321.8269912	5.017258302	18.14786666	0.003900809	28.2108848	14631.60529	35.58692799
3381.968333	5.601936598	58.90718906	0.002333998	78.34645386	120065.4035	116.1494452
2042.018629	4.854483717	45.52690573	0.015369038	35.44711481	54973.92016	90.25049605
817.0575329	5.479546428	28.99920191	0.002656337	63.29580885	74576.15786	56.83401853
1609.030342	5.94725461	40.23432406	0.001953658	95.07615544	326855.6927	79.18409537
427.3299764	5.444446706	20.95300776	0.002856979	57.33355285	51440.22557	40.7964386
775.3830711	5.076962284	28.24214438	0.004926153	36.51111881	26607.70308	55.6204765
782.452169	5.231957343	28.32561434	0.004869019	42.97224742	45159.59436	55.74351387
1582.64664	5.943670424	40.36289296	0.00172617	122.1491106	425416.2562	78.89422862
825.9142223	5.30314696	29.06695145	0.003581804	43.36152009	41777.11211	57.2059957
2291.302547	5.719553748	48.93265135	0.002533467	110.246047	271464.3847	95.58635585
497.4035471	5.155972556	22.58297168	0.003840913	38.65963254	35648.23136	44.30162638
751.4892314	5.464488884	27.78982265	0.002918007	59.77589263	72326.6298	54.43639896
520.5928075	5.033861926	23.05879946	0.004373301	30.24237044	19220.345	45.43483918
691.1086425	5.274736176	26.4481209	0.003520513	38.07986658	36290.87357	52.15669386
645.6166106	5.619773348	25.68534326	0.002196816	69.06531764	122045.6893	50.11051599
401.3932573	4.933601266	20.14965739	0.011749771	32.58793151	47849.60838	39.69554784
736.9257499	5.457699168	27.33665203	0.002441834	47.33514474	51914.22054	53.78920302
465.9924927	4.98744547	21.63717643	0.00650501	25.45971041	21038.44326	42.80153318
591.4440352	5.477305175	24.7126208	0.002175421	54.68573774	55112.45146	48.28244182
1045.251993	4.988731808	32.75932628	0.010689932	106.6504792	291382.8589	63.96645524
326.8758126	5.432286828	18.02703344	0.003762003	57.71384122	56173.14335	35.24519459
692.9804391	5.291915811	26.69237974	0.003156278	45.42761239	52195.6734	52.37836798
717.8614296	5.567455038	27.00274001	0.002223139	54.87016742	67630.94209	52.99115943
1132.017773	5.129720513	34.0741648	0.003989979	37.22863194	31289.5969	67.18330316
345.3172349	4.838802261	18.89226832	0.004731905	24.03588686	10296.56547	37.05756148
681.7147867	5.413678115	26.53106545	0.00291726	53.41611184	52986.85279	51.87310092
932.4591606	5.329294834	31.04316517	0.003003487	51.50902195	60615.20593	60.90935713
431.9609436	5.221535215	21.01352362	0.002978014	38.18385946	23747.87892	41.25483936
536.401663	5.606723605	23.60581952	0.001984648	69.35868703	90805.27296	45.83971641
504.0565095	5.27936358	22.8321492	0.002780237	45.64398823	34340.82823	44.66940817
1448.393684	5.997370061	38.49122641	0.001319293	105.2318329	241641.6171	75.32927419
330.5750401	4.867671171	18.35844986	0.006079121	25.28954448	17065.75151	36.15705398
4052.789149	5.815228733	64.54939213	0.001736369	110.2402867	369145.4612	127.2138085
453.6714925	5.36810548	21.65621607	0.003075829	56.03911514	49373.40947	42.18549847
363.649396	4.956647437	19.04805112	0.008925379	50.41060741	47621.6135	37.36903596
615.6380946	5.241718015	25.0315455	0.003488498	43.16986014	30914.46304	49.30547715
606.2029558	5.206976485	24.89104071	0.003196346	37.28167623	24732.60271	48.99875736
607.6766255	5.615464835	24.49364816	0.002503528	60.82659064	69362.7299	48.16974546
784.9857897	5.55455005	28.30280271	0.00233626	56.05051247	81267.29669	55.50514368
276.9292984	4.990229287	16.9234706	0.004052728	31.54576017	12894.29034	33.07959999
609.088494	4.947895602	24.90701526	0.014760424	38.01819691	43396.3988	49.05228099
877.7058924	5.504725912	30.08759699	0.002373876	60.18000786	72297.59326	58.92601883
1472.970738	6.047876178	39.10951753	0.001161765	126.3791068	302867.3839	76.14619678
749.399141	5.352850028	27.89900687	0.002750496	51.20104151	53757.13431	54.58324784
652.2665327	5.115048487	25.83581742	0.005769969	37.78350971	30735.73447	50.87296879
2381.931786	5.252997978	49.59961123	0.005253042	89.36017361	358560.4365	97.63717193
736.4408615	5.5191075	27.51232265	0.0021897	57.92016725	78775.41537	53.85242803
1814.587812	5.73703447	43.1110842	0.001697539	73.88410899	125285.638	84.80133944
1102.776494	5.00329363	33.62637941	0.005584264	34.79839252	28789.32269	66.36039102
1452.354755	4.703241493	38.27262371	0.014663193	21.67981847	15158.21674	76.08913929
250.5008673	5.17712333	15.68617044	0.005446016	47.02517431	28774.02464	30.83847124
1897.13261	5.883133328	44.34385648	0.001742906	112.1862474	333900.2917	86.73168229
759.1235748	5.687906556	27.77474029	0.002905874	87.26302141	119389.9632	54.19639355
596.6542417	5.427959671	24.64544752	0.002923467	48.39110821	54348.15807	48.32845169
424.0418292	5.224767344	20.93806189	0.00355533	45.05398915	29350.64653	40.94229607
1103.839488	5.592002429	33.80709578	0.002010049	68.71376022	105685.6283	66.14229794
596.1376236	5.496073476	24.72110802	0.003329073	59.16574795	82140.43455	48.32070099
411.0186823	5.317032906	20.43501277	0.002982072	41.6799766	33114.37599	40.04430347
1627.41304	5.348046801	40.78342266	0.00623135	58.52243232	98986.56329	80.49620666
731.6572092	5.083422209	27.28404339	0.007487552	33.3099892	29541.01647	53.85328365
990.2946132	5.074163433	31.83335318	0.00437799	34.25686075	29389.33138	62.79719342
445.304251	5.291185404	21.34594354	0.00292889	41.45581082	31235.76405	41.85579104
624.6707235	5.258200083	25.41294148	0.002944331	43.60339253	38392.09332	49.75394369
1383.62301	5.930330989	37.50661845	0.002275758	107.1949055	266409.2697	73.42106519
643.2026244	5.690256811	25.68786773	0.001837712	71.57680807	96065.92451	50.06635664
840.3682251	5.591084696	29.32274297	0.00203055	59.71736448	77920.23281	57.46802213
780.6978694	5.24180859	28.25269643	0.003880311	47.56439545	50227.62256	55.58351785
879.1438521	5.555169822	30.15140792	0.002073336	63.07046735	73769.50207	58.9848998
1521.976904	5.812789078	39.66277906	0.001452731	85.61891554	166918.2583	77.6073159
332.9871848	5.123640751	18.43060164	0.007539273	62.66097859	68758.76413	35.75818577
783.3637114	5.551037131	28.39222912	0.00260878	64.93303527	92841.95807	55.57901219
1022.389248	5.620123799	32.27435919	0.002051068	59.60248386	92772.81071	63.4023739
1324.605432	5.475623465	36.86039082	0.002873915	60.39447562	87891.66484	72.45649536
925.9805836	5.796478513	30.79303226	0.001646207	77.98369282	118680.8917	60.16068695
344.9718364	4.378937615	18.69073014	0.012459286	17.90776581	7060.02977	37.05955429
1215.683996	4.962236829	35.10521259	0.01099358	87.70718086	187568.2163	69.04882037
1219.24241	5.170614931	35.15424774	0.005231201	35.80600882	36181.35371	69.57777843
771.9470175	5.907411097	27.8561681	0.002153944	100.3674438	209821.988	54.28313492
1002.531113	5.507935507	32.15156781	0.002256465	60.14122638	65007.42584	63.03824026
1279.198778	5.735879714	36.04005962	0.002377452	75.78515499	189751.5931	70.8308261
852.0804989	5.027216354	29.41227875	0.004806658	27.86238988	19618.74939	58.17619245
1536.955908	6.015996416	40.39324557	0.003551524	252.781999	879615.7841	77.51983459
1408.278282	6.019660619	37.79167743	0.001837343	105.4288716	330276.7815	73.91685718
358.9119601	5.192417206	18.69236334	0.008026933	75.22583002	89420.35094	36.66182876
1977.208976	5.462505682	45.12257657	0.003796492	84.4414623	252295.0807	88.67256067
1322.270334	5.539373072	36.90854587	0.003205674	80.17563492	176408.4091	72.39625009

wavelet.LHL_glcm_Imc2	wavelet.LHL_glcm_Imc1	wavelet.LHL_glcm_DifferenceAverage	wavelet.LHL_glcm_Id	wavelet.LHL_glcm_ClusterTendency	wavelet.LHL_firstorder_InterquartileRange	wavelet.LHL_firstorder_Skewness
0.954916778	-0.241089126	8.197262681	0.217458061	178.0742078	237.5454468	-0.101217953
0.794796822	-0.11183813	5.31083929	0.3130268	93.40469159	146.9576544	0.274671316
0.966339383	-0.279756824	7.991428617	0.206642629	131.3450918	226.7084488	0.231277714
0.877594532	-0.140132763	9.891728952	0.200423549	241.0127106	262.654284	0.314577817
0.961284312	-0.276219732	6.369126305	0.244492335	115.7821405	204.0656001	-0.614705149
0.897929106	-0.176077472	6.874256246	0.227757599	95.22294507	220.1903898	0.050842843
0.965663105	-0.284973228	7.46757817	0.213526967	112.9088184	205.9904121	-0.563920257
0.944473857	-0.209950163	9.513268289	0.185912036	271.5875627	343.8289373	-0.161455001
0.87175108	-0.140528985	8.553907804	0.209506996	199.3585401	243.6247891	-0.355010698
0.783588732	-0.10015248	6.548484843	0.245142723	142.4046863	199.4261	0.061638865
0.906261652	-0.178686554	6.627397326	0.243734223	126.86701	222.0595172	-0.034226771
0.912240054	-0.172677847	7.782815959	0.217671663	254.0821921	278.6389131	0.423667409
0.938638525	-0.201883643	8.362758452	0.221483809	396.3001788	272.6589847	1.024029926
0.779916859	-0.108945313	5.172320709	0.2764045	67.01801757	178.93927	-0.054577905
0.929989213	-0.20080178	8.776135973	0.204679627	175.0512816	266.7534743	-0.144532375
0.768893567	-0.112187158	4.722678145	0.371659857	83.03378856	74.43892508	-0.068679057
0.935035284	-0.212356548	7.91308357	0.22086904	148.5839001	260.9038984	-0.546983936
0.904026041	-0.166443577	7.259542438	0.243229592	270.5836033	242.0835814	0.55536536
0.939558522	-0.220959771	7.591959229	0.216986422	139.9425167	241.1142165	-0.103414661
0.78600903	-0.107777739	5.760057881	0.304956671	77.01861916	150.8037477	0.126398636
0.707575248	-0.080092618	6.128828234	0.285668883	96.81679925	151.5138369	0.245584485
0.942430801	-0.205041553	9.516814081	0.207265851	296.2083189	282.0591206	0.422708734
0.770589143	-0.102480441	6.172096026	0.270618607	102.8887491	174.8908627	0.007597369
0.985291439	-0.332566214	11.20378773	0.169242673	243.9322068	328.8924409	-0.563192869
0.828645592	-0.127691674	5.885303153	0.267979034	89.0105274	174.6615956	0.577493925
0.936946715	-0.214909573	7.413040578	0.228584543	140.8796348	224.5504422	0.113726164
0.644167139	-0.061620514	5.226707522	0.291591104	68.77798387	140.9555739	0.206011198
0.825912023	-0.125011166	5.467395626	0.27410823	98.02614396	187.6788149	0.062519578
0.873238966	-0.145445876	7.590888602	0.224687185	171.7930014	250.6930521	0.495729884
0.812111891	-0.129337641	4.666158103	0.360273645	79.1775271	107.2631442	0.615472369
0.746283969	-0.087174465	6.24585283	0.250294562	122.4179911	201.0384594	-0.04616821
0.676191279	-0.075885889	4.076602723	0.351163092	65.76564271	115.2902673	0.665025204
0.798125668	-0.105038424	7.29099213	0.230882772	127.4627648	231.5186514	0.085081056
0.997187445	-0.561195208	9.727792419	0.212164609	269.2427383	216.0198679	-0.979652276
0.97169115	-0.298973104	7.194912635	0.220639816	148.1114735	270.5604449	-0.144818287
0.814007709	-0.119948218	6.290593321	0.262041095	105.3327696	172.7152307	0.095435217
0.78734081	-0.100585314	6.560507793	0.248346388	141.0677971	216.1806194	0.116792075
0.704054568	-0.077781685	5.996926008	0.269889094	81.81720862	163.0918898	-0.130206355
0.778572258	-0.108962266	5.181407902	0.272405767	53.68168101	152.1974732	-0.280682695
0.887863959	-0.161979916	7.067798349	0.233851002	124.909126	201.7735993	0.14592382
0.846557468	-0.132866673	6.963106901	0.24090112	112.1703184	184.1774363	0.187997877
0.831288693	-0.12895409	6.272790273	0.247931673	92.50736741	215.8413356	-0.271305043
0.869248799	-0.143618147	8.182099913	0.213927414	161.9546707	239.8018343	0.002033707
0.854519044	-0.138665397	6.856092398	0.235869654	101.732855	208.0340453	0.174580159
0.895444292	-0.15282169	9.440752105	0.190177822	274.4123744	329.2107075	-0.395950727
0.732976945	-0.092064872	4.737816278	0.314232275	58.79602501	119.8920122	0.306702656
0.84873763	-0.123489925	9.761582029	0.210983147	235.1968348	243.1327935	-0.161819555
0.932879508	-0.211385527	7.467062049	0.217902811	127.0138963	238.4310689	0.390508158
0.99151377	-0.453201224	6.696307616	0.225849676	129.3245156	223.9358364	-0.469958273
0.922176311	-0.203191949	6.600800192	0.234379649	103.1146923	202.267469	-0.30916515
0.711527081	-0.080231267	5.720703579	0.278968421	85.26825553	179.1342786	0.090669353
0.92233045	-0.193683961	6.381991056	0.245885261	179.6784091	293.6187399	-0.15069071
0.756139989	-0.088580584	6.72333167	0.248530485	142.1171342	208.5384202	0.099114955
0.849782073	-0.144150172	5.786022038	0.254767496	67.51097823	181.4690551	0.095952362
0.797751477	-0.119873357	4.899895908	0.372174777	90.2406706	82.08197574	0.313704392
0.907969057	-0.176937737	7.74603621	0.217923984	142.5477892	234.9111679	-0.377266161
0.902172531	-0.158448267	10.85975207	0.18077273	303.9858178	354.1280514	-0.33655002
0.852429118	-0.13616855	7.095569646	0.234909984	112.5243915	197.1257998	-0.032713214
0.890141257	-0.17473591	5.865972279	0.29158169	87.72552895	151.4900342	-0.404243917
0.955263026	-0.25741799	8.120519097	0.265905737	182.265915	153.277774	-1.05859432
0.817600197	-0.115771415	7.227331725	0.233335401	139.1367556	224.9052796	0.106640615
0.747387616	-0.082421147	7.861668951	0.221744091	181.3004887	242.0551517	0.160206485
0.75239432	-0.09500825	5.618799075	0.300926098	73.23743229	125.0034591	0.215230617
0.792433561	-0.124986501	3.74432929	0.400748937	54.32120013	91.77777308	-0.116355778
0.977313474	-0.336018098	6.215748721	0.244088529	116.1047426	237.2069943	-0.068063502
0.937508155	-0.199099929	10.04989974	0.198078061	258.7574425	270.2168064	0.040622668
0.983655101	-0.332699778	8.717234472	0.1934221	224.2650343	321.4397256	-0.166367649
0.758819909	-0.094503822	6.214815545	0.263992422	122.0622357	188.6491752	0.16832109
0.935683557	-0.222139436	7.111245029	0.222452515	102.9030938	233.2451989	-0.200769366
0.855401525	-0.131179122	8.021454422	0.216498701	157.4870942	230.2848736	0.221343664
0.817221227	-0.119754928	6.839795484	0.267302106	143.3625513	179.6987474	0.187458752
0.849338148	-0.139562169	6.064226431	0.259500708	105.0540676	203.8576942	0.080267434
0.81526135	-0.121854894	6.556603366	0.301910477	131.9841725	130.4961814	-0.159732037
0.695934528	-0.080799732	5.029520292	0.33080239	80.22650783	115.1297073	0.089079086
0.60162153	-0.051278739	5.55481345	0.286603489	76.95616138	142.7552206	0.120608597
0.835584415	-0.128570904	6.380822348	0.246414794	99.2777781	190.061959	0.026285629
0.844722114	-0.133297044	6.702742412	0.232397075	98.9656078	201.1652964	-0.007704022
0.978628707	-0.299173113	9.088998622	0.198286826	287.8754814	315.362728	-0.125919926
0.850624372	-0.128471686	7.952583071	0.213583176	176.2396036	258.2179939	0.198467849
0.775391813	-0.094472416	7.18212627	0.231579933	148.5340148	226.9510755	0.207611242
0.937497397	-0.226144422	6.484285483	0.24883841	109.8944206	189.7206302	0.059239119
0.875473817	-0.146600637	8.06867391	0.206684369	146.603926	246.7224965	-0.140824534
0.832276865	-0.113703449	9.024174643	0.19602287	205.1863745	269.1077681	-0.031428887
0.993758613	-0.467622458	7.878125645	0.204895852	151.7433671	239.5265783	-0.072352142
0.812378858	-0.112961422	7.375151091	0.248054987	152.1851376	193.9780303	0.208341548
0.725203864	-0.079020142	6.705246545	0.245559628	152.8493232	221.1523163	0.267628734
0.937652684	-0.216787326	7.268781684	0.231488349	146.4138887	233.9505097	-0.10137857
0.875182242	-0.141376464	8.33275513	0.200337546	199.6502624	272.5427509	-0.124779174
0.945467137	-0.286963089	3.976741443	0.342714691	37.88481839	101.0149662	-0.825975228
0.997893936	-0.588460234	8.65213014	0.207883746	229.7189028	277.0163514	-1.000810045
0.728839951	-0.087342686	5.186560761	0.308918205	90.30692873	143.6914865	-0.279290293
0.968575878	-0.265051217	8.69171438	0.201643372	269.3684672	327.2643767	0.311897727
0.896092167	-0.164314643	7.783195927	0.216841234	139.6347623	245.1474663	-0.092252539
0.818310275	-0.114607705	7.083991045	0.254152288	199.2313999	221.6320812	0.480919129
0.615133309	-0.058775424	4.837823883	0.307194406	68.31239077	135.7008021	0.060008688
0.999083121	-0.55161438	16.12315912	0.132175158	574.3724731	650.8617466	0.207404794
0.952146712	-0.222708281	8.412934868	0.216060521	300.1831436	309.8970564	-0.209882163
0.997096264	-0.536295131	8.0350298	0.204929312	197.4225375	261.0153049	-0.227657733
0.982538422	-0.336276375	8.403708951	0.213901513	197.0940984	225.709412	-0.614263258
0.95001038	-0.234053308	8.096163356	0.237016041	181.4203303	206.3461186	-0.250618016

wavelet.LHL_firstorder_Uniformity	wavelet.LHL_firstorder_MeanAbsoluteDeviation	wavelet.LHL_firstorder_Energy	wavelet.LHL_firstorder_RobustMeanAbsoluteDeviation	wavelet.LHL_firstorder_Median
0.038291777	163.0874275	33714137.26	105.540517	-14.80239348
0.061185754	105.6281688	43660642.9	64.34215446	-10.36607441
0.042738219	144.2089577	15992722.32	94.37445474	-34.04714826
0.03403044	182.0138972	163144989.6	112.1216663	0.537955366
0.050628099	126.279349	9502196.065	85.85618362	2.713722171
0.045260721	128.0120576	16598517.19	90.47000127	-22.22395189
0.046606028	129.8791125	10186257.96	88.20217668	54.41033212
0.02911343	199.5692546	61674996.33	142.8517532	-22.77475009
0.036973644	162.8273353	99784077.36	104.5286215	-0.338117377
0.044237499	135.7798508	105387769	85.16707583	-20.77835081
0.042655012	136.0113029	25006001.58	93.15321555	9.584475397
0.033967666	176.1121527	77818710.84	117.3240865	-39.89268572
0.033287644	199.1469539	164442025.3	118.3143608	-18.1750151
0.054843334	104.6144025	19335212.08	72.98034193	10.95624532
0.035787744	168.4371694	45382602.39	113.0471781	17.89262878
0.103142801	86.60027147	147713419.5	39.04738464	-3.131277057
0.038837941	156.2140655	26494268.07	109.6698565	7.354309213
0.036484705	168.3819681	123233746	104.6089222	-15.35259049
0.039867267	146.8833389	18950532.28	105.6806751	9.538126777
0.061458262	102.0518078	56374235.81	62.21615961	-0.614967356
0.059080015	113.3231504	135337686.5	66.84961975	-14.08186591
0.03194159	197.549166	126437648.6	120.4853523	-14.32643
0.052357214	118.9726968	71515665.19	74.56989444	-11.5303227
0.029343753	206.4428613	36138913.84	133.9970592	-4.424111744
0.051493637	116.7582006	29576162.62	74.8203268	-21.03291595
0.040698785	146.0730392	24996750.25	97.01412785	-9.969390402
0.061873166	97.39463824	60639360.23	61.00515749	0.001284861
0.049736327	117.2898708	35765424.2	77.28826361	-33.36113421
0.037338234	159.5684429	84326013.52	105.9079963	-97.0945244
0.082255557	90.82577412	47724104.45	49.77357936	-8.628525247
0.044953607	128.2126674	88967239.62	84.64816504	6.051900054
0.074850604	84.72958435	85428254.13	50.13169802	-7.06599465
0.041067831	139.8630248	65319008.44	95.11947272	-7.575428097
0.049001479	174.888332	6584491.001	94.73317221	-0.867107209
0.039029612	152.6500808	11368642.31	111.0312979	-10.27228417
0.051318498	118.14522	51988317.53	73.73419962	-9.50318261
0.041646653	139.2818666	99517419.4	93.16671623	-39.25764484
0.05632058	109.0041574	74471872.74	67.82117132	-3.827082147
0.062500627	94.23846885	12377174.55	63.87349616	15.30600931
0.044666444	134.0315157	32840441.11	86.48661999	-17.75139402
0.046989059	127.9067186	45400754.44	80.27279398	-23.77531973
0.046937434	123.1693696	22475336.09	87.49753845	-0.402648415
0.03783301	153.7918272	65814927.92	101.0536514	-1.663003634
0.045305413	128.615652	28321272.68	86.75208372	-17.05545235
0.028907103	200.416869	121664558.3	137.9120934	24.45532039
0.071651722	88.50250551	34415370.47	52.7056042	-10.77593238
0.036004879	179.119133	286526482.6	104.2262246	-7.529551563
0.041410578	144.3857868	19803143.22	100.7888609	-46.53382387
0.048785921	134.8903138	4104548.571	92.97203025	-26.82465649
0.047905137	124.1852384	12354809.27	85.40577943	-0.307081386
0.051967045	110.8624557	71266726.95	73.92994008	-7.181796318
0.036461387	158.2803397	30988360.41	117.1998486	-23.64106777
0.043456933	136.4942433	126905135.9	87.75486682	0.285146288
0.052476373	109.6724583	11519358.72	77.2480746	-34.38301043
0.105384128	87.85883211	124146410.4	41.9286507	1.738287275
0.040516877	144.6717184	31450106.75	95.9818757	-9.100317935
0.027263092	213.3472624	163541403.6	145.1922129	-8.4921045
0.046167447	128.6002847	37873841.59	83.73067438	2.148130966
0.058965041	110.0629297	18996505.55	67.18454883	-12.15507257
0.055683035	143.4398829	49715203.2	69.12264762	-17.31860994
0.041213444	141.3272535	72979907.94	92.93920272	-12.77637843
0.037420951	157.2764639	236914983.2	101.9912998	-7.688329331
0.06853154	97.10092913	60272643.65	56.59645813	3.761212609
0.094899	76.60299383	34101269.44	43.34621761	3.653054057
0.043895602	136.9982079	5790838.184	102.2998908	3.048168844
0.033915206	183.9106828	98766546.43	114.5444236	2.261709302
0.031745214	186.5053985	20813281.97	134.8050008	-22.46695076
0.047437243	125.7383692	89624470.9	81.09242262	-8.730352643
0.044340325	135.1333774	12489815.59	97.27628177	-5.266648691
0.039320535	150.7281717	83853353.41	97.13444334	-39.77361333
0.048194252	131.307232	98984887.98	79.94283691	4.785602369
0.04711052	122.0235497	27308854.89	83.82846911	-7.147234422
0.068456293	116.0603381	164227976.8	59.4511676	-2.99730432
0.075467819	93.41631565	125087052.1	52.42394205	-6.873999375
0.060960386	101.0149953	180179353.8	61.39482271	-0.6715739
0.049116147	119.7298919	26961509.74	79.32991525	-1.917799328
0.046985266	123.9862777	28853546.45	83.65331506	-8.180382098
0.03011556	195.7760868	39319948.74	133.5038957	-46.36666054
0.035039413	163.4346297	78057202.97	111.0835812	-36.75837256
0.039910288	145.1694754	102852323.4	96.07162865	-22.43045365
0.048603238	123.3500593	13913694.34	79.45486391	-12.62266962
0.038110284	151.7987828	51253112.56	102.2208933	6.873941368
0.034177675	169.7959243	138690762.6	112.662559	-15.91211883
0.047705113	143.7825039	4893055.58	96.8666513	31.62843049
0.04528745	140.1974518	124395533.9	85.59407354	-6.140920912
0.041537387	140.6794026	189019537.4	92.23606773	-2.64508588
0.03949551	150.5300033	31208011.61	99.52013309	-24.85959702
0.034957388	164.977221	68440327.59	113.0239264	1.605072645
0.085012989	75.01028112	2014138.754	44.2731556	6.343989008
0.0430839	187.6246521	6327335.31	119.079108	38.91649915
0.061707369	103.9031691	83981039.7	62.37302818	3.956907291
0.030282724	197.1065261	42953335.34	137.0283522	-32.76081314
0.038453437	149.3230705	37362965.3	101.6249956	-1.95027033
0.040945116	151.3407213	175623570.8	94.54105229	-13.72455256
0.065447043	93.0146539	94749022.13	57.53229875	-10.42314625
0.020950179	336.8255922	41899731.42	251.8586401	-112.9150381
0.031059286	193.4099101	80213668.59	126.9116076	-23.89901443
0.040782603	158.3095725	4747306.204	109.9724446	1.424358257
0.040371482	164.4206595	20572198.6	99.77653608	25.98571125
0.041325207	157.6513996	40521609.34	92.28785315	-13.02177673

wavelet.LHL_firstorder_TotalEnergy	wavelet.LHL_firstorder_Maximum	wavelet.LHL_firstorder_RootMeanSquared	wavelet.LHL_firstorder_90Percentile	wavelet.LHL_firstorder_Minimum	wavelet.LHL_firstorder_Entropy
910281706.1	691.94639	218.526281	228.0477522	-750.6318331	5.045713608
1178837358	571.9017747	144.9862492	189.5974895	-500.3108491	4.474983689
431803502.5	710.3243996	191.9624718	206.1512593	-594.0504074	4.867638531
4404914720	1146.510299	248.8264597	324.6413784	-953.8176228	5.271147254
256559293.8	418.8272572	162.6914648	159.718928	-615.4628374	4.603207173
448159964.1	531.5466669	160.6679157	192.3652712	-451.6666737	4.68065881
275028964.8	498.3629187	173.8570814	253.6493441	-697.276917	4.669182945
1665224901	655.3428843	248.0965388	295.0706667	-927.9699089	5.287974039
2694170089	796.5959905	219.0803259	258.6520691	-1063.716721	5.098436378
2845469763	777.9721398	184.4396964	208.9194012	-866.017111	4.859319185
675162042.5	550.1564712	173.8879039	237.6456707	-612.7512608	4.794070316
2101105193	778.0460352	229.1489725	289.0132126	-895.644921	5.163181404
4439934684	1395.108526	281.5801478	371.1107549	-1273.219393	5.319952122
522050726.3	436.3413821	131.5084225	178.4121944	-423.3465872	4.423850104
1225330264	658.321989	223.1955387	346.2238876	-1395.749178	5.066702414
3988262327	1008.029998	145.8180636	139.1202895	-1121.242531	4.115127969
715345237.8	417.5450608	194.8266001	213.2780875	-723.8630221	4.911936428
3327311143	1024.181167	229.8796631	290.5934252	-959.3721988	5.14732475
511664371.4	484.6162831	180.4468122	235.9280072	-487.8460855	4.833953965
1522104367	700.3946626	141.8677822	178.1683381	-662.6149855	4.456371225
3654117537	759.9092033	160.9562296	181.4992782	-693.2742429	4.593828129
3413816513	1211.354816	271.1277574	350.885458	-936.5234572	5.367249565
1930922960	688.2800955	161.8228111	199.8688733	-708.8447101	4.659048078
975750673.7	711.4302643	274.1037705	315.4272337	-1177.532354	5.363902544
798556390.7	810.1694304	156.7319977	197.842549	-547.0876768	4.605717179
674912256.6	639.6974455	189.7850682	221.1115314	-651.4932898	4.897836682
1637262726	717.6226516	132.2705094	173.0648324	-539.2810067	4.384386564
965666453.3	558.4891763	157.9823592	159.9176286	-652.0208642	4.626795663
2276802365	779.0609938	218.5789976	205.2190907	-678.9136383	5.03234265
1288550820	824.3165426	137.0191032	136.3740804	-486.1090726	4.231905763
2402115470	627.7639599	167.2899279	217.1647655	-636.582208	4.764693954
2306562861	705.6001413	120.025471	142.1467879	-518.9780564	4.188341767
1763613228	633.483756	179.2466726	229.5707716	-578.3113399	4.864046284
177781257	525.2653014	251.6195809	210.9234588	-815.8484549	4.685419108
306953342.4	427.9747824	185.0483681	228.2302176	-431.8352478	4.831364765
1403684573	711.8107002	160.4269019	179.4792382	-634.9155668	4.650795217
2686970324	791.7626182	185.8888364	180.1305183	-682.5626788	4.871945321
2010740564	588.8942913	149.0097234	187.4212016	-808.5296754	4.546455977
334183712.9	470.1958365	121.7495936	144.4462874	-448.3843473	4.262724558
886691909.9	639.7019901	176.9362208	226.9666881	-630.4244413	4.808868266
1225820370	784.515371	175.5022922	180.1971347	-765.9256634	4.760809838
606834074.4	367.6008699	153.5700929	172.6918124	-514.2663692	4.616311613
1777003054	679.4594079	200.2053269	240.2417494	-571.9099723	5.011411357
764674362.2	626.2783319	166.224276	189.8779329	-559.9037111	4.725849997
3284943075	841.1678257	254.5272282	322.5260967	-904.0979774	5.343807739
929215002.6	660.1088828	124.2849755	142.19085	-437.9029673	4.243501726
7736215030	1054.808374	259.1623794	286.7313652	-1564.723301	5.2611592
534684866.8	609.3787274	184.3031622	210.8799579	-530.212523	4.830101849
110822811.4	298.8569827	175.0170263	150.4518435	-488.0468711	4.547751224
333579850.3	392.4878045	157.6666457	174.9899978	-608.8343182	4.613696264
1924201628	560.2297167	144.2701651	179.9538705	-584.9489745	4.556121116
836685730.9	391.8260349	193.5737861	230.2135569	-602.4200907	4.91756666
3426438670	915.6833086	182.2430942	229.2740521	-670.3152405	4.868252505
311022685.4	382.2609562	139.6112485	150.7450719	-414.8034357	4.449077886
3351953081	674.7663982	137.9568896	180.9193526	-563.3771375	4.080440015
849152882.4	532.636736	189.1544768	204.5135385	-733.0780739	4.891243562
4415617897	649.5468007	273.0829241	319.2357192	-973.9947866	5.442256037
1022593723	977.0318231	171.1474437	192.4628012	-650.3104621	4.741029097
512905649.9	422.2522723	152.7652788	156.2886116	-633.2356766	4.519213141
1342310486	742.7330202	237.5508864	226.8961922	-1203.854167	4.795291424
1970457514	830.1202335	185.5821195	227.74662	-662.7339779	4.902121867
6396704547	830.2700553	207.9626055	266.8222331	-1049.728408	5.063369382
1627361379	776.3634252	139.6628437	184.9153588	-797.2639274	4.363538631
920734274.9	437.306089	111.7440539	154.9832743	-912.4454023	3.987319633
156352631	341.8055174	163.3581655	211.1064962	-351.9150161	4.635439547
2666696754	1136.800997	252.1849739	316.7015716	-1062.067485	5.273757598
561958613.1	562.9538384	229.8381795	267.2511729	-694.0625269	5.149731352
2419860714	679.255661	167.4333568	210.0186059	-597.7269672	4.740792992
337225020.8	410.1061968	167.3441688	193.7923261	-519.6051958	4.696278647
2264040542	887.1592804	204.1992687	203.9523647	-825.1207274	4.994160045
2672591975	727.3407282	180.4461649	246.8287951	-570.259767	4.797856212
737339082.1	530.610738	155.5266344	192.5721562	-481.1631199	4.655700391
4434155375	768.4958748	178.2634547	184.4530632	-982.1149035	4.588122498
3377350406	803.6431097	136.4742873	149.2005646	-667.2811278	4.312906239
4864842552	769.7176361	140.0977537	164.6946625	-758.2121523	4.451857168
727960763.1	505.5705441	155.4318465	181.439203	-506.6021451	4.640580957
779045754.2	652.8546233	160.7211436	183.4316791	-618.3657247	4.685377811
1061638616	625.1240431	253.8876373	280.6315645	-930.3547554	5.285448592
2107544480	665.7998587	210.3571132	247.0948896	-648.3802294	5.075340263
2777012731	842.0254886	190.1032779	223.7892096	-724.7352167	4.937322211
375669747.2	628.1862426	166.1521483	167.8599434	-687.9640253	4.668923423
1383834039	658.5466576	195.4995371	251.0359796	-704.9107954	4.981795037
3744650590	1108.405591	222.8774145	260.1397677	-965.1055742	5.161733823
132112500.7	575.2257361	183.698696	219.376692	-424.9931499	4.673906689
3358679416	677.499239	191.5307456	247.2472804	-651.4479864	4.887757665
5103527509	967.1903105	185.7896978	238.3480514	-763.3319394	4.90285022
842616313.4	764.7754619	200.282614	205.2444044	-901.0329482	4.955557961
1847888845	695.872241	209.9955933	245.1482572	-725.05291	5.083915876
54381746.35	265.299122	104.9105462	113.6292863	-434.5595935	3.909727873
170838053.4	400.7321352	245.4798083	276.9133301	-863.8016718	4.811971357
2267488072	499.2061415	145.3523498	187.7167113	-839.4491617	4.473695917
1159740054	700.3711006	246.6585055	333.3395335	-655.3832032	5.270624289
1008800063	664.0928276	191.2032282	250.4763542	-761.0451891	4.952134929
4741836412	1013.118975	207.219083	252.8464101	-865.5893002	5.008422318
2558223597	518.0668675	127.7464536	144.9372816	-721.7149724	4.326887351
1131292748	808.9054399	406.9540351	520.5721489	-1008.493984	5.78163382
2165769052	917.4836254	256.8368898	279.0122626	-948.9123223	5.320263201
128177267.5	360.1933992	196.4586099	240.5387747	-465.3422322	4.775921575
555449362.3	814.8060124	232.981131	259.4293667	-1057.342599	4.988800371
1094083452	785.0286159	224.0817953	245.8670223	-895.2203105	5.013511498

wavelet.LHL_firstorder_StandardDeviation	wavelet.LHL_firstorder_Range	wavelet.LHL_firstorder_Variance	wavelet.LHL_firstorder_10Percentile	wavelet.LHL_firstorder_Kurtosis	wavelet.LHL_firstorder_Mean
217.0466608	1442.578223	47109.25296	-294.9421218	3.889744918	-25.3866607
144.9835739	1072.212624	21020.2367	-172.2361667	3.988510283	-0.88077722
189.2870644	1304.374807	35829.59276	-256.0306006	3.843471222	-31.9374048
248.4319625	2100.327922	61718.43997	-281.4729554	4.351588527	14.00596658
160.6024027	1034.290095	25793.13175	-229.3525162	3.683882941	-25.98809279
159.4179451	983.2133405	25414.08124	-226.2224707	2.806862707	-20.00244756
168.0950424	1195.639836	28255.94327	-165.7334106	4.262682521	44.38852854
246.5848582	1583.312793	60804.09232	-339.7492196	2.830252592	-27.34593628
218.9930467	1860.312711	47957.9545	-265.8223955	4.839498735	-6.183421871
183.577699	1643.989251	33700.77158	-241.8741379	4.301250089	-17.81095271
173.3412031	1162.907732	30047.1727	-199.3275733	3.206341302	13.77789574
228.1223616	1673.690956	52039.81188	-293.8809406	3.397159889	-21.66655713
280.5231083	2668.327919	78693.21431	-246.6968199	5.994055972	24.37550634
131.1528677	859.6879693	17201.0747	-154.1324449	3.100567169	9.663875487
220.091067	2054.071167	48440.07779	-215.3128269	5.134938548	37.096775
145.7957959	2129.272529	21256.4141	-144.923263	9.997237325	-2.548247182
193.980577	1141.408083	37628.46427	-283.4717612	3.144973032	-18.13669865
229.879172	1983.553366	52844.43373	-265.6827116	4.769045619	-0.475186185
180.4454063	972.4623686	32560.54467	-248.5290264	2.552153672	-0.712284157
141.77169	1363.009648	20099.21209	-156.3542203	4.858263788	5.220683107
160.2525567	1453.183446	25680.88192	-201.6748654	4.768623421	-15.03415843
270.9368127	2147.878273	73406.75647	-285.5659824	4.644712404	10.17370879
161.1279101	1397.124806	25962.2034	-220.374193	3.763590497	-14.9806135
273.6027956	1888.962618	74858.48976	-352.2792623	4.148339244	-16.56463843
156.5131459	1357.257107	24496.36483	-192.0262407	4.633003539	-8.279751776
189.666806	1291.190735	35973.49729	-240.5357688	3.674073505	-6.698867599
132.2083714	1256.903658	17479.05348	-154.7204516	4.417280433	4.053909648
154.1602661	1210.510041	23765.38766	-225.5863028	3.927230596	-34.54038452
205.9974126	1457.974632	42434.93401	-312.5427147	3.528391846	-73.08792097
136.2884641	1310.425615	18574.54544	-168.1989297	7.028394817	-14.13114269
167.162084	1264.346168	27943.16233	-198.9585447	3.589495826	6.538933182
120.0234288	1224.578198	14405.62346	-136.7233911	5.714133847	-0.700159278
179.1779808	1211.795096	32104.7488	-227.7474174	3.290124633	-4.96193681
250.2309187	1341.113756	62615.51268	-407.9464754	4.650501807	-26.39887873
184.6165204	859.8100302	34083.25961	-256.038073	2.389564299	-12.63482953
160.235972	1346.726267	25675.56671	-193.9127244	4.579251443	-7.824585856
180.8663635	1474.325297	32712.64145	-276.0705656	3.631468869	-42.91873796
149.0053808	1397.423967	22202.6035	-172.264266	4.64548057	1.13761097
121.5907242	918.5801839	14784.30421	-147.1004244	3.975353697	6.217662564
176.6466867	1270.126431	31204.05191	-229.5348794	3.584459532	-10.11801912
173.9309033	1550.441034	30251.95912	-225.0567553	4.899316203	-23.43278575
153.1478251	881.8672391	23454.25634	-226.0369867	2.777888427	-11.38055795
199.9951159	1251.36938	39998.04638	-275.0150867	3.279775647	-9.172051671
165.0719898	1186.182043	27248.76182	-229.4859068	3.448893612	-19.53837527
254.4440469	1745.265803	64741.77302	-337.6564502	3.158857049	6.506677777
124.1184911	1098.01185	15405.39984	-147.7879946	5.090019413	-6.430808859
[truncated: 1,205,792 more chars]
